# Synthesis, Evaluation, and Context for Applications of SF_5_‑Containing [1.1.1]Bicyclopentane and [*2*]Staffane “Hybrid Bioisosteres”

**DOI:** 10.1021/jacs.6c12832

**Published:** 2026-09-18

**Authors:** Jón Atiba Buldt, Masiel M. Belsuzarri, Ansh Hiten Patel, Yannick Kraemer, Tyson Vu, Riddhiman Banerjee, Jake Anthony Olvera, Dean J. Tantillo, Lauren M. Holder, Cody Ross Pitts

**Affiliations:** † Department of Chemistry, University of California, Davis, California 95616, United States; ‡ Novartis BioMedical Research, Emeryville, California 94608, United States

## Abstract

Bioisosteric replacements are conventionally applied as single-variable changes to a parent molecule during drug discovery campaigns. Herein, we evaluate hybrid bioisosteric replacements as strategic combinations of multiple substitutions that counterbalance the physicochemical consequences of incorporating single bioisosteres individually. In this context, we validate SF_5_-substituted [1.1.1]­bicyclopentanes (SF_5_-BCPs) and SF_5_-[*2*]­staffanes (SF_5_-BCP-BCPs) as *hybrid bioisostere* motifs that have not been field-tested due to lack of access to versatile building blocks. We address a key synthetic limitation by developing scalable routes to carboxylic acid-containing intermediates that facilitate incorporation of these motifs into drug-like molecules *via* carbonyl group manipulations. These advances enable (1) crystallographic studies that contextualize structural features of these motifs, (2) syntheses of structurally diverse pharmaceutical and agrochemical derivatives, and (3) systematic evaluation of single (SF_5_-Ph → SF_5_-BCP) and multicomponent “hybrid” bioisosteric replacements (CF_3_-Ph → SF_5_-BCP and CF_3_-Ph-Ph → SF_5_-BCP-BCP) through *in vitro* ADME profiling. Finally, we investigate the chemical stability profile of these novel SF_5_-[*2*]­staffanes, establish an extensive suite of building blocks, and demonstrate their compatibility with reactions frequently used in medicinal chemistry. Together, the results of this extensive study position SF_5_-BCPs and SF_5_-[*2*]­staffanes as practical hybrid bioisosteres for tuning molecular properties in drug discovery.

## Graphical abstract

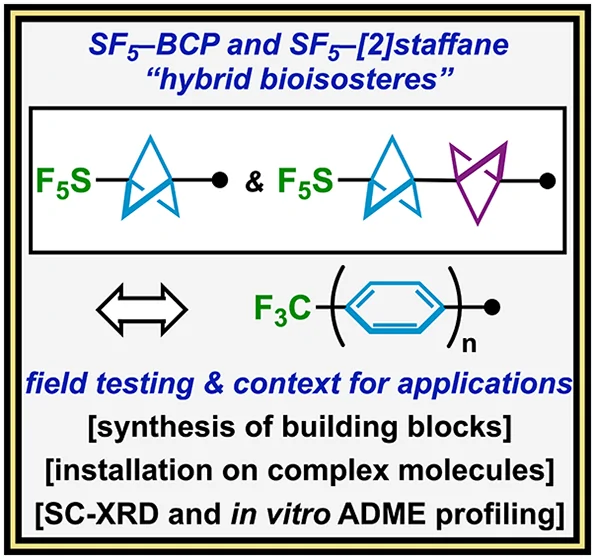

## Introduction

In practice, bioisosteres are employed in drug design either to preserve key geometric features while improving physicochemical and pharmacokinetic (ADME) properties or, conversely, to maintain desirable ADME properties while modulating molecular shape, rigidity, or volume to optimize target engagement.^1^ In this context, the pentafluorosulfanyl (SF_5_) group has attracted increasing interest due to its strong inductive effects, metabolic stability, and unique steric profile; however, its incorporation is often accompanied by a substantial increase in lipophilicity, which can negatively impact overall drug-like properties.^1,2^ In contrast, the incorporation of saturated bioisosteres such as [1.1.1]­bicyclopentanes (BCPs) into pharmaceutically relevant compounds is frequently associated with reduced lipophilicity and increased three-dimensionality.^3–5^ These opposing trends suggest a potentially useful opportunity to balance physicochemical properties through the combination of such motifs within a single structural unit.

In 2022, our laboratory^6^ established proof-of-concept for *strain-release pentafluorosulfanylation* by adding SF_5_Cl^7–38^ across [1.1.1]­propellane,^39–46^ providing the earliest examples of SF_5_-containing [1.1.1]­bicyclopentane (SF_5_-BCP) and [*2*]­staffane (SF_5_-BCP-BCP) motifs. Over the next few years, this sparked a crescendo in the development of synthetic methods centered on SF_5_ radical addition to strain-release reagents^47–52^ beyond [1.1.1]­propellane,^53–62^ including 1-substituted [1.1.0]­bicyclobutanes (BCBs),^63–68^ 1,3-disubstituted BCBs,^69–76^ and 3-substituted aza[1.1.0]­bicyclobutanes (ABBs).^77–85^

These advancements were motivated by the hypothesis that these SF_5_-containing microcycles could serve as a new class of “hybrid bioisosteres” that combine two distinct elements of bioisosterism^86–94^ in a single motif for applications in medicinal chemistry and other arenas.^95–99^ That is, the SF_5_ group has previously been applied as a bioisostere for trifluoromethyl (CF_3_) and *tert*-butyl (*t*-Bu) groups,^100–103^ while BCP and CB rings are known bioisosteres for *para*-substituted phenyl (Ph) rings^104–108^ and azetidines may be considered in place of piperidine rings.^109,110^

Individually, these substitutions have been implemented (Figure 1, *top*),^111–114^ and their effects on physicochemical properties are reasonably well understood.^115–119^ However, the consequences of combining these bioisosteres—such as replacing a CF_3_-Ph motif with an SF_5_-BCP—remain largely unknown. As a result, there is currently limited guidance as to whether these hybrid motifs can be deployed strategically in medicinal chemistry (Figure 1, *bottom*). Moreover, while broader scopes of SF_5_-CB and SF_5_-azetidine building blocks have recently become accessible,^65,66,80,81^ more synthetically versatile SF_5_-BCP and SF_5_-[*2*]­staffane building blocks that can engage in carbonyl functionalization chemistry remain elusive. This has hindered systematic incorporation of these motifs into complex molecules, and thus, evaluation of their effects on parent compound properties.

**Figure 1:**
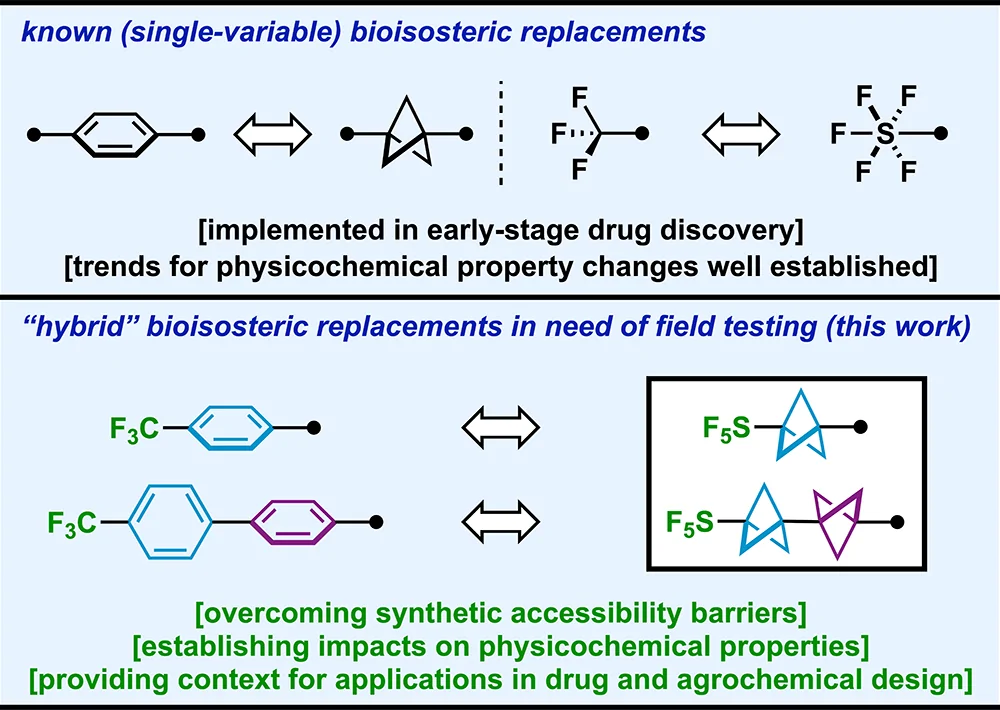
(*Top*) Examples of conventional bioisosteric replacements (Ph → BCP and CF_3_ → SF_5_). (*bottom*) Heretofore unevaluated “hybrid” bioisosteric replacements that serve as the focus of this synthetic, structural, and comparative *in vitro* ADME study.

Accordingly, the present work establishes both synthetic accessibility and functional utility of SF_5_-BCP and SF_5_-[*2*]­staffane hybrid bioisosteres. First, we disclose routes for gram-scale syntheses of SF_5_-BCP and SF_5_-[*2*]­staffane building blocks containing a carboxylic acid and note synthetic challenges associated with SF_5_-BCP-X (X = Cl, I) bridgehead functionalization. We then apply these building blocks to the synthesis and *in vitro* ADME profiling for a series of structurally diverse drug and agrochemical derivatives to evaluate the effects of single (SF_5_-Ph → SF_5_-BCP) and multicomponent “hybrid” bioisosteric replacements (CF_3_-Ph → SF_5_-BCP and CF_3_-Ph-Ph → SF_5_-BCP-BCP) on physicochemical properties. Recognizing that the impact of bioisosteric substitution on ADME properties is highly context-dependent, the aim of this study was to determine whether these hybrid motifs can be incorporated without compromising key drug-relevant properties across a range of molecular scaffolds.

During this evaluation, we also identified and studied an unexpected degradation pathway for SF_5_-[*2*]­staffanes upon prolonged storage in DMSO, which carries important implications for assay design and *in vitro* ADME data interpretation. Although this observation suggests limited application *prima facie*, we demonstrate that SF_5_-[*2*]­staffanes are chemically stable under the most commonly employed reactions in medicinal chemistry, as highlighted by Brown and Boström.^120^ Lastly, we leverage the synthetic accessibility of SF_5_-[*2*]­staffanes to establish an extensive suite of building blocks that we anticipate will be useful to medicinal chemists and others seeking to incorporate these motifs in applied synthetic contexts.

## Results and Discussion

### Access to Carboxylic Acid Building Blocks

The only reported SF_5_-BCP building blocks to date contain a halogen (*viz*. Cl or I) at the remaining bridgehead position (Figure 2, *top left*). In 2022, we disclosed the synthesis and structural analysis of SF_5_-BCP-Cl (**1**), as well as several unsuccessful attempts to functionalize the recalcitrant C–Cl bond.^6^ In 2023, the Qing laboratory disclosed the synthesis of SF_5_-BCP-I (**2**) and demonstrated it to be a more suitable SF_5_-BCP radical precursor, enabling SF_5_-BCP addition to alkenes/alkynes (using a photoredox strategy adapted from Anderson and co-workers^121^) and arenediazonium salts^122^ (inspired by a base-promoted XAT strategy demonstrated for iodo-BCP functionalization by Ni, Shao, and co-workers^123^). While these SF_5_-BCP-I transformations represent important advances in the installation of the SF_5_-BCP motif onto organic molecules, the methods require expensive reagents (Ir- or Rh-based) and present selectivity and/or scalability challenges.

**Figure 2:**
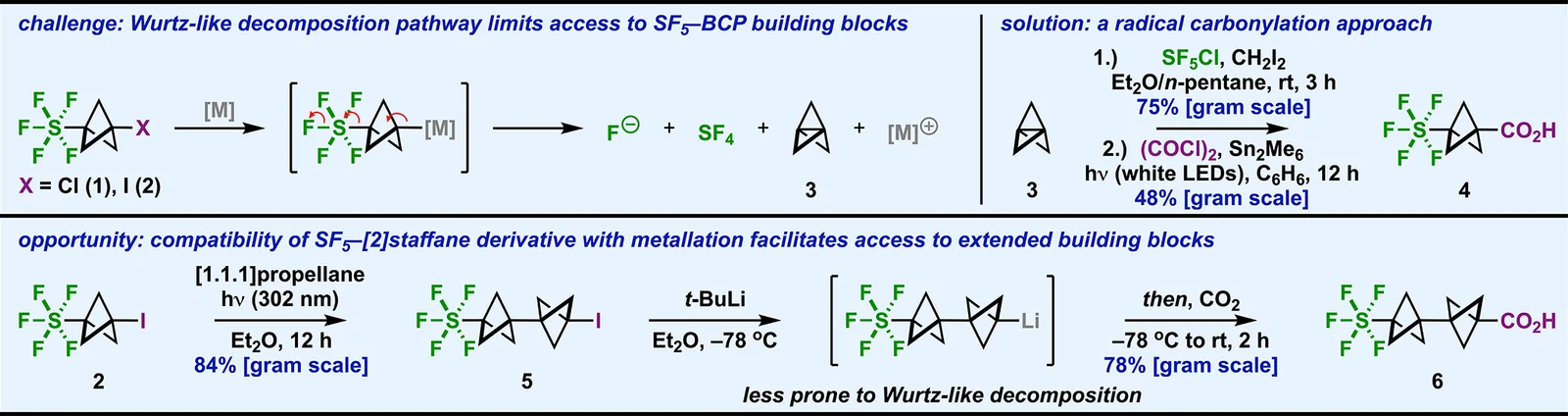
(*Top left*) Decomposition pathway identified upon SF_5_-BCP metalation at the transannular bridgehead position. (*top right*) Gram-scale synthesis of SF_5_-BCP-CO_2_H (**4**). (*bottom*) Gram-scale synthesis of SF_5_-BCP-BCP-CO_2_H (**6**).

We envisioned that SF_5_-BCP-I could be converted to the corresponding carboxylic acid, providing admission for SF_5_-BCP installation to the vast landscape of carbonyl manipulation reactions. Initial attempts at lithiation or magnesiation and subsequent trapping with CO_2_ ^124,125^ led to complete decomposition of the starting material, ostensibly through a Wurtz-like reaction pathway to re-establish [1.1.1]­propellane (**3**),^126–128^ as depicted in Figure 2, *top left*. We have previously noted a similar SF_5_ group decomposition upon putative metalation of the transannular site in SF_5_-CBs, which produced the corresponding BCB and SF_4_, detected by NMR analyses.^44,129^ Considering metalation of the bridgehead position in BCPs is also known to prompt BCP ring opening,^130,131^ we sought to develop a radical carboxylation method to circumvent these challenges.

Inspired by previous work from Michl^132^ and Kim and Jon,^133^ we found that irradiation of SF_5_-BCP-I with oxalyl chloride and hexamethylditin in benzene with white LEDs, followed by aqueous quench, provided SF_5_-BCP-CO_2_H (**4**) in 69% yield (see Supporting Information for optimization table). Note that the syntheses of both SF_5_-BCP-I^44^ and SF_5_-BCP-CO_2_H can be performed on a gram scale in 75% and 48% yields, respectively (Figure 2, *top right*). Furthermore, we found that SF_5_-BCP-CO_2_H can be obtained under tin-free conditions using supersilane instead of hexamethylditin, albeit in diminished yield (37%) due to competitive hydrogen atom abstraction.

Regarding the SF_5_-[*2*]­staffane motif, only one building block has been reported to date (i.e., SF_5_-BCP-BCP-Cl)^45^ and no tactics for its C–Cl bond functionalization have been established. To facilitate bridgehead functionalization, we envisioned that the corresponding iodide (i.e., SF_5_-BCP-BCP-I) would be accessible using a photochemical BCP homologation strategy pioneered by Michl and co-workers.^132^ Accordingly, we found that irradiation of SF_5_-BCP-I in Et_2_O at 302 nm in the presence of [1.1.1]­propellane provided the corresponding SF_5_-[*2*]­staffane (**5**) in 84% yield on a gram scale (Figure 2, *bottom*). In contrast to SF_5_-BCP, the SF_5_ group in the [*2*]­staffane is located distal to the C–I bond, rendering the Wurtz-like decomposition pathway following metalation no longer operative. Thus, SF_5_-BCP-BCP-CO_2_H (**6**) can be synthesized more directly *via* straightforward lithiation and CO_2_ trapping, providing the product in 78% yield on a gram scale without detriment to the SF_5_ group (Figure 2, *bottom*).

### Single Replacement: SF_5_-Ph **→** SF_5_-BCP

With access to these building blocks in gram quantities, we explored their application in the syntheses of various drug and agrochemical derivatives. Our general approach throughout this portion of the study was to (1) select structurally diverse bioactive “reference” molecules from academic, industrial, and patent literature, (2) establish synthetic routes to their corresponding SF_5_-BCP or SF_5_-[*2*]­staffane derivatives, and (3) subject both the reference compounds and their derivatives to *in vitro* ADME profiling and assess changes in physicochemical properties.

As an initial point of reference, we began by evaluating the SF_5_-BCP motif in the context of a single matched-pair Ph → BCP bioisostere prior to examining hybrid replacements. We selected two small molecules containing SF_5_-Ph motifs that could be installed in the final synthetic step using the corresponding carboxylic acid building block. The first compound (SF_5_-BP897; **7**) was reported by Stark and co-workers as a dopamine D_3_ receptor ligand with potential applications in the treatment of addiction and other neurological diseases.^134^

The SF_5_-BCP derivative of SF_5_-BP897 was synthesized from piperidine **8** by intercepting a known intermediate from the reported route (Figure 3, *top*).^134^ The SF_5_-BCP motif was installed successfully during the last step under standard EDC/HOBt amide coupling conditions using **4** to provide SF_5_-BCP-BP897 (**9**) in 54% total isolated yield over 3 steps. The reference molecule was synthesized according to literature,^134^ and both compounds were subjected to *in vitro* ADME profiling.

**Figure 3:**
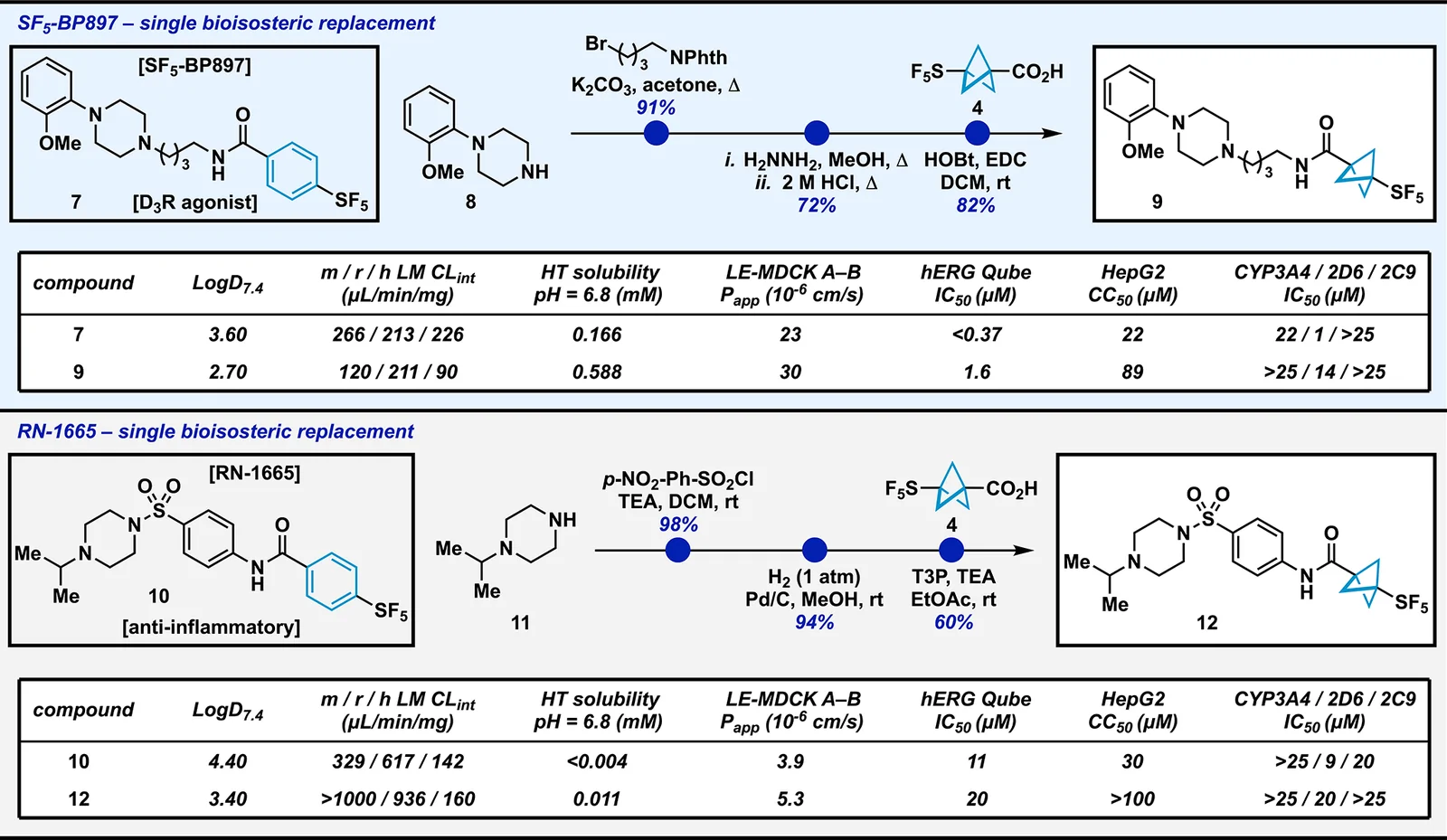
Evaluation of the SF_5_-Ph → SF_5_-BCP single bioisosteric replacement. (*top*) Synthesis of a derivative (**9**) of a previously reported D_3_R agonist (**7**) and summary of *in vitro* ADME properties. (*bottom*) Synthesis of a derivative (**12**) of a previously reported TRPV4 ion-channel antagonist (**10**) and summary of *in vitro* ADME properties.

Consistent with previous accounts of Ph → BCP replacements,^61–63^ we found that incorporation of the SF_5_-BCP motif resulted in substantially reduced lipophilicity (LogD_7.4_ = 3.60 → 2.70). The *in vitro* profile measured for **9** was largely comparable to that of SF_5_-BP897 (**7**), with values for human and rodent liver microsomal clearance (LM CL_int_), permeability (MDCK A-B *P*_app_), and CYP3A4/2C9 inhibition falling within standard ∼3-fold experimental variability. Notable improvements (>3x) were observed for high throughput (HT) solubility, cytotoxicity, and hERG/CYP2D6 inhibition. These results are consistent with an overall favorable outcome for this SF_5_-Ph → SF_5_-BCP bioisosteric replacement.

For a second point of comparison, an SF_5_-containing antagonist of the transient receptor potential vanilloid (TRPV4) ion-channel RN-1665 (**10**)^135^ was selected for single matched-pair bioisosteric replacement. The corresponding SF_5_-BCP derivative of RN-1665 (**12**) was prepared using a modified synthesis route reported for its reference molecule (Figure 3, *bottom*).^135^ Following sulfonamide formation on piperidine **11** and subsequent nitro group reduction, the SF_5_-BCP motif was installed *via* amide coupling with **4** using propanephosphonic acid anhydride (T3P), providing compound **12** in 55% total isolated yield over three steps.

As in the previous example, incorporation of the SF_5_-BCP motif in **12** resulted in decreased lipophilicity relative to the parent compound (LogD_7.4_ = 4.40 → 3.40). The *in vitro* ADME profiles were generally comparable with marginal or moderate improvements observed for the SF_5_-BCP derivative with respect to HT solubility, permeability, and inhibition of hERG, CYP2D6/2C9. Perhaps most significantly, the moderate cytotoxicity observed for RN-1665 (HepG2 CC_50_ = 30 μM) was dialed out in the SF_5_-BCP derivative (HepG2 CC_50_ > 100 μM).

In both examples of SF_5_-Ph → SF_5_-BCP replacement, this modification was associated with decreased lipophilicity and reduced cytotoxicity, while other physicochemical properties were broadly maintained. Thus, the *in vitro* ADME data from these examples support the SF_5_-BCP motif as a viable modification for incorporation into downstream structure–activity relationship (SAR) studies.

### Double “Hybrid” Replacement: CF_3_-Ph **→** SF_5_-BCP

Our next objective was to examine the effects of a double, or “hybrid” bioisosteric replacement of a *para*-CF_3_-substituted phenyl ring (CF_3_-Ph) with SF_5_-BCP. While only two aryl-SF_5_-containing molecules, DSM265 ^136^ and NVP-IWY357,^137^ have advanced to clinical trials, approximately 15% of fluorinated pharmaceuticals^138^ and 28% of fluorinated agrochemicals^139^ contain a trifluoromethylated arene. Ostensibly, opportunities to implement CF_3_-Ph → SF_5_-BCP replacements may present themselves more frequently given the relative synthetic accessibility of arene trifluoromethylation chemistry^140–142^ vs arene pentafluorosulfanylation.^8,19,143^ Accordingly, we selected one representative example of a commercial agrochemical and one FDA-approved pharmaceutical that each contain a CF_3_-Ph motif and synthesized the corresponding SF_5_-BCP derivatives (Figure 4).

**Figure 4:**
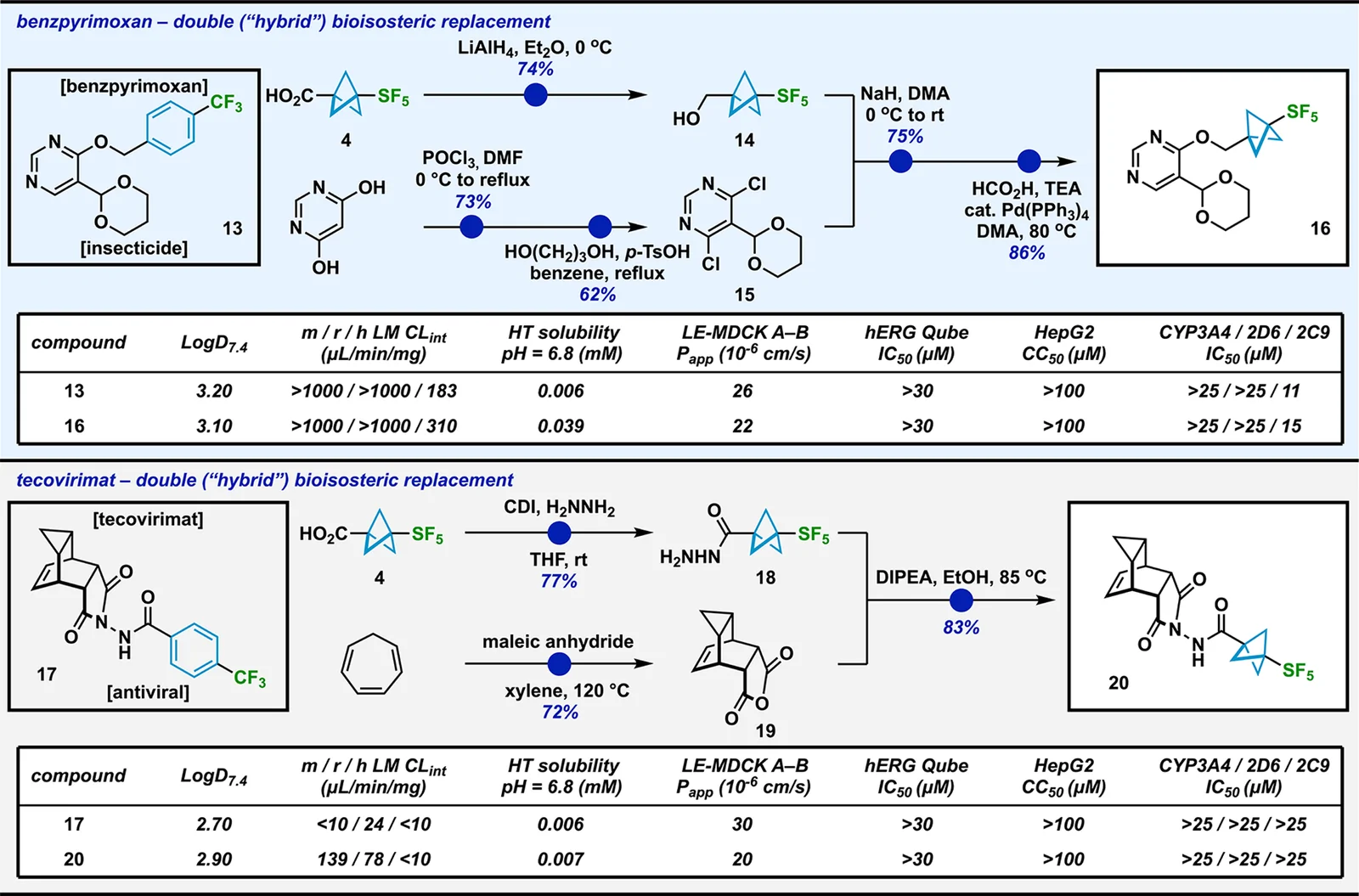
Evaluation of the CF_3_-Ph → SF_5_-BCP double hybrid bioisosteric replacement. (*top*) Synthesis of a derivative (**16**) of an FDA-approved insecticide—benzpyrimoxan (**13**)—and summary of *in vitro* ADME properties. (*bottom*) Synthesis of a derivative (**20**) of an FDA-approved antiviral—tecovirimat (**17**)—and summary of *in vitro* ADME properties.

Our first target for derivatization was benzypyrimoxan (**13**), an insecticide used in rice paddy fields.^144^ To access its hybrid bioisostere, carboxylic acid **4** was subjected to standard LiAlH_4_ reduction conditions, providing alcohol **14** in 74% isolated yield. This building block was appended to pyrimidine intermediate **15** *via* nucleophilic aromatic substitution (S_N_Ar), and subsequent Pd-catalyzed aromatic hydrodehalogenation afforded the SF_5_-BCP derivative of benzpyrimoxan (**16**) in 48% yield over three steps from **4** (Figure 4, *top*).

Substitution of an aryl-CF_3_ group with SF_5_ is typically accompanied by a stark increase in lipophilicity, whereas a Ph → BCP replacement generally results in the opposite trend. A key question is whether the composite effect of replacing both motifs simultaneously (CF_3_-Ph → SF_5_-BCP) leads to a cancellation of these effects (i.e., no change in lipophilicity) or whether the influence of one bioisostere predominates. In this case, the net result of hybrid bioisosteric replacement was *minimal change in measured lipophilicity*, with LogD_7.4_ decreasing slightly from 3.20 in benzpyrimoxan to 3.10 in the SF_5_-BCP derivative. Gratifyingly, we observed a 6.5-fold boost in solubility, while the remaining ADME properties measured for **16** were largely preserved. This suggests that the SF_5_-BCP motif can serve as a reasonable physicochemical mimic of CF_3_-Ph in this context.

Our next target for derivatization was tecovirimat (**17**), a first-in-class FDA-approved antiviral with activity against smallpox and Mpox.^145^ Carboxylic acid **4** was converted into acyl hydrazide building block **18**, which was condensed with known anhydride intermediate^146^ **19** to afford the SF_5_-BCP derivative of tecovirimat (**20**) in 64% isolated yield over 2 steps (Figure 4, *bottom*). Comparative *in vitro* ADME profiling of **17** and **20** revealed only a small increase in lipophilicity (LogD_7.4_ = 2.70 → 2.90). As observed in the RN-1665 system (*vide supra*), tecovirimat exhibited lower intrinsic clearance in rodent liver microsomes, while human CL_int_ values were more closely aligned between the two compounds. All other physicochemical properties closely matched those for tecovirimat.

In both examples of CF_3_-Ph → SF_5_-BCP replacement, this hybrid bioisostere permitted maintenance of physicochemical properties relative to the reference compounds, while affording a distinct three-dimensional structure. These results suggest that CF_3_-Ph → SF_5_-BCP substitution may provide a viable strategy for preserving ADME profiles while introducing structural novelty. In this context, the SF_5_-BCP motif may also be particularly attractive to industrial chemists as a means to access differentiated intellectual property (IP) space.^147^

### Structural Comparison of SF_5_-BCP Relative to Other Fluorinated Rings

Having established that SF_5_-BCP incorporation is generally well tolerated from an ADME perspective, we next examined its structural features relative to other fluorinated rings (i.e., in compounds **21**-**24**)^102^ to provide context for molecular length and volume—key design-relevant parameters that can influence biological target engagement. Note that structural comparisons between SF_5_-BCP and other BCPs have been reported elsewhere.^6,108^

Crystal structures were determined for CF_3_-BCP-CO_2_H (**21**), SF_5_-BCP-CO_2_H (**4**), and SF_5_-Ph-CO_2_H (**24**) and analyzed alongside X-ray data for F-Ph-CO_2_H (**22**) and CF_3_-Ph-CO_2_H (**23**) that were previously deposited in the CCDC (Figure 5).^148,149^ (An identical structure of compound **24** was also previously reported.^150^) Upon measuring the end-to-end distances for each building block, the observed trend was **21** < **22** < **23** < **4** < **24**. Key takeaways include (1) replacing Ph → BCP in CF_3_-Ph-CO_2_H (**23**) shortens the end-to-end distance below that of a CF_3_ → F substitution, (2) replacing CF_3_ → SF_5_ in CF_3_-Ph-CO_2_H (**23**) results in a significant increase in length (>1.3 Å), whereas the hybrid CF_3_-Ph → SF_5_-BCP replacement leads to a more modest increase of ∼0.4 Å, and (3) a hybrid F-Ph → SF_5_-BCP replacement may maintain the fluorine exit vector in a linear array, while increasing the overall end-to-end distance by ∼20%.

**Figure 5:**
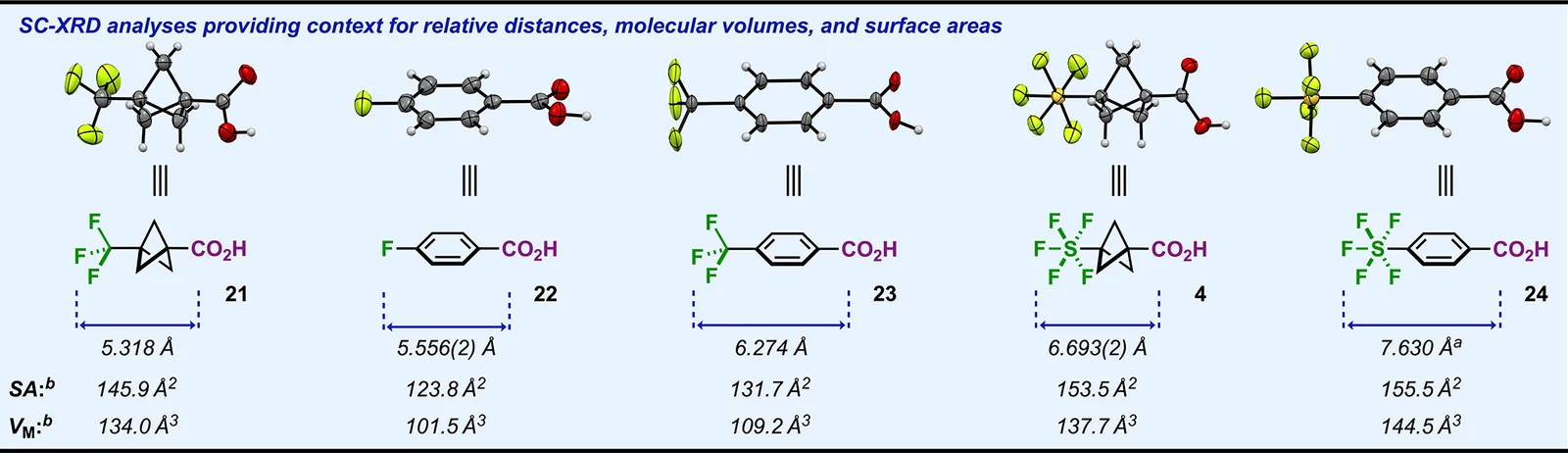
X-ray crystal structure of SF_5_-BCP building block **4** amid various fluorinated ring building blocks, determined by SC-XRD (displacement ellipsoids depicted at 50% probability). End-to-end distances are measured from carbonyl carbon atoms to the terminal fluorine atom in the linear array for **4**, **22**, and **24**, or to a calculated centroid for CF_3_-containing compounds **21** and **23**. ^a^Average end-to-end distance of the two symmetry-independent moieties in the unit cell (only one shown). ^b^Molecular volumes (*V*_M_) and surface areas (*SA*) were calculated in UCSF Chimera.^151^

Analysis of solvent-excluded surfaces (SES) using the program UCSF Chimera^151^ also enabled comparisons of approximate molecular volumes (*V*_M_) and surface areas (*SA*). Compounds **4** and **22**-**24** follow the same trend observed for end-to-end distances; however, CF_3_-BCP-CO_2_H (**21**) displays both a larger volume and surface area than either **22** or **23**. This highlights the three-dimensional impact of the Ph → BCP replacement in isolation. Interestingly, the differences in molecular volume and surface area between **21** and **4** are less pronounced than those observed between **23** and **24**, indicating that the CF_3_ → SF_5_ replacement on BCP rings has a smaller impact on these properties than the analogous CF_3_ → SF_5_ replacement on phenyl rings.

To complement these structural comparisons with corresponding physicochemical data, we selected the BP897 scaffold for ease of preparation of an expanded set of analogs incorporating building blocks **21**-**23.** The resulting compounds **25**-**27** were subjected to *in vitro* ADME profiling and compared alongside **7** and **9** (Figure 6).

**Figure 6:**
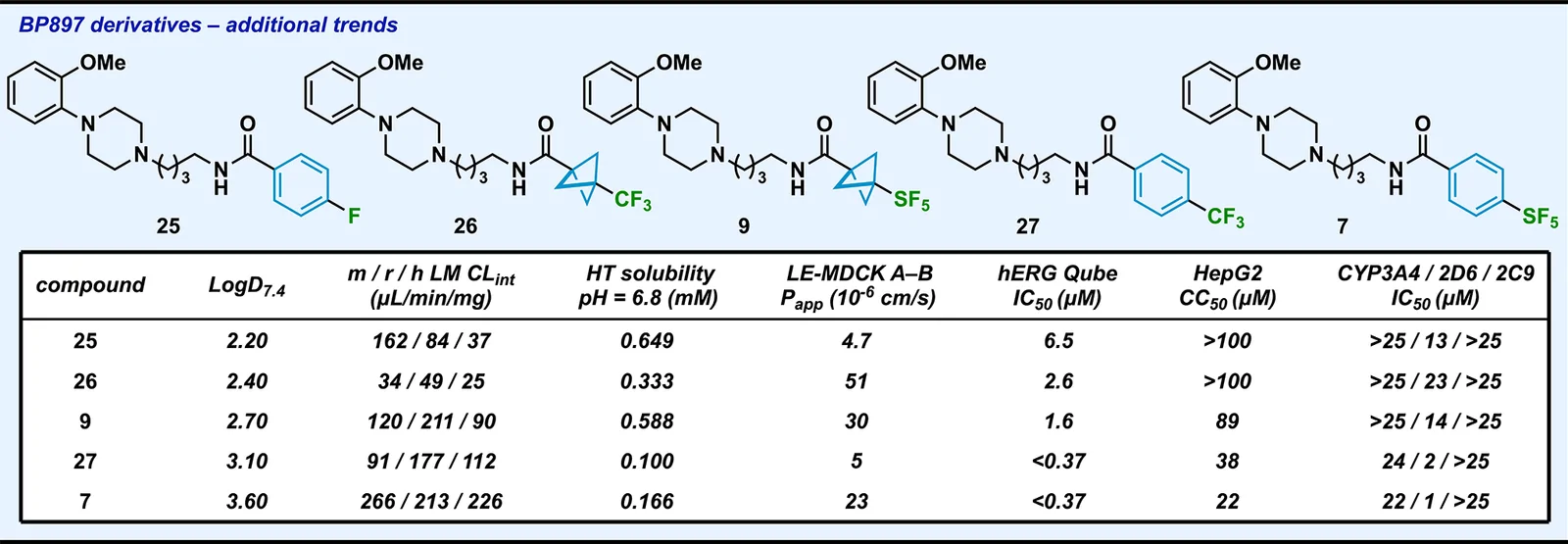
Evaluation of the SF_5_-BCP motif in the context of various single and hybrid bioisosteric replacements involving other fluorinated rings, using SF_5_-BCP-BP897 **9** as the reference molecule for comparative *in vitro* ADME profiling.

The measured lipophilicity of the SF_5_-BCP derivative **9** (LogD_7.4_ = 2.70) falls in the middle of the series, exceeding that of the F-Ph and CF_3_-BCP derivatives **25** and **26** (LogD_7.4_ = 2.20 and 2.40, respectively) and lower than the CF_3_-Ph and SF_5_-Ph derivatives **27** and **7** (LogD_7.4_ = 3.10 and 3.60, respectively). Regarding the hybrid CF_3_-Ph → SF_5_-BCP replacement in BP897, intrinsic clearance, hERG, CYP3A4, and CYP2C9 inhibition are generally maintained, while improvements in solubility and membrane permeability were observed alongside reduced cytotoxicity and CYP2D6 inhibition.

As for the hybrid F-Ph → SF_5_-BCP replacement, the most notable effect is an ∼6-fold increase in membrane permeability (from 4.7 to 30 × 10^–6^ cm/s), while other physicochemical properties are largely similar. Likewise, comparison of the single CF_3_-BCP → SF_5_-BCP replacement demonstrates that the SF_5_-BCP derivative **9** exhibits broadly comparable physicochemical properties to **26**, albeit with reduced metabolic stability, likely reflecting its higher lipophilicity.

Within this series, the *in vitro* data suggest that properties such as liver microsomal clearance and cytotoxicity generally track with lipophilicity, as is often expected. Significant hERG inhibition is observed across all BP897 analogs—a trend well established for lipophilic basic amines, which can also contribute to favorable solubility despite relatively high lipophilicities.

Taken together, these results indicate that each of these bioisosteric motifs are broadly compatible with drug-like physicochemical properties and may be considered viable for use to explore SAR in medicinal chemistry. As such, SF_5_-BCP can be strategically deployed with an understanding of its molecular length, volume, and surface area to potentially optimize target engagement in a context-dependent manner.

Beyond playing a role in modulation of lipophilicity and solubility and offering a different three-dimensional structure, we postulate that the SF_5_-BCP motif may also carry a long-term advantage over CF_3_-BCP (or other CF_3_-containing ring structures) from an environmental perspective. That is, the long-term fate of the fluorine atoms following SF_5_ degradation is likely mineralization, as opposed to formation of persistent trifluoroacetic acid—a common degradation product of CF_3_-containing compounds.^152,153^

### Triple “Hybrid” Replacement: CF_3_-Ph-Ph **→** SF_5_-BCP-BCP

Considering a [*2*]­staffane can be viewed as a double hybrid replacement for a biphenyl motif, the SF_5_-[*2*]­staffane may qualify as a *triple* hybrid replacement for a *para*-CF_3_-substituted biphenyl. To evaluate the impact of this higher-order substitution, we identified two bioactive reference molecules containing a CF_3_-Ph-Ph motif (Figure 7).

**Figure 7:**
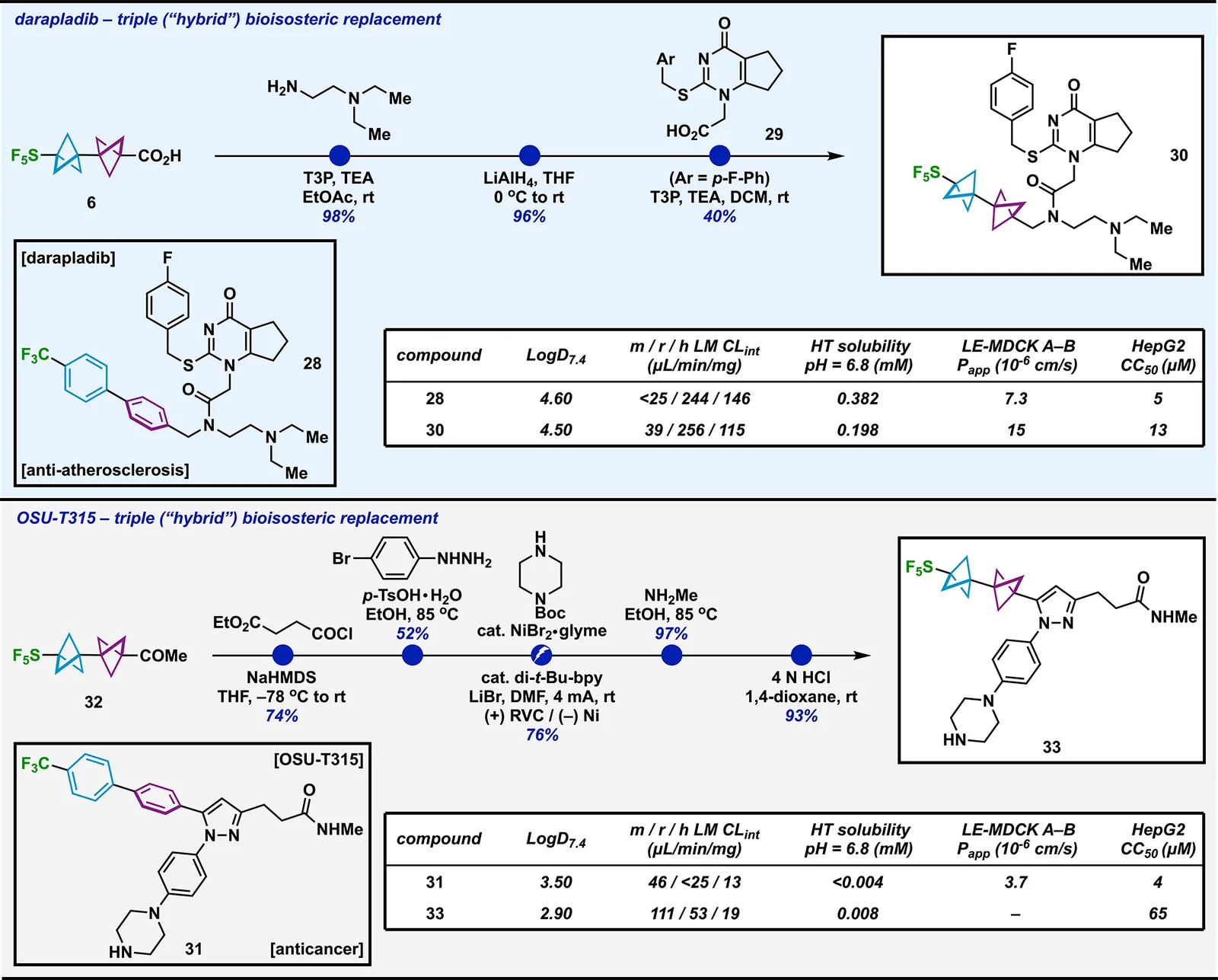
Evaluation of the CF_3_-Ph-Ph → SF_5_-BCP-BCP triple hybrid bioisosteric replacement. (*top*) Synthesis of a derivative (**30**) of darapladib (**28**) and summary of *in vitro* ADME properties. (*bottom*) Synthesis of a derivative (**33**) of OSU-T315 (**31**) and summary of *in vitro* ADME properties.

Darapladib (**28**) is a lipoprotein-associated phospholipase A2 (Lp-PLA_2_) inhibitor originally developed by GSK for the treatment of atherosclerosis.^154^ The SF_5_-[*2*]­staffane derivative was synthesized^104^ by carrying carboxylic acid building block **6** through sequential amide bond formation with *N*,*N*-diethylethane-1,2-diamine, reduction using LiAlH_4_, and amide bond formation with commercially available heterocycle **29** to afford compound **30** in 38% yield over three steps (Figure 7, *top*).

Comparative *in vitro* ADME profiling determined that the lipophilicity of **30** is similar to that of darapladib (LogD_7.4_ = 4.50 vs 4.60) and intrinsic clearance in both rodent and human liver microsomes is likewise comparable. While solubility is modestly reduced for **30**, the SF_5_-[*2*]­staffane derivative exhibits improved permeability and reduced cytotoxicity in this case.

For a second point of comparison, we targeted derivatization of OSU-T315 (**31**), an integrin-linked kinase (ILK) inhibitor with demonstrated potential as an anticancer agent.^155^ In this instance, it was necessary to develop an alternative synthesis to enable incorporation of the [*2*]­staffane building block (Figure 7, *bottom*). Ketone **32** (synthesized in 2 steps from **6**, *vide infra*) was converted into a 1,3-dicarbonyl in 74% yield upon stirring with NaHMDS and ethyl 4-chloro-4-oxobutanoate in THF at −78 °C. Subsequently, regioselective pyrazole formation proceeded in 52% yield, followed by installation of an *N*-Boc-protected piperazine using Baran’s method for electrochemical nickel-catalyzed amination of aryl bromides.^156^ Notably, the SF_5_-[*2*]­staffane motif was well tolerated under electrochemical reaction conditions, which delivered the desired product in 76% yield. Finally, amide bond formation using methylamine and subsequent Boc deprotection provided derivative **33**, which was subjected to *in vitro* ADME profiling.

In contrast to the darapladib example, measured LogD values reveal a more pronounced decrease in lipophilicity for **33**, relative to OSU-T315 (LogD_7.4_ = 3.50 vs 2.90). While intrinsic clearance in rodent liver microsomes is modestly increased for the SF_5_-[*2*]­staffane derivative **33**, human clearance remains comparable to that of the reference molecule. Lastly, a more substantial reduction in cytotoxicity is observed from **31** to **33**, whereas only modest improvement was noted in the darapladib series. Despite multiple attempts, membrane permeability could not be reliably determined for **33** due to low mass recovery in the assay.

Both examples of CF_3_-Ph-Ph → SF_5_-BCP-BCP triple hybrid replacement demonstrate that this multicomponent modification may result in similar or reduced lipophilicity and cytotoxicity, along with shortening of the end-to-end molecular distance. Moreover, physicochemical properties were generally maintained, supporting SF_5_-[*2*]­staffane as viable motifs to incorporate into SAR studies.

### Structural Comparison of SF_5_-[*2*]­Staffane Relative to Other Fluorinated Biphenyls

For additional context, crystal structures were determined for SF_5_-BCP-BCP-CO_2_H (**6**), F-Ph-Ph-CO_2_H (**34**), CF_3_-Ph-Ph-CO_2_H (**35**), and SF_5_-Ph-Ph-CO_2_H (**36**) and subjected to comparative structural analyses (Figure 8). In brief, the end-to-end distance measurements reveal that the SF_5_-[*2*]­staffane motif is ∼4–5% shorter than CF_3_-biphenyl, and virtually on par with that of F-biphenyl. Moreover, analyses of the SESs showcase that the SF_5_-[*2*]­staffane motif falls somewhere in between F-biphenyl and CF_3_-biphenyl in terms of surface area and molecular volume, in line with the trend for end-to-end distances. Altogether, this suggests that, depending on the context, SF_5_-[*2*]­staffanes may be considered more broadly as potential replacements for 4′-fluorinated biphenyl rings.

**Figure 8:**
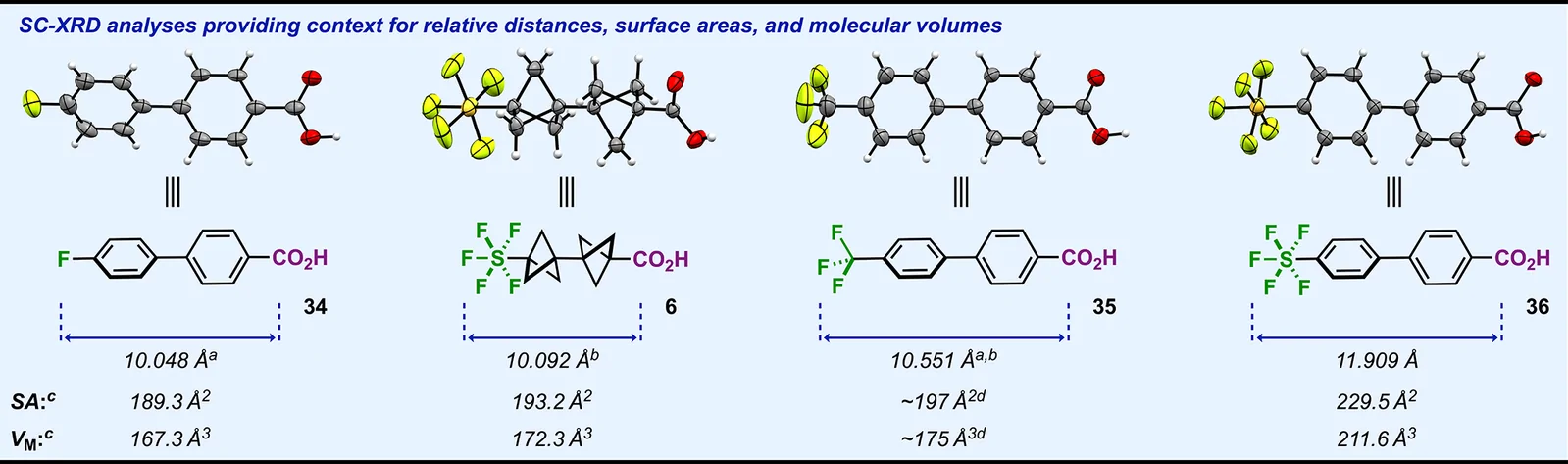
X-ray crystal structure of SF_5_-[*2*]­staffane building block **6** amid various 4′-fluorinated biphenyl building blocks, determined by SC-XRD (displacement ellipsoids depicted at 50% probability). End-to-end distances are measured from carbonyl carbon atoms to the terminal fluorine atom in the linear array for **6**, **34**, and **36**, or to a calculated centroid for CF_3_-containing compound **35**. ^a^Average end-to-end distance of the two symmetry-independent moieties in the unit cell (only one shown). ^b^Solvent molecule from cocrystal omitted for clarity. ^c^Molecular volumes (*V*_M_) and surface areas (SA) were calculated in UCSF Chimera.^151^ ^d^Estimated based on calculated differences between compounds **22** and **23**.

To parlay this idea, an additional matched pair series of proline transporter (PROT) inhibitors (compounds **37**-**40**) was synthesized to further evaluate SF_5_-[*2*]­staffanes amid an extended set of biphenyl derivatives through *in vitro* ADME profiling (Figure 9).^157^ In general, we observed that the physicochemical properties of the 4′-fluorinated biphenyl derivatives **37**, **39**, and **40** are largely preserved in the SF_5_-[*2*]­staffane derivative **38**; that is, solubility, permeability, hERG inhibition, cytotoxicity, and CYP inhibition are nearly identical across the board. Additionally, the measured lipophilicity of **38** (LogD_7.4_ = 4.30) is identical to **39** and situated in between that of **37** (LogD_7.4_ = 3.60) and **40** (LogD_7.4_ = 4.60). One notable difference in metabolic stability was observed, whereby compound **38** exhibited higher intrinsic clearance vs the biphenyl matched pairs across the series. Collectively, these data further support SF_5_-[*2*]­staffanes as viable hybrid bioisosteres for 4′-fluorinated biphenyl motifs. The higher microsomal clearance observed for compound **38** illustrates that topologically distinct bioisosteres may interact differently with biological targets; as highlighted above, these motifs offer a unique three-dimensional profile.

**Figure 9:**
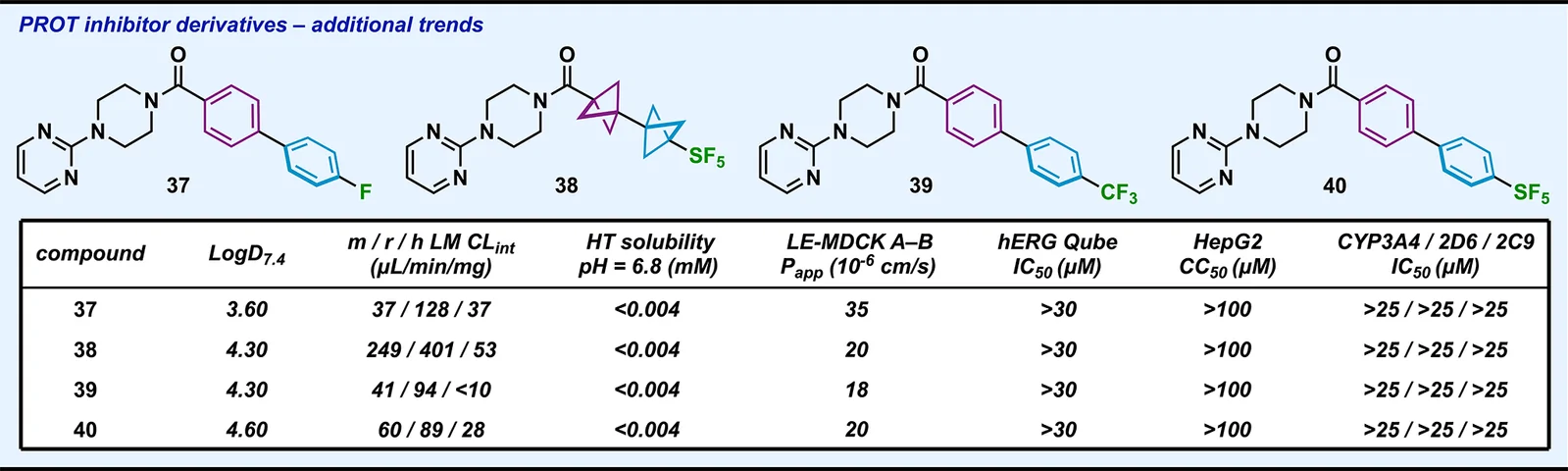
Evaluation of the SF_5_-[*2*]­staffane motif in the context of various hybrid bioisosteric replacements involving other 4′-fluorinated biphenyl rings, using SF_5_-[*2*]­staffane **38** as the reference molecule for comparative *in vitro* ADME profiling.

### Long-Term Instability of SF_5_-[*2*]­Staffanes in DMSO: An Unexpected Degradation Pathway

During the course of *in vitro* ADME profiling for **30** and **33**, we identified an unexpected chemical stability issue associated with SF_5_-[*2*]­staffanes upon prolonged storage in DMSO (QC analyses conducted after 1–4 weeks; see Supporting Information for details), prompting further investigation into their chemical stability. This same phenomenon was not observed for the SF_5_-BCP analogs during the same time frames, suggesting a degradation pathway that may be unique to SF_5_-[*2*]­staffanes.

A solution of OSU-T315 derivative **33** (*m*/*z* = 572.2) in DMSO was stored at rt for 7 days and subsequently analyzed by electrospray ionization in positive ion mode. While some of the SF_5_-[*2*]­staffane remained intact, a major degradation product was observed with an *m*/*z* ratio of 462.3, suggesting that loss of the SF_5_ group and hydration had occurred. This mass is consistent with formation of cyclobutanol derivative **41** (Figure 10). Note that the major degradation product from SF_5_-[*2*]­staffane **30** also displayed an identical mass differential.

**Figure 10:**
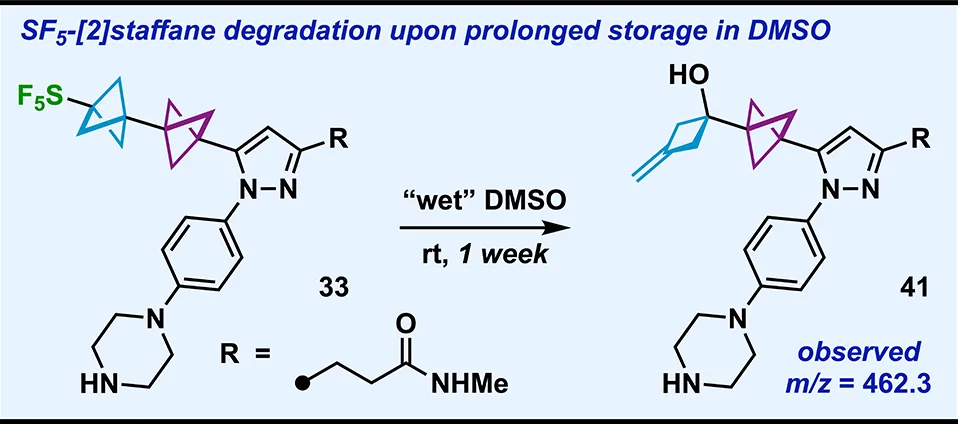
Degradation of SF_5_-[*2*]­staffane **33** to putative cyclobutanol **41** after extended exposure to “wet” DMSO observed *via* ESI+.

Ostensibly, these cyclobutanols arise from ionization of the SF_5_ group concomitant with BCP ring opening,^158–161^ followed by hydration from “wet” DMSO. Nonetheless, a C­(sp^3^)-substituted SF_5_ is not typically considered a leaving group, and since the analogous transformation did not occur for the SF_5_-BCPs, we regarded this unusual observation worthy of additional study.

Accordingly, we synthesized SF_5_-BCP (**42**) and SF_5_-[*2*]­staffane (**43**) model substrates that were equipped with remote aryl fluorides as ^19^F NMR handles to assess relative rates of decomposition (Figure 11, *top*). Stock solutions of each compound (0.047 M) were prepared in various polar organic solvents, including off-the-shelf DMSO-*d*_6_, MeOH-*d*_4_, MeCN-*d*_3_, and the same three solvents doped with 5% D_2_O. The solutions were sealed in NMR tubes under argon at rt with trifluorotoluene as an internal standard, and the SF_5_ and aryl fluoride signals monitored by ^19^F NMR over a period of 5 days at *t* = 0, 12, 24, 48, 72, 96, and 120 h.

**Figure 11:**
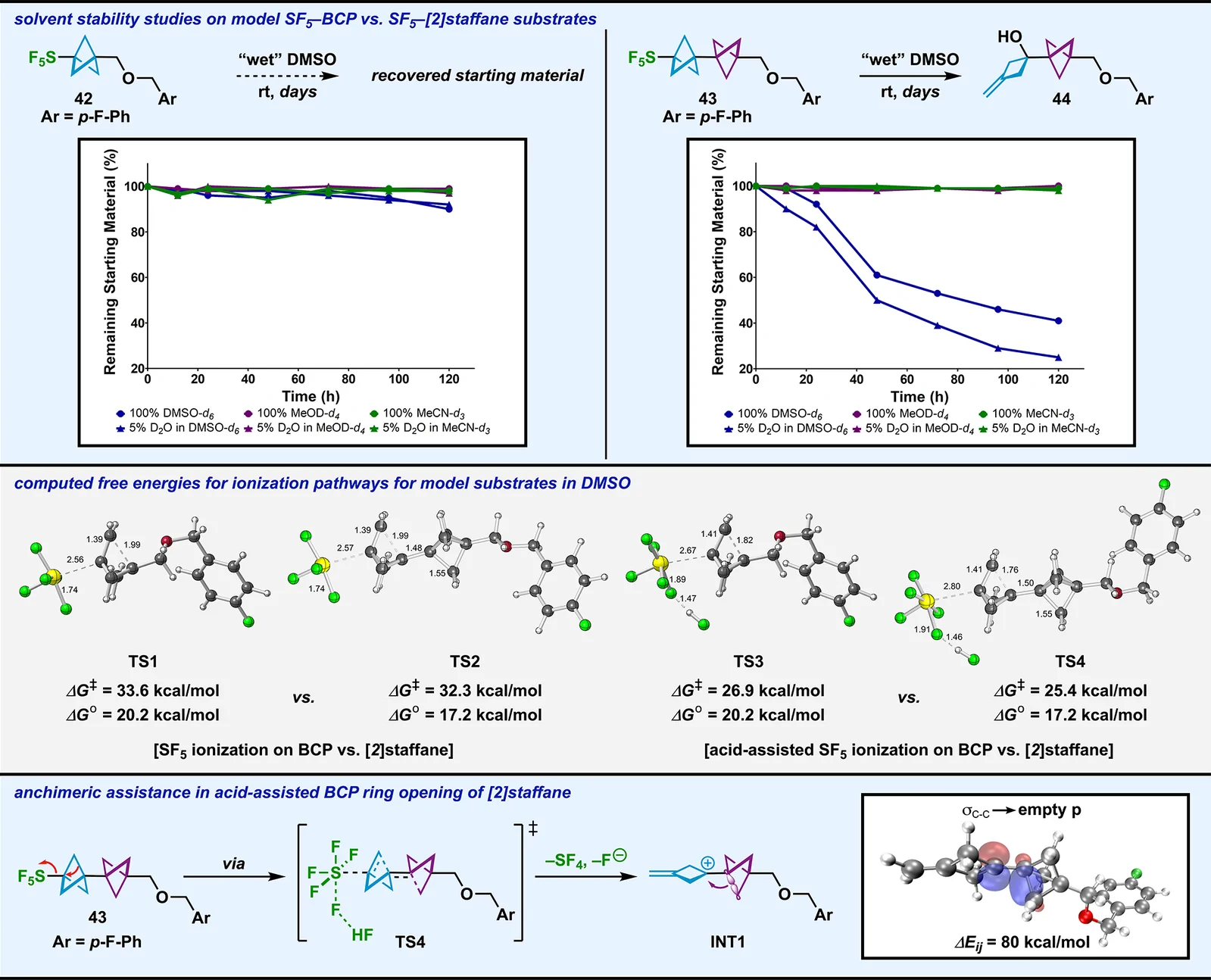
(*Top*) Relative rates of decomposition of model SF_5_-BCP substrate **42** and SF_5_-[*2*]­staffane substrate **43** in various solvents and identification of decomposition product **44**. (*middle*) Transition structures and free energies computed for SF_5_ ionization from **42** (**TS1** and **TS3**) and **43** (**TS2** and **TS4**), with and without acid assistance, at the PCM­(DMSO)-ωB97X-D/def2-SVP level of theory. (*bottom*) Proposed mechanism for SF_5_-[*2*]­staffane ionization, highlighting anchimeric assistance from adjacent BCP “wing” C–C σ-bond. NBOs were generated at the PCM­(DMSO)-ωB97X-D/def2-SVP level of theory.

Consistent with our prior observations in the analytical QC data, the SF_5_-BCP model substrate **42** exhibited negligible degradation during this period in DMSO-*d*_6_, as well as in all other solvents studied. On the other hand, SF_5_–[*2*]­staffane model substrate **43** showed evidence of slow degradation in DMSO at earlier time points, with 99% and 92% starting material remaining at *t* = 12 h and *t* = 24 h, respectively, followed by an accelerated rate after 48 h. By 120 h, only 41% starting material remained; the majority of the material balance corresponded to cyclobutanol **44**, which was isolated and fully characterized. The relative rate of decomposition of **43** was faster in “wet” DMSO (5% D_2_O), with 90% starting material remaining at 12 h, 82% after 24 h, and only 25% after 120 h.

Intriguingly, SF_5_-[*2*]­staffane degradation appears to be unique to DMSO within this solvent series. In MeCN and MeOH (representing alternative polar aprotic and polar protic solvents), virtually all of **43** remained after 120 h. Moreover, **43** remained intact upon addition of water to each of these solvents, indicating that the SF_5_-[*2*]­staffane is not inherently hydrolytically unstable; rather, exogenous water only appears to be deleterious to **43** in DMSO.^162^

To ensure that the reported physicochemical properties for SF_5_-[*2*]­staffanes **30** and **33** in Figure 7 were reliable, samples were prepared from fresh powder immediately prior to assays. The resulting data were largely consistent with those obtained using automated compound plating protocols, in which DMSO stock solutions are prepared in advance (see Supporting Information for comparisons). Nevertheless, these findings suggest that assay results involving SF_5_-[*2*]­staffanes should be interpreted with caution when samples are stored in DMSO for extended periods.

Lastly, compounds **42** and **43** were both subjected to aqueous and thermal stability tests. In brief, only recovered starting material was observed by ^19^F NMR in each case after stirring **42** and **43** in acidic (citrate buffer, pH ∼2.5), neutral (PBS, pH = 7.4), basic (carbonate buffer, pH ∼9), and biological (plasma) media for 24 h at 37 °C. In addition, no evidence of degradation was observed upon heating these compounds to 120 °C in toluene-*d*_6_ for 72 h (see Supporting Information). These results corroborate that SF_5_-[*2*]­staffane degradation is primarily associated with prolonged storage in DMSO.

### Computational Insight on the Mechanism of SF_5_-[*2*]­Staffane Degradation in DMSO

To rationalize the observed differences in SF_5_ ionization between SF_5_-[*2*]­staffanes and SF_5_-BCPs, as well as the apparent role of the solvent, we computed the free energy profiles for the SF_5_ ionization step for SF_5_-BCP **42** and SF_5_-[*2*]­staffane **43** at the PCM­(DMSO)-ωB97X-D/def2-SVP level of theory.^163–166^ The free energy of activation for the ionization of the SF_5_ group in **43** through **TS2** was computed to be 32.3 kcal/mol, which is lower than the barrier computed for ionization of the SF_5_ group in **42** through **TS1** (ΔΔ*G* ^‡^ = 1.2 kcal/mol; Figure 11, *middle*). This is consistent with the experimentally observed faster relative rate of decomposition for the SF_5_-[*2*]­staffane vs the SF_5_-BCP. However, this barrier is too high to account for the observed rate of degradation under the experimental conditions (discussed below). We also observed that the decrease in barrier height is inversely proportional to the dielectric constant of the solvent. This may explain why ionization is more favorable in polar DMSO (ε = 47.2 at 20 °C) than in MeCN (ε = 37.5) or MeOH (ε = 33.6); note that the corresponding computed barriers for the SF_5_-[*2*]­staffane **43** (in kcal/mol) are 32.27, 32.32, and 32.95. This trend holds for the SF_5_-BCP **42**: (in kcal/mol) 33.55, 33.84, and 33.95 (see Supporting Information for details).^167,168^

Analysis of the transition structure (TS) and the resultant ring-opened carbocation intermediate formed upon ionization of SF_5_-[*2*]­staffane **43** revealed several interesting features. First, the S–C bond is elongated from 1.81 Å in the starting material to 2.57 Å in the TS, as anticipated; in addition, S–F_eq_ bond distances increase by ∼8% in the most extreme case (i.e., the F_eq_ atom located antiperiplanar to the opening wing C–C bond). This may suggest that SF_5_ degradation to SF_4_ and fluoride occurs concomitantly with S–C bond homolysis.^129^ In addition, one of the wing C–C bonds of the second BCP ring is poised to overlap with the formally empty p orbital of the resultant carbocation intermediate and is ∼7% longer than the other wing C–C bonds. This suggests that the wing C–C bond aids in carbocation stabilization *via* σ_C–C_ → p orbital hyperconjugation (Figure 11, *bottom*). Indeed, the calculated donor–acceptor orbital interaction (Δ*E*_*ij*_) for this phenomenon in **INT1** is ∼80 kcal/mol.^169–171^ This substantial participation from the neighboring BCP ring accounts for the lower activation barrier, as well as the lower free energy of reaction (ΔΔ*G*° = 3.0 kcal/mol), computed for the SF_5_-[*2*]­staffane vs the SF_5_-BCP.

The sigmoidal-shaped curves obtained from the decomposition studies provided an additional hint to guide our computational studies, as they are suggestive of an autocatalytic process—analogous to how buildup of HBr accelerates electrophilic aromatic bromination of phenols.^172,173^ Following SF_5_ ionization, the group is liable to hydrolyze and extrude HF; note that this process would also be accelerated by an increase in concentration of water. If accumulation of HF in DMSO facilitates subsequent SF_5_ ionization through an acid-assisted mechanism, this could account for the observed accelerated rate of decomposition at later time points.^151,161^

Consistent with this hypothesis (Figure 11, *middle*), coordination of one HF molecule to the F_eq_ atom on the SF_5_ group when optimizing the ionization TSs lowers the activation energy barriers significantly for both the SF_5_-[*2*]­staffane (from Δ*G* ^‡^ = 32.3 to 25.4 kcal/mol; **TS4**) and the SF_5_-BCP (from Δ*G* ^‡^ = 33.6 to 26.9 kcal/mol; **TS3**). These computed free energies of activation are now in agreement with our relative rate experiments, as 25.4 kcal/mol corresponds to a half-life of ∼4 days, whereas 26.9 kcal/mol would correspond to a half-life of ∼49 days.^174–176^

### Programmed Degradation of SF_5_-[*2*]­Staffanes: A Path to SF_5_-BCP Building Blocks Inspired from Mechanistic Insight

The participation of the neighboring BCP ring during ionization/BCP ring opening can conceivably be leveraged from a synthetic standpoint. For instance, ionization of “X” in SF_5_-BCP-BCP-X would prompt an adjacent BCP ring-opening event, while leaving the residual SF_5_-BCP motif intact, thereby providing access to new classes of SF_5_-BCP-containing building blocks (Figure 12).

**Figure 12:**
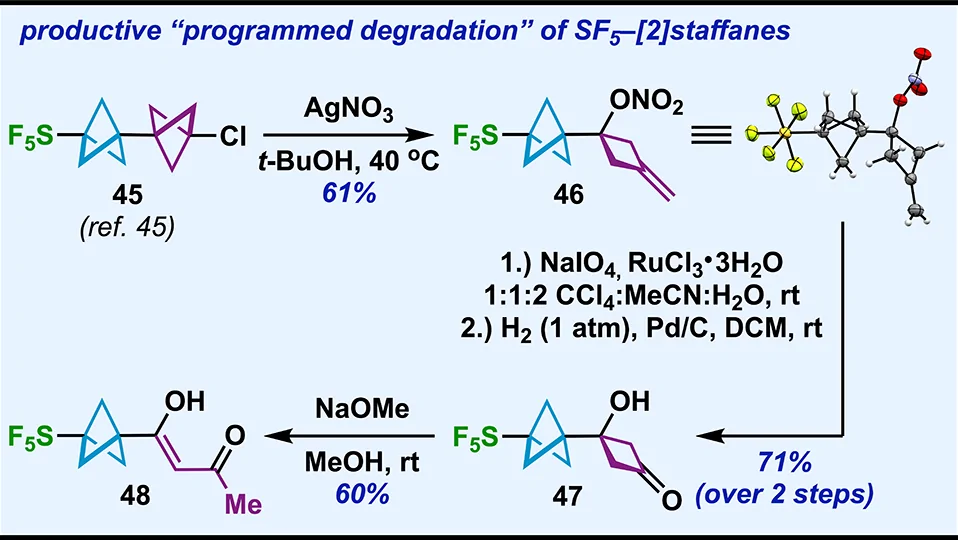
Exploring a programmed degradation strategy to utilize SF_5_-[*2*]­staffanes as a means to access other classes of SF_5_-BCP building blocks.

We explored this *programmed degradation* concept using SF_5_-BCP-BCP-Cl (**45**), which we previously reported is accessible in a single step from [1.1.1]­propellane.^45^ After screening a series of Ag­(I) salts to promote ionization through AgCl formation,^177^ we found that stirring **45** with AgNO_3_ in *t*-BuOH at 40 °C provides nitrate-containing exocyclic cyclobutene **46** in 61% isolated yield. The structure of this organic nitrate was confirmed by SC-XRD. Oxidation of the terminal alkene to the corresponding cyclobutanone using NaIO_4_ and RuCl_3_·3H_2_O^178^ and removal of the nitrate under standard hydrogenation conditions^179^ provided 3-hydroxy-cyclobutanone **47** after recrystallization in 71% yield over 2 steps. Interestingly, this sequence highlights the potential for organic nitrates to serve as unconventional protecting groups for alcohols under highly oxidizing reaction conditions.^180^

Lastly, we found that 3-hydroxy-cyclobutanone **47** may undergo a retro-aldol ring opening^181^ upon stirring with NaOMe in MeOH at rt to provide β-dicarbonyl **48** (characterized as the enol tautomer) in 60% yield. Collectively, these examples demonstrate that intentionally orchestrated degradation of SF_5_-[*2*]­staffanes can be applied to access diverse SF_5_-BCP-containing building blocks that would not be readily obtainable through direct strain-release functionalization or related approaches.

### Extensive Examination of Chemical Stability Establishes a Versatile Suite of SF_5_-[*2*]­Staffane Building Blocks

At this juncture, we sought to demonstrate the compatibility of SF_5_-[*2*]­staffanes with a wide variety of reaction conditions, while establishing a suite of building blocks that may be useful within, and beyond, the context of a medicinal chemistry setting (Figure 13, *top*).

**Figure 13:**
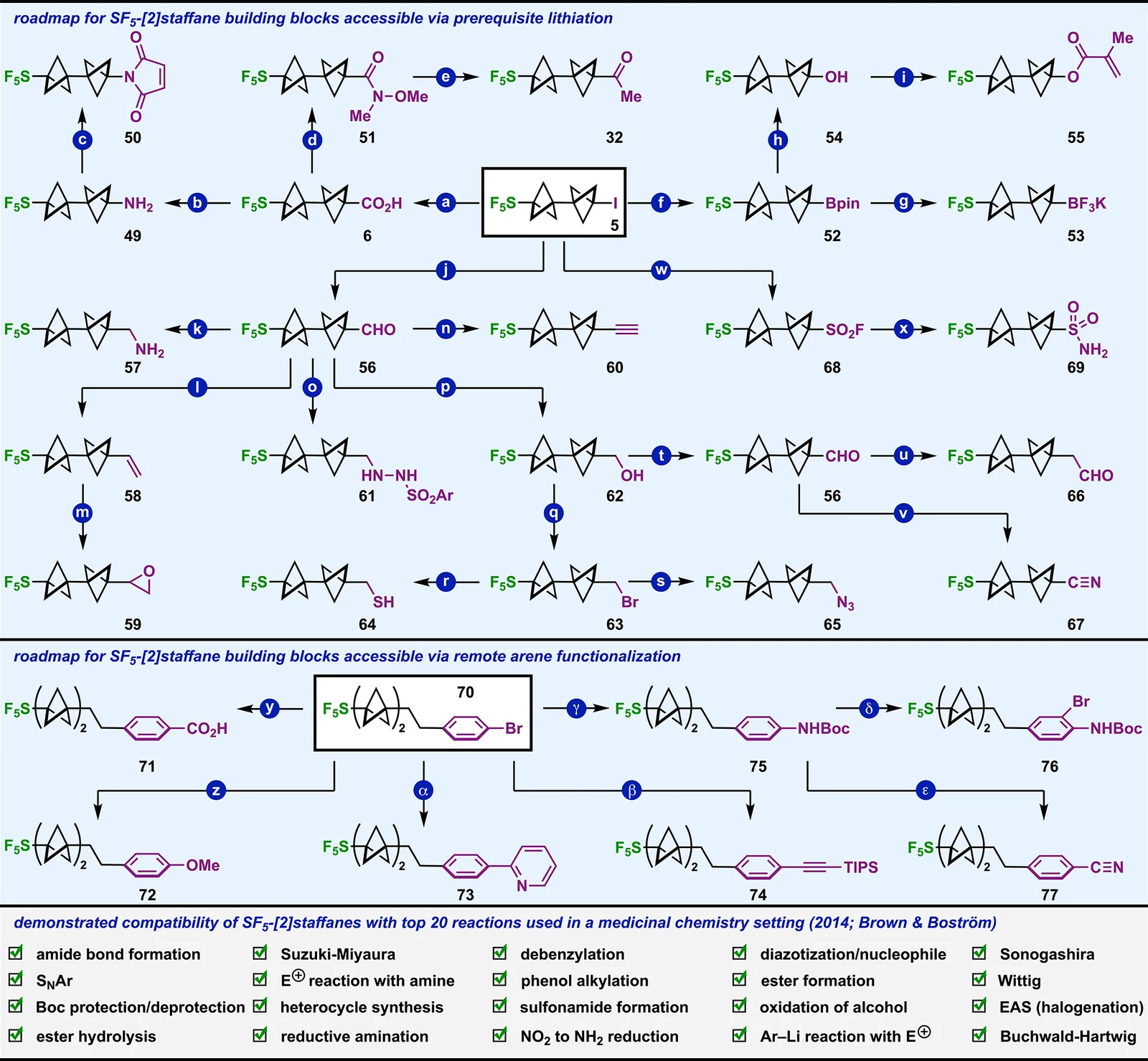
(*Top*) Reaction conditions: (a) *t*-BuLi, CO_2_, Et_2_O, −78 °C to rt, 2 h, (78%); (b) *i.* DPPA, TEA, benzene, 80 °C, 3 h, *then*, BnOH, 80 °C, 24 h (72%); *ii.* Pd/C, MeOH/THF (1:1), H_2_ (1 atm), rt, 16 h (96%); (c) *i.* maleic anhydride, DCM, 0 to 45 °C, 18 h; *ii.* KOAc, Ac_2_O, 60 °C, 5 h (63%); (d) *i.* (COCl)_2_, cat. DMF, 0 °C to rt, 3 h; *ii.* DIPEA, *N*,*O*-dimethylhydroxylamine hydrochloride, 0 °C to rt, 3 h (73% over 2 steps); (e) MeLi (1.6 M in Et_2_O), Et_2_O, 0 °C to rt, 2 h (94%); (f) *t*-BuLi, *i*-OPr-Bpin, Et_2_O, −78 °C to rt, 2 h (99%); (g) KHF_2_, MeOH/H_2_O (4:1), rt, 20 h; (h) UHP, KOAc, THF, 0 °C to rt, 20 h (96%); (i) methacryloyl chloride, TEA, DCM, 0 °C to rt, 12 h (81%); (j) *t*-BuLi, ethyl formate, Et_2_O, −78 °C to rt, 2 h (63%); (k) *i.* 7 M NH_3_ in MeOH, 4 Å MS, rt, 7 h; *ii*. NaBH_4_, 0 °C to rt, 12 h (50%); (l) methyltriphenylphosphonium bromide, *t*-BuOK, THF, 0 °C to rt, 3 h (81%); (m) *m*-CPBA, DCM, 0 °C to rt, 12 h (95%); (n) dimethyl 1-diazo-2-oxopropylphosphonate, K_2_CO_3_, MeOH, 0 °C to rt, 3 h (65%); (o) *i.* sulfonyl hydrazide, MeOH, rt, 2 h; *ii.* NaBH_4_, DCM/MeOH (1:1), 0 °C, 6 h (40% over 2 steps); (p) NaBH_4_, MeOH, 0 °C, 1 h (66%); (q) PPh_3_, CBr_4_, DCM, 0 °C to rt, 2 h (97%); (r) *i.* KSAc, DMF, rt, 12 h; *ii.* 2 M NaOH/acetone (1:1), rt, 1 h (48% over 2 steps); (s) NaN_3_, DMSO, rt, 24 h, (51%); (t) (COCl)_2_, DMSO, TEA, DCM, −78 °C to rt, 3 h (51%); (u) *i.* (methoxymethyl)­triphenylphosphonium chloride, NaHMDS, THF, 0 °C to rt, 4 h; *ii.* 3 M HCl, 0 °C to rt, 1 h (53%); (v) DPPH, toluene, rt to 85 °C, 16 h; (w) *i. t*-BuLi, SO_2_Cl_2_, Et_2_O, −78 °C to rt, 2 h (38%); *ii.* KF, 18-crown-6, MeCN, rt, 12 h (93%); (x) 7 M NH_3_ in MeOH, rt, 12 h (93%). (*middle*) Reaction conditions: (y) *t*-BuLi, CO_2_, Et_2_O, −78 °C to rt, 2 h, (62%); (z) *i. t*-BuLi, *i*-OPr-Bpin, Et_2_O, −78 °C to rt, 2 h *ii.* UHP, KOAc, THF, 0 °C to rt, 20 h (75%) *iii.* NaH, MeI, THF, 0 °C to rt, 12 h (59%); α) 2-bromopyridine, Pd­(dppf)­Cl_2_, K_3_PO_4_, DME, 85 °C, 12 h (50%); β) TIPS acetylene, Pd­(PPh_3_)_2_Cl_2_, CuI, PPh_3_, TEA, 90 °C, 18 h, (70%); γ) NH_2_Boc, Pd­(OAc)_2_, XPhos, Cs_2_CO_3_, 1,4-dioxane, 90 °C, 3 h (91%); δ) NBS, MeCN, 50 °C, 3 h (68%); ε) *i.* 4 N HCl in 1,4-dioxane, rt, 12 h; *ii.* sat. aq. NaHCO_3_ (75%); *iii*. NaCN, CuCN, 2 M HCl, NaNO_2,_ H_2_O, 0 °C (51%). (*bottom*) Checklist of reactions performed throughout this study demonstrating compatibility of the SF_5_-[*2*]­staffane motif with the top 20 reactions used in a medicinal chemistry setting in 2014, as outlined by Brown and Boström.^120^

Starting from SF_5_-BCP-BCP-I (**5**), we previously demonstrated access to carboxylic acid **6** *via* straightforward lithiation and CO_2_ trapping. From **6**, the corresponding amine **49** was obtained in 72% yield over two steps through a Curtius rearrangement to furnish the *N*-Cbz-protected amine, followed by hydrogenolysis.^124^ Alternatively, the *N*-Boc-protected amine was accessible in 88% yield and deprotected under acidic conditions to afford the HCl salt of **49** in 79% yield (see Supporting Information for details).^124^ Amine **49** can also be converted to maleimide **50** in 63% yield over two steps,^182^ providing a functional group handle that may be useful for bioconjugation.^183^

Carboxylic acid **6** was converted into the corresponding Weinreb amide **51** *via* amide bond formation from an acid chloride intermediate in 73% yield.^184^ Subsequent alkylation with MeLi provided methyl ketone **32** in 94% yield.^182^ In addition, we established that the corresponding methyl ester of **6** can be synthesized using MeI and K_2_CO_3_ (85% yield) and that the SF_5_-[*2*]­staffane motif also tolerates mild saponification conditions to revert the methyl ester back into **6** (91% yield; see Supporting Information for details).^185,186^

We further explored the use of other trapping reagents following bridgehead lithiation of **5**. For instance, borylation using *i*-OPr-Bpin afforded boronate ester **52** in 99% yield.^125^ In turn, this intermediate was successfully converted to BF_3_K salt **53** using KHF_2_ (78% yield),^125^ and oxidized to the corresponding alcohol **54** using urea hydrogen peroxide (96% yield).^187^ Alcohol **54** provides a convenient handle to install, e.g., a methacrylate moiety *via* addition to methacryloyl chloride to access **55** in 81% yield;^188^ this compound may be of interest as a monomer for polymerization applications.^189^

Beyond synthesizing the carboxylic acid and Bpin derivatives, aldehyde **56** was obtained in 63% yield by trapping the lithiated SF_5_-[*2*]­staffane with ethyl formate.^125^ This intermediate provided access to a diverse set of downstream building blocks. First, we found that reductive amination proceeded under standard conditions to afford amine **57** in 50% yield.^190^ Aldehyde **56** was also subjected to Wittig reaction conditions to provide terminal alkene **58** in 81% yield,^80^ which could be further oxidized to epoxide **59** with *m-*CPBA in 95% yield.^191^ Both derivatives, alongside methacrylate **55**, may be of interest in the context of polymerization.^192,193^ Alkyne **60** was accessed *via* Seyferth–Gilbert homologation in 65% yield.^124^ Thereafter, we demonstrated that aldehyde **56** can be transformed into arylsulfonyl hydrazide **61** in 40% yield over two steps,^194^ which may enable installation of SF_5_-[*2*]­staffanes using modern methods for redox-neutral radical cross-coupling.^193^ Lastly, reduction of **56** with NaBH_4_ afforded primary alcohol **62** in 65% yield,^195^ allowing further evaluation of the compatibility of the SF_5_-[*2*]­staffane motif with substitution and oxidation conditions.

With alcohol **62** in hand, we synthesized the corresponding alkyl bromide **63** in 99% yield under Appel conditions.^196^ Despite β-branching from the BCP ring, the alkyl bromide proved amenable to S_N_2 functionalization, providing thiol **64** *via* nucleophilic attack of KSAc followed by base-promoted hydrolysis (48% yield over 2 steps)^197^ or azide **65** *via* substitution with NaN_3_ (51% yield).^198^ The SF_5_-[*2*]­staffane motif was also found to be compatible with Swern conditions,^199^ as alcohol **62** was oxidized to aldehyde **56** in 51% yield. Furthermore, we found that (1) Wittig–Levine homologation can be employed on this substrate to provide compound **66** in 53% yield,^200^ opening the possibility of exploring enolate chemistry in the presence of an SF_5_-[*2*]­staffane and (2) oxime formation/dehydration afforded nitrile **67** in 69% yield,^201^ creating additional opportunities to incorporate SF_5_-[*2*]­staffanes into heterocycle synthesis.^202^

Lithiation and trapping logic also lends way to C–S bond formation at the bridgehead position. For instance, the bridgehead sulfonyl chloride was synthesized in 38% yield by using SO_2_Cl_2_ as the trapping reagent; in turn, sulfonyl fluoride **68** can be accessed in 93% yield *via* Cl–F exchange on this intermediate using KF and 18-crown-6.^203,204^ Note that sulfonyl fluoride **68**, azide **65**, and alkyne **60** all render the SF_5_-[*2*]­staffane motif amenable to click chemistry.^205,206^ Additionally, the sulfonyl fluoride serves as a precursor to sulfonamides such as **69** (93% yield),^207^ which are used frequently in drug discovery.^208^

Although the SF_5_-[*2*]­staffane motif proved robust over a wide range of reaction conditions outlined above, its compatibility with remote arene functionalization had not yet been explored (Figure 13, *middle*). Accordingly, we synthesized aryl bromide **70** from aldehyde **56** *via* Wittig olefination^209^ and subsequent alkene hydrogenation using Wilkinson’s catalyst^210^ (see Supporting Information for details). Aryl bromide **70** was then subjected to a series of transformations, including lithiation and CO_2_ trapping to provide carboxylic acid **71** in 62% yield and lithiation and trapping with *i*-PrO-Bpin,^125^ followed by oxidation with UHP^186^ and phenol alkylation^211^ with MeI to provide anisole **72** in 44% yield over three steps. The same pinacol boronate intermediate underwent Suzuki cross-coupling to access 2-phenylpyridine (ppy) **73** in 50% yield.^212^ Sonogashira cross-coupling furnished alkyne **74** in 70% yield,^213^ and Buchwald–Hartwig amination provided *N*-Boc-protected aniline **75** in 91% yield.^214^ Subsequent electrophilic aromatic bromination of **75** gave **76** in 68% yield.^215^ Finally, **75** was deprotected and converted to an arenediazonium salt, which was treated with CuCN to afford benzonitrile **77** in 38% yield over two steps.^124,216^

Note that the Supporting Information document contains additional proof-of-concept experiments that demonstrate feasibility for a few more transformations not highlighted above, including radical arylation, decarboxylative Giese, and Matteson homologation reactions.

This derivatization exercise establishes a diverse suite of versatile SF_5_-[*2*]­staffane-based building blocks that enable incorporation of this motif into molecules for use in medicinal chemistry and beyond. Notably, the transformations described herein demonstrate that the SF_5_-[*2*]­staffane motif is compatible with the top 20 reactions employed in a medicinal chemistry setting^120^ (Figure 13, *bottom*).

## Conclusion

The purpose of this multidimensional study is to provide foundational perspective on the synthesis, properties, and potential applications of compounds containing the SF_5_-BCP and SF_5_-[*2*]­staffane motifs. Herein, scalable access to carboxylic acid building blocks **4** and **6** enabled structural comparisons with other fluorinated rings, synthesis of drug and agrochemical derivatives, and comparative *in vitro* ADME profiling with several bioactive reference compounds. Consequently, our results establish proof-of-concept that SF_5_-BCPs and SF_5_-[*2*]­staffanes can serve as viable “hybrid bioisosteres” for CF_3_-Ph and CF_3_-Ph-Ph motifs.^6^

Beyond expanding synthetic access to SF_5_-BCP and SF_5_-[*2*]­staffane building blocks, this work aims to provide a framework for when and how these unusual motifs can be employed in drug or agrochemical design. Specifically, SF_5_-BCP and SF_5_-[*2*]­staffanes may be considered as either single matched-pair replacements, or as part of multivariable “hybrid” substitutions, offering a strategy to balance competing physicochemical effects. The *in vitro* ADME results discussed herein provide initial guidance through realistic examples of the potential physicochemical property changes that may arise across diverse drug-like molecular scaffolds (Figure 14).

**Figure 14:**
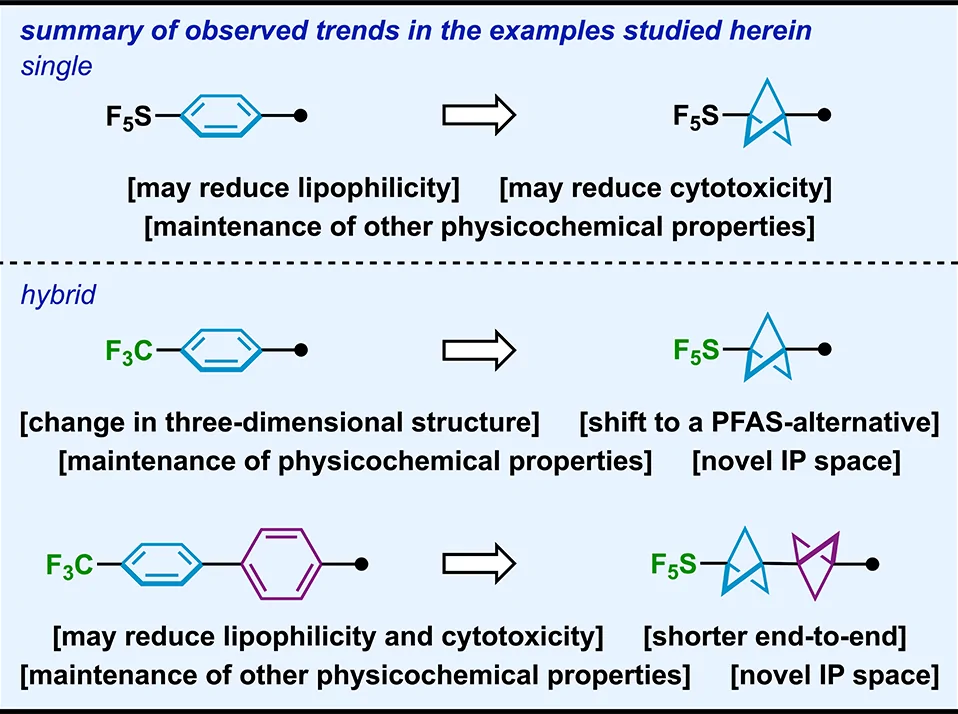
Condensed summary of effects of single and multivariable (hybrid) bioisosteric replacements involving SF_5_-BCP and SF_5_-[*2*]­staffane motifs observed in this study.

Over the course of these studies, we also identified a unique degradation pathway for SF_5_-[*2*]­staffanes upon prolonged storage in DMSO, with important implications for assay design and data interpretation. This observation prompted experimental and computational (DFT) studies that led to elucidation of the decomposition mechanism, inspired development of a programmed degradation strategy to expand the repertoire of SF_5_-BCP building blocks, and motivated a campaign to explore the broader chemical stability of SF_5_-[*2*]­staffanes under various reaction conditions, including those most frequently employed in medicinal chemistry. These efforts established the first comprehensive suite of SF_5_-[*2*]­staffane building blocks—the potential utility of which conceivably spans well beyond the fields of medicinal and agrochemistry.

## Supplementary Material



## Notes

Experimental procedures, characterization data, NMR spectra, crystallographic data, computational details, and *in vitro* ADME profiling details (PDF)
