# Supplementary material for: Synthesis, Evaluation, and Context for Applications of SF5‑Containing [1.1.1]Bicyclopentane and [2]Staffane “Hybrid Bioisosteres”
Source: J Am Chem Soc. 2026 Sep 18;148(38):41302–21. doi: 10.1021/jacs.6c12832 (PMC13632559; doi:10.1021/jacs.6c12832)
Supplement: Supplementary file 1 [file ja6c12832_si_001.pdf]

Synthesis, Evaluation, and Context for Applications of SF_5-Containing [1.1.1]Bicyclopentane and [*2*]Staffane "Hybrid Bioisosteres"

Jón Atiba Buldt,[a] Masiel M. Belsuzarri,[a] Ansh Hiten Patel,[a] Yannick Kraemer,[a] Tyson Vu,[a] Riddhiman Banerjee,[a] Jake Anthony Olvera,[a] Dean Tantillo,[a,*] Lauren M. Holder,[b,*] Cody Ross Pitts[a,*]

[a] Department of Chemistry, University of California, Davis, Davis, California 95616, United States
[b] Novartis BioMedical Research, Emeryville, California 94608, United States

Table of Contents

General Information 7
Synthesis of SF_5Cl 8
Synthesis of [1.1.1]Propellane (3) 10
Synthesis Overview of SF_5-BP897 (7) and Derivatives 12
Synthesis of SF_5-BP897 and Derivatives 13
Compound S-1 13
Compound S-2 14
Compound 7 15
Compound 9 16
Compound 25 17
Compound 26 18
Compound 27 19
Synthesis Overview of RN-1665 (10) and SF_5–BCP Derivative (12) 20
Synthesis of RN-1665 (10) and Derivative (12) 21
Compound S-3 21
Compound S-4 22
Compound 10 23
Compound 12 24
Synthesis Overview of Benzpyrimoxan (13) and SF_5–BCP Derivative (16) 25
Synthesis of Benzpyrimoxan (13) and SF_5–BCP Derivative (16) 26
Compound S-5 26
Compound 15 27

Compound S-6 .... *28*
Compound 13 .... *29*
Compound S-7 .... *30*
Compound 16 .... *31*
Synthesis Overview of Tecovirimat (17) and SF_5–BCP Derivative (20) *32*
Synthesis of Tecovirimat (17) and SF_5–BCP Derivative (20) *33*
Compound 19 .... *33*
Compound S-8 .... *34*
Compound 17 .... *35*
Compound 20 .... *36*
Synthesis Overview of SF_5–[2]staffane Darapladib Derivative (30) *36*
Synthesis of SF_5–[2]staffane Darapladib Derivative (30) *38*
Compound S-9 .... *38*
Compound S-10 .... *39*
Compound 30 .... *40*
Synthesis Overview of OSU-T315 (31) *41*
Compound S-11 .... *42*
Compound S-12 .... *43*
Compound S-13 .... *44*
Compound S-14 .... *45*
Compound 31 .... *46*
Synthesis Overview of SF_5–[2]staffane OSU-T315 Derivative (33) *47*
Compound S-15 .... *48*
Compound S-16 .... *49*
Compound S-17 .... *50*
Compound S-18 .... *51*
Compound 33 .... *52*
Synthesis Overview of SF_5–[2]staffane PROT inhibitor Derivatives (37–40) *53*
Compound S-19 .... *54*
Compound 34 .... *55*
Compound 37 .... *56*

Compound 38 .... *57*
Compound S-20 .... *58*
Compound 35 .... *59*
Compound 39 .... *60*
Compound S-21 .... *61*
Compound 36 .... *62*
Compound 40 .... *63*
Syntheses of Compounds 42–44 for Decomposition Study *64*
Compound 42 .... *64*
Compound 43 .... *65*
Compound 44 .... *66*
Solvent Stability Experiments *67*
Thermal Stability Experiments *73*
Aqueous Stability Experiments *77*
Compounds 38-41 Toward Programmed Decomposition of SF_5-[2]staffanes *80*
Compound 45 .... *80*
Compound 46 .... *81*
Compound 47 .... *82*
Compound 48 .... *83*
Procedures for SF_5–BCP and SF_5–[2]staffane Building Blocks *84*
Compound 2 .... *84*
Compound S-22 .... *85*
Compound 4 .... *86*
Compound 14 .... *87*
Compound 18 .... *88*
Compound 5 .... *89*
Compound 6 .... *90*
Compound S-23 .... *91*
Compound 49 .... *92*
Compound S-24 .... *93*
Compound S-25 .... *94*

Compound S-26 .... *95*
Compound 50 .... *96*
Compound 51 .... *97*
Compound 32 .... *98*
Compound S-27 .... *99*
Saponification of Compound S-27 .... *100*
Compound 52 .... *101*
Compound 53 .... *102*
Compound 54 .... *103*
Compound 55 .... *104*
Compound 56 .... *105*
Compound 57 .... *106*
Compound 58 .... *107*
Compound 59 .... *108*
Compound 60 .... *109*
Compound 61 .... *110*
Compound 62 .... *111*
Compound 63 .... *112*
Compound 64 .... *113*
Compound 65 .... *114*
Compound 66 .... *115*
Compound 67 .... *116*
Compound S-28 .... *117*
Compound 68 .... *118*
Compound 69 .... *119*
Compound S-29 .... *120*
Compound S-30 .... *121*
Compound 70 .... *122*
Compound 71 .... *123*
Compound S-31 .... *124*
Compound 72 .... *125*

Compound S-32 .... *126*
Compound 73 .... *127*
Compound 74 .... *128*
Compound 75 .... *129*
Compound 76 .... *130*
Compound S-33 .... *131*
Compound 77 .... *132*
Compound S-34 .... *133*
Compound S-35 .... *134*
Optimization Tables *135*
Optimization for Compound 4 .... *135*
Optimization for Compound 5 .... *137*
Initial Optimization for Compound 39 (in Ethanol) *138*
Optimization for Compound 39 (in t-BuOH) *139*
First-Generation Synthesis Route Toward Tecovirimat and Derivative *140*
Compound S-36 .... *141*
Compound S-37 .... *142*
Compound 17 .... *143*
Compound 20 .... *144*
First-Generation (Unsuccessful) Synthesis Route Toward OSU-T315 Derivative *145*
Compound S-38 .... *146*
Compound S-39 .... *147*
Compound S-40 .... *148*
Compound 33 .... *149*
Additional Attempted Transformations Toward Remote Arene Functionalization *150*
Compound S-41 .... *150*
Compound S-42 .... *151*
Compound S-43 .... *152*
Compound S-44 .... *153*
Compound S-45 .... *154*
Compound S-46 .... *155*

Compound S-47 .... *156*
Compound S-48 .... *157*
Compound S-49 .... *158*
Compound S-50 .... *159*
Compound S-51 .... *160*
Compound S-52 .... *161*
Compound S-53 .... *162*
Unsuccessful Transformations Regarding Synthesis of Building Blocks to Explore Remote Arene Functionalization *163*
Summary of in vitro ADME Profiling *168*
X-ray Crystallography *174*
Computational Data *192*
NMR Spectra *201*
References *543*

General Information

Unless otherwise stated, all reactions were carried out strictly under anhydrous conditions and under N_2 or Ar atmosphere and were stirred with Teflon-coated magnetic stir bars. Liquid reagents and solvents were transferred *via* syringe using standard Schlenk techniques. Dichloromethane (CH_2Cl_2), tetrahydrofuran (THF), diethyl ether (Et_2O) and toluene (PhMe) were obtained from a solvent purification system directly before use. Methanol (MeOH), acetonitrile (MeCN), *n*-pentane, *n*-hexane, dimethyl sulfoxide (DMSO), dimethylformamide (DMF), and ethyl acetate (EtOAc) were purchased in sealed bottles containing molecular sieves and were used as received. All other solvents were used as received unless otherwise stated. Spray-dried KF was always weighed out under glovebox atmosphere. All NMR data were collected on either a 300, 400, 600, or 800 MHz spectrometer. For ^{19}F NMR yield determination, α,α,α-trifluorotoluene (TFT) or fluorobenzene was introduced after each reaction as an internal standard, and the relaxation delay (d1) was increased to 30 s during data collection. The ^{1}H, ^{13}C, and ^{19}F NMR chemical shifts are given in parts per million (δ) and calibrated to either residual solvent or TMS signal (^{1}H and ^{13}C), α,α,α-trifluorotoluene (TFT) (δ = –62.61 ppm in $CDCl_3$), or fluorobenzene (δ = –112.96 in $CDCl_3$).[1,2] NMR data are reported in the following fashion: chemical shift (multiplicity (s = singlet, d = doublet, q = quartet, p = pentet, sept = septet, dt = doublet of triplets, dd = doublet of doublets, tt = triplet of triplets, ttp = triplet of triplet of pentets, m = multiplet), integration, coupling constants (Hz)). Infrared spectroscopy (IR) data were collected on a Bruker Tensor 27 FT-IR spectrometer with ATR-IR attachment. IR signals are reported in reciprocal centimeters (cm^{-1}) and are rounded to the nearest 1 cm^{-1}. High-resolution mass spectrometry (HRMS) data was collected on a Thermo Q-Exactive HF. Melting point data was collected on a Stanford Research Systems MPA120EZ-Melt automated melting point system.

Synthesis of SF_5Cl

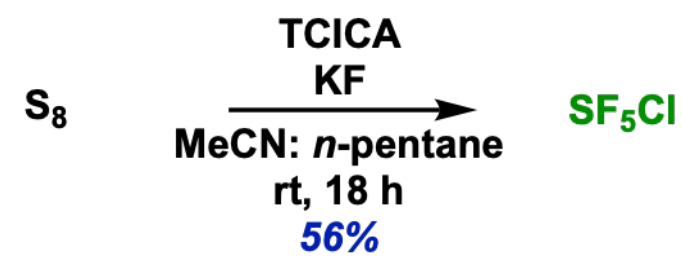

Synthesis of SF_5Cl was adapted from a known literature procedure.[3] An oven-dried 350 mL glass pressure vessel equipped with a large stir bar was introduced to a nitrogen-filled glove box. Under inert atmosphere, trichloroisocyanuric acid (33.7 g, 145.0 mmol, 4.5 equiv.) and spray-dried potassium fluoride (16.7 g, 287.4 mmol, 9.0 equiv.) were added, followed by anhydrous MeCN (80 mL) and *n*-pentane (180 mL). The vessel was wrapped in aluminum foil and sealed with a Teflon screwcap; the mixture then was vigorously stirred under inert atmosphere for 15 to 20 min. to break up solid aggregates. Then, sulfur (1.03 g, 32.13 mmol, 1.0 equiv.) was added, and the vessel was sealed, removed from the glovebox, and quickly transported to a fume hood. The mixture was stirred vigorously at room temperature for 18 h (~750 rpm). Upon reaction completion, stirring was ceased, and the mixture was allowed to settle for approximately 30 min., allowing the MeCN and *n*-pentane layers to fully separate. The vessel was carefully opened, and the MeCN and *n*-pentane layers were decanted into an oven-dried separatory funnel. The MeCN layer and any residual solids were discarded, and the SF_5Cl solution in *n*-pentane was transferred to a Schlenk flask under Ar atmosphere *via* syringe. Typically, at this scale, approximately 170 mL of the stock solution was obtained with a concentration ranging from 0.10–0.15 M as determined by ^{19}F NMR analysis. *Caution: Although working with a dilute solution may not pose the same hazards as working with SF_5Cl in pure gaseous form, SF_5Cl is toxic, and the solution should only be handled in a well-ventilated fume hood.*

Concentration determination:
An oven-dried 3 mm NMR tube was fitted with a septum cap, evacuated, and backfilled with Ar. Subsequently, 10 μL of α,α,α-trifluorotoluene (TFT) was added, followed by 0.4 mL anhydrous $CDCl_3$ and a 0.2 mL aliquot of the SF_5Cl stock solution. During data collection the relaxation delay (d1) was set to 30 sec and O1P to +30 ppm.

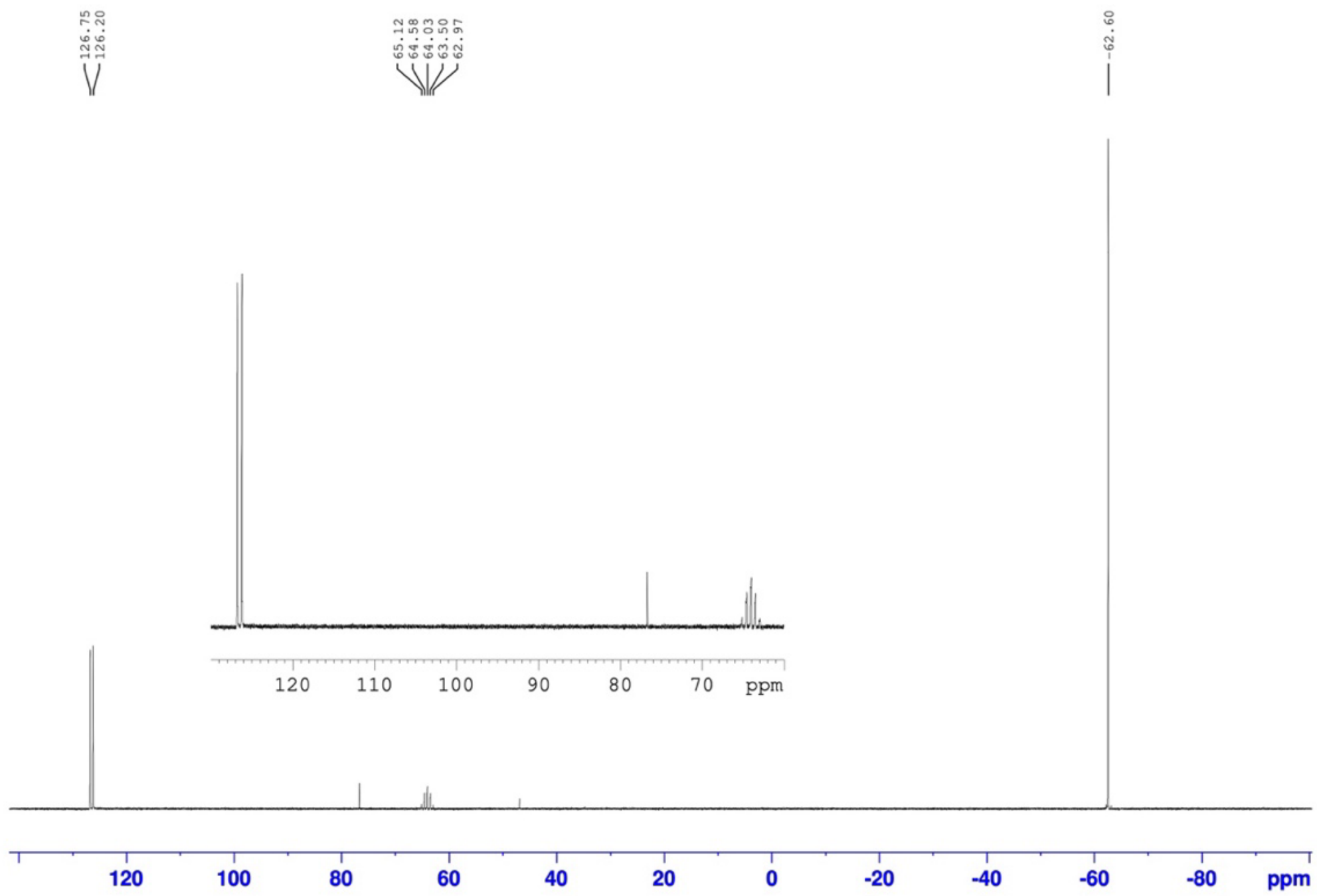

Figure S1: Typical ^{19}F NMR spectrum of SF_5Cl stock solution in *n*-pentane with TFT as an internal standard.

Synthesis of [1.1.1]Propellane (3)

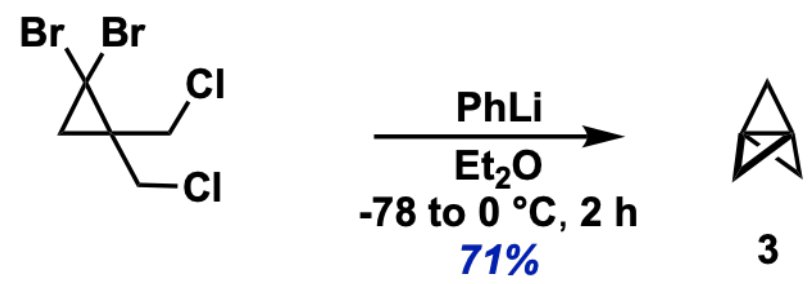

The synthesis of [1.1.1]propellane was adapted from a known literature procedure.[4] An oven-dried 1 L three-neck round bottom flask equipped with a stir bar was charged with 1,1-dibromo-2,2-bis(chloromethyl)cyclopropane (56.3 g, 190 mmol, 1.0 equiv.). The flask was evacuated and backfilled with Ar. Then, 190 mL of anhydrous Et_2O was added *via* syringe. The solution was cooled to –78 °C leading to the formation of a precipitate. Phenyllithium (PhLi) (1.8 M in *n*-Bu_2O, 200 mL, 380 mmol, 2.0 equiv.) was added dropwise. Upon addition of PhLi, the reaction mixture turned brown, and the precipitate dissolved. The reaction mixture was allowed to stir at –78 °C for 20 min, and the reaction vessel was then transferred to an ice bath and stirred at 0 °C for 2 h. After 2 h, a short path distillation head connected to a 250 mL 2-neck receiver flask was introduced. The system was placed under dynamic vacuum for 2 min, then static vacuum was maintained. (Vigorous bubbling was observed as [1.1.1]propellane in Et_2O is distilled into receiving flask. Excessive bubbling can result in the crude reaction mixture transferring over.) Vacuum may be reapplied periodically to increase the rate of the distillation. After the distillation is complete, the resulting solution of [1.1.1]propellane in Et_2O was transferred *via* cannula to a Teflon-capped 250 mL Schlenk tube. *Note: the solution of [1.1.1]propellane can be stored over 3 Å molecular sieves in a freezer at –20 °C for multiple weeks without noticeable decomposition.*

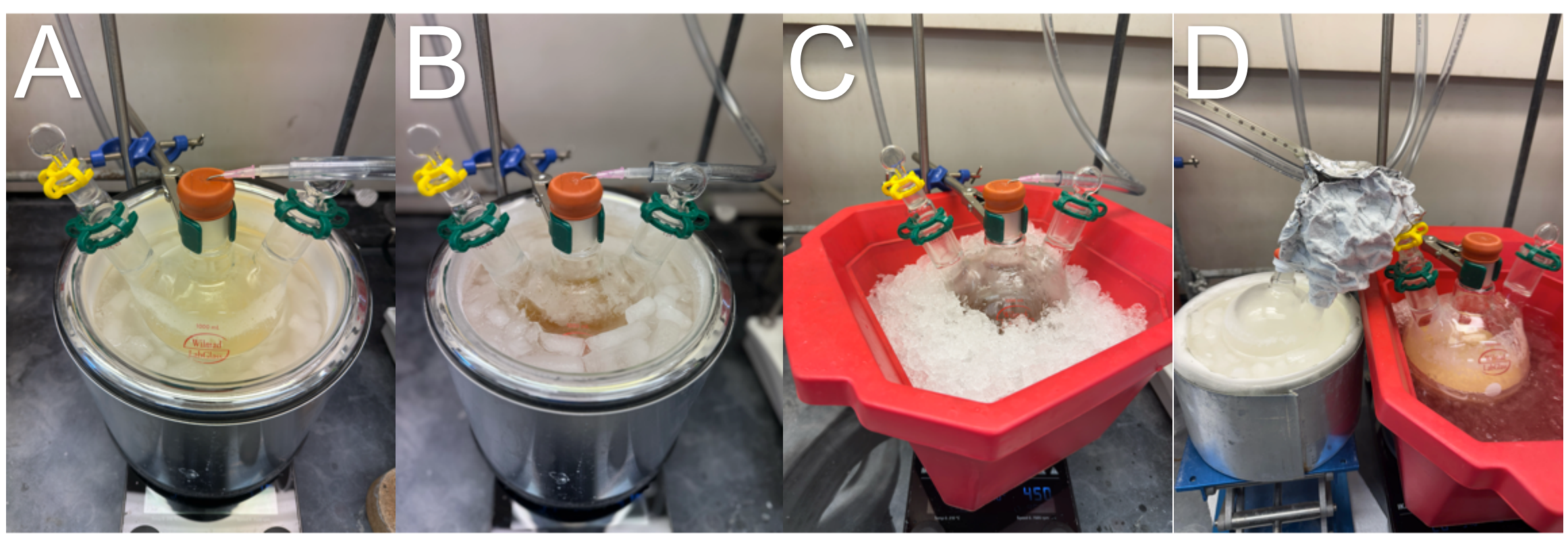

Figure S2. A: Setup for 200 mmol scale synthesis of [1.1.1]propellane; 1,1-dibromo-2,2-bis(chloromethyl)cyclopropane was dissolved in Et_2O and cooled to –78 °C, prior to PhLi addition. **B:** Following PhLi addition, the off-white precipitate completely dissolves, and the reaction mixture changes to a dark-orange/brown color. **C:** The reaction vessel was transferred to an ice-bath to keep it at 0 °C for 2 h. **D**: Distillation setup following reaction completion. The glass stopper on the left was removed and replaced with a short-path distillation head attached to a two-neck 250 mL receiving flask. The distillation head was wrapped in aluminum foil that was filled with finely crushed dry ice.

Concentration determination:

An internal standard stock solution was prepared by dissolving 1,3,5-trimethoxybenzene (10–20 mg) in 0.5 mL anhydrous $CDCl_3$. Then, 0.1 mL of the internal standard stock solution was added to an NMR tube. Then, 0.3 mL anhydrous $CDCl_3$ was added, followed by a 0.2 mL aliquot of the [1.1.1]propellane solution. During data collection, the relaxation delay (d1) was set to 10 s, with 8 scans and 0 dummy scans.

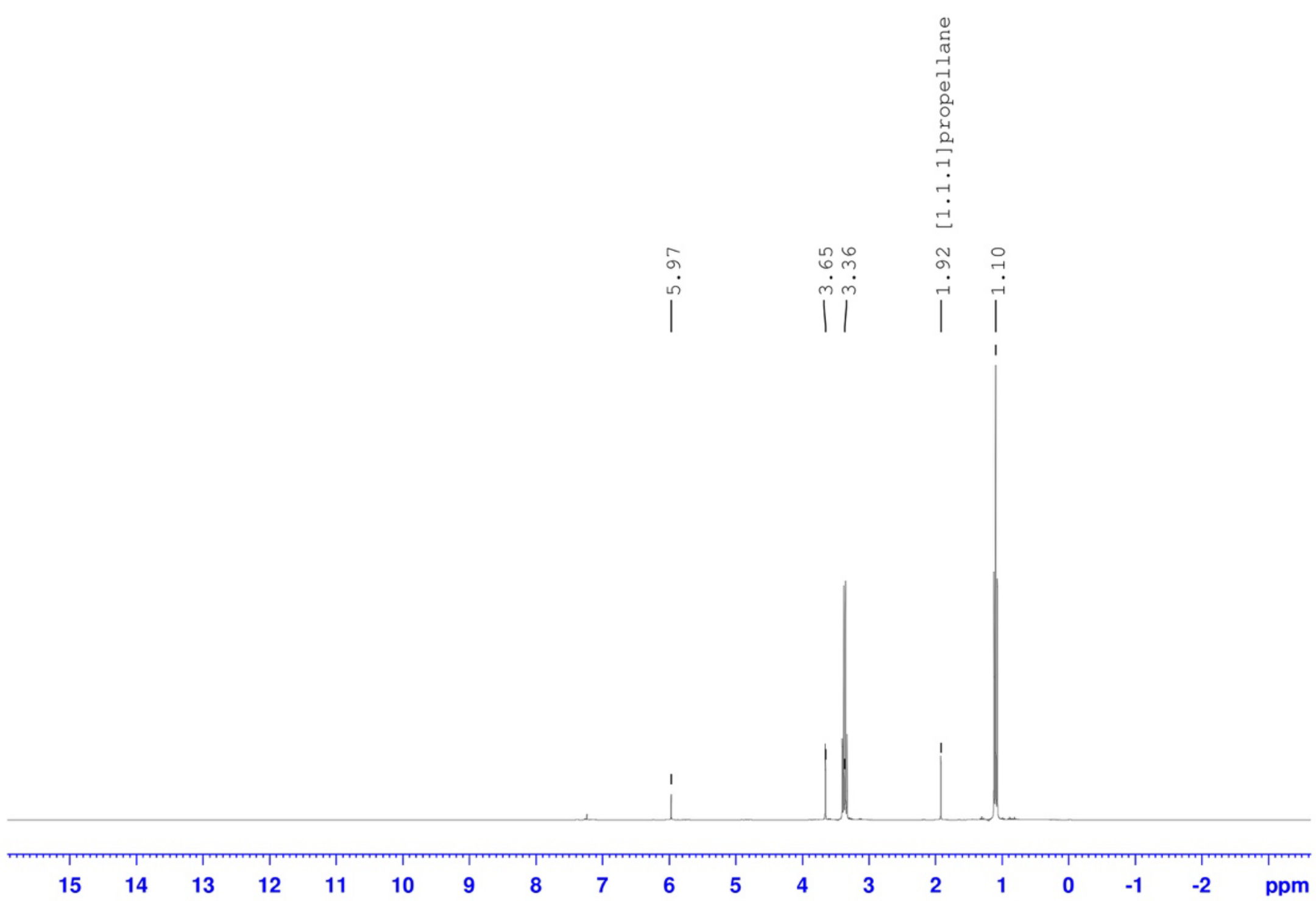

Figure S3: Typical ^1H NMR spectrum of [1.1.1]propellane stock solution in Et_2O with 1,3,5-trimethoxybenzene as the internal standard.

Synthesis Overview of SF_5-BP897 (7) and Derivatives

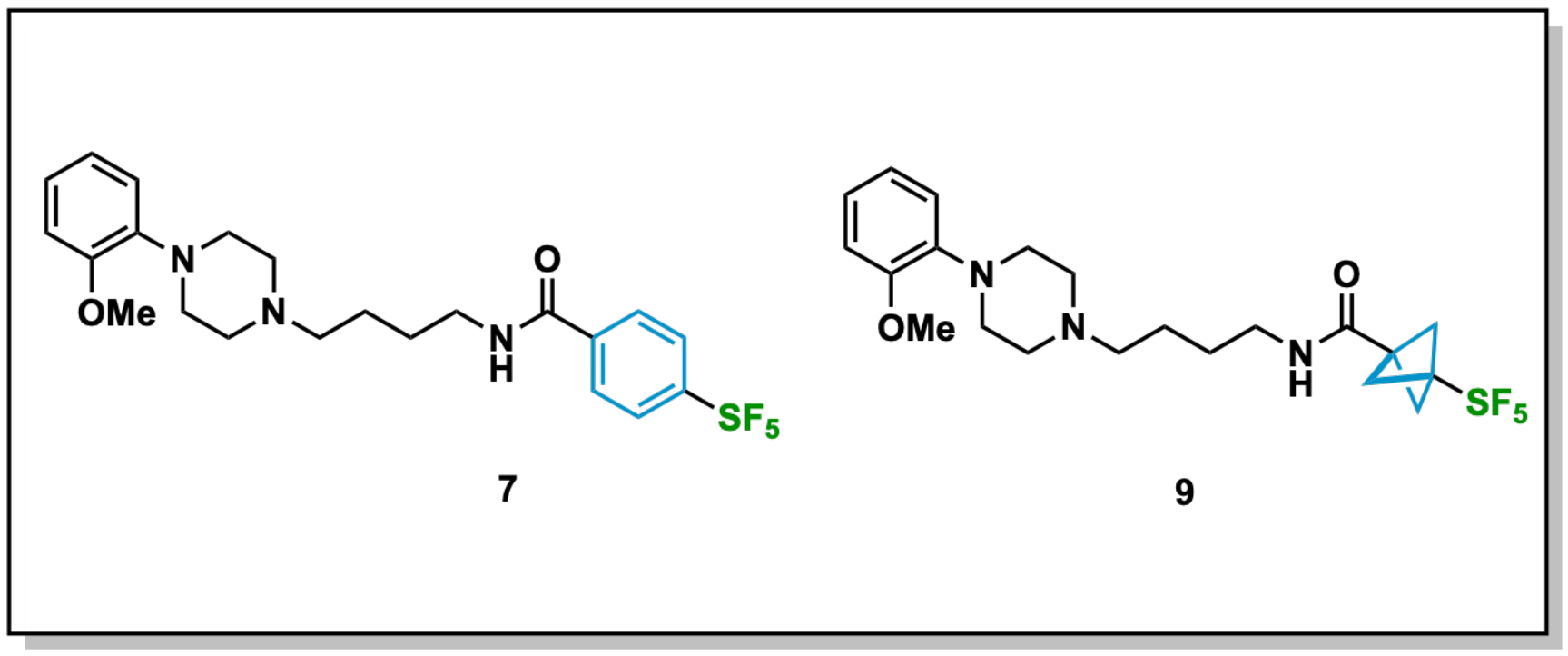

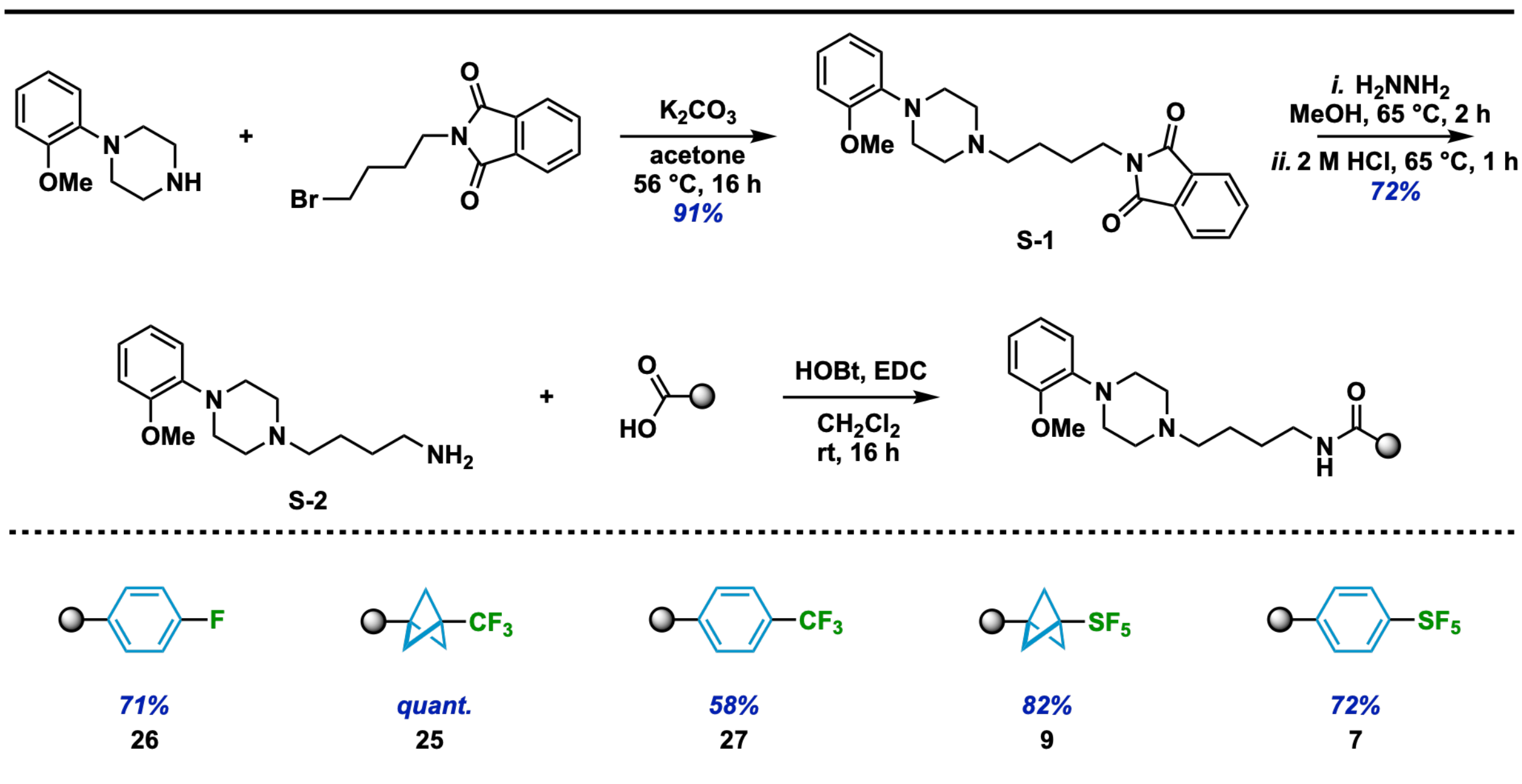

Scheme S1: Synthesis overview of SF_5-BP897 (**7**) and various derivatives.[5] See *pages S12-S18 for detailed procedures and corresponding characterization data.*

Synthesis of SF_5-BP897 and Derivatives

Compound S-1

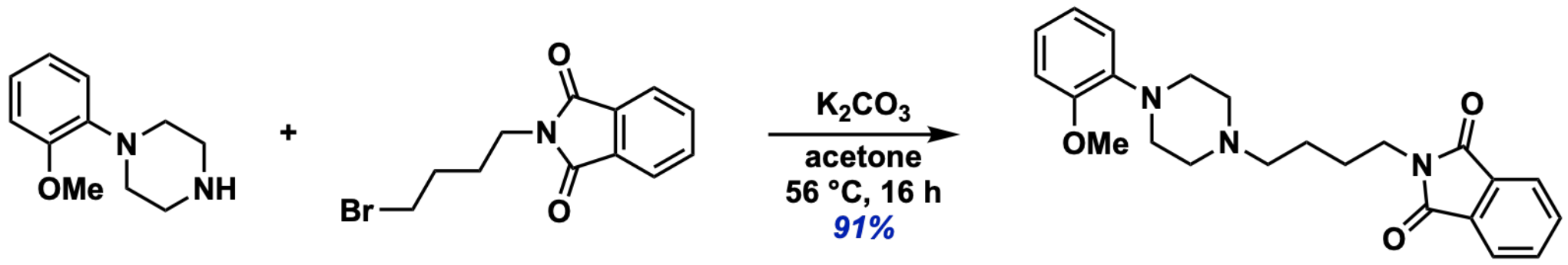

The synthesis of compound **S-1** was adapted from a known literature procedure.[5] To an oven-dried round bottom flask equipped with a stir bar was added 2-(4-bromobutyl)isoindoline-1,3-dione (0.205 g, 1.04 mmol, 1.0 equiv.). Acetone (5.0 mL) was added, then the reaction mixture was stirred. Subsequently, 1-(2-methoxyphenyl)piperazine (0.354 g, 1.25 mmol, 1.2 equiv.) was added followed by K_2CO_3 (0.862 g, 12.5 mmol, 6.0 equiv.). The reaction was sealed with a septum, and the mixture was stirred at 56 °C for 16 h. Upon reaction completion, the reaction was cooled to room temperature. The inorganic salts were removed *via* filtration through Celite, and the filtrate was concentrated. The crude mixture was extracted with EtOAc (3 x 10 mL). The combined organic layers were washed with brine (3 x 25 mL), dried over anhydrous Na_2SO_4, filtered through Celite, and concentrated. The crude residue was purified *via* column chromatography on silica gel eluting with 97:3 CH_2Cl_2:MeOH to afford a dark yellow oil (0.371 g, 91%).

2-(4-(4-(2-methoxyphenyl)piperazin-1-yl)butyl)isoindoline-1,3-dione (**S-1**):
R_f: 0.14 (97:3 CH_2Cl_2:MeOH), vis: UV (254 nm).
^{1}H (300 MHz, DMSO-*d_6*): δ = 7.87-7.79 (m, 4H), 6.94-6.82 (m, 4H), 3.75 (s, 3H), 3.61-3.56 (t, 2H, $^3J_{H-H}$ = 6.9 Hz), 2.90 (s, 4H), 2.45 (s, 4H), 2.33-2.28 (t, 2H, $^3J_{H-H}$ = 7.2 Hz), 1.66-1.57 (m, 2H,), 1.49-1.40 (m, 2H).
^{1}H (300 MHz, $CDCl_3$): δ = 7.87-7.80 (m, 2H), 7.73-7.67 (m, 2H), 7.01-6.83 (m, 4H), 3.85 (s, 3H), 3.75-3.70 (t, 2H, $^3J_{H-H}$ = 7.0 Hz), 3.08 (s, 4H), 2.64 (s, 4H), 2.46-2.41 (t, 2H, $^3J_{H-H}$ = 7.4 Hz), 1.78-1.69 (m, 2H), 1.49-1.40 (m, 2H).
^{13}C{^{1}H} (101 MHz, DMSO-*d_6*): δ = 167.9, 151.9, 141.2, 134.3, 131.6, 122.9, 122.2, 120.8, 117.8, 111.8, 57.3, 55.2, 53.0, 49.0, 37.3, 25.9, 23.6.
ATR-IR (ν_{max}): 2940, 2814, 1707, 1239, 718 cm^{-1}.
HRMS (*m/z*): (ESI) calc'd for $C_{23}H_{28}N_3O_3^+$ [M-H]$^+$: 394.2125, found 394.2121.

Compound S-2

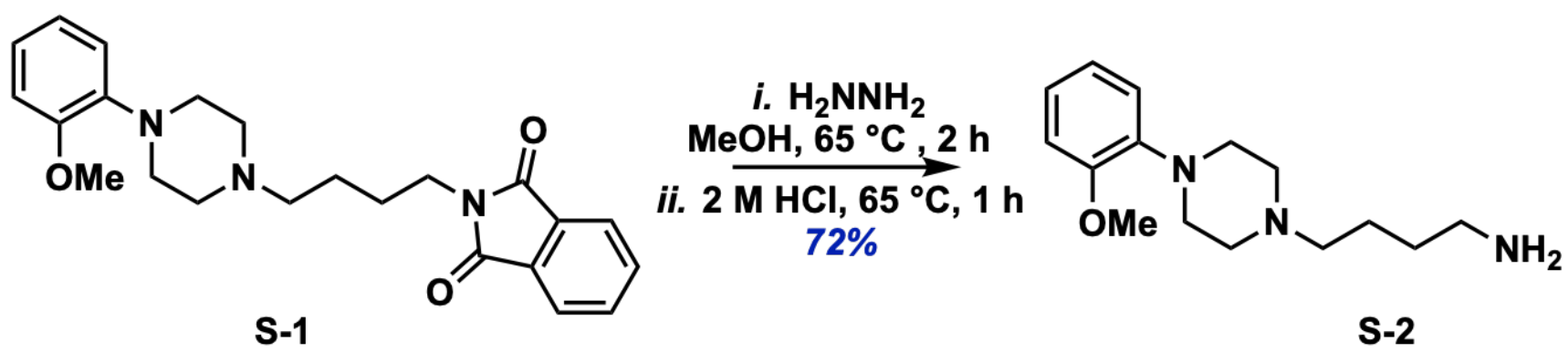

The synthesis of compound **S-2** was adapted from a known literature procedure.[5] To an oven-dried round bottom equipped with a stir bar was added compound **S-1** (1.10 g, 2.82 mmol, 1.0 equiv.) and MeOH (30 mL). The reaction mixture was stirred. Subsequently, H_2NNH_2•H_2O (0.50 mL) was added, and the reaction mixture was heated to 65 °C for 2 h. After 2 h, 2 M HCl (5.0 mL) was added dropwise; the reaction mixture was stirred at 65 °C for an additional 1 h. The reaction mixture was then cooled to room temperature, filtered through Celite, and washed with MeOH (10 mL). The filtrate was concentrated and to it was added 2 M NaOH (20 mL). The crude mixture was extracted with EtOAc (3 x 10 mL). The combined organic layers were washed with brine (3 x 25 mL), dried over anhydrous Na_2SO_4, filtered through Celite, and concentrated. The crude reside was purified *via* column chromatography on silica gel eluting with 92:8 CH_2Cl_2:7 M NH_3 in MeOH to afford a yellow solid (0.536 g, 72%).

4-(4-(2-methoxyphenyl)piperazin-1-yl)butan-1-amine (**S-2**):
R_f: 0.18 (92:8 CH_2Cl_2:7 M NH_3 in MeOH), vis: UV (254 nm).
m.p: 99.8–103.3 ºC.
^{1}H (300 MHz, DMSO-*d$_6$*): δ = 6.92-6.86 (m, 4H), 3.76 (s, 3H), 3.21 (s, 2H), 2.94 (s, 4H), 2.48 (s, 4H), 2.32-2.27 (t, 2H $^3J_{H-H}$ = 7.3 Hz), 1.50- 1.31 (m, 4H).
^{1}H (300 MHz, $CDCl_3$): δ = 7.01-6.84 (m, 4H), 3.87 (s, 3H), 3.10 (s, 4H), 2.74-2.71 (t, 2H), 2.65 (s, 4H), 2.44-2.40 (t, 2H), 1.61-1.44 (m, 4H).
^{13}C{^{1}H} (76 MHz, $CDCl_3$): δ = 152.2, 141.3, 122.8, 120.9, 118.1, 111.1, 58.4, 55.3, 53.4, 50.6, 41.5, 30.7, 24.2.
ATR-IR (ν_{max}): 3364, 2913, 1500, 1224, 737 cm^{-1}.
HRMS (*m/z*): (ESI) calc'd for $C_{15}H_{26}N_3O^+$ [M-H]$^+$: 264.2070, found 264.2076.

Compound 7

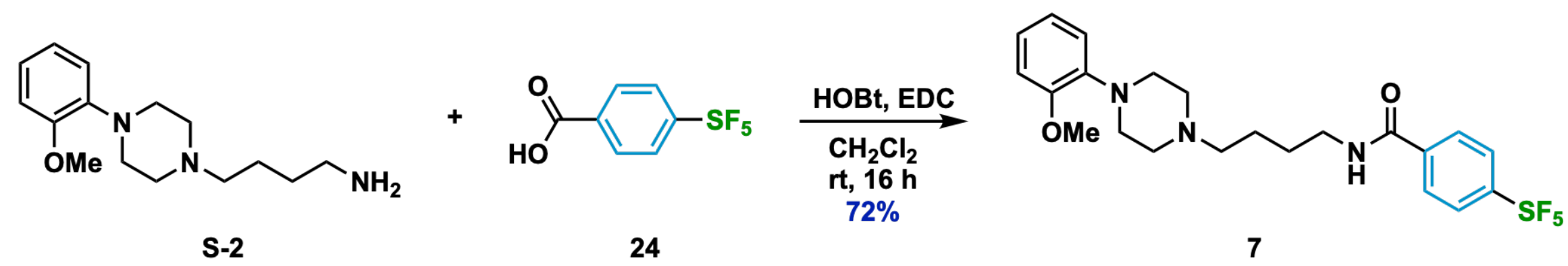

The synthesis of compound **7** was adapted from a known literature procedure.[5] To an oven-dried microwave vial equipped with a stir bar was added compound **S-2** (25.0 mg, 0.095 mmol, 1.0 equiv.) and compound **24** (26.0 mg, 0.104 mmol, 1.1 equiv.), followed by CH_2Cl_2 (1.25 mL). The reaction mixture was stirred. Subsequently, HOBt (16.0 mg, 0.104 mmol, 1.1 equiv.) and EDC (20.0 mg, 0.104 mmol, 1.1 equiv.) were added. The vial was sealed and stirred at room temperature. After 16 h, the reaction was quenched with sat. aq. $NaHCO_3$ (1 mL). The crude mixture was extracted with CH_2Cl_2 (3 x 10 mL). The combined organic layers were washed with brine (3 x 25 mL), dried over anhydrous Na_2SO_4, filtered through Celite, and concentrated. The crude reside was purified *via* preparatory TLC eluting with 95:5 CH_2Cl_2:MeOH to afford an off-white solid (33.5 mg, 72 %).

N-(4-(4-(2-methoxyphenyl)piperazin-1-yl)butyl)-4-(pentafluoro-λ^6-sulfanyl)benzamide (**7**):
R_f: 0.27 (95:5 CH_2Cl_2:MeOH), vis: UV (254 nm).
m.p: 112.2–114.5 ºC.
^{1}H (300 MHz, DMSO-*d*$_6$): δ = 8.76-8.72 (t, 1H, $^3J_{H-H}$ = 5.5 Hz), 8.02 (s, 4H), 6.96-6.84 (m, 4H), 3.76 (s, 3H), 3.33-3.27(m, 2H), 2.94 (s, 4H), 2.5 (s, 4H), 2.37-2.32 (m, 2H), 1.62-1.45 (m, 4H).
^{1}H (300 MHz, $CDCl_3$): δ = 7.86-7.79 (m, 4H), 7.10 (s, 1H), 7.03-6.83 (m, 4H), 3.85 (s, 3H), 3.53-3.47 (m, 2H), 3.02 (s, 4H), 2.64 (s, 4H), 2.50-2.46 (m, 2H), 1.75-1.66 (m, 4H).
^{19}F (283 MHz, DMSO-*d*$_6$): δ = 87.53-85.39 (p, 1F, $^2J_{F-F}$ = 152 Hz), 64.08-63.54 (d, 4F, $^2J_{F-F}$ = 151 Hz).
^{13}C{^{1}H} (101 MHz, DMSO-*d*$_6$): δ = 164.4, 154.4-154.1 (t, $^2J_{C-F}$ = 16.2 Hz), 151.9, 141.2, 138.2, 128.3, 125.9-125.9 (t, $^2J_{C-F}$ = 4.7 Hz), 122.31, 120.82, 117.84, 111.90, 57.56, 55.28, 54.90, 53.01, 50.02, 26.92, 23.75.
ATR-IR (ν_{max}): 3328, 2935, 2816, 2358, 1645, 1242, 843 cm^{-1}.
HRMS (*m/z*): (ESI) calc'd for $C_{22}H_{29}F_5N_3O_2S^+$ [M-H]$^+$: calculated 494.1895, found 494.1892.

Compound 9

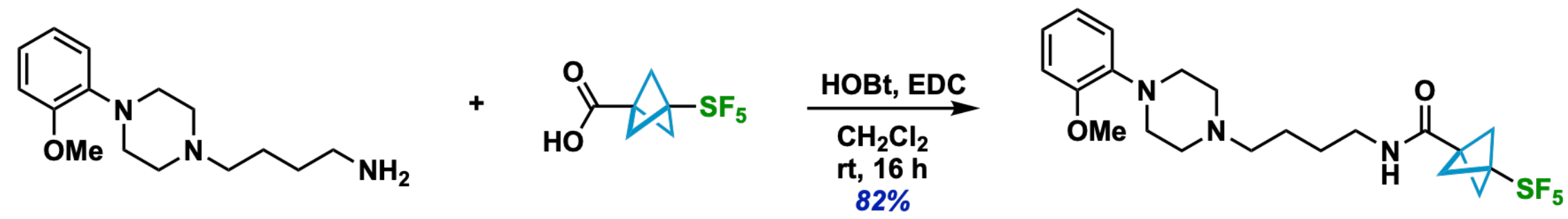

The synthesis of compound **9** was adapted from a known literature procedure.[5] To an oven-dried microwave vial equipped with a stir bar was added compound **S-2** (10.0 mg, 0.038 mmol, 1.0 equiv.) and compound **4** (9.80 mg, 0.042 mmol, 1.1 equiv.), followed by CH_2Cl_2 (0.5 mL). The reaction mixture was stirred. Subsequently, HOBt (6.40 g, 0.042 mmol, 1.1 equiv.) and EDC•HCl (8.00 mg, 0.042 mmol, 1.1 equiv.) were added. The vial was sealed with a cap with a septum using a crimper and stirred at rt for 16 h. The reaction was quenched with sat. aq. $NaHCO_3$ (1.0 mL). The crude mixture was extracted with CH_2Cl_2 (3 x 10 mL). The combined organic layers were washed with brine (3 x 25 mL), dried over anhydrous Na_2SO_4, filtered through Celite, and concentrated. The crude reside was purified *via* preparatory TLC eluting with 95:5 CH_2Cl_2:MeOH to afford an off-white solid (15.1 mg, 82%).

N-(4-(4-(2-methoxyphenyl)piperazin-1-yl)butyl)-3-(pentafluoro-λ^6-sulfanyl)bicyclo[1.1.1]pentane-1-carboxamide (**9**):
R_f: 0.15 (95:5 CH_2Cl_2:MeOH), vis: UV (254 nm).
m.p: 137.6–138.2 ºC.
^{1}H (300 MHz, $CDCl_3$): δ = 7.02-6.8 (m, 4H), 5.82 (s, 1H), 3.85 (s, 3H), 3.31-3.25 (m, 2H), 3.10 (s, 1H, 2.65 (s, 4H), 2.5 (s, 6H), 2.45-2.40 (2H), 1.59-1.54 (m, 4H).
^{19}F (283 MHz, $CDCl_3$): δ = 79.58-77.51 (p, 1F, $^2J_{F\text{-}F}$ = 146 Hz), 49.19-48.67 (d, 4F, $^2J_{F\text{-}F}$ = 146 Hz).
^{13}C{^{1}H} (101 MHz, $CDCl_3$): δ = 166.6, 152.4, 141.3, 123.1, 121.1, 118.3, 111.3, 58.1, 55.7-55.7(t, $^2J_{C\text{-}F}$ = 2.2 Hz), 55.4, 53.6, 50.7, 39.6, 35.4, 27.5, 24.3.
ATR-IR (ν_{max}): 3365, 2941, 2818, 1646, 1238, 800 cm^{-1}.
HRMS (*m/z*): (ESI) calc'd for $C_{21}H_{31}F_5N_3O_2S^+$ $[M\text{-}H]^+$: calculated 484.2052, found 484.2056.

Compound 25

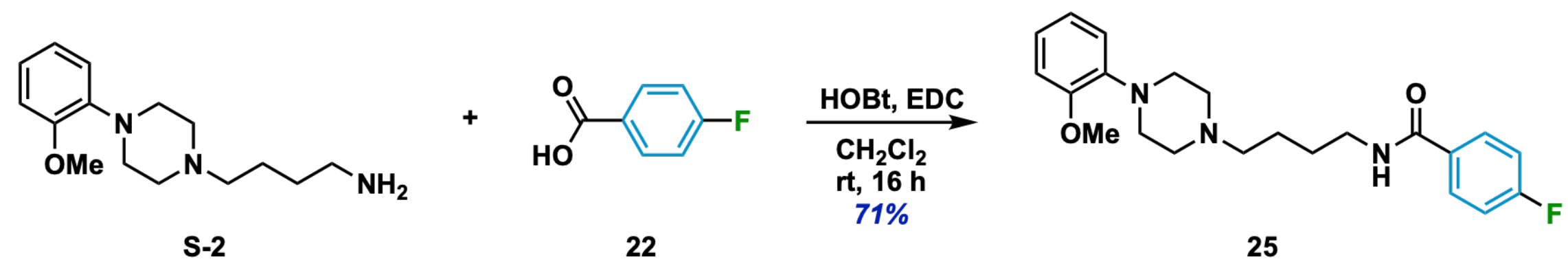

The synthesis of compound **25** was adapted from a known literature procedure.[5] To an oven-dried microwave vial equipped with a stir bar was added compound **S-2** (25.0 mg, 0.095 mmol, 1.0 equiv.) and compound **22** (14.6 mg, 0.104 mmol, 1.1 equiv.) followed by CH_2Cl_2 (1.25 mL). The reaction mixture was stirred. Subsequently, HOBt (15.9 mg, 0.104 mmol, 1.1 equiv.) and EDC (20.0 mg, 0.104 mmol, 1.1 equiv.) were added. The vial was sealed with a cap with a septum using a crimper, and the mixture was stirred at room temperature for 16 h. The reaction was then quenched with sat. aq. $NaHCO_3$ (1 mL). The crude mixture was extracted with CH_2Cl_2 (3 x 10 mL). The combined organic layers were washed with brine (3 x 25 mL), dried over anhydrous Na_2SO_4, filtered through Celite, and concentrated. The crude residue was purified *via* preparatory TLC eluting with 9:1 CH_2Cl_2:MeOH to afford an off-white solid (25.9 mg, 71%).

4-fluoro-N-(4-(4-(2-methoxyphenyl)piperazin-1-yl)butyl)benzamide (**25**):
R_f: 0.14 (9:1 CH_2Cl_2:MeOH), vis: UV (254 nm).
m.p: 127.4–128.7 °C.
^{1}H (300 MHz, $CDCl_3$): δ = 7.79- 7.74 (m, 2H), 7.10- 6.83 (m, 7H), 3.84 (s, 3H), 3.49-3.43 (m, 2H), 3.05 (s, 4H), 2.64 (s, 4H), 2.47-2.43 (t, 2H, $^2J_{H-H}$ = 6.9 Hz), 1.70-1.62 (m, 4H).
^{19}F (283 MHz, $CDCl_3$): δ = -108.58 – -108.68 (m, 1F).
^{13}C{^{1}H} (101 MHz, $CDCl_3$): δ = 166.8, 165.9-163.4 (d, $^1J_{C-F}$ = 250.82 Hz), 152.3, 141.2, 131.3-131.2 (d, $^4J_{C-F}$ = 2.92 Hz), 129.4-129.3 (d, $^3J_{C-F}$ = 8.86 Hz), 123.1, 121.1, 118.3, 115.7-155.5 (d, $^2J_{C-F}$ = 22.01 Hz), 111.3, 58.1, 55.5, 50.6, 40.1, 27.5, 24.5.
ATR-IR (ν_{max}): 3295, 2940, 2818, 2359, 1638, 1502, 1240, 749 cm^{-1}.
HRMS (*m/z*): (ESI) calc'd for $C_{22}H_{29}FN_3O_2^+$ $[M-H]^+$: calculated 386.2238, found 386.2239.

Compound 26

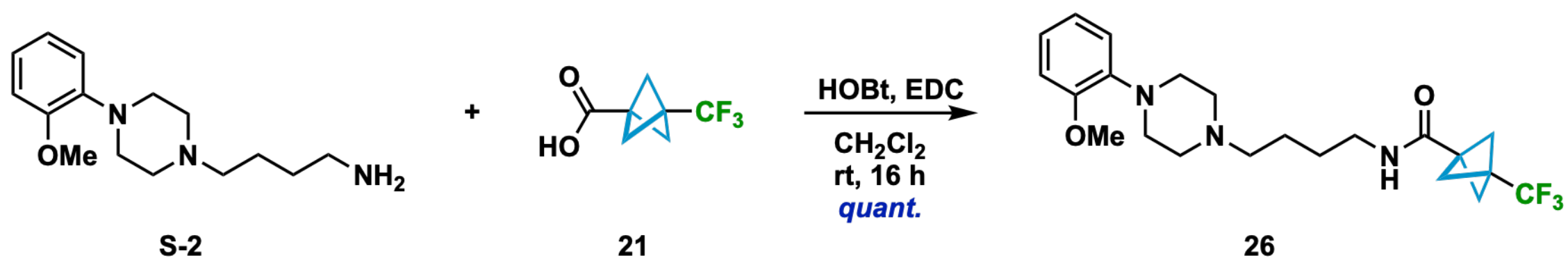

The synthesis of compound **26** was adapted from a known literature procedure.[5] To an oven-dried microwave vial equipped with a stir bar was added compound **S-2** (40.0 mg, 0.152 mmol, 1.0 equiv.) and compound **21** (30.0 mg, 0.167 mmol, 1.1 equiv.), followed by CH_2Cl_2 (2.0 mL). The reaction mixture was stirred. Subsequently, HOBt (26.0 mg, 0.167 mmol, 1.10 equiv.) and EDC (32.0 mg, 0.167 mmol, 1.1 equiv.) were added. The vial was sealed and the mixture was stirred at room temperature for 16 h. The reaction was quenched with sat. aq. $NaHCO_3$ (1 mL). The crude mixture was extracted with CH_2Cl_2 (3 x 10 mL). The combined organic layers were washed with brine (3 x 25 mL), dried over anhydrous Na_2SO_4, filtered through Celite, and concentrated. The crude residue was purified *via* preparatory TLC eluting with 9:1 CH_2Cl_2:MeOH to afford an off pink solid (40.0 mg, quant.).

N-(4-(4-(2-methoxyphenyl)piperazin-1-yl)butyl)-3-(trifluoromethyl)bicyclo[1.1.1]pentane-1-carboxamide (**26**):

R_f: 0.44 (9:1 CH_2Cl_2:MeOH), vis: UV (254 nm).

m.p.: 127.5–129.6 ºC.

^{1}H (300 MHz, $CDCl_3$): δ = 7.01-6.83 (m, 4H), 5.86 (s, 1H), 3.84 (s, 3H), 3.30-3.24 (m, 2H), 3.09 (s, 4H), 2.64 (s, 4H), 2.44-2.40 (m, 2H), 2.18 (s, 6H), 1.58- 1.53 (m, 4H).

^{19}F (283 MHz, $CDCl_3$): δ = -73.27 (s, 3F).

^{13}C{^{1}H} (76 MHz, $CDCl_3$): δ = 168.3, 152.3, 141.2, 128.0-117.0 (q, $^2J_{C\text{-}F}$ = 275.6 Hz), 123.0, 121.0, 118.2, 111.2, 58.0, 55.39, 53.5, 50.5, 49.5-49.4 (m), 39.3, 38.8-38.7 (m), 37.1-35.6 (q, $^2J_{C\text{-}F}$ = 39.09 Hz), 27.4, 24.2.

ATR-IR (ν_{max}): 3361, 2939, 2817, 1642, 1130, 738 cm^{-1}.

HRMS (*m/z*): (ESI) calc'd for $C_{22}H_{31}F_3N_3O_2^+$ [M-H]$^+$: calculated 426.2363, found 426.2355.

Compound 27

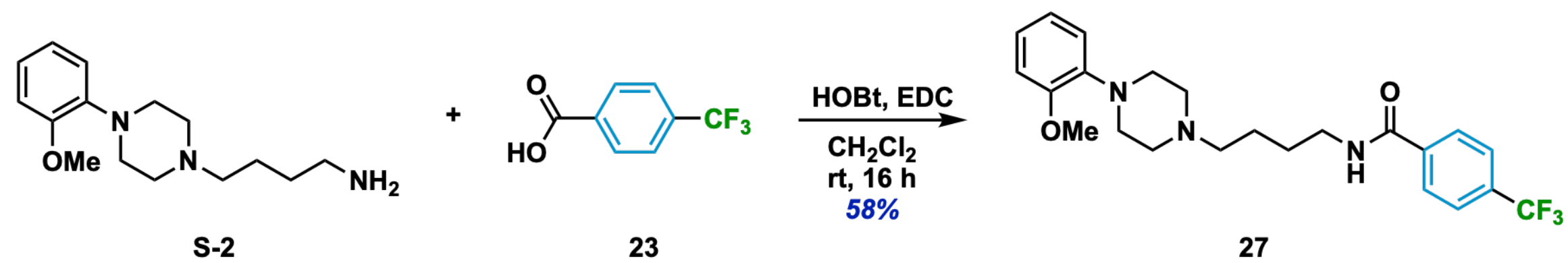

The synthesis of compound **27** was adapted from a known literature procedure.[5] To an oven-dried microwave vial equipped with a stir bar was added compound **S-2** (25.0 mg, 0.095 mmol, 1.0 equiv.) and compound **23** (19.9 mg, 0.104 mmol, 1.1 equiv.) followed by CH_2Cl_2 (1.25 mL). The reaction mixture was stirred. Subsequently, HOBt (15.9 mg, 0.104 mmol, 1.1 equiv.) and EDC (20.0 mg, 0.104 mmol, 1.1 equiv.) were added. The vial was sealed and the mixture was stirred at room temperature for 16 h. The reaction was then quenched with sat. aq. $NaHCO_3$ (1 mL). The crude mixture was extracted with CH_2Cl_2 (3 x 10 mL). The combined organic layers were washed with brine (3 x 25 mL), dried over anhydrous Na_2SO_4, filtered through Celite and concentrated. The crude residue was purified *via* preparatory TLC eluting with 9:1 CH_2Cl_2:MeOH to afford an off-white solid (24.0 mg, 58%).

N-(4-(4-(2-methoxyphenyl)piperazin-1-yl)butyl)-4-(trifluoromethyl)benzamide (**27**):
R_f: 0.19 (19:1 CH_2Cl_2:MeOH), vis: UV (254 nm).
m.p: 138.8–139.0 ºC.
^{1}H (300 MHz, $CDCl_3$): δ = 7.87-7.85 (d, 2H, $^3J_{H\text{-}H}$ = 8.2 Hz), 7.68-7.65 (d, 2H, $^3J_{H\text{-}H}$ = 8.2 Hz), 7.12 (s, 1H), 7.02-6.83 (m, 4H), 3.84 (s, 3H), 3.51-3.45 (m, 3H), 3.02 (s, 4H), 2.63 (s, 4H), 2.49-2.44 (t, 2H, $^3J_{H\text{-}H}$ = 6.7 Hz), 1.73-1.64 (m, 4H).
^{19}F (283 MHz, $CDCl_3$): δ = -62.87 (s, 3F).
^{13}C{^{1}H} (201 MHz, $CDCl_3$): δ = 166.6, 152.3, 141.1, 138.5, 133.3-132.8 (q, $^2J_{C\text{-}F}$ = 32.3 Hz), 127.6, 125.6-125.6 (q, $^3J_{C\text{-}F}$ = 3.8 Hz), 123.1, 121.0, 118.2, 111.2, 58.0, 55.4, 53.5, 50.5, 40.2, 27.4, 24.5.
ATR-IR (ν_{max}): 3296, 2941, 2817, 2361, 1638, 1327, 747 cm^{-1}.
HRMS (*m/z*): (ESI) calc'd for $C_{23}H_{29}F_3N_3O_2^+$ [M-H]$^+$: calculated 436.2206, found 436.2204.

Synthesis Overview of RN-1665 (10) and SF_5–BCP Derivative (12)

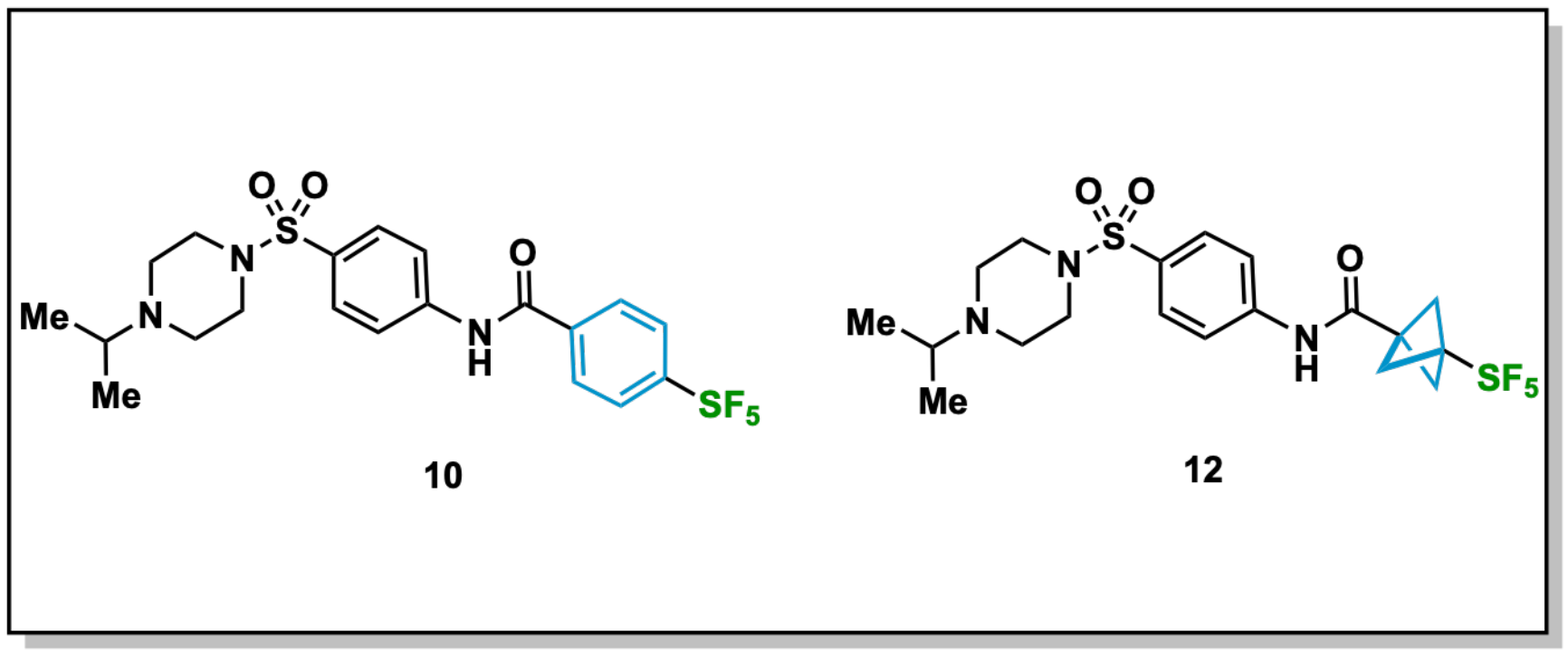

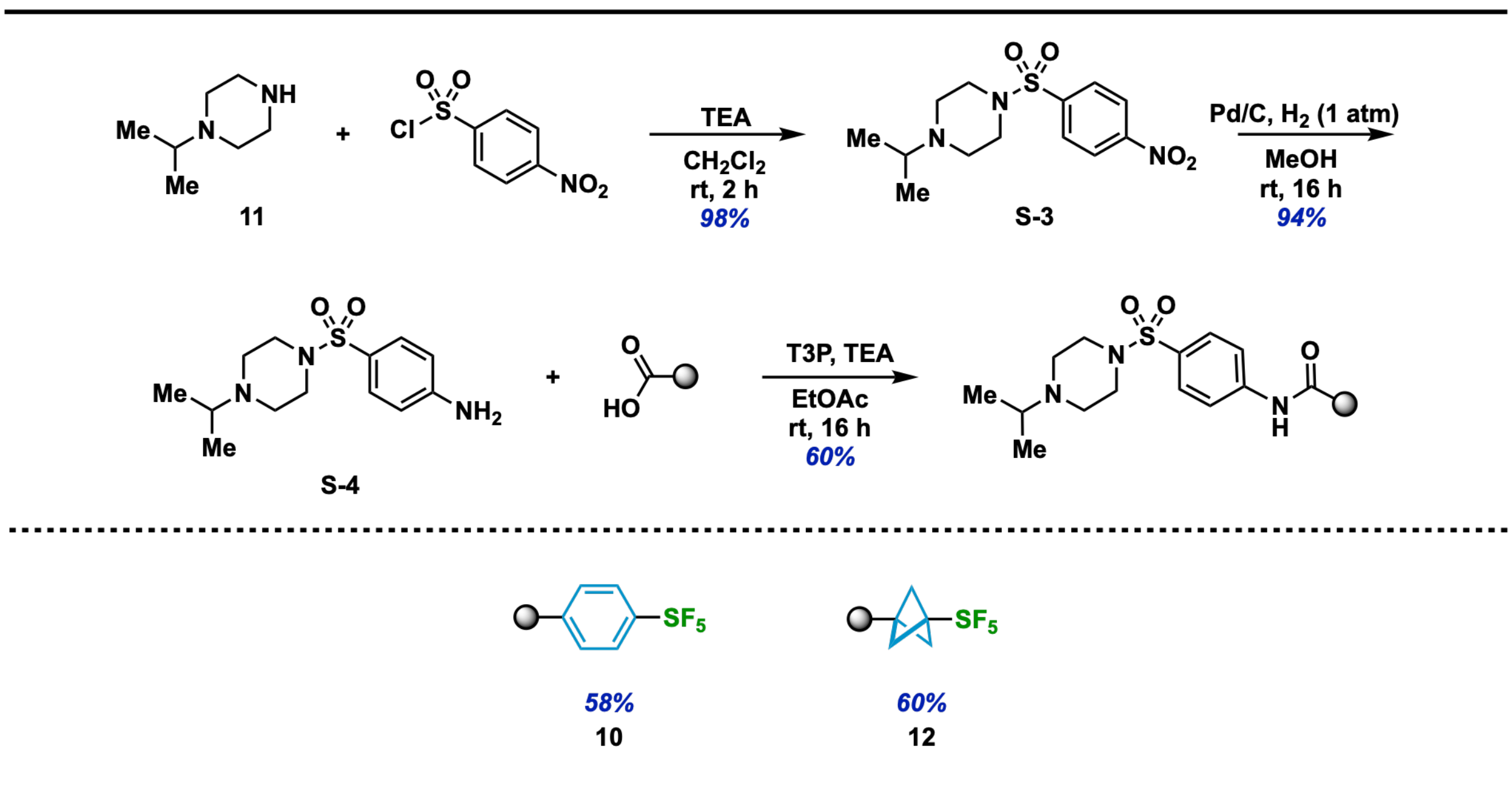

Scheme S2: Synthesis overview of RN-1665 (**10**) and its SF_5–BCP derivative (**12**).[6] *See pages S21-S24 for detailed procedures and corresponding characterization data.*

Synthesis of RN-1665 (10) and Derivative (12)

Compound S-3

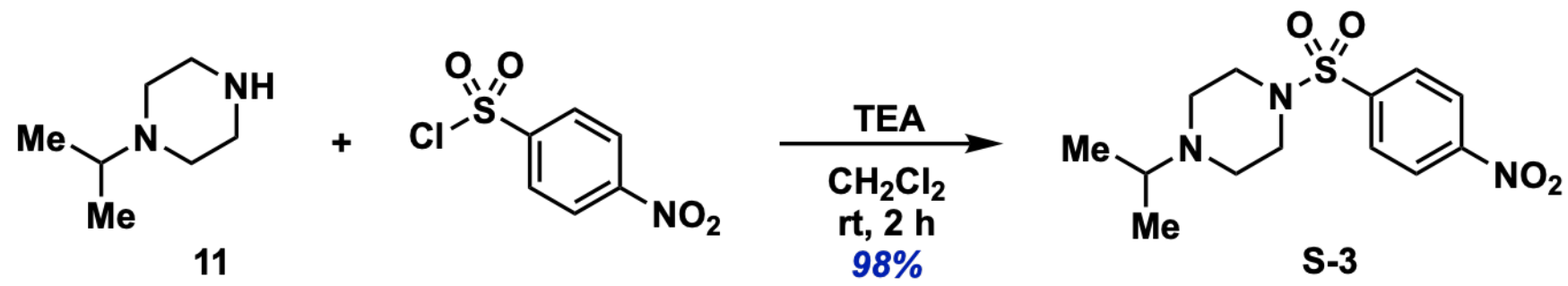

The synthesis of compound **S-3** was adapted from a known literature procedure.[6] To an oven-dried round bottom flask equipped with a stir bar was added *p*-nitrophenylsulfonyl chloride (0.461 g, 2.08 mmol, 1.0 equiv.). The vial was evacuated and backfilled with Ar three times. Anhydrous CH_2Cl_2 (15 mL), TEA (0.30 mL, 2.08 mmol, 1.0 equiv.), and 1-isopropylpiperazine (0.30 mL, 2.08 mmol, 1.0 equiv.) were added sequentially *via* syringe. The reaction mixture was stirred at room temperature for 2 h. Upon reaction completion, the reaction was diluted with CH_2Cl_2 (10 mL) and H_2O (10 mL). The aqueous layer was extracted with EtOAc (3 x 10 mL). The combined organic layers were washed with brine (3 x 10 mL), dried over anhydrous Na_2SO_4, filtered through Celite, and concentrated to afford a yellow solid (0.644 g, 98%).

1-isopropyl-4-((4-nitrophenyl)sulfonyl)piperazine (**S-3**):
R_f: 0.34 (7:3 hexanes:EtOAc), vis: UV (254 nm).
m.p.: 116.2–118.6 ºC.
^{1}H (300 MHz, $CDCl_3$): δ = 8.38-8.36 (m, 2H), 7.95-7.92 (m, 2H), 3.09-3.06 (m, 4H), 2.75-2.66 (p, 1H, $^3J_{H\text{-}H}$ = 6.53 Hz), 2.61-2.58 (m, 4H), 1.00-0.98 (d, 6H, $^3J_{H\text{-}H}$ = 6.59 Hz).
^{13}C{^{1}H} (151 MHz, $CDCl_3$): δ = 150.2, 141.6, 129.0, 124.3, 54.4, 47.7, 46.4, 18.3.
ATR-IR (ν_{max}): 2969, 2931, 2857, 2821, 1528, 1347, 1167, 944, 855, 754, 743, 686, 611 cm^{-1}.
HRMS (*m/z*): (ESI) calc'd for $C_{13}H_{20}N_3O_4S^+$ [M-H]$^+$: calculated 314.1169, found 314.1162.

Compound S-4

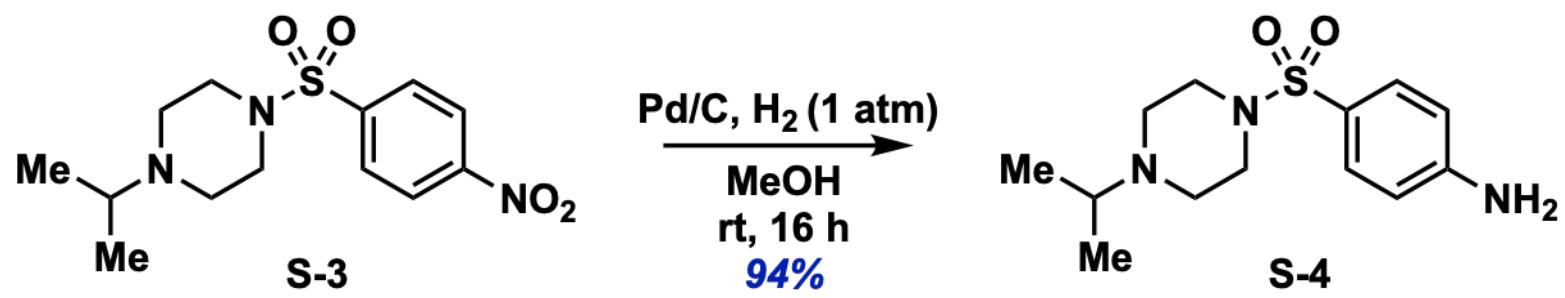

The synthesis of compound **S-4** was adapted from a known literature procedure.[6] To an oven-dried round bottom flask equipped with a stir bar was added compound **S-3** (0.644 g, 2.04 mmol, 1.0 equiv.), MeOH (18 mL), and followed by 10 wt.% Pd/C (40.0 mg). The flask was sealed, purged three times with a H_2 balloon. The reaction mixture was stirred at room temperature for 18 h under an H_2 atmosphere. Upon reaction completion, the reaction was diluted with MeOH (5.0 mL), filtered through Celite, and concentrated to afford a yellow-white solid (0.542 g, 94%).

4-((4-isopropylpiperazin-1-yl)sulfonyl)aniline (**S-4**):
R_f: 0.41 (19:1 EtOAc:TEA), vis: UV (254 nm).
m.p.: 148.4–150.3 ºC.
^{1}H (300 MHz, $CDCl_3$): δ = 7.53-7.50 (m, 2H) 6.69-6.66 (m, 2H), 4.08 (s, 2H), 2.99-2.97 (m, 4H), 2.0-2.64 (p, 1H, $^3J_{H-H}$ = 6.08 Hz), 2.60-2.56 (m, 4H), 1.01-0.99 (d, 6H, $^3J_{H-H}$ = 6.37 Hz).
^{13}C{^{1}H} (151 MHz, $CDCl_3$): δ = 150.7, 130.0, 123.3, 113.9, 54.4, 47.8, 46.4, 18.4.
ATR-IR (ν_{max}): 3375, 2968, 2819, 2359, 2341, 1630, 1596, 1316, 1266, 1154, 1093, 932, 738, 667, 612 cm^{-1}.
HRMS (*m/z*): (ESI) calc'd for $C_{13}H_{22}N_3O_2S^+$ [M-H]$^+$: calculated 284.1427, found 284.1419.

Compound 10

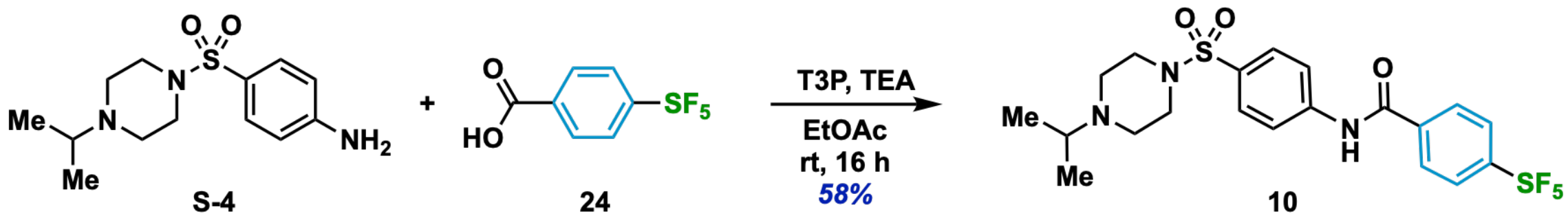

The synthesis of compound **10** was adapted from a known literature procedure.[6] To an oven-dried microwave vial equipped with a stir bar was added compound **24** (20.0 mg, 0.081 mmol, 1.0 equiv.). The vial was sealed, then evacuated and backfilled with Ar three times. Anhydrous EtOAc (1.0 mL), T3P (50% in EtOAc) (0.071 mL, 0.121, 1.5 equiv.), and TEA (0.022 mL, 0.161 mmol, 2.0 equiv.) were added sequentially *via* syringe. The reaction mixture was stirred at room temperature for 30 min. Then, a solution of compound **S-4** (0.025 g, 0.089 mmol 1.1 equiv.) in anhydrous EtOAc (1.0 mL) was added *via* syringe. The reaction mixture was stirred at room temperature for 16 h. Upon reaction completion, the reaction was diluted with water (2 mL). The aqueous layer was extracted with EtOAc (3 x 5 mL). The combined organic layers were washed with brine (3 x 5 mL), dried over anhydrous Na_2SO_4, filtered through Celite, and concentrated. The crude residue was purified *via* preparatory TLC eluting with 19:1 EtOAc:TEA to afford a light yellow solid (24.0 mg, 58%).

N-(4-((4-isopropylpiperazin-1-yl)sulfonyl)phenyl)-3-(pentafluoro-λ^6-sulfanyl)bicyclo[1.1.1]pentane-1-carboxamide (**10**):

R_f: 0.56 (19:1 EtOAc :TEA), vis: UV (254 nm).

m.p.: 162.8–164.1 ºC.

^{1}H (300 MHz, $CDCl_3$): δ = 8.28 (s, 1H), 8.01-7.99 (m, 2H), 7.9-7.82 (m, 4H), 7.74-7.71 (m, 2H), 3.02 (m. 4H), 2.71-2.65 (p, 1H, $^3J_{H-H}$ = 6.56 Hz), 2.59-2.58 (m, 4H), 1.00-0.98 (d, 6H, $^3J_{H-H}$ = 6.46 Hz).

^{19}F (282 MHz, $CDCl_3$): δ = 83.77-78.64 (p, 1F, $^2J_{F-F}$ = 146.83 Hz), 62.78-62.25 (d, 4F, $^2J_{F-F}$ = 150.95 Hz).

^{13}C{^{1}H} (151 MHz, $CDCl_3$): δ = 164.2, 156.4-156.0 (t, $^2J_{C-F}$ = 19.48 Hz), 141.8, 137.2, 130.7, 129.2, 127.9, 126.7-126.6 (t, $^3J_{C-F}$ = 4.35 Hz), 120.1, 54.4, 47.7, 46.4, 45.9, 18.3, 1.0.

ATR-IR (ν_{max}): 2973, 1684, 1592, 1529, 1326, 1266, 1163, 905, 844, 726, 671, 649 cm^{-1}.

HRMS (*m/z*): (ESI) calc'd for $C_{20}H_{25}F_5N_3O_3S_2^+$ [M-H]$^+$: calculated 514.1252, found 514.1241.

Compound 12

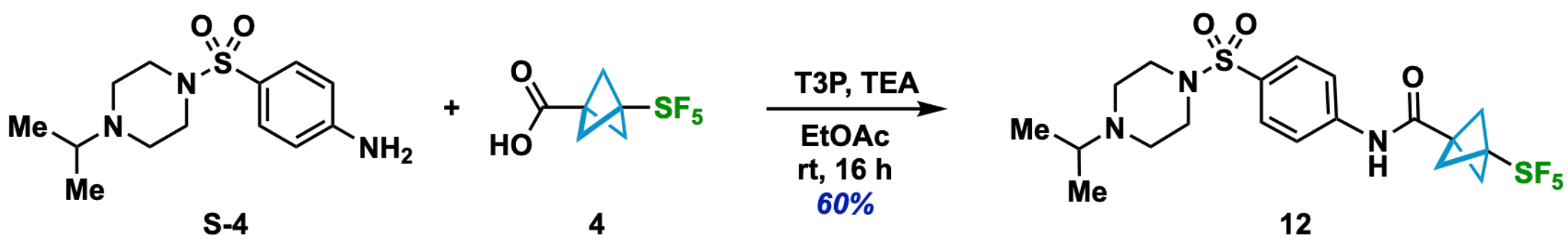

The synthesis of compound **12** was adapted from a known literature procedure.[6] To an oven-dried microwave vial equipped with a stir bar was added compound **4** (20.0 mg, 0.084 mmol, 1.0 equiv.). The vial was sealed, then evacuated and backfilled with Ar three times. Anhydrous EtOAc (1.0 mL), T3P (50% in EtOAc; 0.080 mL, 0.126, 1.5 equiv.), and TEA (0.024 mL, 0.168 mmol, 2.0 equiv.) were added sequentially *via* syringe and stirred for 30 min. Then compound **S-4** (26.0 mg, 0.092 mmol 1.1 equiv.) in anhydrous EtOAc (1 mL) was added, and the reaction mixture was stirred for 16 h. The reaction was diluted with water (2 mL), and the contents were transferred to a separatory funnel. The aqueous layer was extracted with EtOAc (3 x 5 mL). The combined organic layers were washed with brine (5 mL), dried over anhydrous Na_2SO_4, filtered through Celite, and concentrated. The crude residue was purified *via* preparatory TLC eluting with 19:1 EtOAc to afford a light yellow solid (24.0 mg, 60%).

N-(4-((4-isopropylpiperazin-1-yl)sulfonyl)phenyl)-3-(pentafluoro-λ^6-sulfanyl)bicyclo[1.1.1]pentane-1-carboxamide (**12**):
R_f: 0.59 (19:1 EtOAc:TEA), vis: UV (254 nm).
m.p.: 162.8–163.1 ºC.
^{1}H (300 MHz, $CDCl_3$): δ = 7.69 (m, 4H), 7.58 (s, 1H), 3.08 (m, 4H), 2.86-2.78 (p, 1H,$^3J_{H\text{-}H}$ = 6.08 Hz), 2.68 (m, 10H), 1.05-1.03 (d, 6H, $^3J_{H\text{-}H}$ = 6.32 Hz).
^{19}F (282 MHz, $CDCl_3$): δ = 79.04-76.97 (p, 1F, $^2J_{F\text{-}F}$ = 148.33 Hz), 49.40-48.89 (d,4F, $^2J_{F\text{-}F}$ = 146.77).
^{13}C{^{1}H} (151 MHz, $CDCl_3$): δ = 165.2, 141.4, 130.6, 129.1, 119.7, 55.9, 54.9, 47.7, 46.1, 36.1, 29.7, 22.7, 18.2, 1.0.
ATR-IR (ν_{max}): 2967, 2930, 1694, 1591, 1530, 1326, 1265, 1253, 1162, 1095, 908, 828, 801, 732, 670, 648, 611 cm^{-1}.
HRMS (*m/z*): (ESI) calc'd for $C_{19}H_{27}F_5N_3O_3S_2^+$ [M-H]$^+$: calculated 504.1408, found 504.1401.

Synthesis Overview of Benzpyrimoxan (13) and SF_5–BCP Derivative (16)

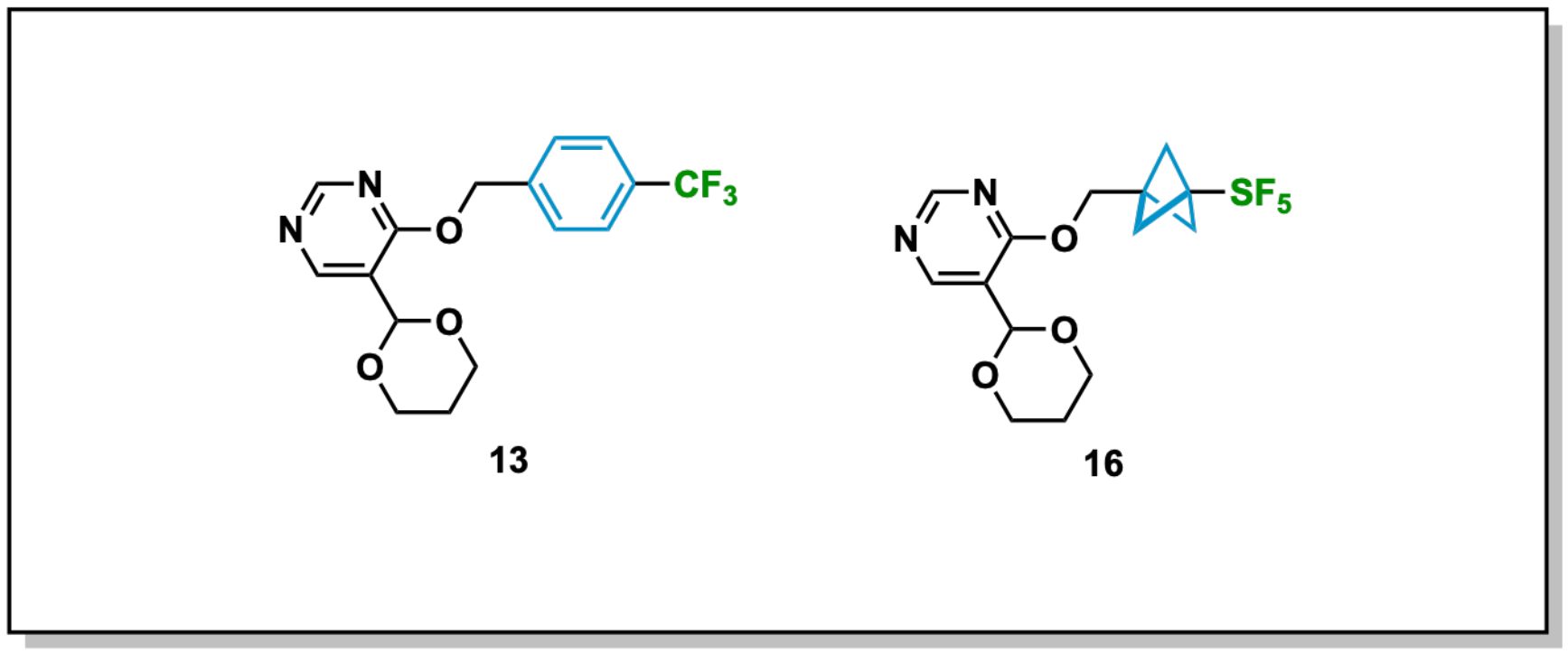

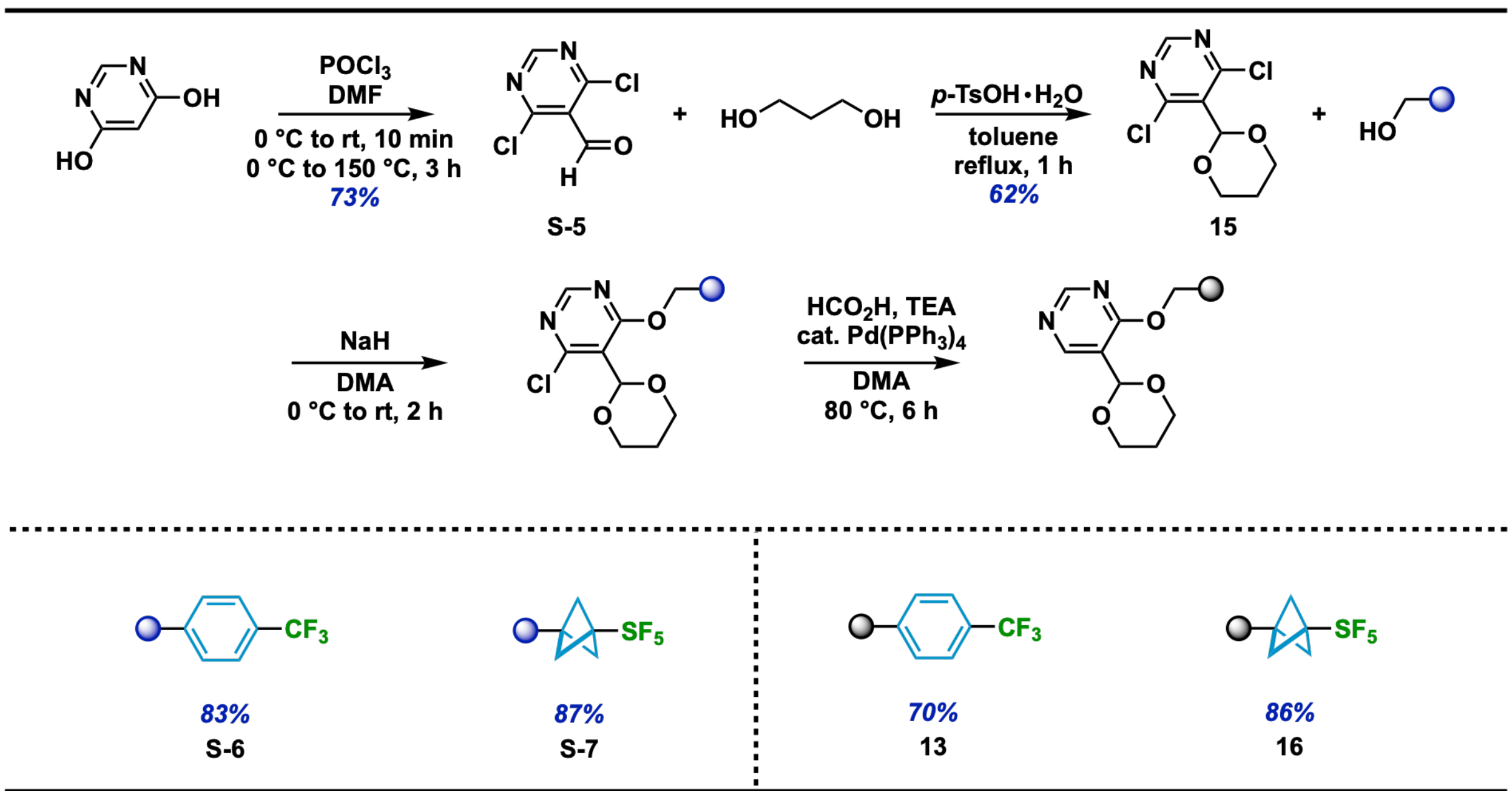

Scheme S3: Synthesis overview of Benzpyrimoxan (**13**) and its SF_5–BCP derivative (**16**).[7] *See pages S26-S31 for detailed procedures and corresponding characterization data.*

Synthesis of Benzpyrimoxan (13) and SF_5–BCP Derivative (16)

Compound S-5

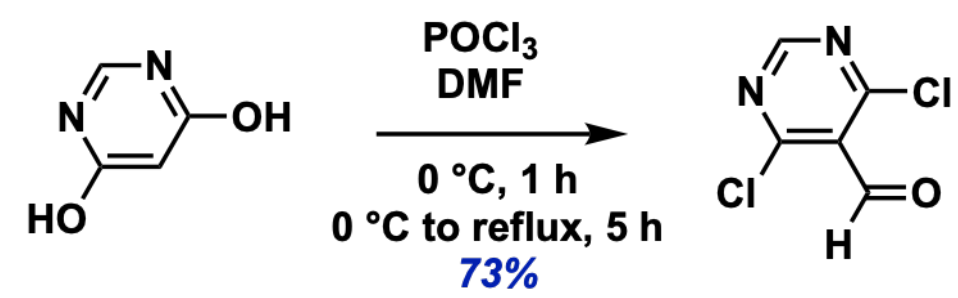

The synthesis of compound **S-5** was adapted from a known literature procedure.[7] An oven-dried two neck round bottom was equipped with a stir bar and a reflux condenser. The round bottom was then evacuated and backfilled with Ar three times. Freshly distilled $POCl_3$ (30 mL) was added *via* syringe and cooled to 0 °C in an ice water bath. Once cooled, anhydrous DMF (9.4 mL) was added dropwise *via* syringe. The reaction mixture was stirred. Subsequently, the reaction mixture was stirred for 1 h at 0 °C in an ice water bath. (*Note: If the reaction mixture solidifies while in the ice bath, bring the solution back to room temperature and continue stirring at 0 °C for the remainder of the hour*.) After 1 h, pyrimidine-4,6-diol (7.29 g, 65.0 mmol, 1.0 equiv.) was added, and the reaction mixture was heated to 150 °C in an oil bath for 5 h. As the reaction proceeded, the orange heterogenous mixture became homogenous with a clear brown-red color. After 5 h, the reaction mixture was cooled to room temperature, and the excess $POCl_3$ was removed *in vacuo*. The crude reaction mixture was poured over ice and extracted with Et_2O (6 x 250 mL). The combined organic layers were washed with brine (3 x 500 mL), dried over anhydrous Na_2SO_4, filtered through Celite, and concentrated. The crude solid was redissolved in Et_2O (100 mL). The organic layer was washed with sat. aq. $NaHCO_3$ (3 x 500 mL), dried over anhydrous Na_2SO_4, filtered through Celite, and concentrated. The desired product was obtained without further purification as a yellow solid (8.43 g, 73%).

4,6-dichloropyrimidine-5-carbaldehyde (**S-5**):
R_f: 0.60 (7:3 hexanes:EtOAc), vis: UV (254 nm).
m.p: 66.6–67.8 °C.
^{1}H (300 MHz, $CDCl_3$): δ = 10.40 (s, 1H), 8.85 (s, 1H).
^{13}C{^{1}H} (76 MHz, $CDCl_3$): δ = 185.7, 162.7, 159.6, 124.9.
ATR-IR (ν_{max}): 3381, 1692, 1509, 805 cm^{-1}.
HRMS (*m/z*): (ESI) calc'd for $C_5H_3Cl_2N_2O^+$ $[M-H]^+$: calculated 176.9617, found 176.9610.

Compound 15

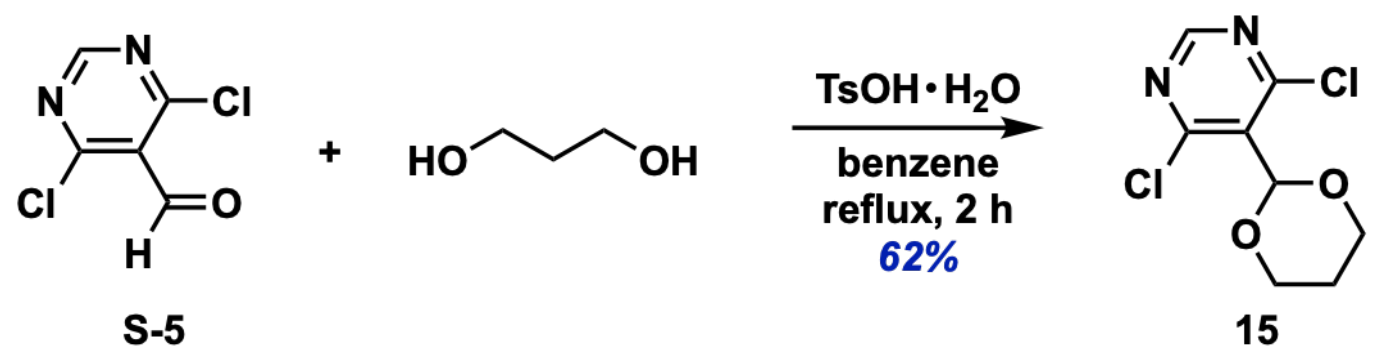

The synthesis of compound **15** was adapted from a known literature procedure.[7] To an oven-dried two neck round bottom equipped with a stir bar, attached to a dean stark trap, was added compound **S-5** (5.60 g, 32.0 mmol, 1.0 equiv.), sealed, evacuated and backfilled with Ar three times. Anhydrous benzene (50 mL) was added *via* syringe. The reaction mixture was stirred. Subsequently, propane-1,3-diol (2.80 mL, 38.4 mmol, 1.2 equiv.) and *p*-toluenesulfonic acid monohydrate (0.600 g, 3.20 mmol, 0.1 equiv.) were added. The reaction mixture was heated to 150 °C in an oil bath for 2 h. The reaction was monitored by TLC. Once completed, the reaction mixture was cooled to room temperature and concentrated. The crude mixture was extracted with Et_2O (3 x 50 mL). The combined organic layers were washed with brine (3 x 100 mL), dried over anhydrous Na_2SO_4, filtered through Celite, and concentrated. The crude residue was purified *via* column chromatography on silica gel eluting with 9:1 hexanes:EtOAc to afford a white solid (5.94 g, 62%).

4,6-dichloro-5-(1,3-dioxan-2-yl)pyrimidine (**15**):
R_f: 0.23 (9:1 hexanes:EtOAc), vis: UV (254 nm).
m.p: 121.8–122.9 °C.
^{1}H (300 MHz, $CDCl_3$): δ = 8.69 (s, 1H), 6.09 (s, 1H), 4.30-4.25 (m, 2H), 3.40-3.91 (m, 2H), 2.40-2.24 (m, 1H), 1.47-1.43 (m, 1H).
$^{13}C\{^1H\}$ (76 MHz, $CDCl_3$): δ = 161.6, 157.4, 128.13, 98.3, 67.8, 25.4.
ATR-IR (ν_{max}): 2991, 2959, 2869, 1520, 797.cm^{-1}.
HRMS (*m/z*): (ESI) calc'd for $C_8H_9Cl_2N_2O_2^+$ [M-H]$^+$: calculated 235.0036, found 235.0030.

Compound S-6

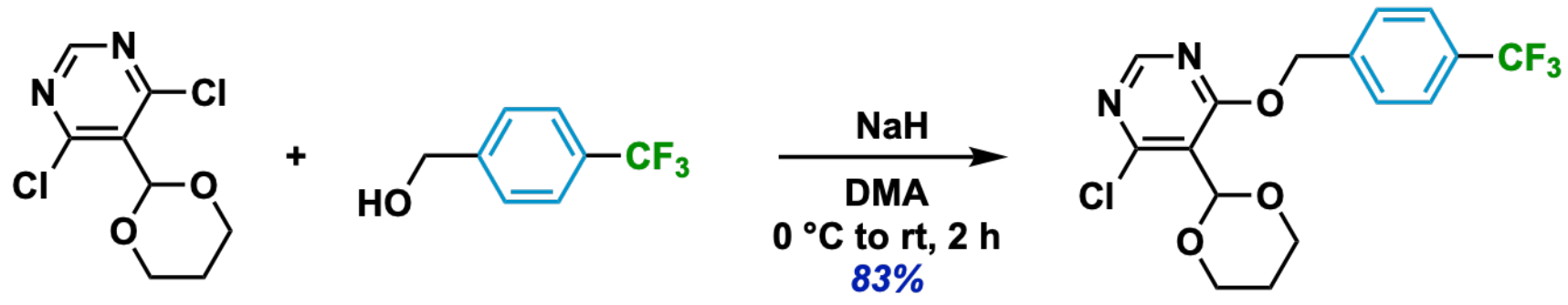

15 S-6

The synthesis of compound **S-6** was adapted from a known literature procedure.[7] To an oven-dried microwave vial equipped with a stir bar were added compound **15** (30.0 mg, 0.096 mmol, 1.0 equiv.) and (4-(trifluoromethyl)phenyl)methanol (0.020 mL, 0.109 mmol, 1.1 equiv.). The vial was sealed, evacuated, and backfilled with Ar three times. Anhydrous DMA (1.0 mL) was added *via* syringe. The resulting mixture was cooled to 0°C in an ice water bath. After the reaction mixture was cooled, NaH (60% dispersion in mineral oil; 4.70 mg, 0.118 mmol, 1.2 equiv.) was added. The reaction mixture was stirred at room temperature for 6 h. The reaction mixture was poured over ice and extracted with EtOAc (3 x 10 mL). The combined organic layers were washed with brine (3 x 10 mL), dried over anhydrous Na_2SO_4, filtered through Celite, and concentrated. The crude residue was purified *via* preparatory TLC eluting with 3:2 hexanes:EtOAc to afford an off-white solid (30.7 mg, 83%).

4-chloro-5-(1,3-dioxan-2-yl)-6-((4-(trifluoromethyl)benzyl)oxy)pyrimidine (**S-6**):
R_f: 0.39 (3:2 hexanes:EtOAc), vis: UV (254 nm).
m.p: 111.5–112.4 °C.
^{1}H (300 MHz, $CDCl_3$): δ = 8.51 (s, 1H), 7.66-7.58 (m, 4H), 6.04 (s, 1H), 5.56 (s, 2H), 4.30-4.24 (m, 2H), 4.00- 3.91 (m, 2H), 2.35-2.19 (m, 1H), 1.47-1.43 (m, 1H).
^{19}F (283 MHz, $CDCl_3$): δ = -62.56 (s, 3F).
^{13}C{^{1}H} (101 MHz, $CDCl_3$): δ = 167.6, 159.8, 157.4, 140.2, 130.7-129.8 (q, $^2J_{C-F}$ = 32.2 Hz), 127.5, 125.6-125.5 (q, $^3J_{C-F}$ = 4.0 Hz), 122.8, 120.1, 116.5, 97.5, 68.3, 67.8, 25.7.
ATR-IR (ν_{max}): 2982, 2929, 2875, 1555, 1015, 820 cm^{-1}.
HRMS (*m/z*): (ESI) calc'd for $C_{16}H_{15}ClF_3N_2O_3^+$ [M-H]$^+$: calculated 375.0718, found 375.0710.

Compound 13

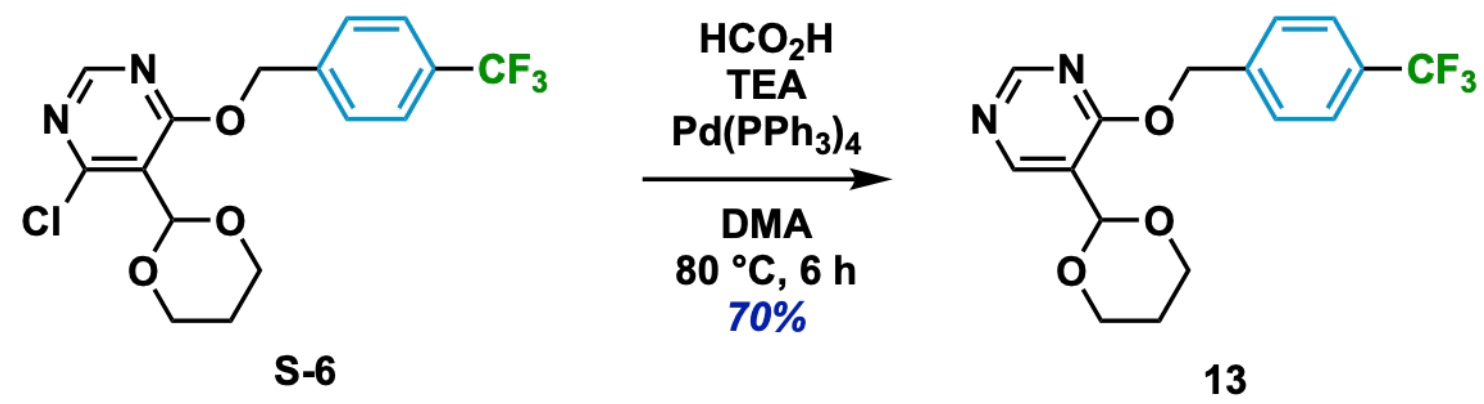

The synthesis of compound **13** was adapted from a known literature procedure.[7] To an oven-dried microwave vial equipped with a stir bar were added compound **S-6** (29.6 mg, 0.079 mmol, 1.0 equiv.) and $Pd(PPh_3)_4$ (9.10 mg, 0.008 mmol, 0.1 equiv.). The vial was sealed, evacuated, and backfilled with Ar three times. Anhydrous DMA (0.25 mL), HCO_2H (0.010 mL, 0.160 mmol, 2.0 equiv.), and TEA (0.030 mL, 0.174 mmol, 2.2 equiv.) were added sequentially *via* syringe. The reaction mixture was heated to 80 °C in an oil bath for 2 h. Upon reaction completion, the crude mixture was extracted with CH_2Cl_2 (3 x 10 mL). The combined organic layers were washed with brine (3 x 10 mL), dried over anhydrous Na_2SO_4, filtered through Celite, and concentrated. The crude residue was purified *via* preparatory TLC eluting with 3:2 hexanes:EtOAc to afford a white solid (18.8 mg, 70%).

5-(1,3-dioxan-2-yl)-4-((4-(trifluoromethyl)benzyl)oxy)pyrimidine (**13**):
R_f: 0.42 (3:2 hexanes:EtOAc), vis: UV (254 nm).
m.p: 114.3–115.3 °C.
^{1}H (300 MHz, $CDCl_3$): δ = 88.76 (s, 1H), 8.75 (s, 1H), 7.65-7.53 (d, 4H, $^2J_{H\text{-}H}$ = 8.2Hz), 5.76 (s, 1H), 5.55 (s, 2H), 4.29- 4.23 (m, 2H), 4.02-3.93 (m, 2H), 2.32- 2.17 (m, 1H), 1.50- 1.43 (m, 1H).
^{19}F (283 MHz, $CDCl_3$): δ = -62.59 (s, 3F).
^{13}C{^{1}H} (101 MHz, $CDCl_3$): δ = 65.5, 158.4, 155.6, 140.4, 130.8- 129.8 (q, $^2J_{C\text{-}F}$ = 32.7 Hz), 127.7, 125.7-125.5 (q, $^2J_{C\text{-}F}$ = 3.1 Hz), 122.8, 119.2, 95.8, 67.7, 67.2, 25.7.
ATR-IR (ν_{max}): 2923, 2361, 1332, 1017 cm^{-1}.
HRMS (*m/z*): (ESI) calc'd for $C_{16}H_{16}F_3NO_3^+$ [M-H]$^+$: calculated 341.1108, found 341.1109.

Compound S-7

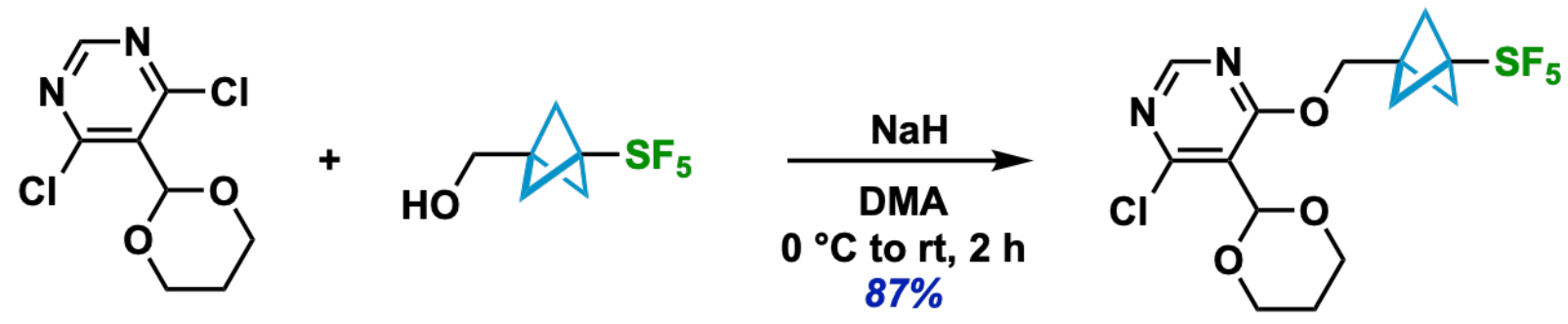

15 14 S-7

The synthesis of compound **S-7** was adapted from a known literature procedure.[7] To an oven-dried microwave vile equipped with a stir bar were added compound **15** (50.0 mg, 0.164 mmol, 1.0 equiv.) and compound **14** (40.5 mg, 0.181 mmol, 1.1 equiv.). The vial was sealed, evacuated, and backfilled with Ar three times. Anhydrous DMA (1.7 mL) was added *via* syringe. The resulting mixture was cooled to 0 °C in an ice water bath. After the reaction mixture was cooled, NaH (60% dispersion in mineral oil; 7.90 mg, 0.197 mmol, 1.2 equiv.) was added. The reaction mixture stirred at room temperature for 6 h. The reaction mixture was poured over ice and extracted with EtOAc (3 x 10 mL). The combined organic layers were washed with brine (3 x 10 mL), dried over anhydrous Na_2SO_4, filtered through Celite, and concentrated. The crude residue was purified *via* column chromatography on silica gel eluting with 8:2 hexanes:EtOAc to afford an off-white solid (60.3 mg, 87%).

4-chloro-5-(1,3-dioxan-2-yl)-6-((3-(pentafluoro-λ^6-sulfanyl)bicyclo[1.1.1]pentan-1-yl)methoxy)pyrimidine (**S-7**):
R_f: 0.39 (8:2 hexanes:EtOAc), vis: UV (254 nm).
m.p: 114.0–117.0 °C.
^{1}H (300 MHz, $CDCl_3$): δ = 8.47 (s, 1H), 5.98 (s, 1H), 4.59 (s, 2H), 4.29-4.24 (m, 2H), 4.00-3.91 (m, 2H), 2.37 (s, 6H), 2.34- 2.18 (m, 1H), 1.50-1.46 (m, 1H).
^{19}F (283 MHz, $CDCl_3$): δ = 80.18-78.11 (p, 1F, $^2J_{F-F}$ = 146 Hz), 48.75-48.23 (d, 4F, $^2J_{F-F}$ = 146 Hz).
^{13}C{^{1}H} (101 MHz, $CDCl_3$): δ = 167.7, 159.8, 157.3, 116.2, 97.5, 67.8-66.5 (p, $^2J_{C-F}$ = 15.4 Hz), 65.0, 54.8-54.8 (t, $^2J_{C-F}$ = 2.2 Hz), 33.7, 25.8.
ATR-IR (ν_{max}): 2982, 2934, 2866, 1550, 826, 796 cm^{-1}.
HRMS (*m/z*): (ESI) calc'd for $C_{14}H_{17}ClF_5N_2O_3S^+$ [M-H]$^+$: calculated 423.0563, found 423.0565.

Compound 16

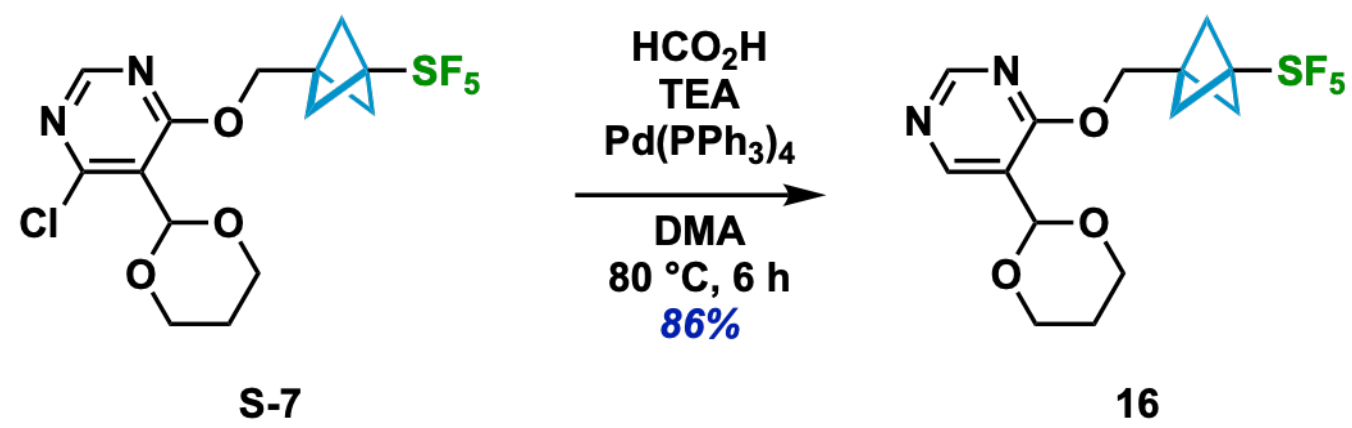

The synthesis of compound **16** was adapted from a known literature procedure.[7] To an oven-dried microwave vial equipped with a stir bar were added compound **S-7** (10.0 mg, 0.024 mmol, 1.0 equiv.) and $Pd(PPh_3)_4$ (5.50 mg, 0.005 mmol, 0.2 equiv.). The vial was sealed, evacuated, and backfilled with Ar three times. Anhydrous DMA (0.25 mL) was added *via* syringe. To the resulting mixture was added HCO_2H (0.010 mL, 0.095 mmol, 4.0 equiv.) and TEA (0.020 mL, 0.104 mmol, 4.4 equiv.) sequentially *via* syringe. The reaction mixture was heated to 80 °C in an oil bath for 2 h. The crude mixture was extracted with CH_2Cl_2 (3 x 10 mL). The combined organic layers were washed with brine (3 x 10 mL), dried over anhydrous Na_2SO_4, filtered through Celite, and concentrated. The crude residue was purified *via* preparatory TLC eluting with 3:2 hexanes:EtOAc to afford a white solid (7.90 mg, 86%).

5-(1,3-dioxan-2-yl)-4-((3-(pentafluoro-λ^6-sulfanyl)bicyclo[1.1.1]pentan-1-yl)methoxy)pyrimidine (**16**):
R_f: 0.36 (3:2 hexanes:EtOAc), vis: UV (254 nm).
m.p: 97.5–98.7 °C.
^{1}H (300 MHz, $CDCl_3$): δ = 8.71 (s, 2H), 5.66 (s, 1H), 4.56 (s, 2H), 4.28-4.22 (m, 2H), 4.00-3.92 (m, 2H), 2.32 (s, 6H), 2.29-2.15 (m, 1H), 1.50-1.42 (m, 1H).
^{19}F (283 MHz, $CDCl_3$): δ = 80.17-78.11 (p, 1F, $^2J_{F\text{-}F}$ = 145 Hz), 48.76-48.24 (d, 4F, $^2J_{F\text{-}F}$ = 145 Hz).
^{13}C{^{1}H} (101 MHz, $CDCl_3$): δ = 165.5, 158.3, 155.5, 119.1, 95.7, 67.7, 67.1-66.3 (p, $^2J_{C\text{-}F}$ = 15.8 Hz), 63.9, 54.8-54.7 (t, $^2J_{C\text{-}F}$ = 2.7 Hz), 33.7, 25.7.
ATR-IR (ν_{max}): 2930, 1578, 1094, 803, 792 cm^{-1}.
HRMS (*m/z*): (ESI) calc'd for $C_{14}H_{18}F_5N_2O_3S^+$ $[M+H]^+$: calculated 389.0953, found 389.0955.

Synthesis Overview of Tecovirimat (17) and SF_5–BCP Derivative (20)

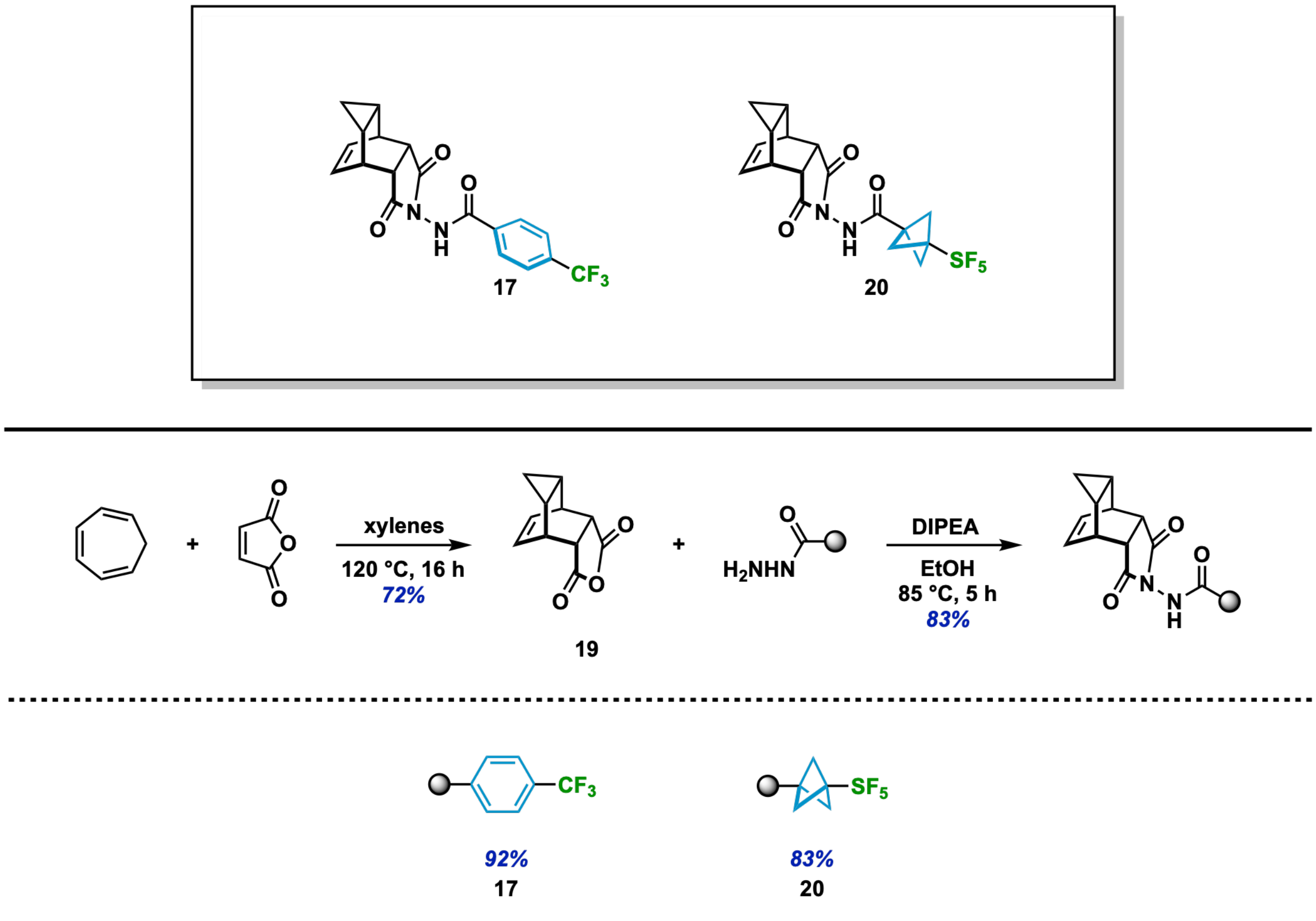

Scheme S4: Synthesis overview of Tecovirimat (**17**) and its SF_5–BCP derivative (**20**).[8] *See pages S33-S36 for detailed procedures and corresponding characterization data.*

Synthesis of Tecovirimat (17) and SF_5–BCP Derivative (20)

Compound 19

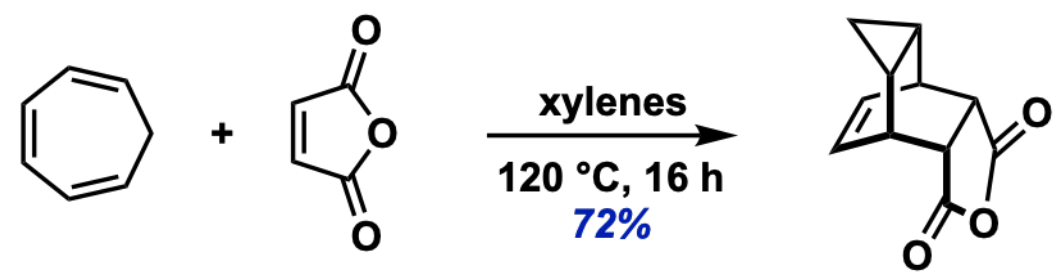

19

The synthesis of compound **19** was adapted from a known literature procedure.[8] To an oven-dried two neck round bottom equipped with a stir bar and a reflux condenser was added maleic anhydride (3.06 g, 31.2 mmol, 1.15 equiv.). The round bottom flask was sealed, evacuated, and backfilled with Ar three times. Then, xylenes (15 mL) and cycloheptatriene (2.82 mL, 27.13 mmol, 1.0 equiv.) were added sequentially *via* syringe. The reaction mixture was then stirred at 120 °C in an oil bath for 16 h. Upon reaction completion, the reaction was cooled to room temperature. The crude reaction mixture was filtered, and the precipitate was washed with xylenes to afford a light brown solid (3.72 g, 72%). (*Note: The filtrate was concentrated and the resulting material recrystallized from EtOH to afford more of the desired product.)*

(4*R*,4a*R*,5a*S*,6*S*,6a*S*)-4,4a,5,5a,6,6a-hexahydro-1*H*-4,6-ethenocyclopropa[*f*]isobenzofuran-1,3(3a*H*)-dione (**19**):
R_f: 0.19 (17:3 hexanes:EtOAc), vis: $KMnO_4$.
m.p: 100.8–101.6 ºC.
^{1}H (300 MHz, DMSO-d_6): δ = 5.87-5.84 (m, 2H), 3.40- 3.38 (m, 2H), 3.30-2.25 (m, 2H), 1.18-1.13 (m, 2H), 0.32-0.25 (m, 1H), 0.05-0.01 (m, 1H).
^{13}C{^{1}H} (151 MHz, DMSO-d_6): δ = 173.3, 128.3, 45.7, 33.1, 8.9, 4.6.
ATR-IR (ν_{max}): 3010, 1769, 910 cm^{-1}.
HRMS (*m/z*): (ESI) calc'd for $C_{11}H_{11}O_3^+$ $[M+H]^+$: calculated 191.0703, found 190.0706.

Compound S-8

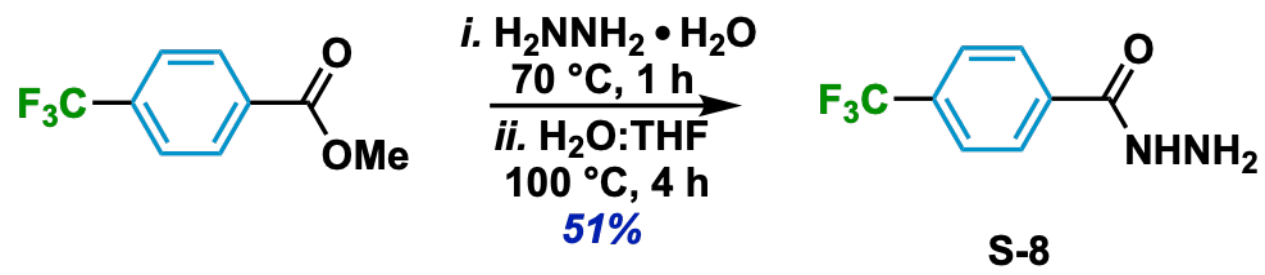

The synthesis of compound **S-8** was adapted from a known literature procedure.[8] An oven-dried microwave vial equipped with a stir bar was sealed, evacuated, and backfilled with Ar three times. Then, methyl 4-(trifluoromethyl)benzoate (0.160 mL, 0.980 mmol, 1.0 equiv.) and $H_2NNH_2{\bullet}H_2O$ (0.100 mL, 1.47 mmol, 1.5 equiv.) were added *via* syringe. The mixture was stirred at 70 °C in an oil bath for 1 h. A solution of 8:1 H_2O:MeOH (0.9 mL) was added dropwise *via* syringe. The reaction was stirred at 100 °C in an oil bath for 4 h. The crude reaction mixture was extracted with EtOAc (3 x 10 mL). The combined organic layers were washed with brine (3 x 20 mL), dried over anhydrous Na_2SO_4, filtered through Celite, and concentrated. The crude solid was purified *via* column chromatography on silica gel eluting with 97:3 CH_2Cl_2:MeOH to afford a white solid (0.204 g, 51%).

4-(trifluoromethyl)benzohydrazide (**S-8**):
R_f: 0.11 (97:3 CH_2Cl_2:MeOH), vis: *p*-anisaldehyde.
m.p.:135.1–137.0 ºC.
^{1}H (300 MHz, DMSO-*d6*): δ = 10.02 (s, 1H), 8.03-8.00 (d, 2H, $^3J_{H-H}$ = 8.09 Hz), 7.84-7.81 (d, 2H, $^3J_{H-H}$ = 8.19 Hz), 4.59 (s, 2H).
^{19}F (283 MHz, DMSO-*d6*): δ = -61.38 (s, 3F).
^{13}C{^{1}H} (101 MHz, DMSO-*d6*): δ = 164.5, 137.1, 131.4-130.5 (m), 127.8, 125.4-125.2 (m), 122.5.
ATR-IR (ν_{max}): 3303, 1639, 1329, 858, 650 cm^{-1}.
HRMS (*m/z*): (ESI) calc'd for $C_8H_6F_3N_2O^-$ [M-H]$^-$: calculated 203.0438, found 203.0429.

Compound 17

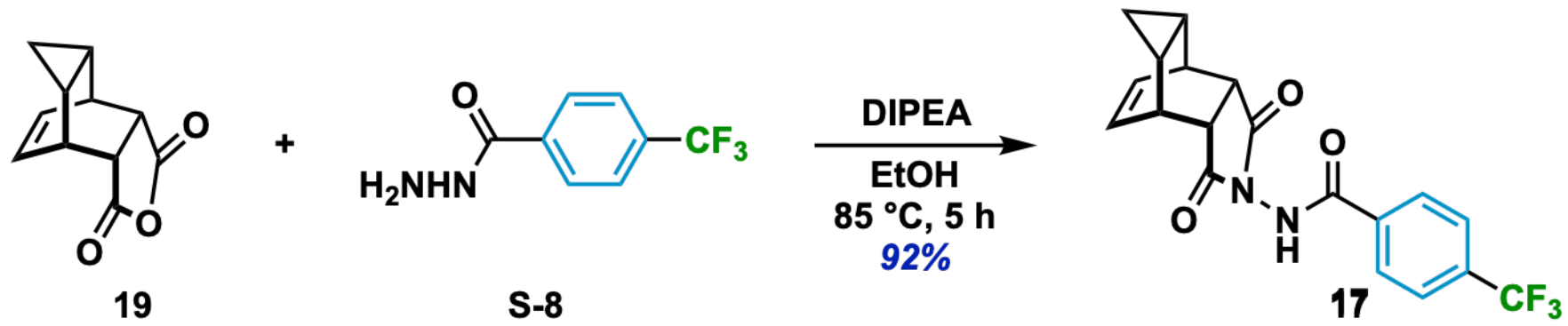

The synthesis of compound **17** was adapted from a known literature procedure.[8] To an oven-dried microwave vial equipped with a stir bar were added compounds **19** (25.0 mg, 0.131 mmol, 1.0 equiv.) and **S-8** (28.4 mg, 0.138 mmol, 1.05 equiv.). The vial was then sealed with a cap with a septum using a crimper, evacuated, and backfilled with Ar three times. Anhydrous EtOH (0.75 mL) and DIPEA (0.010 mL, 0.005 mmol, 0.04 equiv.) were added sequentially *via* syringe. The reaction mixture was then heated to 85 °C and stirred in an oil bath for 5 h. Upon reaction completion, the reaction mixture was concentrated, and purified *via* preparatory TLC eluting with 98:2 CH_2Cl_2:MeOH to afford a white solid (46.3 mg, 92%).

N-((4a*R*,5a*S*,6a*S*)-1,3-dioxo-3,3a,4,4a,5,5a,6,6a-octahydro-4,6-ethenocyclopropa[*f*]isoindol-2(1*H*)-yl)-4-(trifluoromethyl)benzamide (**17**):
R_f: 0.43 (97:3 CH_2Cl_2:MeOH), vis: *p*-anisaldehyde.
m.p: 191.0–193.0 °C.
^{1}H (300 MHz, DMSO-*d₆*): δ = 11.24 (s, 1H), 8.10-7.91 (m, 4H), 5.8 (s, 2H), 3.29-3.24 (m, 4H), 1.18 (s, 1H), 0.30-0.24 (m, 1H), 0.07 (s, 1H).
^{19}F (283 MHz, DMSO-d_6): δ = -61.54 (s, 3F).
^{13}C{^{1}H} (201 MHz, DMSO-d_6): δ = 174.8, 163.6-163.1 (m), 135.0, 134.6, 133.6-143.1 (m), 128.7-128.6 (m), 127.6-127.3 (m), 125.8, 43.4-43.0 (m), 33.1-32.8 (m), 9.2, 4.1.
ATR-IR (ν_{max}): 3394, 3008, 2957, 1714, 1132, 735 cm^{-1}.
HRMS (*m/z*): (ESI) calc'd $C_{19}H_{16}F_3N_2O_3^+$ [M-H]$^+$: calculated 377.1108, found 377.1110.
HRMS (*m/z*): (ESI) calc'd for $C_{19}H_{16}F_3N_2O_3^-$ [M-H]$^-$: calculated 375.0962, found 375.0963.

Compound 20

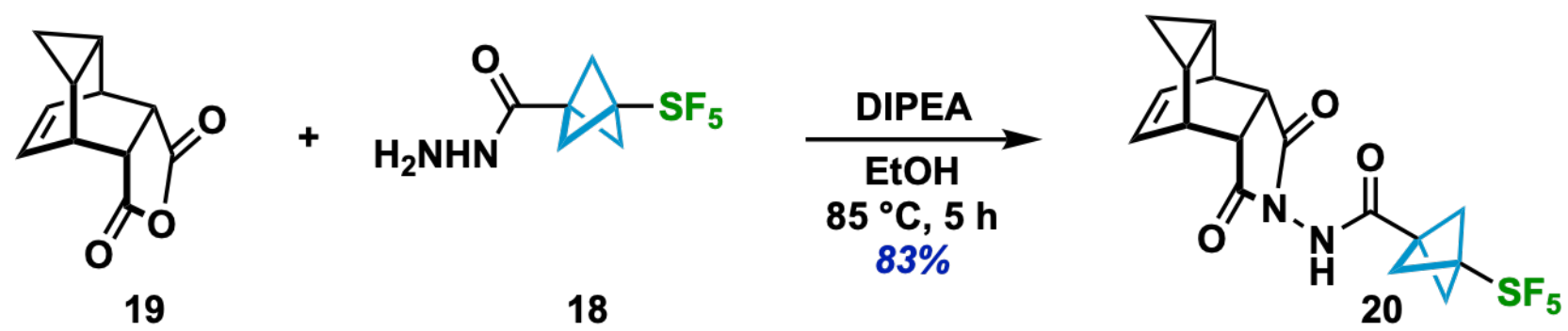

The synthesis of compound **20** was adapted from a known literature procedure.[8] To an oven-dried microwave vial equipped with a stir bar were added compounds **19** (25.0 mg, 0.131 mmol, 1.0 equiv.) and **18** (34.8 mg, 0.138 mmol, 1.05 equiv.). The vial was then sealed with a cap with a septum using a crimper, evacuated, and backfilled with Ar three times. Anhydrous EtOH (0.75 mL) and DIPEA (0.010 mL, 0.005 mmol, 0.04 equiv.) were then added sequentially *via* syringe. The reaction mixture was heated to 85 °C in an oil bath for 5 h. Upon reaction completion, the reaction was concentrated and purified *via* preparatory TLC eluting with 98:2 CH_2Cl_2:MeOH to afford a white solid (46.3 mg, 83%).

N-((4R,4aR,5aS,6S,6aS)-1,3-dioxo-3,3a,4,4a,5,5a,6,6a-octahydro-4,6-ethenocyclopropa[f]isoindol-2(1H)-yl)-3-(pentafluoro-λ^6-sulfanyl)bicyclo[1.1.1]pentane-1-carboxamide (**20**):
R_f: 0.37 (98:2 CH_2Cl_2:MeOH), vis: *p*-anisaldehyde stain.
m.p: 189.8–190.8 °C.
^{1}H (300 MHz, MeCN-*d*$_3$): δ = 5.76-5.70 (m, 2H), 3.32-3.27 (m, 2H), 3.10-3.09 (m, 2H), 2.64 (s, 6H), 1.17-1.12 (m, 2H), 0.30-0.23 (m, 1H), 0.16-0.11 (m,1H).
^{19}F (282 MHz, MeCN-*d*$_3$): δ = 79.27-77.22 (p, 1F, $^2J_{F\text{-}F}$ = 145 Hz), 48.35-47.84 (d, 4F, $^2J_{F\text{-}F}$ = 145 Hz).
^{13}C{^{1}H} (76 MHz, MeOD-*d*$_4$): δ = 176.4, 128.8, 67.5- 66.7 (p, $^2J_{C\text{-}F}$ = 16.4 Hz), 57.1-57.0 (t, $^2J_{C\text{-}F}$ = 2.7 Hz), 44.9, 34.9, 34.7, 10.3, 4.7.
ATR-IR (ν_{max}): 3524,1724, 1189, 829, 796 cm^{-1}.
HRMS (*m/z*): (ESI) calc'd for $C_{17}H_{18}F_5N_2O_3S^+$ $[M\text{-}H]^+$: calculated 425.0953, found 425.0958.

Synthesis Overview of SF_5–[2]staffane Darapladib Derivative (30)

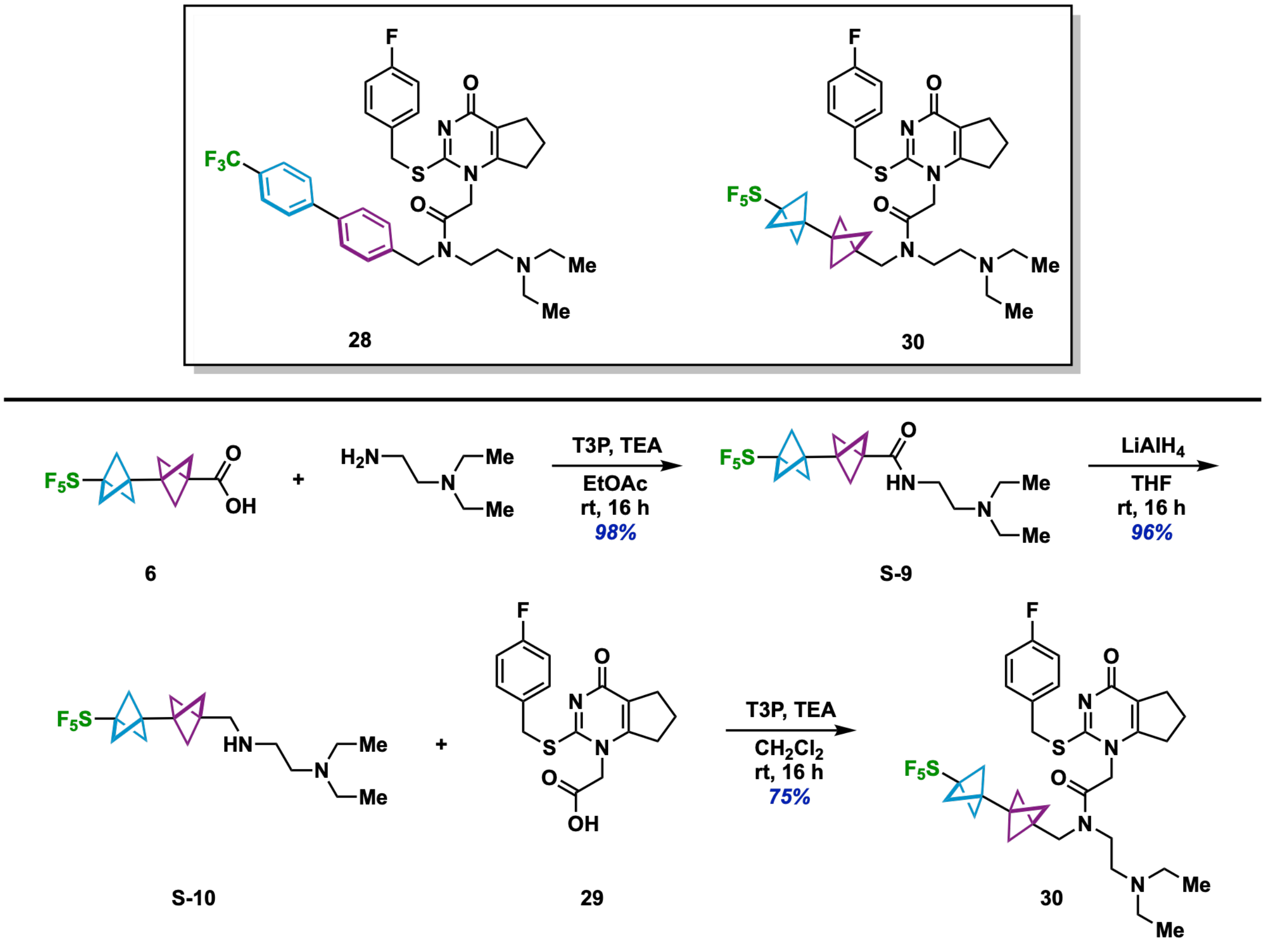

Scheme S5: Synthesis overview of the SF_5– [2]staffane derivative of Darapladib (**30**).[9] *See pages S38-S40 for detailed procedures and corresponding characterization data. Note: Darapladib (**28**) was purchased from Ambeed.*

Synthesis of SF_5–[*2*]staffane Darapladib Derivative (30)

Compound S-9

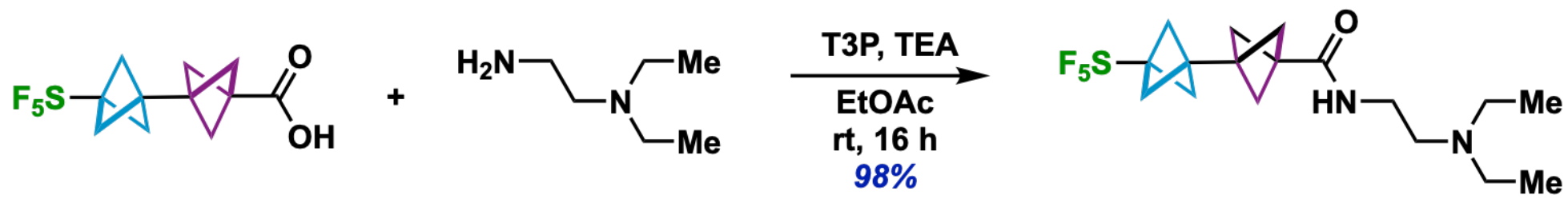

6 S-9

The synthesis of compound **S-9** was adapted from a known literature procedure.[9] To an oven-dried microwave vial equipped with a stir bar was added compound **6** (0.200 g, 0.657 mmol, 1.0 equiv.). The vial was sealed, evacuated, and backfilled with Ar three times. Anhydrous EtOAc (2.1 mL), T3P (50% in EtOAc; 0.627 mL, 0.986 mmol, 1.5 equiv.), and TEA (0.182 mL, 1.31 mmol, 2.0 equiv.) were added sequentially *via* syringe and stirred for 30 min. Then, *N^1,N^1*-diethylethane-1,2-diamine (0.138 mL, 0.985 mmol 1.5 equiv.) was added, and the reaction mixture was stirred at room temperature for 16 h. Upon reaction completion, the reaction was diluted with water (2 mL). The resulting layers were separated and the aqueous layer extracted with CH_2Cl_2 (3 x 5 mL). The combined organic layers were washed with brine (3 x 5 mL), dried over anhydrous Na_2SO_4, filtered through Celite, and concentrated. The crude residue was purified *via* column chromatography on silica gel eluting with 10% TEA in 4:1 hexanes:EtOAc to afford a light yellow solid (0.259 g, 98%).

N-(2-(diethylamino)ethyl)-3'-(pentafluoro-λ^6-sulfanyl)-[1,1'-bi(bicyclo[1.1.1]pentane)]-3-carboxamide (**S-9**):
R_f: 0.17 (10% TEA in 4:1 hexanes:EtOAc), vis: *p*-anisaldehyde.
m.p.: 122.3–124.5 ºC.
^{1}H (300 MHz, $CDCl_3$): δ = 7.31 (bs, 1H), 3.45-3.39 (m, 2H), 2.84-2.77 (m, 6H), 2.15 (s, 6H), 1.91 (s, 6H), 1.22-1.17 (t, 6H, $^3J_{H-H}$= 7.20 Hz).
^{19}F (282 MHz, $CDCl_3$): δ = 80.47-78.34 (p, 1F, $^2J_{F-F}$ = 145.6 Hz), 48.42-47.91 (d, 4F, $^2J_{F-F}$ = 145.6 Hz).
^{13}C{^{1}H} (151 MHz, $CDCl_3$): δ = 170.2, 66.4-66.0 (m), 54.3, 52.1, 50.4, 47.8, 38.9, 36.6, 36.6, 34.7, 10.4.
ATR-IR (ν_{max}): 3340, 3204, 2982, 2928, 2879, 1633, 1548, 1536, 1227, 1210, 1177, 903, 802, 734, 708 cm^{-1}.
HRMS (*m/z*): (ESI) calc'd for $C_{17}H_{28}F_5N_2OS^+$ [M-H]$^+$: calculated 403.1837, found 403.1848.

Compound S-10

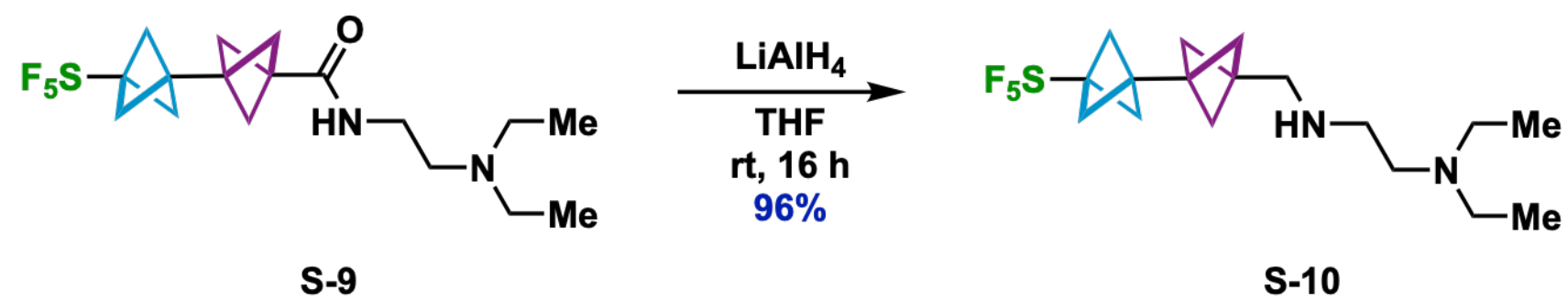

The synthesis of compound **S-10** was adapted from a known literature procedure.[9] To an oven-dried microwave vial equipped with a stir bar was added compound **S-9** (0.149 g, 0.370 mmol, 1.0 equiv.). The vial was sealed, evacuated, and backfilled with Ar three times. Anhydrous THF (2.0 mL) was added *via* syringe. To the resulting mixture, 2.0 M solution of $LiAlH_4$ in THF (0.370 mL, 0.740 mmol, 2.0 equiv.) was added dropwise and left to stir at room temperature for 16 h. Upon reaction completion, the reaction mixture was cooled to 0 °C and diluted with THF (4 mL) and then carefully quenched with H_2O (4 mL). Followed by 1 M HCl (4 mL) and EtOAc (4 mL). The aqueous layer was extracted with EtOAc (3 x 10 mL). The combined organic layers were washed with brine (3 x 25 mL), dried over anhydrous Na_2SO_4, filtered through Celite, and concentrated. The crude residue was purified *via* column chromatography on silica gel eluting with 1.5% TEA in 19:1 CH_2Cl_2:MeOH to afford an orange oil (0.137 g, 96%).

N^1,N^1-diethyl-N^2-((3'-(pentafluoro-λ^6-sulfanyl)-[1,1'-bi(bicyclo[1.1.1]pentan)]-3-yl)methyl)ethane-1,2-diamine (**S-10**):

R_f: 0.27 (1.5% TEA in 19:1 CH_2Cl_2:MeOH), vis: *p*-anisaldehyde.

^1H (300 MHz, $CDCl_3$): δ = 2.67-2.63 (m, 4H), 2.54-2.46 (m, 6H), 2.14 (s, 6H), 1.57 (s, 6H), 1.02-0.97 (t, 6H, $^3J_{H\text{-}H}$ = 7.20 Hz).

^{19}F (282 MHz, $CDCl_3$): δ = 80.72-78.66 (p, 1F, $^2J_{F\text{-}F}$ = 145.6 Hz), 48.30-47.78 (d, 4F, $^2J_{F\text{-}F}$ = 145.6 Hz).

$^{13}C\{^1H\}$ (151 MHz, $CDCl_3$): δ = 66.9-66.1 (m), 54.3, 52.6, 51.2, 49.1, 48.0, 47.3, 38.8, 37.4, 35.1, 12.0.

ATR-IR (ν_{max}): 2976, 2926, 2909, 2872, 2807, 1451, 1284, 1210, 1119, 1065, 997, 935, 903, 883, 822, 793 cm^{-1}.

HRMS (*m/z*): (ESI) calc'd for $C_{17}H_{30}F_5N_2S^+$ $[M\text{-}H]^+$: calculated 389.2044, found 389.2045.

Compound 30

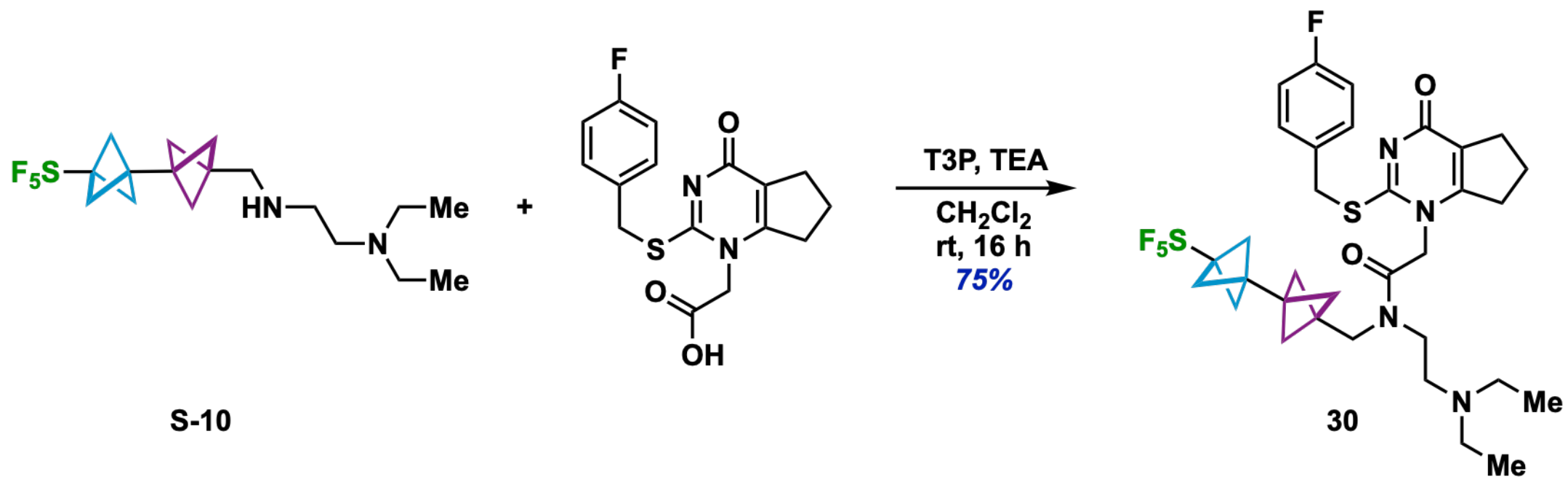

The synthesis of compound **30** was adapted from a known literature procedure.[9] To an oven-dried microwave vial equipped with a stir bar was added 2-(2-((4-Fluorobenzyl)thio)-4-oxo-4,5,6,7-tetrahydro-1H-cyclopenta[d]pyrimidin-1-yl)acetic acid (0.120 g, 0.359 mmol, 1.5 equiv.). The vial was sealed, evacuated, and backfilled with Ar three times. Anhydrous CH_2Cl_2 (1.0 mL), T3P (50% in EtOAc; 0.230 mL, 0.359 mmol, 1.5 equiv.), and TEA (0.07 mL, 0.479 mmol, 2.0 equiv.) were added sequentially *via* syringe and stirred for 30 min. Then **compound S-10** (93.0 mg, 0.239 mmol, 1.0 equiv.) in anhydrous CH_2Cl_2 (1.0 mL) was added via syringe, and the reaction mixture was stirred at room temperature for 16 h. Upon reaction completion, the reaction was diluted with water (2 mL). The aqueous layer was extracted with CH_2Cl_2 (3 x 5 mL). The combined organic layers were washed with brine (3 x 5 mL), dried over anhydrous Na_2SO_4, filtered through Celite, and concentrated. The crude residue was purified *via* preparative TLC eluting with 19:1 CH_2Cl_2:7 M NH_3 in MeOH to afford a clear waxy solid (0.126 g, 75%).

N-(2-(diethylamino)ethyl)-2-(2-((4-fluorobenzyl)thio)-4-oxo-4,5,6,7-tetrahydro-1*H*-cyclopenta[*d*]pyrimidin-1-yl)-*N*-((3'-(pentafluoro-λ^6-sulfanyl)-[1,1'-bi(bicyclo[1.1.1]pentan)]-3-yl)methyl)acetamide (**30**):

R_f: 0.33 (19:1 CH_2Cl_2:7 M NH_3 MeOH), vis: UV (254 nm).

^{1}H (300 MHz, $CDCl_3$): δ = 7.35-7.29 (m, 2H), 6.97-6.92 (m, 2H), 4.82 (s, 2H), 4.48-4.47 (m, 2H), 4.48-4.47 (m, 2H), 3.43-3.36 (m, 4H), 3.25-3.21 (m, 2H), 2.83-2.78 (m, 2H), 2.75-2.71 (m, 2H), 2.55-2.45 (m, 9H), 2.08 (s, 6H), 1.52 (s, 6H), 0.99-0.93 (m, 9H).

^{19}F (282 MHz, $CDCl_3$): δ = 80.55-78.25 (m, 1F), 48.49-47.89 (m, 4F), -114.16– -114.27 (m, 1F).

^{13}C{^{1}H} (151 MHz, $CDCl_3$): δ = 167.1, 167.0, 164.7, 164.1, 163.9, 160.8, 160.7, 155.9, 155.5, 131.7, 131.60, 131.1, 131.0, 121.3, 121.2, 115.7, 115.4, 66.5-65.9 (m), 54.2, 51.4, 49.7, 49.5, 47.9, 47.5, 47.2, 37.5, 37.22, 36.4, 36.3, 31.9, 28.3, 20.7, 11.8.

ATR-IR (ν_{max}): 2969, 2926, 2872, 1658, 1629, 1509, 1485, 1424, 1213, 1196, 1158, 903, 825, 794, 728 cm^{-1}.

HRMS (*m/z*): (ESI) calc'd for $C_{33}H_{43}F_6N_4O_2S_2^+$ $[M-H]^+$: calculated 705.2726, found 705.2731.

Synthesis Overview of OSU-T315 (31)

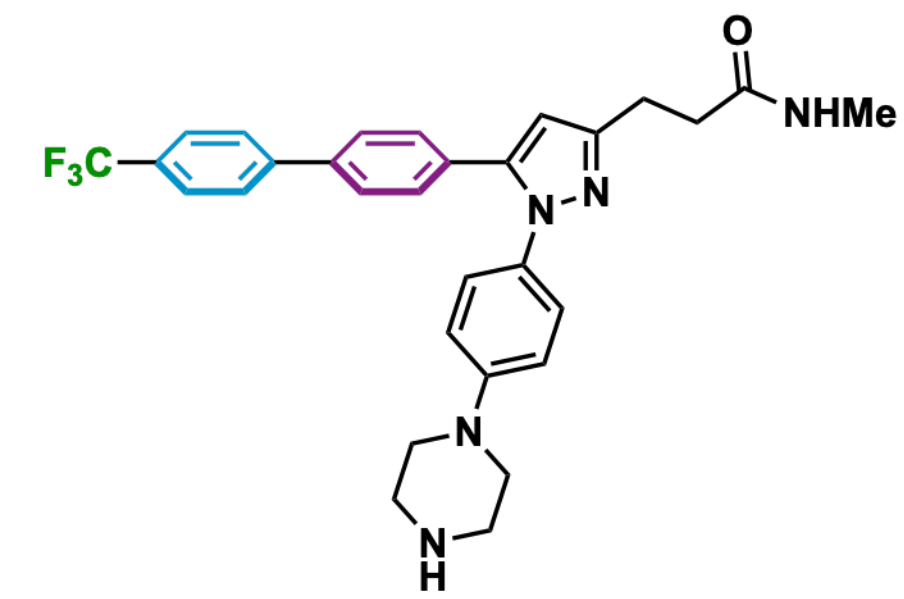

31

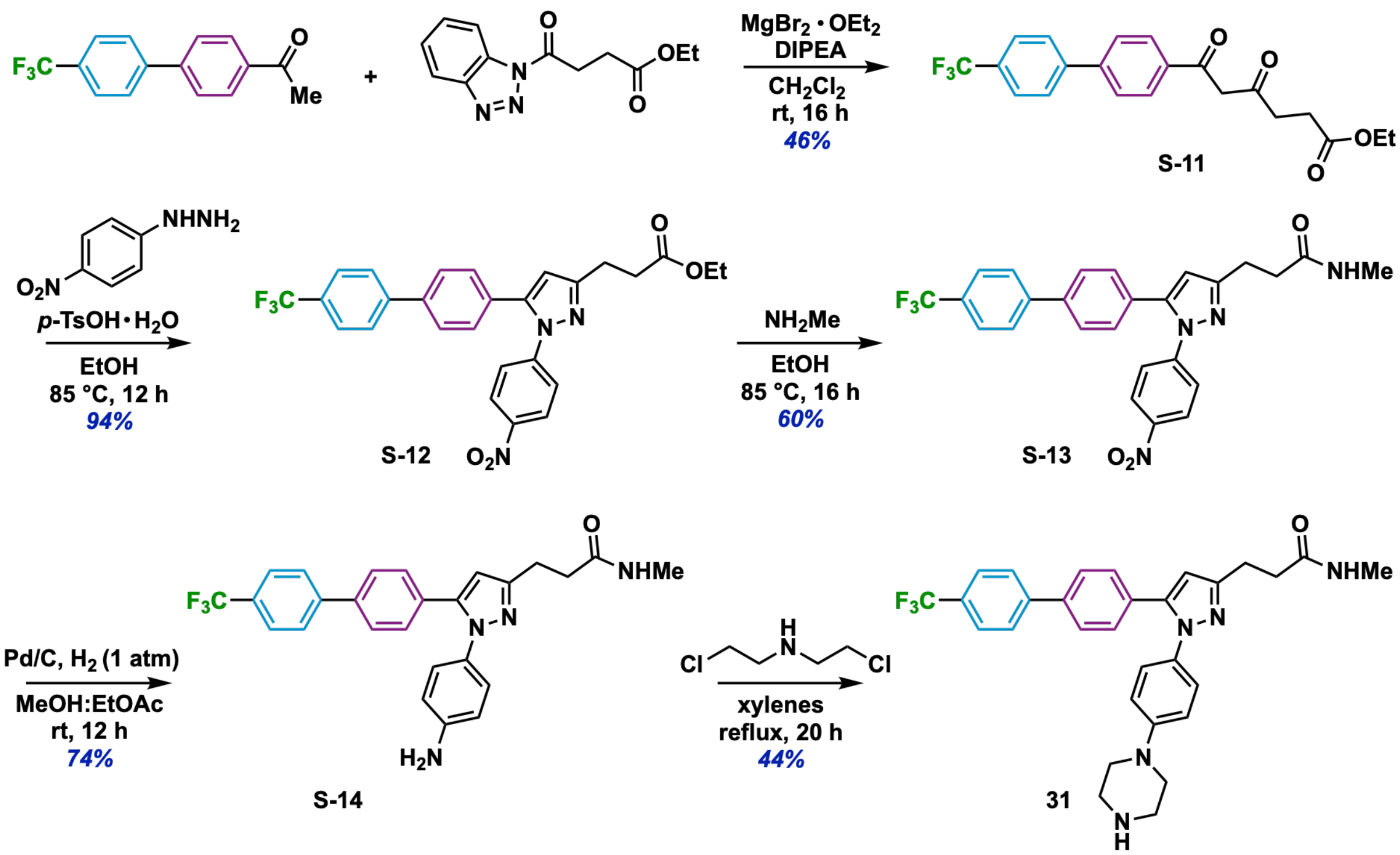

Scheme S6: Synthesis overview of OSU-T315 (**31**).[10] *See pages S42-S46 for detailed procedures and corresponding characterization data.*

Compound S-11

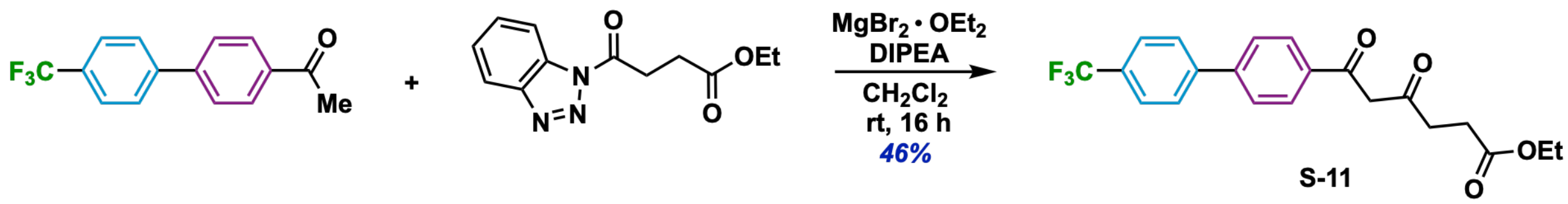

The synthesis of compound **S-11** was adapted from a known literature procedure.[10] To an oven-dried round bottom flask equipped with a stir bar was added 1-(4'-(trifluoromethyl)-[1,1'-biphenyl]-4-yl)ethan-1-one (0.391 g, 1.48 mmol, 1.0 equiv.). The round bottom flask was sealed, evacuated, and purged with Ar three times. Anhydrous CH_2Cl_2 (18 mL) was added, followed by ethyl 4-(1*H*-benzo[*d*][1,2,3]triazol-1-yl)-4-oxobutanoate (0.440 g, 1.78 mmol, 1.2 equiv.), and magnesium bromide ethyl etherate (1.53 g, 5.92 mmol, 4.0 equiv.). The resulting mixture was stirred for 10 min, then DIPEA (1.03 mL, 5.92 mmol, 4.0 equiv.) was added dropwise, and the reaction mixture was stirred at room temperature for 16 h. Upon reaction completion, the reaction was diluted with 1 M HCl (15 mL), water (15 mL), and extracted with CH_2Cl_2 (3 x 10 mL). The combined organic layers were washed with brine (3 x 4 mL), dried over anhydrous Na_2SO_4, filtered through Celite, and concentrated. The crude residue was purified *via* column chromatography on silica gel eluting with 4:1 hexanes:EtOAc to afford a light yellow solid (0.267 g, 46% yield).

ethyl 4,6-dioxo-6-(4'-(trifluoromethyl)-[1,1'-biphenyl]-4-yl)hexanoate (**S-11**):
R_f: 0.29 (4:1 hexanes:EtOAc), vis: UV (254 nm).
m.p.: 150.3–152.3 ºC.
^{1}H (300 MHz, $CDCl_3$): δ = 15.81 (bs, 1H), 7.98-7.96 (m, 2H), 7.73 (m, 4H), 7.69-7.67 (m, 2H), 6.25 (s, 1H), 4.21-4.14 (q, 2H, $^3J_{H\text{-}H}$ = 7.22 Hz), 2.86-2.69 (m, 4H), 1.30-1.25 (t, 3H, $^3J_{H\text{-}H}$ = 7.22 Hz).
^{19}F (282 MHz, $CDCl_3$): δ = -62.53 (s, 3F).
^{13}C{^{1}H} (151 MHz, $CDCl_3$): δ = 197.4, 179.7, 172.7, 143.6, 143.4, 134.0, 130.9-129.7 (m), 129.6, 127.8, 127.7 (m), 126.1-125.9 (m), 96.5, 60.8, 34.6, 29.2, 14.2.
ATR-IR (ν_{max}): 2992, 2931, 2913, 1719, 1613, 1324, 1191, 1163, 1115, 1071, 1003, 836, 802, 771, 748 cm^{-1}.
HRMS (*m/z*): (ESI) calc'd for $C_{21}H_{20}F_3O_4^+$ $[M\text{-}H]^+$: calculated 393.1308, found 393.1302.

Compound S-12

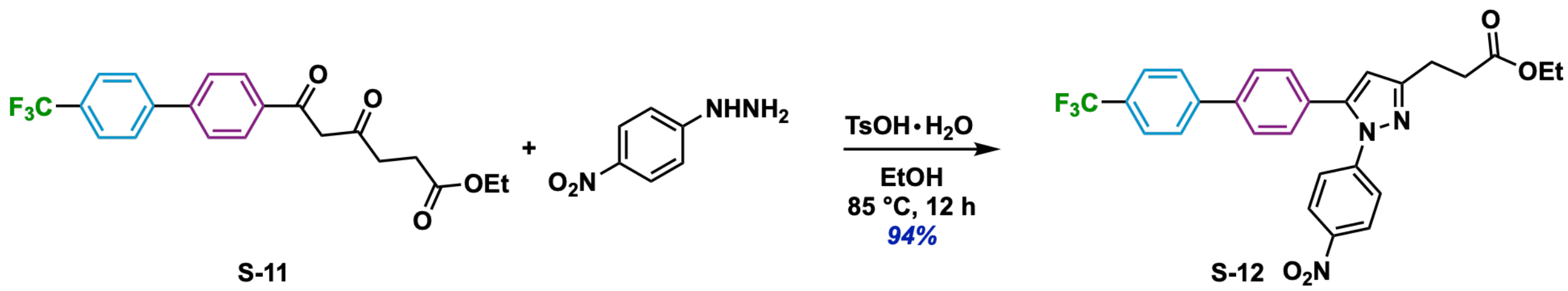

The synthesis of compound **S-12** was adapted from a known literature procedure.[10] To an oven-dried microwave vial equipped with a stir bar were added compound **S-11** (0.144 g, 0.380 mmol, 1.0 equiv.), 4-nitro-phenylhydrazine hydrochloride (87.0 mg, 0.457 mmol, 1.2 equiv.), and 4-methylbenzene-sulfonic acid (72.0 mg, 0.390 mmol, 1.0 equiv.). The vial was sealed, evacuated, and purged with Ar three times. Anhydrous EtOH (4.0 mL) was added *via* syringe and the resulting solution was heated to 85 °C in an oil bath for 12 h. Upon reaction completion, the reaction was concentrated and recrystallized from EtOH to afford a yellow solid (0.181 g, 94% yield).

ethyl 3-(1-(4-nitrophenyl)-5-(4'-(trifluoromethyl)-[1,1'-biphenyl]-4-yl)-1*H*-pyrazol-3-yl)propanoate (**S-12**):
R_f: 0.20 (4:1 hexanes:EtOAc), vis: UV (254 nm).
m.p.: 134.5–135.5 ºC.
^{1}H (300 MHz, $CDCl_3$): δ = 8.40-8.37 (m, 2H), 7.96-7.94 (m, 2H), 7.80-7.77 (m, 8H), 6.66 (s, 1H), 4.20-4.13 (q, 2H, $^3J_{H-H}$ = 7.22 Hz), 3.17-3.12 (m, 2H), 2.78-2.73 (m, 2H), 1.28-1.24 (t, 3H, $^3J_{H-H}$ = 7.22 Hz).
^{19}F (282 MHz, $CDCl_3$): δ = -62.38 (s, 3F).
^{13}C{^{1}H} (151 MHz, $CDCl_3$): δ = 171.9,152.4, 146.5, 144.8, 144.2, 144.1, 139.7, 132.5, 130.2-128.9 (q, $^2J_{C-F}$ = 32.85 Hz), 127.7, 127.4, 126.5, 125.9-125.8 (q, $^3J_{C-F}$ = 3.83 Hz), 125.0, 124.9, 104.8, 61.1, 33.1, 22.2, 14.3.
ATR-IR (ν_{max}): 2981, 2922, 1723, 1593, 1501, 1330, 1160, 1109, 1094, 1072, 1014, 1006, 855, 829, 816, 750 cm^{-1}.
HRMS (*m/z*): (ESI) calc'd for $C_{27}H_{23}F_3N_3O_4^+$ [M-H]$^+$: calculated 510.1635, found 510.1631.

Compound S-13

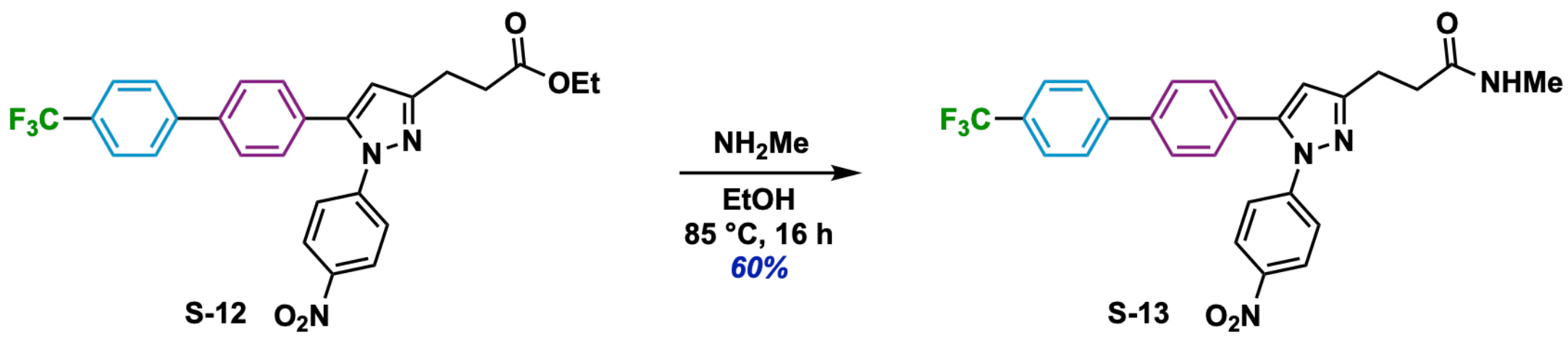

The synthesis of compound **S-13** was adapted from a known literature procedure.[10] To an oven-dried microwave vial equipped with a stir bar was added compound **S-12** (0.226 g, 0.446 mmol, 1.0 equiv.). The vial was sealed, evacuated, and purged with Ar three times. Then, a 1 M methylamine in ethanol solution (4.0 mL) was added *via* syringe and the resulting solution was heated to 85 °C in an oil bath for 16 h. Upon reaction completion, the reaction was concentrated and recrystallized from EtOH to afford a yellow solid (0.132 g, 60% yield).

N-methyl-3-(1-(4-nitrophenyl)-5-(4'-(trifluoromethyl)-[1,1'-biphenyl]-4-yl)-1*H*-pyrazol-3-yl)propanamide (**S-13**):

R_f: 0.42 (9:1 EtOAc:TEA), vis: UV (254 nm).

m.p.: 235.3–237.0 ºC.

^{1}H (300 MHz, $CDCl_3$): δ = 8.20-8.18 (m, 2H), 7.70 (s, 4H), 7.61-7.58 (m, 2H), 7.49-7.46 (m, 2H), 7.34-7.31 (m, 2H), 6.45 (s, 1H), 5.79 (s, 1H), 3.12-3.07 (m, 2H), 2.84-2.83 (d, 3H, $^3J_{H\text{-}H}$ = 4.68 Hz), 2.69-2.64 (m, 2H).

^{19}F (282 MHz, $CDCl_3$): δ = -60.86 (s, 3F).

^{13}C{^{1}H} (151 MHz, $CDCl_3$): δ = 172.8, 154.4, 145.9, 144.9, 143.8, 143.5, 140.3, 130.2-129.7 (q, $^2J_{C\text{-}F}$ = 32.85 Hz), 129.9, 129.4, 127.8, 127.5, 126.3-122.2 (q, $^1J_{C\text{-}F}$ = 272.08 Hz), 126.0-125.9 (q, $^3J_{C\text{-}F}$ = 3.54 Hz), 124.7, 124.6, 109.4, 35.5, 26.5, 24.2.

ATR-IR (ν_{max}): 3315, 2922, 1639, 1594, 1501, 1331, 1157, 1118, 1109, 1072, 855, 828, 808 cm^{-1}.

HRMS (*m/z*): (ESI) calc'd for $C_{26}H_{22}F_3N_4O_3^+$ [M-H]$^+$: calculated 495.1638, found 495.1636.

Compound S-14

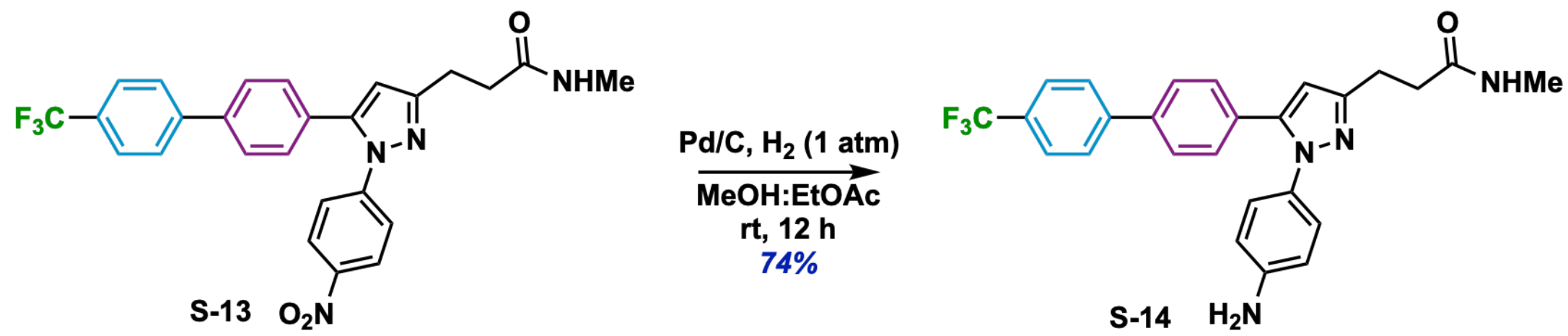

The synthesis of compound **S-14** was adapted from a known literature procedure.[10] To an oven-dried microwave vial equipped with a stir bar was added compound **S-13** (0.089 g, 0.180 mmol, 1.0 equiv.), dissolved in 1:1 MeOH:EtOAc (2.0 mL) and followed by 10 wt. % Pd/C (10.0 mg, 0.094 mmol, 0.5 equiv.). The flask was sealed and purged three times with a H_2 balloon. The reaction mixture was stirred at room temperature for 12 h under an H_2 atmosphere. Upon reaction completion, the reaction was diluted with MeOH (5.0 mL), filtered through Celite, and concentrated. The crude residue was then recrystallized from EtOH to afford a white solid (62.0 g, 74% yield).

3-(1-(4-aminophenyl)-5-(4'-(trifluoromethyl)-[1,1'-biphenyl]-4-yl)-1*H*-pyrazol-3-yl)-*N*-methylpropanamide (**S-14**):

R_f: 0.42 (9:1 EtOAc:TEA), vis: UV (254 nm).

m.p.: 167.5–170.5 ºC.

^{1}H (300 MHz, CDCl$_3$): δ = 7.91 (m, 2H), 7.72-7.65 (m, 4H), 7.62-7.59 (m, 2H), 7.21-7.17 (m, 2H), 6.71-6.67 (m, 2H), 6.50 (s, 1H), 5.81-5.75 (m, 1H), 3.87 (bs, 2H), 2.99-2.94 (m, 2H), 2.74-2.72 (d, 3H, $^3J_{H\text{-}H}$ = 4.68 Hz), 2.43-2.38 (m, 2H).

^{19}F (282 MHz, CDCl$_3$): δ = -62.32 (s, 3F).

^{13}C{^{1}H} (151 MHz, CDCl$_3$): δ = 171.9, 150.4, 146.9, 144.3, 144.1, 138.8, 133.4, 130.5, 129.8-128.7 (q, $^2J_{C\text{-}F}$ = 32.85 Hz), 127.5, 127.2, 127.1, 126.2, 125.9-125.7 (q, $^3J_{C\text{-}F}$ = 3.83 Hz), 115.2, 102.3, 35.2, 26.3, 22.2.

ATR-IR (ν_{max}): 3412, 3334, 3059, 2934, 1631, 1612, 1518, 1324, 1281, 1266, 1159, 1109, 1069, 829, 795, 733 cm^{-1}.

HRMS (*m/z*): (ESI) calc'd for $C_{26}H_{24}F_3N_4O^+$ [M-H]$^+$: calculated 465.1898, found 465.1895.

Compound 31

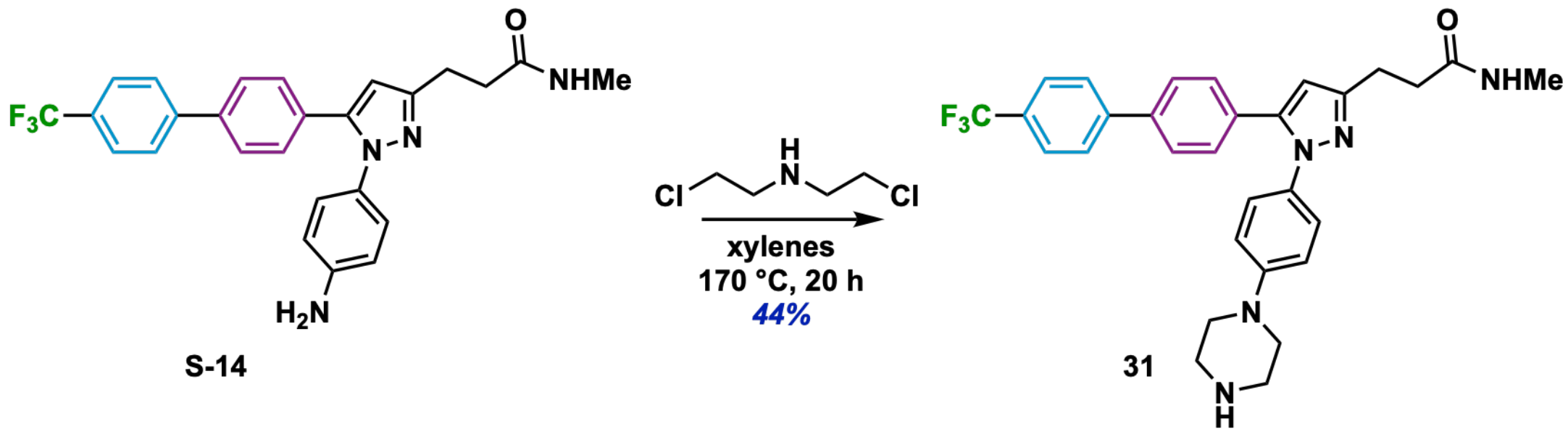

The synthesis of compound **31** was adapted from a known literature procedure.[10] To an oven-dried microwave vial equipped with a stir bar was added compound **S-14** (20.0 mg, 0.043 mmol, 1.0 equiv.). the vial was sealed with a cap with a septum using a crimper, evacuated, and backfilled with Ar three times. Dry xylenes (0.5 mL) was then added *via* syringe. The reaction mixture was stirred, subsequently bis(2-chloroethyl)amine hydrochloride (10.0 mg, 0.052 mmol, 1.2 equiv.) was. The reaction mixture was heated to 170 °C in an oil bath for 20 h. Upon reaction completion, the reaction was cooled to room temperature and concentrated. The crude residue was purified *via* column chromatography on silica gel eluting with 9:1 CH_2Cl_2:7 M NH_3 in MeOH to afford a white solid (10.0 mg, 44%).

N-methyl-3-(1-(4-(piperazin-1-yl)phenyl)-5-(4'-(trifluoromethyl)-[1,1'-biphenyl]-4-yl)-1*H*-pyrazol-3-yl)propanamide (**31**):

R_f: 0.13 (19:1 CH_2Cl_2:7 M NH_3 in MeOH), vis: UV (254 nm).

m.p.: 167.5–170.6 ºC.

^{1}H (300 MHz, $CDCl_3$): δ = 7.94-7.91 (m, 2H), 7.74-7.66 (m, 4H), 7.64-7.61 (m, 2H), 7.36-7.33 (m, 2H), 6.99-6.96 (m, 2H), 6.53 (s, 1H), 5.56-5.51 (m, 1H), 3.21-3.18 (m, 4H), 3.06-2.99 (m, 3H), 2.78-2.76 (d, 3H, $^3J_{H-H}$= 4.68 Hz), 2.47-2.42 (m, 2H).

^{19}F (282 MHz, $CDCl_3$): δ = -62.34 (s, 3F).

^{13}C{^{1}H} (151 MHz, $CDCl_3$): δ = 171.9, 151.7, 150.5, 144.4, 144.0, 138.9, 133.4, 131.3 ,129.6-129.1 (q, $^2J_{C-F}$ = 32.85 Hz), 127.5, 127.3, 126.6, 126.3, 126.4-122.4 (q, $^1J_{C-F}$ = 271.42 Hz), 125.8-125.7 (q, $^3J_{C-F}$ = 3.83 Hz), 116.2, 102.5, 50.0, 46.0, 35.3, 26.5, 22.2.

ATR-IR (ν_{max}): 3302, 3069, 2922, 2844, 2360, 2341, 1650, 1613, 1518, 1324, 1237, 1164, 1112, 1070, 908, 827, 795, 728 cm^{-1}.

HRMS (*m/z*): (ESI) calc'd for $C_{30}H_{31}F_3N_5O^+$ [M-H]$^+$: calculated 534.2475, found 534.2472.

Synthesis Overview of SF_5–[*2*]staffane OSU-T315 Derivative (33)

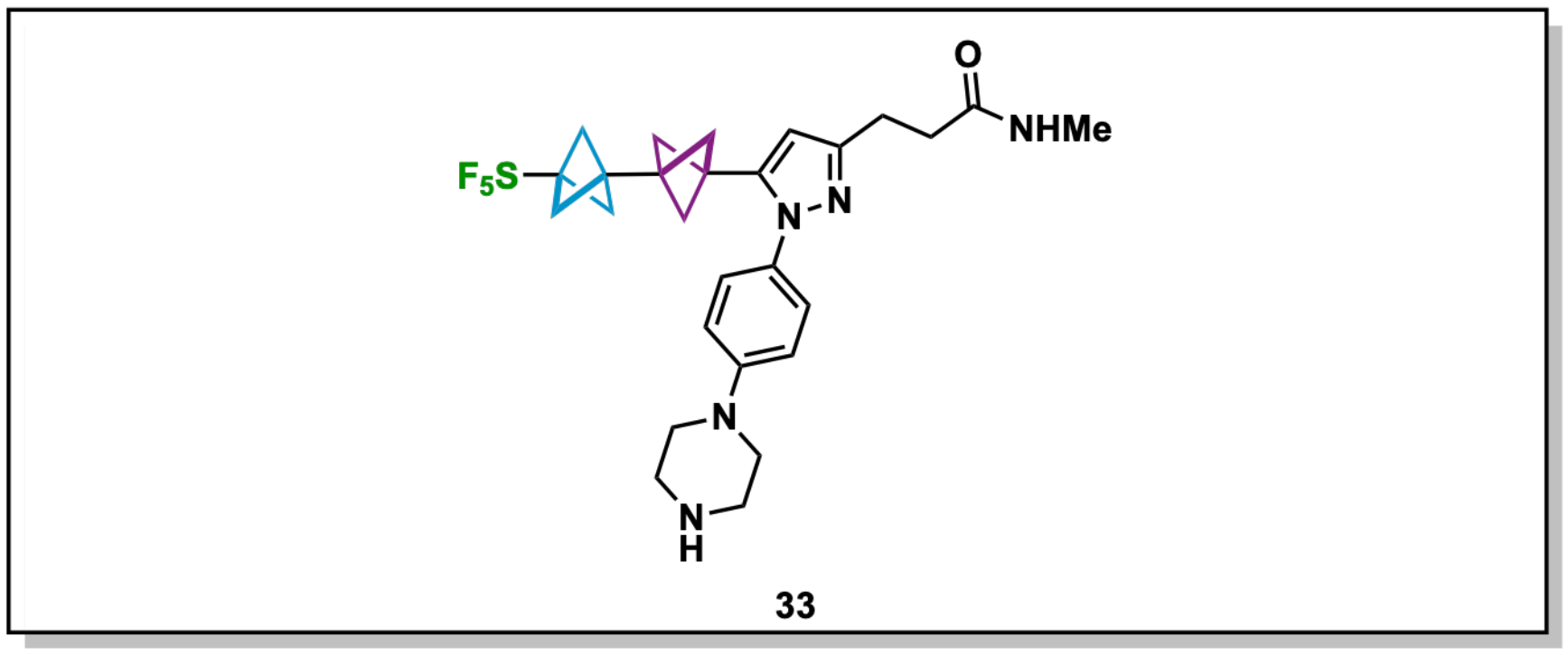

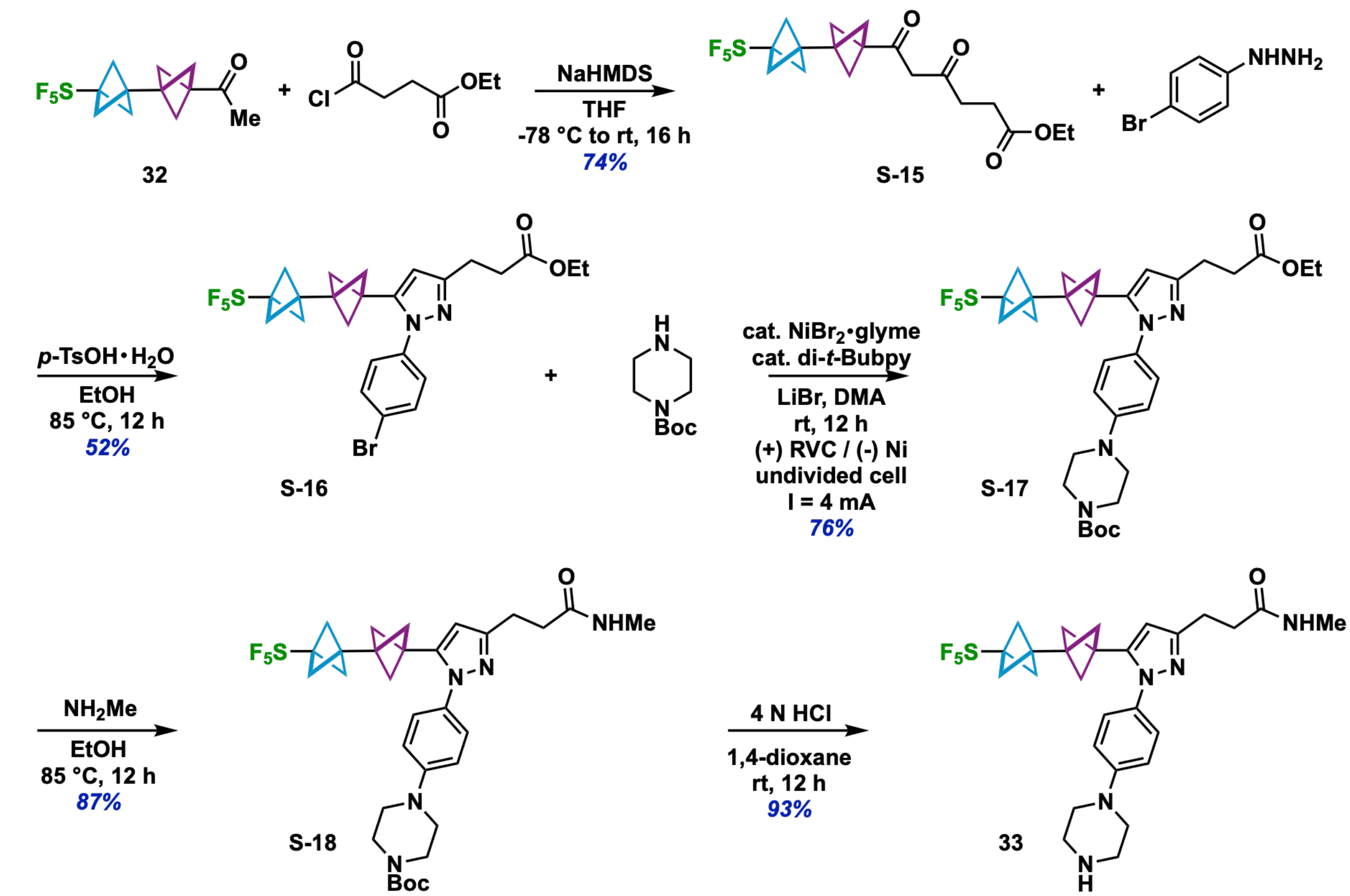

Scheme S7: Synthesis overview of the SF_5–[2]staffane derivative of OSU-T315 (**33**).[10] *See pages S48-S52 for detailed procedures and corresponding characterization data.*

Compound S-15

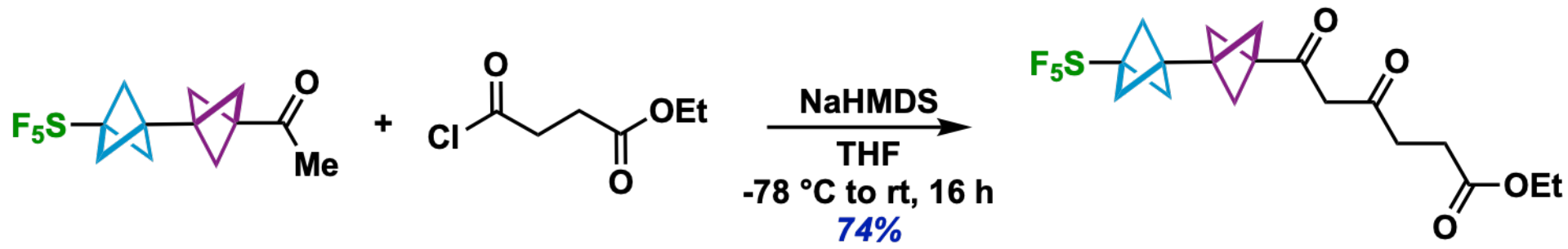

The synthesis of compound **S-15** was adapted from a known literature procedure.[10] To an oven-dried 20 mL microwave vial equipped with a stir bar was added compound **32** (0.300 g, 0.990 mmol, 1.0 equiv.). The vial was sealed, evacuated, and backfilled with Ar three times. Anhydrous THF (12 mL) was added *via* syringe. The resulting mixture was cooled to -78 °C, a 2.0 M solution of sodium bis(trimethylsilyl)amide in THF (1.49 mL, 2.97 mmol, 3.0 equiv.) was added dropwise, and the reaction mixture stirred at -78 °C for 30 min. Subsequently, ethyl 4-chloro-4-oxobutanoate (0.210 mL, 1.49 mmol, 1.5 equiv.) was added dropwise, and the reaction mixture was warmed to room temperature. After 16 h, the reaction was quenched with sat. aq. NH_4Cl (10 mL). The aqueous layer was extracted with EtOAc (3 x 10 mL). The combined organic layers were washed with brine (3 x 25 mL), dried over anhydrous Na_2SO_4, filtered through Celite, and concentrated. The crude residue was purified *via* column chromatography on silica gel eluting with 4:1 hexanes:EtOAc to afford a light yellow solid (0.314 g, 74%).

ethyl 4,6-dioxo-6-(3'-(pentafluoro-λ^6-sulfanyl)-[1,1'-bi(bicyclo[1.1.1]pentan)]-3-yl)hexanoate (**S-15**):
R_f: 0.30 (4:1 hexanes:EtOAc), vis: UV (254 nm).
m.p.: 80.9–81.0 ºC.
^{1}H (300 MHz, $CDCl_3$): δ = 14.79 (s, 1H), 5.47 (s, 1H), 4.17-4.10 (q, 2H, $^3J_{H\text{-}H}$ = 7.05 Hz), 2.70-2.57 (m, 4H), 2.18 (s, 6H), 1.88 (s, 6H), 1.27-1.22 (t, 3H, $^3J_{H\text{-}H}$ = 7.05 Hz).
^{19}F (282 MHz, $CDCl_3$): δ = 80.40-78.34 (p, 1F, $^2J_{F\text{-}F}$ = 145.6 Hz), 48.43-47.91 (d, 4F, $^2J_{F\text{-}F}$ = 145.6 Hz).
^{13}C{^{1}H} (151 MHz, $CDCl_3$): δ = 198.1, 183.0, 172.4, 97.5, 66.6-65.8 (m), 60.8, 54.3, 50.7, 50.5, 39.7, 34.8, 34.6, 28.8, 14.2.
ATR-IR (ν_{max}): 2979, 2920, 2883, 1722, 1613, 1213, 1169, 1123, 903, 818, 792, 758 cm^{-1}.
HRMS (*m/z*): (ESI) calc'd for $C_{18}H_{24}F_5O_4S^+$ [M-H]$^+$: calculated 431.1310, found 431.1319.

Compound S-16

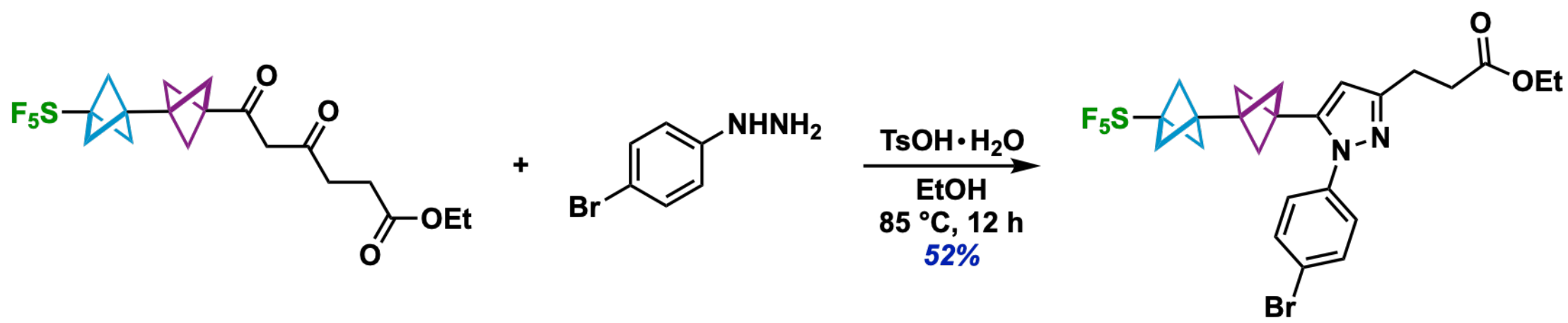

S-15 S-16

The synthesis of compound **S-16** was adapted from a known literature procedure.[10] To an oven-dried microwave vial equipped with a stir bar were added compound **S-15** (0.227 g, 0.527 mmol, 1.0 equiv.), 4-bromophenylhydrazine hydrochloride (0.141 g, 0.633 mmol, 1.2 equiv.), and *p*-toluenesulfonic acid monohydrate (0.100 g, 0.527 mmol, 1.0 equiv.). The vial was sealed, evacuated, and backfilled with Ar three times. Anhydrous EtOH (4.0 mL) was added *via* syringe. The resulting mixture was heated to 85 °C in an oil bath and refluxed for 12 h. Upon reaction completion, the vial was cooled to room temperature and concentrated. The crude residue was purified *via* column chromatography on silica gel eluting with 4:1 hexanes:EtOAc to afford an orange-yellow oil (0.159 g, 52%).

ethyl 3-(1-(4-bromophenyl)-5-(3'-(pentafluoro-λ^6-sulfanyl)-[1,1'-bi(bicyclo[1.1.1]pentan)]-3-yl)-1*H*-pyrazol-3-yl)propanoate (**S-16**):

R_f: 0.33 (4:1 hexanes:EtOAc), vis: UV (254 nm).

^{1}H (300 MHz, $CDCl_3$): δ = 7.59-7.56 (m, 2H), 7.31-7.28 (m, 2H), 6.02 (s, 1H), 4.16-4.08 (q, 2H, $^3J_{H\text{-}H}$ = 7.05 Hz), 2.93-2.88 (m, 2H), 2.60-2.55 (m, 2H), 2.21 (s, 6H), 1.98 (s, 6H), 1.25-1.20 (t, 3H, $^3J_{H\text{-}H}$ = 7.05 Hz).

^{19}F (282 MHz, $CDCl_3$): δ = 80.64-78.58 (p, 1F, $^2J_{F\text{-}F}$ = 145.6 Hz), 48.38-47.87 (d, 4F, $^2J_{F\text{-}F}$ = 145.6 Hz).

^{13}C{^{1}H} (151 MHz, $CDCl_3$): δ = 172.0, 152.3, 142.8, 138.6, 132.4, 127.2, 121.8, 103.9, 66.8-66.1 (p, $^2J_{C\text{-}F}$ = 17.4 Hz), 60.8, 54.4, 51.8, 37.8, 35.7, 35.0, 33.1, 21.7, 14.2.

ATR-IR (ν_{max}): 2976, 2925, 2872, 1732, 1494, 1372, 1211, 1181, 1166, 1008, 902, 820, 791 cm^{-1}.

HRMS (*m/z*): (ESI) calc'd for $C_{24}H_{27}BrF_5N_2O_2S^+$ [M-H]$^+$: calculated 581.08913, found 581.0885.

Compound S-17

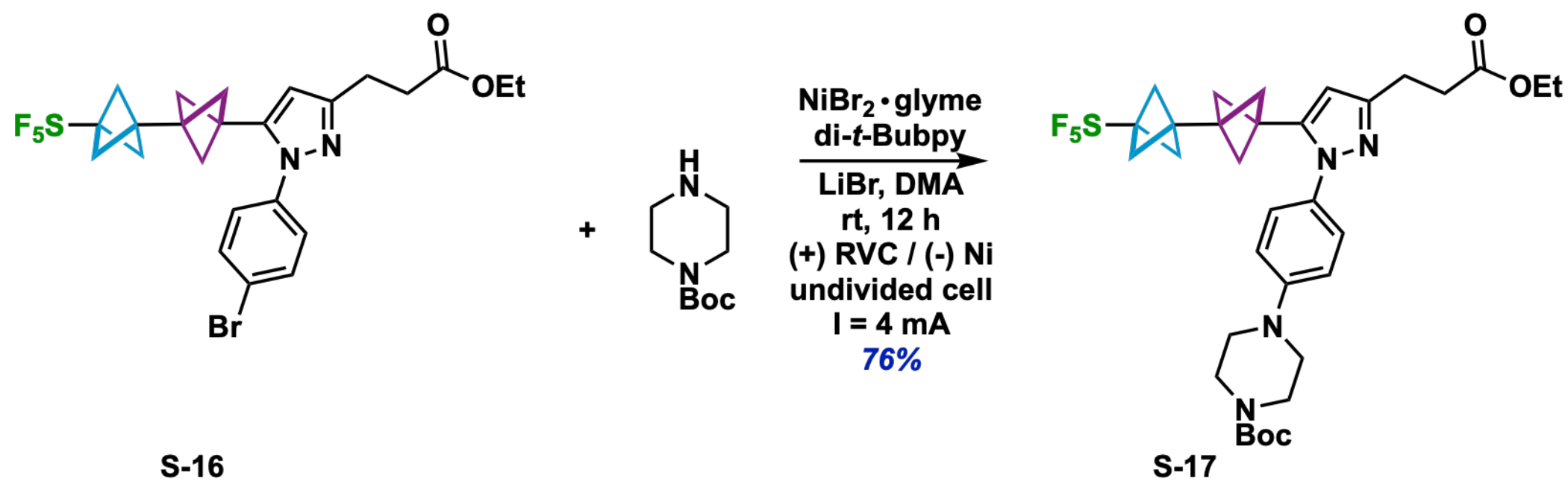

The synthesis of compound **S-17** was adapted from a known literature procedure.[11] An ElectraSyn vial (5 mL) with a stir bar was charged with compound **S-16** (0.116 g, 0.200 mmol, 1.0 equiv.) and *tert*-butyl piperazine-1-carboxylate (0.112 g, 0.600 mmol, 3.0 equiv.). The vial was sealed with the EletraSyn vial cap—equipped with anode (RVC) and cathode (Ni foam)—was inserted into the mixture, evacuated, and backfilled with Ar three times. The solids were dissolved in anhydrous DMA (1.35 mL), followed by the addition of a 2.0 M solution of LiBr in DMA (0.400 mL, 0.200 mmol, 4.0 equiv.) and a 10 mol % nickel bromide dimethoxyethane adduct and 4,4′-di-tert-butyl-2,2′-dipyridine solution (0.250 mL, 0.020 mmol, 0.1 equiv.) The reaction mixture was electrolyzed under a constant current of 4 mA until complete consumption of the starting material was observed by TLC. Upon reaction completion, the EletraSyn vial cap was removed, and the electrodes were rinsed with EtOAc. Sat. aq. NH_4Cl (10 mL) was then added, and the resulting mixture was extracted with EtOAc (3 x 5 mL). The combined organic layers were washed with brine (3 x 10 mL), dried over anhydrous Na_2SO_4, filtered through Celite, and concentrated. The crude residue was purified *via* column chromatography on silica gel eluting with 10% TEA in 4:1 hexanes:EtOAc to afford a clear oil (53.0 mg, 76%).

tert-butyl 4-(4-(3-(3-ethoxy-3-oxopropyl)-5-(3'-(pentafluoro-λ^6-sulfanyl)-[1,1'-bi(bicyclo[1.1.1]pentan)]-3-yl)-1*H*-pyrazol-1-yl)phenyl)piperazine-1-carboxylate (**S-17**):
R_f: 0.46 (10% TEA in 4:1 hexanes:EtOAc), vis: UV (254 nm).
^{1}H (300 MHz, $CDCl_3$): δ = 7.27-7.25 (m, 2H), 6.95-6.92 (m, 2H), 5.98 (s, 1H), 4.14-4.07 (q, 2H, $^3J_{H-H}$ = 7.05 Hz), 3.60-3.57 (m, 4H), 3.18-3.14(m, 4H), 2.89-2.84 (m, 2H), 2.57-2.52 (m, 2H), 2.20 (s, 6H), 1.98 (s, 6H),1.49 (s, 9H), 1.24-1.20 (t, 3H, $^3J_{H-H}$ = 7.05 Hz).
^{19}F (282 MHz, $CDCl_3$): δ = 80.68-78.63 (p, 1F, $^2J_{F-F}$ = 145.6 Hz), 48.36-47.85 (d, 4F, $^2J_{F-F}$ = 145.6 Hz).
^{13}C{^{1}H} (151 MHz, $CDCl_3$): δ = 172.35, 154.80, 151.48, 151.10, 142.78, 131.90, 126.89, 116.72, 102.97, 80.18, 66.87-66.43 (m), 60.80, 54.43, 51.89, 49.27, 37.76, 35.86, 35.12, 33.20, 28.55, 28.43, 21.64, 14.31.
ATR-IR (ν_{max}): 2975, 2925, 2870, 2360, 2341, 1735, 1692, 1519, 1420, 1366, 1232, 1211, 1165, 1122, 902, 827, 796, 731 cm^{-1}.
HRMS (*m/z*): (ESI) calc'd for $C_{33}H_{44}F_5N_4O_4S^+$ [M-H]$^+$: calculated 687.2997, found 687.2989.

Compound S-18

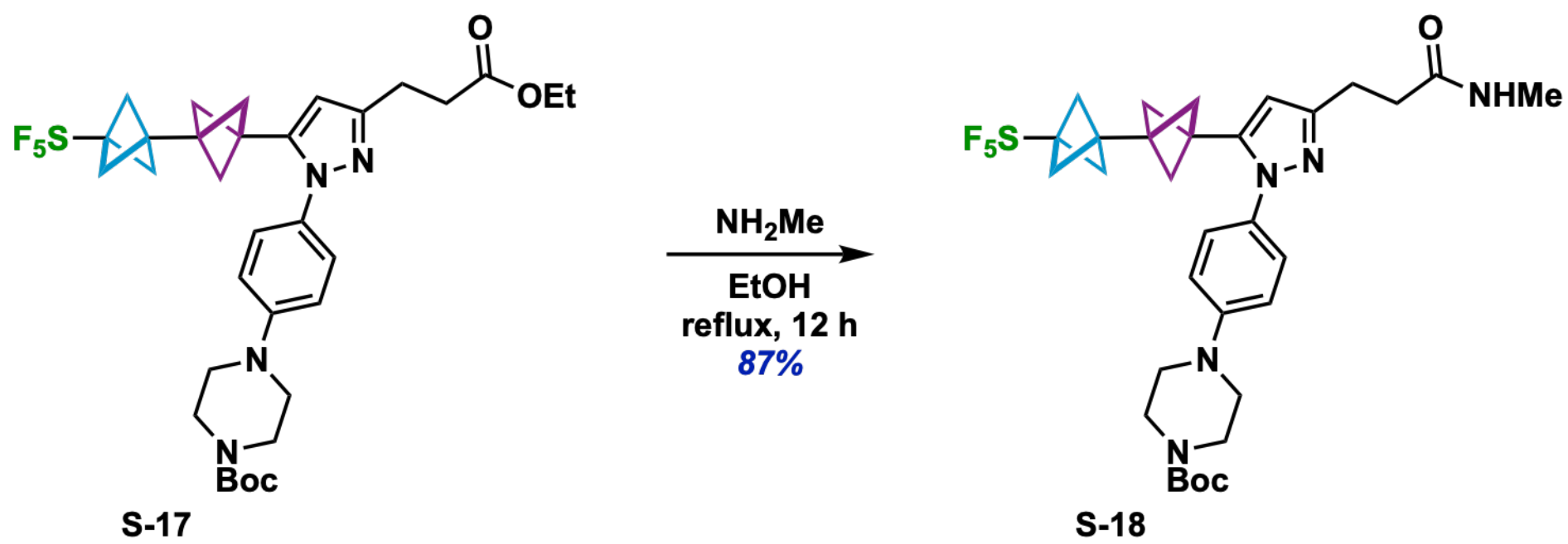

The synthesis of compound **S-18** was adapted from a known literature procedure.[10] To an oven-dried microwave vial equipped with a stir was added compound **S-17** (20.0 mg, 0.029 mmol, 1.00 equiv.). The vial was sealed, evacuated, and backfilled with Ar three times. To the vial, was added a 33 wt. % methylamine in ethanol (0.5 mL) and heated to 85 °C in an oil bath and refluxed for 12 h. Upon reaction completion, the reaction was cooled to room temperature and concentrated. The crude residue was purified *via* preparatory TLC eluting with 10% TEA in 2:3 hexanes:EtOAc to afford a white solid (17.0 mg, 87%).

tert-butyl 4-(4-(3-(3-(methylamino)-3-oxopropyl)-5-(3'-(pentafluoro-λ^6-sulfanyl)-[1,1'-bi(bicyclo[1.1.1] pentan)]-3-yl)-1*H*-pyrazol-1-yl)phenyl)piperazine-1-carboxylate (**S-18**):

R_f: 0.26 (10% TEA in 2:3 hexanes:EtOAc), vis: UV (254 nm).

m.p.: 104.8–106.8 ºC.

^{1}H (300 MHz, $CDCl_3$): δ = 7.27-7.24 (m, 2H), 6.94-6.91 (m, 2H), 5.96 (s, 1H), 5.41-5.36 (m, 1H), 3.60-3.57 (m,4H), 3.17-.3.14 (m, 4H), 2.92-2.87 (m, 2H), 2.78-2.76 (d, 3H, $^3J_{H-H}$ = 5.04 Hz), 2.40-2.35 (m, 2H), 2.20 (s, 6H), 1.97 (s, 6H), 1.48 (s, 9H).

^{19}F (282 MHz, $CDCl_3$): δ = 80.68-78.62 (p, 1F, $^2J_{F-F}$ = 145.6 Hz), 48.36-47.85 (d, 4F, $^2J_{F-F}$ = 145.6 Hz).

^{13}C{^{1}H} (151 MHz, $CDCl_3$): δ = 171.8, 154.8, 151.5, 151.0, 143.1, 131.8, 126.8, 116.7, 102.9, 80.1, 66.6-66.3 (m), 54.4, 51.9, 49.2, 35.8, 35.2, 35.1, 28.5, 26.4, 22.1.

ATR-IR (ν_{max}): 3304, 2972, 2924, 2870, 2360, 2341, 1692, 1658, 1519, 1232, 1166, 1125, 1050, 903, 829, 796, 730 cm^{-1}.

HRMS (*m/z*): (ESI) calc'd for $C_{32}H_{43}F_5N_5O_3S^+$ [M-H]$^+$: calculated 672.3001, found 672.2995.

Compound 33

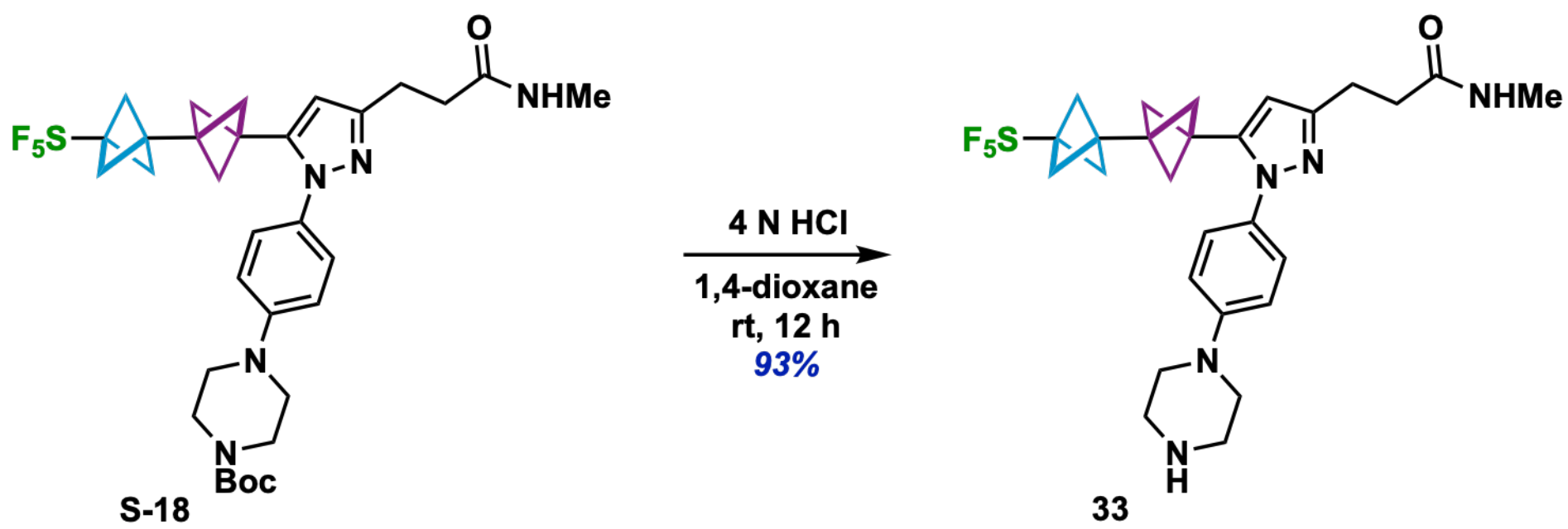

To an oven-dried microwave vial equipped with a stir bar was added compound **S-18** (17.0 mg, 0.025 mmol, 1.0 equiv.). The vial was sealed, evacuated, and backfilled with Ar three times. A 4 N solution of HCl in 1,4-dioxane (0.3 mL) was added, and the reaction mixture was stirred at room temperature for 12 h. Upon reaction completion, the crude reaction mixture was diluted with CH_2Cl_2 (1 mL) and transferred to a separatory funnel. Sat. aq. $NaHCO_3$ (3 x 3 mL) was then added, and the mixture was extracted with CH_2Cl_2 (3 x 1 mL). The combined organic layer was dried over anhydrous Na_2SO_4, filtered through Celite, and concentrated. The crude residue was purified *via* preparatory TLC eluting with 19:1 CH_2Cl_2:7 M NH_3 in MeOH to afford a white solid (13.0 mg, 92%).

N-methyl-3-(5-(3'-(pentafluoro-λ^6-sulfanyl)-[1,1'-bi(bicyclo[1.1.1]pentan)]-3-yl)-1-(4-(piperazin-1-yl)phenyl)-1*H*-pyrazol-3-yl)propenamide (**33**):

R$_f$: 0.13 (19:1 CH_2Cl_2:7 M NH_3 in MeOH), vis: UV (254 nm).

m.p.: 167.5–170.5 ºC.

^{1}H (300 MHz, CDCl$_3$): δ = 7.25-7.22 (m, 2H), 6.94-6.91 (m, 2H), 5.96 (s, 1H), 5.40-5.34 (m, 1H), 3.18-3.14 (m, 4H), 3.05-3.02 (m, 4H), 2.93-2.87 (m, 2H), 2.77-2.76 (d, 3H, $^3J_{H\text{-}H}$ = 4.91Hz), 2.39-2.34 (m, 2H), 2.20 (s, 6H), 1.97 (s, 6H).

^{19}F (282 MHz, CDCl$_3$): δ = 80.70-78.63 (p, 1F, $^2J_{F\text{-}F}$ = 145.6 Hz), 48.37-47.85 (d, 4F, $^2J_{F\text{-}F}$ = 145.6 Hz).

^{13}C{^{1}H} (151 MHz, CDCl$_3$): δ = 171.8, 151.6, 151.4, 143.1, 131.3, 126.7, 116.1, 102.9, 66.9-66.3 (m), 54.42, 51.9, 50.1, 46.0, 37.7, 35.8, 35.2, 35.1, 26.4, 22.1.

ATR-IR (ν_{max}): 3282, 2972, 2926, 2871, 2828, 2215, 1641, 1548, 1518, 1452, 1373, 1236, 1211, 1051, 902, 832, 817, 794, 725, 644 cm^{-1}.

HRMS (*m/z*): (ESI) calc'd for $C_{27}H_{35}F_5N_5OS^+$ [M-H]$^+$: calculated 572.2477, found 572.2476.

Synthesis Overview of SF_5–[2]staffane PROT inhibitor Derivatives (37–40)

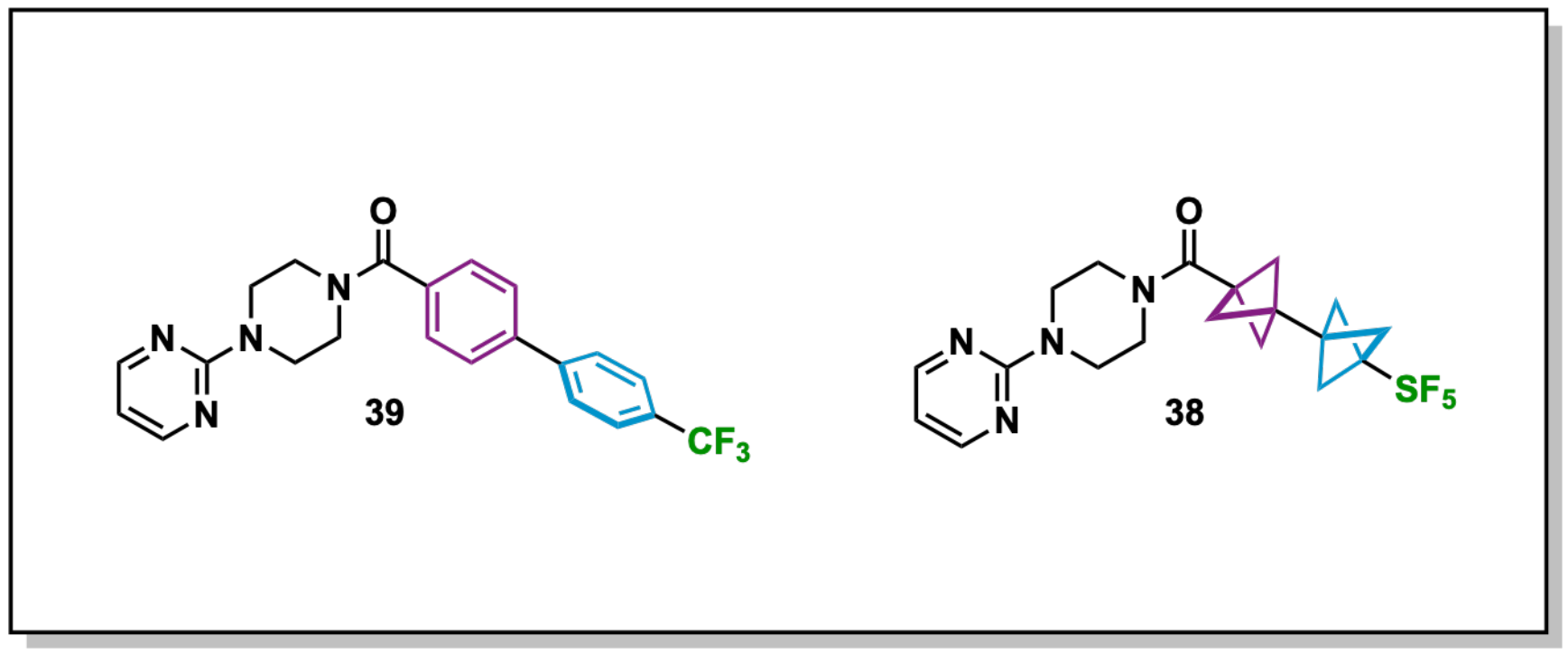

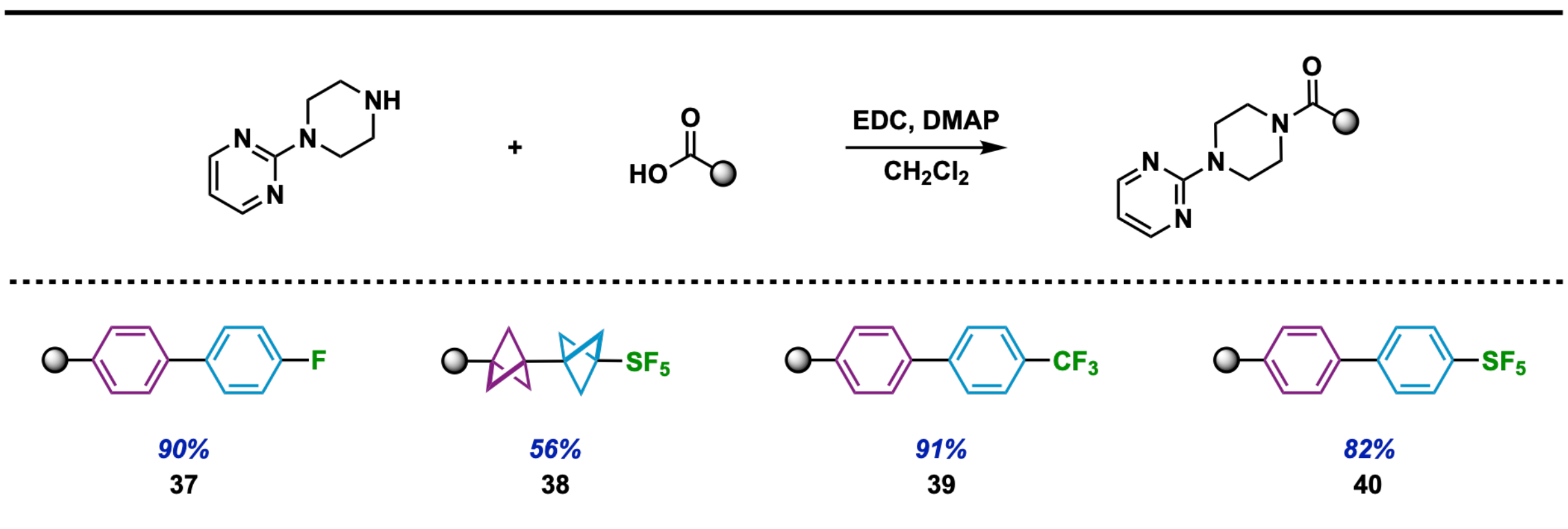

Scheme S8: Synthesis overview of CF_3–PROT inhibitor (**39**) and various derivatives.[12] *See pages S54-S63 for detailed procedures and corresponding characterization data.*

Compound S-19

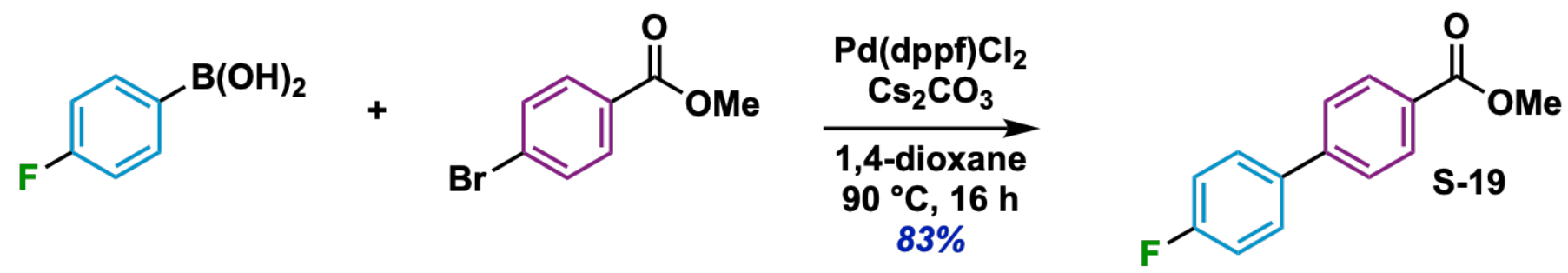

To an oven-dried microwave vial equipped with a stir bar was added methyl-4-bromobenzoate (430 mg, 2.00 mmol, 1.0 equiv.), 4-fluorophenylboronic acid (336 mg, 2.40 mmol, 1.2 equiv.), Pd(dppf)Cl_2 (14.6 mg, 0.02 mmol, 0.01 equiv.), and Cs_2CO_3 (1.63 g, 5.00 mmol, 2.5 equiv.). The vial was sealed with a cap with a septum using a crimper, then evacuated and backfilled with Ar three times. Anhydrous 1,4-dioxane (4.3 mL) was added *via* syringe and the resulting reaction mixture sparged with Ar for 10 mins. The reaction mixture was stirred at 90 °C in an oil bath for 16 h. Upon reaction completion, the reaction mixture was cooled to room temperature, diluted with EtOAc (3 mL) and filtered through celite. The filtrate was then transferred to a separatory funnel and washed with H_2O (3 x 40 mL) followed by brine (3 x 40 mL) and dried over anhydrous Na_2SO_4, filtered through Celite, and concentrated. The crude residue was then purified *via* column chromatography on silica gel eluting with 40:1 hexanes:EtOAc to afford a white solid (386 mg, 83%).

methyl 4'-fluoro-[1,1'-biphenyl]-4-carboxylate (**S-19**):
R_f: 0.32 (20:1 hexanes:EtOAc), vis: UV (254 nm).
m.p.: 100.4–101.7 ºC.
^{1}H (300 MHz, $CDCl_3$): δ = 8.09 (d, 2H, $^3J_{H\text{-}H}$ = 8.5 Hz), 7.64-7.50 (m, 4H), 7.13 (t, 2H, $^3J_{H\text{-}H}$ = 8.5 Hz), 3.93 (s, 3H).
^{19}F (282 MHz, $CDCl_3$): δ = -114.18 – -114.28 (m, 1F).
^{13}C{^{1}H} (76 MHz, $CDCl_3$): δ = 166.9, 163.0 (d, $^1J_{C\text{-}F}$ = 247.9 Hz), 144.6, 136.2 (d, $^4J_{C\text{-}F}$ = 3.3 Hz), 130.2, 129.0, 129.0 (d, $^3J_{C\text{-}F}$ = 8.2 Hz), 126.9, 115.9 (d, $^2J_{C\text{-}F}$ = 21.6 Hz), 52.2.
ATR-IR (ν_{max}): 2958, 1721, 1432, 1283, 1269, 1252, 1192, 1110, 832, 770 cm^{-1}.
HRMS (*m/z*): (ESI) calc'd for $C_{14}H_{12}FO_2^+$ [M-H]$^+$: calculated 231.0816, found 231.0810.

Compound 34

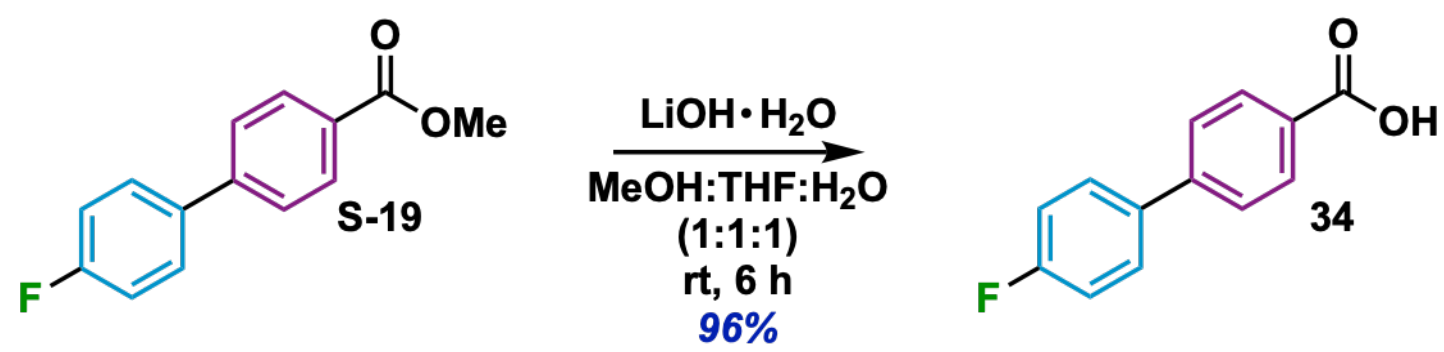

To an oven-dried microwave vial equipped with a stir bar was added compound **S-19** (138 mg, 0.600 mmol, 1.0 equiv.) followed by a solution of H_2O:MeOH:THF (6 mL). Subsequently, lithium hydroxide monohydrate (57.2 mg, 1.2 mmol, 2.0 equiv.) was added in one portion, and the vial was sealed with a septum and stirred at room temperature for 6 h. Upon reaction completion, as indicated by TLC, the reaction mixture was carefully acidified with 1 M HCl to ca. pH 4-5, and the aqueous layer was extracted with EtOAc (3 x 2 mL). The combined organic layers were washed with H_2O (3 x 40 mL), brine (3 x 40 mL), dried over anhydrous Na_2SO_4, filtered through Celite, and concentrated. The resulting crude residue was washed with MeOH (2.0 mL) to afford a white solid (124 mg, 96%).

4'-fluoro-[1,1'-biphenyl]-4-carboxylic acid (**34**):
R_f: 0.31 (3:2 hexanes:EtOAc), vis: UV (254 nm).
m.p.: 101.1–105.5 ºC.
^{1}H (300 MHz, $CDCl_3$): δ = 12.99 (s, 1H), 8.01 (d, 2H, $^3J_{H-H}$ = 8.9 Hz), 7.75-7.8 (m, 4H), 7.32 (t, 2H, $^3J_{H-H}$ = 8.9 Hz).
^{19}F (283 MHz, $CDCl_3$): δ = -114.17 – -114.27 (m, 1F).
^{13}C{^{1}H} (101 MHz, $CDCl_3$): δ = 167.2, 162.4 (d, $^1J_{C-F}$ = 247.5 Hz), 143.3, 135.6 (d, $^4J_{C-F}$ = 3.1 Hz), 130.1, 129.7, 129.1 (d, $^3J_{C-F}$ = 8.4 Hz), 126.8, 116.0 (d, $^2J_{C-F}$ = 21.3 Hz).
ATR-IR (ν_{max}): 3070, 2962, 2872, 2662, 2545, 1080, 1600, 1427, 1296, 1227, 823, 771, 713 cm^{-1}.
HRMS (*m/z*): (ESI) calc'd for $C_{13}H_{10}FO_2^+$ $[M-H]^+$: calculated 217.0659, found 217.0653.

Compound 37

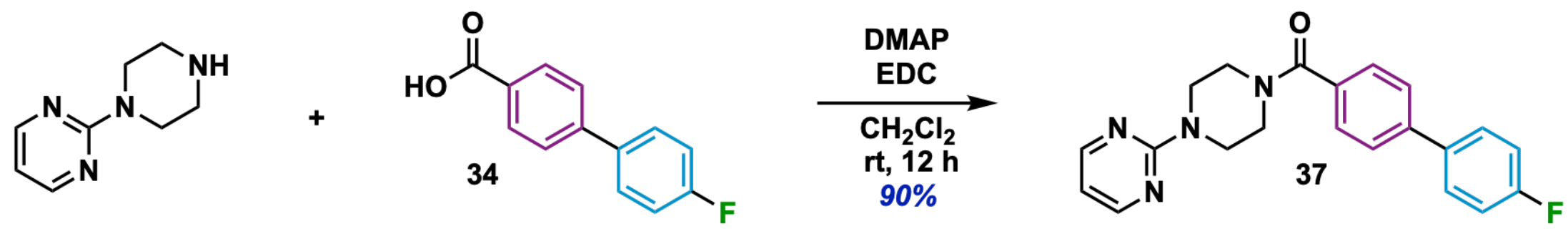

The synthesis of compound **37** was adapted from a known literature procedure.[12] To an oven-dried microwave vial equipped with a stir bar was added compound **34** (108 mg, 0.500 mmol, 1.0 equiv.), 2-(piperazin-1-yl)pyrimidine (98.5 mg, 0.600 mmol, 1.2 equiv.), DMAP (12.2 mg, 0.100 mmol, 0.2 equiv.), and EDC (239.6 mg, 1.25 mmol, 2.5 equiv.). The vial was sealed with a septum, then evacuated and backfilled with Ar three times. Subsequently, anhydrous CH_2Cl_2 (12 mL) was added *via* syringe. The resulting reaction mixture was stirred at room temperature for 12 h. Upon reaction completion, the reaction mixture was diluted with CH_2Cl_2 (5 mL). The organic layer was washed with H_2O (3 x 40 mL), brine (3 x 40 mL), dried over anhydrous Na_2SO_4, filtered through Celite, and concentrated. The crude residue was purified *via* column chromatography on silica gel eluting with 3:2 to 2:3 hexanes:EtOAc to afford a white solid (163 mg, 90%).

(4'-fluoro-[1,1'-biphenyl]-4-yl)(4-(pyrimidin-2-yl)piperazin-1-yl)methanone (**37**):
R_f: 0.34 (3:5 hexanes:EtOAc), vis: UV (254 nm).
m.p.: 201.4–205.1 ºC.
^{1}H (400 MHz, CDCl$_3$): δ = 8.29 (d, 2H, $^3J_{H-H}$ = 4.8 Hz), 7.59 – 7.44 (m, 6H), 7.10 (t, 2H, $^3J_{H-H}$ = 8.7 Hz), 6.50 (t, 1H, $^3J_{H-H}$ = 4.7 Hz), 3.84 (s, 6H), 3.55 (s, 2H).
^{19}F (283 MHz, CDCl$_3$): δ = -114.62 – -114.71 (m, 1 F).
^{13}C{^{1}H} (76 MHz, CDCl$_3$): δ = 170.4, 162.8 (d, $^1J_{C-F}$ = 248.5 Hz), 161.6, 157.9, 141.9, 136.4 (d, $^4J_{C-F}$ = 3.2 Hz), 134.5, 128.9 (d, $^3J_{C-F}$ = 8.2 Hz), 127.9, 127.2, 115.9 (d, $^2J_{C-F}$ = 21.5 Hz), 110.6, 47.7, 43.9, 42.3.
ATR-IR (ν_{max}): 2903, 2852, 1624, 1589, 1549, 1514, 1429, 1005, 982, 832, 791 cm^{-1}.
HRMS (*m/z*): (ESI) calc'd for $C_{21}H_{20}FN_4O^+$ [M-H]$^+$: calculated 363.1616, found 363.1593.

Compound 38

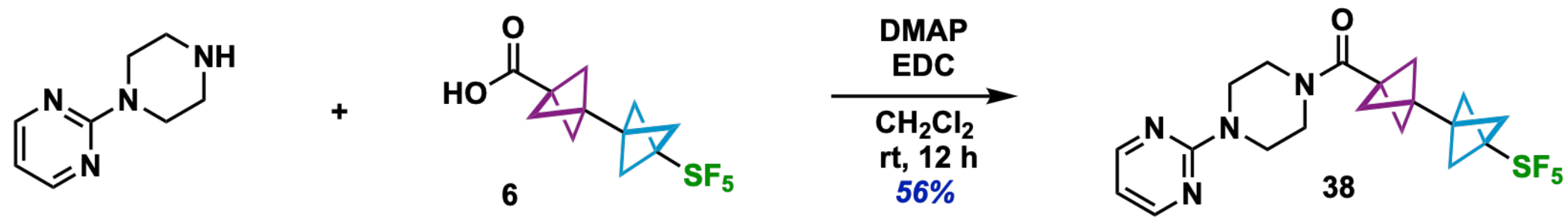

The synthesis of compound **38** was adapted from a known literature procedure.[12] To an oven-dried microwave vial equipped with a stir bar added with compound **6** (17.7 mg, 0.058 mmol, 1.0 equiv.), 2-(piperazin-1-yl)pyrimidine (14.3 mg, 0.087 mmol, 1.5 equiv.), DMAP (1.4 mg, 0.012 mmol, 0.2 equiv.), and EDC (27.9 mg, 0.145 mmol, 2.5 equiv.). The vial was sealed with a septum, then evacuated and backfilled with Ar three times. Anhydrous CH_2Cl_2 (5.0 mL) was added *via* syringe. The resulting reaction mixture was stirred at room temperature for 12 h. Upon reaction completion, the reaction mixture was diluted with CH_2Cl_2 (5 mL). The organic layer was washed with H_2O (3 x 40 mL), brine (3 x 40 mL), dried over anhydrous Na_2SO_4, filtered through Celite, and concentrated. The crude reside was purified *via* preparatory TLC eluting with 1:1 Hex:EtOAc to afford a white solid (15.4 mg, 56%).

(3'-(pentafluoro-λ^6-sulfaneyl)-[1,1'-bi(bicyclo[1.1.1]pentan)]-3-yl)(4-(pyrimidin-2-yl)piperazin-1-yl)methanone (**38**):

R_f: 0.2 (1:1 hexanes:EtOAc), vis: UV (254 nm).

m.p.: decomposes above 168.2 ºC.

^{1}H (400 MHz, $CDCl_3$): δ = 8.32 (s, 1H), 8.31 (s, 1H), 6.55-6.52 (m, 1H), 3.83-3.80 (m, 4H), 3.67-3.64 (m, 4H), 2.19 (s, 6H), 2.04 (s, 6H).

^{19}F (376 MHz, $CDCl_3$): δ = 80.10-78.55 (p, 1F, $^2J_{F\text{-}F}$ = 146.0 Hz), 48.39-48.00 (d, 4F, $^2J_{F\text{-}F}$ = 145.5 Hz).

^{13}C{^{1}H} (100 MHz, $CDCl_3$): δ = 168.0, 161.7, 157.9, 110.7, 66.4-66.1 (m), 54.4, 52.3, 45.4, 44.0, 43.6, 41.0, 39.4, 38.6, 34.8.

ATR-IR (ν_{max}): 2986, 2922, 2880, 2852, 1613, 824, 790 cm^{-1}.

HRMS (*m/z*): (ESI) calc'd for $C_{19}H_{24}F_5N_4OS^+$ $[M+H]^+$: calculated 451.1585, found 451.1577.

Compound S-20

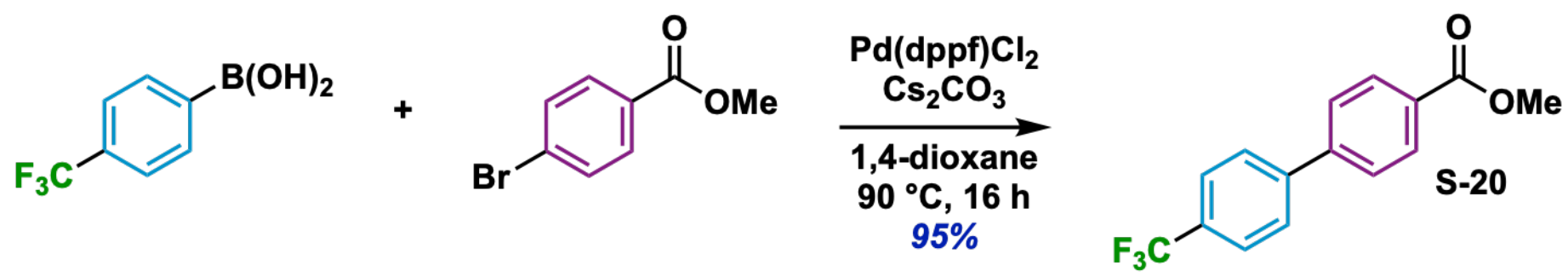

To an oven-dried microwave vial equipped with a stir bar was added compound methyl-4-bromobenzoate (430 mg, 2.00 mmol, 1.0 equiv.), 4-(Trifluoromethyl)phenylboronic acid (456 mg, 2.40 mmol, 1.2 equiv.), Pd(dppf)Cl_2 (15.0 mg, 0.02 mmol, 0.01 equiv.), and Cs_2CO_3 (1.63 g, 5.00 mmol, 2.5 equiv.). The vial was sealed with a cap with a septum with a crimper, then evacuated and backfilled with Ar three times. Anhydrous 1,4-dioxane (4.4 mL) was added *via* syringe, and the resulting reaction mixture was sparged with Ar for 10 mins. The reaction mixture was then stirred at 90 °C in an oil bath for 16 h. Upon reaction completion, the reaction mixture was cooled to room temperature, diluted with EtOAc (3 mL) and filtered through celite and the filtrate was transferred to a separatory funnel. The organic layer was washed with H_2O (3 x 40 mL), brine (3 x 40 mL), dried over anhydrous Na_2SO_4, filtered through Celite, and concentrated. The crude residue was purified *via* column chromatography on silica gel eluting with a gradient of 40:1 to 24:1 hexanes:EtOAc to afford a white solid (534 mg, 95%).

methyl 4'-(trifluoromethyl)-[1,1'-biphenyl]-4-carboxylate (**S-20**):
R_f: 0.38 (20:1 Hexanes:EtOAc), vis: UV (254 nm).
m.p.: 123.8–125.3 ºC.
^{1}H (400 MHz, $CDCl_3$): δ = 8.13 (d, 2H, $^3J_{H-H}$ = 8.4 Hz), 7.71 (s, 4H), 7.65 (d, 2H, $^3J_{H-H}$ = 8.4 Hz), 3.95 (s, 3H).
^{19}F (282 MHz, $CDCl_3$): δ = -62.54 (s, 3F).
^{13}C{^{1}H} (101 MHz, $CDCl_3$): δ = 166.8, 144.1, 143.6 (d, $^4J_{C-F}$ = 1.5 Hz), 130.4, 130.3 (q, $^2J_{C-F}$ = 32.3 Hz), 129.9, 127.7, 127.3, 126.0 (q, $^3J_{C-F}$ = 3.8 Hz), 124.3 (q, $^1J_{C-F}$ = 271.1 Hz), 52.3.
ATR-IR (ν_{max}): 2953.7, 1710.8, 1331.8, 1316.6, 1282.7, 1156.0, 1103.0, 1073.3, 1022.1, 1006.4, 830.7, 772.7, 738.6, 699.8, 666.9 cm^{-1}.
HRMS (*m/z*): (ESI) calc'd for $C_{15}H_{12}F_3O_2^+$ [M-H]$^+$: calculated 281.0784, found 281.0789.

Compound 35

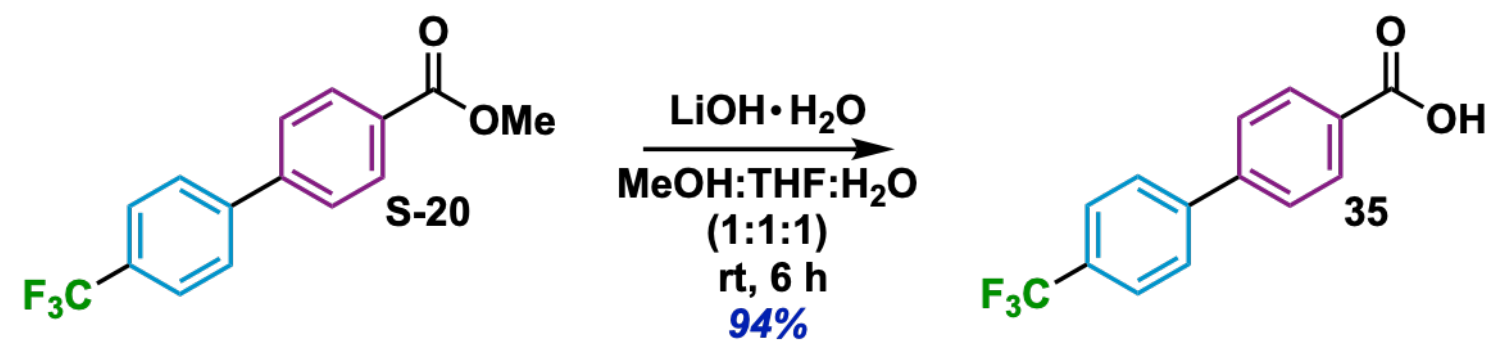

To an oven-dried microwave vial equipped with a stir bar was added compound **S-20** (280 mg, 1.00 mmol, 1.0 equiv.) followed by a solution of H_2O:MeOH:THF (9.6 mL). Subsequently, lithium hydroxide monohydrate (84 mg, 2.00 mmol, 2.0 equiv.) was added in one portion, and the vial was sealed with a septum. The reaction mixture was stirred vigorously at room temperature until the starting material was consumed as indicated by TLC. The reaction mixture was then carefully acidified with 1 M HCl to ca. pH 4-5, and the aqueous layer was extracted with EtOAc (3 x 2 mL). The combined organic layers were washed with H_2O (3 x 40 mL), brine (3 x 40 mL), dried over anhydrous Na_2SO_4, filtered through Celite, and concentrated. The crude residue was washed with MeOH (3.0 mL) to afford a white solid (250 mg, 94%).

4'-(trifluoromethyl)-[1,1'-biphenyl]-4-carboxylic acid (**35**):
R_f: 0.25 (2:1 hexanes:EtOAc), vis: UV (254 nm).
m.p.: decomposes above 284.5 ºC.
^{1}H (400 MHz, DMSO-d_6): δ = 13.09 (s, 1H), 8.06 (d, 2H, $^3J_{H-H}$ = 8.4 Hz), 7.96 (d, 2H, $^3J_{H-H}$ = 8.41 Hz), 7.88-7.84 (m, 4H).
^{19}F (376 MHz, DMSO-d_6): δ = -61.13 (s, 3F).
^{13}C{^{1}H} (101 MHz, DMSO-d_6): δ = 167.0, 143.0, 142.7, 130.6, 130.1, 128.6 (q, $^2J_{C-F}$ = 31.3 Hz), 127.8, 127.3, 125.9 (q, $^3J_{C-F}$ = 3.4 Hz), 124.3 (q, $^1J_{C-F}$ = 273.7 Hz).
ATR-IR (ν_{max}): 2962.2, 2930.9, 2829.0, 2673.5, 2546.5, 1685.7, 1608.4, 1427.4, 1317.3, 1292.4, 1161.1, 1117.1, 1069.0, 1006.4, 930.5, 833.1, 775.2, 736.9, 662.8.cm^{-1}.
HRMS (*m/z*): (ESI) calc'd for $C_{14}H_{10}F_3O_2^+$ [M-H]$^+$: calculated 267.0627, found 267.0635.

Compound 39

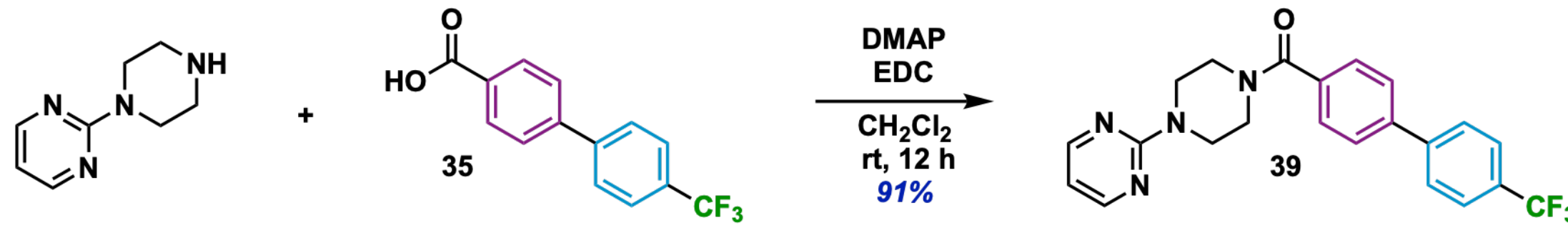

The synthesis of compound **39** was adapted from a known literature procedure.[12] To an oven-dried microwave vial equipped with a stir bar was added with compound **35** (100 mg, 0.380 mmol, 1.0 equiv.), 2-(piperazin-1-yl)pyrimidine (73.9 mg, 0.450 mmol, 1.2 equiv.), DMAP (9.8 mg, 0.08 mmol, 0.2 equiv.), and EDC (180 mg, 0.94 mmol, 2.5 equiv.). The vial was sealed with a septum, then evacuated and backfilled with Ar three times. Anhydrous CH_2Cl_2 (9.0 mL) was added *via* syringe. The resulting reaction mixture was stirred at room temperature for 12 h. Upon reaction completion, the reaction mixture was diluted with CH_2Cl_2 (5 mL). The organic layer was washed with H_2O (3 x 40 mL), brine (3 x 40 mL), dried over anhydrous Na_2SO_4, filtered through Celite, and concentrated. The crude residue was purified by *via* column chromatography on silica gel eluting with 1:1 hexanes:EtOAc to afford a white solid (141 mg, 91%).

(4-(pyrimidin-2-yl)piperazin-1-yl)(4'-(trifluoromethyl)-[1,1'-biphenyl]-4-yl)methanone (**39**):
R_f: 0.2 (1:1 hexanes:EtOAc), vis: UV (254 nm).
m.p.: 219.0–222.2 ºC.
^{1}H (300 MHz, $CDCl_3$): δ = 8.32 (d, $^3J_{H\text{-}H}$ = 4.8 Hz, 2H), 7.67 (d, $^3J_{H\text{-}H}$ = 18.1 Hz, 6H), 7.55 (d, $^3J_{H\text{-}H}$ = 8.2 Hz, 2H), 6.54 (t, $^3J_{H\text{-}H}$ = 4.8 Hz, 1H), 3.88 (s, 6H), 3.58 (s, 2H).
^{19}F (283 MHz, $CDCl_3$): δ = -62.44 (s, 3F).
^{13}C{^{1}H} (76 MHz, $CDCl_3$): δ = 170.2, 161.7, 157.9, 143.8 (q, $^4J_{C\text{-}F}$ = 1.5 Hz), 141.4, 135.4, 130.0 (q, $^2J_{C\text{-}F}$ = 32.6 Hz), 128.0, 127.6, 125.9 (q, $^3J_{C\text{-}F}$ = 3.8 Hz), 124.3 (q, $^1J_{C\text{-}F}$ = 272.0 Hz), 110.7, 47.6, 43.9, 42.3.
ATR-IR (ν_{max}): 2904, 2871, 1635, 1628, 1589, 1437, 1255, 1167, 1118, 1072, 826 cm^{-1}.
HRMS (*m/z*): (ESI) calc'd for $C_{22}H_{20}F_3N_4O^+$ [M-H]$^+$: calculated 413.1584, found 413.1569.

Compound S-21

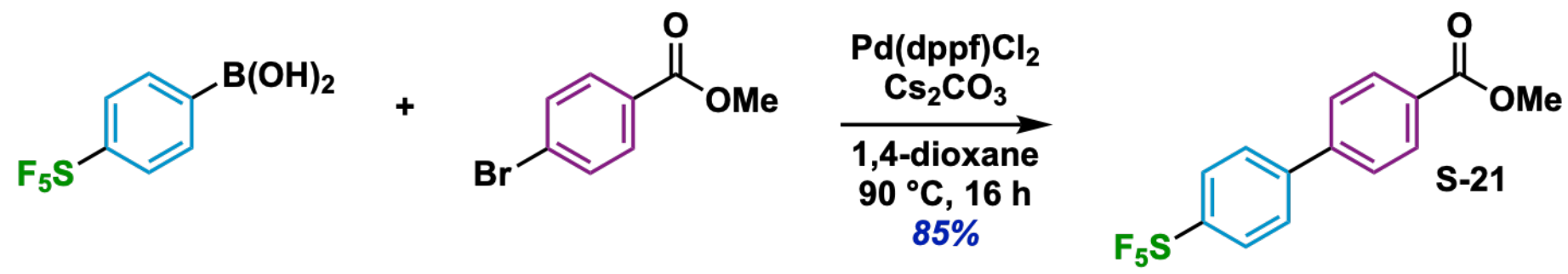

To an oven-dried microwave vial equipped with a stir bar was added compound methyl-4-bromobenzoate (258 mg, 1.20 mmol, 1.0 equiv.), 4-(4,4,5,5-tetramethyl-1,3,2-dioxaborolan-2-yl)-phenylsulfur pentafluoride (475 mg, 1.44 mmol, 1.2 equiv.), Pd(dppf)Cl_2 (9.0 mg, 0.012 mmol, 0.01 equiv.), and Cs_2CO_3 (975 mg, 3.00 mmol, 2.5 equiv.). The vial was sealed with a cap with a septum using a crimper, evacuated and backfilled with Ar three times. Anhydrous 1,4-dioxane (4.3 mL) was added *via* syringe, and the resulting reaction mixture was sparged with Ar for 10 min. The reaction mixture was heated to 90 °C in an oil bath for 16 h. Upon reaction completion, the reaction mixture was cooled to room temperature, diluted with EtOAc (3 mL), filtered through Celite, and the filtrate was transferred to a separatory funnel. The organic layer was washed with H_2O (3 x 10 mL), brine (3 x 10 mL), dried over anhydrous Na_2SO_4, filtered through Celite, and concentrated. The crude residue was purified *via* column chromatography on silica gel eluting with a gradient of 40:1 to 24:1 hexanes:EtOAc hexanes to afford a white solid (347 mg, 85%).

methyl 4'-(pentafluoro-λ^6-sulfaneyl)-[1,1'-biphenyl]-4-carboxylate (**S-21**):
R_f: 0.34 (20:1 hexanes:EtOAc), vis: UV (254 nm).
m.p.: 136.2–137.6 ºC.
^{1}H (300 MHz, $CDCl_3$): δ = 8.13 (d, 2H, $^3J_{H-H}$ = 8.3 Hz), 7.84 (d, 2H, $^3J_{H-H}$ = 8.7 Hz), 7.72-7.59 (m, 4H), 3.95 (s, 3H).
^{19}F (282 MHz, $CDCl_3$): δ = 84.28 (p, 1F, J = 150.0 Hz), 63.04 (d, 4F, J = 150.0 Hz).
^{13}C{^{1}H} (76 MHz, $CDCl_3$): δ = 166.8, 153.6 (p, $^2J_{C-F}$ = 17.5 Hz), 143.4, 130.4, 130.2, 127.6, 127.4, 126.7 (p, $^3J_{C-F}$ = 4.6 Hz), 52.4.
ATR-IR (ν_{max}): 2972, 1713, 1437, 1281, 1102, 819, 768, 750 cm^{-1}.
HRMS (*m/z*): (ESI) calc'd for $C_{14}H_{12}F_5O_2S^+$ $[M-H]^+$: calculated 339.0472, found 339.0462.

Compound 36

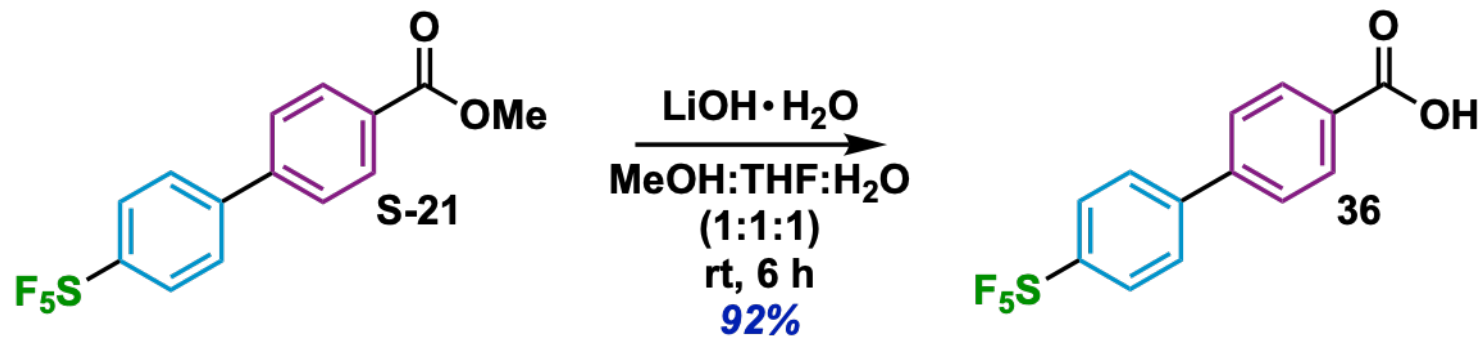

To an oven-dried microwave vial equipped with a stir bar was added compound **S-21** (169 mg, 0.50 mmol, 1.0 equiv.) followed by a solution of H_2O:MeOH:THF (4.8 mL). Subsequently, lithium hydroxide monohydrate (42 mg, 1.00 mmol, 2.0 equiv.) was added in one portion, sealed with a septum, the reaction mixture was stirred vigorously at room temperature until the starting material was consumed as indicated by TLC. The reaction mixture was then carefully acidified with 1 M HCl to ca. pH 4-5, and the aqueous layer was extracted with EtOAc (3 x 2 mL). The combined organic layers were washed with H_2O (3 x 40 mL), brine (3 x 40 mL), dried over anhydrous Na_2SO_4, filtered through Celite, and concentrated. The crude residue was washed with MeOH (3.0 mL) to afford a white solid (148 mg, 92%).

4'-(pentafluoro-λ^6-sulfaneyl)-[1,1'-biphenyl]-4-carboxylic acid (**36**):
R_f: 0.15 (3:2 hexanes:EtOAc), vis: UV (254 nm).
m.p.: 278.8–285.2 ºC.
^{1}H (300 MHz, DMSO-*d*$_6$): δ = 13.10 (s, 1H), 8.08-7.93 (m, 6H), 7.86 (d, 2H, $^3J_{H\text{-}H}$ = 8.4 Hz).
^{19}F (283 MHz, DMSO-*d*$_6$): δ = 87.18 (p, 1F, $^2J_{F\text{-}F}$ = 150.5 Hz), 64.20 (d, 4F, $^2J_{F\text{-}F}$ = 150.5 Hz).
^{13}C{^{1}H} (101 MHz, DMSO-*d*$_6$): δ = 167.0, 152.4 (p, $^2J_{C\text{-}F}$ = 16.1 Hz), 142.9, 142.0, 130.8, 130.1, 128.0, 127.4, 126.5 (p, $^3J_{C\text{-}F}$ = 4.4 Hz).
ATR-IR (ν_{max}): 2969, 2810, 2667, 2550, 1679, 1609, 1306, 806, 771, 651 cm^{-1}.
HRMS (*m/z*): (ESI) calc'd for $C_{13}H_{10}F_5O_2S^+$ [M-H]$^+$: calculated 325.0316, found 325.0299.

Compound 40

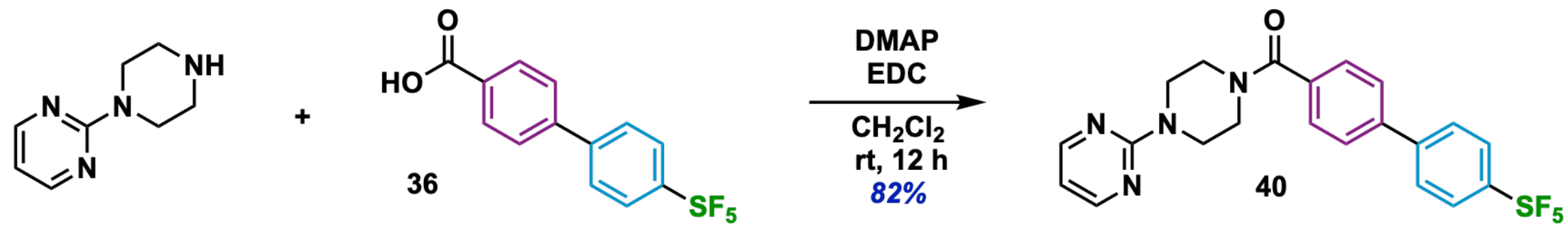

The synthesis of compound **40** was adapted from a known literature procedure.[12] To an oven-dried microwave vial equipped with a stir bar was added with compound **S-24** (140 mg, 0.430 mmol, 1.0 equiv.), 2-(piperazin-1-yl)pyrimidine (85.3 mg, 0.52 mmol, 1.2 equiv.), DMAP (11.0 mg, 0.09 mmol, 0.2 equiv.), and EDC (207.0 mg, 1.08 mmol, 2.5 equiv.). The vial was sealed with a septum evacuated and backfilled with Ar three times. Anhydrous CH_2Cl_2 (10.5 mL) was added *via* syringe. The resulting reaction mixture was stirred at room temperature for 12 h. Upon reaction completion, the reaction mixture was diluted with CH_2Cl_2 (5 mL). The organic layer was washed with H_2O (3 x 40 mL), brine (3 x 40 mL), dried over anhydrous Na_2SO_4, filtered through Celite, and concentrated. The crude residue was purified by *via* column chromatography on silica gel eluting with 1:1 hexanes:EtOAc to afford a white solid (165 mg, 82%).

(4'-(pentafluoro-λ^6-sulfaneyl)-[1,1'-biphenyl]-4-yl)(4-(pyrimidin-2-yl)piperazin-1-yl)methanone (**40**):
R_f: 0.31 (1:1 Hexanes:EtOAc), vis: UV (254 nm).
m.p.: 201.2–203.8 ºC.
^{1}H (400 MHz, $CDCl_3$): δ = 8.31 (d, 2H, *J* = 4.7 Hz), 7.85 – 7.78 (m, 2H), 7.64 (dd, 4H, $^3J_{H\text{-}H}$ = 8.9, 7.3 Hz), 7.57 – 7.50 (m, 2H), 6.52 (t, 1H, $^3J_{H\text{-}H}$ = 4.7 Hz), 3.86 (s, 6H), 3.55 (s, 2H).
^{19}F (283 MHz, $CDCl_3$): δ = 84.38 (p, 1F, $^2J_{F\text{-}F}$ = 149.9 Hz), 63.09 (d, 4F, $^2J_{F\text{-}F}$ = 149.9 Hz).
^{13}C{^{1}H} (101 MHz, $CDCl_3$): δ = 170.1, 161.6, 157.9, 153.3 (p, $^2J_{C\text{-}F}$ = 17.2 Hz), 143.5, 140.6, 135.7, 128.0, 127.6, 127.4, 126.6 (p, $^3J_{C\text{-}F}$ = 4.6 Hz), 110.7, 47.6, 43.9, 42.3.
ATR-IR (ν_{max}): 3020, 2980, 2869, 1626, 1585, 1551, 1497, 1437, 1253, 882, 824, 813, 766 cm^{-1}.
HRMS (*m/z*): (ESI) calc'd for $C_{21}H_{20}F_5N_4OS^+$ $[M\text{-}H]^+$: calculated 471.1272, found 471.1250.

Syntheses of Compounds 42–44 for Decomposition Study

Compound 42

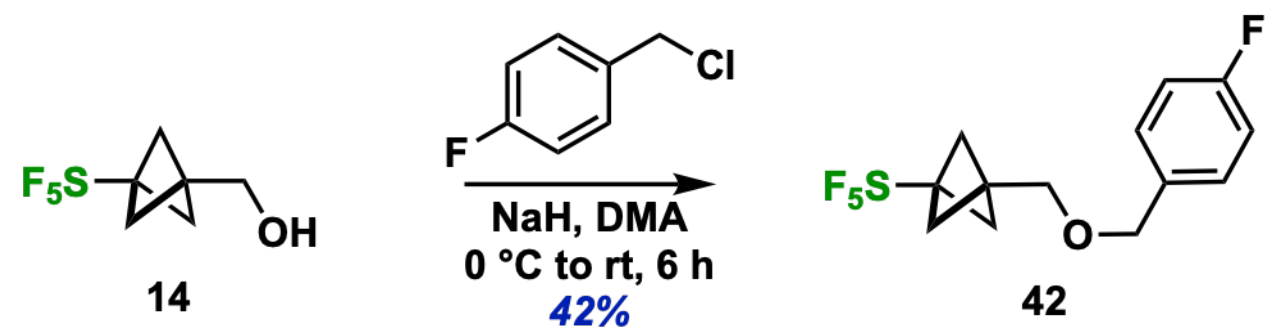

To an oven-dried microwave vial equipped with a stir bar was added compound **14** (71.0 mg, 0.317 mmol, 1.0 equiv.) and dissolved in DMA (1.58 mL). Then the mixture was cooled to 0 °C in an ice water bath. At 0 °C, sodium hydride 60% dispersion in mineral oil (11.0 mg, 0.475 mmol, 1.5 equiv.) was added, followed by 4-fluorobenzyl chloride (0.073 mL, 0.475, 1.5 equiv.). The reaction was stirred at 0 °C for 6 h. Upon reaction completion, the reaction was quenched with sat. aq. NH_4Cl (3 mL). The aqueous layer was extracted with EtOAc (3 x 3 mL). The combined organic layers were washed with brine (3 × 4 mL), dried over anhydrous Na_2SO_4, filtered through Celite, and concentrated. The crude residue was purified *via* column chromatography on silica gel eluting with 4:1 hexanes:CH_2Cl_2 to afford a clear oil (44.0 mg, 42%).

pentafluoro(3-(((4-fluorobenzyl)oxy)methyl)bicyclo[1.1.1]pentan-1-yl)- λ^6-sulfane (**42**):

R_f: 0.26 (4:1 hexanes:CH_2Cl_2), vis: UV (254 nm).

^{1}H (300 MHz, $CDCl_3$): δ = 7.30-7.25 (m, 2H), 7.07-7.01 (m, 2H), 4.48 (s, 2H), 3.56 (s, 2H), 2.28 (s, 6H).

^{19}F (282 MHz, $CDCl_3$): δ = 80.46-78.40 (p, 1F, $^2J_{F\text{-}F}$ = 143.6 Hz), 48.50-47.99 (d, 4F, $^2J_{F\text{-}F}$ = 143.6 Hz), -114.6– -114.7 (m, 1H).

^{13}C{^{1}H} (151 MHz, $CDCl_3$): δ = 164.2-160.9 (d, $^1J_{C\text{-}F}$ = 245.3 Hz), 133.9-133.8 (d, $^4J_{C\text{-}F}$ = 2.99 Hz), 129.4-129.3 (d, $^2J_{C\text{-}F}$ = 8.15 Hz), 115.6-115.3 (d, $^3J_{C\text{-}F}$ = 21.42 Hz), 72.6, 68.0-67.9 (m), 67.4-66.6 (m), 54.7-54.5 (m), 34.4.

ATR-IR (ν_{max}): 2988, 2928, 2894, 1601, 1508, 1359, 1223, 1186, 1091, 810, 788, 774 cm^{-1}.

HRMS (*m/z*): (ESI) calc'd for $C_{13}H_{15}F_6OS^+$ [M-H]$^+$: calculated 333.0742, found 333.0744.

Compound 43

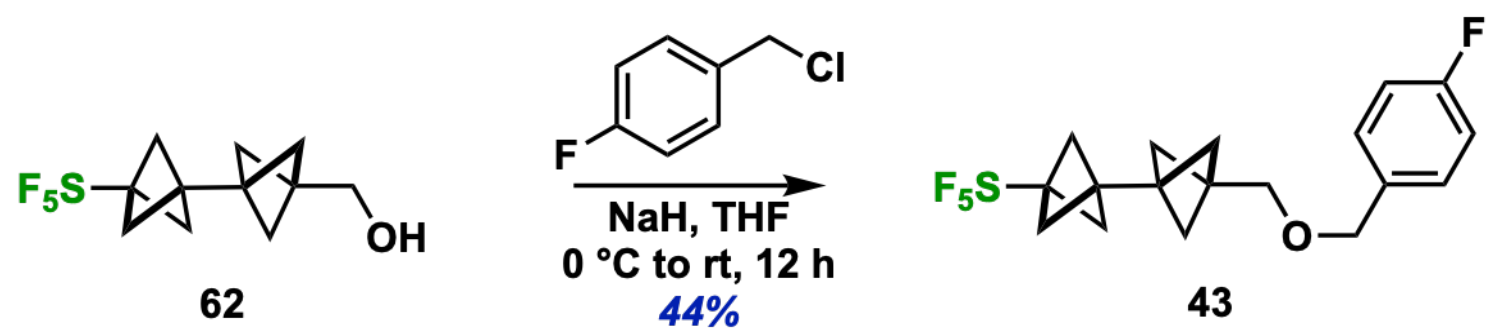

To an oven-dried microwave vial equipped with a stir bar was added compound **62** (0.100 g, 0.344 mmol, 1.0 equiv.) and THF (1.8 mL). Then, the reaction mixture was cooled to 0 °C in an ice water bath. At 0 °C, sodium hydride 60% dispersion in mineral oil (21.0 mg, 0.517 mmol, 1.5 equiv.) was added, followed by 4-fluorobenzyl chloride (0.058 mL, 0.379, 1.1 equiv.). The reaction mixture was stirred at room temperature for 12 h. Upon reaction completion, the reaction was quenched with sat. aq. NH_4Cl (3 mL). The aqueous layer was extracted with EtOAc (3 x 3 mL). The combined organic layers were washed with brine (3 x 4 mL), dried over anhydrous Na_2SO_4, filtered through Celite, and concentrated. The crude residue was purified *via* column chromatography on silica gel eluting with 4:1 hexanes:CH_2Cl_2 to afford a white solid (60.0 mg, 44%).

pentafluoro(3'-(((4-fluorobenzyl)oxy)methyl)-[1,1'-bi(bicyclo[1.1.1]pentan)]-3-yl)-λ^6-sulfane (**43**):
R_f: 0.16 (4:1 hexanes:CH_2Cl_2), vis: UV (254 nm).
m.p.: 58.1–59.9 ºC.
^{1}H (300 MHz, $CDCl_3$): δ = 7.30-7.25 (m, 2H), 7.05-6.99 (m, 2H), 4.47 (s, 1H), 3.43 (s, 1H), 2.16 (s, 6H), 1.63 (s, 6H).
^{19}F (282 MHz, $CDCl_3$): δ = 80.66-78.60 (p, 1F, $^2J_{F\text{-}F}$ = 143.6 Hz), 48.34-47.82 (d, 4F, $^2J_{F\text{-}F}$ = 143.6 Hz), -115.0– -115.1 (m, 1H).
^{13}C{^{1}H} (151 MHz, $CDCl_3$): δ = 164.0-160.8 (d, $^1J_{C\text{-}F}$ = 245.3 Hz), 134.4-134.4 (d, $^4J_{C\text{-}F}$ = 2.99 Hz), 129.3-129.2 (d, $^2J_{C\text{-}F}$ = 8.15 Hz), 115.4-115.2 (d, $^3J_{C\text{-}F}$ = 21.42 Hz), 72.4, 70.1, 66.8-66.1 (m), 54.4-54.2 (m), 49.0, 38.0, 35.1, 29.8.
ATR-IR (ν_{max}): 2981, 2915, 2878, 1509, 1210, 1090, 814, 783 cm^{-1}.
HRMS (*m/z*): (ESI) calc'd for $C_{18}H_{21}F_6OS^+$ $[M\text{-}H]^+$: calculated 399.1212, found 399.1207.

Compound 44

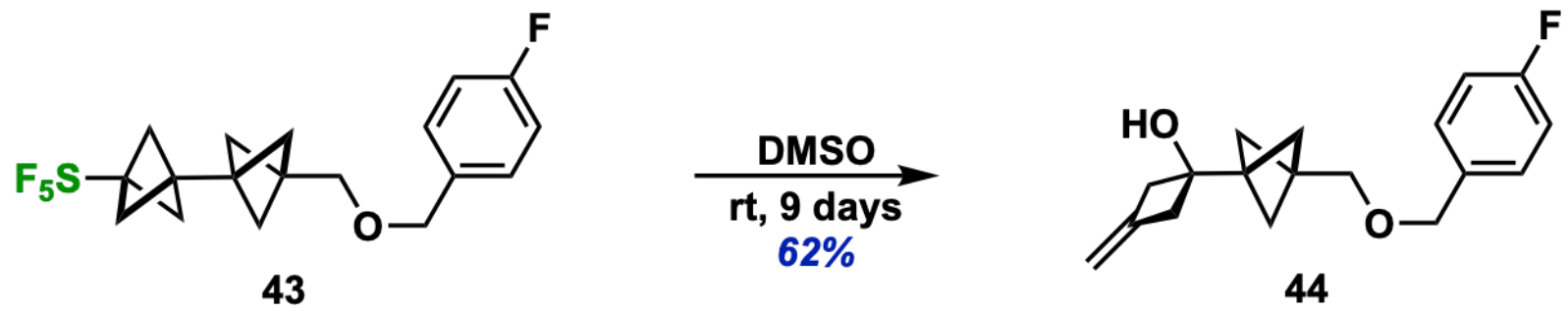

To an oven-dried microwave vial equipped with a stir bar was added compound **43** (42.0 mg, 0.105 mmol, 1.0 equiv.) and dissolved in DMSO (1.0 mL). The reaction was left to stir at room temperature for 9 days. Upon reaction completion, the reaction was diluted with H_2O (3 mL). The aqueous layer was extracted with EtOAc (3 x 3 mL). The combined organic layers were washed with brine (3 x 4 mL), dried over anhydrous Na_2SO_4, filtered through Celite, and concentrated. The crude residue was dissolved in a minimal amount of CH_2Cl_2 and loaded on to a silica plug eluting with 1:1 hexanes:EtOAc to afford a yellow oil (18.8 mg, 62%).

1-(3-(((4-fluorobenzyl)oxy)methyl)bicyclo[1.1.1]pentan-1-yl)-3-methylenecyclobutan-1-ol (**44**):
R_f: 0.29 (4:1 hexanes:EtOAc), vis: UV (254 nm).
^{1}H (300 MHz, $CDCl_3$): δ = 7.30-7.28 (m, 2H), 7.04-7.01 (m, 2H), 4.85-4.83 (m, 2H), 4.50 (s, 2H), 3.48 (s, 2H), 2.82-2.79 (m, 2H), 2.69-2.66 (m, 2H), 1.79 (s, 1H), 1.68 (s, 6H).
^{19}F (282 MHz, $CDCl_3$): δ = -115.1– -115.1 (m, 1H).
^{13}C{^{1}H} (151 MHz, $CDCl_3$): δ = 163.0-160.8 (d, $^1J_{C\text{-}F}$ = 245.3 Hz), 140.3, 134.4-134.4 (d, $^4J_{C\text{-}F}$ = 2.99 Hz), 129.3-129.3 (d, $^2J_{C\text{-}F}$= 8.15 Hz), 115.4-115.3 (d, $^3J_{C\text{-}F}$ = 21.42 Hz), 107.4, 72.4, 70.3, 69.8, 46.4, 44.8, 42.5, 37.1.
ATR-IR (ν_{max}): 3412, 2967, 2912, 2870, 1678, 1603, 1509, 1222, 877, 822 cm^{-1}.
HRMS (*m/z*): (ESI) calc'd for $C_{18}H_{22}FO_2^+$ [M-H]$^+$: calculated 289.1598, found 289.1599.

Solvent Stability Experiments

To an NMR tube was added **compound 42** (11.0 mg, 0.026 mmol, 1.0 equiv.). The NMR tube was sealed, evacuated, and backfilled with Ar three times. The material was dissolved in the designated deuterated NMR solvent (0.70 mL) and monitored over the time span of 120 h. See detailed NMR data below.

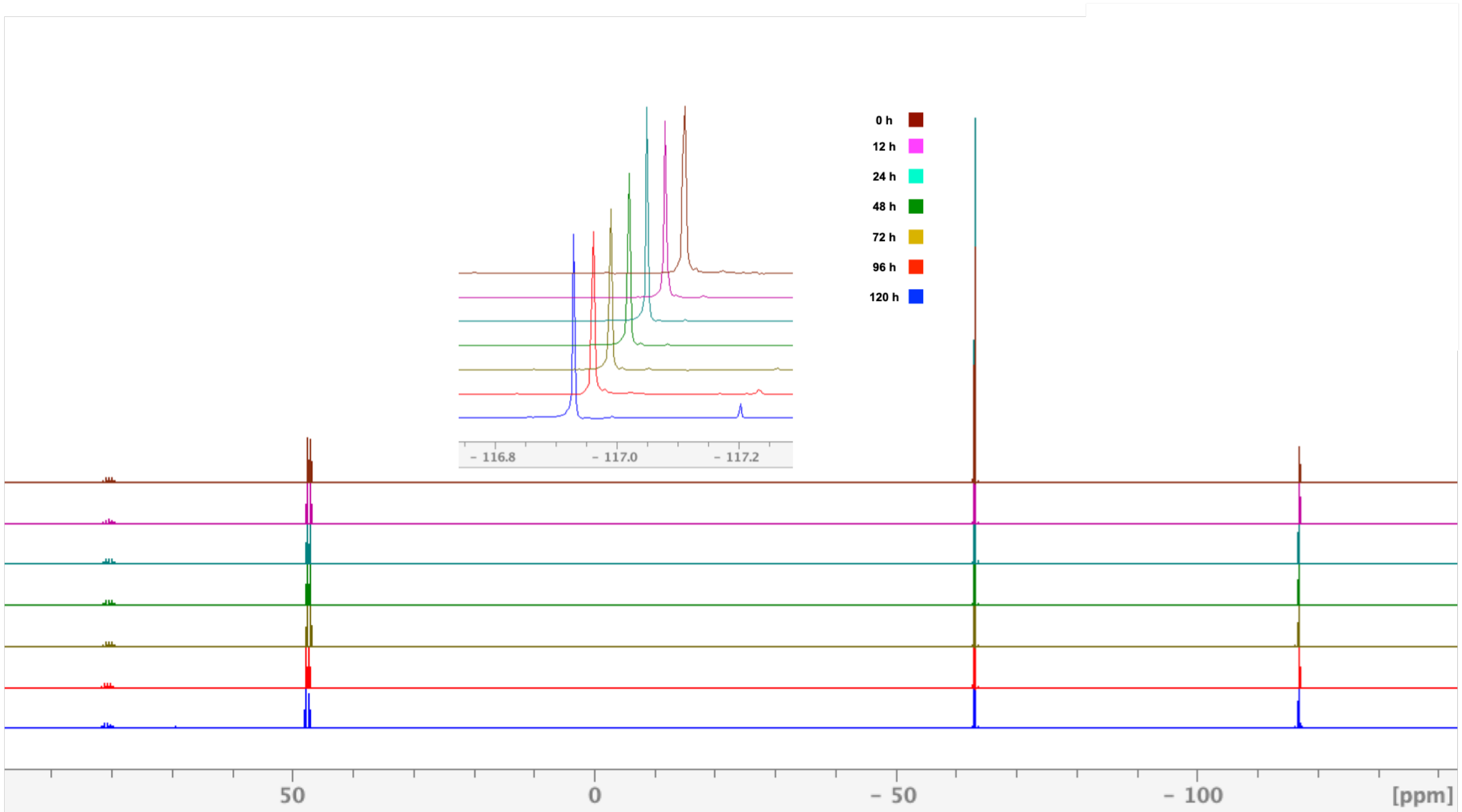

Figure S4. Compound **42** over the time span of 120 h in 100% DMSO-d_6.

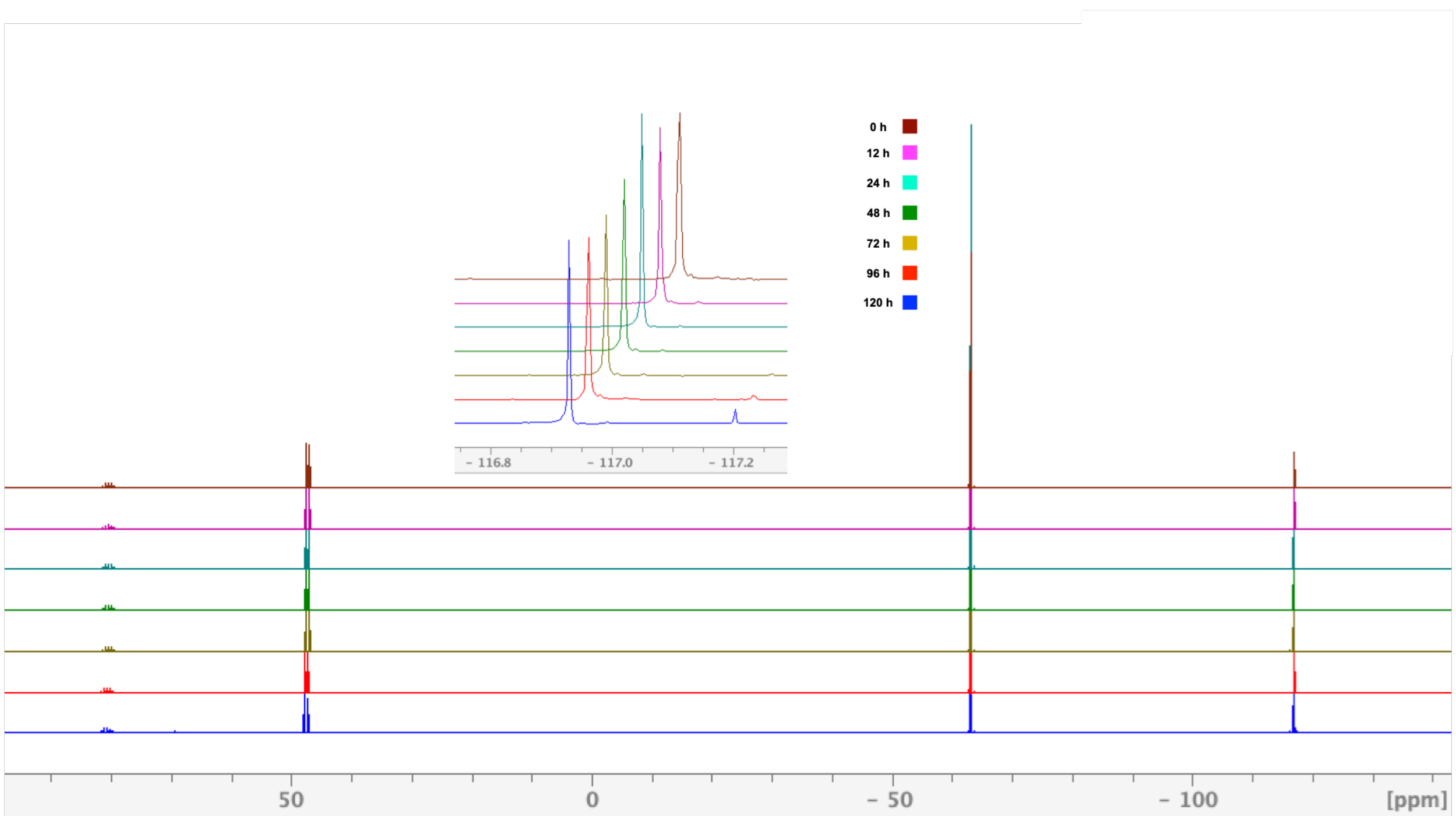

Figure S5. Compound **42** over the time span of 120 h in 5% D_2O:95% DMSO-d_6.

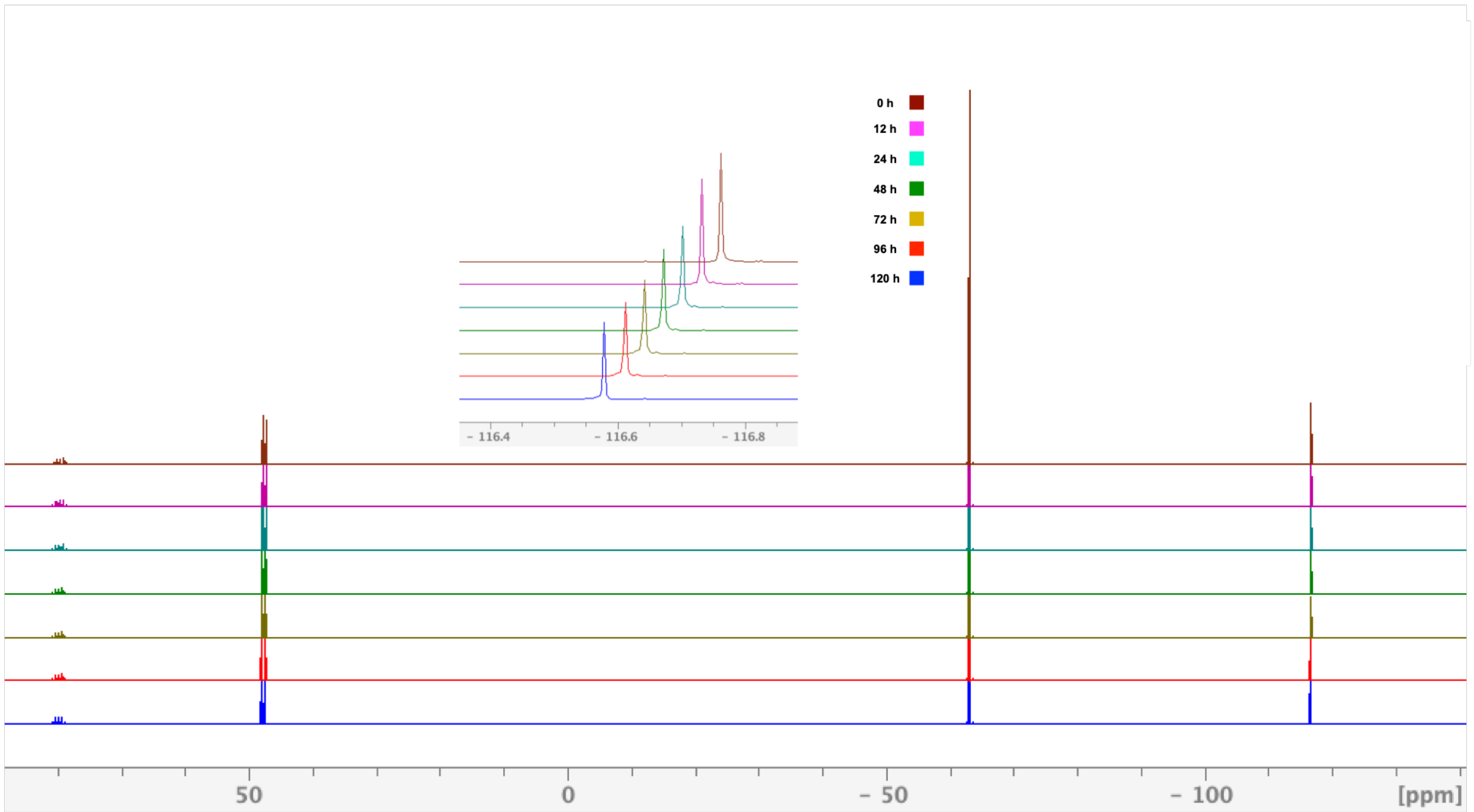

Figure S6. Compound **42** over the time span of 120 h in 100% MeCN-d_3.

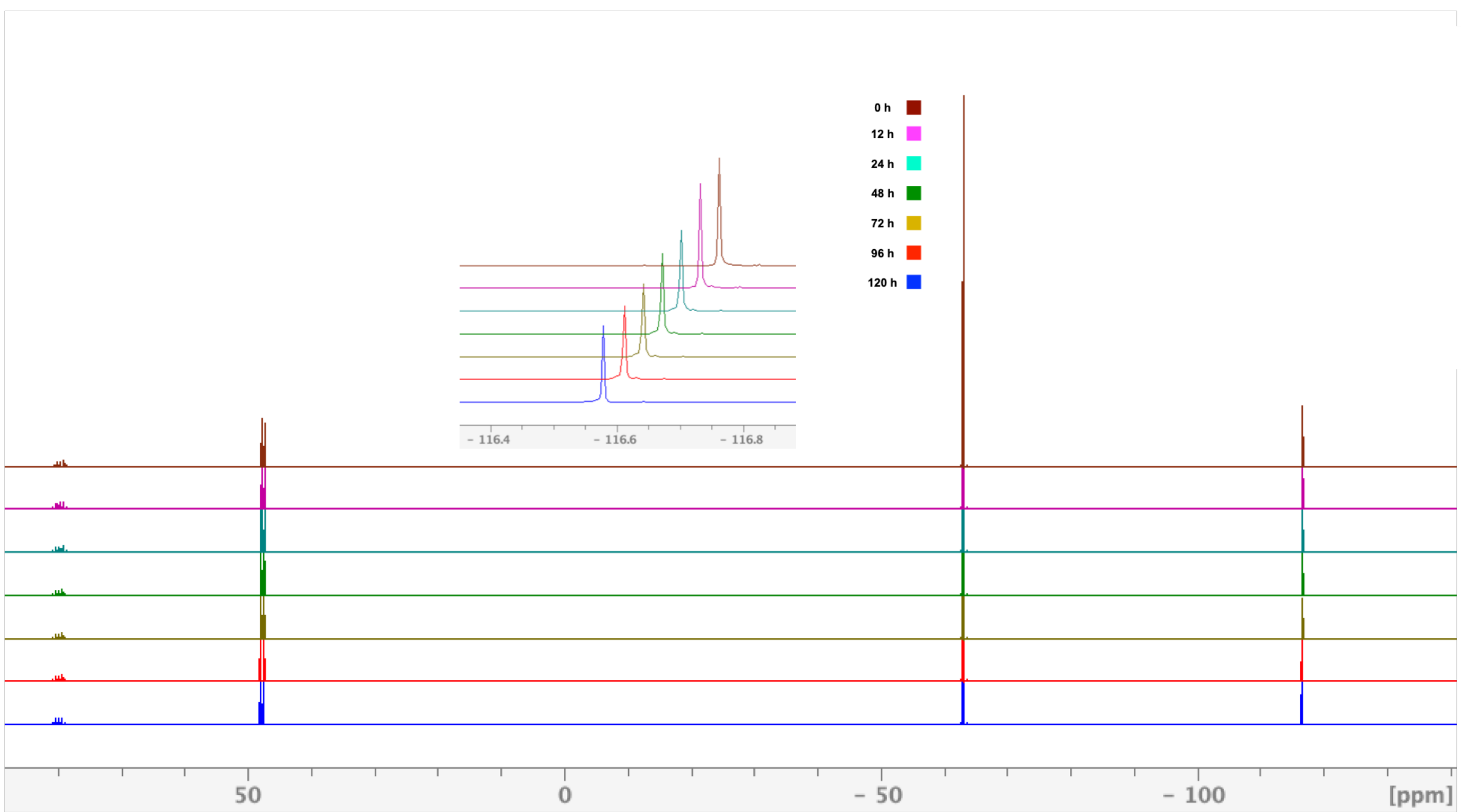

Figure S7. Compound **42** over the time span of 120 h in 5% D_2O:95% MeCN-d_3.

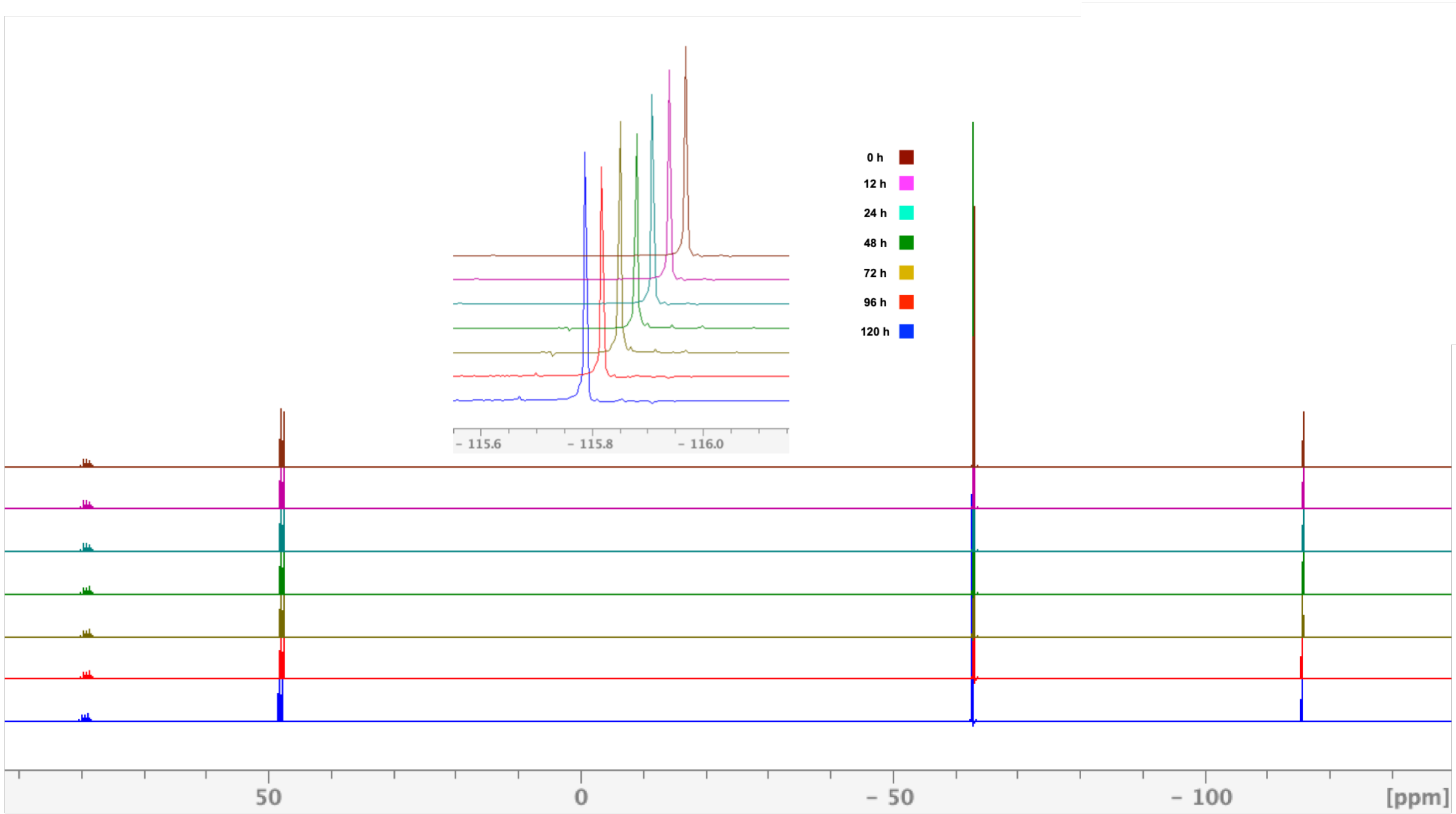

Figure S8. Compound **42** over the time span of 120 h in 100% MeOD-d_4.

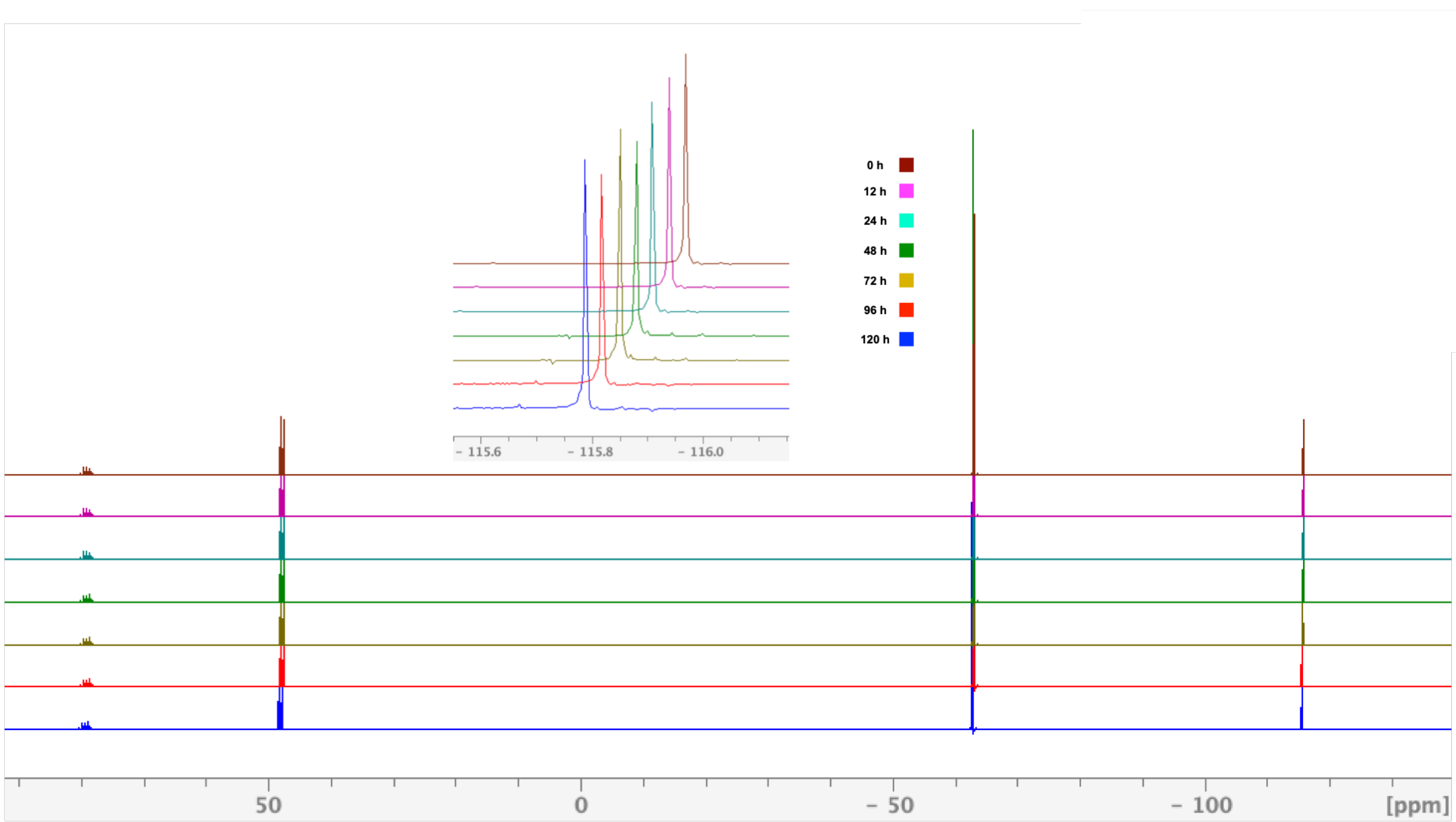

Figure S9. Compound **42** over the time span of 120 h in 5% D_2O:95% MeOD-d_4.

To an NMR tube was added **compound 43** (11.0 mg, 0.026 mmol, 1.0 equiv.). The NMR tube was sealed, evacuated, and backfilled with Ar three times. The material was dissolved in the designated deuterated NMR solvent (0.70 mL) and monitored over the time span of 120 h. See detailed NMR data below.

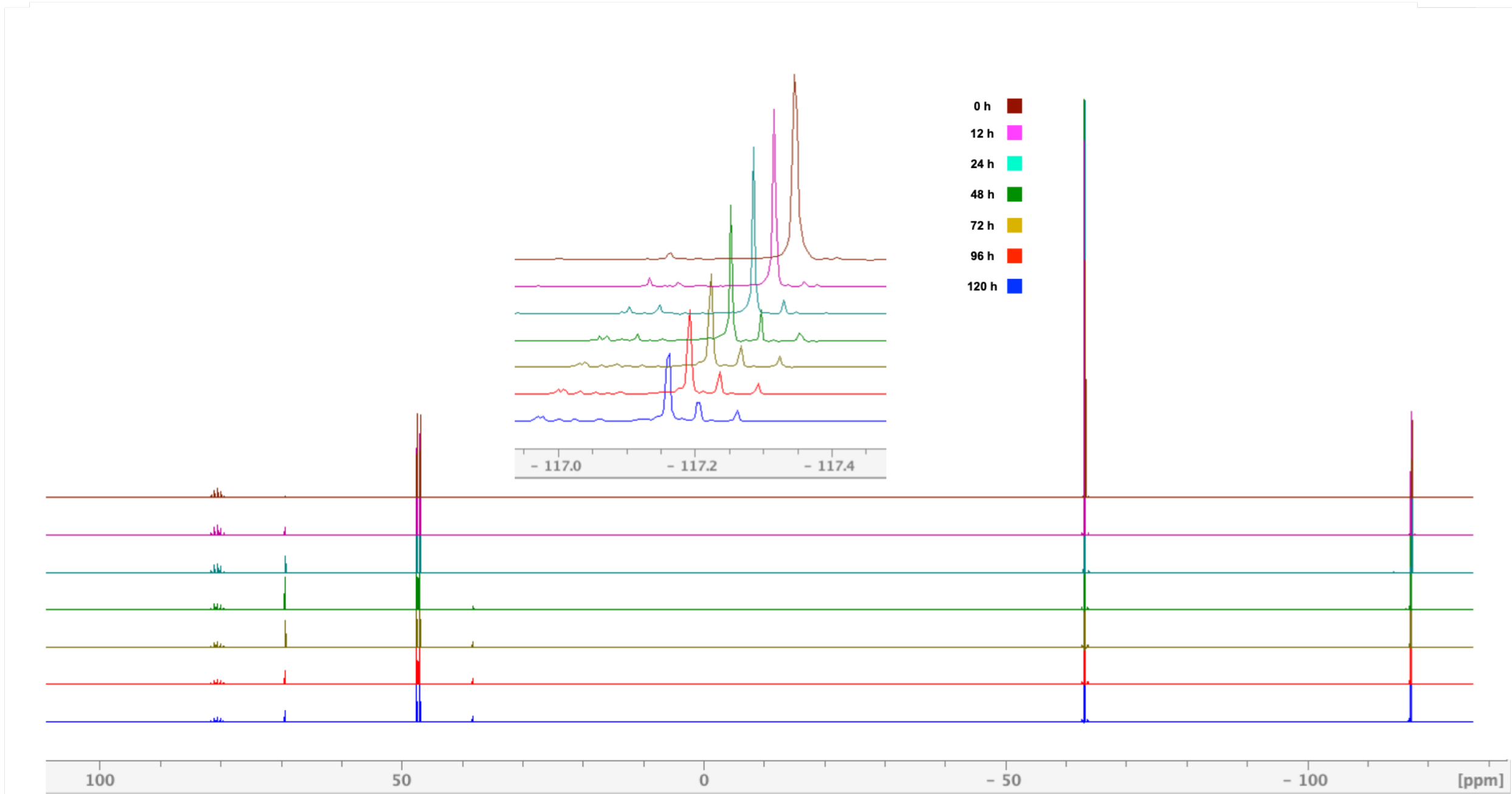

Figure S10. Compound **43** over the time span of 120 h in 100% DMSO-d_6. *Note that compound **43** (-117.16 ppm) decreases in concentration over time concomitant with a rise in concentration of compound **44** (-117.20 ppm).*

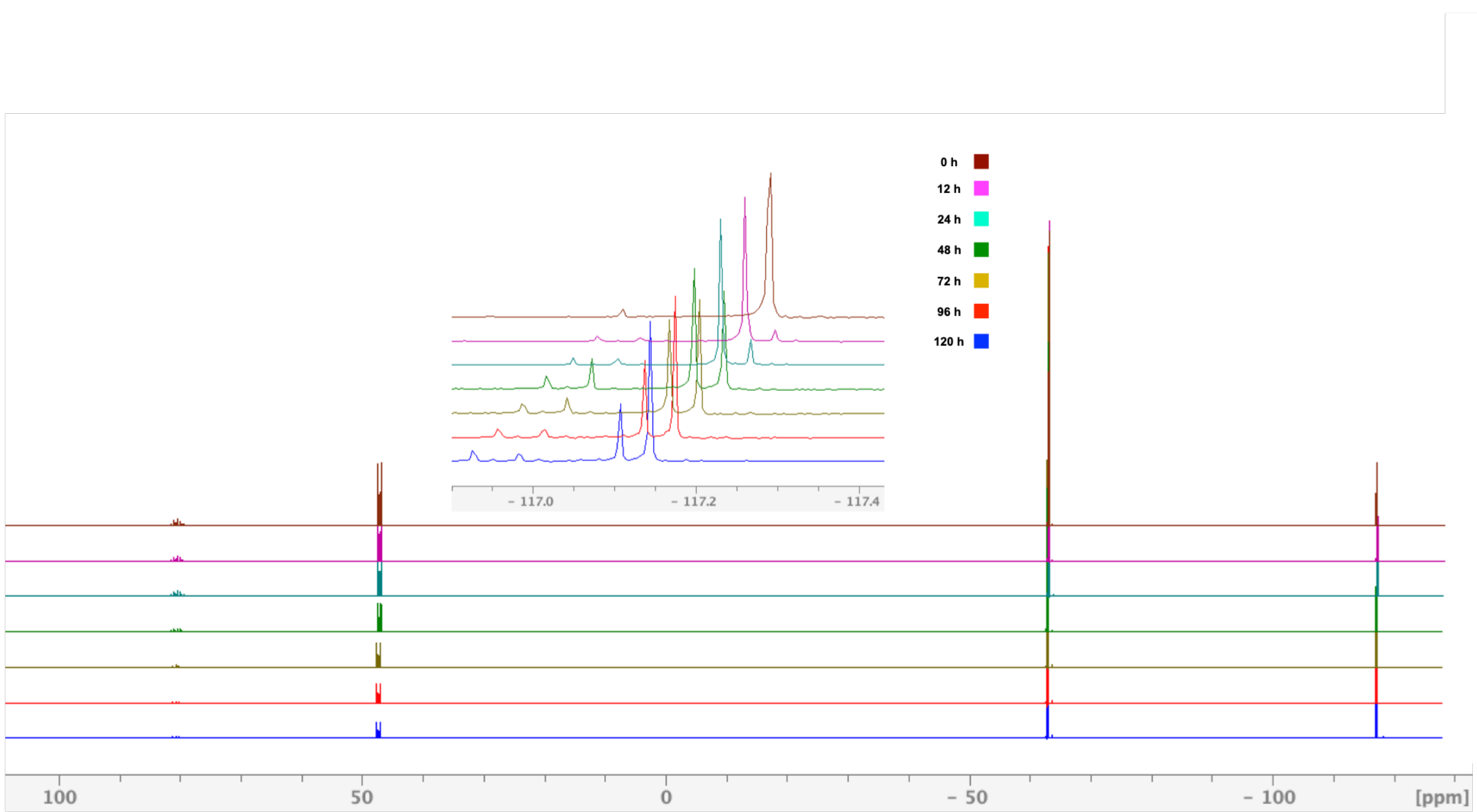

Figure S11. Compound **43** over the time span of 120 h in 5% D_2O:95% DMSO-d_6. *Note that compound **43** (-117.10 ppm) decreases in concentration over time concomitant with a rise in concentration of compound **44** (-117.14 ppm).*

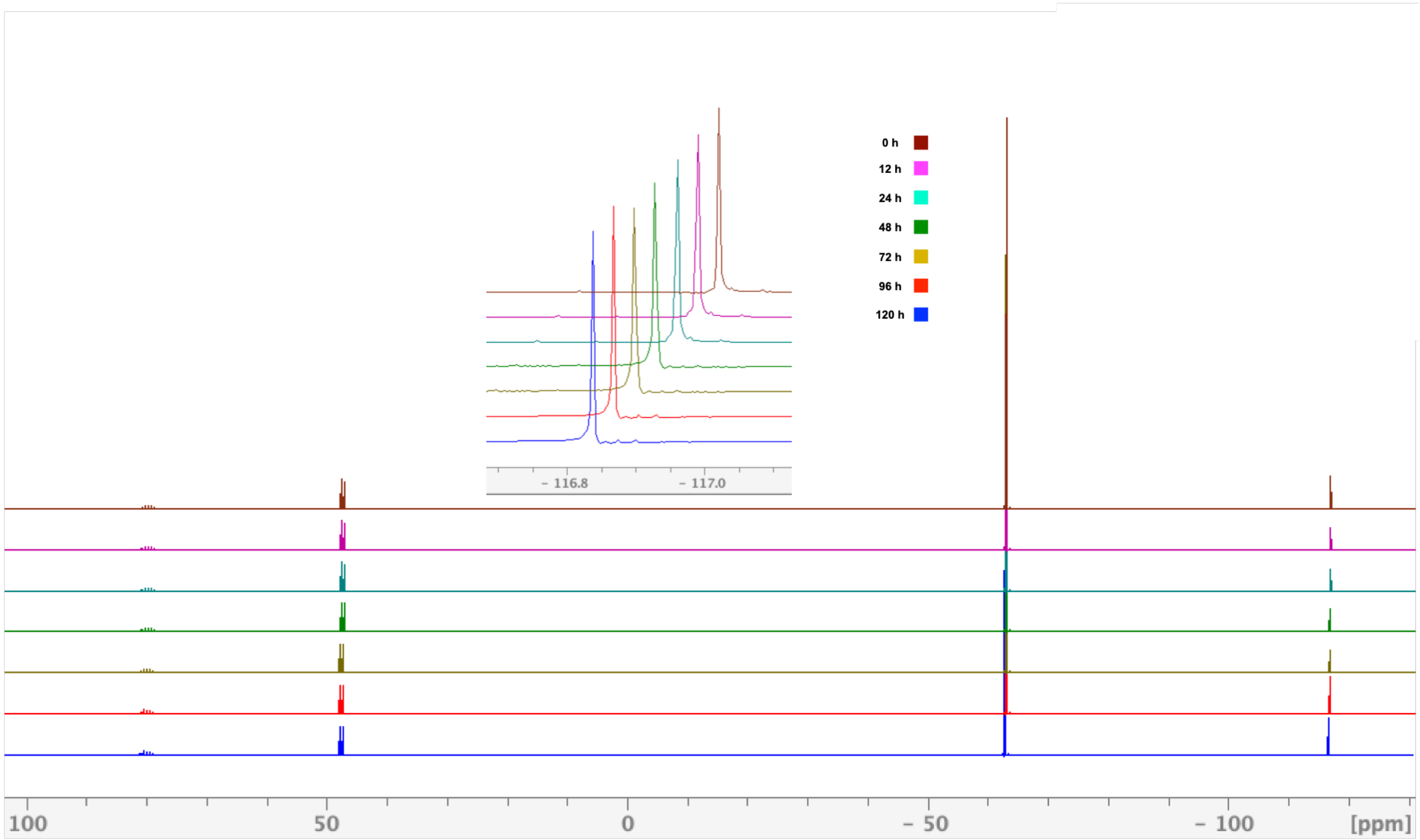

Figure S12. Compound **43** over the time span of 120 h in 100% MeCN-d_3.

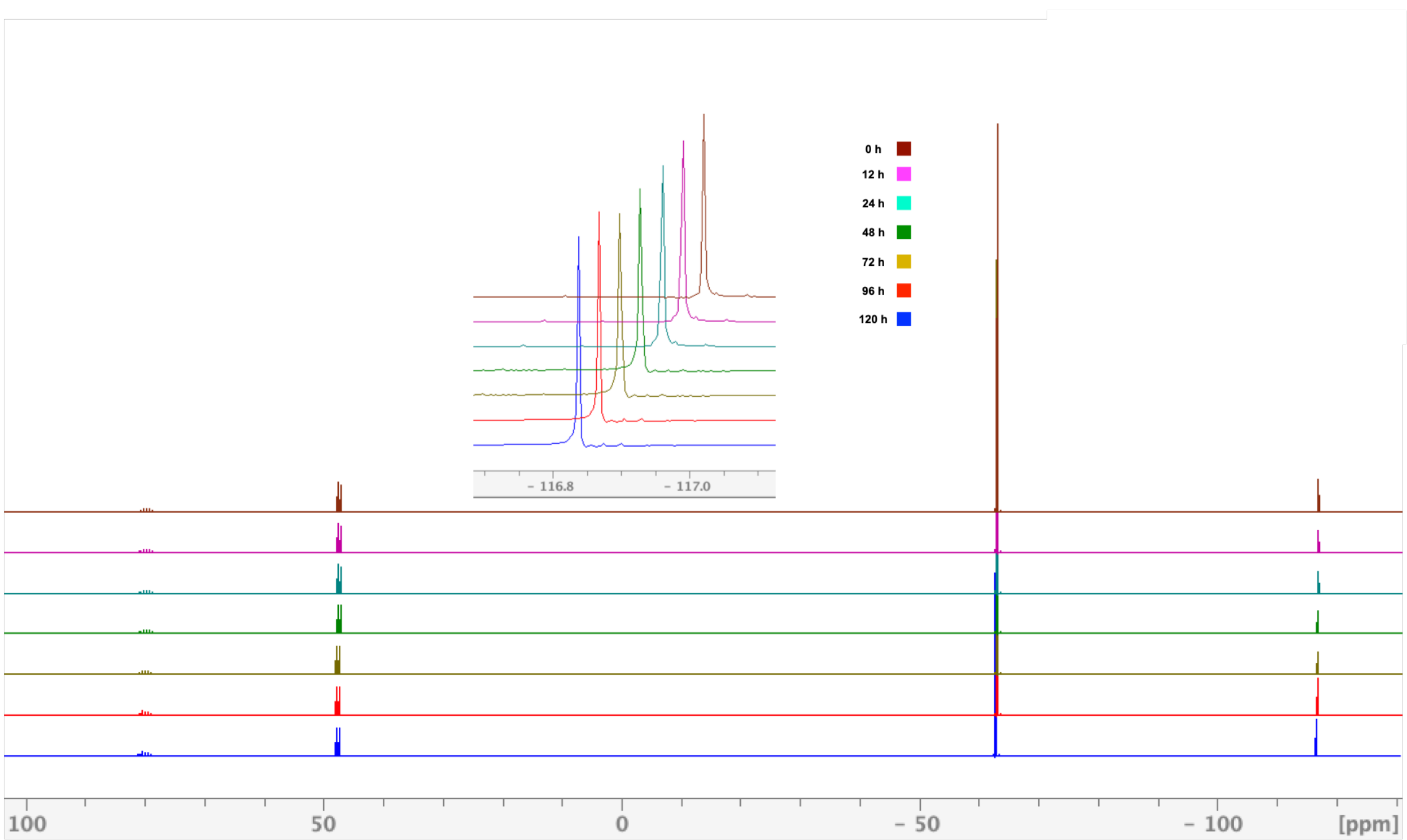

Figure S13. Compound **43** over the time span of 120 h in 5% D_2O:95% MeCN-d_3.

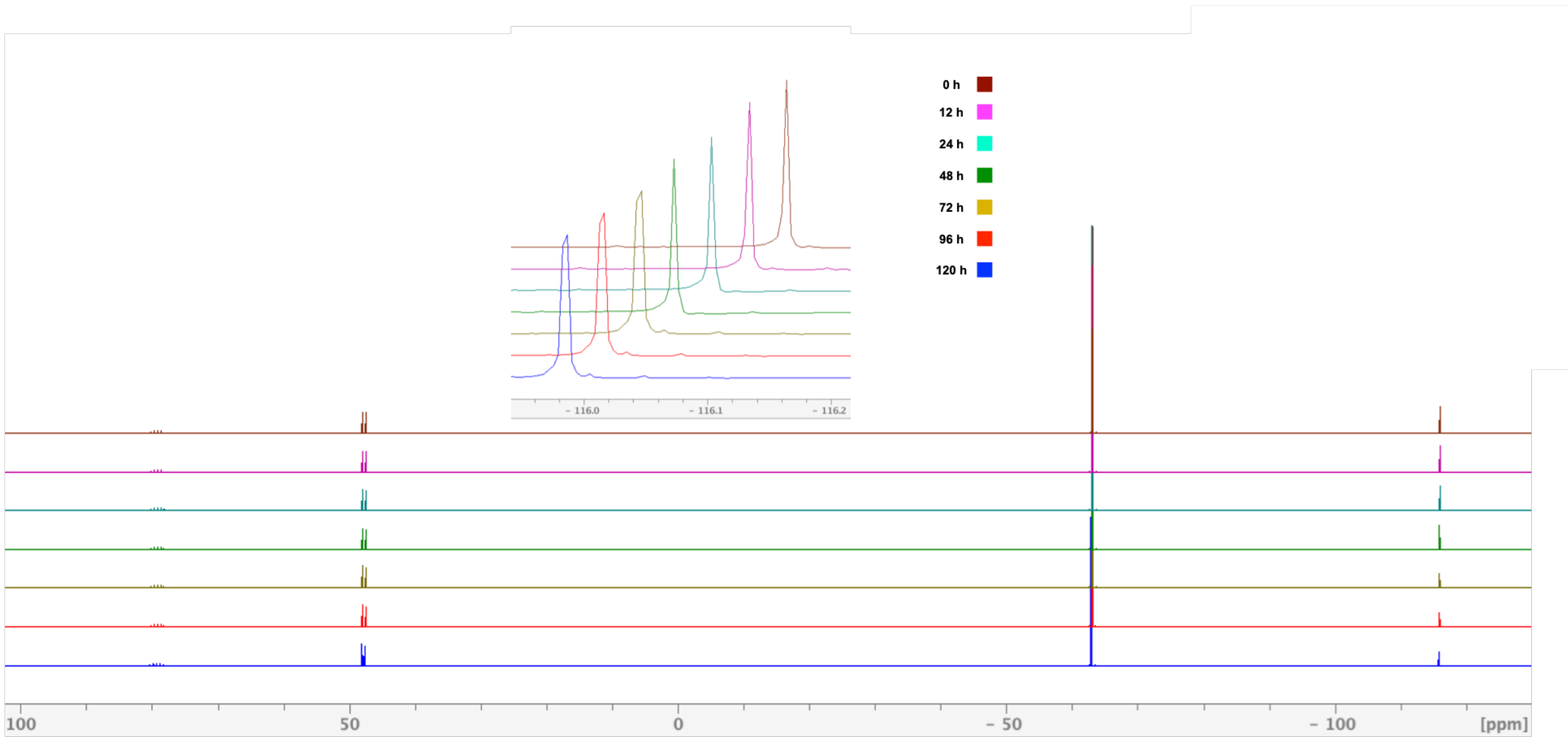

Figure S14. Compound **43** over the time span of 120 h 100% MeOD-d_4.

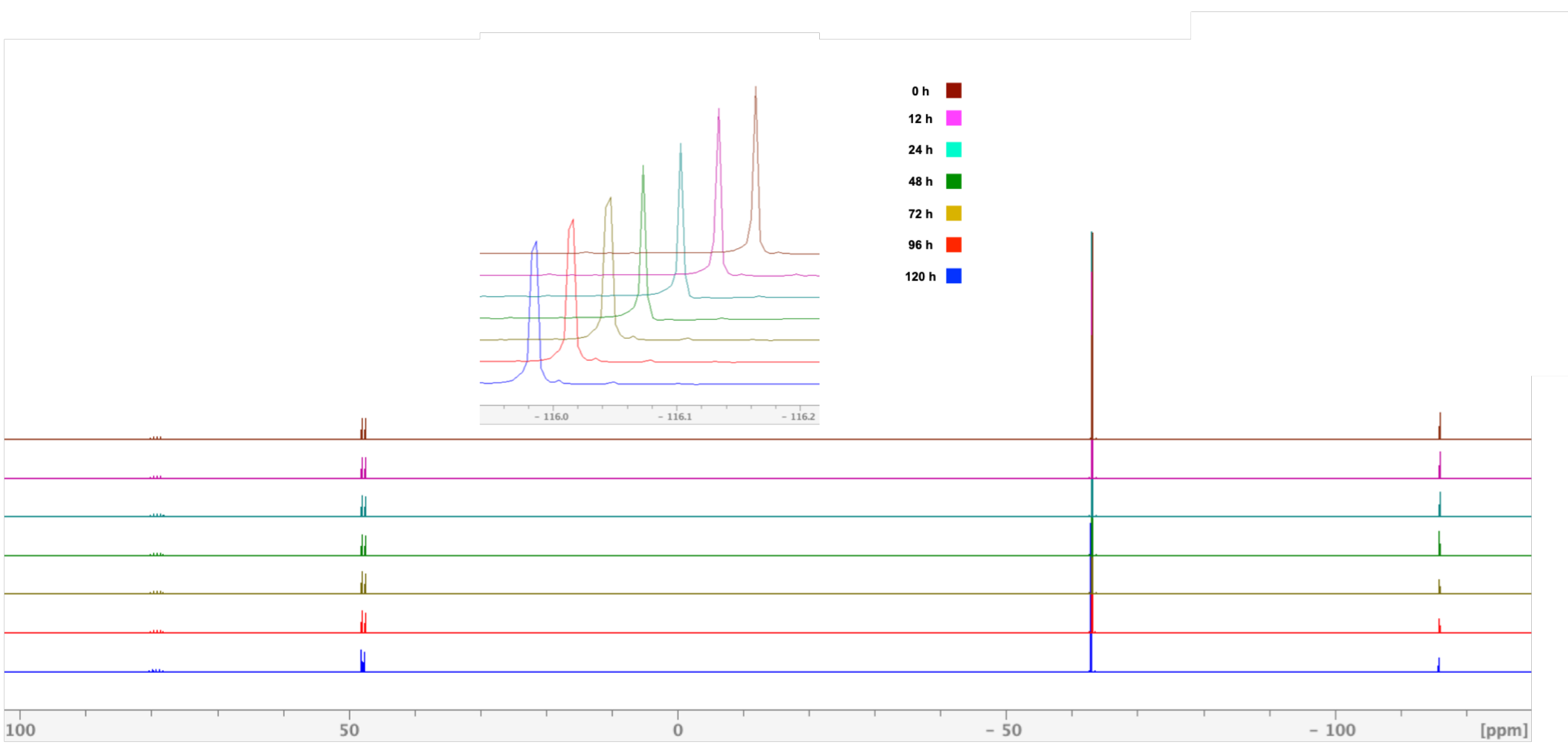

Figure S15. Compound **43** over the time span of 120 h in 5% D_2O:95% MeOD-d_4.

Thermal Stability Experiments

An oven-dried NMR tube was sealed, evacuated, and backfilled with Ar three times. A 0.038 mM stock solution of compound **42** in toluene-d_8 (0.40 mL, 5.0 mg, 0.015 mmol) and a 0.076 mM stock solution of trifluorotoluene in toluene-d_8 (0.1 mL, 11.1 mg, 0.0076 mmol) was added *via* syringe. Two additional NMR tubes were prepared. All three NMR tubes were individually monitored over the time span of 24 h, 48 h, and 72 h at 80 °C, 100 °C, and 120°C.

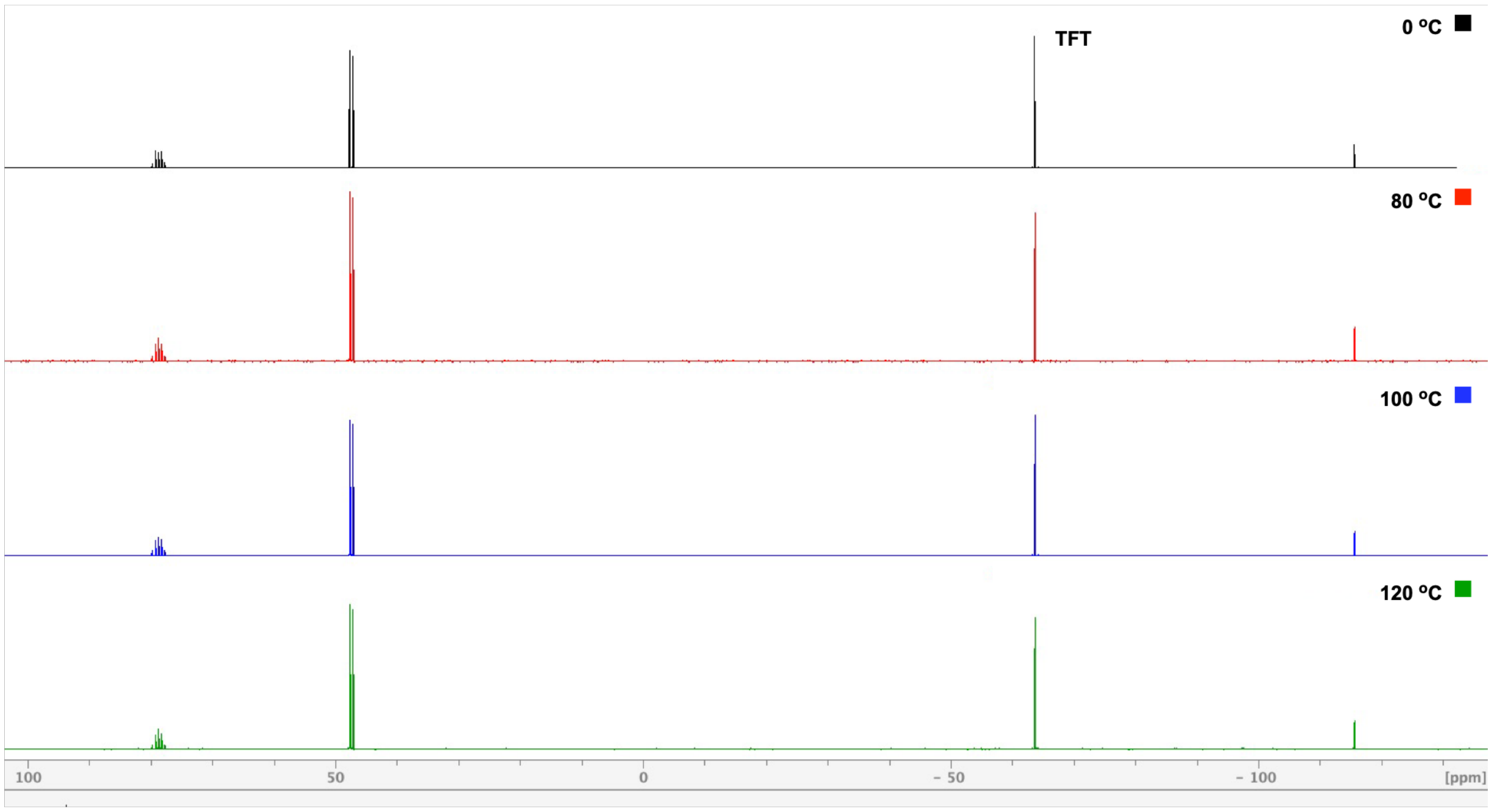

Figure S16. Compound **42** and TFT in toluene-d_8 at 80 °C, 100 °C, and 120 °C for 24 h.

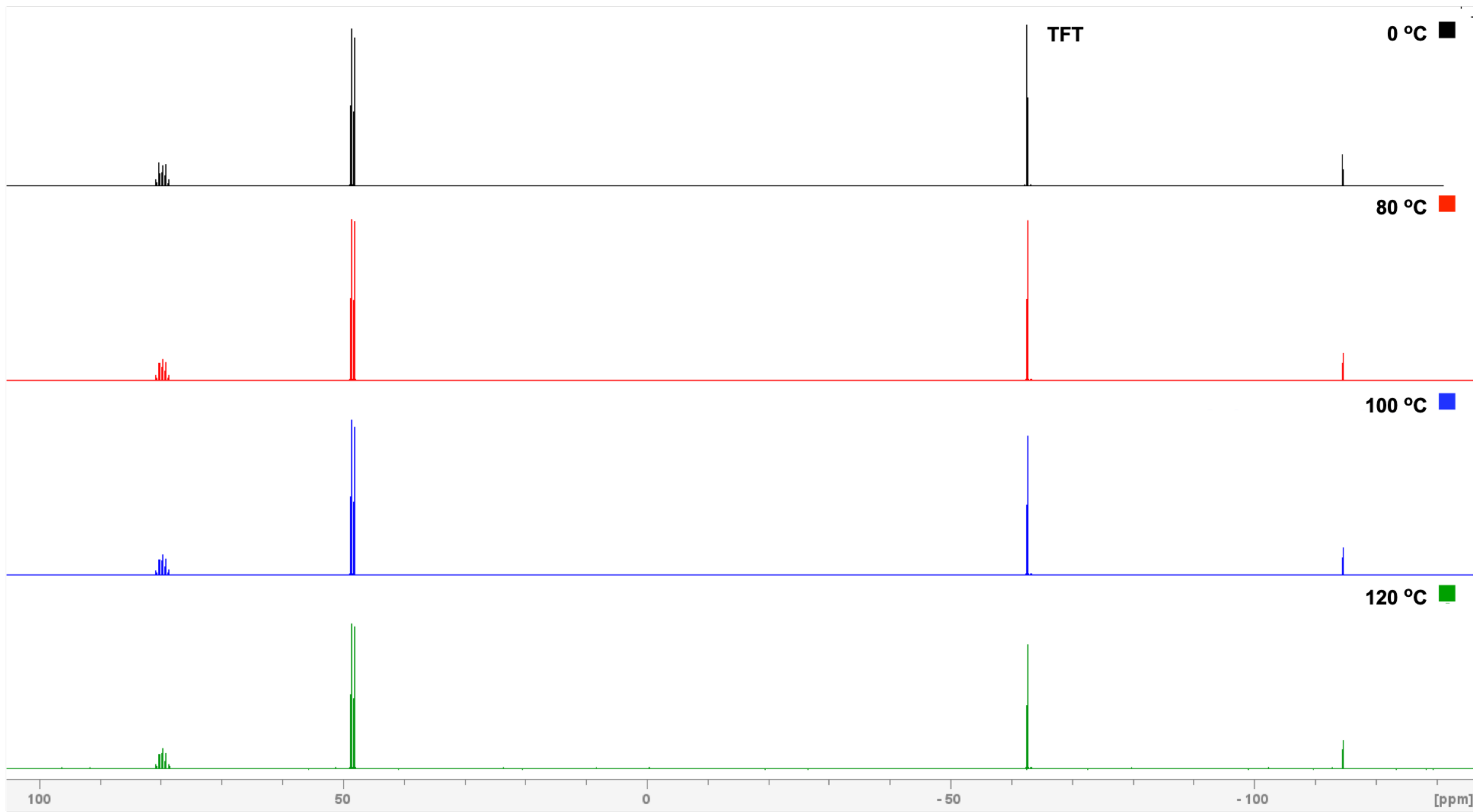

Figure S17. Compound **42** and TFT in toluene-d_8 at 80 °C, 100 °C, and 120 °C for 48 h.

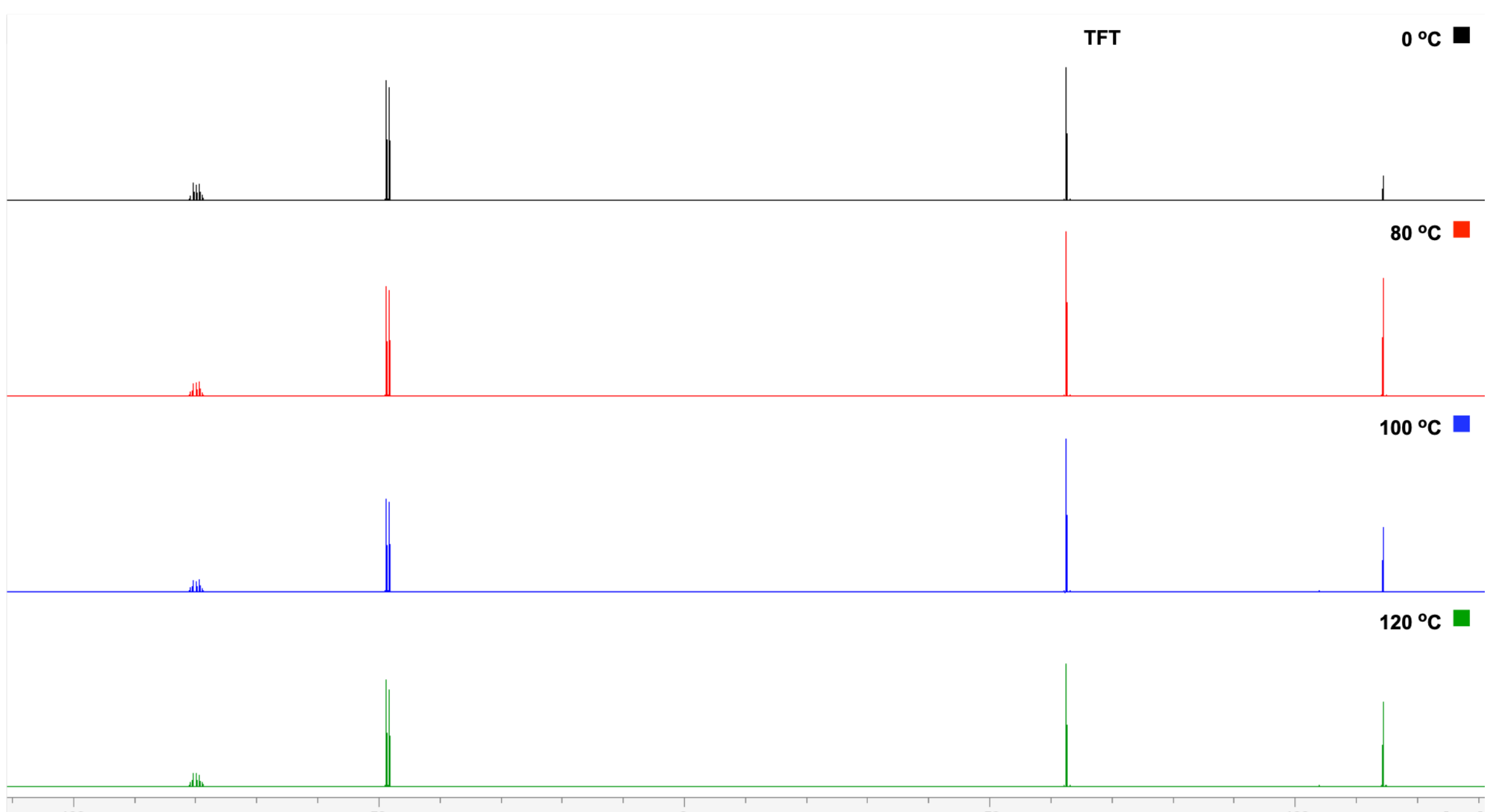

Figure S18. Compound **42** and TFT in toluene-d_8 at 80 °C, 100 °C, and 120 °C for 72 h.

An oven-dried NMR tube was sealed, evacuated, and backfilled with Ar three times. A 0.031 mM stock solution of compound **43** in toluene-d_8 (0.40 mL, 5.0 mg, 0.013 mmol) and a 0.084 mM stock solution of trifluorotoluene in toluene-d_8 (0.10 mL, 12.3 mg, 0.0084 mmol) was added *via* syringe. Two additional NMR tubes were prepared. All three NMR tubes were individually monitored over the time span of 24 h, 48 h, and 72 h at 80 °C, 100 °C, and 120°C.

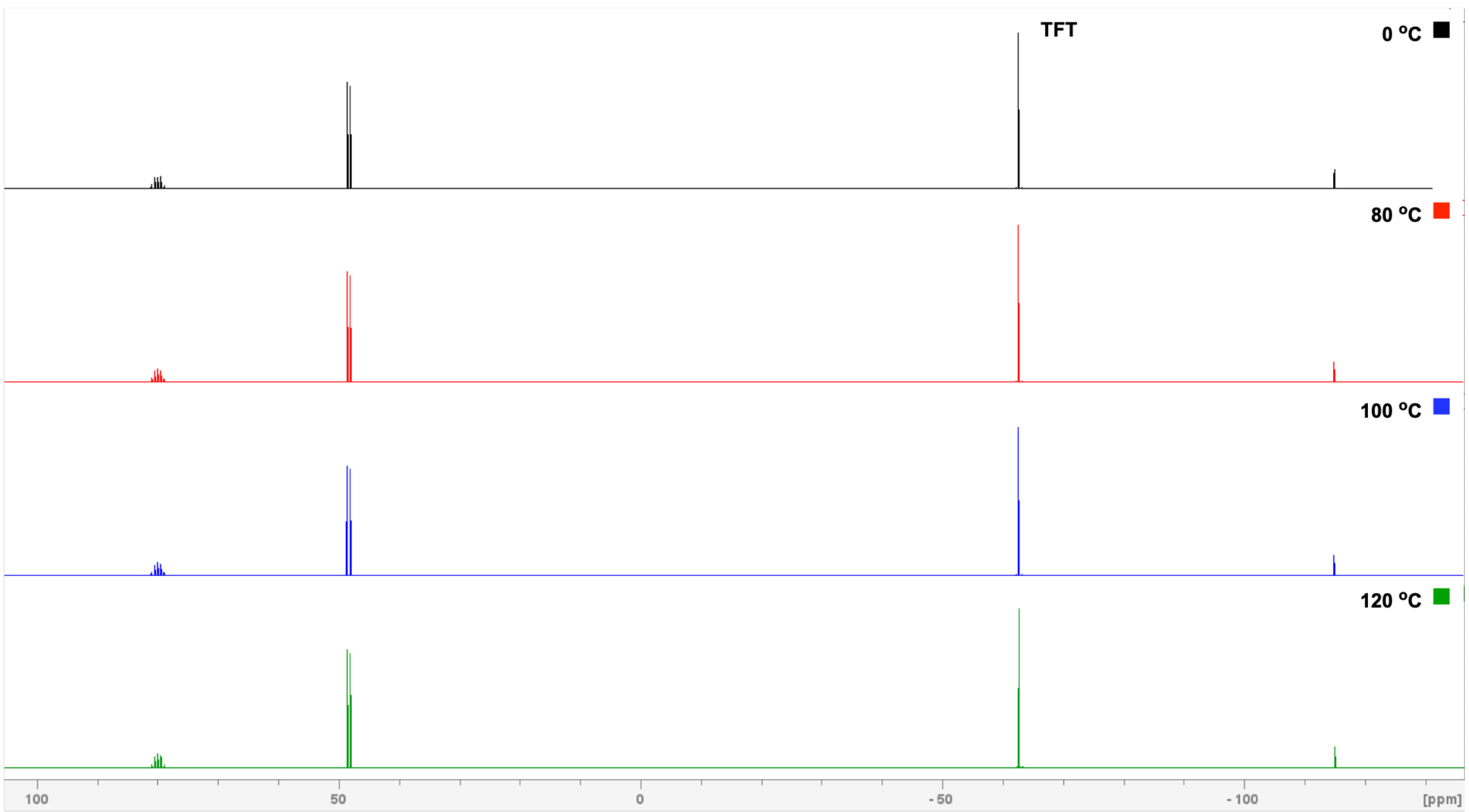

Figure S19. Compound **43** and TFT in toluene-d_8 at 80 °C, 100 °C, and 120 °C for 24 h.

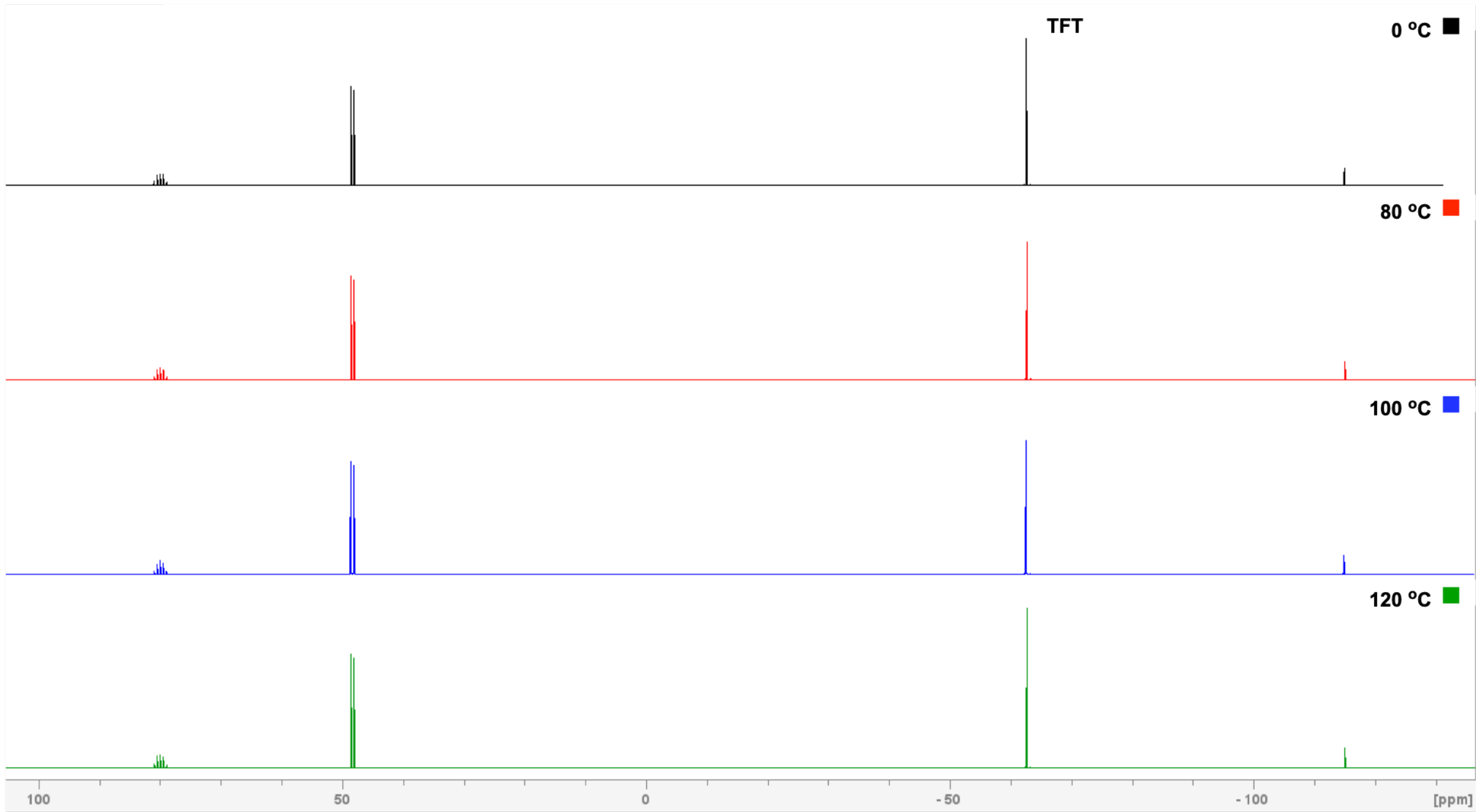

Figure S20. Compound **43** and TFT in toluene-d_8 at 80 °C, 100 °C, and 120 °C for 48 h.

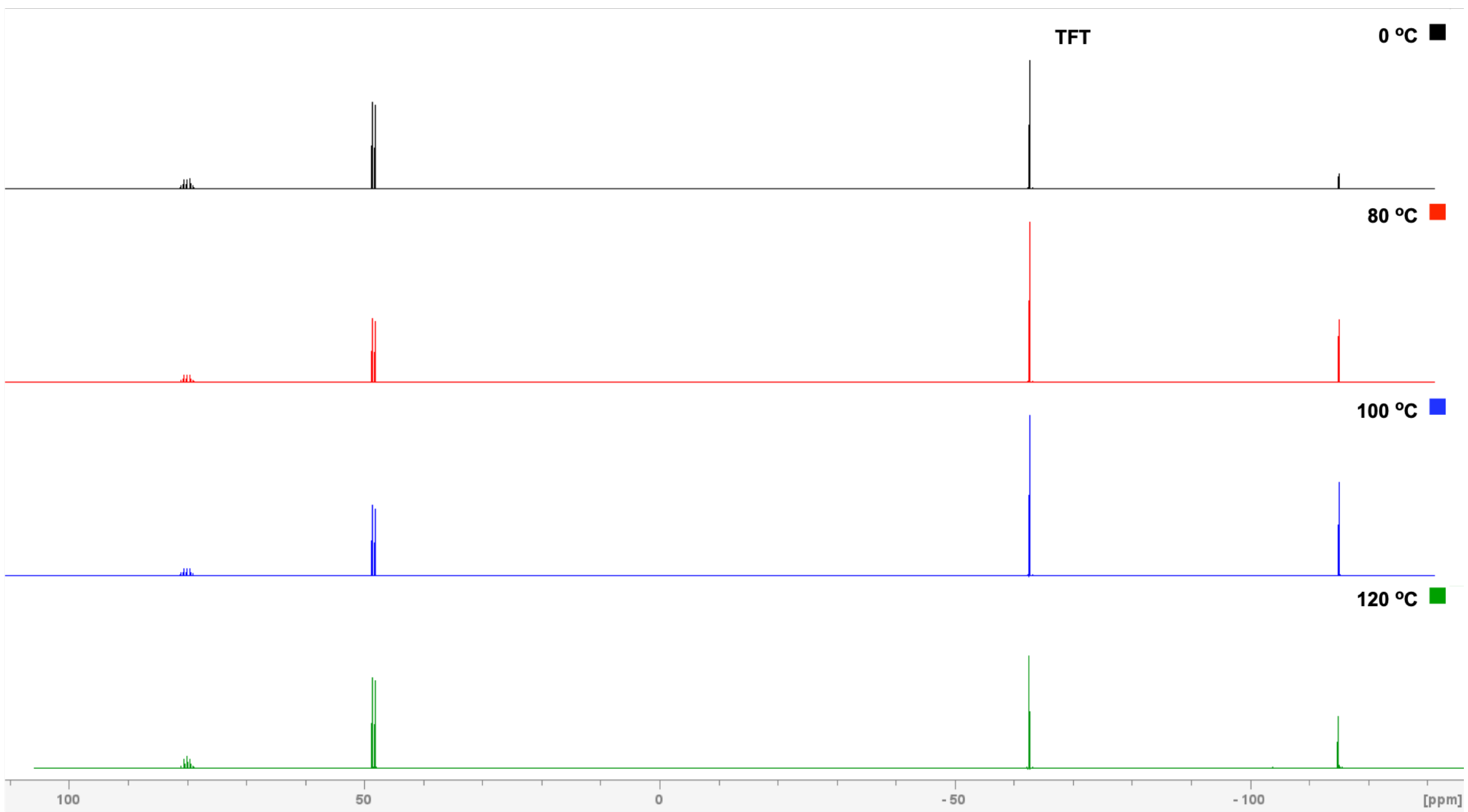

Figure S21. Compound **43** and TFT in toluene-d_8 at 80 °C, 100 °C, and 120 °C for 72 h.

Aqueous Stability Experiments

citrate buffer solution procedure

To a beaker equipped with a stir bar was added citric acid (2.10 g) and distilled H_2O (100 mL). The solution was stirred until all solids were dissolved. To a separate beaker equipped with a stir bar, sodium citrate (2.94 g) and distilled H_2O (100 mL) were added. The solution was stirred until all solids were dissolved. In a plastic bottle, citric acid solution (46.5 mL), sodium citrate solution (3.5 mL), and distilled water (100 mL) were added. The solution was mixed thoroughly, and a pH meter was used to confirm a resulting pH of 2.5.

phosphate buffered Saline (PBS) procedure

To a beaker equipped with a stir bar was added distilled H_2O (400 mL), sodium chloride (4.00 g), potassium chloride (100 mg), sodium phosphate dibasic (720 mg), and potassium phosphate monobasic (123 mg). The solution was stirred until all solids were dissolved, and a pH meter was used to confirm a resulting pH of 7.4.

carbonate buffer solution procedure

To a beaker equipped with a stir bar was added anhydrous sodium carbonate (2.12 g) and distilled H_2O (100 mL). The solution was stirred until all solids were dissolved. To a separate beaker equipped with a stir bar, sodium bicarbonate (1.68 g) and distilled H_2O (100 mL) were added. The solution was stirred until all solids were dissolved. To a plastic bottle, sodium carbonate solution (27.5 mL), sodium bicarbonate solution (22.5 mL), and distilled H_2O (50 mL) were added. The solution was mixed thoroughly, and a pH meter was used to confirm a resulting pH of 10.2.

rodent plasma solution procedure

Rodent plasma was purchased from Millipore Sigma (P2516-ML) and hydrolyzed in Milli-Q water (1.0 mL).

aqueous stability experiment procedure and NMR spectra

To test the stability of compound **42** in different aqueous environments, a 0.121 mM stock solution of compound **42** (36.2 mg, 0.109 mmol) was prepared in MeCN (0.9 mL). Four oven-dried microwave vials equipped with a stir bar were sealed, evacuated, and backfilled with Ar three times. The appropriate buffer solution (0.5 mL) was added *via* syringe. While stirring, the stock solution of compound **42** was added (0.1 mL, 4.0 mg, 0.012 mmol) was added dropwise *via* syringe. To test the stability of compound **42** in a biologically relevant environment, the stock solution of compound **42** (0.1 mL) was added dropwise *via* syringe to a stirring solution of rodent plasma (0.25 mL) and Milli-Q water (0.25 mL). (*Note: that compound **42** is only marginally soluble in aqueous media*). The resulting suspension was stirred at 37 °C in an oil bath for 24 h. After 24 h, the aqueous layer was extracted with EtOAc (3 x 2 mL). The combined organic layers were dried over anhydrous Na_2SO_4 and filtered through a glass pipette containing cotton and Celite. The resulting organic layer was concentrated and analyzed by ^{19}F NMR.

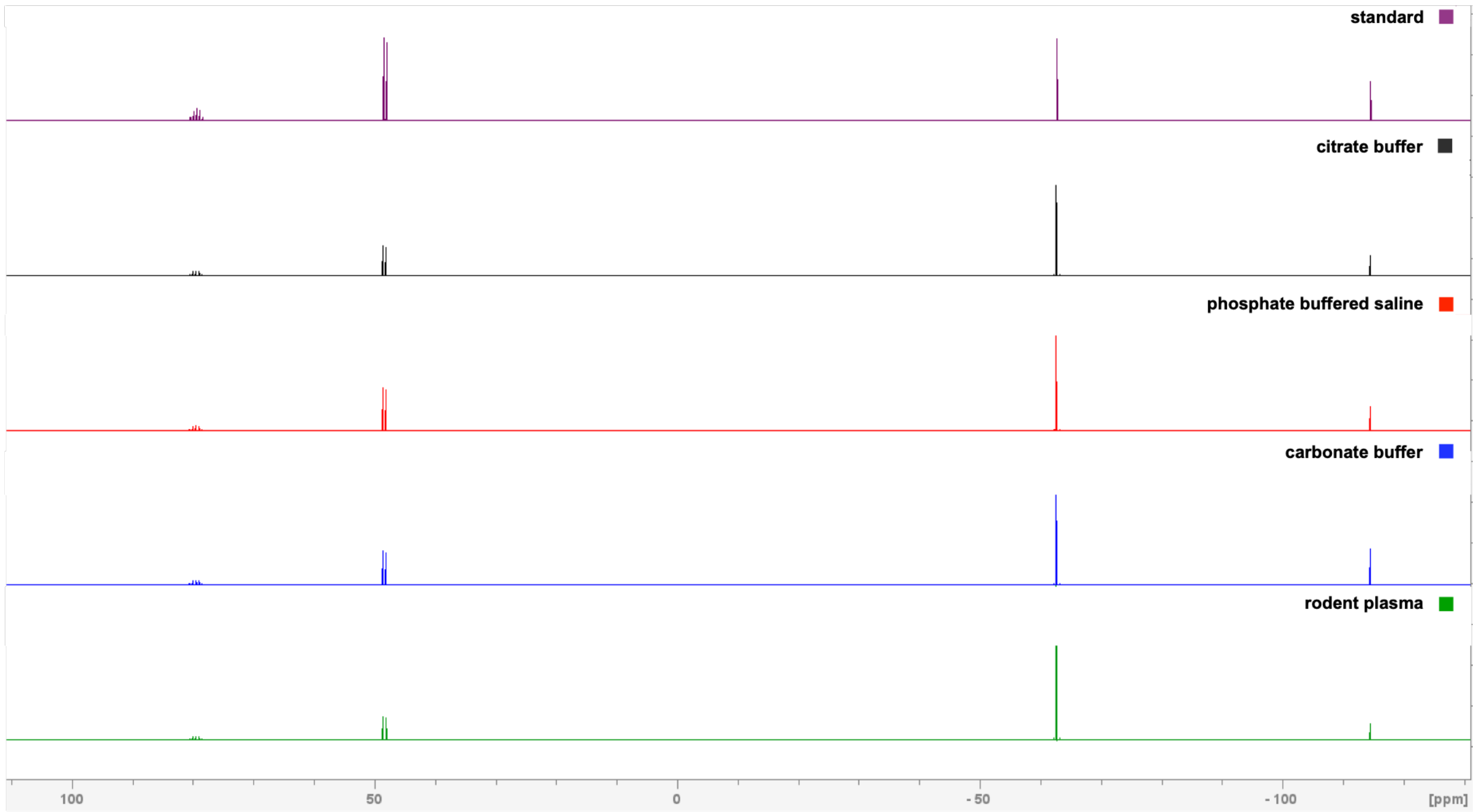

Figure S22. Compound **42** and TFT in $CDCl_3$.

To test the stability of compound **43** in different aqueous environments, a 0.100 mM stock solution of compound **43** (38.4 mg, 0.096 mmol) was prepared in MeCN (0.96 mL). Four oven-dried microwave vials equipped with a stir bar were sealed, evacuated, and backfilled with Ar three times. The appropriate buffer solution (0.5 mL) was added *via* syringe. While stirring, the stock solution of compound **43** was added (0.10 mL, 4.0 mg, 0.0100 mmol) dropwise *via* syringe. To test the stability of compound **43** in a biologically relevant environment, the stock solution of compound **43** (0.1 mL) was added dropwise *via* syringe to a stirring solution of rodent plasma (0.25 mL) and Milli-Q water (0.25 mL). (*Note: that compound **43** is only marginally soluble in aqueous medium*). The resulting suspension was stirred at 37 °C in an oil bath for 24 h. After 24 h, the aqueous layer was extracted with EtOAc (3 x 2 mL). The combined organic layers were dried over anhydrous Na_2SO_4 and filtered through a glass pipette containing cotton and Celite. The resulting organic layer was concentrated and analyzed by ^{19}F NMR using TFT as an internal standard.

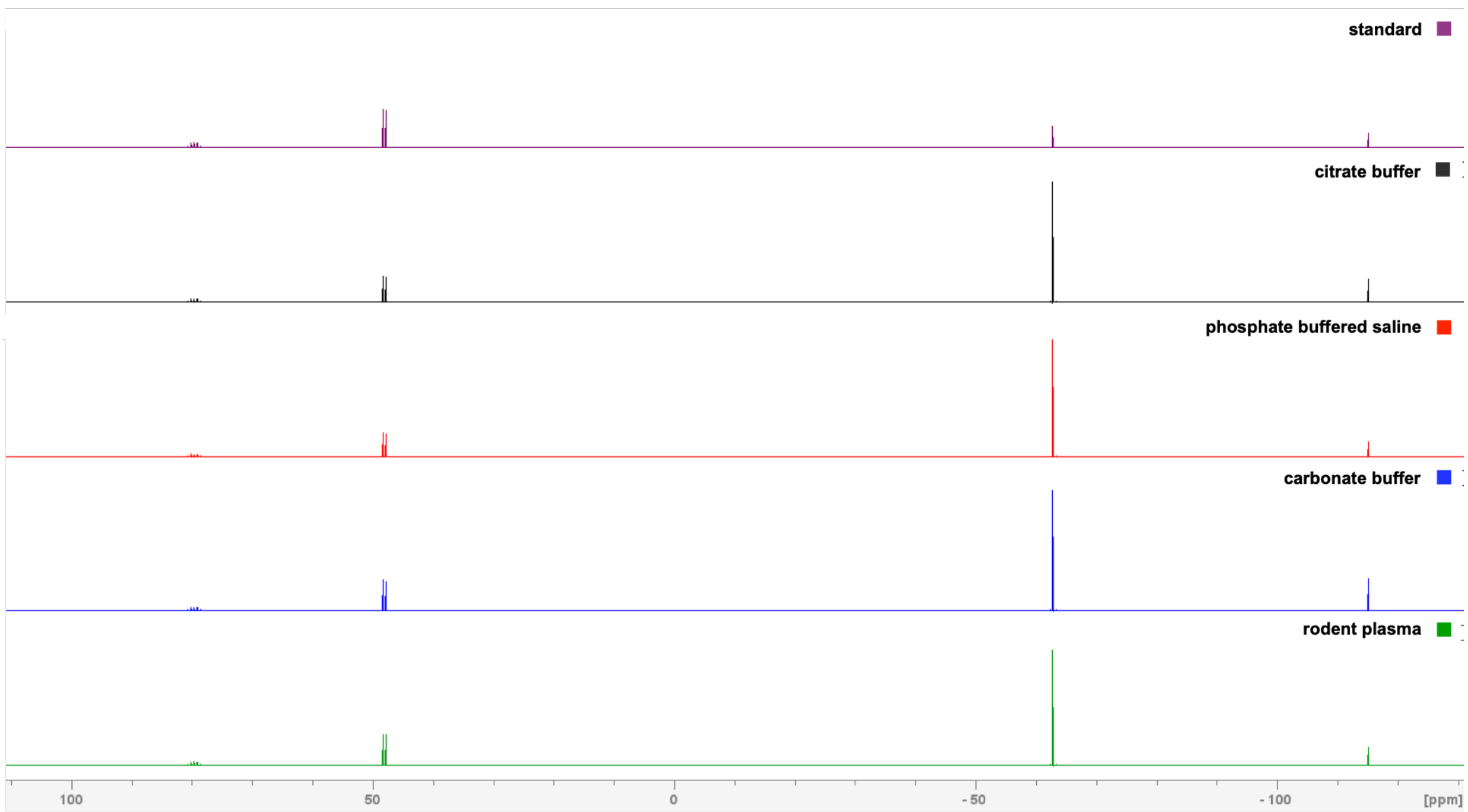

Figure S23. Compound **43** and TFT in $CDCl_3$.

Compounds 38-41 Toward Programmed Decomposition of SF_5-[*2*]staffanes

Compound 45

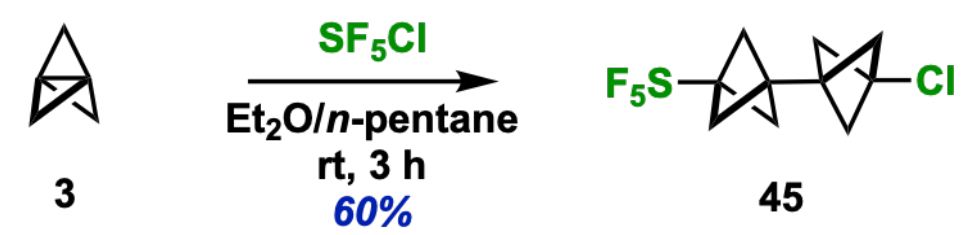

The synthesis of compound **45** followed a known literature procedure.[13] An oven-dried round bottom equipped with a stir bar was cooled to room temperature under vacuum and purged with Ar three times. An ~0.8 M solution of [1.1.1]propellane in Et_2O (24.0 mmol, 6.0 equiv.) was added *via* syringe in one portion. The reaction mixture was stirred. Subsequently, a ~0.1 M solution SF_5Cl in *n*-pentane (4.00 mmol, 1.0 equiv.) was added in three portions over a time span of 30 min. (*Note: The Schlenk line or Ar balloon was removed after each SF₅Cl portion addition.)* The resulting mixture was stirred at room temperature for 3 h. Upon completion, the reaction was concentrated. The crude residue was purified via column chromatography eluting with 100% hexanes to afford a white solid (0.707 g, 60%).

(3'-chloro-[1,1'-bi(bicyclo[1.1.1]pentan)]-3-yl)pentafluoro-λ^6-sulfane (**45**):
R_f: 0.42 (hexanes), vis: *p*-anisaldehyde.
m.p.: 122.4–124.2 ºC.
^{1}H (300 MHz, $CDCl_3$): δ = 2.19 (s, 6H), 2.05 (s, 6H).
^{19}F (282 MHz, $CDCl_3$): δ = 80.19-78.13 (p, 1F, $^2J_{F\text{-}F}$ = 143.6 Hz), 48.75-48.23 (d, 4F, $^2J_{F\text{-}F}$ = 143.6 Hz).
^{13}C{^{1}H} (151 MHz, $CDCl_3$): δ = 66.3-65.7 (p, $^2J_{C\text{-}F}$ = 15.3 Hz), 56.5, 54.7, 48.1, 35.6, 34.1.
ATR-IR (ν_{max}): 2996, 2919, 1210, 1193, 1144, 1031, 904, 897, 816, 784, 644 cm^{-1}.
HRMS (*m/z*): (ESI) calc'd for $C_{10}H_{11}ClF_5S^-$ [M-H]$^-$: calculated 293.0195, found 293.0193.

Compound 46

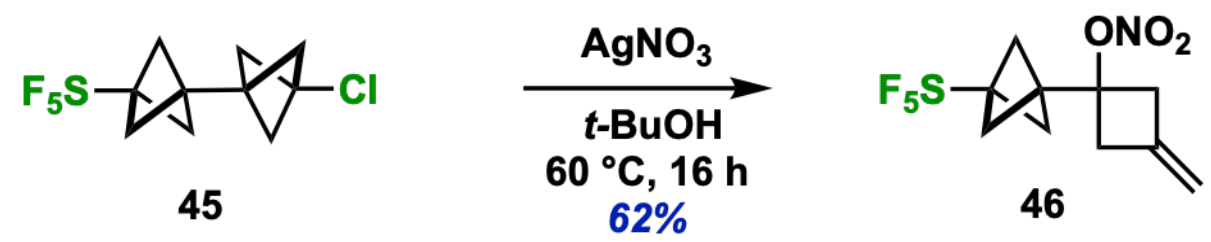

To an oven-dried microwave vial equipped with a stir bar were added compound **45** (0.500 g, 1.70 mmol, 1.0 equiv.) and silver nitrate (0.577 g, 3.40 mmol, 2.0 equiv.), followed by *tert*-butanol (17 mL). The resulting suspension was heated to 60 °C in an oil bath for 16 h. Upon reaction completion, the reaction was cooled to room temperature, diluted with EtOAc (20 mL), filtered through Celite, and concentrated. The crude residue was purified *via* column chromatography on silica gel eluting with 100% hexanes to afford a white solid (0.338 g, 62%).

3-methylene-1-(3-(pentafluoro-λ^6-sulfanyl)bicyclo[1.1.1]pentan-1-yl)cyclobutyl nitrate (**46**):
R_f: 0.39 (hexanes), vis: *p*-anisaldehyde.
m.p.: 49.5–52.5 ºC.
^{1}H (300 MHz, CDCl$_3$): δ = 5.01-4.98 (m, 2H), 3.13-2.94 (m, 4H), 2.37 (s, 6H).
^{19}F (282 MHz, CDCl$_3$): δ = 79.67-77.60 (p, 1F, $^2J_{F-F}$ = 143.6 Hz), 49.35-48.84 (d, 4F, $^2J_{F-F}$ = 143.6 Hz).
^{13}C{^{1}H} (151 MHz, CDCl$_3$): δ = 137.0, 109.7, 80.8, 65.8-65.1 (m), 54.1-54.0 (m), 41.0, 38.0.
ATR-IR (ν_{max}): 2986, 2932, 1629, 1294, 1212, 821, 792, 752 cm^{-1}.
HRMS (*m/z*): (ESI) calc'd for $C_{10}H_{13}F_5NO_3S^+$ [M-H]$^+$: calculated 322.0531, found 322.0537.

Compound 47

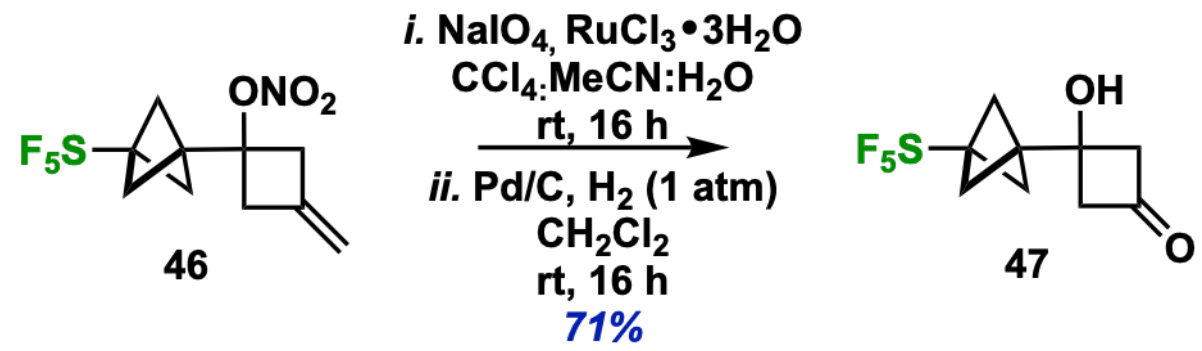

To an oven-dried microwave vial equipped with a stir bar was added compound **46** (0.420 g, 1.31 mmol, 1.0 equiv.) and a solvent mixture of 1:1:2 CCl_4:MeCN:H_2O (15 mL). Then, sodium periodate (1.12 g, 5.23 mmol, 4.0 equiv.) was added and allowed to completely dissolve before addition of ruthenium trichloride trihydrate (11.0 mg, 0.040 mmol, 0.03 equiv.). The resulting reaction mixture stirred at room temperature for 16 h. Upon reaction completion, the reaction was quenched with sat. aq. NH_4Cl (20 mL). The aqueous layer was extracted with EtOAc (3 x 10 mL). The combined organic layers were washed with brine (3 x 10 mL), dried over anhydrous Na_2SO_4, filtered through Celite, and concentrated. The crude residue was moved forward to the next step without further purification. (*Note: The material is meta stable on silica gel but is clean enough by ^1H NMR to be used in the next reaction.)*

To an oven-dried microwave vial equipped with a stir bar the crude material from the previous step was redissolved in CH_2Cl_2 (6.15 mL). Then, 10 wt.% Pd/C (30.0 mg) was added, and the reaction was sparged with a H_2 balloon. The H_2 atmosphere was maintained over the course of the reaction. Upon reaction completion by TLC analysis, the reaction mixture was filtered through Celite and concentrated. The crude residue was recrystallized from 9:1 hexanes:CH_2Cl_2 to afford a white solid (0.259 g, 71%, over two steps). (*Note: The material is metastable on silica gel, resulting in ring opening of the cyclobutane to yield the retro-aldol product. See next page for details on synthesis procedure to obtain exclusively the retro-aldol product.)*

3-hydroxy-3-(3-(pentafluoro-λ^6-sulfanyl)bicyclo[1.1.1]pentan-1-yl)cyclobutan-1-one (**47**):
m.p.: 115.9–118.2 ºC.
^{1}H (300 MHz, $CDCl_3$): δ = 3.15 (s, 4H), 2.35 (s, 6H), 1.99 (s, 1H).
^{19}F (282 MHz, $CDCl_3$): δ = 79.57-77.50 (p, 1F, $^2J_{F\text{-}F}$ = 143.6 Hz), 49.02-48.51 (d, 4F, $^2J_{F\text{-}F}$ = 143.6 Hz).
^{13}C{^{1}H} (151 MHz, $CDCl_3$): δ = 202.9, 66.6, 65.8-65.4 (m), 56.9, 52.6-52.5 (m), 40.3.
ATR-IR (ν_{max}): 3432, 3012, 2941, 2882, 1773, 1232, 1214, 813, 792, 780 cm^{-1}.
HRMS (*m/z*): (ESI) calc'd for $C_9H_{12}F_5O_2S^+$ [M-H]$^+$: calculated 279.0473, found 279.0480.

Compound 48

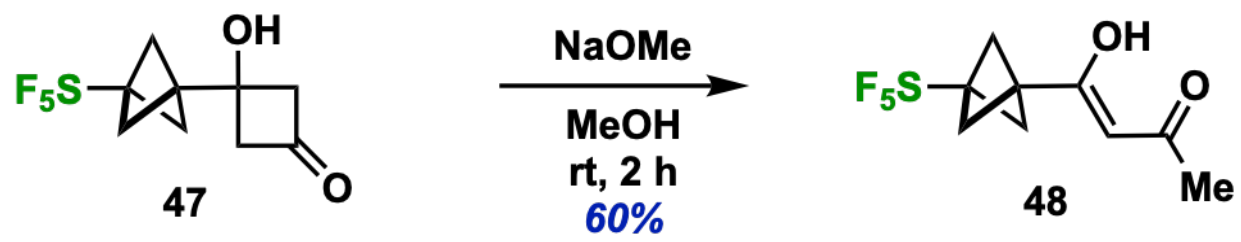

To an oven-dried microwave vial equipped with a stir bar was added compound **47** (20.0 mg, 0.072 mmol, 1.0 equiv.). The vial was evacuated and purged three times with Ar. Anhydrous MeOH (0.36 mL) was added, followed by sodium methoxide (8.00 mg, 0.144 mmol, 2.0 equiv.). The resulting reaction mixture was stirred at room temperature for 2 h. Upon reaction completion, the reaction was quenched with sat. aq. NH_4Cl (2 mL). The aqueous layer was extracted with EtOAc (3 x 3 mL). The combined organic layers were washed with brine (3 x 3 mL), dried over Na_2SO_4, filtered through Celite, and concentrated to afford a white solid (12.0 mg, 60%). (*Note: If necessary, the product can be further purified on silica eluting with 1:1 hexanes:CH_2Cl_2.)*

(*Z*)-4-hydroxy-4-(3-(pentafluoro-λ^6-sulfanyl)bicyclo[1.1.1]pentan-1-yl)but-3-en-2-one (**48**):
R_f: 0.38 (1:1 hexanes:CH_2Cl_2), vis: *p*-anisaldehyde.
m.p.: 54.5–58.6 ºC.
^{1}H (300 MHz, $CDCl_3$): δ = 15.08 (s, 1H), 5.50 (s, 1H), 2.53 (s, 6H), 2.10 (s, 3H).
^{19}F (282 MHz, $CDCl_3$): δ = 79.45-77.38 (p, 1F, $^2J_{F-F}$ = 143.6 Hz), 49.07-48.56 (d, 4F, $^2J_{F-F}$ = 143.6 Hz).
^{13}C{^{1}H} (151 MHz, $CDCl_3$): δ = 193.8, 184.7, 98.5, 66.2-65.8 (m), 55.9-55.8 (m), 36.7, 25.6.
ATR-IR (ν_{max}): 3433. 3027, 2991, 2934, 1616, 1423, 1194, 906, 793, 771 cm^{-1}.
HRMS (*m/z*): (ESI) calc'd for $C_9H_{12}F_5O_2S^+$ [M-H]$^+$: calculated 279.0473, found 279.0480.

Procedures for SF_5–BCP and SF_5–[*2*]staffane Building Blocks

Compound 2

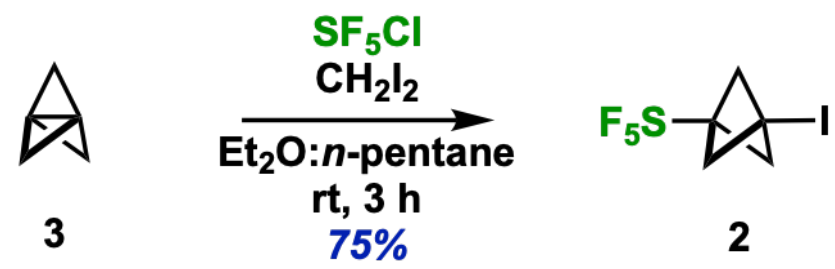

3 2

The synthesis of compound **2** was adapted from a known literature procedure.[14] An oven-dried 250 mL round bottom flask equipped with a stir bar was cooled to room temperature under vacuum and purged with Ar three times. The Ar line was removed and replaced with a fresh Ar balloon to provide an Ar cushion throughout the reaction duration. The septum was wrapped with electrical tape to ensure a tight seal during the reaction. Then, a ~0.1 M solution of SF_5Cl in *n*-pentane (18.7 mmol, 1.0 equiv.) was added *via* syringe, followed by CH_2I_2 (1.51 mL, 18.7 mmol, 1.0 equiv.). To the reaction mixture was added an ~0.8 M solution of [1.1.1]propellane in Et_2O (37.5 mmol, 2.0 equiv.), and the reaction was left to stir at room temperature for 3 h. Upon reaction completion, the stir bar was removed, and the reaction mixture was carefully concentrated. (*Note: Compound **2** is a volatile white solid; special precautions were taken when subjecting it to reduced pressures. While concentrating on the rotary evaporator, the water bath was set to 20 °C, and the pressure was reduced to no lower than 200 mBar. Compound **2** will sublime if left under high vacuum for more than ~15-20 min.)* The crude residue was purified *via* column chromatography on silica gel eluting with 100% hexanes to afford a white solid (4.50 g, 75%).

pentafluoro(3-iodobicyclo[1.1.1]pentan-1-yl)- λ^6-sulfane (**2**):
R_f: 0.67 (hexanes), vis: UV (254 nm).
m.p.: 74.1–77.9 ºC.
^{1}H (300 MHz, $CDCl_3$): δ = 2.73 (s, 6H).
^{19}F (283 MHz, $CDCl_3$): δ = 79.18-76.10 (p, 1F, $^2J_{F\text{-}F}$ = 145.3 Hz), 51.30-50.78 (d, 4F, $^2J_{F\text{-}F}$ = 146.4 Hz).
^{13}C{^{1}H} (76 MHz, $CDCl_3$): δ = 69.3-68.8 (m), 63.2-63.1 (m), -4.3.
ATR-IR (ν_{max}): 3029, 2984, 2931, 1200, 822, 788 cm^{-1}.
HRMS (*m/z*): (ESI) calc'd for $C_5H_5F_5IS^-$ [M-H]$^-$: calculated 318.9082, found 318.9072.

Compound S-22

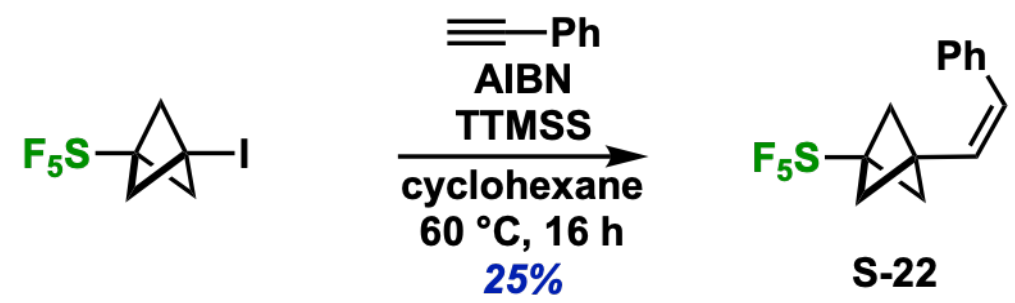

The synthesis of compound **S-22** was adapted from a known literature procedure.[14] To an oven-dried round bottom equipped with a stir bar was added compound **2** (1.30 g, 4.06 mmol, 1.0 equiv.) and AIBN (0.334 g, 2.03 mmol, 0.5 equiv.). The round bottom was sealed with a septum, then evacuated and backfilled with Ar three times. Anhydrous cyclohexane (13 mL) was added followed by phenyl acetylene (2.87 mL, 32.5 mmol, 8.0 equiv.), and TTMSS (2.02 mL, 6.09 mmol, 1.5 equiv.) *via* syringe. The reaction mixture was stirred at 60 °C in an oil bath for 12 h. The reaction mixture was cooled to room temperature and concentrated. The crude residue was purified *via* column chromatography on silica gel eluting with 100% hexanes to afford a light-yellow oil. (0.301 mg, 25 %).

(*Z*)-pentafluoro(3-styrylbicyclo[1.1.1]pentan-1-yl)-λ^6-sulfane (**S-22**):

R_f: 0.44 (hexanes), vis: UV (254 nm).

^{1}H (300 MHz, $CDCl_3$): δ = 7.37-7.28 (m, 3H), 7.17-7.14 (m, 2H), 6.71-6.67 (d, 1H, $^2J_{H\text{-}H}$ = 11.64 Hz), 5.70-5.66 (d, 1H, $^2J_{H\text{-}H}$ = 11.81 Hz), 2.25 (s, 6H).

^{19}F (282 MHz, $CDCl_3$): δ = 80.75-78.68 (p, 1F, $^2J_{F\text{-}F}$ = 145.3 Hz), 48.83-48.32 (d, 4F, $^2J_{F\text{-}F}$ = 145.4 Hz).

^{13}C{^{1}H} (76 MHz, $CDCl_3$): δ = 136.6, 133.8, 128.8, 128.0, 127.4, 126.3-126.2 (m), 67.5-67.1 (m), 57.9-57.7 (m), 34.4.

ATR-IR (ν_{max}): 3013, 2928, 1492, 1195, 904, 797 cm^{-1}.

HRMS (*m/z*): (ESI) calc'd for $C_{13}H_{12}F_5S^-$ [M-H]$^-$: calculated 295.0585, found 295.0591.

Compound 4

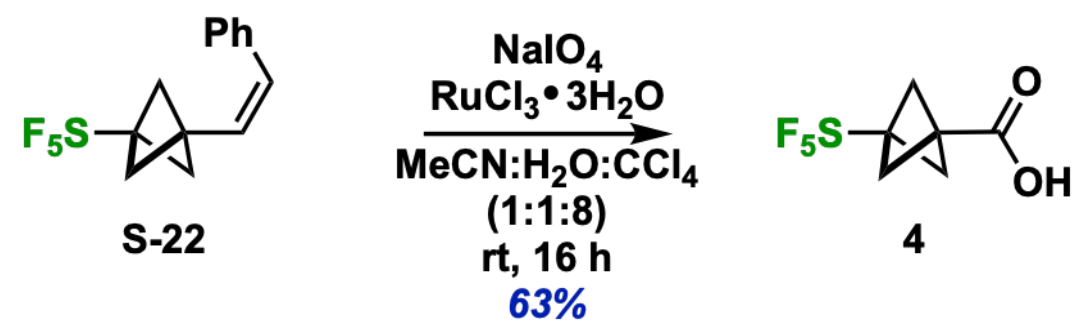

To a round bottom equipped with a stir bar was added compound **S-25** (2.00 g, 6.78 mmol, 1.0 equiv.) followed by a solvent mixture of 1:1:8 MeCN:CCl_4:H_2O (56 mL). Sodium periodate (5.81 g, 27.1 mmol, 4.0 equiv.) was added, and the mixture was stirred at room temperature until all solids dissolved. Subsequently, ruthenium trichloride trihydrate (53.0 mg, 0.200 mmol, 0.03 equiv.) was added. The round bottom was sealed, and the reaction mixture was stirred at room temperature for 19 h. The reaction mixture was then diluted with 1 M HCl (100 mL). The aqueous layer was extracted with CH_2Cl_2 (3 x 20 mL). The combined organic layers were washed with brine (3 x 25 mL), dried over anhydrous Na_2SO_4, filtered through Celite, and concentrated. The crude residue was purified *via* column chromatography on silica gel eluting with 4:1 hexanes:EtOAc to remove the benzoic acid side product. The column was then flushed with 3% AcOH in 4:1 hexanes:EtOAc (2 L) to obtain compound **4.** Once concentrated, the resulting solid was triturated with CH_2Cl_2 (3 mL) and filtered. The precipitate was washed with CH_2Cl_2 (0.25 mL) to afford a white solid (1.02 g, 63%).

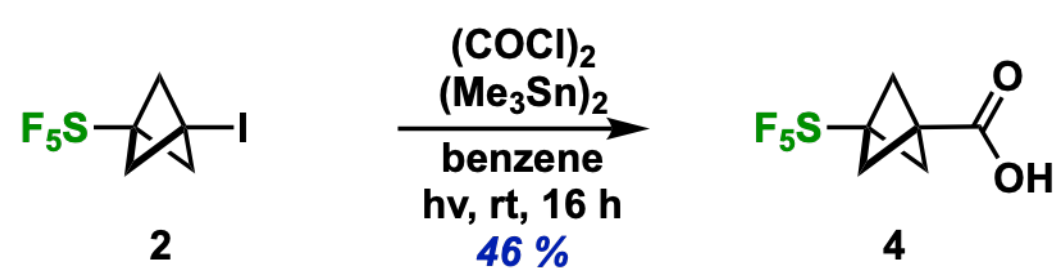

To an oven-dried microwave vial equipped with a stir bar was added compound **2** (1.00 g, 3.12 mmol, 1.0 equiv.). The vial was sealed, evacuated, and backfilled with Ar three times. Anhydrous benzene (16 mL) was added *via* syringe, followed by oxalyl chloride (0.374 mL, 4.37 mmol, 1.4 equiv.) and hexamethylditin (1.30 mL, 6.25 mmol, 2.0 equiv.). The resulting reaction mixture was irradiated with white LEDs (Kessil A160WE Tuna Blue, maximum intensity) for 16 h (the vial was placed ca. 5 cm away from the light source). Upon reaction completion, the reaction was cooled to room temperature, transferred to an Erlenmeyer flask open to air, diluted with 1 M HCl (16 mL), and left to stir for 1 h. The reaction mixture was washed with EtOAc (3 x 30 mL). The combined organic layers were washed with brine (3 x 40 mL), dried over anhydrous Na_2SO_4, filtered through Celite, and concentrated. The crude residue was purified *via* column chromatography on silica gel eluting with 4:1 hexanes:EtOAc to first remove any impurities followed by a 3% AcOH in 4:1 hexanes:EtOAc flush to afford a white solid (0.331 g, 46%). (*Note: Both oxalyl chloride and hexamethylditin are extremely moisture sensitive and were kept in a nitrogen filled glovebox for long-term storage; portions were transferred into sealed microwave vials and brought outside of the glovebox as needed.)*

3-(pentafluoro-λ^6-sulfanyl)bicyclo[1.1.1]pentane-1-carboxylic acid (**4**):
R_f: 0.19 (3% AcOH in 4:1 hexanes:EtOAc), vis: $KMnO_4$.
m.p.: 184.0–184.4ºC.
^{1}H (300 MHz, $CDCl_3$): δ = 2.61 (s, 6H).
^{19}F (282 MHz, $CDCl_3$): δ = 80.41-78.35 (p, 1F, $^2J_{F\text{-}F}$ = 148.6 Hz), 48.59-48.07 (d, 4F, $^2J_{F\text{-}F}$ = 146.3 Hz).
^{13}C{^{1}H} (101 MHz, $CDCl_3$): δ = 173.4, 66.0-65.3 (m), 56.2, 33.8.
ATR-IR (ν_{max}): 2925, 2852, 1659, 1422, 1220, 827, 802 cm^{-1}.
HRMS (*m/z*): (ESI) calc'd for $C_6H_6F_5OS^-$ $[M-H]^-$: calculated 237.0014, found 237.0010.

Compound 14

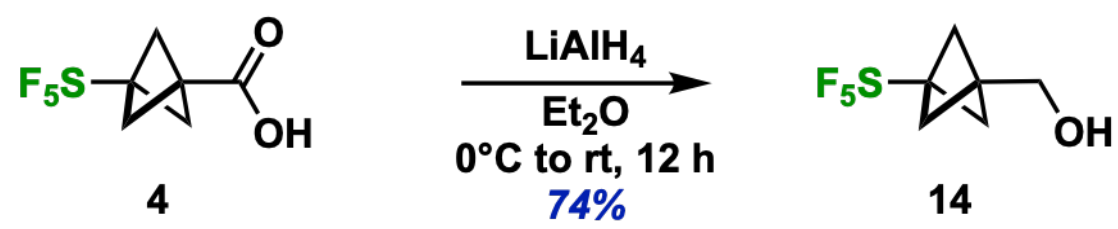

To an oven-dried microwave vial equipped with a stir bar was added compound **4** (0.040 g, 0.168 mmol, 1.0 equiv.). The vial was sealed with a cap with a septum using a crimper and then evacuated and backfilled with Ar three times. Et_2O (1.0 mL) was added via syringe. The reaction mixture was cooled to 0 °C in an ice bath and. 2 M $LiAlH_4$ in THF (0.42 mL, 0.840 mmol, 5.0 equiv.) was added dropwise *via* syringe. The reaction mixture was stirred at room temperature for 12 h. The reaction mixture was quenched with H_2O (0.5 mL). The aqueous layer was extracted with Et_2O (3 x 5 mL). The combined organic layers were washed with brine (20 mL) and sat. Rochelle salts (1 ml), dried over anhydrous Na_2SO_4, filtered through Celite, and concentrated at 250 mbar to afford a white solid (28.0 mg, 74%). (*Note: the solid is volatile under vacuum.*)

(3-(pentafluoro-λ^6-sulfanyl)bicyclo[1.1.1]pentan-1-yl)methanol (**14**):
R_f: 0.17 (3:1 hexanes:EtOAc), vis: *p*-anisaldehyde.
m.p.: 96.2–98.8 ºC.
^{1}H (300 MHz, $CDCl_3$): δ = 3.74 (s, 2H), 2.26 (s, 6H).
^{19}F (282 MHz, $CDCl_3$): δ = 80.32-78.26 (p, 1F, $^2J_{F\text{-}F}$ = 146.3 Hz), 48.43-47.92 (d, 4F, $^2J_{F\text{-}F}$ = 146.5 Hz).
^{13}C{^{1}H} (101 MHz, $CDCl_3$): δ = 67.3-66.7 (m), 61.1-61.1 (m), 53.9-53.9 (m), 35.9.
ATR-IR (ν_{max}): 3313, 2931, 1188, 813, 784 cm^{-1}.
HRMS (*m/z*): (ESI) calc'd for $C_6H_{10}F_5OS^+$ [M-H]$^+$: calculated 225.0367, found 225.0364.

Compound 18

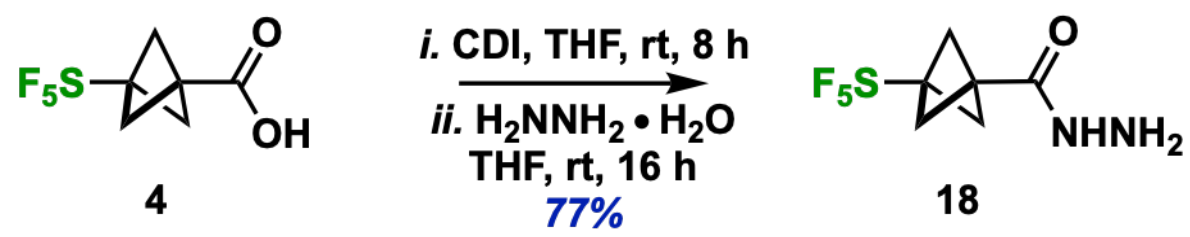

To an oven-dried microwave vial equipped with a stir bar were added compound **4** (0.100 g, 0.420 mmol, 1.0 equiv.) and CDI (0.089 g, 0.546 mmol, 1.3 equiv.). The vial was sealed with a septum, evacuated, and backfilled with Ar three times. Anhydrous THF (1.0 mL) was added via syringe. The reaction mixture was stirred at room temperature for 4 h. Hydrazine hydrate (0.130 mL, 3.78 mmol, 9.0 equiv.) was added dropwise *via* syringe. The reaction mixture was stirred at room temperature for 16 h. The crude reaction mixture was extracted with EtOAc (3 x 10 mL). The combined organic layers were washed with brine (3 x 20 mL), dried over anhydrous Na_2SO_4, filtered through Celite, and concentrated. The crude solid was precipitated from MTBE and filtered to afford a white solid (81.1 mg, 77%).

(3-(pentafluoroλ^6-sulfanyl)bicyclo[1.1.1]pentane-1-carbohydrazide (**18**):
R_f: 0.28 (19:1 CH_2Cl_2:MeOH), vis: *p*-anisaldehyde.
m.p.: 150.2–153.0 ºC.
^{1}H (300 MHz, $CDCl_3$): δ = 6.90 (s, 1H), 3.89 (d, 2H), 2.56 (s, 6H).
^{19}F (283 MHz, $CDCl_3$): δ = 79.02-76.95 (p, 1F, $^2J_{F\text{-}F}$ = 145.5 Hz), 49.10-48.58 (d, 4F, $^2J_{F\text{-}F}$ = 145.3 Hz).
^{13}C{^{1}H} (101 MHz, MeOD-*d₄*): δ = 168.3, 67.6-67.0 (m), 56.8-56.7 (m), 35.1.
ATR-IR (ν_{max}): 3292, 1659, 796, 647 cm^{-1}.
HRMS (*m/z*): (ESI) calc'd for $C_6H_8F_5OS^-$ [M-H]$^-$: calculated 251.0283, found 251.0277.

Compound 5

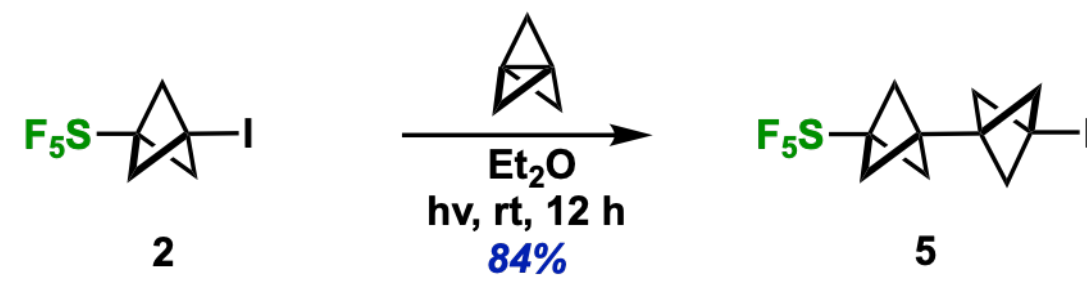

Six oven-dried microwave vials equipped with a stir bar were charged with compound **2** (0.300 g, 0.937 mmol, 1.0 equiv.), sealed with septa, and purged with Ar three times. Then, a ~0.8 M solution of [1.1.1]propellane in Et_2O (0.937 mmol, 1.0 equiv.) was added *via* syringe to each of the 6 microwave vials. The resulting reaction mixtures were irradiated using a 302 nm UV Pen lamp for 12 h. (*Note: See **Figure S24** for detailed reaction setup*.) Upon reaction completion, the stir bars were removed, and the combined reaction mixtures were combined and concentrated. The crude residue was purified *via* column chromatography on silica gel eluting with 100% hexanes to afford a white solid (1.82 g, 84%).

pentafluoro(3'-iodo-[1,1'-bi(bicyclo[1.1.1]pentan)]-3-yl)-λ^6-sulfane (**5**):
R_f: 0.31 (hexanes), vis: *p*-anisaldehyde.
m.p.: 131.8–133.2 ºC.
^{1}H (300 MHz, $CDCl_3$): δ = 2.22 (s, 6H), 2.18 (s, 6H).
^{19}F (282 MHz, $CDCl_3$): δ = 80.14-78.08 (p, 1F, $^2J_{F-F}$ = 145.6 Hz), 48.83-48.31 (d, 4F, $^2J_{F-F}$ = 145.6 Hz)
^{13}C{^{1}H} (151 MHz, $CDCl_3$): δ = 66.3-65.8 (p, $^2J_{C-F}$ = 17.4 Hz), 59.5, 54.7-54.6 (m), 45.8, 35.0, 5.9.
ATR-IR (ν_{max}): 2999, 2968, 2922, 2882, 1213, 1126, 1014, 899, 888, 847, 812, 780 cm^{-1}.
HRMS (*m/z*): (ESI) calc'd for $C_{10}H_{13}F_5IS^-$ [M-H]$^-$: calculated 386.9708, found 386.9716.

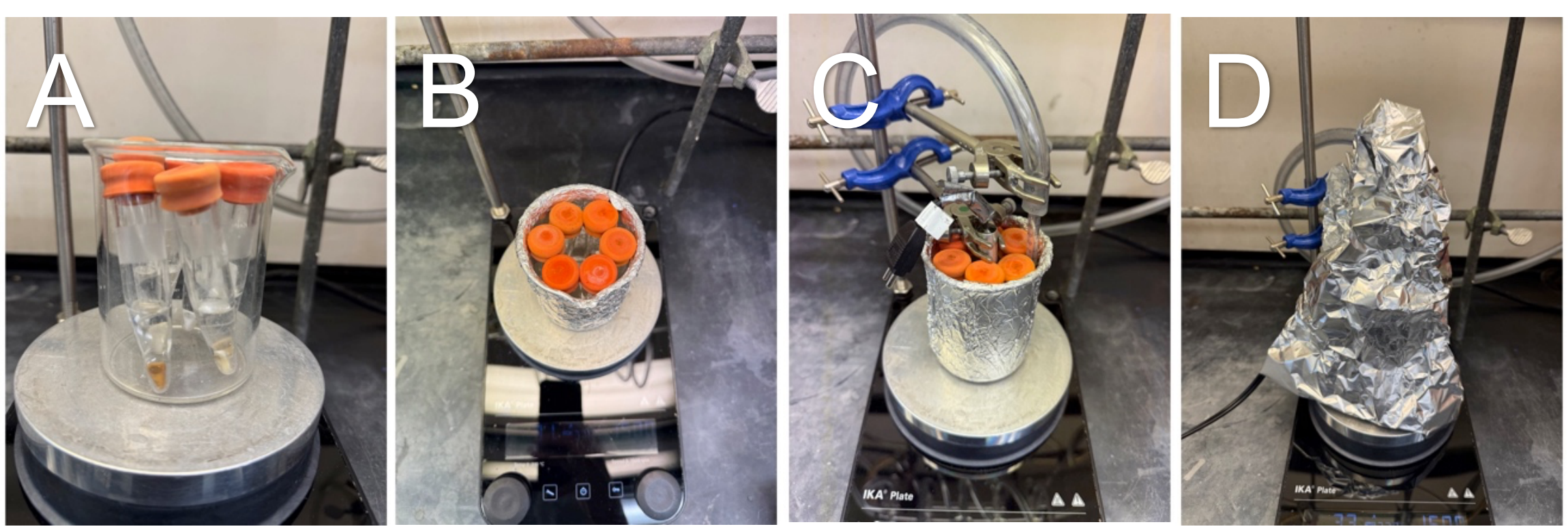

Figure S24. A: A 500 mL beaker containing six identical reaction vessels containing compound **2** and [1.1.1]propellane. **B:** The beaker is completely wrapped in aluminum foil, and the six vials are neatly arranged along the perimeter of the beaker. **C:** A 302 nm pen lamp is placed into the center of the six reaction vessels, and compressed air is blown through the beaker over the course of the reaction to mitigate heating. **D:** The entire reaction setup is covered with a large piece of aluminum foil to ensure stray light cannot escape while reaction mixtures are being irradiated.

Compound 6

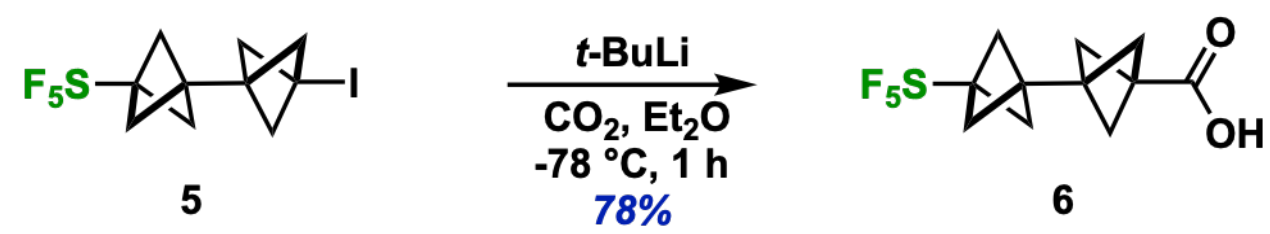

The synthesis of compound **6** was adapted from a known literature procedure.[15] To an oven-dried 100 mL round bottom flask equipped with a stir bar was added compound **5** (7.45 g, 19.3 mmol, 1.0 equiv.), and was sealed with a septum, evacuated, and backfilled with Ar three times. Then, anhydrous Et_2O (168 mL) was added *via* a syringe. The resulting mixture was cooled to -78 °C. To the cooled reaction mixture, 1.7 M solution of *t*-BuLi in pentane (25.0 mL, 42.5 mmol, 2.2 equiv.) was added dropwise *via* a cannula. After addition was complete, the reaction was left to stir at -78 °C for 1 h. Subsequently, the reaction was quenched at -78 °C using anhydrous CO_2 that was carefully bubbled through the reaction mixture using a balloon. The reaction mixture was diluted with 1 M HCl (40 mL) and extracted with EtOAc (3 x 30 mL). The combined organic layers were washed with brine (3 x 40 mL), dried over anhydrous Na_2SO_4, filtered through Celite, and concentrated. The crude residue was recrystallized from 4:1 CH_2Cl_2:EtOAc to afford a white solid (4.59 g, 78%). (*Note: Recrystallized product should only be washed with ice-cold CH_2Cl_2.)*

3'-(pentafluoro-λ^6-sulfanyl)-[1,1'-bi(bicyclo[1.1.1]pentane)]-3-carboxylic acid (**6**):

R_f: 0.45 (5% AcOH in 4:1 hexanes:EtOAc), vis: *p*-anisaldehyde.

m.p.: 166.4–167.3 ºC.

^{1}H (300 MHz, DMSO-*d6*): δ = 12.31 (bs, 1H), 2.20 (s, 6H), 1.85 (s, 6H).

^{19}F (282 MHz, DMSO-*d6*): δ = 83.17-81.11 (p, 1F, $^2J_{F\text{-}F}$ = 145.6 Hz), 49.35-48.82 (d, 4F, $^2J_{F\text{-}F}$ = 145.6 Hz).

^{13}C{^{1}H} (151 MHz, DMSO-*d6*): δ = 170. 8, 67.0-66.4 (p, $^2J_{C\text{-}F}$ = 17.4 Hz), 53.9, 50.2, 36.9, 36.6, 34.1.

ATR-IR (ν_{max}): 3008, 2985, 2927, 2889, 2596, 1696, 1434, 1318, 1216, 900, 814, 780, 749 cm^{-1}.

HRMS (*m/z*): (ESI) calc'd for $C_{11}H_{12}F_5O_2S^-$ [M-H]$^-$: calculated 303.0484, found 304.0477.

Compound S-23

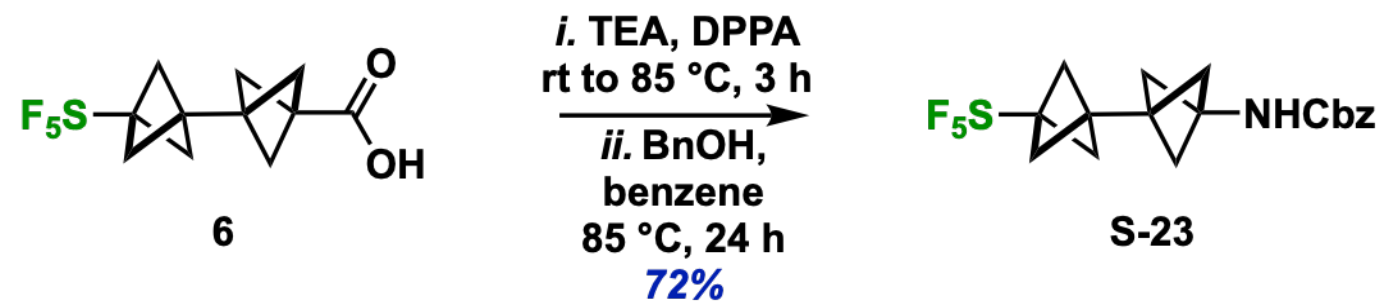

The synthesis of compound **S-23** was adapted from a known literature procedure.[15] To an oven-dried microwave vial equipped with a stir bar was added compound **6** (0.100 g, 0.329 mmol, 1.0 equiv.). The vial was sealed with a cap with a septum using a crimper, evacuated, and backfilled with Ar three times. Anhydrous benzene (1.64 mL) was added *via* syringe, followed by TEA (0.055 mL, 0.395 mmol, 1.2 equiv.), and dropwise addition of DPPA (0.082 mL, 0.378 mmol, 1.15 equiv.). The resulting mixture was stirred for 3 h at 85 °C in an oil bath. Then, benzyl alcohol (0.041 mL, 0.395, 1.2 equiv.) was added dropwise, and the resulting solution was stirred at 85 °C for 24 h. Subsequently, the reaction mixture was cooled, diluted with EtOAc (2 mL), and washed with 2 M NaOH (3 x 4 mL) and brine (3 x 4 mL). The combined organic layers were dried over anhydrous Na_2SO_4, filtered through Celite, and concentrated. The crude residue was purified *via* column chromatography on silica gel eluting with 5:1 hexanes:EtOAc to afford a white solid (97.0 mg, 72%).

benzyl (3'-(pentafluoro-λ^6-sulfanyl)-[1,1'-bi(bicyclo[1.1.1]pentan)]-3-yl)carbamate (**S-23**):
R_f: 0.26 (4:1 hexanes:EtOAc), vis: *p*-anisaldehyde.
m.p.: 142.9–144.0 ºC.
^{1}H (300 MHz, $CDCl_3$): δ = 7.37-7.31 (m, 5H), 5.14 (bs, 1H), 5.08 (s, 2H), 2.18 (s, 6H), 1.92 (s, 6H).
^{19}F (282 MHz, $CDCl_3$): δ = 80.50-78.44 (p, 1F, $^2J_{F-F}$ = 145.6 Hz), 48.53-48.01 (d, 4F, $^2J_{F-F}$ = 145.6 Hz).
^{13}C{^{1}H} (151 MHz, $CDCl_3$): δ = 150.5, 136.3, 129.9, 128.6, 128.2, 125.6, 120.1, 66.2-65.9 (m), 54.5-54.4 (m), 52.0, 45.3, 34.2.
ATR-IR (ν_{max}): 3321, 2982, 2921, 2882, 1697, 1528, 1517, 1285, 1247, 1212, 1099, 1052, 902, 813, 790, 734 cm^{-1}.
HRMS (*m/z*): (ESI) calc'd for $C_{18}H_{21}F_5NO_2S^+$ $[M-H]^+$: calculated 410.1208, found 410.1209.

Compound 49

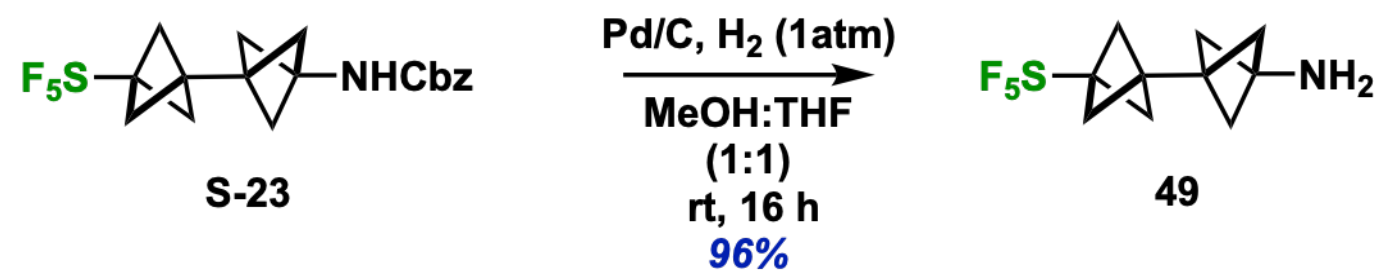

The synthesis of compound **49** was adapted from a known literature procedure.[15] To an oven-dried microwave vial equipped with a stir bar was added compound **S-23** (0.100 g, 0.244 mmol, 1.0 equiv.) and a solvent mixture of 1:1 MeOH:THF (1.2 mL). Then, 10 wt. % Pd/C (3.00 mg, 0.024 mmol, 0.1 equiv.) was added, and the reaction vessel was sealed and sparged with a H_2 balloon. The reaction mixture stirred under H_2 atmosphere for 16 h. Upon completion, the reaction mixture was filtered through Celite and concentrated to afford a white solid (64.0 mg, 96%), which required no further purification.

3'-(pentafluoro-λ^6-sulfanyl)-[1,1'-bi(bicyclo[1.1.1]pentan)]-3-amine (**49**):
R_f: 0.17 (10% TEA in hexanes:EtOAc), vis: *p*-anisaldehyde.
m.p.: 74.3–76.8 ºC.
^{1}H (300 MHz, $CDCl_3$): δ = 2.15 (s, 6H), 1.68 (s, 6H), 1.64 (bs, 2H).
^{19}F (282 MHz, $CDCl_3$): δ = 80.70-78.64 (p, 1F, $^2J_{F\text{-}F}$ = 145.6 Hz), 48.41-47.90 (d, 4F, $^2J_{F\text{-}F}$ = 145.6 Hz).
^{13}C{^{1}H} (151 MHz, $CDCl_3$): δ = 66.7-66.0 (m), 54.6-54.5 (m), 52.9, 48.7, 34.6, 32.1.
ATR-IR (ν_{max}): 3369, 3268, 2978, 2910, 2872, 1304, 1206, 901, 815, 785 cm^{-1}.
HRMS (*m/z*): (ESI) calc'd for $C_{18}H_{21}F_5NO_2S^+$ [M-H]$^+$: calculated 410.1208, found 410.1209.

Compound S-24

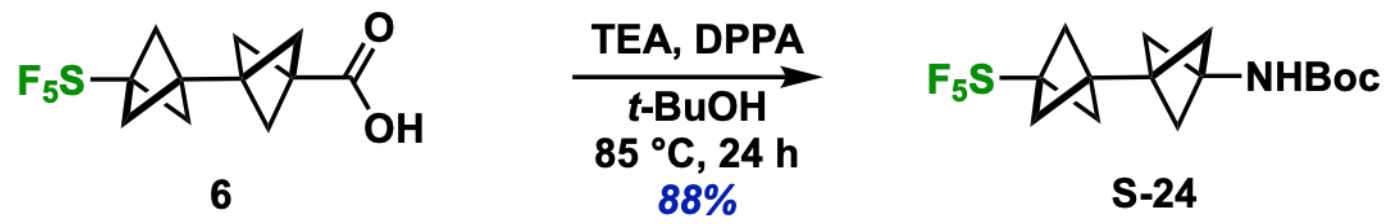

The synthesis of compound **S-24** was adapted from a known literature procedure.[15] To an oven-dried microwave vial equipped with a stir bar was added compound **6** (0.050 g, 0.164 mmol, 1.00 equiv.). The vial was then sealed with a cap with a septum using a crimper, evacuated and backfilled with Ar three times. Anhydrous *t*-BuOH (0.66 mL) was added *via* syringe, followed by TEA (0.027 mL, 0.197 mmol, 1.2 equiv.) and dropwise addition of DPPA (0.039 mL, 0.180 mmol, 1.1 equiv.). The resulting mixture was heated to 85 °C in an oil bath and stirred for 24 h or until all starting material was consumed by TLC. Upon reaction completion, the mixture was cooled and EtOAc (3 mL) was added. The solution was washed with 2 M NaOH (3 x 3 mL) and brine (3 x 4 mL). The combined organic layers were dried over anhydrous Na_2SO_4, filtered through Celite, and concentrated. The crude residue was purified *via* column chromatography on silica gel eluting with a gradient of 19:1 hexanes:EtOAc to 9:1 hexanes:EtOAc to afford a white solid (54.0 mg, 88%).

tert-butyl (3'-(pentafluoro-λ^6-sulfanyl)-[1,1'-bi(bicyclo[1.1.1]pentan)]-3-yl)carbamate (**S-24**):
R_f: 0.21 (9:1 hexanes:EtOAc), vis: *p*-anisaldehyde.
m.p.: 126.9–128.0 ºC.
^{1}H (300 MHz, $CDCl_3$): δ = 4.97 (bs,1H), 2.18 (s, 6H), 1.90 (s, 6H), 1.45 (s, 9H).
^{19}F (282 MHz, $CDCl_3$): δ = 80.56-78.50 (p, 1F, $^2J_{F\text{-}F}$ = 145.6 Hz), 48.49-47.97 (d, 4F, $^2J_{F\text{-}F}$ = 145.6 Hz).
^{13}C{^{1}H} (151 MHz, $CDCl_3$): δ = 155.0, 79.7, 66.6-65.9 (m), 54.6-54.5 (m), 52.1, 45.4, 34.5, 34.4, 28.4.
ATR-IR (ν_{max}): 3708, 3681, 3248, 3122, 2978, 2922, 2879, 1702, 1364, 1300, 1210, 1166, 1045, 1033, 1019, 842, 824, 793, 748 cm^{-1}.
HRMS (*m/z*): (ESI) calc'd for $C_{15}H_{23}F_5NO_2S^+$ $[M\text{-}H]^+$: calculated 376.1364, found 376.1365.

Compound S-25

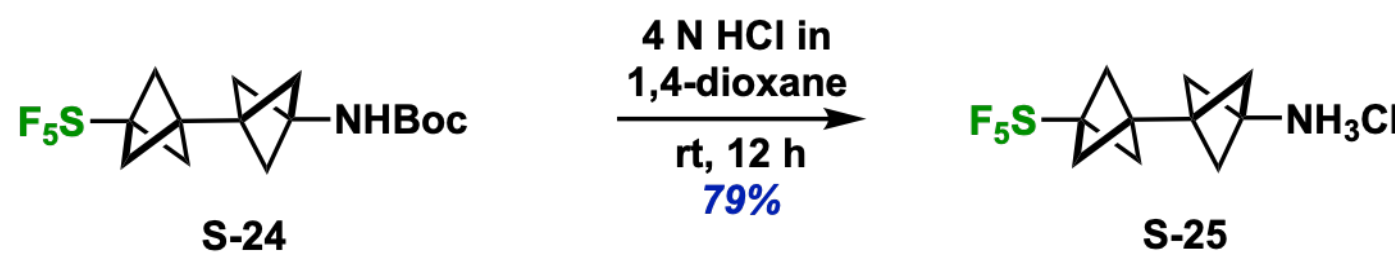

The synthesis of compound **S-25** was adapted from a known literature procedure.[15] To an oven-dried microwave vial equipped with a stir bar was added compound **S-24** (60.0 mg, 0.160 mmol, 1.0 equiv.). The vial was sealed with a septum, evacuated and backfilled with Ar three times. Then, 4 N HCl in 1,4-dioxane (1.0 mL) was added *via* syringe, and the reaction mixture was left to stir at room temperature for 12 h. Upon reaction completion, the resulting precipitate was filtered and washed with Et_2O (20 mL) to afford a white solid (39.0 mg, 79%), which required no further purification.

chloro(3'-(pentafluoro-λ^6-sulfanyl)-[1,1'-bi(bicyclo[1.1.1]pentan)]-3-yl)-λ^5-azane (**S-25**):

R_f: 0.39 (9:1 CH_2Cl_2:MeOH), vis: *p*-anisaldehyde.

m.p.: decomposes above 140.0 ºC.

^{1}H (300 MHz, DMSO-*d6*): δ = 9.00 (s, 1H), 2.23 (s, 6H), 1.88 (s, 6H).

^{19}F (282 MHz, DMSO-*d6*): δ = 83.35-81.28 (p, 1F, $^2J_{F-F}$ = 145.6 Hz), 49.63-49.11 (d, 4F, $^2J_{F-F}$ = 145.6 Hz).

^{13}C{^{1}H} (151 MHz, DMSO-*d6*): δ = 67.3-66.7 (m), 54.6, 51.3, 43.3, 34.7, 33.7.

ATR-IR (ν_{max}): 2972, 2924, 2886, 2821, 2725, 1241, 1212, 909, 817, 806, 787 cm^{-1}.

HRMS (*m/z*): (ESI) calc'd for $C_{10}H_{15}F_5NS^+$ $[M-H]^+$: calculated 276.0840, found 276.0848.

Compound S-26

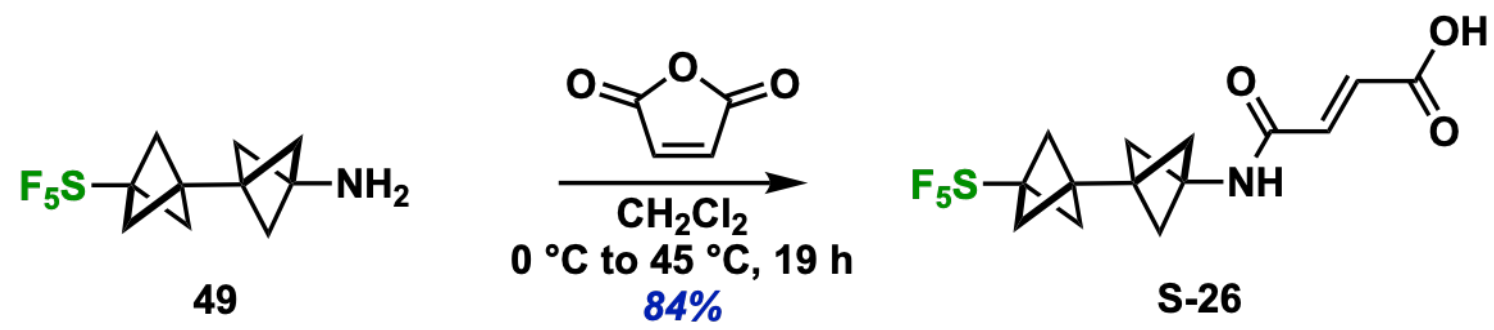

49 S-26

The synthesis of compound **S-26** was adapted from a known literature procedure.[16] To an oven-dried microwave vial equipped with a stir bar was added maleic anhydride (22.0 mg, 0.218 mmol, 1.0 equiv.). The vial was then sealed with a with a cap with a septum using a crimper, evacuated and backfilled with Ar three times. Anhydrous CH_2Cl_2 (0.3 mL) was added *via* syringe, and the reaction mixture was then cooled to 0 °C. In a separate oven-dried microwave vial, compound **49** (60.0 mg, 0.218 mmol, 1.0 equiv.) was added. The vial was sealed with a septum, evacuated, and backfilled with Ar three times. Anhydrous CH_2Cl_2 (0.9 mL) was added *via* syringe. The solution of compound **49** was then added to the cooled reaction mixture of maleic anhydride dropwise. After addition was complete, the reaction mixture was left to stir at 45 °C in an oil bath for 19 h. The resulting precipitate was filtered and washed with CH_2Cl_2 to afford a white solid (69.0 mg, 84%), which required no further purification.

4-oxo-4-((3'-(pentafluoro-λ^6-sulfanyl)-[1,1'-bi(bicyclo[1.1.1]pentan)]-3-yl)amino)but-2-enoic acid (**S-26**)
R_f: 0.14 (6:94, MeOH: CH_2Cl_2), vis: *p*-anisaldehyde.
m.p.: decomposes above 181.2 ºC.
^{1}H (300 MHz, (Acetone-*d*$_6$): δ = 15.52 (br s, 1H), 9.11 (br s, 1H), 6.49-6.45 (d, 1H, $^3J_{H-H}$ = 12.9 Hz), 6.29-6.25 (d, 1H, $^3J_{H-H}$ = 12.8 Hz), 2.29 (s, 6H), 2.10 (s, 6H).
^{19}F (283 MHz, (Acetone-*d*$_6$): δ = 80.45-78.45 (p, 1F, $^2J_{F-F}$ = 142.2 Hz), 47.83-47.32 (d, 4F, $^2J_{F-F}$ = 142.2 Hz).
^{13}C{^{1}H} (76 MHz, (DMSO-*d*$_6$) δ = 165.8, 165.5, 132.8, 130.9, 66.8-66.5 (m), 54.2, 51.8, 44.8, 35.1, 33.6.
ATR-IR (ν_{max}): 3252, 1625, 1529, 1215, 816, 787 cm^{-1}.
HRMS (*m/z*): (ESI) calc'd for $C_{14}H_{17}F_5NO_3S^+$ $[M-H]^+$: calculated 374.0844, found 374.0827.

Compound 50

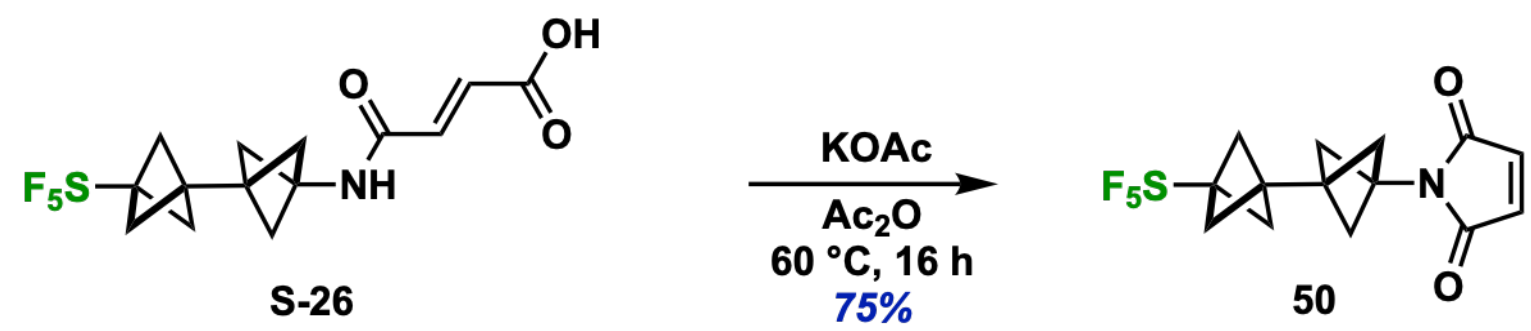

The synthesis of compound **50** was adapted from a known literature procedure.[16] To an oven-dried microwave vial equipped with a stir bar was added compound **S-26** (20.0 mg, 0.054 mmol, 1.0 equiv.) and KOAc (7.00 mg, 0.070 mmol, 1.3 equiv.). The vial was sealed with a cap with a septum using a crimper, evacuated, and backfilled with Ar three times. Ac_2O (0.3 mL) was then added *via* syringe. The resulting mixture was heated to 60 °C in an oil bath for 5 h. The reaction mixture was diluted with H_2O (5 mL) and washed with EtOAc (3 x 3 mL). The combined organic layers were washed with sat. aq. $NaHCO_3$ (3 x 4 mL), brine (3 x 4 mL), dried over anhydrous Na_2SO_4, filtered through Celite, and concentrated. The crude residue was purified *via* preparatory TLC eluting with 3:7 EtOAc:hexanes to afford a white solid (14.3 mg, 75%).

1-(3'-(pentafluoro-λ^6-sulfanyl)-[1,1'-bi(bicyclo[1.1.1]pentan)]-3-yl)-1*H*-pyrrole-2,5-dione (**50**):
R_f: 0.22 (86:14 hexanes:EtOAc), vis: *p*-anisaldehyde.
m.p.: decomposes above 157.5 ºC.
^{1}H (300 MHz, $CDCl_3$): δ = 6.61 (s, 2H), 2.20 (s, 6H), 2.20 (s, 6H).
^{19}F (282 MHz, $CDCl_3$): δ = 80.41-78.35 (p, 1F, $^2J_{F\text{-}F}$ = 145.9 Hz), 48.59-48.07 (d, 4F, $^2J_{F\text{-}F}$ = 145.9 Hz).
^{13}C{^{1}H} (101 MHz, $CDCl_3$): δ = 170.5, 134.3, 66.4-65.9 (m), 54.6-54.6 (m), 52.9, 43.8, 36.6, 34.2.
ATR-IR (ν_{max}): 2926, 1704, 1396, 1212, 791, 691 cm^{-1}.
HRMS (*m/z*): (ESI) calc'd for $C_{14}H_{15}F_5NO_2S^+$ $[M\text{-}H]^+$: calculated 356.0738, found 356.0730.

Compound 51

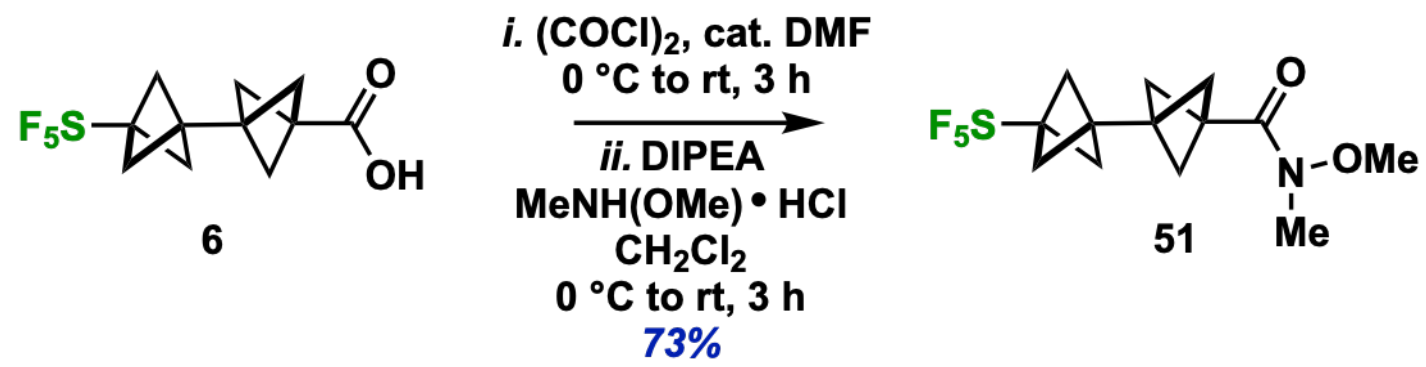

The synthesis of compound **51** was adapted from a known literature procedure.[17] To an oven-dried round bottom flask equipped with a stir bar was added compound **6** (1.25 g, 4.11 mmol, 1.00 equiv.). The round bottom flask was sealed with a septum, evacuated, and backfilled with Ar three times. Anhydrous CH_2Cl_2 (24 mL) was added *via* syringe. The resulting mixture was cooled to 0 °C in an ice-water bath. To the cooled reaction mixture was added anhydrous DMF (0.05 mL) followed by dropwise addition of $(COCl)_2$ (2.0 M in CH_2Cl_2; 2.26 mL, 4.52 mmol, 1.1 equiv.). After addition was complete, the reaction mixture was stirred at room temperature for 3 h. Subsequently, MeNH(OMe)·HCl (0.400 g, 4.11 mmol, 1.0 equiv.) was added quickly in one portion to conserve the Ar atmosphere and cooled to 0 °C. To the cooled reaction, was added DIPEA (2.51 mL, 14.4 mmol, 3.5 equiv.) dropwise. The reaction mixture was slowly warmed to room temperature and stirred for 3 h before being quenched with H_2O (10 mL). The aqueous layer was extracted with CH_2Cl_2 (3 x 10 mL). The combined organic layers were washed with brine (3 x 10 mL), dried over anhydrous Na_2SO_4, filtered through Celite, and concentrated. The crude residue was purified *via* column chromatography on silica gel eluting with 3:2 hexanes:EtOAc to afford a white solid (1.04 g, 73%). *(Caution: gas evolution occurs after oxalyl chloride addition, and the addition of DIPEA results in an exothermic reaction.)*

N-methoxy-*N*-methyl-3'-(pentafluoro-λ^6-sulfanyl)-[1,1'-bi(bicyclo[1.1.1]pentane)]-3-carboxamide (**51**):
R_f: 0.40 (1:1 hexanes:EtOAc), vis: *p*-anisaldehyde.
m.p.: 106.8–108.5 ºC.
^{1}H (300 MHz, $CDCl_3$): δ = 3.65 (s, 3H), 3.16 (s, 3H), 2.18 (s, 6H), 1.98 (s, 6H).
^{19}F (282 MHz, $CDCl_3$): δ = 80.19-78.64 (p, 1F, $^2J_{F-F}$ = 145.6 Hz), 48.33-47.94 (d, 4F, $^2J_{F-F}$ = 145.6 Hz).
^{13}C{^{1}H} (151 MHz, $CDCl_3$): δ = 170.0, 66.5-66.0 (p, $^2J_{C-F}$ = 17.4 Hz), 61.5, 54.3-54.3 (m), 51.5, 38.4, 38.0, 34.8, 32.5.
ATR-IR (ν_{max}): 2996, 2919, 2881, 1656, 1416, 1376, 1213, 989, 900, 819, 782, 700 cm^{-1}.
HRMS (*m/z*): (ESI) calc'd for $C_{13}H_{19}F_5NO_2S^+$ $[M-H]^+$: calculated 348.1051, found 348.1051.

Compound 32

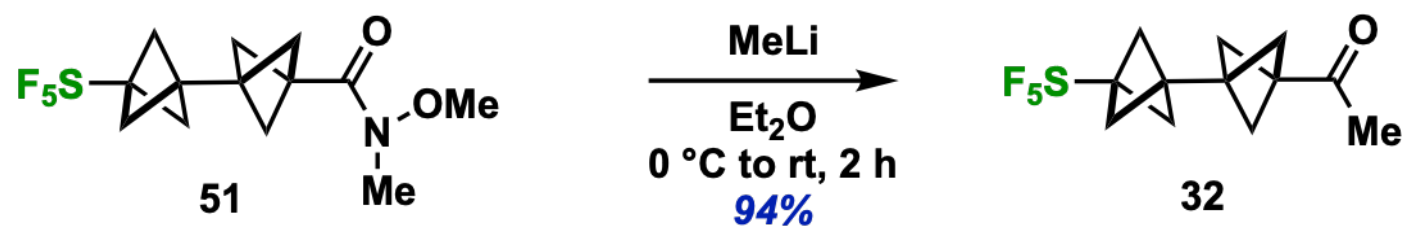

The synthesis of compound **32** was adapted from a known literature procedure.[18] To an oven-dried round bottom flask equipped with a stir bar was added compound **51** (1.43 g, 4.11 mmol, 1.0 equiv.). The round bottom flask was sealed with a septum, evacuated, and backfilled with Ar three times. Anhydrous Et_2O (24 mL) was added *via* syringe. The reaction mixture was cooled to 0 °C in an ice-water bath. To the cooled reaction mixture 1.6 M solution of MeLi in Et_2O (3.86 mL, 6.17 mmol, 1.5 equiv.) was added dropwise, and the reaction mixture was then stirred at room temperature for 2 h before being quenched with sat. aq. NH_4Cl (10 mL). The aqueous layer was extracted with EtOAc (3 x 10 mL). The combined organic layers were washed with brine (3 x 10 mL), dried over anhydrous Na_2SO_4, filtered through Celite, and concentrated. The crude residue was purified *via* column chromatography on silica gel eluting with 4:1 hexanes:EtOAc to afford a white solid (1.17 g, 94%).

1-(3'-(pentafluoro-λ^6-sulfanyl)-[1,1'-bi(bicyclo[1.1.1]pentan)]-3-yl)ethan-1-one (**32**):
R_f: 0.36 (4:1 hexanes:EtOAc), vis: *p*-anisaldehyde.
m.p.: 102.9–103.5 ºC.
^{1}H (300 MHz, $CDCl_3$): δ = 2.18 (s, 6H), 2.10 (s, 3H), 1.90 (s, 6H).
^{19}F (282 MHz, $CDCl_3$): δ = 80.07-78.53 (p, 1F, $^2J_{F-F}$ = 145.6 Hz), 48.35-47.96 (d, 4F, $^2J_{F-F}$ = 145.6 Hz).
^{13}C{^{1}H} (151 MHz, $CDCl_3$): δ = 206.0, 66.5-65.9 (p, $^2J_{C-F}$ = 17.4 Hz), 54.3-54.2 (m), 50.5, 43.7, 36.9, 34.8, 26.2.
ATR-IR (ν_{max}): 2990,2918, 2883, 1701, 1357, 1212, 899, 880, 813, 780 cm^{-1}.
HRMS (*m/z*): (ESI) calc'd for $C_{12}H_{16}F_5OS^+$ $[M-H]^+$: calculated 303.0836, found 303.0836.

Compound S-*27*

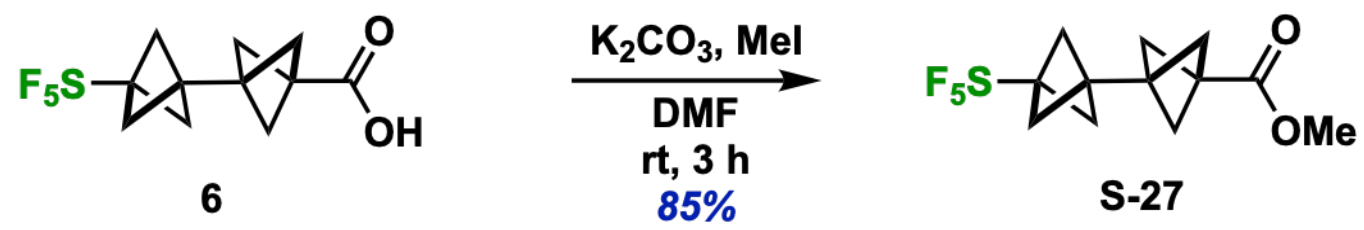

The synthesis of compound **S-30** was adapted from a known literature procedure.[19] An oven-dried microwave vial equipped with a stir bar was charged with compound **6** (0.100 g, 0.329 mmol, 1.0 equiv.) and K_2CO_3 (0.136 g, 0.987 mmol, 3.0 equiv.). The vial was sealed with a septum, evacuated and backfilled with Ar three times. Then, anhydrous DMF (1.65 mL) was added followed by dropwise addition of MeI (0.030 mL, 0.494 mmol, 1.5 equiv.). The resulting reaction mixture was left to stir at room temperature for 3 h. Upon completion, the reaction mixture was diluted with sat. aq. NH_4Cl (3 mL) and washed with EtOAc (3 x 3 mL). The combined organic layers were washed with brine (3 x 4 mL), dried over anhydrous Na_2SO_4, filtered through Celite, and concentrated to afford a white solid (89.0 mg, 85%), which required no further purification.

methyl 3'-(pentafluoro-λ^6-sulfanyl)-[1,1'-bi(bicyclo[1.1.1]pentane)]-3-carboxylate (**S-27**):
R_f: 0.40 (9:1 hexanes:EtOAc), vis: *p*-anisaldehyde.
m.p.: 122.3–124.4 ºC.
^{1}H (300 MHz, $CDCl_3$): δ = 3.66 (s, 3H), 2.18 (s, 6H), 1.93 (s, 6H).
^{19}F (282 MHz, $CDCl_3$): δ = 80.34-78.28 (p, 1F, $^2J_{F\text{-}F}$ = 145.6 Hz), 48.41-47.90 (d, 4F, $^2J_{F\text{-}F}$ = 145.6 Hz).
^{13}C{^{1}H} (151 MHz, $CDCl_3$): δ = 170.2, 66.5-65.8 (m), 54.3-54.2 (m), 51.8, 51.0, 37.5, 37.2, 34.7.
ATR-IR (ν_{max}): 2983, 2920, 2883, 1721, 1310, 1215, 1203, 1154, 1139, 1061, 904, 820, 787, 717 cm^{-1}.
HRMS (*m/z*): (ESI) calc'd for $C_{12}H_{16}F_5O_2S^+$ [M-H]$^+$: calculated 319.0786, found 319.0795.

Saponification of Compound S-27

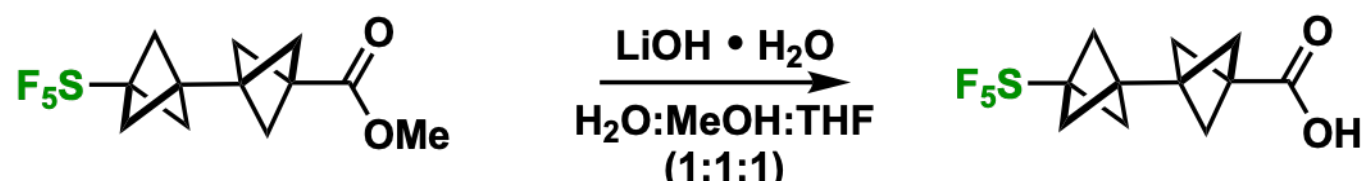

S-27 → 6: LiOH • H_2O, H_2O:MeOH:THF (1:1:1), rt, 2 h, *91%*

The synthesis of compound **6** was adapted from a known literature procedure.[20] To a microwave vial equipped with a stir bar was added compound **S-27** (16.0 mg, 0.050 mmol, 1.0 equiv.), followed by a solution of H_2O:MeOH:THF (0.45 mL). The reaction mixture was stirred. Subsequently, lithium hydroxide monohydrate (2.10 mg, 0.05 mmol, 1.0 equiv.) was added in one portion. The reaction mixture was stirred vigorously at room temperature until the starting material was consumed as indicated by TLC. The reaction mixture was then carefully acidified with 1 M HCl to ca. pH 4-5, and the aqueous layer was extracted with EtOAc (3 x 2 mL). The combined organic layers were dried over anhydrous $Na_2SO_4$and concentrated. The crude residue was recrystallized from 4:1 CH_2Cl_2:EtOAc to afford a white solid (14.0 mg, 91%). *(Note: Recrystallized product should only be washed with ice cold CH_2Cl_2.)*

3'-(pentafluoro-λ^6-sulfanyl)-[1,1'-bi(bicyclo[1.1.1]pentane)]-3-carboxylic acid (**6**):
R_f: 0.45 (5% AcOH in 4:1 hexanes:EtOAc), vis: *p*-anisaldehyde.
m.p.: 166.4–167.3 ºC.ºC.
^{1}H (300 MHz, DMSO-d_6): δ = 12.31 (bs, 1H), 2.20 (s, 6H), 1.85 (s, 6H).
^{19}F (282 MHz, DMSO-d_6): δ = 83.17-81.11 (p, 1F, $^2J_{F-F}$ = 145.6 Hz), 49.35-48.82 (d, 4F, $^2J_{F-F}$ = 145.6 Hz).
^{13}C{^{1}H} (151 MHz, DMSO-d_6): δ = 170. 8, 67.0-66.4 (p, $^2J_{C-F}$ = 17.4 Hz), 53.9, 50.2, 36.9, 36.6, 34.1.
ATR-IR (ν_{max}): 3008, 2985, 2927, 2889, 2596, 1696, 1434, 1318, 1216, 900, 814, 780, 749 cm^{-1}.
HRMS (*m/z*): (ESI) calc'd for $C_{11}H_{12}F_5O_2S^-$ [M-H]$^-$: calculated 303.0484, found 304.0477.

Compound 52

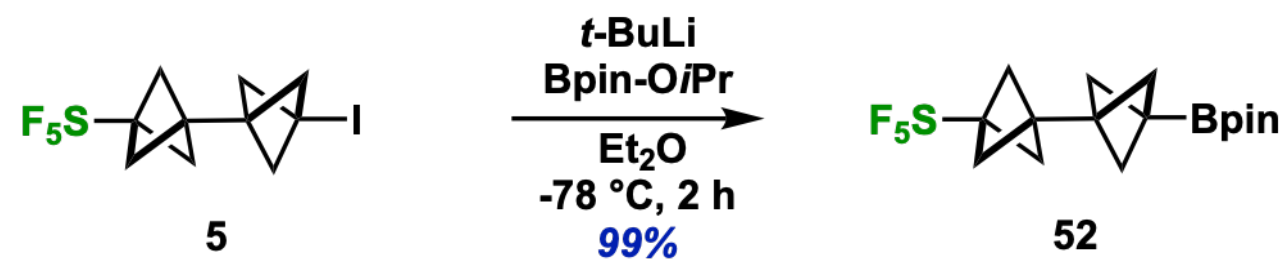

The synthesis of compound **52** was adapted from a known literature procedure.[21] To an oven-dried microwave vial equipped with a stir bar was added compound **5** (0.200 g, 0.518 mmol, 1.0 equiv.). The vial was sealed with a septum, evacuated and backfilled with Ar three times. Anhydrous Et_2O (3.0 mL) was added *via* syringe. The reaction mixture was then cooled to -78 °C, and 1.7 M solution of *t*-BuLi in pentane (0.67 mL, 1.14 mmol, 2.2 equiv.) was added dropwise *via* syringe. After addition was complete, the reaction mixture was stirred at -78 °C for 1 h. Then, Bpin-O*i*Pr (0.127 mL, 0.621 mmol, 1.2 equiv.) was added dropwise at -78 °C, and the cooling bath was removed to let the reaction mixture warm gradually to room temperature. Upon completion, the reaction mixture was diluted with 1 M HCl (3 mL) and washed with EtOAc (3 x 3 mL). The combined organic layers were washed with H_2O (3 x 4 mL), brine (3 x 4 mL), dried over anhydrous Na_2SO_4, filtered through Celite, and concentrated. The crude residue was recrystallized from boiling hexanes to afford a white solid (0.132 g, 99%). *(Note: Compound **52** is not stable on silica gel.)*

4,4,5,5-tetramethyl-2-(3'-(pentafluoro-λ^6-sulfanyl)-[1,1'-bi(bicyclo[1.1.1]pentan)]-3-yl)-1,3,2-dioxaborolane (**52**):

m.p.: 114.4–117.2 ºC.

^{1}H (300 MHz, $CDCl_3$): δ = 2.13 (s, 6H), 1.76 (s, 6H), 1.22 (s, 12H).

^{19}F (282 MHz, $CDCl_3$): δ = 80.17-78.65 (p, 1F, $^2J_{F\text{-}F}$ = 145.6 Hz), 48.18-47.67 (d, 4F, $^2J_{F\text{-}F}$ = 145.6 Hz).

^{13}C{^{1}H} (151 MHz, $CDCl_3$): δ = 83.5, 67.0-66.2 (m), 54.1-54.0 (m), 50.5, 42.9, 35.4, 24.8.

ATR-IR (ν_{max}): 2966, 2923, 2908, 2872, 1407, 1371, 1320, 1212, 1168, 1139, 1043, 1034, 899, 851, 822, 792 cm^{-1}.

HRMS (*m/z*): (ESI) calc'd for $C_{10}H_{13}F_5S^+$ $[M\text{-}H]^+$: calculated 260.0653, found 260.0561.

Compound 53

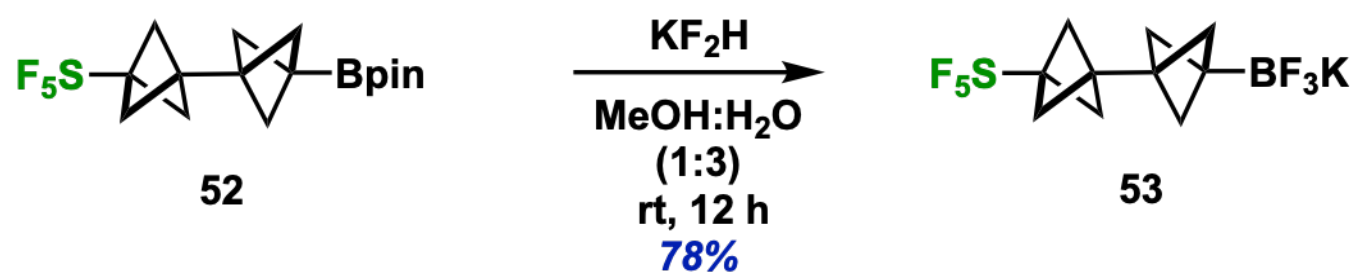

The synthesis of compound **53** was adapted from a known literature procedure.[21] To an oven-dried microwave vial equipped with a stir bar was added compound **52** (0.100 g, 0.259 mmol, 1.0 equiv.) and MeOH (5.0 mL). Then, KF_2H (55.0 mg, 0.699 mmol, 2.7 equiv.) was dissolved in H_2O (1.0 mL) and added dropwise to the stirred solution of compound **52**. The reaction mixture was stirred at room temperature for 12 h. *(Note: Upon, KF_2H addition a fine white precipitate formed that persisted throughout the course of the reaction.)* The reaction mixture was concentrated, and the residue was washed with hot acetone (3 x 5 mL). The combined acetone washes were concentrated, and the residue was washed with hot MTBE (3 x 5 mL) and dried under vacuum to afford a white solid (77.8 mg, 78%).

trifluoro(3'-(pentafluoro-λ^6-sulfanyl)-[1,1'-bi(bicyclo[1.1.1]pentan)]-3-yl)-λ^4-borane, potassium salt (**53**):
m.p.: decomposes above 200.0 ºC.
^{1}H (300 MHz, MeOD-*d₄*): δ = 2.13 (s, 6H), 1.49 (s, 6H).
^{19}F (282 MHz, MeOD-*d₄*): δ = 82.76-81.25 (p, 1F, $^2J_{F-F}$ = 145.6 Hz), 50.37-49.86 (d, 4F, $^2J_{F-F}$ = 145.6 Hz), -144.82 (s, 3F).
^{13}C{^{1}H} (151 MHz, MeOD-*d₄*:Acetone-*d₆*): δ = 54.9, 49.3, 40.6, 39.2, 36.8.
ATR-IR (ν_{max}): 2952, 2901, 2861, 1558, 1473, 1456, 1379, 1306, 1208, 975, 946, 810 cm^{-1}.
HRMS (*m/z*): (ESI) calc'd for $C_{10}H_{12}BF_8S^-$ [M-K+]$^-$: calculated 327.0631, found 327.0606.

Compound 54

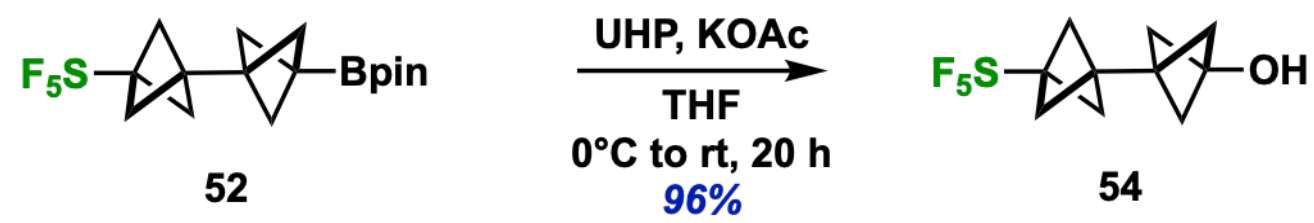

The synthesis of compound **54** was adapted from a known literature procedure.[22] To an oven-dried microwave vial equipped with a stir bar was added compound **52** (0.100 g, 0.259 mmol, 1.0 equiv.) and THF (1.29 mL). The solution was cooled to 0 °C, then urea hydrogen peroxide (29.0 mg, 0.311 mmol, 1.2 equiv.) was added, followed by potassium acetate (30.0 mg, 0.311 mmol, 1.2 equiv.). The cooling bath was removed, and the reaction mixture was stirred at room temperature for 20 h. Upon completion, the reaction mixture was diluted with sat. aq. NH_4Cl (3 mL) and washed with EtOAc (3 x 3 mL). The combined organic layers were washed with brine (3 x 4 mL), dried over anhydrous Na_2SO_4, filtered through Celite, and concentrated. The crude residue was purified *via* column chromatography on silica gel eluting with 100% CH_2Cl_2 to afford a white solid (68.7 mg, 96%).

3'-(pentafluoro-λ^6-sulfanyl)-[1,1'-bi(bicyclo[1.1.1]pentan)]-3-ol (**54**):

R_f: 0.26 (CH_2Cl_2), vis: *p*-anisaldehyde.

m.p.: 130.7–133.5 ºC.

^{1}H (300 MHz, $CDCl_3$): δ = 2.36 (s, 1H), 2.17 (s, 6H), 1.82 (s, 6H).

^{19}F (282 MHz, $CDCl_3$): δ = 80.54-78.48 (p, 1F, $^2J_{F\text{-}F}$ = 145.6 Hz), 48.56-48.04 (d, 4F, $^2J_{F\text{-}F}$ = 145.6 Hz).

^{13}C{^{1}H} (151 MHz, $CDCl_3$): δ = 66.3-66.0 (m), 62.3, 54.8-54.7 (m), 53.4, 34.1, 28.9.

ATR-IR (ν_{max}): 3201, 2980, 2915, 2877, 1263, 1209, 1073, 905, 816, 798, 648, 633 cm^{-1}.

HRMS (*m/z*): (ESI) calc'd for $C_{10}H_{12}F_5OS^-$ [M-H]$^-$: calculated 275.0535, found 275.0533.

Compound 55

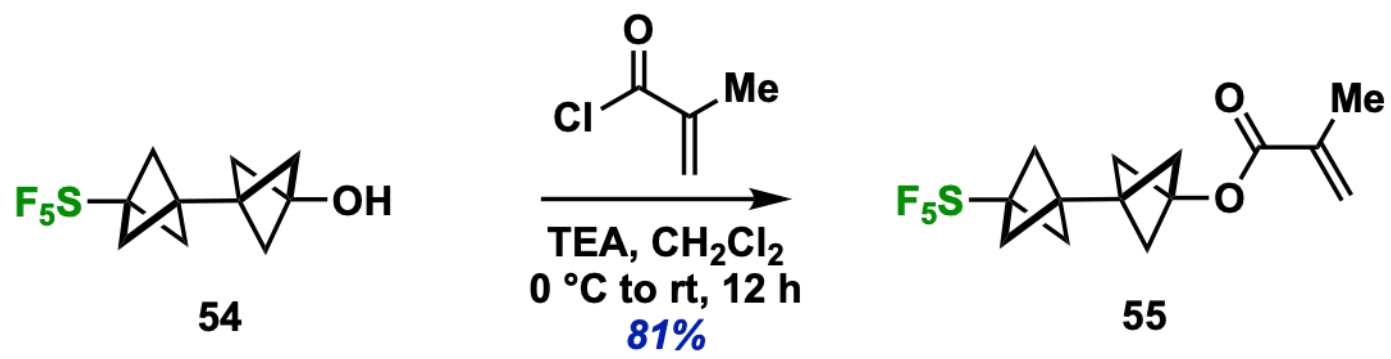

The synthesis of compound **55** was adapted from a known literature procedure.[23] An oven-dried microwave vial equipped with a stir bar was charged with compound **54** (0.034 g, 0.124 mmol, 1.0 equiv.). The vial was sealed with a septum, evacuated and backfilled with Ar three times. Then, anhydrous CH_2Cl_2 (0.62 mL) was added, and the reaction mixture was cooled to 0 °C using an ice-water bath. Then, TEA (0.021 mL, 0.149 mmol, 1.2 equiv.) was added dropwise, followed by 1 M solution of methacryloyl chloride in CH_2Cl_2 (0.149 mL, 0.149 mmol, 1.2 equiv.). The cooling bath was removed, and the reaction mixture was stirred for 12 h at room temperature. Upon completion, the reaction mixture was diluted with H_2O (3 mL) and extracted with CH_2Cl_2 (3 x 3 mL). The combined organic layers were washed with brine (3 x 4 mL), dried over anhydrous Na_2SO_4, filtered through Celite, and concentrated. The crude residue was purified *via* column chromatography on silica gel eluting with 9:1 hexanes:CH_2Cl_2 to 3:2 hexanes:CH_2Cl_2 to afford a white solid (32.9 mg, 81%).

3'-(pentafluoro-λ^6-sulfanyl)-[1,1'-bi(bicyclo[1.1.1]pentan)]-3-yl methacrylate (**55**):

R_f: 0.36 (19:1 hexanes:EtOAc), vis: *p*-anisaldehyde.

m.p.: 117.0–119.0 ºC.

^{1}H (300 MHz, $CDCl_3$): δ = 6.08-6.07 (m, 1H), 5.58-5.56 (m, 1H), 2.21 (s, 6H), 2.08 (s, 6H), 1.91-1.90 (m, 3H).

^{19}F (282 MHz, $CDCl_3$): δ = 80.41-78.35 (p, 1F, $^2J_{F\text{-}F}$ = 145.6 Hz), 48.63-48.11 (d, 4F, $^2J_{F\text{-}F}$ = 145.6 Hz).

^{13}C{^{1}H} (151 MHz, $CDCl_3$): δ = 166.88, 136.43, 126.07, 66.56-65.76 (m), 63.13, 54.87-54.74 (m), 53.09, 33.09, 32.29, 18.27.

ATR-IR (ν_{max}): 3006, 2984, 2925, 2886, 1714, 1235, 1212, 1161, 1143, 902, 820, 795, 782, 645 cm^{-1}.

HRMS (*m/z*): (ESI) calc'd for $C_{14}H_{18}F_5O_2S^+$ [M-H]$^+$: calculated 345.0942, found 345.0945.

Compound 56

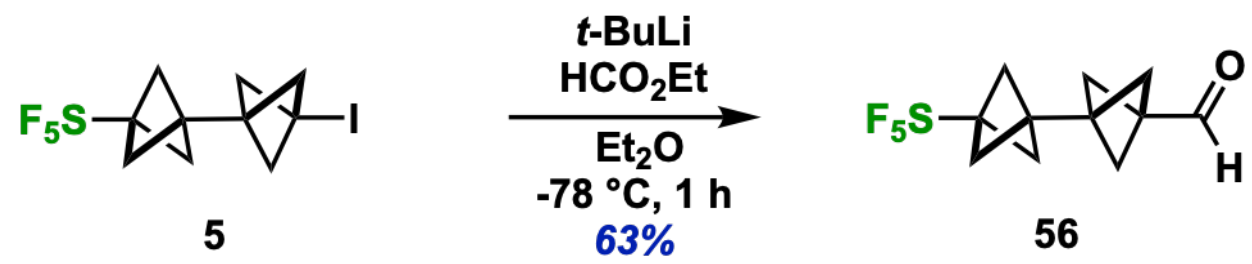

The synthesis of compound **56** was adapted from a known literature procedure.[21] To an oven-dried microwave vial equipped with a stir bar was added compound **5** (0.800 g, 2.07 mmol, 1.0 equiv.). The vial was sealed with a septum, evacuated, and backfilled with Ar three times. Anhydrous Et_2O (10 mL) was then added *via* syringe, and the reaction mixture was cooled to -78 °C. To the cooled reaction mixture, 1.7 M solution of *t*-BuLi in pentane (2.68 mL, 4.56 mmol, 2.2 equiv.) was added dropwise *via* syringe. After addition was complete, the reaction was stirred at -78 °C for 30 min. The solution containing lithiated staffane was transferred dropwise *via* cannula into a second 20 mL microwave vial at -78 °C containing ethyl formate (0.502 mL, 6.22 mmol, 3.0 equiv.) dissolved in anhydrous Et_2O (5.0 mL). The resulting mixture was left to stir for 30 min, then the cooling bath was removed to let the reaction mixture warm gradually to room temperature. Upon completion, the reaction mixture was then diluted with 1 M HCl (5 mL). The aqueous layer was extracted with EtOAc (3 x 10 mL). The combined organic layers were washed with brine (3 x 10 mL), dried over anhydrous Na_2SO_4, filtered through Celite, and concentrated. The crude residue was purified *via* column chromatography on silica gel eluting with 3:2 hexanes:CH_2Cl_2 to afford a white solid (0.376 g, 63%). *(Note: Anhydrous ethyl formate that is either freshly distilled or dried over 4 Å molecular sieves for 24 h will yield the best results.)*

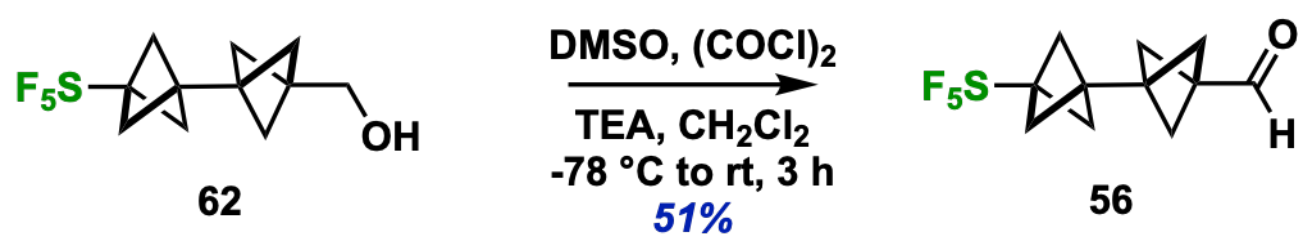

The synthesis of compound **56** was adapted from a known literature procedure.[15] An oven-dried microwave vial was sealed with a septum, evacuated, and backfilled with Argon three times. Anhydrous CH_2Cl_2 (1.0 mL) was added to the vial *via* syringe followed by oxalyl chloride (2 M in CH_2Cl_2; 0.011 mL, 0.127 mmol, 1.2 equiv.). The reaction mixture was cooled down to -78º C before adding a solution of DMSO (0.018 mL, 0.255, 2.4 equiv.) in anhydrous CH_2Cl_2 (1.0 mL). The reaction mixture was stirred for 5 min followed by addition of compound **62** (30.7 mg, 0.106 mmol, 1.0 equiv.) dissolved in anhydrous CH_2Cl_2 (1.0 mL). The resultant mixture was stirred for 5 min before adding TEA (0.073 mL, 0.532, 5.0 equiv.) *via* syringe. Subsequently, the reaction mixture was stirred at room temperature and monitored for completion *via* TLC. Upon completion, the reaction mixture was diluted with Et_2O (2 mL) and H_2O (2 mL). The aqueous layer was extracted with Et_2O (3 x 2 mL). The combined organic layers were washed with brine (3 x 3 mL), dried over anhydrous Na_2SO_4, filtered through Celite, and concentrated. The crude material was purified *via* preparative TLC eluting with 3:2 hexanes:CH_2Cl_2 to afford a white solid (15.7 mg, 51%).

3'-(pentafluoro-λ^6-sulfanyl)-[1,1'-bi(bicyclo[1.1.1]pentane)]-3-carbaldehyde (**56**):

R_f: 0.24 (3:2 hexanes:CH_2Cl_2), vis: *p*-anisaldehyde.

m.p.: 98.2–101.7 ºC.

^{1}H (300 MHz, $CDCl_3$): δ = 9.56 (s, 1H), 2.20 (s, 6H), 1.94 (s, 6H).

^{19}F (282 MHz, $CDCl_3$): δ = 80.25-78.18 (p, 1F, $^2J_{F\text{-}F}$ = 145.6 Hz), 48.45-47.93 (d, 4F, $^2J_{F\text{-}F}$ = 145.6 Hz).

^{13}C{^{1}H} (151 MHz, $CDCl_3$): δ = 198.5, 66.5-65.8 (m), 54.3-54.1 (m), 49.7, 43.3, 38.3, 34.9.

ATR-IR (ν_{max}): 2990, 2920, 1710, 1213, 814, 780 cm^{-1}.

HRMS (*m/z*): (ESI) calc'd for $C_{11}H_{14}F_5OS^+$ $[M\text{-}H]^+$: calculated 289.0680, found 289.0674.

Compound 57

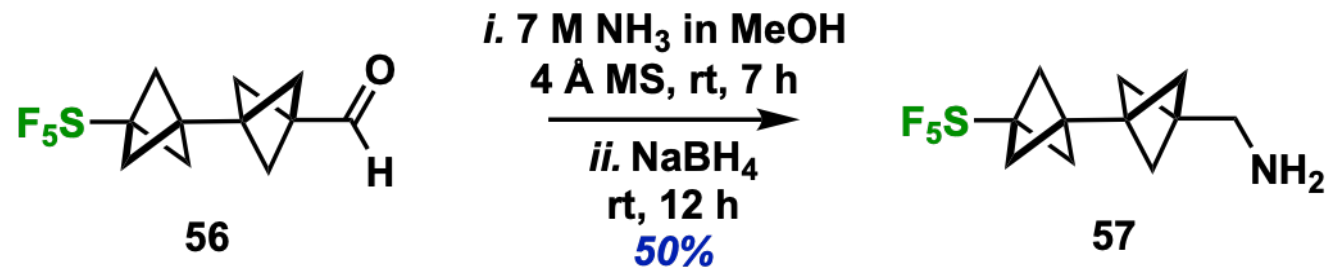

The synthesis of compound **57** was adapted from a known literature procedure.[24] To an oven-dried microwave vial equipped with a stir bar was added activated 4 Å powdered molecular sieves (0.05 g), followed by compound **56** (0.100 g, 0.347 mmol, 1.0 equiv.). The vial was sealed with a septum, evacuated and backfilled with Ar three times. Anhydrous 7 M solution of NH_3 in MeOH (1.73 mL) was then added *via* syringe. The reaction mixture was stirred at room temperature for 7 h or until all starting material was consumed by TLC. Once all starting material was consumed, sodium borohydride (0.026 g, 0.694 mmol, 2.0 equiv.) was added, and the reaction mixture was stirred at room temperature for 12 h. Upon completion, the reaction was diluted with sat. aq. NH_4Cl (3 mL). The aqueous layer was extracted with EtOAc (3 x 3 mL). The combined organic layers were washed with brine (3 x 4 mL), dried over anhydrous Na_2SO_4, filtered through Celite, and concentrated. The crude residue was purified *via* column chromatography on silica gel eluting with 19:1 CH_2Cl_2:7 M NH_3 in MeOH to afford a white solid (50.2 mg, 50%).

(3'-(pentafluoro-λ^6-sulfanyl)-[1,1'-bi(bicyclo[1.1.1]pentan)]-3-yl)methanamine (**57**):
R_f: 0.37 (19:1 CH_2Cl_2:7 M NH_3 in MeOH), vis: *p*-anisaldehyde.
m.p.: 63.5–65.2 ºC.
^{1}H (300 MHz, $CDCl_3$): δ = 2.70 (s, 2H), 2.16 (s, 6H), 1.53 (s, 6H), 1.13 (bs, 2H).
^{19}F (282 MHz, $CDCl_3$): δ = 80.68-78.62 (p, 1F, $^2J_{F\text{-}F}$ = 145.6 Hz), 48.32-47.80 (d, 4F, $^2J_{F\text{-}F}$ = 145.6 Hz).
^{13}C{^{1}H} (151 MHz, $CDCl_3$): δ = 66.6-66.4 (m), 54.3-54.2 (m), 47.9, 43.6, 40.5, 37.2, 35.2.
ATR-IR (ν_{max}): 3372, 3301, 2967, 2909, 2873, 1476, 1453, 1210, 900, 881, 808, 779 cm^{-1}.
HRMS (*m/z*): (ESI) calc'd for $C_{11}H_{17}F_5NS^+$ $[M\text{-}H]^+$: calculated 290.0996, found 290.0986.

Compound 58

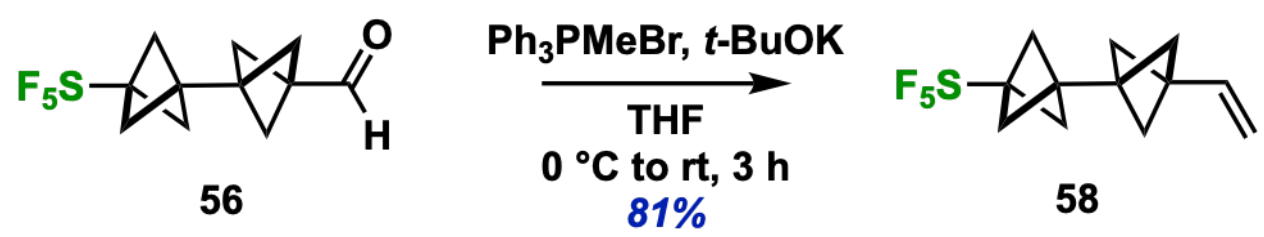

The synthesis of compound **58** was adapted from a known literature procedure.[3] To an oven-dried microwave vial equipped with a stir bar was added methyl triphenylphosphonium bromide (0.155 g, 0.434 mmol, 2.5 equiv.). The vial was sealed with a septum, evacuated, and backfilled with Ar three times. Subsequently, anhydrous THF (0.3 mL) was added to the vial, and the reaction mixture was cooled to 0 °C in an ice-water bath. Then, *t*-BuOK (0.049 g, 0.434 mmol, 2.5 equiv.) was added and the reaction mixture was left to stir at 0 °C for 1 h. A stock solution containing compound **56** (0.050 g, 0.173 mmol, 1.0 equiv.) in 0.3 mL THF was then added dropwise. *(Note: ylide formation can be observed visually when the reaction mixture turns a canary yellow.)* The cooling bath was then removed, and the reaction mixture was stirred at room temperature for 2 h. Upon completion, the reaction mixture was concentrated. The crude residue was diluted with a minimal amount of CH_2Cl_2 and loaded on to a silica plug; elution with 100% *n*-pentane and careful concentration afforded a volatile white solid (40.1 mg, 81%).

pentafluoro(3'-vinyl-[1,1'-bi(bicyclo[1.1.1]pentan)]-3-yl)-λ^6-sulfane (**58**):
R_f: 0.60 (hexanes), vis: *p*-anisaldehyde.
m.p.: 57.5–59.8 ºC.
^{1}H (300 MHz, $CDCl_3$): δ = 5.94-5.85 (m, 1H), 5.03 (s, 1H), 5.00-4.97 (m, 1H), 2.16 (s, 6H), 1.68 (s, 6H).
^{19}F (282 MHz, $CDCl_3$): δ = 80.66-78.60 (p, 1F, $^2J_{F\text{-}F}$ = 145.6 Hz), 48.30-47.79 (d, 4F, $^2J_{F\text{-}F}$ = 145.6 Hz).
^{13}C{^{1}H} (151 MHz, $CDCl_3$): δ = 137.3, 115.2, 66.9-66.1 (m), 54.4-54.3 (m), 50.8, 40.6, 37.0, 35.1.
ATR-IR (ν_{max}): 2966, 2927, 2907, 2866, 1635, 1451, 1422, 1275, 1209, 987, 928, 901, 881, 794, 646 cm^{-1}.
HRMS (*m/z*): (ESI) calc'd for $C_{12}H_{16}F_5S^+$ [M-H]$^+$: calculated 287.0887, found 287.0881.

Compound 59

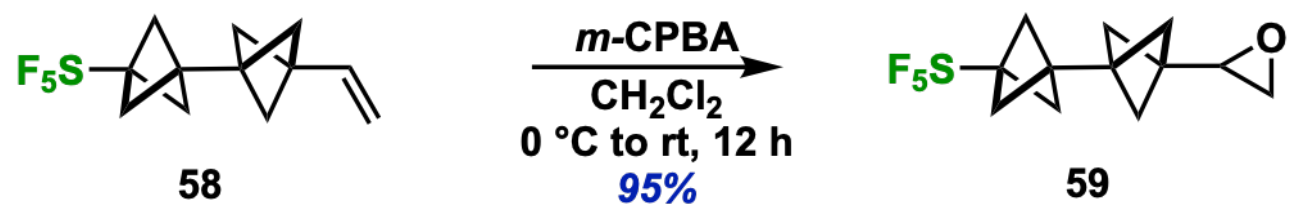

The synthesis of compound **59** was adapted from a known literature procedure.[25] To an oven-dried microwave vial equipped with a stir bar was added compound **58** (0.120 g, 0.419 mmol, 1.0 equiv.). The vial was sealed with a septum, evacuated and backfilled with Ar three times. Then, anhydrous CH_2Cl_2 (2.0 mL) was added, and the reaction mixture was cooled to 0 °C using an ice-water bath. Then, *m*-CPBA (0.108 g, 0.629 mmol, 1.5 equiv.) was added, the cooling bath was removed, and the reaction mixture was stirred at room temperature for 12 h. Upon completion, the reaction mixture was cooled to 0 °C, diluted with sat. aq. $Na_2S_2O_4$ (3.0 mL), and transferred to a separatory funnel. The aqueous layer was extracted with CH_2Cl_2 (3 x 3 mL). The combined organic layers were washed with brine (3 x 3 mL), dried over anhydrous Na_2SO_4, filtered through Celite, and concentrated. The crude residue was purified *via* column chromatography on silica gel eluting with 9:1 hexanes:EtOAc to afford a white solid (0.120 g, 95%).

2-(3'-(pentafluoro-λ^6-sulfanyl)-[1,1'-bi(bicyclo[1.1.1]pentan)]-3-yl)oxirane (**59**):

R_f: 0.15 (19:1 hexanes:EtOAc), vis: *p*-anisaldehyde.

m.p.: 112.9–114.2 ºC.

^{1}H (300 MHz, CDCl$_3$): δ = 2.94-2.91 (m, 1H), 2.73-2.70 (m, 1H), 2.47-2.45 (m, 1H), 2.16 (s, 6H), 1.61 (s, 6H).

^{19}F (282 MHz, CDCl$_3$): δ = 80.54-78.48 (p, 1F, $^2J_{F\text{-}F}$ = 145.6 Hz), 48.37-47.86 (d, 4F, $^2J_{F\text{-}F}$ = 145.6 Hz).

^{13}C{^{1}H} (151 MHz, CDCl$_3$): δ = 66.7-66.0 (m), 54.3-54.2 (m), 50.3, 48.3, 45.5, 38.1, 37.3, 35.0.

ATR-IR (ν_{max}): 2981, 2916, 2881, 1479, 1451, 1419, 1289, 1246, 1210, 899, 813, 779, 642 cm^{-1}.

HRMS (*m/z*): (ESI) calc'd for $C_{12}H_{16}F_5OS^+$ [M-H]$^+$: calculated 303.0837, found 303.0825.

Compound 60

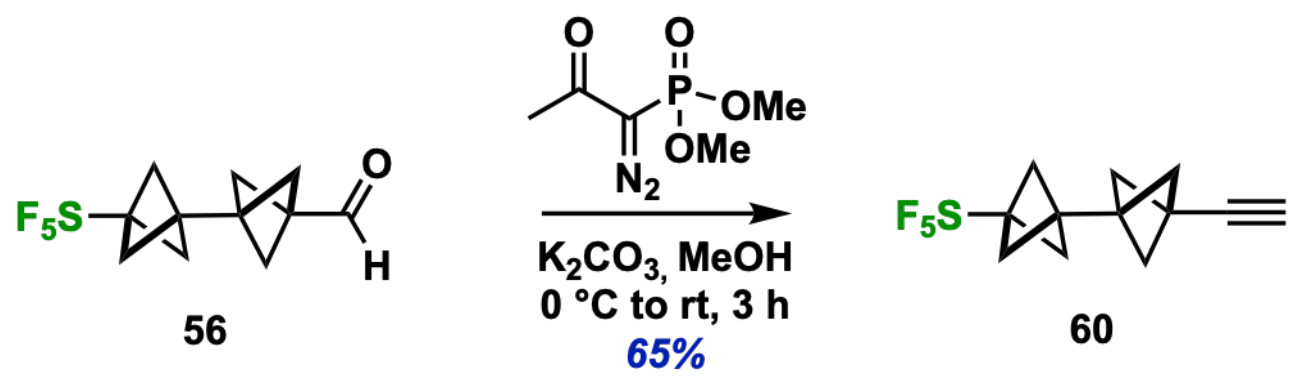

The synthesis of compound **60** was adapted from a known literature procedure.[15] To an oven-dried microwave vial equipped with a stir bar was added compound **56** (50.0 mg, 0.173 mmol, 1.0 equiv.). The vial was sealed with a septum, evacuated, and backfilled with Ar three times. Then, anhydrous MeOH (1.0 mL) was added and was cooled to 0 °C using an ice-water bath. Then, K_2CO_3 (25.0 mg, 0.182 mmol, 1.05 equiv.) and dimethyl 1-diazo-2-oxopropylphosphonate (0.087 mL, 0.363 mmol, 2.1 equiv.) were added. The resulting reaction mixture was left to stir at room temperature for 3 h. Upon completion, the reaction mixture was concentrated. The crude residue was diluted with a minimal amount of CH_2Cl_2 and loaded on to a silica plug; elution with 100% *n*-pentane and careful concentration afforded a volatile white solid (32.1 mg, 65%).

(3'-ethynyl-[1,1'-bi(bicyclo[1.1.1]pentan)]-3-yl)pentafluoro-λ^6-sulfane (**60**):
R_f: 0.20 (hexanes), vis: *p*-anisaldehyde.
m.p.: 111.4–113.9 ºC.
^{1}H (300 MHz, $CDCl_3$): δ = 2.16 (s, 6H), 2.09 (s, 1H), 1.95 (s, 6H).
^{19}F (282 MHz, $CDCl_3$): δ = 80.40-78.35 (p, 1F, $^2J_{F-F}$ = 145.6 Hz), 48.41-47.90 (d, 4F, $^2J_{F-F}$ = 145.6 Hz).
^{13}C{^{1}H} (151 MHz, $CDCl_3$): δ = 82.7, 68.3, 66.5-65.8 (m), 54.4-54.3 (m), 53.9, 39.6, 34.7, 27.6.
ATR-IR (ν_{max}): 3286, 2984, 2917, 2880, 1212, 904, 816, 794, 656, 646, 630 cm^{-1}.
HRMS (*m/z*): (ESI) calc'd for $C_{12}H_{14}F_5S^+$ $[M-H]^+$: calculated 285.0731, found 285.0725.

Compound 61

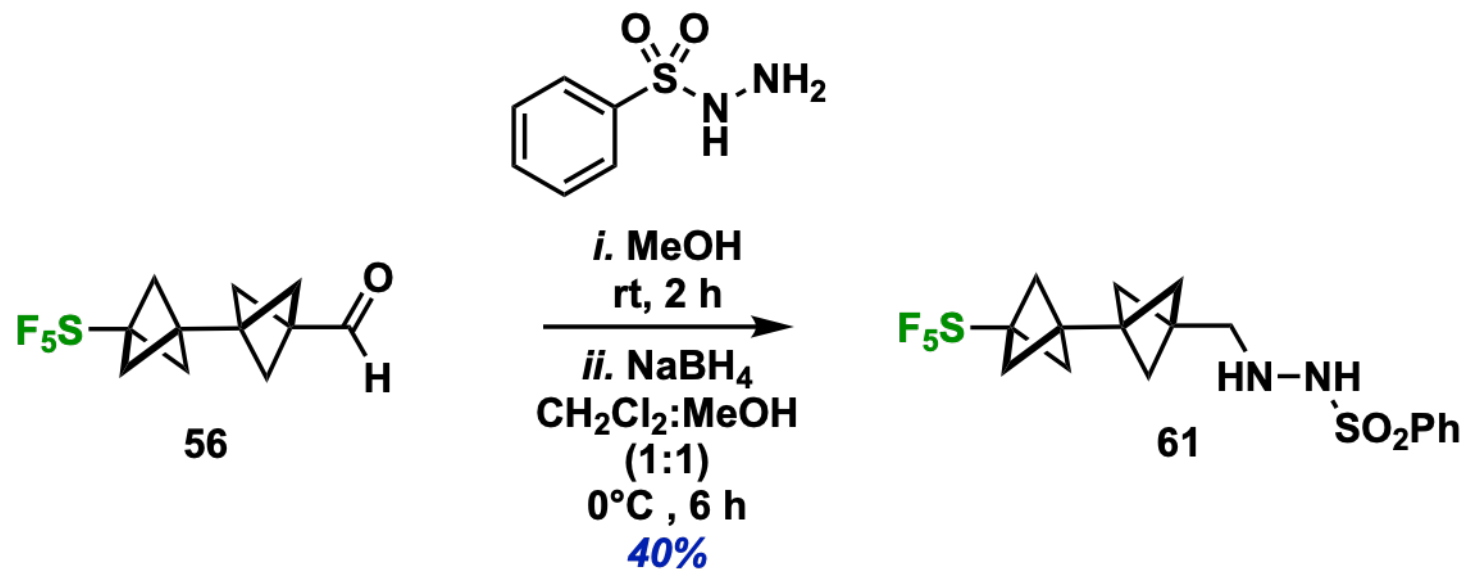

The synthesis of compound **61** was adapted from a known literature procedure.[26] To an oven-dried microwave vial equipped with a stir bar was added compound **56** (0.100 g, 0.347 mmol, 1.0 equiv.) and MeOH (0.69 mL). *(Note: It is important to completely dissolve the aldehyde, as the product may precipitate out.)* Next, the sulfonyl hydrazide (60.0 mg, 0.347 mmol, 1.0 equiv.) was added in a single portion. The reaction mixture was stirred for 2 h at room temperature or until all starting material is consumed by TLC. Upon reaction completion, the reaction mixture was concentrated. Then, *n*-pentane (5.0 mL) was added, and the undissolved solid was sonicated for 30 min to turn the hydrazones into white powders. *(Note: in case no solid is formed, the n-pentane is evaporated, and this step is repeated.)* The slurry was filtered and washed with *n*-pentane (3 x 5 mL), then dried under vacuum to afford the hydrazone in a crude mixture, which can be used directly in the next step without further purification.

To an oven-dried microwave vial was added the crude mixture containing sulfonyl hydrazone (0.347 mmol), CH_2Cl_2 (1.5 mL), and MeOH (1.5 mL). The mixture was then cooled to 0 °C in an ice water bath. Sodium borohydride (20.0 mg, 0.521 mmol, 1.5 equiv.) was added, and the reaction mixture was stirred at 0 °C for 1 h. If, at this point, the reaction did not go to completion as monitored by TLC, more sodium borohydride was added (20.0 mg, 0.521 mmol, 1.5 equiv.) every hour until full conversion was observed (in this case, 6 h and 0.141 g of $NaBH_4$). *(Note: 1. It is important to keep the reaction mixture at 0 °C to suppress side-product formation and decomposition of the product. 2. TLC is a reliable way to track conversion.)* Upon reaction completion, the reaction was quenched with sat. aq. NH_4Cl (3 mL), and the mixture was transferred to a separatory funnel. The aqueous layer was extracted with CH_2Cl_2 (3 x 3 mL). The combined organic layers were washed with brine (3 x 4 mL), dried over anhydrous Na_2SO_4, filtered through Celite, and concentrated. The crude residue was redissolved in a minimal amount of Et_2O, sonicated for 10 min, filtered, and washed with Et_2O to afford a white solid (64.0 mg, 40%).

N'-((3'-(pentafluoro-λ^6-sulfanyl)-[1,1'-bi(bicyclo[1.1.1]pentan)]-3-yl)methyl)benzenesulfonohydrazide (**61**):
R_f: 0.61 (3:2 hexanes:EtOAc), vis: *p*-anisaldehyde.
m.p.: 126.0–129.0 ºC.
^{1}H (300 MHz, DMSO-*d6*): δ = 8.53 (bs, 1H), 7.81-7.79 (m, 2H), 7.67-7.55 (m, 3H), 4.50 (bs, 1H), 3.35 (bs, 2H), 2.15 (s, 6H), 1.45 (s, 6H).
^{19}F (282 MHz, DMSO-*d6*): δ = 83.49-81.41 (p, 1F, $^2J_{F-F}$ = 145.6 Hz), 49.38-48.86 (d, 4F, $^2J_{F-F}$ = 145.6 Hz).
^{13}C{^{1}H} (151 MHz, DMSO-*d6*): δ = 139.3, 132.4, 128.8, 127.5, 67.2-66.6 (m), 53.9, 51.8, 48.8, 37.1, 37.0, 34.4.
ATR-IR (ν_{max}): 3305, 3245,2962, 2869, 1319, 1211, 1157, 814, 787, 751 cm^{-1}.
HRMS (*m/z*): (ESI) calc'd for $C_{17}H_{22}F_5N_2O_2S_2^+$ [M-H]$^+$: calculated 445.1037, found 445.1032.

Compound 62

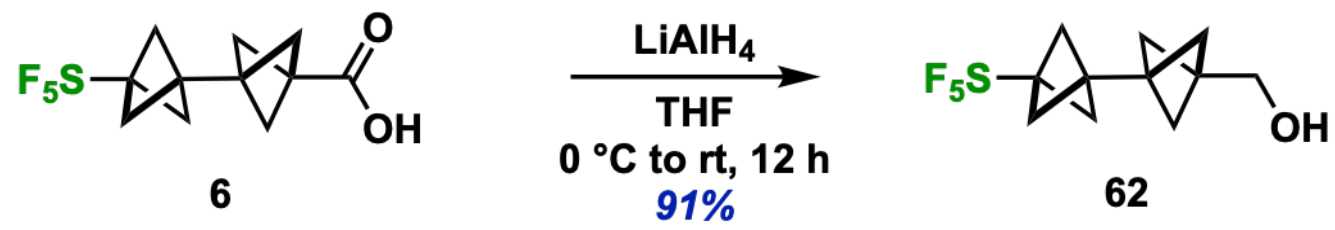

To an oven-dried microwave vial equipped with a stir bar was added compound **6** (45.0 mg, 0.148 mmol, 1.00 equiv.). The vial was sealed with a septum, evacuated, and backfilled with Ar three times. Anhydrous THF (1.0 mL) was added, and the reaction vessel was cooled to 0 ºC in an ice water bath. Then, 1.0 M solution of $LiAlH_4$ solution in THF (0.360 mL, 0.360 mmol, 2.0 equiv.) was added dropwise, and the resulting mixture was stirred at room temperature for 12 h. Upon reaction completion, the reaction mixture was cooled down to 0 ºC, quenched with 1 M HCl (2 mL), diluted with EtOAc (4 mL), and transferred to a separatory funnel. The aqueous layer was extracted with EtOAc (3 x 1mL). The combined organic layers were washed with H_2O (3 x 1mL), brine (3 x 1 mL), dried over anhydrous Na_2SO_4, filtered through Celite, and concentrated to afford a white solid (39.1 mg, 91%), which required no further purification.

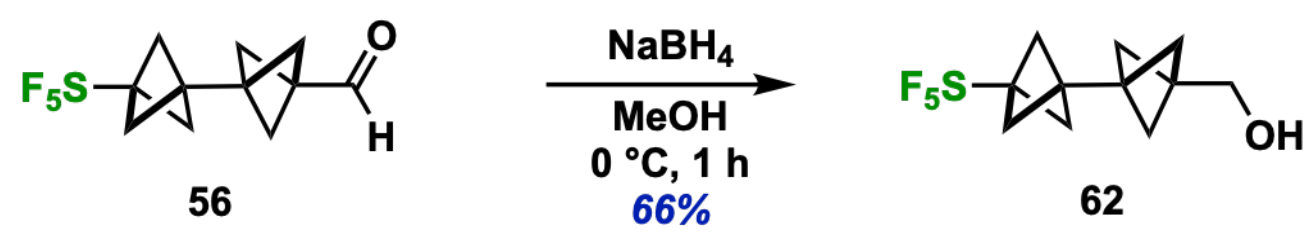

The synthesis of compound **62** was adapted from a known literature procedure.[27] To an oven-dried microwave vial equipped with a stir bar was added compound **56** (50.0 mg, 0.1734 mmol, 1.0 equiv.). The vial was sealed with a septum, evacuated, and backfilled with Ar three times. Anhydrous MeOH (0.45 mL) was added *via* syringe. The reaction mixture was cooled to 0 °C in an ice water bath and $NaBH_4$ (7.00 mg, 0.191 mmol, 1.1 equiv.) was added. The reaction mixture was then stirred for 1 h at 0 °C in an ice water bath. Upon reaction completion, the reaction mixture was quenched with 1 M HCl (1 mL) and transferred to a separatory funnel. The aqueous layer was extracted with EtOAc (3 x 5 mL). The combined organic layers were washed with brine (3 x 5 mL), dried over anhydrous Na_2SO_4, filtered through Celite, and concentrated. The crude residue was purified *via* preparatory TLC eluting with 100% CH_2Cl_2 to afford a white solid (33.0 mg, 66%).

(3'-(pentafluoro-λ^6-sulfanyl)-[1,1'-bi(bicyclo[1.1.1]pentan)]-3-yl)methanol (**62**):
Rf: 0.35 (CH_2Cl_2), vis: *p*-anisaldehyde.
m.p.: 111.0–113.2 ºC.
^{1}H (300 MHz, $CDCl_3$): δ = 3.57 (s, 2H), 2.16 (s, 6H), 1.59 (s, 6H), 1.47 (s, 1H).
^{19}F (282 MHz, $CDCl_3$): δ = 80.64-78.59 (p, 1F, J = 146.3 Hz), 48.34-47.82 (d, 4F, J = 145.0 Hz).
^{13}C{^{1}H} (75 MHz, $CDCl_3$): δ = 77.4, 66.8-66.1 (p, J = 10.9 Hz), 63.2, 54.3-54.3 (t, J = 2.1 Hz), 48.1, 39.5, 37.7, 35.2, 29.8.
ATR-IR (ν_{max}): 3215, 2974, 2912, 2874, 1211, 1043, 1032, 1012, 936, 902, 827, 808, 795, 785 cm^{-1}.
HRMS (*m/z*): (ESI) calc'd for $C_{11}H_{16}F_5OS^+$ $[M-H]^+$: calculated 291.0837, found 291.0837.

Compound 63

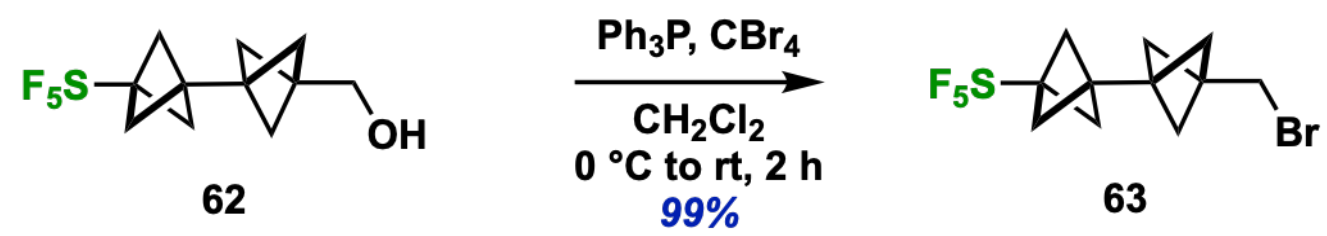

The synthesis of compound **63** was adapted from a known literature procedure.[28] To an oven-dried microwave vial equipped with a stir bar was added compound **62** (42.0 mg, 0.145 mmol, 1.0 equiv.). The vial was sealed with a septum, evacuated, and backfilled with Ar three times. Anhydrous CH_2Cl_2 (0.73 mL) was added *via* syringe and the resulting mixture was cooled to 0 °C using an ice-water bath, and triphenylphosphine (65.0 mg, 0.247 mmol, 1.7 equiv.) and tetrabromomethane (82.0 mg, 0.247 mmol, 1.7 equiv.) were added. The reaction mixture was then warmed to room temperature and stirred for 2 h. Upon reaction completion, the reaction mixture was concentrated. The crude residue was diluted with a minimal amount of CH_2Cl_2 and loaded on to a silica plug; elution with 100% hexanes and concentrated to afford a white solid (50.5 mg, 99%).

(3'-(bromomethyl)-[1,1'-bi(bicyclo[1.1.1]pentan)]-3-yl)pentafluoro-λ^6-sulfane (**63**):
R_f: 0.29 (hexanes), vis: *p*-anisaldehyde.
m.p.: 90.5–92.5 ºC.
^{1}H (300 MHz, $CDCl_3$): δ = 3.42 (s, 2H), 2.17 (s, 6H), 1.64 (s, 6H).
^{19}F (282 MHz, $CDCl_3$): δ = 80.56-78.50 (p, 1F, $^2J_{F\text{-}F}$ = 145.6 Hz), 48.40-47.89 (d, 4F, $^2J_{F\text{-}F}$ = 145.6 Hz).
^{13}C{^{1}H} (151 MHz, $CDCl_3$): δ = 66.7-66.0 (m), 54.4-54.3 (m), 49.3, 38.9, 36.8, 34.8, 33.9.
ATR-IR (ν_{max}): 2971, 2918, 2876, 2853, 1227, 1210, 899, 829, 812, 781 cm^{-1}.
HRMS (*m/z*): (ESI) calc'd for $C_{11}H_{15}BrF_5S^+$ [M-H]$^+$: calculated 352.9993, found 352.9999.

Compound 64

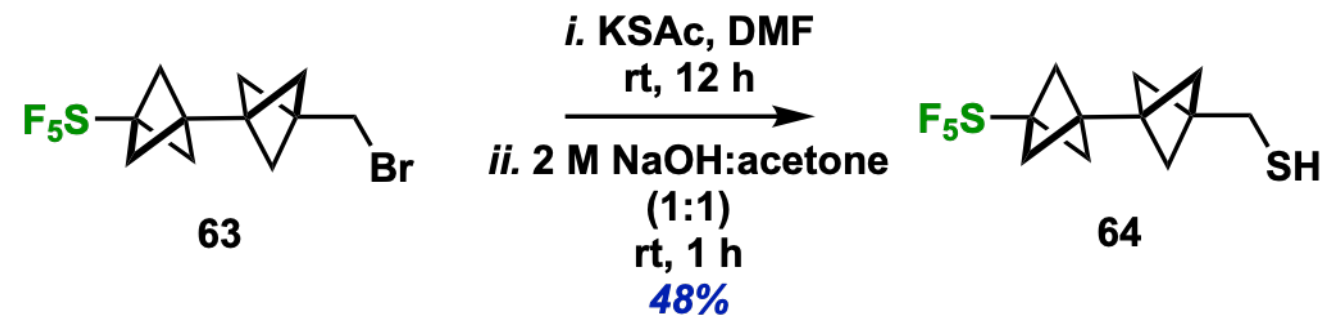

The synthesis of compound **64** was adapted from a known literature procedure.[29] To an oven-dried microwave vial equipped with a stir bar was added compound **63** (53.0 mg, 0.150 mmol, 1.0 equiv.) and potassium thioacetate (17.0 mg, 0.150 mmol, 1.0 equiv.). The vial was sealed with a septum, evacuated, and backfilled with Ar three times. Anhydrous DMF (0.75 mL) was then added, and the reaction mixture was stirred at room temperature for 12 h. Upon reaction completion, the reaction mixture was diluted with sat. aq. NH_4Cl (3 mL) and transferred to a separatory funnel. The aqueous layer was extracted with EtOAc (3 x 3 mL). The combined organic layers were washed with H_2O (3 x 3 mL) and brine (3 x 3 mL), dried over anhydrous Na_2SO_4, filtered through Celite, and concentrated. The crude residue was redissolved in acetone (1.0 mL), and then 2 M NaOH (1.0 mL) was added, and the reaction mixture was stirred at room temperature for 1 h. The reaction was quenched with 1 M HCl (3 mL), and the reaction mixture was transferred to a separatory funnel. The aqueous layer was extracted with EtOAc (3 x 3 mL). The combined organic layers were washed sequentially with H_2O (3 x 3 mL) and brine (3 x 3 mL), dried over anhydrous Na_2SO_4, filtered through Celite, and concentrated. The crude residue was purified *via* preparatory TLC eluting with 4:1 hexanes:CH_2Cl_2 to afford a white solid (22.2 mg, 48%).

(3'-(pentafluoro-λ^6-sulfanyl)-[1,1'-bi(bicyclo[1.1.1]pentan)]-3-yl)methanethiol (**64**):

R_f: 0.23 (9:1 hexanes:CH_2Cl_2), vis: *p*-anisaldehyde.

m.p.: 66.9–69.3 ºC.

^{1}H (300 MHz, $CDCl_3$): δ = 2.63-2.61 (d, 2H, $^2J_{H-H}$= 7.90 Hz), 2.16 (s, 6H), 1.58 (s, 6H), 1.22-1.17 (t, 1H, $^2J_{H-H}$ = 7.90 Hz).

^{19}F (282 MHz, $CDCl_3$): δ = 80.64-78.58 (p, 1F, $^2J_{F-F}$ = 145.6 Hz), 48.36-47.85 (d, 4F, $^2J_{F-F}$ = 145.6 Hz).

^{13}C{^{1}H} (151 MHz, $CDCl_3$): δ = 66.6-66.3 (m), 54.3-54.3 (m), 49.9, 48.7, 39.9, 35.0, 26.7.

ATR-IR (ν_{max}): 2977, 2927, 2911, 2874, 2565, 1253, 1210, 900, 814, 782 cm^{-1}.

HRMS (*m/z*): (ESI) calc'd for $C_{11}H_{14}F_5S_2^-$ [M-H]$^-$: calculated 305.0463, found 305.0453.

Compound 65

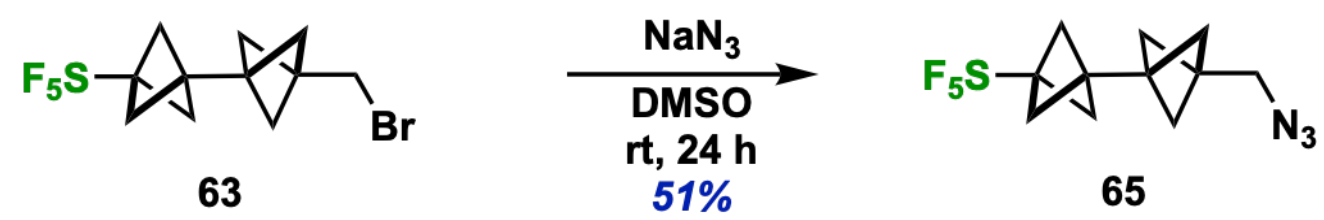

The synthesis of compound **65** was adapted from a known literature procedure.[30] To an oven-dried microwave vial equipped with a stir bar was added compound **63** (41.0 mg, 0.116 mmol, 1.0 equiv.) and sodium azide (15.0 mg, 0.232 mmol, 2.0 equiv.). The vial was sealed with a septum, evacuated, and backfilled with Ar three times. Anhydrous DMSO (1.0 mL) was added *via* syringe and the reaction mixture was then stirred at room temperature for 24 h. Upon reaction completion, the reaction mixture was diluted with H_2O (3.0 mL) and transferred to a separatory funnel. The aqueous layer was extracted with CH_2Cl_2 (3 x 3 mL). The combined organic layers were washed with brine (3 x 4 mL), dried over anhydrous Na_2SO_4, filtered through Celite, and concentrated. The crude residue was purified *via* preparatory TLC eluting with 4:1 hexanes:CH_2Cl_2 to afford a white solid (18.7 mg, 51%).

(3'-(azidomethyl)-[1,1'-bi(bicyclo[1.1.1]pentan)]-3-yl)pentafluoro-λ^6-sulfane (**65**):

R_f: 0.34 (4:1 hexanes: CH_2Cl_2), vis: *p*-anisaldehyde.

m.p.: 53.1–54.3 ºC.

^{1}H (300 MHz, $CDCl_3$): δ = 3.25 (s, 2H), 2.17 (s, 6H), 1.69 (s, 6H).

^{19}F (282 MHz, $CDCl_3$): δ = 80.51-78.45 (p, 1F, $^2J_{F\text{-}F}$ = 145.6 Hz), 48.40-47.89 (d, 4F, $^2J_{F\text{-}F}$ = 145.6 Hz).

^{13}C{^{1}H} (151 MHz, $CDCl_3$): δ = 66.5-66.2 (m), 54.3-54.3 (m), 52.1, 49.5, 38.2, 38.2, 35.9.

ATR-IR (ν_{max}): 2981,2912, 2875, 2087, 1275, 1210, 814, 783 cm^{-1}.

HRMS (*m/z*): (ESI) calc'd for $C_{11}H_{17}F_5NS^+$ [M-N$_2$]$^+$: calculated 290.0996, found 290.0987.

Compound 66

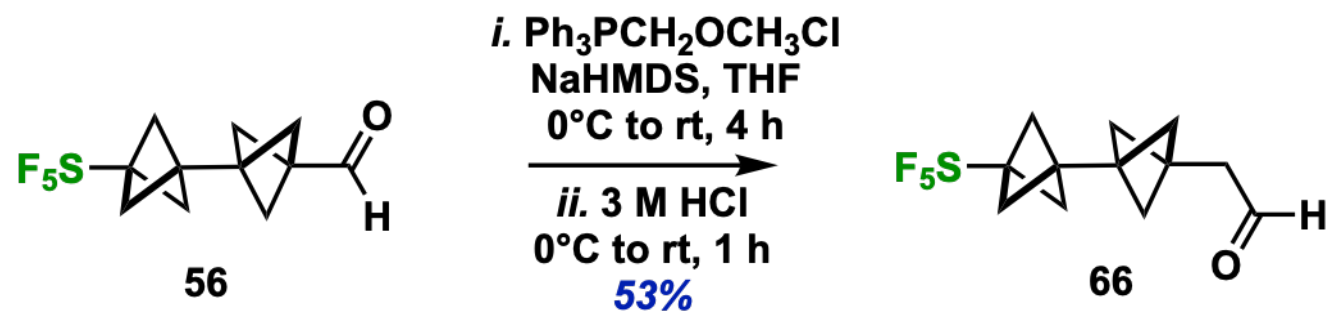

The synthesis of compound **66** was adapted from a known literature procedure.[31] To an oven-dried microwave vial equipped with a stir bar was added (methoxymethyl)triphenylphosphonium chloride (0.178 g, 0.520 mmol, 1.5 equiv. The vial was sealed with a septum, evacuated, and backfilled with Ar three times, and anhydrous THF (1.0 mL) was then added, and the reaction mixture was cooled to 0 °C in a water-ice bath. Subsequently, 2 M NaHMDS in THF (0.260 mL, 0.520 mmol, 1.5 equiv.) was added to the reaction mixture and the resulting solution was stirred at 0 °C for 1 h. A stock solution containing compound **56** (0.100 g, 0.347 mmol, 1.0 equiv.) in 1.0 mL THF was then added dropwise. *(Note: ylide formation can be observed visually when the reaction mixture turns a blood red.)* The cooling bath was then removed, and the reaction mixture was stirred at room temperature for 3 h. Upon reaction completion, the reaction mixture was concentrated. The crude residue was redissolved in THF (2.0 mL) and cooled to 0 °C in an ice water bath. Then, 3 M HCl (1.0 mL) was added, and the reaction mixture was stirred at room temperature for another 1 h. Subsequently, the reaction was quenched with sat. aq. $NaHCO_3$ (10 mL) and transferred into a separatory funnel and washed with EtOAc (3 x 3 mL). The combined organic layers were washed with brine (3 x 4 mL), dried over anhydrous Na_2SO_4, filtered through Celite, and concentrated. The crude residue was purified *via* preparatory TLC eluding with 1:1 hexanes:CH_2Cl_2 to afford a white solid (56.0 mg, 53%).

2-(3'-(pentafluoro-λ^6-sulfanyl)-[1,1'-bi(bicyclo[1.1.1]pentan)]-3-yl)acetaldehyde (**66**):

R_f: 0.32 (3:2 hexanes:CH_2Cl_2), vis: *p*-anisaldehyde.

m.p.: 72.5–74.5 ºC.

^{1}H (300 MHz, $CDCl_3$): δ = 9.71-9.69 (t, 1H,$^3J_{H\text{-}H}$ = 2.28 Hz), 2.57-2.57 (d, 2H, $^3J_{H\text{-}H}$ = 2.28 Hz), 2.15 (s, 6H), 1.71 (s, 6H).

^{19}F (282 MHz, $CDCl_3$): δ = 80.33-78.79 (p, 1F, $^2J_{F\text{-}F}$ = 145.6 Hz), 48.32-47.93 (d, 4F, $^2J_{F\text{-}F}$ = 145.6 Hz).

^{13}C{^{1}H} (151 MHz, $CDCl_3$): δ = 200.7, 66.7-66.1 (m), 54.4-54.3 (m), 50.8, 45.7, 39.3, 34.9, 34.3.

ATR-IR (ν_{max}): 2976, 2916, 2876, 2725, 1723, 1211, 899, 811, 782 cm^{-1}.

HRMS (*m/z*): (ESI) calc'd for $C_{12}H_{16}F_5OS^+$ [M-H]$^+$: calculated 303.0837, found 303.0833.

Compound 67

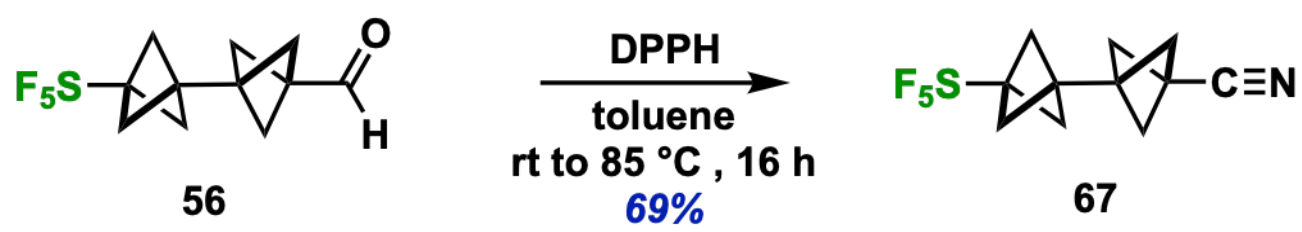

The synthesis of compound **67** was adapted from a known literature procedure.[32] To an oven-dried microwave vial equipped with a stir bar was added compound **56** (50.0 mg, 0.173 mmol, 1.0 equiv.) and *O*-(diphenylphosphinyl)hydroxylamine (48.0 mg, 0.208 mmol, 1.2 equiv.). The vial was sealed with a septum, evacuated, and backfilled with Ar three times. Anhydrous toluene (0.87 mL) was added *via* syringe and the reaction mixture was stirred at room temperature for 4 h. Subsequently, the reaction mixture was placed into an oil bath and gradually heated to 85 °C over the course of 45 min. The reaction mixture was then stirred at 85 °C for 12 h. Upon reaction completion, the reaction mixture was diluted with sat. aq. $NaHCO_3$ (3 mL) and transferred to a separatory funnel. The aqueous layer was extracted with EtOAc (3 x 3 mL). The combined organic layers were washed with brine (3 x 3 mL), dried over anhydrous Na_2SO_4, filtered through Celite, and concentrated. The crude residue was purified *via* column chromatography on silica gel eluting with 1:1 hexanes:CH_2Cl_2 to afford a white solid (34.0 mg, 69%).

3'-(pentafluoro-λ^6-sulfanyl)-[1,1'-bi(bicyclo[1.1.1]pentane)]-3-carbonitrile (**67**):
R_f: 0.28 (1:1 hexanes:CH_2Cl_2), vis: *p*-anisaldehyde.
m.p.: decomposes above 158 ºC.
^{1}H (300 MHz, $CDCl_3$): δ = 2.19 (s, 6H), 2.15 (s, 6H).
^{19}F (282 MHz, $CDCl_3$): δ = 79.99-77.93 (p, 1F, $^2J_{F-F}$ = 145.6 Hz), 48.60-48.08 (d, 4F, $^2J_{F-F}$ = 145.6 Hz).
^{13}C{^{1}H} (151 MHz, $CDCl_3$): δ = 117.4, 66.0-65.5 (m), 54.3-54.2 (m), 53.6, 41.7, 34.4, 23.4.
ATR-IR (ν_{max}): 3015, 2928, 2851, 2229,1645, 1454, 1217, 905, 839, 791 cm^{-1}.
HRMS (*m/z*): (ESI) calc'd for $C_{11}H_{13}F_5NS^+$ $[M-H]^+$: calculated 286.0683, found 286.0673.

Compound S-28

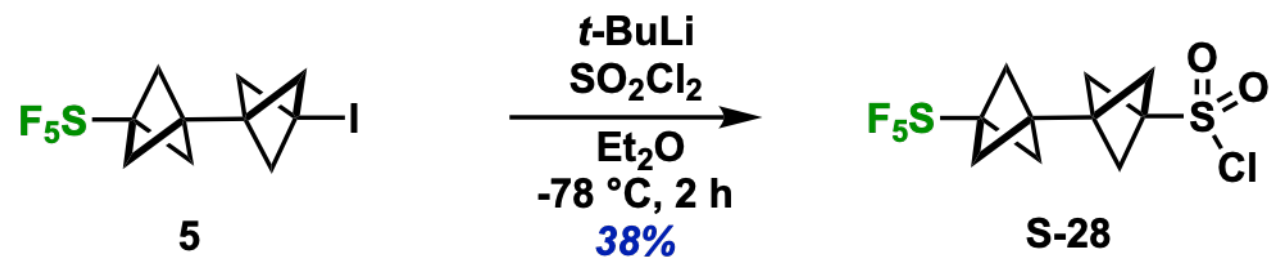

The synthesis of compound **S-28** was adapted from a known literature procedure.[33] To an oven-dried microwave vial equipped with a stir bar was added compound **5** (0.300 g, 0.777 mmol, 1.0 equiv.). The vial was sealed with a septum, evacuated, and backfilled with Ar three times. Anhydrous Et_2O (8.0 mL) was added *via* syringe. The resulting mixture was cooled to -78 °C, and 1.7 M solution of *t*-BuLi in pentane (1.00 mL, 1.71 mmol, 2.2 equiv.) was added dropwise *via* syringe. After addition, the reaction mixture was stirred at -78 °C for 1 h. Then, SO_2Cl_2 (0.125 mL, 1.55 mmol, 2.0 equiv.) was added dropwise at -78 °C and the reaction mixture was gradually warmed to room temperture and stirred for 1 h. The reaction mixture was quenched with 1 M HCl (3 mL), transferred to a separatory funnel, and extracted with EtOAc (3 x 3 mL). The combined organic layers were washed with H_2O (3 x 4 mL), brine (3 x 4 mL), dried over anhydrous Na_2SO_4, filtered through Celite, and concentrated. The crude residue was purified *via* column chromatography on silica gel eluting with 4:1 hexanes:CH_2Cl_2 to afford a white solid (0.107 g, 38%).

3'-(pentafluoro-λ^6-sulfanyl)-[1,1'-bi(bicyclo[1.1.1]pentane)]-3-sulfonyl chloride (**S-28**):
R_f: 0.16 (4:1 hexanes:CH_2Cl_2), vis: *p*-anisaldehyde.
m.p.: 155.0–157.9 ºC.
^{1}H (300 MHz, $CDCl_3$): δ = 2.31 (s, 6H), 2.26 (s, 6H).
^{19}F (282 MHz, $CDCl_3$): δ = 79.63-77.56 (p, 1F, $^2J_{F\text{-}F}$ = 145.6 Hz), 48.84-48.22 (d, 4F, $^2J_{F\text{-}F}$ = 145.6 Hz).
^{13}C{^{1}H} (151 MHz, $CDCl_3$): δ = 66.1-65.3 (m), 58.6, 54.4-54.3, 52.4, 35.9, 33.7.
ATR-IR (ν_{max}): 3017, 2926, 2851, 1368, 1214, 1174, 1133, 818, 792 cm^{-1}.
HRMS (*m/z*): (ESI) calc'd for $C_{10}H_{13}ClF_5O_2S_2$ $^+$ $[M-H]^+$: calculated 358.9960, found 358.9967.

Compound 68

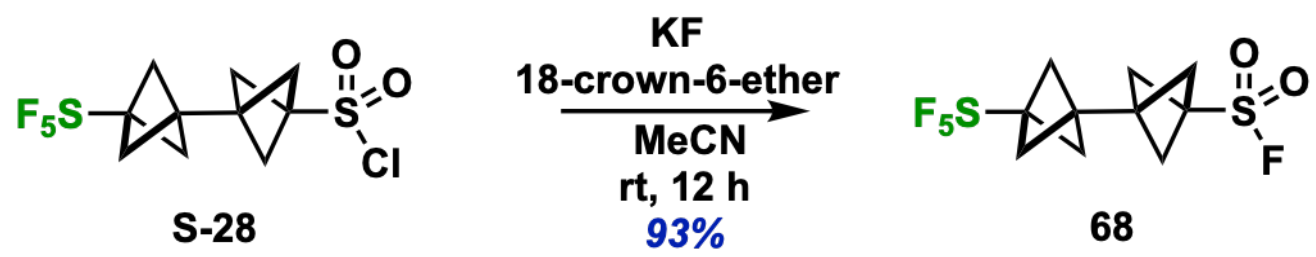

The synthesis of compound **68** was adapted from a known literature procedure.[34] To an oven-dried microwave vial equipped with a stir bar were added compound **S-28** (25.0 mg, 0.070 mmol, 1.0 equiv.), potassium fluoride (22.0 mg, 0.378 mmol, 5.4 equiv.), and 18-crown-6-ether (53.0 mg, 0.200 mmol, 2.88 equiv.). The vial was sealed with a septum, evacuated, and backfilled with Ar three times. Anhydrous MeCN (0.5 mL) was added *via* syringe and the resulting mixture was stirred at room temperature for 12 h. Upon reaction completion, the reaction was diluted with H_2O (3 mL) and washed with EtOAc (3 x 3 mL). The combined organic layers were washed with brine (3 x 4 mL), dried over anhydrous Na_2SO_4, filtered through Celite, and concentrated to afford a white solid (22.0 mg, 93%).

3'-(pentafluoro-λ^6-sulfanyl)-[1,1'-bi(bicyclo[1.1.1]pentane)]-3-sulfonyl fluoride (**68**):

R_f: 0.16 (4:1 hexanes:CH_2Cl_2), vis: *p*-anisaldehyde.

m.p.: 172.6–174.6 ºC.

^{1}H (300 MHz, $CDCl_3$): δ = 2.28 (s, 6H), 2.25 (s, 6H).

^{19}F (282 MHz, $CDCl_3$): δ = 79.68-77.61 (p, 1F, $^2J_{F-F}$ = 145.6 Hz), 48.79-48.27 (d, 4F, $^2J_{F-F}$ = 145.6 Hz), 45.34 (s, 1F).

^{13}C{^{1}H} (151 MHz, $CDCl_3$): δ = 65.8-65.5 (m), 54.3-54.3 (m), 52.0, 48.0-47.8 (d, $^3J_{C-F}$ = 22.28 Hz), 38.0, 33.9.

ATR-IR (ν_{max}): 3007, 2959, 2851, 1400, 1222, 1141, 820, 804, 788, 761, 623 cm^{-1}.

HRMS (*m/z*): (ESI) calc'd for $C_{10}H_{13}F_6O_2S_2^+$ [M-H]$^+$: calculated 343.0256, found 343.0258.

Compound 69

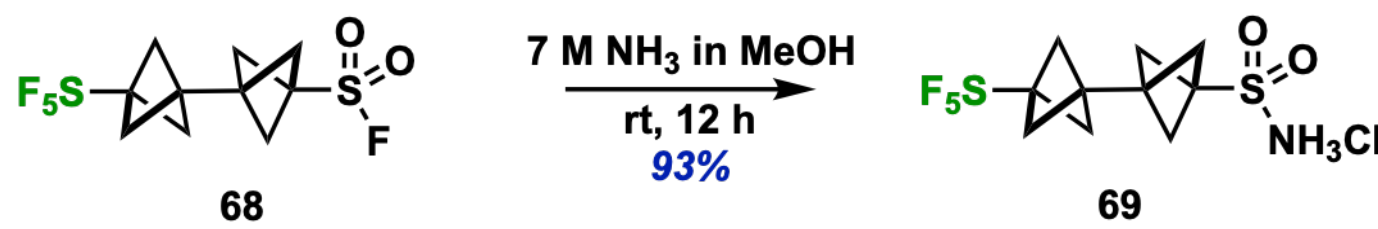

The synthesis of compound **69** was adapted from a known literature procedure.[35] To an oven-dried microwave vial equipped with a stir bar was added compound **68** (40.0 mg, 0.111 mmol, 1.0 equiv.). The vial was sealed with a septum, evacuated, and backfilled with Ar three times. Then, 7 M solution in NH_3 in MeOH (1.0 mL) was added *via* syringe and the resulting mixture was stirred at room temperature for 12 h. Upon completion, the reaction mixture was concentrated to afford a white solid (39.0 mg, 93%), which required no further purification.

chloro((3'-(pentafluoro-λ^6-sulfanyl)-[1,1'-bi(bicyclo[1.1.1]pentan)]-3-yl)sulfonyl)-λ^5-azane (**69**):
R_f: 0.41 (19:1 CH_2Cl_2:MeOH), vis: *p*-anisaldehyde.
m.p.: 172.3 ºC.
^{1}H (300 MHz, MeOD-d_4): δ = 2.26 (s,6H), 2.06 (s, 6H).
^{19}F (282 MHz, MeOD-d_4): δ = 78.81-76.78 (p, 1F, $^2J_{F-F}$ = 145.6 Hz), 47.04-46.53 (d, 4F, $^2J_{F-F}$ = 145.6 Hz).
^{13}C{^{1}H} (151 MHz, MeOD-d_4): δ = 67.8-67.5 (m), 55.2, 51.4, 51.0, 36.8, 35.4.
ATR-IR (ν_{max}): 3334, 3242, 3134, 3014, 2926, 1403, 1318, 1214, 1132, 815, 797 cm^{-1}.
HRMS (*m/z*): (ESI) calc'd for $C_{10}H_{15}F_5NO_2S_2^+$ [M-Cl]$^+$: calculated 340.0464, found 340.0467.

Compound S-29

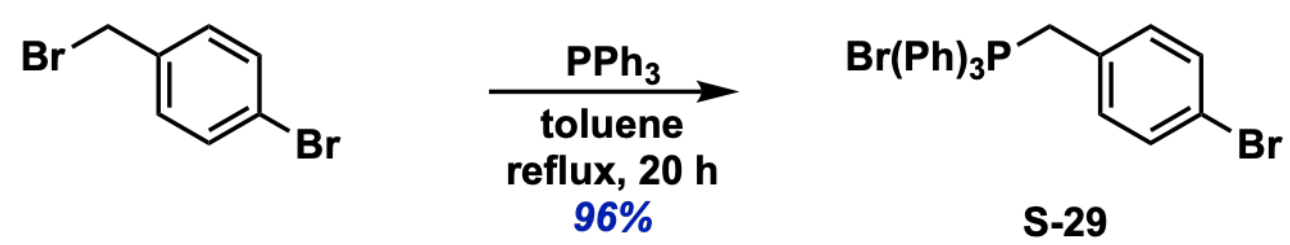

The synthesis of compound **S-29** was adapted from a known literature procedure.[36] To an oven-dried 250 mL two-neck round bottom flask equipped with a stir bar were added 1-bromo-4-(bromomethyl)benzene (4.00 g, 16.0 mmol, 1.00 equiv.) and triphenylphosphine (4.61 g, 17.0 mmol, 1.1 equiv.). The round bottom flask was attached a reflux column, the system was sealed with septa, evacuated, and backfilled with Ar three times. Anhydrous toluene (38 mL) was then added *via* syringe and the reaction mixture was heated to 130 °C in an oil bath for 20 h. The reaction mixture was then cooled to room temperature and concentrated. The resulting slurry was filtered and washed with Et_2O to obtain a white solid (7.90 g, 96%).

(4-bromobenzyl)triphenylphosphonium bromide (**S-29**)
R_f: 0.30 (9:1 CH_2Cl_2:MeOH), vis: UV (254 nm).
m.p.: 278.8–283.9 ºC.
^{1}H (300 MHz, $CDCl_3$): δ = 7.80-7.71 (m, 9H), 7.62-7.56 (m, 6H), 7.18-7.15 (m, 2H), 7.08-7.04 (m, 2H), 5.61 (s, 1H), 5.56 (s, 1H).
^{31}P{^{1}H} (121 MHz, $CDCl_3$): δ = 23.23.
^{13}C{^{1}H} (100 MHz, $CDCl_3$): δ = 134.9, 134.9, 134.5, 134.4, 133.4, 133.3, 131.7, 131.6, 130.1, 130.0, 126.64, 126.5, 122.6, 122.5, 118.0, 117.1, 30.0.
ATR-IR (ν_{max}): 3034, 3009, 2875, 2783, 1486, 1109 cm^{-1}.
HRMS (*m/z*): (ESI) calc'd for $C_{25}H_{21}P^+$ $[M-H]^+$: calculated 431.0559, found 431.0553.

Compound S-30

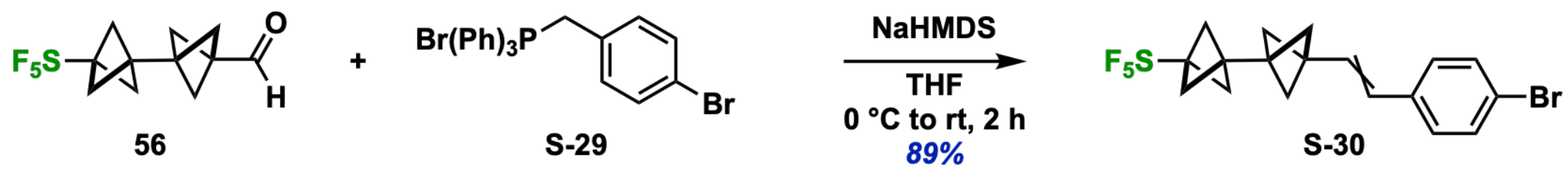

The synthesis of compound **S-30** was adapted from a known literature procedure.[36] To an oven-dried microwave vial equipped with a stir bar was added the compound **S-29** (2.35 g, 4.58 mmol, 1.5 equiv.). The vial was sealed with a septum, evacuated, and backfilled with Ar three times. Anhydrous toluene (8.0 mL) was then added *via* syringe. The reaction mixture was cooled to 0 °C in an ice water bath. Subsequently, 1.0 M solution of NaHMDS in THF (4.60 mL, 4.58 mmol, 1.5 equiv.) was added dropwise. *(Note: the reaction mixture turns bright orange indicative of the ylide formation.)* The reaction mixture was stirred at 0 °C for 1 h. A stock solution containing compound **56** (0.880 g, 3.05 mmol, 1.0 equiv.) dissolved in anhydrous THF (1.8 mL) was then added dropwise to the ylide solution *via* syringe. Subsequently, the reaction mixture was stirred at room temperature for 2 h. The reaction was quenched with sat. aq. NH_4Cl (5 mL), and the aqueous layer was extracted with Et_2O (3 x 20 mL). The combined organic layers were washed with brine (3 x 40 mL), dried over anhydrous Na_2SO_4, filtered through Celite, and concentrated. The crude residue was purified *via* column chromatography on silica gel eluting with 100% hexanes to afford a white solid (1.20 g, 89%). *(Note: a mixture of Z and E isomers was obtained.)*

(3'-(4-bromostyryl)-[1,1'-bi(bicyclo[1.1.1]pentan)]-3-yl)pentafluoro-λ^6-sulfane (**S-30**):
R_f: 0.46 (*cis*) and 0.35 (*trans*) (hexanes), vis: *p*-anisaldehyde.
m.p.: 120.1–121.6 ºC.
^{1}H (300 MHz, $CDCl_3$): δ = 7.06-7.04 (m, 2H), 6.44-6.40 (d, 1H, $^3J_{H-H}$ = 11.9 Hz), 5.69-5.65 (d, 1H, $^3J_{H-H}$ = 11.9 Hz), 2.10 (s, 6H), 1.64 (s, 6H).
^{19}F (283 MHz, $CDCl_3$): δ = 80.65-78.60 (p, 1F, $^2J_{F-F}$ = 145.5 Hz), 48.32-47.81 (d, 4F, $^2J_{F-F}$ = 145.5 Hz).
^{13}C{^{1}H} (201 MHz, $CDCl_3$): δ = 136.2, 136.0, 131.7, 131.4, 130.8, 130.8, 130.1, 129.5, 129.3, 129.8, 121.1, 120.8, 66.5-66.3 (m), 54.4, 54.3, 52.8, 51.2, 40.2, 38.6, 38.2, 37.4, 35.1, 34.9.
ATR-IR (ν_{max}): 3013, 2970, 2924, 2908, 2870, 1211, 814, 785 cm^{-1}.
HRMS (*m/z*): (ESI) calc'd for $C_{18}H_{17}BrF_5S^-$ [M-H]$^-$: calculated 439.0160, found 439.0169.

Compound 70

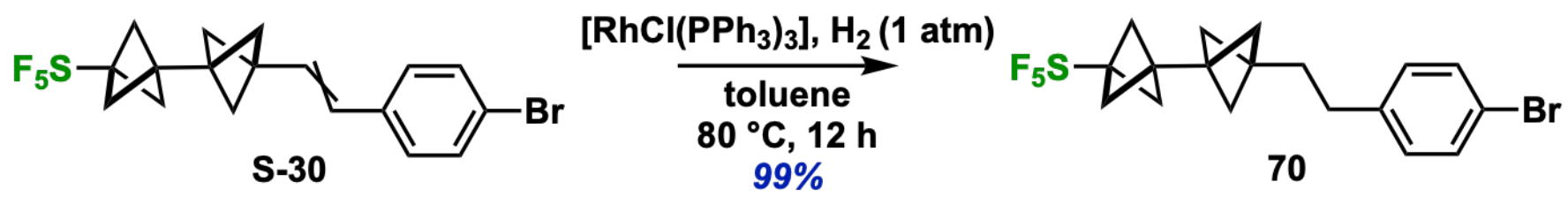

The synthesis of compound **70** was adapted from a known literature procedure.[37] To an oven-dried microwave vial equipped with a stir bar was added compound **S-33** (1.10 g, 2.49 mmol, 1.0 equiv.). The vial was brought into a nitrogen filled glovebox. Wilkinson's catalyst (0.461 g, 0.499 mmol, 0.2 equiv.) was then added and the vial was sealed and brought outside of the glovebox. Anhydrous toluene (10 mL) was added *via* syringe and the reaction mixture was sparged with H_2 for 10 min. The reaction mixture was then heated to 80 °C in an oil bath and stirred for 12 h under H_2 atmosphere. Upon reaction completion, the reaction mixture was cooled to room temperature and concentrated. The crude residue was loaded onto a silica plug eluting with hexanes to afford a white solid (0.929 g, 99%).

(3'-(4-bromophenethyl)-[1,1'-bi(bicyclo[1.1.1]pentan)]-3-yl)pentafluoro-λ^6-sulfane (**70**):
R_f: 0.26 (hexanes), vis: UV (254 nm).
m.p.: 86.6–89.8ºC.
^{1}H (300 MHz, CDCl$_3$): δ = 7.42-7.39 (m, 2H), 7.06-7.03 (m, 2H,), 2.59-2.56 (m, 2H), 2.16 (s, 6H), 1.94-1.89 (m, 2H).
^{19}F (283 MHz, CDCl$_3$): δ = 80.72-78.66 (p, 1F, $^2J_{F-F}$ = 145.6 Hz), 48.50-47.99 (d, 4F, $^2J_{F-F}$ = 145.6 Hz).
^{13}C{^{1}H} (101 MHz, CDCl$_3$): δ = 140.1, 131.6, 130.0, 120.0, 66.8-66.2 (m), 55.2-55.1 (m), 35.4, 32.4, 30.8.
ATR-IR (ν_{max}): 2926, 2359, 1490, 1186, 811 cm^{-1}.
HRMS (*m/z*): (ESI) calc'd for $C_{13}H_{15}BrF_5S^+$ [M-H]$^+$: calculated 376.9993, found 377.0010.

Compound 71

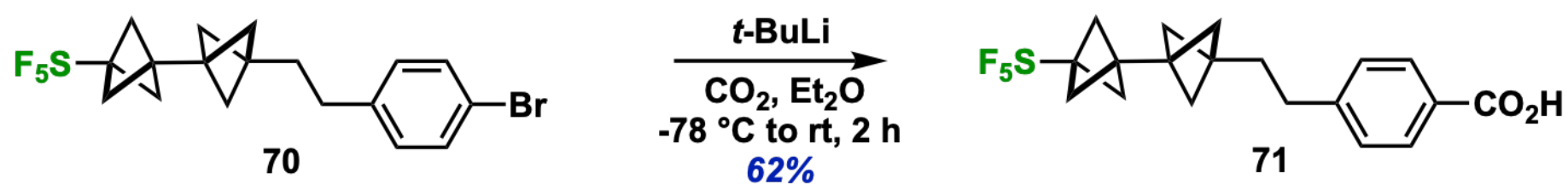

To an oven-dried microwave vial equipped with a stir bar was added compound **70** (0.100 g, 0.226 mmol, 1.0 equiv.). The vial was sealed with a septum, evacuated, and backfilled with Ar three times. Anhydrous Et_2O (2.0 mL) was then added *via* syringe and the reaction mixture was cooled to -78 °C. Subsequently, 1.7 M solution of *t*-BuLi in pentane (0.29 mL, 0.496 mmol, 2.2 equiv.) was added dropwise to the reaction mixture and stirred at -78 °C for 1 h. Then, the reaction was quenched at -78 °C using a CO_2 filled balloon that was carefully bubbled through the solution. The reaction mixture was then quenched with 1 M HCl (3 mL), transferred to a separatory funnel and extracted with EtOAc (3 x 30 mL). The combined organic layers were washed with brine (3 x 40 mL), dried over anhydrous Na_2SO_4, filtered through Celite, and concentrated. The crude residue was triturated with hexanes to afford a white solid (57.2 mg, 62%), which required no further purification.

4-(2-(3'-(pentafluoro-λ^6-sulfanyl)-[1,1'-bi(bicyclo[1.1.1]pentan)]-3-yl)ethyl)benzoic acid (**71**):

R_f: 0.20 (97:3 CH_2Cl_2:MeOH), vis: *p*-anisaldehyde.

m.p.: decomposes above 180 ºC.

^{1}H (300 MHz, Acetone-*d₆*): δ = 7.96-7.93 (m, 2H), 7.36-7.34 (m, 2H), 2.71-2.65 (m, 2H), 2.21 (s, 6H), 1.86-1.80 (m, 2H), 1.57 (s, 6H).

^{19}F (283 MHz, Acetone- d_6): δ = 80.60-78.57 (p, 1F, $^2J_{F-F}$ = 142.9 Hz), 47.62-47.12 (d, 4F, $^2J_{F-F}$ = 142.9 Hz).

^{13}C{^{1}H} (201 MHz, Acetone-d_6): δ = 167.5, 148.8, 130.6, 129.2, 129.0, 67.9-67.7 (m), 54.9, 50.0, 39.8, 37.6, 35.7, 33.6, 33.4.

ATR-IR (ν_{max}): 2971, 2924, 2908, 2873, 1673, 1291, 825, 795 cm^{-1}.

HRMS (*m/z*): (ESI) calc'd for $C_{19}H_{20}F_5O_2S^-$ [M-H]$^-$: calculated 407.1110, found 407.1100.

Compound S-31

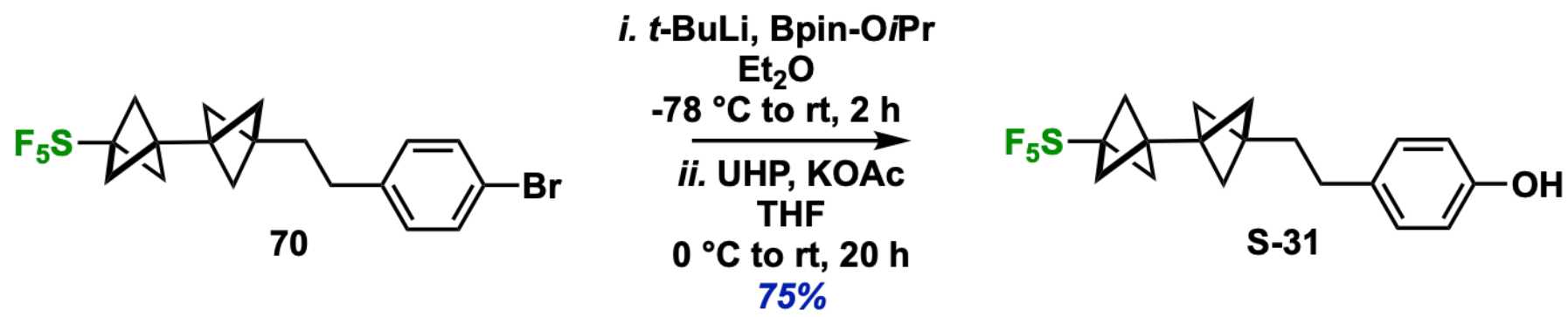

To an oven-dried microwave vial equipped with a stir bar was added compound **70** (0.150 g, 0.338 mmol, 1.0 equiv.). The vial was sealed with a septum, evacuated, and backfilled with Ar three times. Anhydrous Et_2O (1.9 mL) was added *via* syringe, and the resulting mixture was cooled to -78 °C. Then, a 1.7 M solution of *t*-BuLi in pentane (0.438 mL, 0.744 mmol, 2.2 equiv.) was added dropwise *via* syringe. After addition was complete, the reaction mixture was stirred at -78 °C for 1 h. Then, Bpin-O*i*Pr (0.08 mL, 0.406 mmol, 1.2 equiv.) was added dropwise at -78 °C. After addition, the cooling bath was removed, and the reaction was gradually warmed to room temperature. The reaction mixture was quenched with 1 M HCl (3.0 mL), transferred to a separatory funnel, and extracted with EtOAc (3 x 3 mL). The combined organic layers were washed with brine (3 x 4 mL), dried over anhydrous Na_2SO_4, filtered through Celite, and concentrated, and the crude was used without further purification.

The crude residue was added to an oven-dried microwave vial equipped with a stir bar and dissolved in THF (1.69 mL) and cooled to 0 °C. To the cooled solution, was added urea hydrogen peroxide (38.0 mg, 0.406 mmol, 1.2 equiv.), followed by potassium acetate (40.0 mg, 0.406 mmol, 1.2 equiv.). The cooling bath was removed, and the reaction was stirred at rt for 20 h. Upon reaction completion, the reaction mixture was diluted with sat. aq. NH_4Cl (3 mL) and transferred to a separatory funnel and extracted with EtOAc (3 x 3 mL). The combined organic layers were washed with brine (3 x 4 mL), dried over anhydrous Na_2SO_4, filtered through Celite, and concentrated. The crude residue was purified *via* column chromatography on silica gel eluting with 9:1 hexanes:EtOAc to afford a white solid (96.6 mg, 75%).

4-(2-(3'-(pentafluoro-λ^6-sulfanyl)-[1,1'-bi(bicyclo[1.1.1]pentan)]-3-yl)ethyl)phenol (**S-31**):
R_f: 0.22 (9:1 hexanes:EtOAc), vis: UV (254 nm).
m.p.: decomposes above 131.9 ºC.
^{1}H (300 MHz, MeCN-*d*$_3$): δ = 7.02-6.99 (m, 2H), 6.70-6.67 (m, 2H,), 6.66 (s, 1H), 2.50-2.44 (m, 2H), 2.18 (s, 6H), 1.72-1.67 (m, 2H), 1.50 (s, 6H).
^{19}F (283 MHz, MeCN-*d*$_3$): δ = 80.82-78.79 (p, 1F, $^2J_{F\text{-}F}$ = 145.6 Hz), 47.51-47.00 (d, 4F, $^2J_{F\text{-}F}$ = 145.6 Hz).
^{13}C{^{1}H} (101 MHz, MeCN-*d*$_3$): δ = 155.8, 134.6, 130.2, 115.9, 68.4-68.0 (m), 55.1-55.0 (m), 50.0, 39.9, 37.6, 35.8, 34.2, 32.5.
ATR-IR (ν_{max}): 3438, 3011, 2972, 2909, 2868, 1611, 1600, 1232, 1210, 845, 820, 796, 784 cm^{-1}.
HRMS (*m/z*): (ESI) calc'd for $C_{18}H_{21}F_5OS^-$ [M-H]$^-$: calculated 379.1161, found 379.1151.

Compound 72

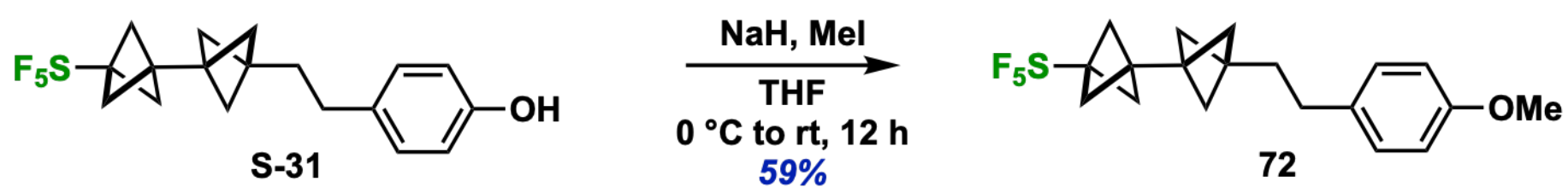

The synthesis of compound **72** was adapted from a known literature procedure.[38] To an oven-dried microwave vial equipped with a stir bar was added compound **S-34** (0.050 g, 0.132 mmol, 1.0 equiv.). The vial was sealed with a septum, evacuated, and backfilled with Ar three times. Anhydrous THF (0.66 mL) was added *via* syringe and cooled to 0 °C in an ice water bath. To the cooled solution, was added sodium hydride 60% dispersion in mineral oil (13.0 mg, 0.528 mmol, 4.0 equiv.) and stirred at 0 °C in a water-ice bath for 1 h. Then, methyl iodide (0.010 mL, 0.158 mmol, 1.2 equiv.) was added *via* syringe, and the cooling bath was removed and the reaction mixture was gradually warmed to room temperature and stirred for 12 h. Upon reaction completion, the reaction mixture was quenched with sat. aq. NH_4Cl (3 mL) and transferred to a separatory funnel and extracted with EtOAc (3 x 3 mL). The combined organic layers were washed with brine (3 × 4 mL), dried over anhydrous Na_2SO_4, filtered through Celite, and concentrated. The crude residue was purified *via* preparatory TLC eluting with 4:1 hexanes:CH_2Cl_2 to afford a white solid (31.0 mg, 59%).

pentafluoro(3'-(4-methoxyphenethyl)-[1,1'-bi(bicyclo[1.1.1]pentan)]-3-yl)-λ^6-sulfane (**72**):
R_f: 0.47 (4:1 hexanes:CH_2Cl_2). vis: UV (254 nm).
m.p.: 120.3–123.6 ºC.
^{1}H (300 MHz, $CDCl_3$): δ = 7.11-7.06 (m, 2H), 6.84-6.80 (m, 2H,), 3.79 (s, 3H), 2.53-2.48 (m, 2H), 2.14 (s, 6H), 1.77-1.72 (m, 2H), 1.51 (s, 6H).
^{19}F (283 MHz, $CDCl_3$): δ = 80.81-78.75 (p, 1F, $^2J_{F-F}$ = 145.6 Hz), 48.30-47.79 (d, 4F, $^2J_{F-F}$ = 145.6 Hz).
^{13}C{^{1}H} (101 MHz, $CDCl_3$): δ = 157.8, 134.4, 129.2, 113.8, 67.0-66.3 (m), 55.3, 54.4-54.3 (m), 49.5, 39.3, 37.1, 35.1, 35.5, 32.0.
ATR-IR (ν_{max}): 2959, 2926, 2864, 1612, 1512, 1244, 1209, 1034, 814, 789 cm^{-1}.
HRMS (*m/z*): (ESI) calc'd for $C_{19}H_{24}F_5OS^+$ $[M-H]^+$: calculated 395.1463, found 395.1466.

Compound S-32

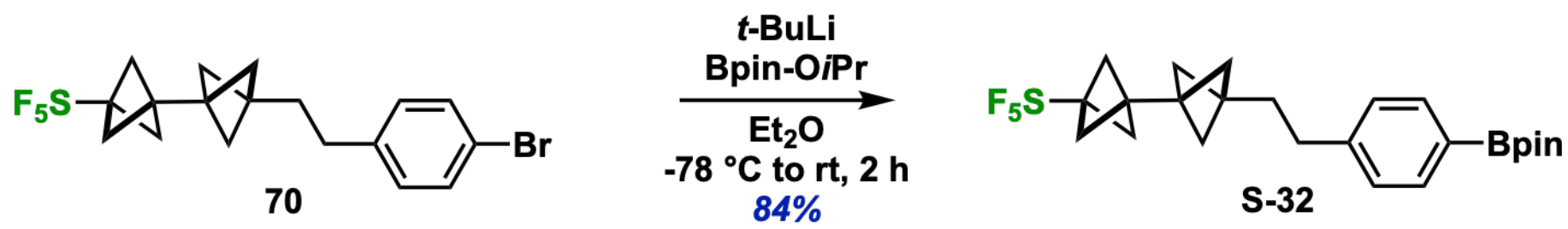

To an oven-dried microwave vial equipped with a stir bar was added compound **70** (0.300 g, 0.677 mmol, 1.0 equiv.). The vial was sealed with a septum, evacuated, and backfilled with Ar three times. Anhydrous Et_2O (5.0 mL) was added *via* syringe. The rection mixture was then cooled to -78 °C. *(Note: As the reaction mixture is cooled the viscosity increases dramatically. This can be remedied with a larger reaction vessel and stir bar or by decreasing the reaction concentration.)* To the cooled reaction mixture, a 1.7 M solution of *t*-BuLi in pentane (0.290 mL, 0.496 mmol, 2.2 equiv.) was added dropwise, and the solution was stirred at -78 °C for 1 h. Subsequently, Bpin-O*i*Pr (0.170 mL, 0.812 mmol, 1.2 equiv.) was added dropwise *via* syringe and the reaction mixture was stirred at -78 °C for 1 h. Subsequently, 1 M HCl (3 mL) was added, and aqueous layer was extracted with EtOAc (3 x 30 mL). The combined organic layers were washed with brine (3 x 40 mL), dried over anhydrous Na_2SO_4, filtered through Celite, and concentrated. The crude residue was purified *via* column chromatography on silica gel eluting with 100% hexanes followed by 95:5 hexanes:EtOAc to afford a white solid (0.232 g, 84%). *(Note: the material is metastable on silica so the material should not sit on silica for extended periods of time.)*

4,4,5,5-tetramethyl-2-(4-(2-(3'-(pentafluoro-λ^6-sulfanyl)-[1,1'-bi(bicyclo[1.1.1]pentan)]-3-yl)ethyl)phenyl)-1,3,2-dioxaborolane (**S-32**):

R_f: 0.42 (95:5 hexanes:EtOAc), vis: *p*-anisaldehyde.

m.p.: 140.3–142.5 ºC.

^{1}H (300 MHz, $CDCl_3$): δ = 7.74-7.72 (m, 2H), 7.20-7.18 (m, 2H), 2.60-2.55 (m, 2H), 2.14 (s, 6H), 1.81-1.76 (m, 2H), 1.51 (s, 6H), 1.35 (s,12H).

^{19}F (283 MHz, $CDCl_3$): δ = 80.79-78.74 (p, 1F, $^2J_{F\text{-}F}$ = 145.4 Hz), 48.30-47.79 (d, 4F, $^2J_{F\text{-}F}$ = 145.4 Hz).

^{13}C{^{1}H} (76 MHz, $CDCl_3$): δ = 145.8, 134.9, 127.8, 83.7, 66.8-66.4 (m), 54.3, 49.5, 39.3, 37.1, 33.2, 33.1, 25.0.

ATR-IR (ν_{max}): 2979, 2928, 2906, 2867, 1360, 817, 797 cm^{-1}.

HRMS (*m/z*): (ESI) calc'd for $C_{24}H_{33}BF_5O_2S^+$ $[M-H]^+$: calculated 491.2209, found 491.2210.

Compound 73

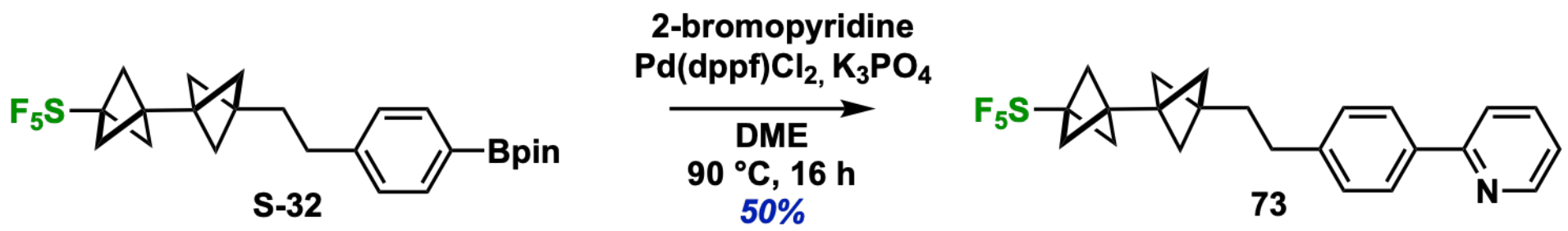

The synthesis of compound **73** was adapted from a known literature procedure.[39] To an oven-dried microwave vial equipped with a stir bar were added compound **S-32** (98.0 mg, 0.204 mmol, 1.0 equiv.), Pd(dppf)Cl_2 (0.015 mg, 0.020 mmol, 0.1 equiv.), and K_3PO_4 (0.216 g, 1.02 mmol, 5.0 equiv.). The vial was sealed with a cap with a septum using a crimper, evacuated, and backfilled with Ar three times. DME (1.5 mL) was added followed by 2-bromopyridine (0.020 mL, 0.204 mmol, 1.0 equiv.) *via* syringe. The reaction mixture was degassed by bubbling Ar through the solution for 5 mins. Subsequently, the degassed mixture was heated to 90 °C in an oil bath for 18 h. Upon reaction completion, the reaction mixture was diluted with EtOAc (1 mL) and transferred to a separatory funnel. The organic layer was washed with H_2O (3 x 40 mL), brine (3 x 40 mL), dried over anhydrous Na_2SO_4, filtered through Celite, and concentrated. The crude residue was purified *via* column chromatography on silica gel eluting with 95:5 hexanes:EtOAc to 1:1 hexanes:EtOAc to afford a white solid (44.0 mg, 50%).

2-(4-(2-(3'-(pentafluoro-λ^6-sulfanyl)-[1,1'-bi(bicyclo[1.1.1]pentan)]-3-yl)ethyl)phenyl)pyridine (**73**):

R_f: 0.19 (98:5 hexanes:EtOAc), vis: UV (254 nm).

m.p.: 160.2–161.2 ºC.

^{1}H (400 MHz, $CDCl_3$): δ = 8.68-8.67 (m, 1H), 7.91-7.89 (m, 2H), 7.75-7.0 (m, 2H), 7.29-7.27 (m, 2H), 7.22-7.19 (m, 1H), 2.64-2.60 (m, 2H), 2.13 (s, 6H), 1.84-1.80 (m, 2H), 1.52 (s, 6H).

^{19}F (283 MHz, $CDCl_3$): δ = 80.82-78.76 (p, 1F, $^2J_{F\text{-}F}$ = 144.4 Hz), 48.32-47.81 (d, 4F, $^2J_{F\text{-}F}$ = 144.4 Hz).

^{13}C{^{1}H} (101 MHz, $CDCl_3$): δ = 157.5, 149.7, 143.3, 137.0, 136.8, 128.8, 126.9, 121.9, 120.4, 66.9-66.3 (m), 54.3, 49.6, 39.3, 37.1, 35.1, 33.1, 32.7.

ATR-IR (ν_{max}): 3001, 2967, 2924, 2904, 2869, 1210, 793, 779 cm^{-1}.

HRMS (*m/z*): (ESI) calc'd for $C_{23}H_{25}F_5NS^+$ $[M-H]^+$: calculated 442.1622, found 442.1602.

Compound 74

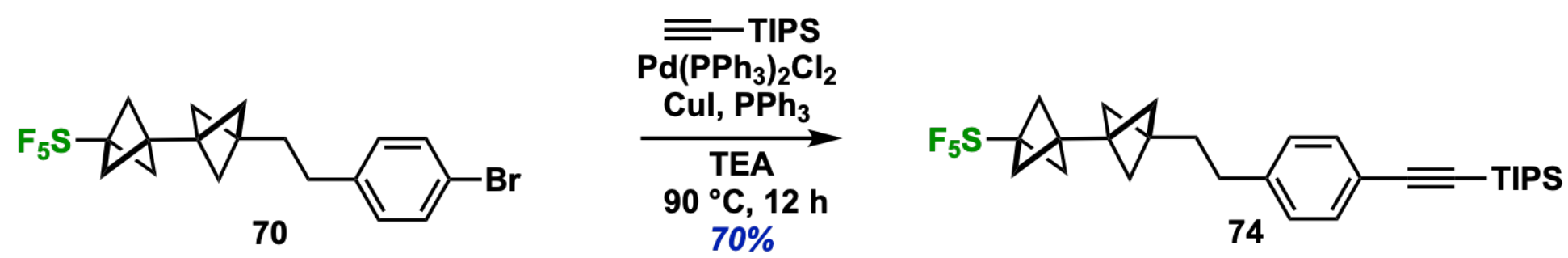

The synthesis of compound **74** was adapted from a known literature procedure.[40] To an oven-dried microwave vial equipped with a stir bar was added compound **70** (50.0 mg, 0.113 mmol, 1.00 equiv.), $Pd(PPh_3)_2Cl_2$ (0.004 mg, 0.006 mmol, 0.05 equiv.), CuI (1.2 mg, 0.006 mmol, 0.06 equiv.), and PPh_3 (3.00 mg, 0.011 mmol, 0.1 equiv.). The vial was sealed with a cap with a septum using a crimper, evacuated, and backfilled with Ar three times. Anhydrous TEA (0.3 mL) and TIPS acetylene (0.03 mL, 0.135 mmol, 1.2 equiv.) were then added *via* syringe. The reaction mixture was sparged with Ar gas for 2 mins and then stirred at 90 °C in an oil bath for 12 h. Upon reaction completion, the reaction mixture was quenched with H_2O (3 mL), transferred to a separatory funnel, and extracted with EtOAc (3 x 30 mL). The combined organic layers were washed with H_2O (3 x 4 mL), brine (3 x 4 mL), dried over anhydrous Na_2SO_4, filtered through Celite, and concentrated. The crude residue was purified *via* column chromatography on silica gel eluting with 100% hexanes to afford a white solid (42.7 mg, 70%).

triisopropyl((4-(2-(3'-(pentafluoro-λ^6-sulfanyl)-[1,1'-bi(bicyclo[1.1.1]pentan)]-3-yl)ethyl)phenyl)ethynyl)silane (**74**):

R_f: 0.25 (hexanes), vis: *p*-anisaldehyde.

m.p.: 84.4–87.9 ºC.

^{1}H (300 MHz, $CDCl_3$): δ = 7.40-7.37 (m, 2H), 7.11-7.09 (m, 2H), 2.58-2.52 (m, 2H), 2.14 (s, 6H), 1.78-1.73 (m, 2H), 1.50 (s, 6H), 1.13 (s, 21H).

^{19}F (283 MHz, $CDCl_3$): δ = 80.76-78.70 (p, 1F, $^2J_{F-F}$ = 144.8 Hz), 48.30-47.79 (d, 4F, $^2J_{F-F}$ = 144.8 Hz).

^{13}C{^{1}H} (101 MHz, $CDCl_3$): δ = 142.8, 132.1, 128.2, 121.0, 107.3, 89.8, 66.7-66.4 (m), 54.3, 49.6, 39.3, 37.1, 35.1, 33.0, 32.9, 18.8, 11.4.

ATR-IR (ν_{max}): 2959, 2940, 2907, 2864, 2159, 1210, 839, 821 cm^{-1}.

HRMS (*m/z*): (ESI) calc'd for $C_{29}H_{42}F_5SSi^+$ [M-H]$^+$: calculated 545.2691, found 545.2692.

Compound 75

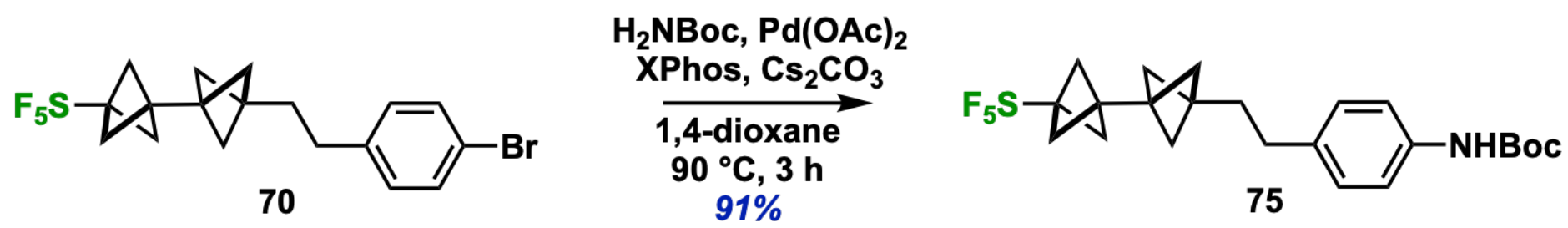

The synthesis of compound **75** was adapted from a known literature procedure.[41] To an oven-dried microwave vial equipped with a stir bar was added compound **70** (0.300 g, 0.677 mmol, 1.0 equiv.), tert-butyl carbamate (95.0 mg, 0.812 mmol, 1.2 equiv.), palladium diacetate (15.0 mg, 0.068 mmol, 0.1 equiv.), XPhos (64.0 mg, 0.135 mmol, 0.2 equiv.), cesium carbonate (0.309 g, 0.948 mmol, 1.4 equiv.), followed by 1,4-dioxane (5.42 mL). The vial was sealed with a cap with a septum using a crimper and sparged with Ar for 10 mins. The resulting solution was stirred at 90 °C in an oil bath for 3 h. Upon reaction completion, the reaction mixture was cooled to room temperature, diluted with CH_2Cl_2 (10 mL), and filtered through Celite. The filtrate was concentrated. The crude residue was purified *via* column chromatography on silica gel eluting with 9:1 hexanes:EtOAc to afford a white solid (0.297 g, 91%).

tert-butyl (4-(2-(3'-(pentafluoro-λ^6-sulfanyl)-[1,1'-bi(bicyclo[1.1.1]pentan)]-3-yl)ethyl)phenyl)carbamate (**75**):
R_f: 0.31 (9:1 hexanes:EtOAc), vis: UV (254nm).

m.p.: 152.5–154.1 ºC.

^{1}H (300 MHz, $CDCl_3$): δ = 7.27-7.22 (m, 2H), 7.10-7.05 (m, 2H,), 6.41 (s, 1H), 2.53-2.47 (m, 2H), 2.13 (s, 6H), 1.76-1.71 (m, 2H), 1.51 (s, 9H), 1.49 (s, 6H).

^{19}F (283 MHz, $CDCl_3$): δ = 80.79-78.73 (p, 1F, $^2J_{F-F}$ = 145.6 Hz), 48.29-47.77 (d, 4F, $^2J_{F-F}$ = 145.6 Hz).

^{13}C{^{1}H} (101 MHz, $CDCl_3$): δ = 153.0, 137.1, 136.2, 128.8, 118.8, 80.5, 66.9-66.3 (m), 54.4-54.3 (m), 49.6, 39.3, 37.1, 35.2, 33.3, 32.2, 28.5.

ATR-IR (ν_{max}): 3397, 2967, 2922, 2867, 1709, 1522, 1507, 1154, 821, 796 cm^{-1}.

HRMS (*m/z*): (ESI) calc'd for $C_{23}H_{31}F_5NO_2S^+$ [M-H]$^+$: calculated 480.1990, found 480.1987.

Compound 76

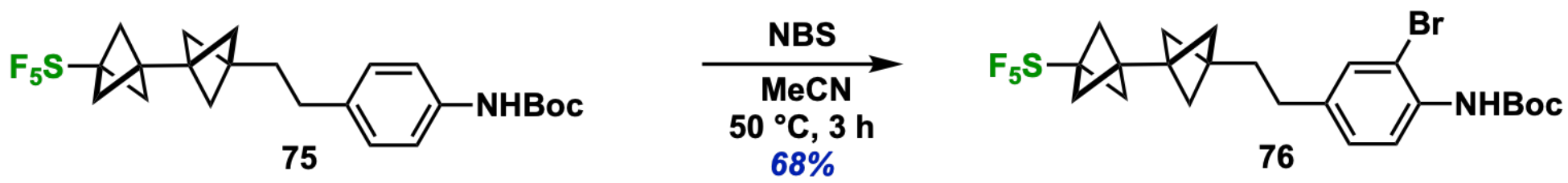

The synthesis of compound **76** was adapted from a known literature procedure.[42] To an oven-dried microwave vial equipped with a stir bar were added compound **75** (48.5 mg, 0.101 mmol, 1.0 equiv.) and NBS (36.0 mg, 0.202 mmol, 2.0 equiv.). The vial was sealed with a cap with a septum using a crimper, evacuated, and backfilled with Ar three times. Anhydrous MeCN (1.25 mL) was then added *via* syringe, and the reaction mixture was heated to 50 °C in an oil bath and stirred for 3 h. The reaction mixture was then diluted with EtOAc (1 mL) and H_2O (10 mL), transferred to a separatory funnel, and extracted with EtOAc (3 x 30 mL). The combined organic layers were washed with brine (3 x 40 mL), dried over anhydrous Na_2SO_4, filtered through Celite, and concentrated. The crude reside was purified *via* column chromatography on silica gel eluting with 95:5 hexanes:EtOAc to afford a white solid (38.7 mg, 68%)

(*tert*-butyl (2-bromo-4-(2-(3'-(pentafluoro-λ^6-sulfanyl)-[1,1'-bi(bicyclo[1.1.1]pentan)]-3-yl)ethyl)phenyl)carbamate (**76**):

R_f: 0.39 (95:5 hexanes:EtOAc), vis: *p*-anisaldehyde.

m.p.: 156.0–158.5 ºC.

^{1}H (400 MHz, CDCl$_3$): δ = 8.01-7.99 (m, 1H), 7.31 (s, 1H), 7.08-7.06 (m, 2H), 6.90 (s, 1H), 2.50-2.46 (m, 2H), 2.13 (s, 6H), 1.75-1.71 (m, 2H), 1.53 (s, 9H), 1.50 (s, 6H).

^{19}F (283 MHz, CDCl$_3$): δ = 80.78-78.72 (p, 1F, $^2J_{F\text{-}F}$ = 144.4 Hz), 48.32-47.81 (d, 4F, $^2J_{F\text{-}F}$ = 144.4 Hz).

^{13}C{^{1}H} (76 MHz, CDCl$_3$): δ = 152.5, 138.1, 133.9, 131.6, 128.1, 120.0, 112.3, 80.9, 66.6-66.3 (m), 54.2, 49.4, 39.0, 37.0, 35.0, 33.0, 31.7, 28.3.

ATR-IR (ν_{max}): 3422, 2966, 2924, 2908, 2867, 1703, 1155, 817, 785 cm^{-1}.

HRMS (*m/z*): (ESI) calc'd for $C_{23}H_{28}BrF_5NO_2S^-$ [M-H]$^-$: calculated 556.0950, found 556.0952.

Compound S-33

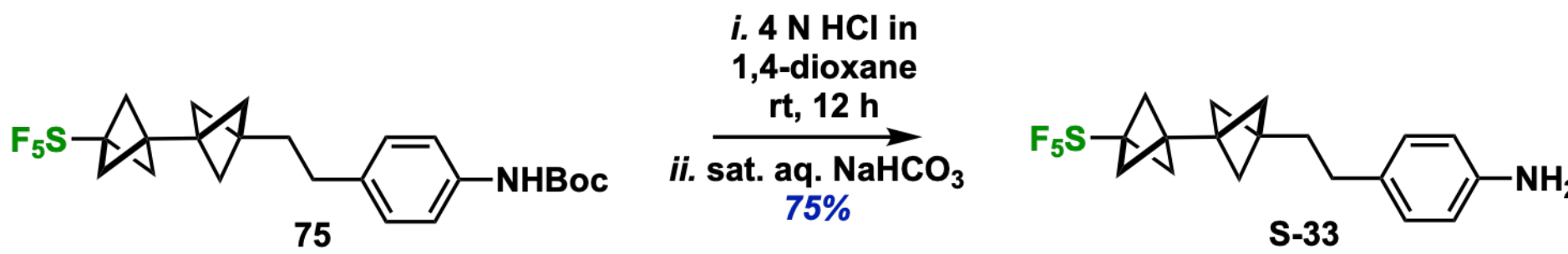

To an oven-dried microwave vial equipped with a stir bar was added compound **75** (0.203 g, 0.423 mmol, 1.0 equiv.) and a solution of 4 N HCl in 1,4-dioxane (5.0 mL). The resulting solution was stirred at rt for 12 h. Upon reaction completion, the precipitate formed was filtered and washed with Et_2O (3 x 10 mL). The residue was dissolved in MeOH (5 mL), transferred to a separatory funnel, followed by addition of H_2O (5 mL), EtOAc (5 mL). Then the aqueous layer was basified with sat. aq. $NaHCO_3$ (10 mL) and extracted with EtOAc (3 x 5 mL). The combined organic layers were washed with H_2O (3 x 3 mL), brine (3 x 4 mL), dried over anhydrous Na_2SO_4, filtered through Celite, and concentrated to afford a white solid (0.121 g, 75%).

4-(2-(3'-(pentafluoro-λ^6-sulfanyl)-[1,1'-bi(bicyclo[1.1.1]pentan)]-3-yl)ethyl)aniline (**S-33**):

R_f: 0.26 (4:1 hexanes:EtOAc), vis: UV (254 nm).

m.p.: decomposes above 128.1 ºC.

^{1}H (300 MHz, $CDCl_3$): δ = 6.98-6.93 (m, 2H), 6.64-6.60 (m, 2H,), 3.55 (s, 2H), 2.47-2.42 (m, 2H), 2.13 (s, 6H), 1.74-1.68 (m, 2H), 1.49 (s, 6H).

^{19}F (283 MHz, $CDCl_3$): δ = 80.87-78.81 (p, 1F, $^2J_{F-F}$ = 145.6 Hz), 48.29-47.77 (d, 4F, $^2J_{F-F}$ = 145.6 Hz).

^{13}C{^{1}H} (101 MHz, $CDCl_3$): δ = 144.2, 132.5, 129.1, 115.4, 66.9-66.4 (m), 54.4-54.3 (m), 49.5, 39.3, 37.1, 35.2, 33.5, 32.0.

ATR-IR (ν_{max}): 3496, 3410, 3011, 2963, 2918, 2866, 1626, 1517, 1447, 1287, 1208, 814, 784 cm^{-1}.

HRMS (*m/z*): (ESI) calc'd for $C_{18}H_{23}F_5NS^+$ [M-H]$^+$: calculated 380.1466, found 380.1468.

Compound 77

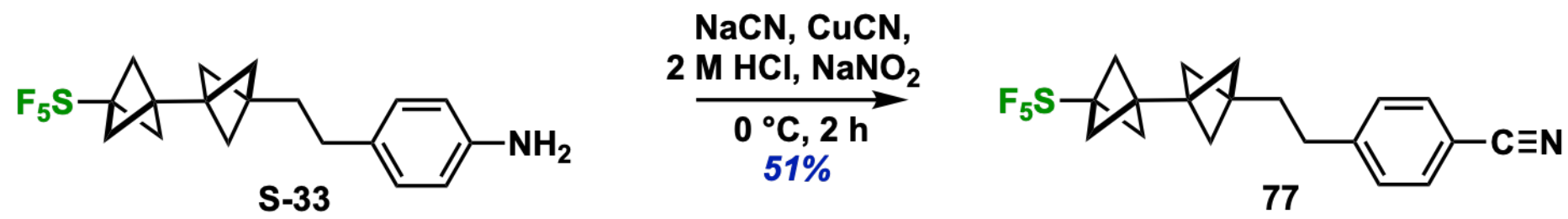

The synthesis of compound **77** was adapted from a known literature procedure.[43] A solution of NaCN (30.0 mg, 0.616 mmol, 3.6 equiv.) in H_2O (0.4 mL) was added at 0 °C to a stirred mixture of CuCN (20.0 mg, 0.239 mmol, 1.4 equiv.) in H_2O (0.3 mL) in a microwave vial. The resulting mixture was stirred at 0 °C for 30 mins. At the same time, in a separate vial compound **S-33** (65.0 mg, 0.171 mmol, 1.0 equiv.) was suspended in 2 N HCl (0.5 mL) and cooled to 0 °C, then a solution of $NaNO_2$ (13.0 mg, 0.188 mmol, 1.1 equiv.) in H_2O (0.2 mL) was added dropwise. The mixture was stirred for 20 mins at 0 °C in an ice water bath and then Na_2CO_3 was added portion wise to quench the excess of HCl. The resulting mixture was then added dropwise at 0 °C to the solution of CuCN/NaCN. The reaction mixture was stirred at 0 °C for 1 h, then 1 M HCl (5 mL) and EtOAc (5 mL) were added, the aqueous phase was extracted with EtOAc (3 x 3 mL). The combined organic phases were washed with brine (3 x 3 mL), dried over anhydrous Na_2SO_4, filtered through Celite, and concentrated. The crude residue was purified *via* preparatory TLC eluting with 4:1 hexanes:EtOAc to afford an orange solid (34.0 mg, 51%).

4-(2-(3'-(pentafluoro-λ^6-sulfanyl)-[1,1'-bi(bicyclo[1.1.1]pentan)]-3-yl)ethyl)benzonitrile (**77**):
R_f: 0.21 (19:1 hexanes:EtOAc), vis: UV (254 nm).
m.p.: 138.9–139.2 ºC.
^{1}H (300 MHz, $CDCl_3$): δ = 7.57-7.54 (m, 2H), 7.28-7.25 (m, 2H,), 2.64-2.59 (m, 2H), 2.14 (s, 6H), 1.81-1.75 (m, 2H), 1.51 (s, 6H).
^{19}F (283 MHz, $CDCl_3$): δ = 80.71-78.65 (p, 1F, $^2J_{F\text{-}F}$ = 145.6 Hz), 48.33-47.82 (d, 4F, $^2J_{F\text{-}F}$ = 145.6 Hz).
^{13}C{^{1}H} (101 MHz, $CDCl_3$): δ = 148.1, 132.3, 129.2, 119.2, 109.8, 66.8-66.2 (m), 54.4-54.2 (m), 49.5, 39.1, 37.2, 35.0, 33.1, 32.8.
ATR-IR (ν_{max}): 3014, 2985, 2928, 2890, 2228, 1218, 905, 838, 791 cm^{-1}.
HRMS (*m/z*): (ESI) calc'd for $C_{19}H_{21}F_5NS^+$ [M-H]$^+$: calculated 390.1309, found 390.1299.

Compound S-34

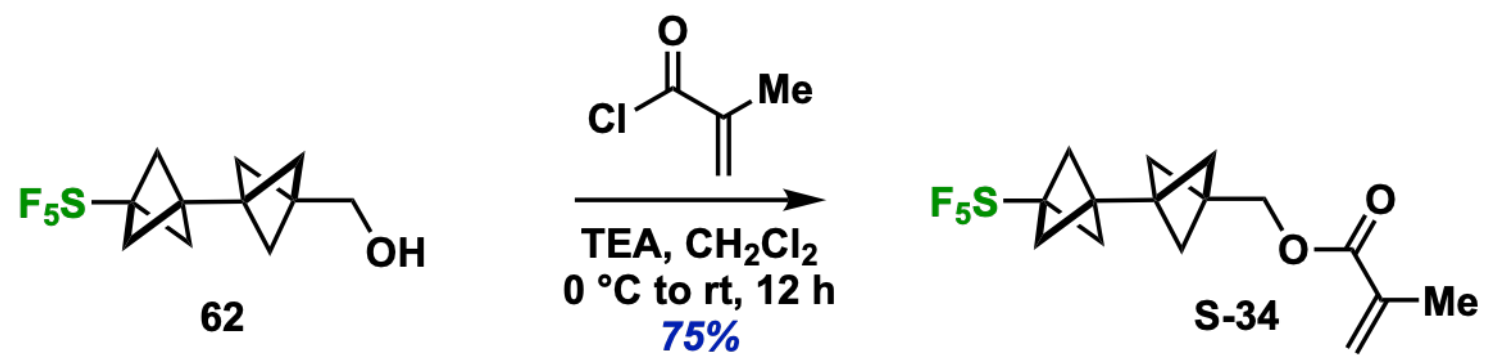

To an oven-dried microwave vial equipped with a stir bar was added compound **62** (0.050 g, 0.172 mmol, 1.0 equiv.). The vial was sealed with a septum, evacuated, and backfilled with Ar three times. Then, anhydrous CH_2Cl_2 (0.86 mL) was added and the reaction mixture was cooled to 0 °C using a water-ice bath. Then, TEA (0.028 mL, 0.206 mmol, 1.2 equiv.) was added dropwise, followed by a 1 M solution of methacryloyl chloride in CH_2Cl_2 (0.21 mL, 0.206 mmol, 1.2 equiv.). The cooling bath was removed, and the reaction mixture was stirred at room temperature for 12 h. Upon reaction completion, the reaction mixture was quenched with H_2O (3 mL), and the aqueous layer was extracted with CH_2Cl_2 (3 x 3 mL). The combined organic layers were washed with brine (3 x 4 mL), dried over anhydrous Na_2SO_4, filtered through Celite, and concentrated. The crude residue was purified *via* column chromatography on silica gel eluting with 9:1 hexanes:CH_2Cl_2 to 3:2 hexanes:CH_2Cl_2 to afford a white solid (46.3 mg, 75%).

(3'-(pentafluoro-λ^6-sulfanyl)-[1,1'-bi(bicyclo[1.1.1]pentan)]-3-yl)methyl methacrylate (**S-34**):
R_f: 0.30 (19:1 hexanes:EtOAc), vis: *p*-anisaldehyde.
m.p.: 69.5–72.3 ºC.
^{1}H (300 MHz, $CDCl_3$): δ = 6.10 (bs, 1H), 5.75-5.55 (m, 1H), 2.16 (s, 6H), 1.94 (bs, 3H), 1.64 (s, 6H).
^{19}F (282 MHz, $CDCl_3$): δ = 80.57-78.51 (p, 1F, $^2J_{F\text{-}F}$ = 145.6 Hz), 48.37-47.85 (d, 4F, $^2J_{F\text{-}F}$ = 145.6 Hz).
^{13}C{^{1}H} (151 MHz, $CDCl_3$): δ = 167.3, 136.4, 125.5, 66.8-66.1 (m), 64.1, 54.4-54.2 (m), 49.0, 38.0, 37.1, 35.0, 18.4.
ATR-IR (ν_{max}): 2978, 2918, 2882, 1704, 1633, 1458, 1326, 1305, 1289, 1209, 1170, 902, 818, 793, 645 cm^{-1}.
HRMS (*m/z*): (ESI) calc'd for $C_{15}H_{20}F_5O_2S^+$ [M-H]$^+$: calculated 359.1099, found 359.1101.

Compound S-35

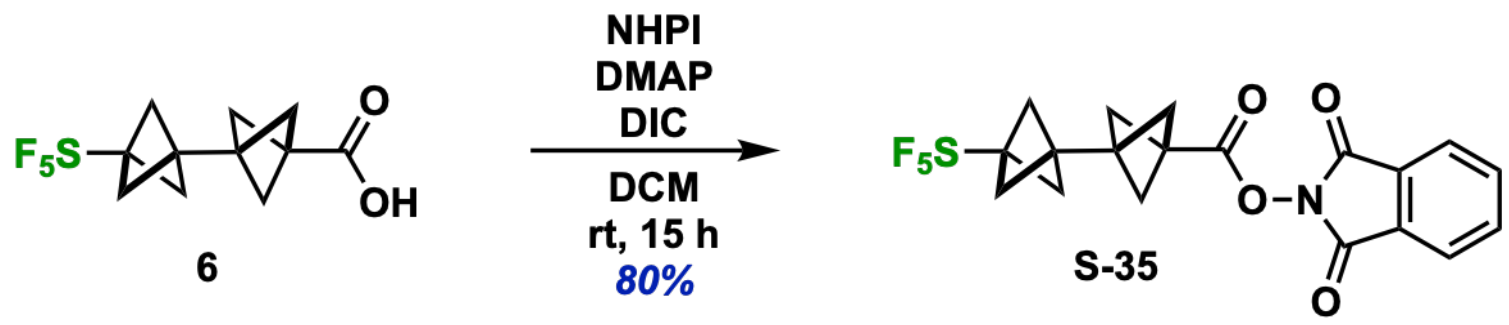

Synthesis of compound **S-35** was adapted from a known literature procedure[44]. An oven-dried microwave vial equipped with a stir bar was charged with compound **6** (0.300 g, 0.986 mmol, 1.0 equiv.), NHPI (0.177 g, 1.09 mmol, 1.1 equiv.) and DMAP (13.3 mg, 0.108 mmol, 0.11 equiv.). The vial was sealed with a septum, evacuated, and backfilled with Ar three times. Anhydrous CH_2Cl_2 (5.0 mL) was then added *via* syringe, and the reaction mixture was stirred at room temperature until solids were dissolved. Once dissolved, DIC (0.19 mL, 1.18 mmol, 1.2 equiv.) was added *via* syringe, and the reaction mixture was stirred at room temperature for 15 h. Upon completion, the reaction mixture was concentrated. The crude residue was purified *via* column chromatography on silica gel eluting with 9:1 hexanes:EtOAc to afford a white solid (0.353 g, 80%).

1,3-dioxoisoindolin-2-yl 3'-(pentafluoro-λ^6-sulfaneyl)-[1,1'-bi(bicyclo[1.1.1]pentane)]-3-carboxylate (**S-35)**:
R_f: 0.32 (4:1 hexanes:EtAOc). vis: *p*-anisaldehyde

m.p.: decomposes above 178.8 ºC.

^{1}H (300 MHz, $CDCl_3$): δ = 7.92-7.80 (m, 2H), 7.84-7.80 (m, 2H), 2.26 (s, 6H), 2.21 (s, 6H).
^{19}F (283 MHz, $CDCl_3$): δ = 80.20-78.13 (p, 1F, $^2J_{F\text{-}F}$ = 145.0 Hz), 48.56-48.05 (d, 4F, $^2J_{F\text{-}F}$ = 145.0 Hz).
^{13}C{^{1}H} (76 MHz, $CDCl_3$): δ = 165.07, 161.92, 134.94, 129.04, 124.12, 66.26-65.86 (m), 54.35, 51.90, 38.88, 35.05, 34.62.
ATR-IR (ν_{max}): 2995, 2926, 2888, 1739, 1001, 819, 800 cm^{-1}.
HRMS (*m/z*): (ESI) calc'd for $C_{19}H_{17}F_5NO_4S^+$ [M+H]$^+$: calculated 450.3995, found 450.3850.

Optimization Tables

Optimization for Compound 4

This general procedure was followed during optimization experiments with a few modifications, as summarized below. To an oven-dried microwave vial equipped with a stir bar was added compound **2** (64.0 mg, 0.200 mmol, 1.0 equiv.). The vial was sealed with a septum, evacuated, and purged with Ar three times. Then, anhydrous benzene (2.0 mL) was added, followed by oxalyl chloride (0.024 mL, 0.280 mmol, 1.4 equiv.) and hexamethylditin (0.059 mL, 0.280 mmol, 1.4 equiv.) sequentially *via* syringe. The resulting reaction mixture was irradiated with white LEDs (Kessil A160WE Tuna Blue, maximum intensity) for 16 h (the vial was placed ca. 5 cm away from the light source). Upon reaction completion, the crude reaction mixture was prepared for ^{19}F NMR analysis. In an NMR tube was combined 0.10 mL crude reaction mixture, 10 μL internal standard (α,α,α-trifluorotoluene), and 0.40 mL $CDCl_3$. During data collection, the relaxation delay (d1) was increased to 30 s, dummy scans were set to 0, no. of scans were set to 8, and the O1P was set to +30 ppm.

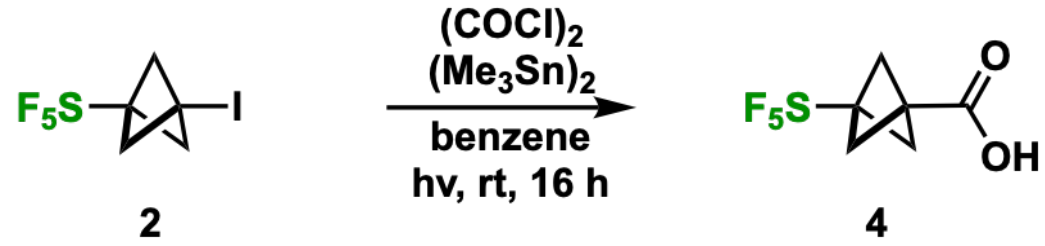

Table S1. Optimization for Compound **4.**

Entry	Deviations from standard conditions	Solvent	$(COCl)_2$ equiv.	Additive (equiv.)	^{19}F NMR Yield (%) *4*[a]
**1**[a]	White LEDs	benzene	1.4	$(Me_3Sn)_2$ (1.4)	77
**2**[b]	302 nm	freon	1.4	TTMSS (1.4)	20
**3**	302 nm	CH_2Cl_2	1.4	TTMSS (1.4)	8
**4**	302 nm	CCl_4	1.4	TTMSS (1.4)	3
**5**	302 nm	benzene	1.4	TTMSS (1.4)	22
**6**	Ambient light	benzene	1.4	TTMSS (1.4)	0
**7**[c]	254 nm	benzene	1.4	TTMSS (1.4)	8
**8**	White LEDs	benzene	1.4	TTMSS (1.4)	0
**9**[a]	Blue LEDs	benzene	1.4	TTMSS (1.4)	0
**10**[d]	Red LEDs	benzene	1.4	TTMSS (1.4)	10
**11**	AIBN (302 nm)	benzene	1.4	TTMSS (1.4)	13
**12**	AIBN (60 ºC)	benzene	1.4	TTMSS (1.4)	11
**12**	302 nm	benzene	1.4	-	0
**13**	302 nm	benzene	1.4	Bu_3SnH (1.4)	1
**14**	302 nm	benzene	1.4	Catechol (1.4)	0
**15**	302 nm	benzene	1.4	Ph_3SiH (1.4)	0
**16**	302 nm	benzene	3.0	TTMSS (1.4)	12
**17**	302 nm	benzene	6.0	TTMSS (1.4)	26
**18**	302 nm	benzene	12	TTMSS (1.4)	8
**19**	302 nm	benzene	24	TTMSS (1.4)	5
**20**	White LEDs	benzene	6.0	TTMSS (1.4)	37
**21**	Blue LEDs	benzene	6.0	TTMSS (1.4)	32
**22**	302 nm	benzene	1.4	$(Me_3Sn)_2$ (1.4)	65
**23**	Blue LEDs	benzene	1.4	$(Me_3Sn)_2$ (1.4)	61
**24**	0.05 M	benzene	1.4	$(Me_3Sn)_2$ (1.4)	62
**25**	0.40 M	benzene	1.4	$(Me_3Sn)_2$ (1.4)	57

[a] 40 W Kessil A160WE Tuna Blue LED light was used. [b] 302 nm Analytikjena Hg pen lamp (PN: 90-0001-04). [c] 254 nm Analytikjena Hg pen lamp (PN: 90-0012-01). 40 W Kessil A160WE Tuna Blue LED light was used. [d] 40 W Kessil H160 Tuna Flora LED light was used.

Optimization for Compound 5

This general procedure was followed during optimization experiments with a few modifications, as summarized below. To an oven-dried microwave vial equipped with a stir bar was added compound **2** (0.032 g, 0.100 mmol, 1.0 equiv.). The vial was sealed with a septum, evacuated, and backfilled with Ar three times. Then a ~0.8 M solution of [1.1.1]propellane in Et_2O (0.200 mmol, 2.0 equiv.) was added. The resulting mixture was irradiated using a 302 nm pen lamp. Upon reaction completion, the crude reaction mixture was prepared for ^{19}F NMR analysis. In an NMR tube was combined 0.10 mL crude reaction mixture, 10 μL internal standard (α,α,α-trifluorotoluene), and 0.40 mL $CDCl_3$. During data collection, the relaxation delay (d1) was increased to 30 s, dummy scans were set to 0, no. of scans were set to 8, and the O1P was set to +30 ppm.

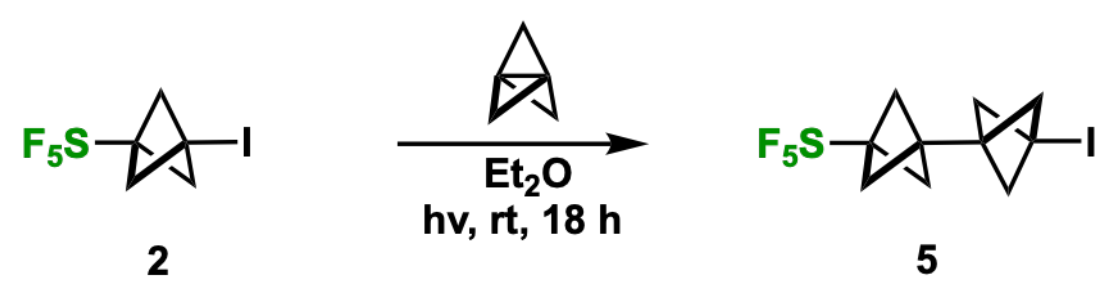

Table S2. Optimization for Compound **5.**

Entry	Deviations from standard conditions	[1.1.1]propellane Equiv.	^{19}F NMR Yield (%) *5*[a]
**1**[a]	302 nm	2.0	75
**2**	Ambient light	2.0	59
**3**[b]	Blue LEDs	2.0	60
**4**[b]	White LEDs	2.0	75
**5**[c]	Dark	2.0	60
**6**[d]	Dark (60 ºC)	2.0	42
**7**	Ambient light (60 ºC)	2.0	40
**8**	-	1.0	78
**9**	-	3.0	71
**10**	-	4.0	6
**11**	-	5.0	0
**12**	Et_2O (0.250 mL)	1.0	68
**13**	Et_2O (0.750 mL)	1.0	52
**14**	Et_2O (1.00 mL)	1.0	34
**15**	Et_2O (2.00 mL)	1.0	17
**16**	0.313 mmol scale	1.0	59
**17**	0.626 mmol scale	1.0	63
**18**	0.937 mmol scale	1.0	81

[a] 302 nm Analytikjena Hg pen lamp (PN: 90-0001-04) [b] 40 W Kessil A160WE Tuna Blue LED light was used. [c] Reaction vessel was wrapped with aluminum foil.

Initial Optimization for Compound 39 (in Ethanol)

This general procedure was followed during optimization experiments with a few modifications, as summarized below. To an oven-dried microwave vial equipped with a stir bar were added compound **38** (29.0 mg, 0.100 mmol, 1.0 equiv.) and $AgNO_3$ (0.356 g, 2.10 mmol, 21.0 equiv.), followed by EtOH (1.0 mL) or solvent specified in Table S3. The resulting mixture was placed into a pre-heated oil bath and stirred for 16 h at 40 °C. *(Note: No special precautions were taken to keep moisture or O_2 out of the reaction mixture.)* Upon reaction completion, the crude reaction mixture was prepared for ^{19}F NMR analysis. In an NMR tube was combined 0.10 mL crude reaction mixture, 10 μL internal standard (α,α,α-trifluorotoluene), and 0.40 mL $CDCl_3$. During data collection, the relaxation delay (d1) was increased to 30 s, dummy scans were set to 0, no. of scans were set to 8, and the O1P was set to +30 ppm.

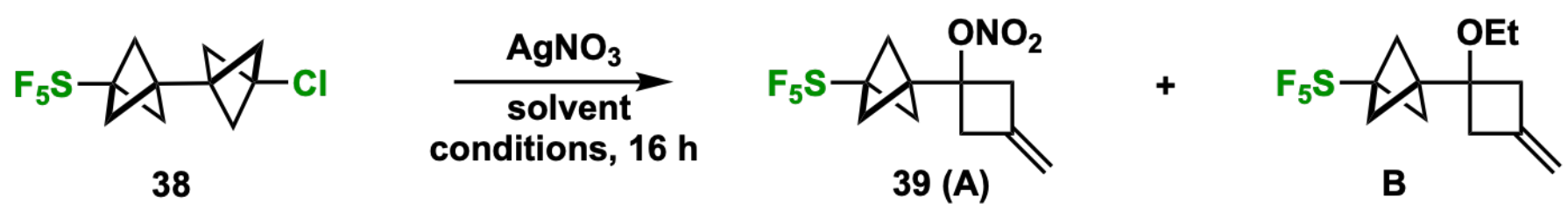

Table S3. Optimization for Compound **39.**

Entry	Solvent	Conditions	Additive	^{19}F Yield (%) *39 (A)*	A:B Ratio[a]	^{19}F NMR Yield (%) *B*
**1**	**EtOH**	**40 ºC**	**$AgNO_3$ (21 equiv.)**	**58**	**1.4:1**	**42**
**2**	EtOH	-	-	0	-	0
**3**	MeOH	-	-	0	-	N/A
**4**	EtOH	-	AcOH (1.5 eqiuv.)	0	-	0
**5**	EtOH	80 ºC	-	0	-	0
**6**	THF/H_2O (1:1)	-	pyridine (1.2 equiv.)	0	-	N/A
**7**	dioxane/H_2O (1:1)	-	TEA (1.2 equiv.)	0	-	N/A
**8**	EtOH	-	$AgNO_3$ (21 equiv.)	21	1.6:1	13
**9**	EtOH	40 ºC	$AgNO_3$ (21 equiv.)	41	1.2:1	36
**10**	EtOH	85 ºC	$AgNO_3$ (21 equiv.)	8	2.2:1	4
**11**	EtOH	85 ºC	$AgNO_3$ (1.0 equiv.)	35	0.8:1	41
**12**	EtOH/H_2O (4:1)	40 ºC	$AgNO_3$ (21 equiv.)	11	1.4:1	8
**13**	MeOH/H_2O (4:1)	40 ºC	$AgNO_3$ (21 equiv.)	8	0.9:1	9[b]
**14**	EtOH/H_2O (1:1)	40 ºC	$AgNO_3$ (21 equiv.)	1	-	0
**15**	H_2O	40 ºC	$AgNO_3$ (21 equiv.)	0	-	N/A
**16**	HFIP	40 ºC	-	0	-	N/A
**17**	EtOH	40 ºC	$AgNO_3$ (3.0 equiv.)	29	0.7:1	40
**18**	EtOH	40 ºC	$AgNO_3$ (10 equiv.)	37	0.9:1	43
**21**	CF_3CH_2OH	40 ºC	$AgNO_3$ (21 equiv.)	42	-	N/A
**22**	MeCN	40 ºC	$AgNO_3$ (21 equiv.)	28	-	N/A
**23**	EtOH	40 ºC	CuI (21 equiv.)	0	-	0
**24**	EtOH	40 ºC	$NiCl_2$ (21 equiv.)	0	-	0
**25**	EtOH	40 ºC	$ZnCl_2$ (21 equiv.)	0	-	0
**26**	EtOH	40 ºC	$Pd(OAc)_2$ (1.0 equiv.)	0	-	0

[a] Ratios determined by ^{19}F NMR. [b] Putative MeOH trapped product instead of EtOH trapped product.

Optimization for Compound 39 (in *t*-BuOH)

This general procedure was followed during optimization experiments with a few modifications, as summarized below. To an oven-dried microwave vial equipped with a stir bar were added compound **38** (29.0 mg, 0.100 mmol, 1.0 equiv.) and $AgNO_3$ (34.0 mg, 0.200 mmol, 2.0 equiv.), followed by *t*-BuOH (1.0 mL). The resulting mixture was placed into a pre-heated oil/sand bath and stirred for 16 h at 60 °C. *(Note: No special precautions were taken to keep moisture or O_2 out of the reaction mixture.)* Upon reaction completion, the crude reaction mixture was prepared for ^{19}F NMR analysis. In an NMR tube was combined 0.10 mL crude reaction mixture, 10 μL internal standard (α,α,α-trifluorotoluene), and 0.40 mL $CDCl_3$. During data collection, the relaxation delay (d1) was increased to 30 s, dummy scans were set to 0, no. of scans were set to 8, and the O1P was set to +30 ppm.

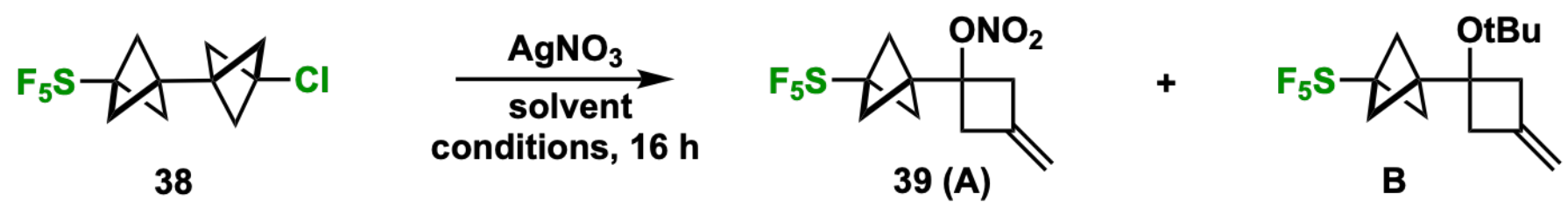

38 39 (A) B

Table S4. Optimization for Compound **39.**

Entry	Solvent	Conditions	Additive	^{19}F Yield (%) *39 (A)*	A:B Ratio[a]	^{19}F Yield (%) *B*
**1**	***t*-BuOH**	**60 ºC**	**$AgNO_3$ (2.0 equiv.)**	**83**	**6.3:1**	**13**
**2**	*t*-BuOH	40 ºC	$AgNO_3$ (2.0 equiv.)	76	3.3:1	23
**3**	*t*-BuOH	40 ºC	$AgNO_3$ (4.0 equiv.)	75	3.7:1	20
**4**	*t*-BuOH	40 ºC	$AgNO_3$ (6.0 equiv.)	72	3.0:1	24
**5**	*t*-BuOH	40 ºC	$AgNO_3$ (8.0 equiv.)	80	4.7:1	17
**6**	*t*-BuOH	40 ºC	$AgNO_3$ (21 equiv.)	61	2.5:1	24
**7**	*t*-BuOH (0.05 M)	40 ºC	$AgNO_3$ (2.0 equiv.)	64	2.3:1	28
**8**	*t*-BuOH (0.10 M)	40 ºC	$AgNO_3$ (2.0 equiv.)	68	4.6:1	15
**9**	*t*-BuOH (0.20 M)	40 ºC	$AgNO_3$ (2.0 equiv.)	62	6.0:1	10
**10**	*t*-BuOH	80 ºC	$AgNO_3$ (2.0 equiv.)	83	5.6:1	15

[a] Ratios determined by ^{19}F NMR.

First-Generation Synthesis Route Toward Tecovirimat and Derivative

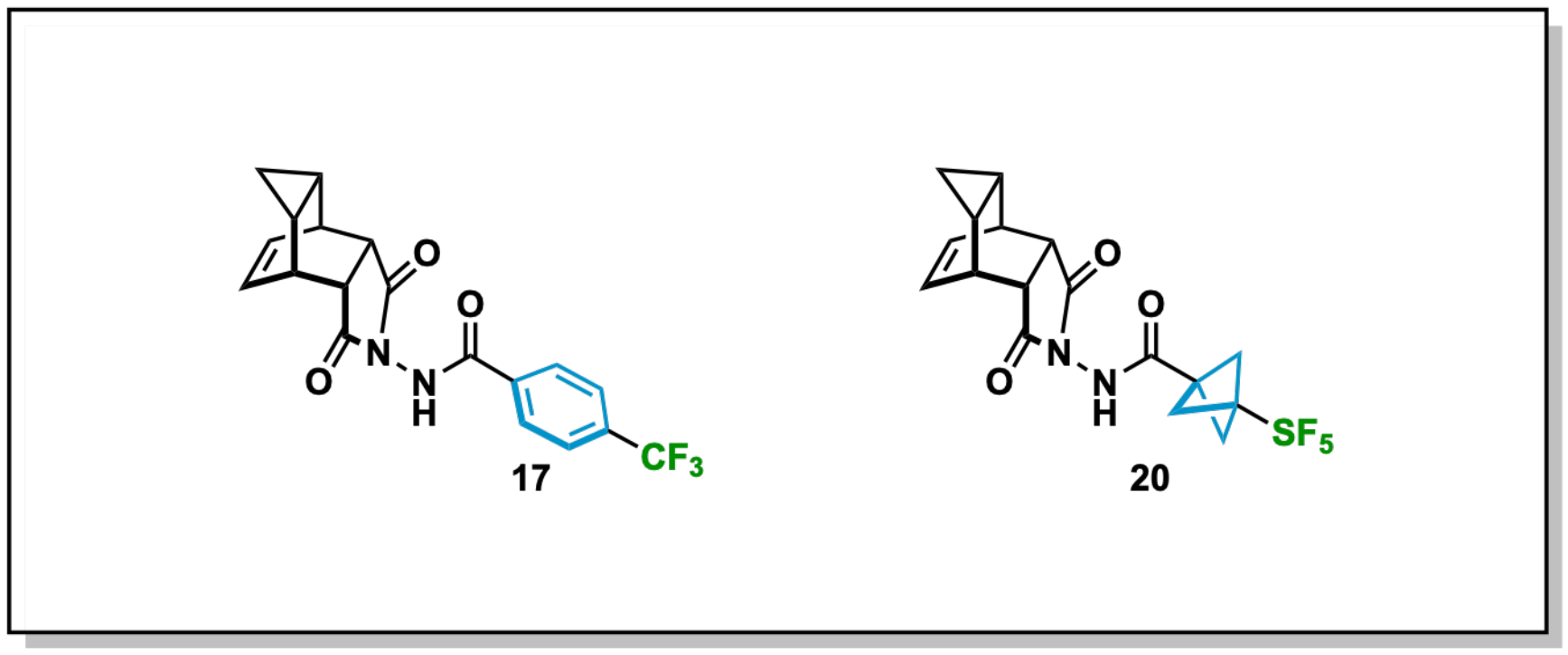

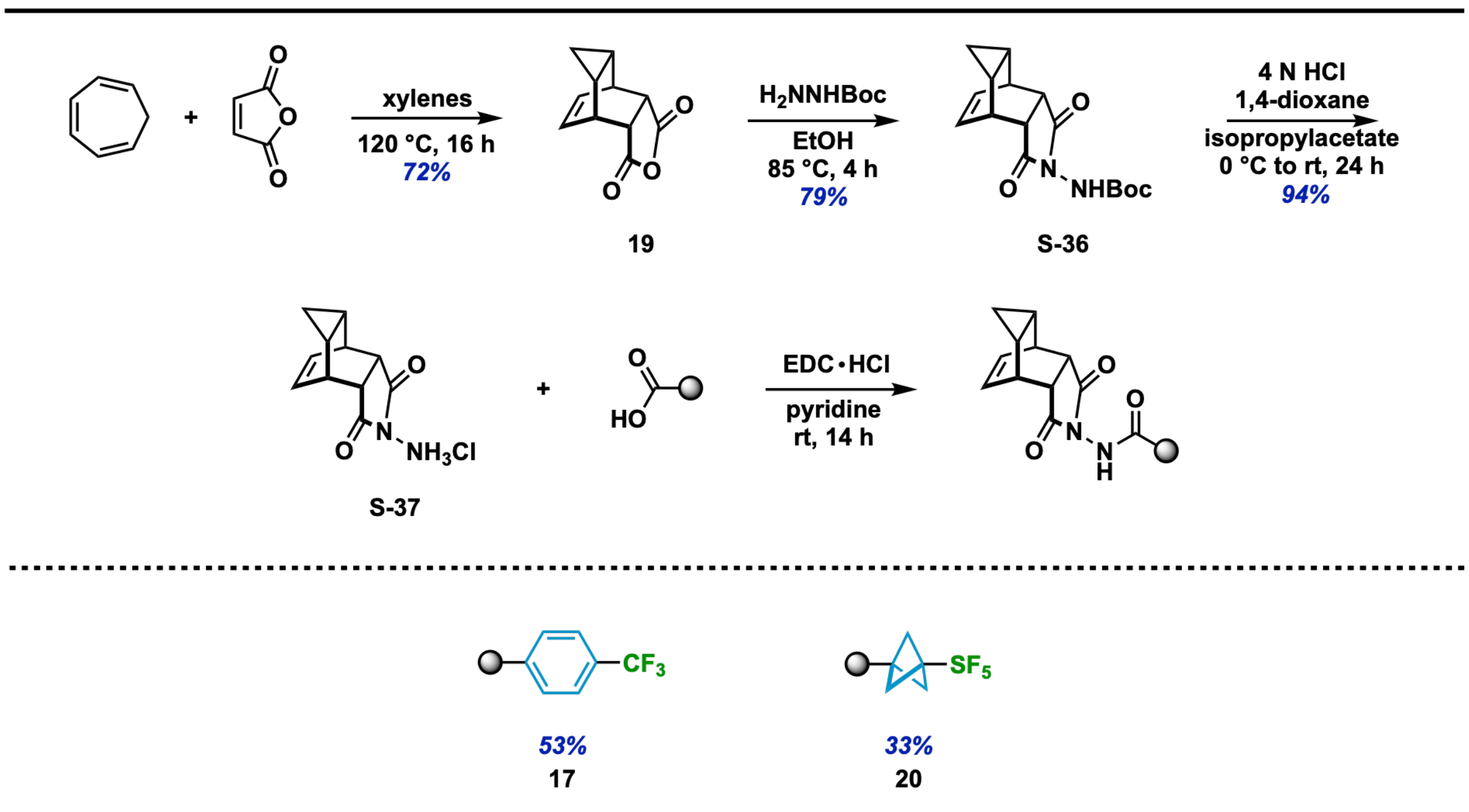

Scheme S9: First-generation synthesis overview of Tecovirimat (**17**) and its SF_5–BCP derivative (**20**).[45] See pages S141-S144 for detailed procedures and corresponding characterization data. *Note that this was ultimately replaced with a more convergent synthesis route that provided overall higher product yields.*

Compound S-36

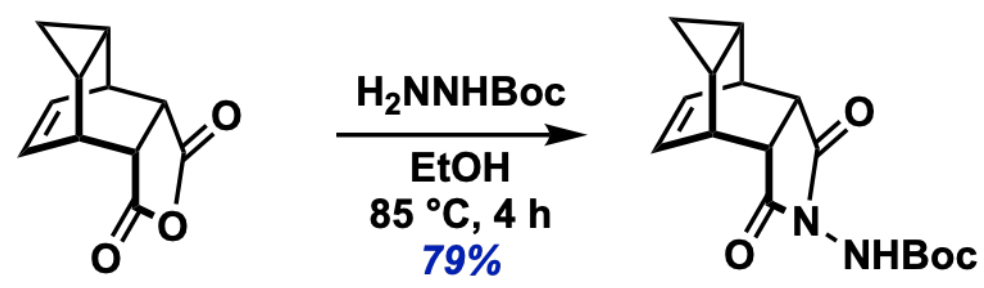

19 S-36

The synthesis of compound **S-36** was adapted from a known literature procedure.[45] To an oven-dried two-neck round bottom equipped with a stir bar and a reflux condenser was added compound **19** (1.00 g, 5.36 mmol, 1.1 equiv.). The round bottom and reflux condenser were sealed with septa, evacuated, and backfilled with Ar three times. EtOH (13 mL) was added *via* syringe followed by $H_2NNHBoc$ (0.730 g, 5.52 mmol, 1.05 equiv.). The reaction mixture was stirred at 85 °C in an oil bath for 4 h. Upon reaction completion, the reaction mixture was cooled to room temperature and concentrated. The resulting crude material was recrystallized from hexanes:EtOAc (3:1). The material was then purified *via* column chromatography on silica gel eluting with 99:1 CH_2Cl_2:MeOH to afford a white solid (1.27 g, 79%).

tert-butyl ((4*R*,4a*R*,5a*S*,6*S*,6a*S*)-1,3-dioxo-3,3a,4,4a,5,5a,6,6a-octahydro-4,6-ethenocyclopropa[*f*]isoindol-2(1*H*)-yl)carbamate (**S-36**):

R_f: 0.36 (99:1 CH_2Cl_2:MeOH), vis: $KMnO_4$.

m.p: 160.2–161.1 °C

^{1}H (300 MHz, $CDCl_3$): δ = 6.4 (s, 1H), 5.80-5.74 (m, 2H), 3.41 (s, 2H), 3.02 (s, 2H), 1.44 (s, 9H), 1.14-1.05 (m, 2H), 0.31-0.19 (s, 2H).

^{13}C{^{1}H} (101 MHz, $CDCl_3$): δ = 175.1, 153.0, 127.8, 82.8, 43.5, 33.3, 28.1, 9.6, 4.4.

ATR-IR (ν_{max}): 3315, 2975, 1709, 1159, 726 cm^{-1}.

HRMS (*m/z*): (ESI) calc'd for $C_{16}H_{21}N_2O_4^+$ $[M-H]^+$: calculated 305.1496 found 305.1497.

Compound S-37

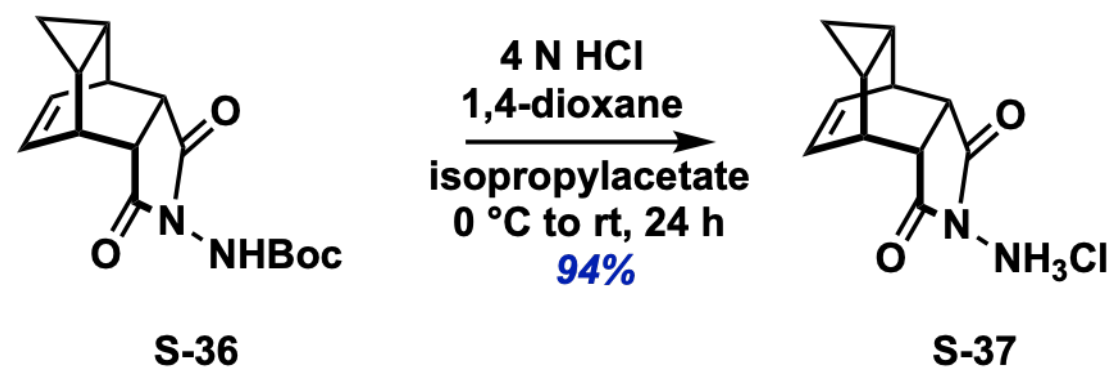

The synthesis of compound **S-37** was adapted from a known literature procedure.[45] To an oven-dried round bottom equipped with a stir bar was added compound **S-36** (0.500 g, 1.64 mmol, 1.0 equiv.). The round bottom was sealed with a septum, evacuated, and backfilled with Ar three times. Isopropylacetate (10 mL) was added *via* syringe, and the reaction mixture was cooled to 0 °C in an ice water bath. Then, 4 N HCl in 1,4-dioxane (12 mL) was added *via* syringe, and the reaction mixture was warmed gradually to room temperature. The reaction mixture was stirred for 24 h, and a precipitate had formed. The precipitate was washed with isopropylacetate and dried to afford a white solid (0.370 g, 94%), which required no further purification.

(4*R*,4a*R*,5a*S*,6*S*,6a*S*)-2-(chloro-λ^5-azaneyl)-4,4a,5,5a,6,6a-hexahydro-4,6-ethenocyclopropa[*f*]isoindole-1,3(2*H*,3a*H*)-dione (**S-37**):

Rf: 0.31 (99:1 CH_2Cl_2:MeOH), vis: $KMnO_4$.

m.p.: 143.9–147.2 °C.

^{1}H (300 MHz, DMSO-*d6*): δ = 6.12 (s, 1H), 5.70-5.64 (m, 2H), 3.22-3.17 (m, 2H), 3.00-2.98 (m, 2H), 1.14-1.09 (m, 2H), 0.26-19 (m, 1H), 0.04-0.01 (m, 1H).

^{13}C{^{1}H} (76 MHz, DMSO-*d6*): δ = 175.5, 127.3, 42.9, 32.7, 9.2, 4.1.

ATR-IR (ν_{max}): 3208, 2949, 1707, 731 cm^{-1}.

HRMS (*m/z*): (ESI) calc'd for $C_{11}H_{13}N_2O_2^+$ [M-H]$^+$: calculated 205.0972 found 205.0963.

Compound 17

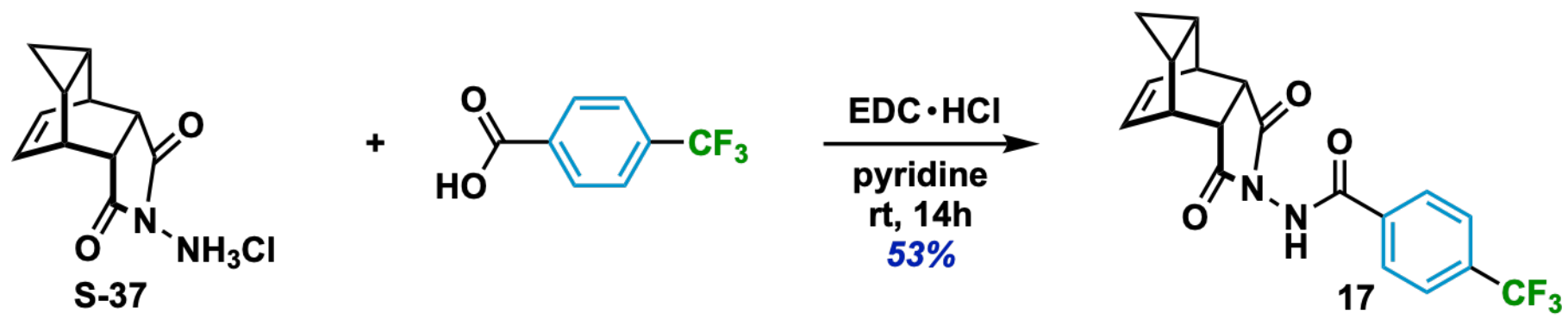

The synthesis of compound **17** was adapted from a known literature procedure.[45] To an oven-dried microwave vial equipped with a stir bar were added compound **S-37** (15.0 mg, 0.079 mmol, 1.0 equiv.), 4-(trifluoromethyl)benzoic acid (31.0 mg, 0.128 mmol, 1.62 equiv.) and EDC•HCl (19.7 mg, 0.103 mmol, 1.3 equiv.). The vial was sealed with a septum, evacuated, and backfilled with Ar three times. Anhydrous pyridine (0.5 mL) was added *via* syringe. The reaction mixture was stirred at room temperature for 14 h. The crude mixture was extracted with CH_2Cl_2 (3 x 10 mL). The combined organic layers were washed with brine (3 x 25 mL), dried over anhydrous Na_2SO_4, filtered through Celite and concentrated. The crude residue was purified *via* preparatory TLC eluting with 98.5:1.5 CH_2Cl_2:MeOH to afford a white solid (15.8 mg, 53%).

Please see page S35 for characterization data.

Compound 20

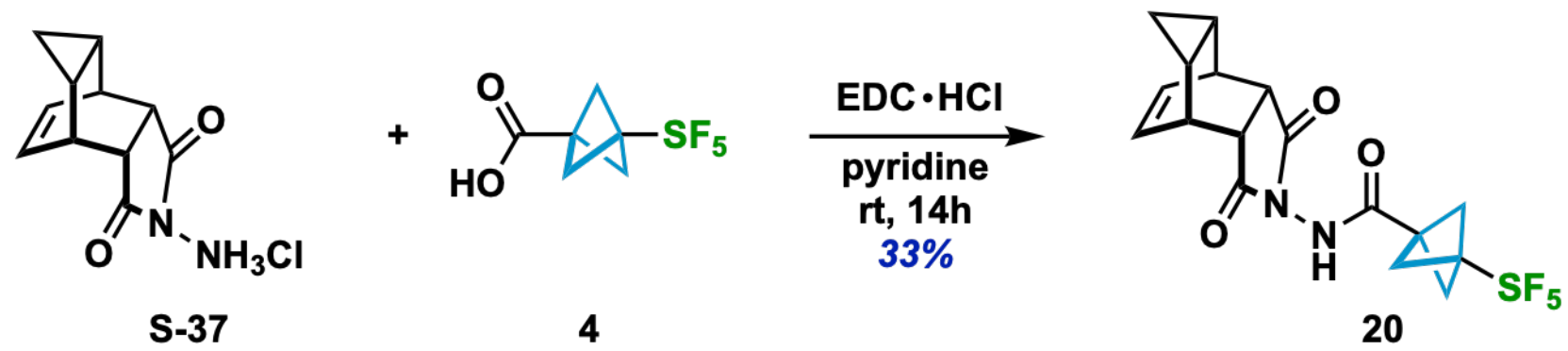

The synthesis of compound **20** was adapted from a known literature procedure.[45] To an oven-dried microwave vial equipped with a stir bar were added compound **4** (20.0 mg, 0.084 mmol, 1.0 equiv.), compound **S-37** (32.7 mg, 0.136 mmol, 1.62 equiv.) and EDC•HCl (20.9 mg, 0.109 mmol, 1.3 equiv.). The vial was sealed with a septum, evacuated, and backfilled with Ar three times. Anhydrous pyridine (1.0 mL) was added *via* syringe. The reaction mixture was stirred at room temperature for 14 h. The crude mixture was extracted with CH_2Cl_2 (3 x 10 mL). The combined organic layers were washed with brine (3 x 25 mL), dried over anhydrous Na_2SO_4, filtered through Celite, and concentrated. The crude reside was purified *via* preparatory TLC eluting with 98.5:1.5 CH_2Cl_2:MeOH to afford a white solid (11.6 mg, 33%).

Please see page S36 for characterization data.

First-Generation (Unsuccessful) Synthesis Route Toward OSU-T315 Derivative

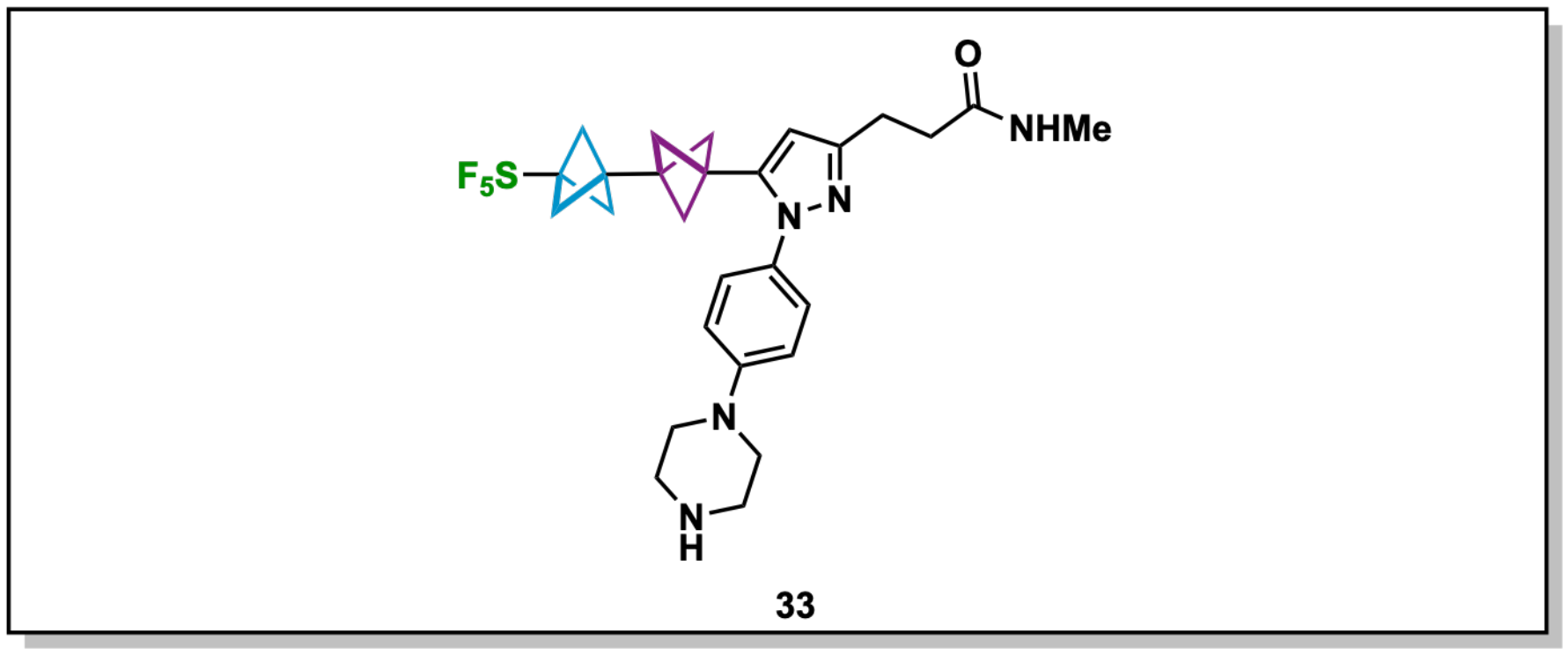

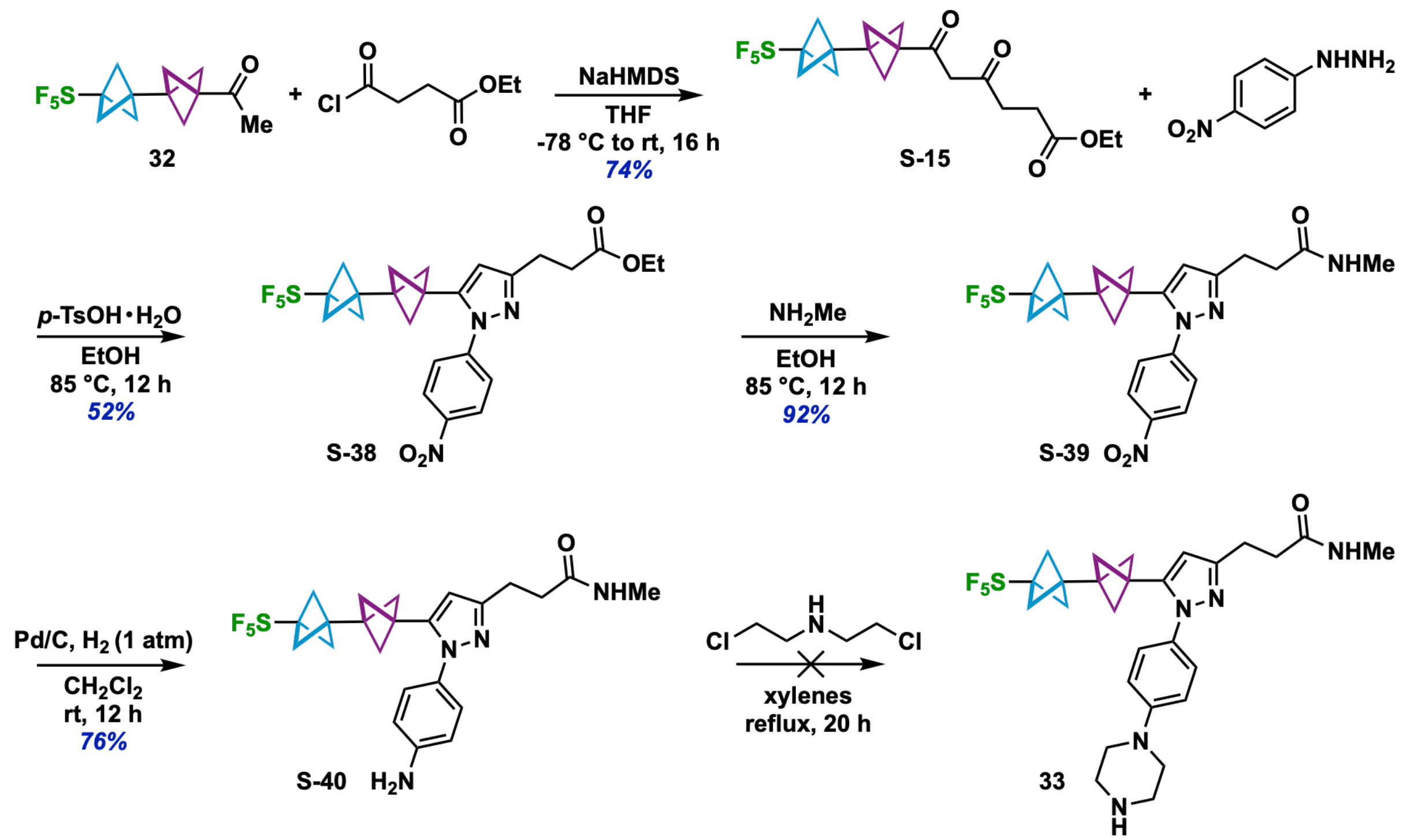

Scheme S10: First-generation (unsuccessful) synthesis overview of the SF_5–[2]staffane derivative of OSU-T315 (**33**).[10] See pages S146-S149 for detailed procedures and corresponding characterization data. *Note that an alternative (successful) route was ultimately developed to install the piperazine moiety via electrochemical Ni-catalyzed amination of the corresponding aryl bromide.*

Compound S-38

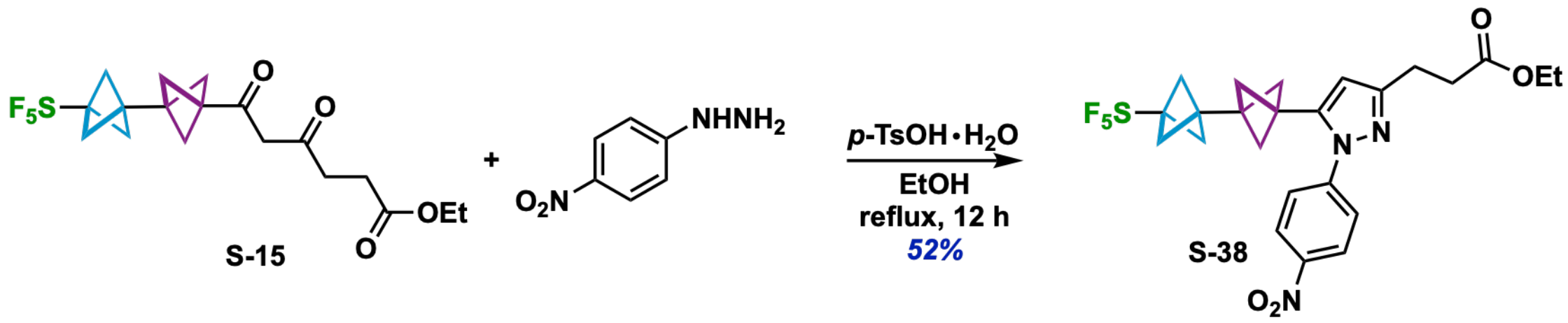

The synthesis of compound **S-38** was adapted from a known literature procedure.[10] To an oven-dried microwave vial equipped with a stir bar were added compound **S-15** (0.227 g, 0.527 mmol, 1.0 equiv.), 4-nitrophenylhydrazine hydrochloride (0.120 g, 0.633 mmol, 1.2 equiv.), and *p*-toluenesulfonic acid monohydrate (0.100 g, 0.527 mmol, 1.0 equiv.). The vial was sealed with a cap with a septum using a crimper, evacuated, and backfilled with Ar three times. Anhydrous EtOH (4.0 mL) was added *via* syringe. The resulting mixture, heated to 85 °C and refluxed in an oil barh for 12 h. Upon reaction completion, the vial was cooled to room temperature and concentrated. The crude residue was purified *via* column chromatography on silica gel eluting with 4:1 hexanes:EtOAc to afford a yellow solid (0.150 g, 52%).

ethyl 3-(1-(4-nitrophenyl)-5-(3'-(pentafluoro-λ^6-sulfanyl)-[1,1'-bi(bicyclo[1.1.1]pentan)]-3-yl)-1*H*-pyrazol-3-yl)propanoate (**S-38**):
R$_f$: 0.41 (4:1 hexanes:EtOAc), vis: UV (254 nm).
m.p.: 114.9–116.7 ºC.
^{1}H (300 MHz, CDCl$_3$): δ = 8.34-8.32 (m, 2H), 7.66-7.64 (m, 2H), 6.10 (s, 1H), 4.14-4.12 (q, 2H, $^3J_{H\text{-}H}$ = 7.05 Hz), 3.03-3.01 (m, 2H), 2.65-2.63 (m, 2H), 2.22 (s, 6H), 2.00 (s, 6H), 1.24-1.22 (t, 3H, $^3J_{H\text{-}H}$ = 7.05 Hz).
^{19}F (282 MHz, CDCl$_3$): δ = 80.58-78.52 (p, 1F, $^2J_{F\text{-}F}$ = 145.6 Hz), 48.40-47.88 (d, 4F, $^2J_{F\text{-}F}$ = 145.6 Hz).
^{13}C{^{1}H} (151 MHz, CDCl$_3$): δ = 171.9, 153.4, 146.4, 144.7, 143.2, 125.1, 124.9, 105.4, 66.5-66.2 (m), 61.04, 54.4, 51.8, 38.0, 35.6, 35.0, 33.0, 22.1, 14.3.
ATR-IR (ν_{max}): 3001, 2925, 2885, 1730, 1594, 1517, 1499, 1336, 1211, 1180, 1112, 1046, 901, 817, 791, 752 cm^{-1}.
HRMS (*m/z*): (ESI) calc'd for $C_{24}H_{27}F_5N_3O_4S$+ [M-H]$^+$: calculated 548.1637, found 548.1640.

Compound S-39

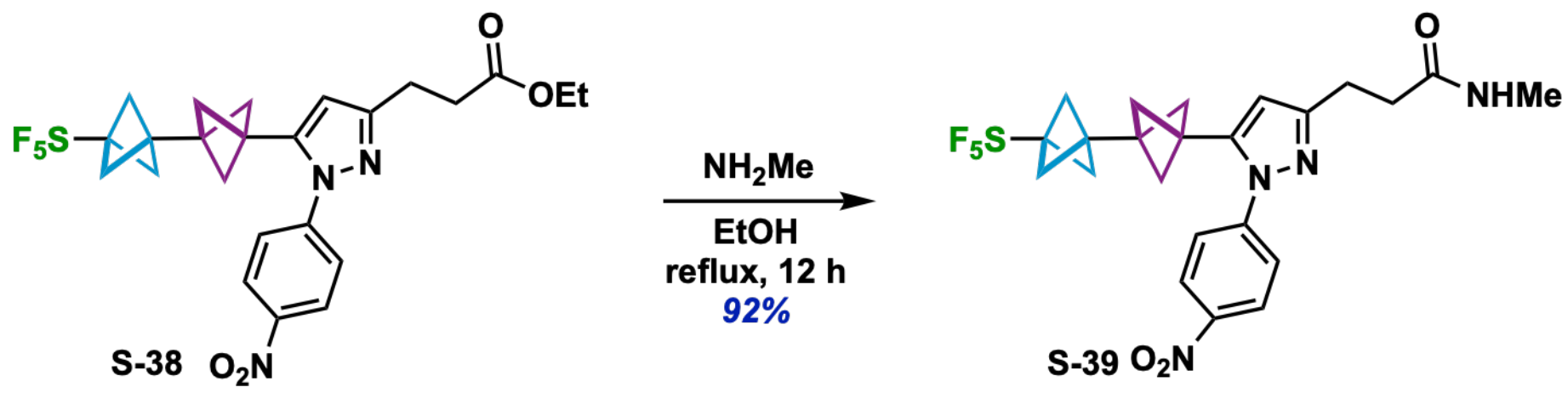

The synthesis of compound **S-39** was adapted from a known literature procedure.[10] To an oven-dried microwave vial equipped with a stir was added compound **S-38** (58.0 mg, 0.105 mmol, 1.0 equiv.). The vial was sealed with with a cap with a septum using a crimper, evacuated, and backfilled with Ar three times. To the vial, was added a 33% solution of methylamine in ethanol (1.0 mL) and heated to 85 °C and refluxed in an oil bath for 12 h. Upon reaction completion, the vial was cooled to room temperature and concentrated. The crude residue was purified *via* preparatory TLC eluting with 9:1 EtOAc:TEA to afford a yellow solid (51.0 mg, 92%).

N-methyl-3-(1-(4-nitrophenyl)-5-(3'-(pentafluoro-λ^6-sulfaneyl)-[1,1'-bi(bicyclo[1.1.1]pentan)]-3-yl)-1*H*-pyrazol-3-yl)propanamide (**S-39**):
R_f: 0.51 (9:1 EtOAc:TEA), vis: UV (254 nm).
m.p.: 141.9–147.9 ºC.
^{1}H (300 MHz, CDCl$_3$): δ = 8.33-8.31 (m, 2H), 7.68-7.66 (m, 2H), 6.08 (s, 1H), 5.48 (s, 1H), 3.07-3.05 (m, 2H), 2.81-2.80 (d, 3H, $^3J_{H-H}$ = 4.91Hz), 2.49-2.47 (m, 2H), 2.21 (s, 6H), 1.99 (s, 6H).
^{19}F (282 MHz, CDCl$_3$): δ = 80.70-78.63 (p, 1F, $^2J_{F-F}$ = 145.6 Hz), 48.37-47.85 (d, 4F, $^2J_{F-F}$ = 145.6 Hz).
^{13}C{^{1}H} (151 MHz, CDCl$_3$): δ = 171.2, 153.4, 146.3, 144.7, 143.6, 125.1, 124.9, 105.3, 66.5-66.2 (m), 54.41, 51.9, 35.6, 35.0, 34.9, 29.8, 26.5, 22.4.
ATR-IR (ν_{max}): 3384, 2919, 2849, 1648, 1595, 1499, 1333, 1209, 1114, 966, 905, 852, 819, 800, 740 cm^{-1}.
HRMS (*m/z*): (ESI) calc'd for $C_{23}H_{26}F_5N_4O_3S$+ $[M-H]^+$: calculated 533.1540, found 533.1638.

Compound S-40

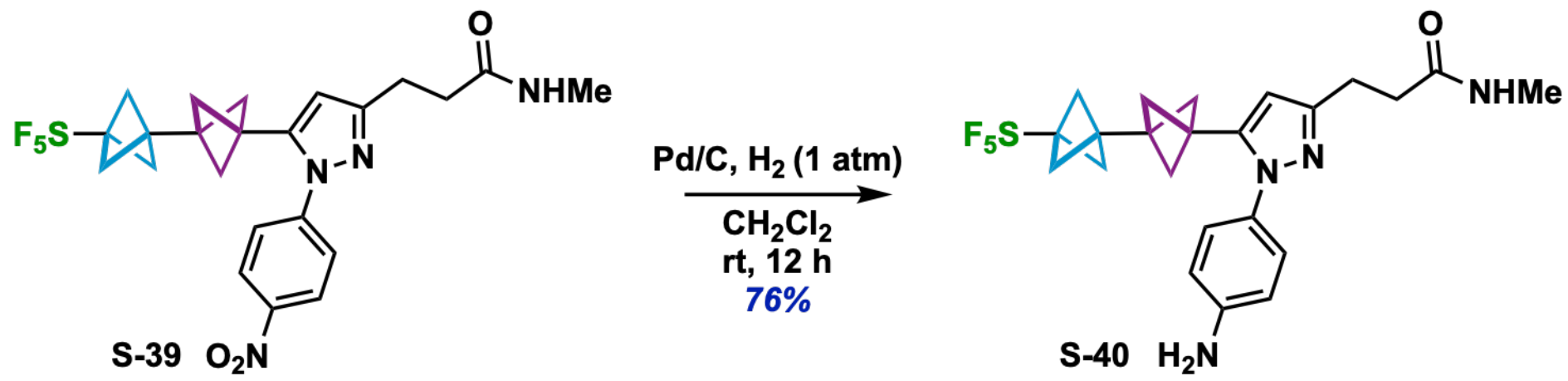

The synthesis of compound **S-40** was adapted from a known literature procedure.[10] To an oven-dried microwave vial equipped with a stir bar was added compound **S-39** (10.0 mg, 0.019 mmol, 1.0 equiv.) dissolved in CH_2Cl_2 (1.0 mL), and 10% wt. Pd/C (1.00 mg, 0.002 mmol, 0.1 equiv.) sequentially. The flask was sealed with a septum, purged three times with a H_2 balloon, and stirred for 12 h under H_2 atmosphere. Upon reaction completion as monitored by TLC, the reaction mixture was diluted with CH_2Cl_2 (2 mL), filtered through Celite, and concentrated and purified via preparatory TLC eluting with 19:1 CH_2Cl_2:MeOH to afford a white solid (7.30 mg, 76% yield).

3-(1-(4-aminophenyl)-5-(3'-(pentafluoro-λ^6-sulfaneyl)-[1,1'-bi(bicyclo[1.1.1]pentan)]-3-yl)-1*H*-pyrazol-3-yl)-*N*-methylpropanamide (**S-40**):

R_f: 0.25 (19:1 CH_2Cl_2:MeOH), vis: UV (254 nm).

m.p.: decomposes above 140.0 ºC.

^{1}H (300 MHz, $CDCl_3$): δ = 7.12-7.07 (m, 2H), 6.68-6.63 (m, 2H), 5.94 (s, 1H), 5.55-5.50 (m, 1H), 3.82 (bs, 2H), 2.89-2.83 (m, 2H), 2.75-2.73 (d, 3H, $^3J_{H-H}$= 4.68 Hz), 2.36-2.31 (m, 2H), 2.20 (s, 6H), 1.96 (s, 6H).

^{19}F (282 MHz, $CDCl_3$): δ = 80.71-78.65 (p, 1F, $^2J_{F-F}$ = 145.6 Hz), 48.38-47.87 (d, 4F, $^2J_{F-F}$ = 145.6 Hz).

^{13}C{^{1}H} (151 MHz, $CDCl_3$): δ = 171.9, 151.2, 146.6, 143.2, 130.4, 127.1, 115.1, 102.7, 66.6-66.3 (m), 54.41, 51.8, 37.7, 35.8, 35.2, 35.1, 26.4, 22.1.

ATR-IR (ν_{max}): 3424, 3335, 3281, 2972, 2927, 2870, 1643, 1608, 1561, 1519, 1286, 1212, 901, 820, 791, 783, 736, 705, 644, 614 cm^{-1}.

HRMS (*m/z*): (ESI) calc'd for $C_{23}H_{28}F_5N_4OS^+$ $[M-H]^+$: calculated 503.1898, found 503.1890.

Compound 33

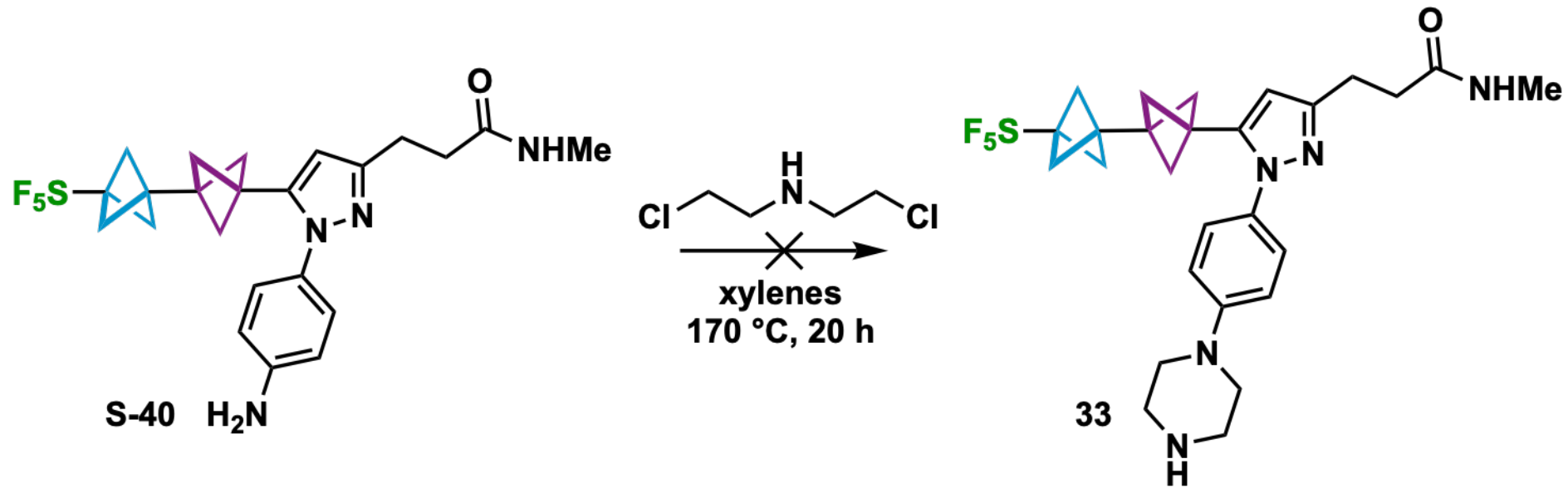

The synthesis of compound **33** was adapted from a known literature procedure.[10] To an oven-dried microwave vial equipped with a stir bar was added compound **S-40** (20.0 mg, 0.043 mmol, 1.0 equiv.). The vial was sealed with a cap with a septum using a crimper, evacuated, and backfilled with Ar three times. Then, dry xylenes (0.5 mL) was added followed by bis(2-chloroethyl)amine hydrochloride (10.0 mg, 0.052 mmol, 1.2 equiv.). The resulting mixture was heated to 170 °C in an oil bath for 20 h. Subsequently, the reaction mixture was cooled to room temperature and concentrated.

Additional Attempted Transformations Toward Remote Arene Functionalization

Compound S-41

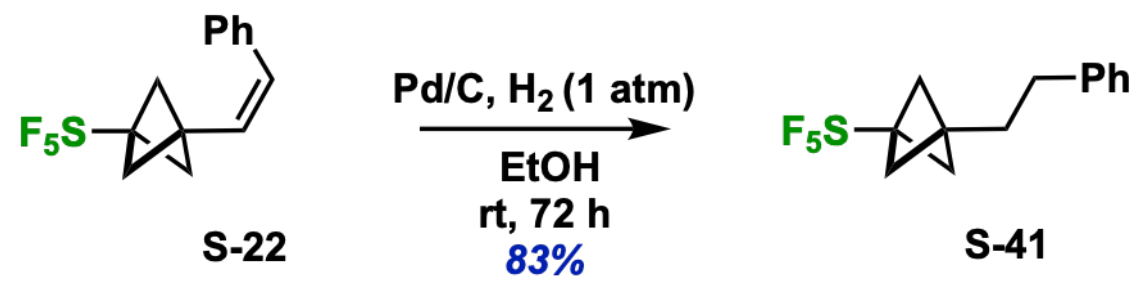

To an oven-dried microwave vial equipped with a stir bar was added compound **S-22** (0.220 g, 0.743 mmol, 1.0 equiv.). Then, EtOH (4.5 mL) was added followed by 10% wt. Pd/C (71.0 mg, 0.067 mmol, 0.09 equiv.). The vial was then sealed with a septum and purged with a H_2 balloon three times. The reaction mixture was then sparged with H_2 gas for 5 min. Subsequently, the reaction mixture was stirred at room temperature for 72 h under a H_2 gas atmosphere. Upon reaction completion, the reaction was filtered through Celite and concentrated. A light-yellow oil was obtained (0.184 g, 83 %), which required no further purification.

pentafluoro(3-phenethylbicyclo[1.1.1]pentan-1-yl)- λ^6-sulfane (**S-41**):
R_f: 0.34 (hexanes), vis: UV (254 nm).
^{1}H (300 MHz, $CDCl_3$): δ = 7.33-7.28 (m, 2H), 7.24-7.17 (m, 3H,), 2.66-2.60 (m, 2H), 2.17 (s, 6H), 1.98-1.93 (m, 2H).
^{19}F (283 MHz, $CDCl_3$): δ = 80.89-78.83 (p, 1F, $^2J_{F\text{-}F}$ = 145.6 Hz), 48.49-47.97 (d, 4F, $^2J_{F\text{-}F}$ = 145.6 Hz).
^{13}C{^{1}H} (76 MHz, $CDCl_3$): δ = 141.2, 128.6, 128.3, 126.2, 67.0-66.6 (m), 55.2-55.2 (m), 35.5, 33.0, 30.9-30.9 (m).
ATR-IR (ν_{max}): 2925, 2360, 2341, 823, 797 cm^{-1}.
HRMS (*m/z*): (ESI) calc'd for $C_{13}H_{16}F_5S^+$ [M-H]$^+$: calculated 299.0887, found 299.0880.

Compound S-42

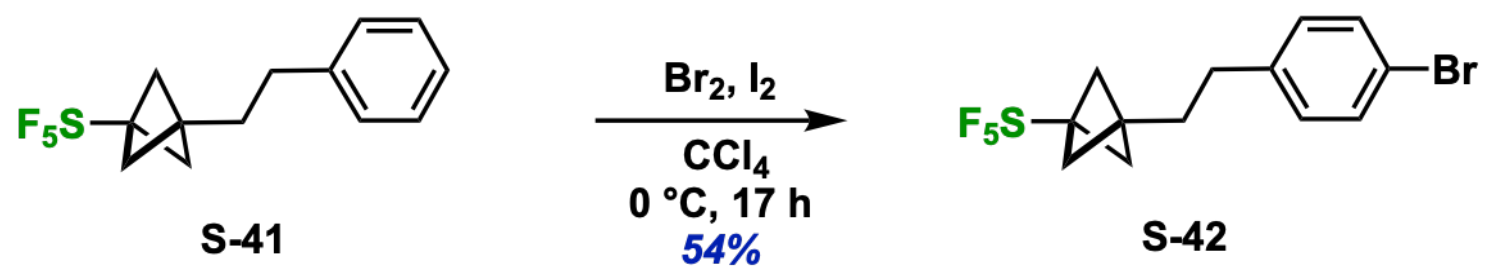

The synthesis of compound **S-42** was adapted from a known literature procedure.[46] To an oven-dried microwave vial equipped with a stir bar was added compound **S-41** (20.0 mg, 0.067 mmol, 1.0 equiv.)**.** The vial was sealed with a septum, evacuated, and backfilled with Ar three times. Then, carbontetrachloride (0.25 mL) was added *via* syringe and the reaction mixture was cooled to 0 °C by using an ice-water bath. Once cooled, I_2 (4.00 mg, 0.010 mmol, 0.24 equiv.) was added followed by the dropwise addition of Br_2 (0.001 mL, 0.20 mmol, 3.0 equiv.). The reaction mixture was then stirred at room temperature for 17 h. The reaction was quenched with aq. $Na_2S_2O_3$ (1 mL) and extracted with Et_2O (3 x 5 mL). The combined organic layers were washed with brine (3 x 10 mL), dried over anhydrous Na_2SO_4, filtered through Celite, and concentrated. The crude solid was purified *via* preparatory TLC eluting with 100% hexanes to afford a white solid (13.6 mg, 54 %). *(Note: The ortho regioisomer was observed by ^{1}H NMR but not characterized.)*

(3-(4-bromophenethyl)bicyclo[1.1.1]pentan-1-yl)pentafluoro-λ^6-sulfane (**S-42**):

R_f: 0.26 (hexanes), vis: UV (254 nm).

m.p.: 86.6–89.8ºC.

^{1}H (300 MHz, $CDCl_3$): δ = 7.42-7.39 (m, 2H), 7.06-7.03 (m, 2H,), 2.59-2.56 (m, 2H), 2.16 (s, 6H), 1.94-1.89 (m, 2H).

^{19}F (283 MHz, $CDCl_3$): δ = 80.72-78.66 (p, 1F, $^2J_{F\text{-}F}$ = 145.6 Hz), 48.50-47.99 (d, 4F, $^2J_{F\text{-}F}$ = 145.6 Hz).

^{13}C{^{1}H} (101 MHz, $CDCl_3$): δ = 140.1, 131.6, 130.0, 120.0, 66.8-66.2 (m), 55.2-55.1 (m), 35.4, 32.4, 30.8.

ATR-IR (ν_{max}): 2926, 2359, 1490, 1186, 811 cm^{-1}.

HRMS (*m/z*): (ESI) calc'd for $C_{13}H_{15}BrF_5S^+$ [M-H]$^+$: calculated 376.9993, found 377.0010.

Compound S-43

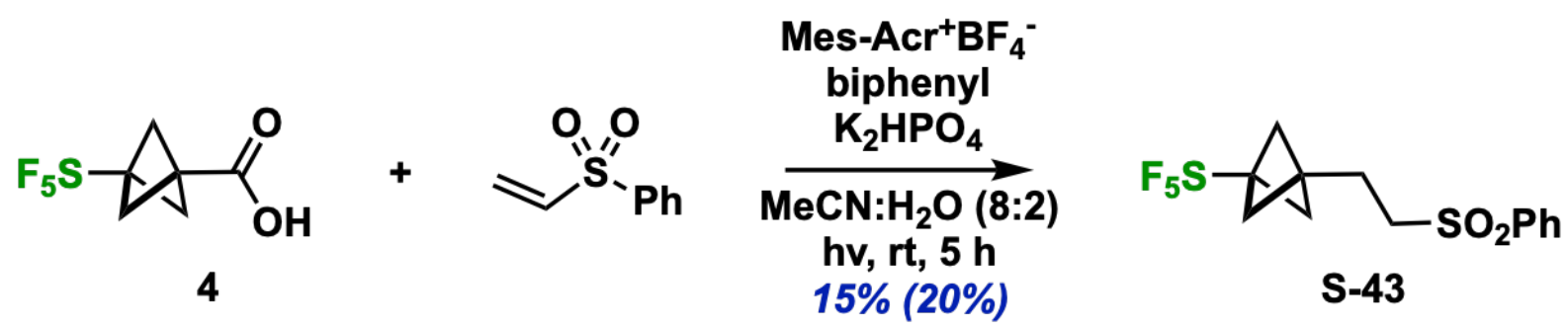

The synthesis of compound **S-43** was adapted from a known literature procedure.[47] To an oven dried microwave vial equipped with a stir bar was added compound **4** (20 mg, 0.084 mmol, 1.0 equiv.), phenyl vinyl sulfone (18.4 mg, 0.109 mmol, 1.3 equiv.), 9-mesityl-3,6-di-*tert*-butyl-10-phenylacridinium tetrafluoroborate (Mes-Acr$^+$$BF_4^-$) (1.0 mg, 0.002 mmol, 0.02 equiv.), biphenyl (0.4 mg, 0.003 mmol, 0.03 equiv.), and K_2HPO_4 (1.5 mg, 0.008 mmol, 0.10 equiv.). The vial was then sealed with a cap with a septum using a crimper, evacuated, and backfilled with Ar three times. Anhydrous MeCN (0.67 mL) and H_2O (0.17 mL) were then sequentially added *via* syringe. The reaction mixture was sparged with Ar for 5 min. The reaction mixture was irradiated with blue LEDs (Kessil A160WE Tuna Blue, maximum intensity) for 5 h (the vial was placed ca. 5 cm away from the light source). Subsequently, the reaction mixture was diluted with EtOAc (1 mL) and H_2O (1 mL). The aqueous layer was extracted with EtOAc (3 x 10 mL). The combined organic layers were washed with brine (3 x 10 mL), dried over anhydrous Na_2SO_4, filtered through Celite, and concentrated. The crude reside was purified *via* preparatory TLC eluting with 4:1 Hexanes:EtOAc to afford a white solid (5.6 mg, 15%). *(Note: the ^{19}F NMR yield for **S-43** was 20%; the mixture contained 60% of unreacted **4**).*

pentafluoro(3-(2-(phenylsulfonyl)ethyl)bicyclo[1.1.1]pentan-1-yl)-λ^6-sulfane (**S-43**).
^{1}H (300 MHz, $CDCl_3$): δ = 7.95-7.92 (m, 2H), 7.74-7.58 (m, 3H,), 3.07-3.01 (m, 2H), 2.20 (s, 6H), 2.12-2.06 (m, 2H).
^{19}F (283 MHz, $CDCl_3$): δ = 80.07-78.00 (p, 1F, $^2J_{F\text{-}F}$ = 145.5 Hz), 48.86-48.34 (d, 4F, $^2J_{F\text{-}F}$ = 145.4 Hz).

*Spectral data for **S–43** are consistent with previous literature.[14] Both ^{1}H and ^{19}F NMR data are provided above for convenience.*

Compound S-44

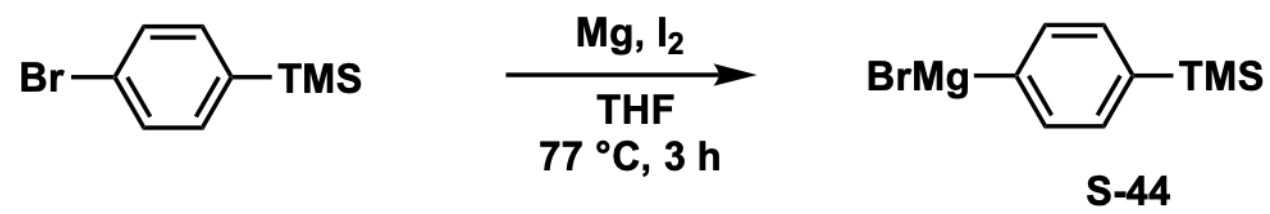

The synthesis of compound **S-44** was adapted from a known literature procedure.[46] An oven-dried three round bottom equipped with a stir bar was charged with magnesium turnings (0.255 g, 10.47 mmol, 1.2 equiv.). The round bottom was heated with a heat gun for 2 min under vacuum. Once cooled to room temperature the round bottom was sealed with a septum, evacuated, and backfilled with Ar three times. Anhydrous THF (7.3 mL) was then added *via* syringe. One crystal of I_2 (25.7 mg, 5.240 mmol, 1.2 equiv.) was added followed by (4-(trimethylsilyl)phenyl)magnesium bromide (1.00 g, 4.26 mmol, 1.0 equiv.). The reaction mixture was refluxed for 3 h at 77 °C in an oil bath. The reaction mixture was then transferred to an oven-dried schlenk flask under argon. The concentration of the grignard reagent was determined by iodometric titration (0.84 M, 7.5 mL).

Iodometric Titration Procedure:

Saturated solution of LiCl in THF:
Anhydrous LiCl (264.9 mg, 6.249 mmol) was placed to an oven-dried 20 mL microwave vial equipped with a stir bar. The vial was sealed with a cap with a septum using a crimper, evacuated, and backfilled with Ar three times. The vial was dried under vacuum for 4 h at 140 °C. After cooling to room temperature, anhydrous THF (12.5 mL) was added *via* syringe. The mixture was stirred at room temperature for 24 h or until the LiCl was completely dissolved, resulting in the formation of a 0.5 M solution of LiCl.

Titration using I_2:

An oven-dried microwave vial equipped with a stir bar was added I_2 (25.7 mg, 0.101 mmol). The vial was sealed with a septum, evacuated, and backfilled with Ar three times. The sat. solution of LiCl in THF (1.0 mL) was added *via* syringe. After the iodine was completely dissolved, the resulting brown solution was cooled to 0 °C in an ice-water bath and the organometallic reagent was added dropwise *via* a syringe (in 0.01 mL graduations) until the brown color disappeared. The amount consumed contains 1 equiv. of the organometallic reagent relative to iodine in the case of monoorganometallic reagents and 0.5 equiv. for diorganometallic reagents.

Compound S-45

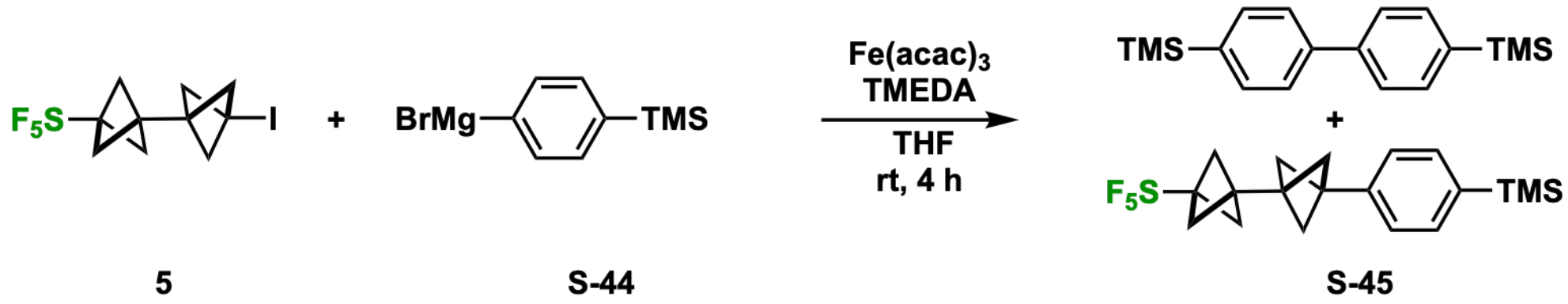

The synthesis of compound **S-45** was adapted from a known literature procedure.[46] To an oven-dried microwave vial equipped with a stir bar were added compound **5** (0.100 g, 0.259 mmol, 1.0 equiv.) and $Fe(acac)_3$ (18.3 mg, 0.052 mmol, 0.2 equiv.). The vial was sealed with a septum, evacuated, and backfilled with Ar three times. Anhydrous THF (0.26 mL) was added *via* syringe followed by freshly distilled TMEDA (0.012 mL, 0.104 mmol, 0.4 equiv.). The reaction mixture was allowed to stir for 5 min. Then, compound **S-44** (0.44 M, 1.00 mL) was diluted to 0.147 M with anhydrous THF (2.0 mL) and added to the reaction mixture dropwise via a syringe pump over 1 h. After addition, the reaction mixture was stirred at room temperature for 5 h. Upon completion, the reaction was quenched with 1 M HCl (1 mL) and sat. aq. NH_4Cl (5 mL). The aqueous layer was extracted with Et_2O (3 x 10 mL). The combined organic layers were washed with brine (3 x 10 mL), dried over anhydrous Na_2SO_4, filtered through Celite, and concentrated. The crude residue was purified *via* column chromatography on silica gel eluting with 100% Hexane to afford a white solid, which was an inseparable mixture of the desired material and homo-coupled product.

trimethyl(4-(3'-(pentafluoro-λ^6-sulfanyl)-[1,1'-bi(bicyclo[1.1.1]pentan)]-3-yl)phenyl)silane (**S-45**):
^{1}H (300 MHz, $CDCl_3$): δ = 7.49-7.46 (m, 2H), 7.21-7.18 (m, 2H), 2.22 (s, 6H), 1.94 (s, 6H), 0.30 (s, 9H).
^{19}F (283 MHz, $CDCl_3$): δ = 80.64-78.58 (p, 1F, $^2J_{F\text{-}F}$ = 145.0 Hz), 48.37-47.86 (d, 4F, $^2J_{F\text{-}F}$ = 145.6 Hz).

Compound S-46

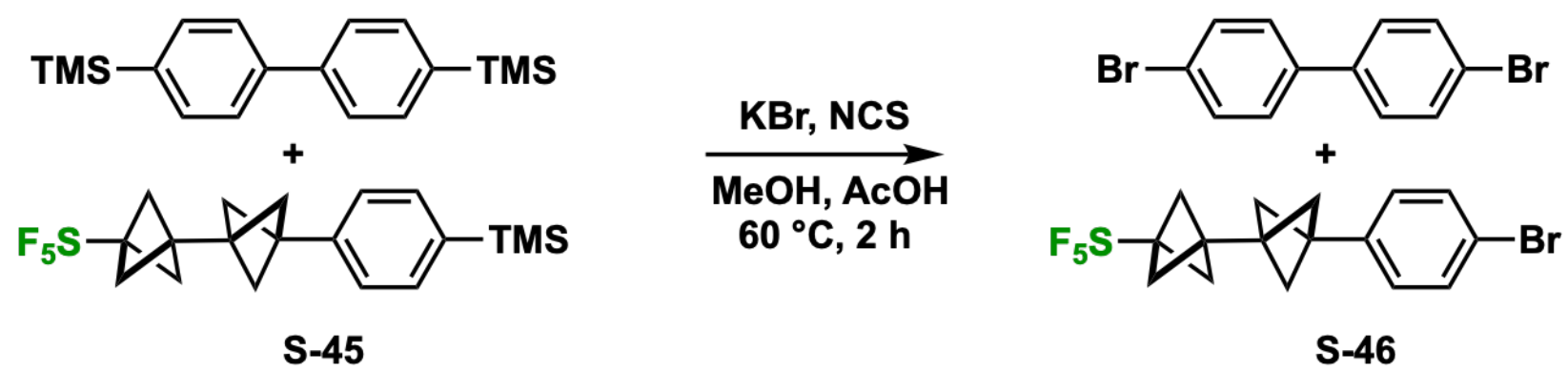

The synthesis of compound **S-46** was adapted from a known literature procedure.[46] To an oven-dried microwave vial equipped with a stir bar were added compound **S-45** (0.163 g, 0.399 mmol, 1.0 equiv.) and KBr (0.143 g, 1.199 mmol, 3.0 equiv.). The round bottom was sealed with a cap with a septum using a crimper, evacuated, and backfilled with Ar three times. Then, anhydrous MeOH (0.3 mL) and acetic acid (2.7 mL) were added *via* syringe and heated to 60 °C for 20 min. Then, NCS (0.128 g, 1.20 mmol, 3.0 equiv.) was added and the reaction mixture was then heated for an additional 2 h. Once cooled to room temperature, the reaction was quenched with H_2O (2 mL). To the crude mixture was added 1 M aq. NaOH (10 mL) and the aq. phase was then extracted with CH_2Cl_2 (3 x 10 mL). The combined organic layers were washed with brine (3 x 4 mL), dried over anhydrous Na_2SO_4, filtered through Celite, and concentrated. The crude solid was purified *via* column chromatography on silica gel eluting with 100% pentane to afford a white solid, which was an inseperable mixture of desired material and homo-coupled product.

(3'-(4-bromophenyl)-[1,1'-bi(bicyclo[1.1.1]pentan)]-3-yl)pentafluoro-λ^6-sulfane (**S-46**):
^{1}H (300 MHz, $CDCl_3$): δ = 7.07-7.04 (m, 2H), 2.22 (s, 6H), 1.93 (s, 6H).
^{19}F (283 MHz, $CDCl_3$): δ = 80.64-78.58 (p, 1F, $^2J_{F\text{-}F}$ = 145.3 Hz), 48.37-47.86 (d, 4F, $^2J_{F\text{-}F}$ = 145.3 Hz).

Compound S-47

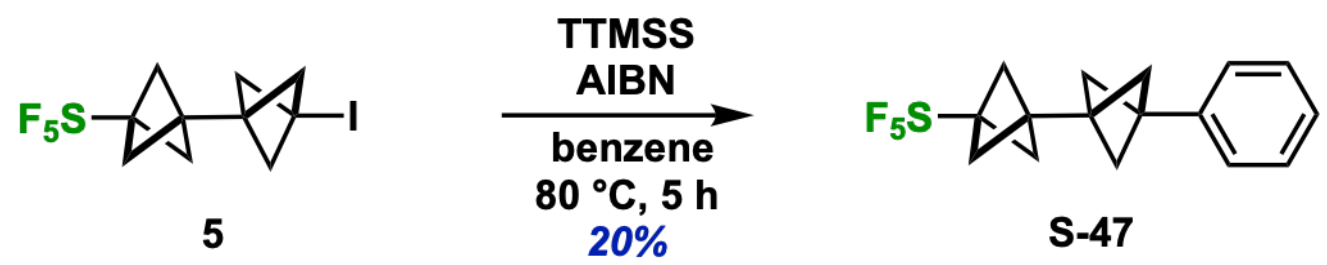

The synthesis of compound **S-47** was adapted from a known literature procedure.[48] To an oven-dried microwave vial equipped with a stir bar were added compound **5** (30.0 mg, 0.078 mmol, 1.0 equiv.) and AIBN (16.2 mg, 0.078 mmol, 1.1 equiv.). The vial was then sealed with a cap with a septum using a crimper. Anhydrous benzene (1.2 mL) and TTMSS (0.02 mL, 0.078 mmol, 1.0 equiv.) were then added sequentially *via* syringe. The reaction mixture was sparged with air for 2 min and heated to 80 °C in an oil bath for 5 h. The crude reaction mixture was concentrated and was purified *via* preparative TLC eluting with 100% hexanes to afford a white solid (5.1 mg, 20%).

pentafluoro(3'-phenyl-[1,1'-bi(bicyclo[1.1.1]pentan)]-3-yl)- λ^6-sulfane (**S-47**):

R_f: 0.37 (hexanes), vis: *p*-anisaldehyde.

m.p.: decomposes above 168.1 ºC.

^{1}H (399 MHz, $CDCl_3$): δ = 7.32-7.28 (m, 2H), 7.24-7.18 (m, 3H,), 2.22 (s, 6H), 1.94 (s, 6H).

^{19}F (376 MHz, $CDCl_3$): δ = 80.38-78.84 (p, 1F, $^2J_{F\text{-}F}$ = 145.5 Hz), 48.30-47.92 (d, 4F, $^2J_{F\text{-}F}$ = 145.5 Hz).

^{13}C{^{1}H} (76 MHz, $CDCl_3$): δ = 140.61, 128.34, 126.74, 126.11, 54.47, 51.52, 41.34, 36.44, 35.20.

ATR-IR (ν_{max}): 2963, 2924, 2874, 2850, 1446, 899 cm^{-1}.

HRMS (*m/z*): (ESI) calc'd for $C_{13}H_{16}F_5S^+$ [M-H]$^+$: not observed.

Compound S-48

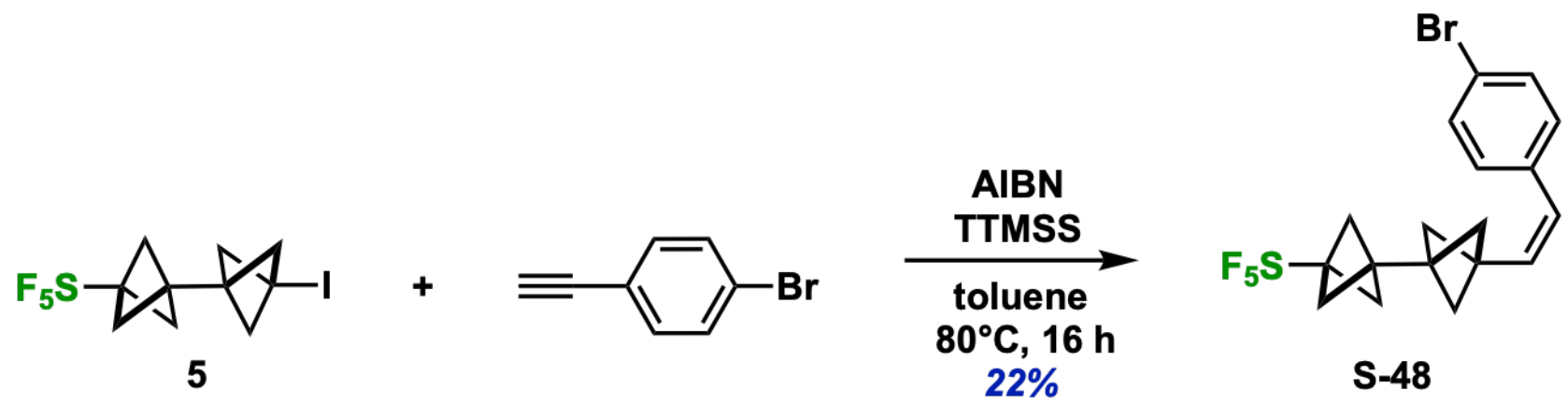

To an oven-dried microwave vial equipped with a stir bar was added compound **5** (0.03 g, 0.078 mmol, 1.0 equiv.) and AIBN (6.4 mg, 0.039 mmol, 0.5 equiv.). The vial was then sealed with a cap with a septum using a crimper, evacuated, and backfilled with Ar three times. Anhydrous toluene (0.5 mL) and TTMSS (0.04 mL, 0.117 mmol, 1.5 equiv.) were then added sequentially *via* syringe. The reaction mixture was sparged with Ar for 2 min. and stirred at 80 °C in an oil bath for 16 h. The crude reaction mixture was concentrated and filtered through silica gel, eluting with hexanes. The filtrate was concentrated and purified *via* preparatory TLC eluting with 100% hexanes to afford a light yellow solid (7.0 mg, 22%). *(Note: only the Z isomer was obtained.)*

(*Z*)-(3'-(4-bromostyryl)-[1,1'-bi(bicyclo[1.1.1]pentan)]-3-yl)pentafluoro-λ^6-sulfane (**S-48**).
R_f: 0.46 (hexanes), vis: *p*-anisaldehyde.
^{1}H (300 MHz, $CDCl_3$): δ = 7.44-7.41 (m, 2H), 7.06-7.03 (m, 2H), 6.44-6.40 (m, 2H), 5.69-5.65 (m, 2H), 2.10 (s, 6H), 1.64 (s, 6H).
^{19}F (283 MHz, $CDCl_3$): δ = 80.62-78.56 (p, 1F, $^2J_{F-F}$ = 145.2 Hz), 48.30-47.78 (d, 4F, $^2J_{F-F}$ = 145.2 Hz).
^{13}C{^{1}H} (201 MHz, $CDCl_3$): δ = 136.23, 131.44, 130.89, 130.83, 130.13, 120.87, 54.36, 52.83, 38.70, 38.24, 34.95.
ATR-IR (ν_{max}): 3002, 2967, 2925, 2873, 1486, 1213, 831.
HRMS (*m/z*): (ESI) calc'd for $C_{18}H_{17}BrF_5S^-$ [M-H]$^-$: calculated 439.0160, found 439.0169.

Compound S-49

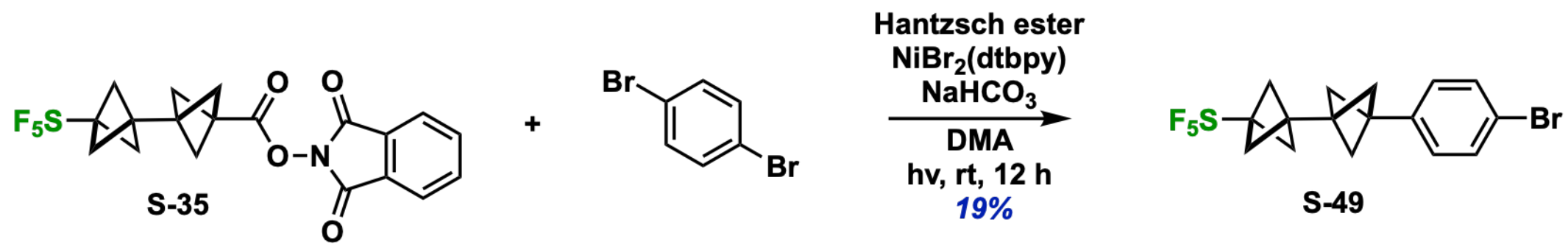

The synthesis of compound **S-49** was adapted from a known literature procedure.[49] To an oven-dried microwave vial equipped with a stir bar was added compound **S-35** (20.0 mg, 0.047 mmol, 1.5 equiv.), 1,4-dibromobenzene (7.60 mg, 0.030 mmol, 1.0 equiv.), Hantzsch ester (15.0 mg, 0.059 mmol, 2.0 equiv.), $NiBr_2$(dtbpy) (2.9 0mg, 0.006 mmol, 0.20 equiv.), and $NaHCO_3$ (9.90 mg, 0.119 mmol, 4.0 equiv.). The vial was sealed with a septum, evacuated, and backfilled with argon three times. Anhydrous DMA (0.31 mL) was added *via* syringe and sparged for 2 min with Ar. The reaction mixture was then irradiated with purple light for 12 h. The reaction was quenched with Et_2O (1.0 mL). The organic layer was extracted with 1 M HCl (3 x 10 mL) and CH_2Cl_2 (3 x 10 mL). The combined organic layers were washed with brine (3 x 4 mL), dried over anhydrous Na_2SO_4, filtered through Celite, and concentrated. The crude solid was purified *via* preparatory TLC eluting with 100% hexanes to afford a white solid (2.30 mg, 19%).

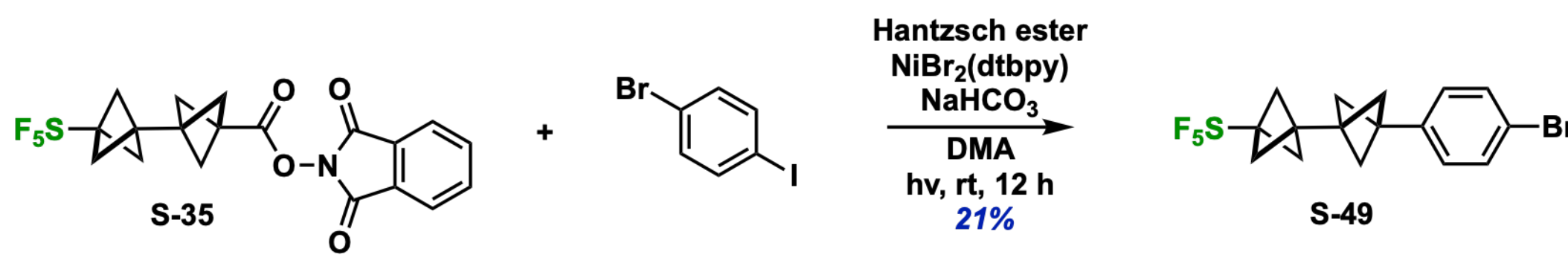

The synthesis of compound **S-49** was adapted from a known literature procedure.[49] To an oven-dried microwave vial equipped with a stir bar was added compound **S-35** (20.0 mg, 0.047 mmol, 1.5 equiv.), 1-bromo-4-iodobenzene (8.40 mg, 0.030 mmol, 1.0 equiv.), Hantzsch ester (15.0 mg, 0.059 mmol, 2.0 equiv.), $NiBr_2$(dtbpy) (2.90 mg, 0.006 mmol, 0.20 equiv.), and $NaHCO_3$ (9.90 mg, 0.119 mmol, 4.0 equiv.). The vial was sealed with a septum, evacuated, and backfilled with Ar three times. Anhydrous DMA (0.31 mL) was added *via* syringe and sparged for 2 min with Ar. The reaction mixture was then irradiated with purple light for 12 h. The reaction was quenched with Et_2O (1.0 mL). The crude reaction mixture was extracted with 1 M HCl (3 x 10 mL) and CH_2Cl_2 (3 x 10 mL). The combined organic layers were washed with brine (3 x 4 mL), dried over anhydrous Na_2SO_4, filtered through Celite, and concentrated. The crude solid was purified *via* preparatory TLC eluting with 100% hexane to afford a white solid (2.60 mg, 21%).

(3'-(4-bromophenyl)-[1,1'-bi(bicyclo[1.1.1]pentan)]-3-yl)pentafluoro-λ^6-sulfane (**S-49**):
^{1}H (300 MHz, $CDCl_3$): δ = 7.42-7.40 (m, 2H), 7.06-7.03 (m, 2H,), 2.21 (s, 6H), 1.92 (s, 6H).
^{19}F (283 MHz, $CDCl_3$): δ = 80.56-78.50 (p, 1F, $^2J_{F\text{-}F}$ = 145.1 Hz), 48.39-47.88 (d, 4F, $^2J_{F\text{-}F}$ = 145.1 Hz).

Compound S-50

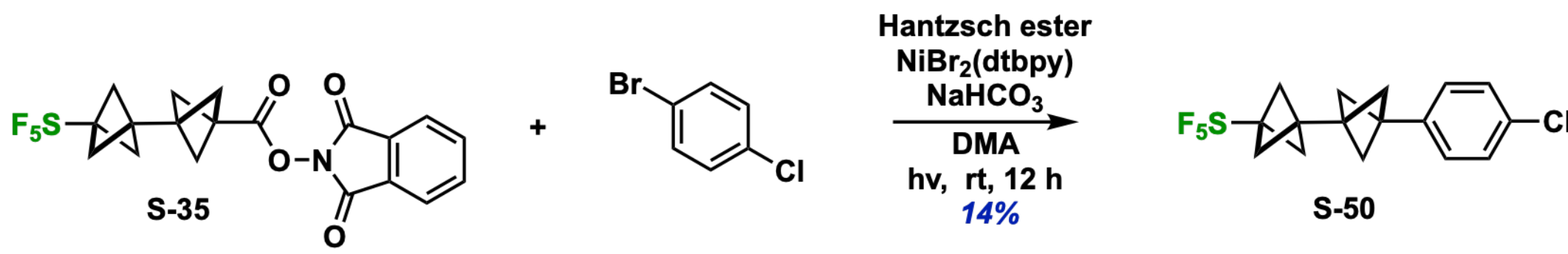

The synthesis of compound **S-50** was adapted from a known literature procedure.[49] To an oven-dried microwave vial equipped with a stir bar were added compound **S-35** (20.0 mg, 0.047 mmol, 1.5 equiv.), 4-chlorobromobenzene (8.40 mg, 0.030 mmol, 1.0 equiv.), Hantzsch ester (15.0 mg, 0.059 mmol, 2.0 equiv.), $NiBr_2$(dtbpy) (2.90 mg, 0.006 mmol, 0.2 equiv.), and $NaHCO_3$ (9.90 mg, 0.119 mmol, 4.0 equiv.). The vial was sealed with a septum, evacuated, and backfilled with Ar three times. Anhydrous DMA (0.31 mL) was added *via* syringe and sparged for 2 min with Ar. The reaction mixture was irradiated with purple light for 12 h. The reaction mixture was quenched with Et_2O (1.0 mL). The crude reaction mixture was extracted with 1 M HCl (3 x 10 mL) and CH_2Cl_2 (3 x 10 mL). The combined organic layers were washed with brine (3 x 4 mL), dried over anhydrous Na_2SO_4, filtered through Celite, and concentrated. The crude solid was purified *via* preparatory TLC eluting with 100% hexane to afford a white solid (2.50 mg, 14%).

(3'-(4-chlorophenyl)-[1,1'-bi(bicyclo[1.1.1]pentan)]-3-yl)pentafluoro-λ^6-sulfane (**S-50**)
^{1}H (300 MHz, $CDCl_3$): δ = 7.27-7.24 (m, 2H), 7.12-7.09 (m, 2H,), 2.21 (s, 6H), 1.92 (s, 6H),
^{19}F (283 MHz, $CDCl_3$): δ = 80.57-78.51 (p, 1F, $^2J_{F-F}$ = 145.2 Hz), 48.39-47.88 (d, 4F, $^2J_{F-F}$ = 145.2 Hz).

Compound S-51

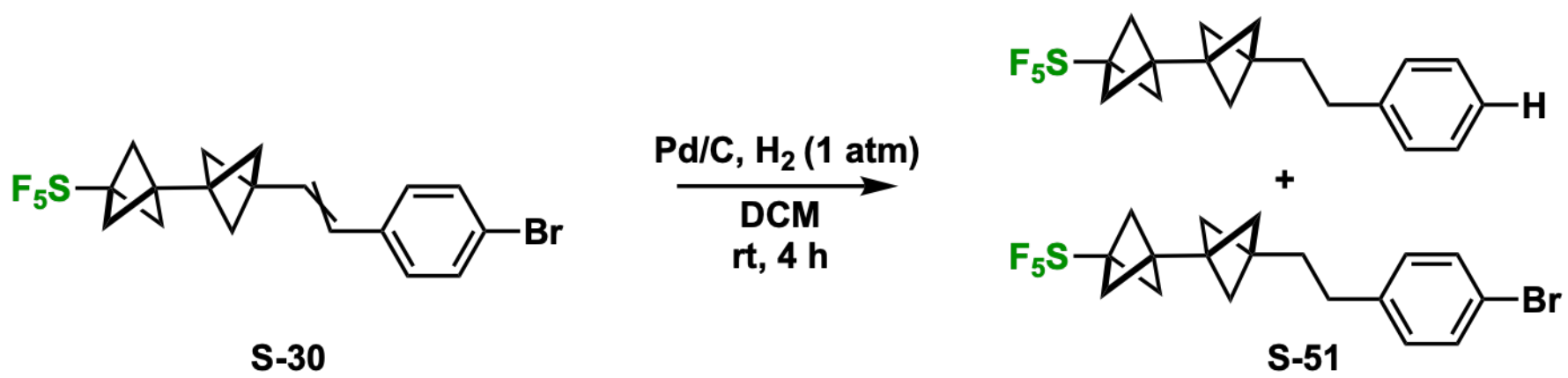

S-30 S-51

To an oven-dried microwave vial equipped with a stir bar was charged with compound **S-30** (20.0 mg, 0.045 mmol, 1.0 equiv.), dissolved in CH_2Cl_2 (1.0 mL) and followed by 10% wt. Pd/C (4.00 mg). The flask was sealed with a septum and purged three times with a H_2 balloon and stirred for 4 h under H_2 atmosphere. Upon reaction completion as monitored by TLC, the reaction was diluted with CH_2Cl_2 (2.0 mL), filtered through Celite, and concentrated to obtain compound **S-51** as a white solid. (*Note: The isolated material was an inseparable mixture of desired product and hydrodehalogenated product).*

Compound S-52

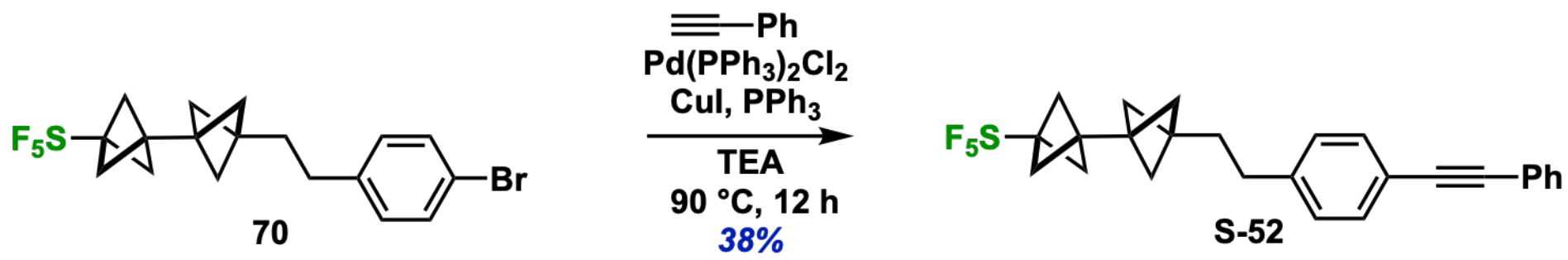

The synthesis of compound **S-52** was adapted from a known literature procedure.[40] To an oven-dried microwave vial equipped with a stir bar was added compound **70** (50.0 mg, 0.113 mmol, 1.0 equiv.), $Pd(PPh_3)_2Cl_2$ (3.90 mg, 0.006 mmol, 0.05 equiv.), CuI (1.20 mg, 0.006 mmol, 0.06 equiv.), and PPh_3 (3.00 mg, 0.011 mmol, 0.1 equiv.). The vial was sealed with a cap with a septum using a crimper, evacuated, and backfilled with Ar three times. Anhydrous TEA (0.3 mL) and phenyl acetylene (0.020 mL, 0.135 mmol, 1.2 equiv.) were added *via* syringe. The reaction mixture was sparged with an Ar balloon for 2 mins and then heated to 90 °C in an oil bath for 20 h. The reaction mixture was diluted with EtOAc and filtered through Celite and concentrated. The crude residue was purified *via* column chromatography on silica gel eluting with 100% hexanes to afford a white solid, followed by recrystallization in EtOAc or precipitation in hexanes to afford a white solid (19.8 mg, 38%).

pentafluoro(3'-(4-(phenylethynyl)phenethyl)-[1,1'-bi(bicyclo[1.1.1]pentan)]-3-yl)-λ^6-sulfane (**S-52**)
R_f: 0.13 (hexanes), vis: *p*-anisaldehyde.

m.p.: decomposes above 175.6 ºC.

^{1}H (300 MHz, $CDCl_3$): δ = 7.54-7.51 (m, 2H), 7.46-7.43 (m, 2H), 7.36-7.33 (m, 3H), 7.17-7.14 (m, 2H), 2.60-2.55 (m, 2H), 2.14 (s, 6H), 1.81-1.76 (m, 2H), 1.51 (s, 6H).
^{19}F (283 MHz, $CDCl_3$): δ = 80.77-78.72 (p, 1F, $^2J_{F-F}$ = 145.4 Hz), 48.31-47.80 (d, 4F, $^2J_{F-F}$ = 145.4 Hz).
^{13}C{^{1}H} (101 MHz, $CDCl_3$): δ = 131.7, 128.4, 128.4, 128.2, 123.5, 120.7, 89.6, 89.0, 66.7-66.5 (m), 54.3, 49.6, 39.3, 37.2, 35.1, 33.0, 32.9.

ATR-IR (ν_{max}): 2966, 2929, 2904, 2867, 1209, 836, 818 cm^{-1}.

HRMS (*m/z*): (ESI) calc'd for $C_{26}H_{26}F_5S^+$ $[M-H]^+$: calculated 465.1670, found 465.1680.

Compound S-53

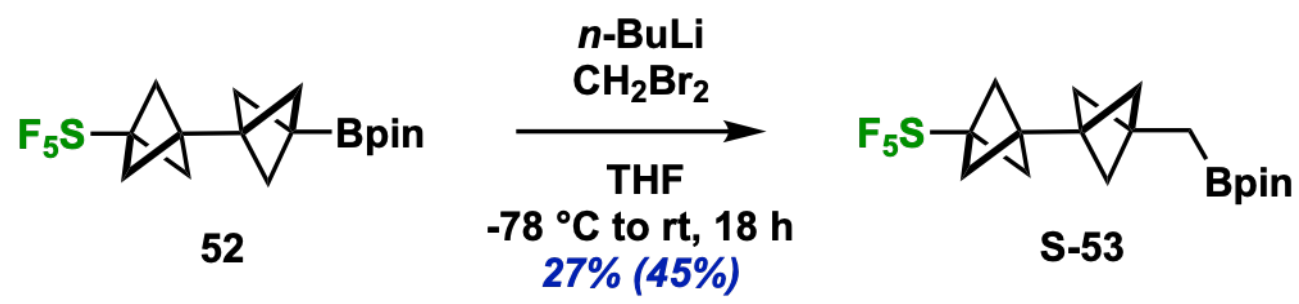

The synthesis of compound **S-53** was adapted from a known literature procedure.[50] To an oven-dried microwave vial equipped with a stir bar was added compound **52** (62 mg, 0.160 mmol, 1.0 equiv.). The vial was sealed with a septum then evacuated and backfilled with Ar three times. Anhydrous THF (2.0 mL) and dibromomethane (0.03 mL, 0.400 mmol, 2.5 equiv.) were added sequentially *via* syringe. The resulting mixture was cooled to –78 °C. To the cooled reaction mixture, 2.5 M solution of *n*-BuLi in hexanes (0.14 mL, 0.224 mmol, 1.4 equiv.) was added dropwise *via* syringe. The reaction mixture was stirred for 30 min at –78 °C, then warmed to rt and stirred for an additional 18 h. Subsequently, the reaction was quenched with sat. aq. NH_4Cl (3 mL). The aqueous layer was extracted with Et_2O (3 x 10 mL). The combined organic layers were washed with brine (3 x 10 mL), dried over anhydrous Na_2SO_4, filtered through Celite, and concentrated. The crude residue was quickly purified by column chromatography on silica gel eluting with 99:1 hexanes:EtOAc to afford a white solid (17 mg, 27%). *(Note: the material is prone to decomposition on silica gel, as confirmed by 2D TLC analysis. A ^{19}F NMR yield of 45% was obtained for compound **S-53**, prior to column chromatography.)*

4,4,5,5-tetramethyl-2-((3'-(pentafluoro-λ^6-sulfaneyl)-[1,1'-bi(bicyclo[1.1.1]pentan)]-3-yl)methyl)-1,3,2-dioxaborolane (**S-53**).
R_f: 0.25 (98:2 Hexanes:EtOAc), vis: *p*-anisaldehyde.
m.p.: 56.2-58.3 ºC.
^{1}H NMR (300 MHz, $CDCl_3$): δ = 2.12 (s, 6H), 1.56 (s, 6H), 1.24 (s, 12H), 1.05 (s, 2H).
^{19}F NMR (283 MHz, $CDCl_3$): δ = 81.24 – 77.47 (m, 1F), 48.00 (d, 4F, *J* = 145.2 Hz).
^{13}C{^{1}H} NMR (201 MHz, $CDCl_3$): δ = 83.19, 67.71 (m), 54.46, 51.66, 37.25, 36.47, 35.17, 25.04.
ATR-IR (ν_{max}): 2981, 2927, 2908, 2870, 1364, 1319, 1211, 1146, 901, 817, 787 cm^{-1}.
HRMS (*m/z*): (ESI) calc'd for $C_{17}H_{27}F_5BO_2S$ [M+H]$^+$: calculated 401.1739, found 401.1726.

Unsuccessful Transformations Regarding Synthesis of Building Blocks to Explore Remote Arene Functionalization

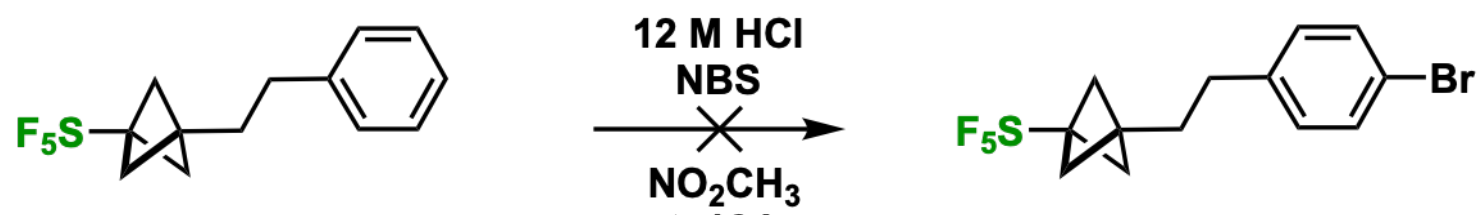

S-41 S-42

The synthesis of compound **S-42** was adapted from a known literature procedure.[51] To an oven-dried microwave vial equipped with a stir bar were added compound **S-41** (20.0 mg, 0.067 mmol, 1.0 equiv.) and NBS (13.2 mg, 0.002 mmol, 1.1 equiv.)**.** The vial was sealed with a septum, evacuated, and backfilled with Ar three times. Then, NO_2CH_3 (0.27 mL) and 12 M HCl (0.01 mL) were added *via* syringe. After addition, the reaction mixture was stirred at room temperature for 16 h. Upon reaction completion, the reaction was concentrated. The crude solid was extracted with CH_2Cl_2 (3 x 5 mL). The combined organic layers were washed with brine (3 x 10 mL), dried over anhydrous Na_2SO_4, filtered through Celite, and concentrated.

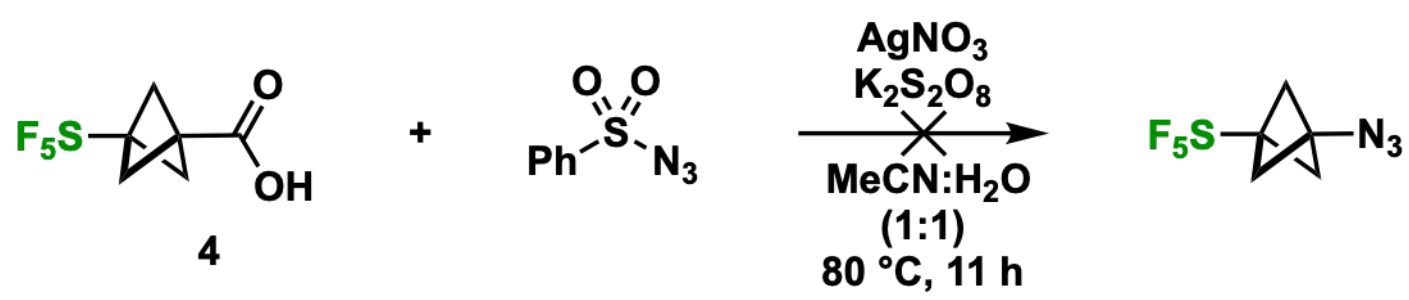

4

The synthesis was adapted from a known literature procedure.[52] To an oven-dried microwave vial equipped with a stir bar was added compound **4** (20 mg, 0.084 mmol, 1.0 equiv.), $AgNO_3$ (2.9 mg, 0.017 mmol, 0.2 equiv.), and $K_2S_2O_8$ (45 mg, 0.168 mmol, 2.0 equiv.). The vial was sealed with a cap with a septum using a crimper, evacuated, and backfilled with Ar three times. Then, MeCN (0.41 mL), H_2O (0.41 mL), and benzenesulfonyl azide (0.04 mL, 0.504 mmol, 6.0 equiv.) were added sequentially *via* syringe. The reaction mixture was sparged with an Ar balloon for 5 min and stirred at 80 °C in an oil bath for 11 h. Subsequently, the reaction mixture was cooled to room temperature and diluted with CH_2Cl_2 (1 mL) and H_2O (1 mL). The aqueous layer was extracted with CH_2Cl_2 (3 x 10 mL). The combined organic layers were washed with brine (3 x 10 mL), dried over anhydrous Na_2SO_4, filtered through Celite, and concentrated.

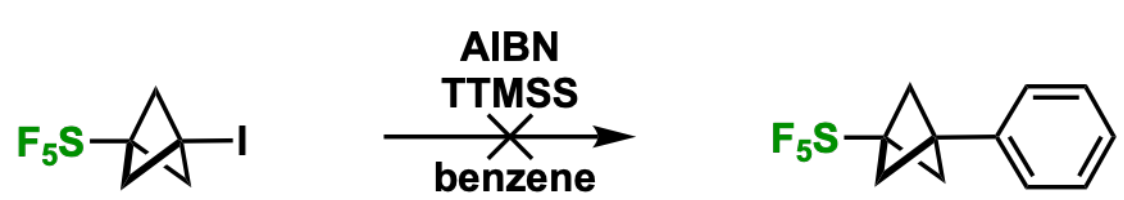

4

The synthesis was adapted from a known literature procedure.[48] To an oven-dried microwave vial equipped with a stir bar was added compound **4** (50 mg, 0.156 mmol, 1.0 equiv.) and AIBN (28.2 mg, 0.172 mmol, 1.1 equiv.). The vial was sealed with a cap with a septum using a crimper. Anhydrous benzene (1.9 mL) was added, followed by TTMSS (0.05 mL, 0.156 mmol, 1.0 equiv.). The reaction mixture was sparged with air and heated to 80 °C in an oil bath for 12 h. The crude reaction mixture was concentrated and filtered through silica gel eluting with hexanes (200 mL).

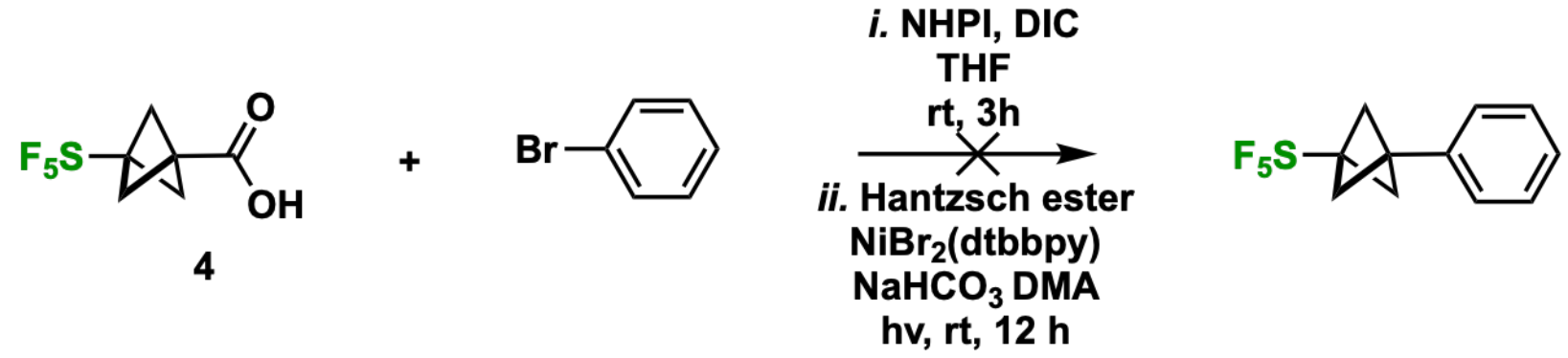

4

The synthesis was adapted from a known literature procedure.[47,53] To an oven-dried microwave vial equipped with a stir bar was added compound **4** (80 mg, 0.340 mmol, 1.0 equiv.) and NHPI (70.2 mg, 0.430 mmol, 1.25 equiv.). The vial was sealed with a cap with a septum using a crimper, then evacuated and backfilled with Ar three times. Then, anhydrous inhibitor-free THF (0.85 mL) and DIC (0.06 mL, 0.374 mmol, 1.1 equiv.) were added sequentially *via* syringe. The reaction mixture was stirred at room temperature for 3 h. Anhydrous DMA (2.6 mL) was then added *via* syringe. In a separate oven-dried microwave vial equipped with a stir bar was added Hantzsch ester (86.1 mg, 0.340 mmol, 2.0 equiv.), $NiBr_2$(dtbbpy) (16.6 mg, 0.034 mmol, 0.2 equiv.), and $NaHCO_3$ (57.1 mg, 0.680 mmol, 4.0 equiv.). The vial was sealed with a cap with a septum using a crimper, then evacuated and backfilled with Ar three times. The solution containing activated compound **4** (1.7 mL, 0.1 M) and phenyl bromide (0.016 mL, 0.150 mmol, 1.0 equiv.) was then added *via* syringe. The resulting reaction mixture was then sparged with Ar for 2 min. and irradiated with purple LEDs (Kessil H160 Tuna Flora, maximum intensity) for 12 h (the vial was placed ca. 5 cm away from the light source). Subsequently, the reaction mixture was diluted with EtOAc (5 mL) and H_2O (5 mL). The aqueous layer was extracted with EtOAc (3 x 10 mL). The combined organic layers were washed with brine (3 x 10 mL), dried over anhydrous Na_2SO_4, filtered through Celite, and concentrated. *(Note: An aliquot taken from the stock solution (0.1 M) containing the putative NHPI-based redox-active ester derived from **4** indicated it had formed in 66% yield by ^{19}F NMR; this was used without further purification due to observed instability toward isolation on silica and alumina – acidic, neutral, or basic).*

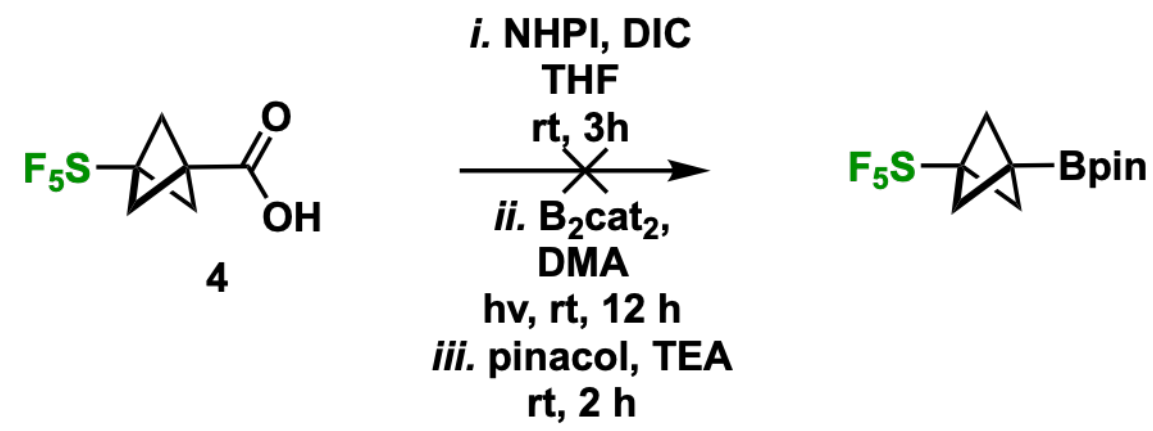

The synthesis was adapted from a known literature procedure.[53,54] To an oven-dried microwave vial equipped with a stir bar was added compound **4** (80 mg, 0.340 mmol, 1.0 equiv.) and NHPI (70.2 mg, 0.430 mmol, 1.25 equiv.). The vial was sealed with a cap with a septum using a crimper, then evacuated and backfilled with Ar three times. Then, anhydrous inhibitor-free THF (0.85 mL) and DIC (0.06 mL, 0.374 mmol, 1.1 equiv.) were added sequentially *via* syringe. The reaction mixture was stirred at room temperature for 3 h. Anhydrous DMA (2.6 mL) was then added *via* syringe. In a separate oven-dried microwave vial equipped with a stir bar was added B_2cat_2 (49.9 mg, 0.21 mmol, 1.25 equiv.). The vial was sealed with a cap with a septum using a crimper, evacuated and backfilled with Ar three times. The solution containing activated compound **4** (1.7 mL, 0.1 M) was then added *via* syringe. The resulting reaction mixture was then sparged with Ar for 2 min. and irradiated with blue LEDs (Kessil A160WE Tuna Blue, maximum intensity) for 12 h (the vial was placed ca. 5 cm away from the light source). After 12 h, pinacol (80.4 mg, 0.68 mmol, 4.0 equiv.) and TEA (0.59 mL) were added *via* syringe. The resulting mixture was sparged with Ar for 2 min, and stirred at room temperature for 2 h. Subsequently, the reaction mixture was diluted with EtOAc (5 mL) and H_2O (5 mL). The aqueous layer was extracted with EtOAc (3 x 10 mL). The combined organic layers were washed with brine (3 x 10 mL), dried over anhydrous Na_2SO_4, filtered through Celite, and concentrated. *(Note: An aliquot taken from the stock solution (0.1 M) containing the putative NHPI-based redox-active ester derived from* ***4*** *indicated it had formed in 66% yield by* ^{19}*F NMR; this was used without further purification due to observed instability toward isolation on silica and alumina – acidic, neutral, or basic).*

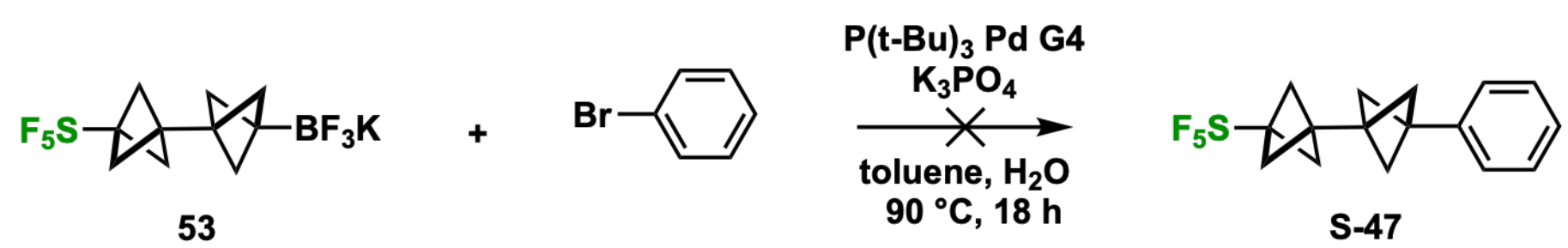

The synthesis was adapted from a known literature procedure.[50] To an oven-dried microwave vial equipped with a stir bar was added compound **53** (30 mg, 0.082 mmol, 1.0 equiv.), P(*t*-Bu)$_3$ Pd G4 (4.8 mg, 0.008 mmol, 0.1 equiv.), and K_3PO_4 (122 mg, 0.574 mmol, 7.0 equiv.). The vial was sealed, then evacuated and backfilled with Ar three times. Anhydrous toluene (0.50 mL), H_2O (0.25 mL), and bromobenzene (0.04 mL, 0.410 mmol, 5.0 equiv.) were sequentially added *via* syringe. The reaction mixture was sparged with Ar for 5 min. and stirred at 90 °C in an oil bath for 18 h. The reaction mixture was cooled to room temperature and quenched with brine (3 mL). The aqueous layer was extracted with EtOAc (3 x 10 mL). The combined organic layers were washed with brine (3 x 10 mL), dried over anhydrous Na_2SO_4, filtered through Celite, and concentrated. The crude residue was filtered through silica eluting with *n*-pentane (200 mL) and concentrated.

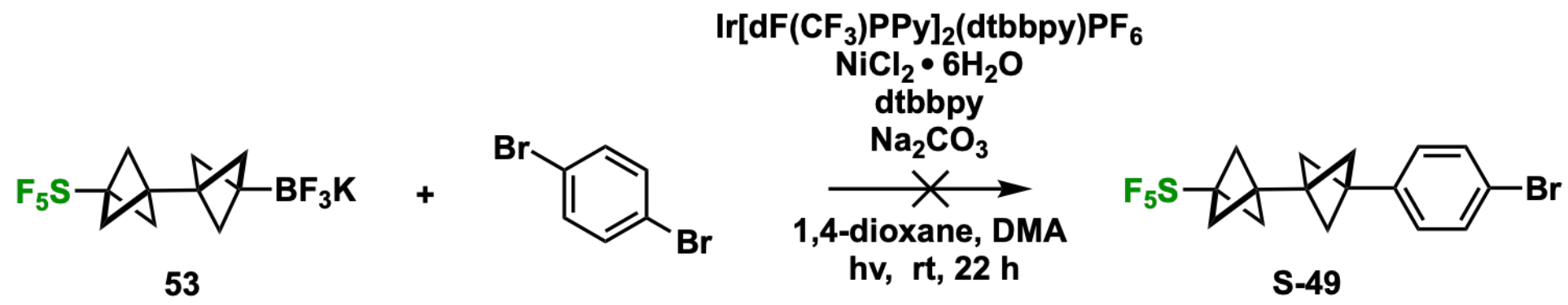

The synthesis of compound **S-49** was adapted from a known literature procedure.[55] To an oven-dried microwave vial equipped with a stir bar were added compound **53** (23.0 mg, 0.064 mmol, 1.5 equiv.), 1,4-dibromobenzene (10.0 mg, 0.042 mmol, 1.0 equiv.), $Ir[dF(CF_3)PPy]_2(dtbbpy)PF_6$ (2.40 mg, 0.002 mmol, 0.050 equiv.), $NiCl_2 \bullet 6H_2O$ (0.70 mg, 0.003 mmol, 0.1 equiv.) and Na_2CO_3 (8.90 mg, 0.085 mmol, 2.0 equiv.). The vial was brought inside of the glove box and dtbbpy (1.70 mg, 0.006 mmol, 0.15 equiv.) was added. The vial was sealed and then taken out of the glove box. Anhydrous DMA (0.32 mL) and 1,4-dioxane (0.11 mL) was added *via* syringe and sparged for 2 min with Ar. The reaction mixture was then irradiated with blue LEDs for 22 h. The reaction was quenched with water (1 mL) and extracted with EtOAc (3 x 10 mL). The combined organic layers were washed with brine (3 x 4 mL), dried over anhydrous Na_2SO_4, filtered through Celite, and concentrated.

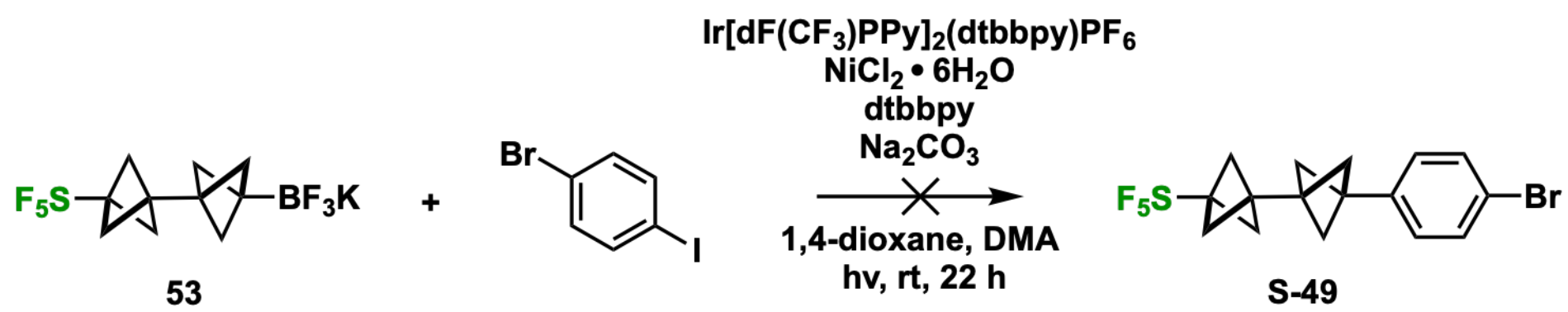

The synthesis of compound **S-49** was adapted from a known literature procedure.[55] To an oven-dried microwave vial equipped with a stir bar were added compound **53** (20.0 mg, 0.053 mmol, 1.5 equiv.), 1-bromo-4-iodobenzene (10.0 mg, 0.035 mmol, 1.0 equiv.), $Ir[dF(CF_3)PPy]_2(dtbbpy)PF_6$ (2.40 mg, 0.002 mmol, 0.050 equiv.), $NiCl_2 \bullet 6H_2O$ (0.70 mg, 0.003 mmol, 0.1 equiv.) and Na_2CO_3 (8.90 mg, 0.085 mmol, 2.0 equiv.). The vial was brought inside of the glove box and dtbbpy (1.70 mg, 0.006 mmol, 0.15 equiv.) was added. The vial was sealed and then taken out of the glove box. Anhydrous DMA (0.32 mL) and 1,4-dioxane (0.11 mL) was added *via* syringe and sparged for 2 min with Ar. The reaction mixture was then irradiated with blue LEDs for 22 h. The reaction was quenched with water (1 mL) and extracted with EtOAc (3 x 10 mL). The combined organic layers were washed with brine (3 x 4 mL), dried over anhydrous Na_2SO_4, filtered through Celite, and concentrated.

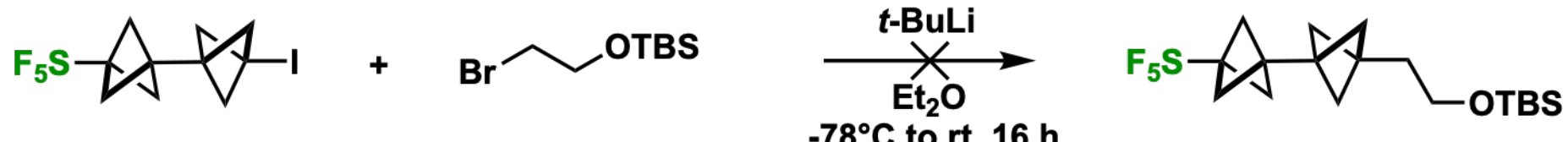

5

To an oven-dried microwave vial equipped with a stir bar were added compound **5** (0.200 g, 0.518 mmol, 1.00 equiv.). The vial was sealed with a septum, evacuated, and backfilled with argon three times. Anhydrous Et_2O (4.5 mL) was added *via* syringe. The reaction mixture was cooled to -78 °C. Then, a 1.7 M solution of *t*-BuLi in pentane (0.67 mL, 0.311 mmol, 2.2 equiv.) was added dropwise *via* syringe. After addition, the reaction was stirred at -78 °C for 1 h. In a separate oven-dried microwave vial, **(**2-bromoethoxy)(*tert*-butyl)dimethylsilane (0.33 mL, 1.554 mmol, 3.00 equiv.) was dissolved in anhydrous Et_2O (1.50 mL) and added dropwise to the cooled reaction mixture. The reaction mixture was then stirred at room temperature for 16 h. The reaction mixture was quenched with sat. aq. NH_4Cl (3 mL). The crude mixture was extracted with Et_2O (3 x 10 mL). The combined organic layers were washed with brine (3 x 4 mL), dried over anhydrous Na_2SO_4, filtered through Celite, and concentrated.

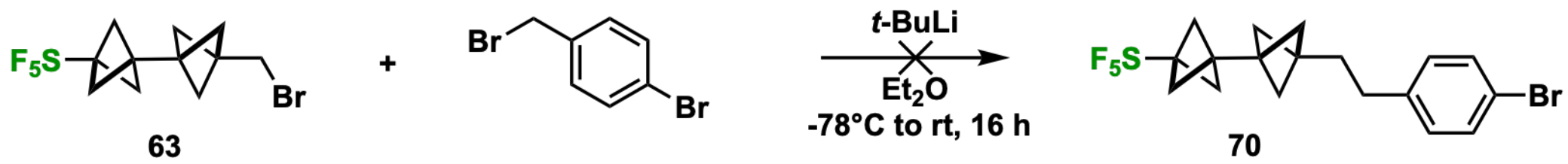

63 70

To an oven-dried microwave vial equipped with a stir bar was charged with compound **63** (50.0 mg, 0.142 mmol, 1.00 equiv.). The vial was sealed, evacuated, and backfilled with Ar three times. Anhydrous Et_2O (4.5 mL) was added *via* syringe. The reaction mixture was cooled to -78 °C. To the cooled reaction mixture, a 1.7 M solution of *t*-BuLi in pentane (0.20 mL, 0.311 mmol, 2.2 equiv.) was added dropwise. After addition was complete, the reaction was stirred at -78 °C for 1 h. In a separate oven-dried microwave vial, 1-bromo-4-(bromomethyl)benzene (42.5 mg, 0.170 mmol, 1.2 equiv.) was dissolved in anhydrous Et_2O (1.2 mL) and added dropwise to the cooled reaction mixture. The reaction mixture was then stirred at room temperature for 16 h. The reaction mixture was quenched with sat. aq. NH_4Cl (3 mL). The aqueous layer was extracted with Et_2O (3 x 10 mL). The combined organic layers were washed with brine (3 x 4 mL), dried over anhydrous Na_2SO_4, filtered through Celite, and concentrated.

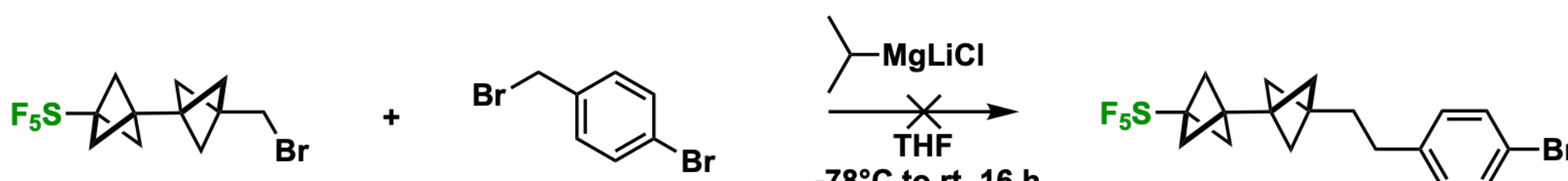

63 70

To an oven-dried microwave vial equipped with a stir bar was charged with compound **63** (50.0 mg, 0.142 mmol, 1.00 equiv.). The vial was sealed, evacuated, and backfilled with Ar three times. Anhydrous Et_2O (4.50 mL) was added *via* syringe. The reaction mixture was cooled to -78 °C. To the cooled reaction mixture, a 1.3 M solution of isopropylmagnesium LiCl in THF (0.08 mL, 0.170 mmol, 1.2 equiv.) was added dropwise. After addition was complete, the reaction was left to stir at -78 °C for 1 h. In a separate oven-dried microwave vial, 1-bromo-4-(bromomethyl)benzene (42.5 mg, 0.170 mmol, 1.20 equiv.) was dissolved in anhydrous Et_2O (1.20 mL) and added dropwise to the cooled reaction mixture. The reaction mixture was then stirred at room temperature for 16 h. The reaction mixture was quenched with sat. aq. NH_4Cl (3 mL). The aqueous layer was extracted with Et_2O (3 x 10 mL). The combined organic layers were washed with brine (3 x 4 mL), dried over anhydrous Na_2SO_4, filtered through Celite, and concentrated.

Summary of *in vitro* ADME Profiling

Table S5. Summary of *in vitro* ADME profiling of compounds **7** and **9**.

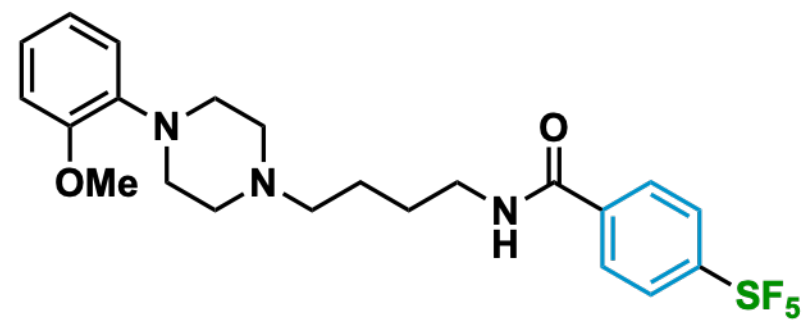

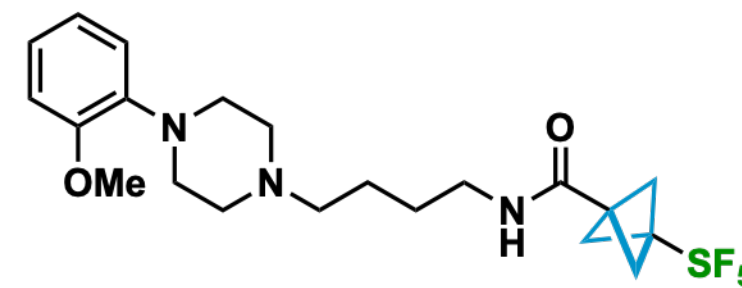

Compound	7	9
Measured $LogD_{7.4}$ / calc'd	3.60 / 4.05	2.70 / 2.86
LM CL_{int} m / r / h (µL/min/mg)[a]	266 / 213 / 226	120 / 211 / 90
HT Solubility pH = 6.8 (mM)[b]	.166	.588
MDCK-LE[c] $P_{app\ A–B}$ ($10\ cm^{-6}/s$) / % Recovery	23 / 61	30 / 78
hERG inhibition Qube IC_{50} (µM)	<0.37	1.6
HepG2 Cytotoxicity CC_{50} (µM)	22	89
CYP3A4 TDI k_{obs} (min^{-1})	.023	.013
CYP3A4 / 2D6 / 2C9 IC_{50} (µM)	22 / 1 / >25	>25 / 14 / >25

[a] LM CL_{int} = liver microsomal intrinsic clearance in mouse, rat, and human, respectively. [b] HT = high throughput. [c] Low efflux Madin–Darby canine kidney permeability.

Table S6. Summary of *in vitro* ADME profiling of compounds **25** and **26**.

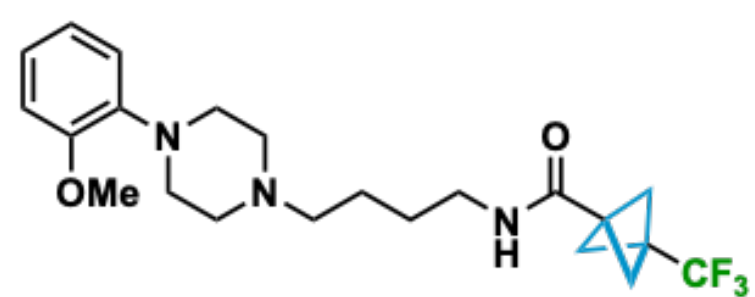

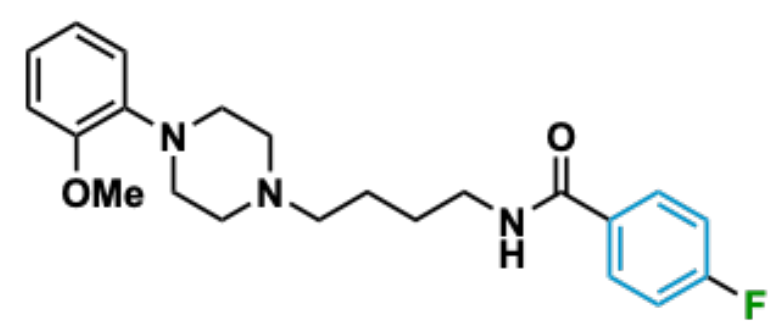

Compound	25	26
Measured $LogD_{7.4}$ / calc'd	2.20 / 2.76	2.40 / 2.64
LM CL_{int} m / r / h (µL/min/mg)[a]	162 / 84 / 37	34 / 49 / 25
HT Solubility pH = 6.8 (mM)[b]	.649	.333
MDCK-LE[c] $P_{app\ A–B}$ ($10\ cm^{-6}/s$) / % Recovery	4.7 / 54	51 / 98
hERG inhibition Qube IC_{50} (µM)	6.5	2.6
HepG2 Cytotoxicity CC_{50} (µM)	>100	>100
CYP3A4 TDI k_{obs} (min^{-1})	.006	.001
CYP3A4 / 2D6 / 2C9 IC_{50} (µM)	>25 / 13 / >25	>25 / 23 / >25

[a] LM CL_{int} = liver microsomal intrinsic clearance in mouse, rat, and human, respectively. [b] HT = high throughput. [c] Low efflux Madin–Darby canine kidney permeability.

Table S7. Summary of *in vitro* ADME profiling of compound **27**.

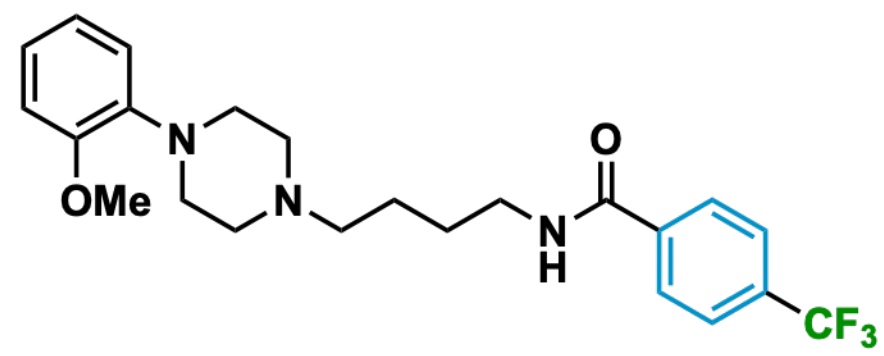

Compound	27
Measured $LogD_{7.4}$ / calc'd	3.10 / 3.57
LM CL_{int} m / r / h (μL/min/mg)[a]	91 / 177 / 112
HT Solubility pH = 6.8 (mM)[b]	.100
MDCK-LE[c] $P_{app\ A\text{–}B}$ (10 cm^{-6}/s) / % Recovery	5 / 60
hERG inhibition Qube IC_{50} (μM)	<0.37
HepG2 Cytotoxicity CC_{50} (μM)	38
CYP3A4 TDI k_{obs} (min^{-1})	.012
CYP3A4 / 2D6 / 2C9 IC_{50} (μM)	24 / 2 / >25

[a] LM CL_{int} = liver microsomal intrinsic clearance in mouse, rat, and human, respectively. [b] HT = high throughput. [c] Low efflux Madin–Darby canine kidney permeability.

Table S8. Summary of *in vitro* ADME profiling of compounds **10** and **12**.

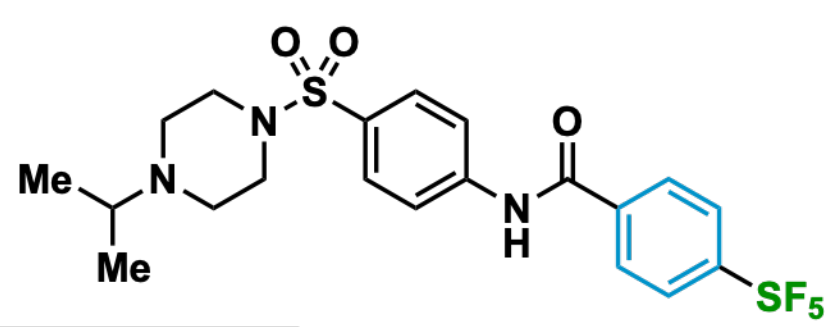

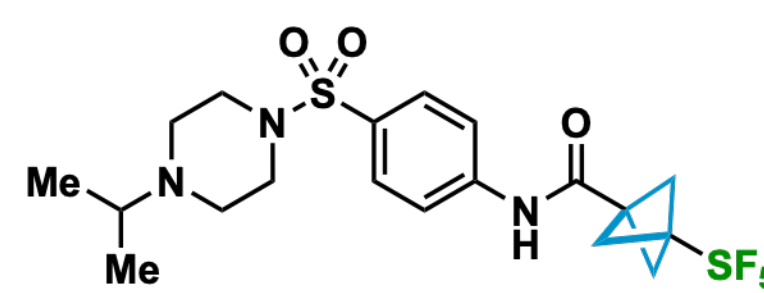

Compound	10	12
Measured $LogD_{7.4}$ / calc'd	4.40 / 4.11	3.40 / 3.27
LM CL_{int} m / r / h (μL/min/mg)[a]	329 / 617 / 142	>1000 / 936 / 160
HT Solubility pH = 6.8 (mM)[b]	<.004	.011
MDCK-LE[c] $P_{app\ A\text{–}B}$ (10 cm^{-6}/s) / % Recovery	3.9 / 53	5.3 / 53
hERG inhibition Qube IC_{50} (μM)	11	20
HepG2 Cytotoxicity CC_{50} (μM)	30	>100
CYP3A4 TDI k_{obs} (min^{-1})	.007	.005
CYP3A4 / 2D6 / 2C9 IC_{50} (μM)	>25 / 9 / 20	>25 / 20 / >25

[a] LM CL_{int} = liver microsomal intrinsic clearance in mouse, rat, and human, respectively. [b] HT = high throughput. [c] Low efflux Madin–Darby canine kidney permeability.

Table S9. Summary of *in vitro* ADME profiling of compounds **13** and **16**.

	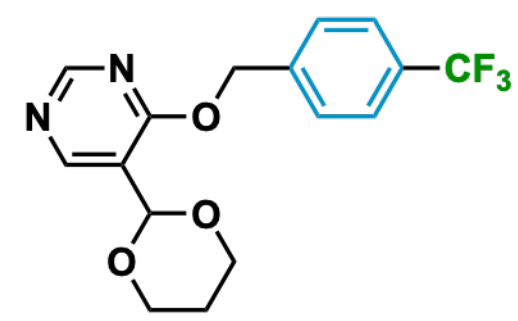	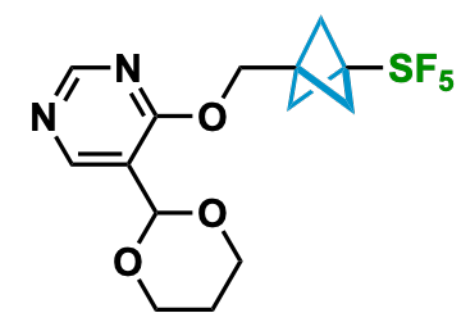
**Compound**	**13**	**16**
Measured Log$D_{7.4}$ / calc'd	3.20 / 2.95	3.10 / 3.40
LM CL_{int} m / r / h (µL/min/mg)[a]	>1000 / >1000 / 183	>1000 / >1000 / 310
HT Solubility pH = 6.8 (mM)[b]	.006	.039
MDCK-LE[c] $P_{app\ A\text{–}B}$ (10 cm^{-6}/s) / % Recovery	26 / 64	22 / 61
hERG inhibition Qube IC_{50} (µM)	>30	>30
HepG2 Cytotoxicity CC_{50} (µM)	>100	>100
CYP3A4 TDI k_{obs} (min^{-1})	.004	.008
CYP3A4 / 2D6 / 2C9 IC_{50} (µM)	>25 / >25 / 11	>25 / >25 / 15

[a] LM CL_{int} = liver microsomal intrinsic clearance in mouse, rat, and human, respectively. [b] HT = high throughput. [c] Low efflux Madin–Darby canine kidney permeability.

Table S10. Summary of *in vitro* ADME profiling of compounds **17** and **20**.

	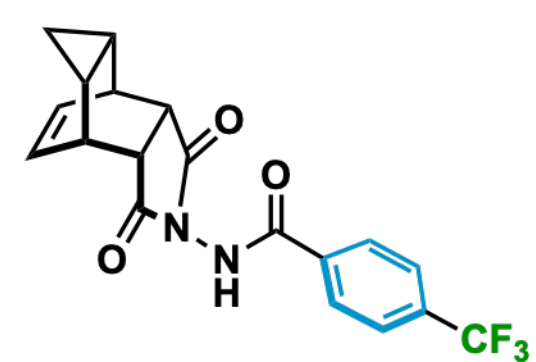	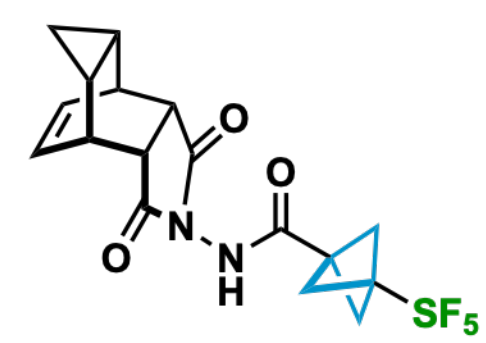
**Compound**	**17**	**20**
Measured Log$D_{7.4}$ / calc'd	2.70 / 2.58	2.90 / 2.05
LM CL_{int} m / r / h (µL/min/mg)[a]	<10 / 24 / <10	127 / 72 / <10
HT Solubility pH = 6.8 (mM)[b]	.006	.007
MDCK-LE[c] $P_{app\ A\text{–}B}$ (10 cm^{-6}/s) / % Recovery	30 / 61	24 / 57
hERG inhibition Qube IC_{50} (µM)	>30	>30
HepG2 Cytotoxicity CC_{50} (µM)	>100	>100
CYP3A4 TDI k_{obs} (min^{-1})	.009	.005
CYP3A4 / 2D6 / 2C9 IC_{50} (µM)	>25 / >25 / >25	>25 / >25 / >25

[a] LM CL_{int} = liver microsomal intrinsic clearance in mouse, rat, and human, respectively. [b] HT = high throughput. [c] Low efflux Madin–Darby canine kidney permeability.

Table S11. Summary of *in vitro* ADME profiling of compounds **28** and **30**.

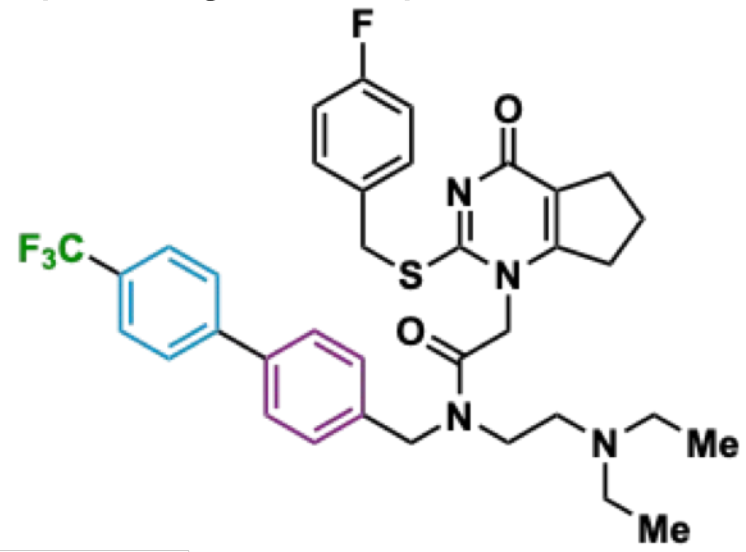

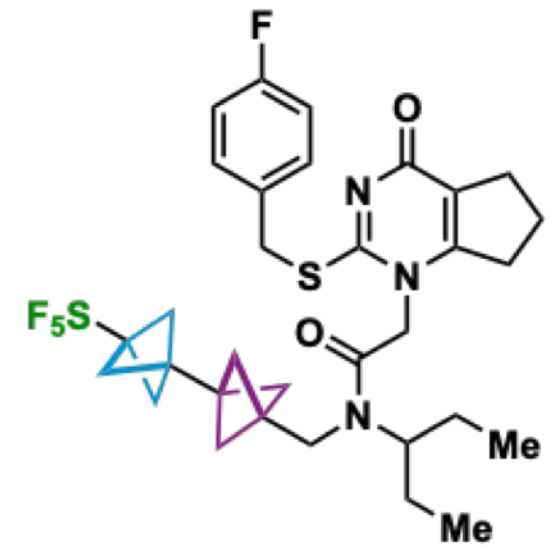

Compound	28	30
Measured $LogD_{7.4}$ / calc'd	4.60 / 4.40	4.50 / 3.55
LM CL_{int} m / r / h (µL/min/mg)[a]	<25 / 244 / 146	39 / 256 / 115
HT Solubility pH = 6.8 (mM)[b]	.382	.198
MDCK-LE[c] $P_{app\ A–B}$ ($10\ cm^{-6}/s$) / % Recovery	7.3 / 63	15 / 33
hERG inhibition Qube IC_{50} (µM)	2.1	5.2
HepG2 Cytotoxicity CC_{50} (µM)	5	45
CYP3A4 TDI k_{obs} (min^{-1})	.003	.007
CYP3A4 / 2D6 / 2C9 IC_{50} (µM)	>25 / >25 / >25	14 / 15 / >25

[a] LM CL_{int} = liver microsomal intrinsic clearance in mouse, rat, and human, respectively. [b] HT = high throughput. [c] Low efflux Madin–Darby canine kidney permeability. [d] Permeability values are not reported for assays with <30% recovery. In vitro parameters for [2]-staffane 30 and 28 are reported as mean values across assays conducted using stored DMSO stocks and freshly prepared solutions, where data for both preparations were available.

Table S12. Summary of *in vitro* ADME profiling of compound **30**.

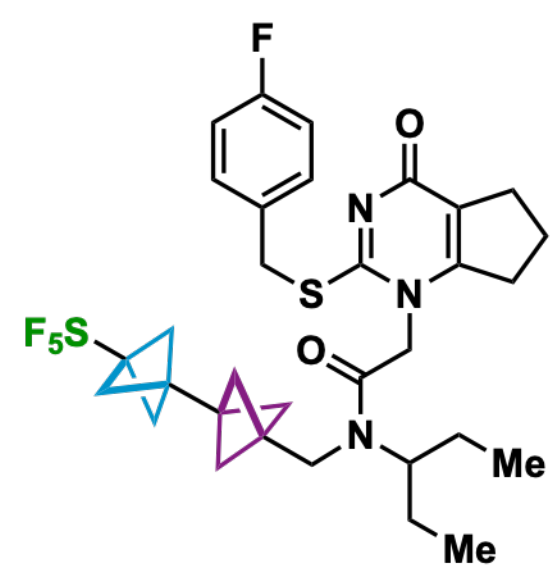

Compound	DMSO stock	fresh powder[e]
Measured $LogD_{7.4}$ / calc'd	- / 3.55	4.50 / 3.55
LM CL_{int} m / r / h (µL/min/mg)[a]	*assay failed[d]*	39 / 256 / 115
HT Solubility pH = 6.8 (mM)[b]	*assay failed[d]*	.198
MDCK-LE[c] $P_{app\ A–B}$ ($10\ cm^{-6}/s$) / % Recovery	*assay failed[d]*	15 / 33
hERG inhibition Qube IC_{50} (µM)	5.2	-
HepG2 Cytotoxicity CC_{50} (µM)	32	13
CYP3A4 TDI k_{obs} (min^{-1})	.007	-
CYP3A4 / 2D6 / 2C9 IC_{50} (µM)	14 / 15 / >25	- / - / -

[a] LM CL_{int} = liver microsomal intrinsic clearance in mouse, rat, and human, respectively. [b] HT = high throughput. [c] Low efflux Madin–Darby canine kidney permeability. [d] assay failed due to insufficient parent mass detected, values not reported. [e] Due to practical constraints with assay execution, values for freshly prepared material could only be obtained for a subset of assays, indicated above. *In vitro parameters for [2]-staffanes **30** when prepared from stored DMSO stocks vs. freshly prepared solutions, where available; assay failed due to insufficient parent mass detected, values not reported.*

Table S13. Summary of *in vitro* ADME profiling of compounds **31** and **33**.

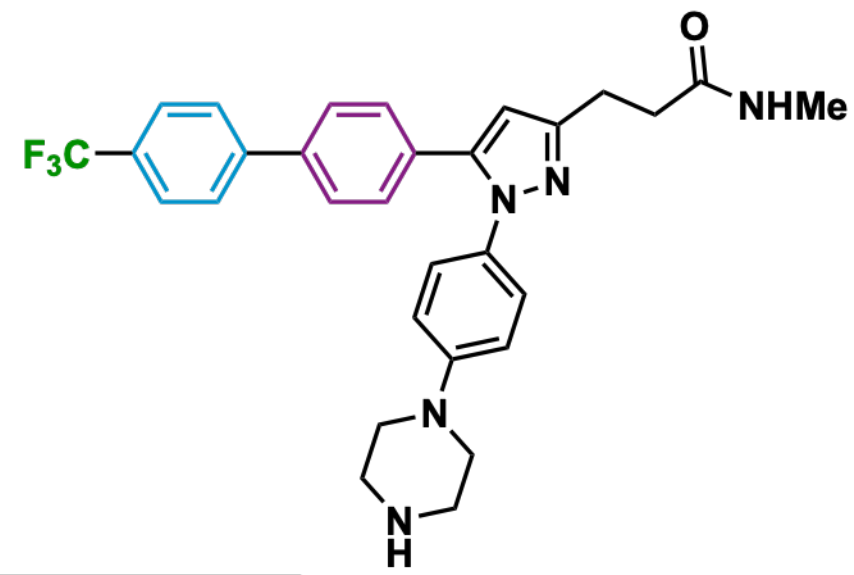

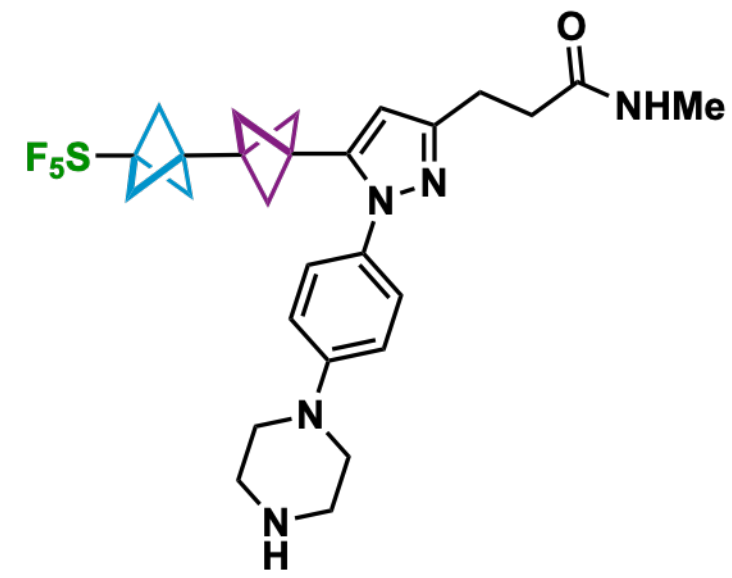

Compound	31	33
Measured $LogD_{7.4}$ / calc'd	3.50 / 3.55	2.90 / 2.52
LM CL_{int} m / r / h (µL/min/mg)[a]	46 / <25 / 13	108 / 51 / 18
HT Solubility pH = 6.8 (mM)[b]	<.004	.016
MDCK-LE[c] $P_{app\ A–B}$ ($10\ cm^{-6}/s$) / % Recovery	3.7 / 30	- / 12[d]
hERG inhibition Qube IC_{50} (µM)	1.8	-
HepG2 Cytotoxicity CC_{50} (µM)	4	65
CYP3A4 TDI k_{obs} (min^{-1})	.004	.006
CYP3A4 / 2D6 / 2C9 IC_{50} (µM)	>25 / >25 / >25	>25 / >25 / >25

*[a] LM CL_{int} = liver microsomal intrinsic clearance in mouse, rat, and human, respectively. [b] HT = high throughput. [c] Low efflux Madin–Darby canine kidney permeability. [d] Permeability values are not reported for assays with <30% recovery. In vitro parameters for [2]-staffane **33** are reported as mean values across assays conducted using stored DMSO stocks and freshly prepared solutions, where data for both preparations were available.*

Table S14. Summary of *in vitro* ADME profiling of compound **33**.

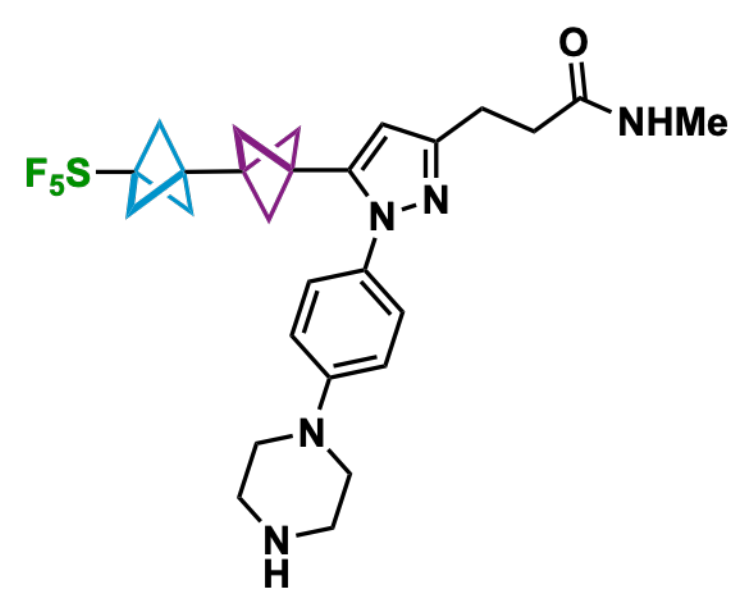

Compound	DMSO stock	fresh powder[e]
Measured $LogD_{7.4}$ / calc'd	2.90 / 2.52	2.90 / 2.52
LM CL_{int} m / r / h (µL/min/mg)[a]	105 / 50 / 18	111 / 53 / 19
HT Solubility pH = 6.8 (mM)[b]	0.033	0.008
MDCK-LE[c] $P_{app\ A–B}$ ($10\ cm^{-6}/s$) / % Recovery	- / 17[d]	- / 9[d]
hERG inhibition Qube IC_{50} (µM)	-	-
HepG2 Cytotoxicity CC_{50} (µM)	64	65
CYP3A4 TDI k_{obs} (min^{-1})	.006	-
CYP3A4 / 2D6 / 2C9 IC_{50} (µM)	>25 / >25 / >25	- / - / -

[a] LM CL_{int} = liver microsomal intrinsic clearance in mouse, rat, and human, respectively. [b] HT = high throughput. [c] Low efflux Madin–Darby canine kidney permeability. [d] Permeability values are not reported for assays with <30% recovery. [e] Due to practical constraints with assay execution, values for freshly prepared material could only be obtained for a subset of assays, indicated above *In vitro parameters for [2]-staffane **33** when prepared from stored DMSO stocks vs. freshly prepared solutions, where available; assay failed due to insufficient parent mass detected, values not reported.*

Table S15. Summary of *in vitro* ADME profiling of compounds **37** and **38**.

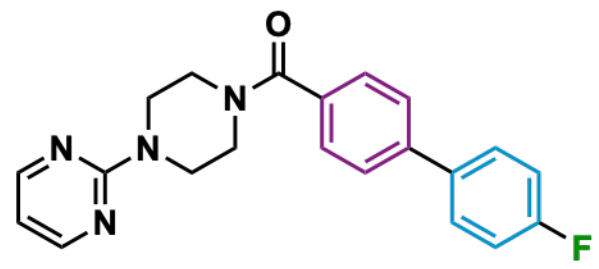

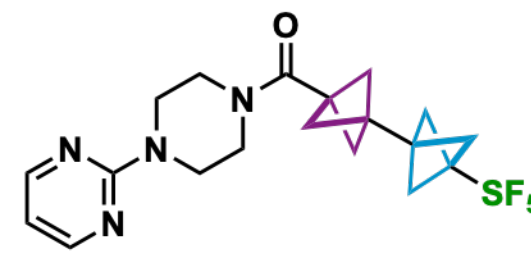

Compound	37	38
Measured $LogD_{7.4}$ / calc'd	3.60 / 4.16	4.30 / 4.11
LM CL_{int} m / r / h (µL/min/mg)[a]	37 / 128 / 37	249 / 401 / 53
HT Solubility pH = 6.8 (mM)[b]	<.004	<.004
MDCK-LE[c] $P_{app\ A–B}$ (10 cm^{-6}/s) / % Recovery	35 / 80	20 / 36
hERG inhibition Qube IC_{50} (µM)	>30	>30
HepG2 Cytotoxicity CC_{50} (µM)	>100	>100
CYP3A4 TDI k_{obs} (min^{-1})	.013	.003
CYP3A4 / 2D6 / 2C9 IC_{50} (µM)	>25 / >25 / >25	>25 / >25 / >25

[a] LM CL_{int} = liver microsomal intrinsic clearance in mouse, rat, and human, respectively. [b] HT = high throughput. [c] Low efflux Madin–Darby canine kidney permeability.

Table S16. Summary of *in vitro* ADME profiling of compounds **39** and **40**.

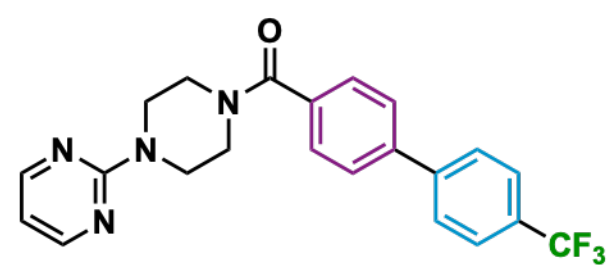

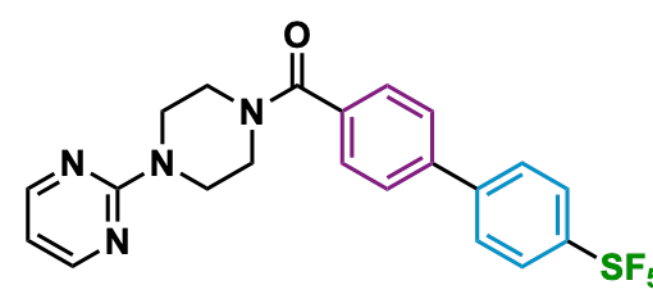

Compound	39	40
Measured $LogD_{7.4}$ / calc'd	4.30 / 4.65	4.60 / 4.99
LM CL_{int} m / r / h (µL/min/mg)[a]	41 / 94 / <10	60 / 89 / 28
HT Solubility pH = 6.8 (mM)[b]	<.004	<.004
MDCK-LE[c] $P_{app\ A–B}$ (10 cm^{-6}/s) / % Recovery	18 / 70	20 / 77
hERG inhibition Qube IC_{50} (µM)	>30	>30
HepG2 Cytotoxicity CC_{50} (µM)	>100	>100
CYP3A4 TDI k_{obs} (min^{-1})	.007	.016
CYP3A4 / 2D6 / 2C9 IC_{50} (µM)	>25 / >25 / >25	>25 / >25 / >25

[a] LM CL_{int} = liver microsomal intrinsic clearance in mouse, rat, and human, respectively. [b] HT = high throughput. [c] Low efflux Madin–Darby canine kidney permeability.

X-ray Crystallography

All single-crystal X-ray diffraction data were collected on either a Bruker APEX II Duo or Bruker D8 VENTURE diffractometer using Mo or Cu radiation (λ = 0.71073, 1.54178 Å). Crystals with suitable optical behavior were mounted onto a cryoloop with Paratone oil and transferred to the goniometer under ambient atmosphere. Data reduction and integration were performed with either the Bruker APEX3 or APEX6 software package. Data were scaled and corrected for absorption effects using the multi-scan procedure implemented in SADABS. The structures were solved by SHELXT and refined by a full-matrix least-squares procedure using SHELXL within the OLEX2 graphical user interface.[56] Graphics were generated using Mercury or OLEX2.[57] Crystallographic data and details of data collection are summarized in the table below.

Table S17. Summary of structure factors.

	**Compound_4.cif**	**Compound_5.cif**	**Compound_6.cif**
CCDC number	2561074	2561076	2561983
Empirical formula	$C_6H_7F_5O_2S$	$C_{10}H_{12}F_5IS$	$C_7H_8F_{2.50}OS_{0.50}$
Formula weight	238.18	386.16	171.66
Temperature [K]	150.00	190(2)	220
Crystal system	triclinic	orthorhombic	monoclinic
Space group (number)	$P\overline{1}$ (2)	$Pnma$ (62)	$C2/m$ (12)
a [Å]	5.6130(12)	18.7416(15)	10.837(16)
b [Å]	6.7066(14)	9.1616(7)	9.534(14)
c [Å]	11.882(3)	7.4733(6)	15.04(2)
α [°]	89.978(4)	90	90
β [°]	86.714(4)	90	97.659(14)
γ [°]	72.272(4)	90	90
Volume [Å^3]	425.27(16)	1283.19(18)	1540(4)
Z	2	4	8
ρ_{calc} [gcm^{-3}]	1.860	1.999	1.480
μ [mm^{-1}]	0.436	2.694	0.266
F(000)	240	744	708
Crystal size [mm^3]	0.345×0.272×0.11	0.834×0.14×0.102	0.985×0.406×0.156
Crystal colour	clear colourless	colourless	clear colourless
Crystal shape	block	rectangular	plate
Radiation	MoK_α (λ=0.71073 Å)	MoK_α (λ=0.71073 Å)	MoK_α (λ=0.71073 Å)
2Θ range [°]	6.38 to 60.14 (0.71 Å)	4.35 to 56.61 (0.75 Å)	2.73 to 61.08 (0.70 Å)
Index ranges	-7 ≤ h ≤ 7<br>-9 ≤ k ≤ 9<br>-16 ≤ l ≤ 16	-24 ≤ h ≤ 25<br>-12 ≤ k ≤ 12<br>-9 ≤ l ≤ 9	-15 ≤ h ≤ 15<br>-13 ≤ k ≤ 13<br>-21 ≤ l ≤ 21
Reflections collected	24808	11865	19415
Independent reflections	2482<br>R_{int} = 0.0276<br>R_{sigma} = 0.0140	1684<br>R_{int} = 0.0184<br>R_{sigma} = 0.0145	2491<br>R_{int} = 0.0335<br>R_{sigma} = 0.0197
Completeness	99.9 %	99.7 %	100.0 %
Data / Restraints / Parameters	2482/0/131	1684/0/91	2491/0/151
Goodness-of-fit on F^2	1.051	1.113	1.081
Final R indexes [I≥2σ(I)]	R_1 = 0.0315<br>wR_2 = 0.0798	R_1 = 0.0231<br>wR_2 = 0.0667	R_1 = 0.0404<br>wR_2 = 0.1175
Final R indexes [all data]	R_1 = 0.0345<br>wR_2 = 0.0821	R_1 = 0.0249<br>wR_2 = 0.0675	R_1 = 0.0463<br>wR_2 = 0.1270
Largest peak/hole [eÅ^{-3}]	0.48/-0.37	1.11/-0.66	0.43/-0.33
Flack X parameter			
Extinction coefficient			

	Compound_21.cif	Compound_24.cif	Compound_34.cif
CCDC number	2561984	2561081	2579262
Empirical formula	$C_7H_7F_3O_2$	$C_7H_5F_5O_2S$	$C_{13}H_9FO_2$
Formula weight	180.13	248.17	216.20
Temperature [K]	200.00	150.00	220.00
Crystal system	triclinic	monoclinic	monoclinic
Space group (number)	$P\overline{1}$ (2)	$P2_1/c$ (14)	$P2_1/c$ (14)
a [Å]	5.6517(5)	14.7999(4)	29.560(2)
b [Å]	6.4451(6)	7.6457(2)	3.7578(2)
c [Å]	10.6063(9)	15.8618(4)	19.3707(14)
α [°]	88.120(2)	90	90
β [°]	82.531(2)	100.1850(10)	109.105(3)
γ [°]	88.101(2)	90	90
Volume [$Å^3$]	382.70(6)	1766.57(8)	2033.2(2)
Z	2	8	8
ρ_{calc} [gcm^{-3}]	1.563	1.866	1.413
μ [mm^{-1}]	0.159	3.940	0.897
F(000)	184	992	896
Crystal size [mm^3]	0.671×0.267×0.094	0.237×0.114×0.032	0.308×0.15×0.087
Crystal colour	clear colourless	colourless	colourless
Crystal shape	plate	plank	Irregular block
Radiation	MoK_α (λ=0.71073 Å)	CuK_α (λ=1.54178 Å)	CuK_α (λ=1.54178 Å)
2Θ range [°]	3.87 to 54.95 (0.77 Å)	6.07 to 144.65 (0.81 Å)	4.83 to 144.98 (0.81 Å)
Index ranges	-7 ≤ h ≤ 7<br>-8 ≤ k ≤ 8<br>-13 ≤ l ≤ 13	-18 ≤ h ≤ 18<br>-9 ≤ k ≤ 9<br>-19 ≤ l ≤ 17	-36 ≤ h ≤ 36<br>-4 ≤ k ≤ 4<br>-22 ≤ l ≤ 23
Reflections collected	3506	24274	57211
Independent reflections	1764<br>R_{int} = 0.0184<br>R_{sigma} = 0.0216	3493<br>R_{int} = 0.0535<br>R_{sigma} = 0.0350	4032<br>R_{int} = 0.0426<br>R_{sigma} = 0.0170
Completeness	100.0 %	99.8 %	100.0 %
Data / Restraints / Parameters	1764/159/169	3493/0/279	4032/2/296
Goodness-of-fit on F^2	1.078	1.057	1.059
Final *R* indexes [*I*≥2σ(*I*)]	R_1 = 0.0537<br>wR_2 = 0.1505	R_1 = 0.0341<br>wR_2 = 0.0885	R_1 = 0.0348<br>wR_2 = 0.0967
Final *R* indexes [all data]	R_1 = 0.0581<br>wR_2 = 0.1561	R_1 = 0.0414<br>wR_2 = 0.0950	R_1 = 0.0375<br>wR_2 = 0.0997
Largest peak/hole [e$Å^{-3}$]	0.42/-0.31	0.36/-0.33	0.16/-0.17
Flack X parameter			
Extinction coefficient			

	Compound_35.cif	Compound_36.cif	Compound_46.cif
CCDC number	2579261	2561985	2561075
Empirical formula	$C_{16}H_{13}F_3O_4$	$C_{13}H_9F_5O_2S$	$C_{10}H_{12}F_5NO_3S$
Formula weight	326.26	324.26	321.27
Temperature [K]	220.00	200.15	145.00
Crystal system	triclinic	monoclinic	orthorhombic
Space group (number)	$P\overline{1}$ (2)	$P2_1/n$ (14)	$Pca2_1$ (29)
a [Å]	7.5443(4)	4.9624(6)	9.5495(4)
b [Å]	11.1603(6)	33.802(4)	9.3885(4)
c [Å]	18.4655(9)	7.7250(10)	14.5140(7)
α [°]	106.2690(10)	90	90
β [°]	96.6930(10)	97.812(3)	90
γ [°]	93.990(2)	90	90
Volume [Å^3]	1473.76(13)	1283.8(3)	1301.26(10)
Z	4	4	4
ρ_{calc} [gcm^{-3}]	1.470	1.678	1.640
μ [mm^{-1}]	0.129	0.315	0.316
F(000)	672	656	656
Crystal size [mm^3]	.331×.290×.046	0.326×0.139×0.07	1.495×0.349×0.306
Crystal colour	colourless	colourless	?
Crystal shape	irregular block	plate	Irregular
Radiation	MoK_α (λ=0.71073 Å)	MoK_α (λ=0.71073 Å)	MoK_α (λ=0.71073 Å)
2Θ range [°]	5.02 to 51.36 (0.82 Å)	4.82 to 50.83 (0.83 Å)	4.34 to 61.99 (0.69 Å)
Index ranges	-9 ≤ h ≤ 9<br>-13 ≤ k ≤ 13<br>-22 ≤ l ≤ 22	-5 ≤ h ≤ 5<br>-40 ≤ k ≤ 40<br>-9 ≤ l ≤ 9	-10 ≤ h ≤ 13<br>-13 ≤ k ≤ 13<br>-20 ≤ l ≤ 21
Reflections collected	64313	9032	19646
Independent reflections	5582<br>R_{int} = 0.0281<br>R_{sigma} = 0.0152	2349<br>R_{int} = 0.0342<br>R_{sigma} = 0.0334	4082<br>R_{int} = 0.0425<br>R_{sigma} = 0.0251
Completeness	99.9 %	100.0 %	100.0 %
Data / Restraints / Parameters	5582/156/485	2349/0/192	4082/1/181
Goodness-of-fit on F^2	1.034	1.085	1.095
Final *R* indexes [$I \geq 2\sigma(I)$]	R_1 = 0.0433<br>wR_2 = 0.1168	R_1 = 0.0414<br>wR_2 = 0.0965	R_1 = 0.0261<br>wR_2 = 0.0650
Final *R* indexes [all data]	R_1 = 0.0538<br>wR_2 = 0.1276	R_1 = 0.0596<br>wR_2 = 0.1022	R_1 = 0.0278<br>wR_2 = 0.0665
Largest peak/hole [eÅ^{-3}]	0.22/-0.26	0.28/-0.27	0.22/-0.36
Flack X parameter			0.01(2)
Extinction coefficient			

Molecular Structures and Packing Diagrams:

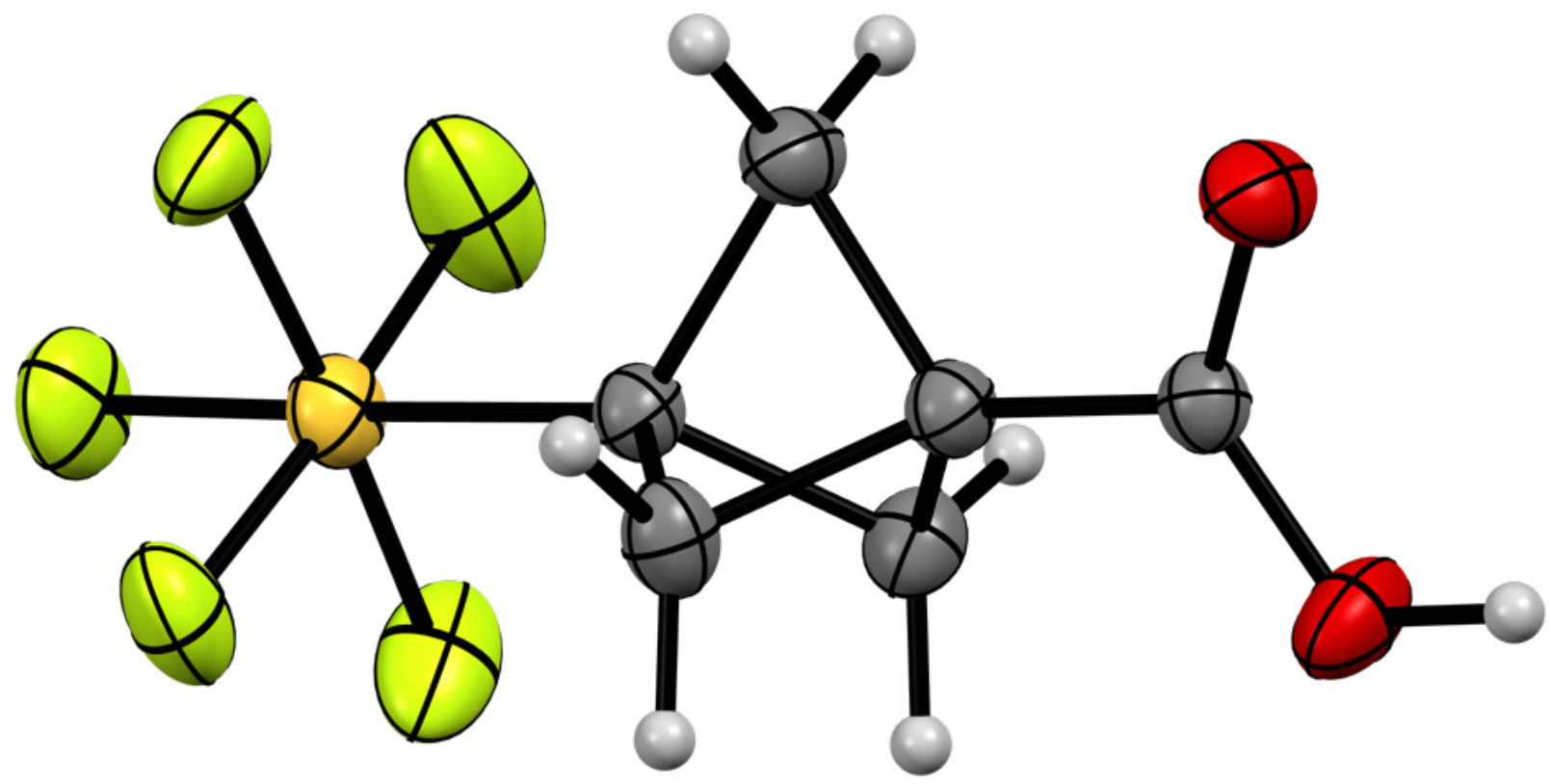

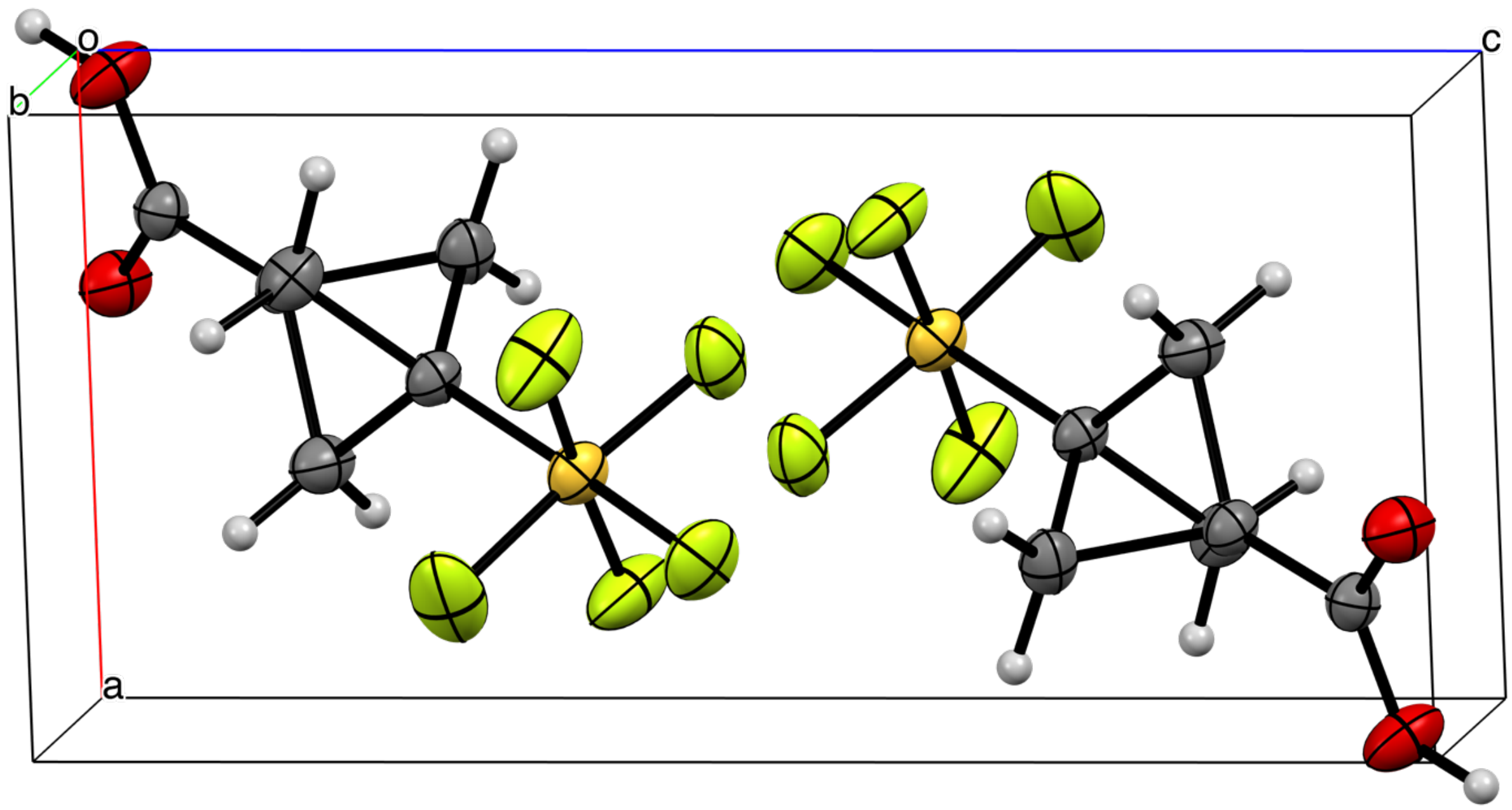

Figure S25. Molecular structure and packing of 3-(pentafluoro-λ[6]-sulfanyl)bicyclo[1.1.1]pentane-1-carboxylic acid (**4**); ellipsoids are depicted at the 50% probability level.

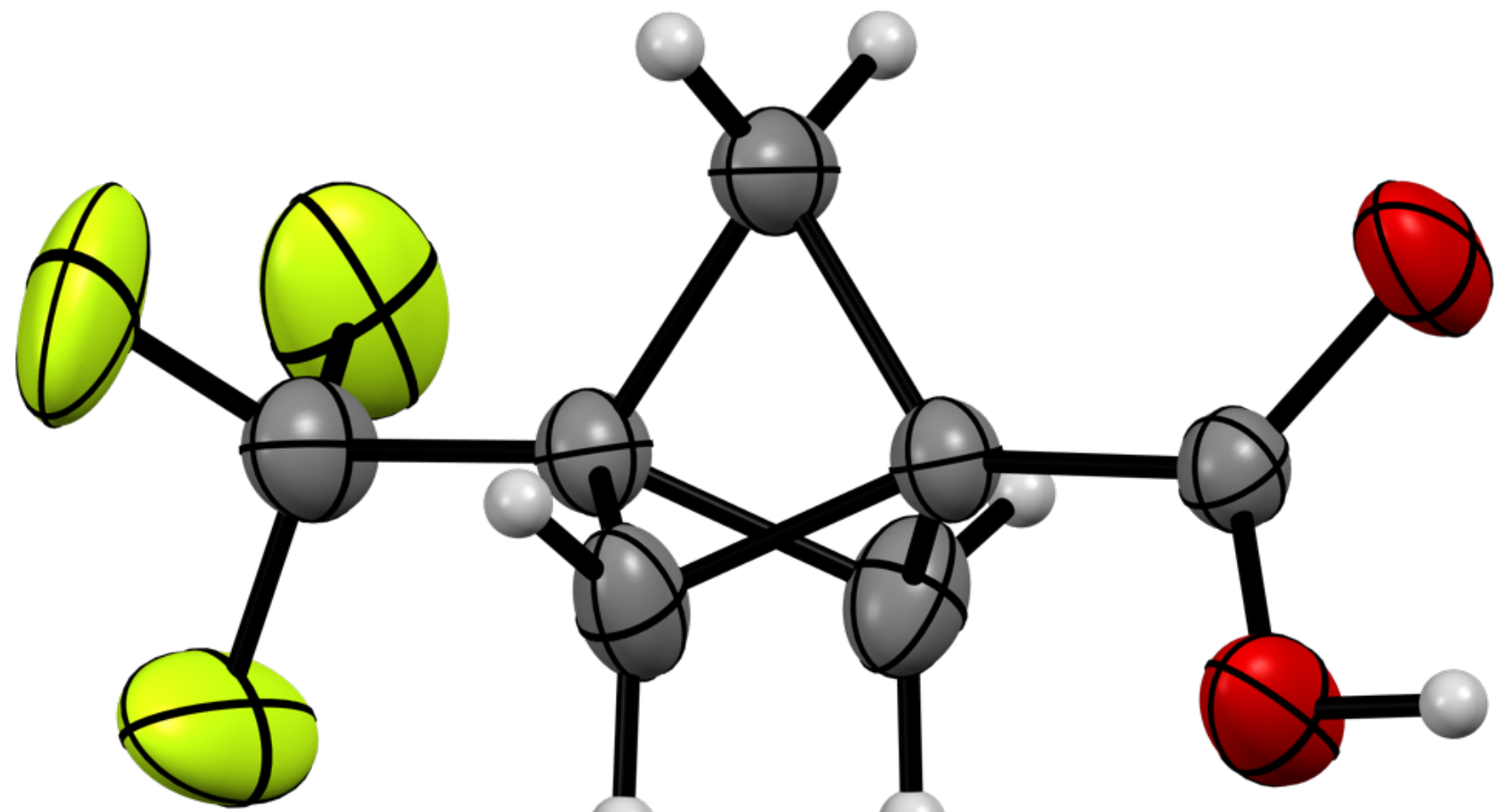

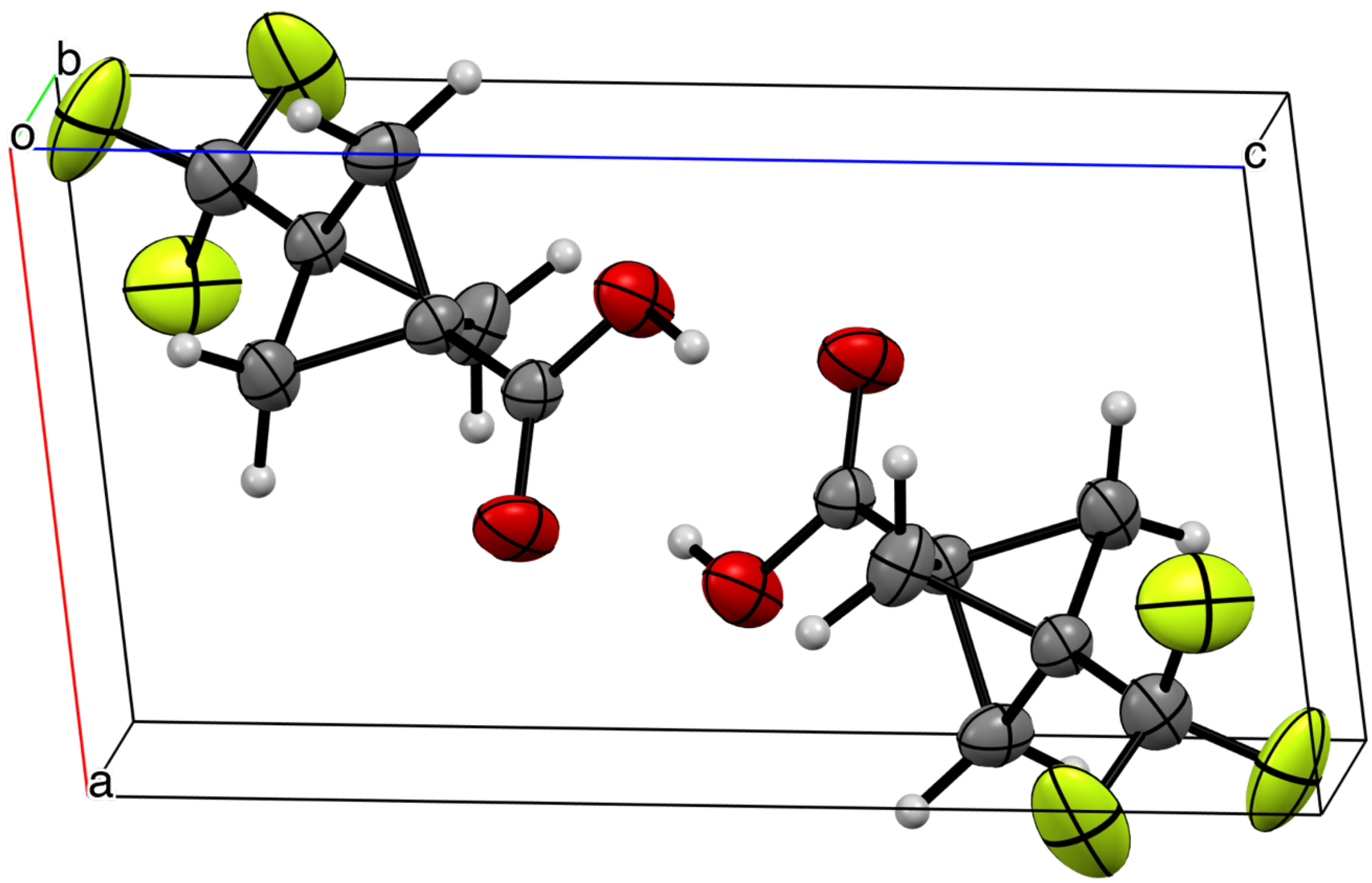

Figure S26. Molecular structure and packing of 3-(trifluoromethyl)bicyclo[1.1.1]pentane-1-carboxylic acid (**21**); ellipsoids are depicted at the 50% probability level.

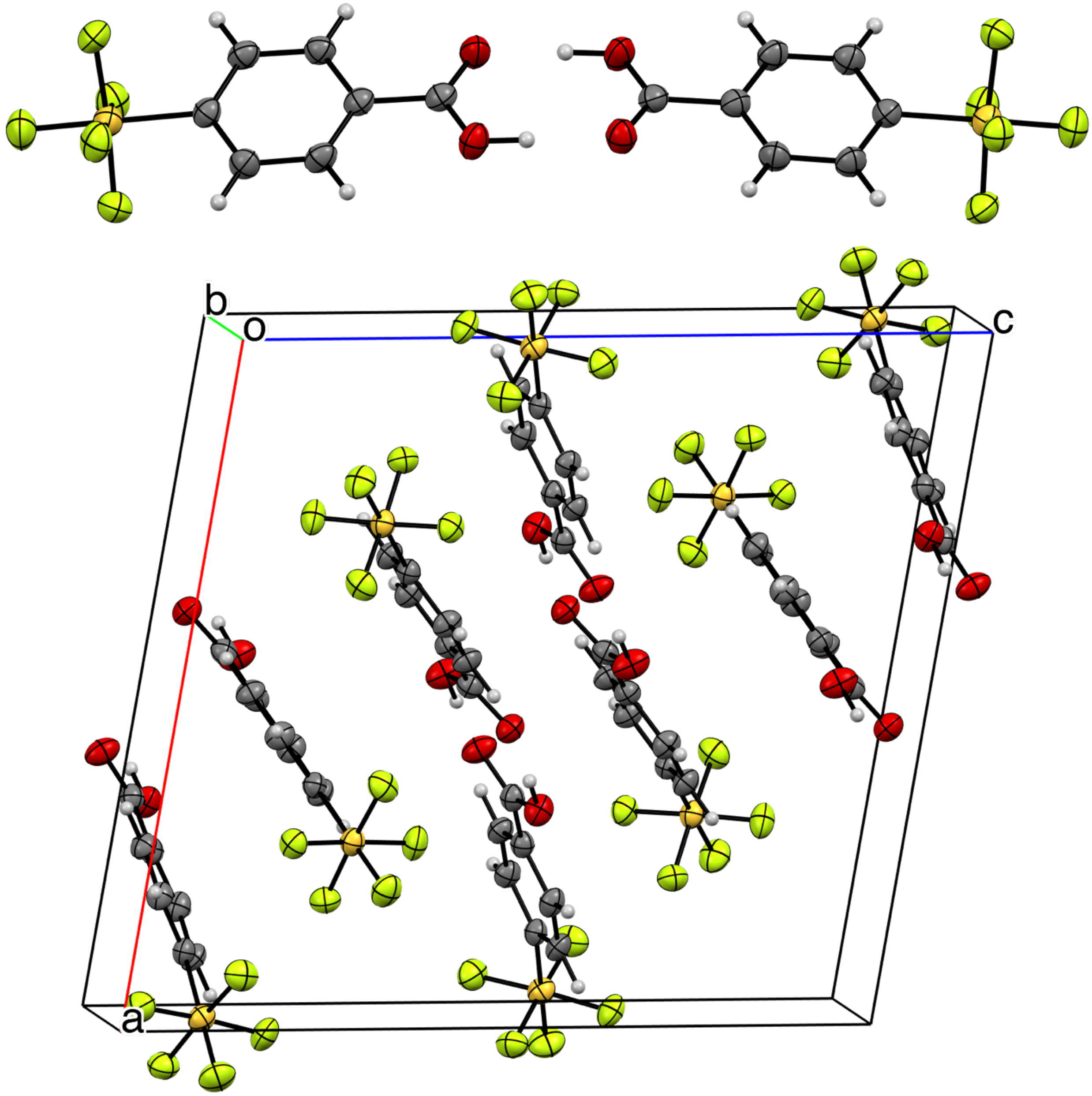

Figure S27. Molecular structure and packing of 4-(pentafluoro-λ^{6}-sulfanyl)benzoic acid (**24**); ellipsoids are depicted at the 50% probability level. There are two symmetry-independent moieties in the unit cell (Z' = 2).

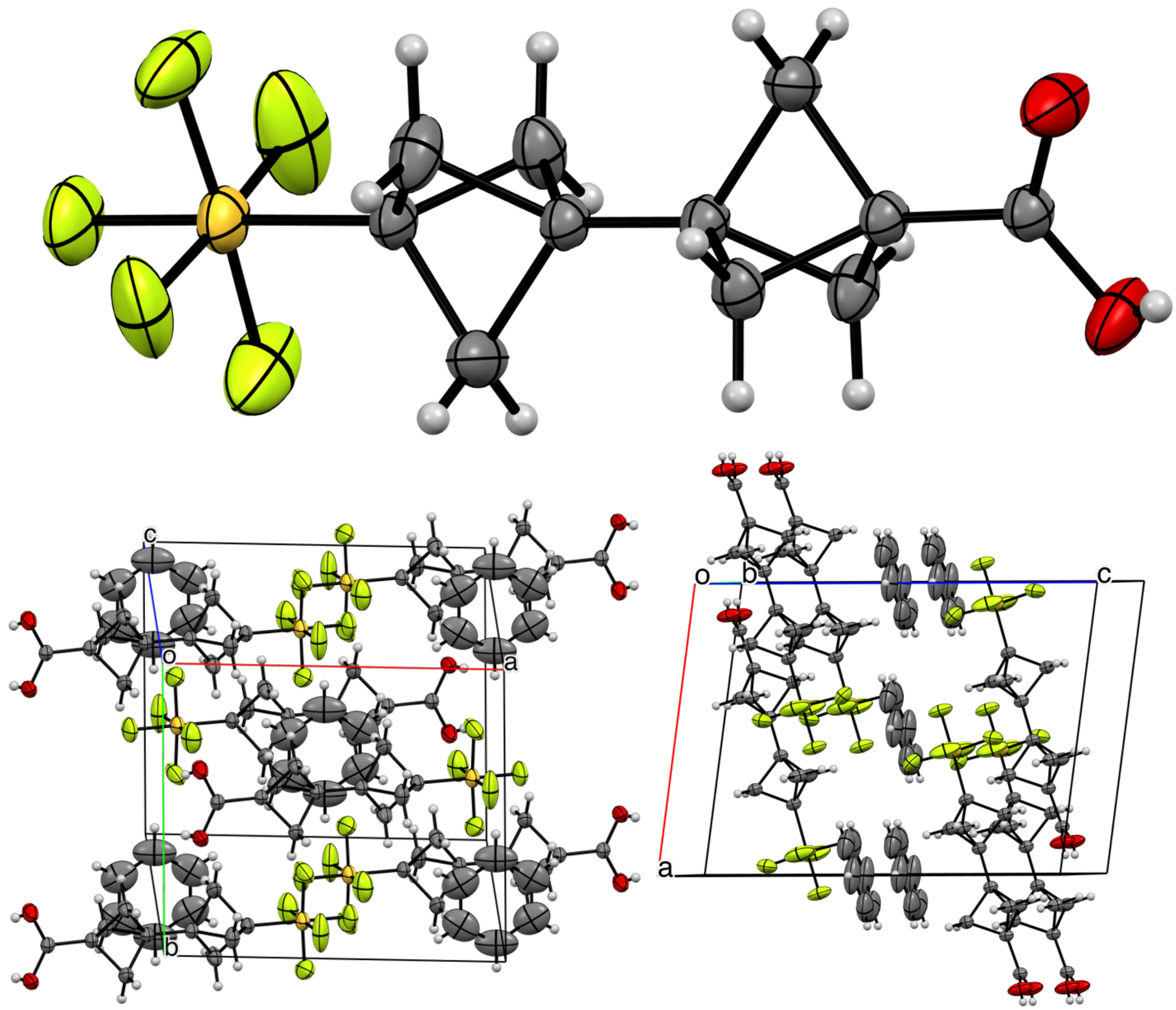

Figure S28. Molecular structure (co-crystallized benzene omitted for clarity) and packing of 3'-(pentafluoro-λ^6-sulfanyl)-[1,1'-bi(bicyclo[1.1.1]pentane)]-3-carboxylic acid (**6**); ellipsoids are depicted at the 50% probability level.

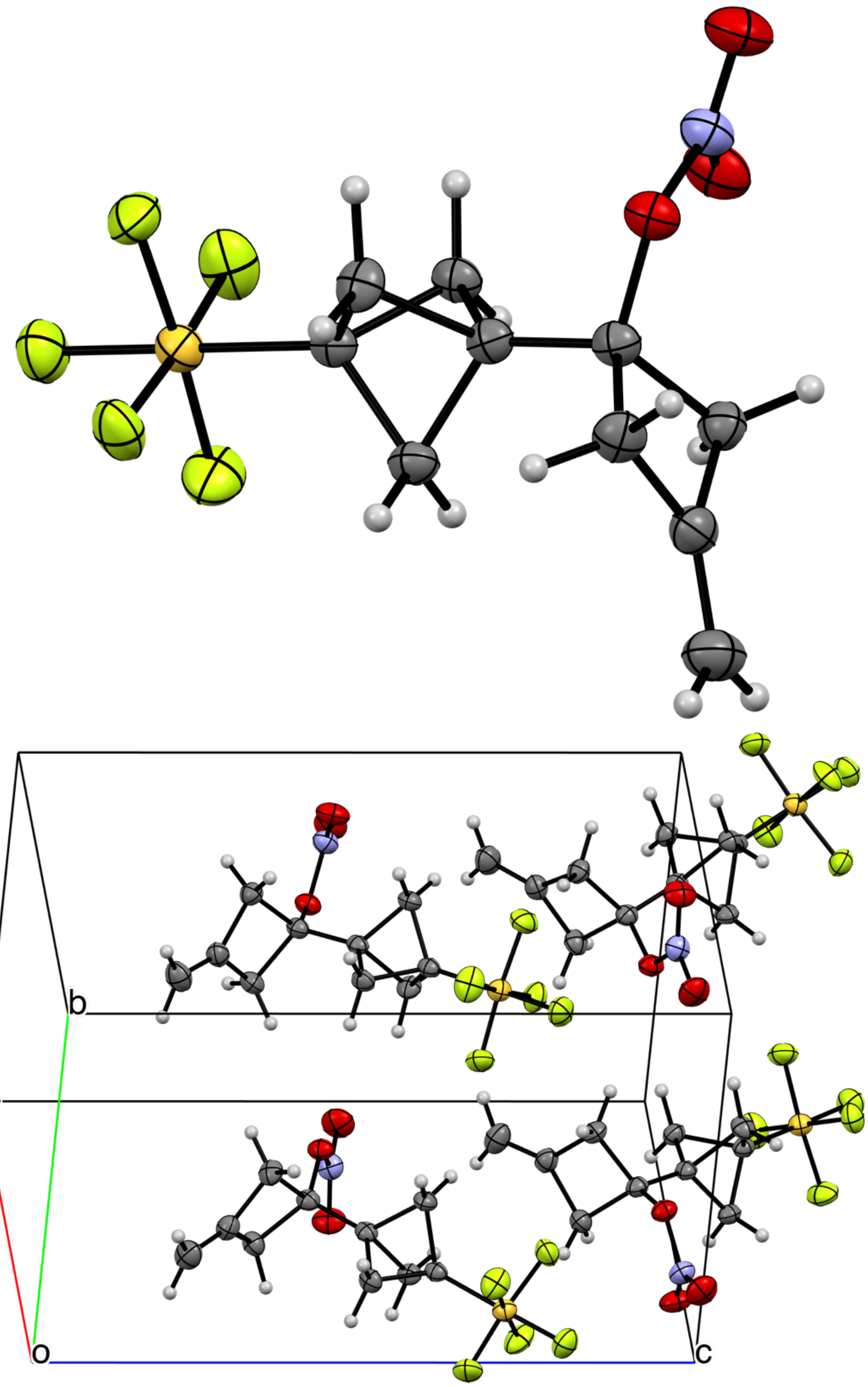

Figure S29. Molecular structure and packing of 3-methylene-1-(3-(pentafluoro-λ^{6}-sulfanyl) bicyclo[1.1.1]pentan-1-yl)cyclobutyl nitrate (**39**); ellipsoids are depicted at the 50% probability level.

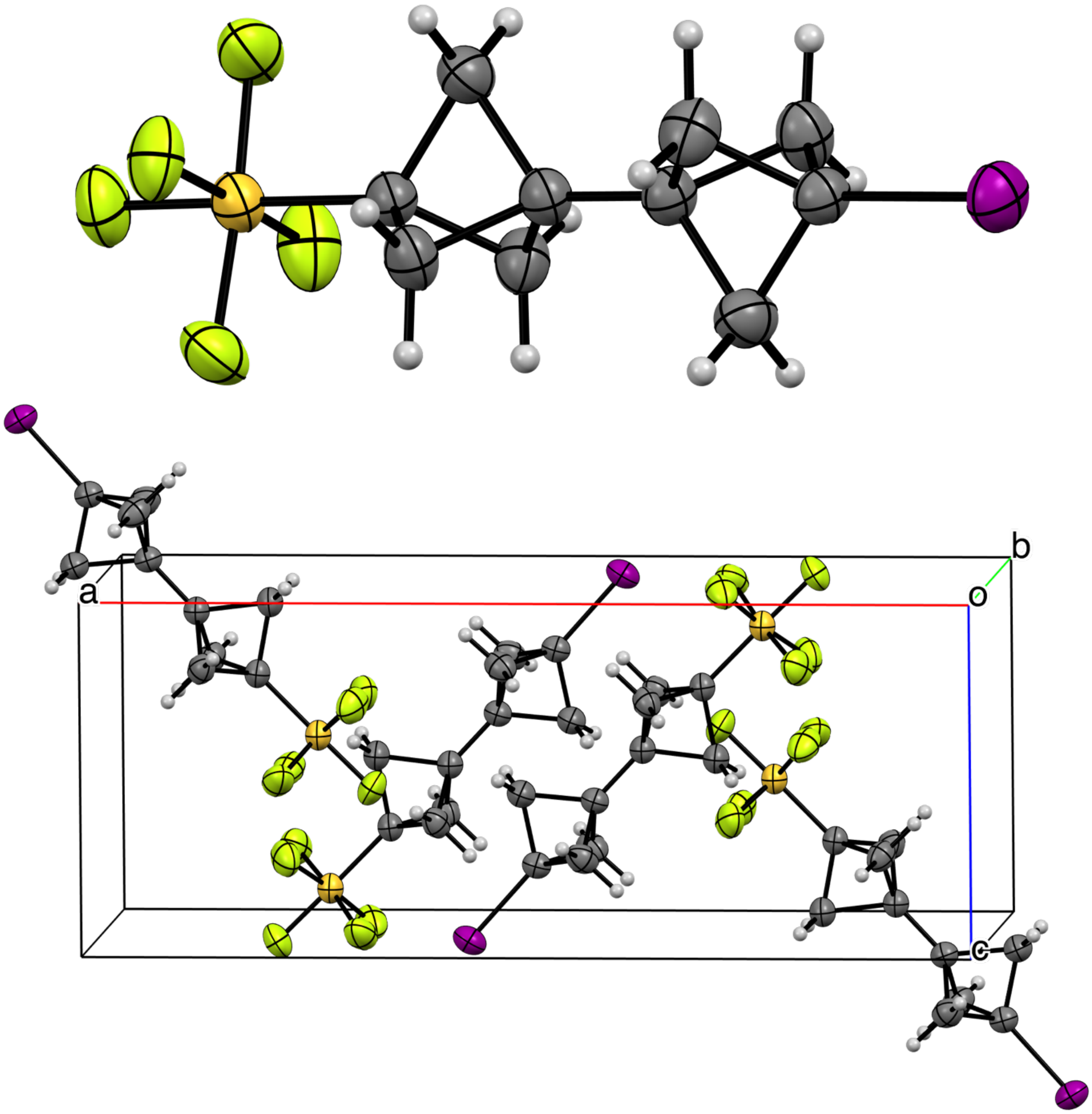

Figure S30. Molecular structure and packing of pentafluoro(3'-iodo-[1,1'-bi(bicyclo[1.1.1]pentan)]-3-yl)-λ^6-sulfane (**5**); ellipsoids are depicted at the 50% probability level.

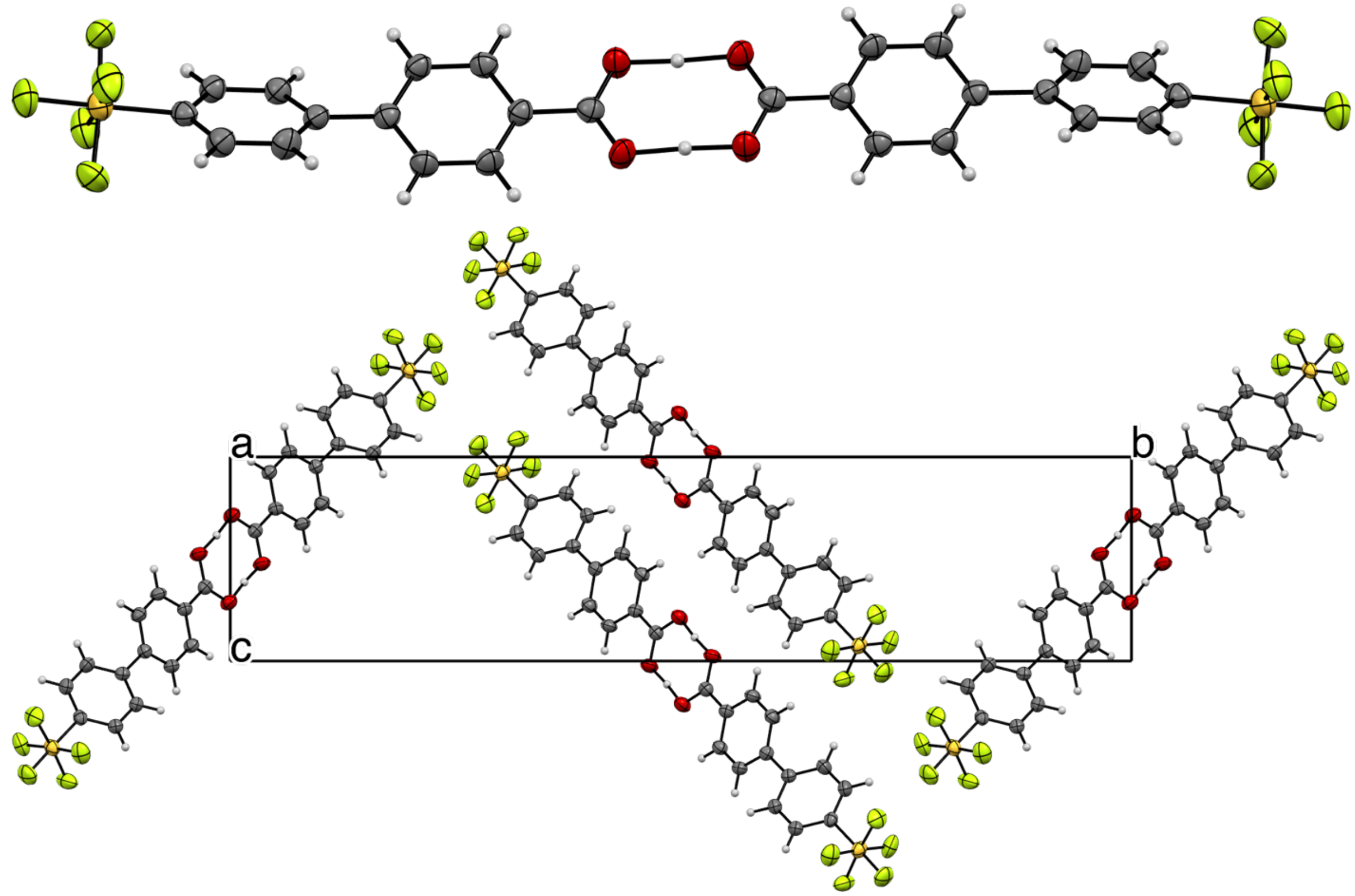

Figure S31. Molecular structure and packing of 4'-(pentafluoro λ^6 --sulfaneyl)-[1,1'-biphenyl]-4-carboxylic acid (**36**); ellipsoids are depicted at the 50% probability level.

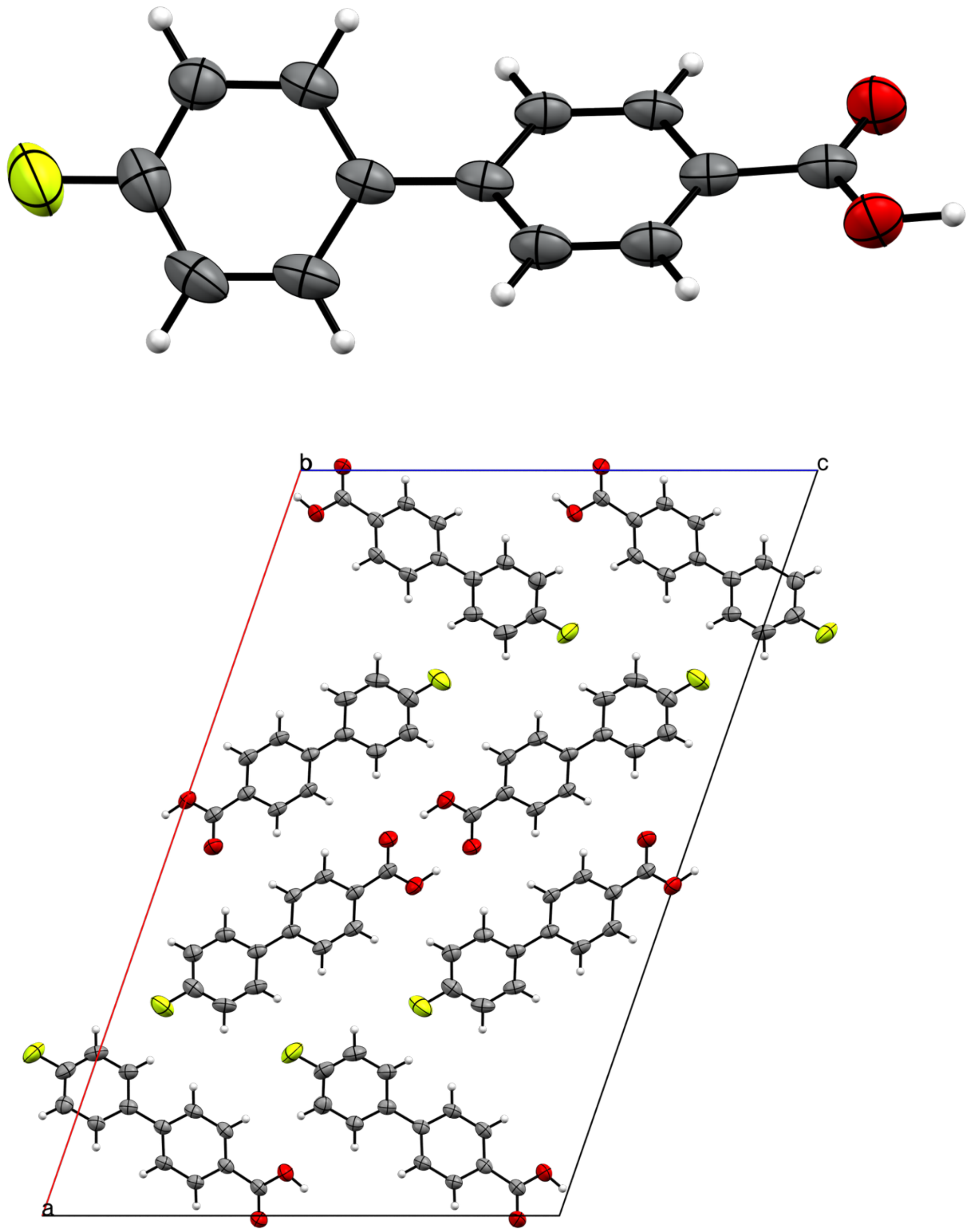

Figure S32. Molecular structure and packing of 4'-fluoro-[1,1'-biphenyl]-4-carboxylic acid (**34**); ellipsoids are depicted at the 50% probability level.

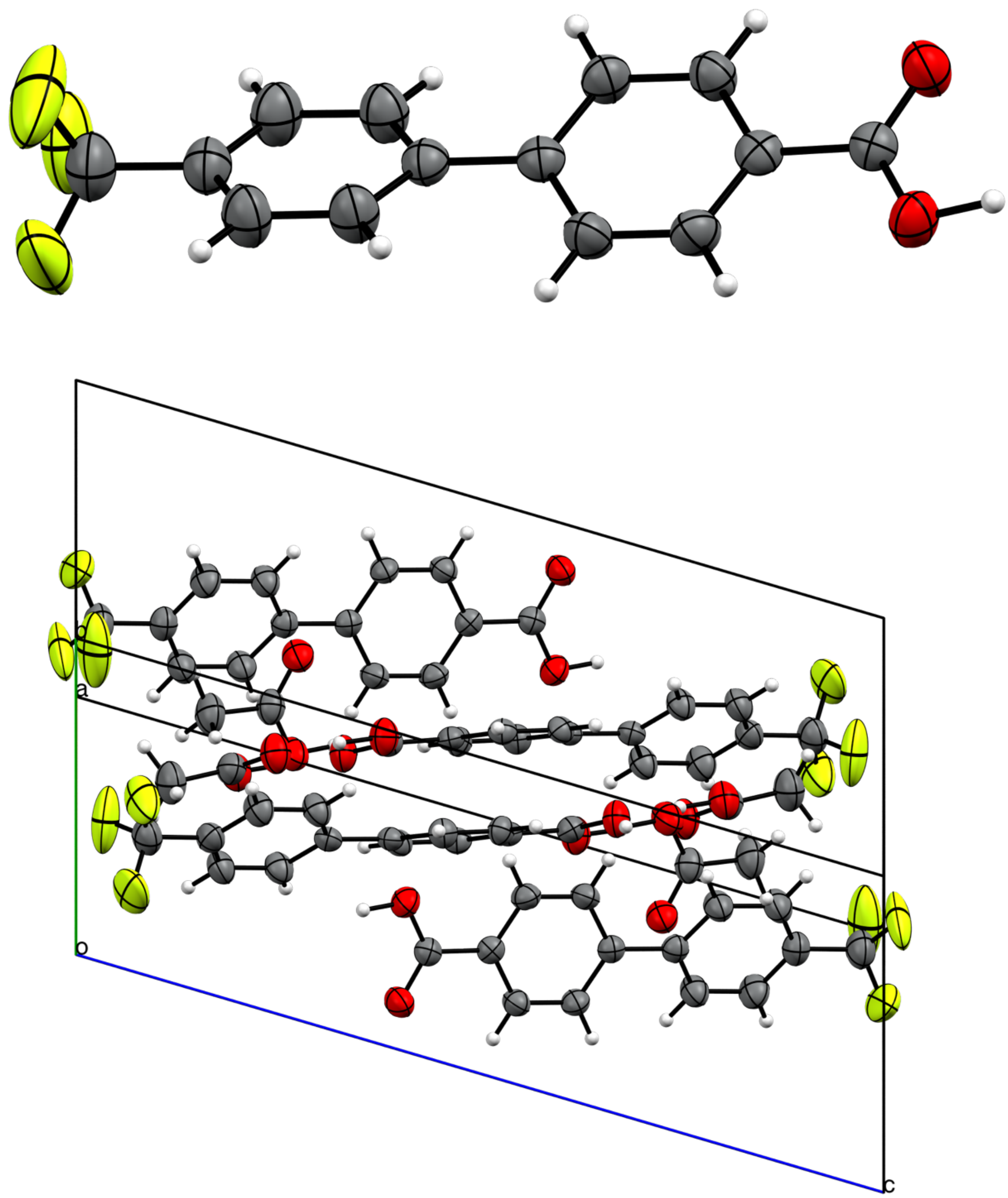

Figure S33. Molecular structure (co-crystallized acetic acid omitted for clarity) and packing of 4'-(trifluoromethyl)-[1,1'-biphenyl]-4-carboxylic acid (**35**); ellipsoids are depicted at the 50% probability level.

Structure Comparisons:

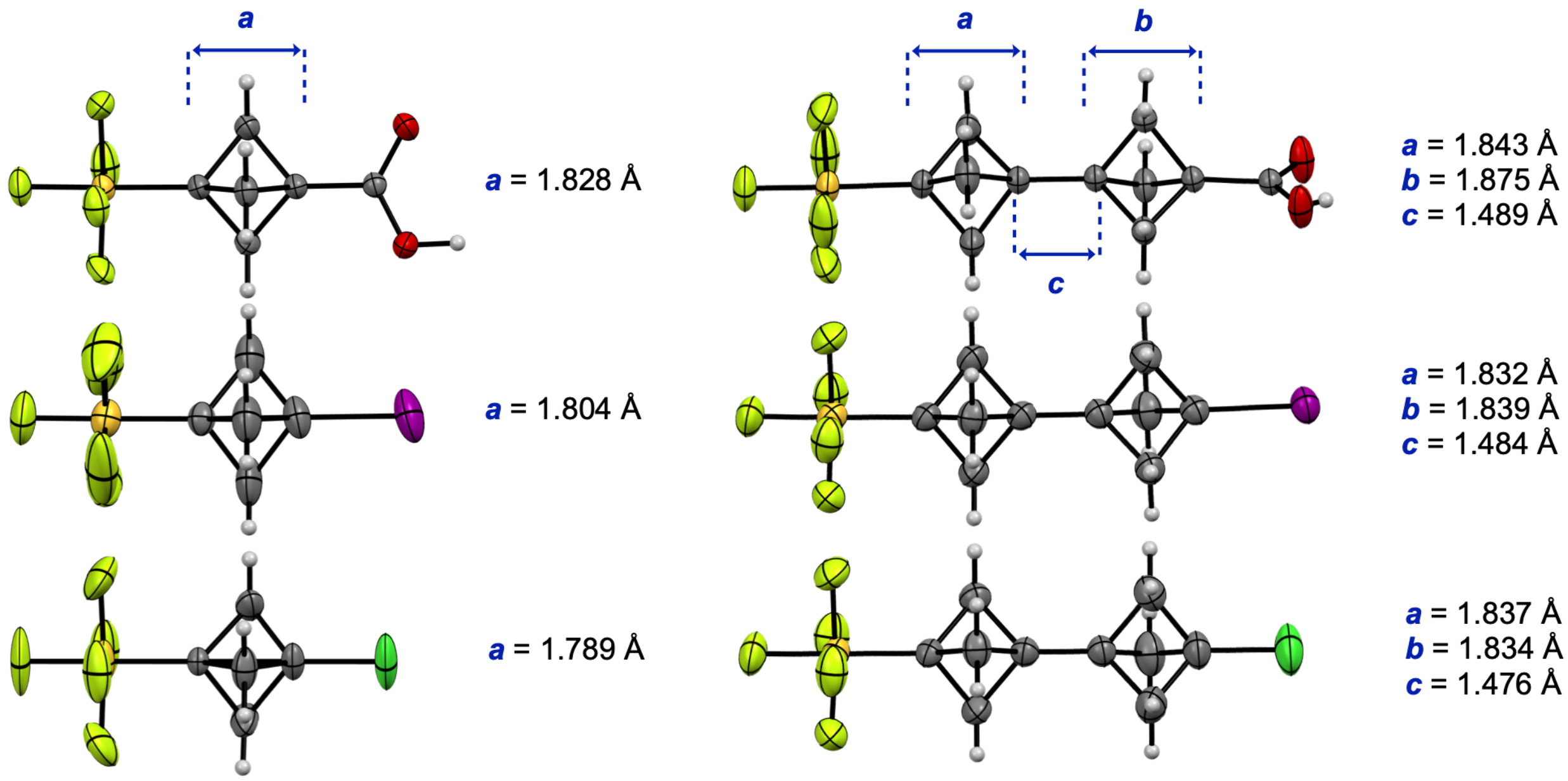

Figure S34. Comparison of select BCP bonding and non-bonding distances between SF_5-BCP-CO_2H (**4**), SF_5-BCP-I (CCDC: 2255639), SF_5-BCP-Cl (CCDC: 2195538) and SF_5-staffane-CO_2H (**6**), SF_5-staffane-I (**5**), SF_5-staffane-Cl (CCDC: 2383115). Ellipsoids are depicted at the 50 % probability level.

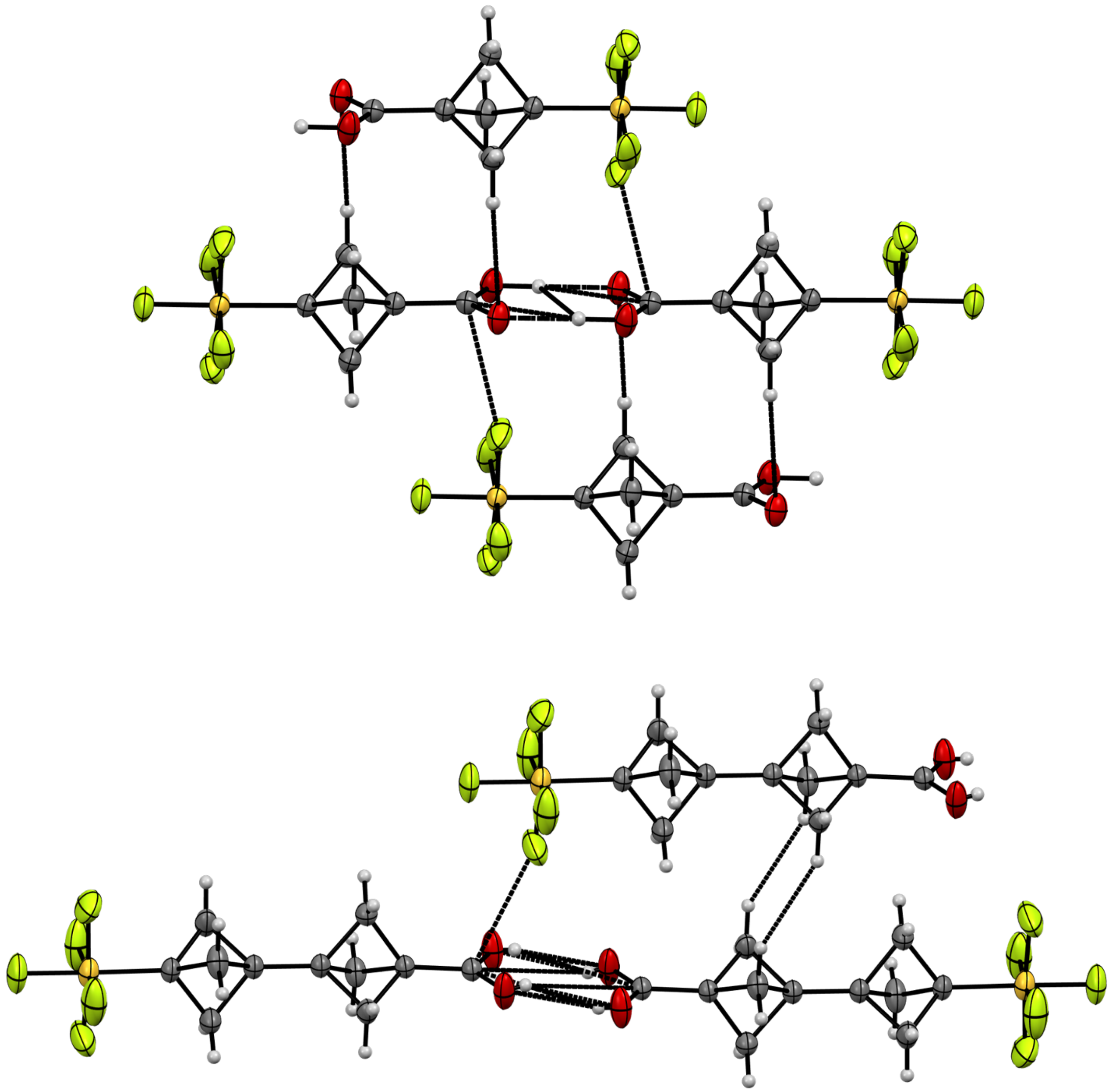

Figure S35. Comparison of the short contacts between SF_5-BCP-CO_2H (**4**, *top*) and SF_5-BCP-BCP-CO_2H (**6**, *bottom*). In both cases, a (likely weak) S–F···CO_2H interaction could be observed in addition to the expected carboxylic acid dimer.

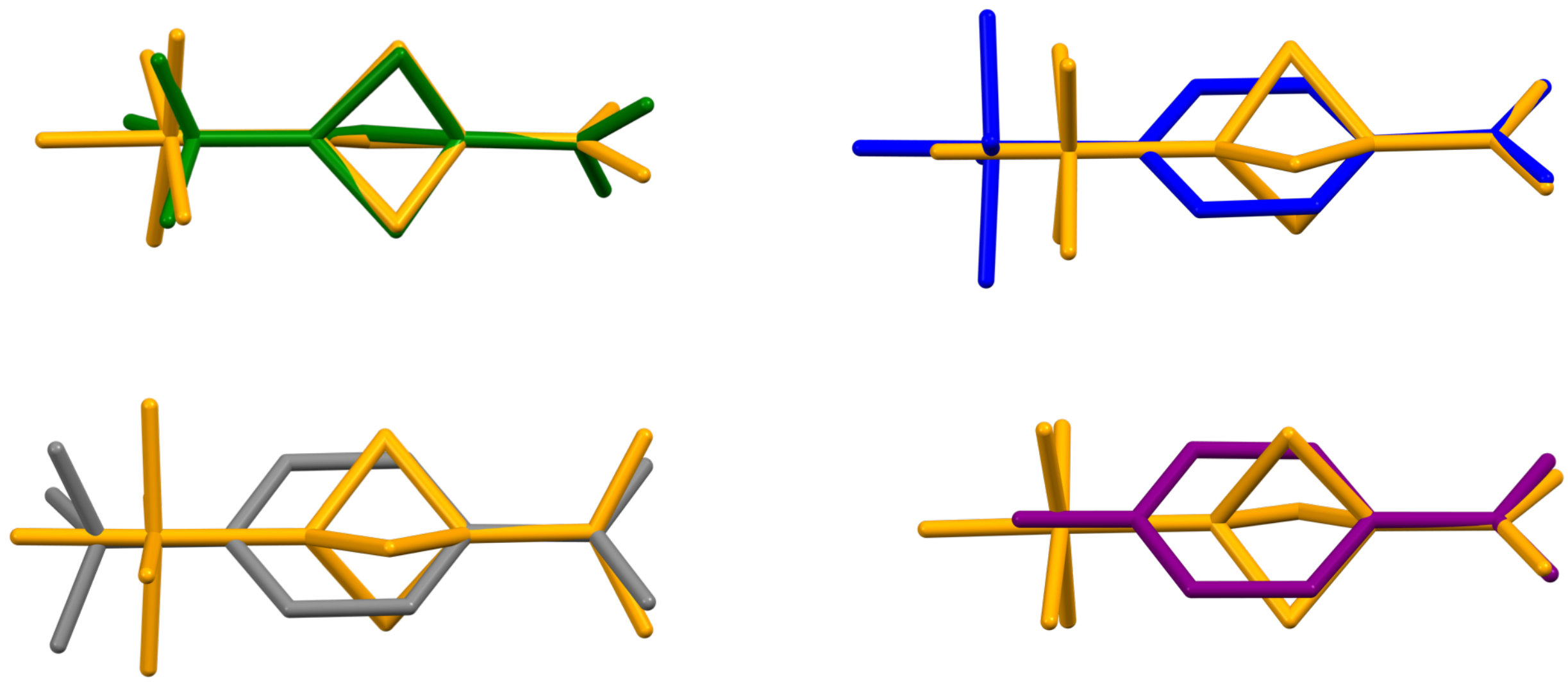

Figure S36. Overlays of SF_5-BCP-CO_2H (**4**, orange) and CF_3-BCP-CO_2H (**21**, green), SF_5-Ph-CO_2H (**24**, blue), CF_3-Ph-CO_2H (**23**, gray), F-Ph-CO_2H (**22**, purple). Hydrogen atoms are omitted for clarity.

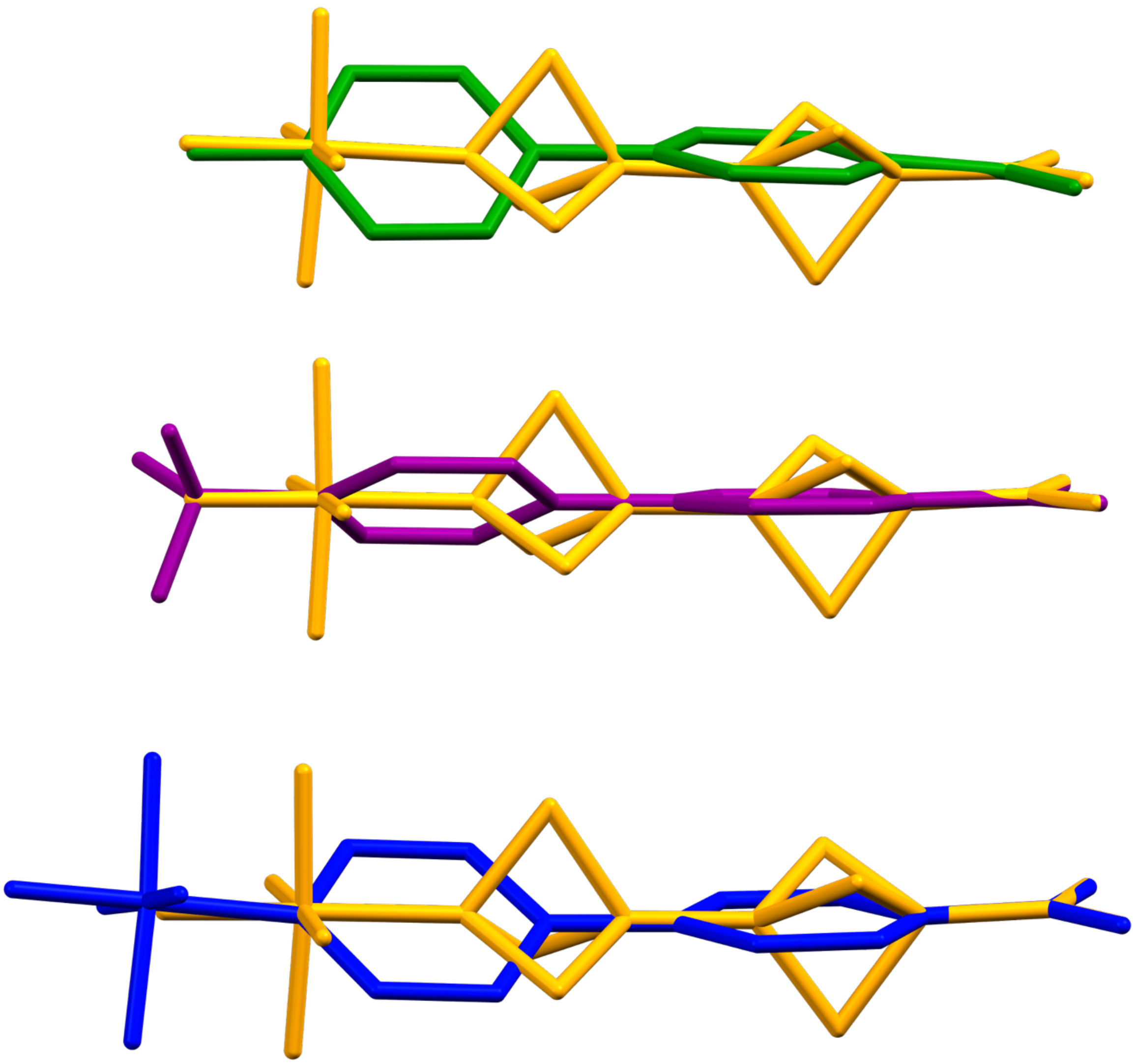

Figure S37. Overlay of SF_5-BCP-BCP-CO_2H (**6**, orange) and F-Ph-Ph-CO_2H (**34**, green) CF_3-Ph-Ph-CO_2H (**35**, purple), and SF_5-Ph-Ph-CO_2H (**36**, blue). Hydrogen atoms are omitted for clarity.

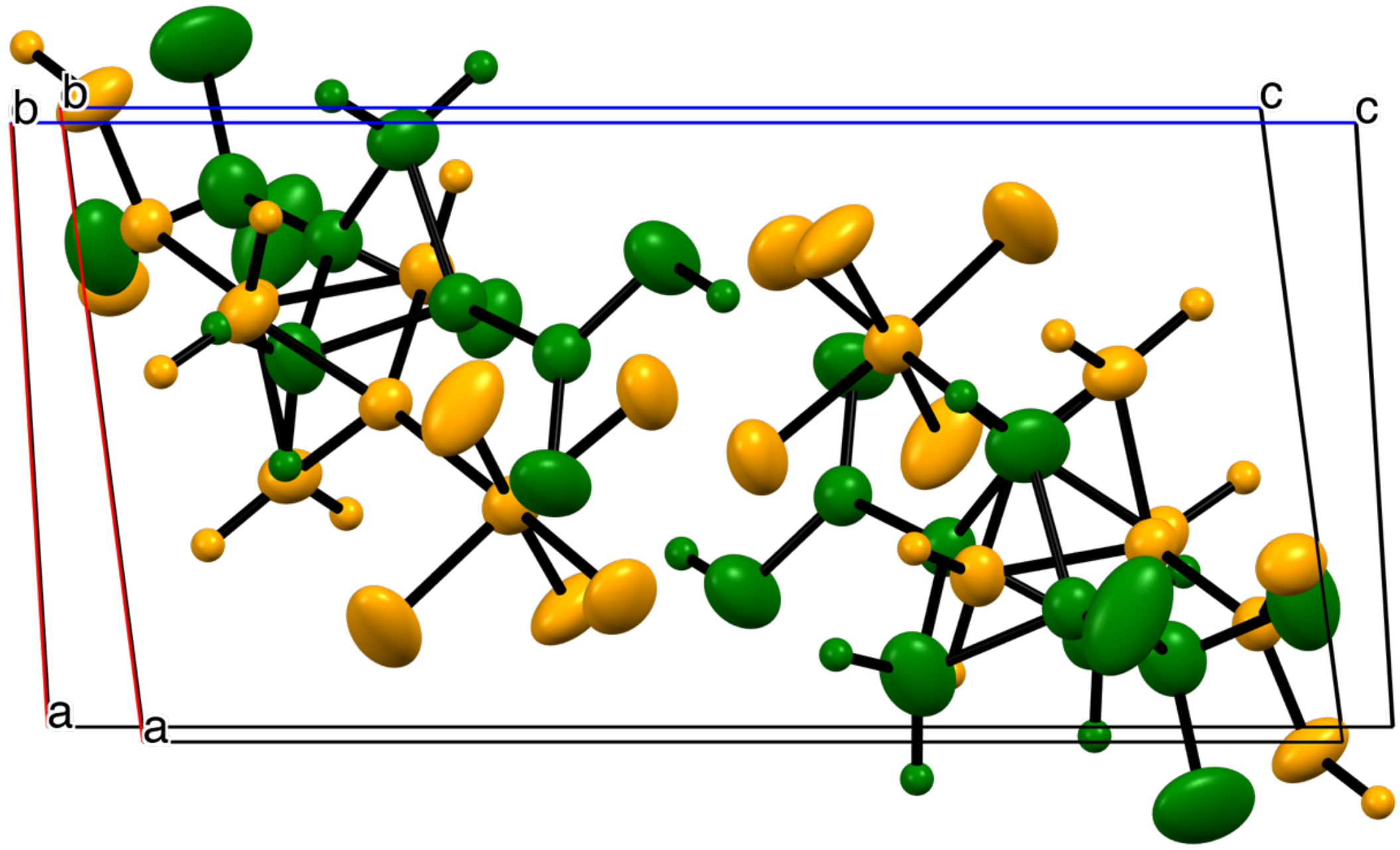

Figure S38. Overlay of the unit cells for SF_5-BCP-CO_2H (**3**, orange) and CF_3-BCP-CO_2H (**21**, green).

Computational Data

Geometry optimization and vibrational analysis were performed with the *Gaussian16* Revision C.01 suite of programs[58] utilizing the Chai and Head-Gordon's dispersion and long-range corrected hybrid exchange-correlation functional (ωB97X-D)[59] in conjunction with Ahlrichs' split valence polarized basis sets (def2-SVP) for all atoms.[60] Solvation effects were considered by the Integral Equation Formalism Polarization Continuum Model (IEFPCM)[61] in water (H_2O, ϵ = 78.36), dimethyl sulfoxide (DMSO, ϵ = 46.83), acetonitrile (MeCN, ϵ = 35.69), methanol (CH_3OH, ϵ = 32.61), spanning a broad range of dielectric constants. The *ORCA* 6.1.0 suite of programs[62] allows manual and automated conformational searching (using GOAT algorithm[63] with GFN2-xTB[64] method). Conformational searching was performed; herein only transition structures and minima of the lowest energy conformers are reported.

Transition structures were verified to possess exactly one vibrational mode with an imaginary frequency and by subsequent Intrinsic Reaction Coordinate calculations[65,66] and/or optimization following displacement alongside the imaginary vibrational mode. Upon these validated structures with "raw" electronic energies (E_{solv}), free energy corrections at 1M and 298.15 K with the quasi-harmonic approximation proposed by Grimme[67] with 100 cm^{-1} cutoffs were calculated with the Goodvibes package,[68] affording the final free energies (G_{solv}) reported in the main text.

Natural Bonding Orbitals and second order perturbation theory analysis of Fock matrix were performed using NBO 7.0[69,70] at ωB97X-D/def2-SVP level of theory. Dataset of optimized geometries available at the ioChem-BD repository[71] *via* [http://dx.doi.org/10.19061/iochem-bd-6-671].

Table S16. Electronic energies (E_{solv}) and free energies (G_{solv}) of the reactants, intermediates, transition structures, and products in **Figure S27** for methanol and acetonitrile. Results for water reported also.

**Structure**	***E_{solv}* (H₂O)**	***G_{solv}* (H₂O)**
SF_5-BCP **42**	-1574.74551	-1574.52655
SF_5-BCP-TS	-1574.68396	-1574.47369
SF_5-BCP-Cation	-677.88034	-677.67634
SF_5-[2]Staffane **43**	-1768.63429	-1768.32242
SF_5-[2]Staffane-TS	-1768.57477	-1768.27088
SF_5-[2]Staffane-Cation	-871.77091	-871.47728
**SF_5^- anion**	-896.80759	-896.81928
	E_{solv} (MeCN)	***G_{solv} (MeCN)***
SF_5-BCP **42**	-1574.74530	-1574.52639
SF_5-BCP-TS	-1574.68306	-1574.47247
SF_5-BCP-Cation	-677.87905	-677.67516
SF_5-[2]Staffane **43**	-1768.63407	-1768.32213
SF_5-[2]Staffane-TS	-1768.57379	-1768.27063
SF_5-[2]Staffane-Cation	-871.76963	-871.47567
**SF_5^- anion**	-896.80620	-896.81793
	E_{solv} (CH_3OH)	***G_{solv} (CH_3OH)***
SF_5-BCP **42**	-1574.74526	-1574.52636
SF_5-BCP-TS	-1574.68291	-1574.47227
SF_5-BCP-Cation	-1574.68291	-677.67495
SF_5-[2]Staffane **43**	-1768.63403	-1768.32209
SF_5-[2]Staffane-TS	-1768.57368	-1768.26958
SF_5-[2]Staffane-Cation	-871.76941	-871.47541
**SF_5^- anion**	-896.80596	-896.81770

Table S17. Electronic energies (E_{solv}) and free energies (G_{solv}) of the reactants, intermediates, transition structures, and products in **Figure S27** for DMSO and autocatalytic (HF-mediated) hypothesis.

**Structure**	***E_{solv} (DMSO)***	***G_{solv} (DMSO)***
SF_5-BCP **42**	-1574.74539	-1574.52646
SF_5-BCP-TS	-1574.68345	-1574.47300
SF_5-BCP-TS-HF	-1675.04362	-1674.82266
SF_5-BCP-Cation	-677.87961	-677.67569
SF_5-[2]Staffane **43**	-1768.63416	-1768.32225
SF_5-[2]Staffane-TS	-1768.57420	-1768.27083
SF_5-[2]Staffane-TS-HF	-1868.93480	-1868.62076
SF_5-[2]Staffane-Cation	-871.77019	-871.47634
**SF_5^- anion**	-896.80681	-896.81852
**HF**	-100.33506	-100.33904

Figure S38. Plots of Intrinsic Reaction Coordinates connecting various intermediates *via* their respective TSs, including geometries at the end points. Atom color code: C = grey, H = white, F = cyan, O = red, S = yellow. (Below)

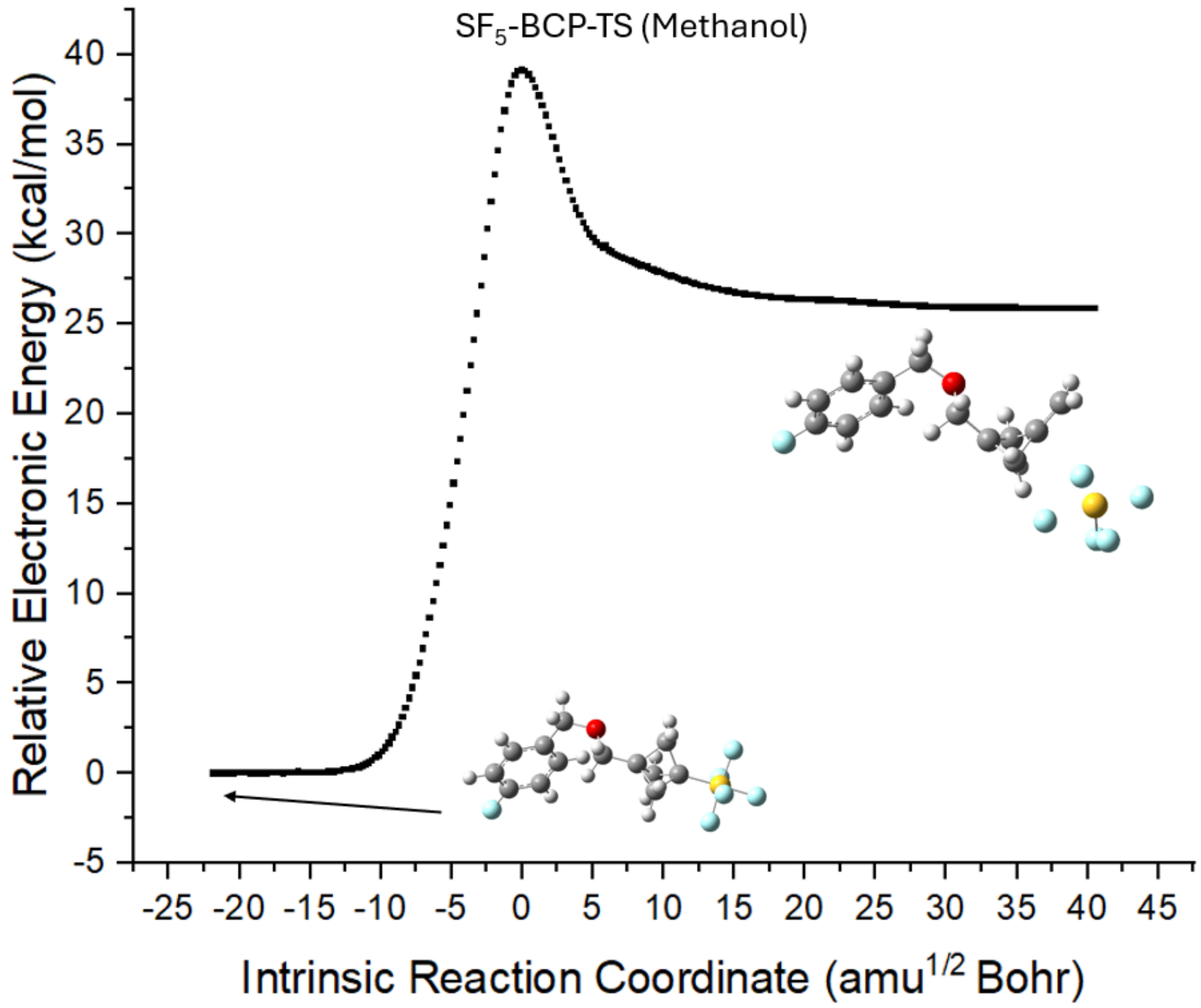

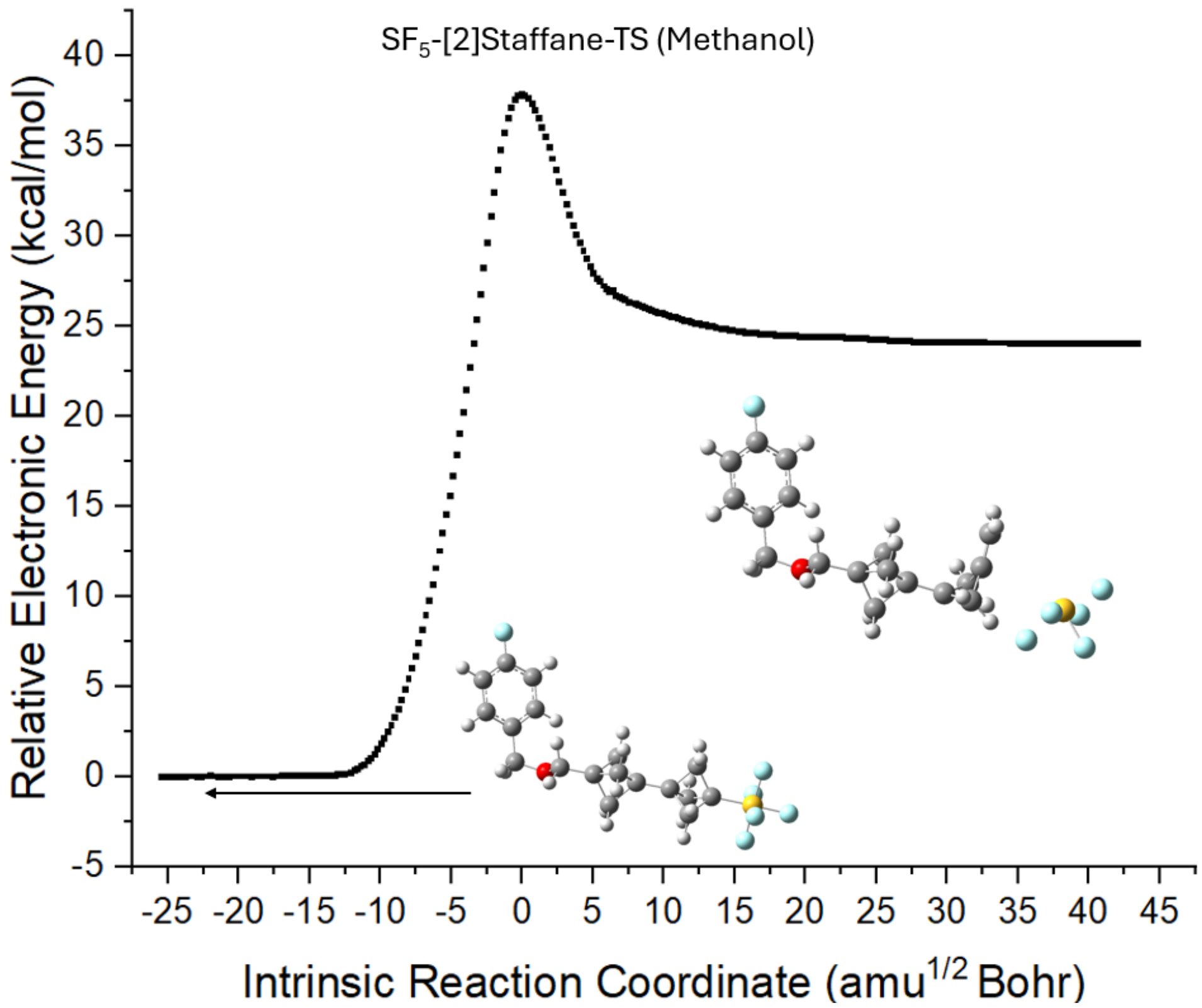
SF$_5$-[2]Staffane-TS (Methanol)
Relative Electronic Energy (kcal/mol)
Intrinsic Reaction Coordinate (amu$^{1/2}$ Bohr)

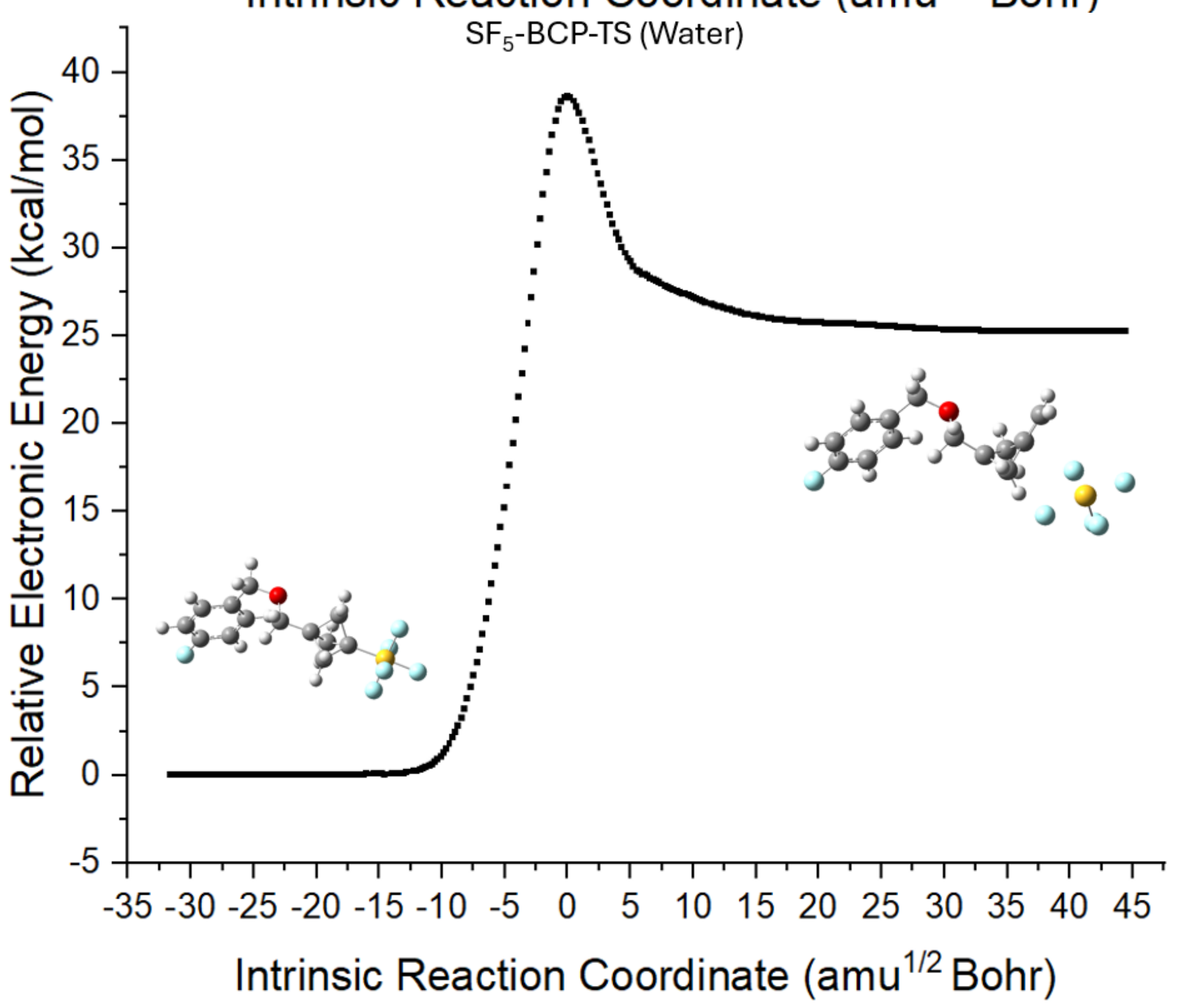
SF$_5$-BCP-TS (Water)
Relative Electronic Energy (kcal/mol)
Intrinsic Reaction Coordinate (amu$^{1/2}$ Bohr)

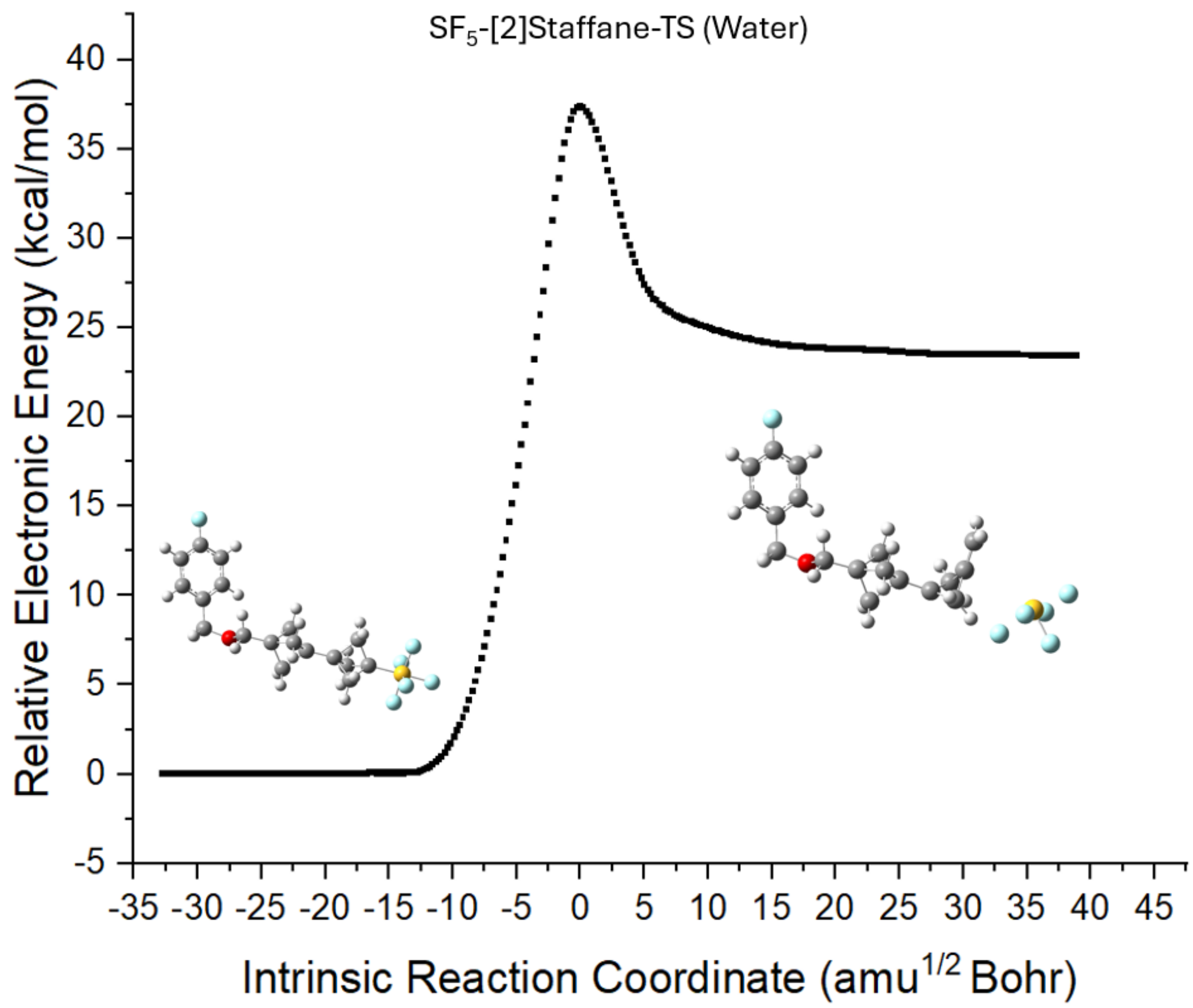
SF5-[2]Staffane-TS (Water)
Relative Electronic Energy (kcal/mol)
Intrinsic Reaction Coordinate (amu1/2 Bohr)
-5
0
5
10
15
20
25
30
35
40
-35 -30 -25 -20 -15 -10 -5 0 5 10 15 20 25 30 35 40 45

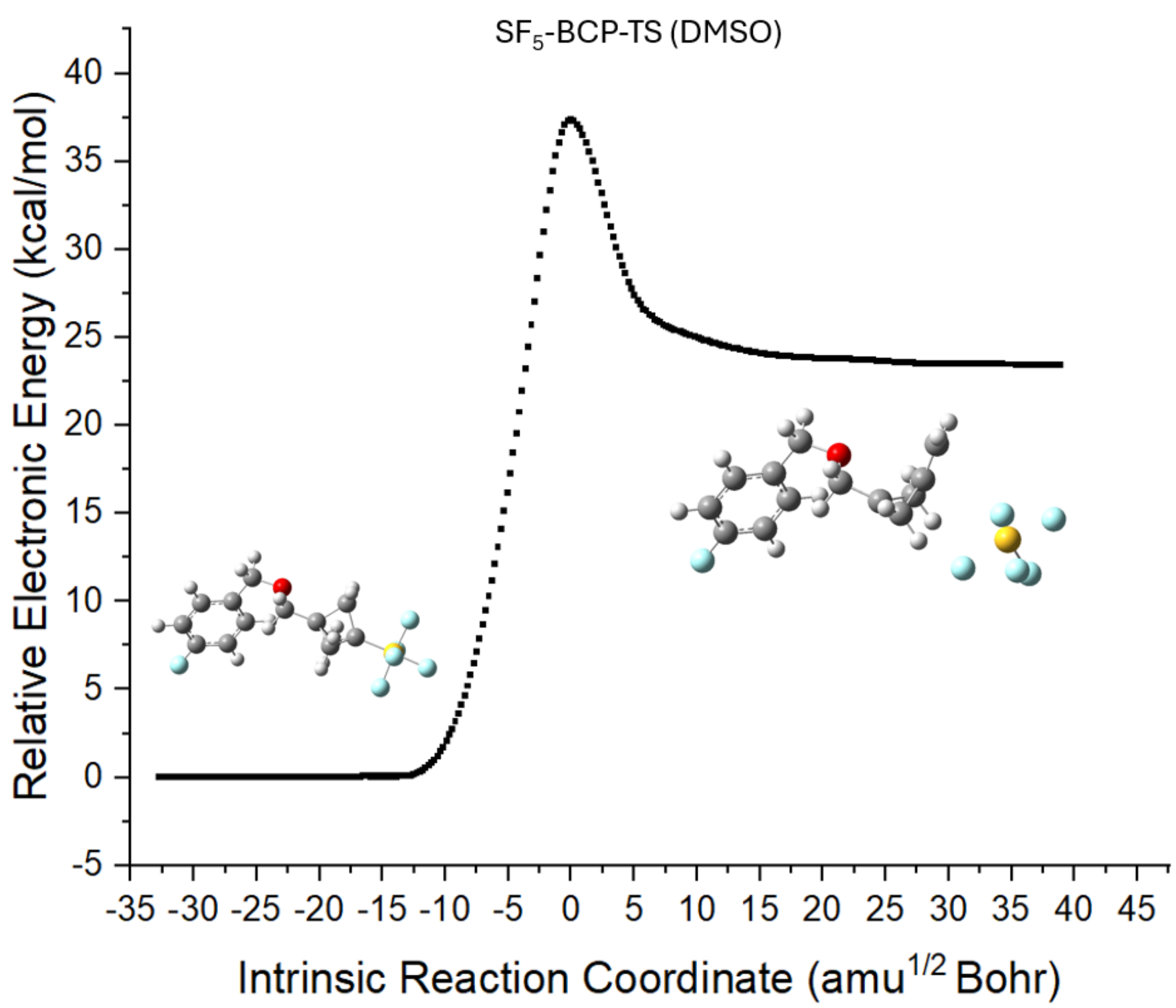
SF5-BCP-TS (DMSO)
Relative Electronic Energy (kcal/mol)
Intrinsic Reaction Coordinate (amu1/2 Bohr)
-5
0
5
10
15
20
25
30
35
40
-35 -30 -25 -20 -15 -10 -5 0 5 10 15 20 25 30 35 40 45

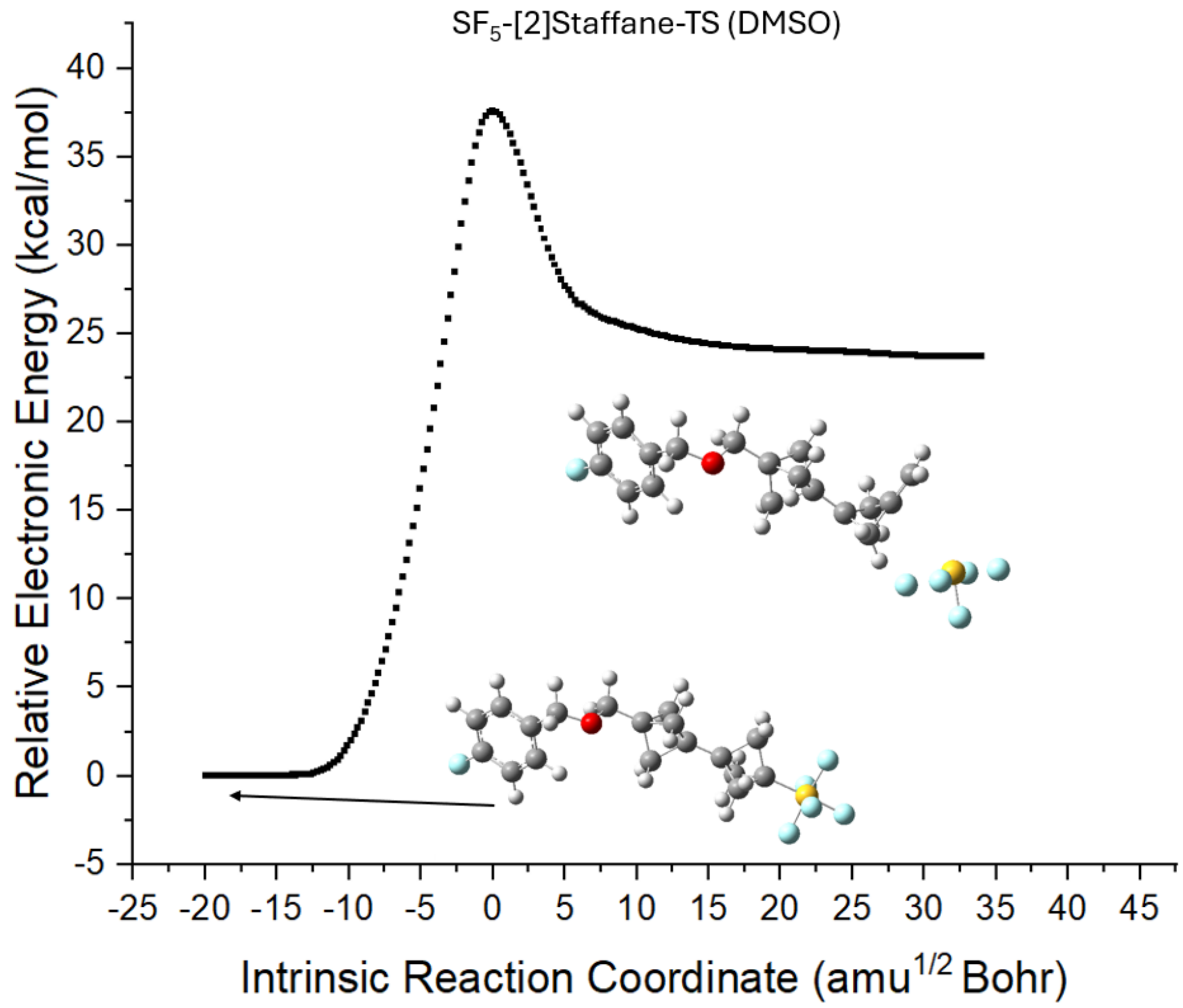
SF5-[2]Staffane-TS (DMSO)
Relative Electronic Energy (kcal/mol)
Intrinsic Reaction Coordinate (amu1/2 Bohr)

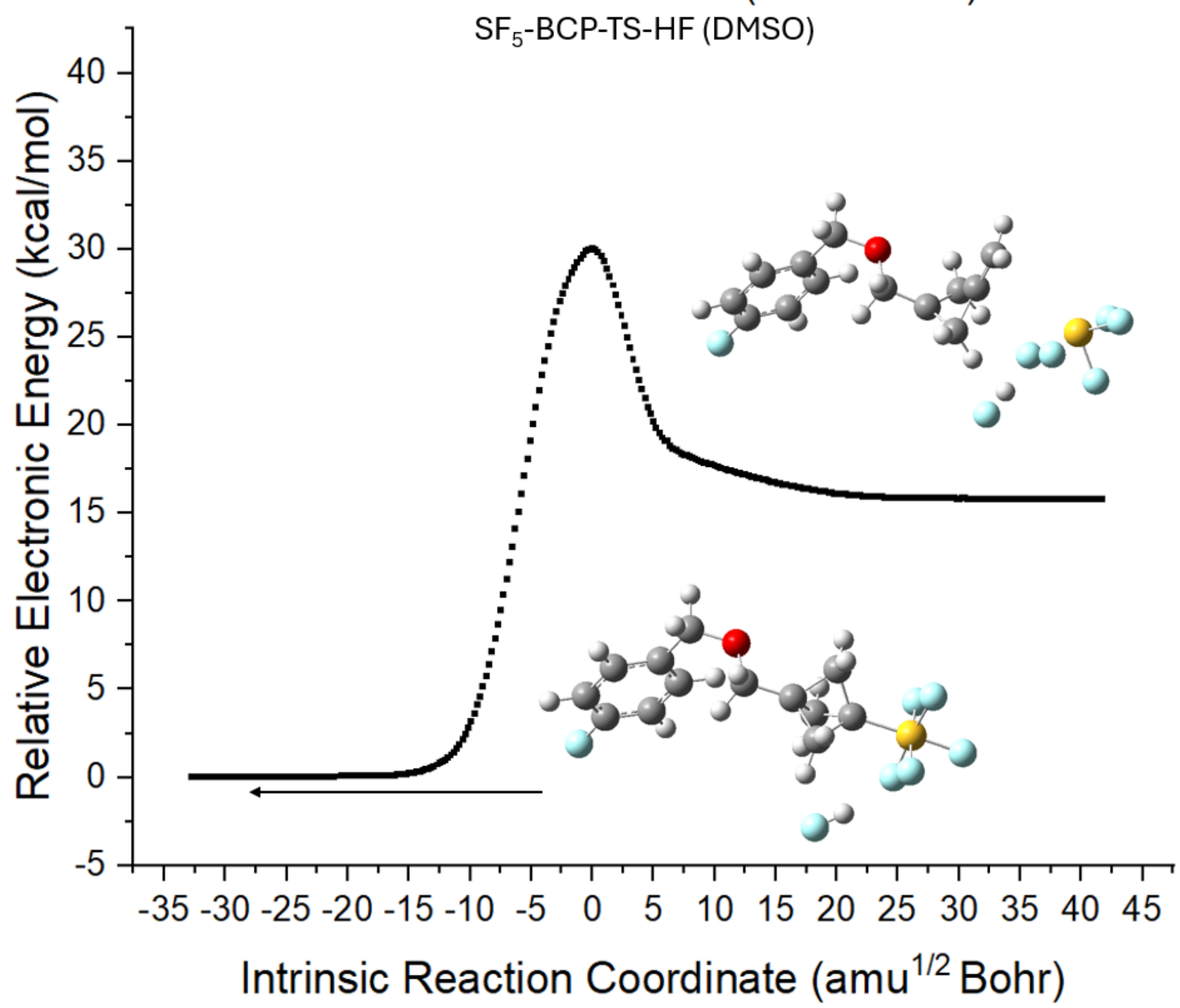
SF5-BCP-TS-HF (DMSO)
Relative Electronic Energy (kcal/mol)
Intrinsic Reaction Coordinate (amu1/2 Bohr)

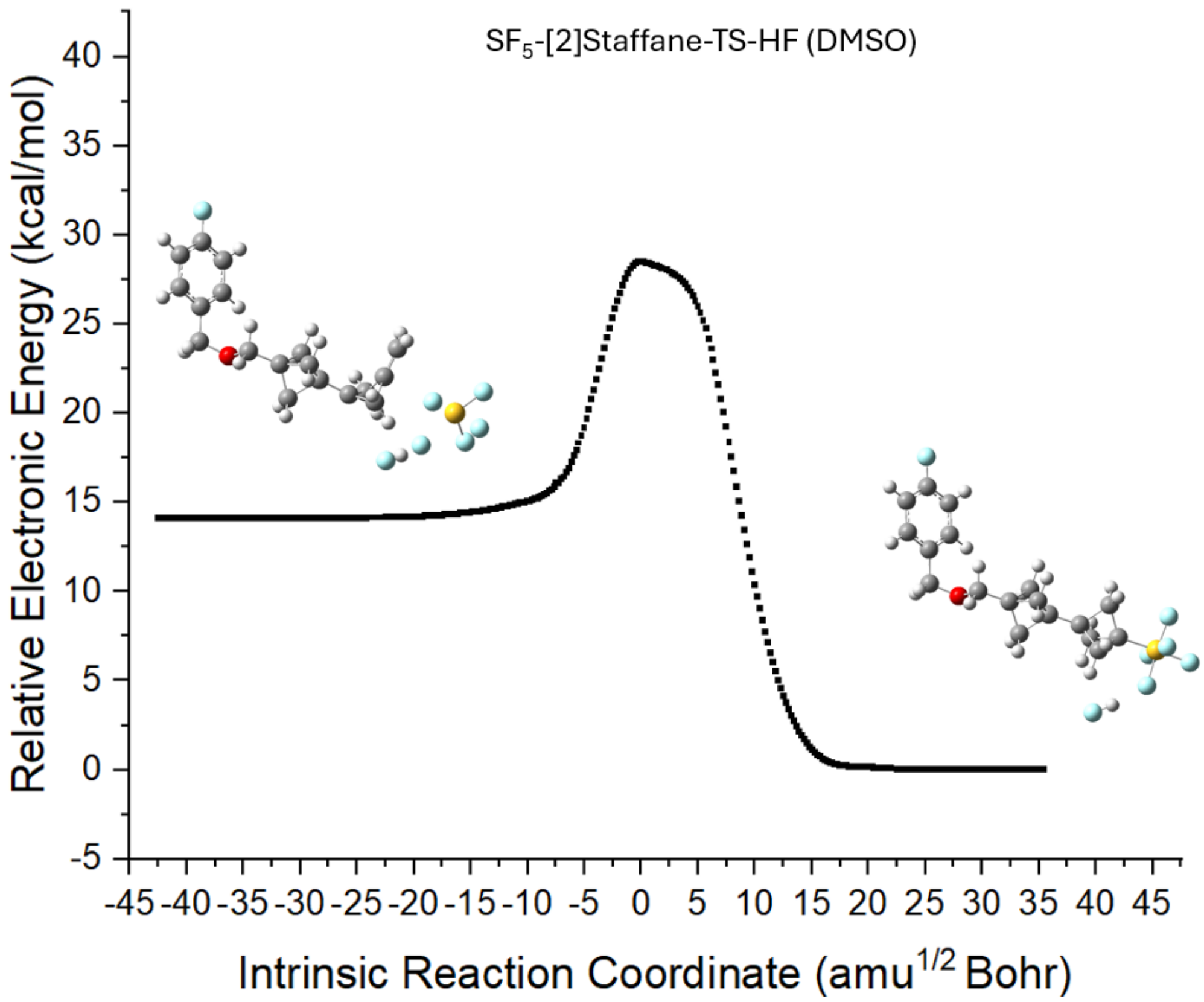

SF$_5$-[2]Staffane-TS-HF (DMSO)
Relative Electronic Energy (kcal/mol)
40
35
30
25
20
15
10
5
0
-5
-45 -40 -35 -30 -25 -20 -15 -10 -5 0 5 10 15 20 25 30 35 40 45
Intrinsic Reaction Coordinate (amu$^{1/2}$ Bohr)

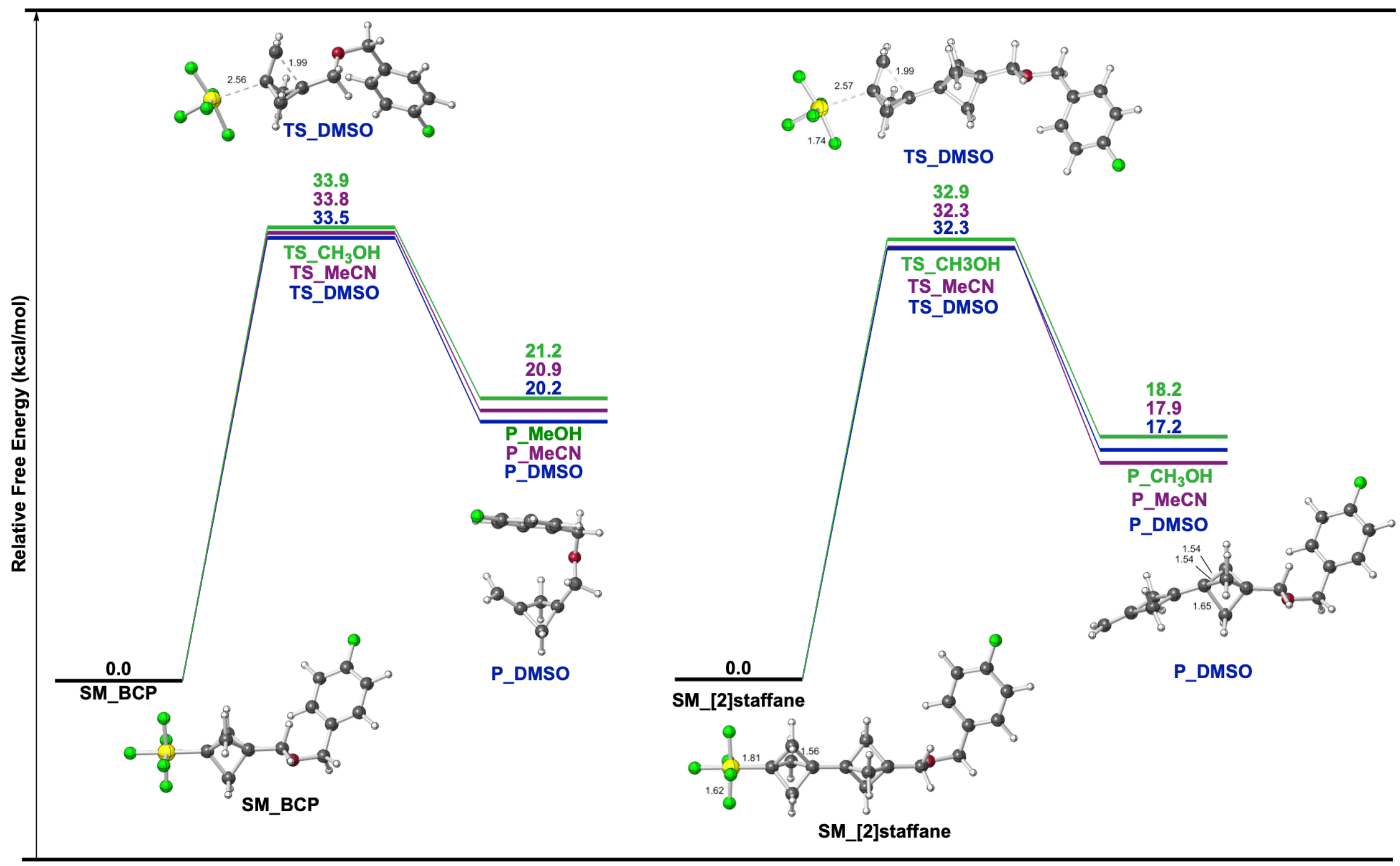

Figure S39. Relative Free energy profiles showing comparison of the SF_5 ionization step for SF_5–BCP **42** and SF_5–[2]staffane **43** at the PCM(DMSO)-ωB97X-D/def2-SVP level of theory. Demonstrate that a decrease in barrier height is inversely proportional to the dielectric constant of the solvent. This may explain why ionization is more favorable in polar DMSO (ε = 47.2 at 20 °C) than in MeCN (ε = 37.5) or MeOH (ε = 33.6); note that the corresponding computed barriers for the SF_5–[2]staffane **43** (in kcal/mol) are 32.27, 32.32, and 32.95. This trend holds for the SF_5–BCP **42**: (in kcal/mol) 33.55, 33.84, and 33.95.

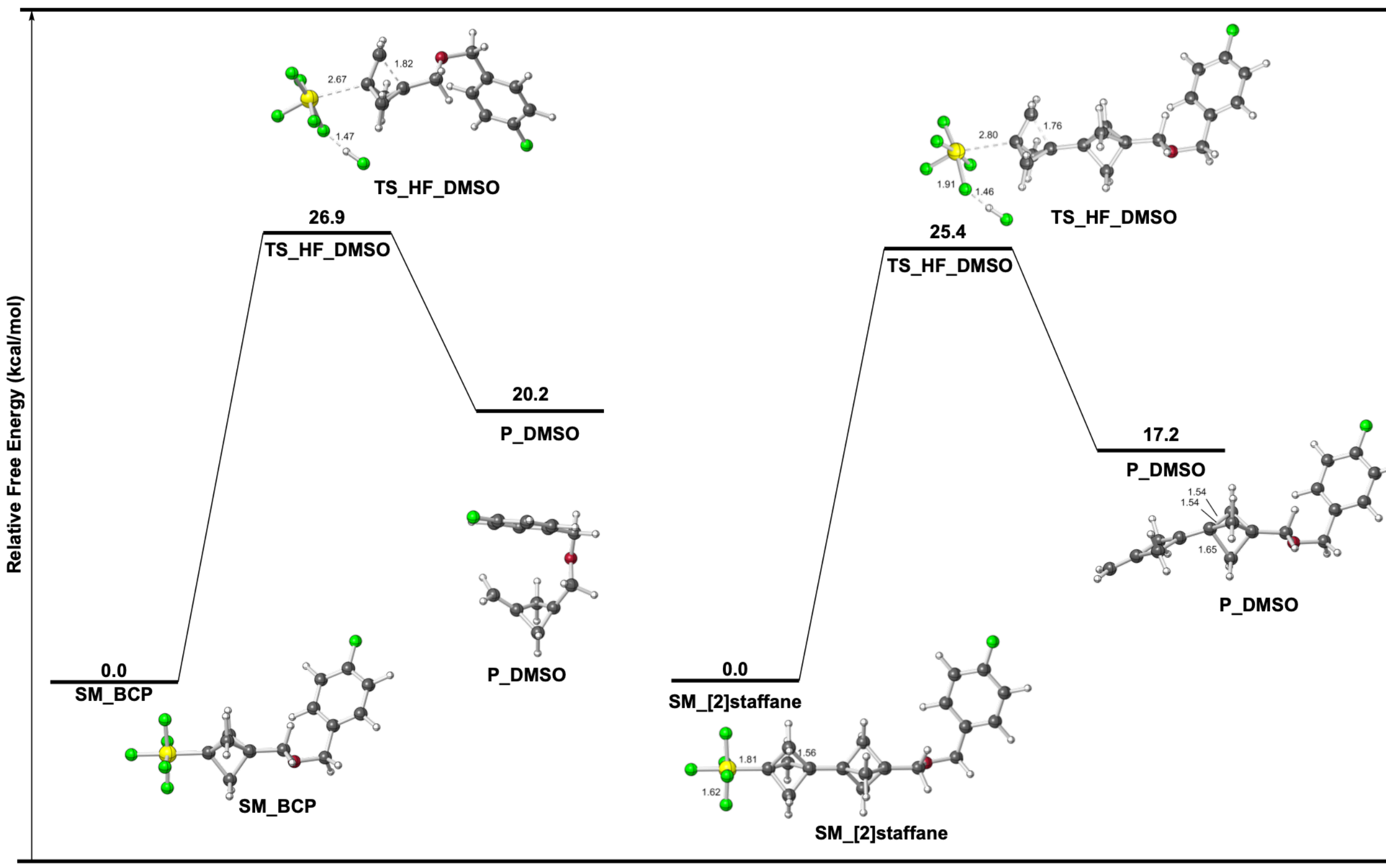

Figure S40. Relative Free energy profiles showing comparison of the SF_5 ionization step for SF_5–BCP **42** and SF_5–[2]staffane **43** at the PCM(DMSO)-ωB97X-D/def2-SVP level of theory. The ionization TSs (with HF) lowers the activation energy barriers significantly for both the SF_5–[2]staffane (from $\Delta G^{\ddagger}$ = 32.3 kcal/mol to 25.4 kcal/mol; TS_HF_DMSO) and the SF_5–BCP (from $\Delta G^{\ddagger}$ = 33.6 kcal/mol to 26.9 kcal/mol; TS_HF_DMSO).

NMR Spectra

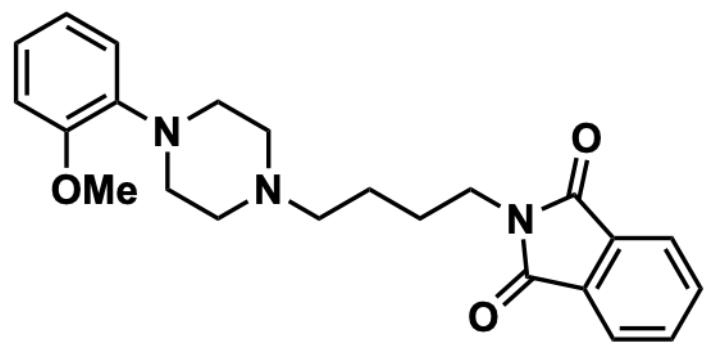

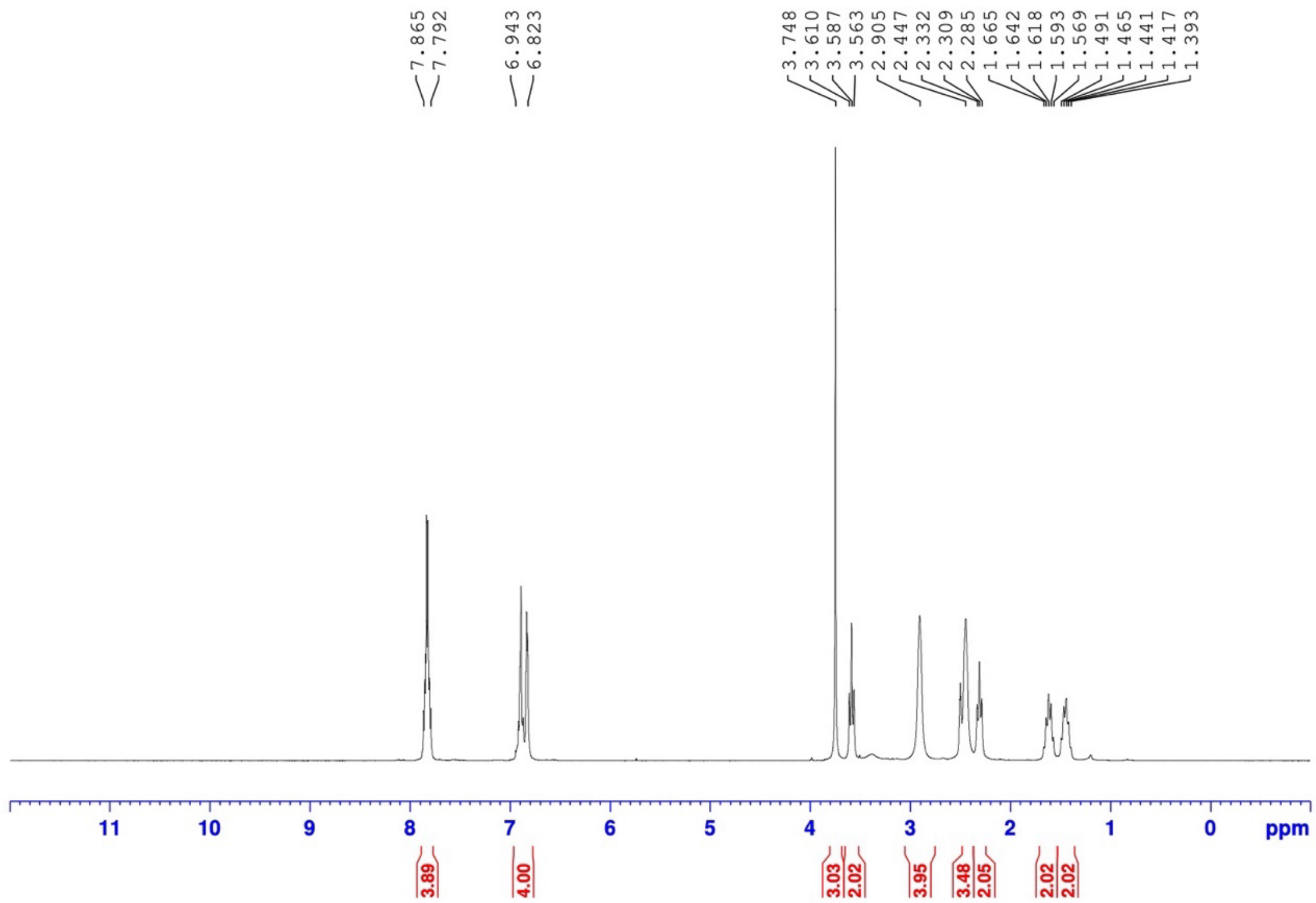

Figure S41. ^{1}H NMR spectrum of 2-(4-(4-(2-methoxyphenyl)piperazin-1-yl)butyl)isoindoline-1,3-dione **(S-1**) in DMSO-d_6.

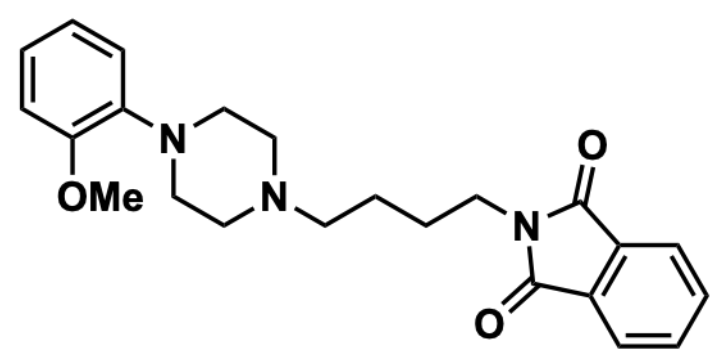

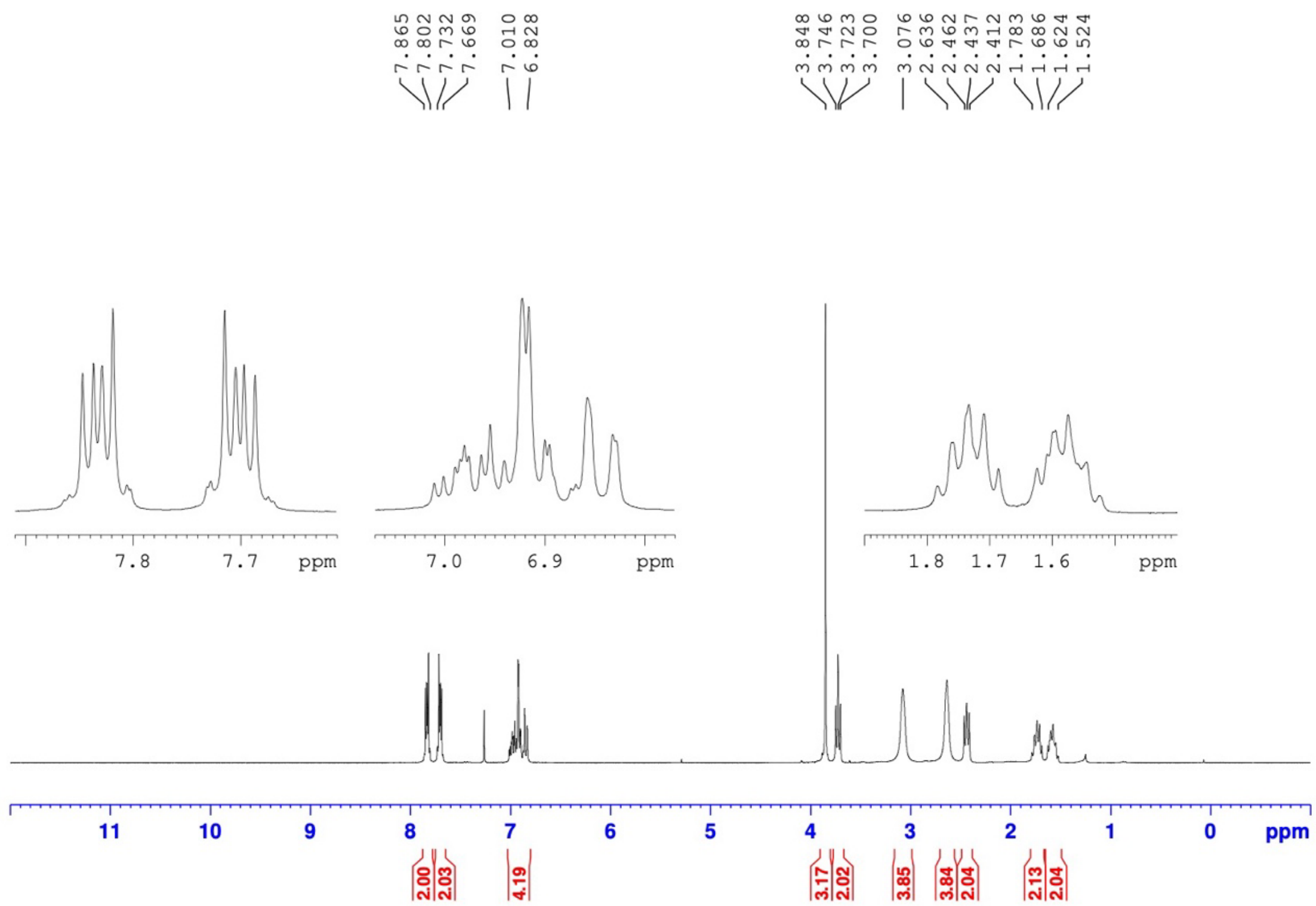

Figure S42. ^{1}H NMR spectrum of 2-(4-(4-(2-methoxyphenyl)piperazin-1-yl)butyl)isoindoline-1,3-dione (**S-1**) in $CDCl_3$.

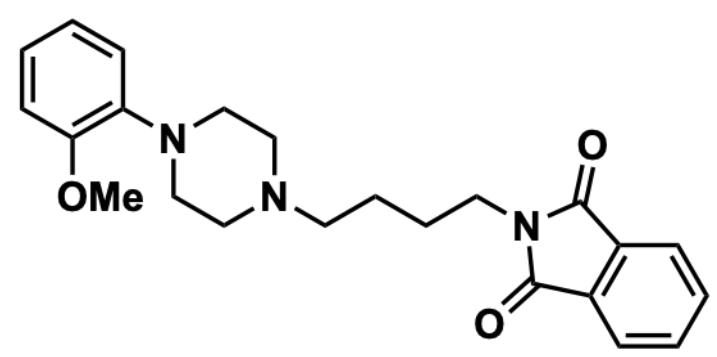

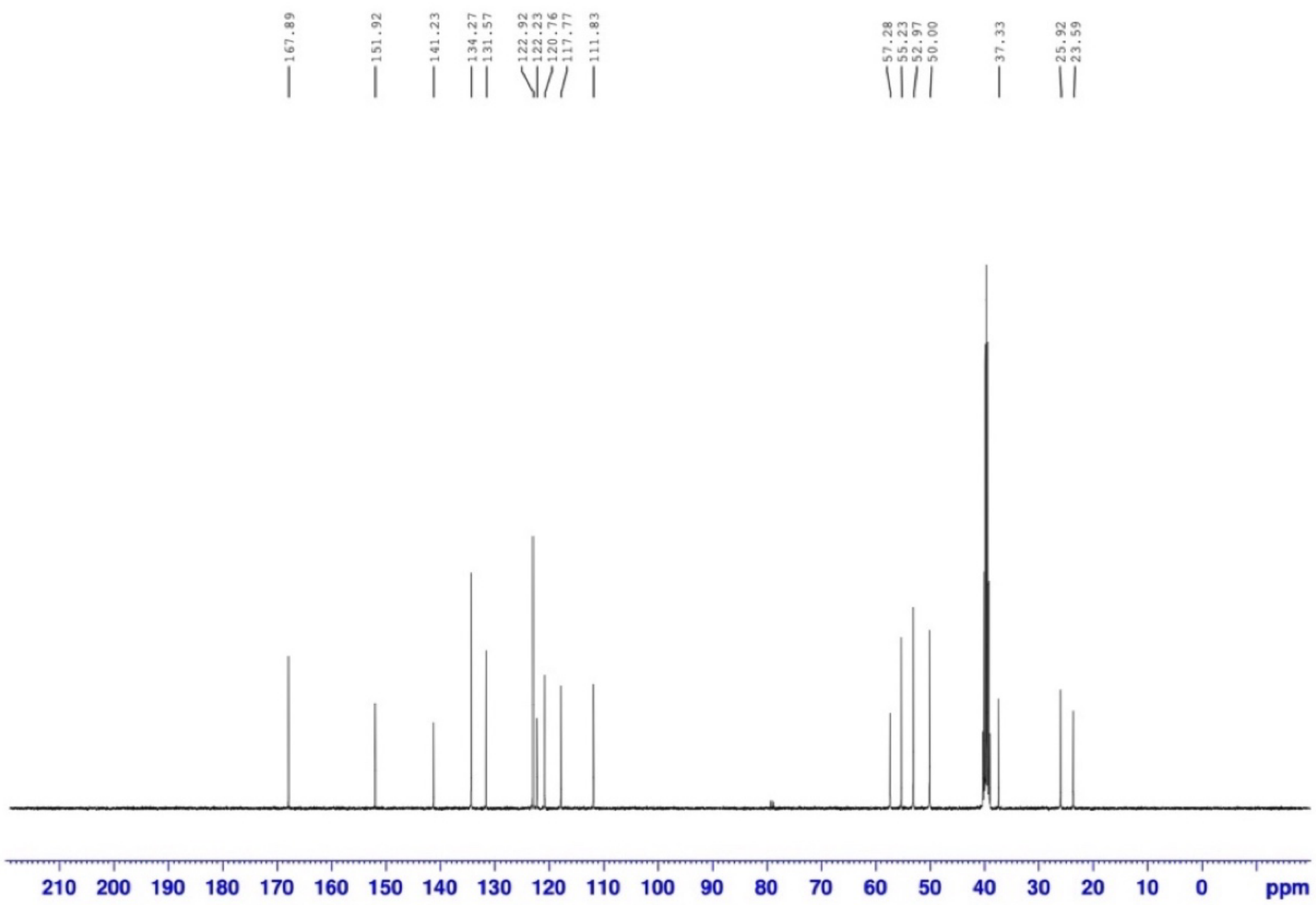

Figure S43. $^{13}C\{^1H\}$ NMR spectrum of 2-(4-(4-(2-methoxyphenyl)piperazin-1-yl)butyl)isoindoline-1,3-dione (**S-1**) in DMSO-d_6.

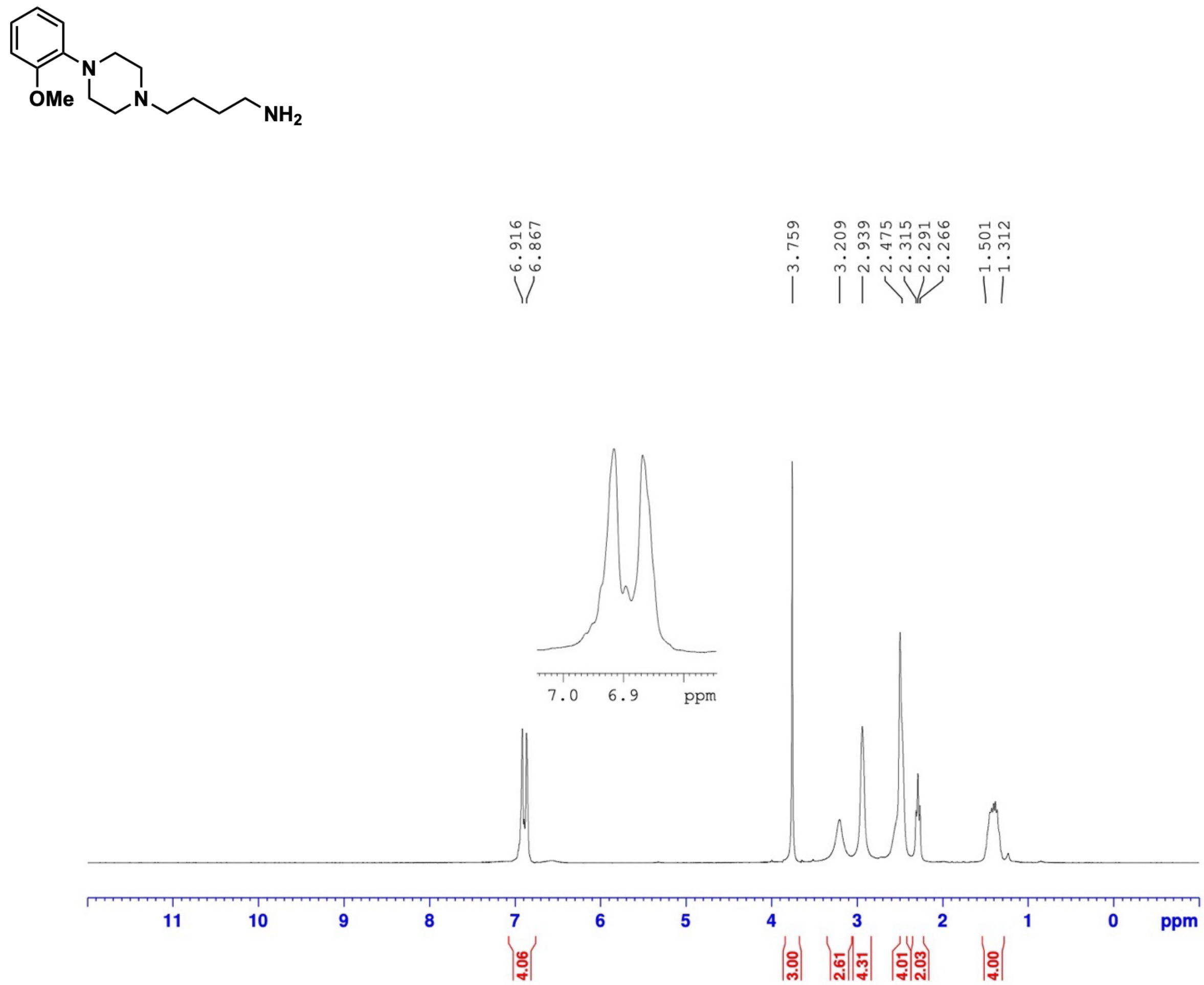

Figure S44. ^{1}H NMR spectrum of 4-(4-(2-methoxyphenyl)piperazin-1-yl)butan-1-amine (**S-2**) in DMSO-d_6.

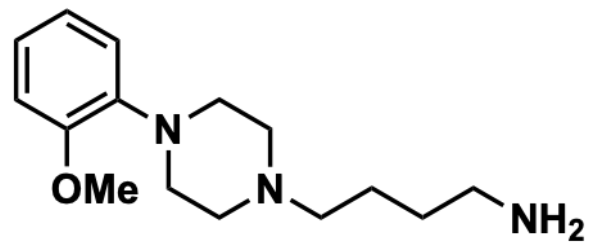

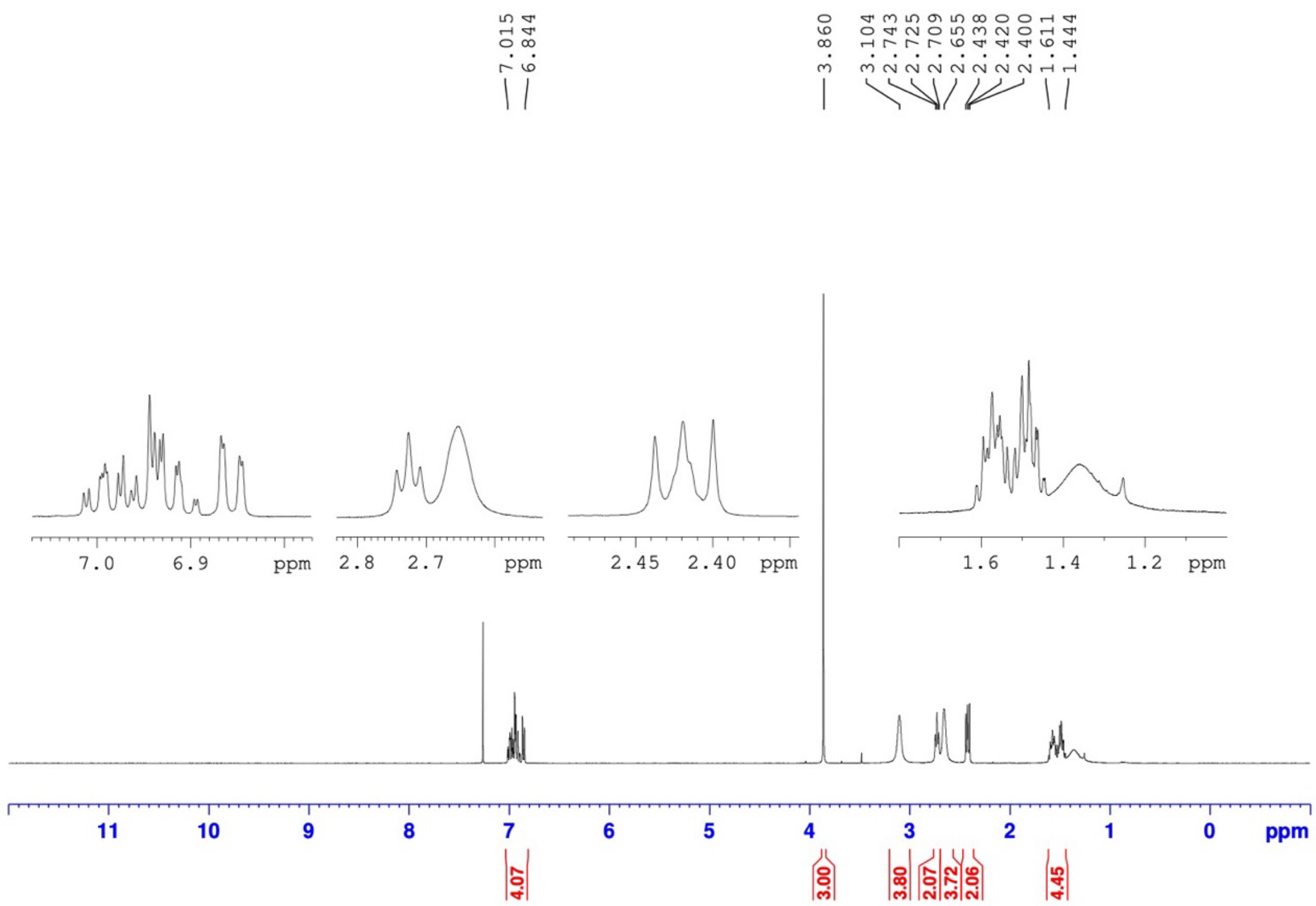

Figure S45. ^{1}H NMR spectrum of 4-(4-(2-methoxyphenyl)piperazin-1-yl)butan-1-amine (**S-2**) in $CDCl_3$.

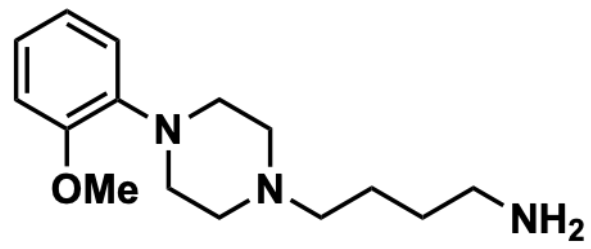

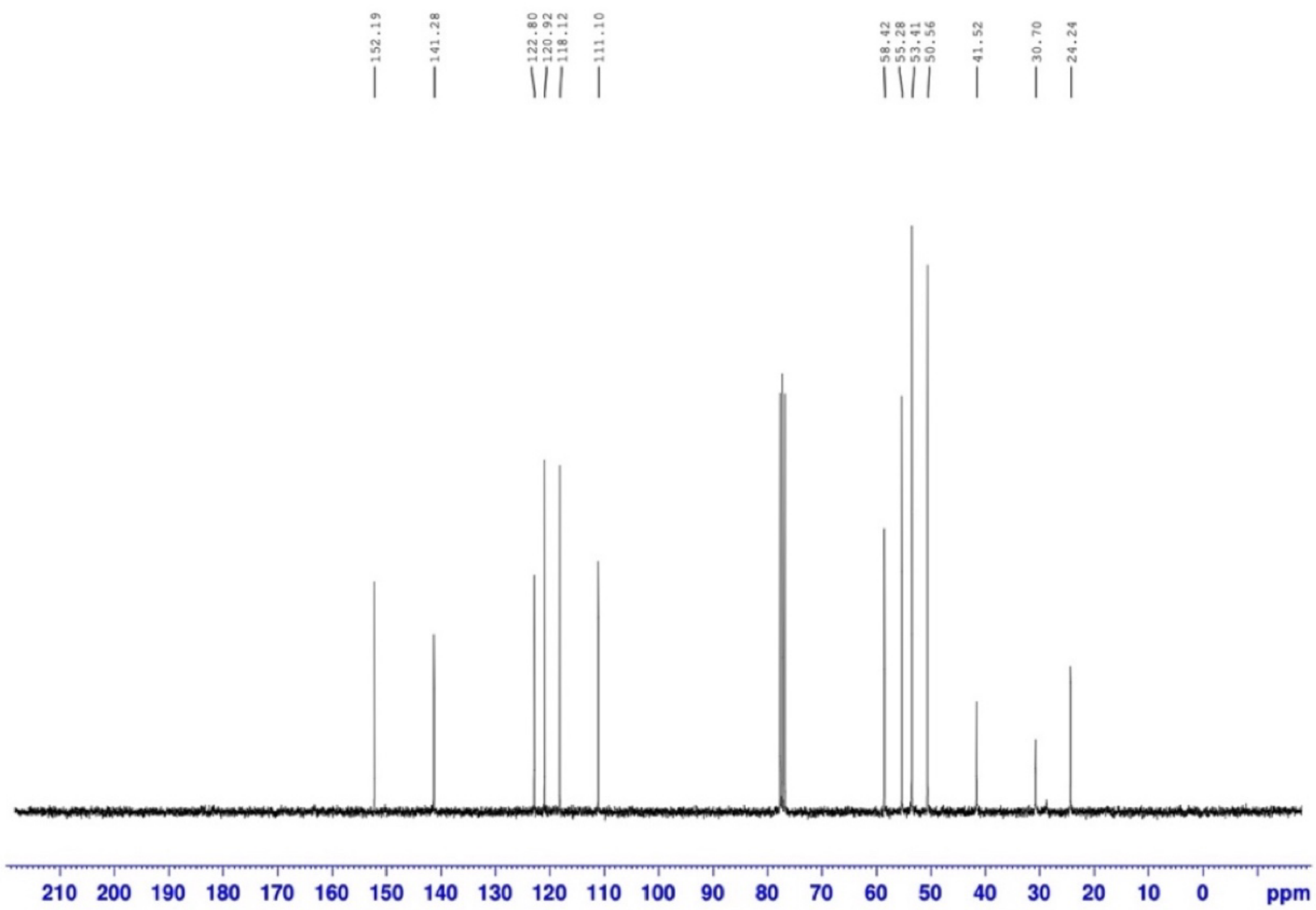

Figure S46. $^{13}C\{^{1}H\}$ NMR spectrum of 4-(4-(2-methoxyphenyl)piperazin-1-yl)butan-1-amine (**S-2**) in $CDCl_3$.

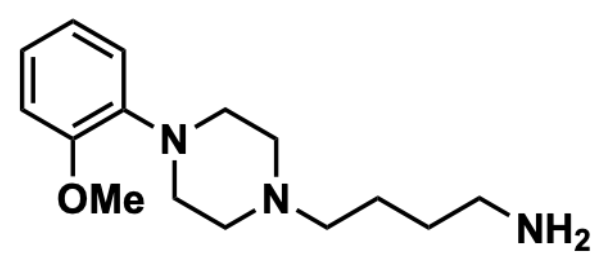

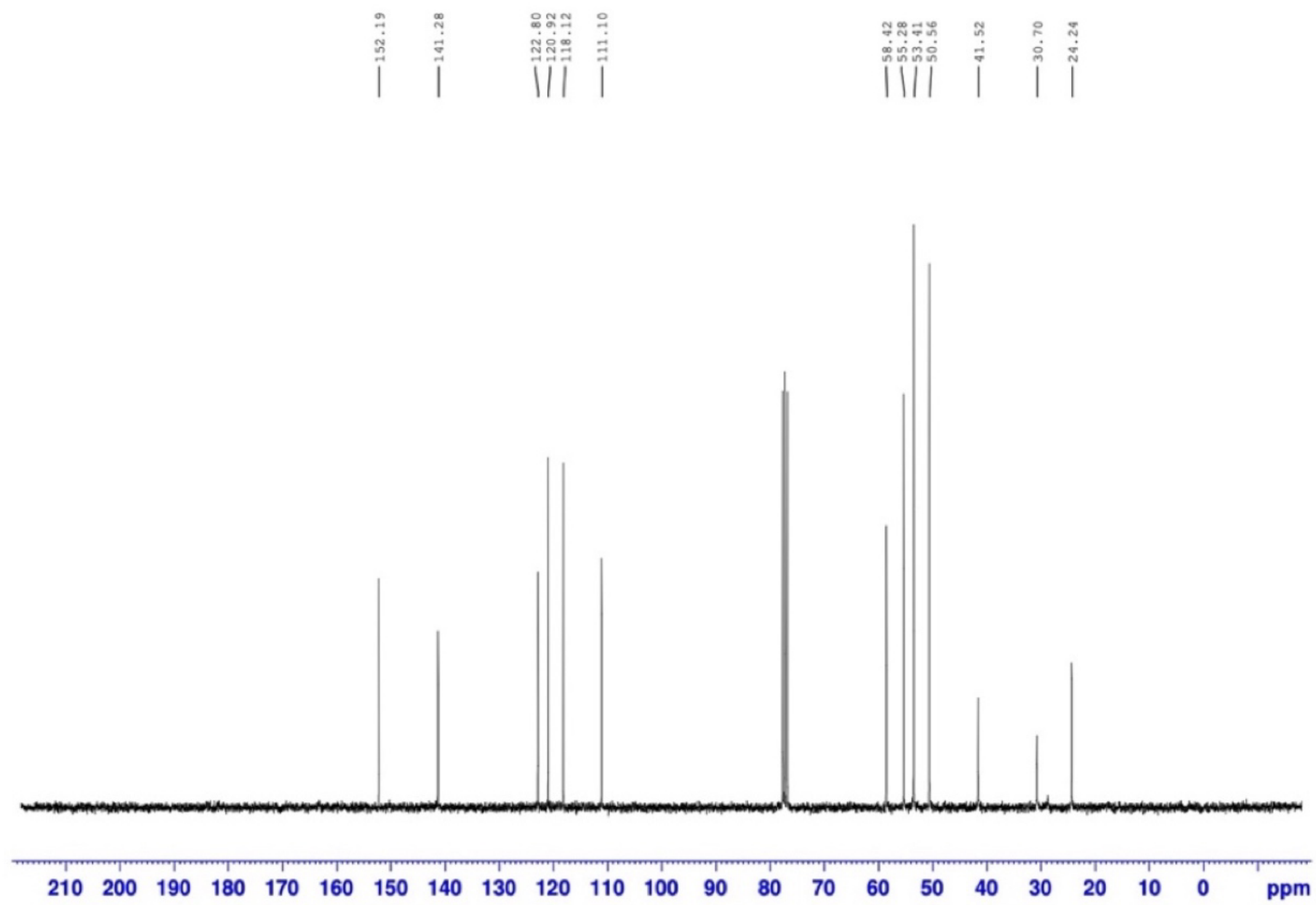

Figure S47. $^{13}C\{^{1}H\}$ NMR spectrum of 4-(4-(2-methoxyphenyl)piperazin-1-yl)butan-1-amine (**S-2**) in $CDCl_3$.

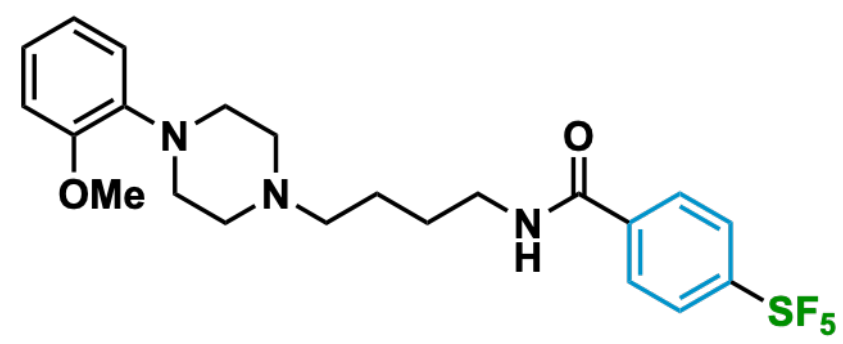

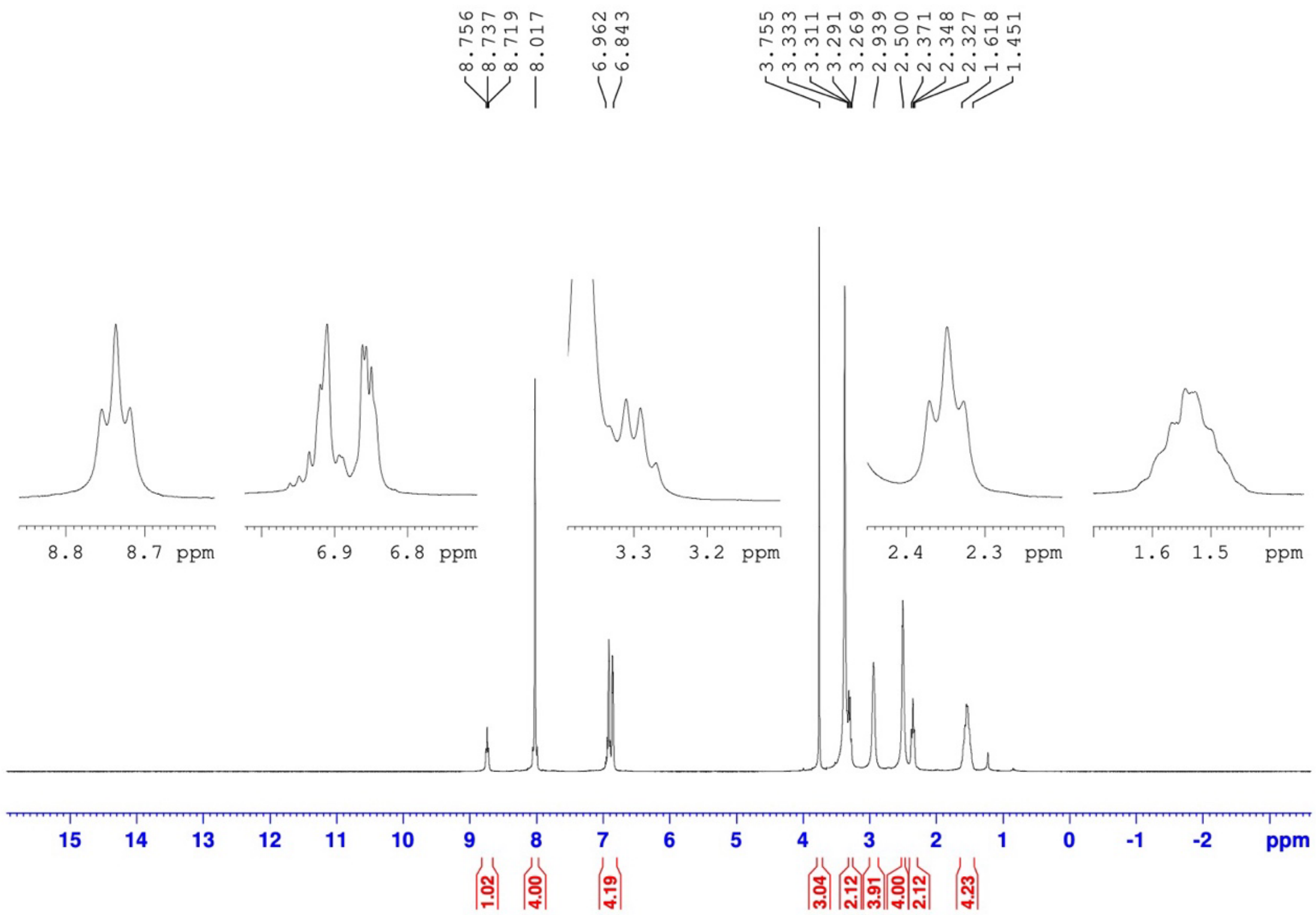

Figure S48. ^{1}H NMR spectrum of *N*-(4-(4-(2-methoxyphenyl)piperazin-1-yl)butyl)-4-(pentafluoro-λ^6-sulfanyl)benzamide (**7**) in DMSO-d_6.

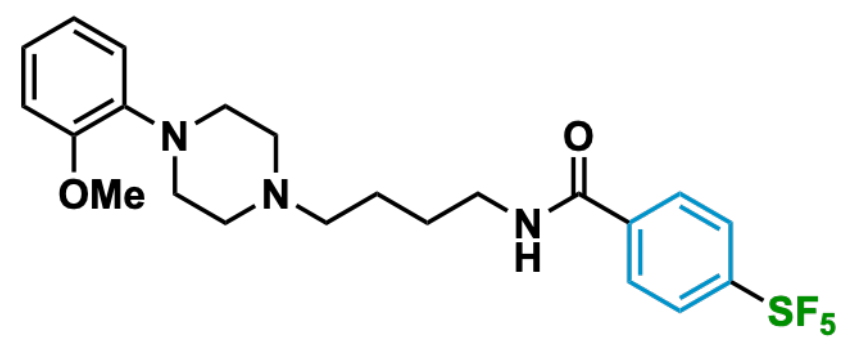

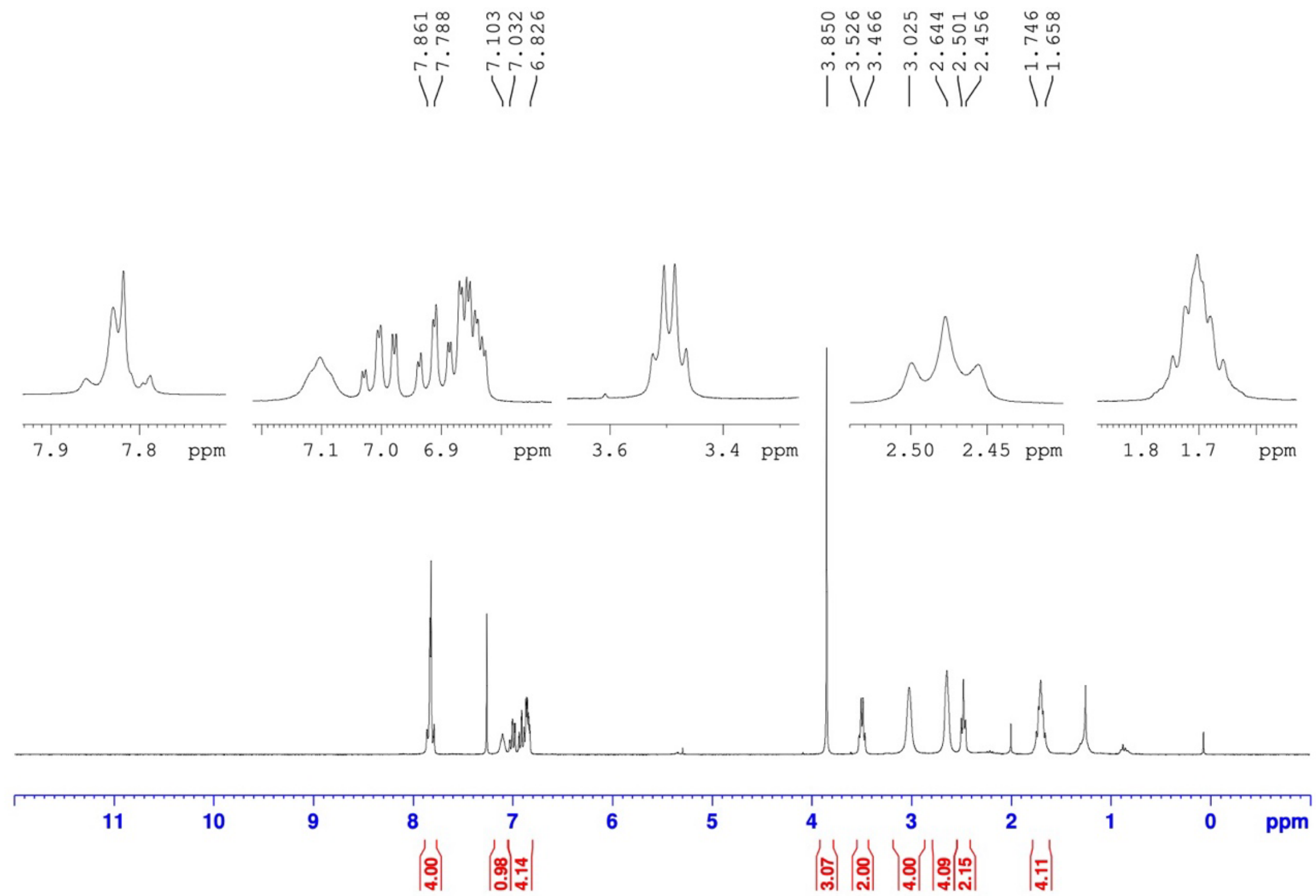

Figure S49. ^{1}H NMR spectrum of *N*-(4-(4-(2-methoxyphenyl)piperazin-1-yl)butyl)-4-(pentafluoro-λ^6-sulfanyl)benzamide (**7**) in $CDCl_3$.

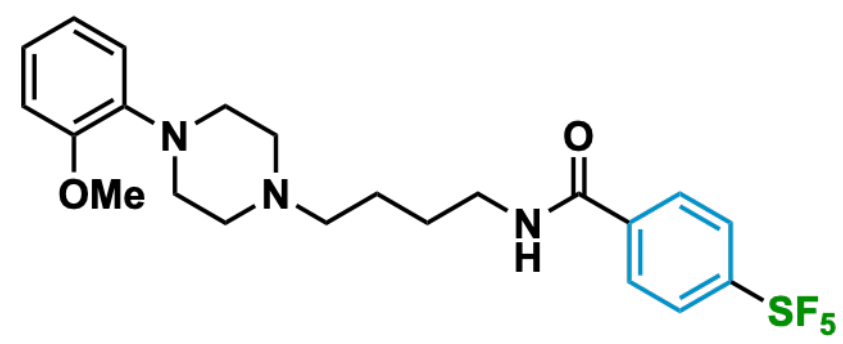

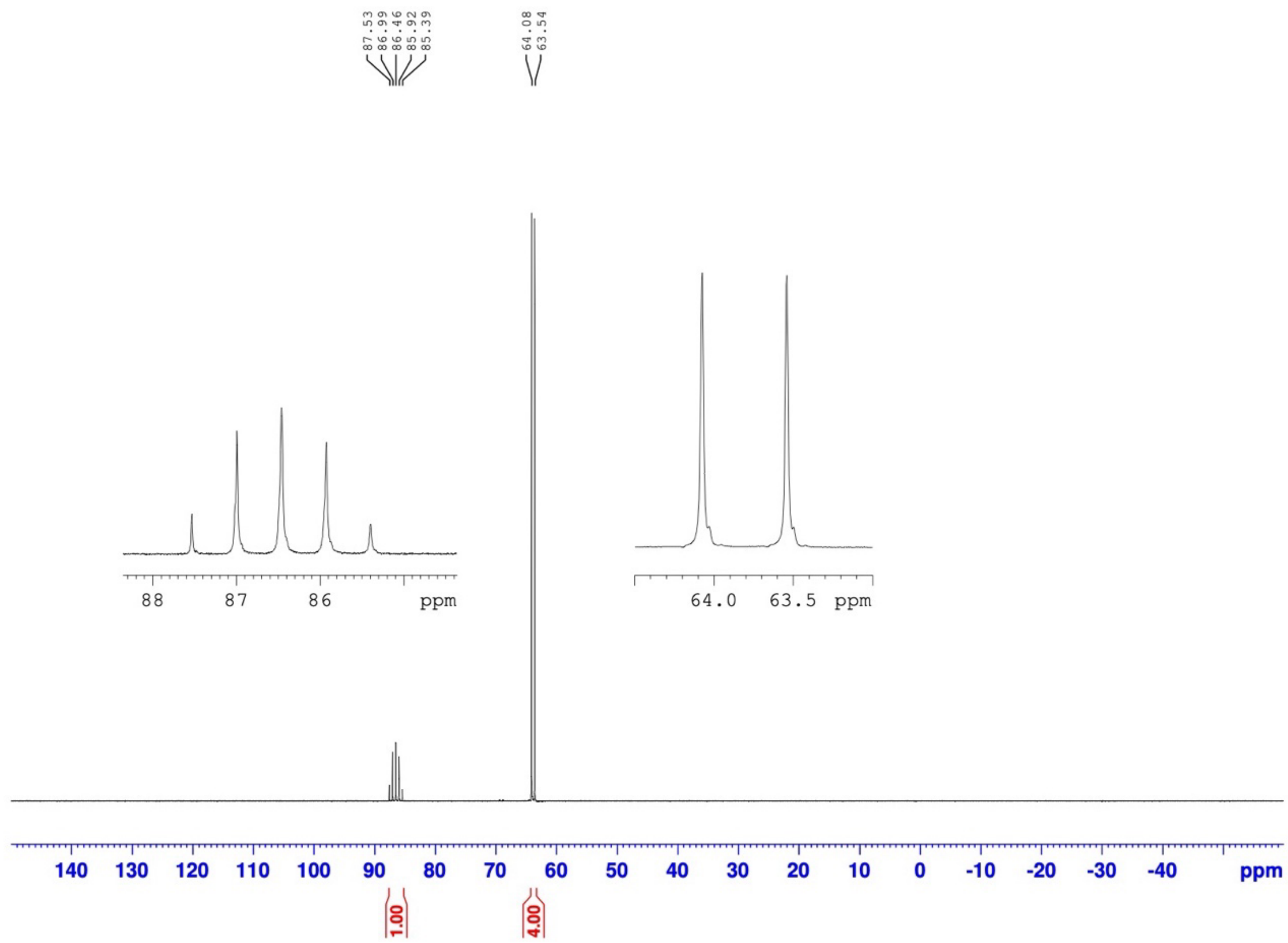

Figure S50. ^{19}F NMR spectrum of *N*-(4-(4-(2-methoxyphenyl)piperazin-1-yl)butyl)-4-(pentafluoro-λ^6-sulfanyl)benzamide (**7**) in DMSO-*d*$_6$.

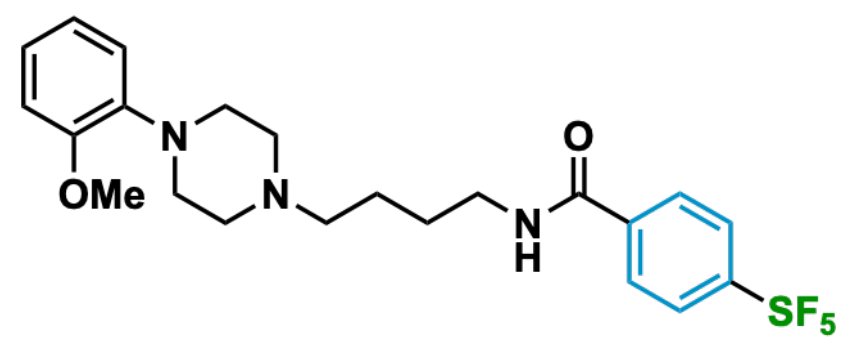

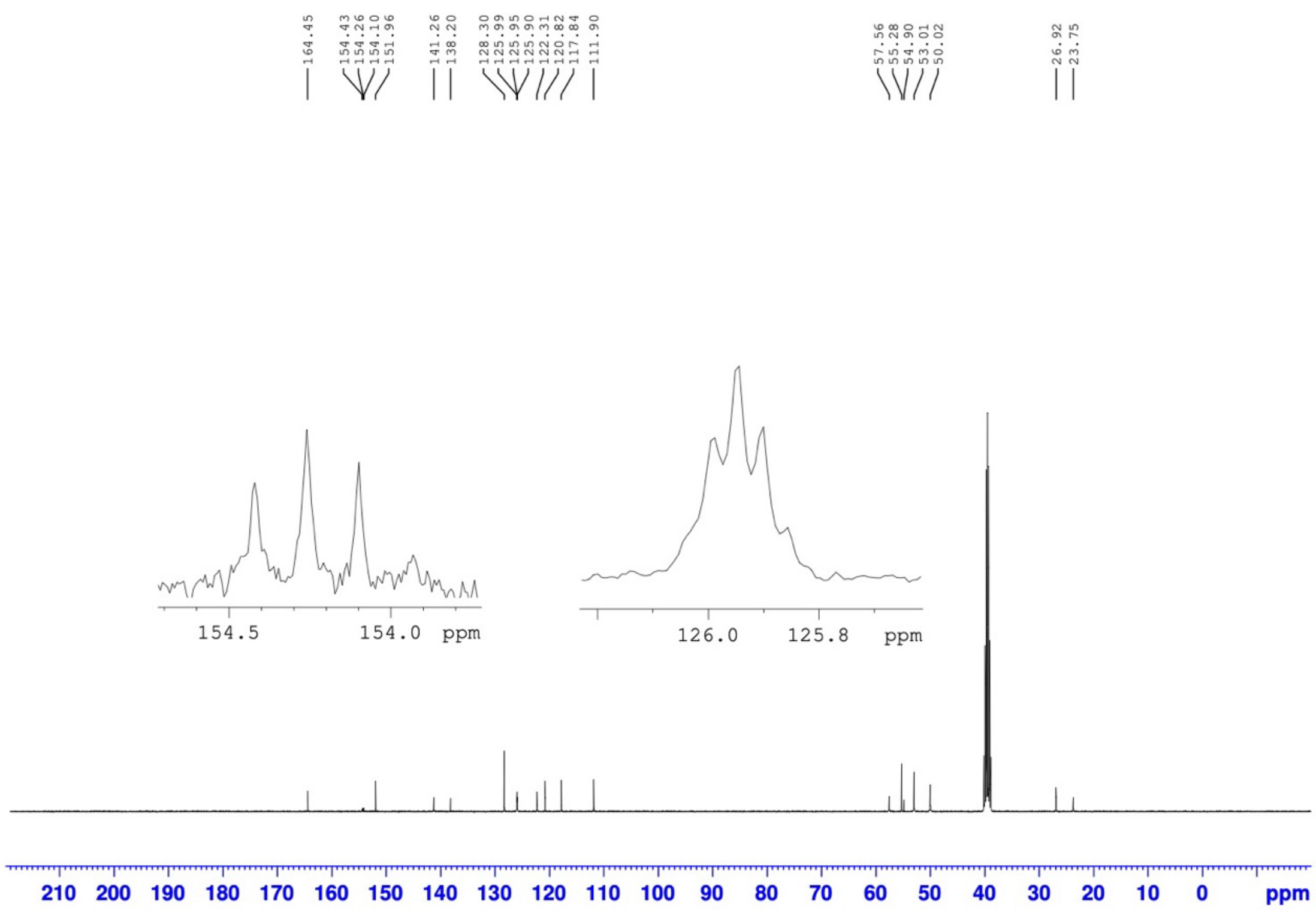

Figure S51. $^{13}C\{^1H\}$ NMR spectrum of *N*-(4-(4-(2-methoxyphenyl)piperazin-1-yl)butyl)-4-(pentafluoro-λ^6-sulfanyl)benzamide (**7**) in DMSO-d_6.

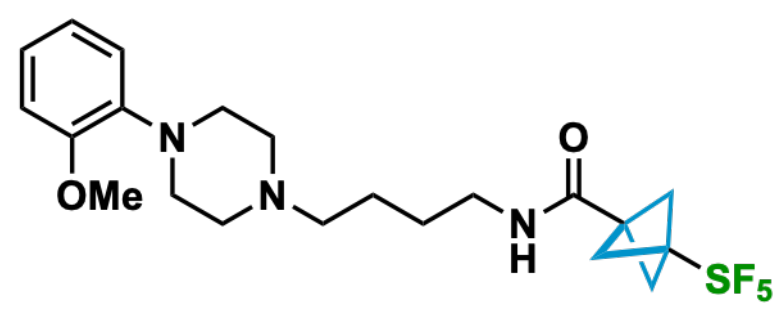

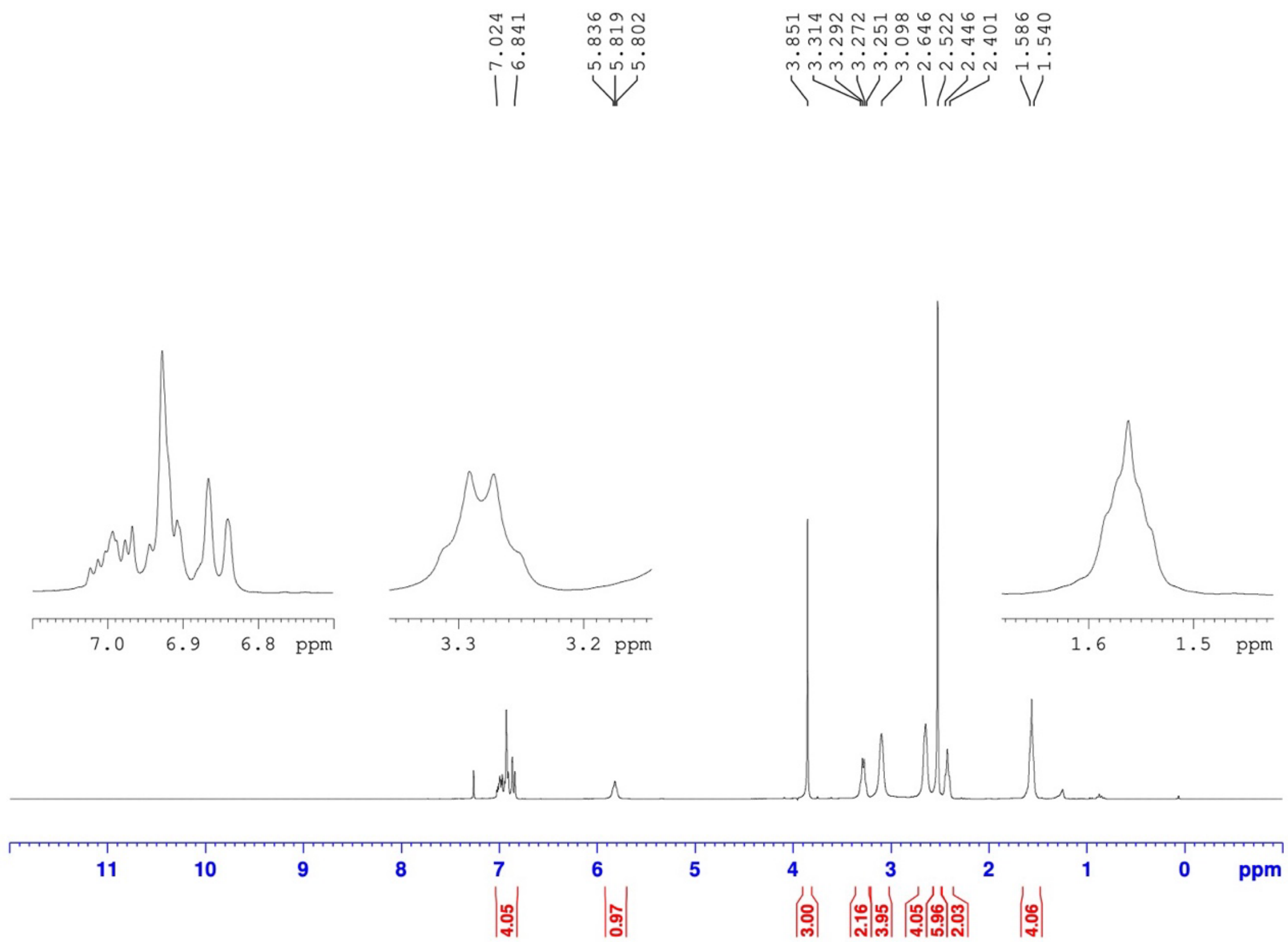

Figure S52. ^{1}H NMR spectrum of *N*-(4-(4-(2-methoxyphenyl)piperazin-1-yl)butyl)-3-(pentafluoro-λ^6-sulfanyl)bicyclo[1.1.1]pentane-1-carboxamide (**9**) in $CDCl_3$.

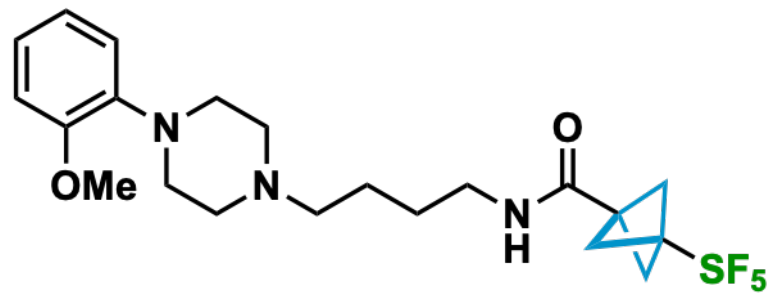

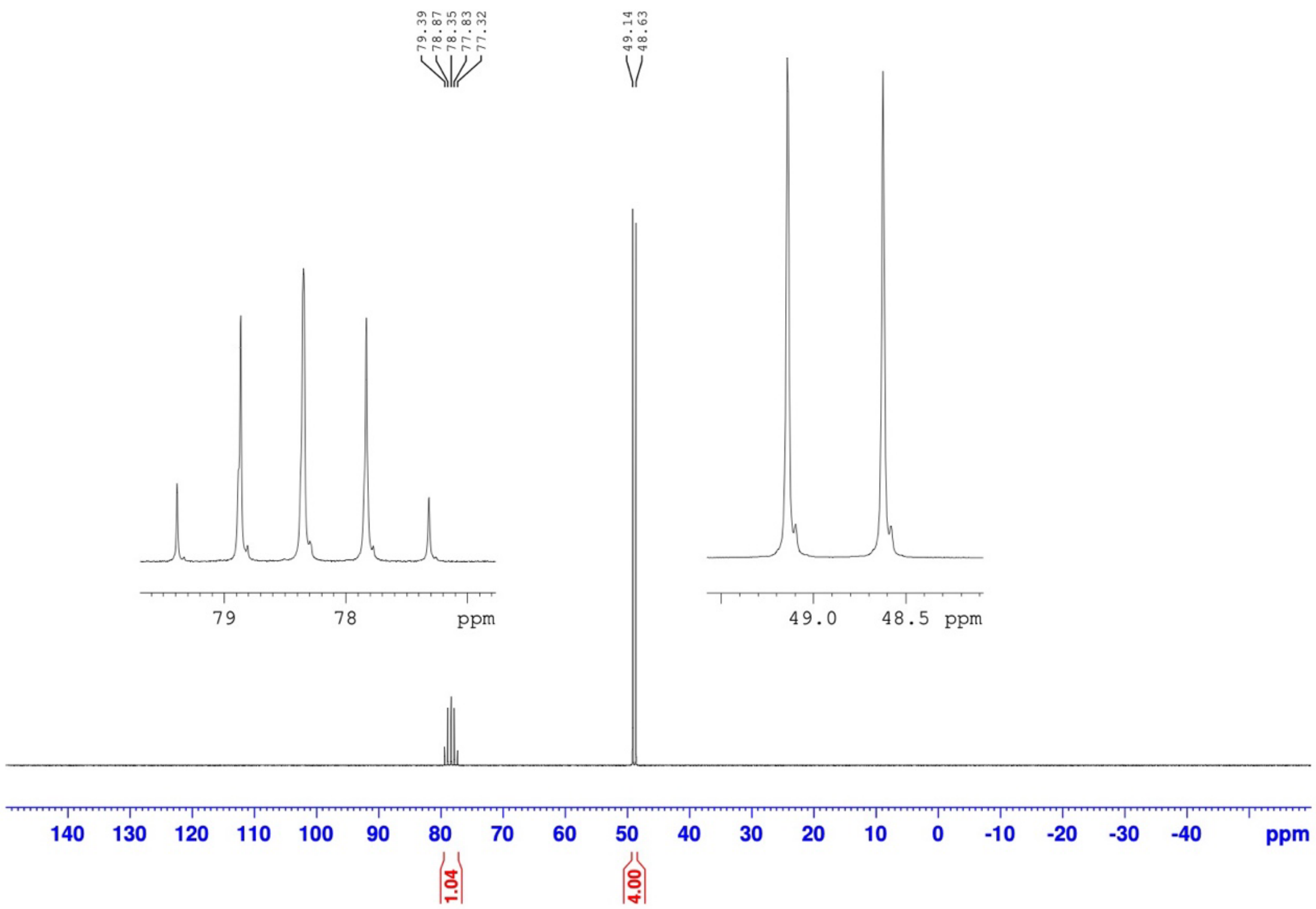

Figure S53. ^{19}F NMR spectrum of *N*-(4-(4-(2-methoxyphenyl)piperazin-1-yl)butyl)-3-(pentafluoro-λ^6-sulfanyl)bicyclo[1.1.1]pentane-1-carboxamide (**9**) in $CDCl_3$.

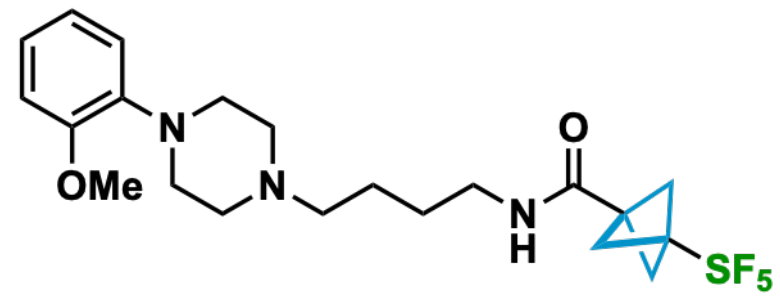

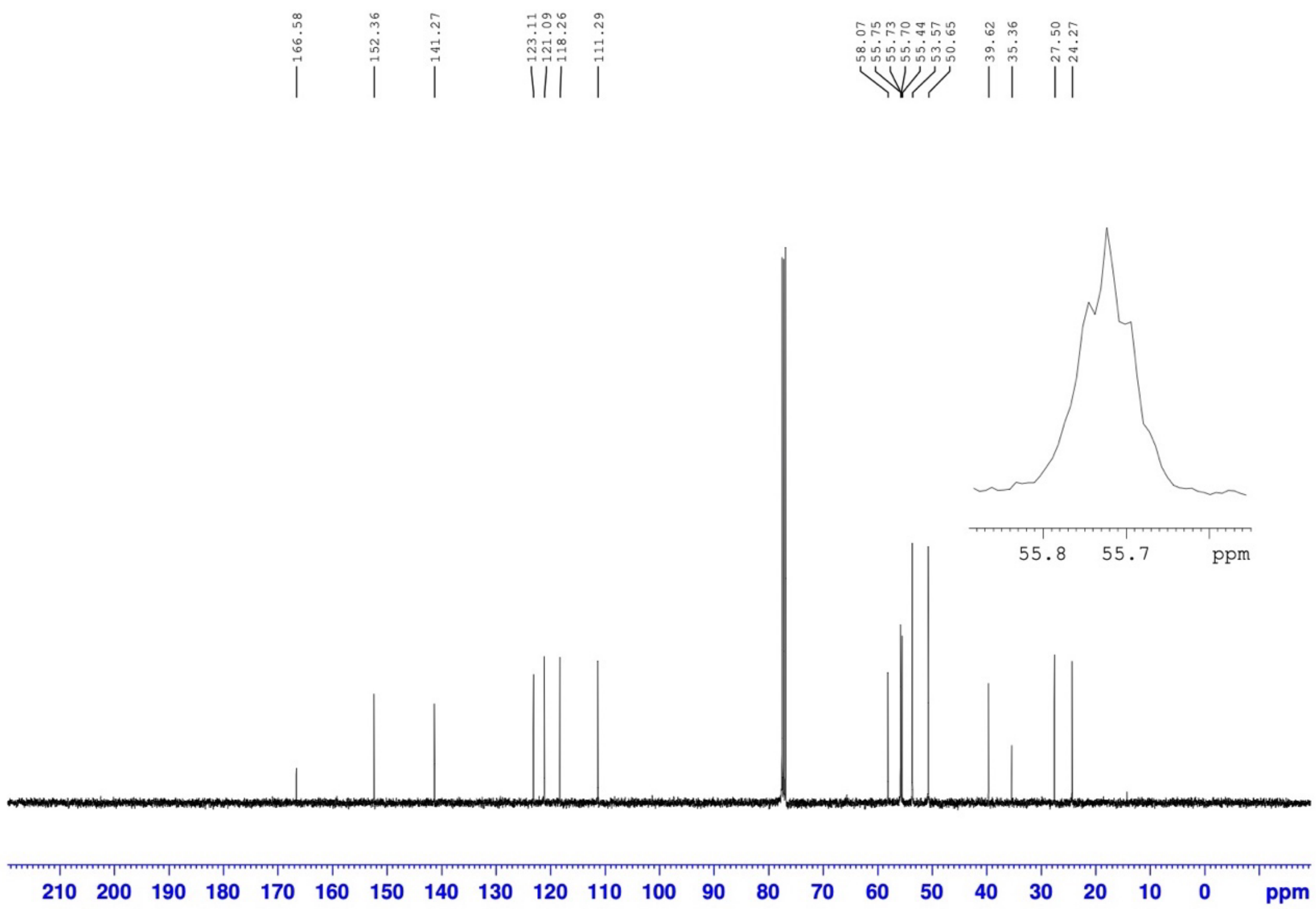

Figure S54. $^{13}C\{^1H\}$ NMR spectrum of *N*-(4-(4-(2-methoxyphenyl)piperazin-1-yl)butyl)-3-(pentafluoro-λ^6-sulfanyl)bicyclo[1.1.1]pentane-1-carboxamide (**9**) in $CDCl_3$.

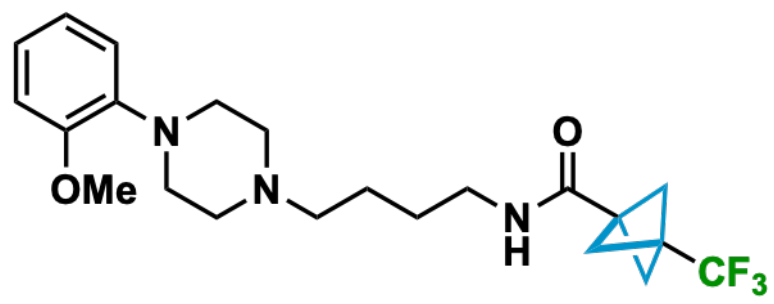

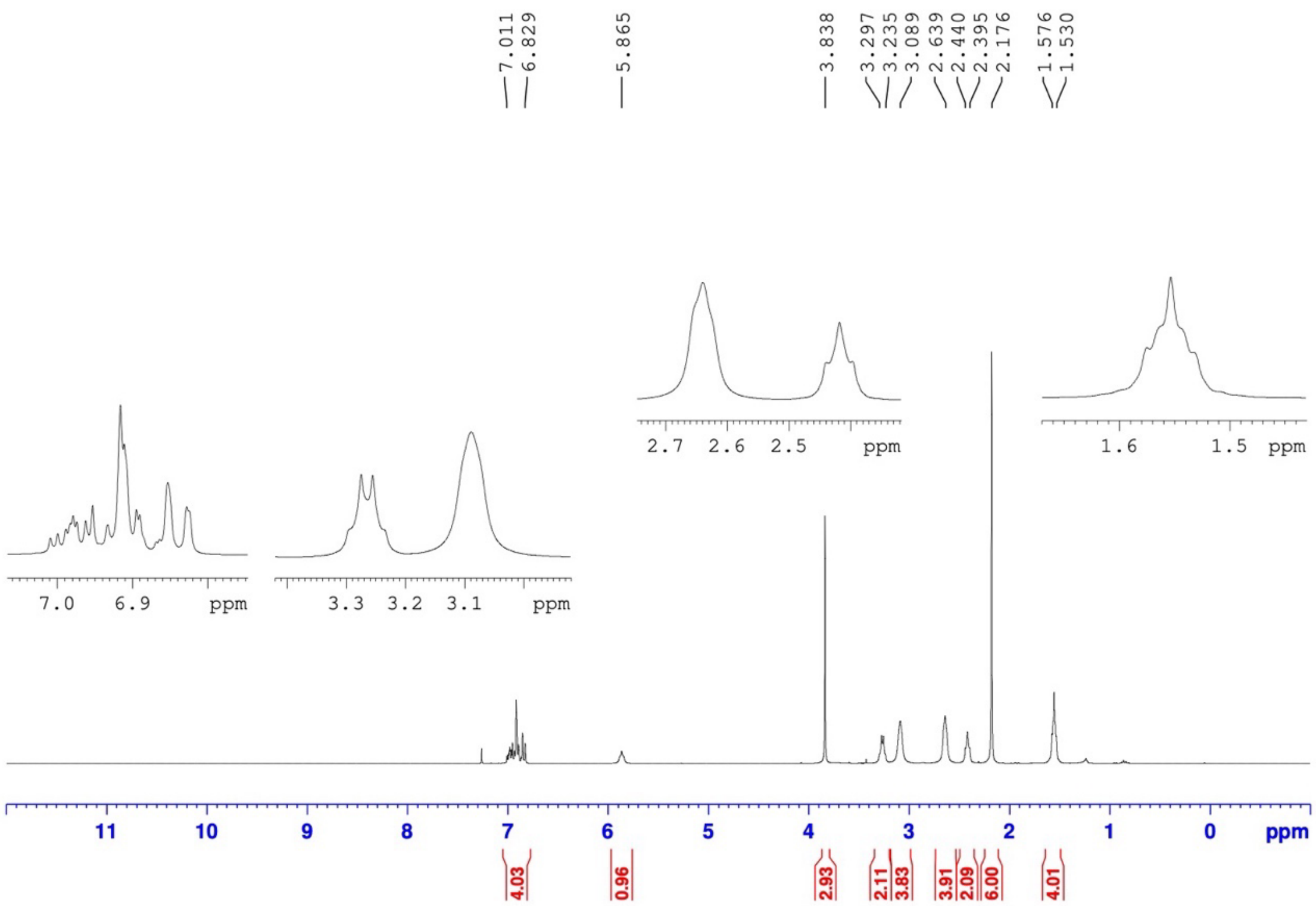

Figure S55. ^{1}H NMR spectrum of *N*-(4-(4-(2-methoxyphenyl)piperazin-1-yl)butyl)-3-(trifluoromethyl)bicyclo[1.1.1]pentane-1-carboxamide (**26**) in $CDCl_3$.

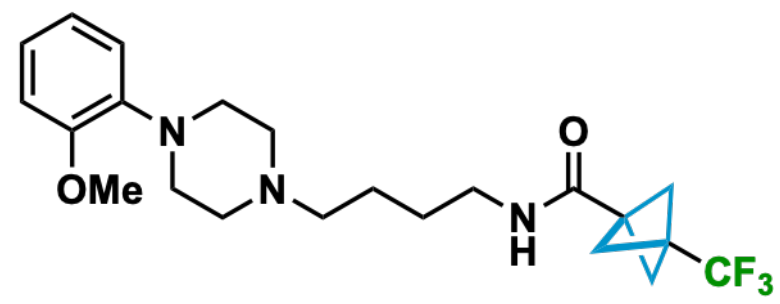

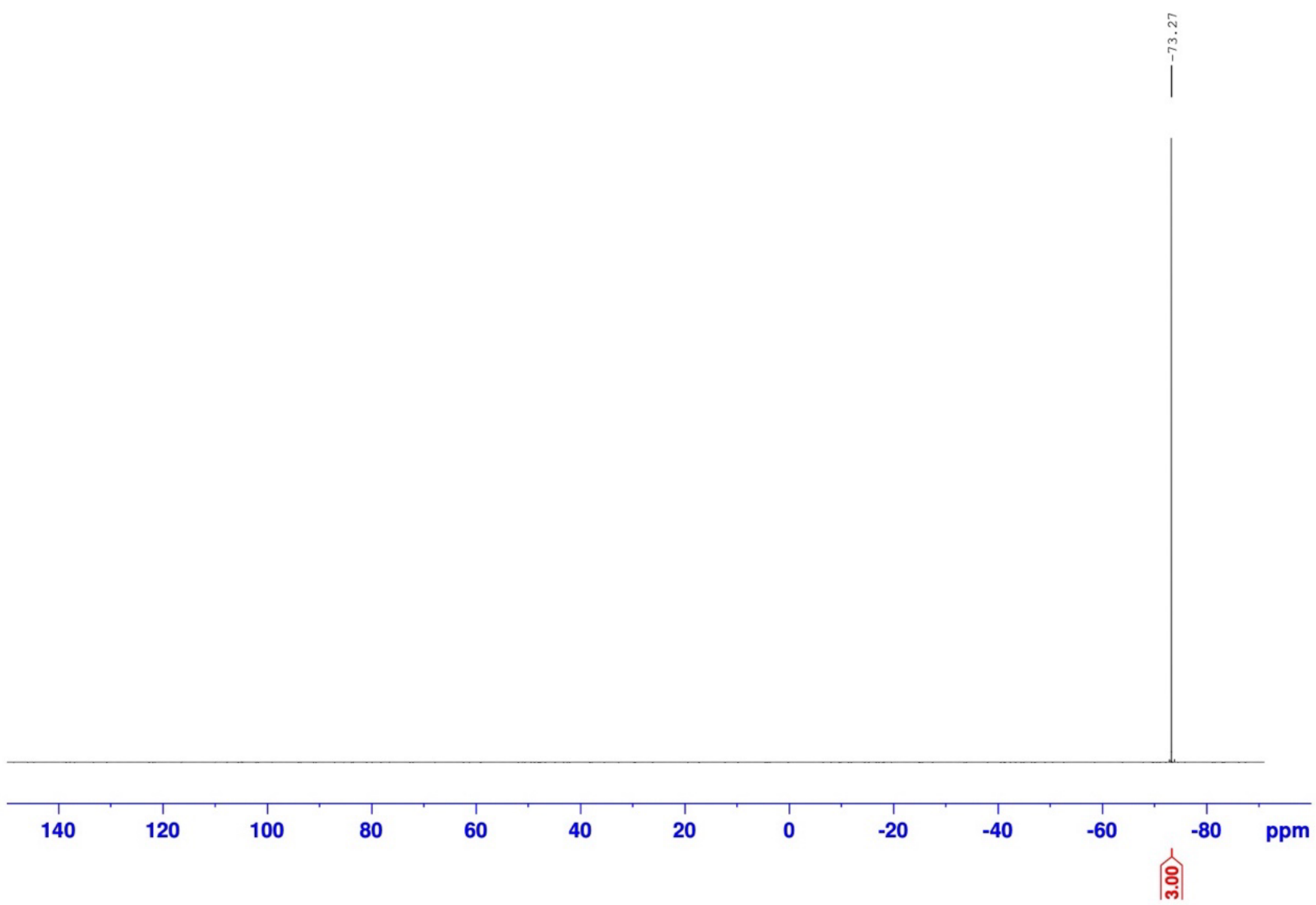

Figure S56. ^{19}F NMR spectrum of *N*-(4-(4-(2-methoxyphenyl)piperazin-1-yl)butyl)-3-(trifluoromethyl)-bicyclo[1.1.1]pentane-1-carboxamide (**26**) in $CDCl_3$.

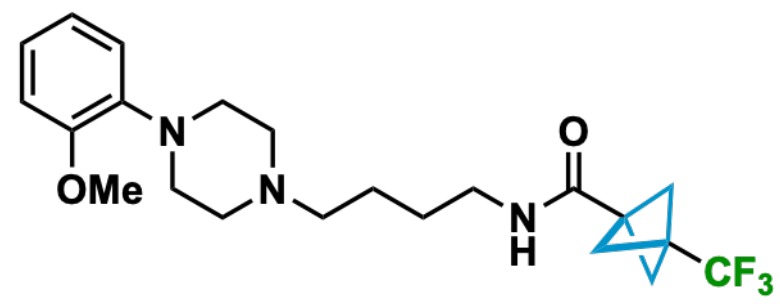

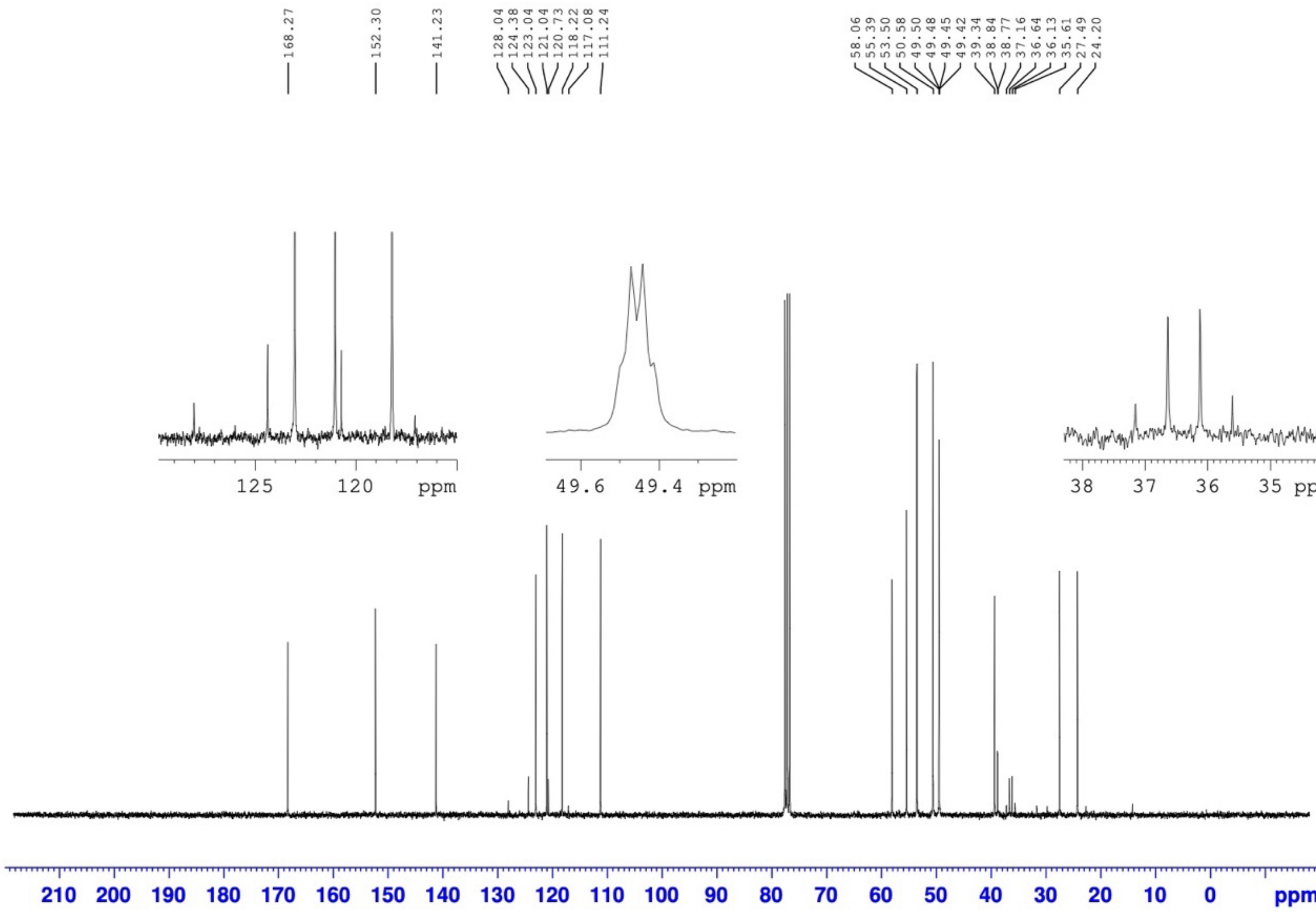

Figure S57. $^{13}C\{^1H\}$ NMR spectrum of *N*-(4-(4-(2-methoxyphenyl)piperazin-1-yl)butyl)-3-(trifluoromethyl)-bicyclo[1.1.1]pentane-1-carboxamide (**26**) in $CDCl_3$.

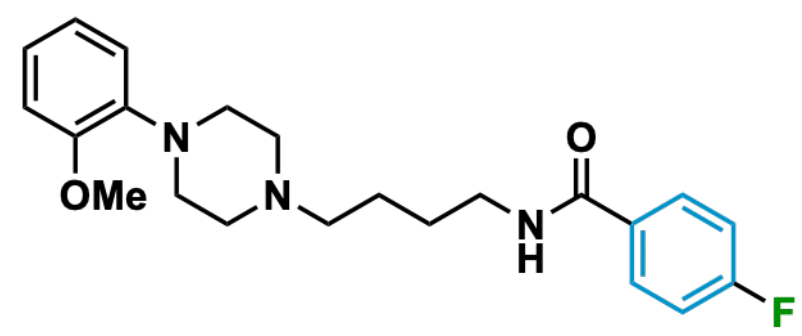

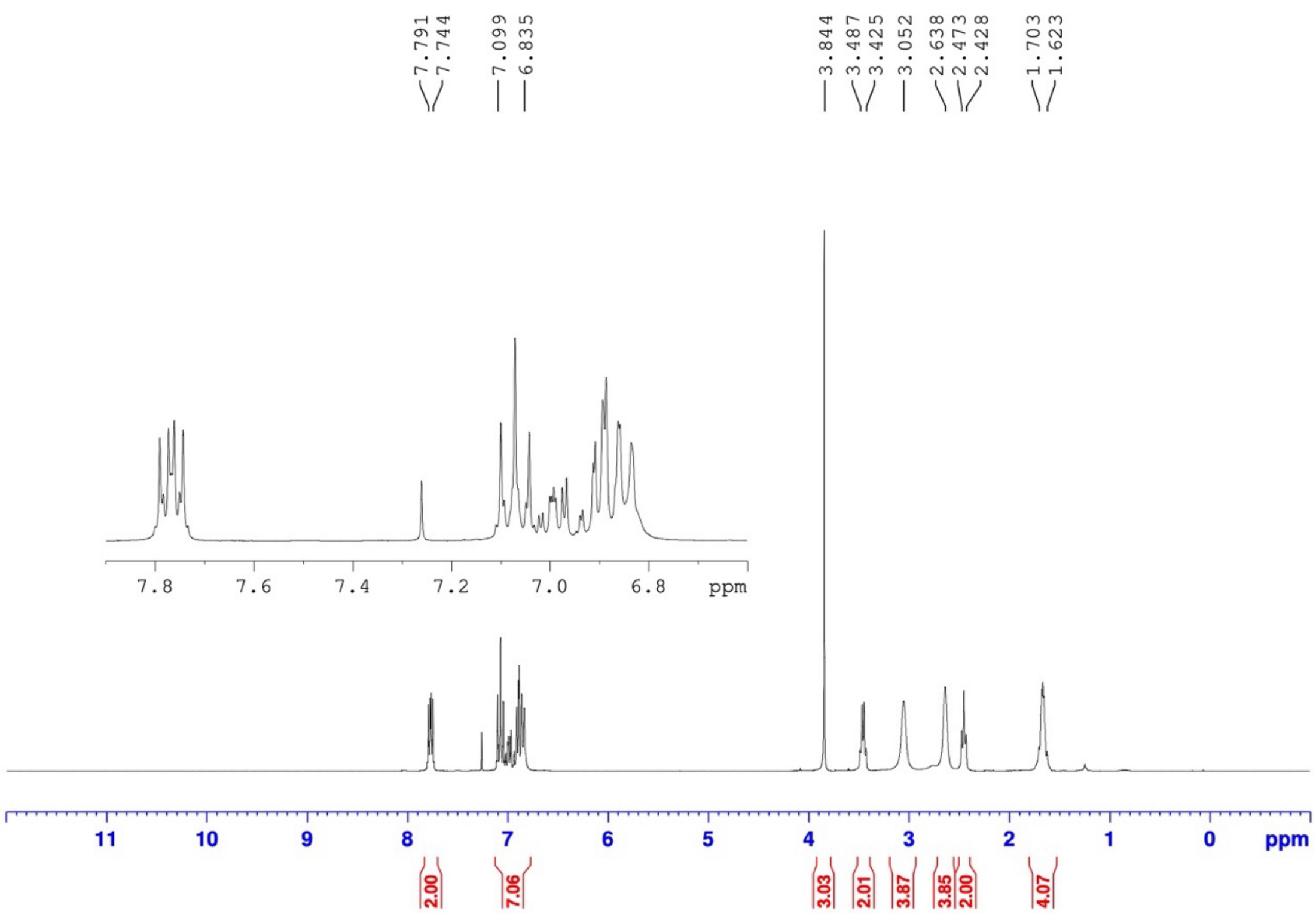

Figure S58. ^{1}H NMR spectrum of 4-fluoro-N-(4-(4-(2-methoxyphenyl)piperazin-1-yl)butyl)benzamide (**25**) in $CDCl_3$.

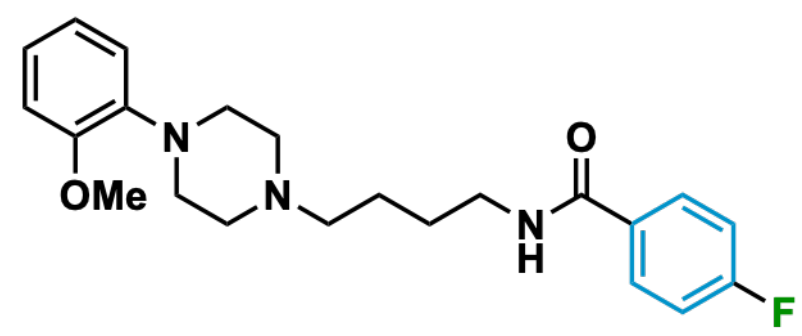

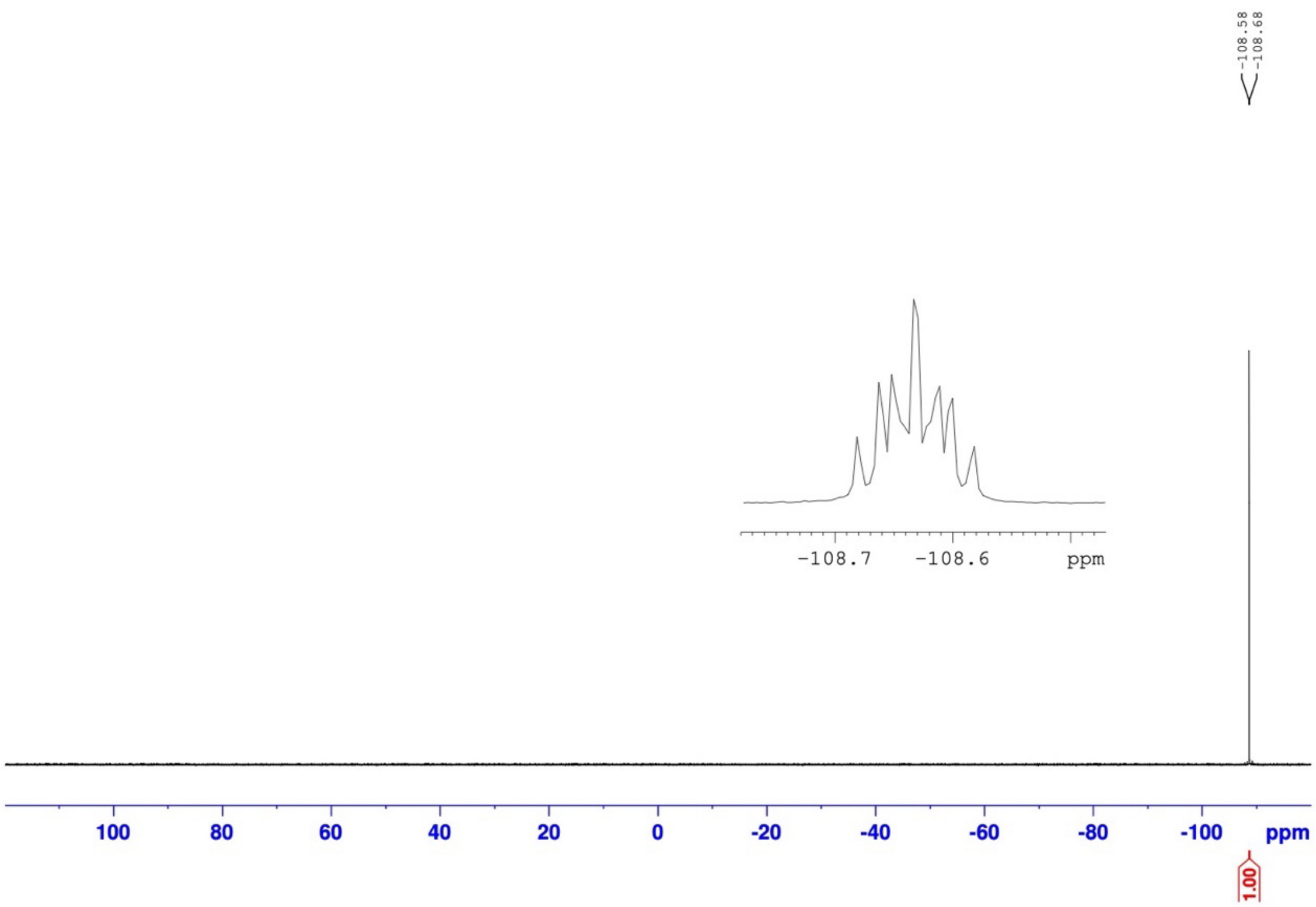

Figure S59. ^{19}F NMR spectrum of 4-fluoro-N-(4-(4-(2-methoxyphenyl)piperazin-1-yl)butyl)benzamide (**25**) in $CDCl_3$.

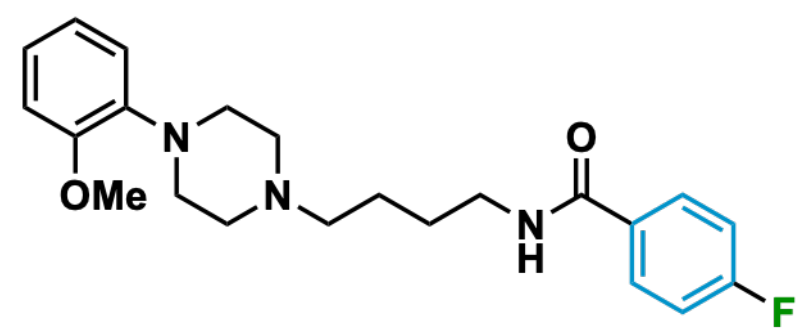

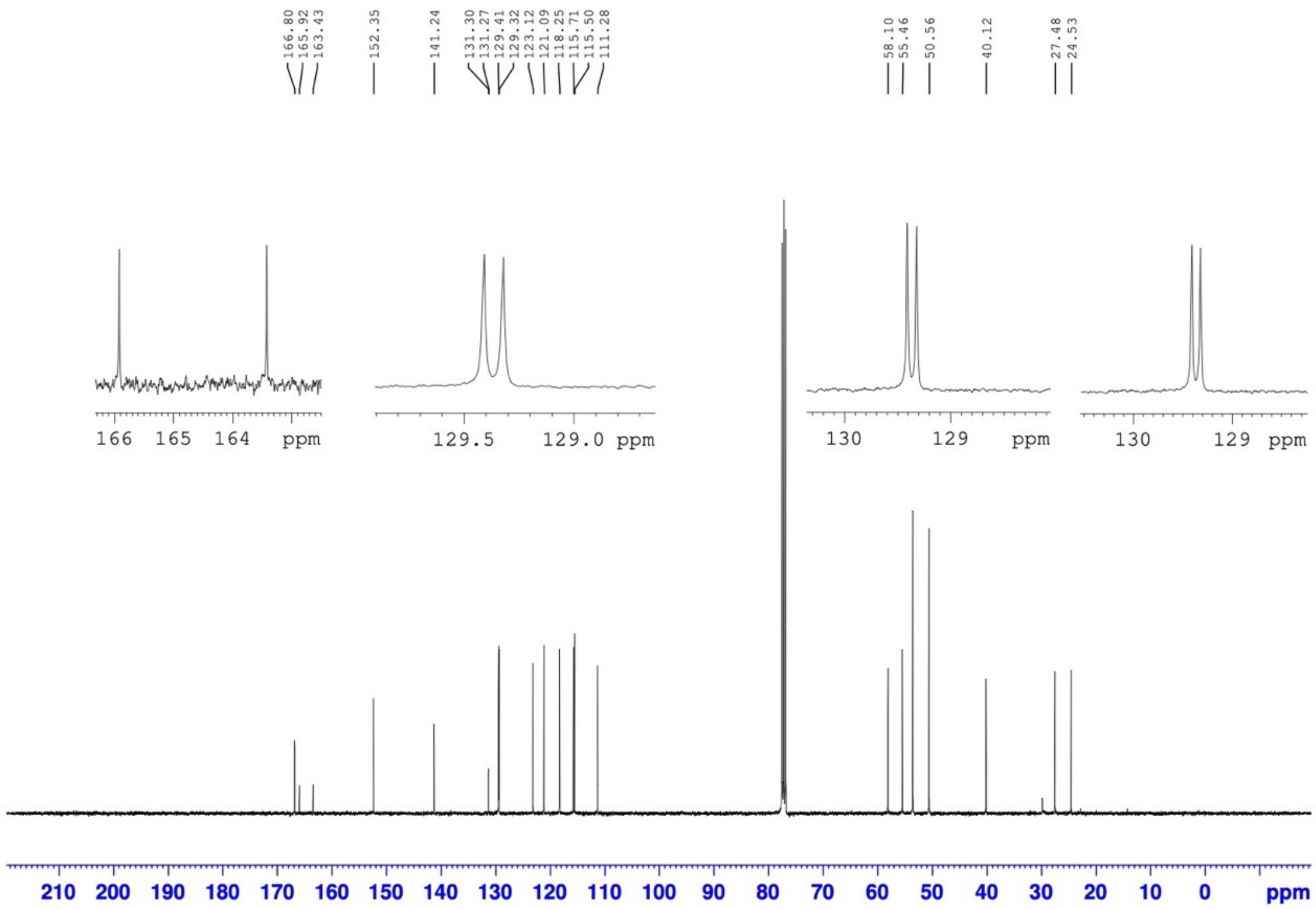

Figure S60. $^{13}C\{^{1}H\}$ NMR spectrum of 4-fluoro-N-(4-(4-(2-methoxyphenyl)piperazin-1-yl)butyl)benzamide (**25**) in $CDCl_3$.

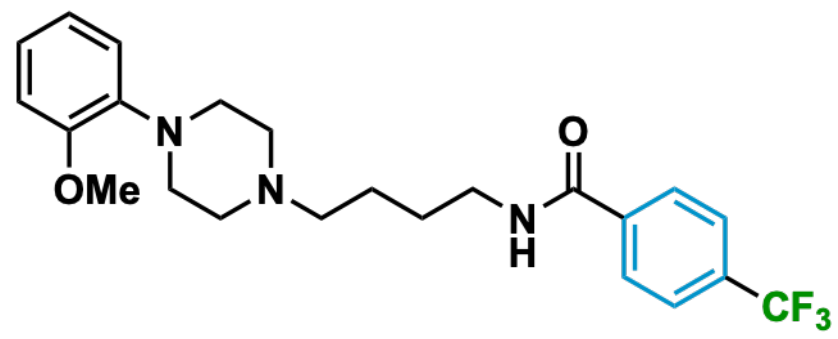

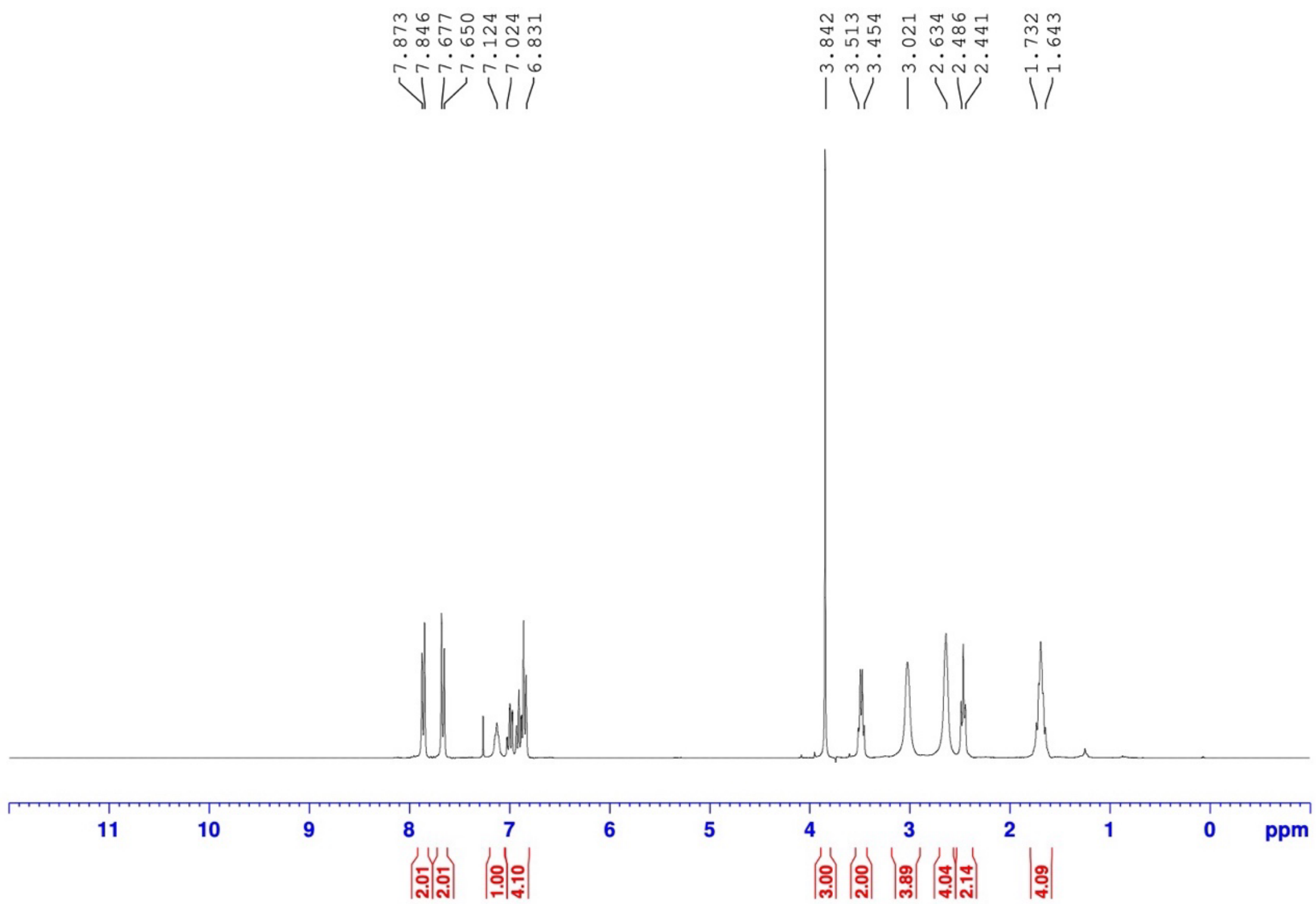

Figure S61. ^{1}H NMR spectrum of *N*-(4-(4-(2-methoxyphenyl)piperazin-1-yl)butyl)-4-(trifluoromethyl)-benzamide (**27**) in $CDCl_3$.

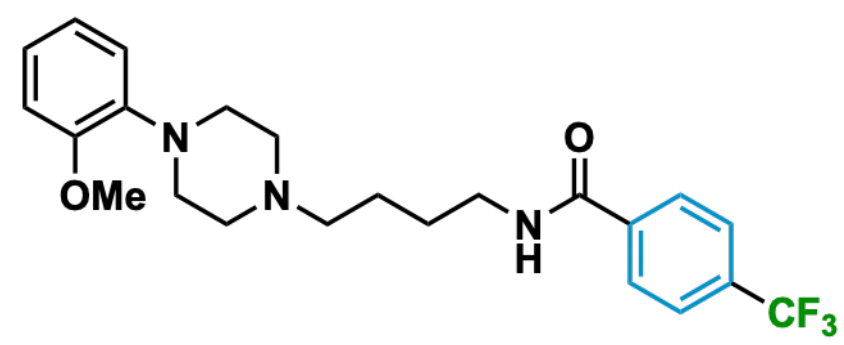

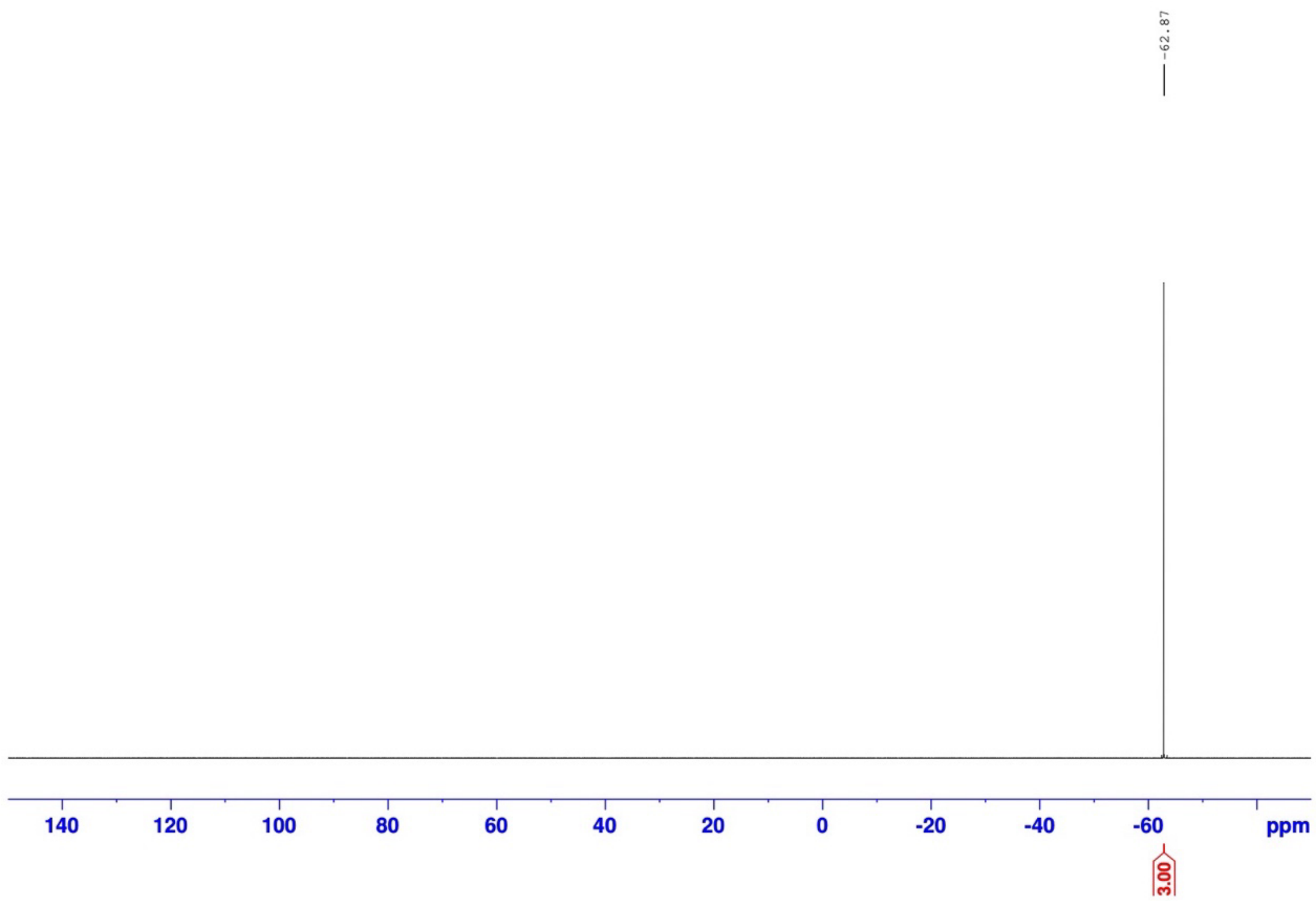

Figure S62. ^{19}F NMR spectrum of *N*-(4-(4-(2-methoxyphenyl)piperazin-1-yl)butyl)-4 (trifluoromethyl)-benzamide (**27**) in $CDCl_3$.

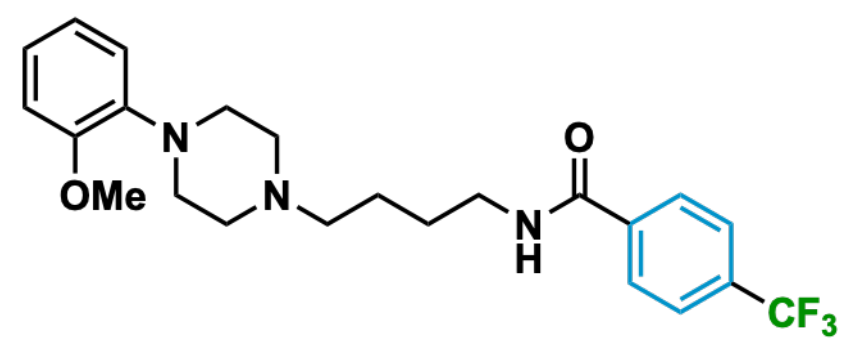

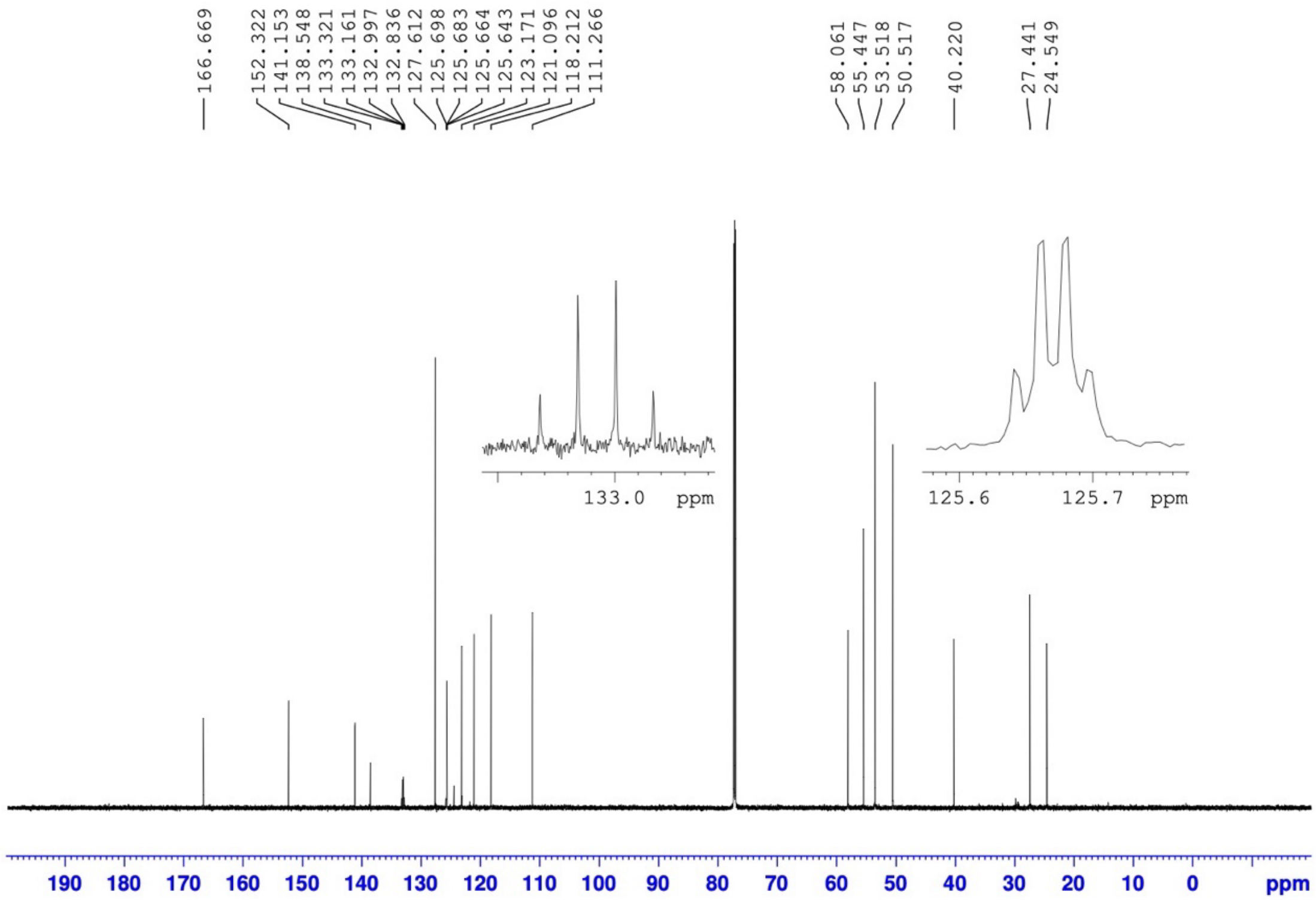

Figure S63. $^{13}C\{^1H\}$ NMR spectrum of *N*-(4-(4-(2-methoxyphenyl)piperazin-1-yl)butyl)-4-(trifluoromethyl)-benzamide (**27**) in $CDCl_3$.

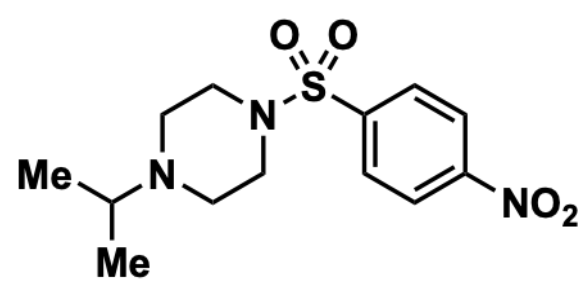

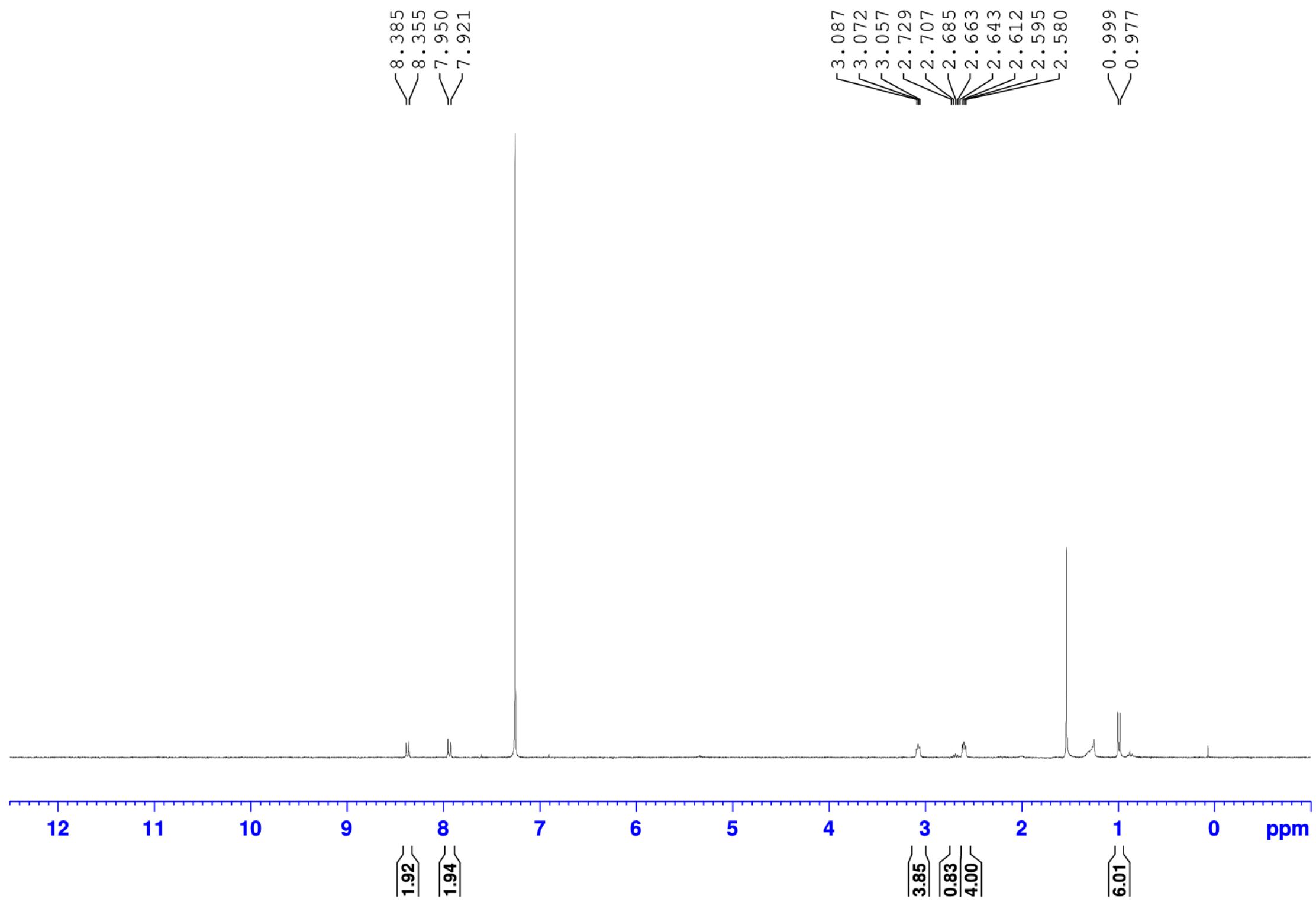

Figure S64. ^{1}H NMR spectrum of 1-isopropyl-4-((4-nitrophenyl)sulfonyl)piperazine (**S-3**) in $CDCl_3$.

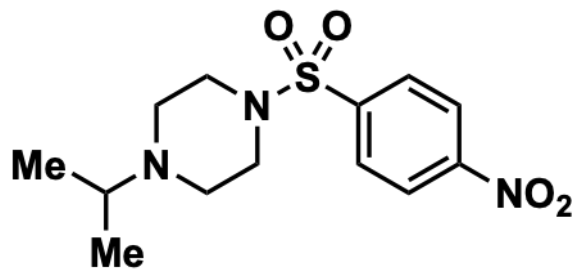

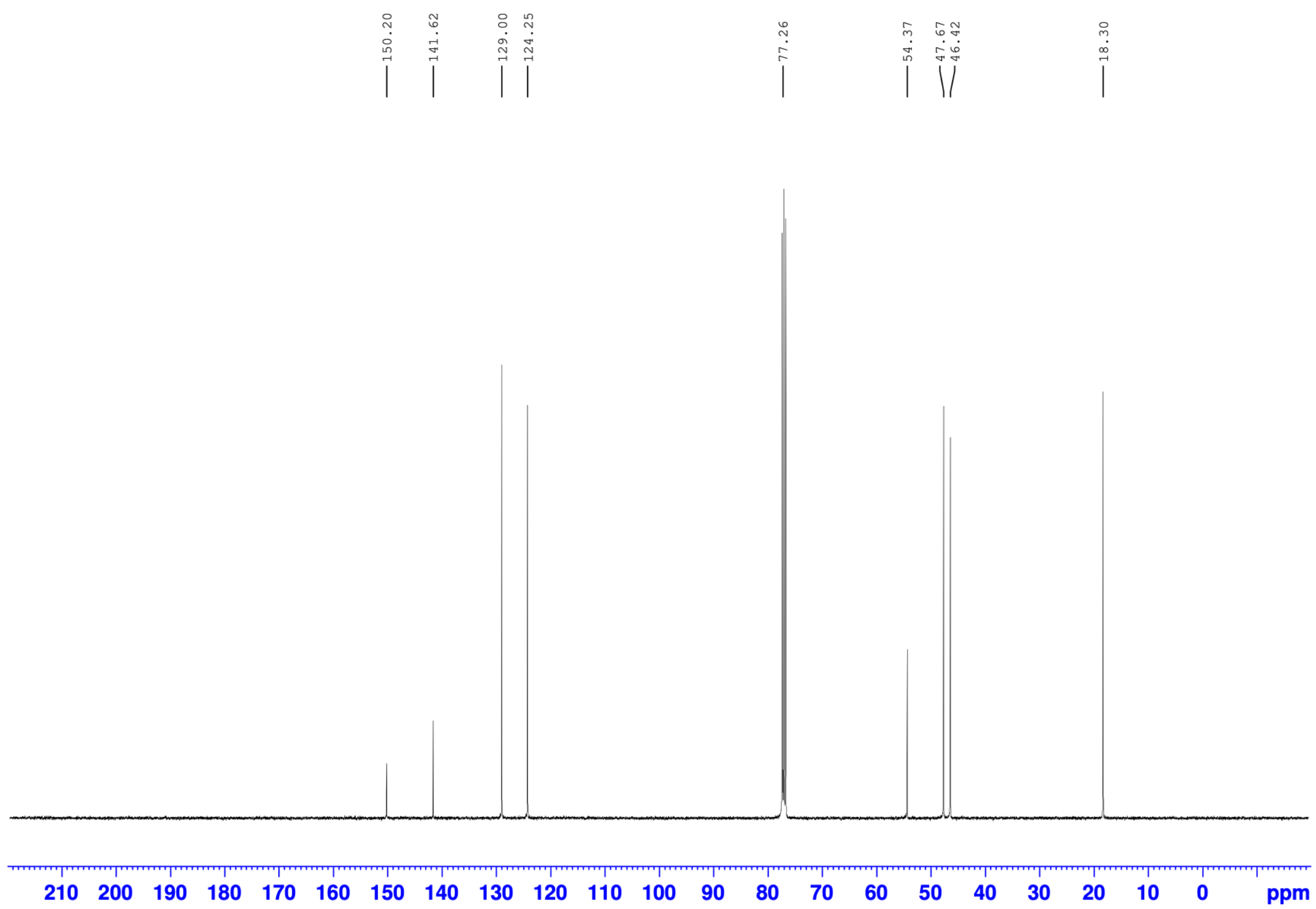

Figure S65. $^{13}C\{^{1}H\}$ NMR spectrum of 1-isopropyl-4-((4-nitrophenyl)sulfonyl)piperazine (**S-3**) in $CDCl_3$.

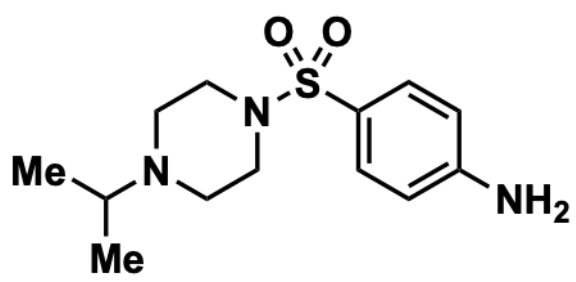

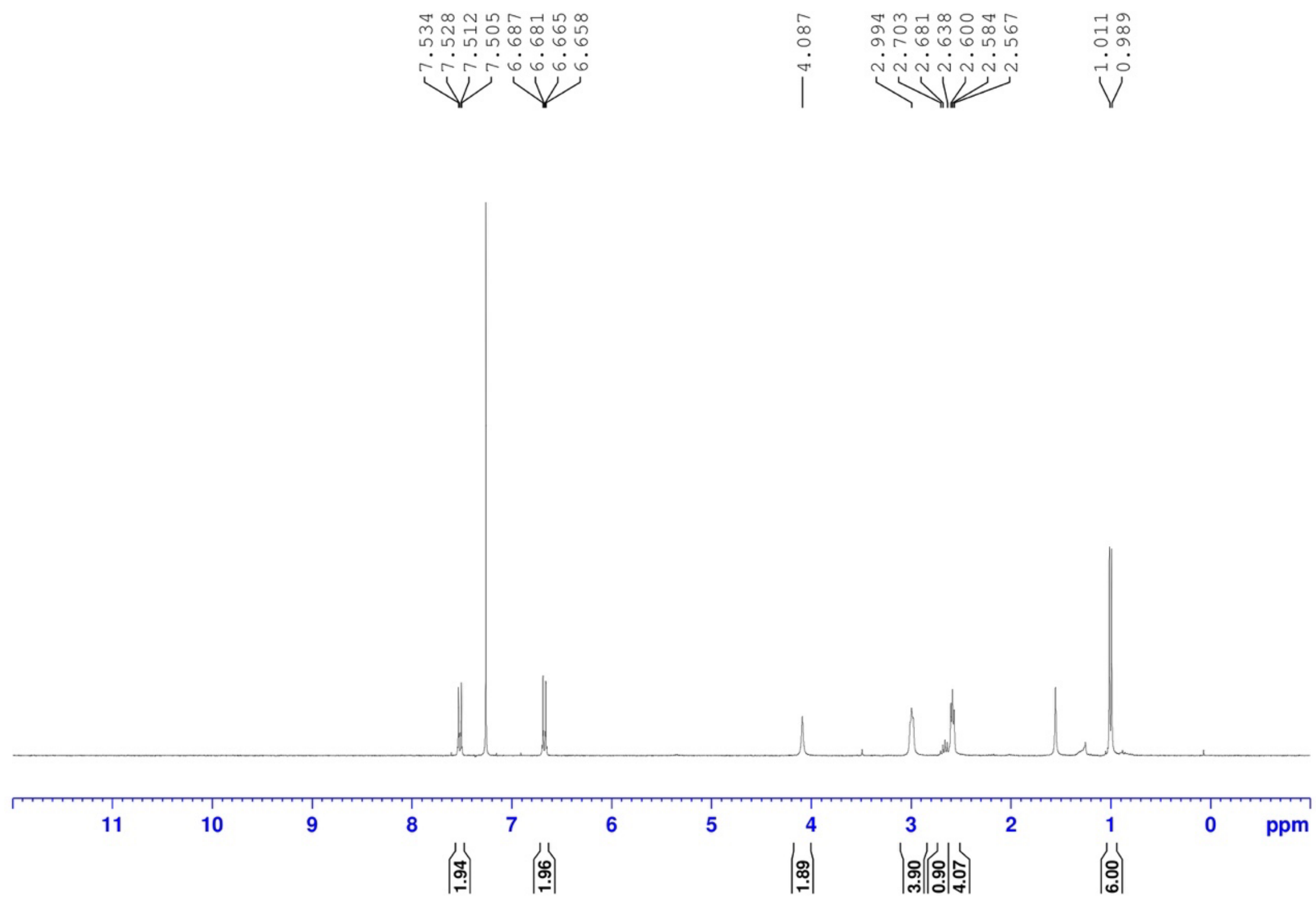

Figure S66. ^{1}H NMR spectrum of 4-((4-isopropylpiperazin-1-yl)sulfonyl)aniline (**S-4**) in $CDCl_3$.

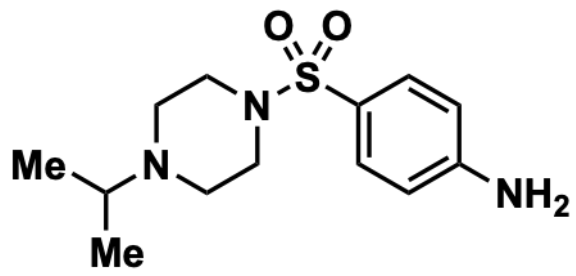

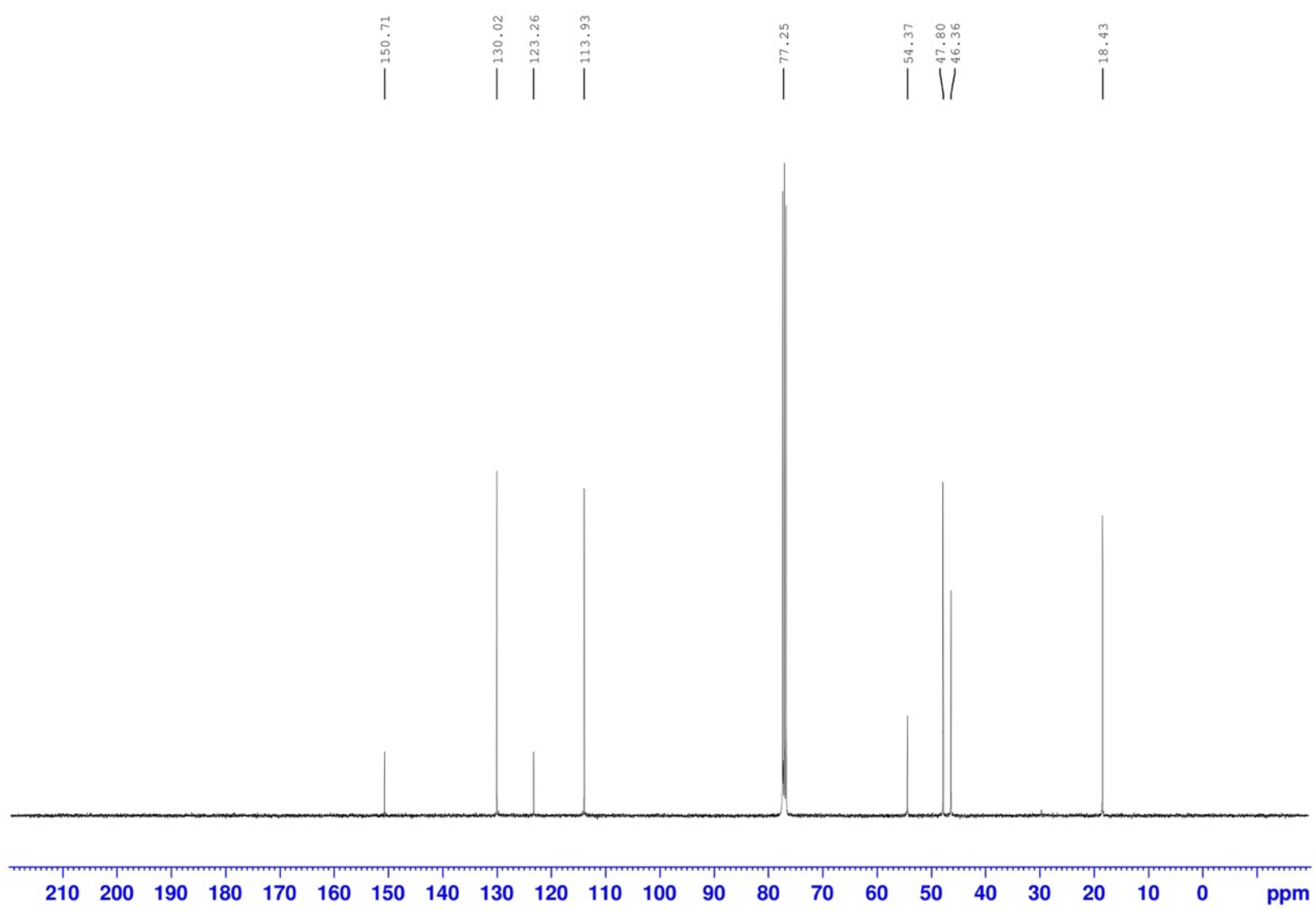

Figure S67. $^{13}C\{^{1}H\}$ NMR spectrum of 4-((4-isopropylpiperazin-1-yl)sulfonyl)aniline (**S-4**) in $CDCl_3$.

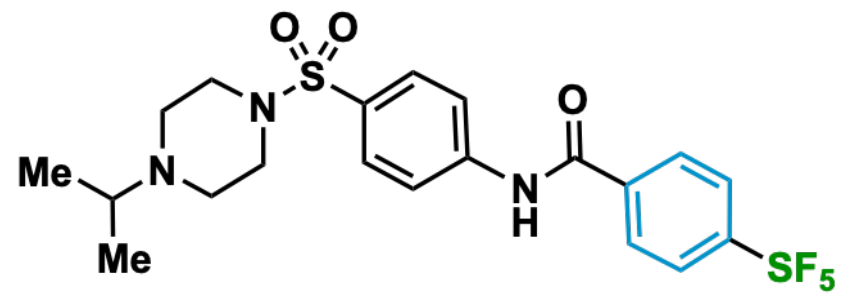

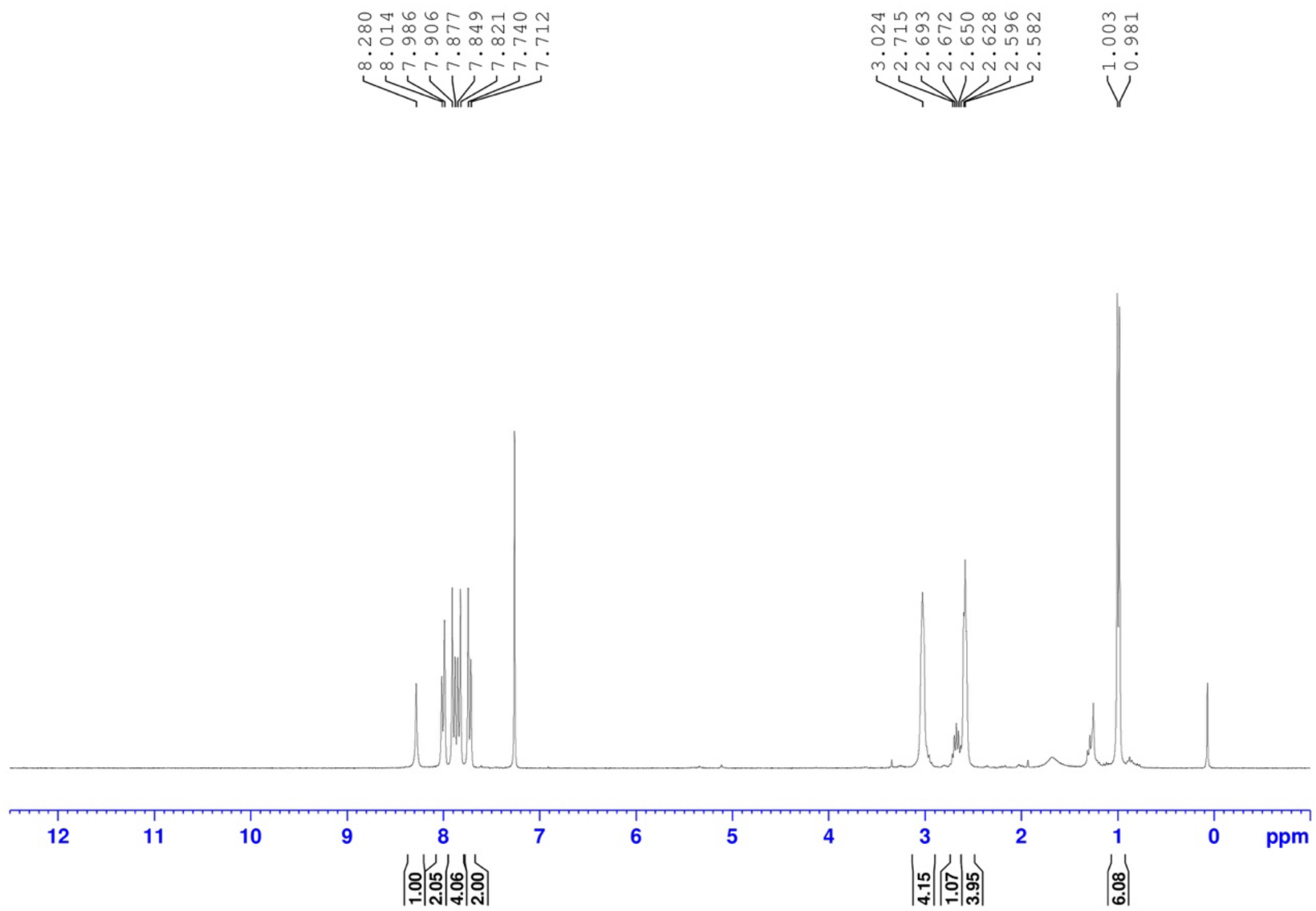

Figure S68. ^{1}H NMR spectrum of *N*-(4-((4-isopropylpiperazin-1-yl)sulfonyl)phenyl)-4-(pentafluoro-λ^6-sulfanyl)benzamide (**10**) in $CDCl_3$.

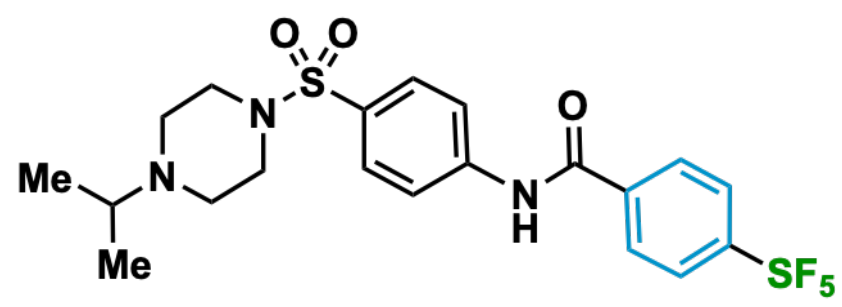

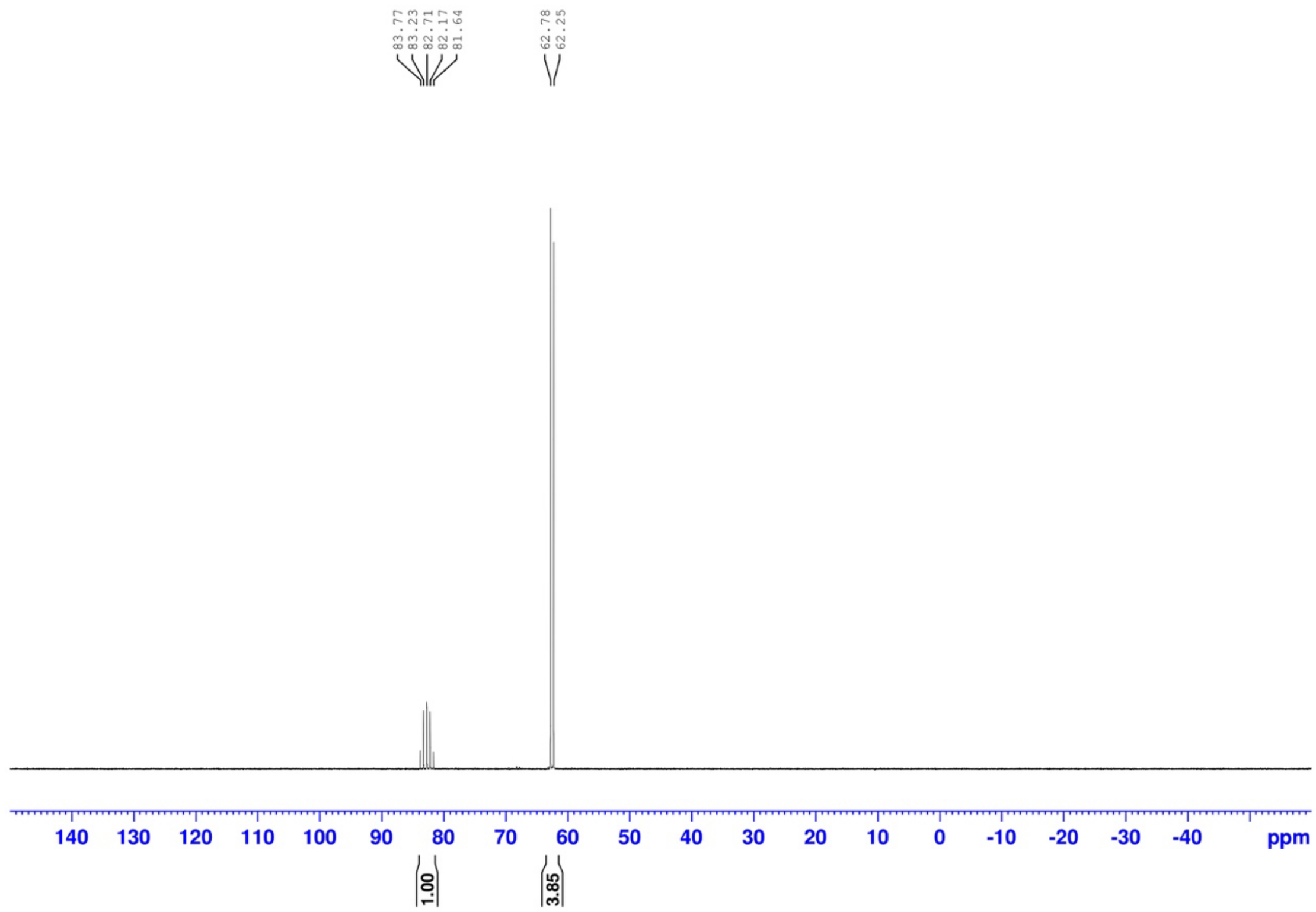

Figure S69. ^{19}F NMR spectrum of *N*-(4-((4-isopropylpiperazin-1-yl)sulfonyl)phenyl)-4-(pentafluoro-λ^6-sulfanyl)benzamide (**10**) in $CDCl_3$.

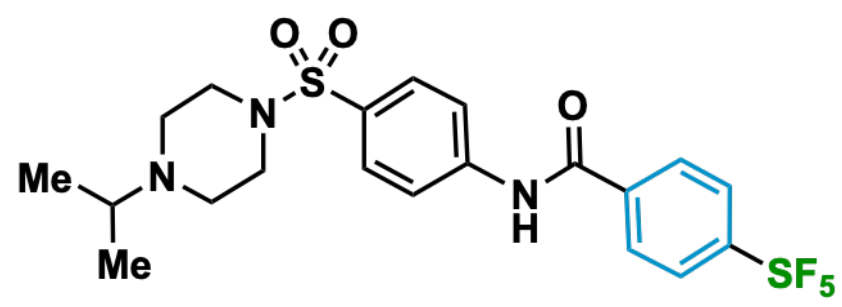

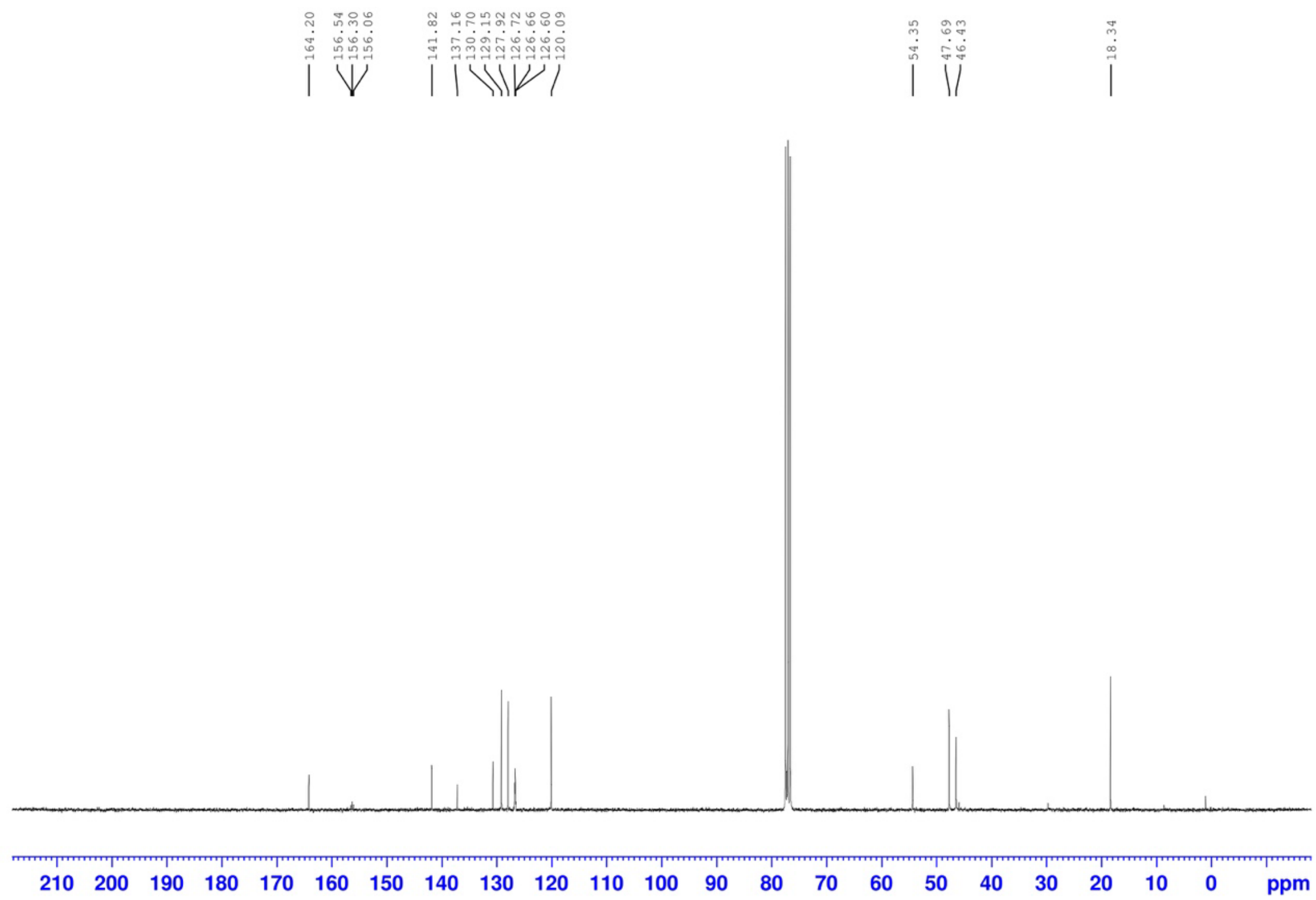

Figure S70. $^{13}C\{^1H\}$ NMR spectrum of *N*-(4-((4-isopropylpiperazin-1-yl)sulfonyl)phenyl)-4-(pentafluoro-λ^6-sulfanyl)benzamide (**10**) in $CDCl_3$.

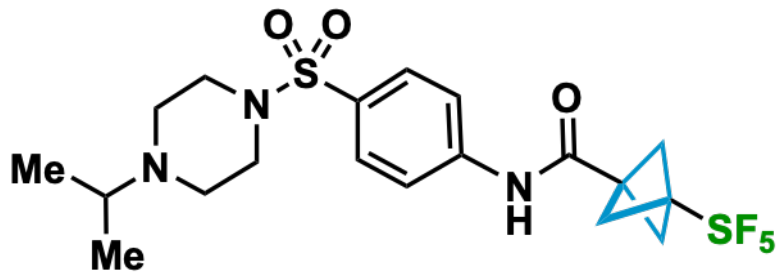

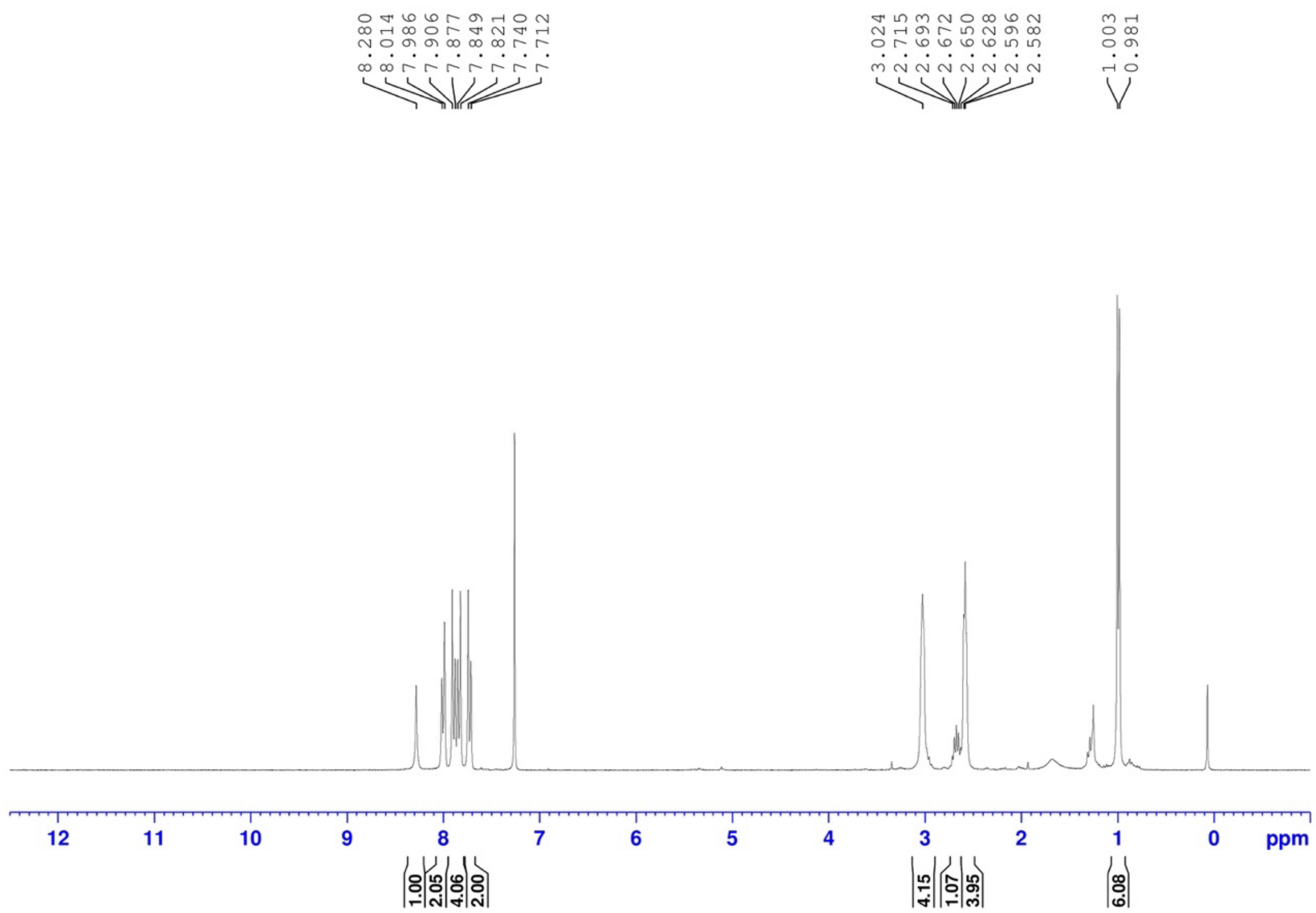

Figure S71. ^{1}H NMR spectrum of *N*-(4-((4-isopropylpiperazin-1-yl)sulfonyl)phenyl)-3-(pentafluoro-λ^6-sulfanyl)bicyclo[1.1.1]pentane-1-carboxamide (**12**) in $CDCl_3$.

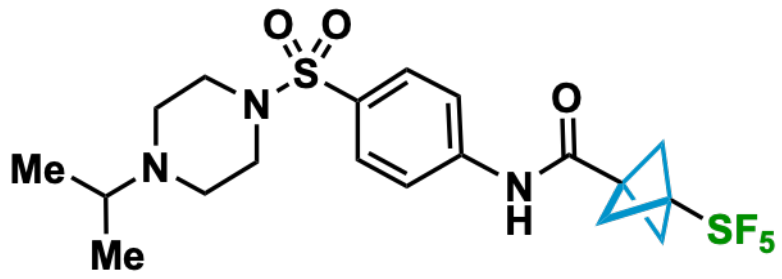

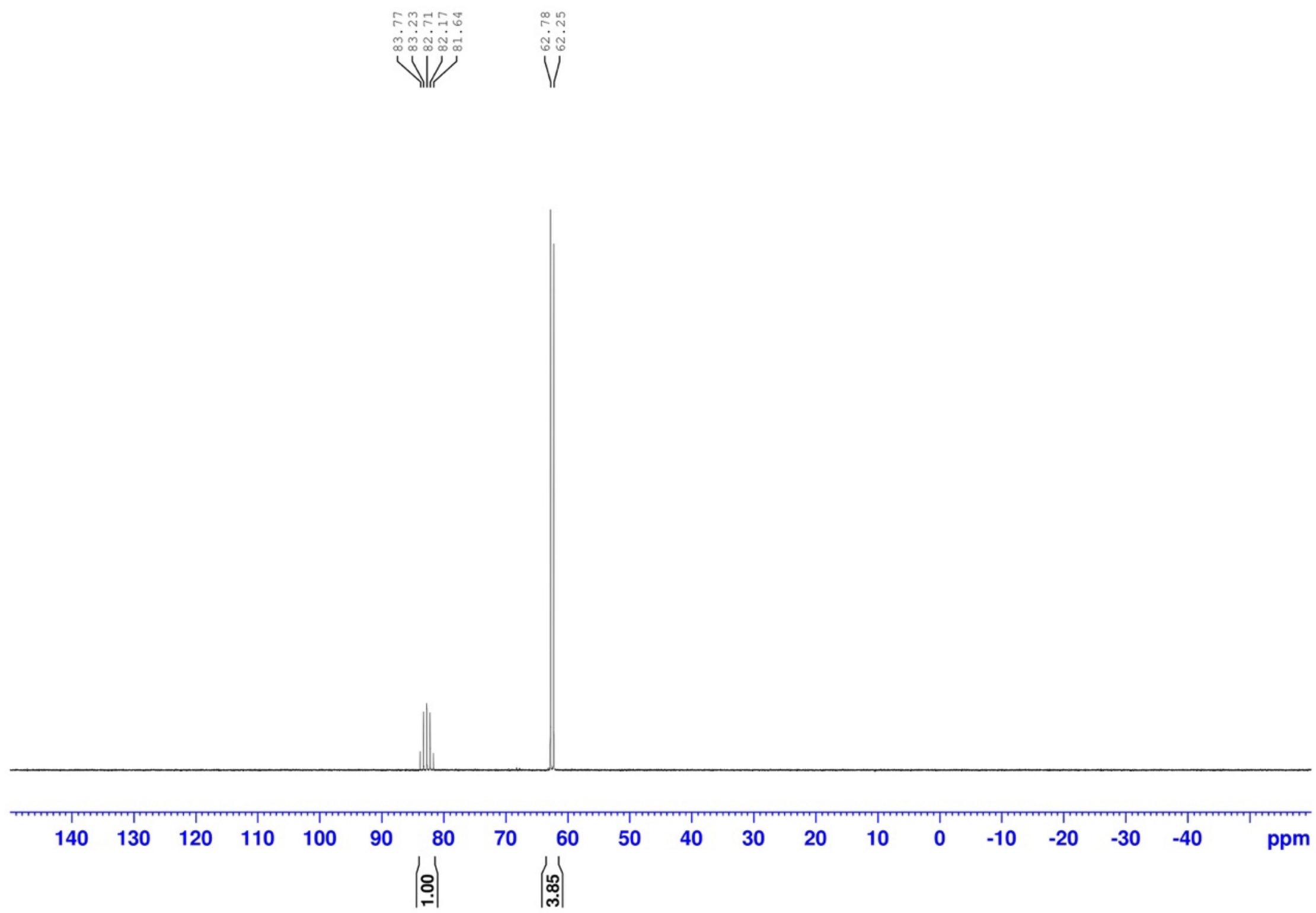

Figure S72. ^{19}F NMR spectrum of *N*-(4-((4-isopropylpiperazin-1-yl)sulfonyl)phenyl)-3-(pentafluoro-λ^6-sulfanyl)bicyclo[1.1.1]pentane-1-carboxamide (**12**) in $CDCl_3$.

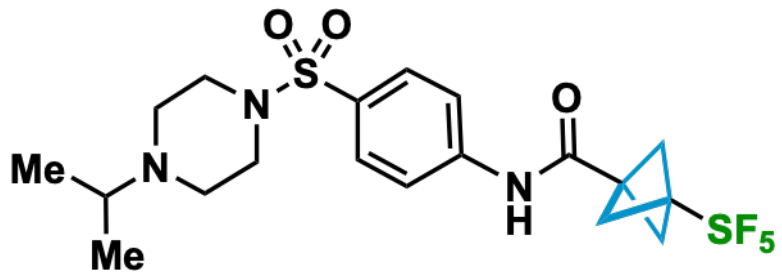

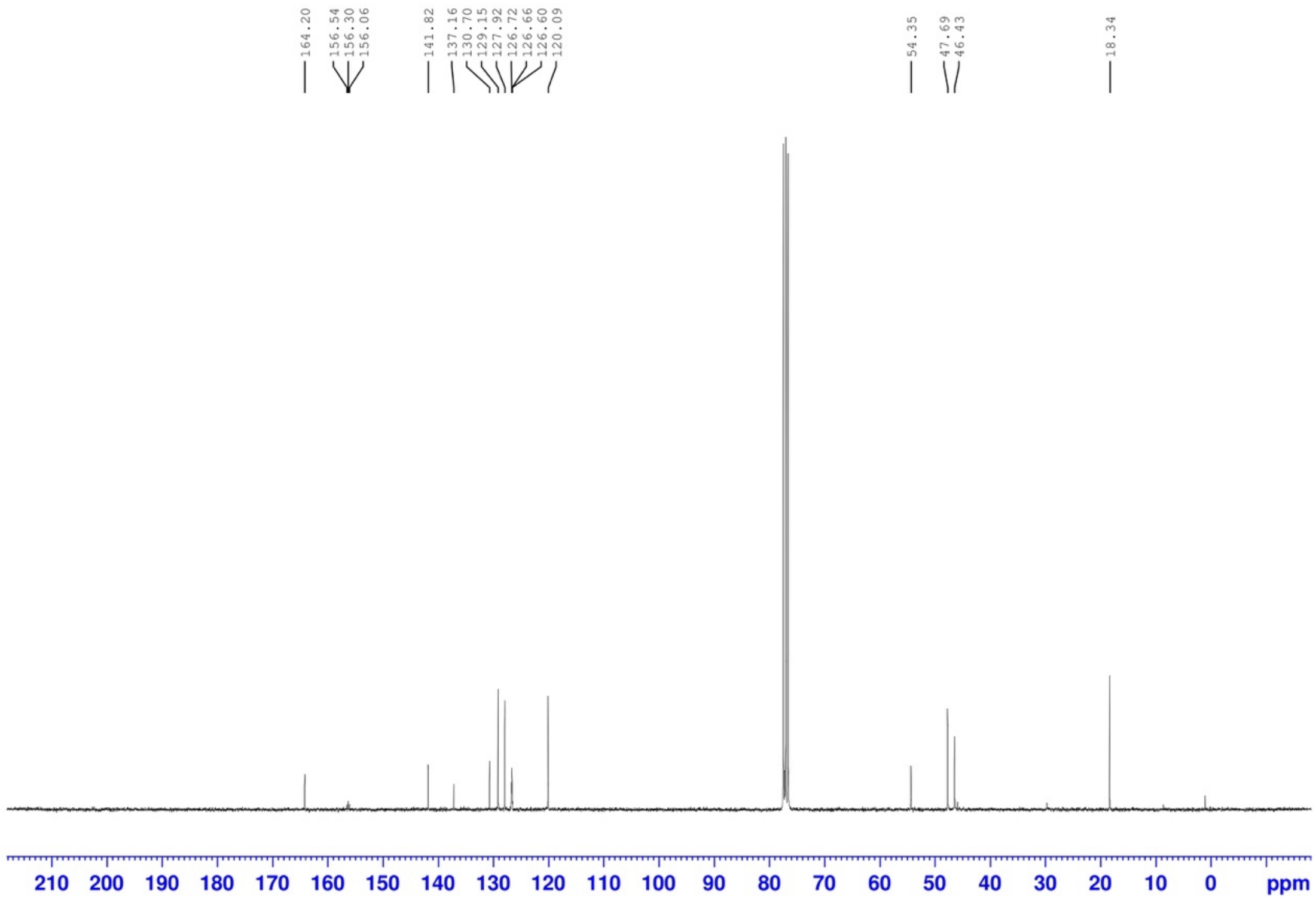

Figure S73. $^{13}C\{^{1}H\}$ NMR spectrum of *N*-(4-((4-isopropylpiperazin-1-yl)sulfonyl)phenyl)-3-(pentafluoro-λ^6-sulfanyl)bicyclo[1.1.1]pentane-1-carboxamide (**12**) in $CDCl_3$.

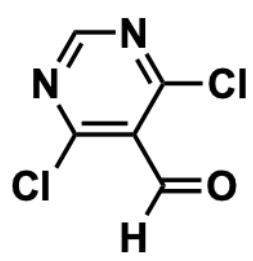

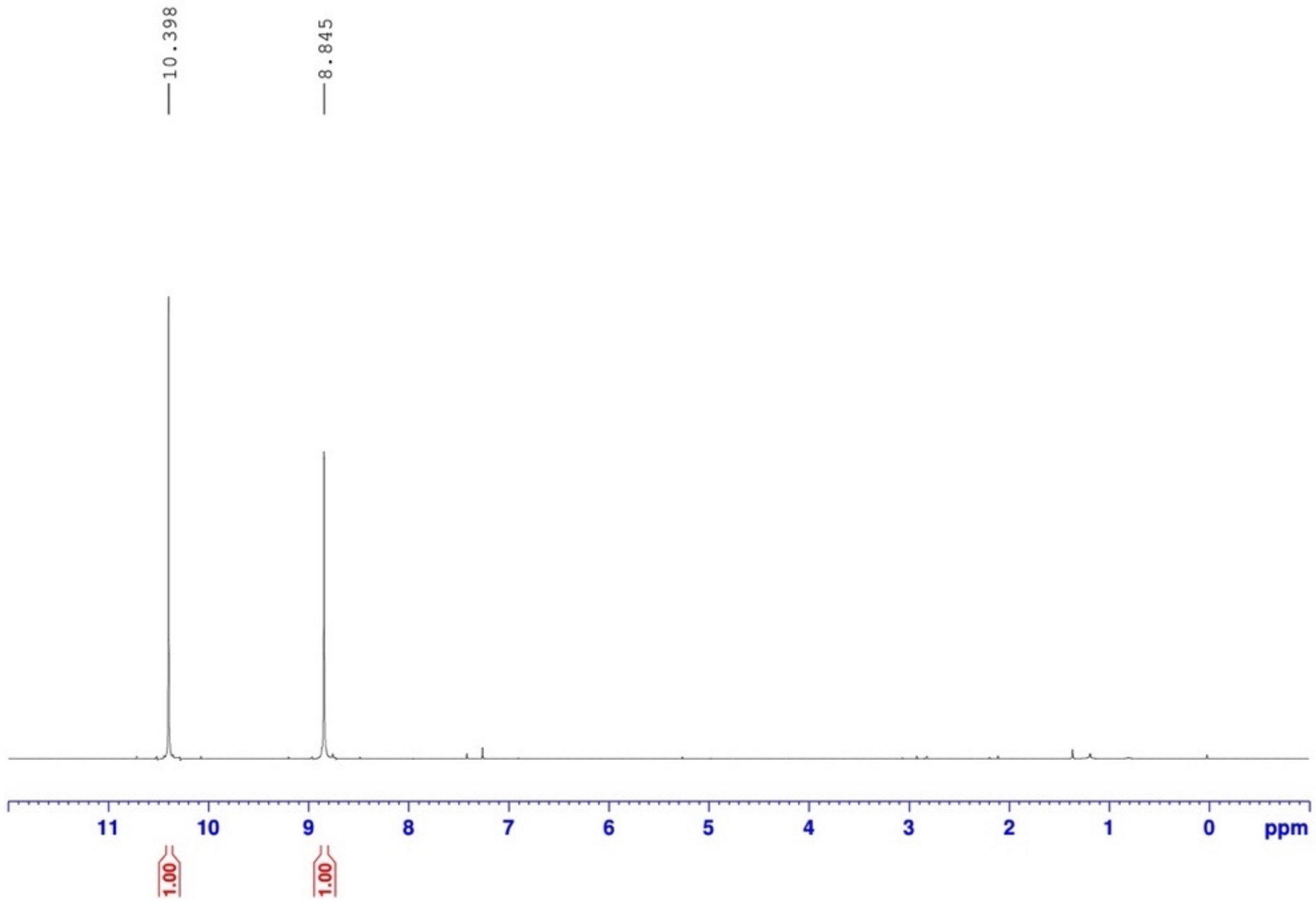

Figure S74. ^{1}H NMR spectrum of 4,6-dichloropyrimidine-5-carbaldehyde (**S-5)** in $CDCl_3$.

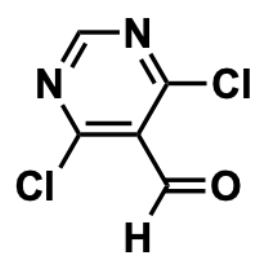

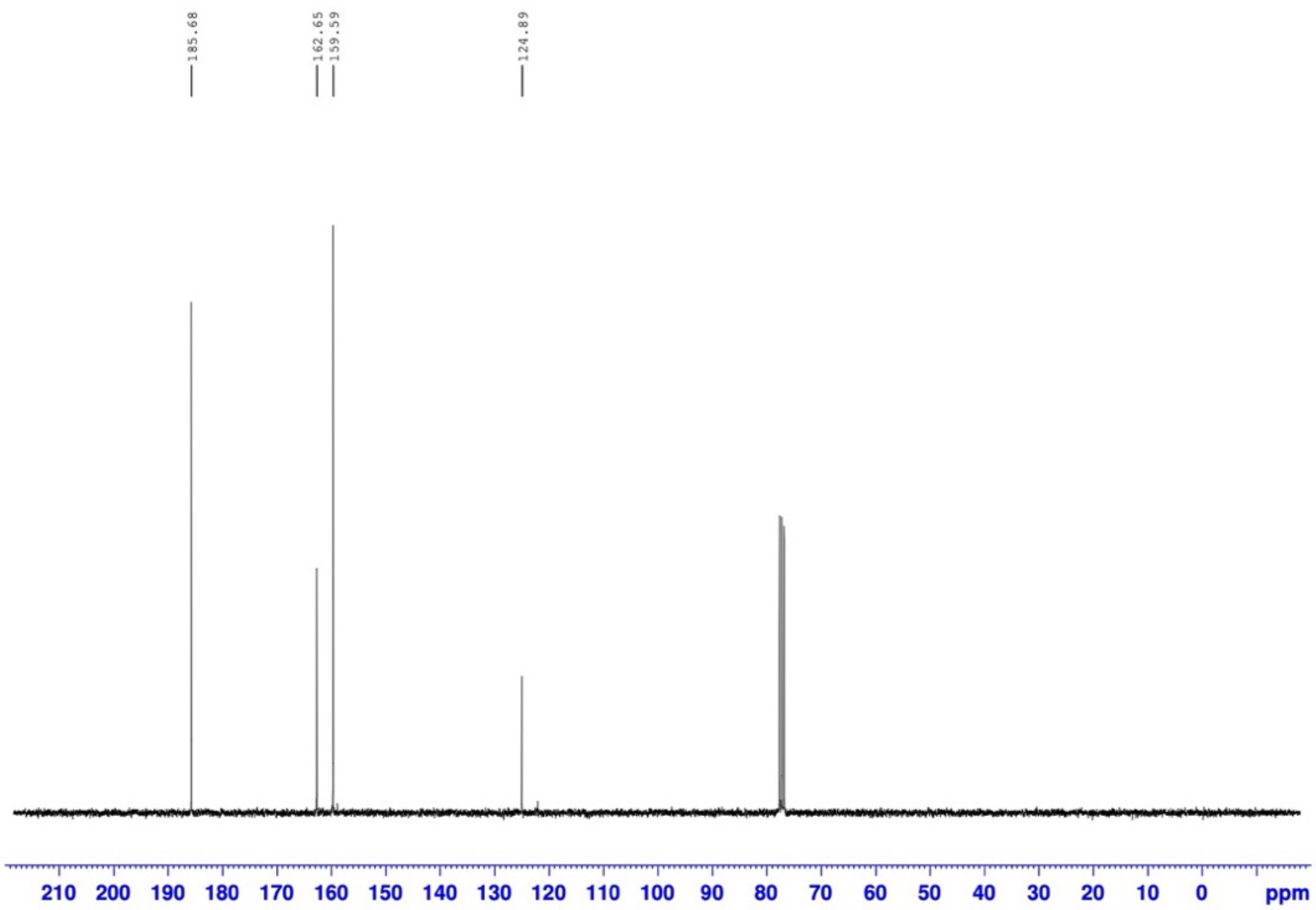

Figure S75. $^{13}C\{^{1}H\}$ NMR spectrum of 4,6-dichloropyrimidine-5-carbaldehyde (**S-5**) in $CDCl_3$.

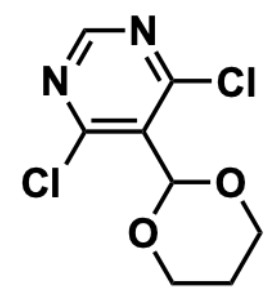

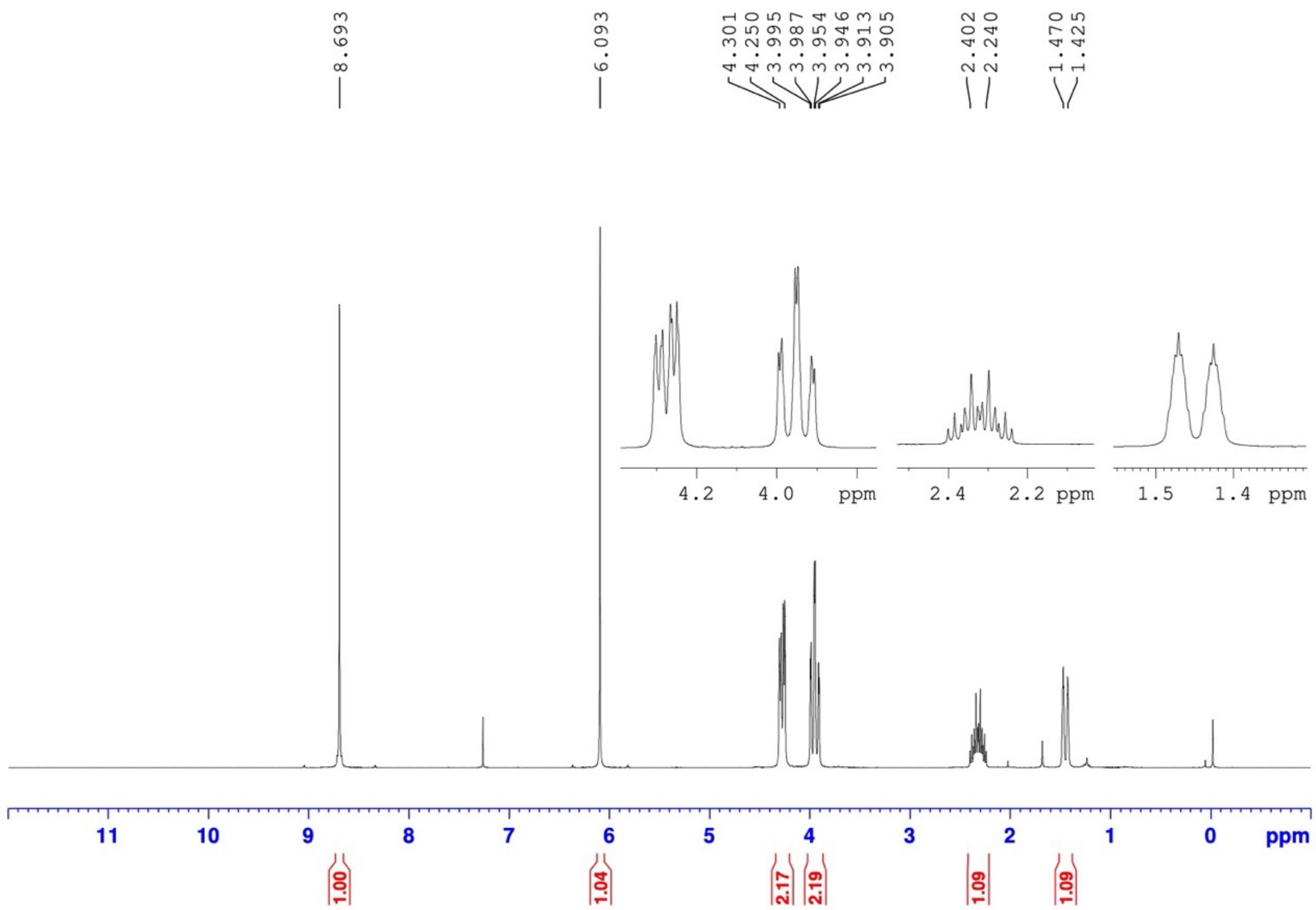

Figure S76. ^{1}H NMR spectrum of 4,6-dichloro-5-(1,3-dioxan-2-yl)pyrimidine (**15**) in $CDCl_3$.

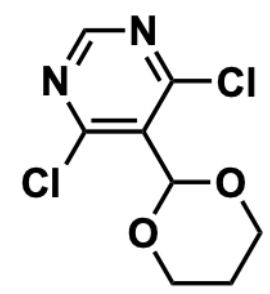

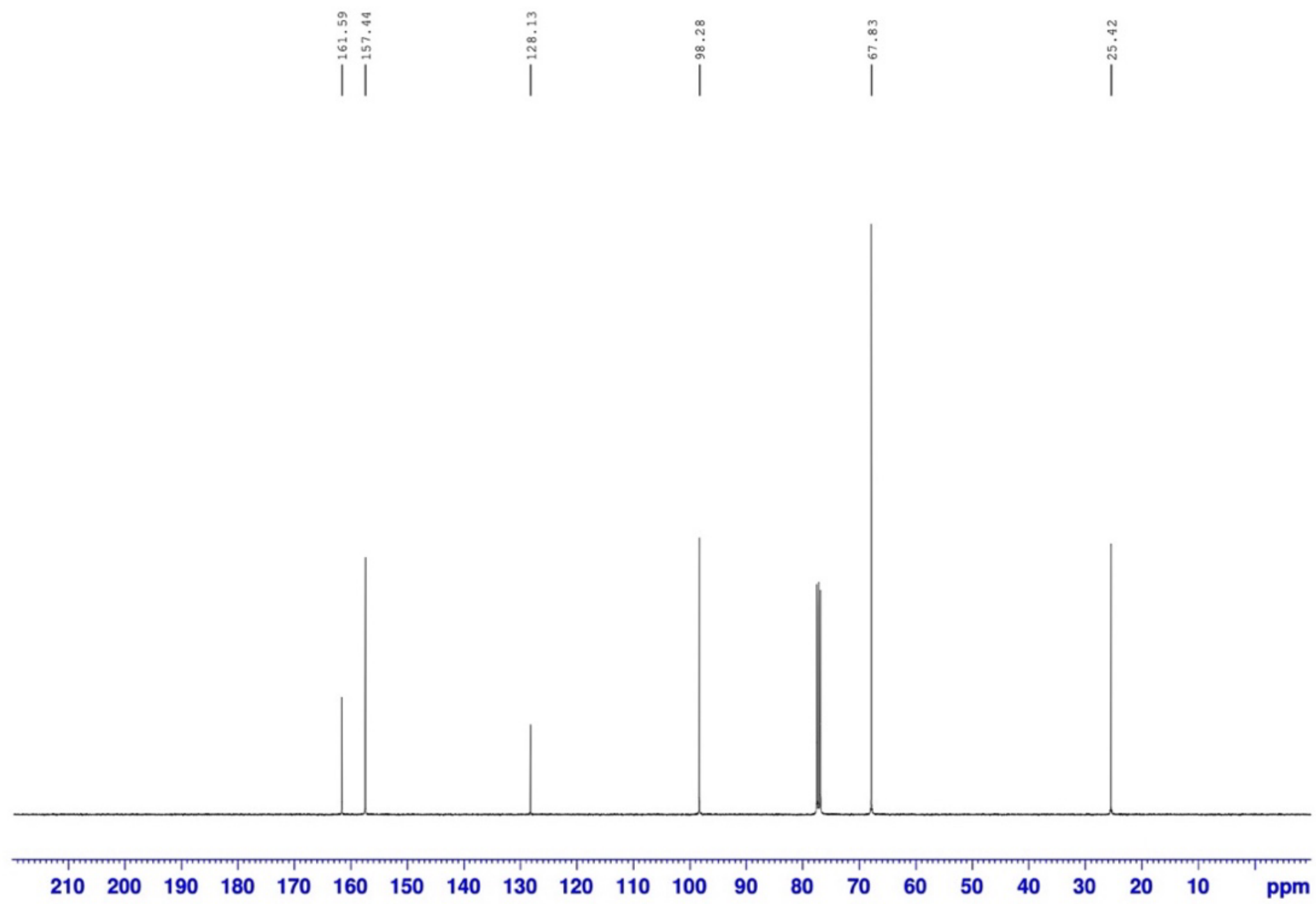

Figure S77. $^{13}C\{^{1}H\}$ NMR spectrum of 4,6-dichloro-5-(1,3-dioxan-2-yl)pyrimidine (**15**) in $CDCl_3$.

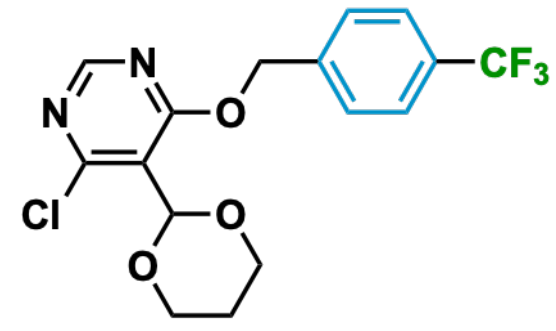

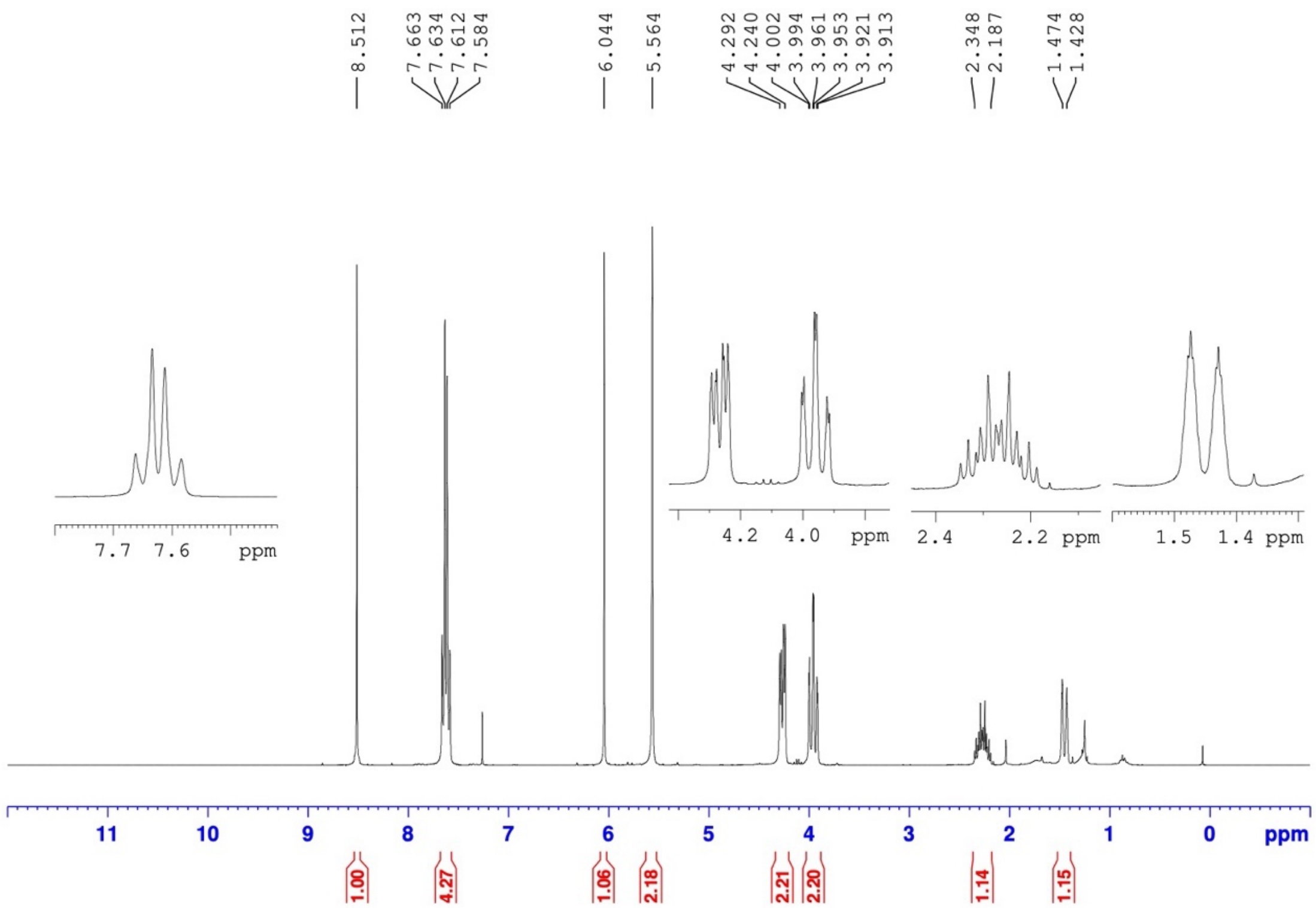

Figure S78. ^{1}H NMR spectrum of 4-chloro-5-(1,3-dioxan-2-yl)-6-((4-(trifluoromethyl)benzyl)oxy)pyrimidine (**S-6**) in $CDCl_3$.

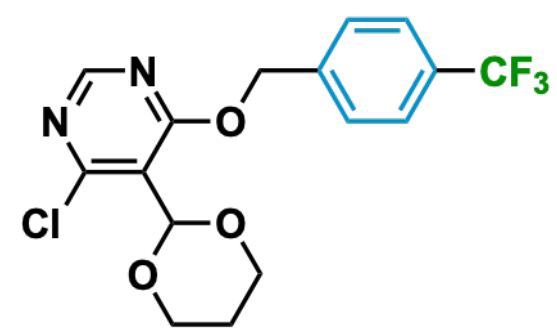

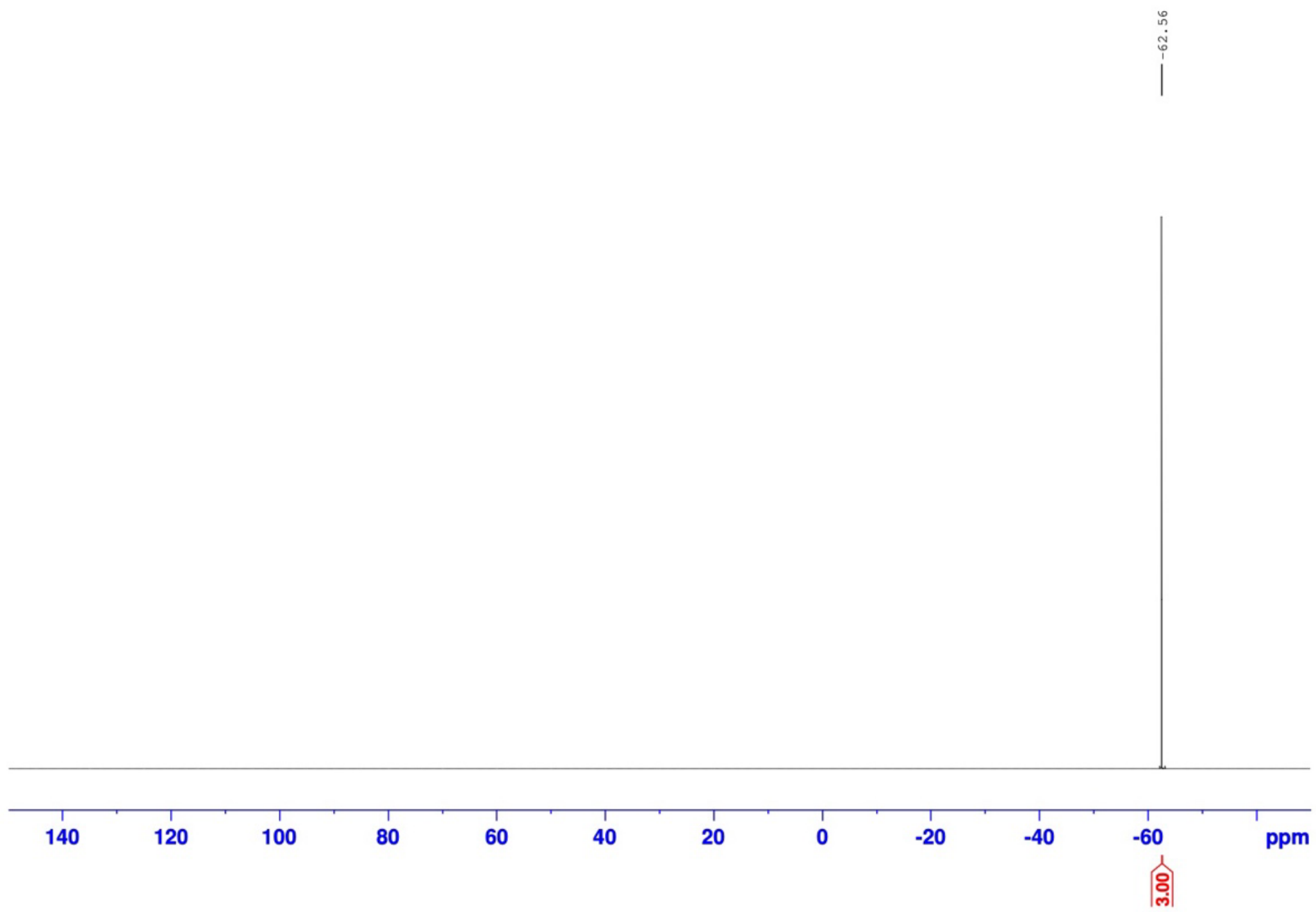

Figure S79. ^{19}F NMR spectrum of 4-chloro-5-(1,3-dioxan-2-yl)-6-((4-(trifluoromethyl)benzyl)oxy)pyrimidine (**S-6**) in $CDCl_3$.

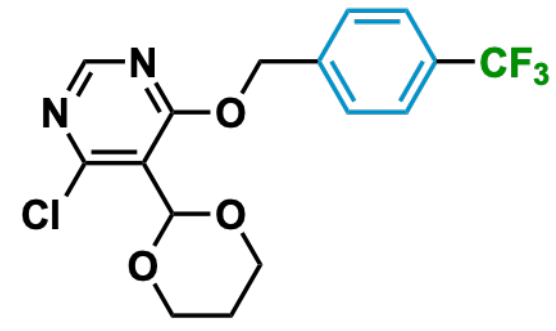

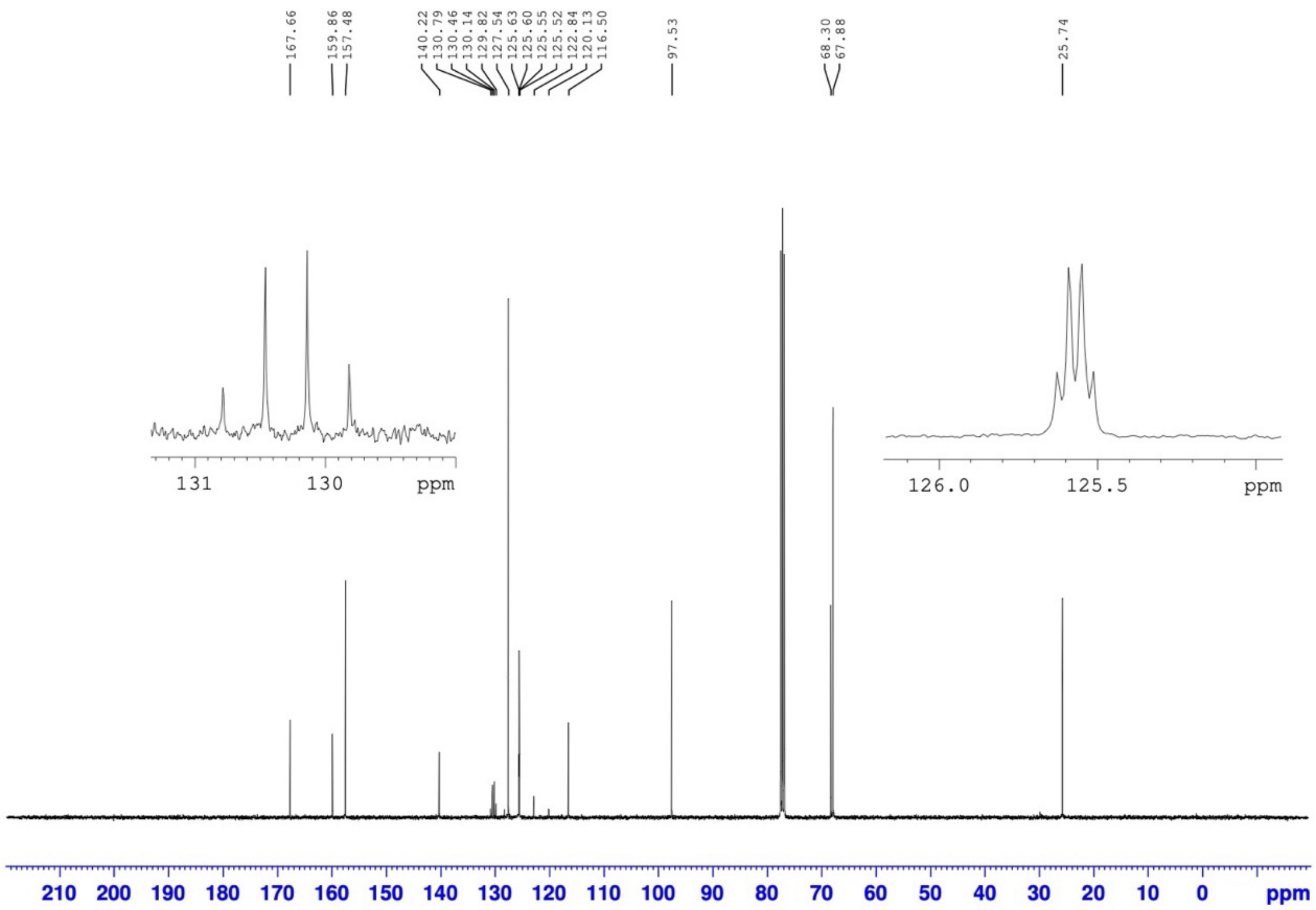

Figure S80. $^{13}C\{^{1}H\}$ NMR spectrum of 4-chloro-5-(1,3-dioxan-2-yl)-6-((4-(trifluoromethyl)benzyl)oxy)-pyrimidine (**S-6**) in $CDCl_3$.

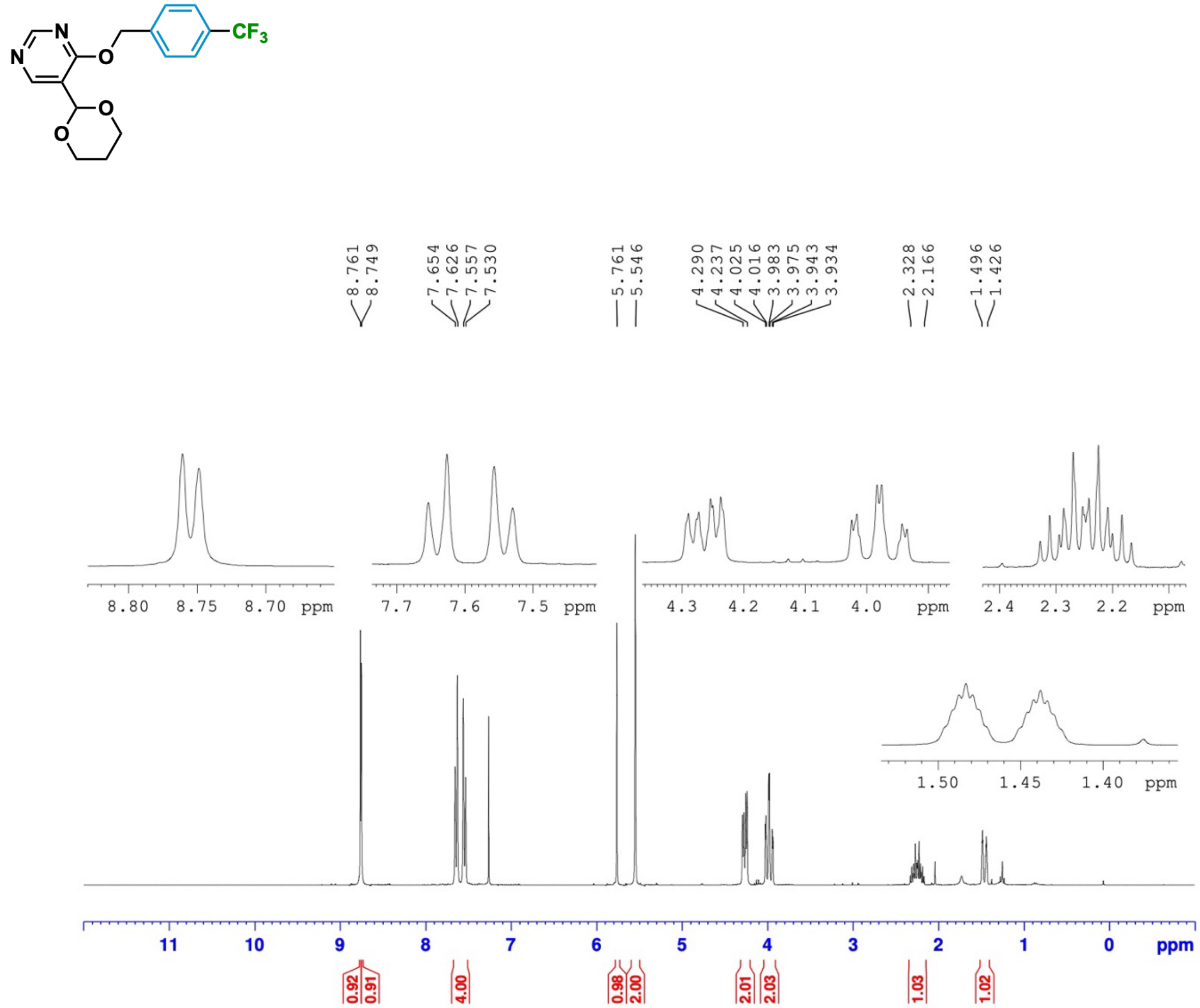

Figure S81. ^{1}H NMR spectrum of 5-(1,3-dioxan-2-yl)-4-((4-(trifluoromethyl)benzyl)oxy)pyrimidine (**13**) in $CDCl_3$.

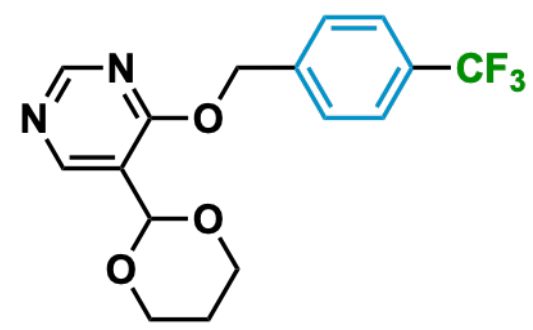

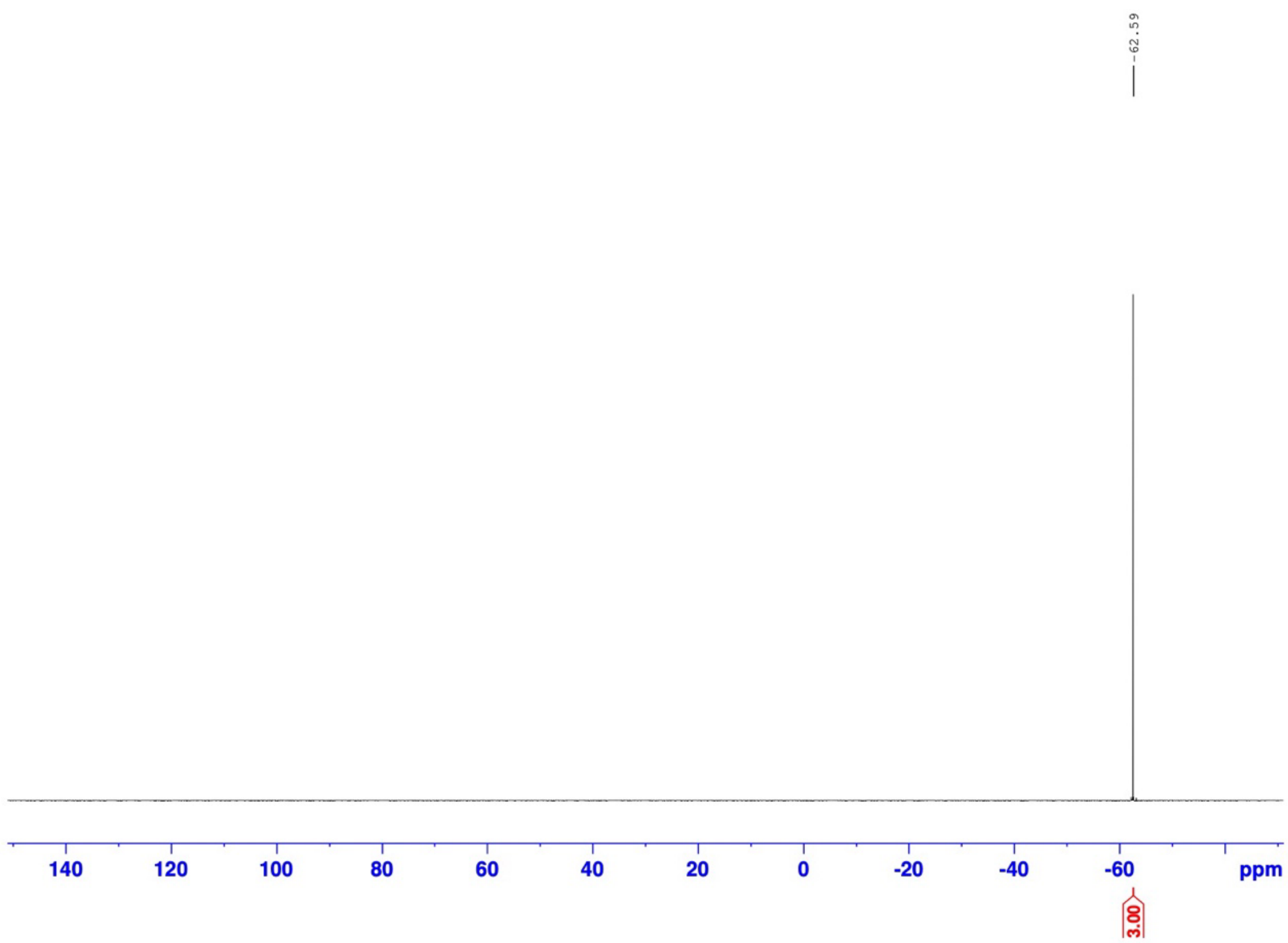

Figure S82. ^{19}F NMR spectrum of 5-(1,3-dioxan-2-yl)-4-((4-(trifluoromethyl)benzyl)oxy)pyrimidine (**13**) in $CDCl_3$.

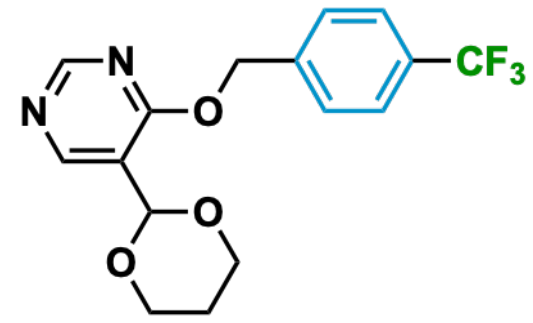

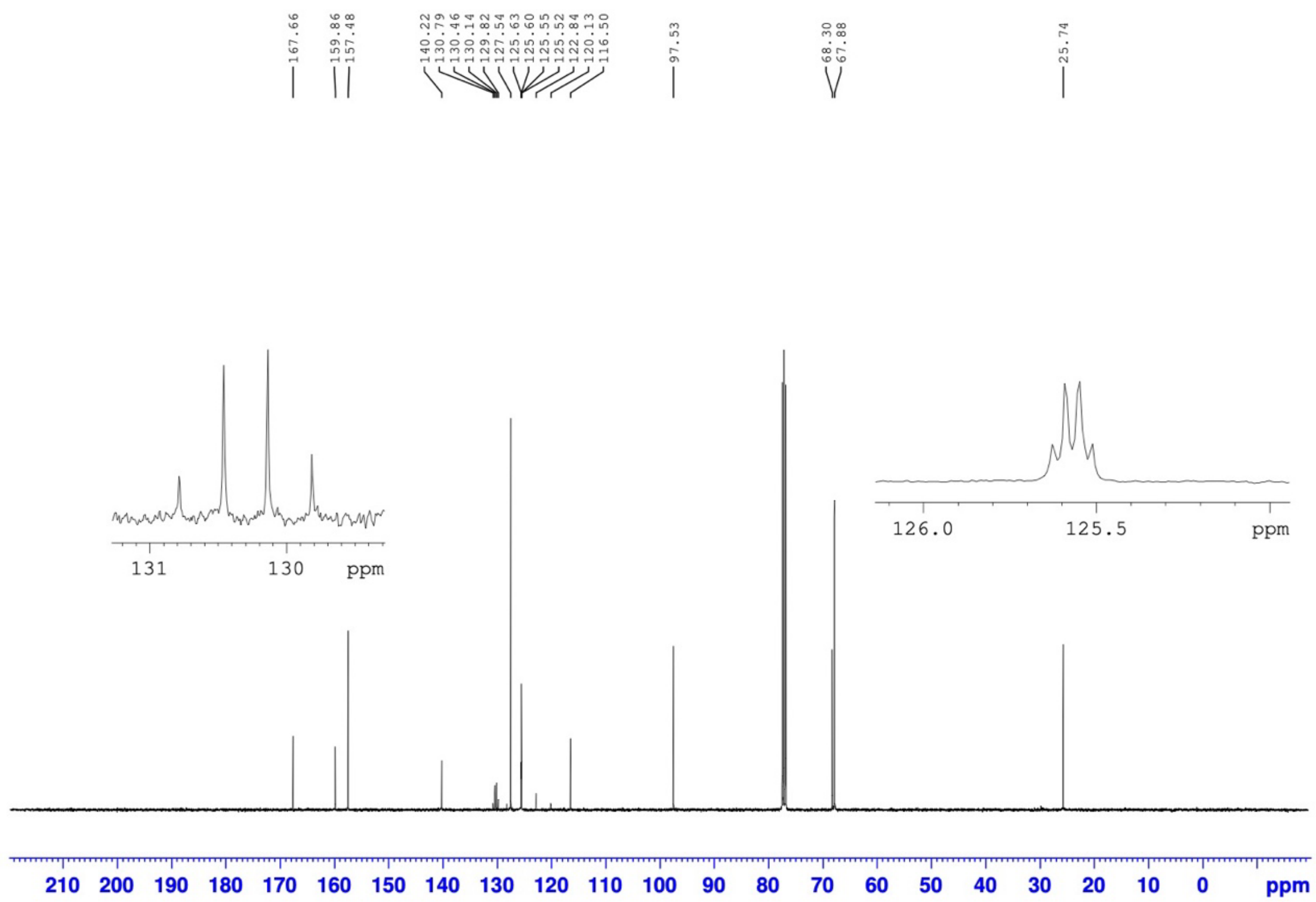

Figure S83. $^{13}C\{^{1}H\}$ NMR spectrum of 5-(1,3-dioxan-2-yl)-4-((4-(trifluoromethyl)benzyl)oxy)pyrimidine (**13**) in $CDCl_3$.

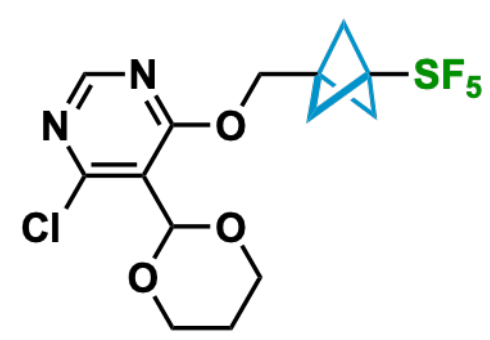

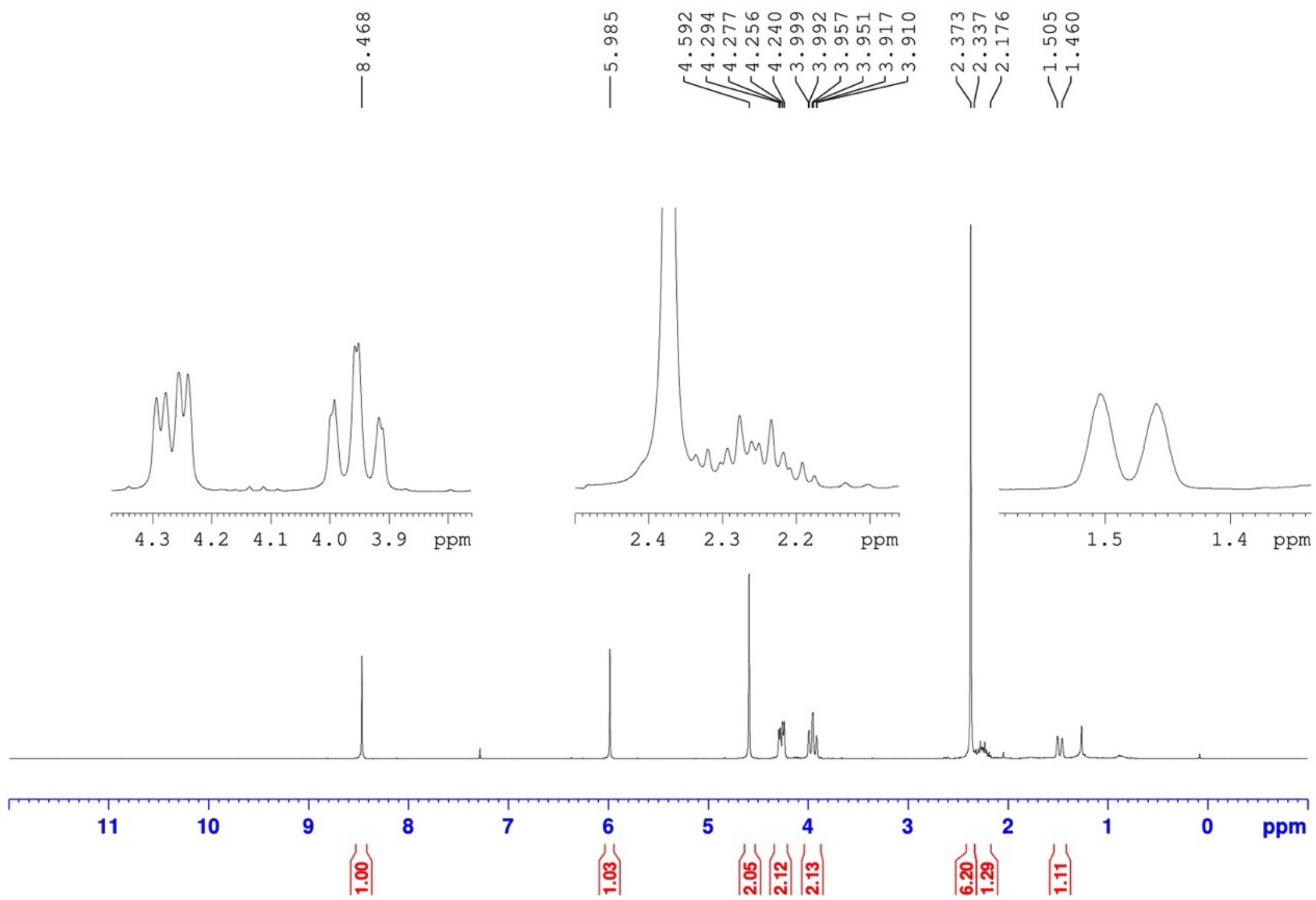

Figure S84. ^1H NMR spectrum of 4-chloro-5-(1,3-dioxan-2-yl)-6-((3-(pentafluoro-λ^6-sulfanyl)bicyclo[1.1.1]-pentan-1-yl)methoxy)pyrimidine (**S-7**) in $CDCl_3$.

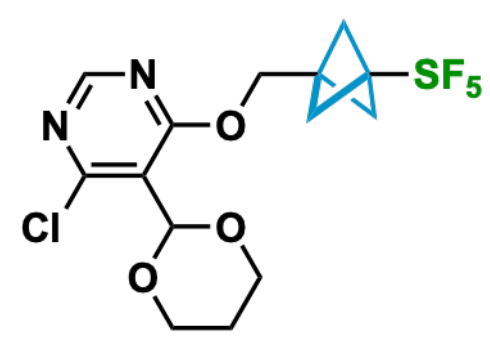

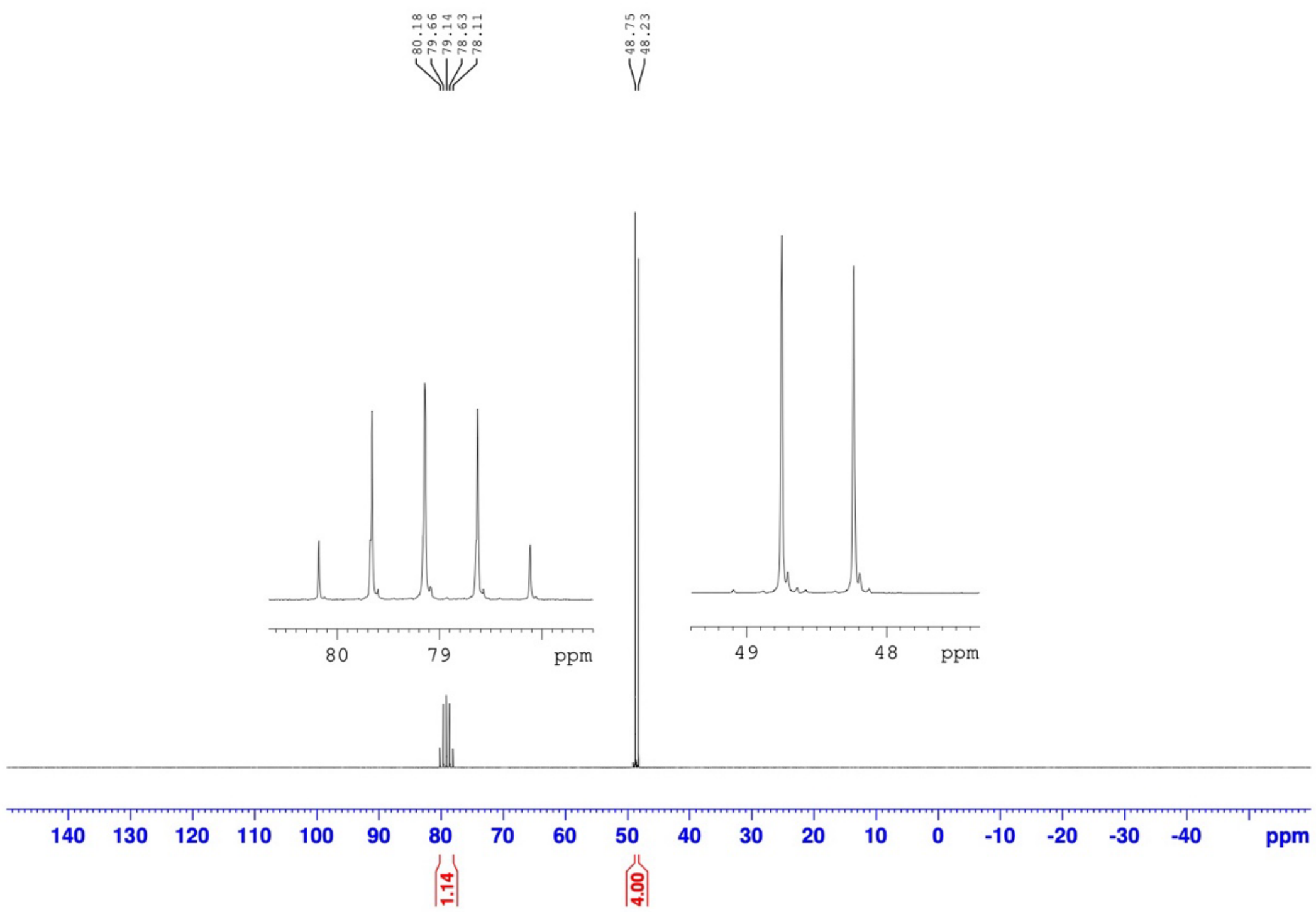

Figure S85. ^{19}F NMR spectrum of 4-chloro-5-(1,3-dioxan-2-yl)-6-((3-(pentafluoro-λ^6-sulfanyl)bicyclo[1.1.1]-pentan-1-yl)methoxy)pyrimidine (**S-7**) in $CDCl_3$.

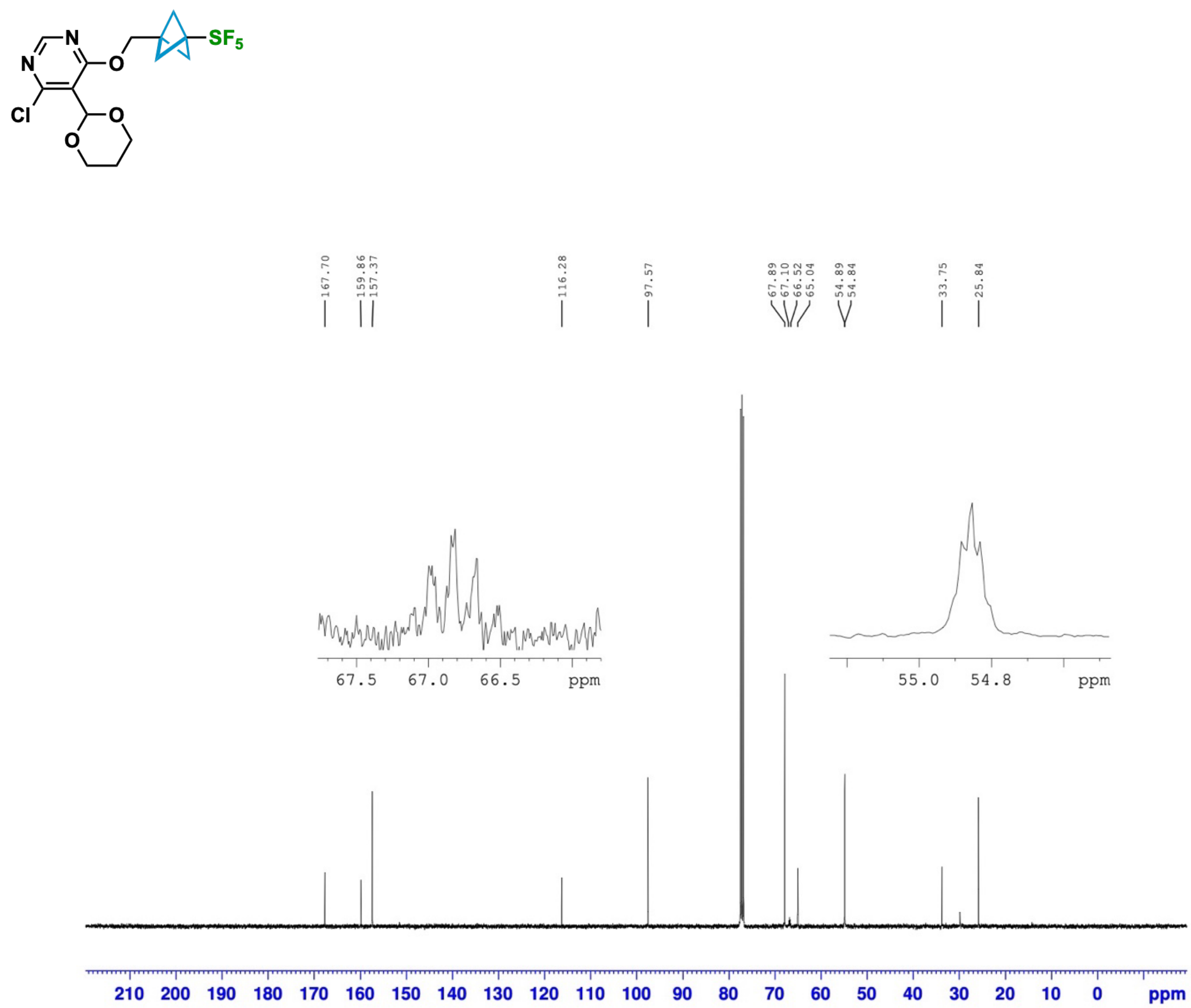

Figure S86. $^{13}C\{^1H\}$ NMR spectrum of 4-chloro-5-(1,3-dioxan-2-yl)-6-((3-(pentafluoro-λ^6-sulfanyl)-bicyclo[1.1.1]pentan-1-yl)methoxy)pyrimidine (**S-7**) in $CDCl_3$.

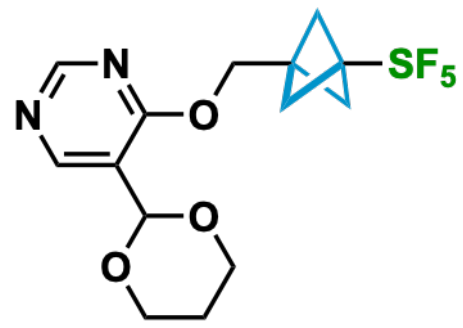

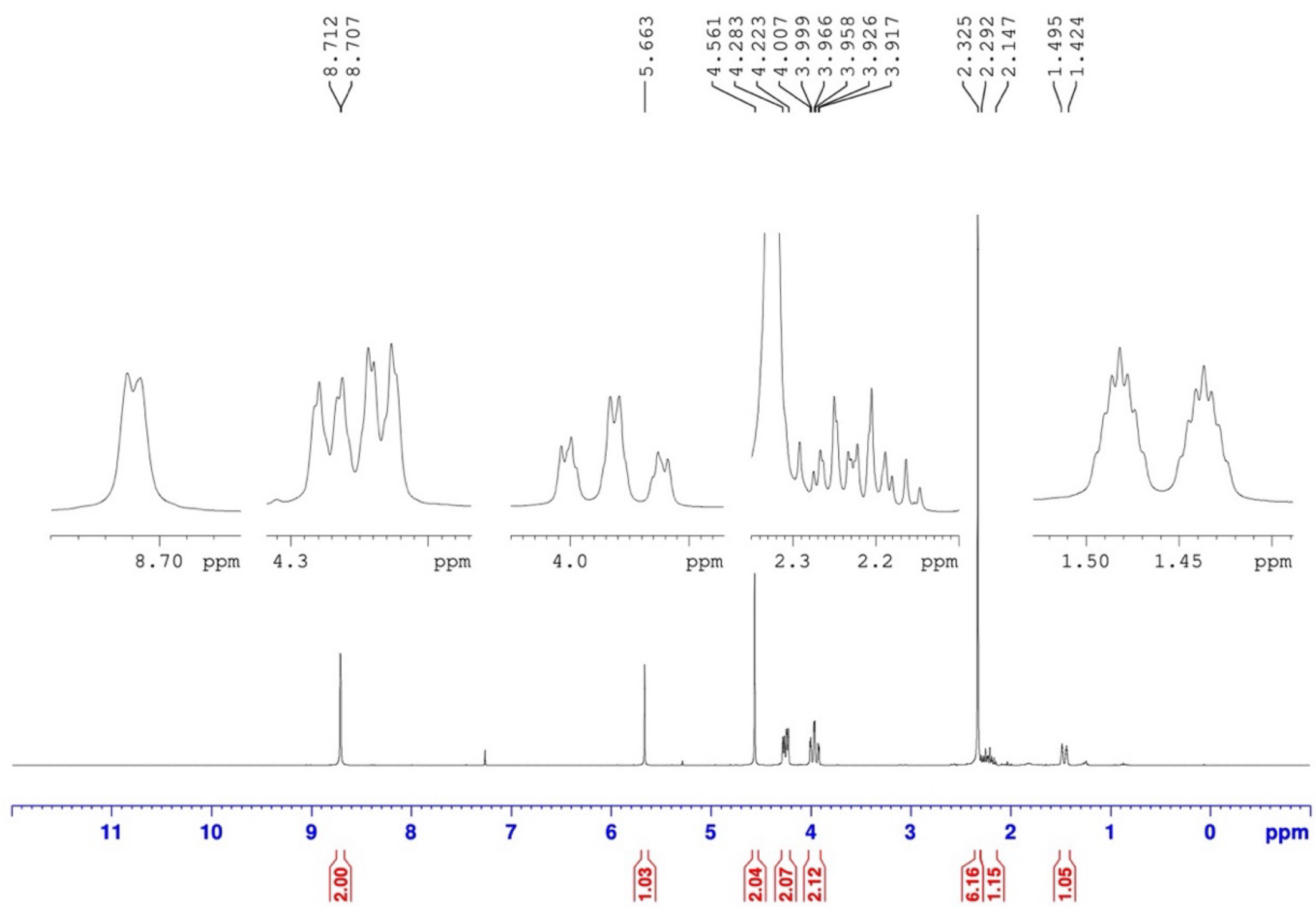

Figure S87. ^{1}H NMR spectrum of 5-(1,3-dioxan-2-yl)-4-((3-(pentafluoro-λ^6-sulfanyl)bicyclo[1.1.1]pentan-1-yl)methoxy)pyrimidine (**16**) in $CDCl_3$.

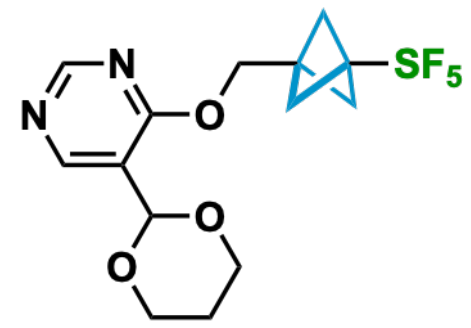

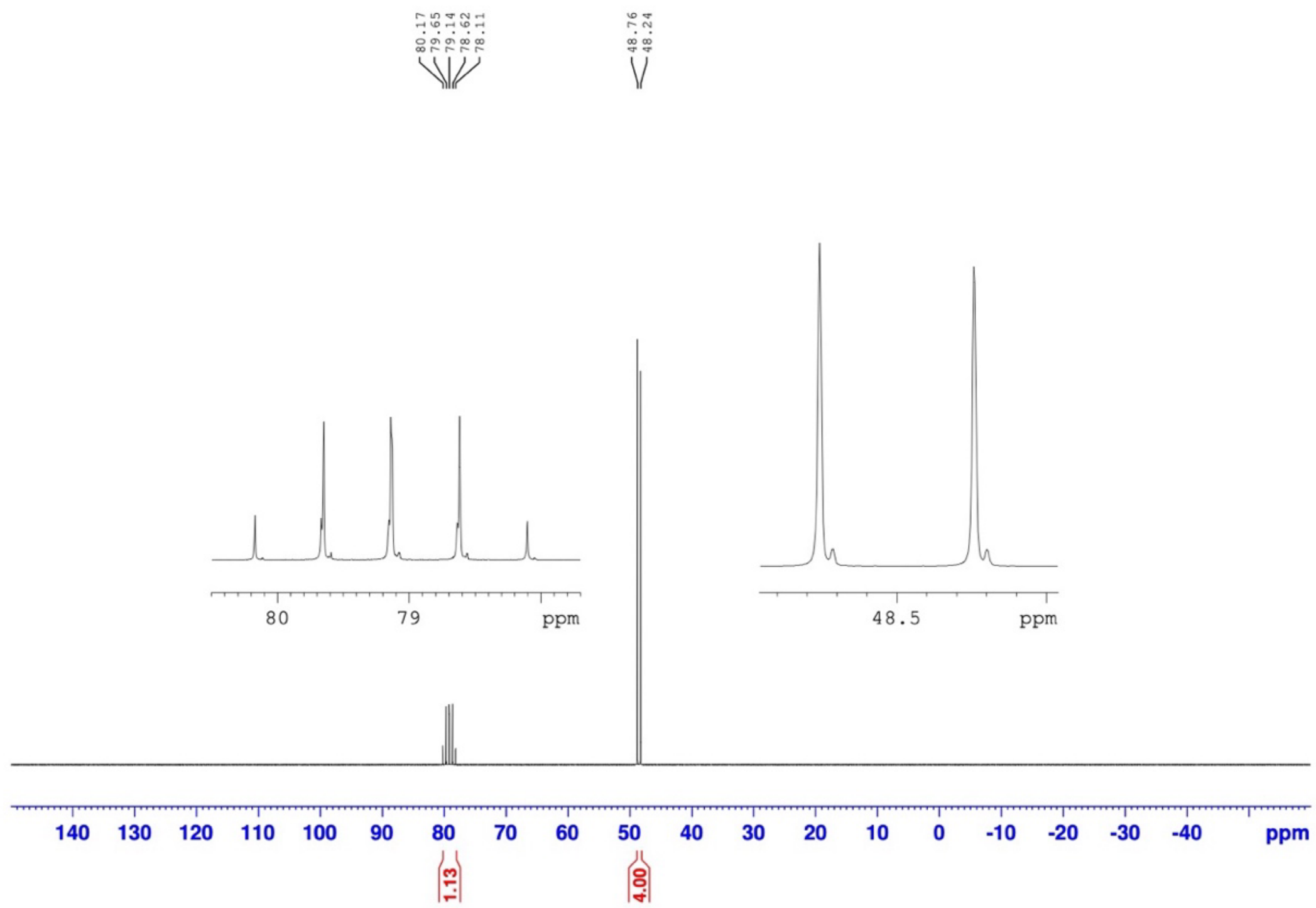

Figure S88. ^{19}F NMR spectrum of 5-(1,3-dioxan-2-yl)-4-((3-(pentafluoro-λ^{6}-sulfanyl)bicyclo[1.1.1]pentan-1-yl)methoxy)pyrimidine (**16**) in $CDCl_3$.

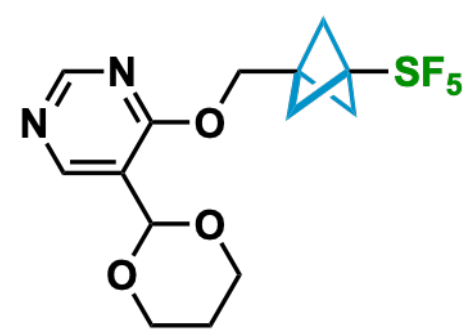

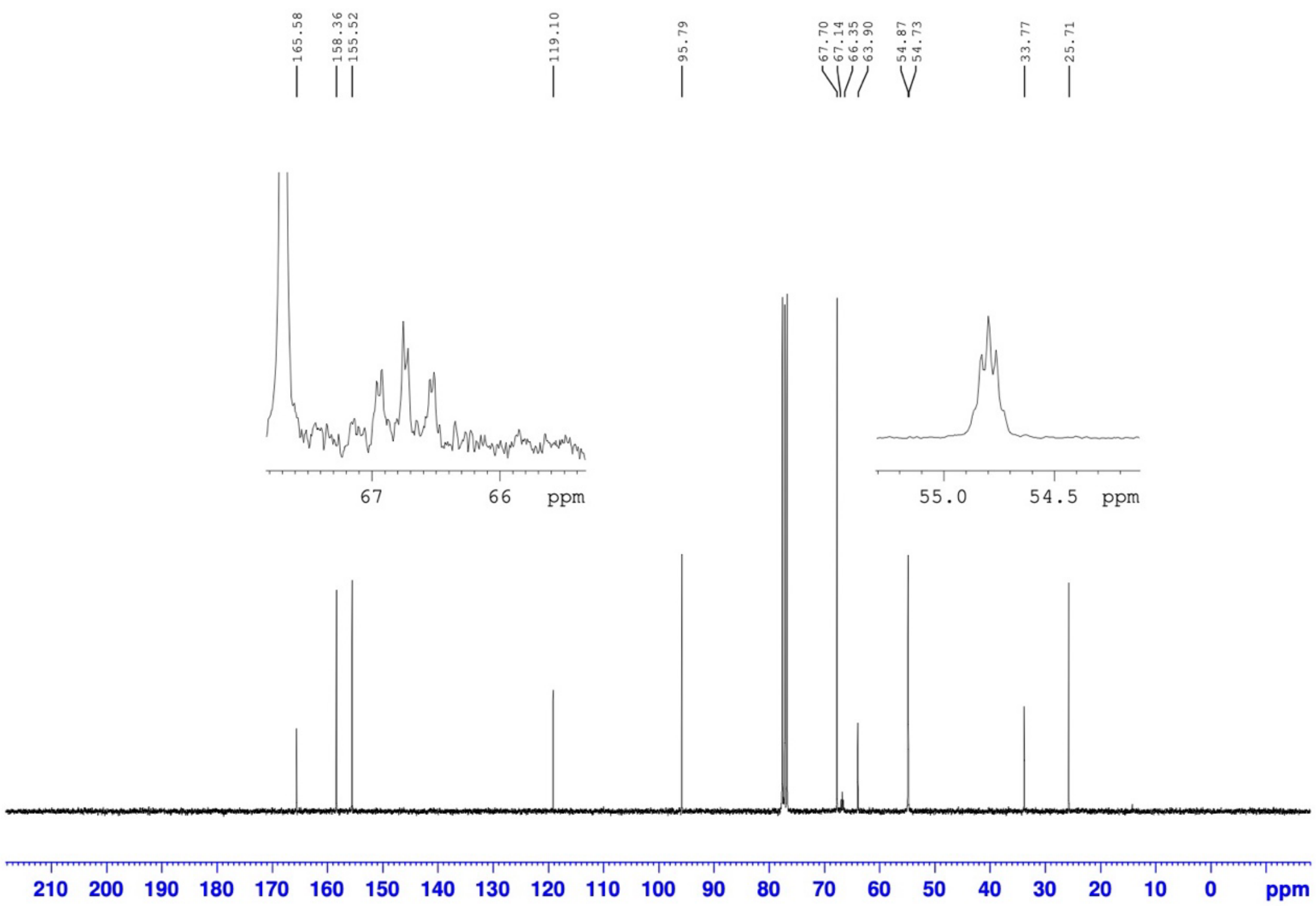

Figure S89. $^{13}C\{^{1}H\}$ NMR spectrum of 5-(1,3-dioxan-2-yl)-4-((3-(pentafluoro-λ^{6}-sulfanyl)bicyclo[1.1.1]-pentan-1-yl)methoxy)pyrimidine (**16**) in $CDCl_3$.

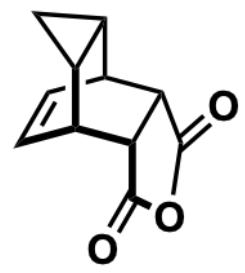

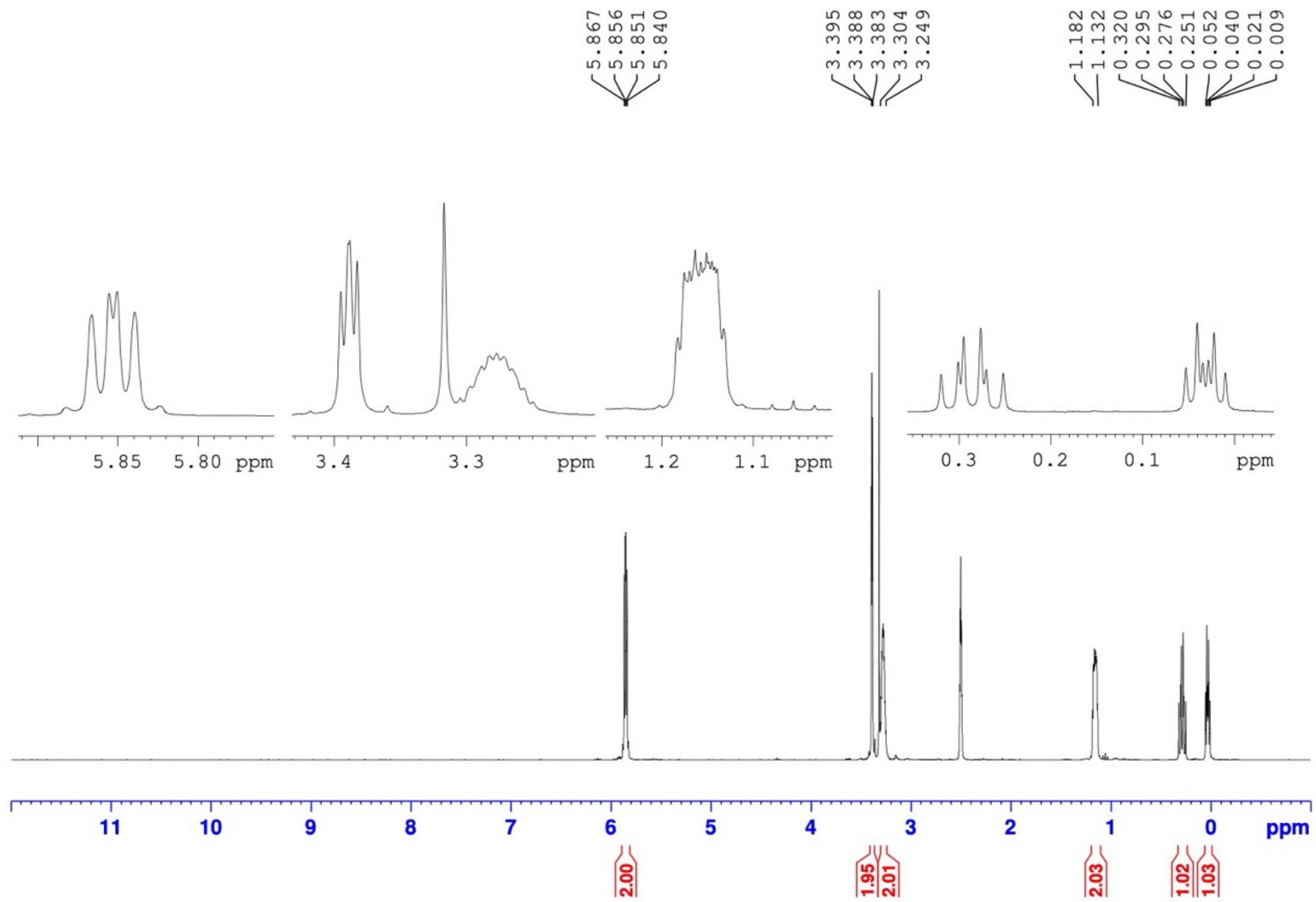

Figure S90. ^{1}H NMR spectrum of (4*R*,4a*R*,5a*S*,6*S*,6a*S*)-4,4a,5,5a,6,6a-hexahydro-1*H*-4,6-ethenocyclopropa[*f*]isobenzofuran-1,3(3a*H*)-dione (**19**) in DMSO-d_6.

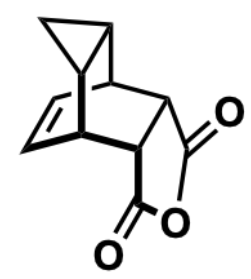

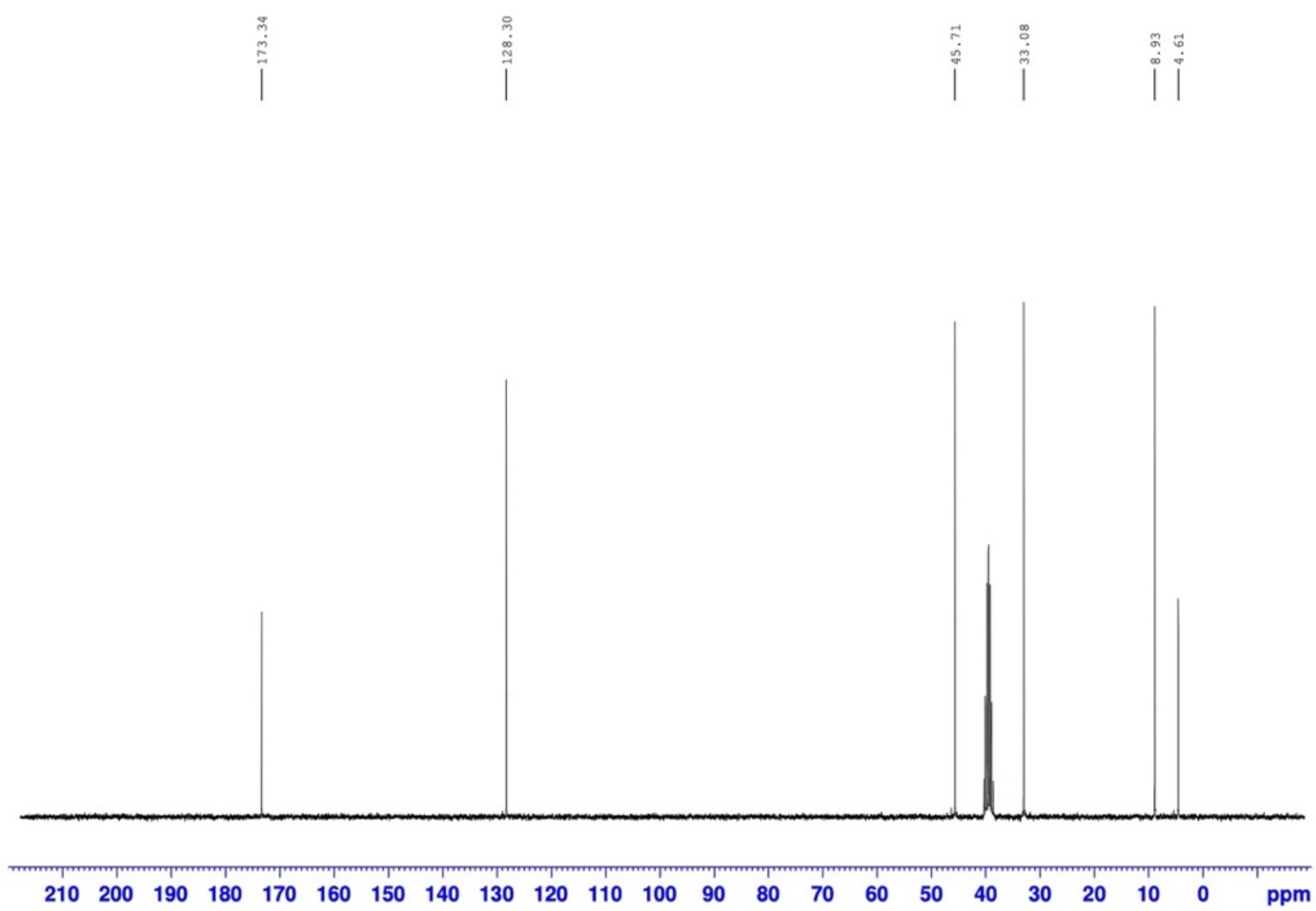

Figure S91. $^{13}C\{^{1}H\}$ NMR spectrum of (4*R*,4a*R*,5a*S*,6*S*,6a*S*)-4,4a,5,5a,6,6a-hexahydro-1*H*-4,6-ethenocyclopropa[*f*]isobenzofuran-1,3(3a*H*)-dione (**19**) in DMSO-*d*6.

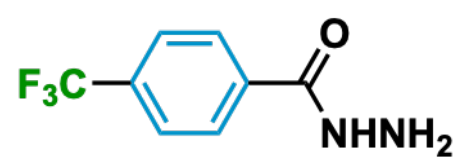

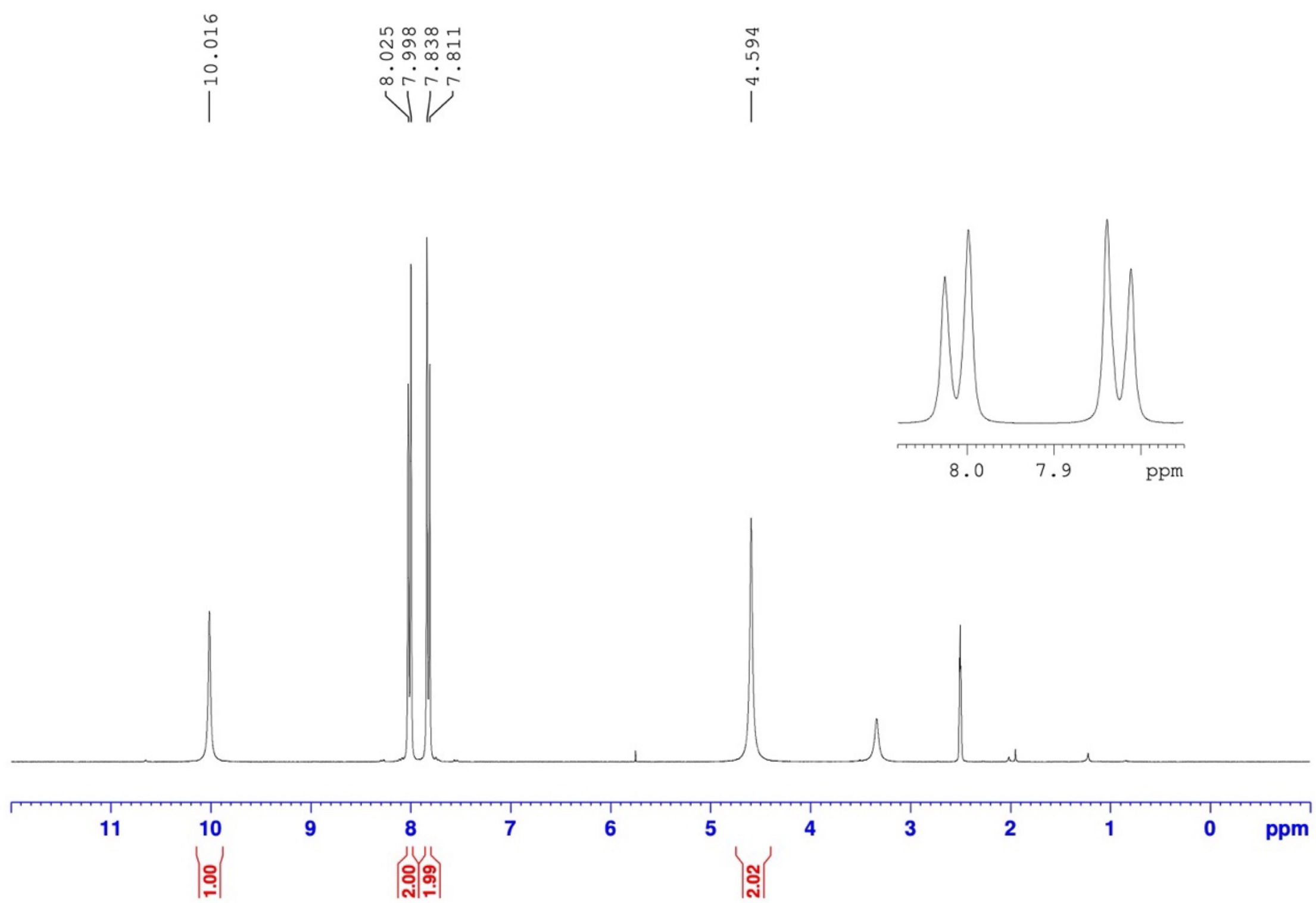

Figure S92. ^{1}H NMR spectrum of 4-(trifluoromethyl)benzohydrazide (**S-8**) in DMSO-d_6.

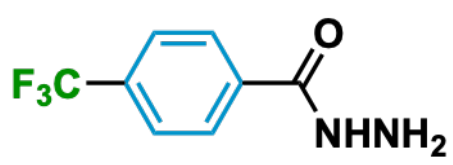

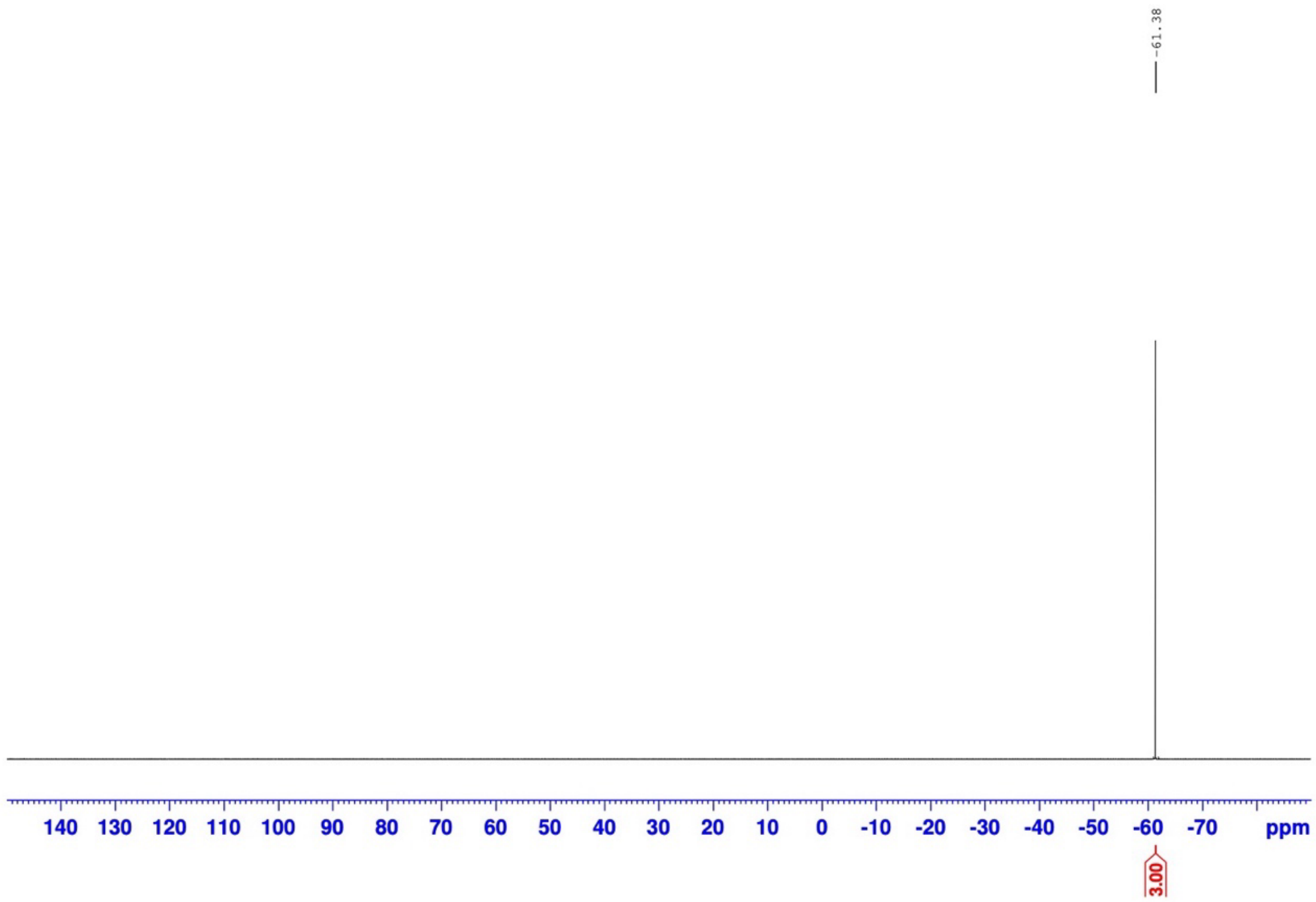

Figure S93. ^{19}F NMR spectrum of 4-(trifluoromethyl)benzohydrazide (**S-8**) in DMSO-d_6.

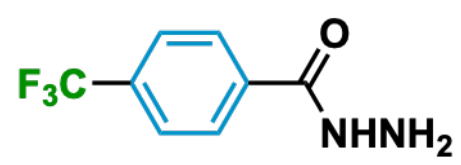

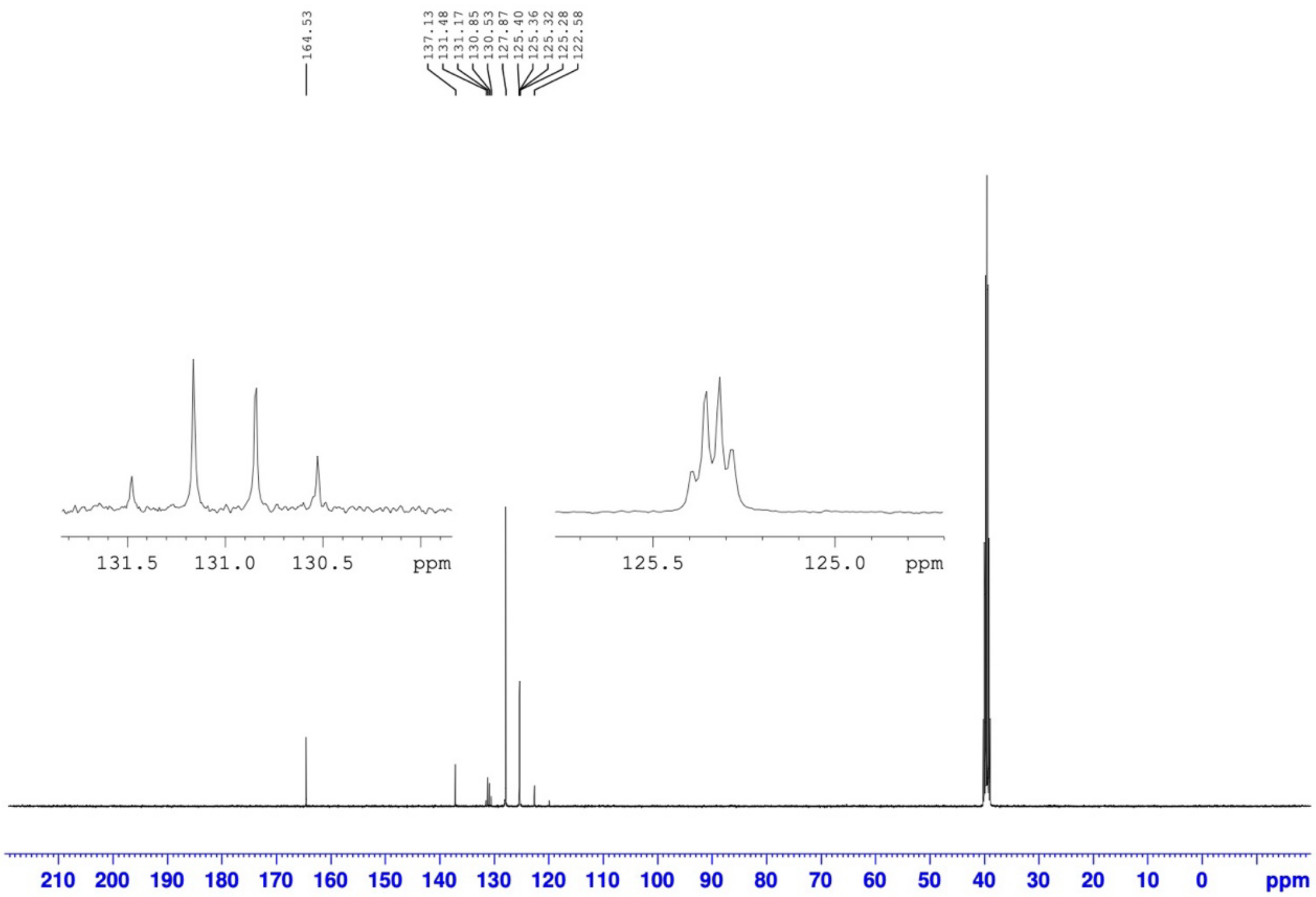

Figure S94. $^{13}C\{^{1}H\}$ NMR spectrum of 4-(trifluoromethyl)benzohydrazide (**S-8**) in DMSO-d_6.

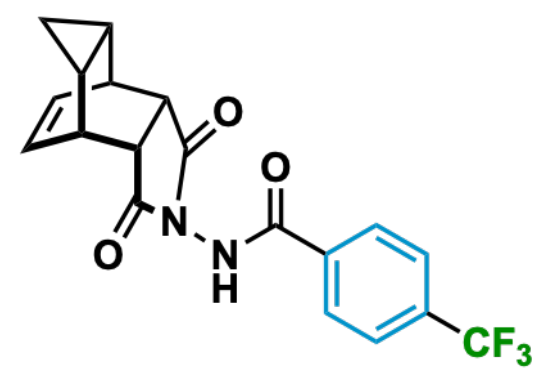

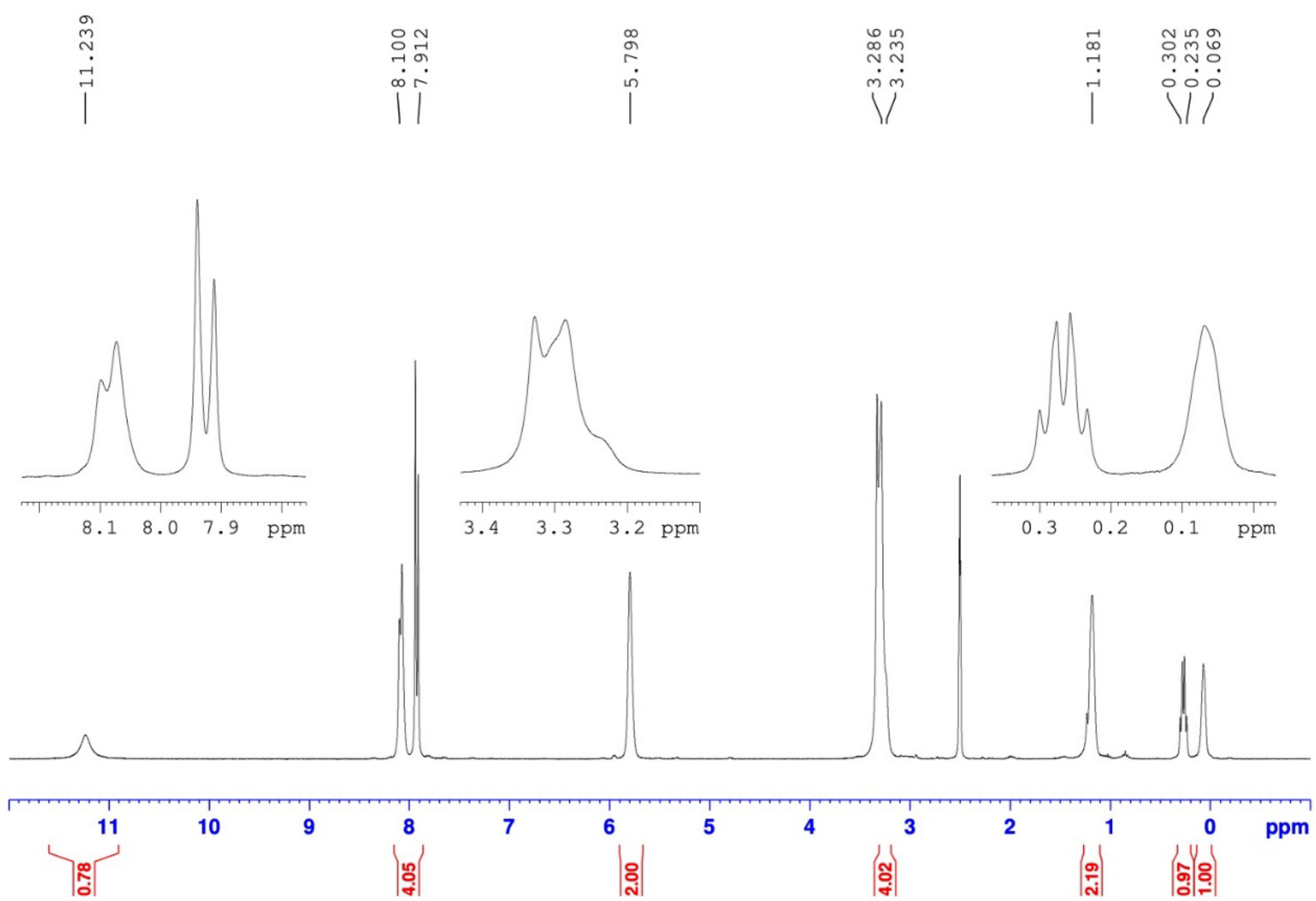

Figure S95. ^{1}H NMR spectrum of *N*-((4a*R*,5a*S*,6a*S*)-1,3-dioxo-3,3a,4,4a,5,5a,6,6a-octahydro-4,6-ethenocyclopropa[*f*]isoindol-2(1*H*)-yl)-4-(trifluoromethyl)benzamide (**17**) in DMSO-d_6.

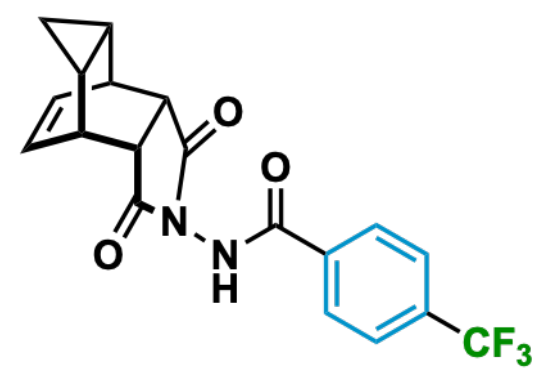

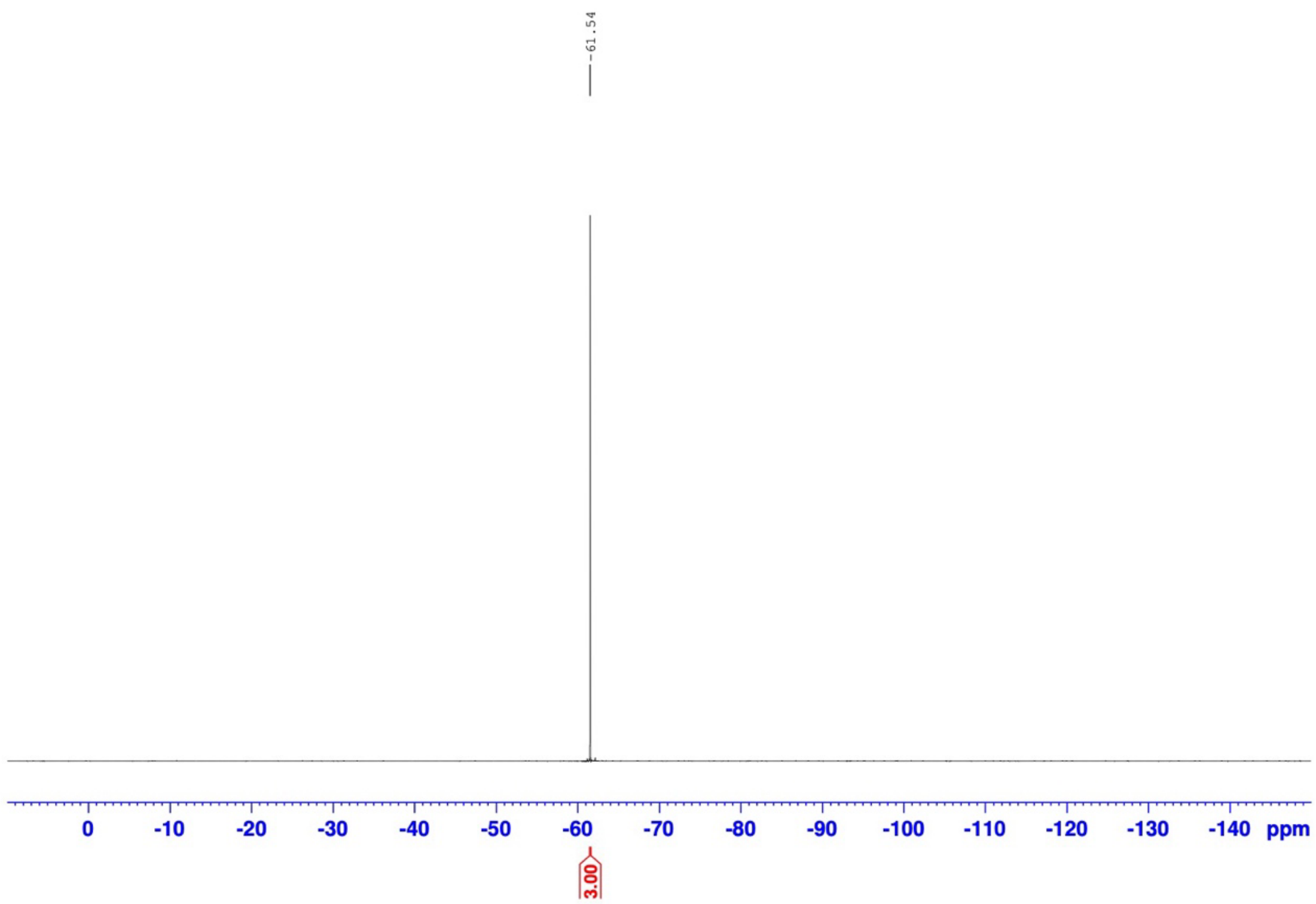

Figure S96. ^{19}F NMR spectrum of *N*-((4a*R*,5a*S*,6a*S*)-1,3-dioxo-3,3a,4,4a,5,5a,6,6a-octahydro-4,6-ethenocyclopropa[*f*]isoindol-2(1*H*)-yl)-4-(trifluoromethyl)benzamide (**17**) in DMSO-*d*$_6$.

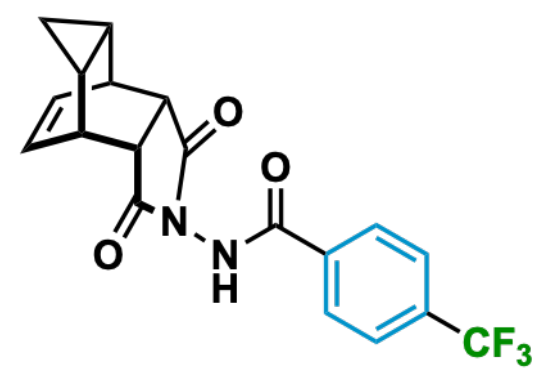

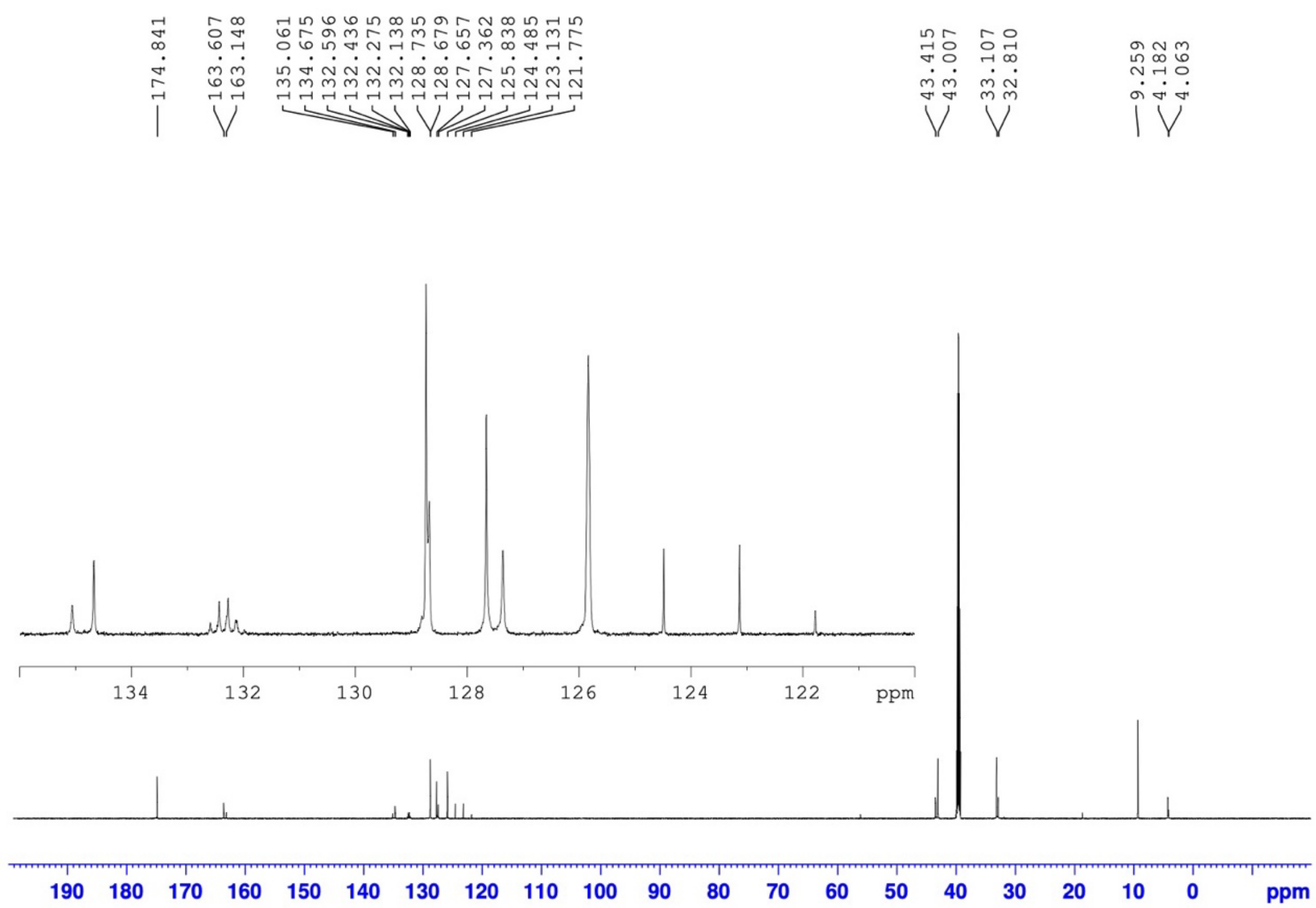

Figure S97. $^{13}C\{^{1}H\}$ NMR *N*-((4a*R*,5a*S*,6a*S*)-1,3-dioxo-3,3a,4,4a,5,5a,6,6a-octahydro-4,6 ethenocyclopropa[*f*]isoindol-2(1*H*)-yl)-4-(trifluoromethyl)benzamide (**17**) in DMSO-d_6.

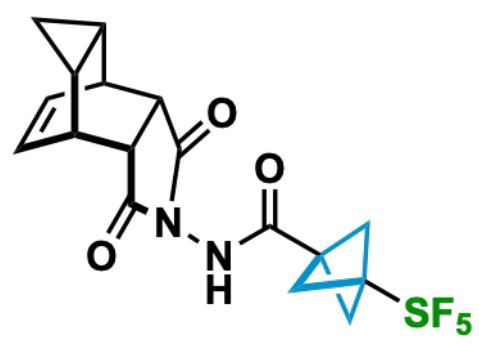

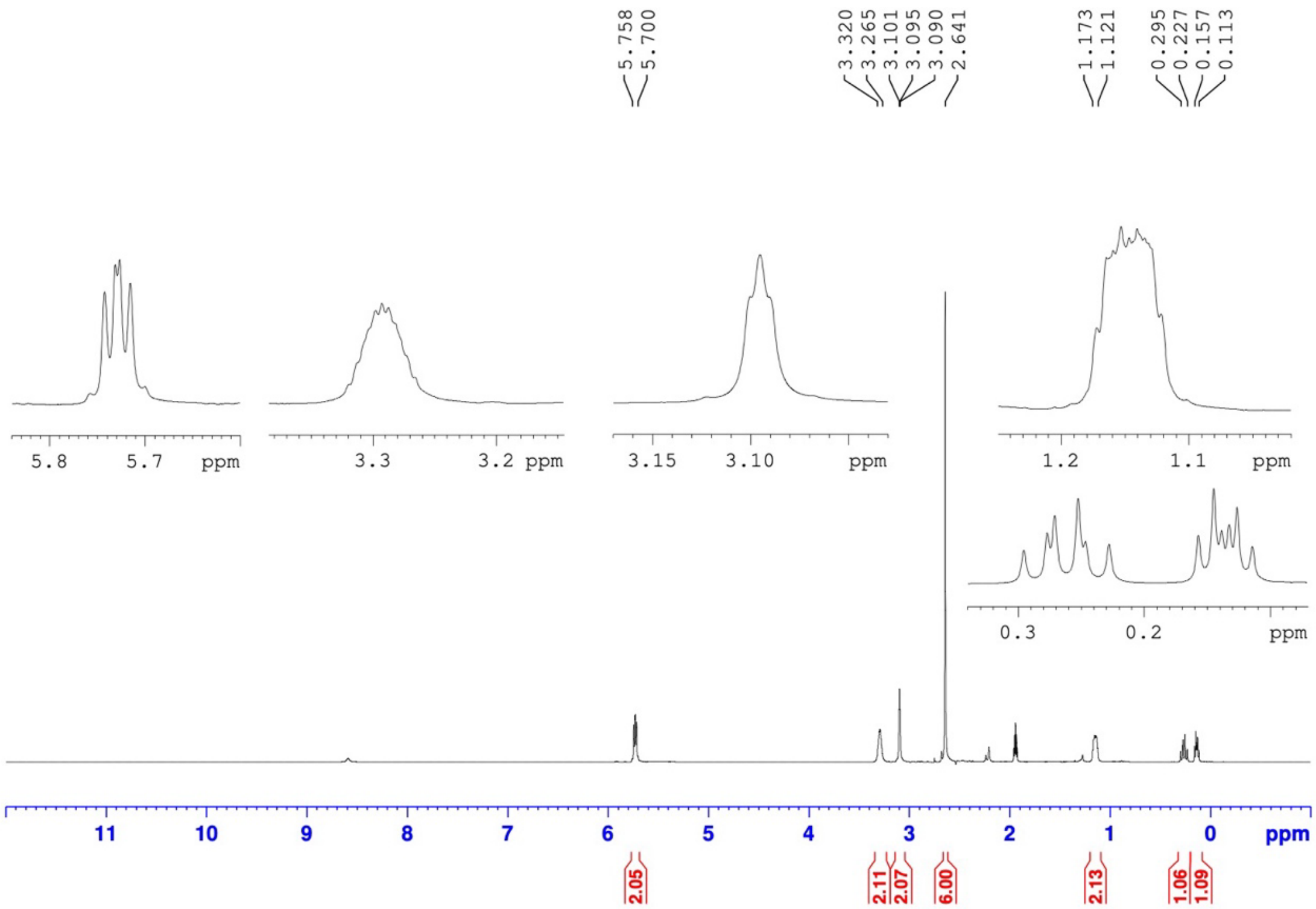

Figure S98. ^{1}H NMR spectrum of *N*-((4*R*,4a*R*,5aS,6S,6aS)-1,3-dioxo-3,3a,4,4a,5,5a,6,6a-octahydro-4,6-ethenocyclopropa[*f*]isoindol-2(1*H*)-yl)-3-(pentafluoro-λ^6-sulfanyl)bicyclo[1.1.1]pentane-1-carboxamide (**20**) in MeCN-d_3.

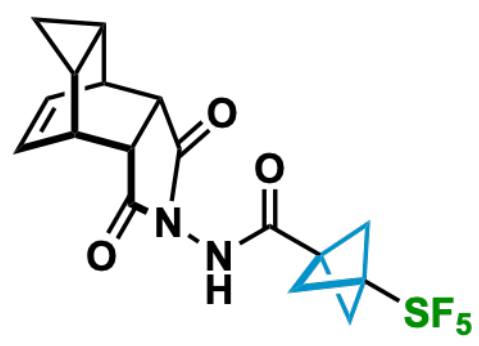

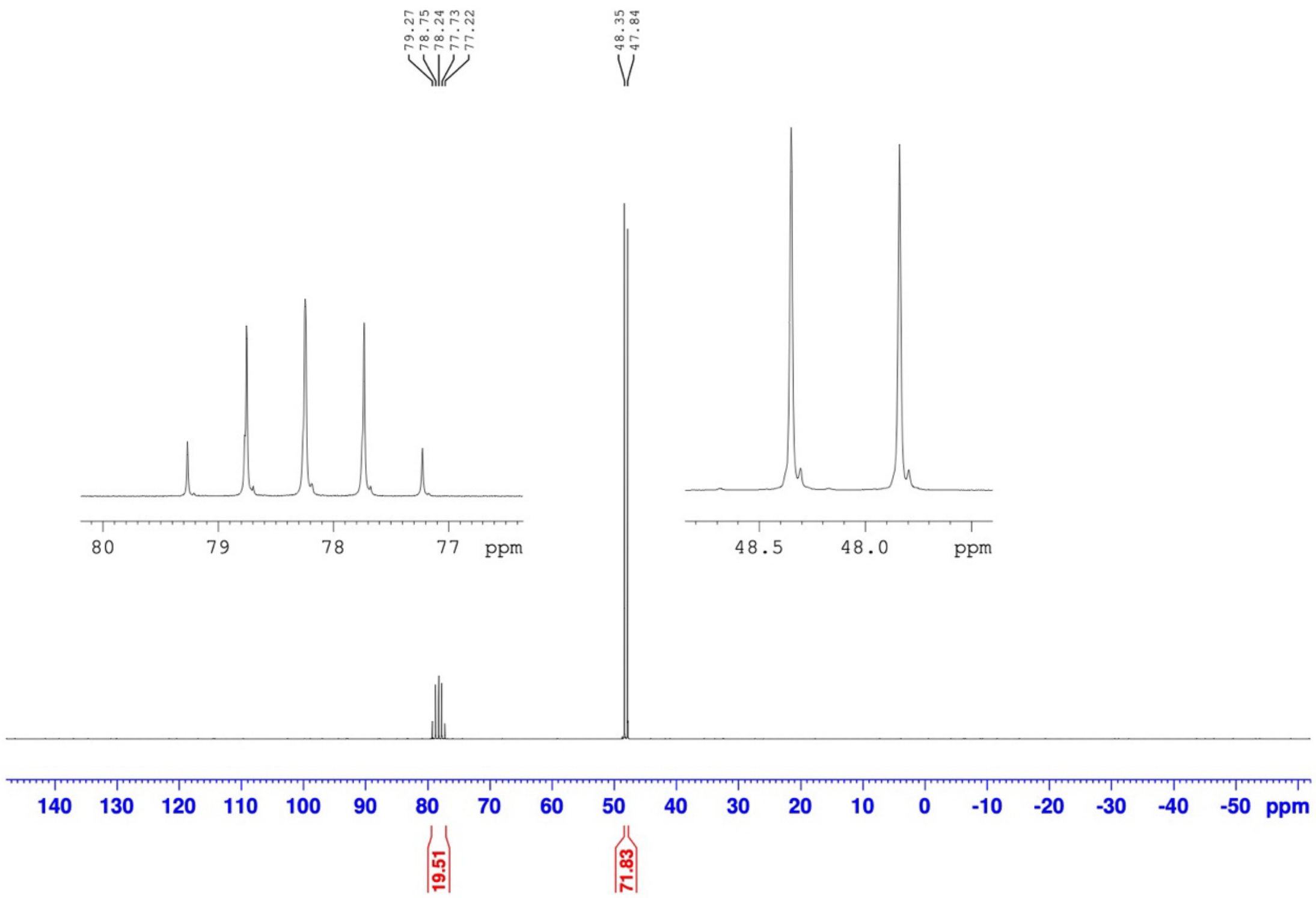

Figure S99. ^{19}F NMR spectrum of *N*-((4*R*,4a*R*,5aS,6S,6aS)-1,3-dioxo-3,3a,4,4a,5,5a,6,6a-octahydro-4,6-ethenocyclopropa[*f*]isoindol-2(1*H*)-yl)-3-(pentafluoro-λ^6-sulfanyl)bicyclo[1.1.1]pentane-1-carboxamide (**20**) in MeCN-d_3.

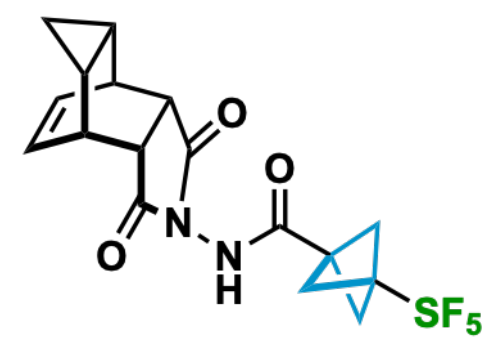

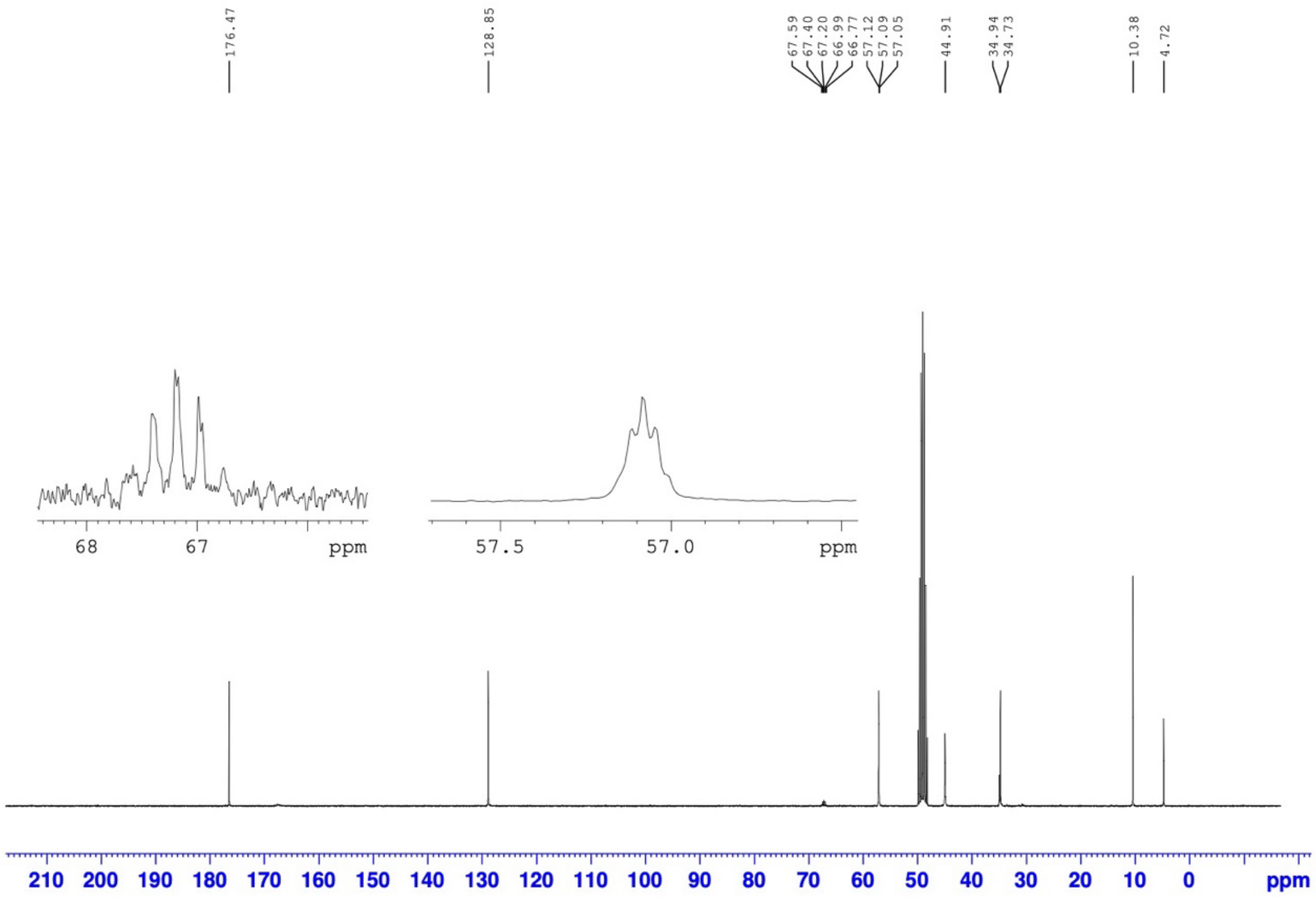

Figure S100. $^{13}C\{^1H\}$ NMR spectrum of *N*-((4*R*,4a*R*,5aS,6S,6aS)-1,3-dioxo-3,3a,4,4a,5,5a,6,6a-octahydro-4,6-ethenocyclopropa[*f*]isoindol-2(1*H*)-yl)-3-(pentafluoro-λ^6-sulfanyl)bicyclo[1.1.1]pentane-1-carboxamide (**20**) in MeOD-*d*$_4$.

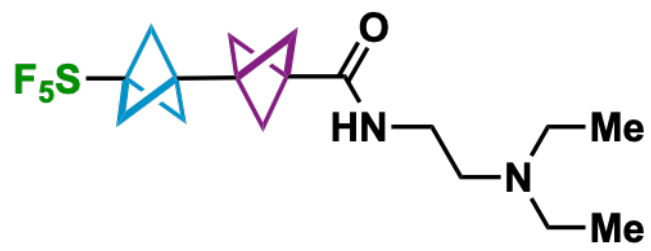

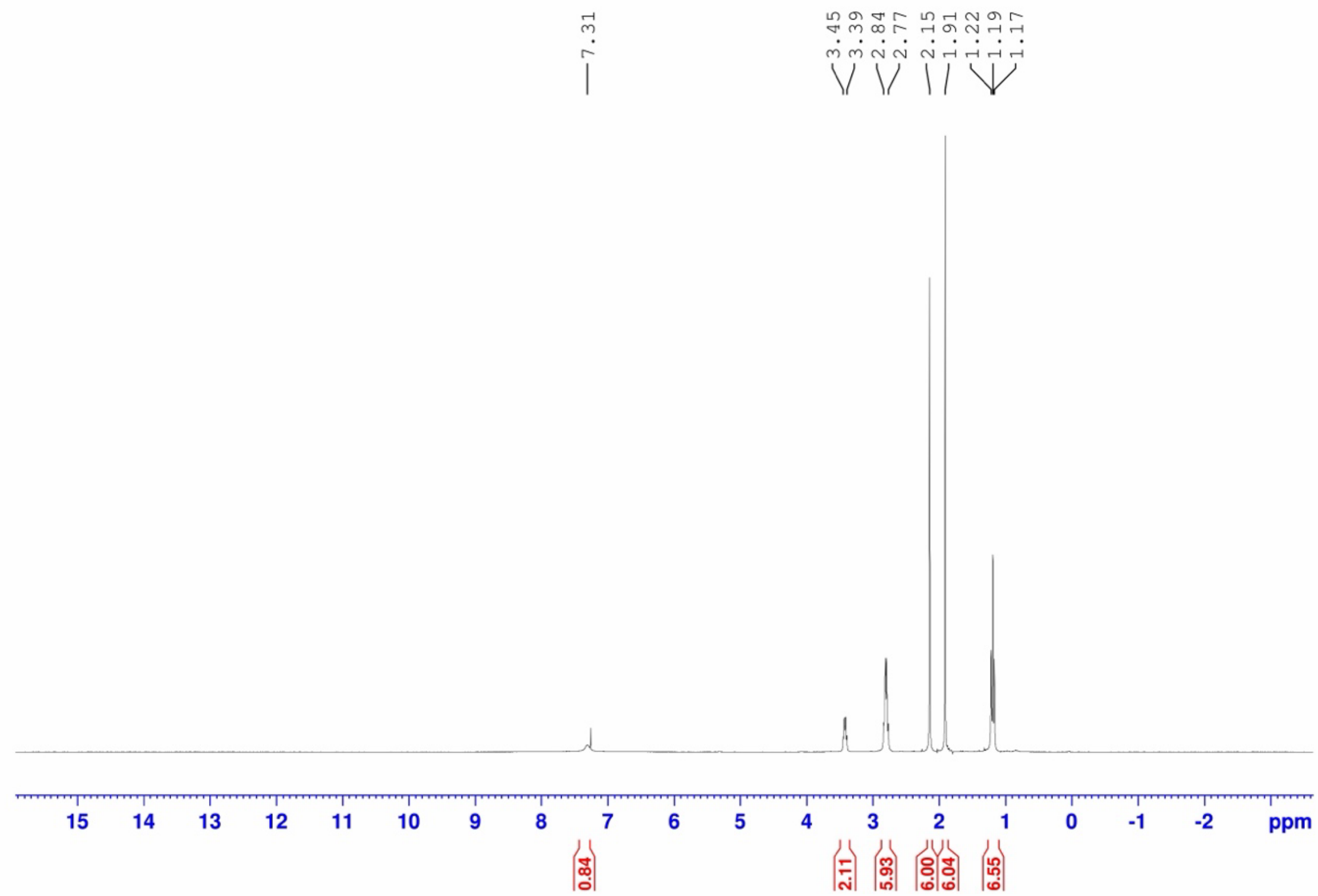

Figure S101. ^{1}H NMR spectrum of *N*-(2-(diethylamino)ethyl)-3'-(pentafluoro-λ^6-sulfanyl)-[1,1'-bi(bicyclo[1.1.1]pentane)]-3-carboxamide (**S-9**) in $CDCl_3$.

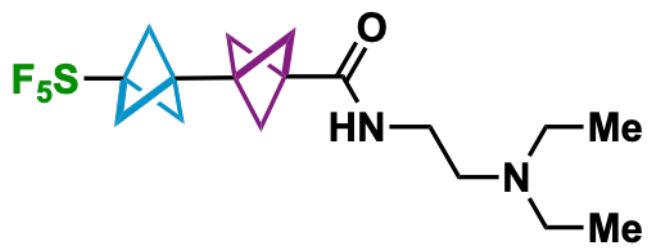

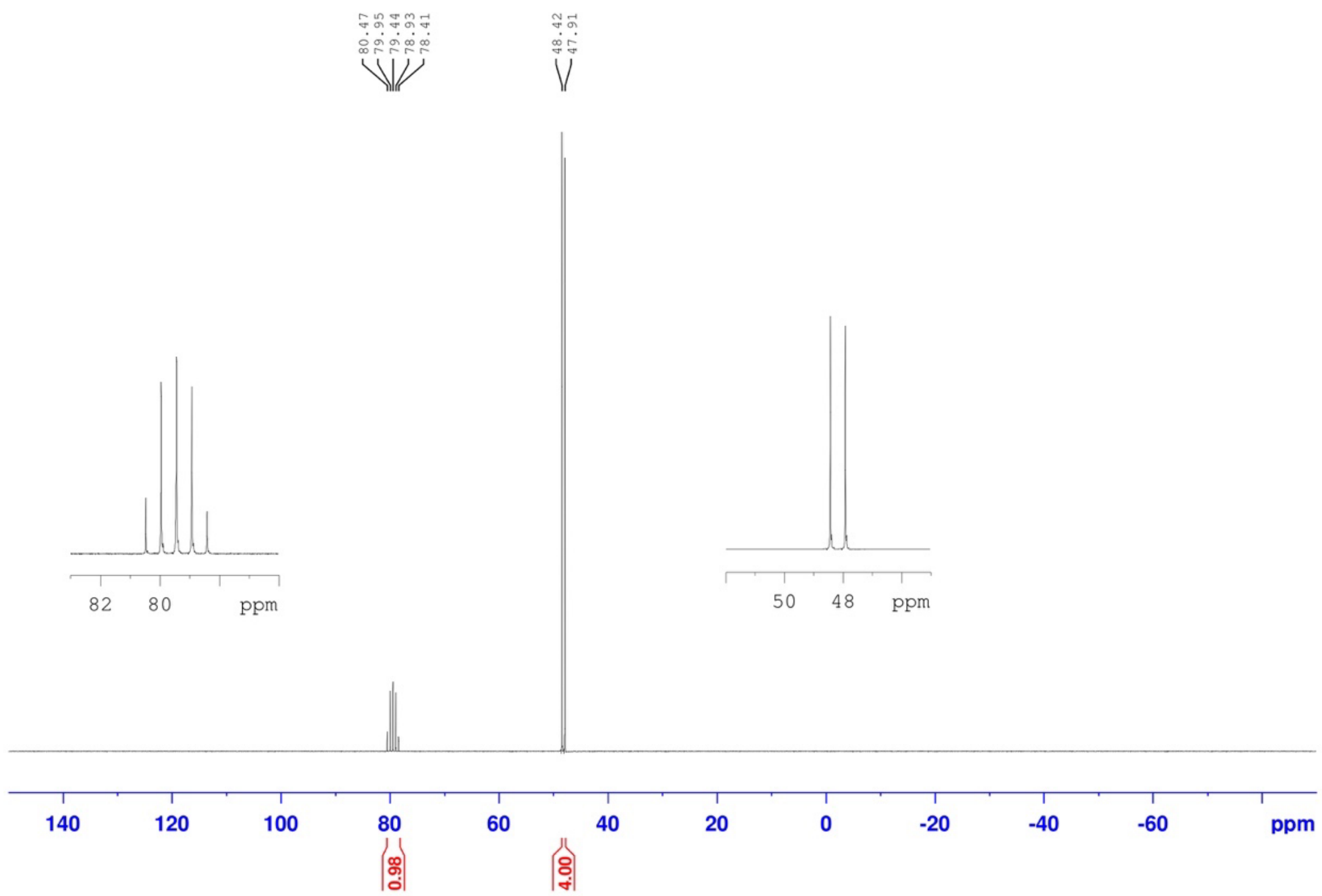

Figure S102. ^{19}F NMR spectrum of *N*-(2-(diethylamino)ethyl)-3'-(pentafluoro-λ^6-sulfanyl)-[1,1'-bi(bicyclo[1.1.1]pentane)]-3-carboxamide (**S-9**) in $CDCl_3$.

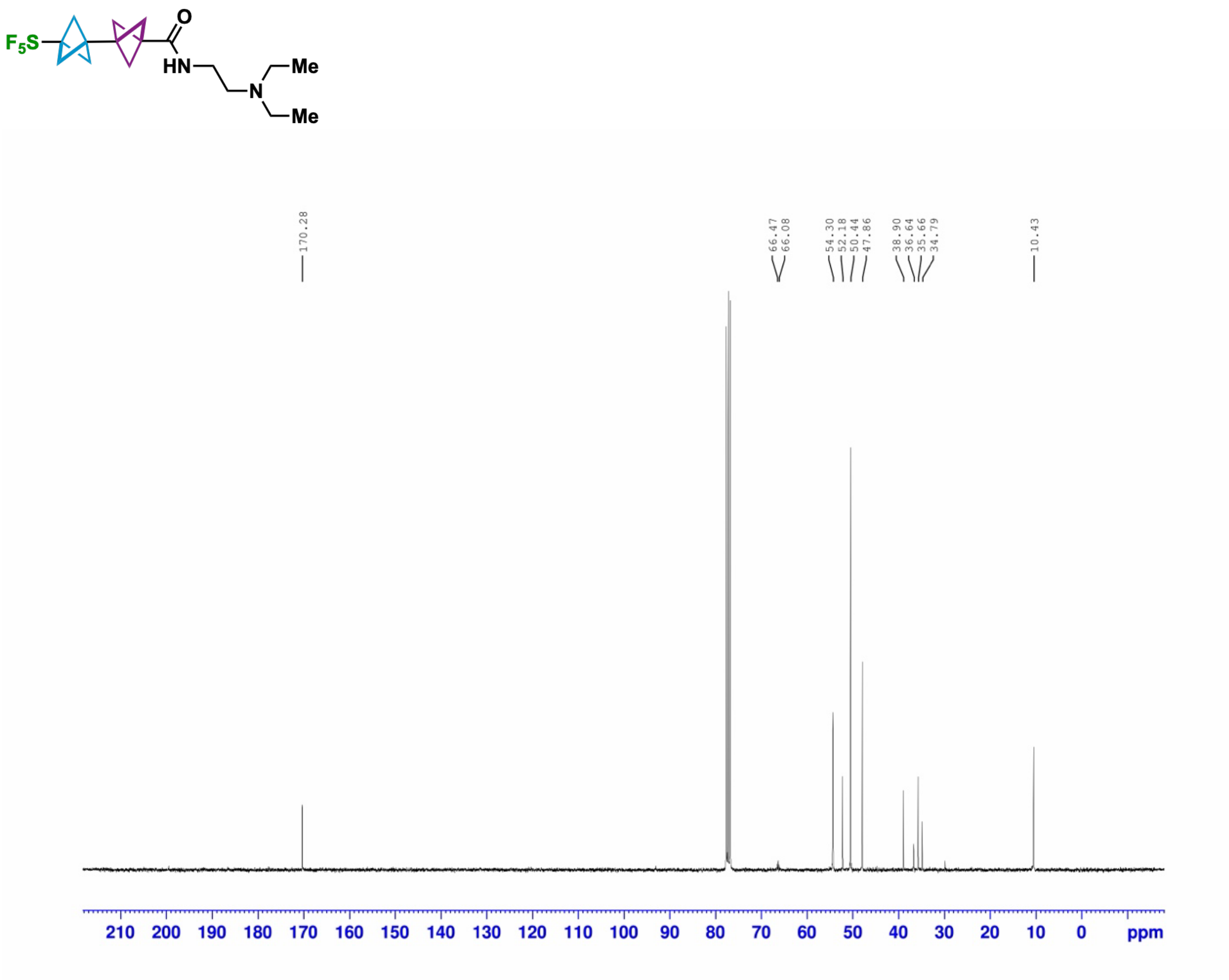

Figure S103. $^{13}C\{^{1}H\}$ NMR spectrum of *N*-(2-(diethylamino)ethyl)-3'-(pentafluoro-λ^6-sulfanyl)-[1,1'-bi(bicyclo[1.1.1]pentane)]-3-carboxamide (**S-9**) in $CDCl_3$.

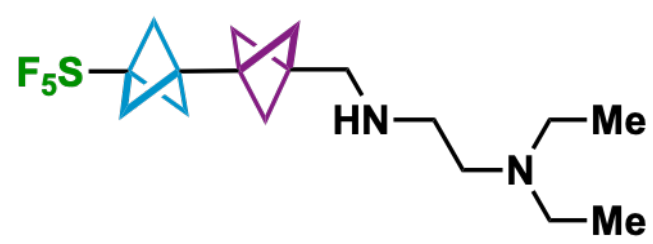

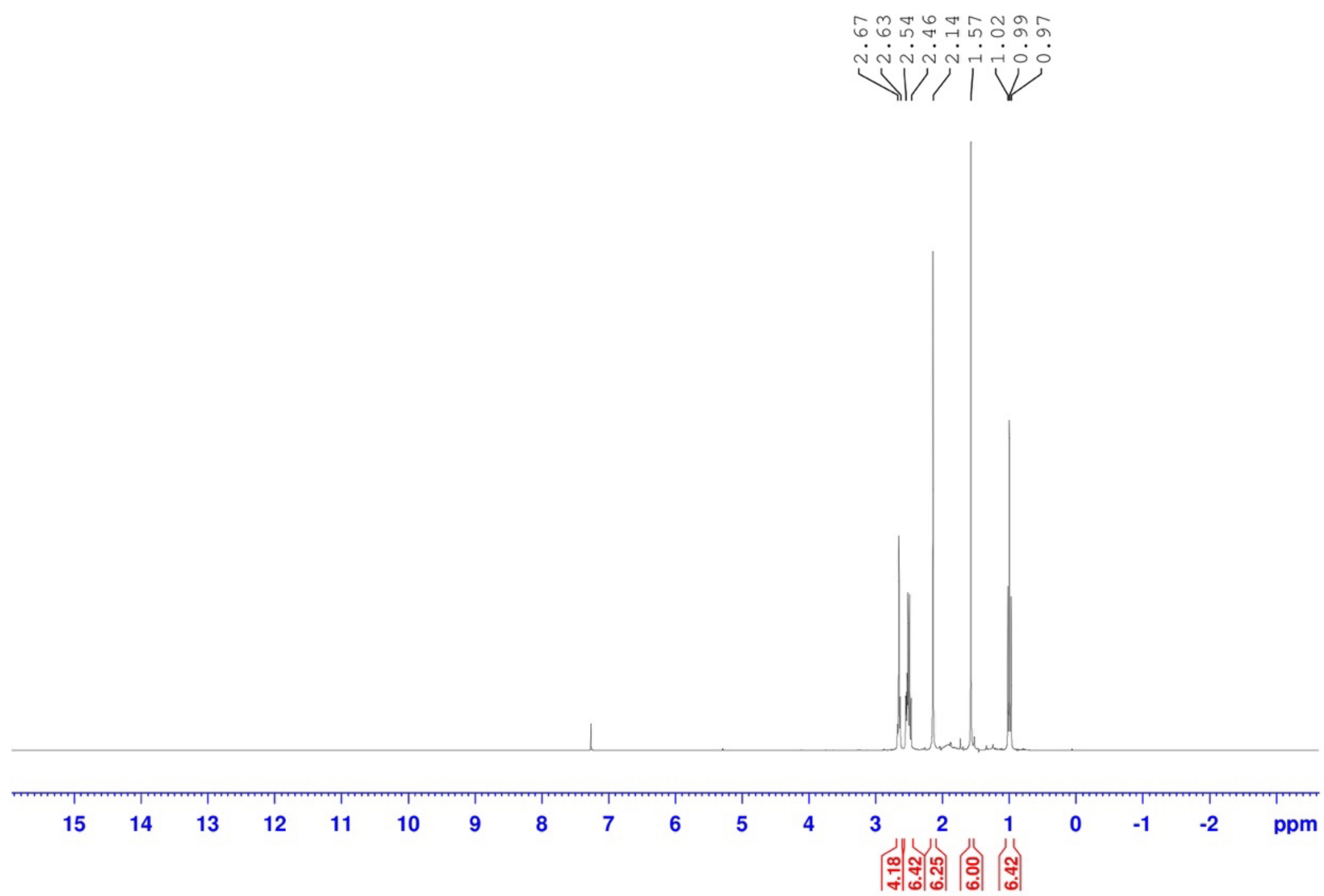

Figure S104. ^{1}H NMR spectrum of N^1,N^1-diethyl-N^2-((3'-(pentafluoro-λ^6-sulfanyl)-[1,1'-bi(bicyclo[1.1.1]pentan)]-3-yl)methyl)ethane-1,2-diamine (**S-10**) in $CDCl_3$.

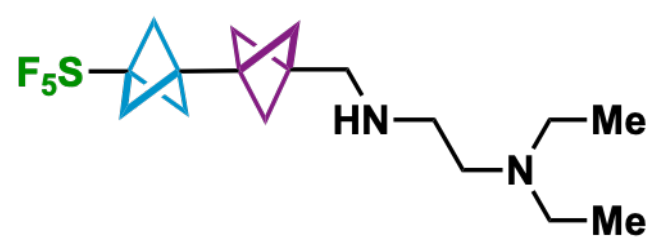

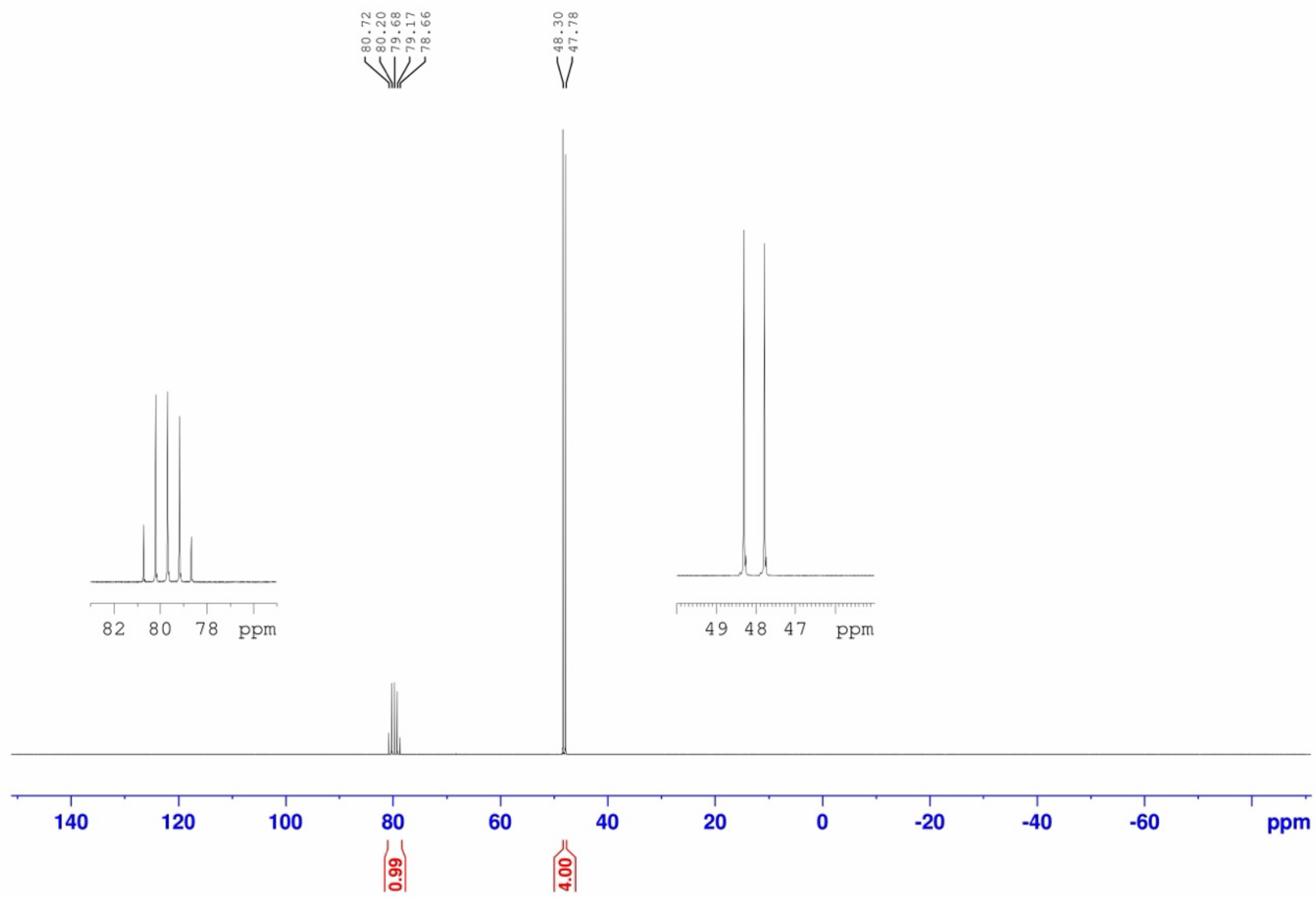

Figure S105. ^{19}F NMR spectrum of *N*[1],*N*[1]-diethyl-*N*[2]-((3'-(pentafluoro-λ[6]-sulfanyl)-[1,1'-bi(bicyclo[1.1.1]pentan)]-3-yl)methyl)ethane-1,2-diamine (**S-10**) in $CDCl_3$.

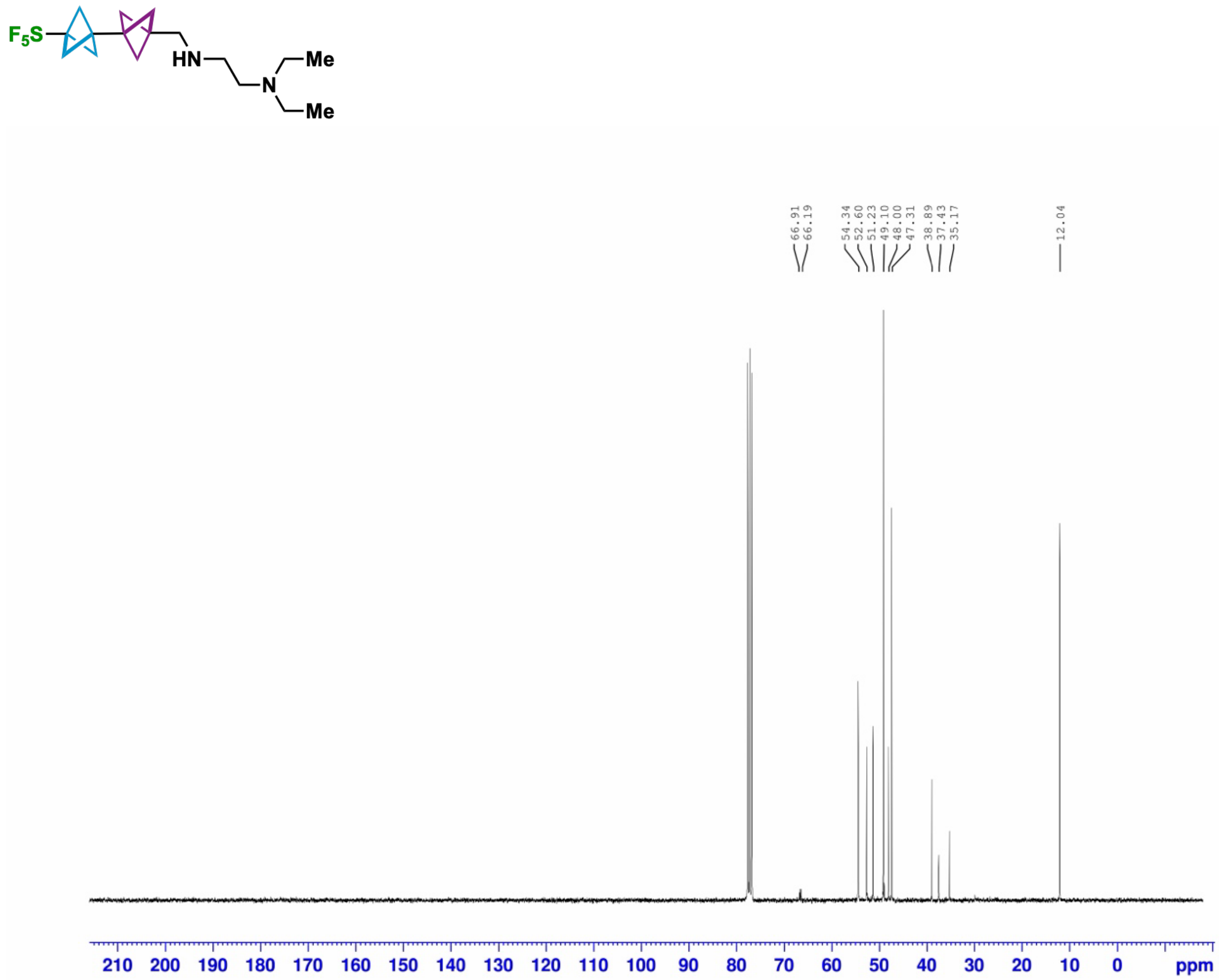

Figure S106. $^{13}C\{^{1}H\}$ NMR spectrum of N^1,N^1-diethyl-N^2-((3'-(pentafluoro-λ^6-sulfanyl)-[1,1'-bi(bicyclo[1.1.1]pentan)]-3-yl)methyl)ethane-1,2-diamine (**S-10**) in $CDCl_3$.

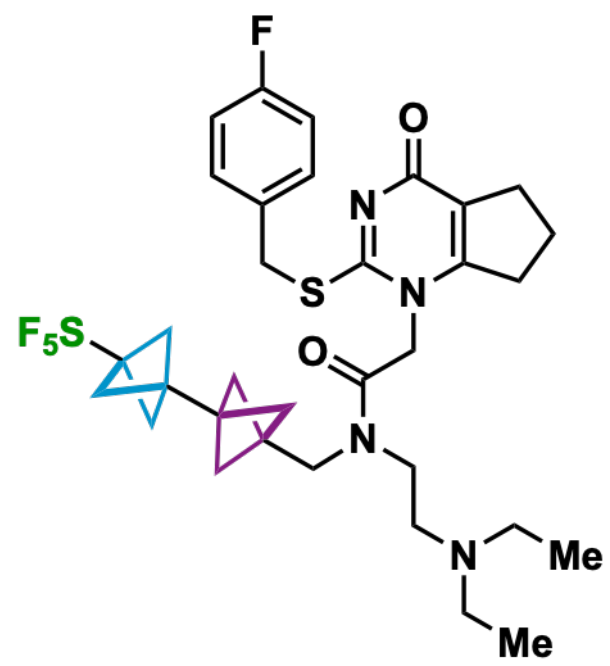

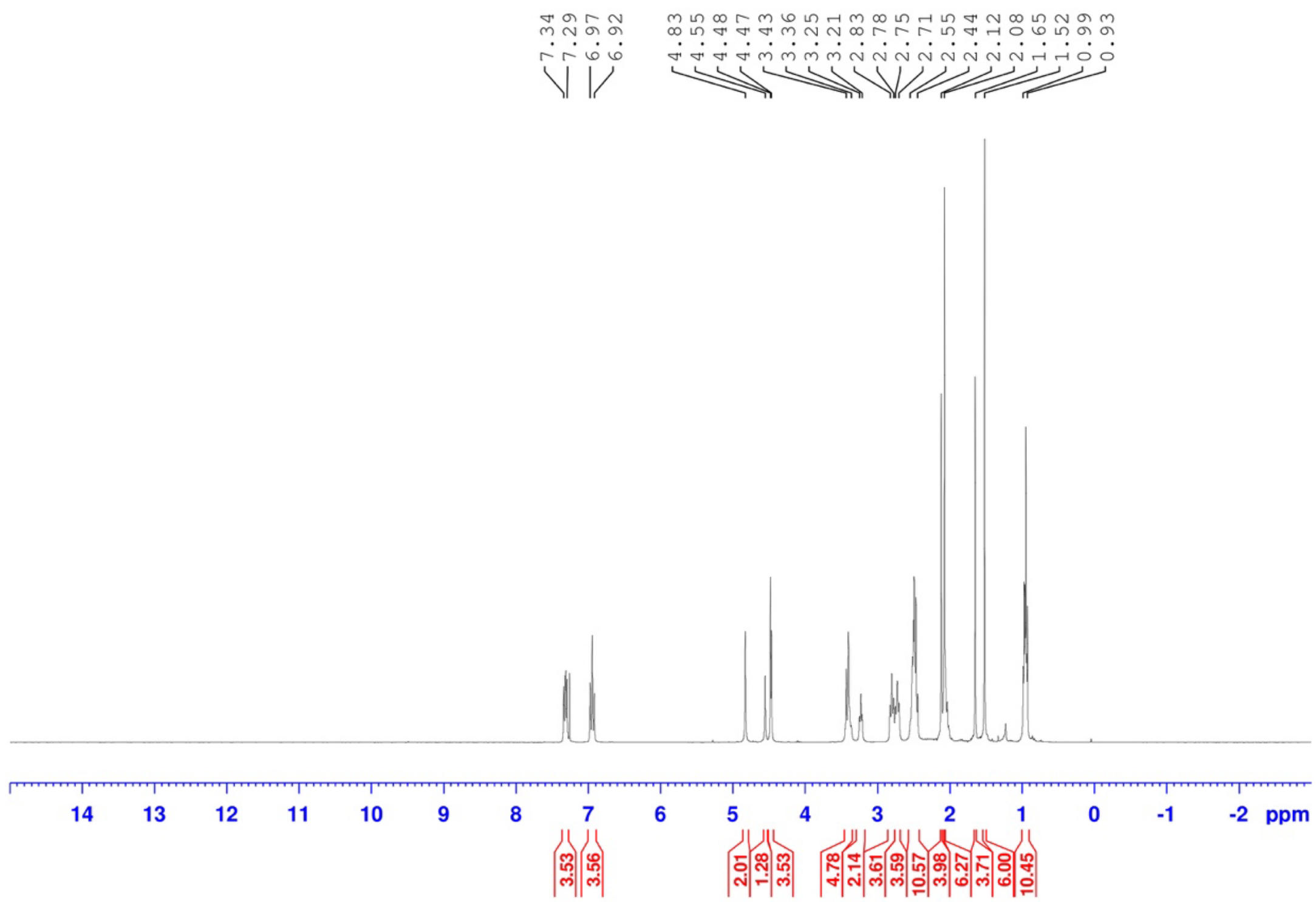

Figure S107. ^{1}H NMR spectrum of *N*-(2-(diethylamino)ethyl)-2-(2-((4-fluorobenzyl)thio)-4-oxo-4,5,6,7-tetrahydro-1*H*-cyclopenta[*d*]pyrimidin-1-yl)-*N*-((3'-(pentafluoro-λ^6-sulfanyl)-[1,1'-bi(bicyclo[1.1.1]pentan)]-3-yl)methyl)acetamide (**30**) in $CDCl_3$.

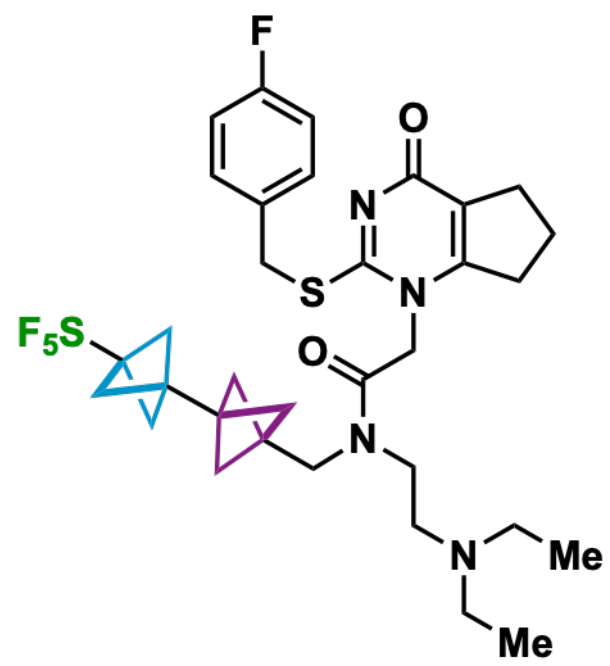

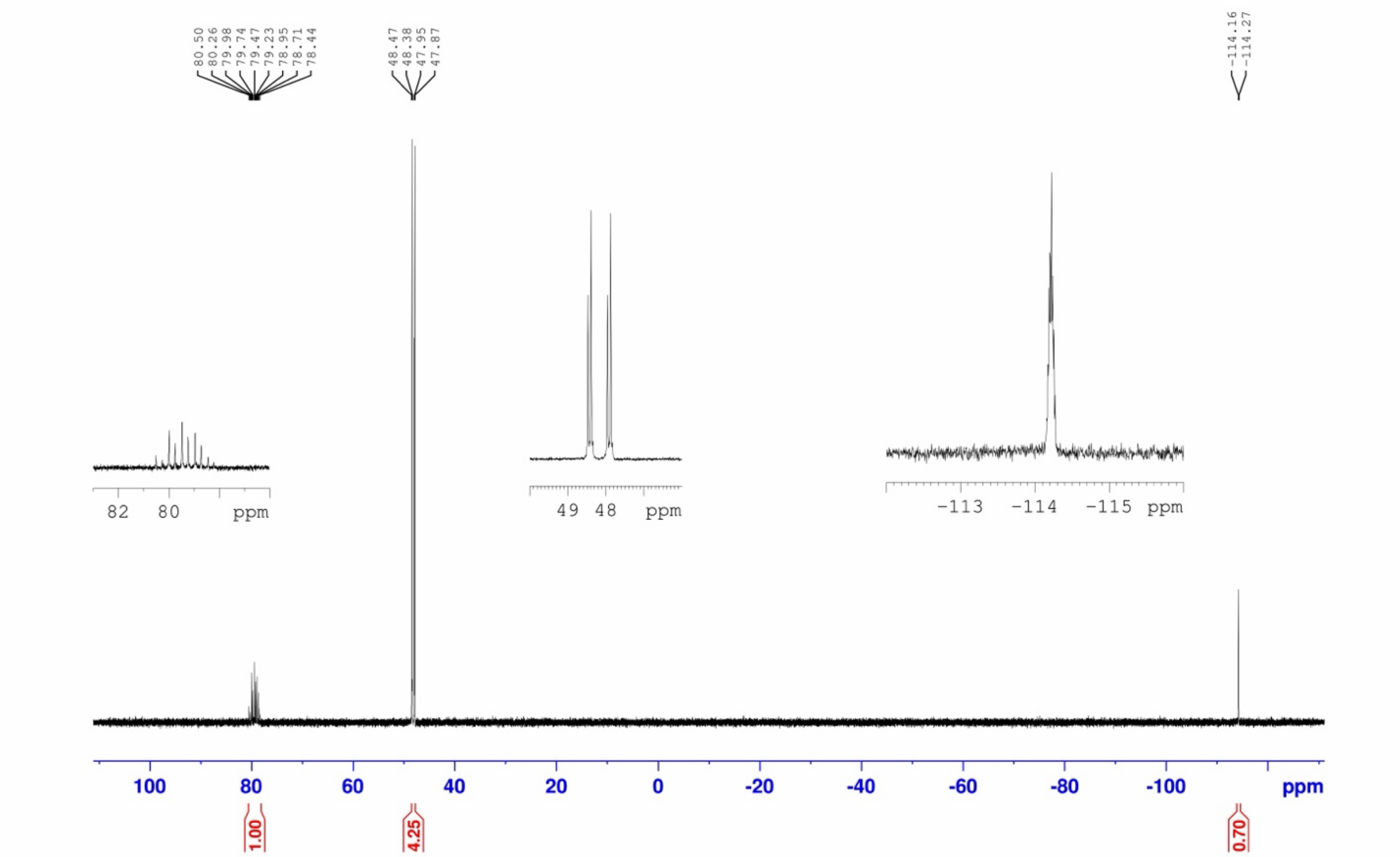

Figure S108. ^{19}F NMR spectrum of *N*-(2-(diethylamino)ethyl)-2-(2-((4-fluorobenzyl)thio)-4-oxo-4,5,6,7-tetrahydro-1*H*-cyclopenta[*d*]pyrimidin-1-yl)-*N*-((3'-(pentafluoro-λ^6-sulfanyl)-[1,1'-bi(bicyclo[1.1.1]pentan)]-3-yl)methyl)acetamide (**30**) in $CDCl_3$.

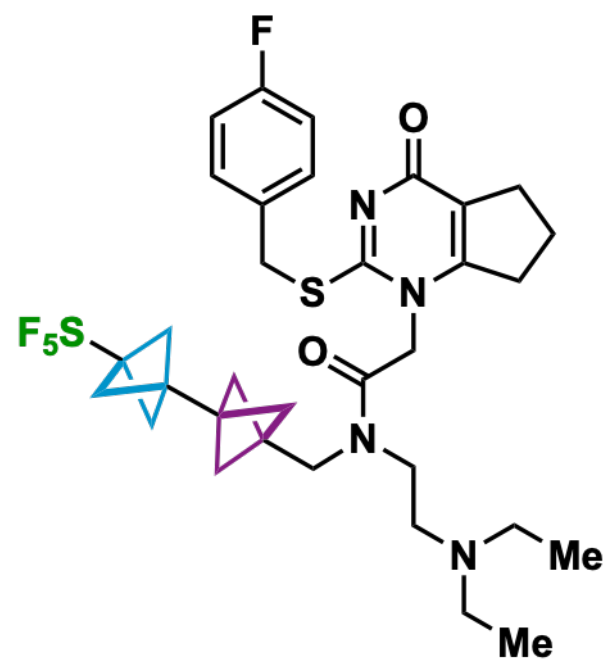

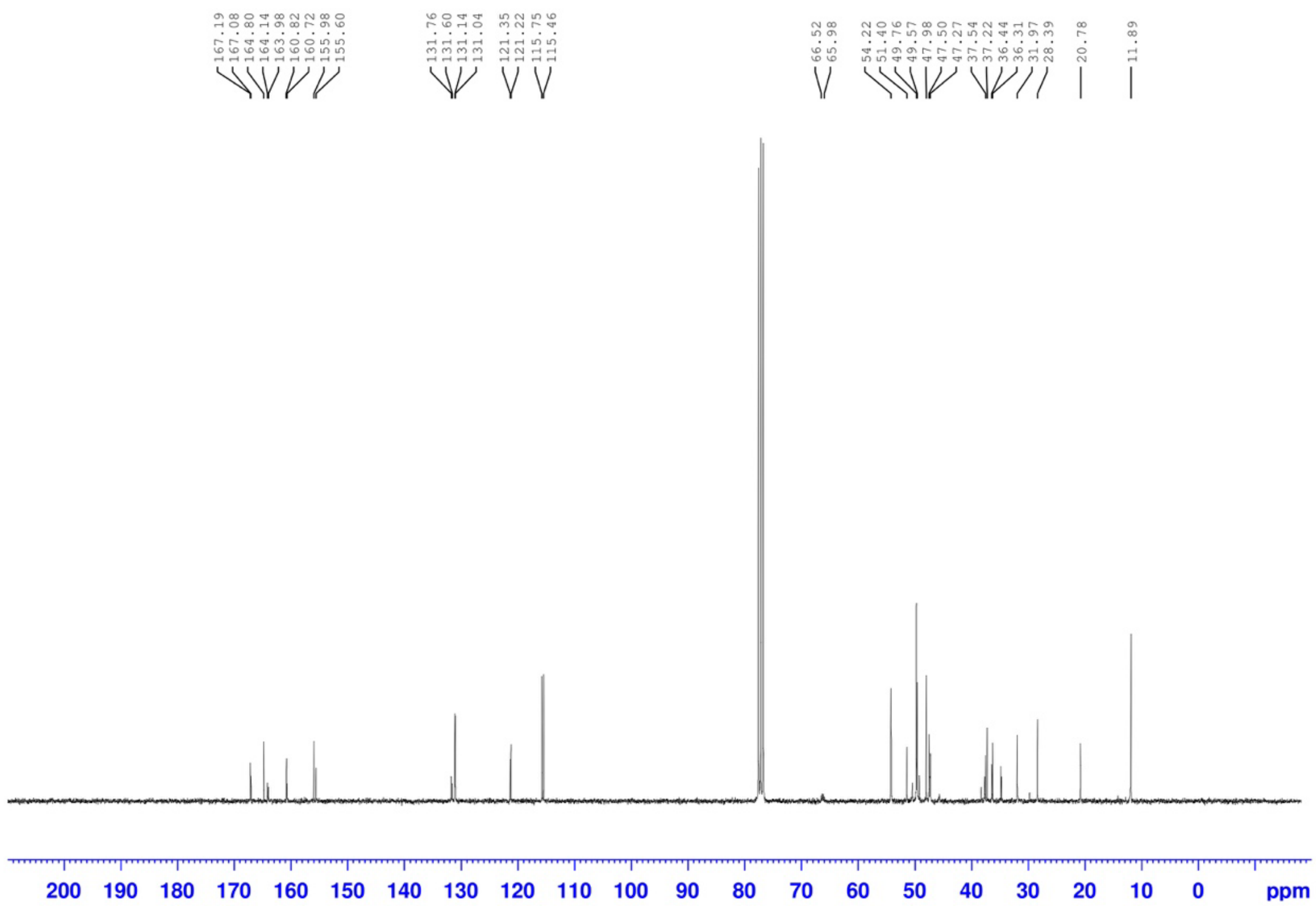

Figure S109. $^{13}C\{^{1}H\}$ NMR spectrum of *N*-(2-(diethylamino)ethyl)-2-(2-((4-fluorobenzyl)thio)-4-oxo-4,5,6,7-tetrahydro-1*H*-cyclopenta[*d*]pyrimidin-1-yl)-*N*-((3'-(pentafluoro-λ^6-sulfanyl)-[1,1'-bi(bicyclo[1.1.1]pentan)]-3-yl)methyl)acetamide (**34**) in $CDCl_3$.

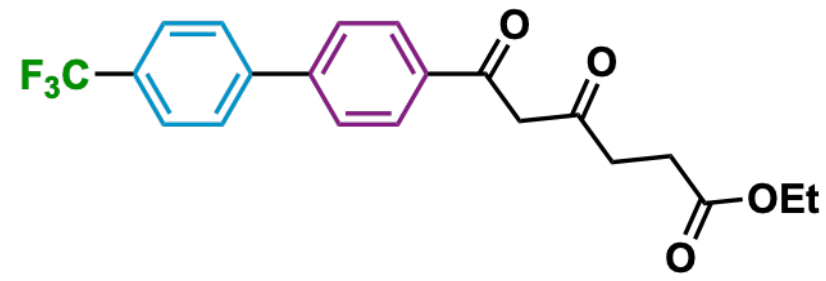

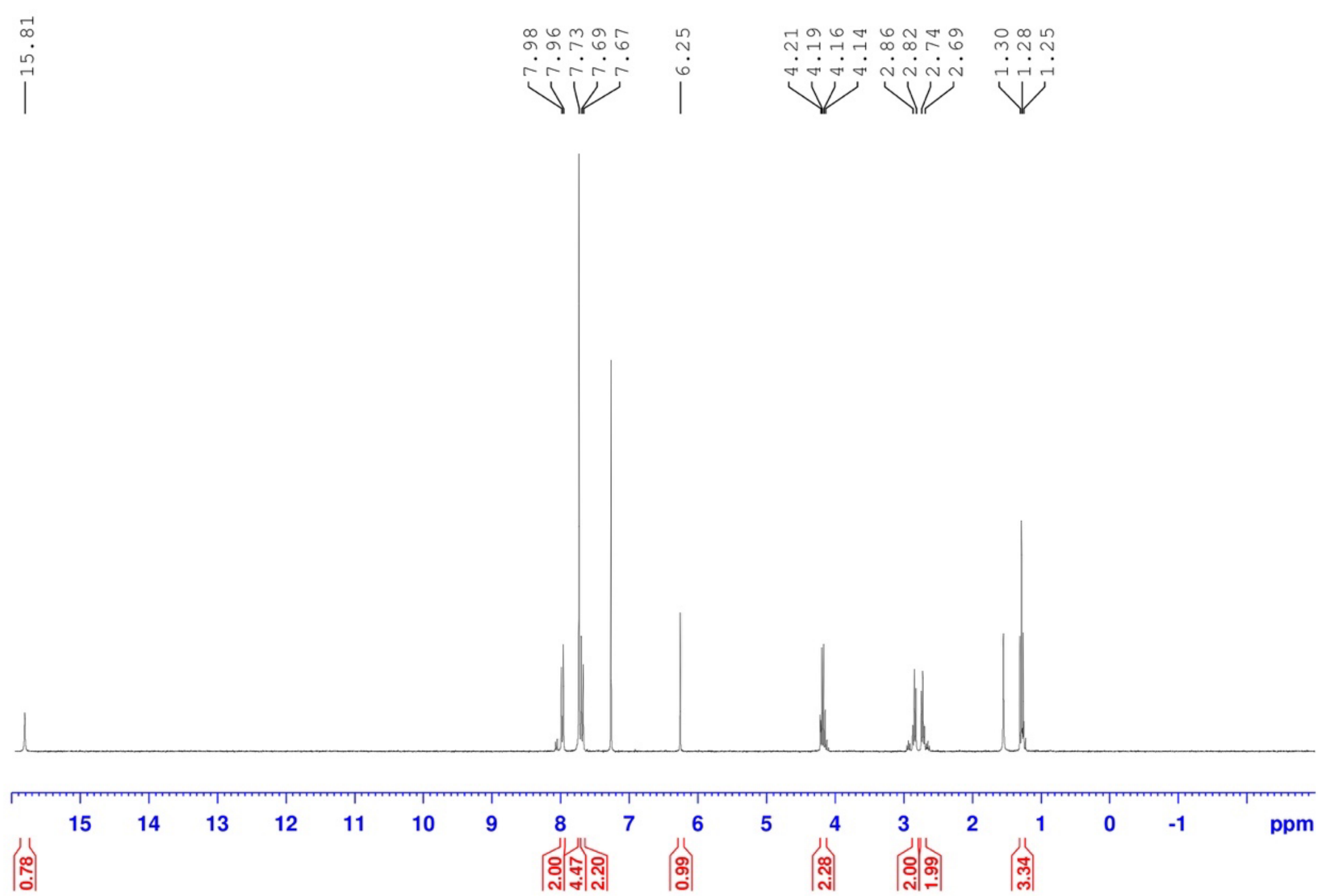

Figure S110. ^{1}H NMR spectrum of ethyl 4,6-dioxo-6-(4'-(trifluoromethyl)-[1,1'-biphenyl]-4-yl)hexanoate (**S-11**) in $CDCl_3$.

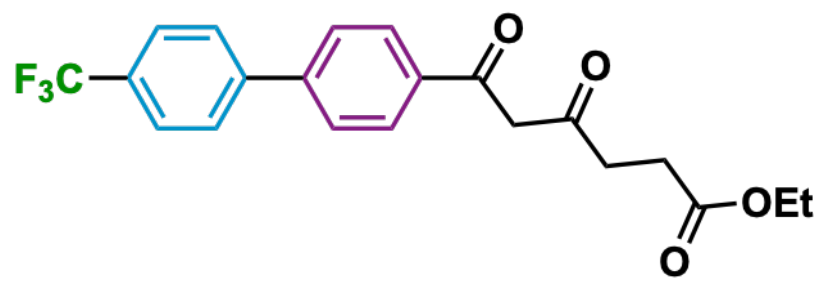

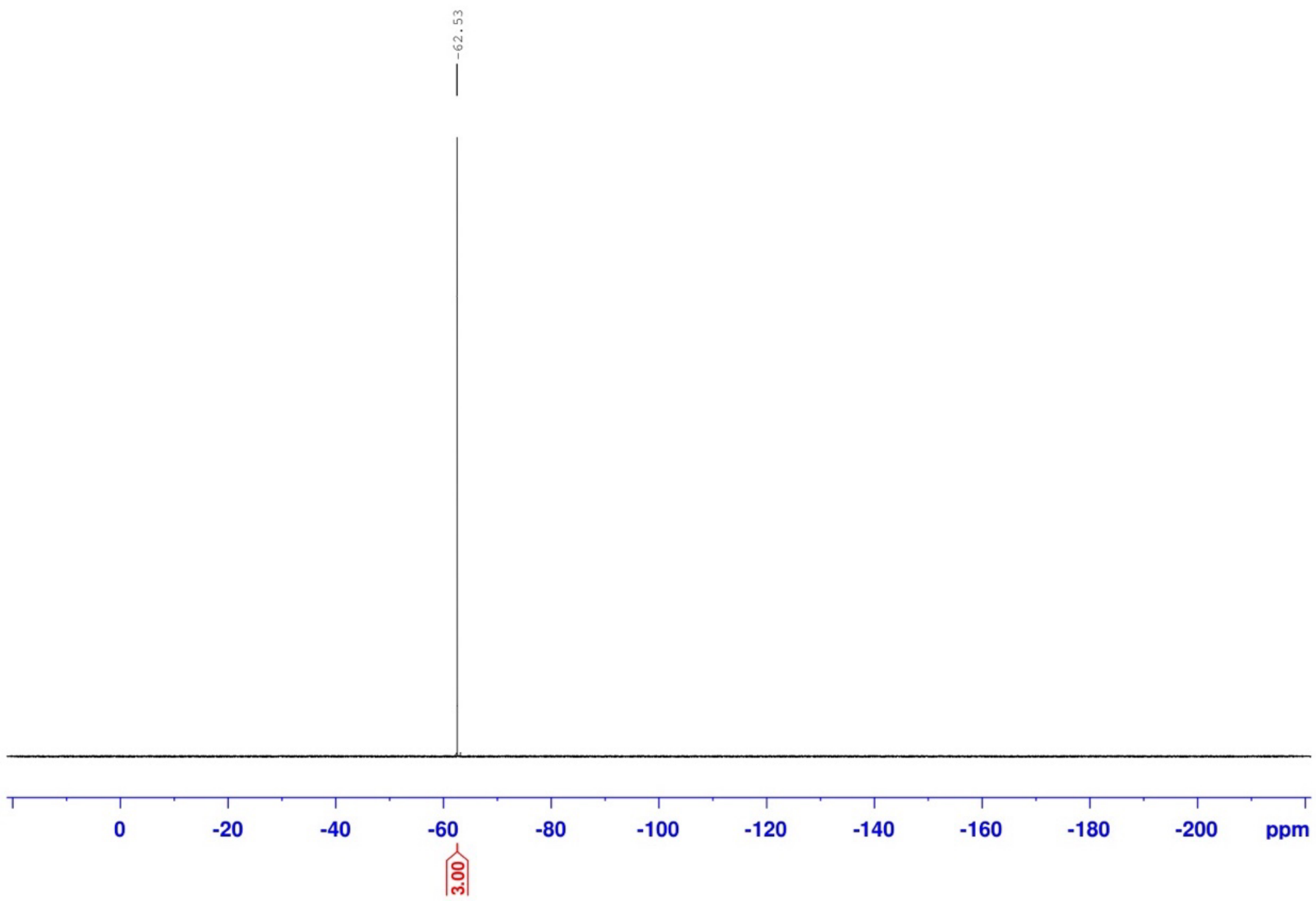

Figure S111. ^{19}F NMR spectrum of ethyl 4,6-dioxo-6-(4'-(trifluoromethyl)-[1,1'-biphenyl]-4-yl)hexanoate (**S-11**) in $CDCl_3$.

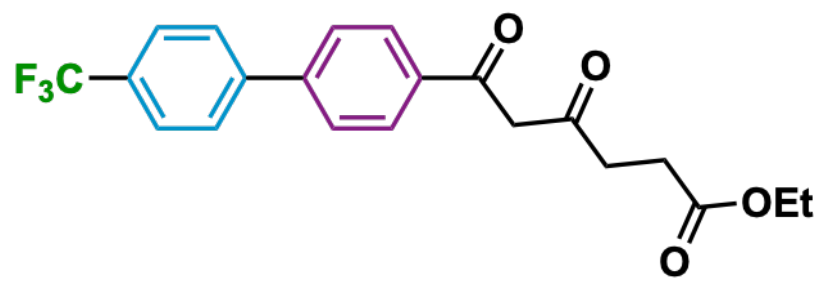

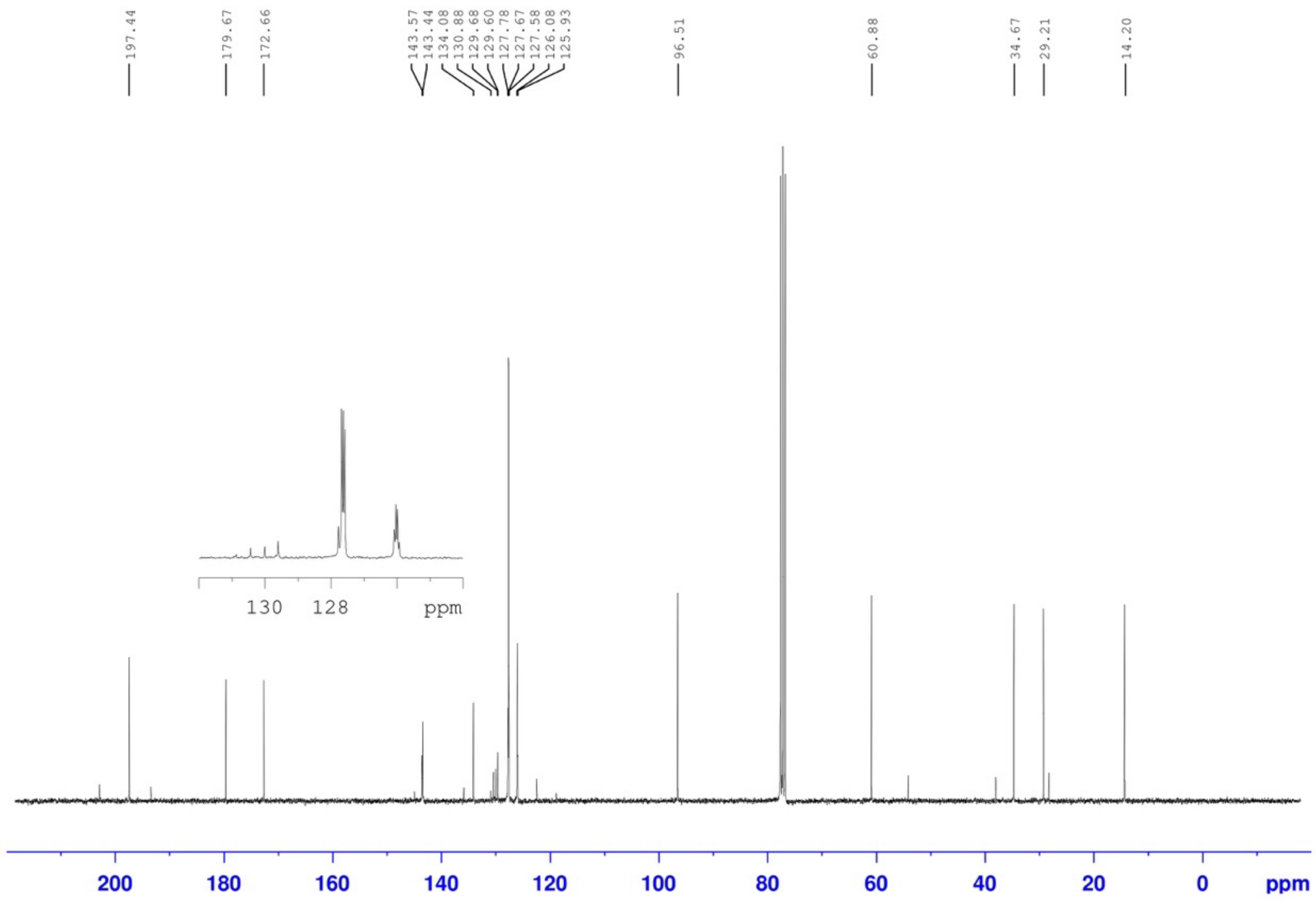

Figure S112. $^{13}C\{^{1}H\}$ NMR spectrum of ethyl 4,6-dioxo-6-(4'-(trifluoromethyl)-[1,1'-biphenyl]-4-yl)hexanoate (**S-11**) in $CDCl_3$.

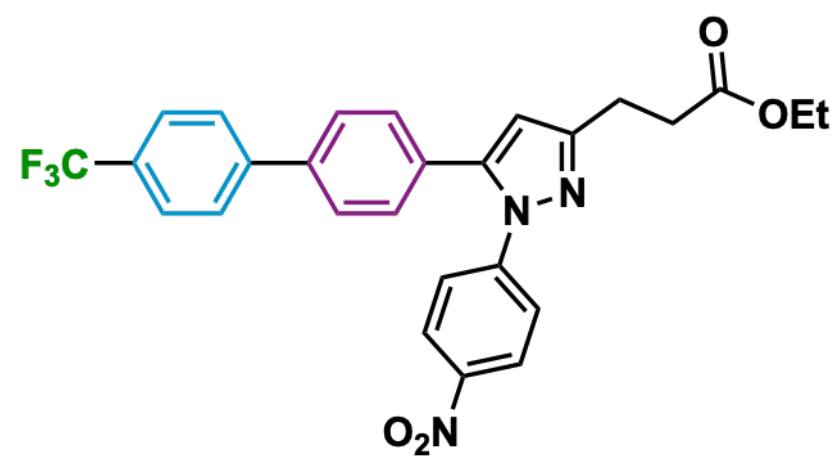

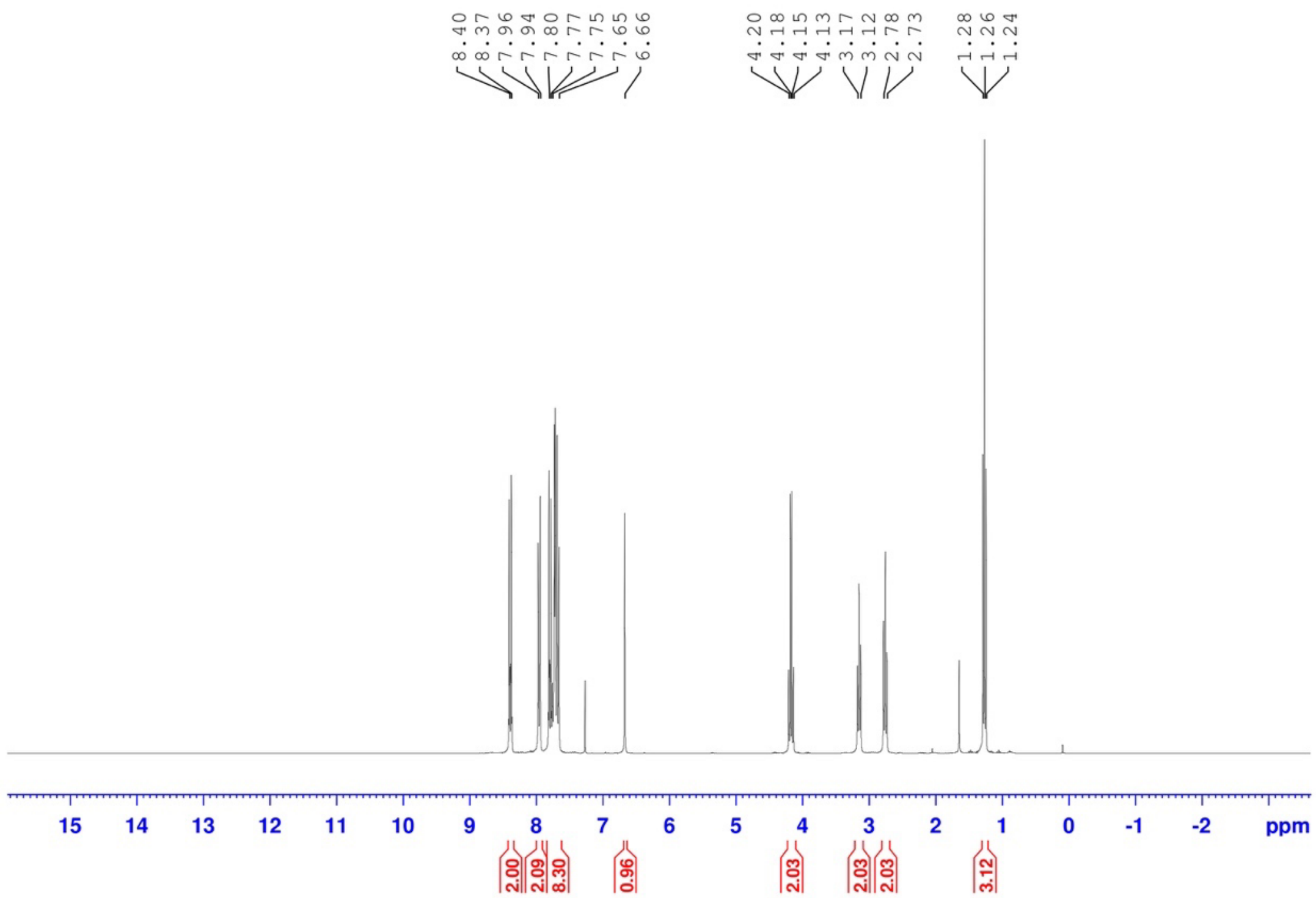

Figure S113. ^{1}H NMR spectrum of ethyl 3-(1-(4-nitrophenyl)-5-(4'-(trifluoromethyl)-[1,1'-biphenyl]-4-yl)-1*H*-pyrazol-3-yl)propanoate (**S-12**) in $CDCl_3$.

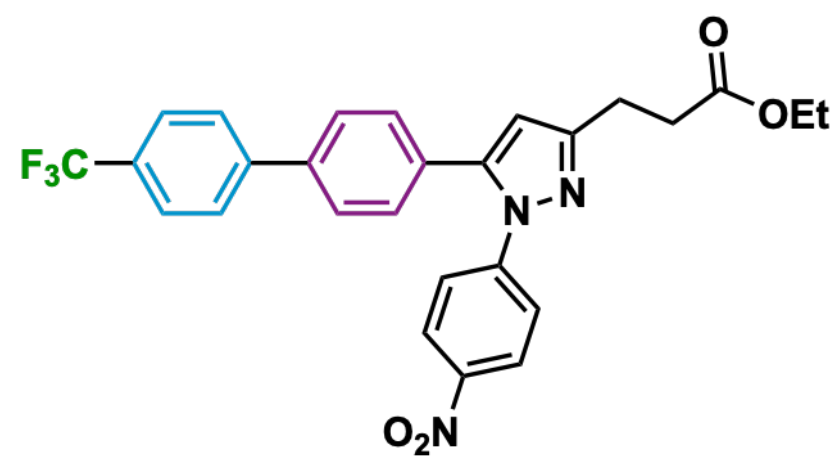

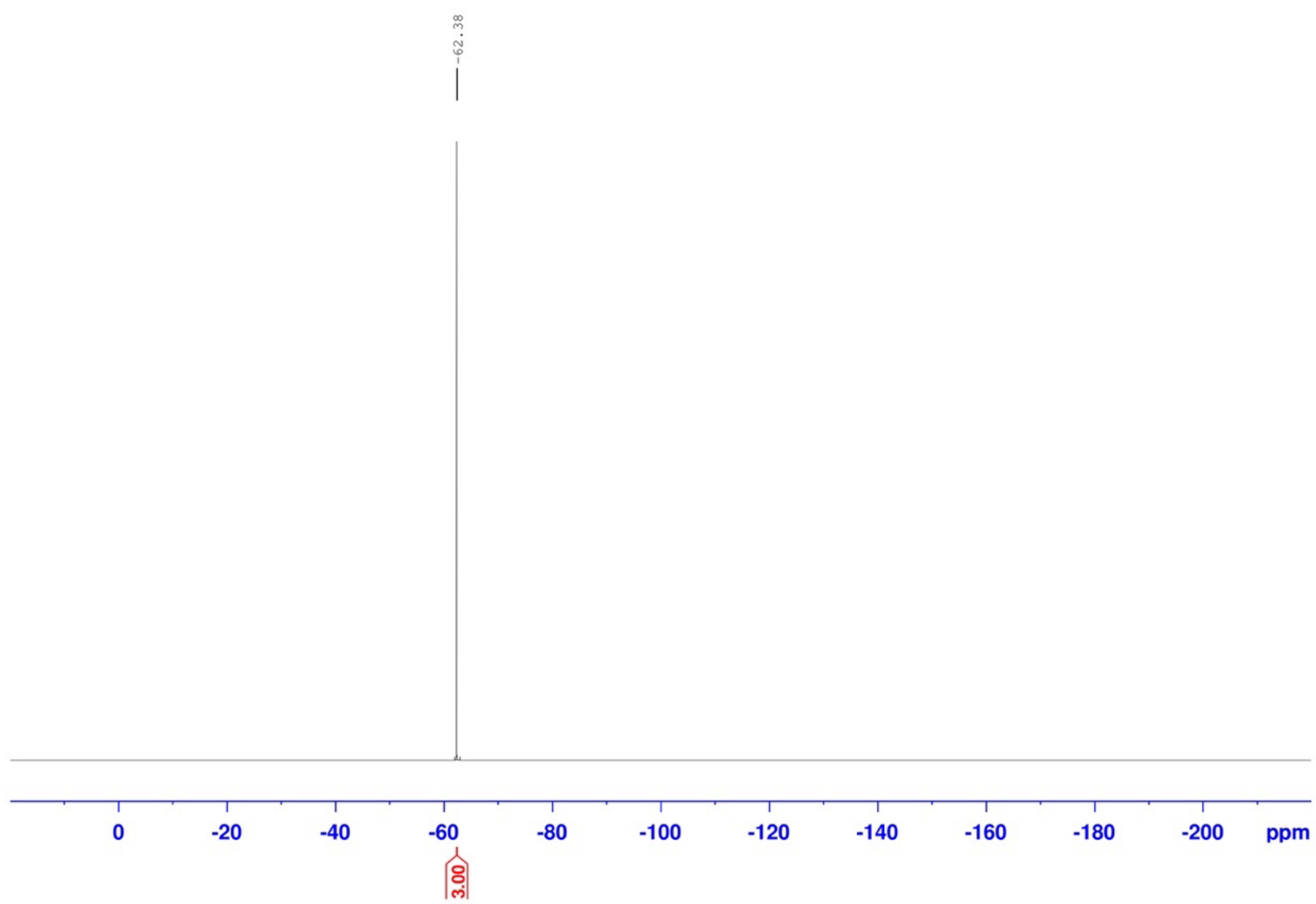

Figure S114. ^{19}F NMR spectrum of ethyl 3-(1-(4-nitrophenyl)-5-(4'-(trifluoromethyl)-[1,1'-biphenyl]-4-yl)-1*H*-pyrazol-3-yl)propanoate (**S-12**) in $CDCl_3$.

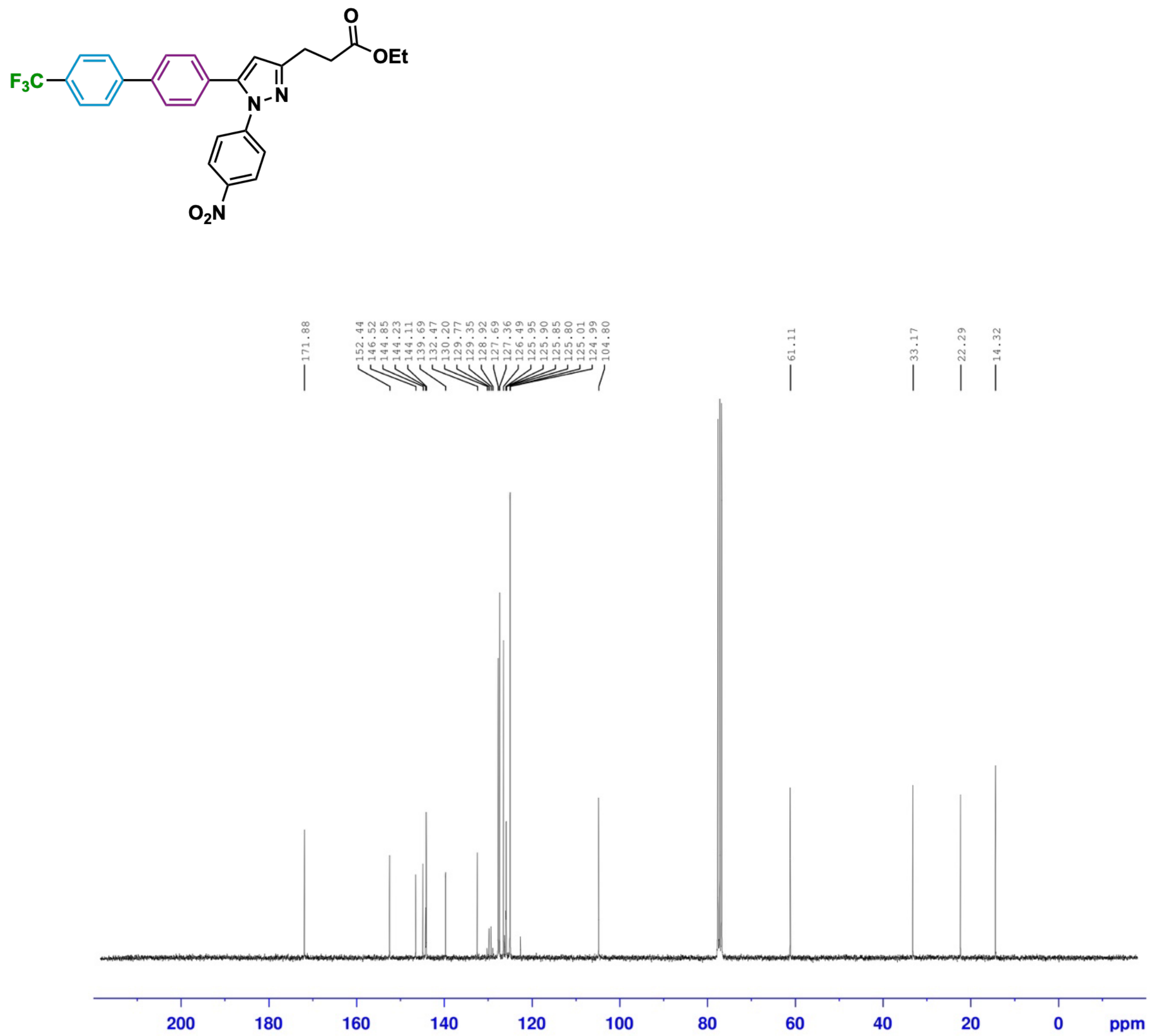

Figure S115. $^{13}C\{^1H\}$ NMR spectrum of ethyl 3-(1-(4-nitrophenyl)-5-(4'-(trifluoromethyl)-[1,1'-biphenyl]-4-yl)-1*H*-pyrazol-3-yl)propanoate (**S-12**) in $CDCl_3$.

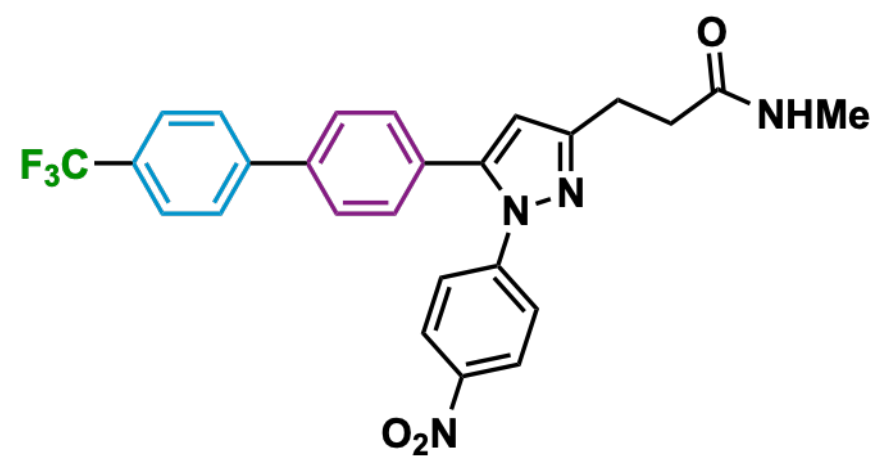

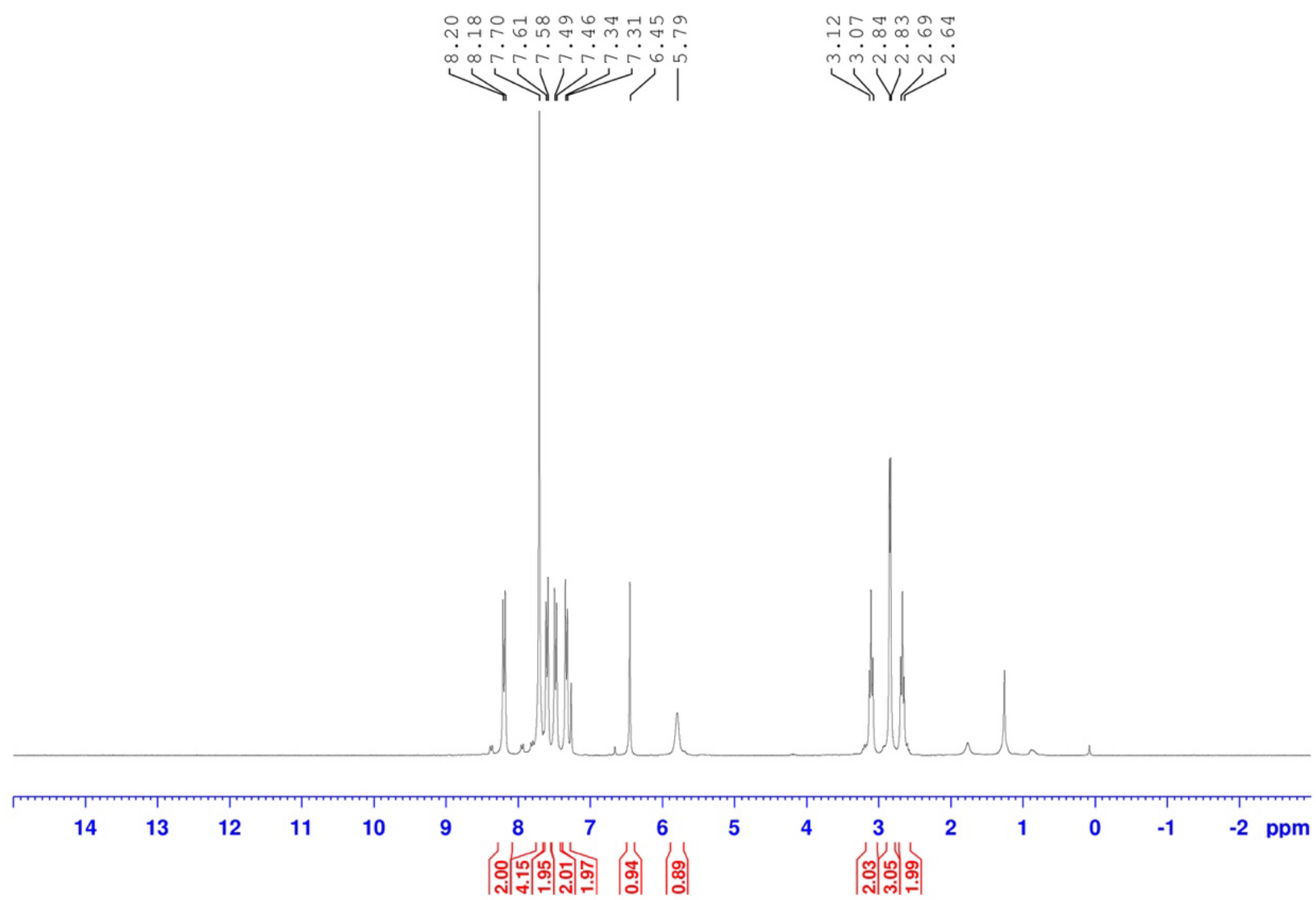

Figure S116. ^{1}H NMR spectrum of *N*-methyl-3-(1-(4-nitrophenyl)-5-(4'-(trifluoromethyl)-[1,1'-biphenyl]-4-yl)-1*H*-pyrazol-3-yl)propanamide (**S-13**) in $CDCl_3$.

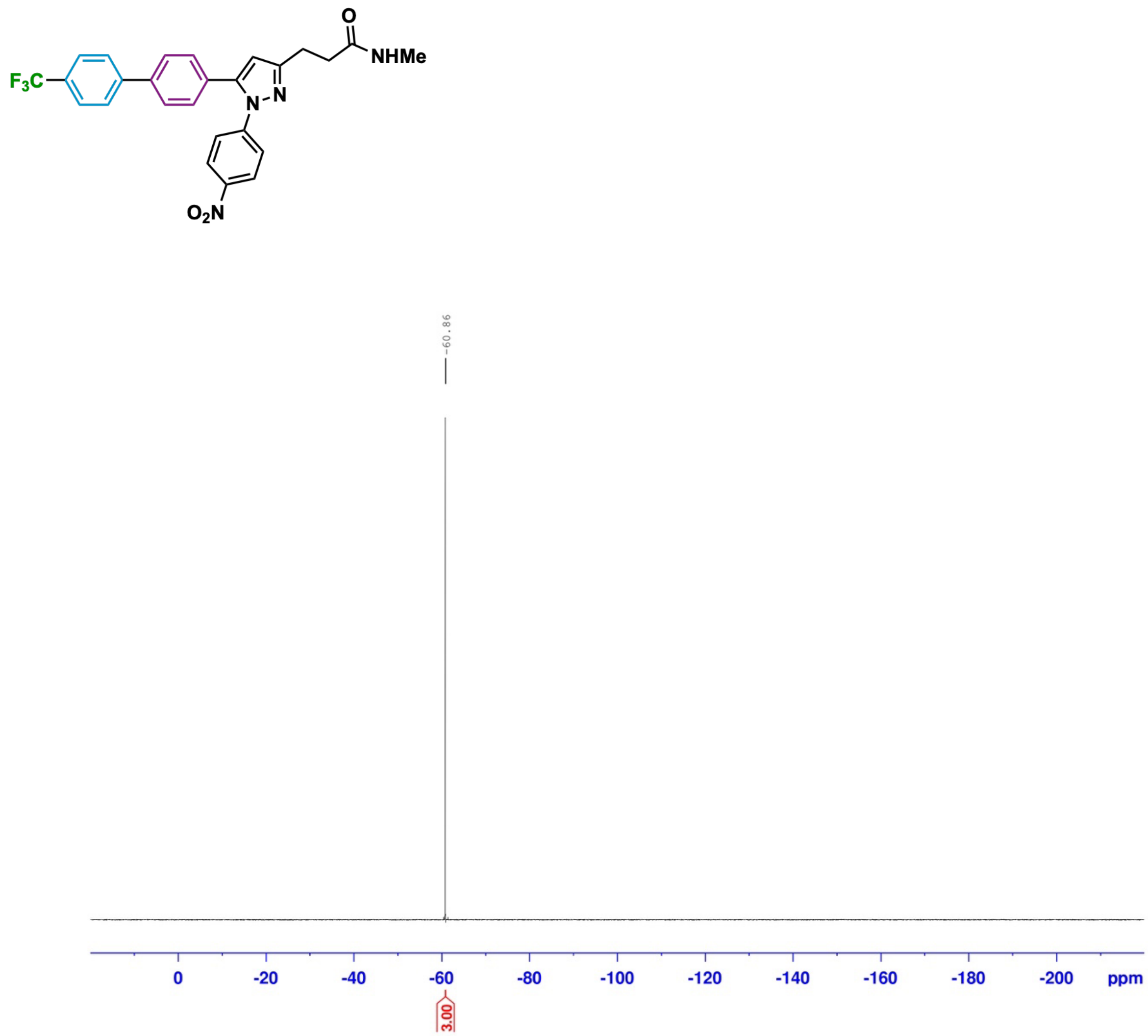

Figure S117. ^{19}F NMR spectrum of *N*-methyl-3-(1-(4-nitrophenyl)-5-(4'-(trifluoromethyl)-[1,1'-biphenyl]-4-yl)-1*H*-pyrazol-3-yl)propanamide (**S-15**) in $CDCl_3$.

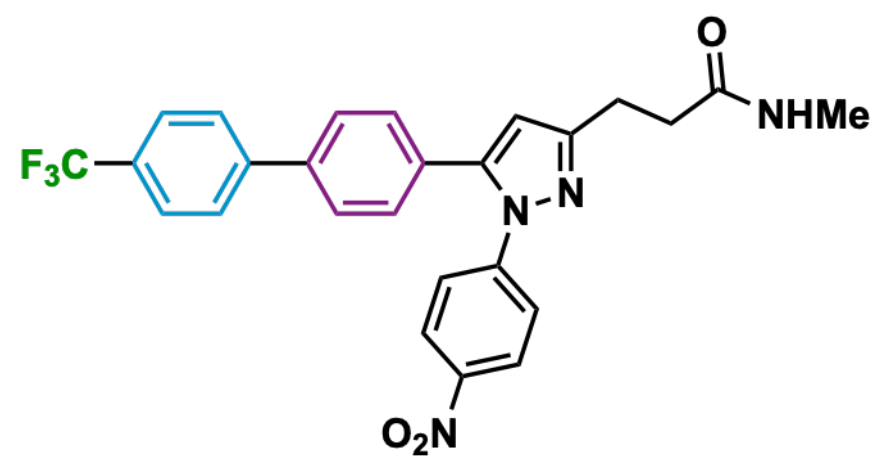

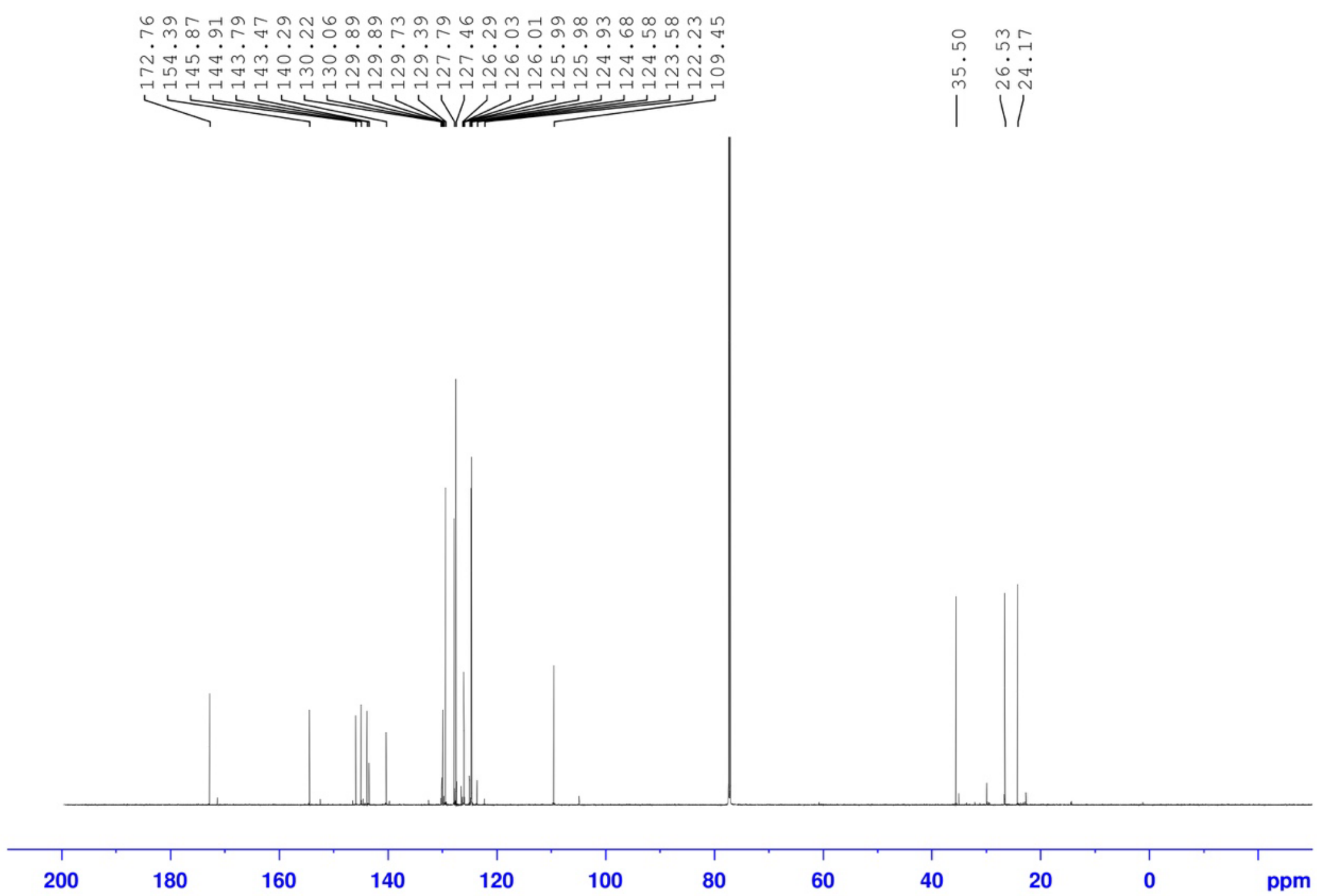

Figure S118. $^{13}C\{^{1}H\}$ NMR spectrum of *N*-methyl-3-(1-(4-nitrophenyl)-5-(4'-(trifluoromethyl)-[1,1'-biphenyl]-4-yl)-1*H*-pyrazol-3-yl)propanamide (**S-13**) in $CDCl_3$.

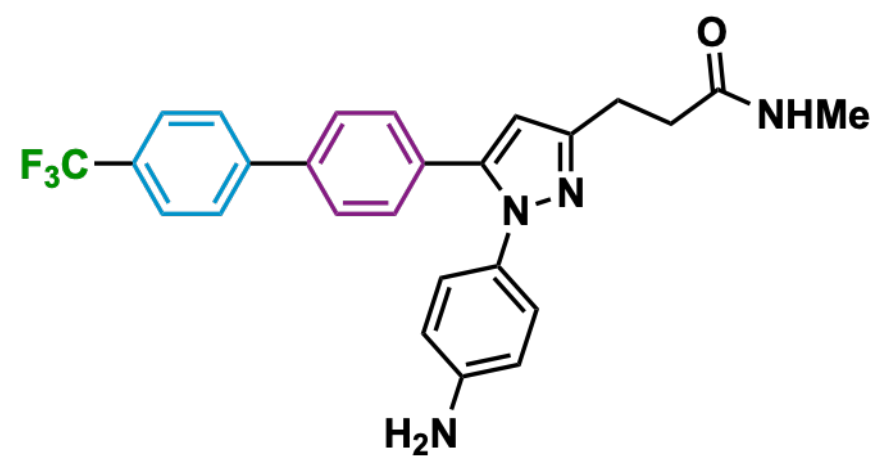

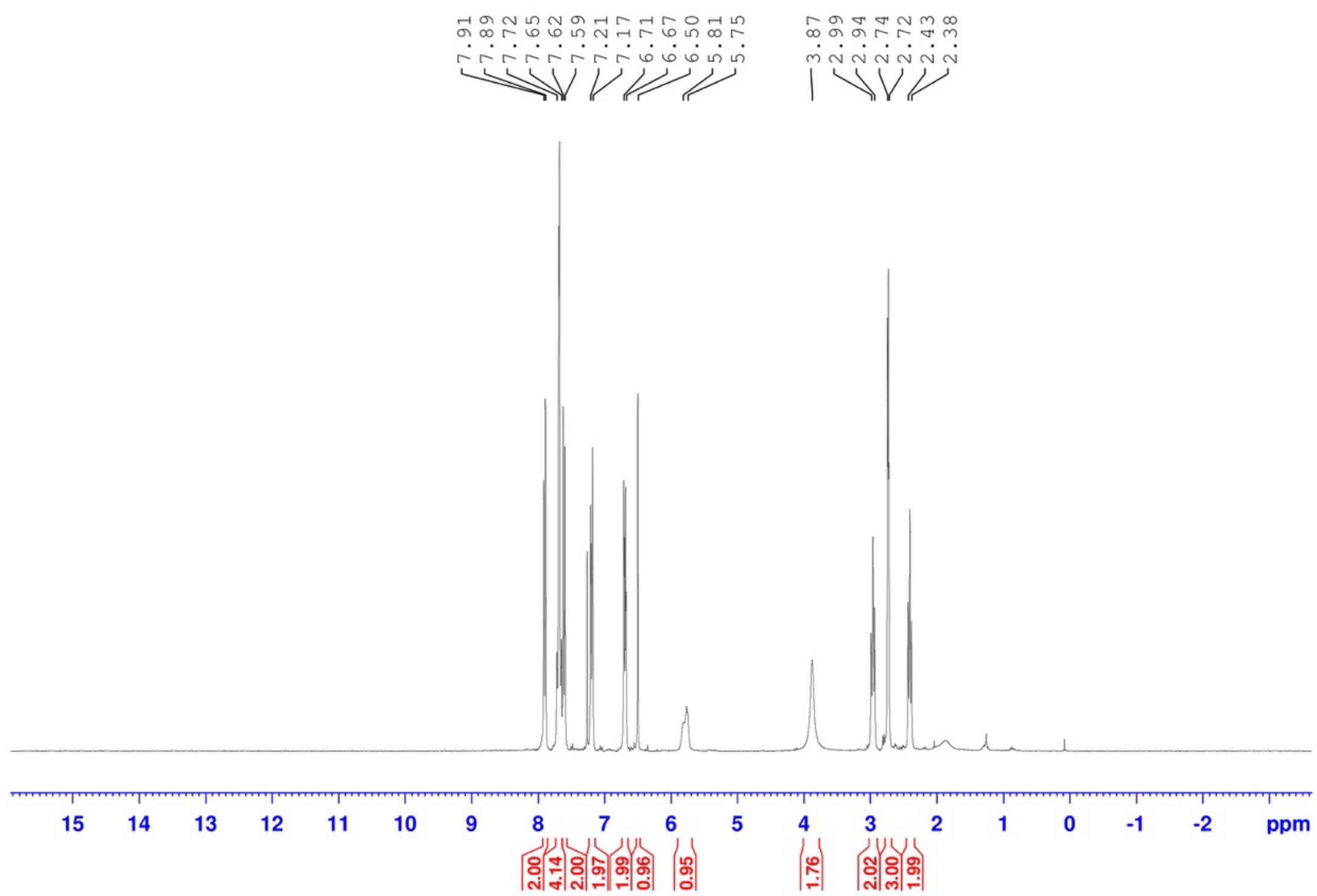

Figure S119. ^{1}H NMR spectrum of 3-(1-(4-aminophenyl)-5-(4'-(trifluoromethyl)-[1,1'-biphenyl]-4-yl)-1*H*-pyrazol-3-yl)-*N*-methylpropanamide (**S-14**) in $CDCl_3$.

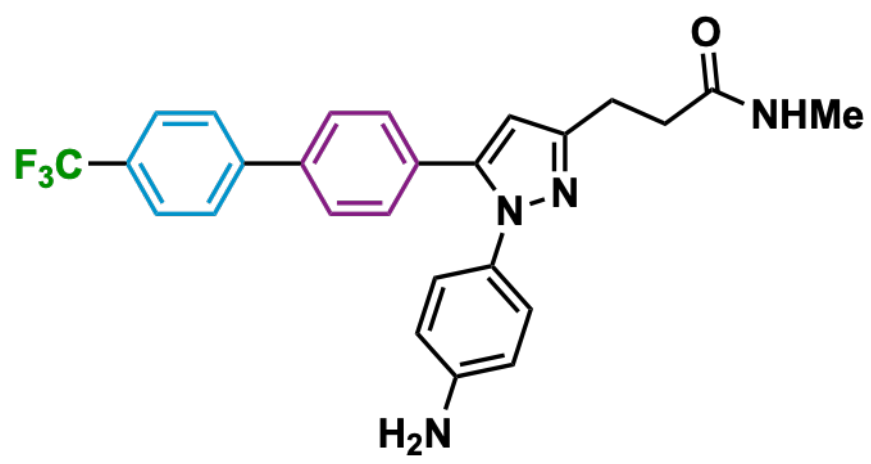

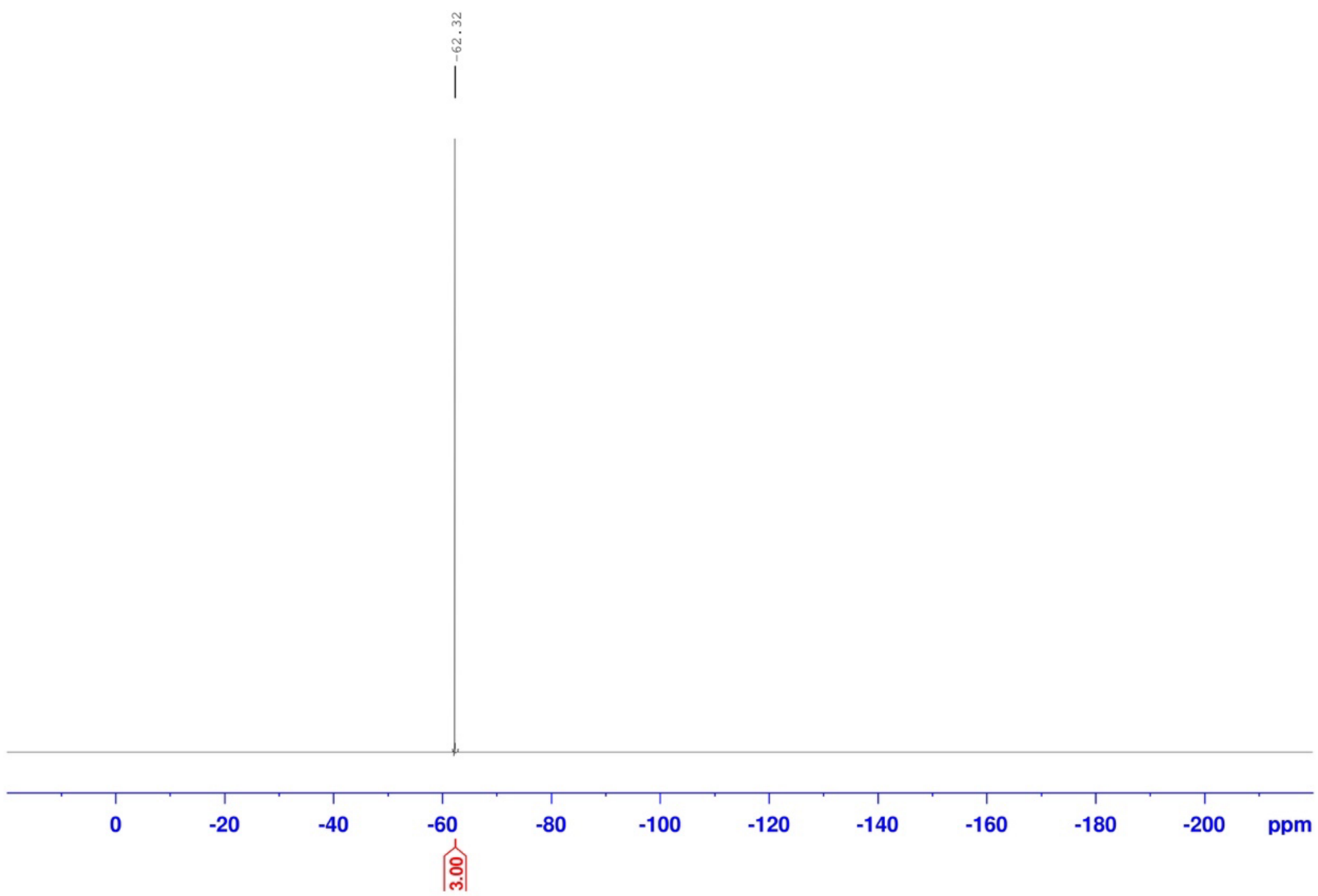

Figure S120. ^{19}F NMR spectrum of 3-(1-(4-aminophenyl)-5-(4'-(trifluoromethyl)-[1,1'-biphenyl]-4-yl)-1*H*-pyrazol-3-yl)-*N*-methylpropanamide (**S-16**) in $CDCl_3$.

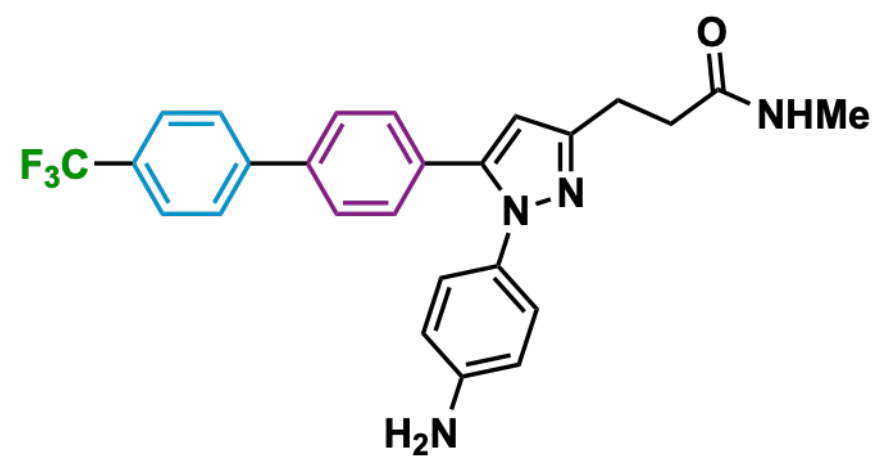

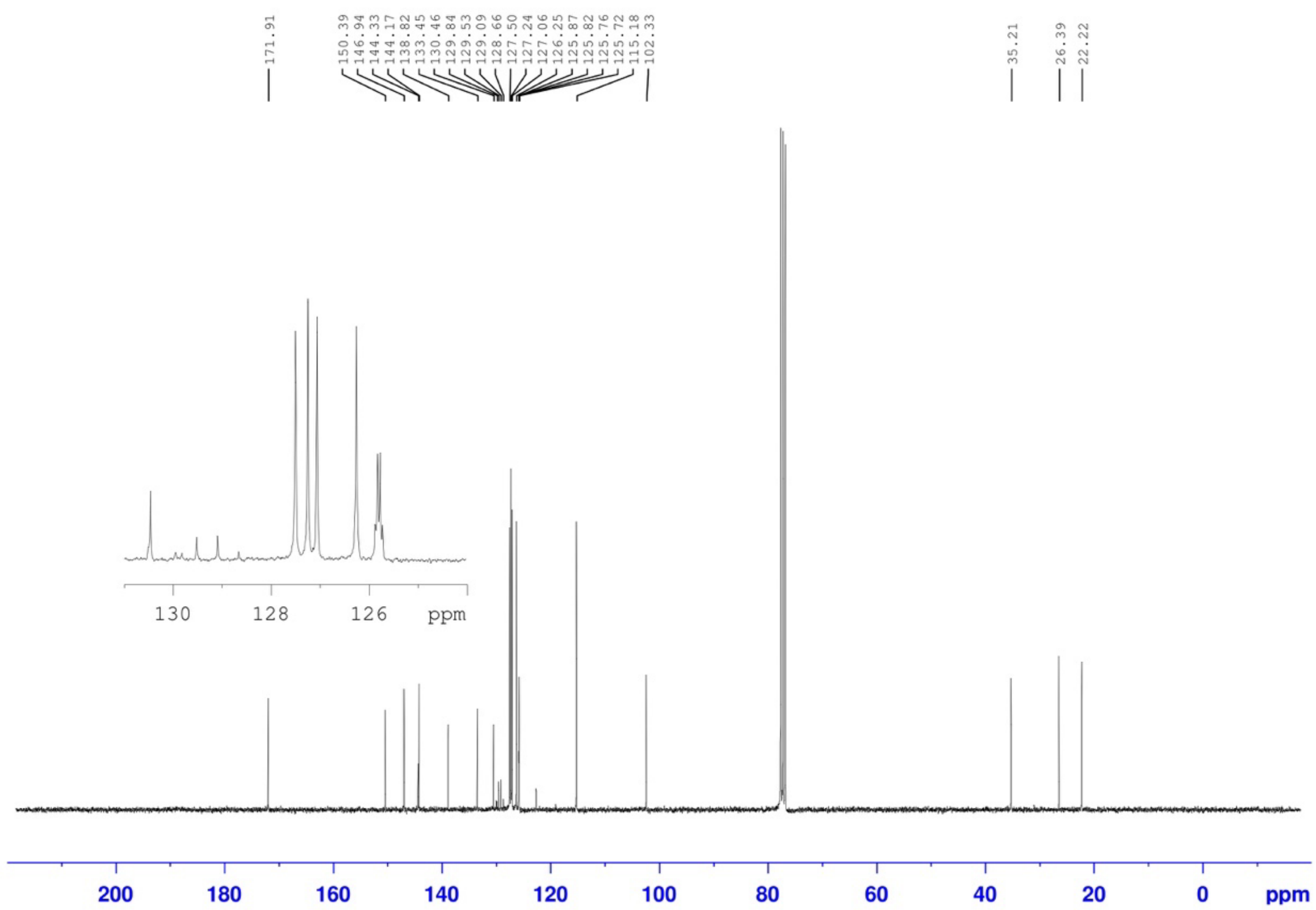

Figure S121. $^{13}C\{^{1}H\}$ NMR spectrum of 3-(1-(4-aminophenyl)-5-(4'-(trifluoromethyl)-[1,1'-biphenyl]-4-yl)-1*H*-pyrazol-3-yl)-*N*-methylpropanamide (**S-14**).

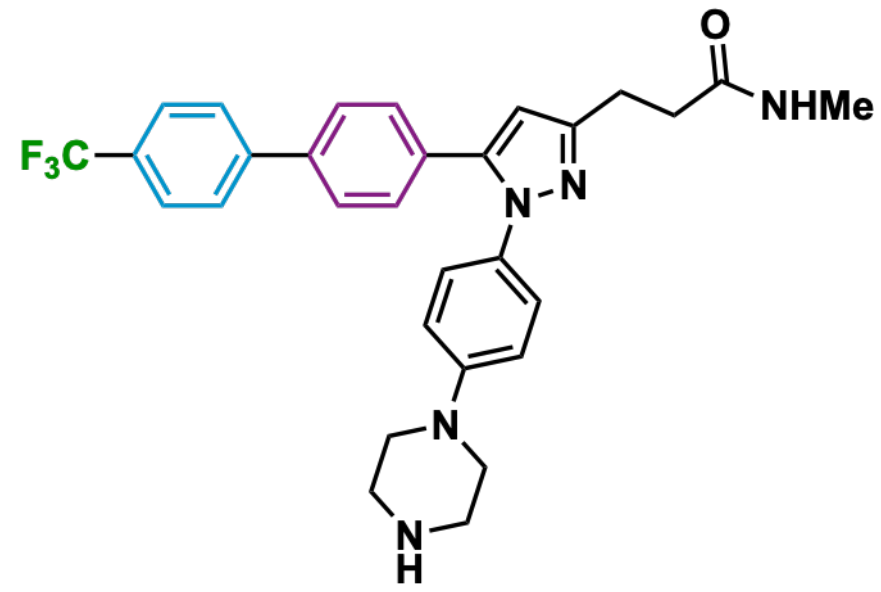

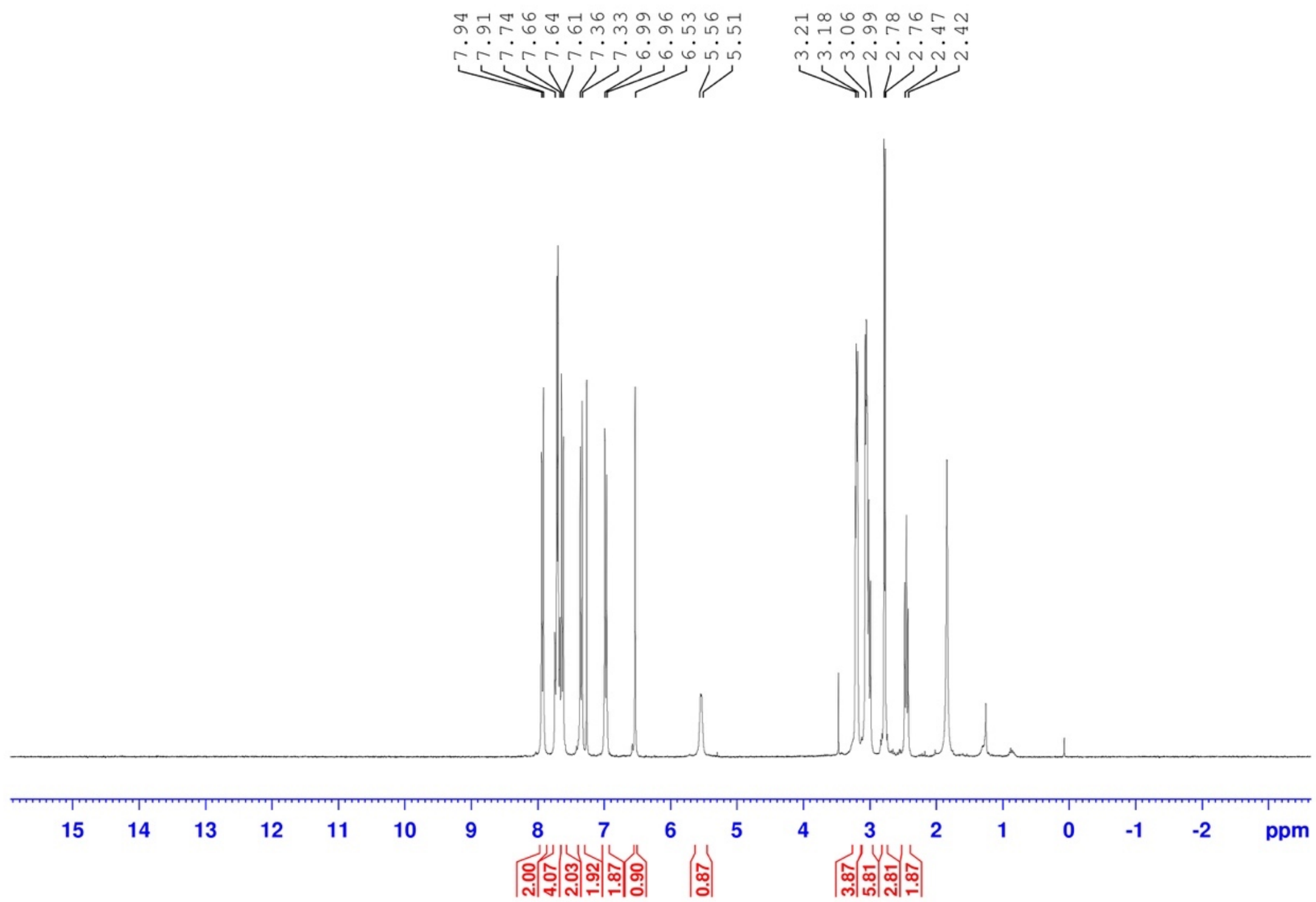

Figure S122. ^{1}H NMR spectrum of *N*-methyl-3-(1-(4-(piperazin-1-yl)phenyl)-5-(4'-(trifluoromethyl)-[1,1'-biphenyl]-4-yl)-1*H*-pyrazol-3-yl)propanamide (**31**) in $CDCl_3$.

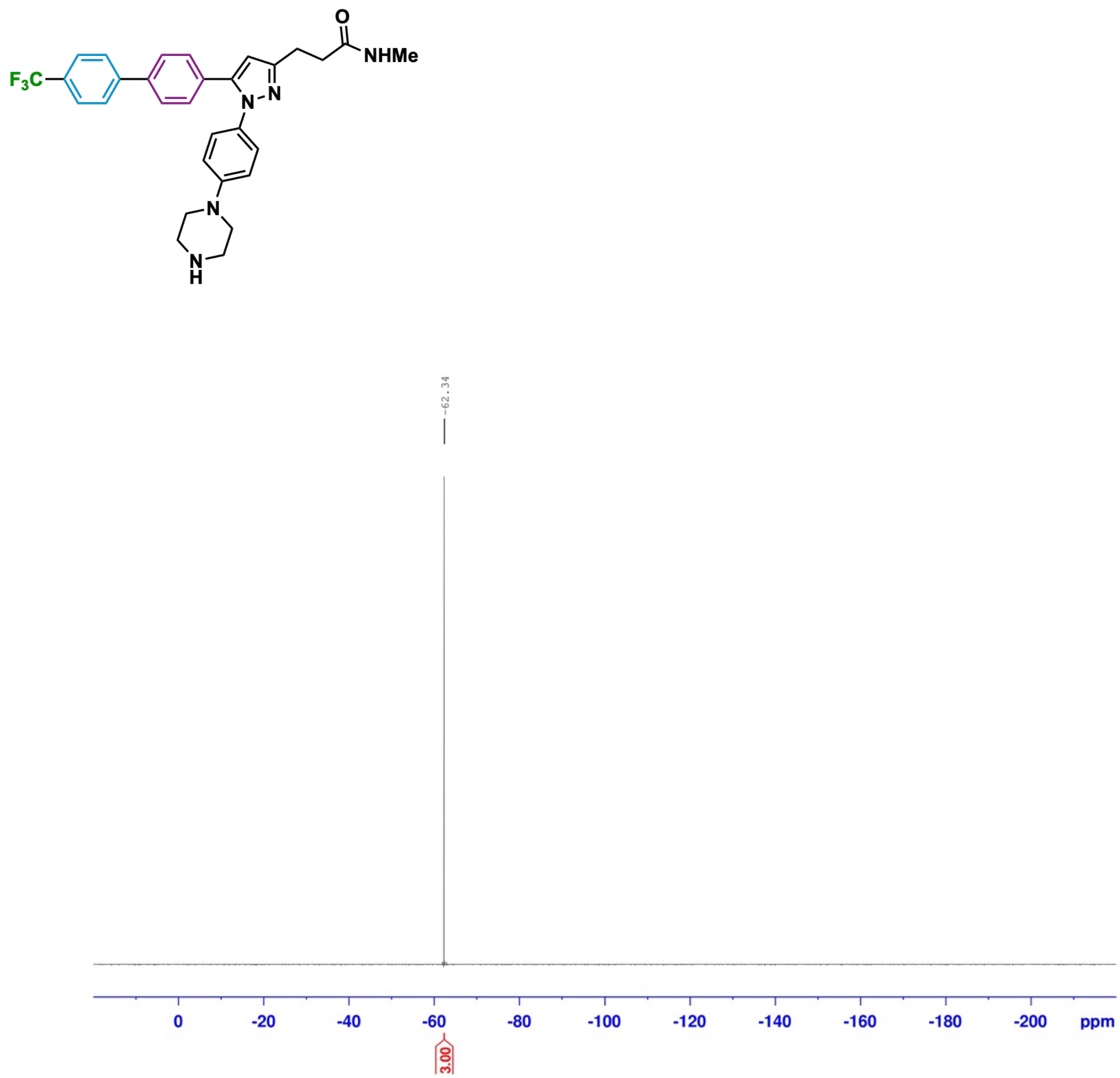

Figure S123. ^{19}F NMR spectrum of *N*-methyl-3-(1-(4-(piperazin-1-yl)phenyl)-5-(4'-(trifluoromethyl)-[1,1'-biphenyl]-4-yl)-1*H*-pyrazol-3-yl)propanamide (**31**) in $CDCl_3$.

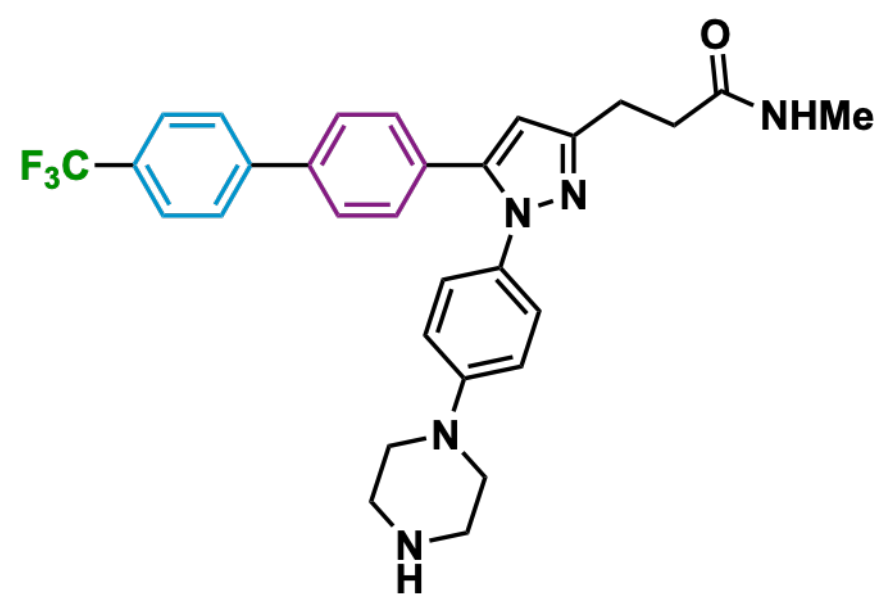

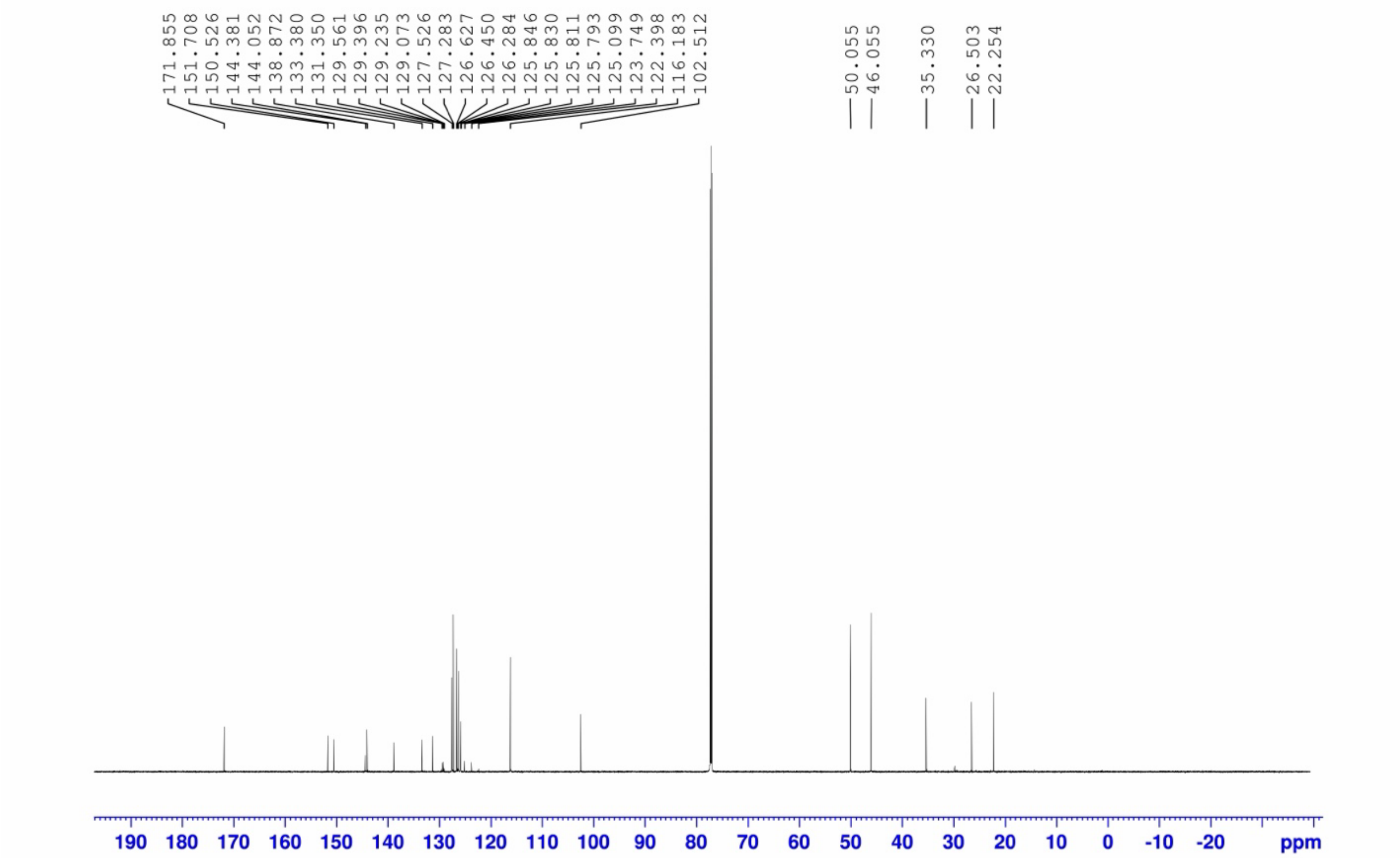

Figure S124. $^{13}C\{^{1}H\}$ NMR spectrum of *N*-methyl-3-(1-(4-(piperazin-1-yl)phenyl)-5-(4'-(trifluoromethyl)-[1,1'-biphenyl]-4-yl)-1*H*-pyrazol-3-yl)propanamide (**31**) in $CDCl_3$.

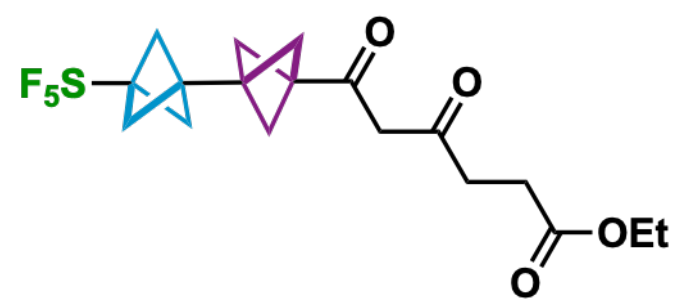

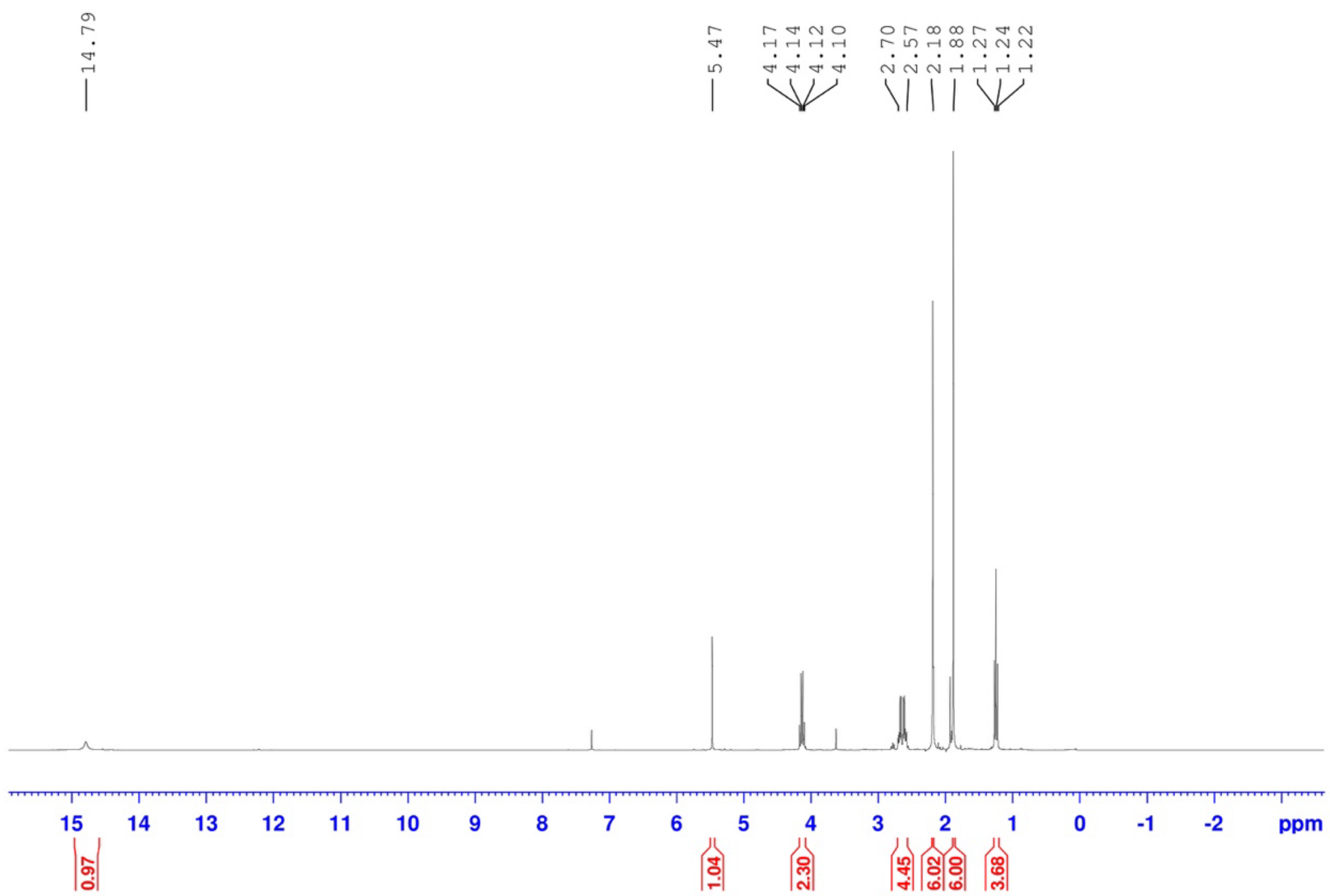

Figure S125. ^{1}H NMR spectrum ethyl 4,6-dioxo-6-(3'-(pentafluoro-λ^6-sulfanyl)-[1,1'-bi(bicyclo[1.1.1]pentan)]-3-yl)hexanoate (**S-15**) in $CDCl_3$.

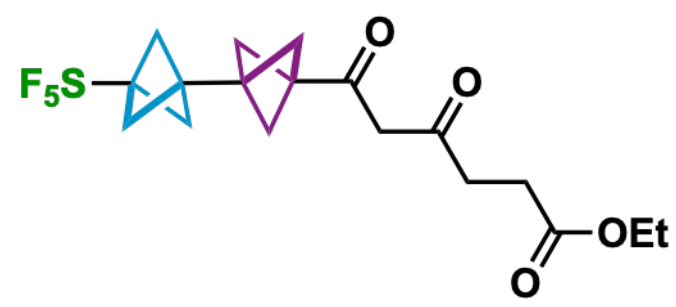

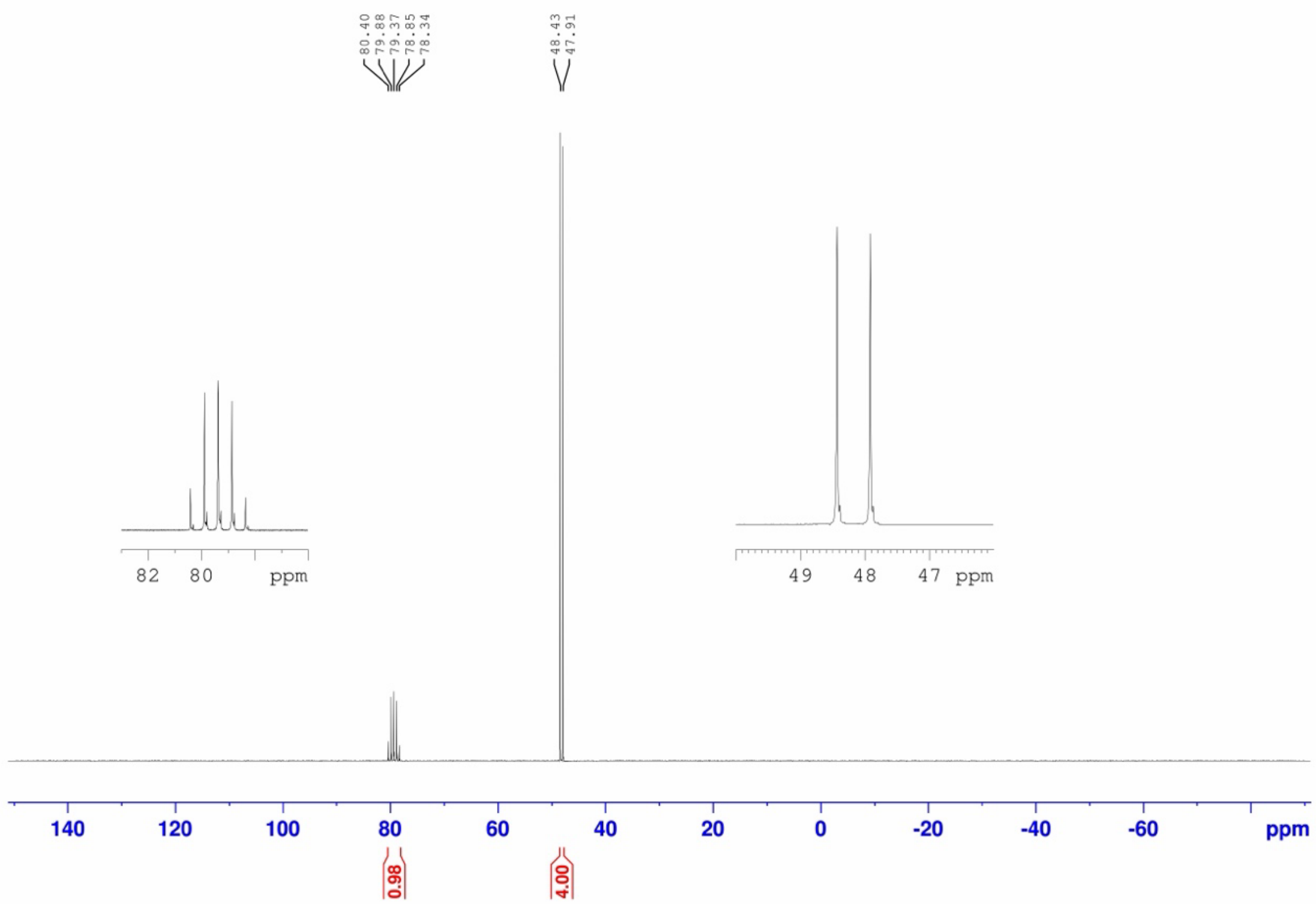

Figure S126. ^{19}F NMR spectrum ethyl 4,6-dioxo-6-(3'-(pentafluoro-λ[6]-sulfanyl)-[1,1'-bi(bicyclo[1.1.1]pentan)]-3-yl)hexanoate (**S-15**) in $CDCl_3$.

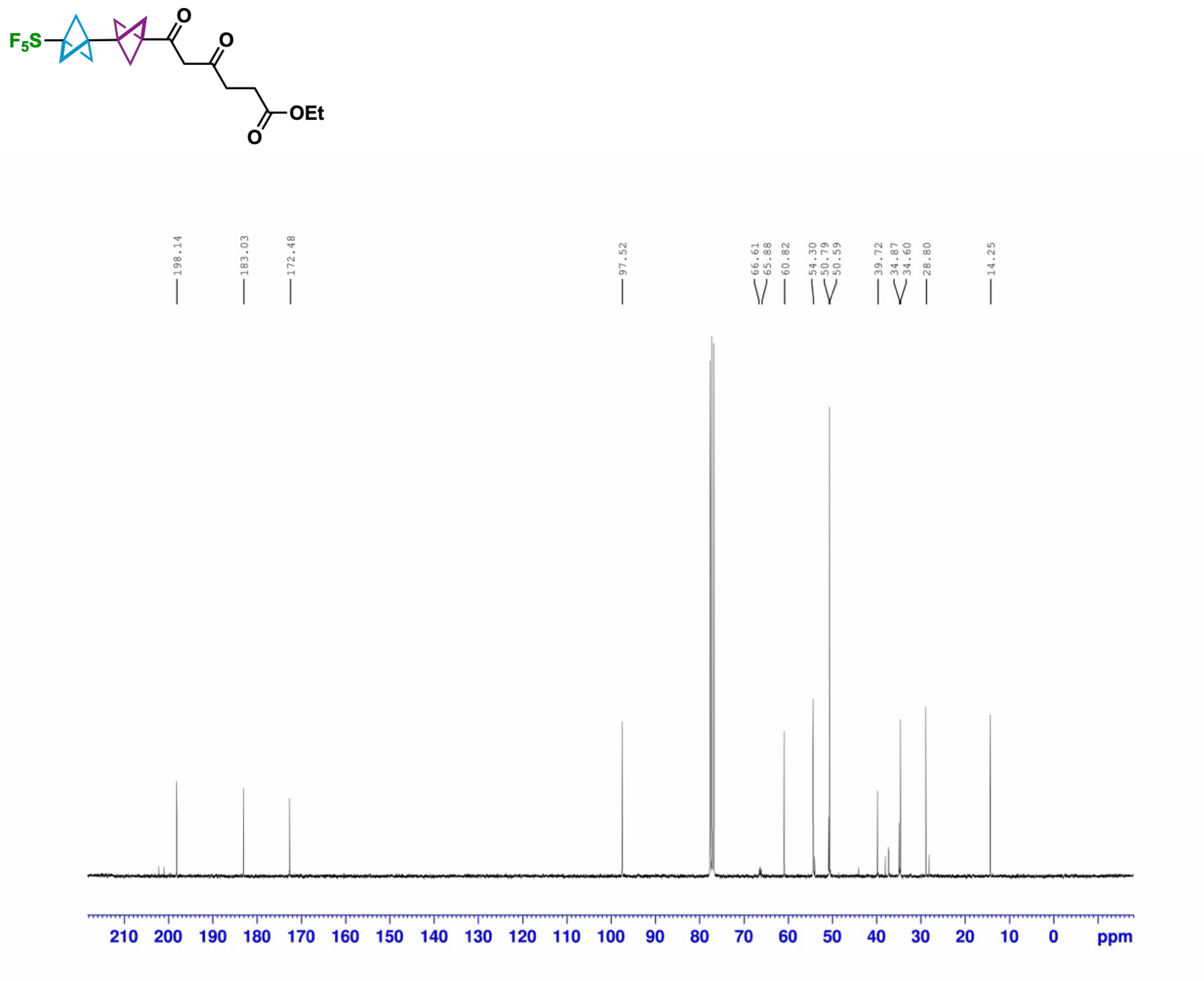

Figure S127. $^{13}C\{^{1}H\}$ NMR spectrum ethyl 4,6-dioxo-6-(3'-(pentafluoro-λ^6-sulfanyl)-[1,1'-bi(bicyclo[1.1.1]pentan)]-3-yl)hexanoate (**S-15**) in $CDCl_3$.

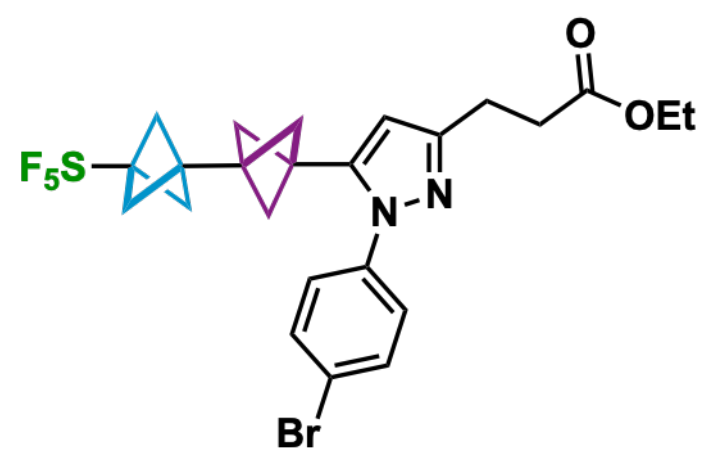

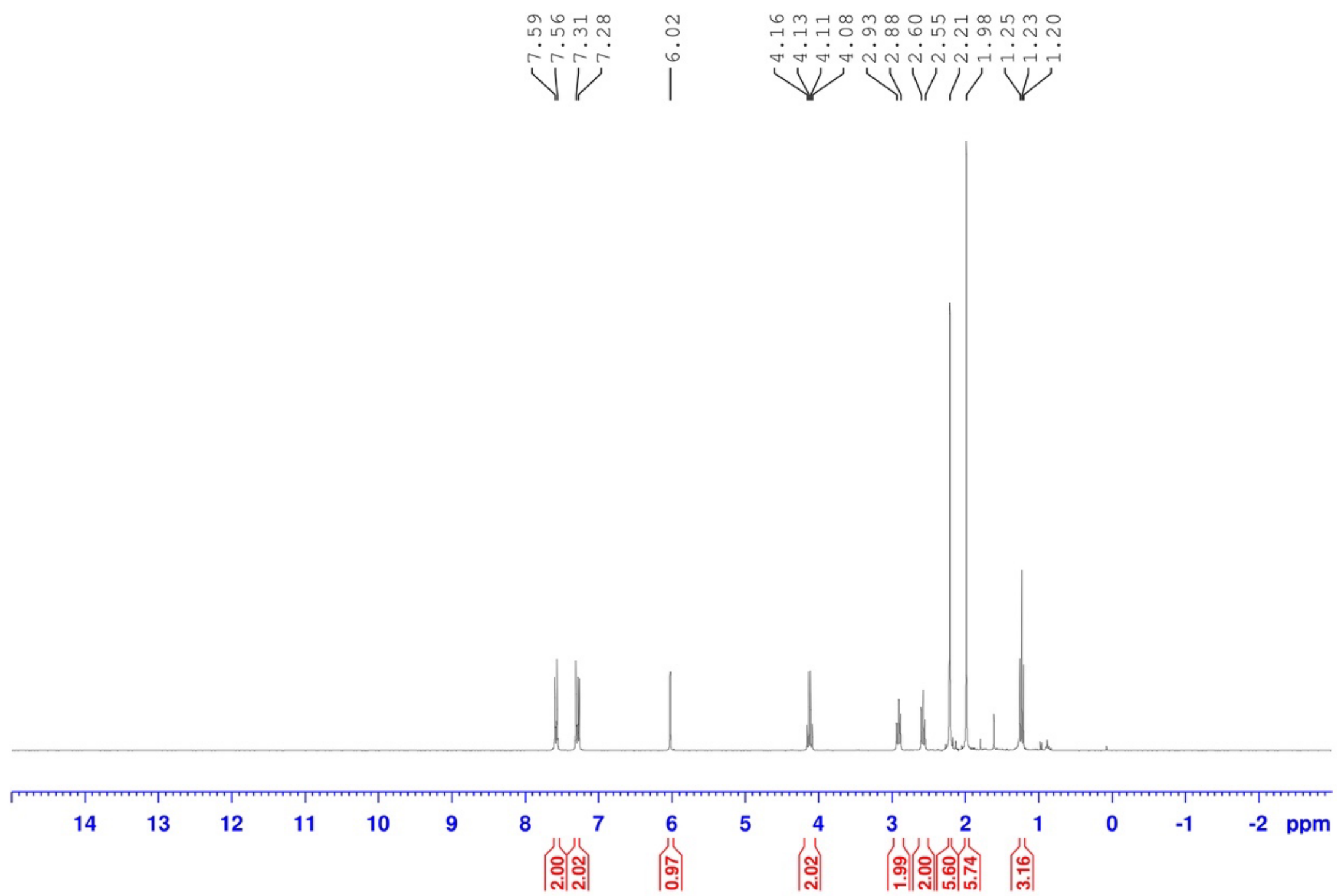

Figure S128. ^{1}H NMR spectrum of ethyl 3-(1-(4-bromophenyl)-5-(3'-(pentafluoro-λ^6-sulfanyl)-[1,1'-bi(bicyclo[1.1.1]pentan)]-3-yl)-1*H*-pyrazol-3-yl)propanoate (**S-16**) in $CDCl_3$.

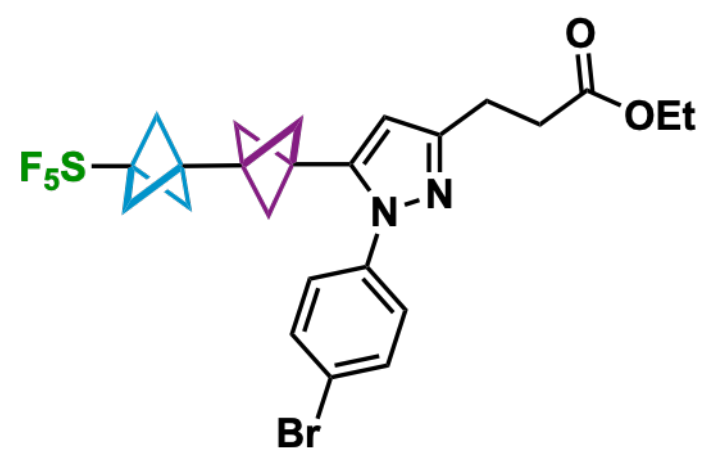

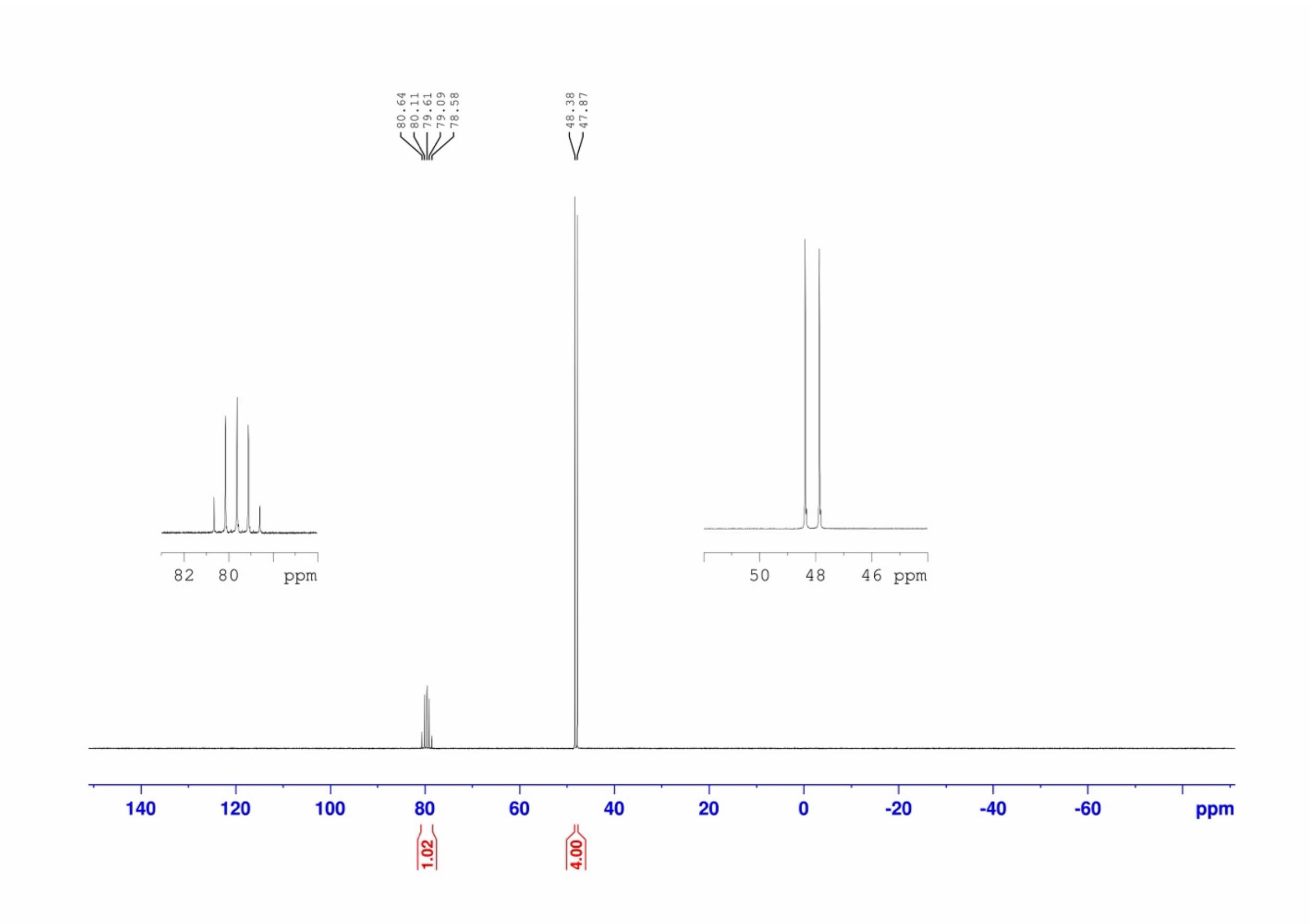

Figure S129. ^{19}F NMR spectrum of ethyl 3-(1-(4-bromophenyl)-5-(3'-(pentafluoro-λ^6-sulfanyl)-[1,1'-bi(bicyclo[1.1.1]pentan)]-3-yl)-1*H*-pyrazol-3-yl)propanoate (**S-16**) in $CDCl_3$.

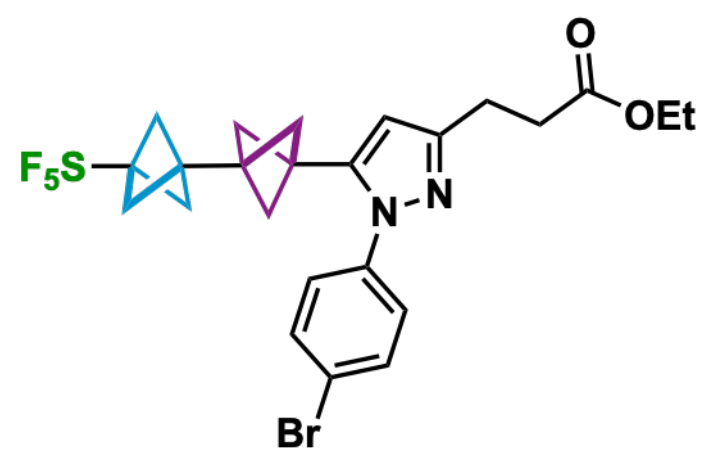

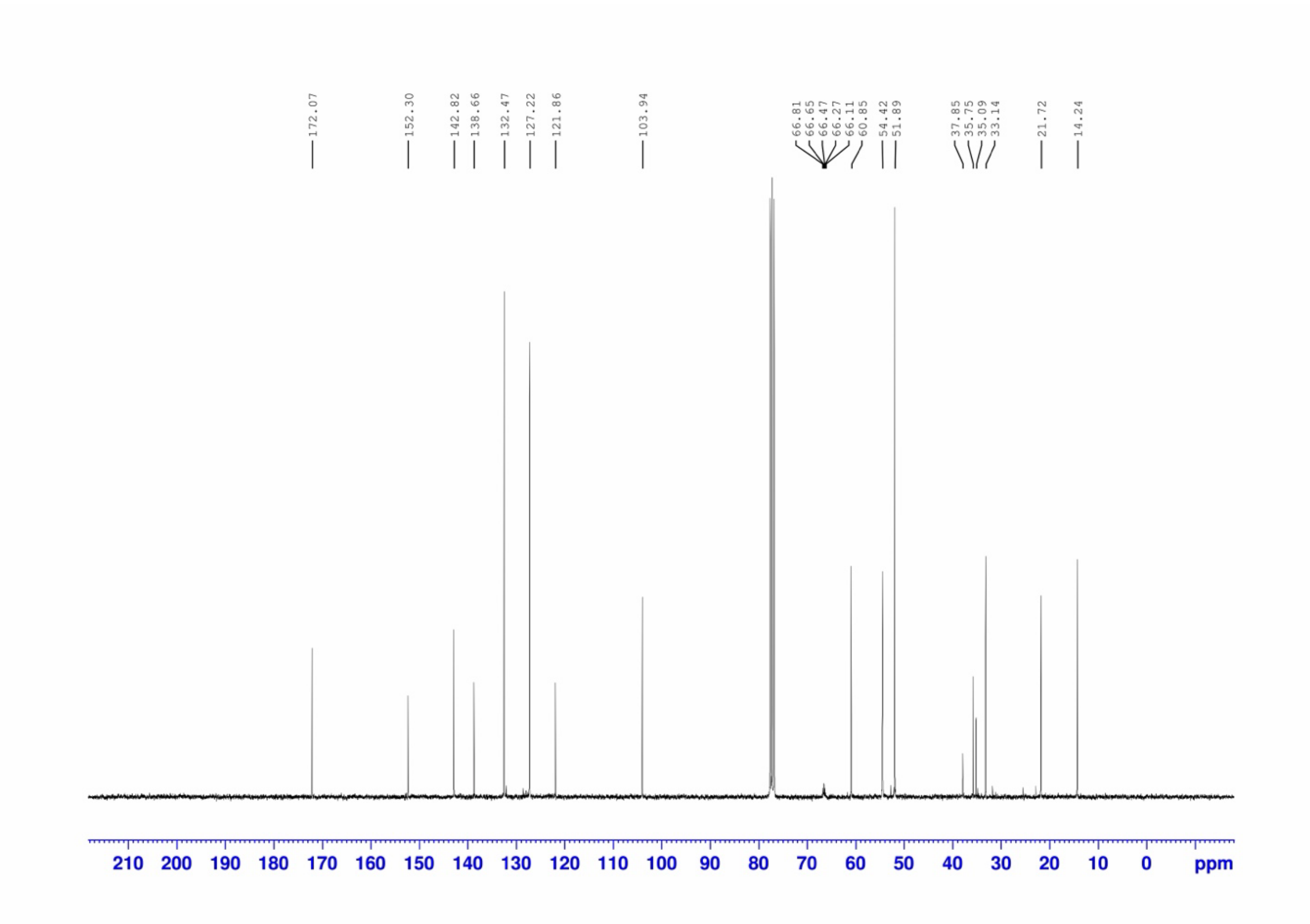

Figure S130. $^{13}C\{^1H\}$ NMR spectrum of ethyl 3-(1-(4-bromophenyl)-5-(3'-(pentafluoro-λ^6-sulfanyl)-[1,1'-bi(bicyclo[1.1.1]pentan)]-3-yl)-1*H*-pyrazol-3-yl)propanoate (**S-16**) in $CDCl_3$.

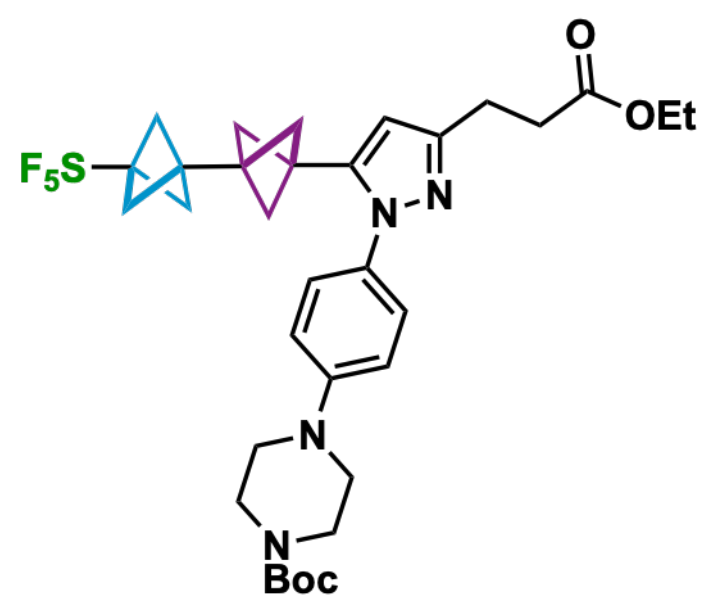

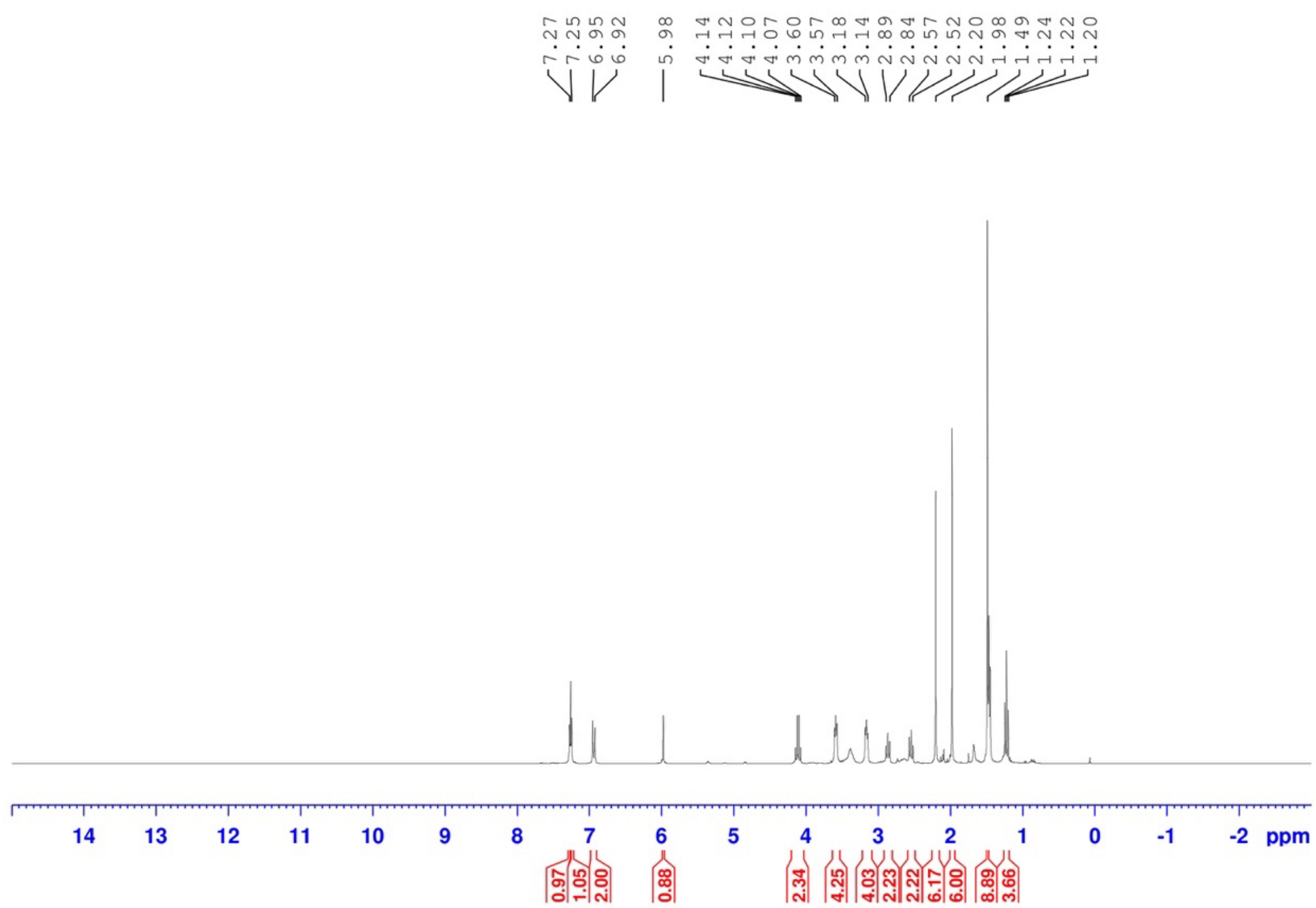

Figure S131. ^{1}H NMR spectrum of *tert*-butyl 4-(4-(3-(3-ethoxy-3-oxopropyl)-5-(3'-(pentafluoro-λ^6-sulfanyl)-[1,1'-bi(bicyclo[1.1.1]pentan)]-3-yl)-1*H*-pyrazol-1-yl)phenyl)piperazine-1-carboxylate (**S-17**) in $CDCl_3$.

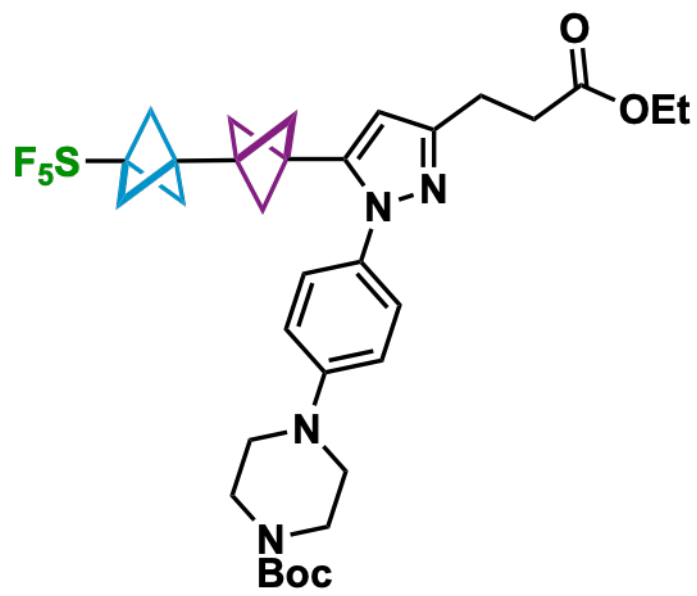

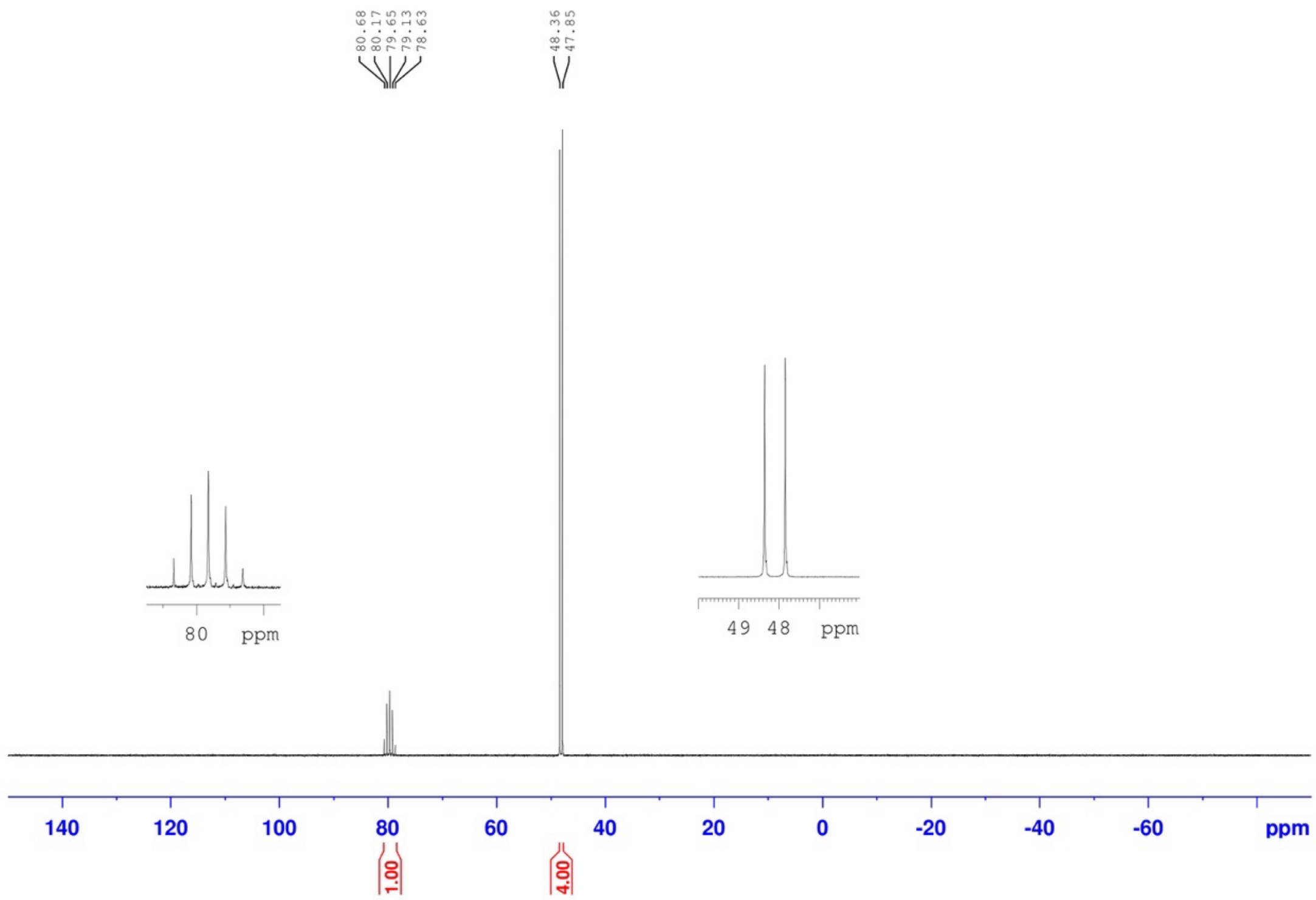

Figure S132. ^{19}F NMR spectrum of *tert*-butyl 4-(4-(3-(3-ethoxy-3-oxopropyl)-5-(3'-(pentafluoro-λ^6-sulfanyl)-[1,1'-bi(bicyclo[1.1.1]pentan)]-3-yl)-1*H*-pyrazol-1-yl)phenyl)piperazine-1-carboxylate (**S-17**) in $CDCl_3$.

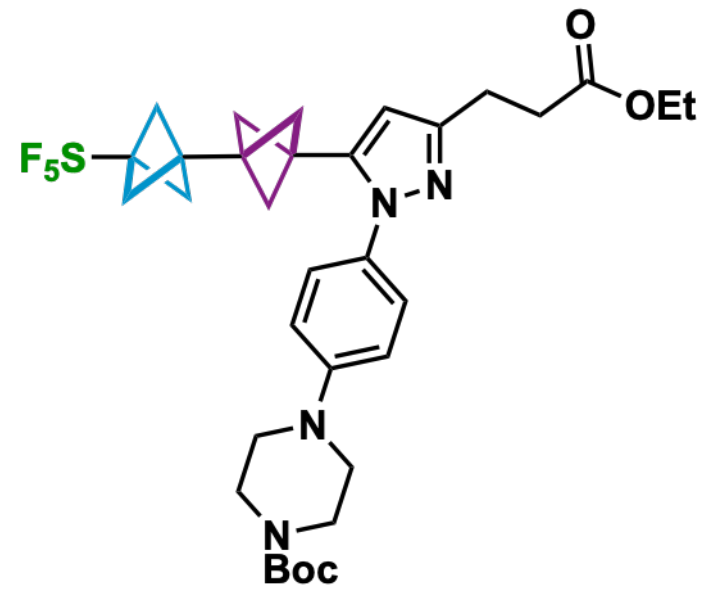

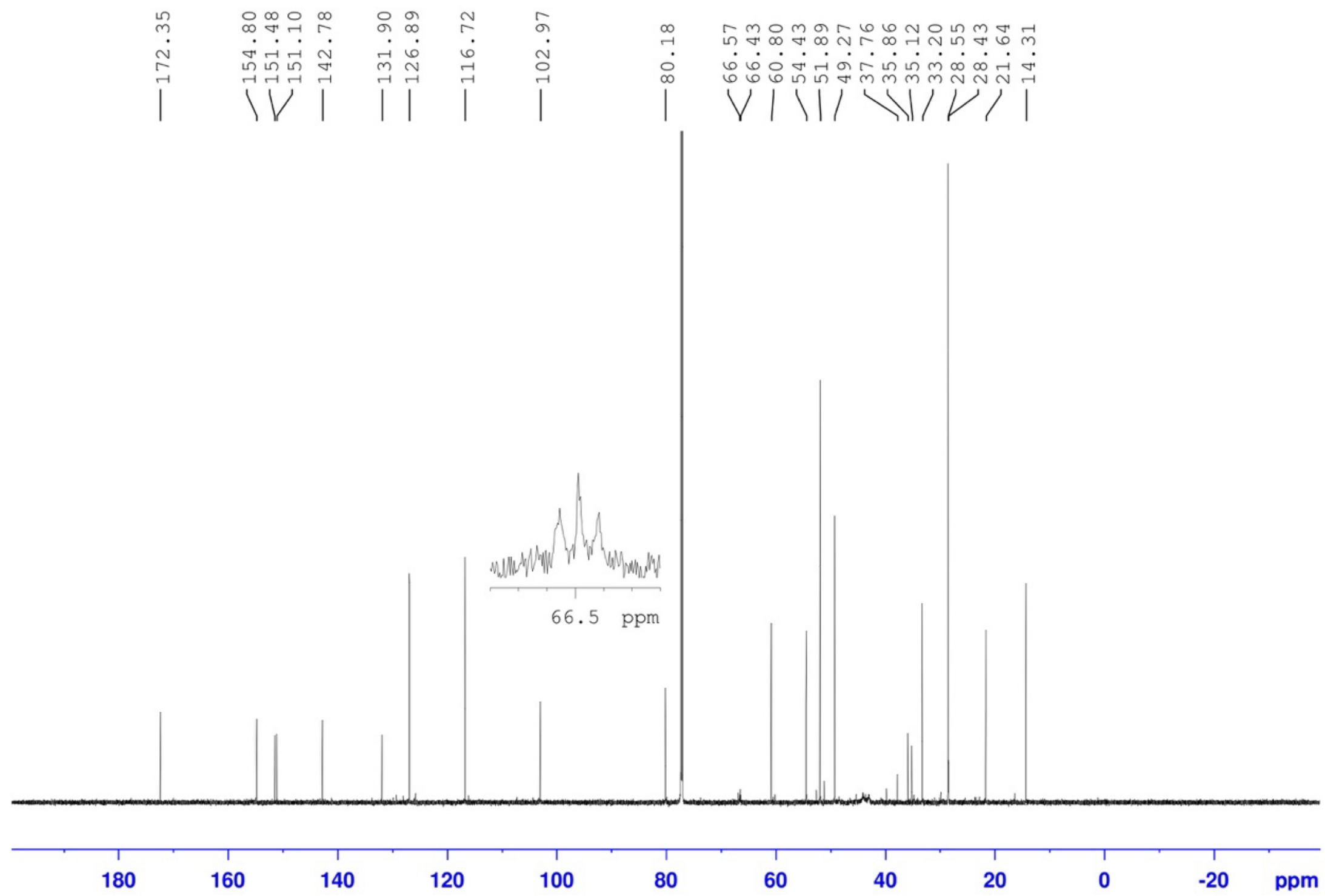

Figure S133. $^{13}C\{^{1}H\}$ NMR spectrum *tert*-butyl 4-(4-(3-(3-ethoxy-3-oxopropyl)-5-(3'-(pentafluoro-λ^{6}-sulfanyl)-[1,1'-bi(bicyclo[1.1.1]pentan)]-3-yl)-1*H*-pyrazol-1-yl)phenyl)piperazine-1-carboxylate (**S-17**) in $CDCl_3$.

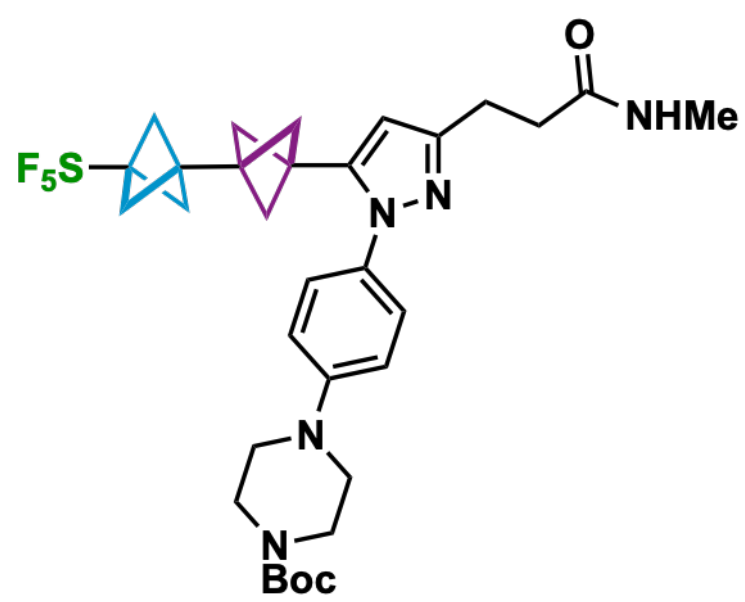

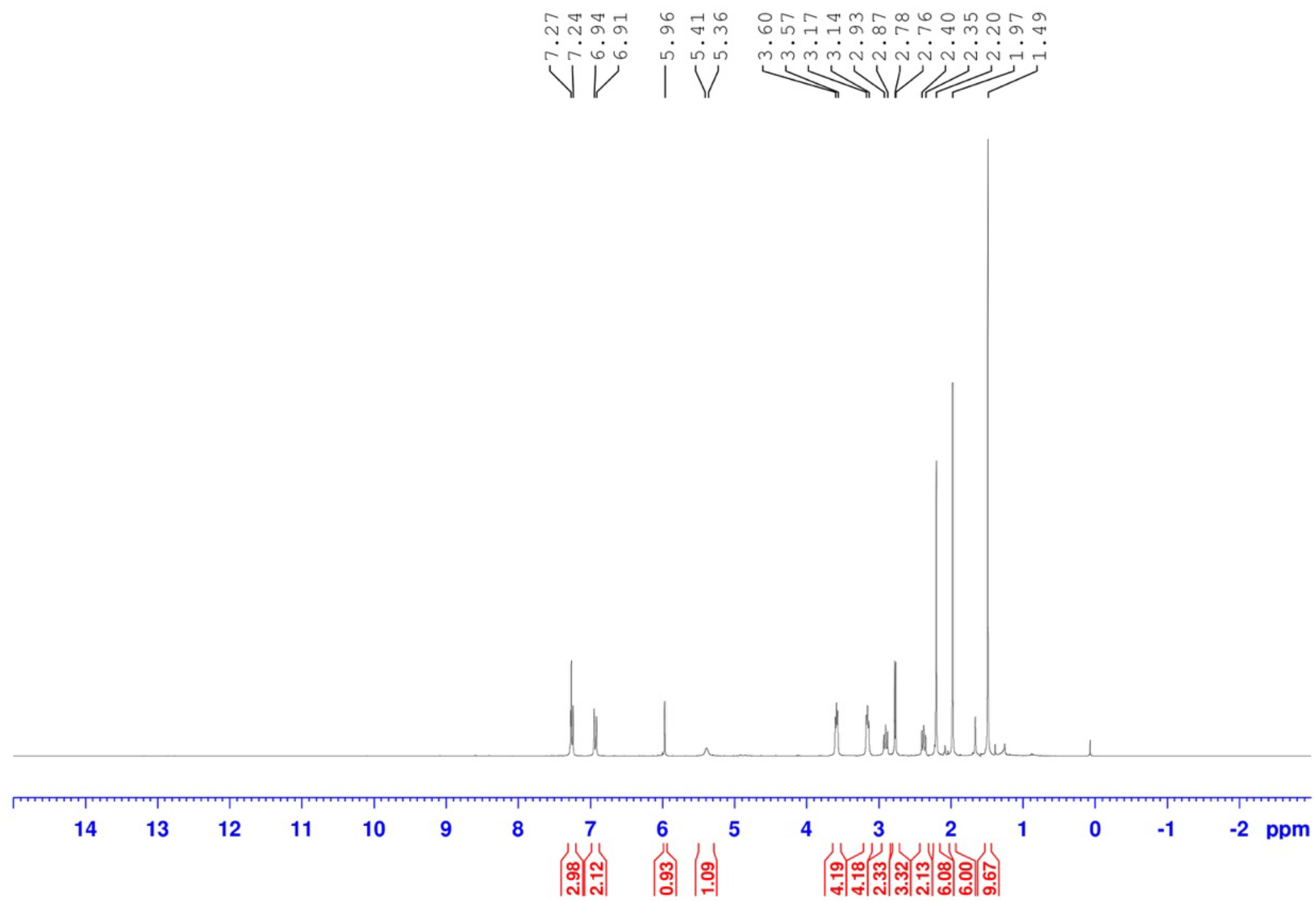

Figure S134. ^{1}H NMR spectrum of *tert*-butyl 4-(4-(3-(3-(methylamino)-3-oxopropyl)-5-(3'-(pentafluoro-λ^6-sulfanyl)-[1,1'-bi(bicyclo[1.1.1] pentan)]-3-yl)-1*H*-pyrazol-1-yl)phenyl)piperazine-1-carboxylate (**S-18**) in $CDCl_3$.

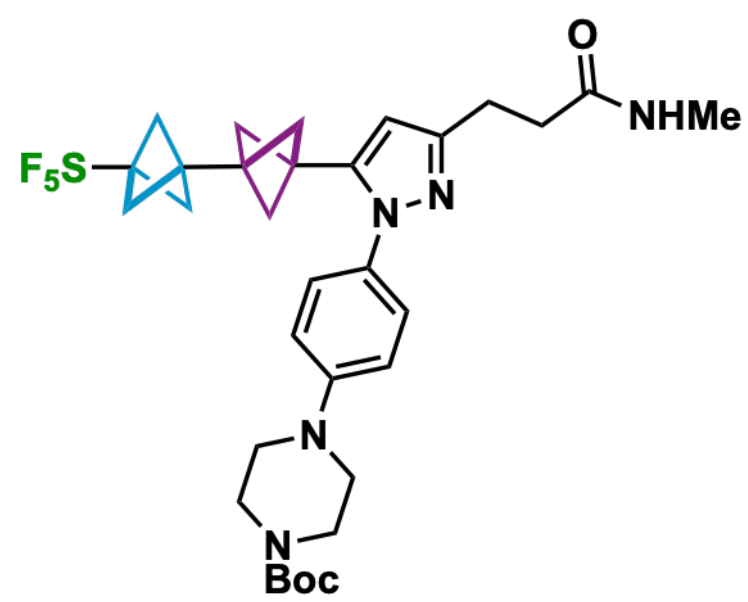

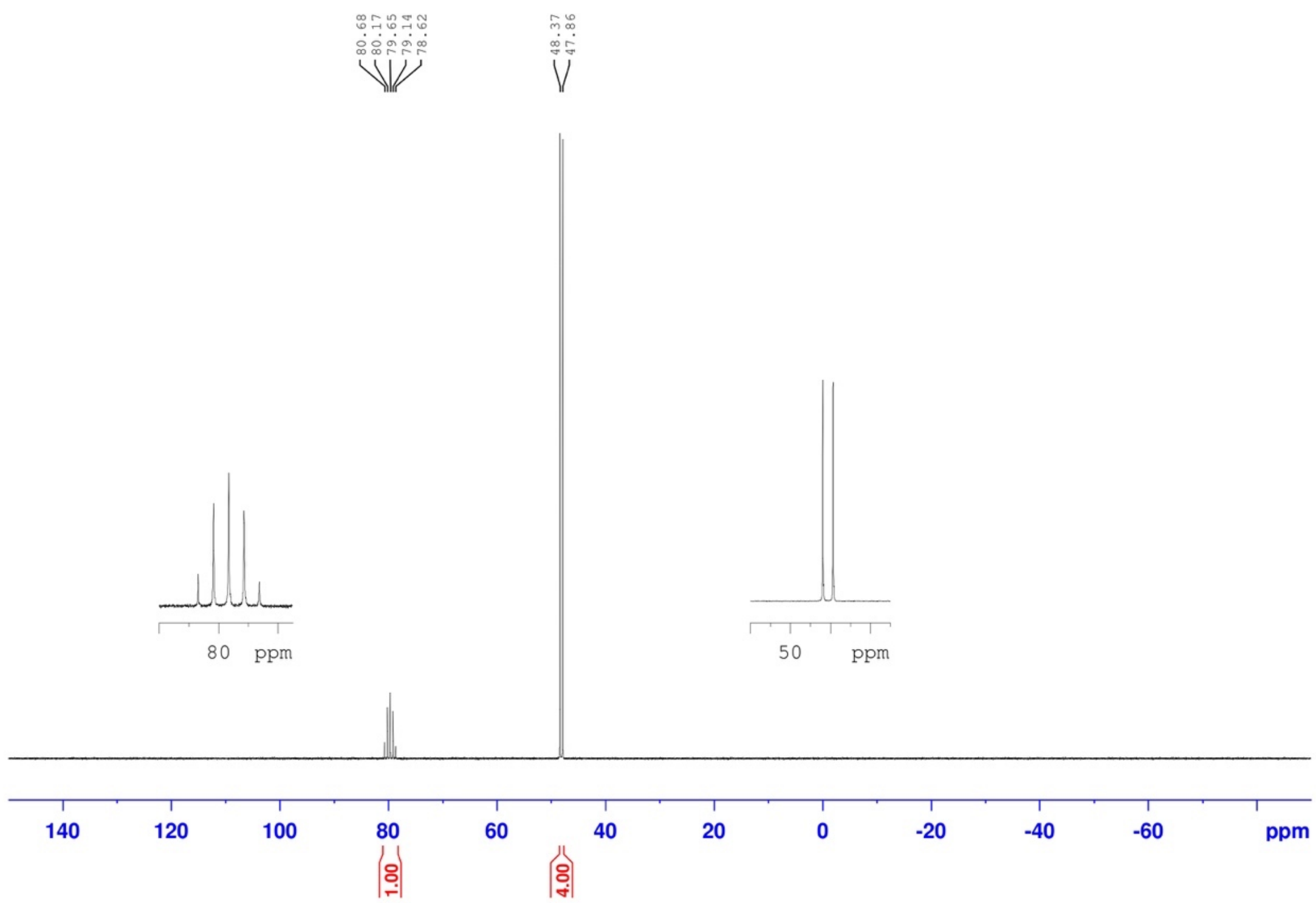

Figure S135. ^{19}F NMR spectrum of *tert*-butyl 4-(4-(3-(3-(methylamino)-3-oxopropyl)-5-(3'-(pentafluoro-λ[6]-sulfanyl)-[1,1'-bi(bicyclo[1.1.1] pentan)]-3-yl)-1*H*-pyrazol-1-yl)phenyl)piperazine-1-carboxylate (**S-18**) in $CDCl_3$.

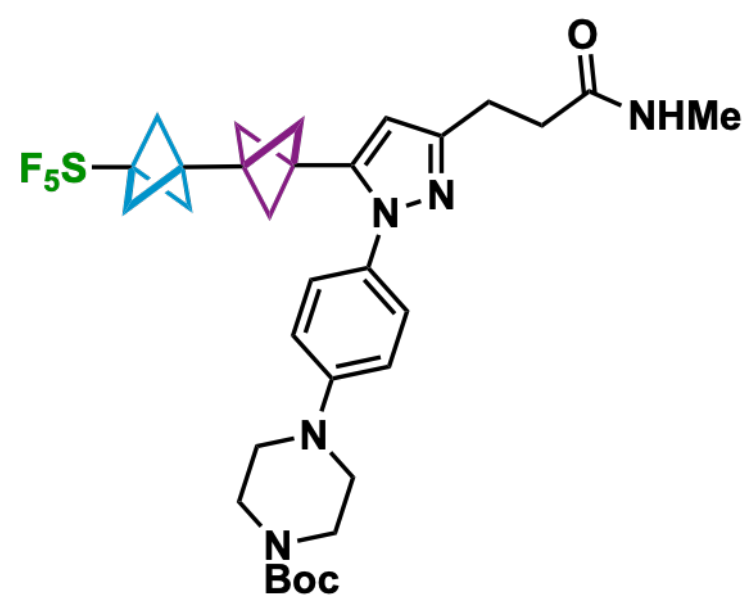

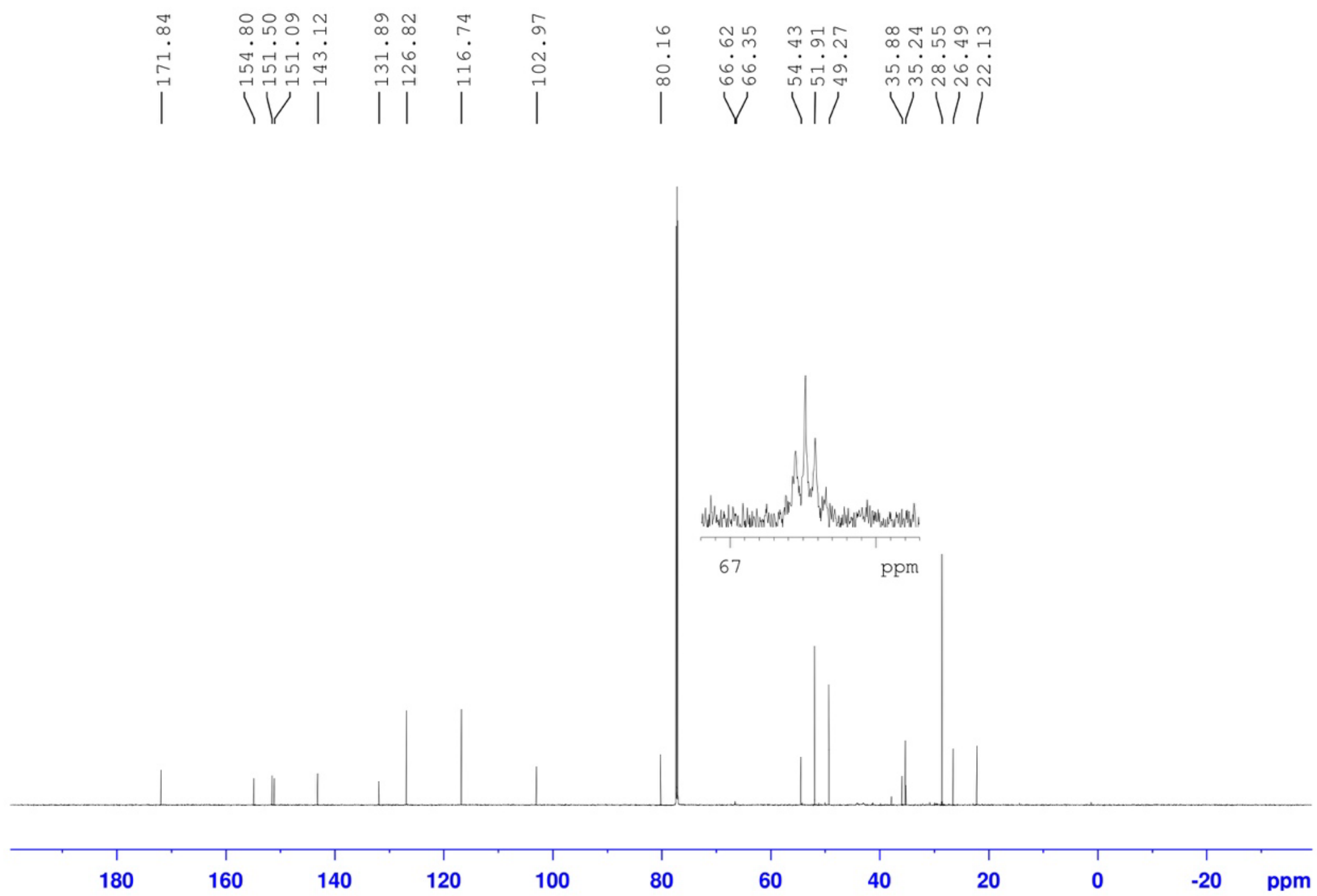

Figure S136. $^{13}C\{^{1}H\}$ NMR spectrum of *tert*-butyl 4-(4-(3-(3-(methylamino)-3-oxopropyl)-5-(3'-(pentafluoro-λ^6-sulfanyl)-[1,1'-bi(bicyclo[1.1.1] pentan)]-3-yl)-1*H*-pyrazol-1-yl)phenyl)piperazine-1-carboxylate (**S-18**) in $CDCl_3$.

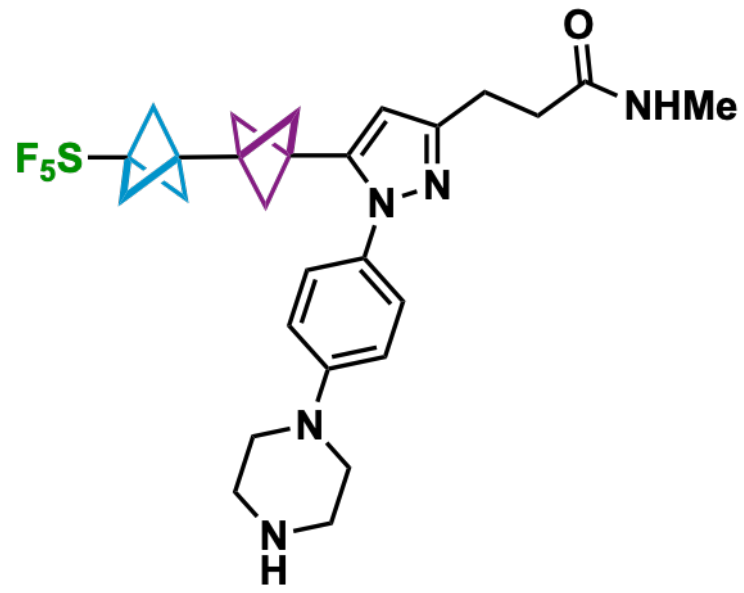

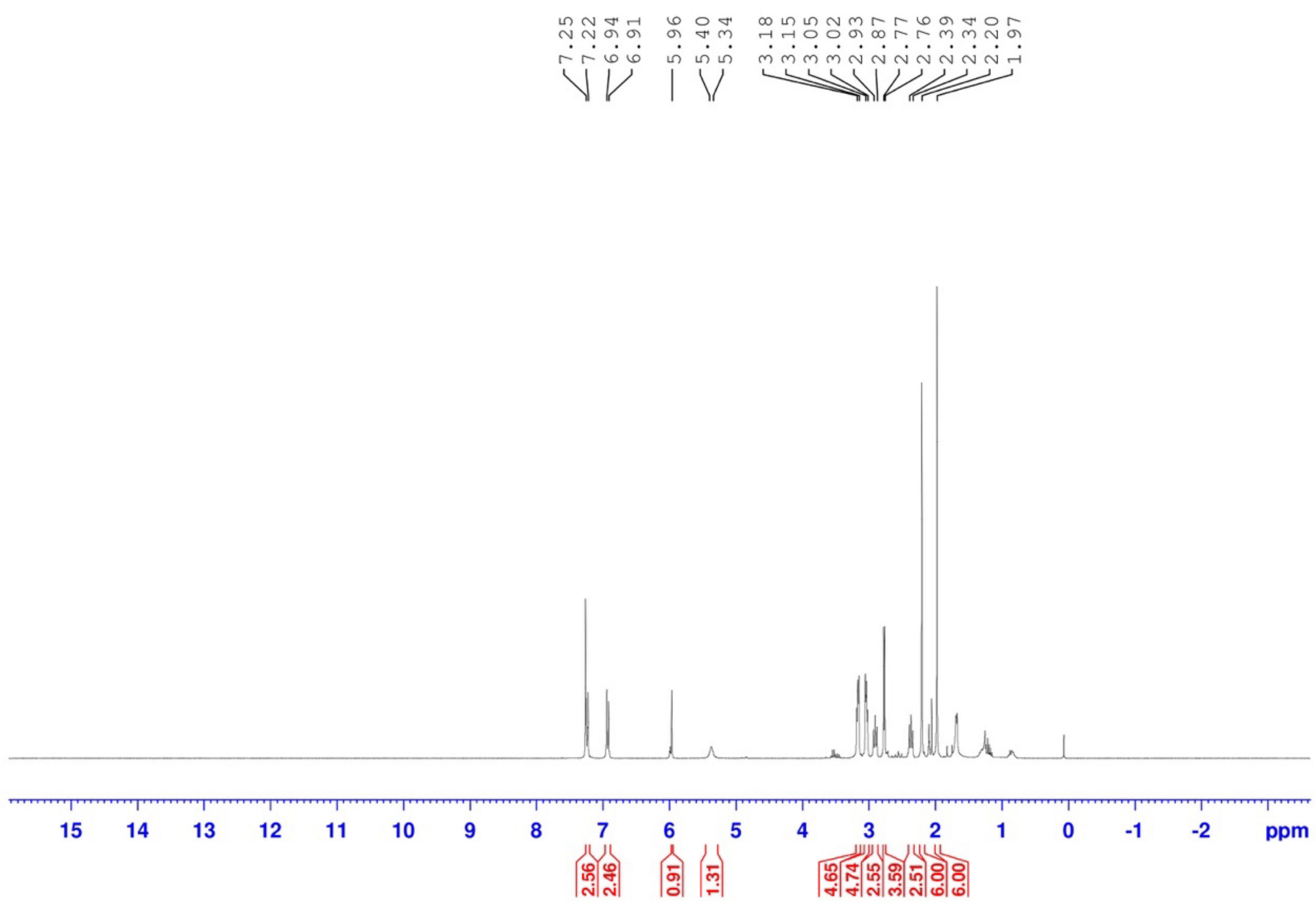

Figure S137. ^{1}H NMR spectrum of *N*-methyl-3-(5-(3'-(pentafluoro-λ[6]-sulfanyl)-[1,1'-bi(bicyclo[1.1.1]pentan)]-3-yl)-1-(4-(piperazin-1-yl)phenyl)-1*H*-pyrazol-3-yl)propenamide (**33**) in $CDCl_3$.

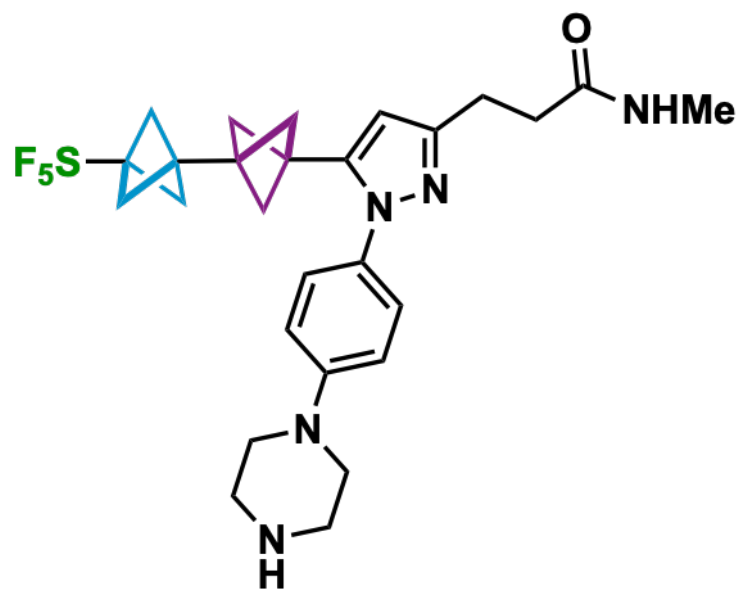

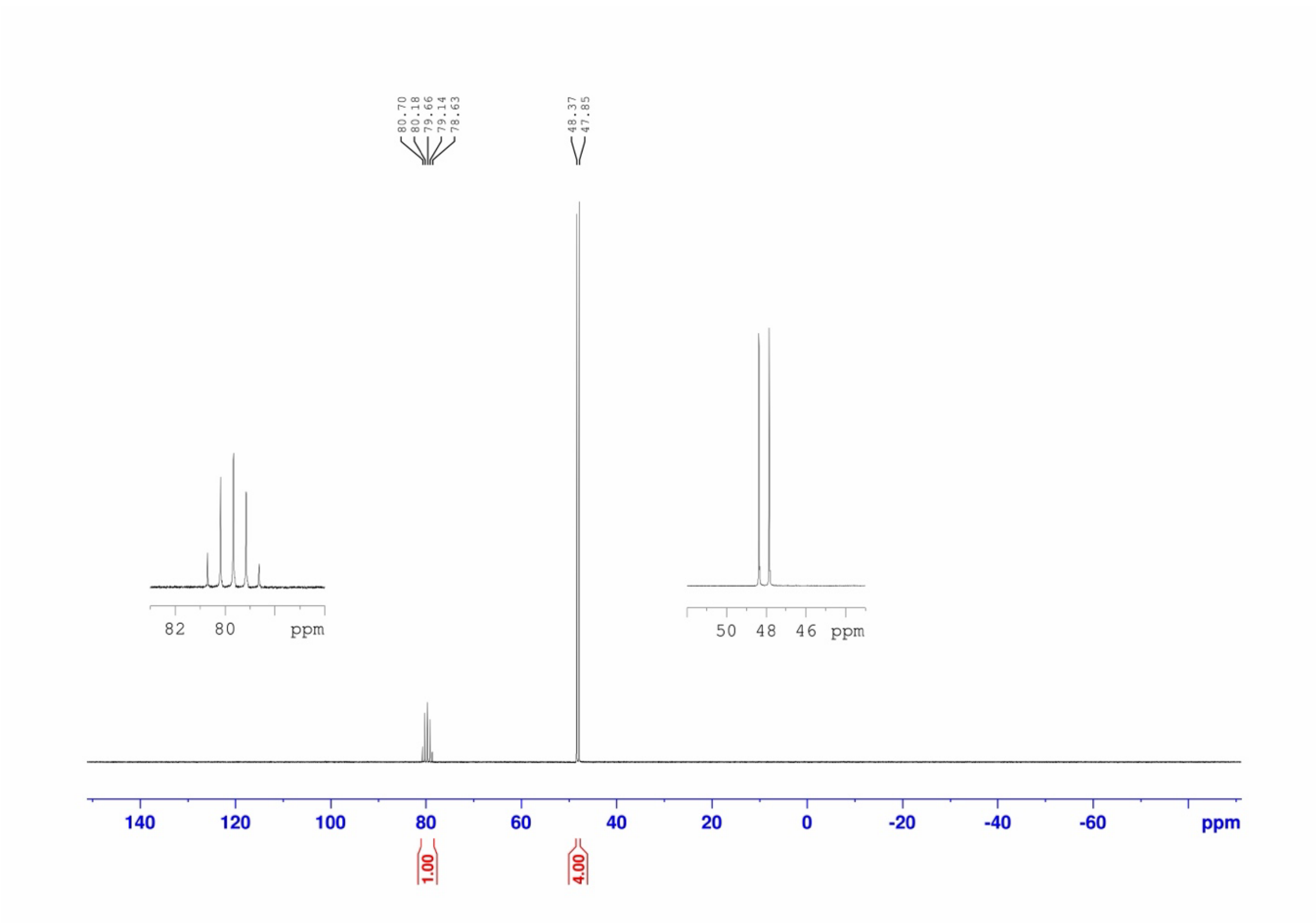

Figure S138. ^{19}F NMR spectrum of *N*-methyl-3-(5-(3'-(pentafluoro-λ^6-sulfanyl)-[1,1'-bi(bicyclo[1.1.1]pentan)]-3-yl)-1-(4-(piperazin-1-yl)phenyl)-1*H*-pyrazol-3-yl)propenamide (**33**) in $CDCl_3$.

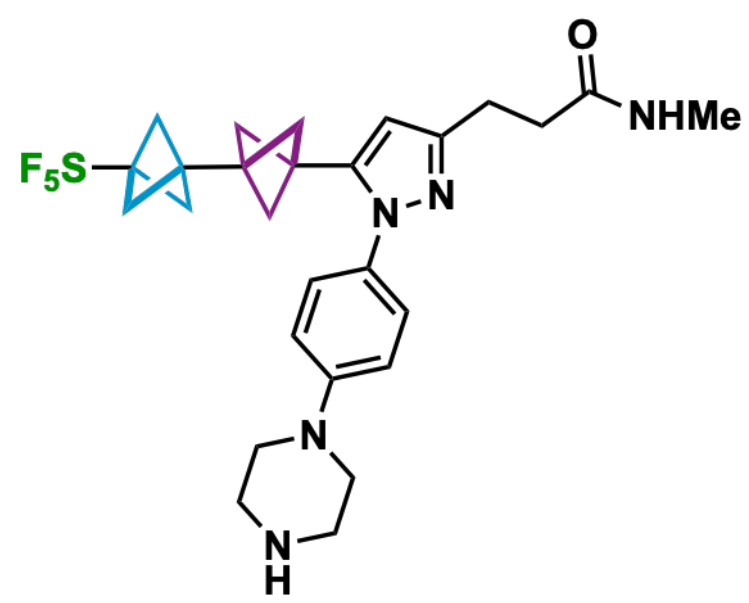

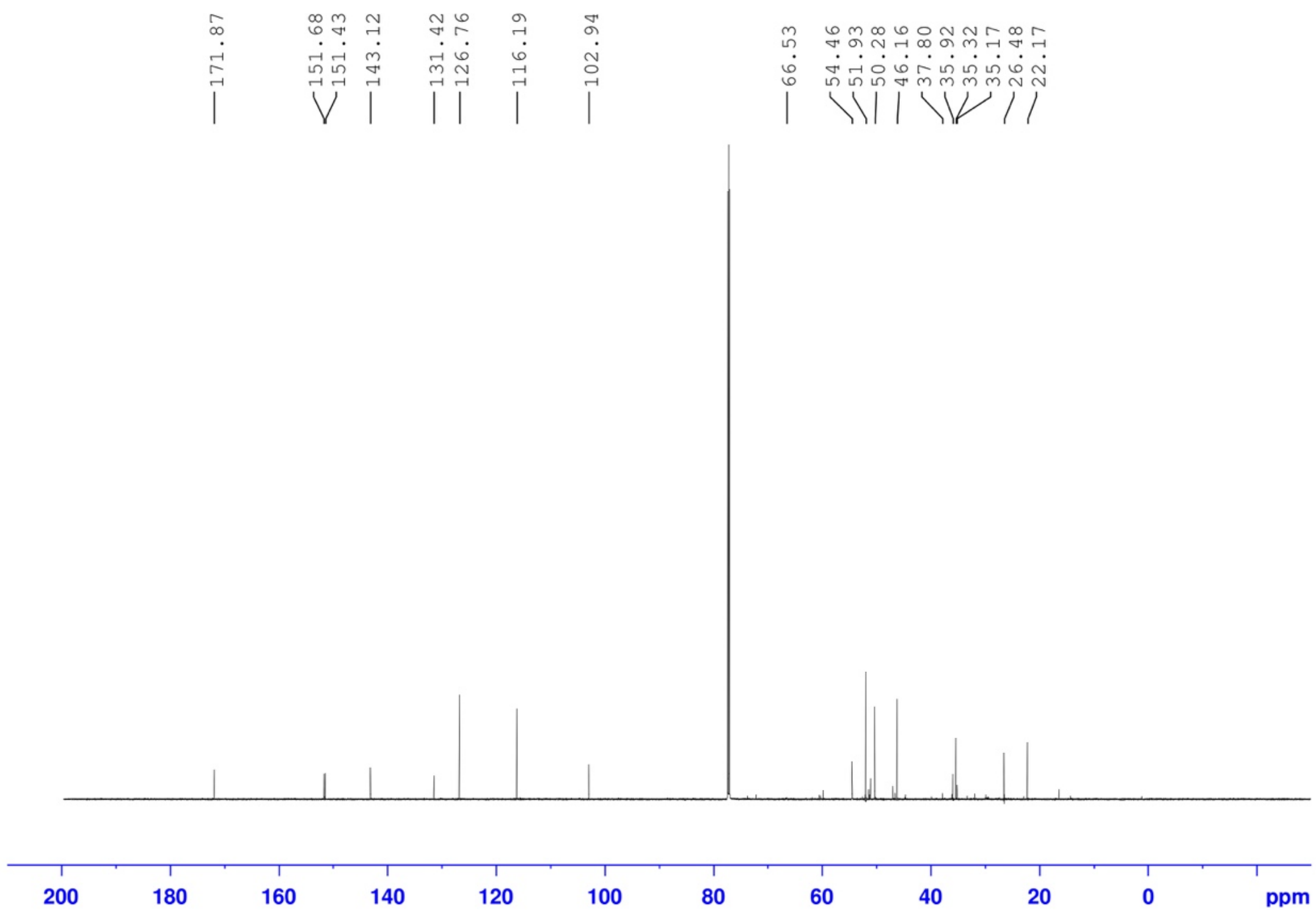

Figure S139. $^{13}C\{^{1}H\}$ NMR spectrum of *N*-methyl-3-(5-(3'-(pentafluoro-λ^{6}-sulfanyl)-[1,1'-bi(bicyclo[1.1.1]pentan)]-3-yl)-1-(4-(piperazin-1-yl)phenyl)-1*H*-pyrazol-3-yl)propenamide (**33**) in $CDCl_3$.

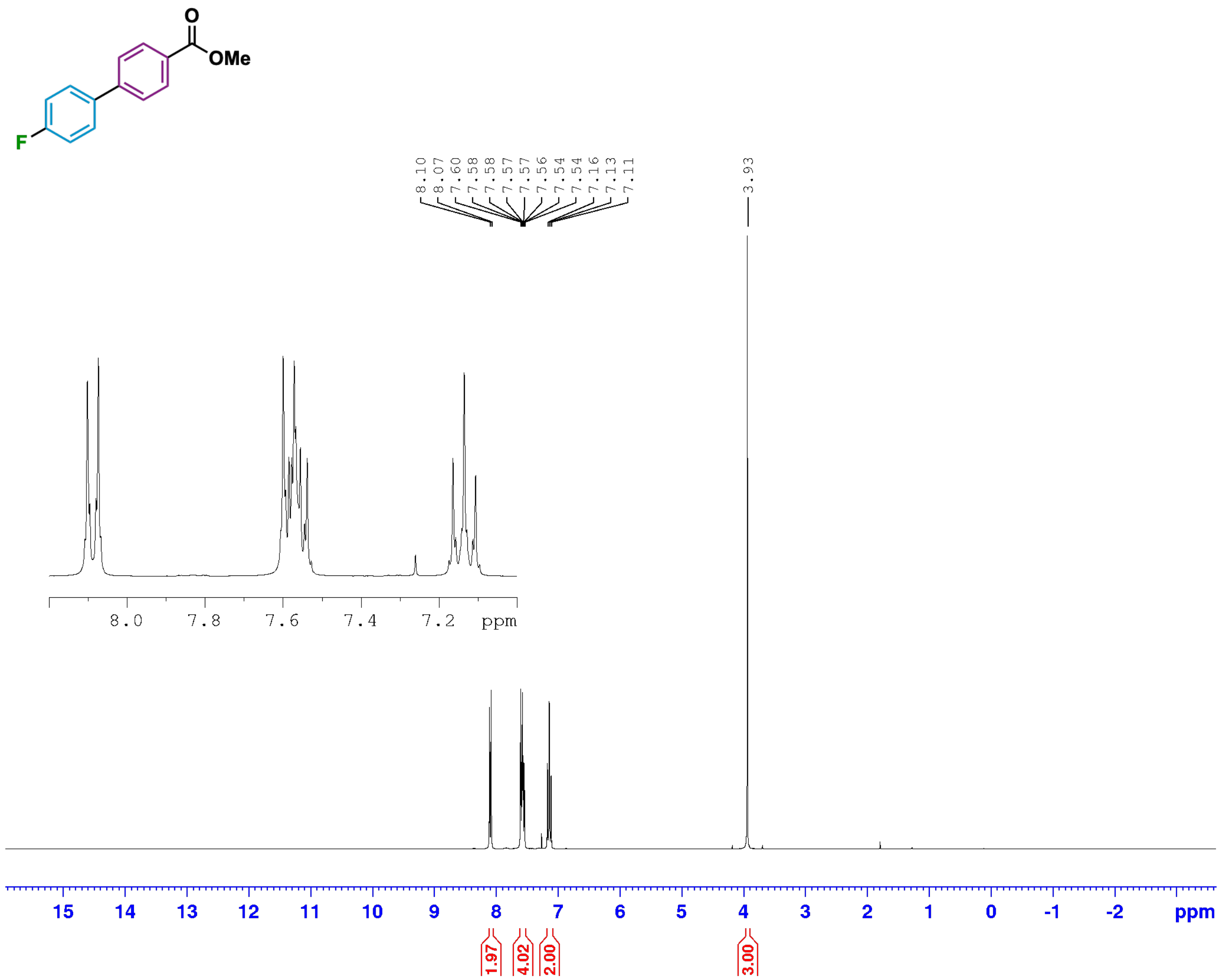

Figure S140. ^{1}H NMR spectrum of methyl 4'-fluoro-[1,1'-biphenyl]-4-carboxylate (**S-19**) in $CDCl_3$.

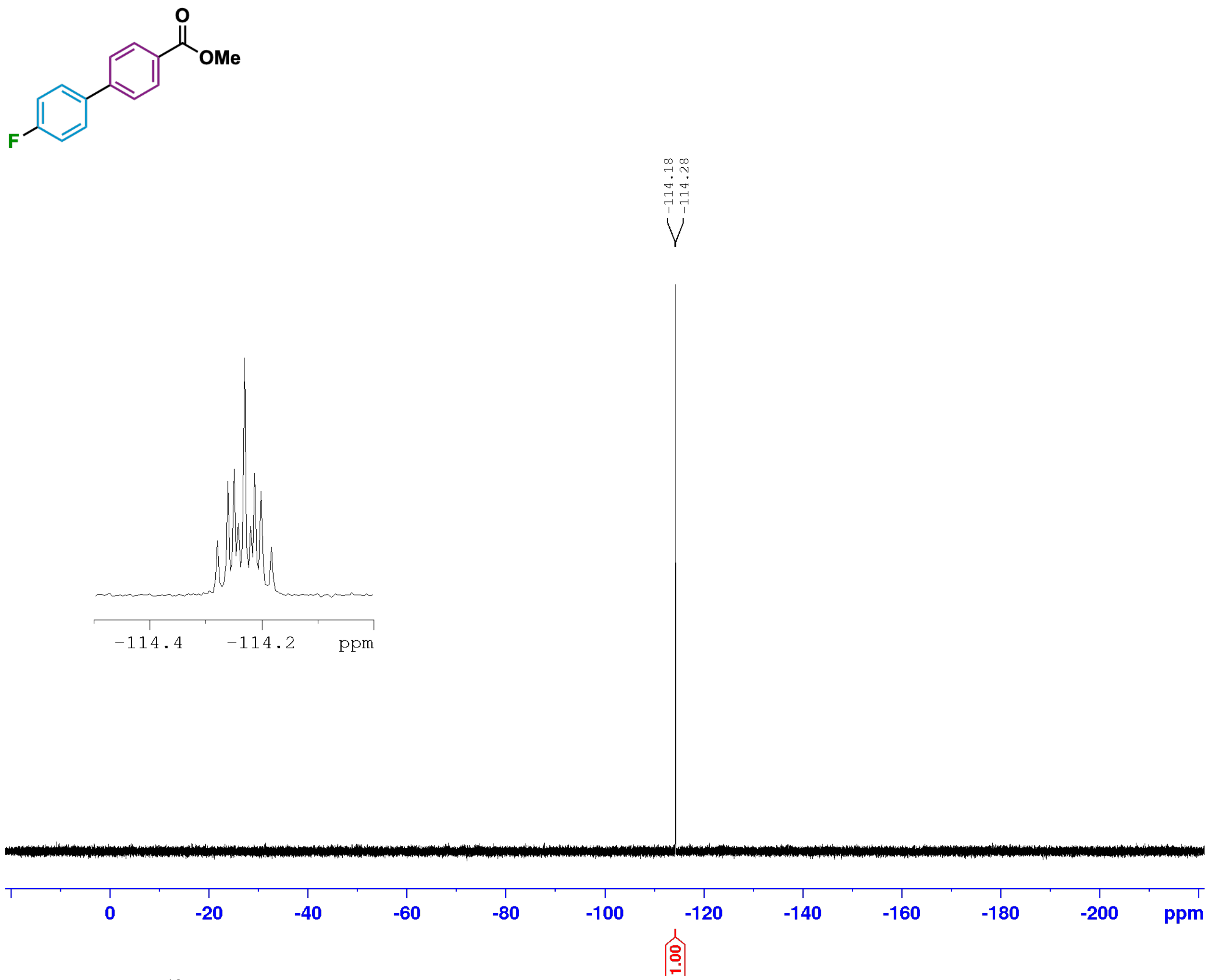

Figure S141. ^{19}F NMR spectrum of methyl 4'-fluoro-[1,1'-biphenyl]-4-carboxylate (**S-19**) in $CDCl_3$.

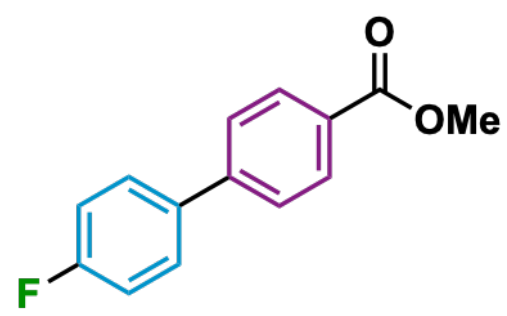

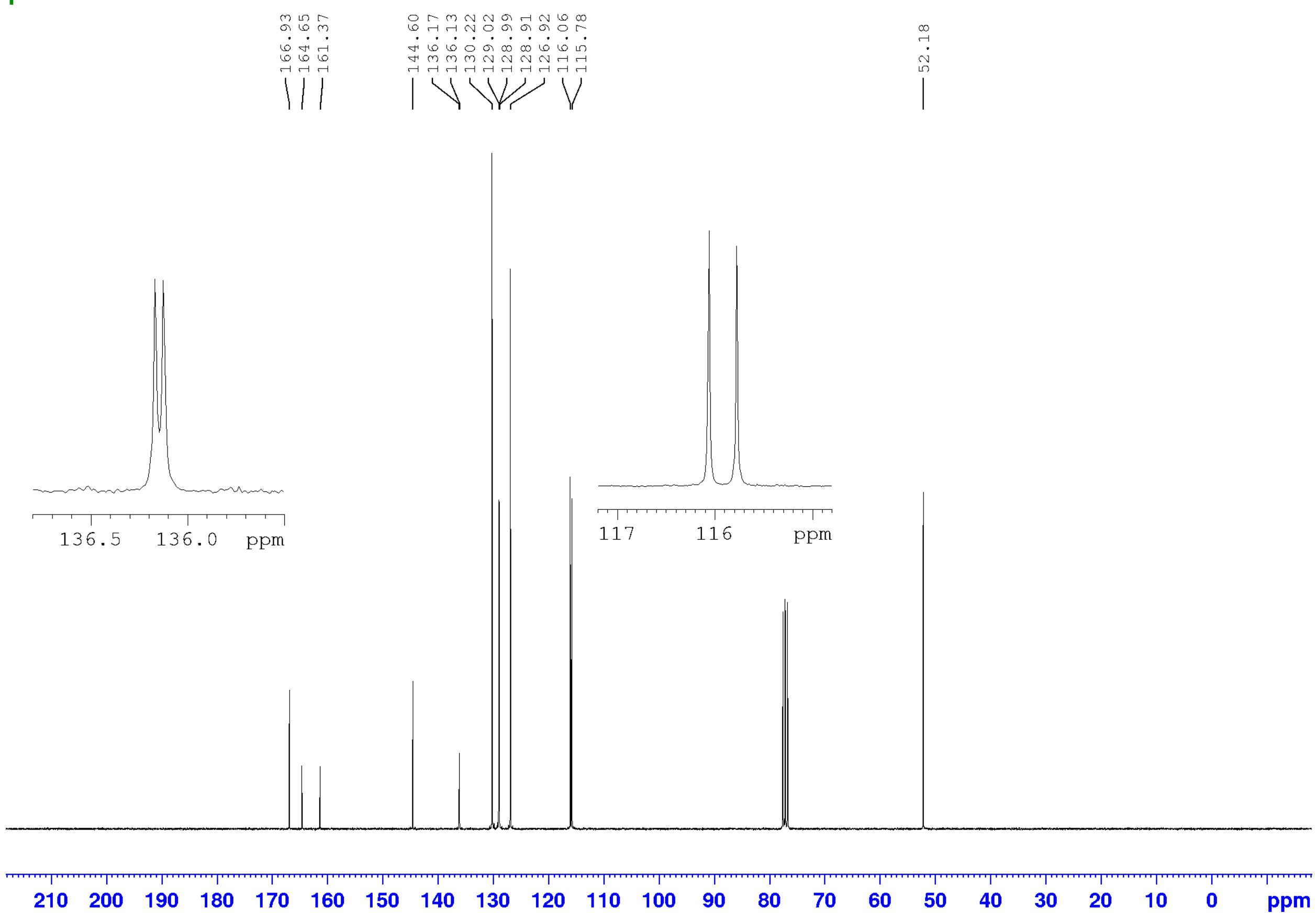

Figure S142. $^{13}C\{^1H\}$ NMR spectrum of methyl 4'-fluoro-[1,1'-biphenyl]-4-carboxylate (**S-19**) in $CDCl_3$.

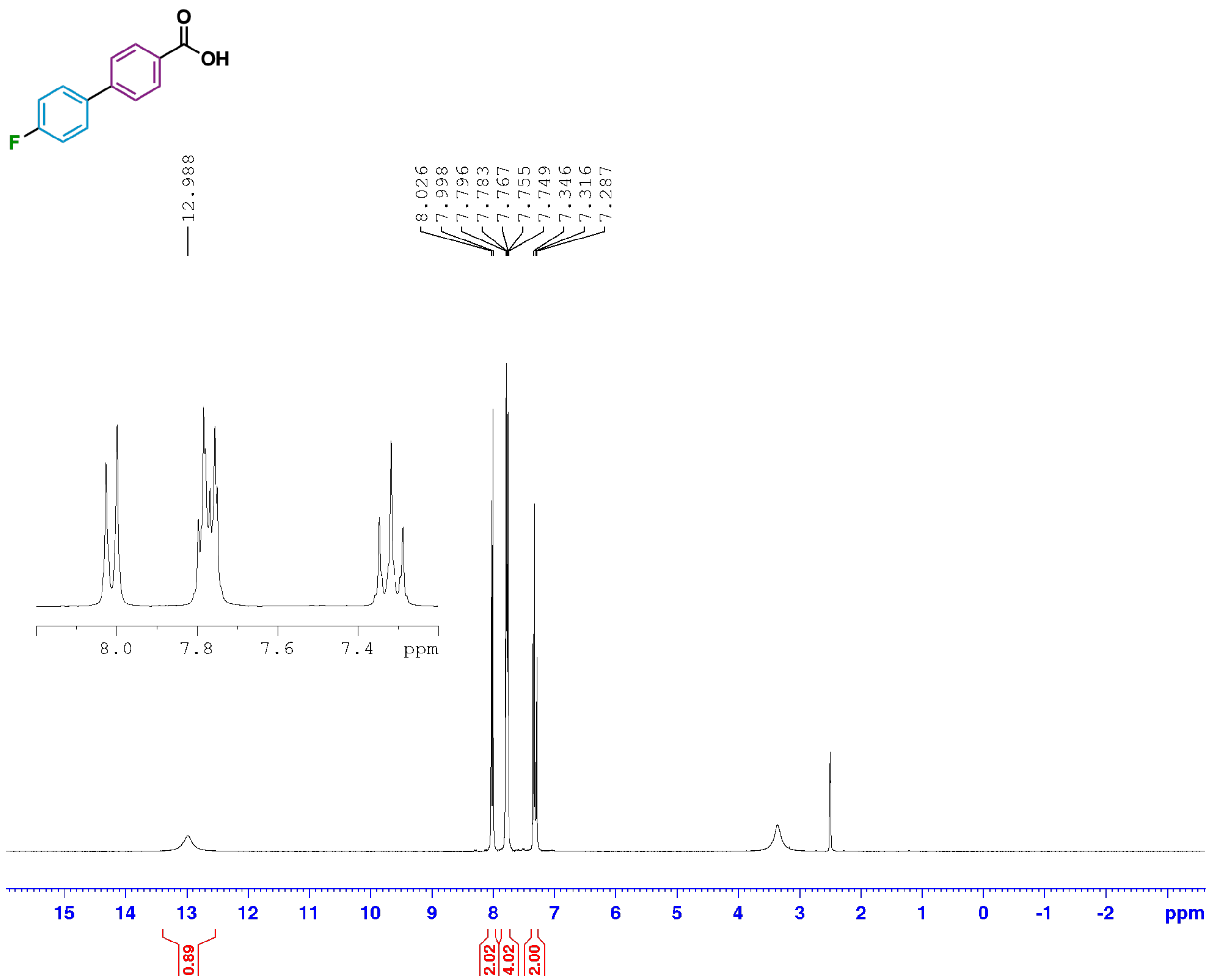

Figure S143. ^{1}H NMR spectrum of 4'-fluoro-[1,1'-biphenyl]-4-carboxylic acid (**34**) in DMSO-d_6.

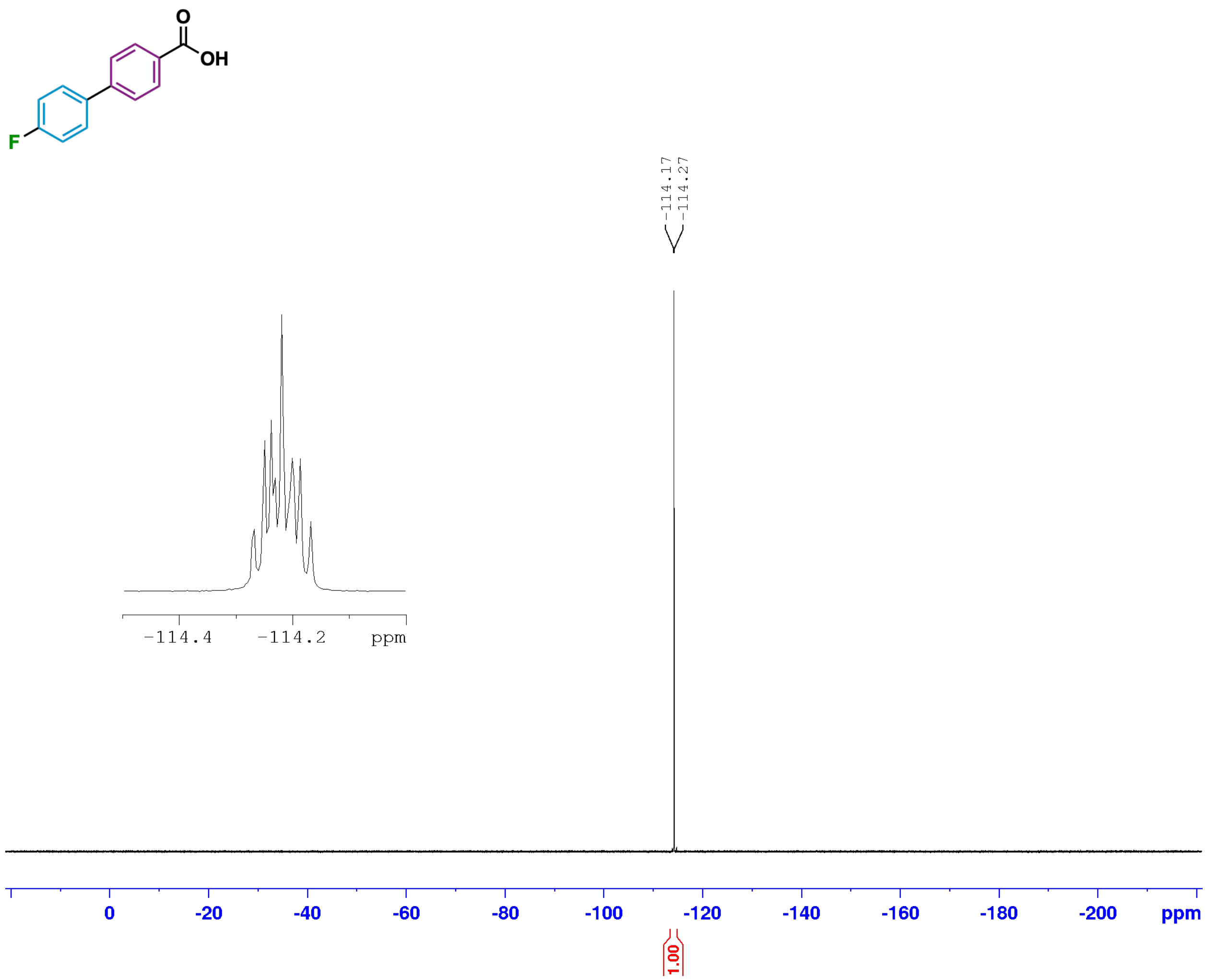

Figure S144. ^{19}F NMR spectrum of 4'-fluoro-[1,1'-biphenyl]-4-carboxylic acid (**34**) in DMSO-d_6.

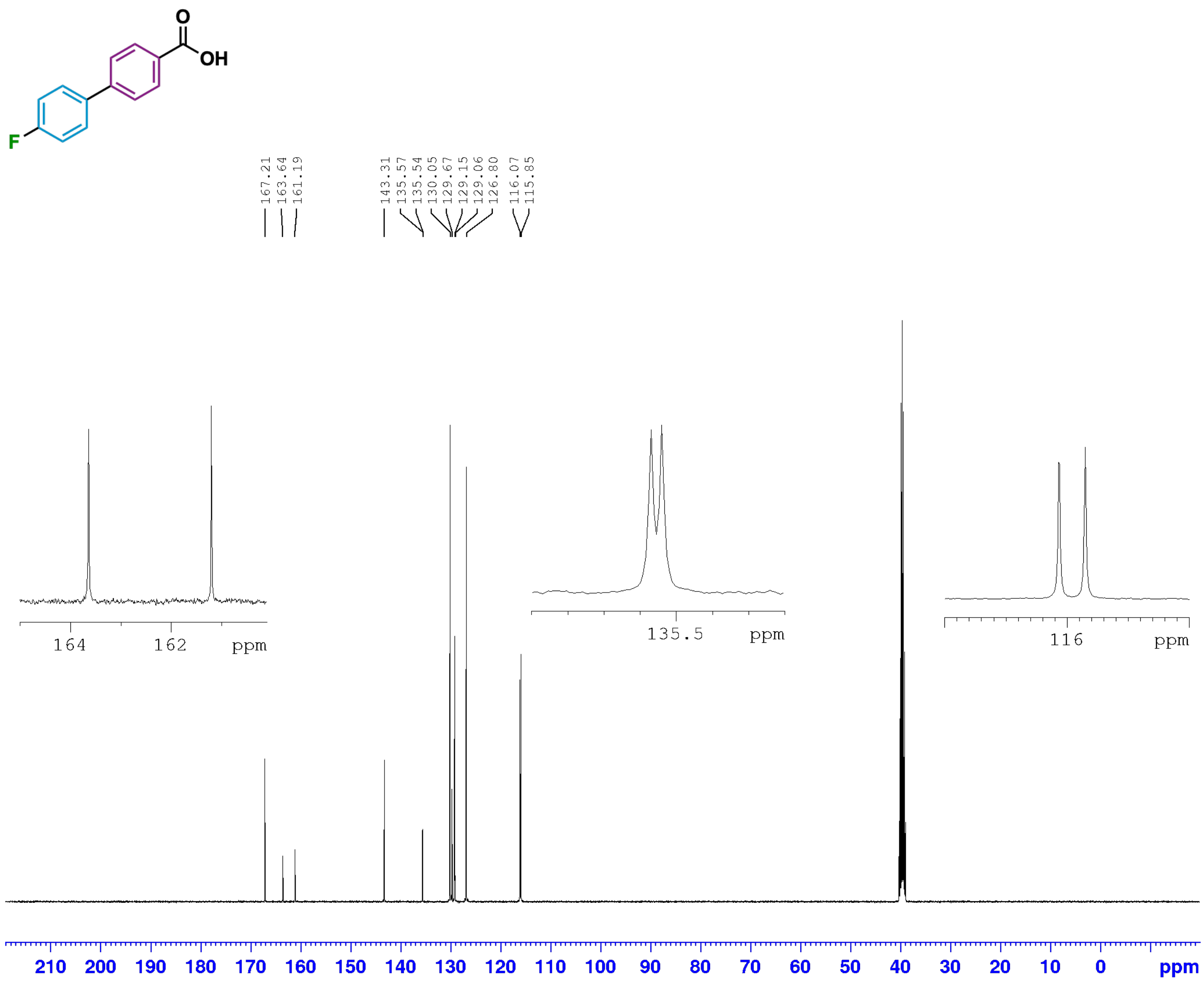

Figure S145. $^{13}C\{^{1}H\}$ NMR spectrum of 4'-fluoro-[1,1'-biphenyl]-4-carboxylic acid (**34**) in DMSO-*d*$_6$.

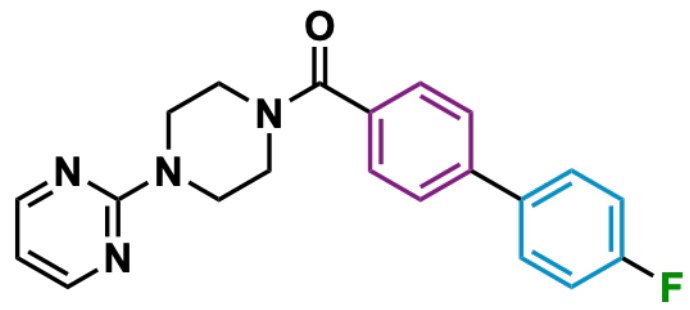

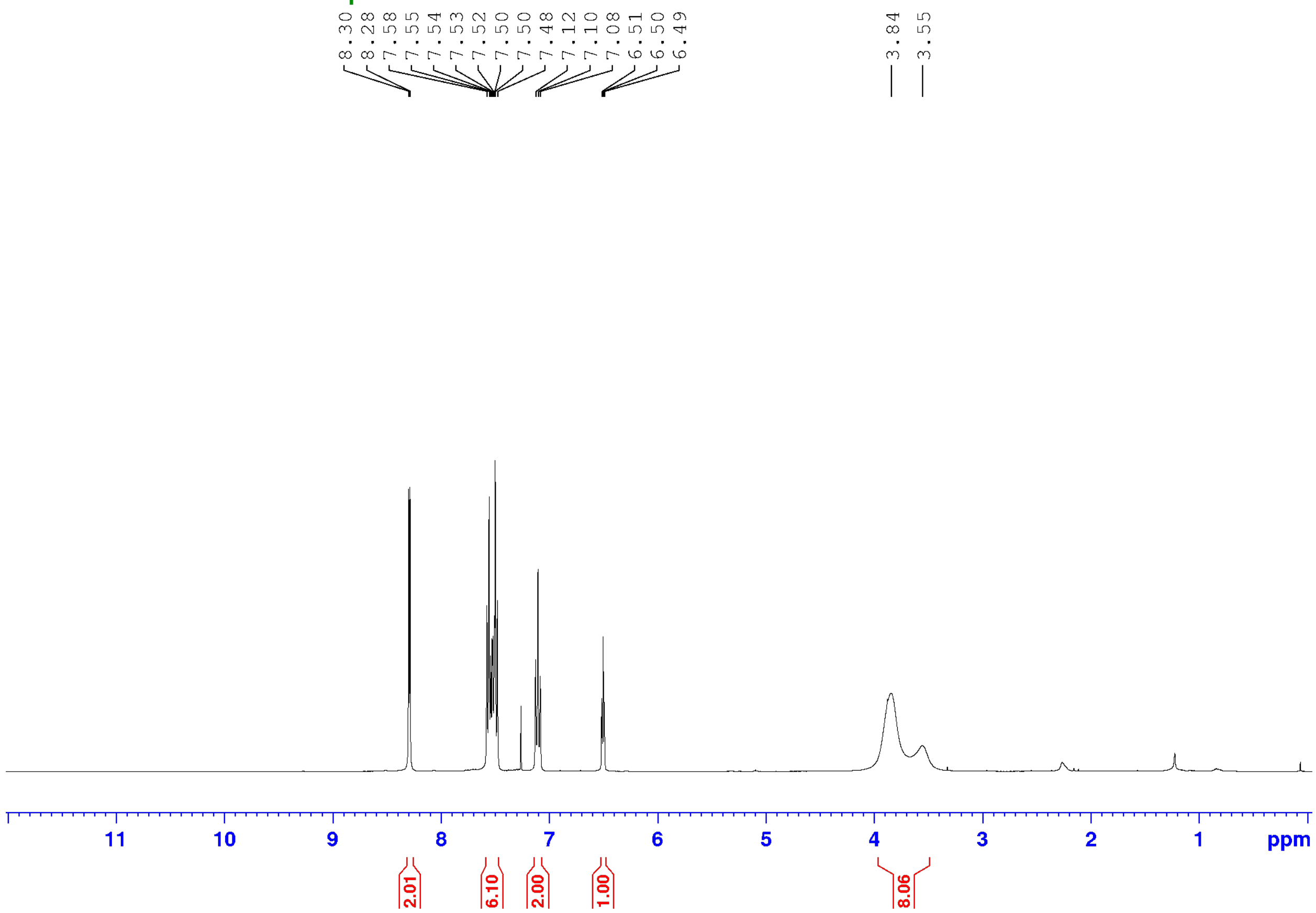

Figure S146. ^{1}H NMR spectrum of (4'-fluoro-[1,1'-biphenyl]-4-yl)(4-(pyrimidin-2-yl)piperazin-1-yl)methanone (**37**) in $CDCl_3$.

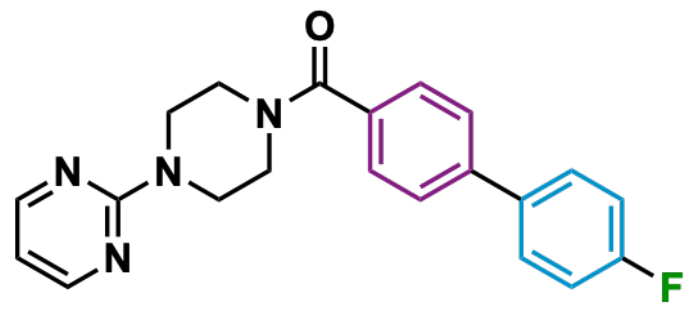

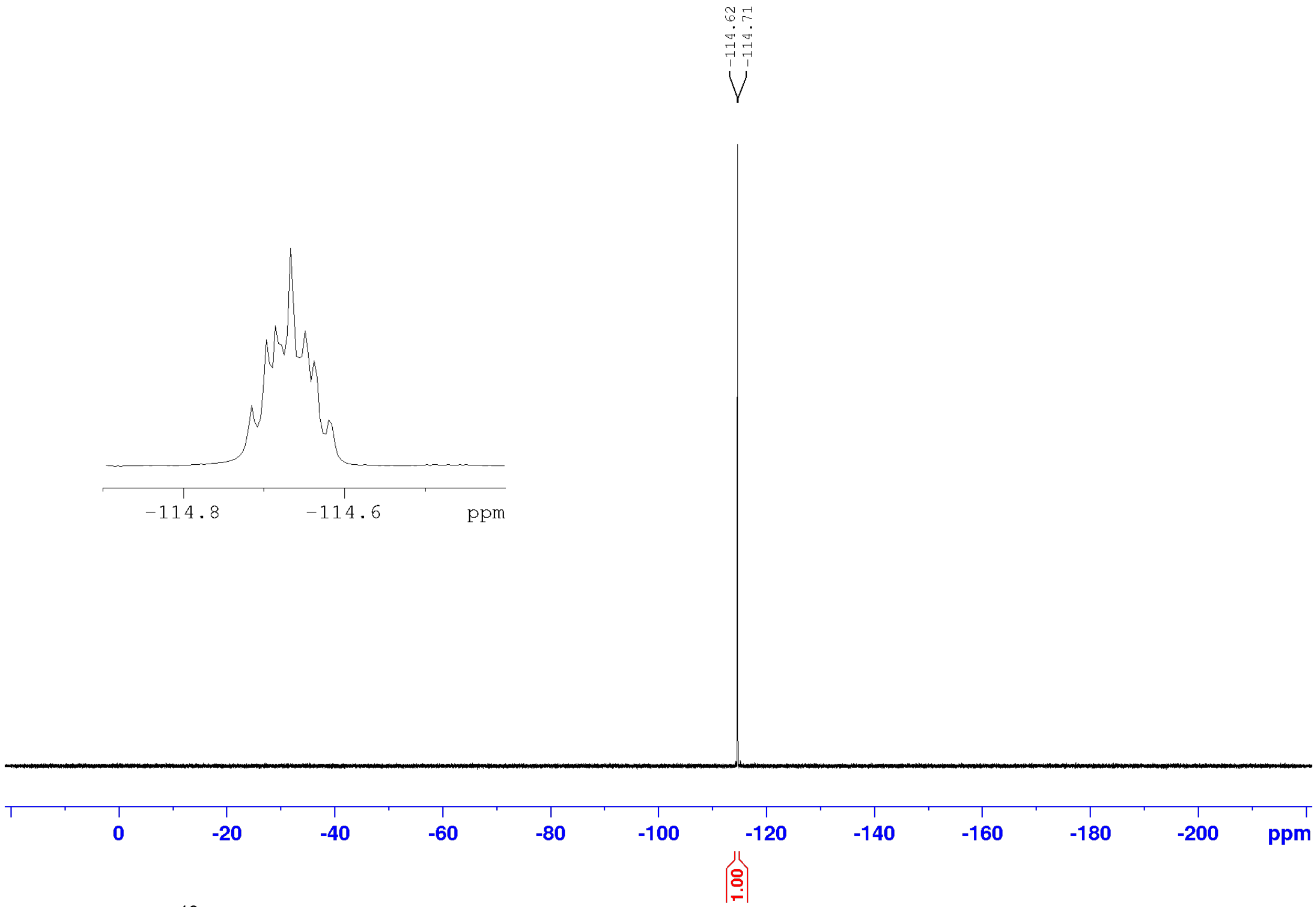

Figure S147. ^{19}F NMR spectrum of (4'-fluoro-[1,1'-biphenyl]-4-yl)(4-(pyrimidin-2-yl)piperazin-1-yl)methanone (**37**) in $CDCl_3$.

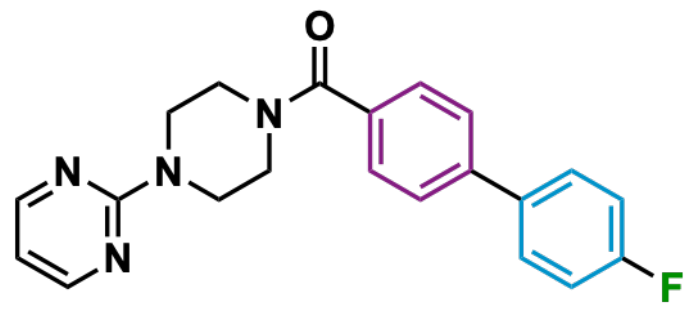

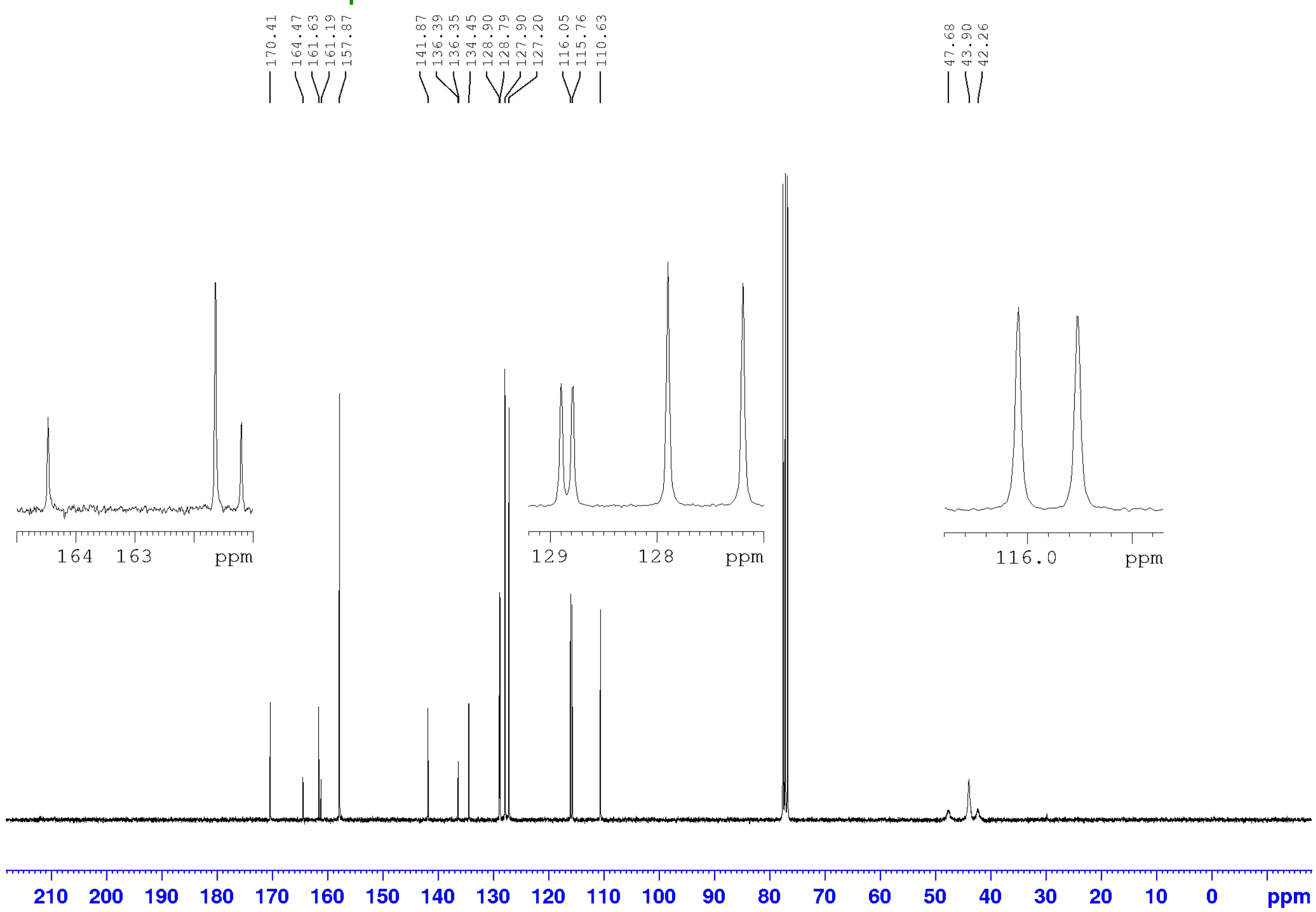

Figure S148. $^{13}C\{^{1}H\}$ NMR spectrum of (4'-fluoro-[1,1'-biphenyl]-4-yl)(4-(pyrimidin-2-yl)piperazin-1-yl)methanone (**37**) in $CDCl_3$.

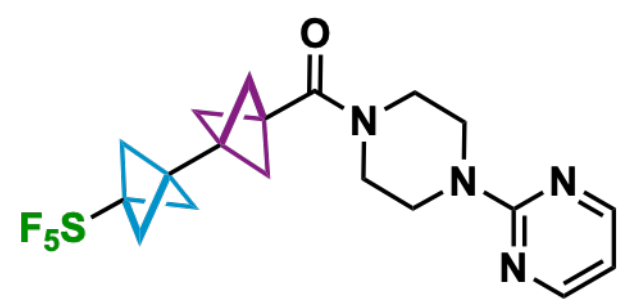

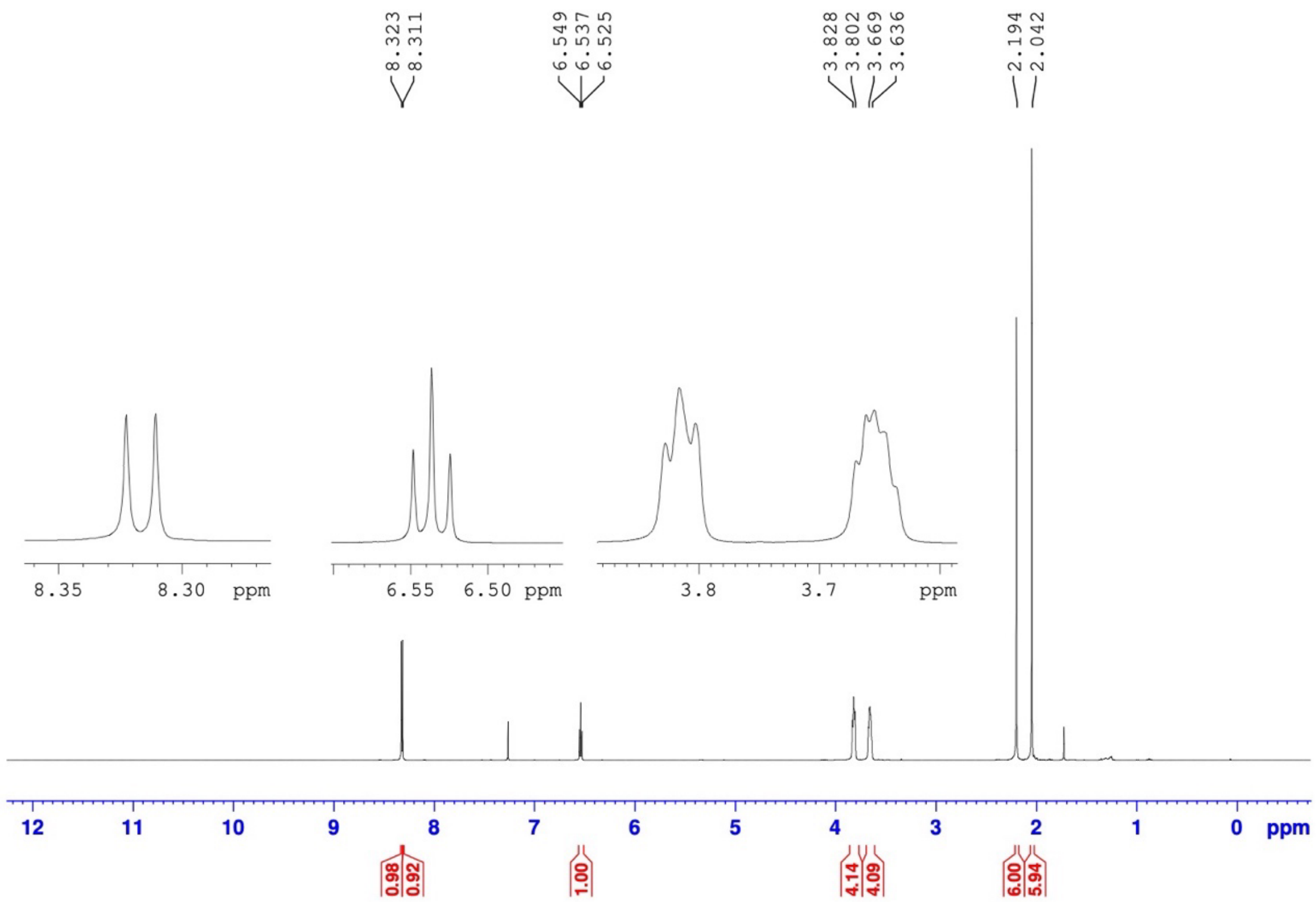

Figure S149. ^{1}H NMR spectrum of ((3'-(pentafluoro-λ[6]-sulfaneyl)-[1,1'-bi(bicyclo[1.1.1]pentan)]-3-yl)(4-(pyrimidin-2-yl)piperazin-1-yl)methanone (**38**) in $CDCl_3$.

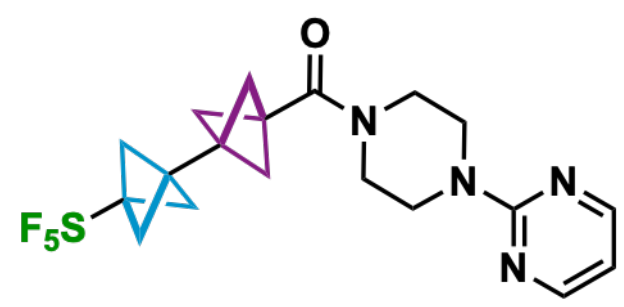

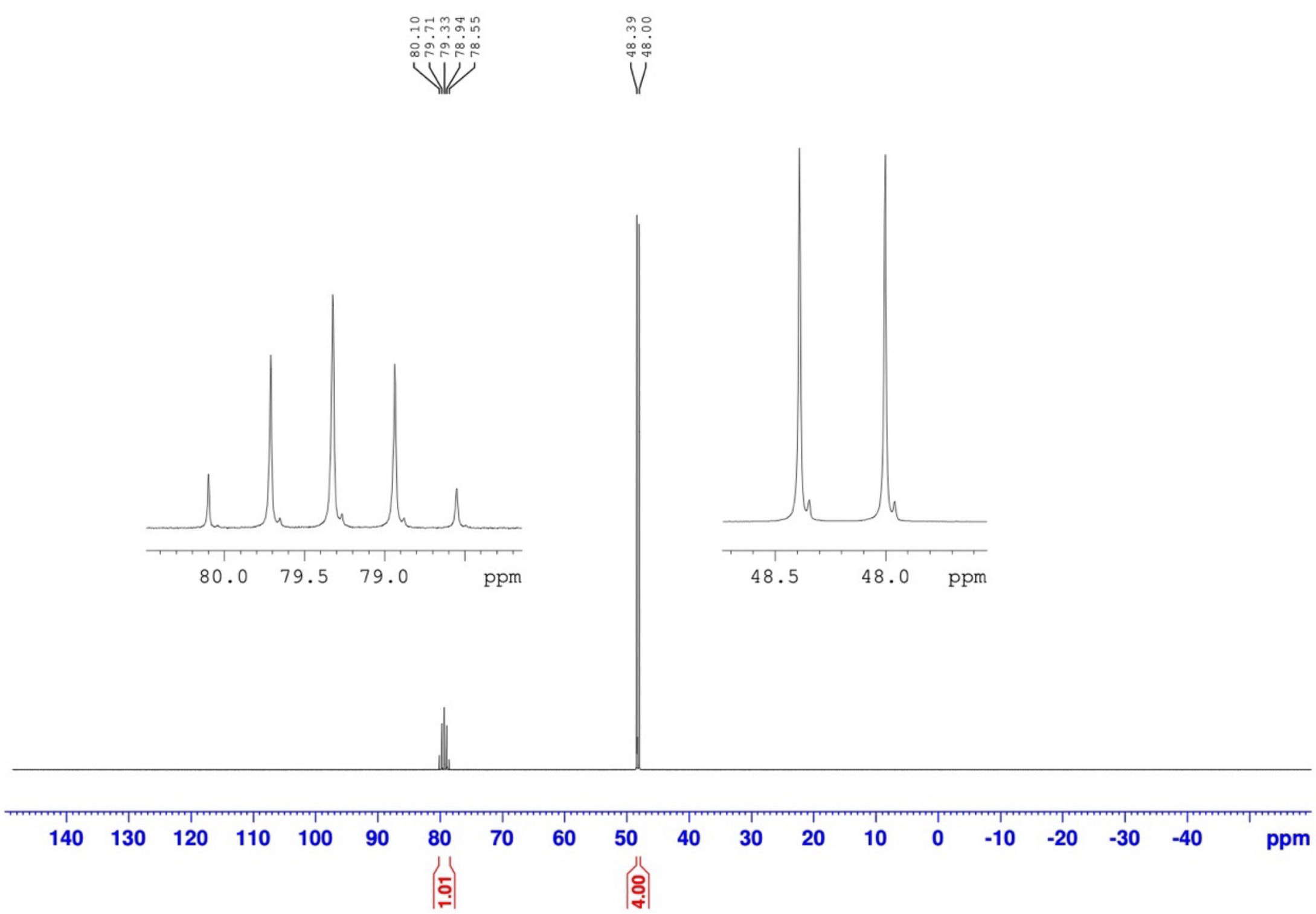

Figure S150. ^{19}F NMR spectrum of ((3'-(pentafluoro-λ^6-sulfaneyl)-[1,1'-bi(bicyclo[1.1.1]pentan)]-3-yl)(4-(pyrimidin-2-yl)piperazin-1-yl)methanone (**38**) in $CDCl_3$.

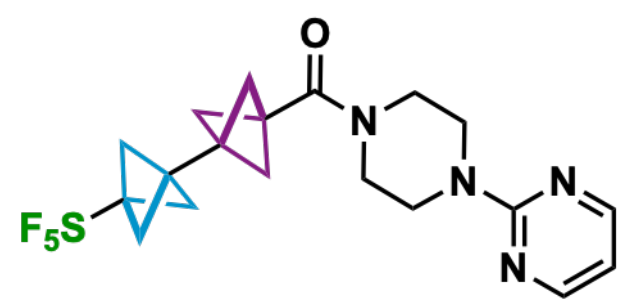

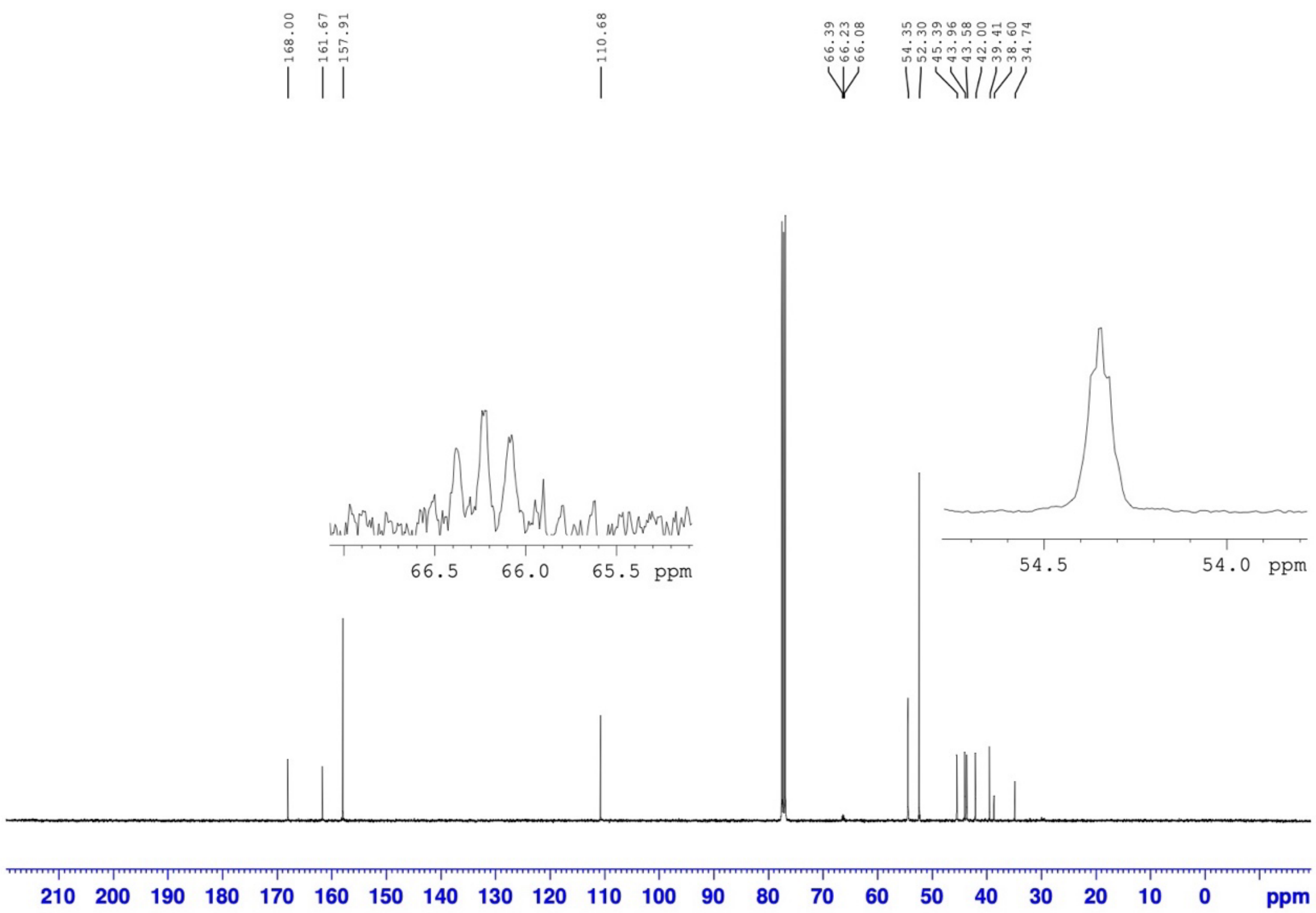

Figure S151. $^{13}C\{^1H\}$ NMR spectrum of ((3'-(pentafluoro-λ^6-sulfaneyl)-[1,1'-bi(bicyclo[1.1.1]pentan)]-3-yl)(4-(pyrimidin-2-yl)piperazin-1-yl)methanone (**38**) in $CDCl_3$.

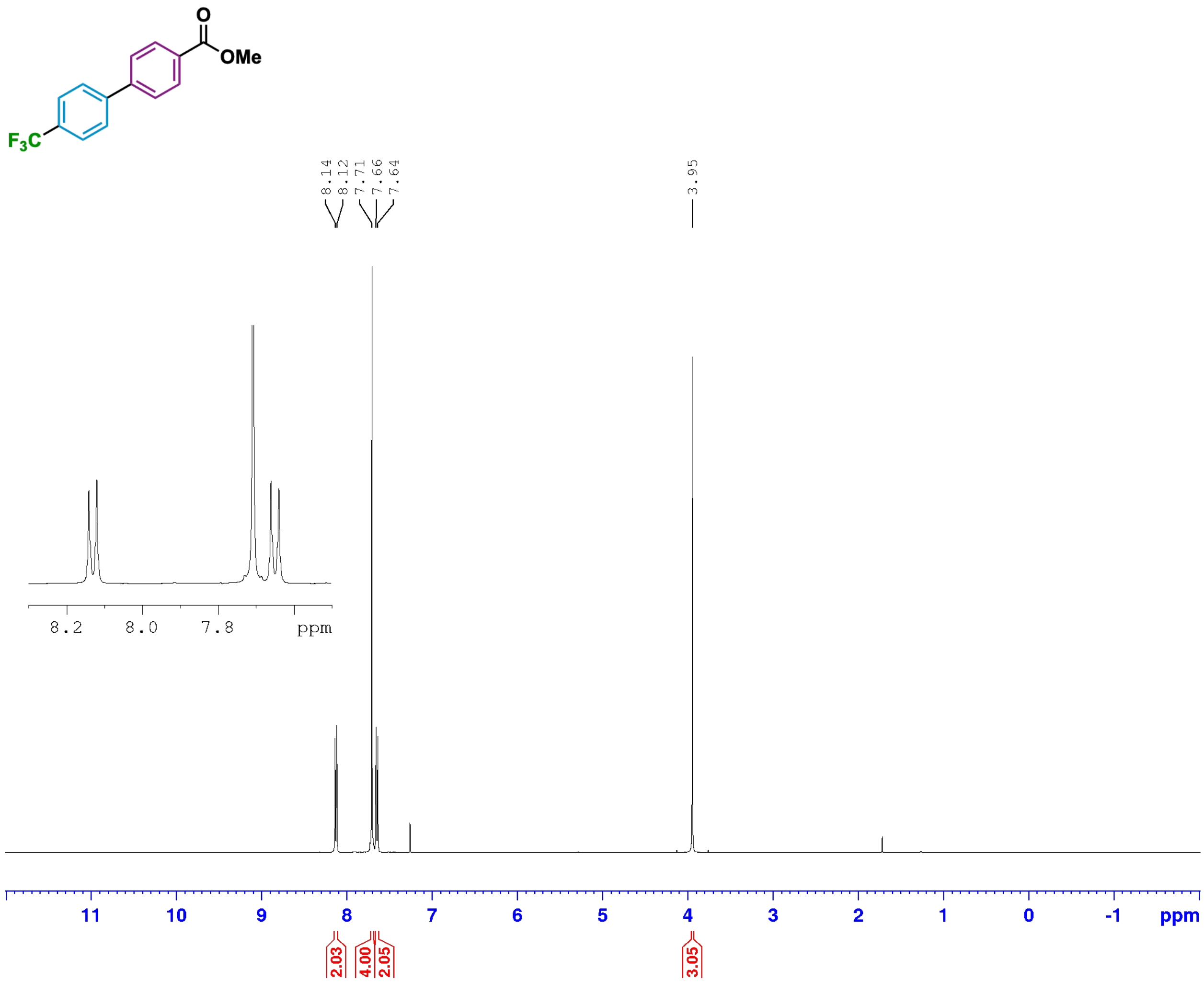

Figure S152. ^{1}H NMR spectrum of methyl 4'-(trifluoromethyl)-[1,1'-biphenyl]-4-carboxylate (**S-20**) in $CDCl_3$.

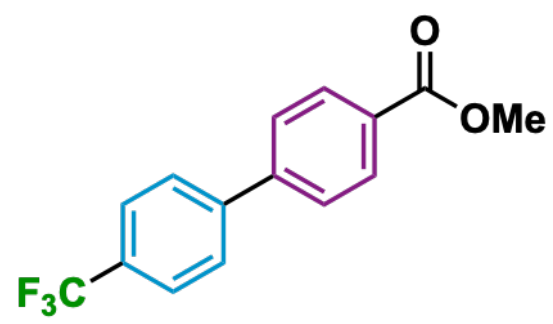

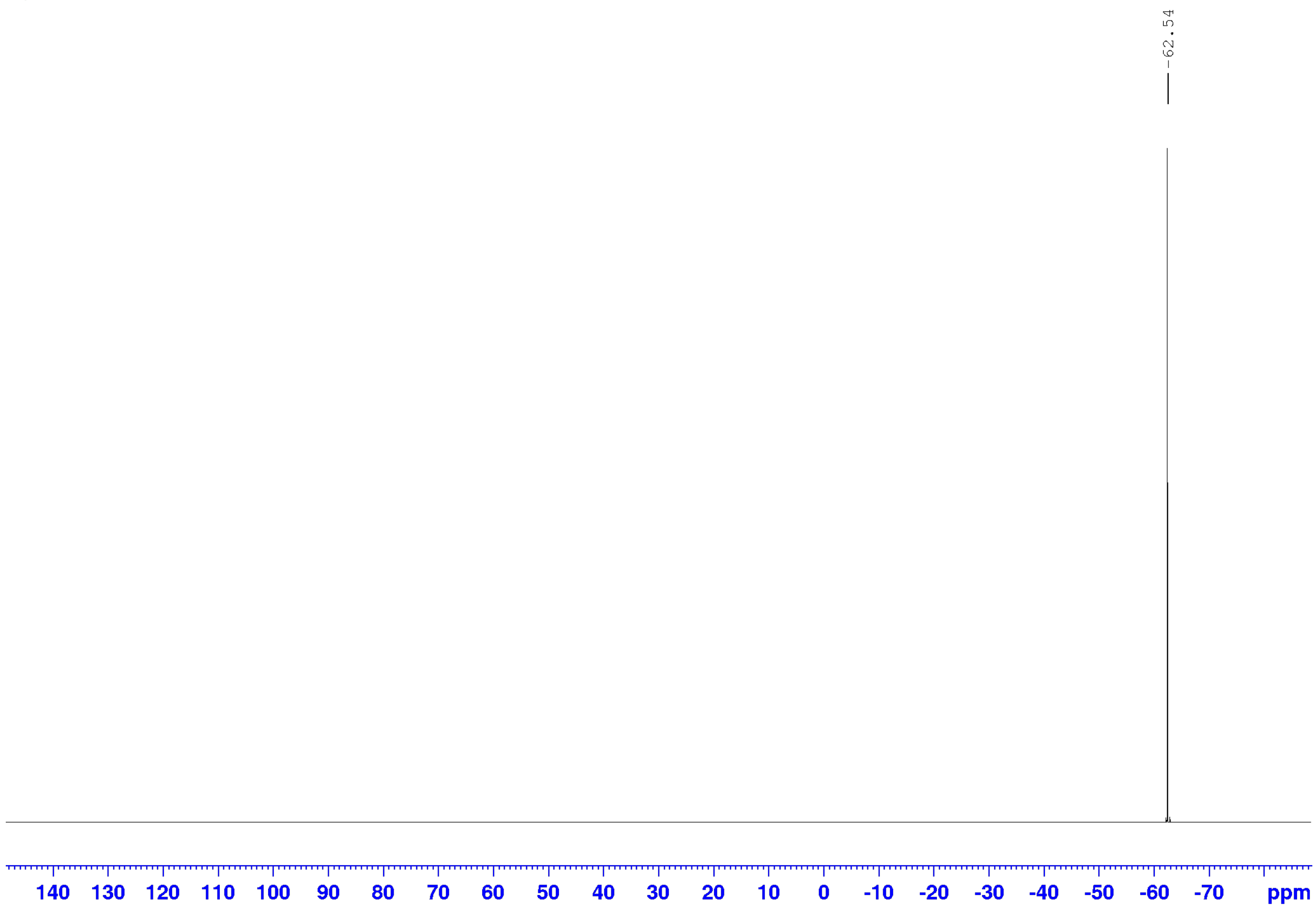

Figure S153. ^{19}F NMR spectrum of methyl 4'-(trifluoromethyl)-[1,1'-biphenyl]-4-carboxylate (**S-20**) in $CDCl_3$.

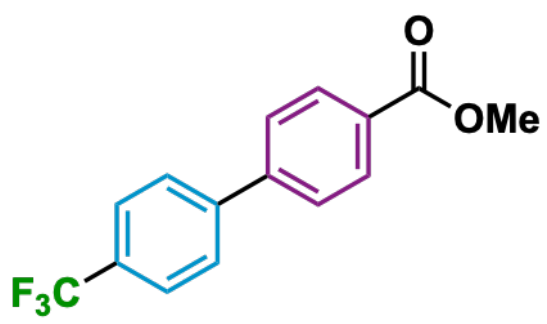

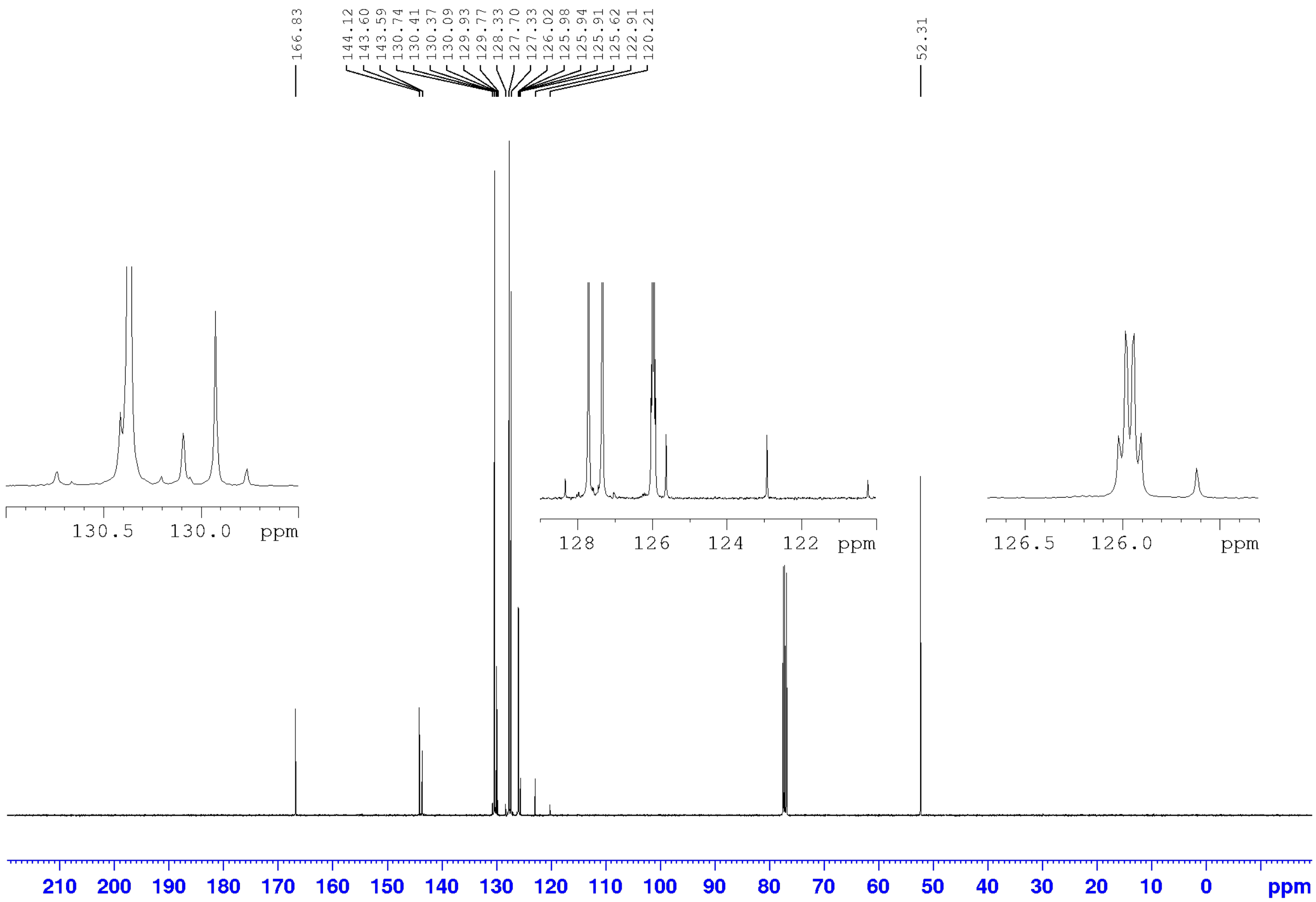

Figure S154. $^{13}C\{^{1}H\}$ NMR spectrum of methyl 4'-(trifluoromethyl)-[1,1'-biphenyl]-4-carboxylate (**S-20**) in $CDCl_3$.

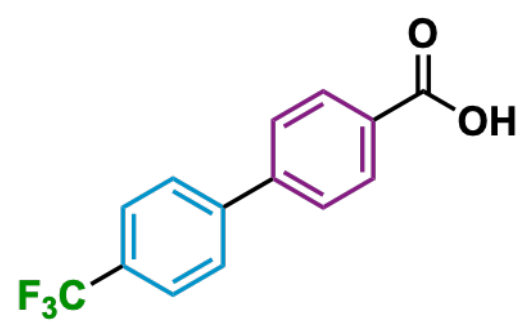

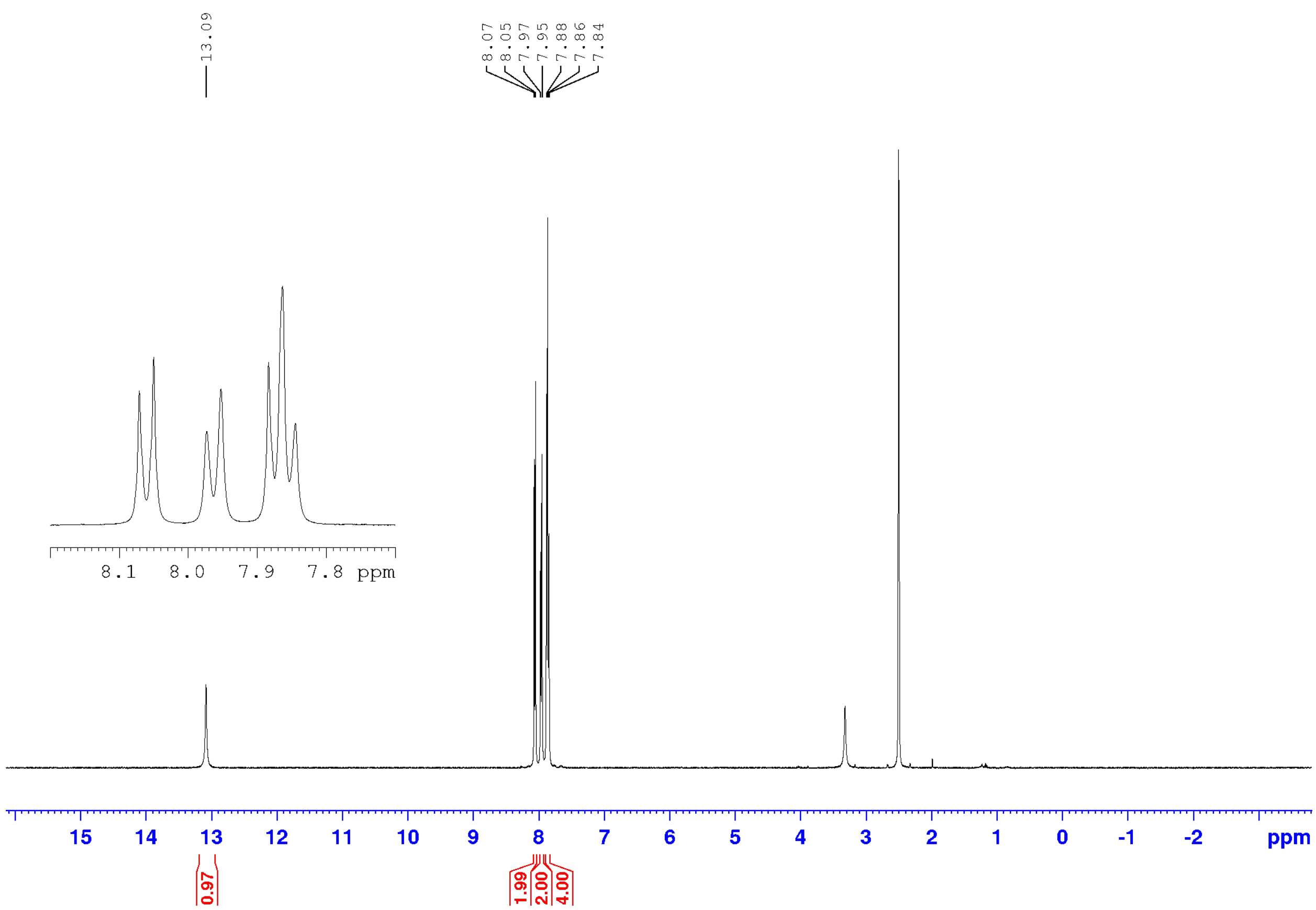

Figure S155. ^{1}H NMR spectrum of 4'-(trifluoromethyl)-[1,1'-biphenyl]-4-carboxylic acid (**35**) in DMSO-d_6.

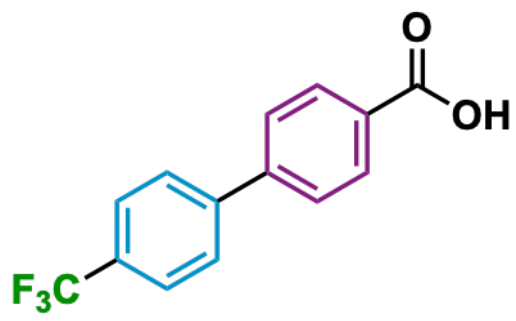

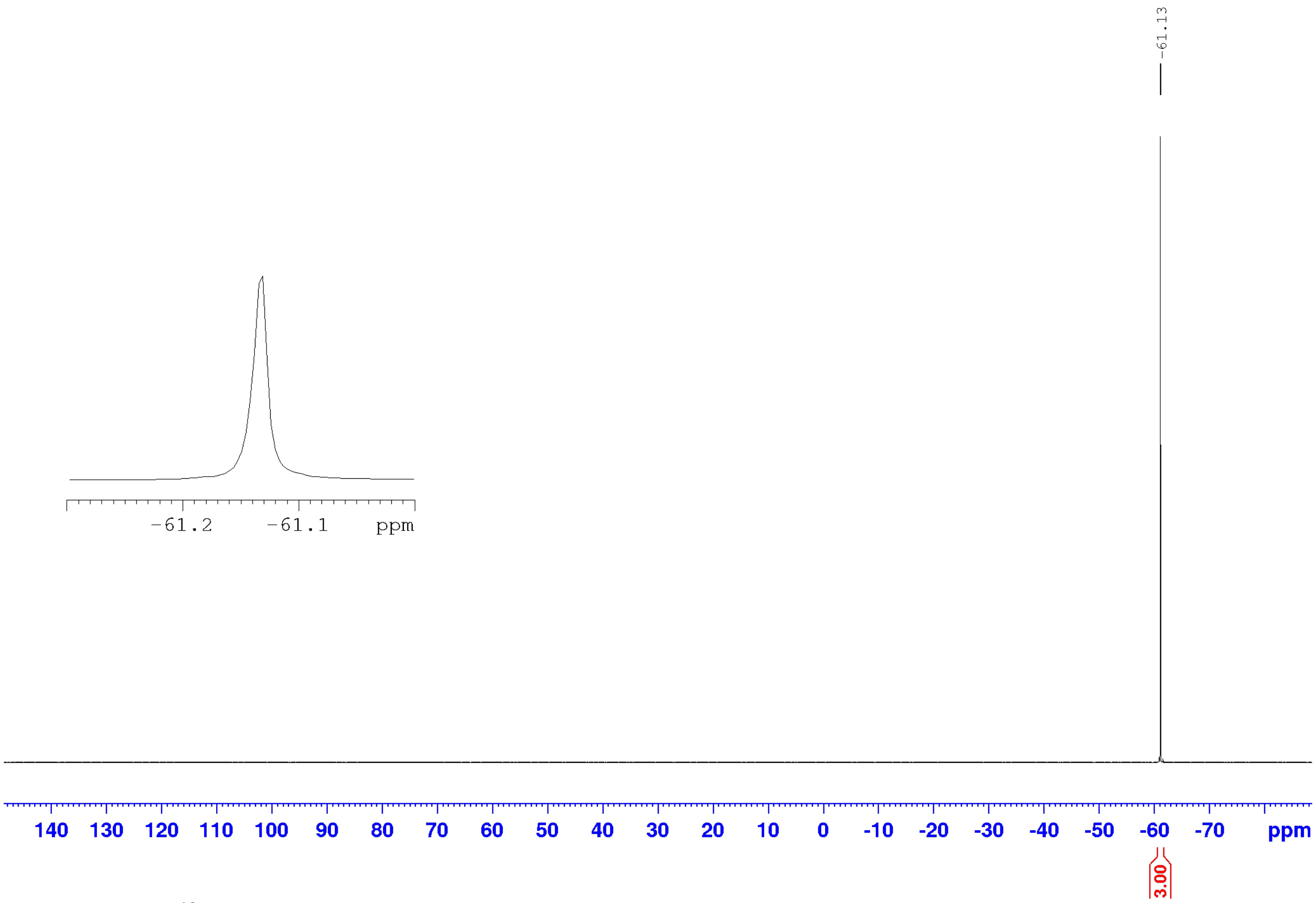

Figure S156. ^{19}F NMR spectrum of 4'-(trifluoromethyl)-[1,1'-biphenyl]-4-carboxylic acid (**35**) in DMSO-d_6.

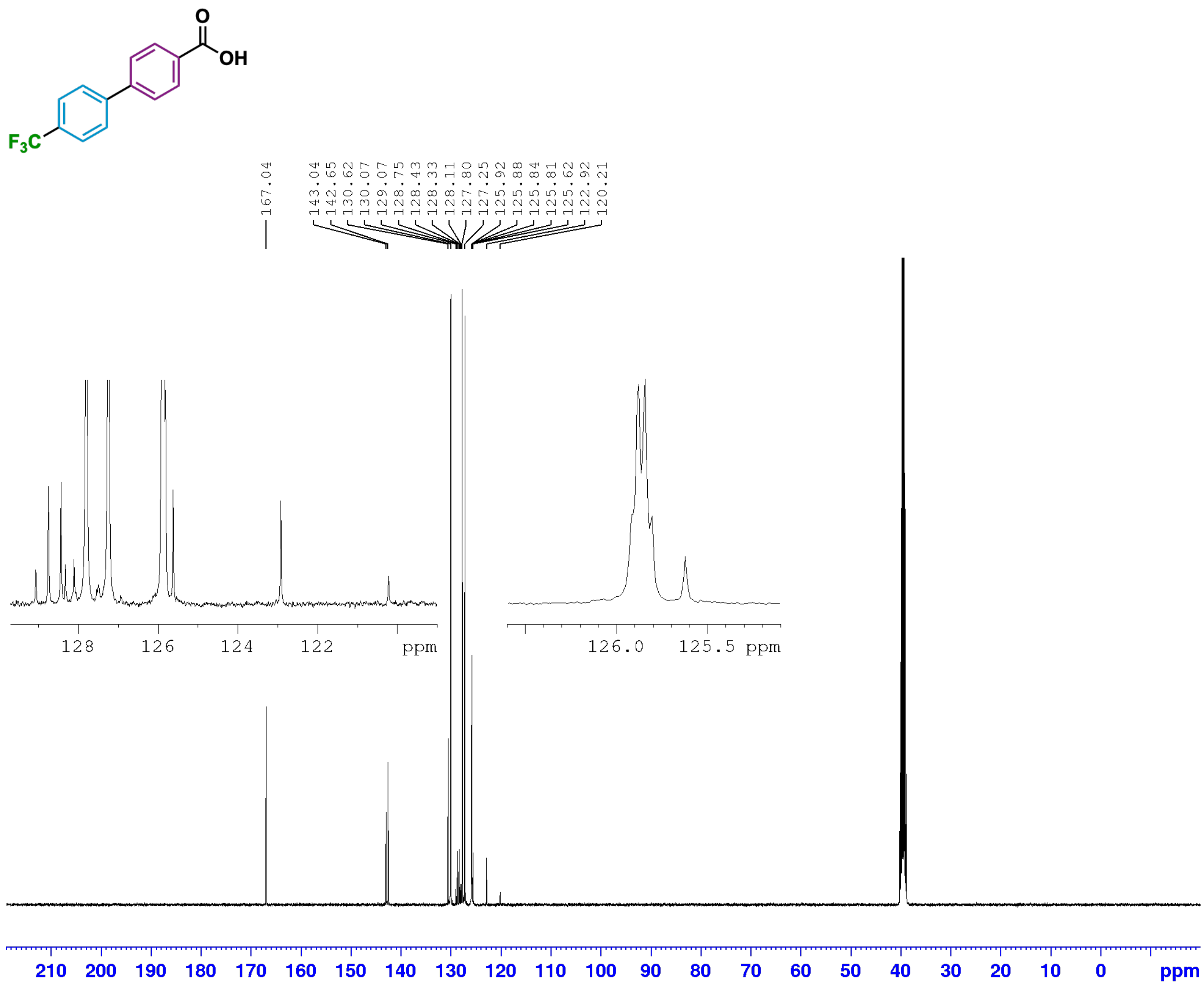

Figure S157. $^{13}C\{^{1}H\}$ NMR spectrum of 4'-(trifluoromethyl)-[1,1'-biphenyl]-4-carboxylic acid (**35**) in DMSO-d_6.

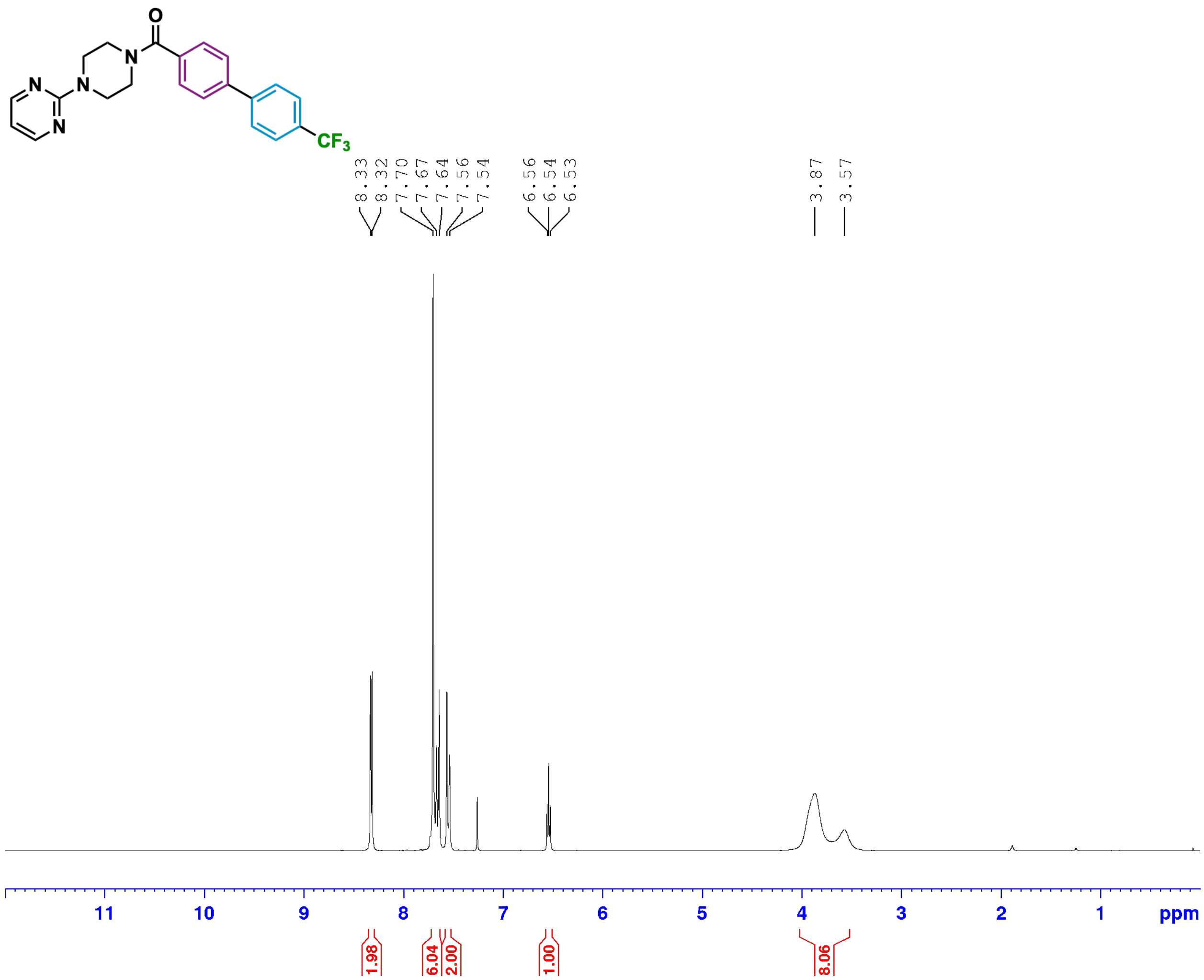

Figure S158. ^{1}H NMR spectrum of (4-(pyrimidin-2-yl)piperazin-1-yl)(4'-(trifluoromethyl)-[1,1'-biphenyl]-4-yl)methanone (**39**) in $CDCl_3$.

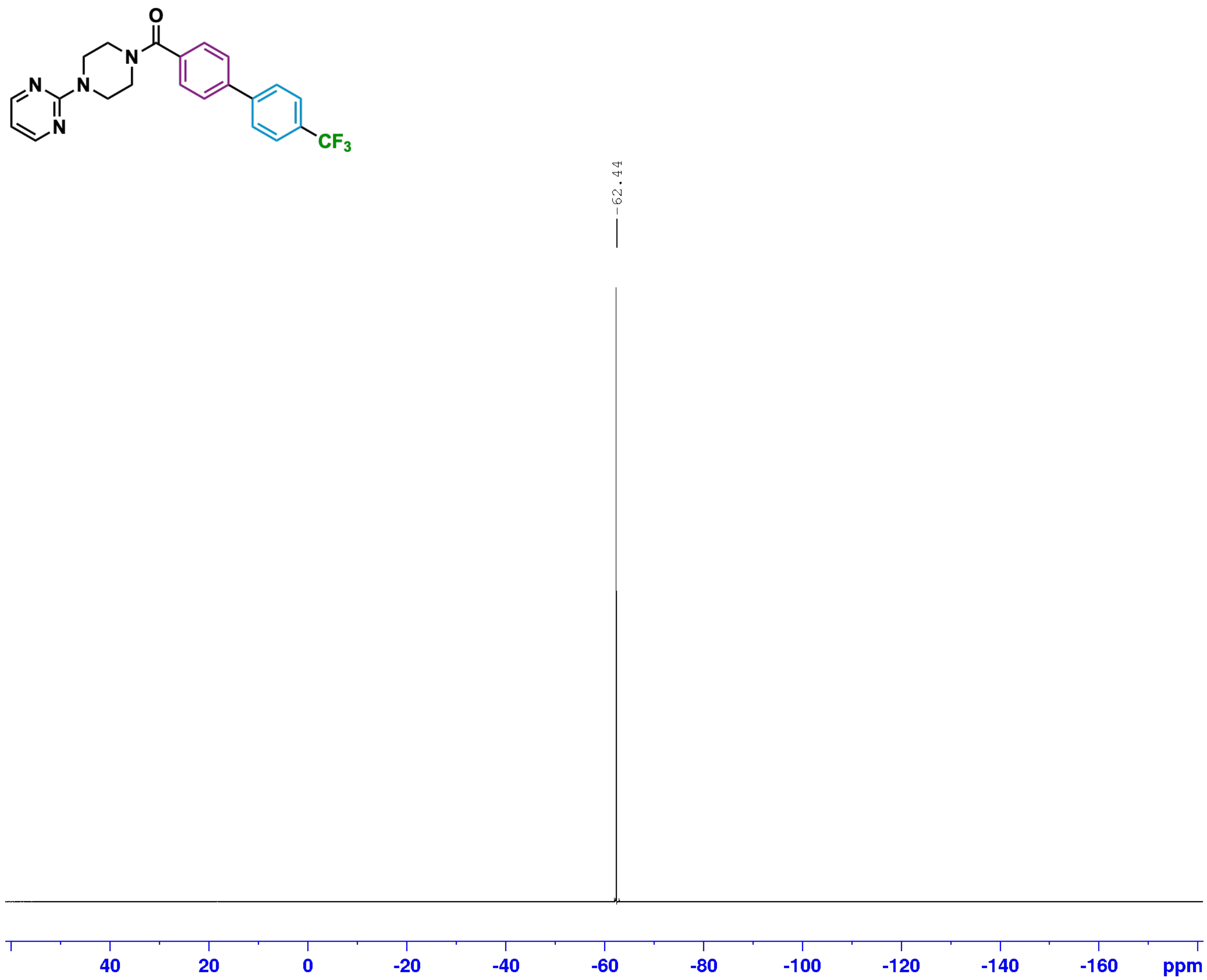

Figure S159. ^{19}F NMR spectrum of (4-(pyrimidin-2-yl)piperazin-1-yl)(4'-(trifluoromethyl)-[1,1'-biphenyl]-4-yl)methanone (**39**) in $CDCl_3$.

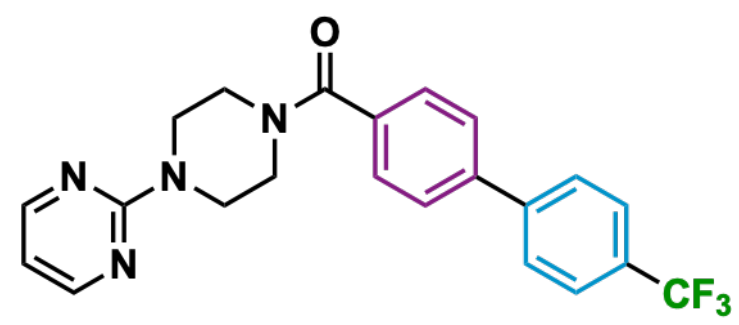

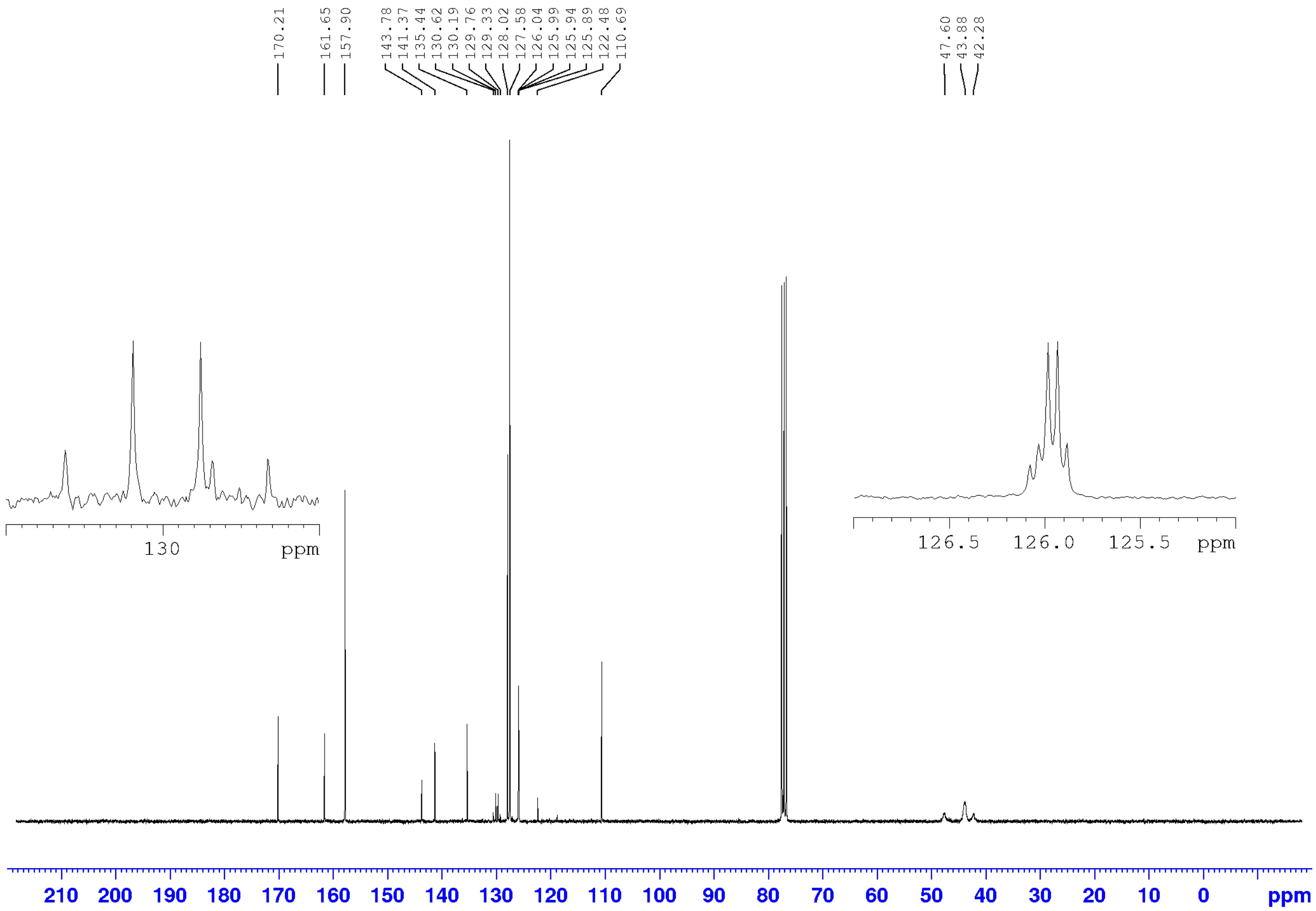

Figure S160. $^{13}C\{^{1}H\}$ NMR spectrum of (4-(pyrimidin-2-yl)piperazin-1-yl)(4'-(trifluoromethyl)-[1,1'-biphenyl]-4-yl)methanone (**39**) in $CDCl_3$.

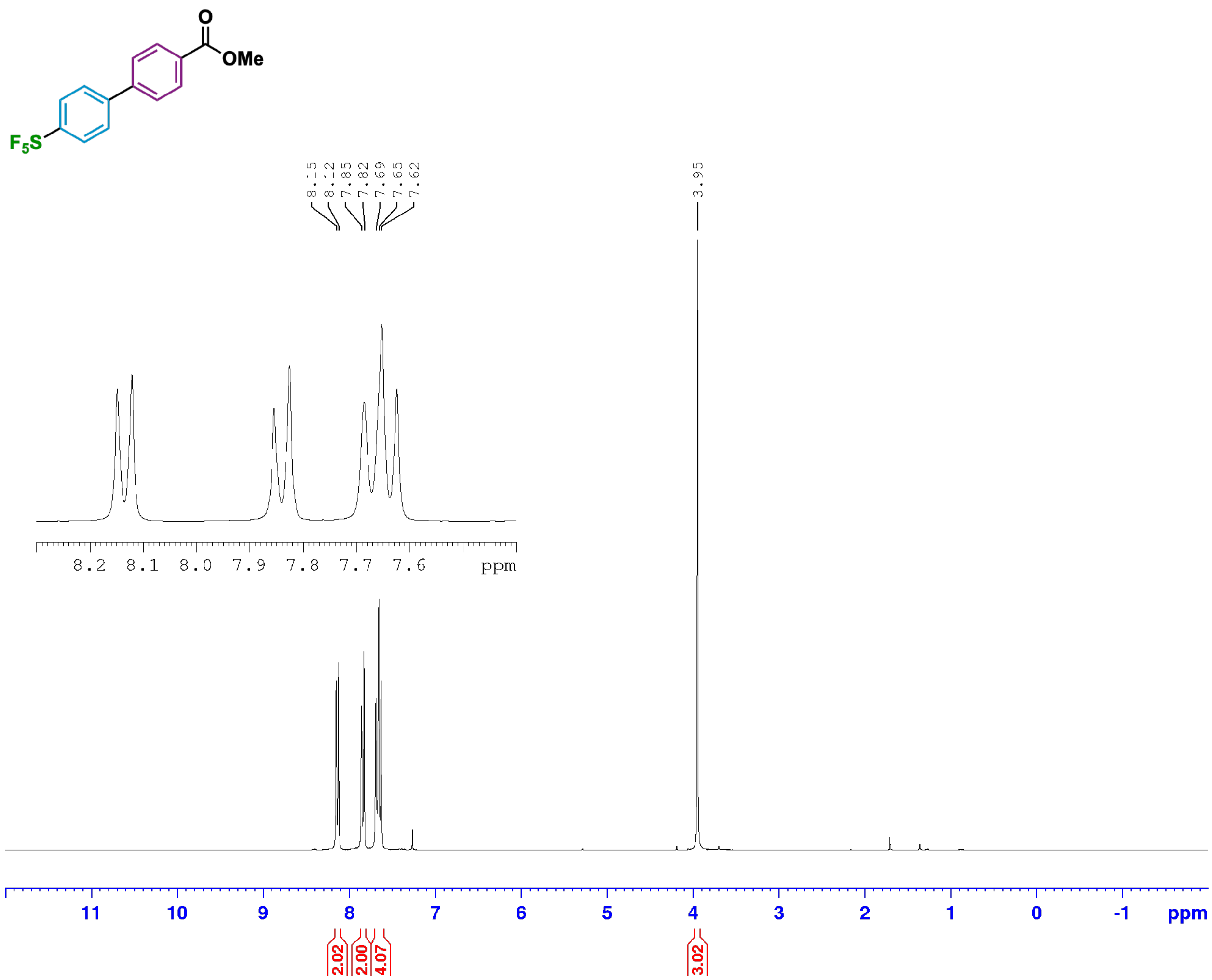

Figure S161. ^{1}H NMR spectrum of methyl 4'-(pentafluoro-λ^{6}-sulfaneyl)-[1,1'-biphenyl]-4-carboxylate (**S-21**) in $CDCl_3$.

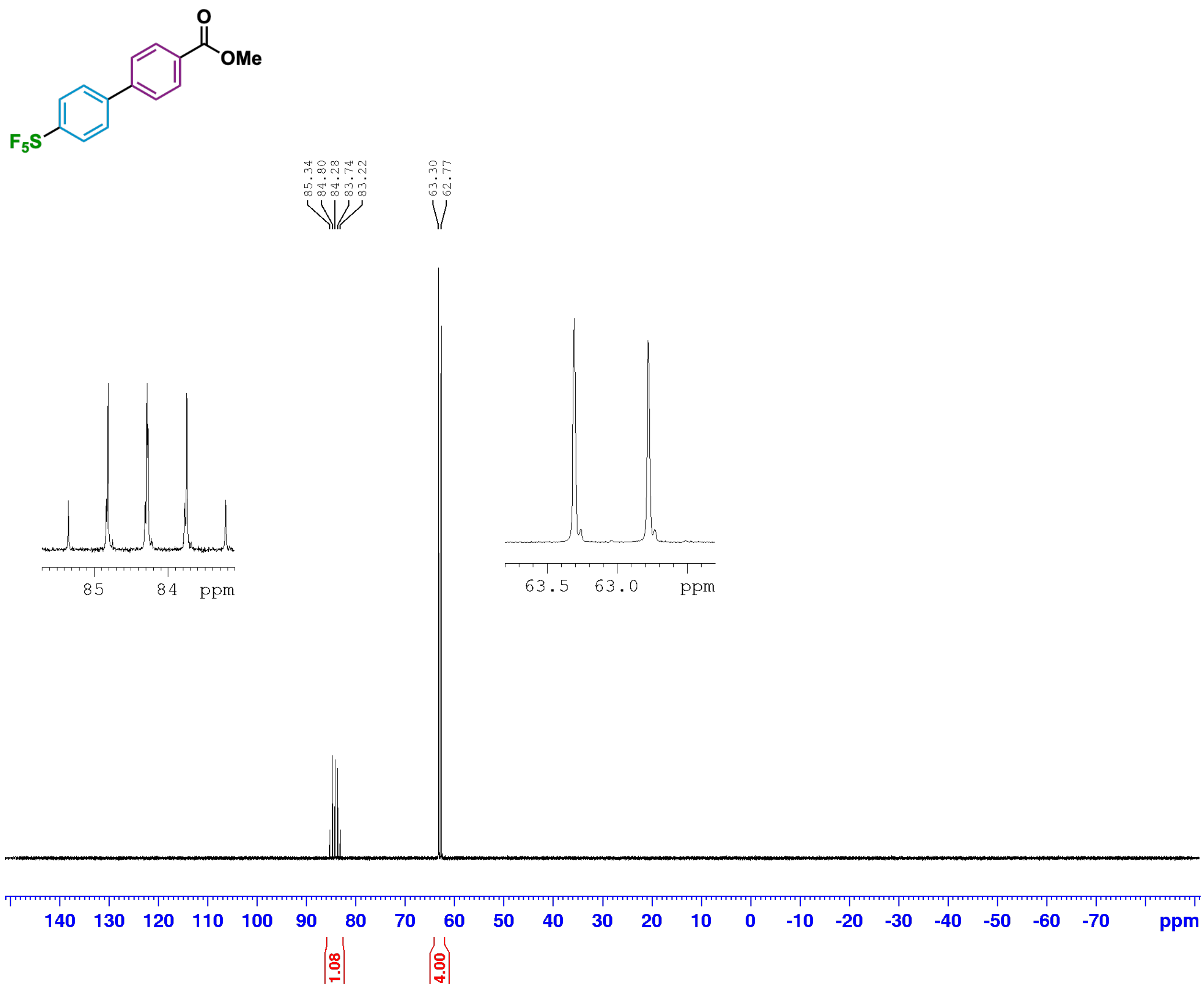

Figure S162. ^{19}F NMR spectrum of methyl 4'-(pentafluoro-λ^6-sulfaneyl)-[1,1'-biphenyl]-4-carboxylate (**S-21**) in $CDCl_3$.

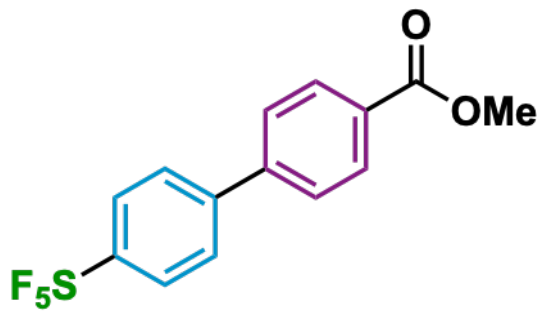

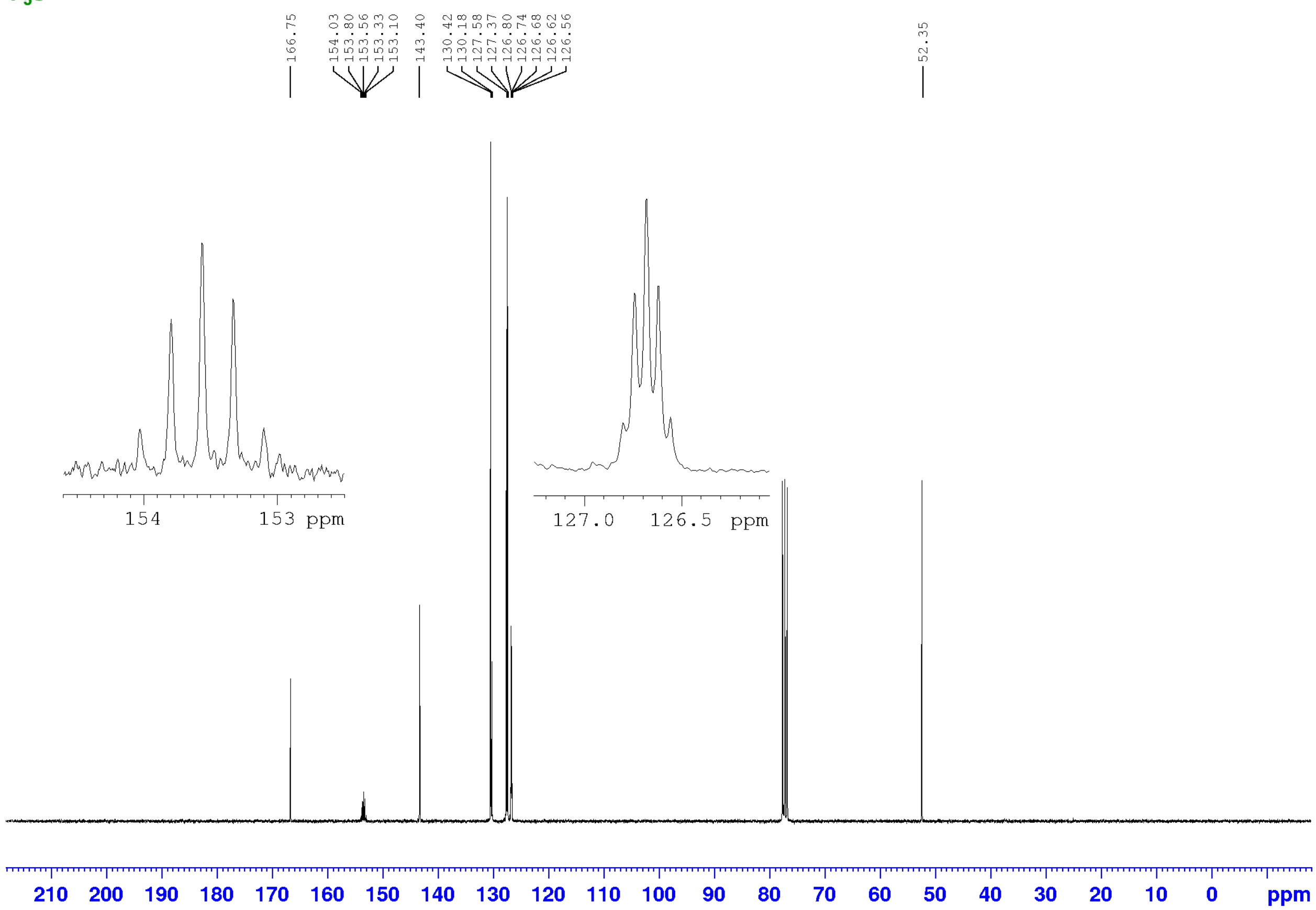

Figure S163. $^{13}C\{^{1}H\}$ NMR spectrum of methyl 4'-(pentafluoro-λ^{6}-sulfaneyl)-[1,1'-biphenyl]-4-carboxylate (**S-21**) in $CDCl_3$.

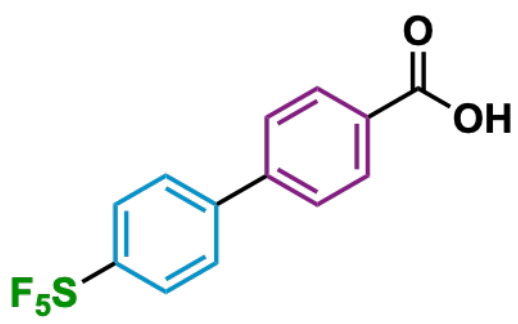

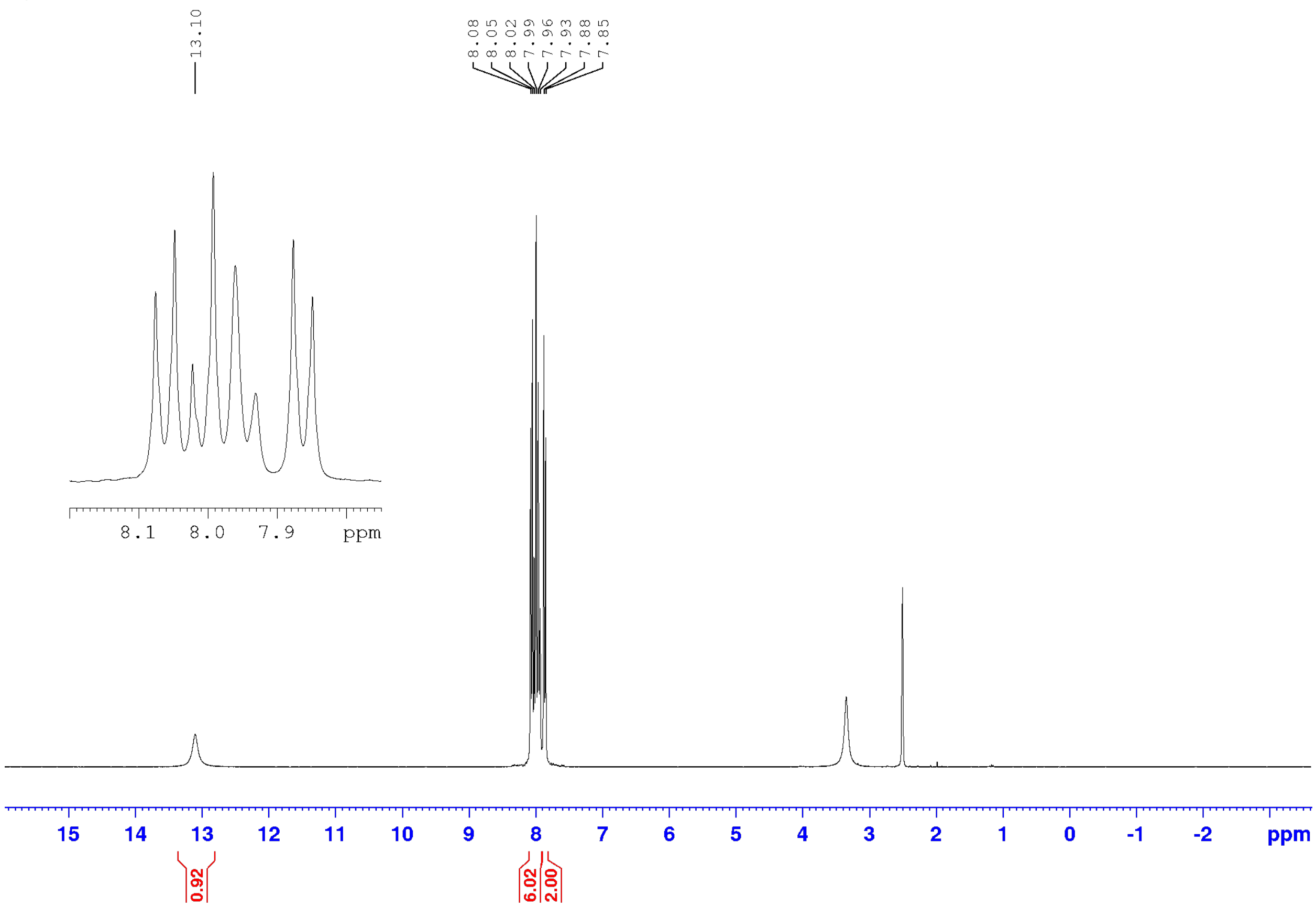

Figure S164. ^{1}H NMR spectrum of methyl 4'-(pentafluoro-λ^{6}-sulfaneyl)-[1,1'-biphenyl]-4-carboxylate (**36**) in DMSO-d_6.

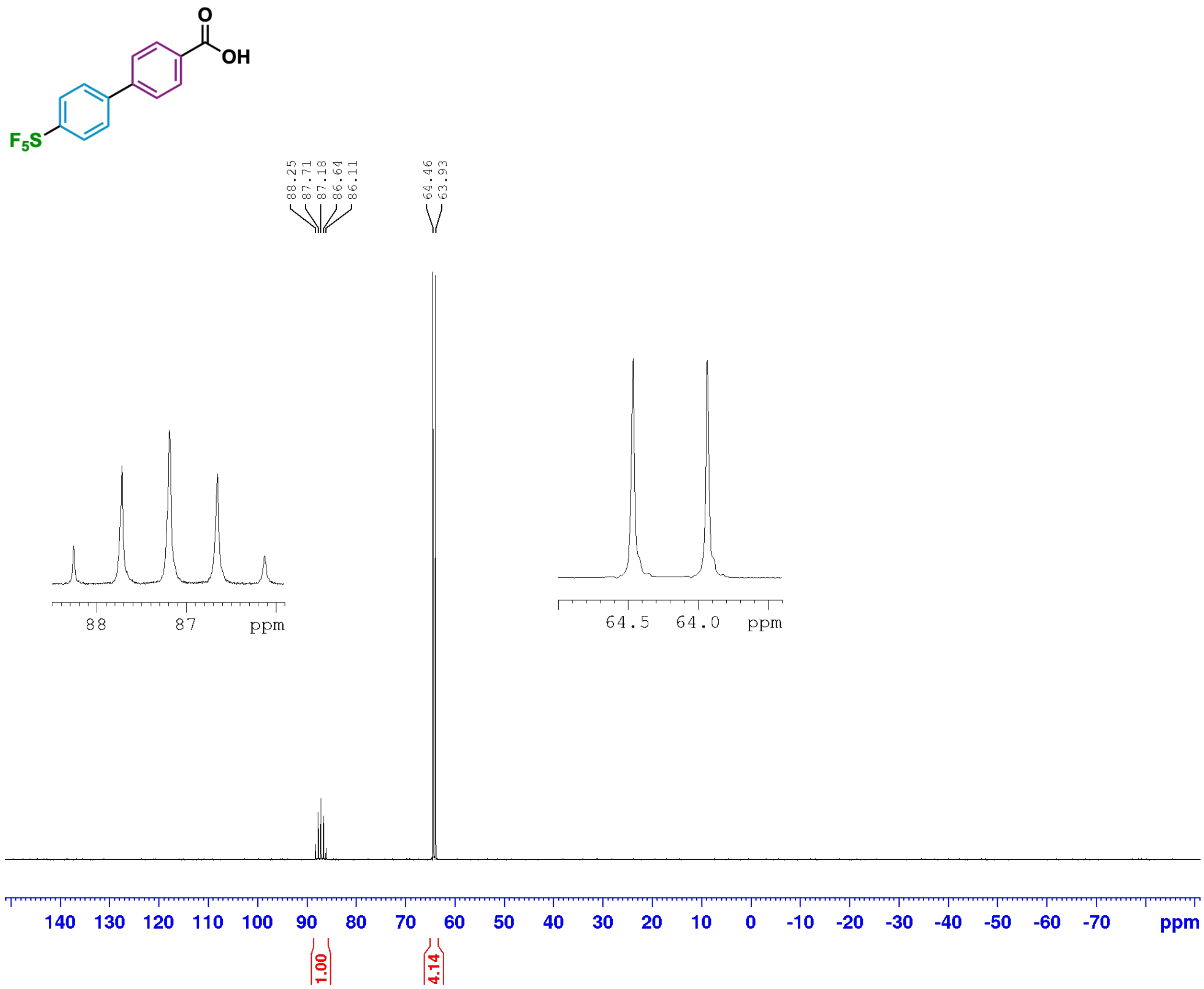

Figure S165. ^{19}F NMR spectrum of 4'-(pentafluoro-λ^6-sulfaneyl)-[1,1'-biphenyl]-4-carboxylic acid (**36**) in DMSO-d_6.

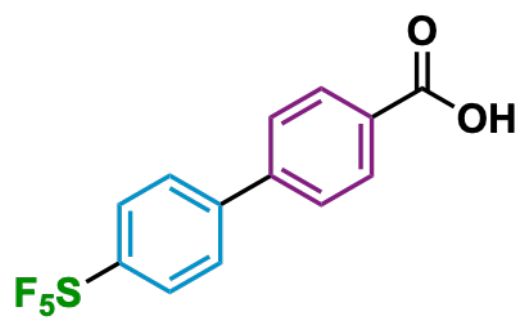

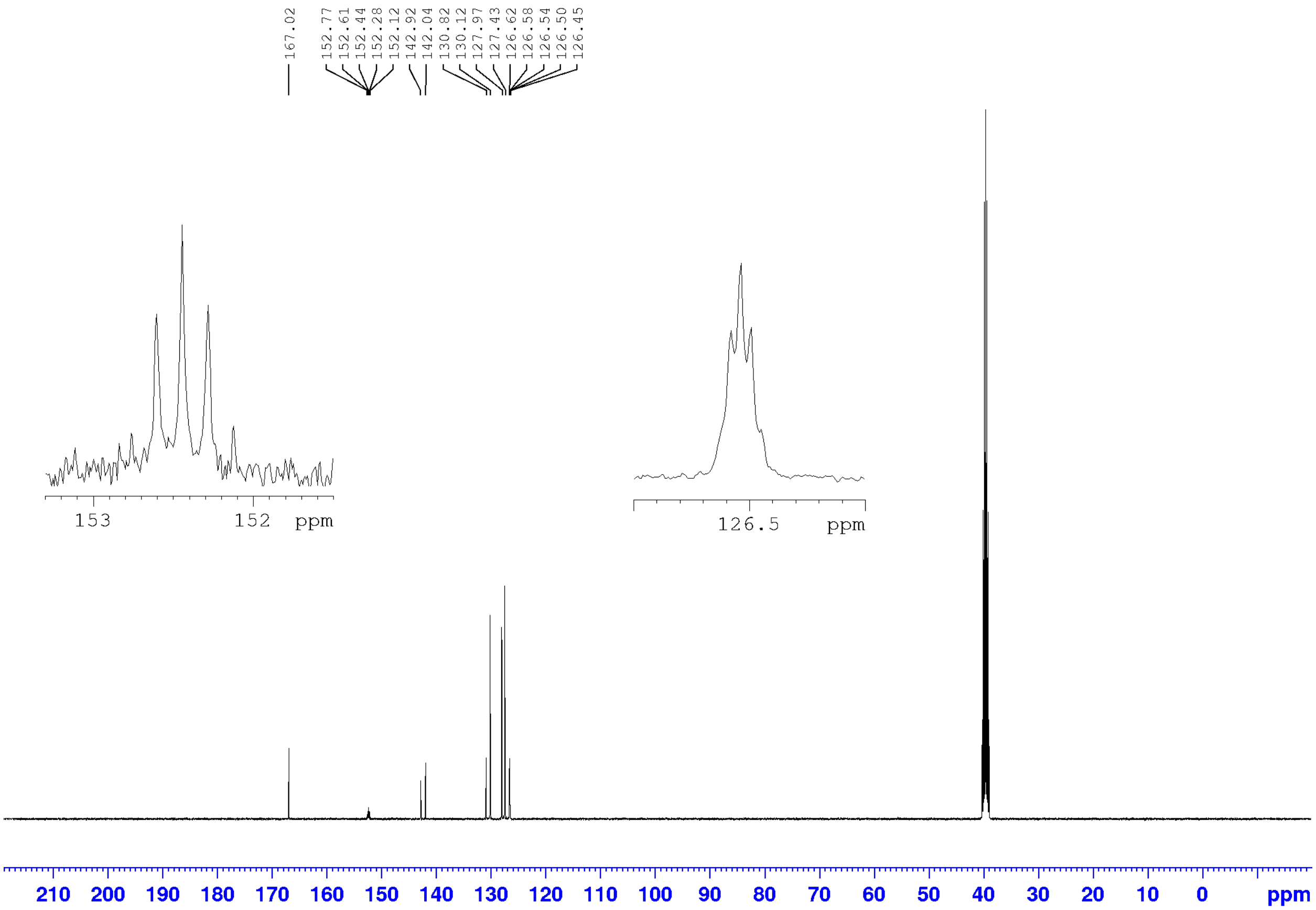

Figure S166. $^{13}C\{^{1}H\}$ NMR spectrum of 4'-(pentafluoro-λ^6-sulfaneyl)-[1,1'-biphenyl]-4-carboxylic acid (**36**) in DMSO-d_6.

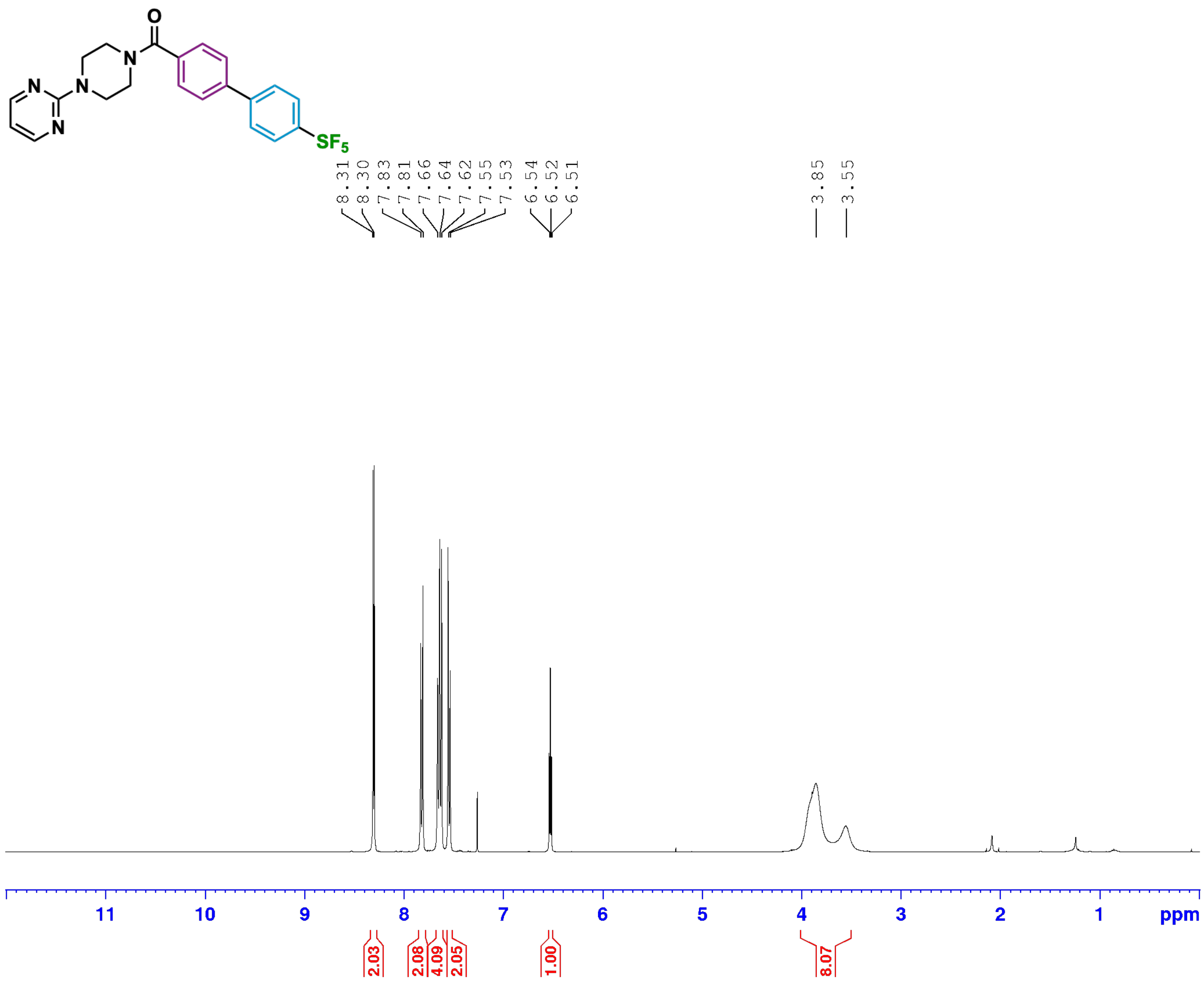

Figure S167. ^{1}H NMR spectrum of (4'-(pentafluoro-λ^{6}-sulfaneyl)-[1,1'-biphenyl]-4-yl)(4-(pyrimidin-2-yl)piperazin-1-yl)methanone (**40**) in $CDCl_3$.

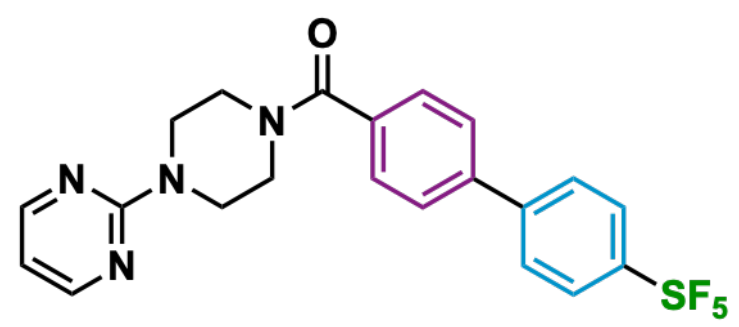

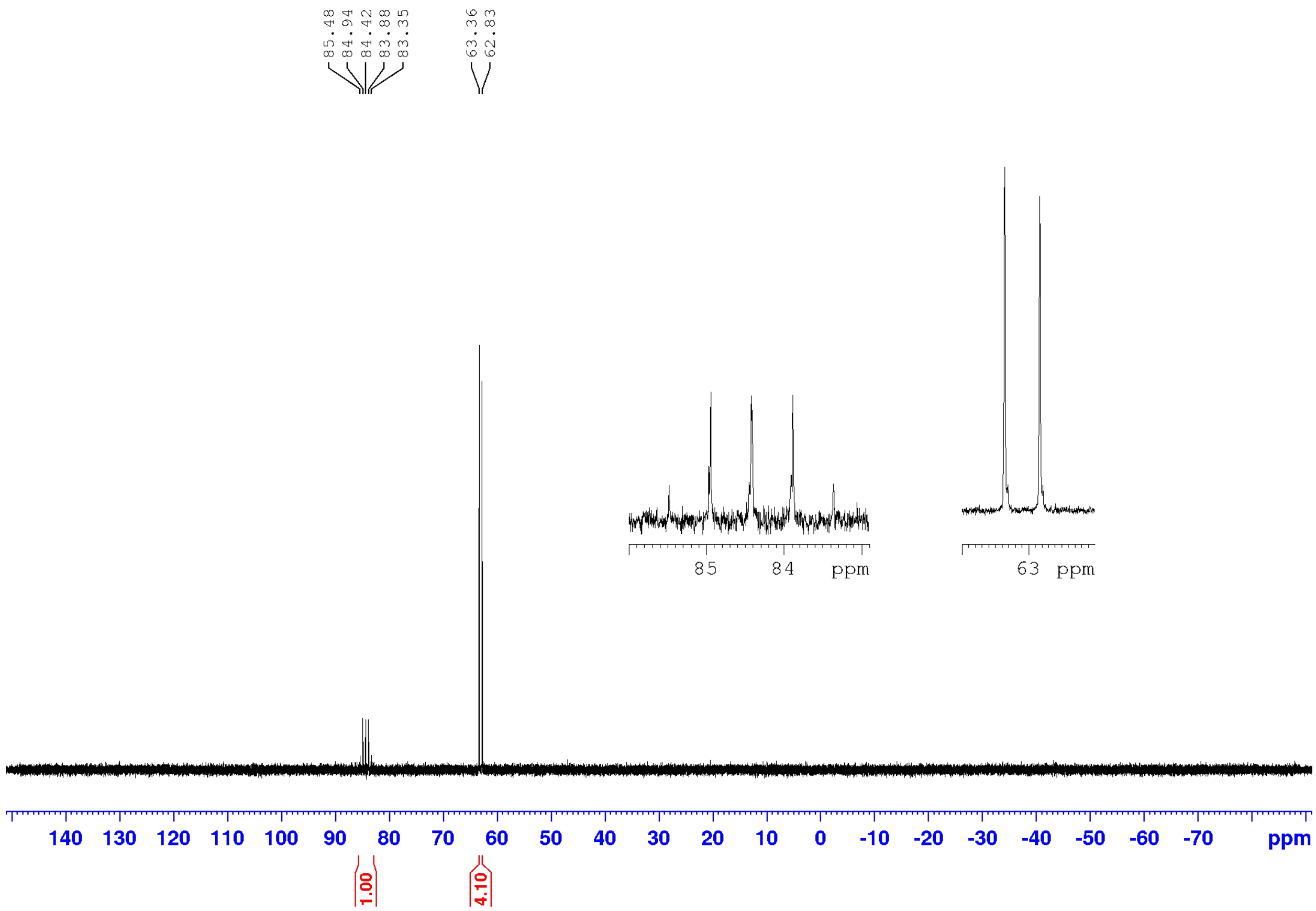

Figure S168. ^{19}F NMR spectrum of (4'-(pentafluoro-λ^6-sulfaneyl)-[1,1'-biphenyl]-4-yl)(4-(pyrimidin-2-yl)piperazin-1-yl)methanone (**40**) in $CDCl_3$.

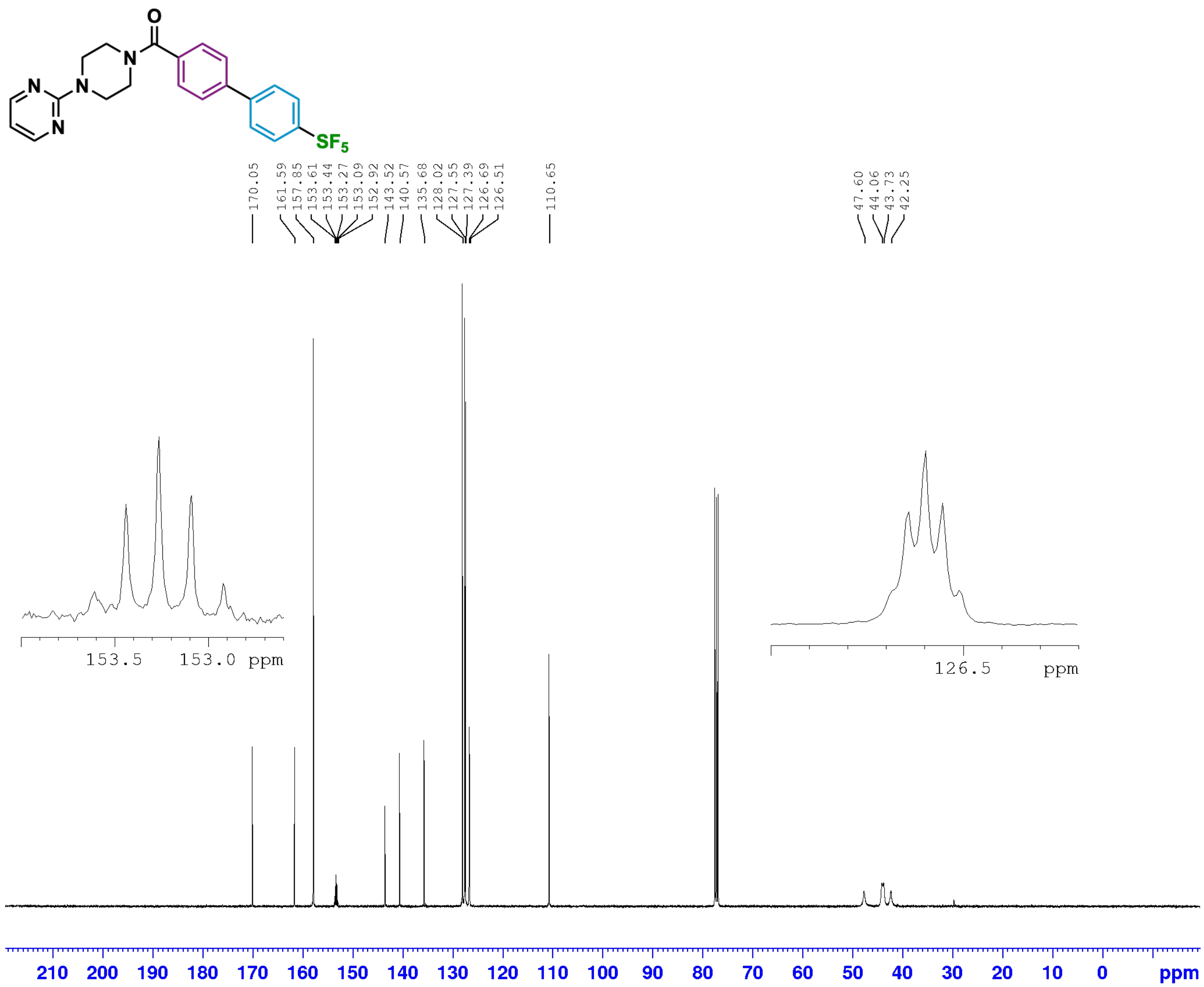

Figure S169. $^{13}C\{^{1}H\}$ NMR spectrum of (4'-(pentafluoro-λ^{6}-sulfaneyl)-[1,1'-biphenyl]-4-yl)(4-(pyrimidin-2-yl)piperazin-1-yl)methanone (**40**) in $CDCl_3$.

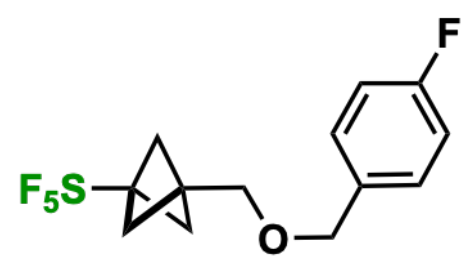

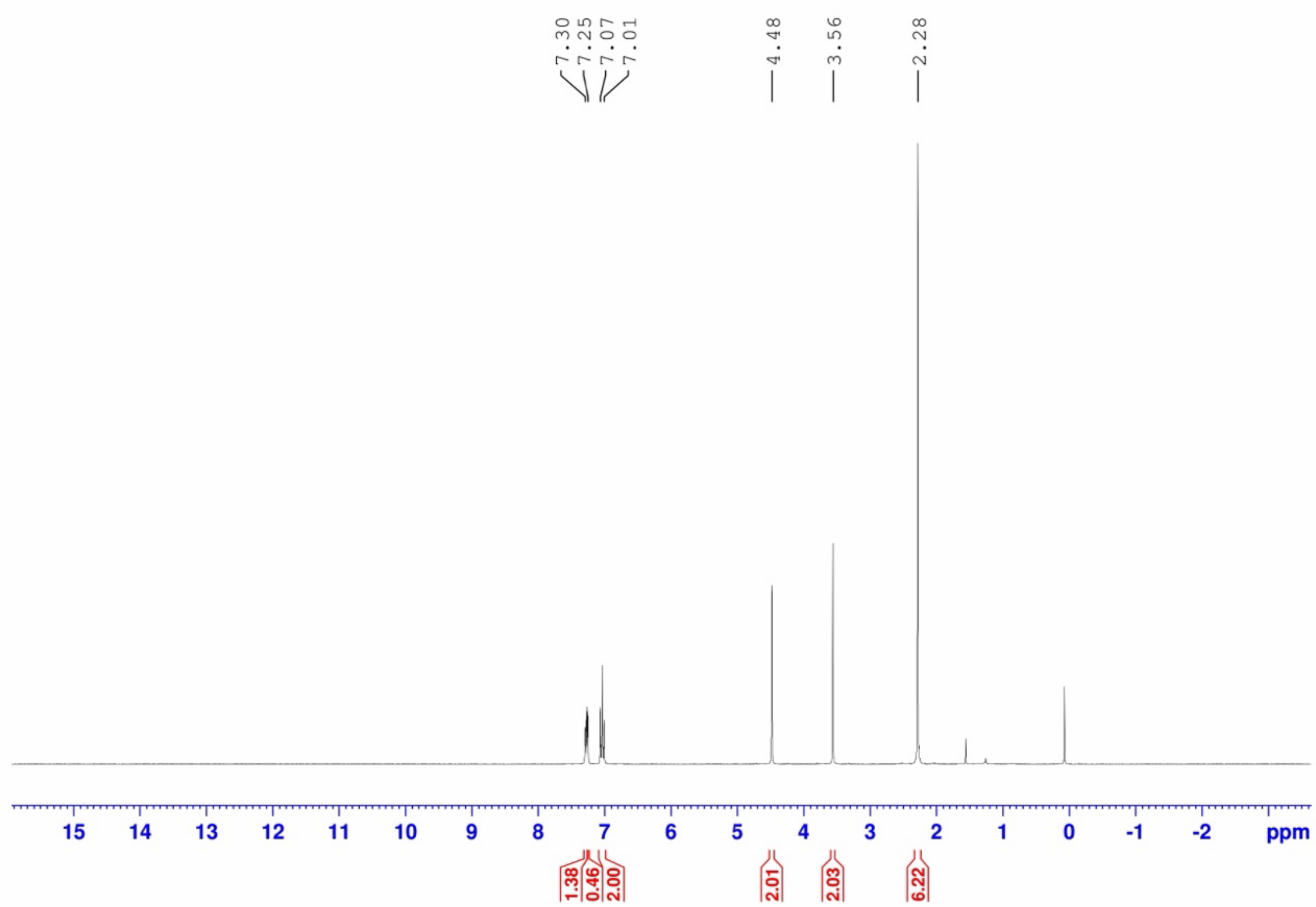

Figure S170. ^{1}H NMR spectrum of pentafluoro(3-(((4-fluorobenzyl)oxy)methyl)bicyclo[1.1.1]pentan-1-yl)-λ^6-sulfane **(42)** in $CDCl_3$.

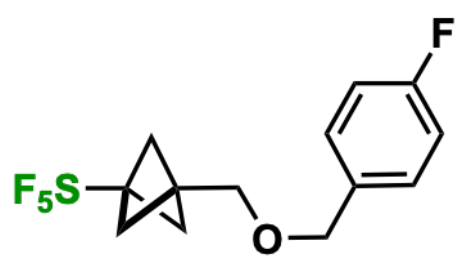

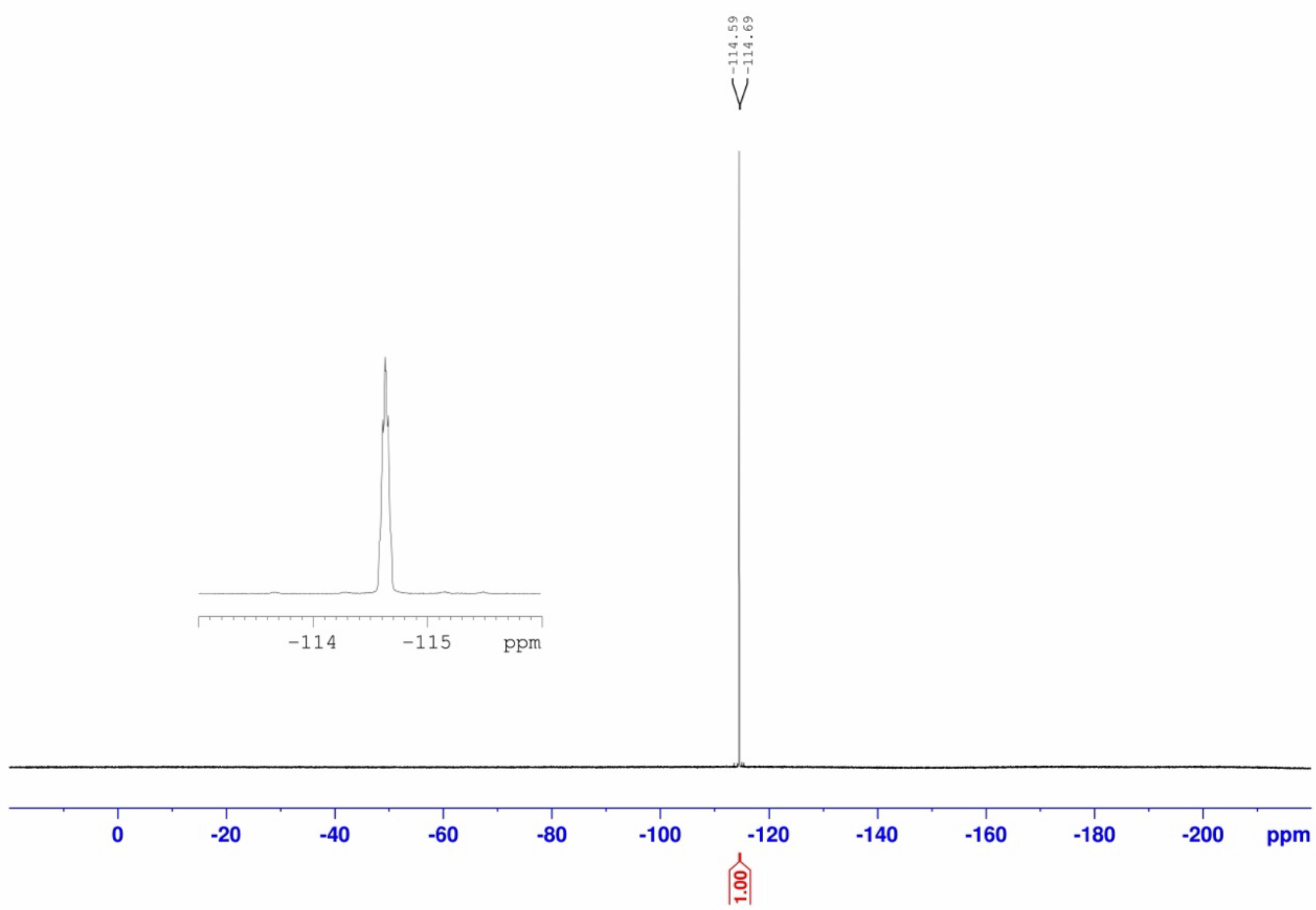

Figure S171. ^{19}F NMR spectrum of pentafluoro(3-(((4-fluorobenzyl)oxy)methyl)bicyclo[1.1.1]pentan-1-yl)-λ^6-sulfane **(42)** in $CDCl_3$.

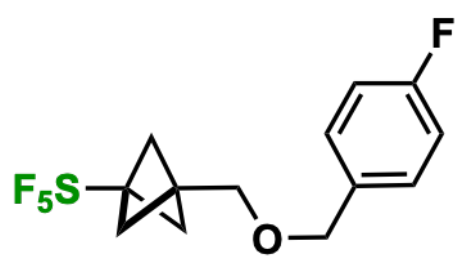

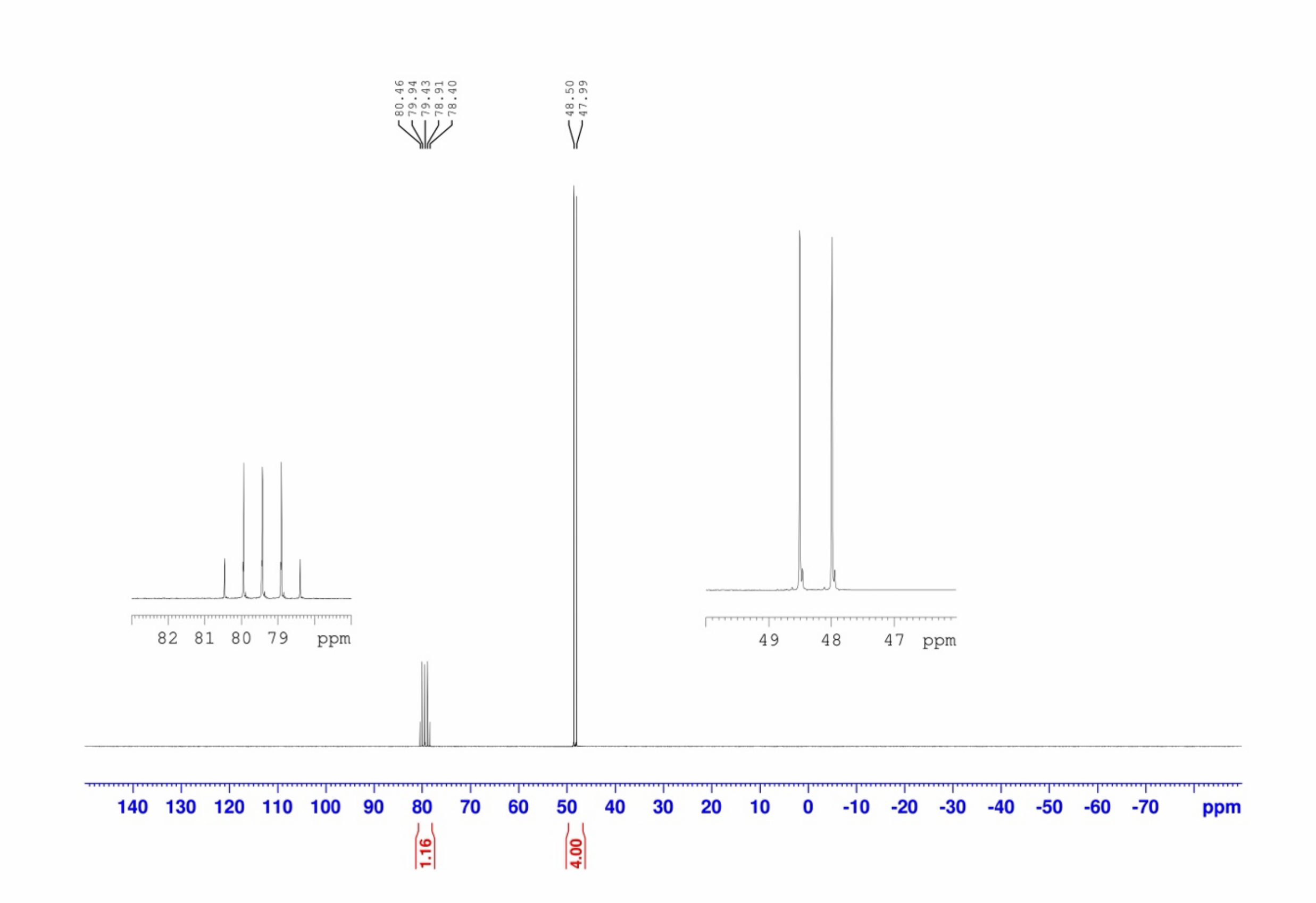

Figure S172. ^{19}F NMR spectrum of pentafluoro(3-(((4-fluorobenzyl)oxy)methyl)bicyclo[1.1.1]pentan-1-yl)-λ^6-sulfane **(42)** in $CDCl_3$.

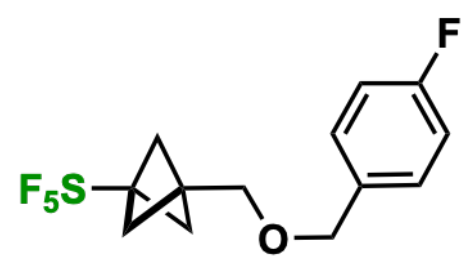

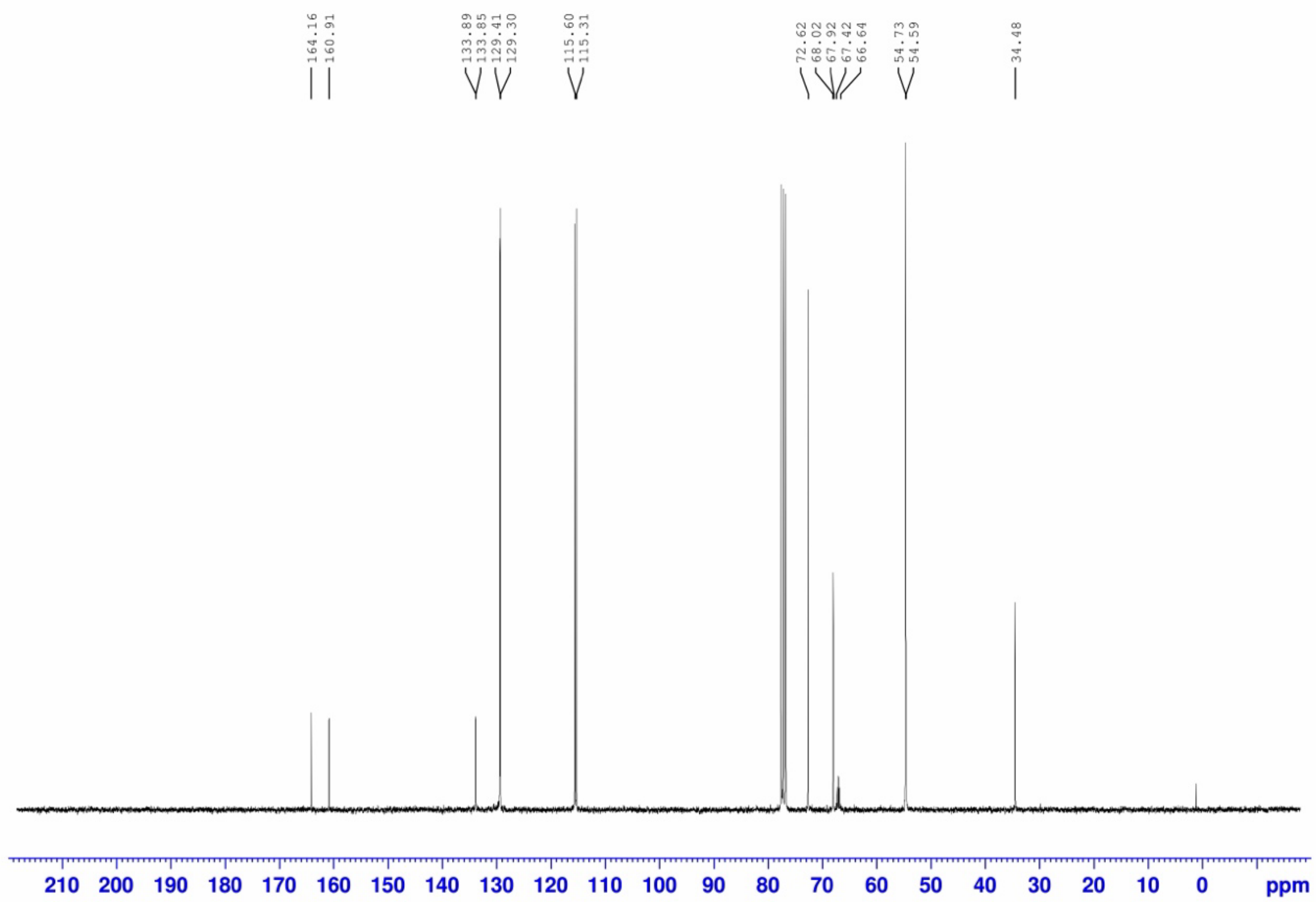

Figure S173. $^{13}C\{^1H\}$ NMR spectrum of pentafluoro(3-(((4-fluorobenzyl)oxy)methyl)bicyclo[1.1.1]pentan-1-yl)- λ^6-sulfane **(42)** in $CDCl_3$.

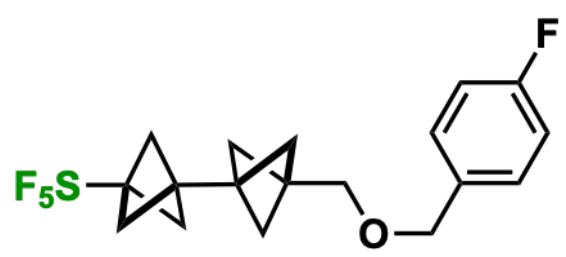

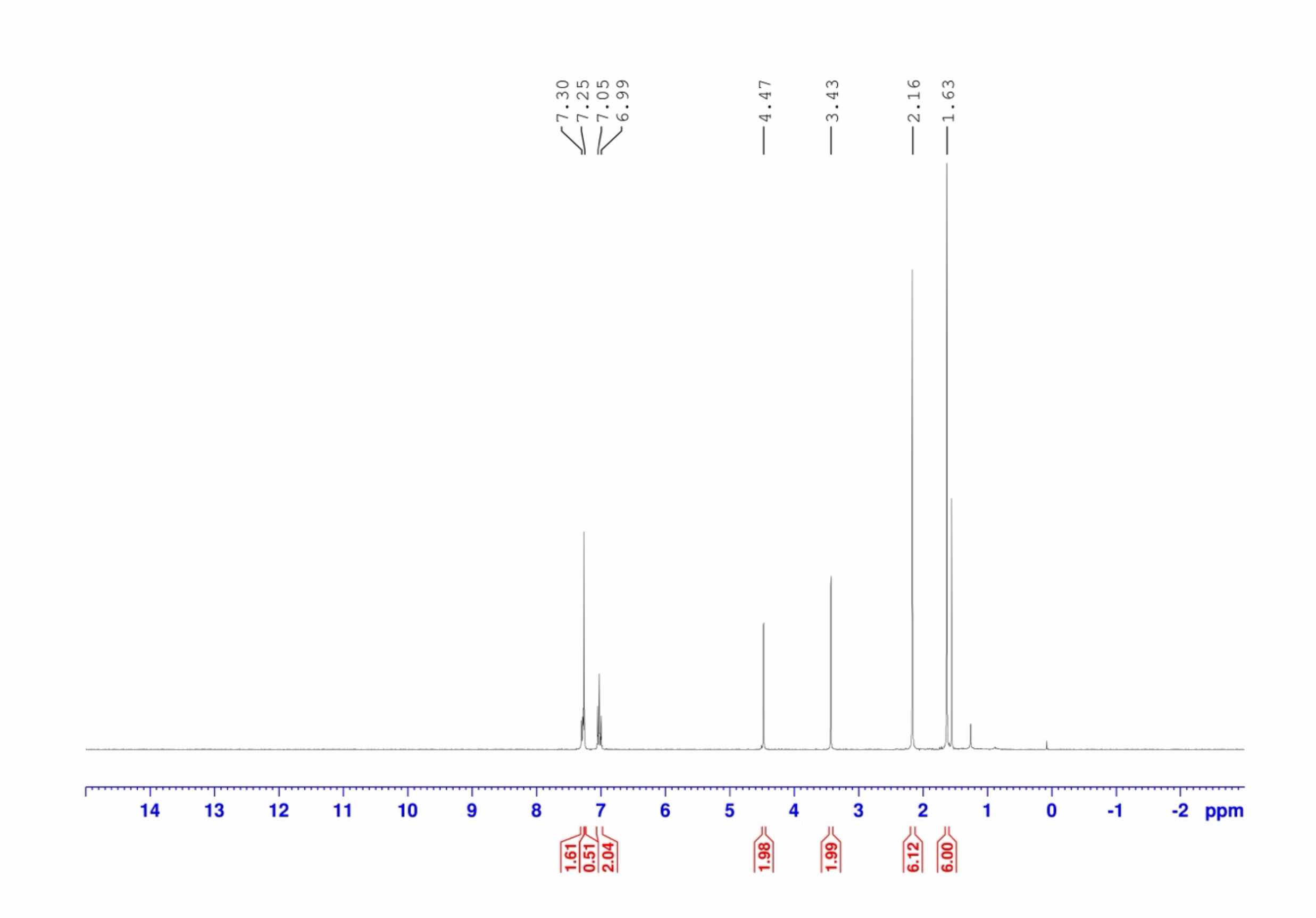

Figure S174. ^{1}H NMR spectrum of pentafluoro(3'-(((4-fluorobenzyl)oxy)methyl)-[1,1'-bi(bicyclo[1.1.1]pentan)]-3-yl)- λ^6-sulfane **(43)** in $CDCl_3$.

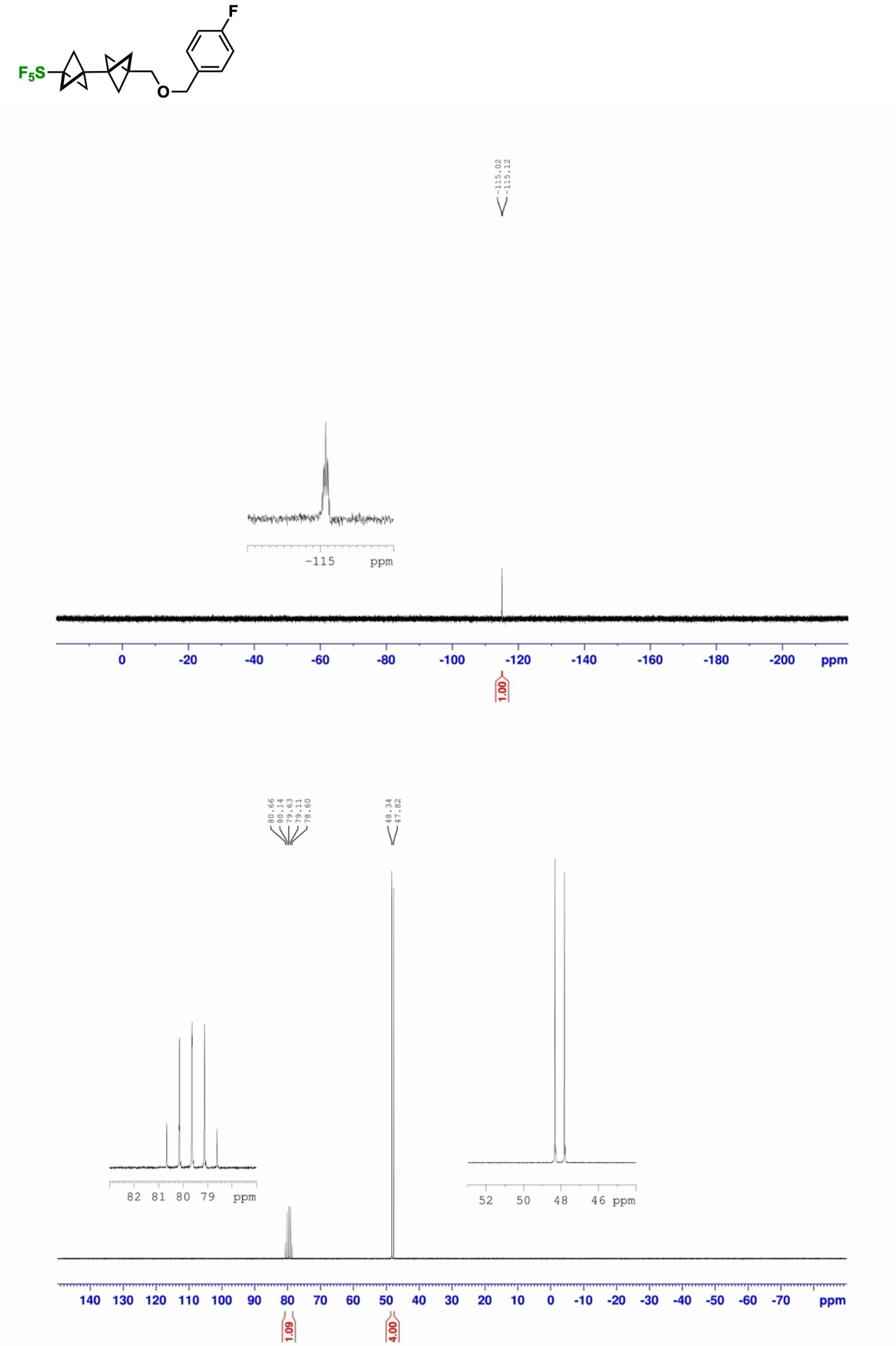

Figure S175. ^{19}F NMR spectrum of pentafluoro(3'-(((4-fluorobenzyl)oxy)methyl)-[1,1'-bi(bicyclo[1.1.1]pentan)]-3-yl)- λ^6-sulfane **(43)** in $CDCl_3$.

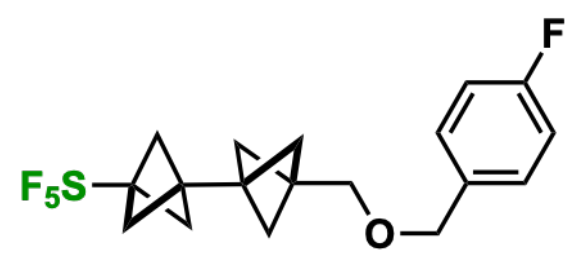

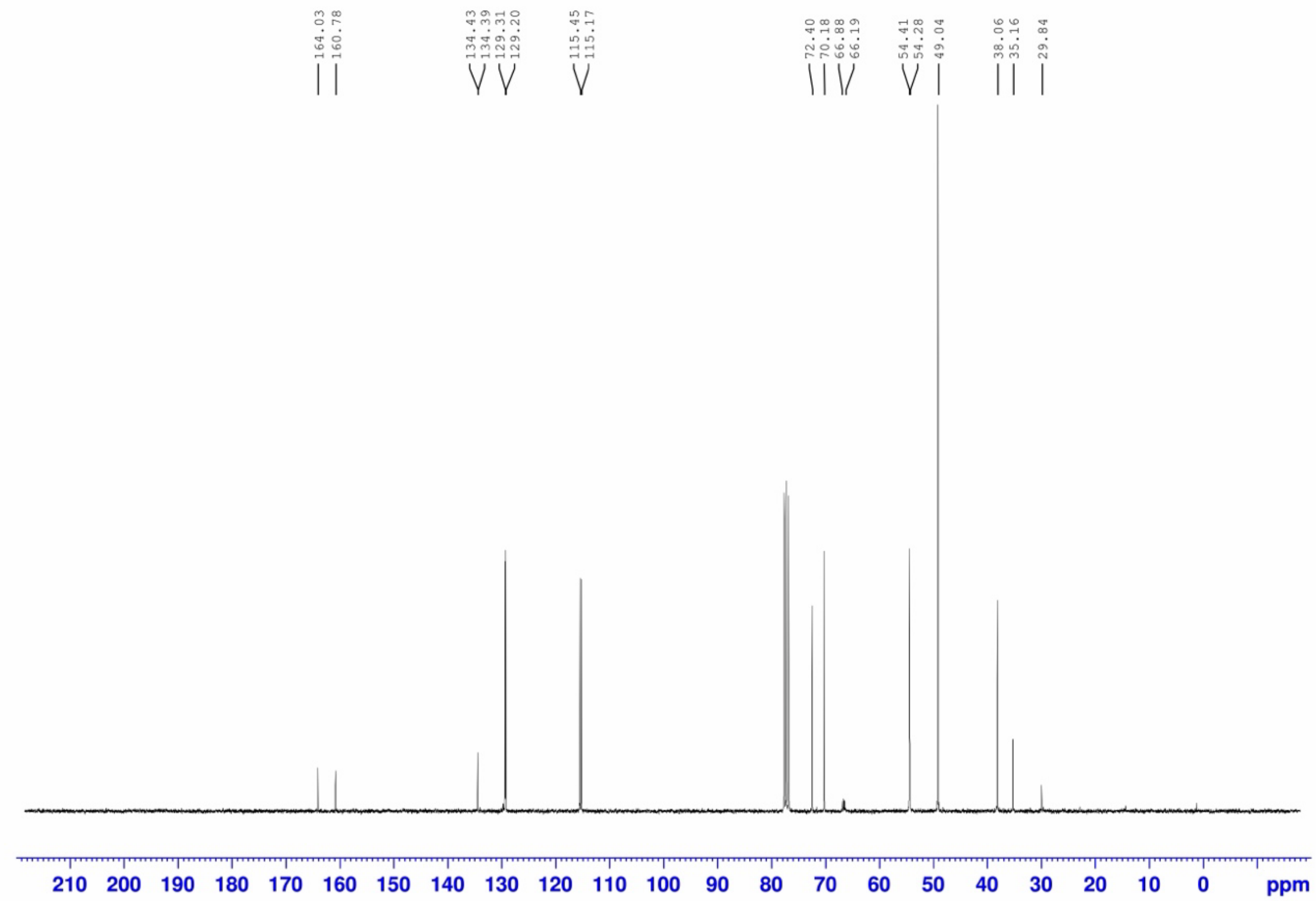

Figure S176. $^{13}C\{^1H\}$ NMR spectrum of pentafluoro(3'-(((4-fluorobenzyl)oxy)methyl)-[1,1'-bi(bicyclo[1.1.1]pentan)]-3-yl)- λ^6-sulfane **(43)** in $CDCl_3$.

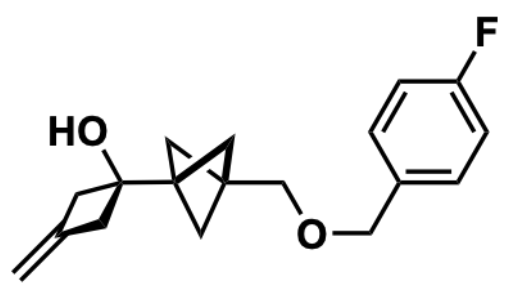

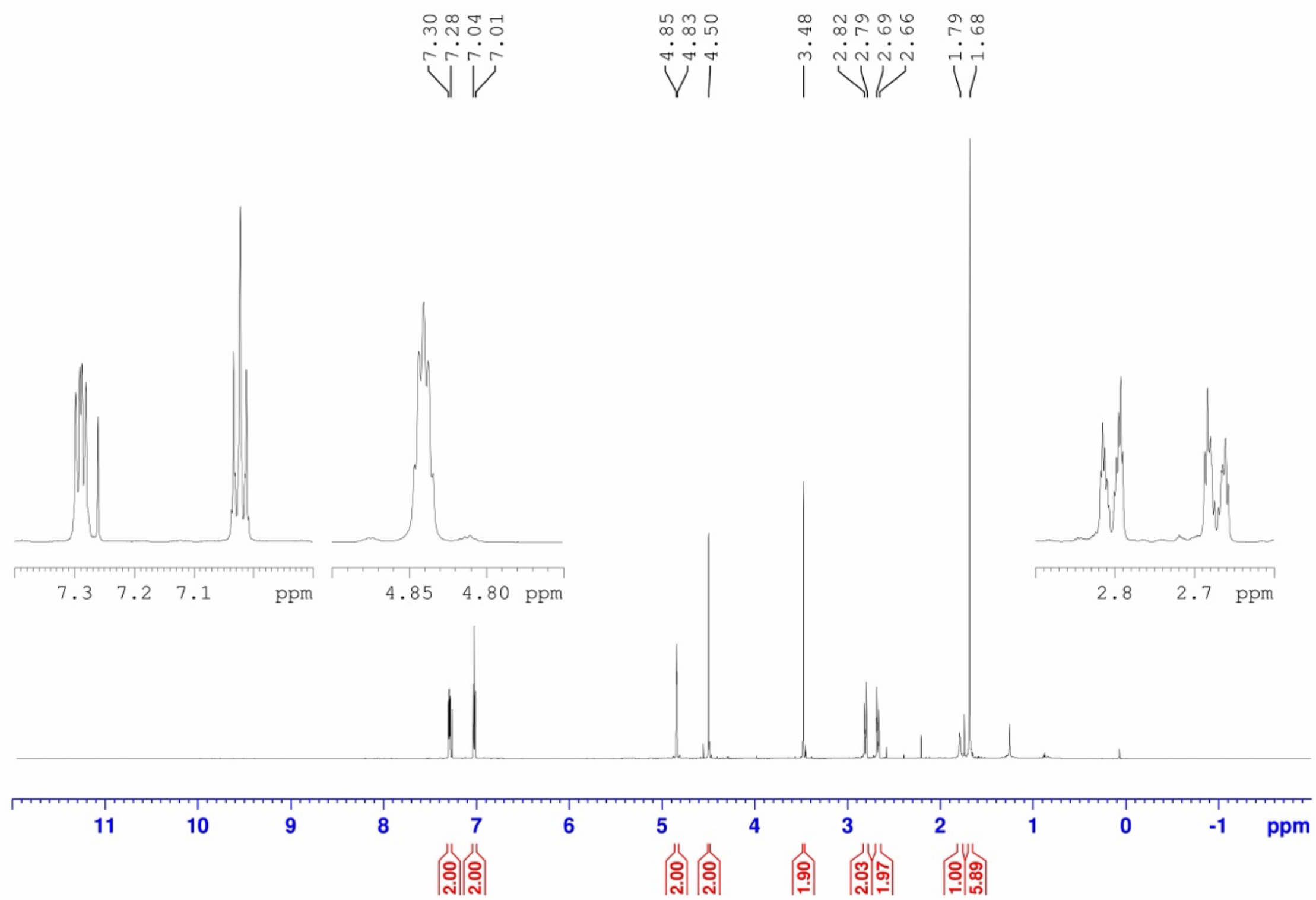

Figure S177. ^{1}H NMR spectrum of 1-(3-(((4-fluorobenzyl)oxy)methyl)bicyclo[1.1.1]pentan-1-yl)-3-methylenecyclobutan-1-ol **(44)** in $CDCl_3$.

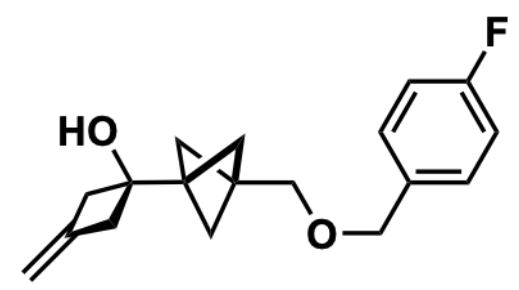

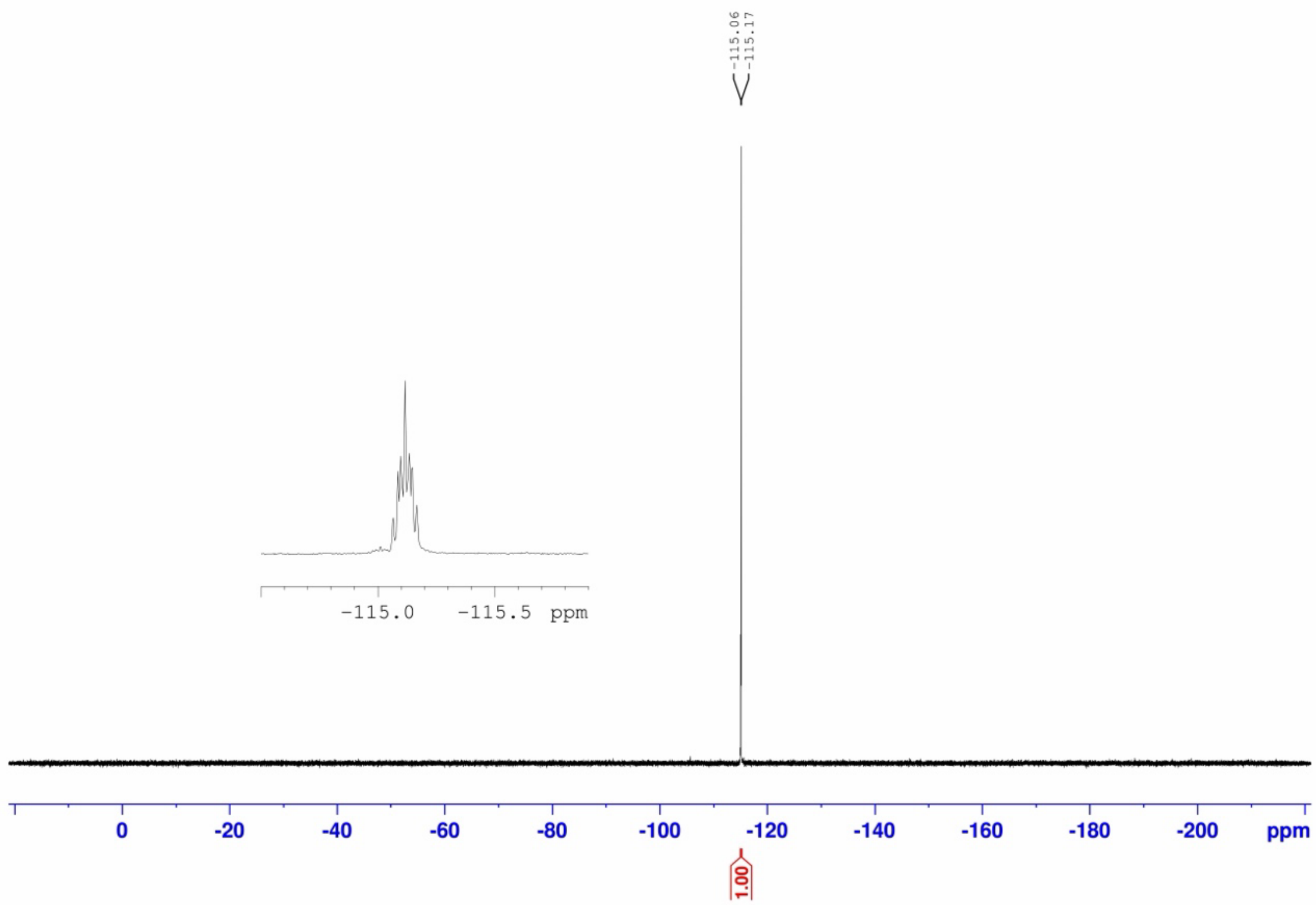

Figure S178. ^{19}F NMR spectrum of 1-(3-(((4-fluorobenzyl)oxy)methyl)bicyclo[1.1.1]pentan-1-yl)-3-methylenecyclobutan-1-ol **(44)** in $CDCl_3$.

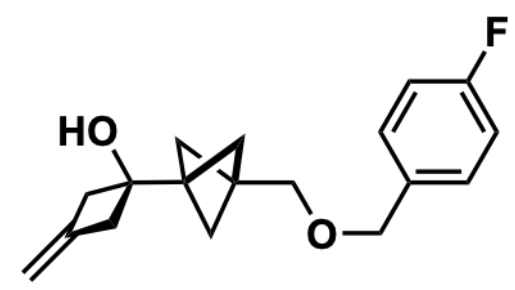

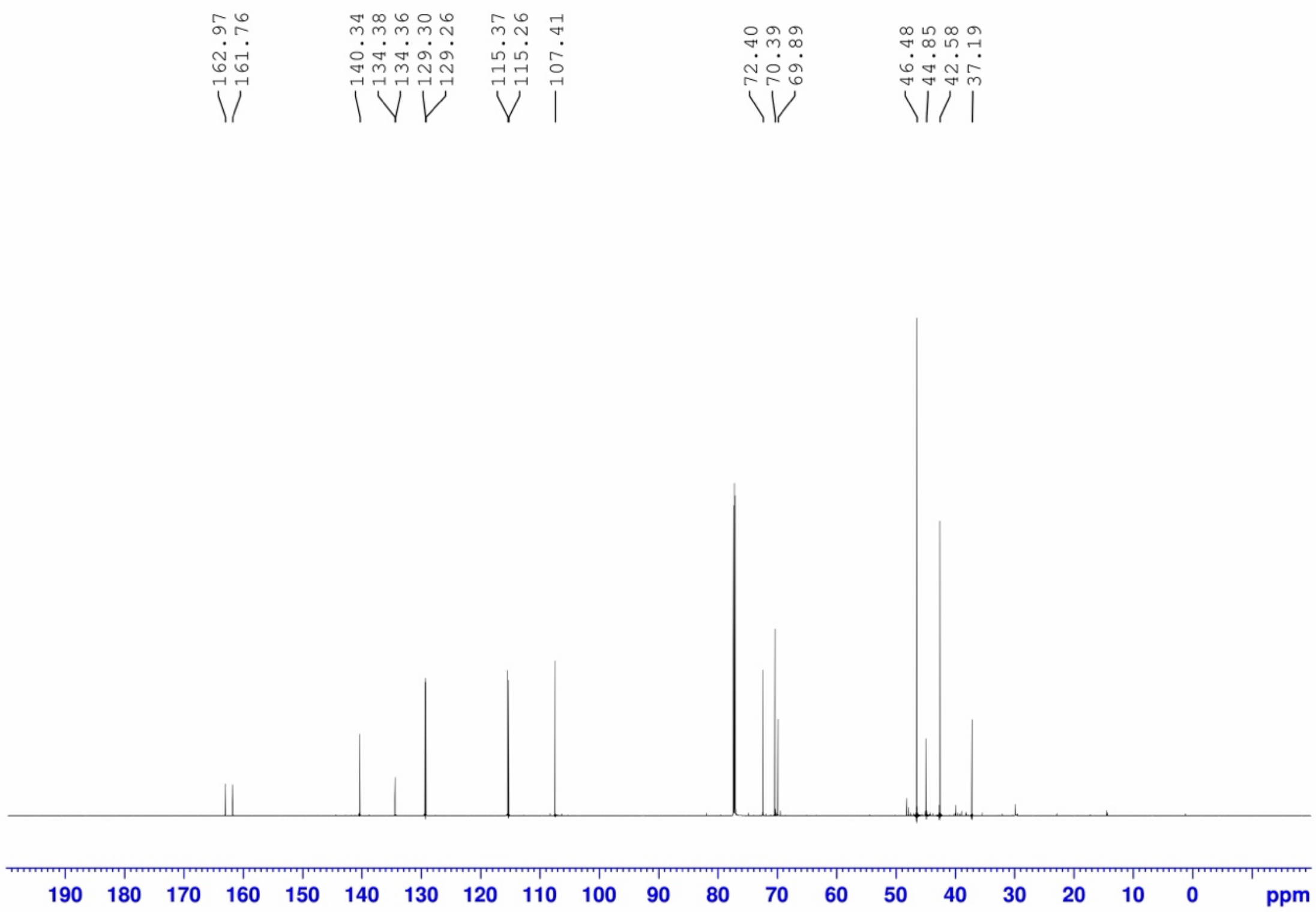

Figure S179. $^{13}C\{^{1}H\}$ NMR spectrum of 1-(3-(((4-fluorobenzyl)oxy)methyl)bicyclo[1.1.1]pentan-1-yl)-3-methylenecyclobutan-1-ol **(44)** in $CDCl_3$.

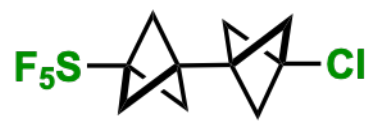

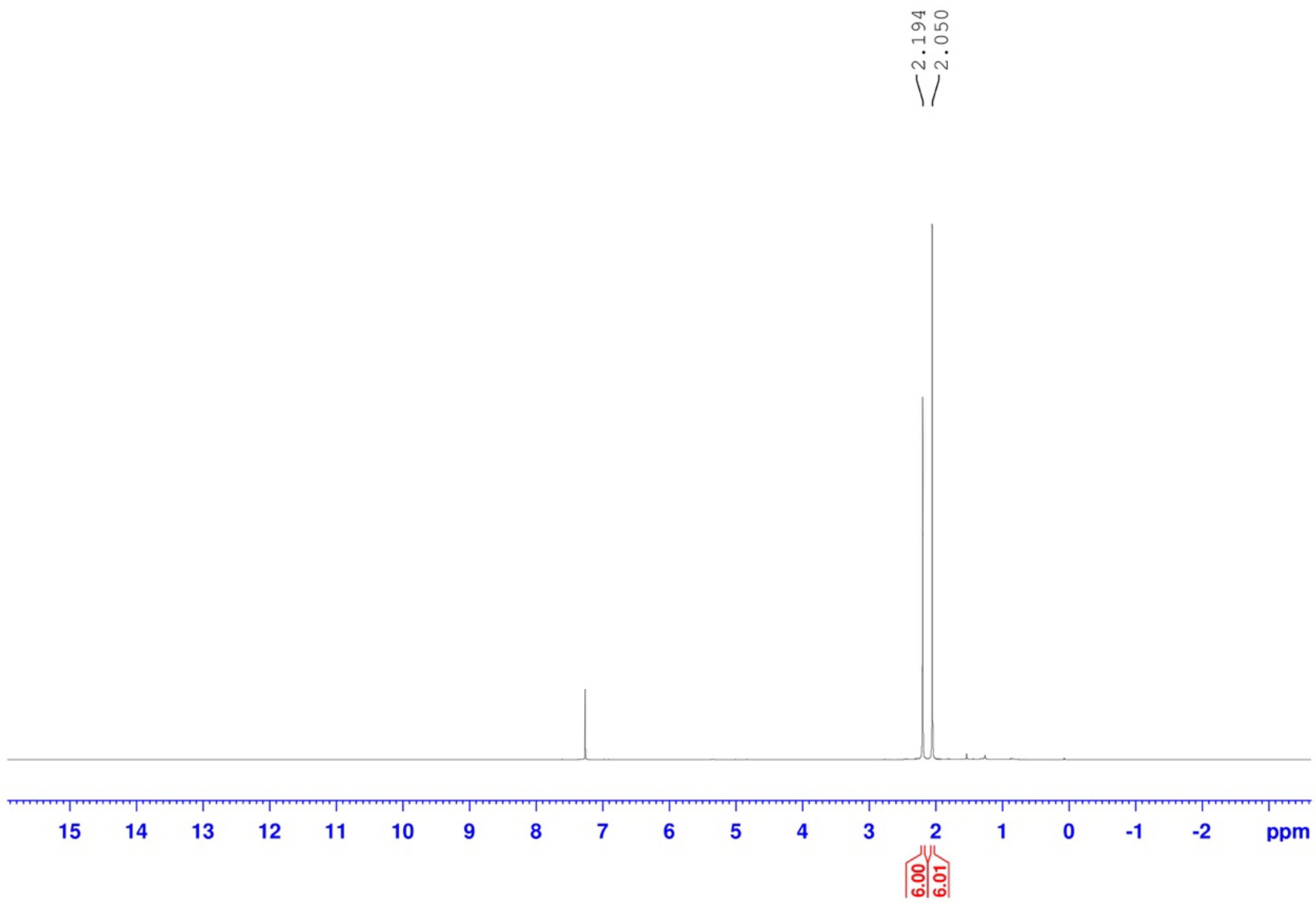

Figure S180. ^{1}H NMR spectrum of (3'-chloro-[1,1'-bi(bicyclo[1.1.1]pentan)]-3-yl)pentafluoro-λ^{6}-sulfane (**45**) in $CDCl_3$.

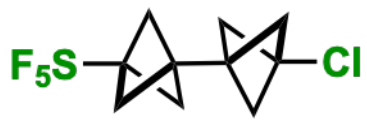

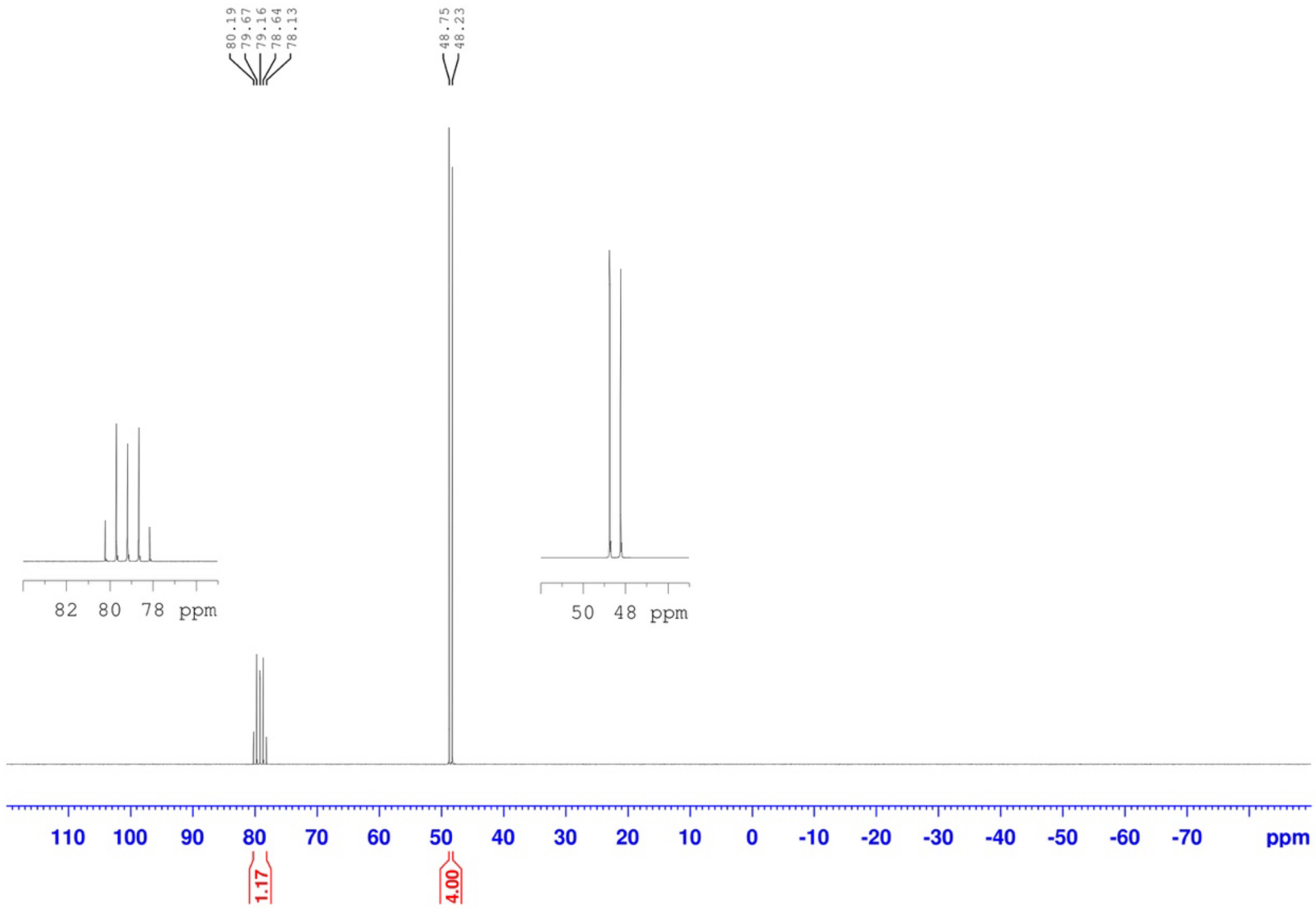

Figure S181. ^{19}F NMR spectrum of (3'-chloro-[1,1'-bi(bicyclo[1.1.1]pentan)]-3-yl)pentafluoro-λ^6-sulfane (**45**) in $CDCl_3$.

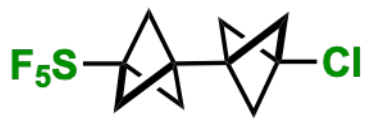

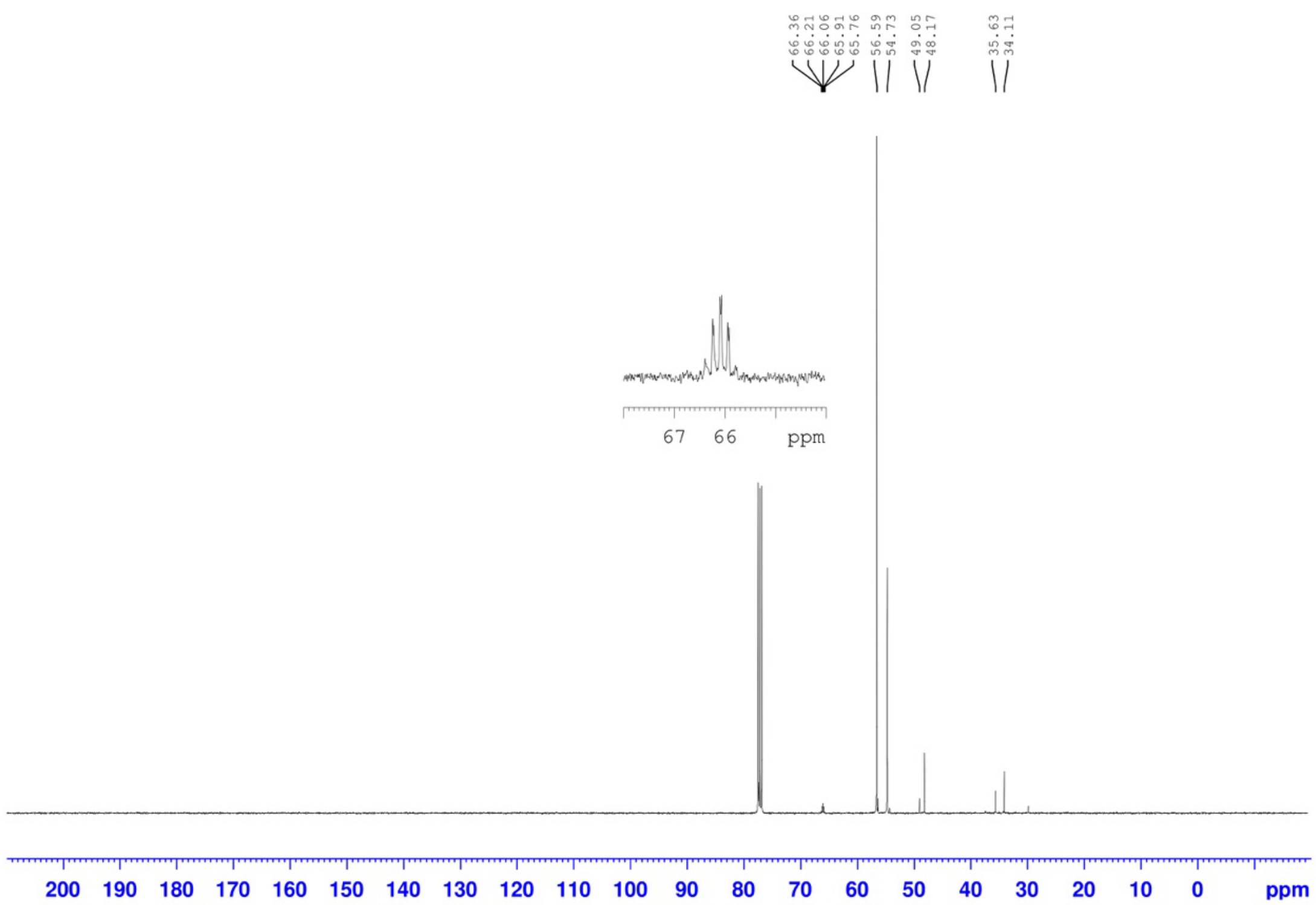

Figure S182. $^{13}C\{^1H\}$ NMR spectrum of (3'-chloro-[1,1'-bi(bicyclo[1.1.1]pentan)]-3-yl)pentafluoro-λ^6-sulfane (**45**) in $CDCl_3$.

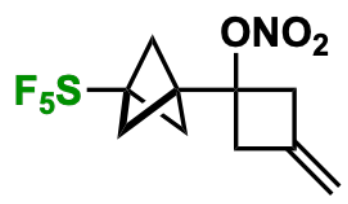

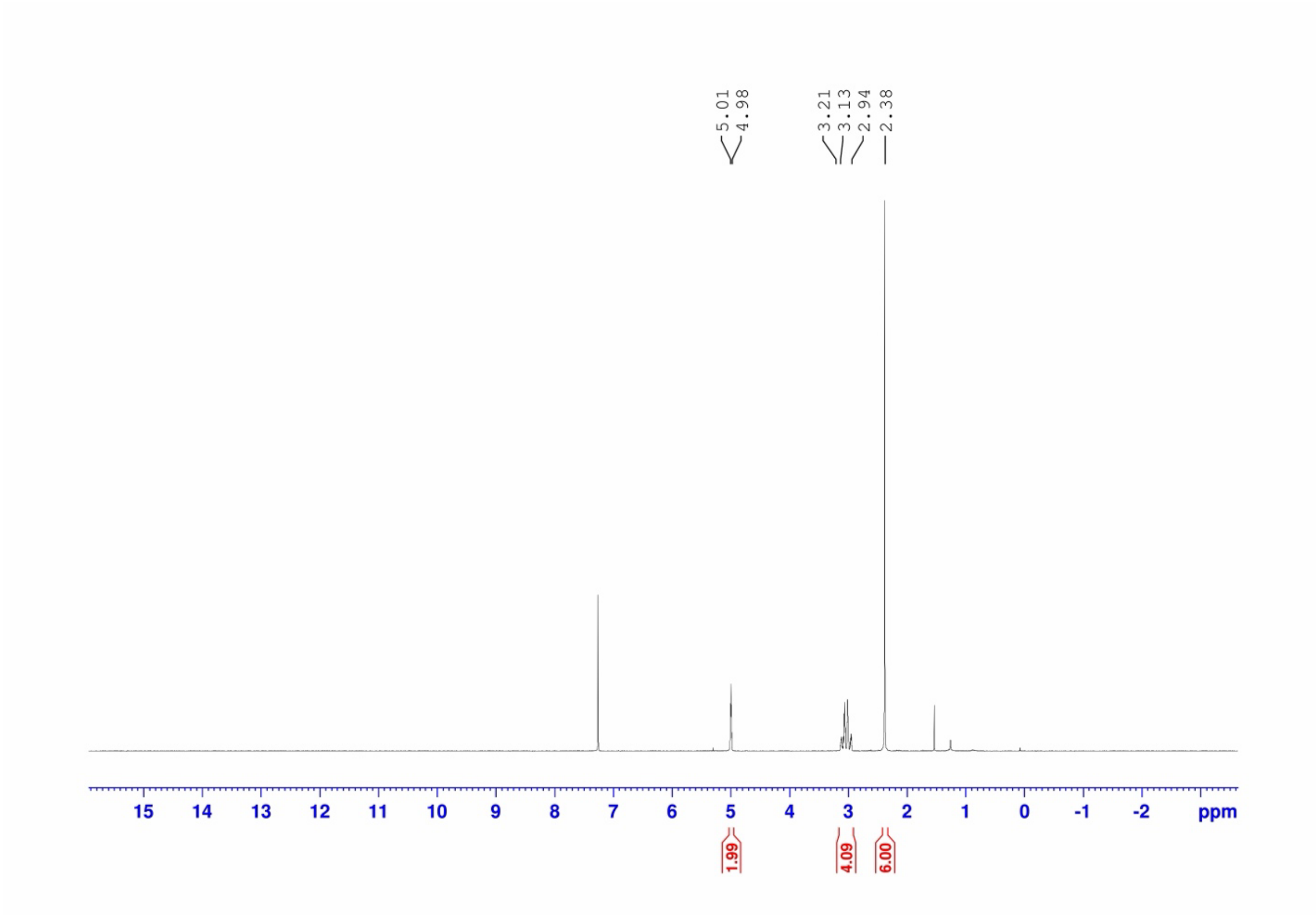

Figure S183. ^{1}H NMR spectrum of 3-methylene-1-(3-(pentafluoro-λ^6-sulfanyl)bicyclo[1.1.1]pentan-1-yl)cyclobutyl nitrate **(46)** in $CDCl_3$.

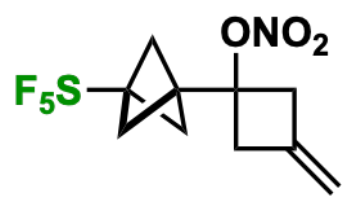

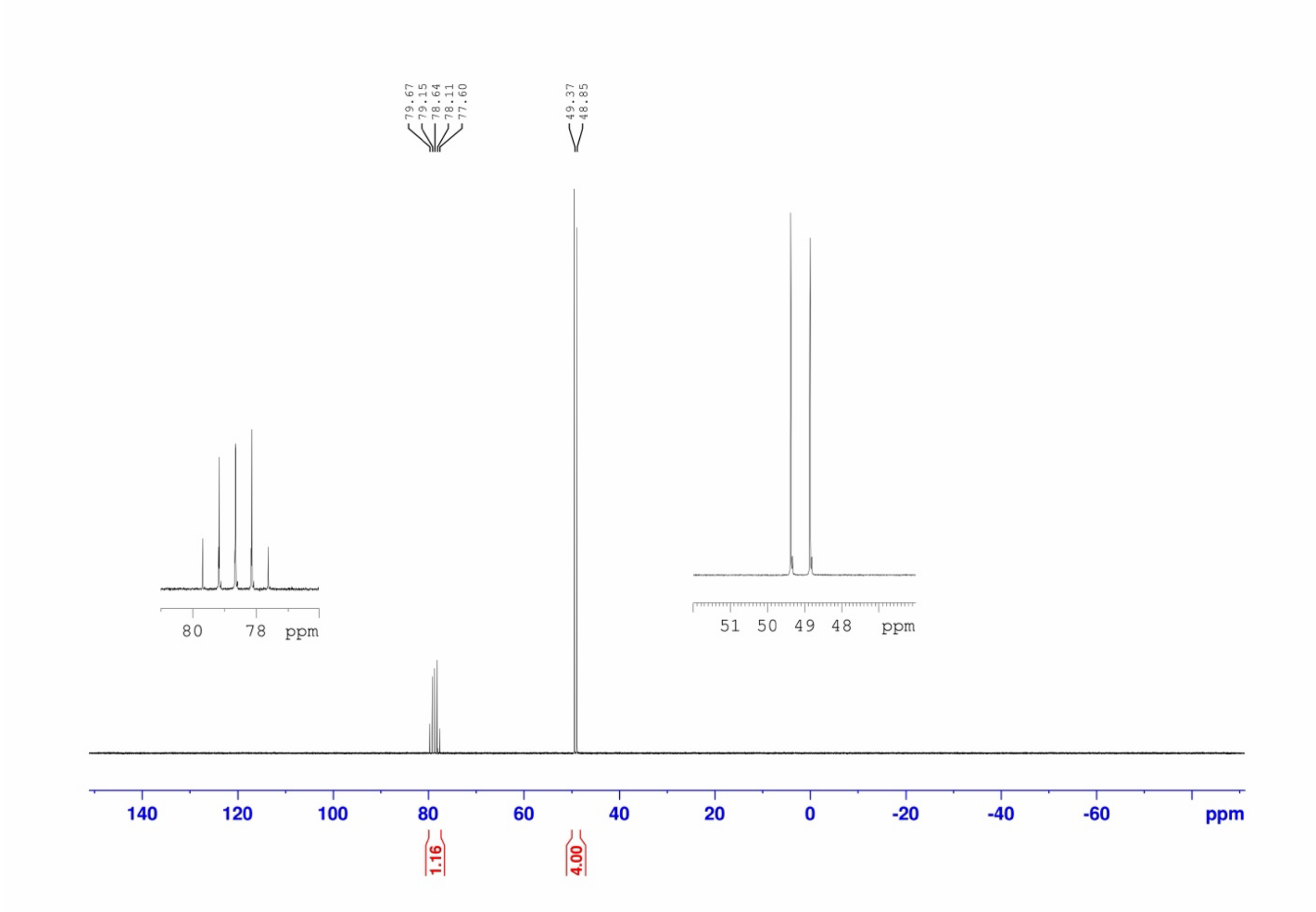

Figure S184. ^{19}F NMR spectrum of 3-methylene-1-(3-(pentafluoro-λ^6-sulfanyl)bicyclo[1.1.1]pentan-1-yl)cyclobutyl nitrate **(46)** in $CDCl_3$.

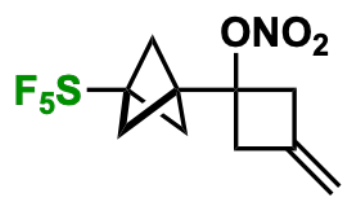

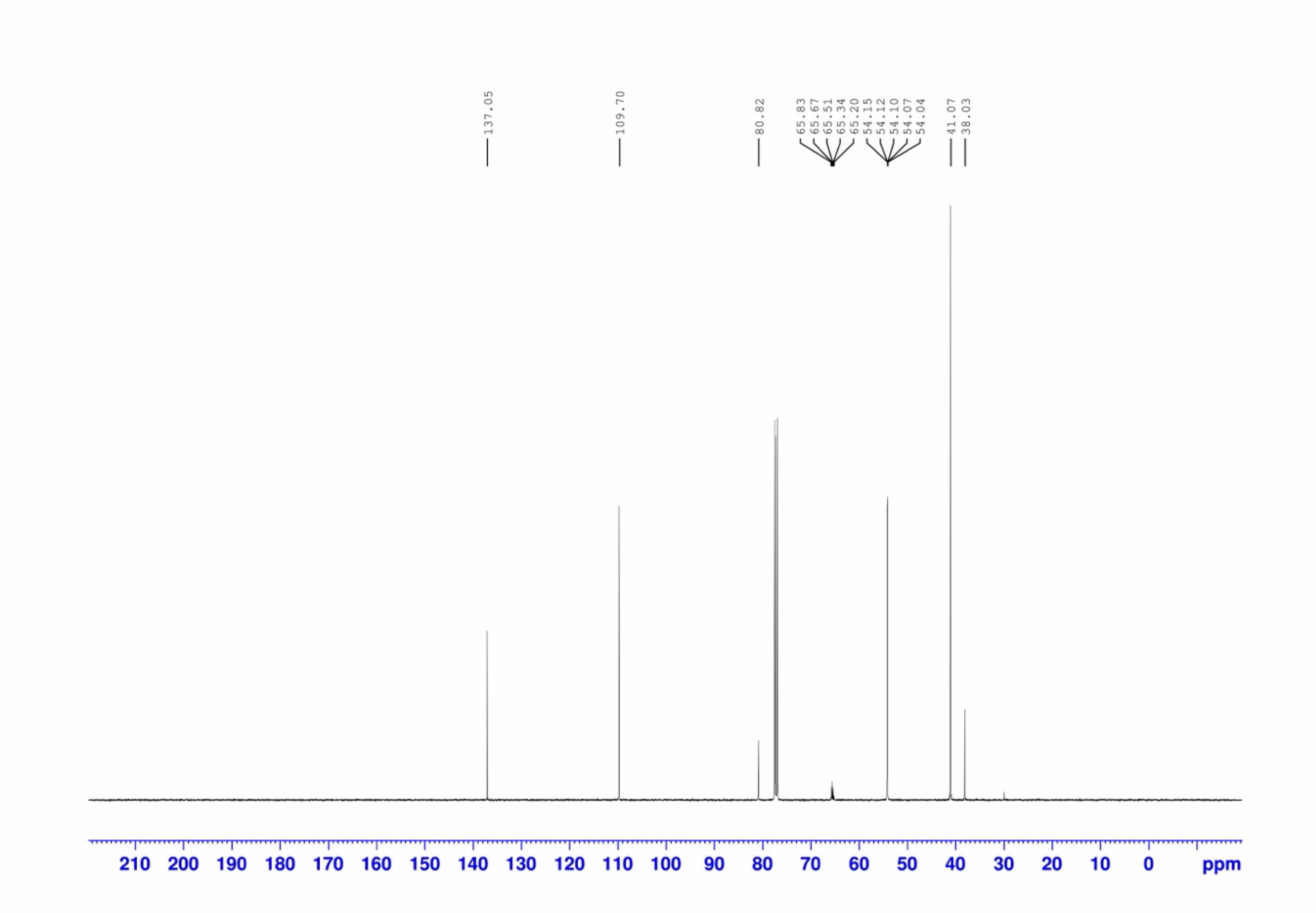

Figure S185. $^{13}C\{^1H\}$ NMR spectrum of 3-methylene-1-(3-(pentafluoro-λ^6-sulfanyl)bicyclo[1.1.1]pentan-1-yl)cyclobutyl nitrate **(46)** in $CDCl_3$.

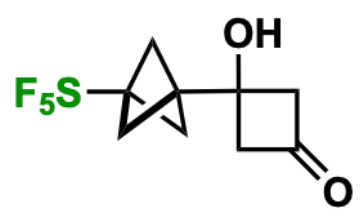

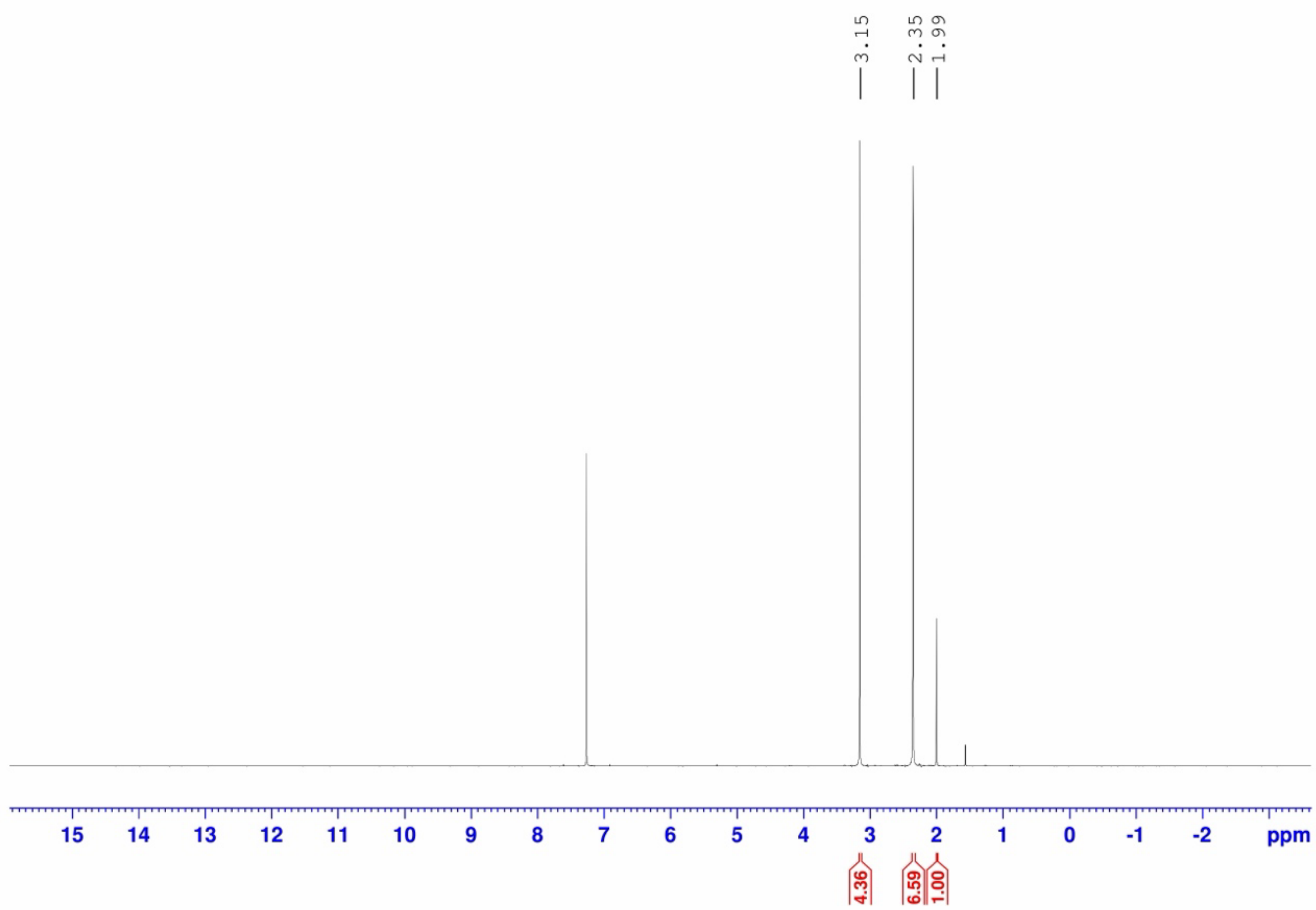

Figure S186. ^{1}H NMR spectrum of 3-hydroxy-3-(3-(pentafluoro-λ^6-sulfanyl)bicyclo[1.1.1]pentan-1-yl)cyclobutan-1-one **(47)** in $CDCl_3$.

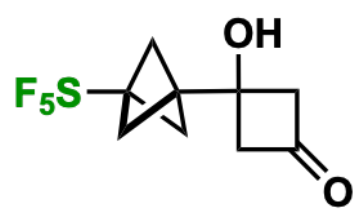

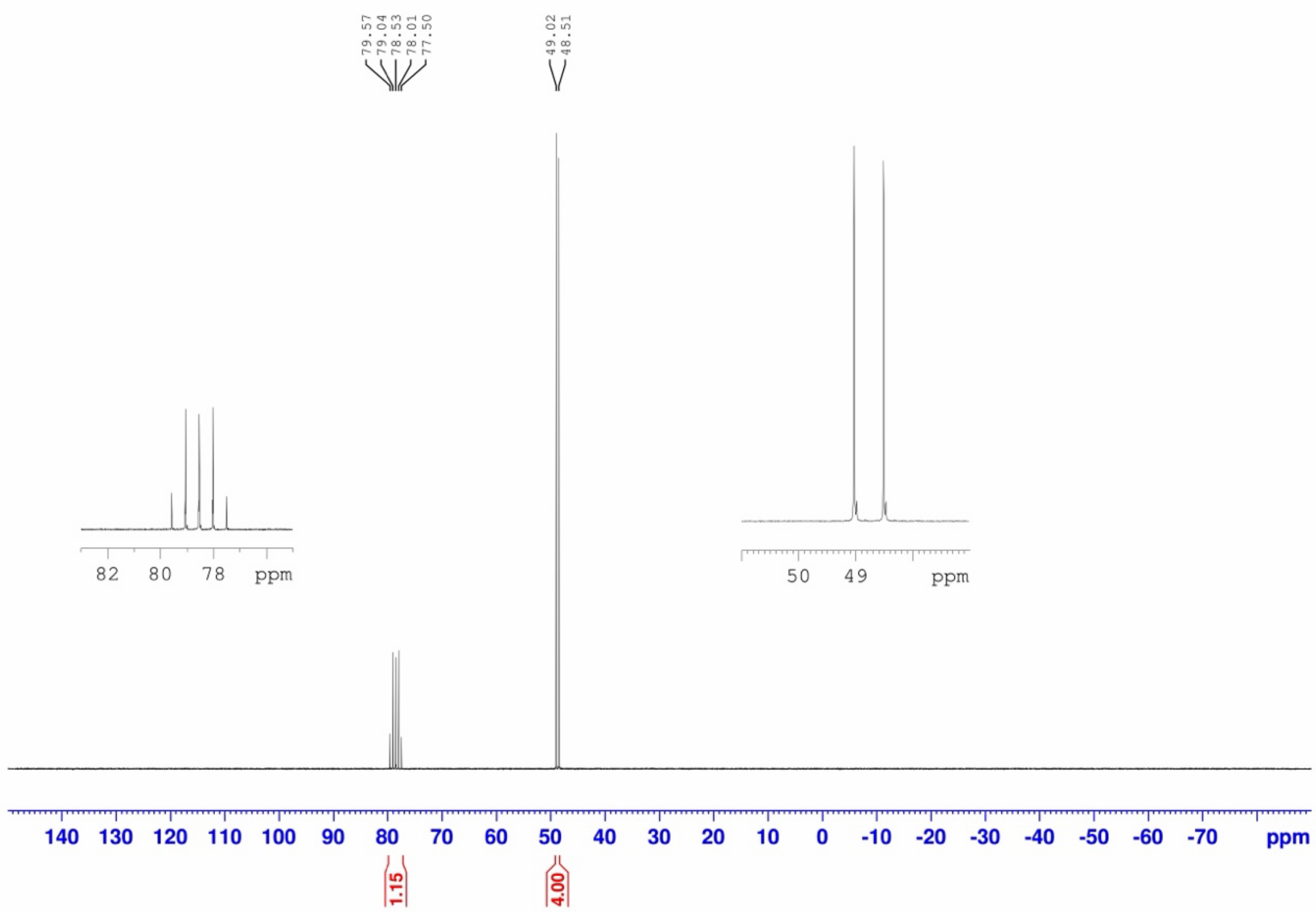

Figure S187. ^{19}F NMR spectrum of 3-hydroxy-3-(3-(pentafluoro-λ^6-sulfanyl)bicyclo[1.1.1]pentan-1-yl)cyclobutan-1-one **(47)** in $CDCl_3$.

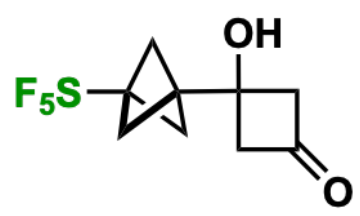

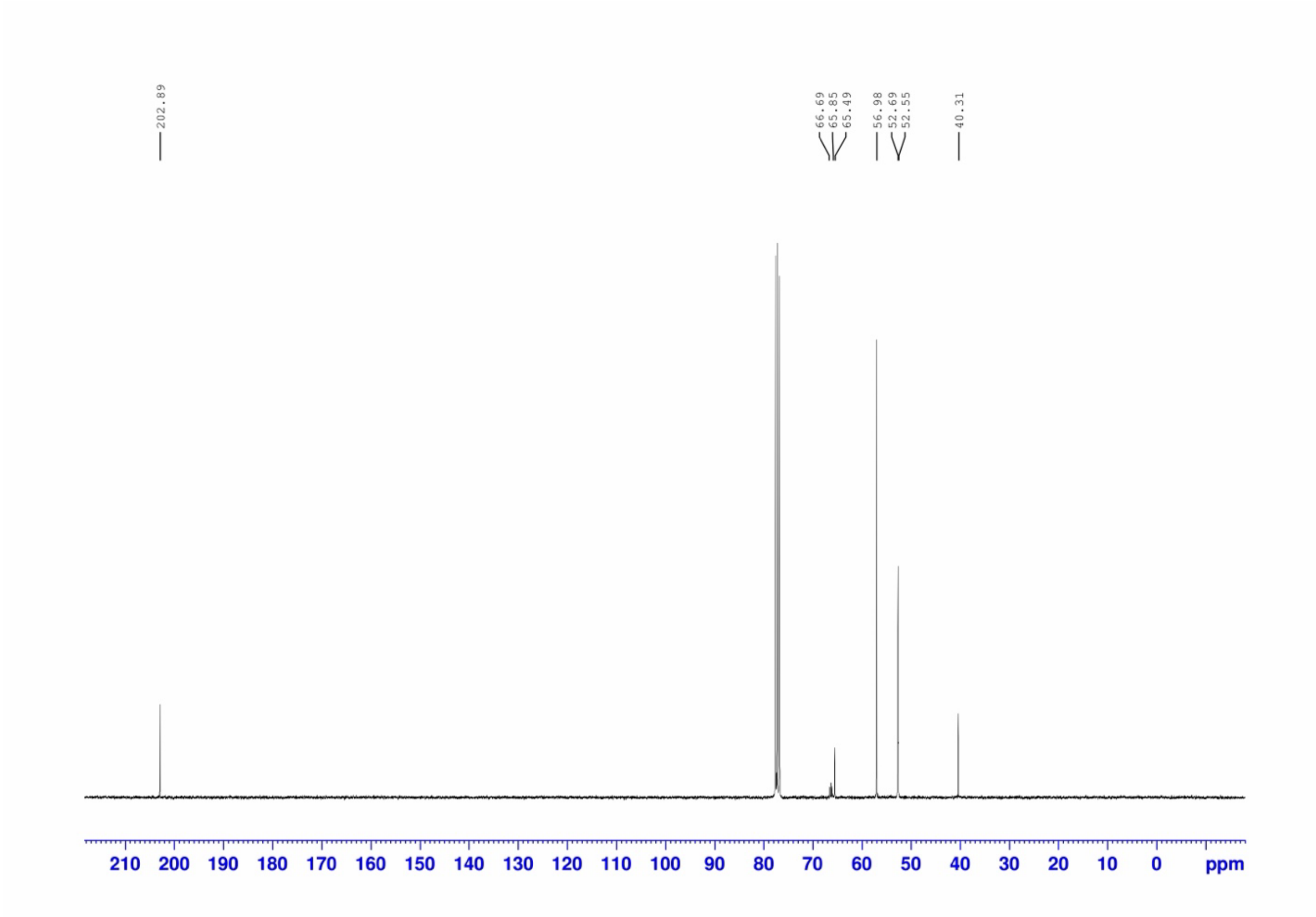

Figure S188. $^{13}C\{^1H\}$ NMR spectrum of 3-hydroxy-3-(3-(pentafluoro-λ^6-sulfanyl)bicyclo[1.1.1]pentan-1-yl)cyclobutan-1-one **(47)** in $CDCl_3$.

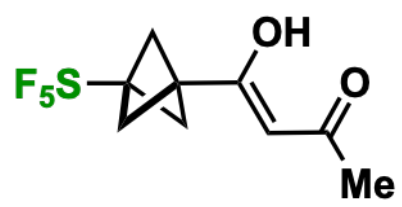

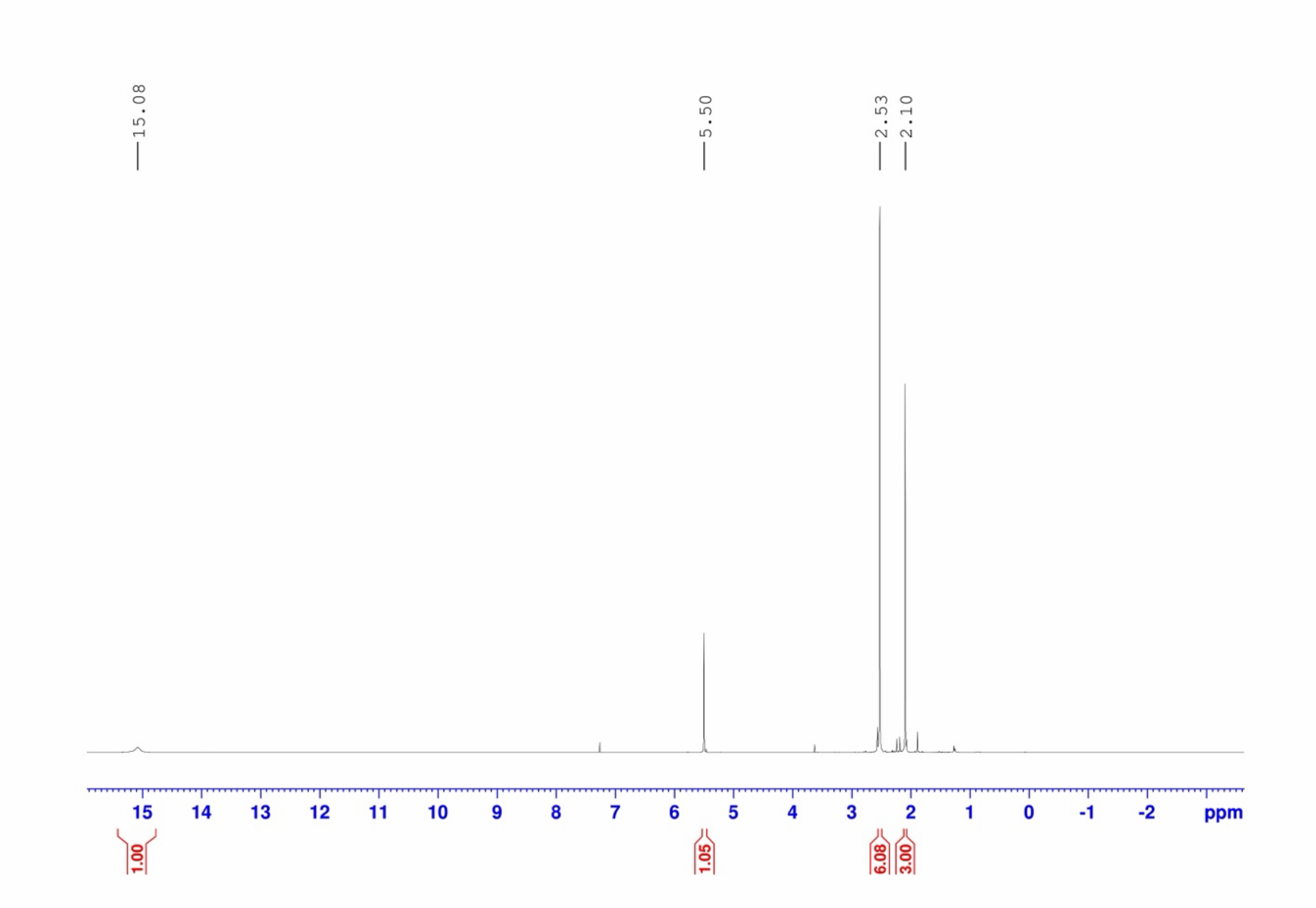

Figure S189. ^{1}H NMR spectrum of (*Z*)-4-hydroxy-4-(3-(pentafluoro-λ^6-sulfanyl)bicyclo[1.1.1]pentan-1-yl)but-3-en-2-one **(48)** in $CDCl_3$.

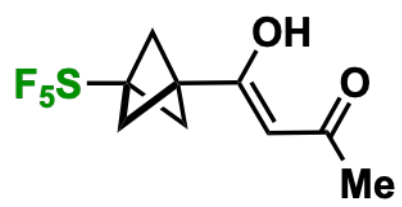

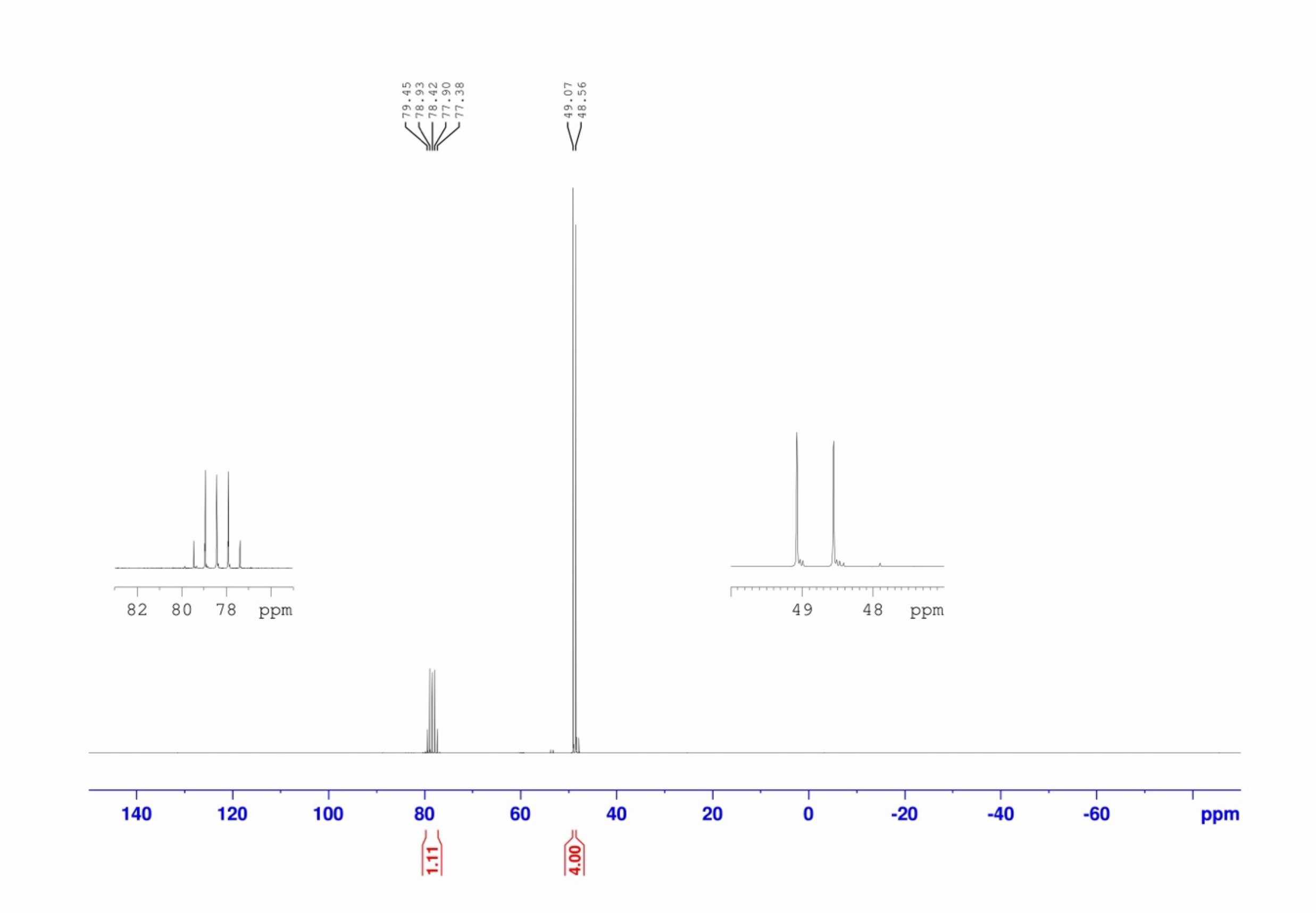

Figure S190. ^{19}F NMR spectrum of (*Z*)-4-hydroxy-4-(3-(pentafluoro-λ^6-sulfanyl)bicyclo[1.1.1]pentan-1-yl)but-3-en-2-one **(48)** in $CDCl_3$.

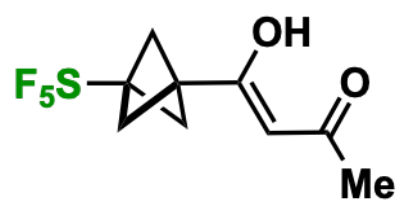

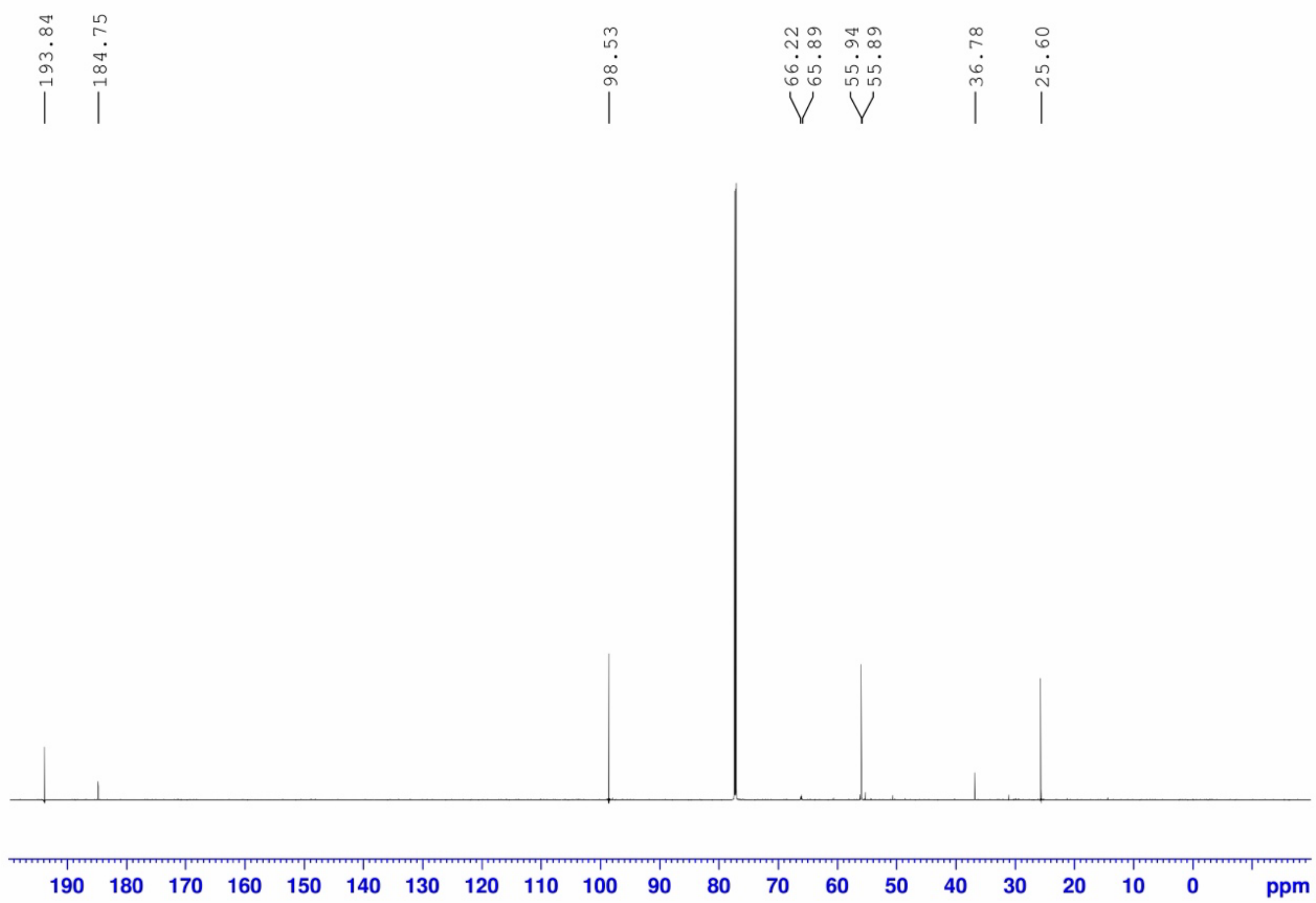

Figure S191. $^{13}C\{^1H\}$ NMR spectrum of (*Z*)-4-hydroxy-4-(3-(pentafluoro-λ^6-sulfanyl)bicyclo[1.1.1]pentan-1-yl)but-3-en-2-one **(48)** in $CDCl_3$.

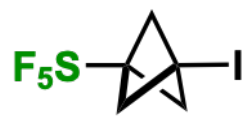

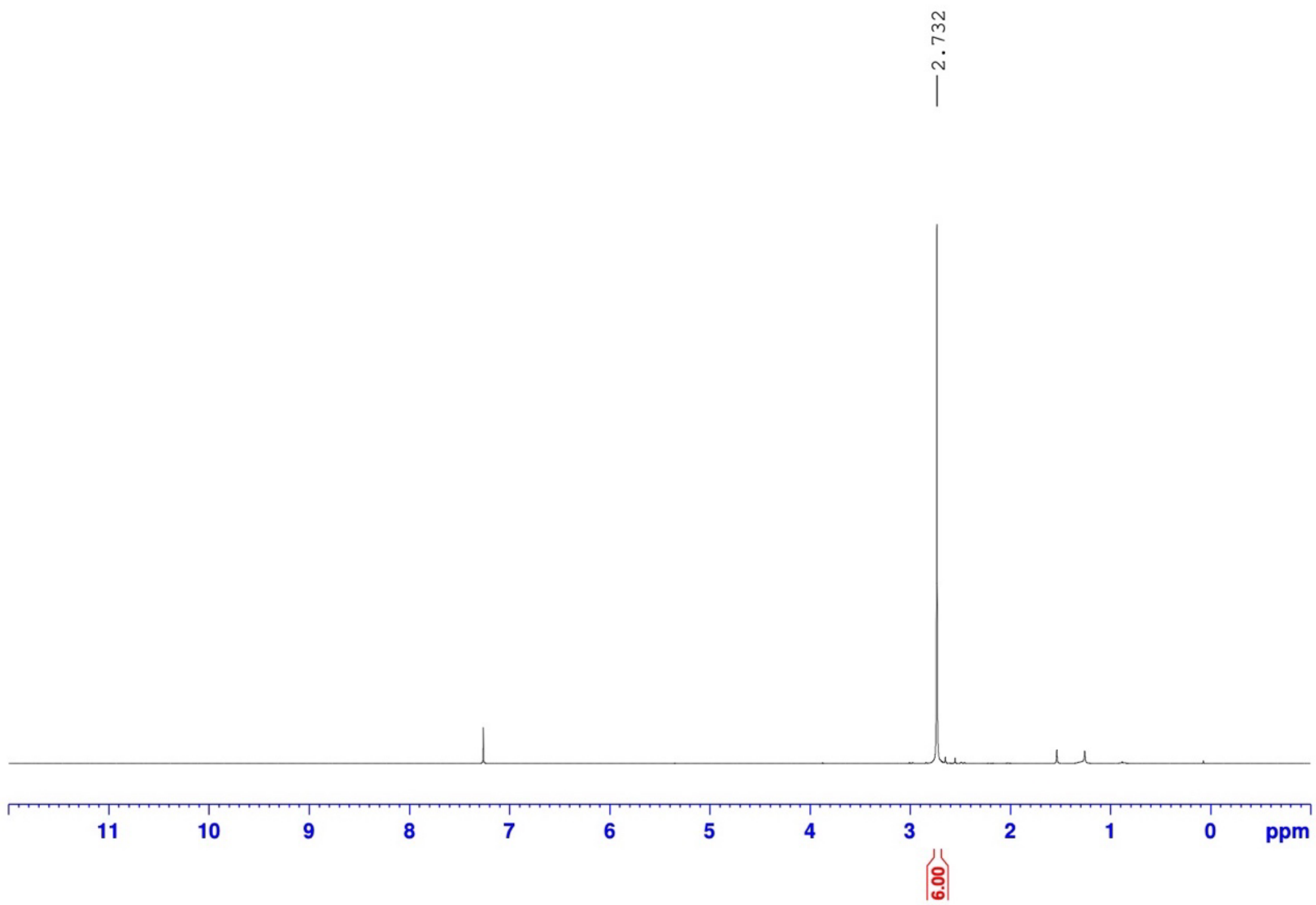

Figure S192.^{1}H NMR spectrum of pentafluoro(3-iodobicyclo[1.1.1]pentan-1-yl)-λ[6]-sulfane (**2**) in $CDCl_3$.

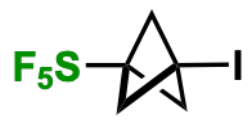

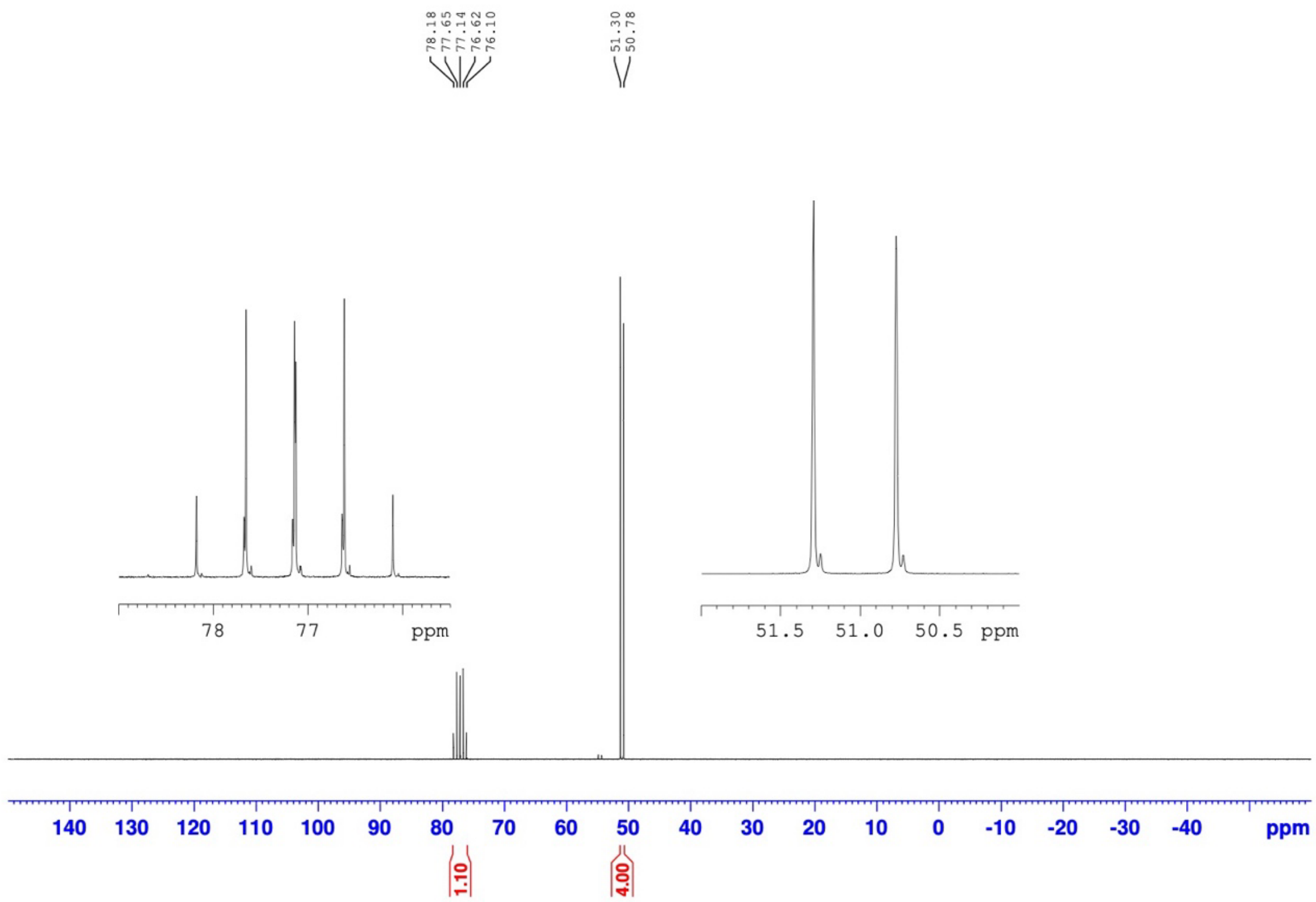

Figure S193. ^{19}F NMR spectrum of pentafluoro(3-iodobicyclo[1.1.1]pentan-1-yl)-λ^6-sulfane (**2**) in $CDCl_3$.

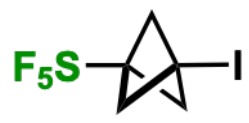

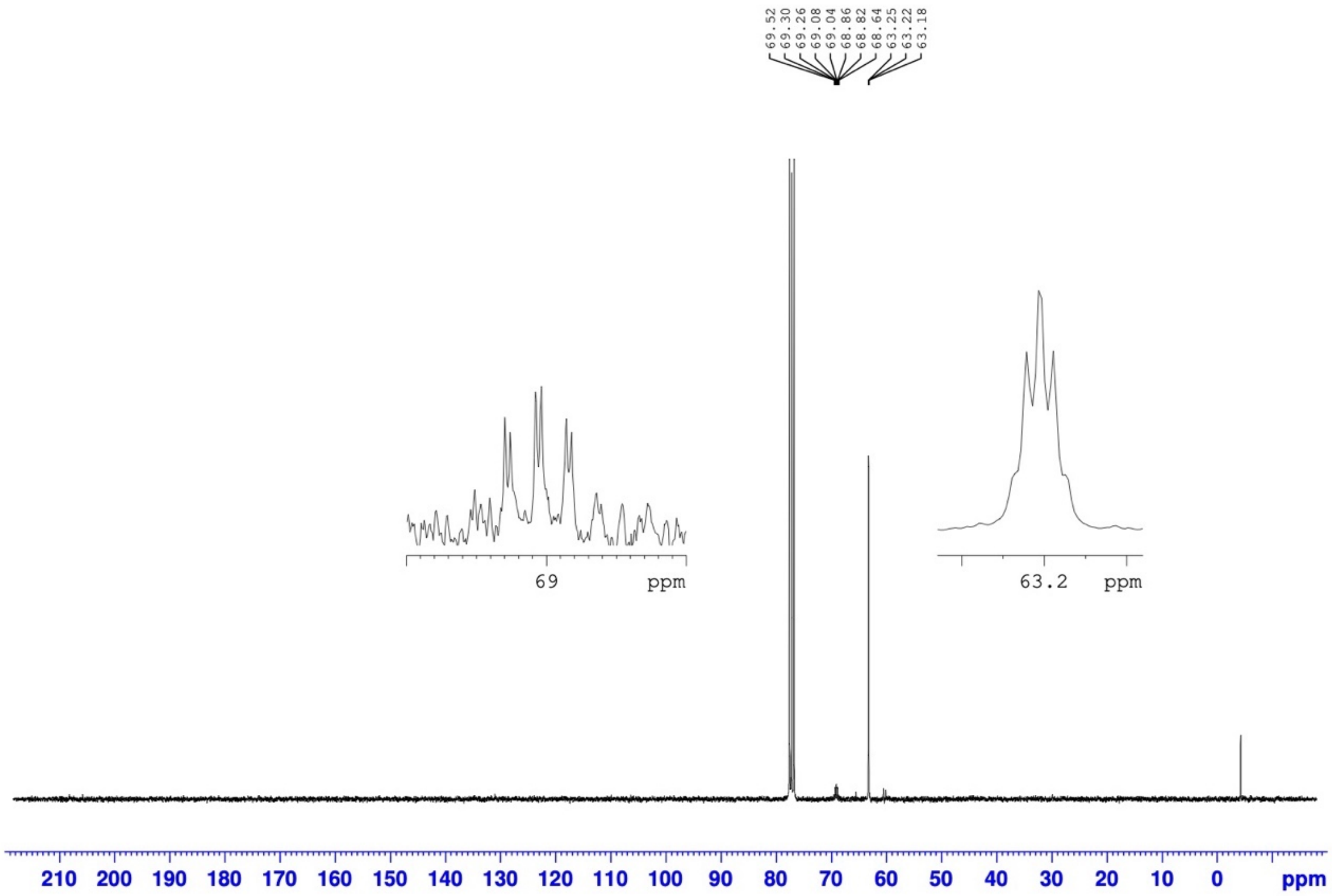

Figure S194. $^{13}C\{^{1}H\}$ NMR spectrum of pentafluoro(3-iodobicyclo[1.1.1]pentan-1-yl)-λ^6-sulfane (**2**) in $CDCl_3$.

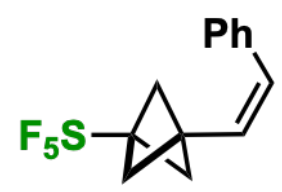

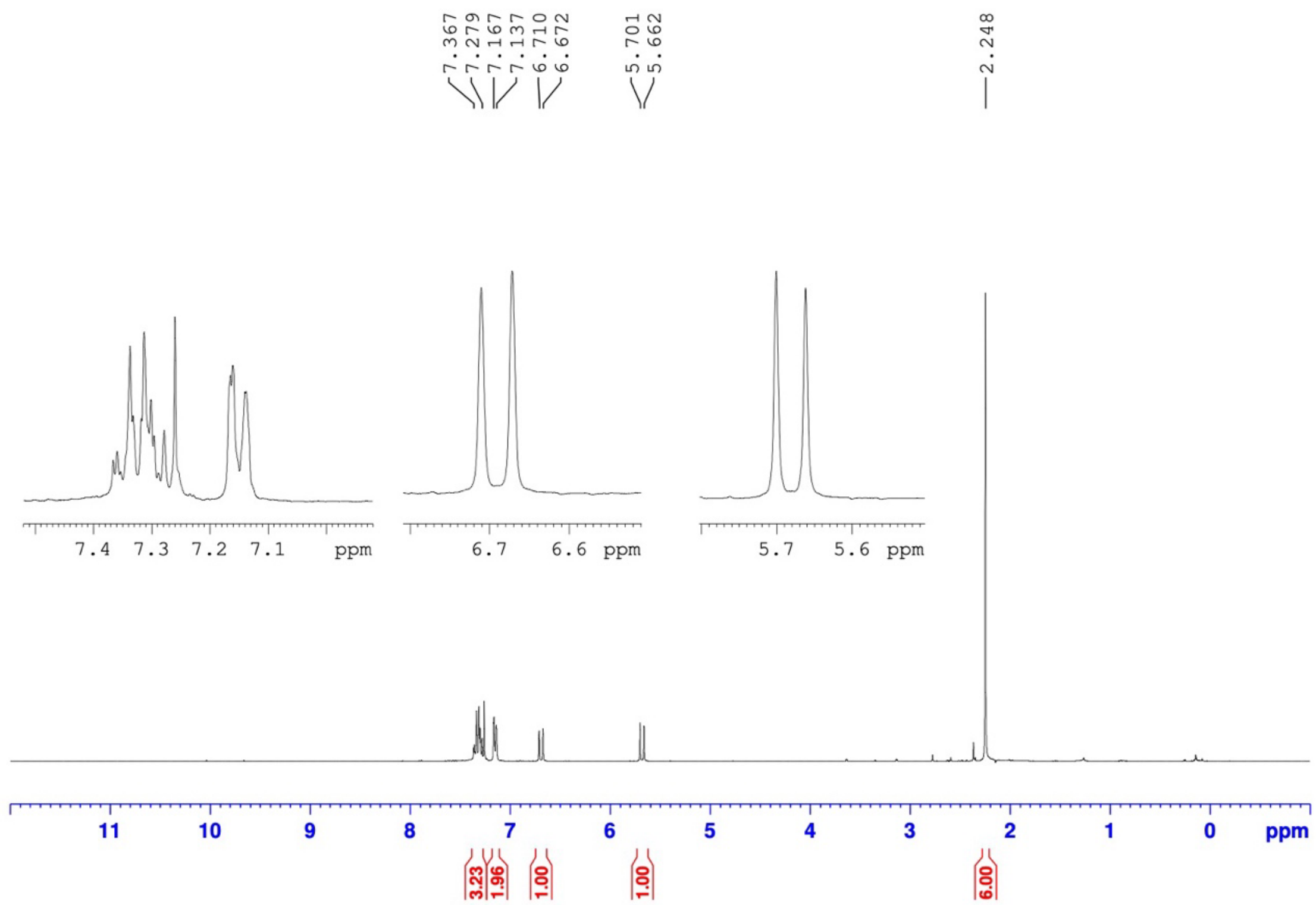

Figure S195. ^1H NMR spectrum of (*Z*)-pentafluoro(3-styrylbicyclo[1.1.1]pentan-1-yl)-λ^6-sulfane (**S-22**) in $CDCl_3$.

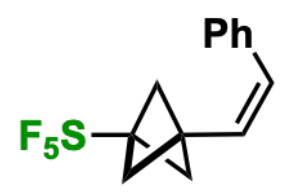

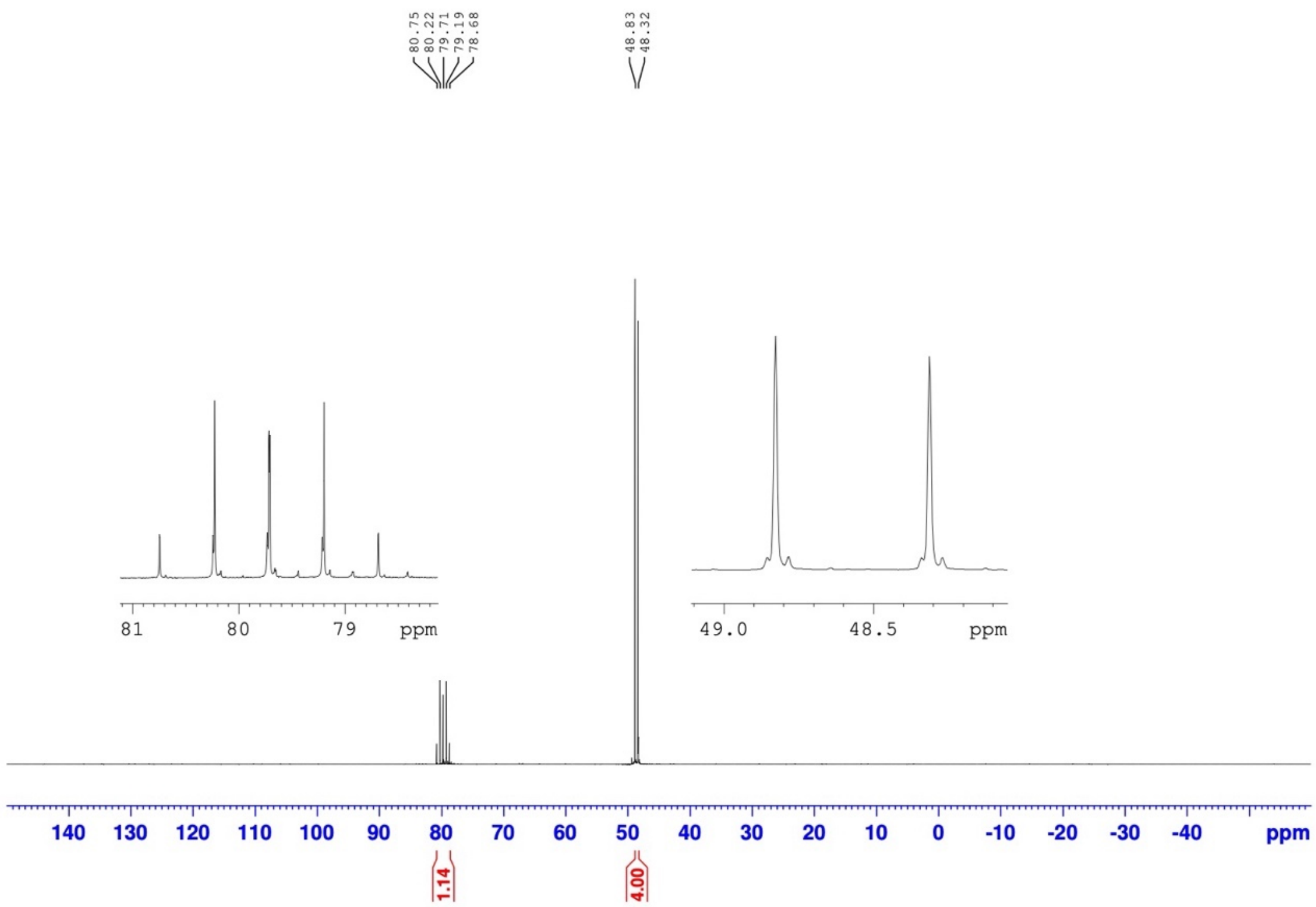

Figure S196. ^{19}F NMR spectrum of (*Z*)-pentafluoro(3-styrylbicyclo[1.1.1]pentan-1-yl)-λ^6-sulfane (**S-22**) in $CDCl_3$.

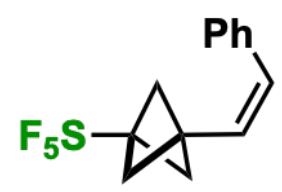

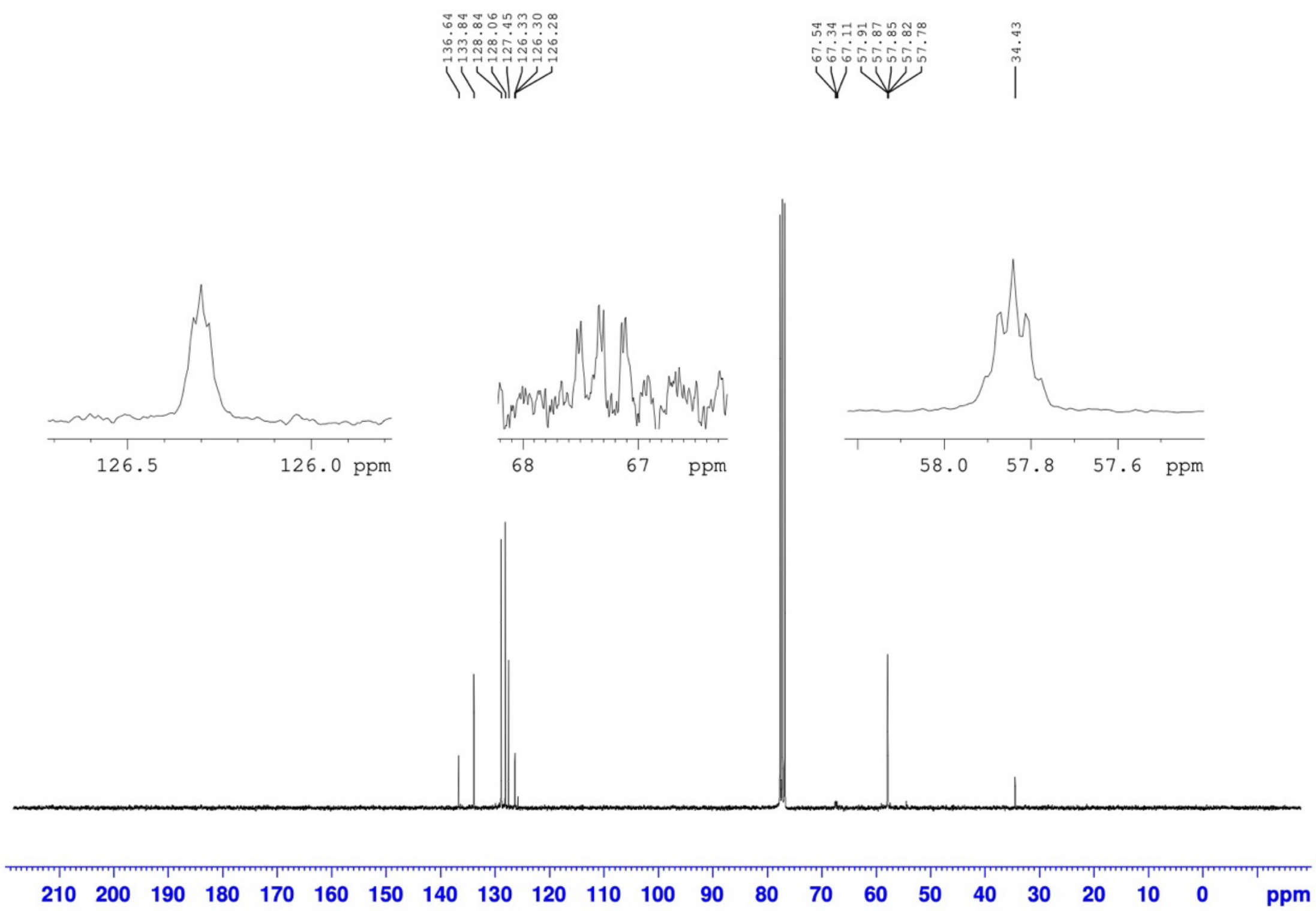

Figure S197. $^{13}C\{^{1}H\}$ NMR (*Z*)-pentafluoro(3-styrylbicyclo[1.1.1]pentan-1-yl)-λ^6-sulfane (**S-22**) in $CDCl_3$.

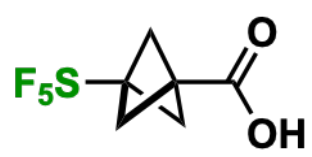

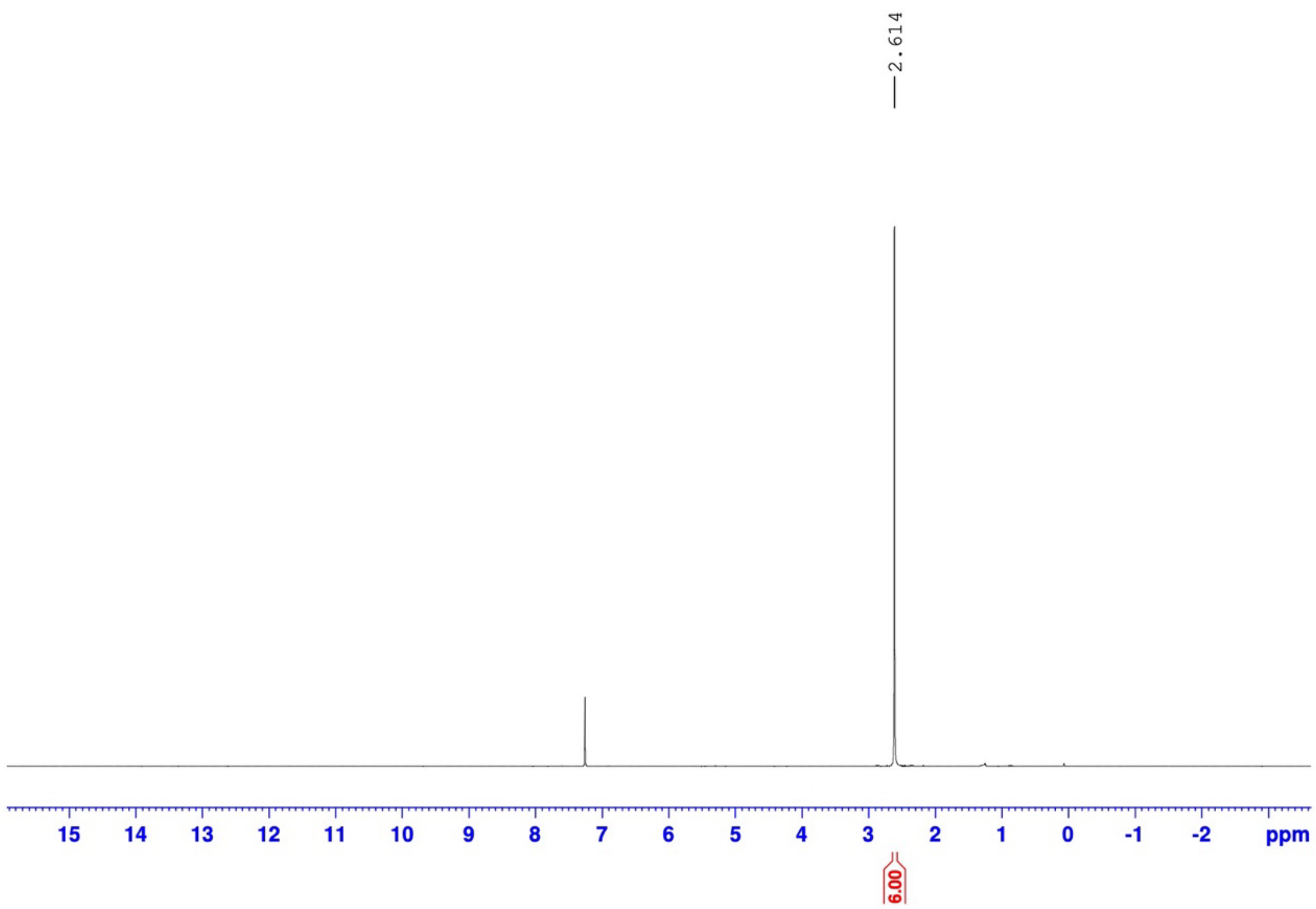

Figure S198. ^{1}H NMR spectrum of 3-(pentafluoro-λ[6]-sulfanyl)bicyclo[1.1.1]pentane-1-carboxylic acid (**4**) in $CDCl_3$.

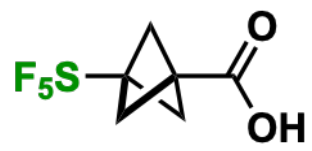

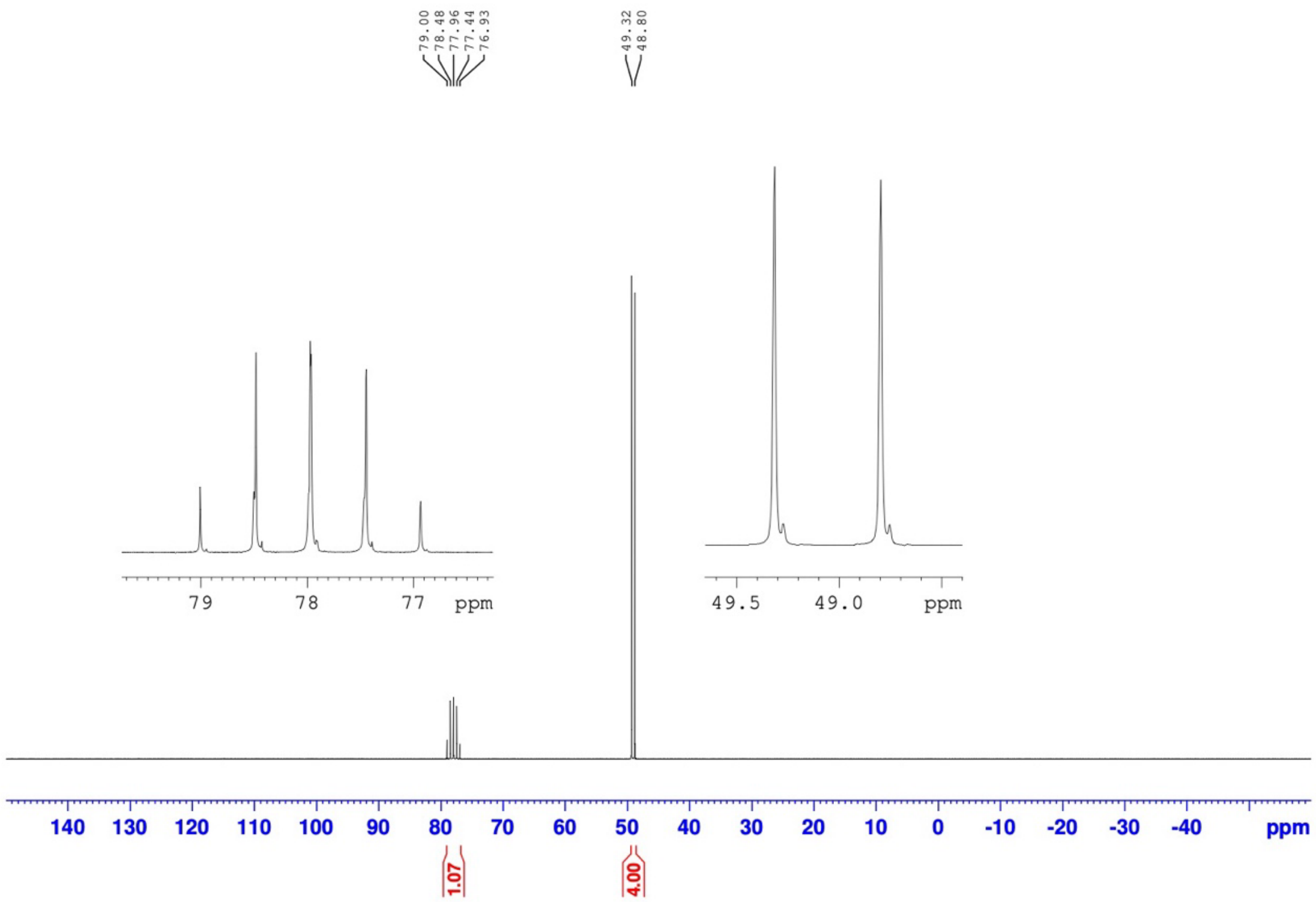

Figure S199. ^{19}F NMR spectrum of 3-(pentafluoro-λ^6-sulfanyl)bicyclo[1.1.1]pentane-1-carboxylic acid (**4**) in $CDCl_3$.

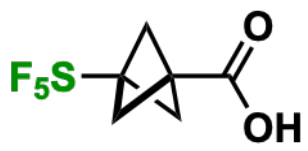

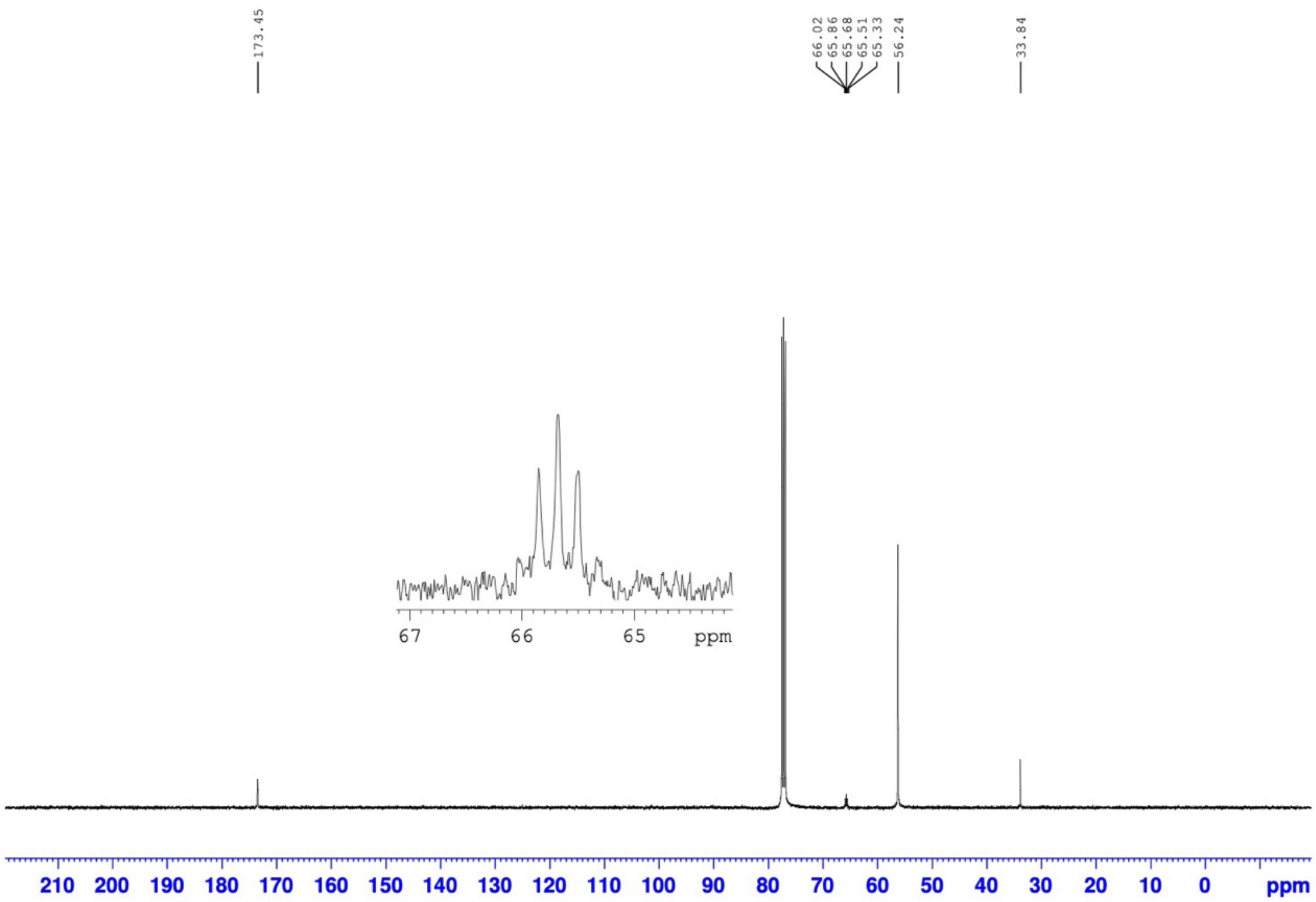

Figure S200. $^{13}C\{^{1}H\}$ NMR spectrum of 3-(pentafluoro-λ^6-sulfanyl)bicyclo[1.1.1]pentane-1-carboxylic acid (**4**) in $CDCl_3$.

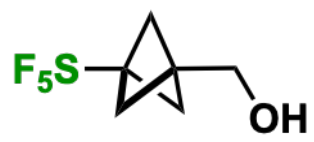

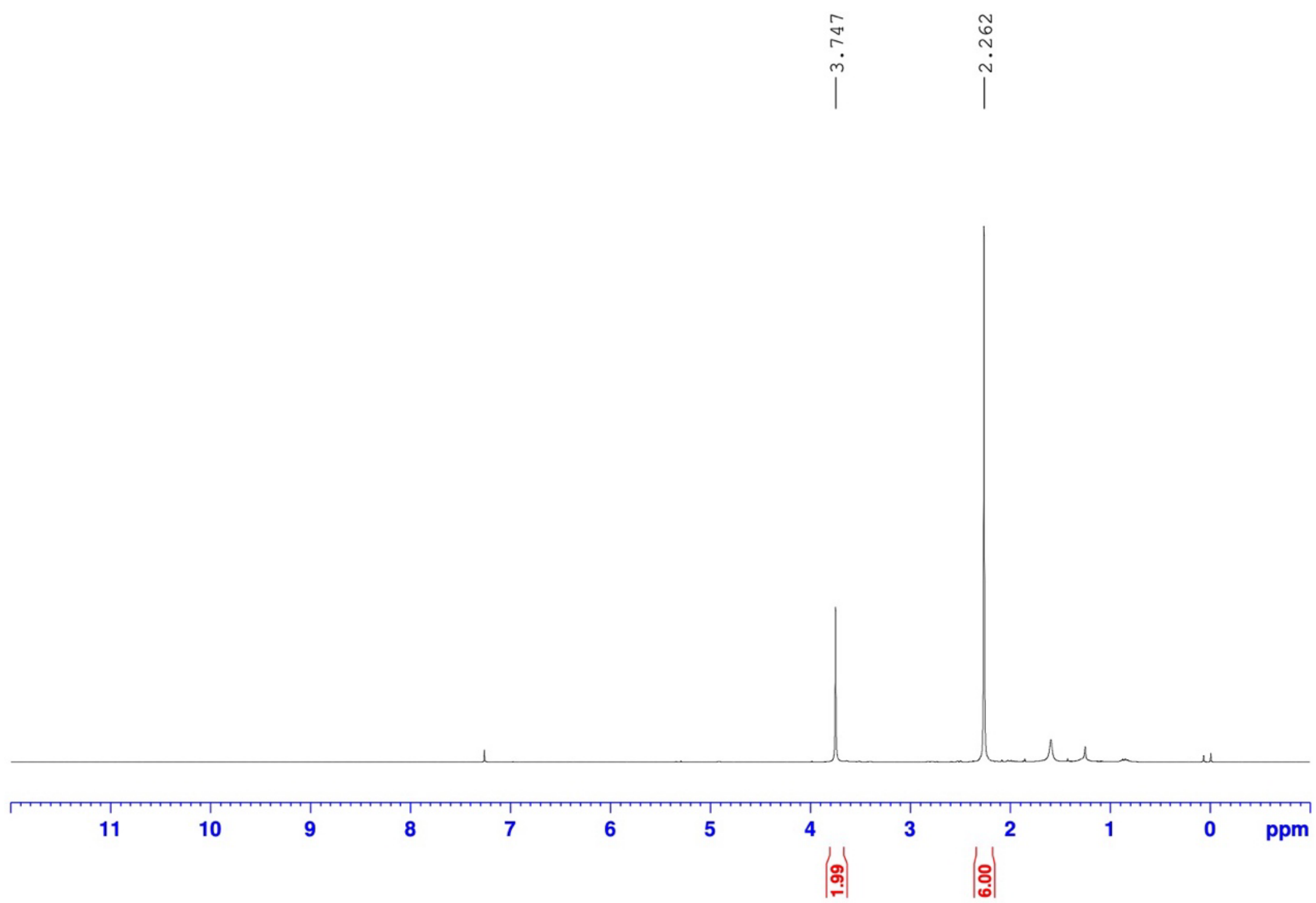

Figure S201. ^{1}H NMR spectrum of (3-(pentafluoro-λ^{6}-sulfanyl)bicyclo[1.1.1]pentan-1-yl)methanol (**14**) in $CDCl_3$.

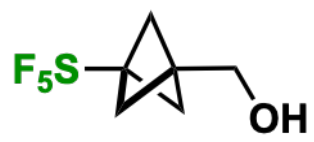

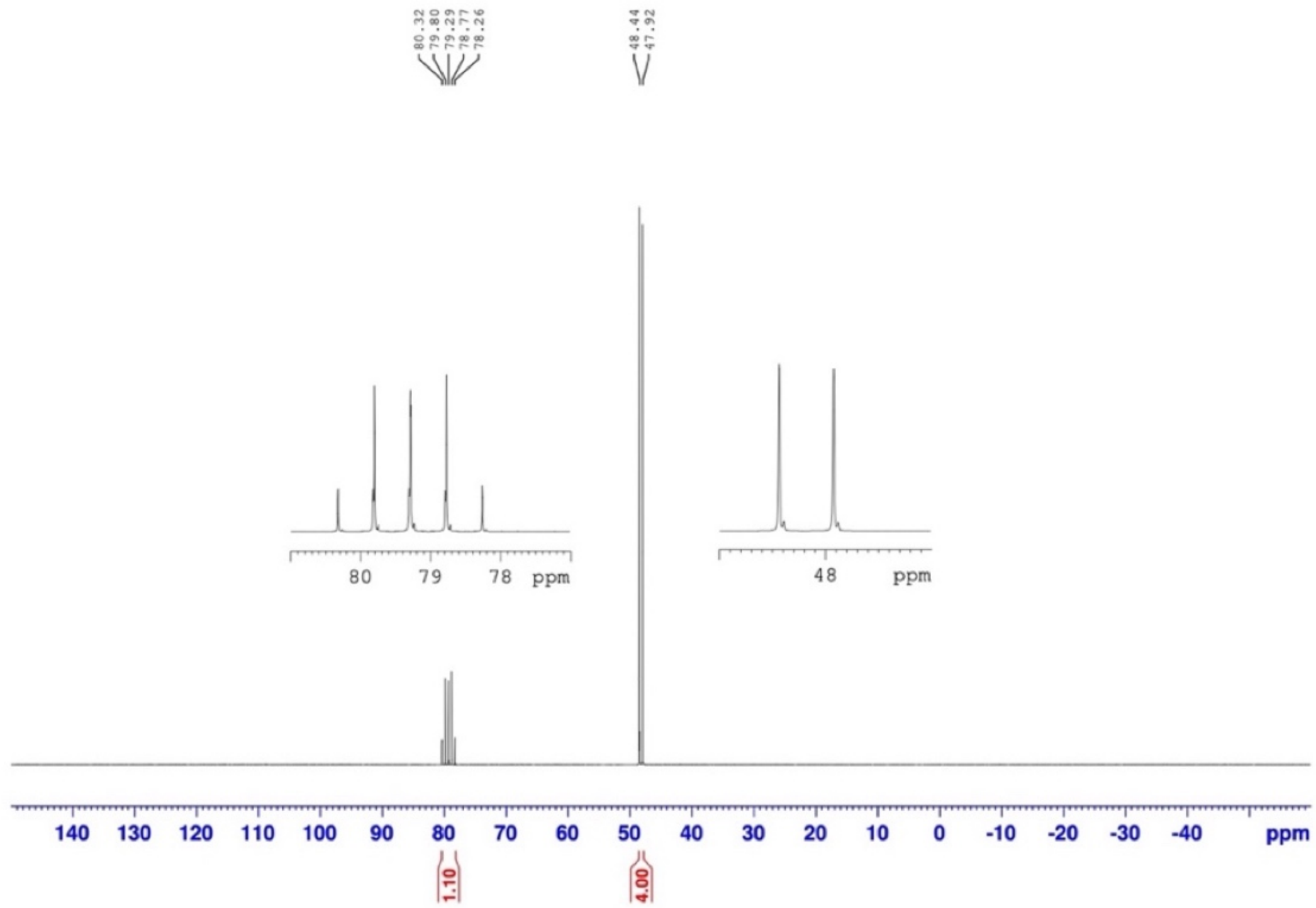

Figure S202. ^{19}F NMR spectrum of (3-(pentafluoro-λ^6-sulfanyl)bicyclo[1.1.1]pentan-1-yl)methanol (**14**) in $CDCl_3$.

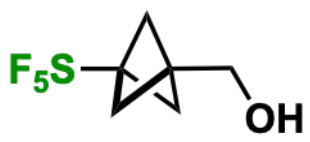

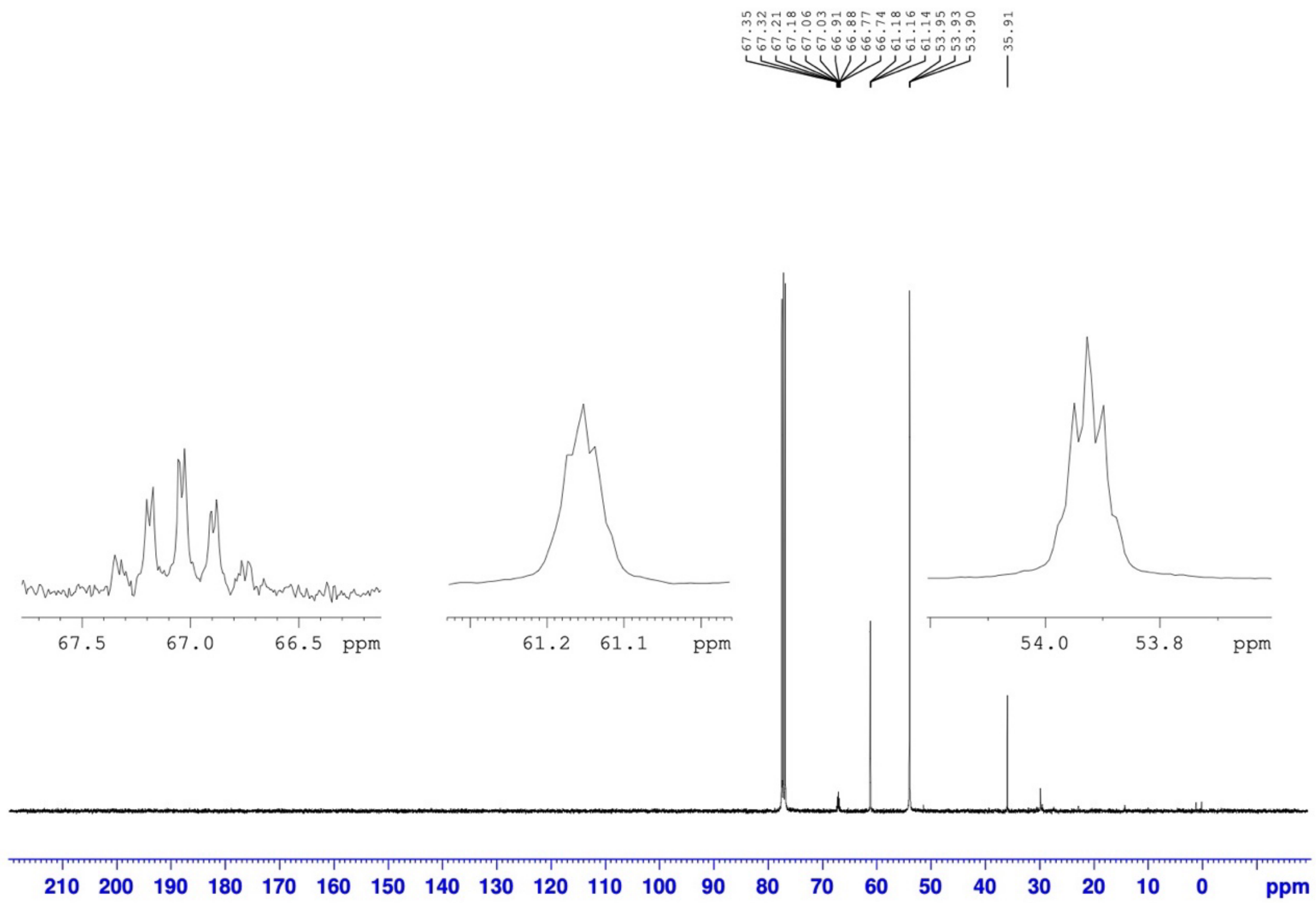

Figure S203. $^{13}C\{^1H\}$ NMR spectrum of (3-(pentafluoro-λ^6-sulfanyl)bicyclo[1.1.1]pentan-1-yl)methanol (**14**) in $CDCl_3$.

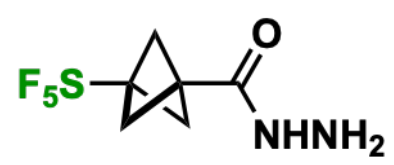

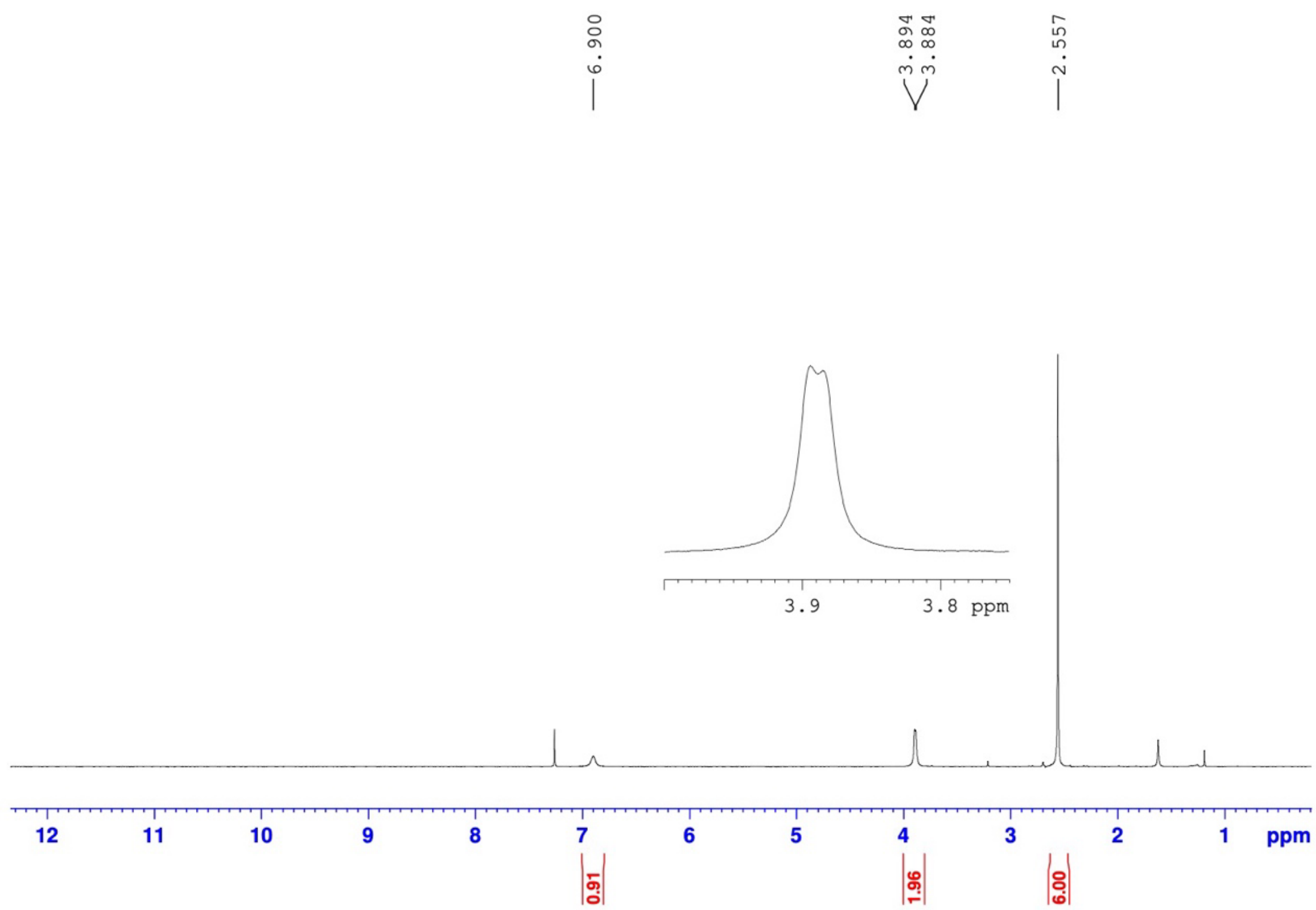

Figure S204. ^{1}H NMR spectrum of (3-(pentafluoroλ^6-sulfanyl)bicyclo[1.1.1]pentane-1-carbohydrazide (**18**) in $CDCl_3$.

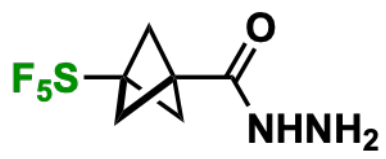

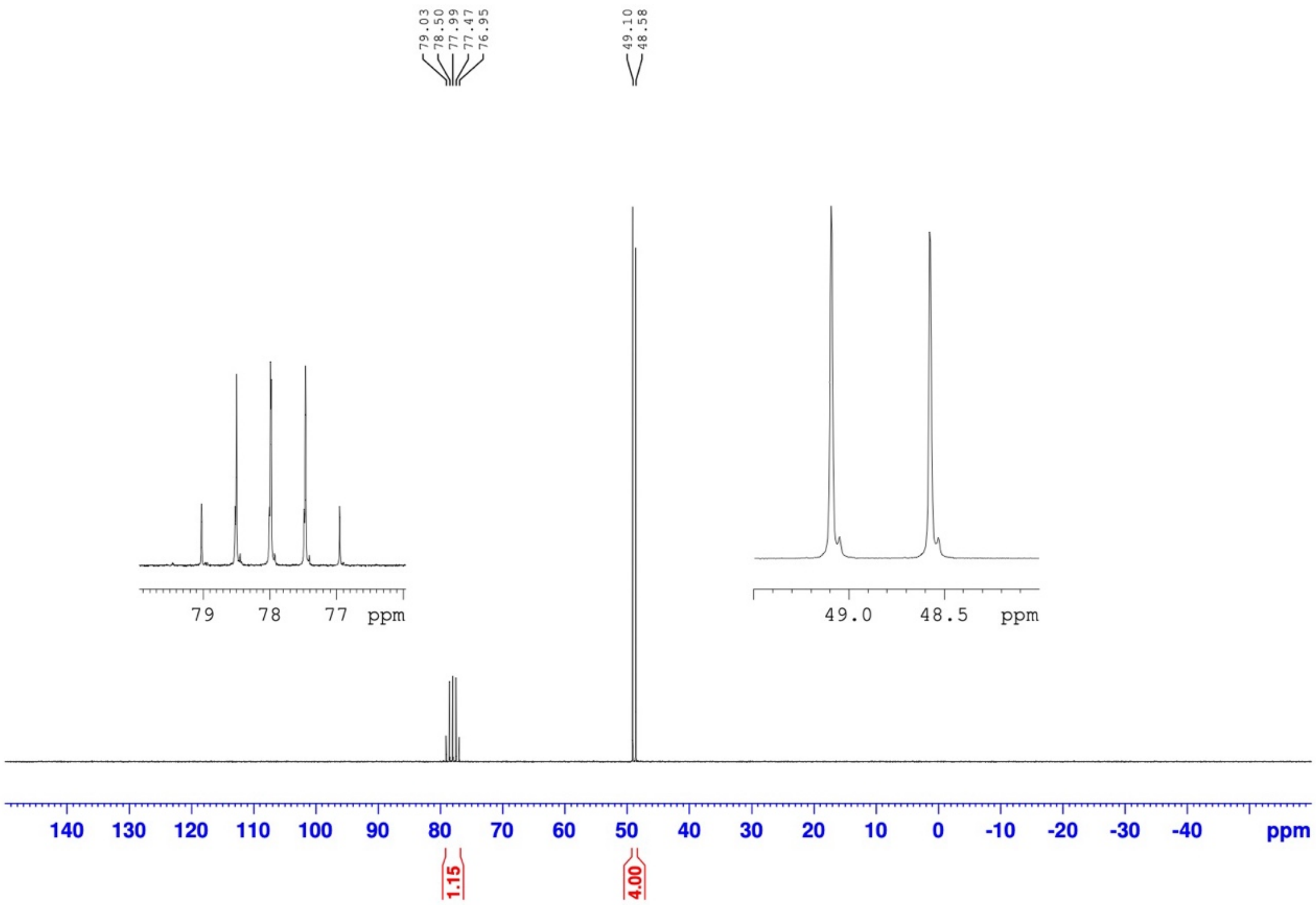

Figure S205. ^{19}F NMR spectrum of (3-(pentafluoroλ^6-sulfanyl)bicyclo[1.1.1]pentane-1-carbohydrazide (**18**) in $CDCl_3$.

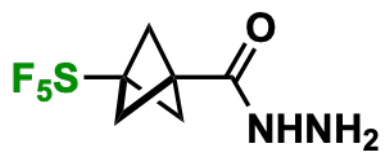

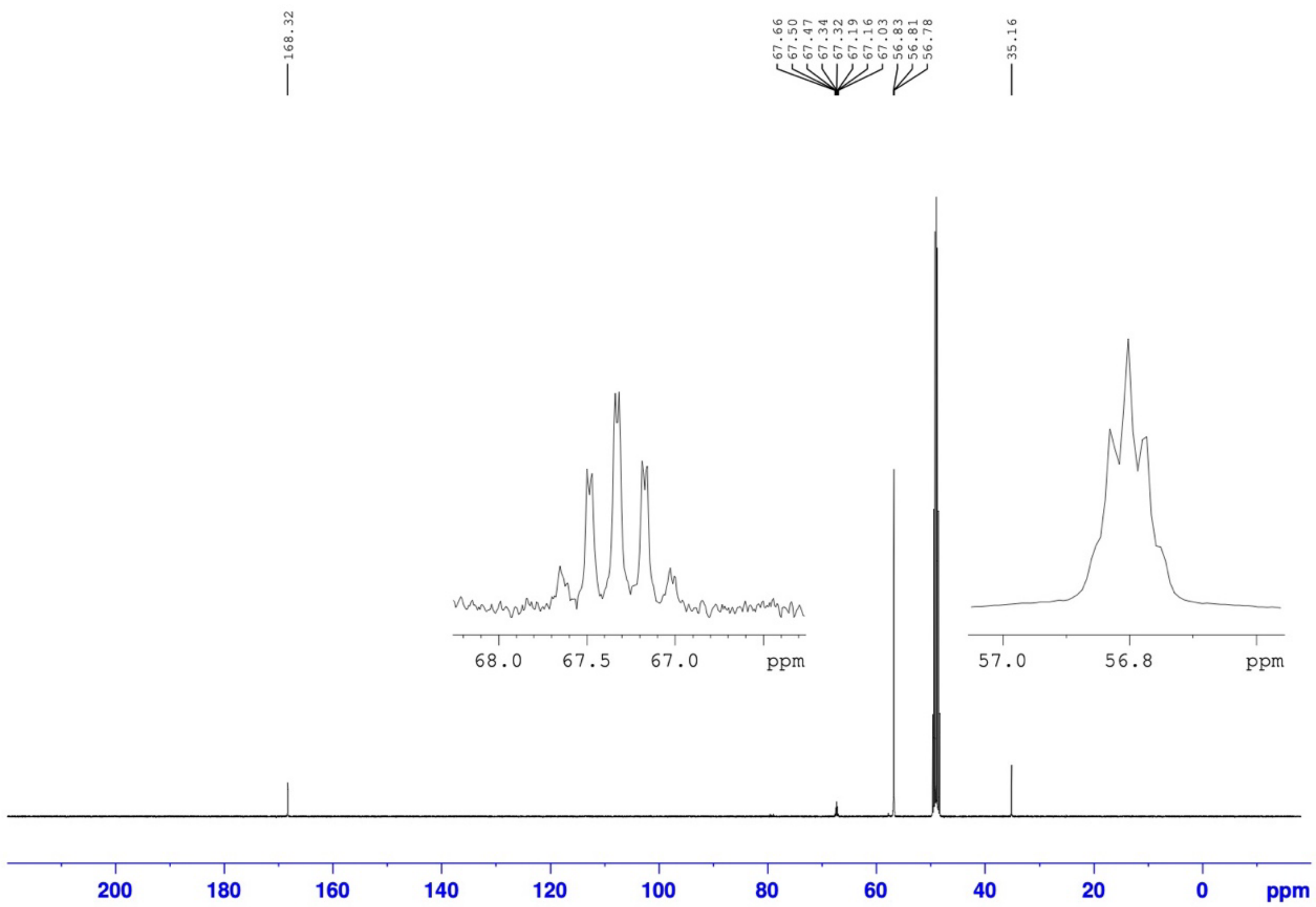

Figure S206. $^{13}C\{^{1}H\}$ NMR spectrum of (3-(pentafluoroλ^{6}-sulfanyl)bicyclo[1.1.1]pentane-1-carbohydrazide (**18**) in MeOD-*d*$_4$.

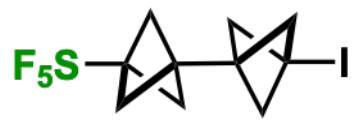

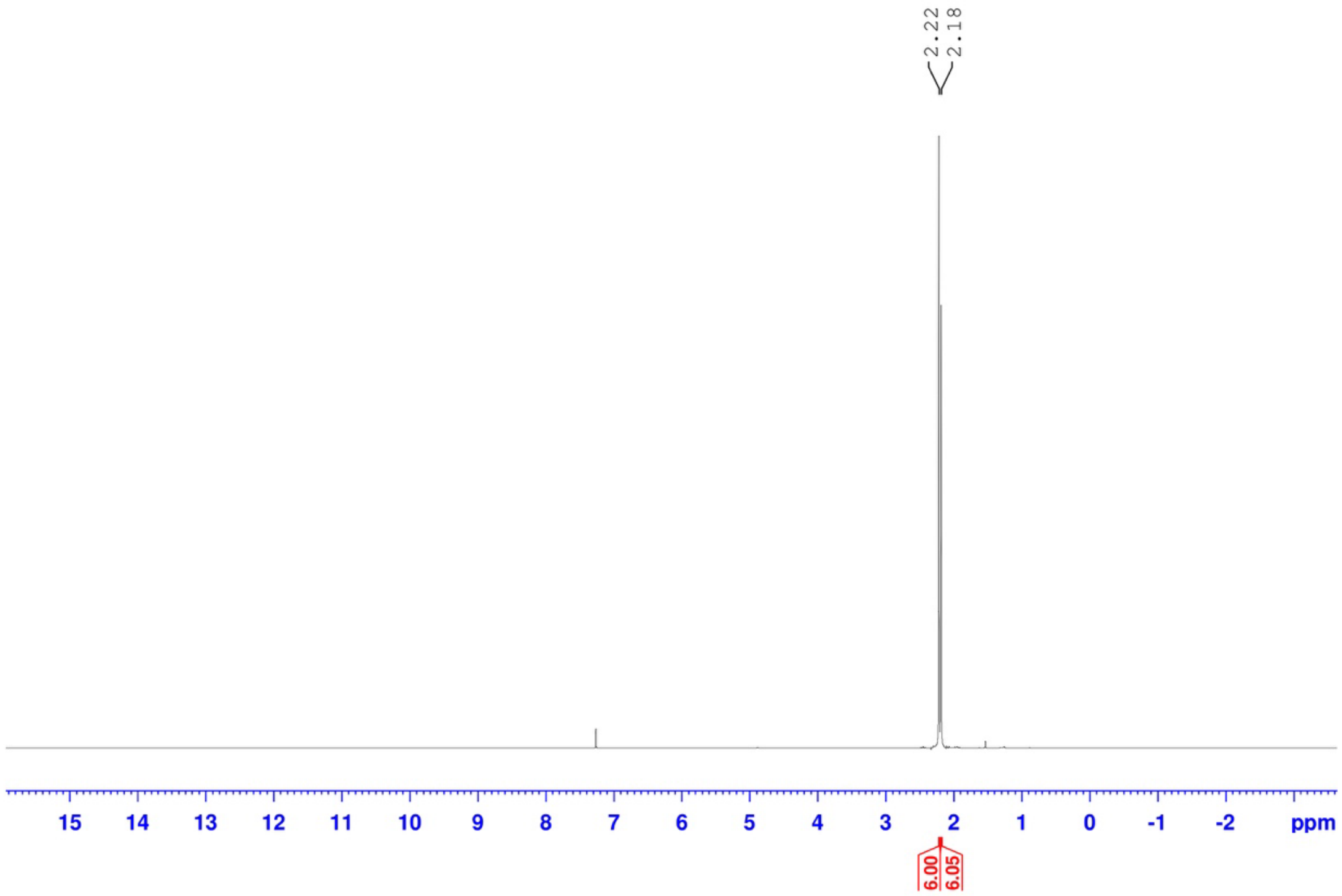

Figure S207. ^{1}H NMR spectrum of pentafluoro(3'-iodo-[1,1'-bi(bicyclo[1.1.1]pentan)]-3-yl)-λ^{6}-sulfane (**5**) in $CDCl_3$.

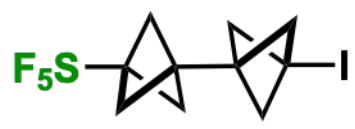

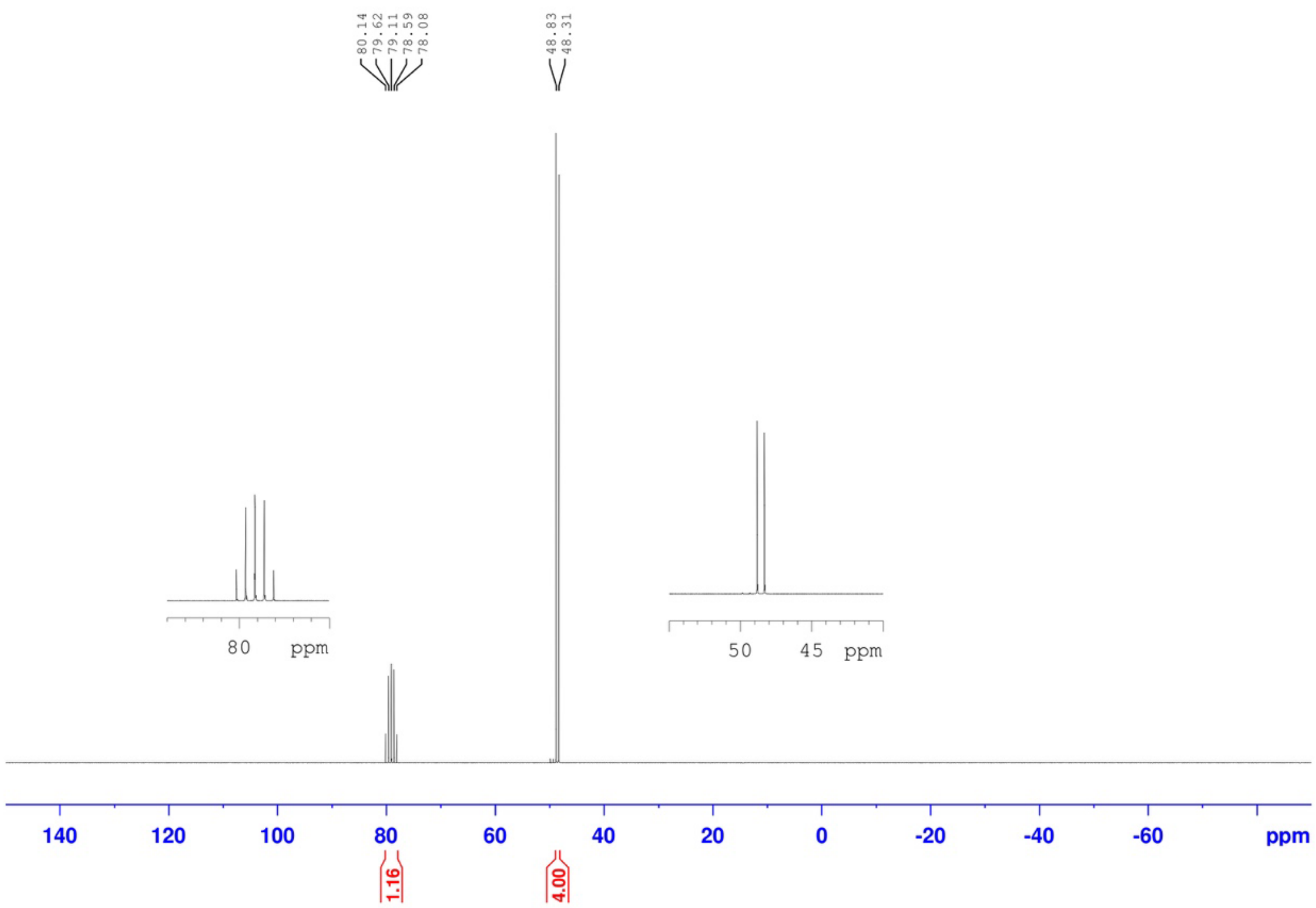

Figure S208. ^{19}F NMR spectrum of pentafluoro(3'-iodo-[1,1'-bi(bicyclo[1.1.1]pentan)]-3-yl)-λ^6-sulfane (**5**) in $CDCl_3$.

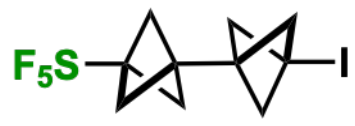

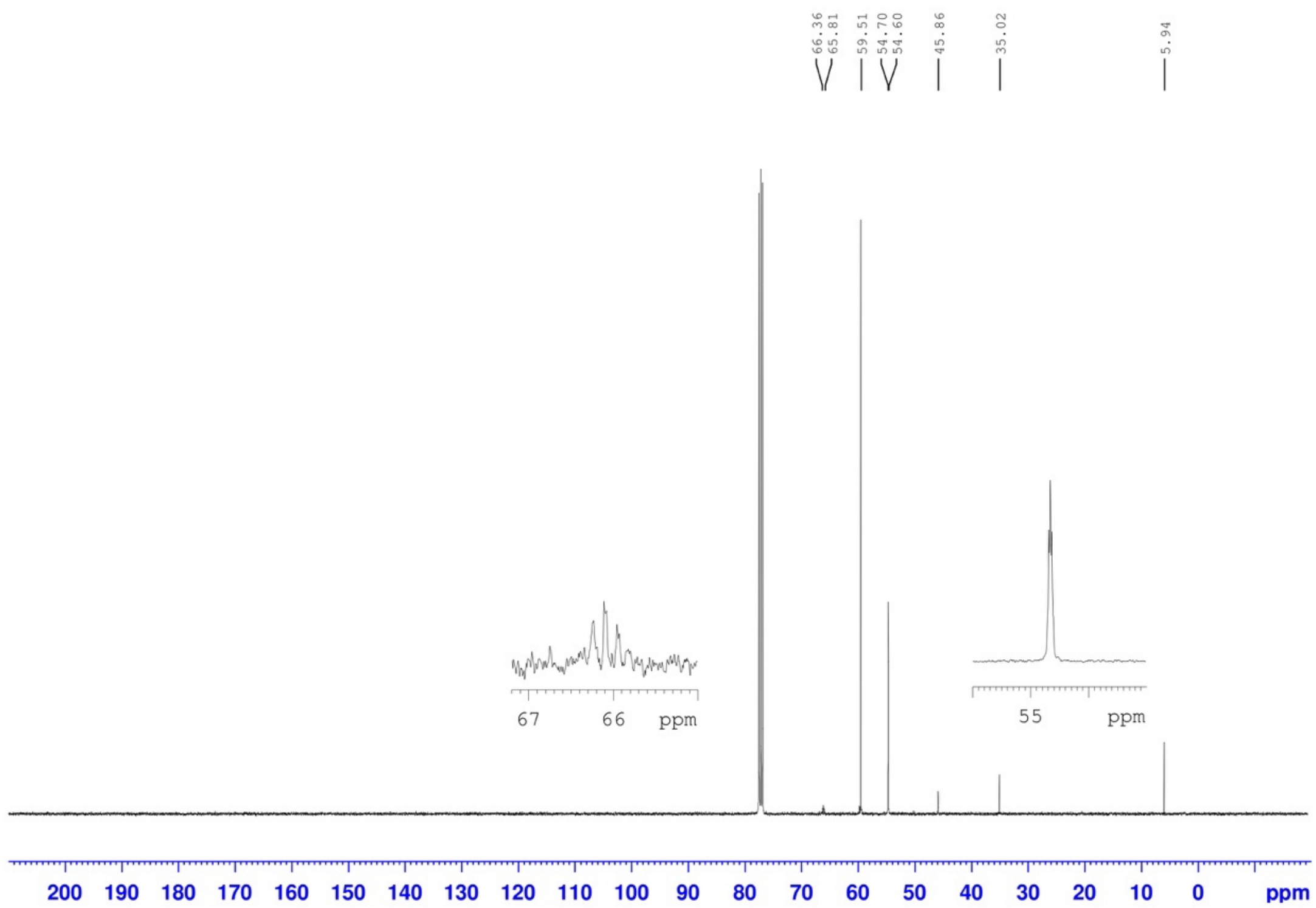

Figure S209. $^{13}C\{^{1}H\}$ NMR spectrum of pentafluoro(3'-iodo-[1,1'-bi(bicyclo[1.1.1]pentan)]-3-yl)-λ^6-sulfane (**5**) in $CDCl_3$.

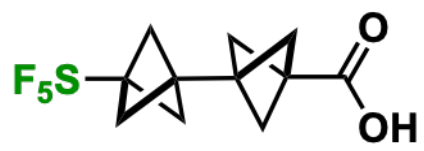

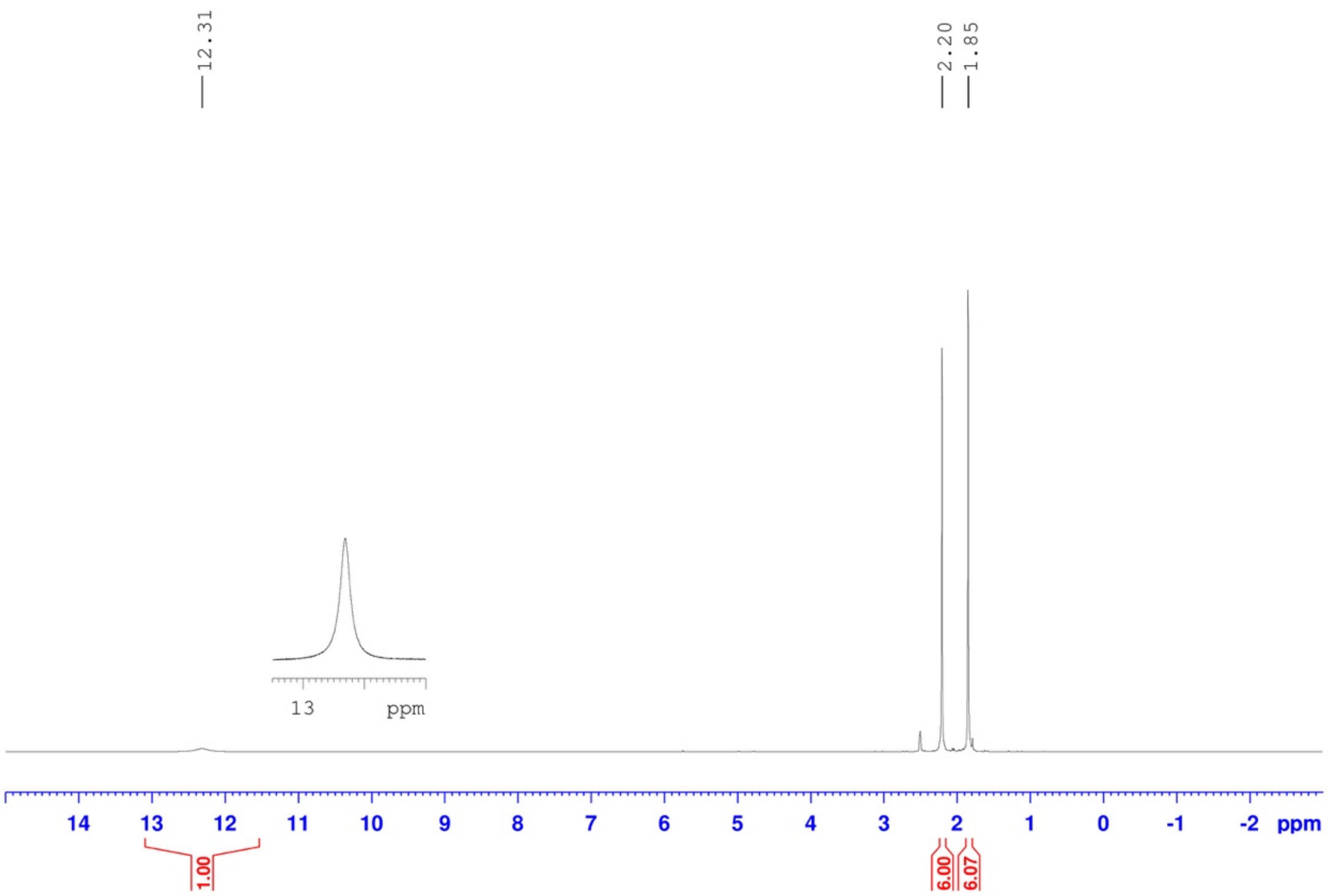

Figure S210. ^{1}H NMR spectrum of 3'-(pentafluoro-λ^6-sulfanyl)-[1,1'-bi(bicyclo[1.1.1]pentane)]-3-carboxylic acid (**6**) in DMSO-d_6.

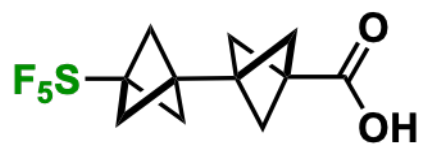

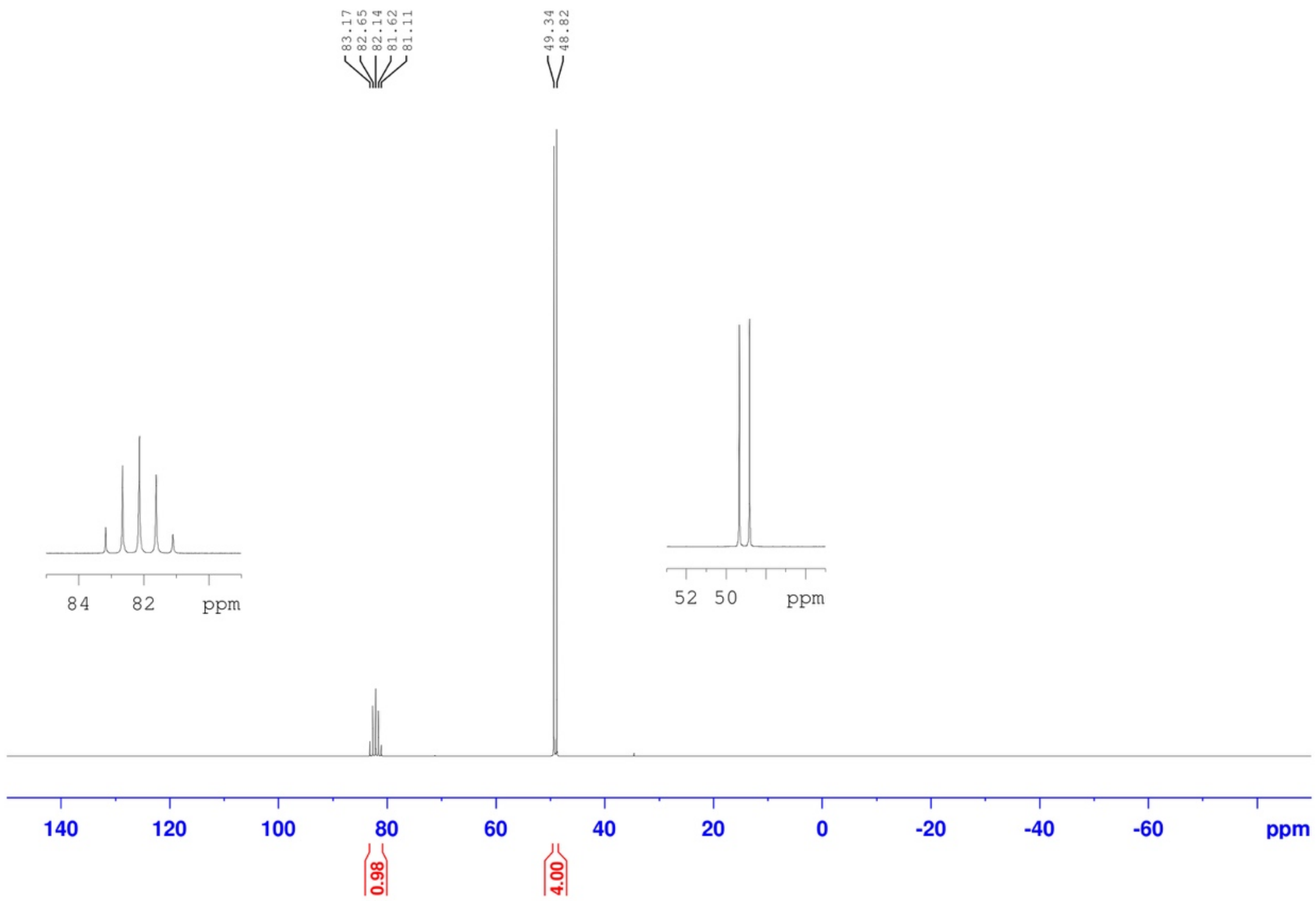

Figure S211. ^{19}F NMR spectrum of 3'-(pentafluoro-λ^6-sulfanyl)-[1,1'-bi(bicyclo[1.1.1]pentane)]-3-carboxylic acid (**6**) in DMSO-d_6.

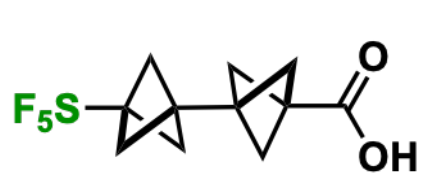

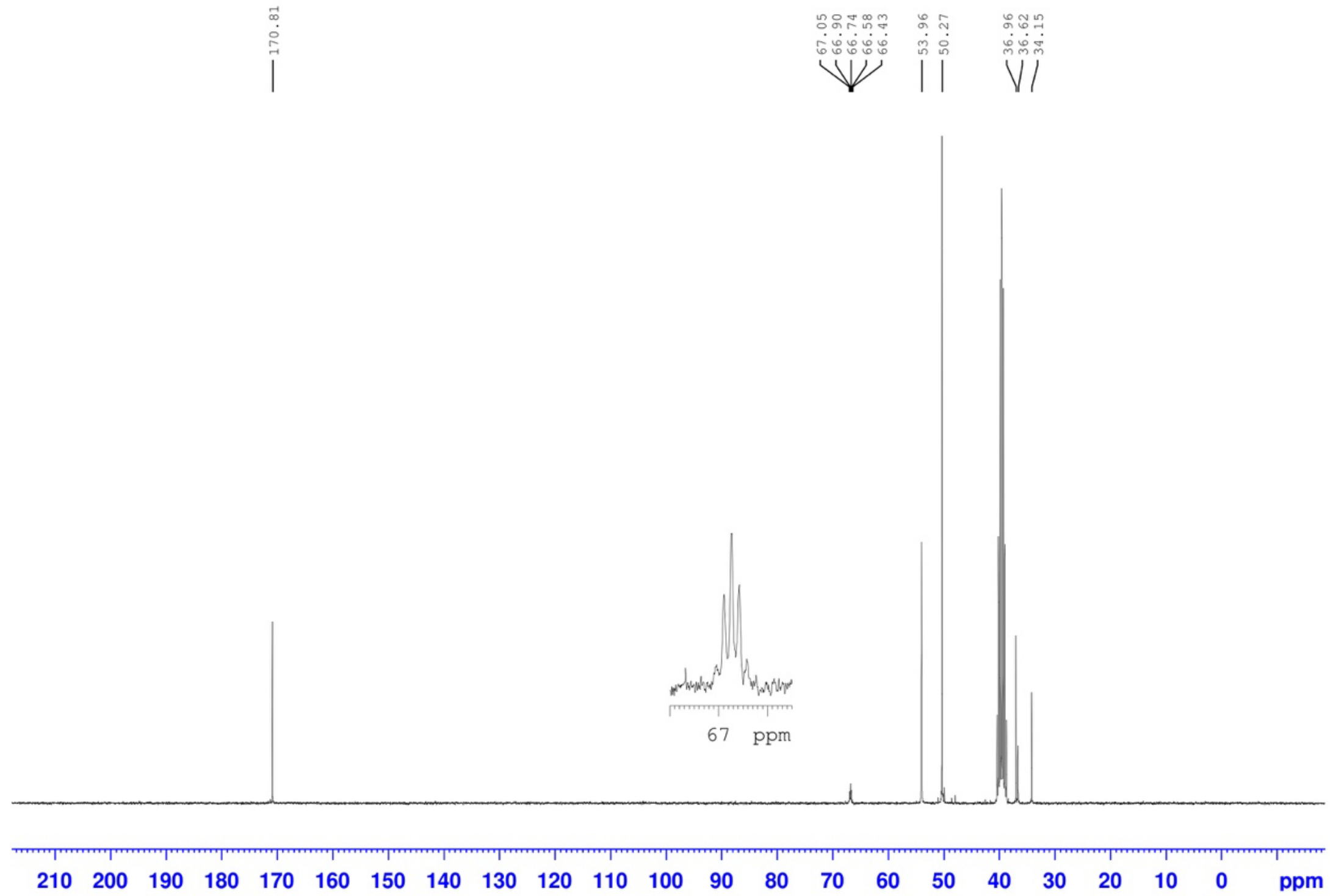

Figure S212. $^{13}C\{^1H\}$ NMR spectrum of 3'-(pentafluoro-λ^6-sulfanyl)-[1,1'-bi(bicyclo[1.1.1]pentane)]-3-carboxylic acid (**6**) in DMSO-d_6.

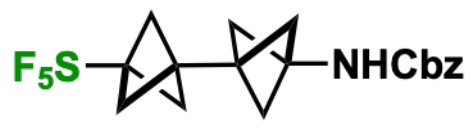

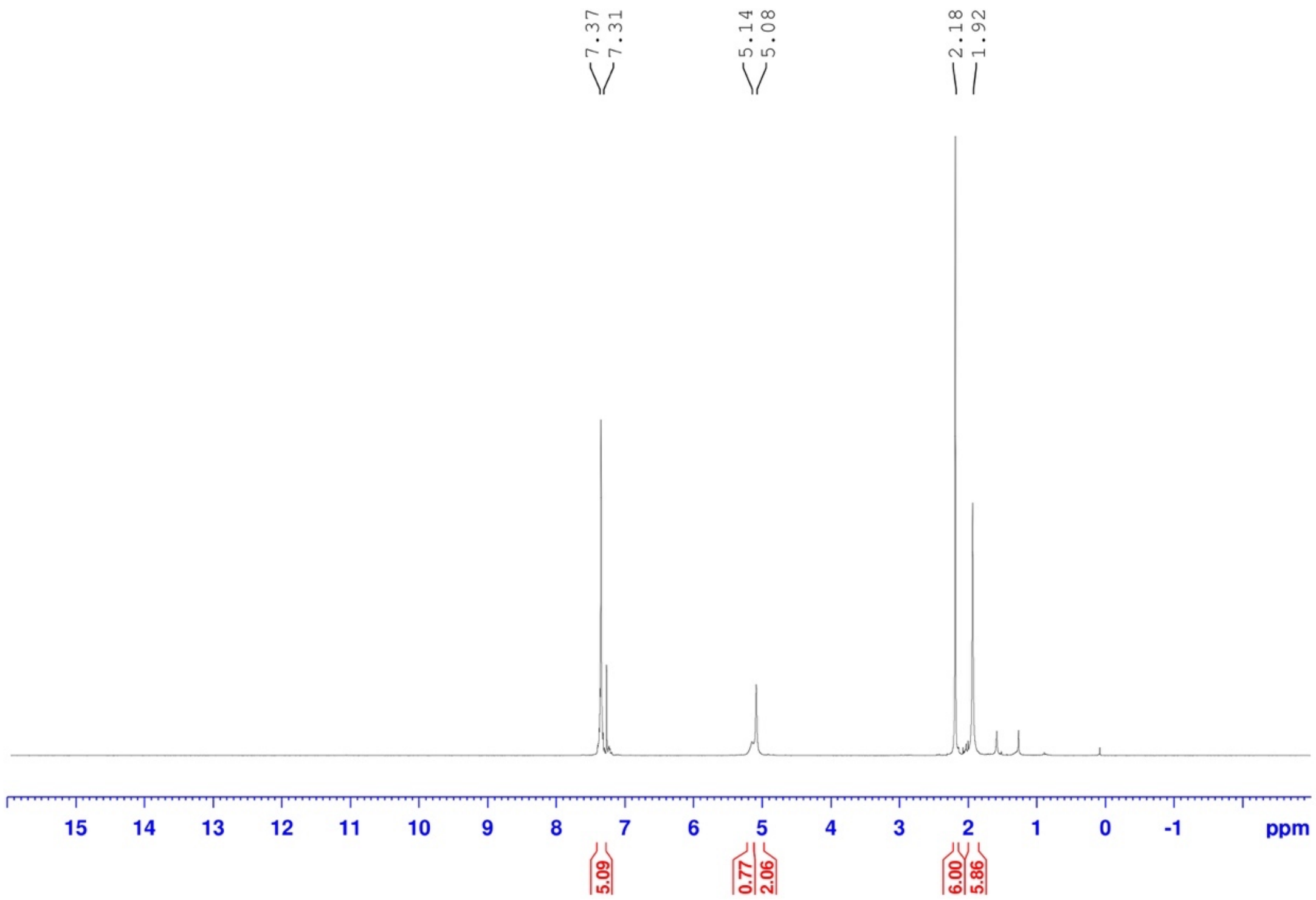

Figure S213. ^{1}H NMR spectrum of benzyl (3'-(pentafluoro-λ^6-sulfanyl)-[1,1'-bi(bicyclo[1.1.1]pentan)]-3-yl)carbamate (**S-23**) in $CDCl_3$.

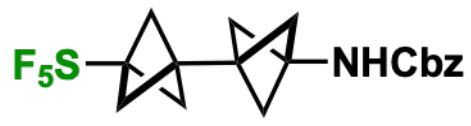

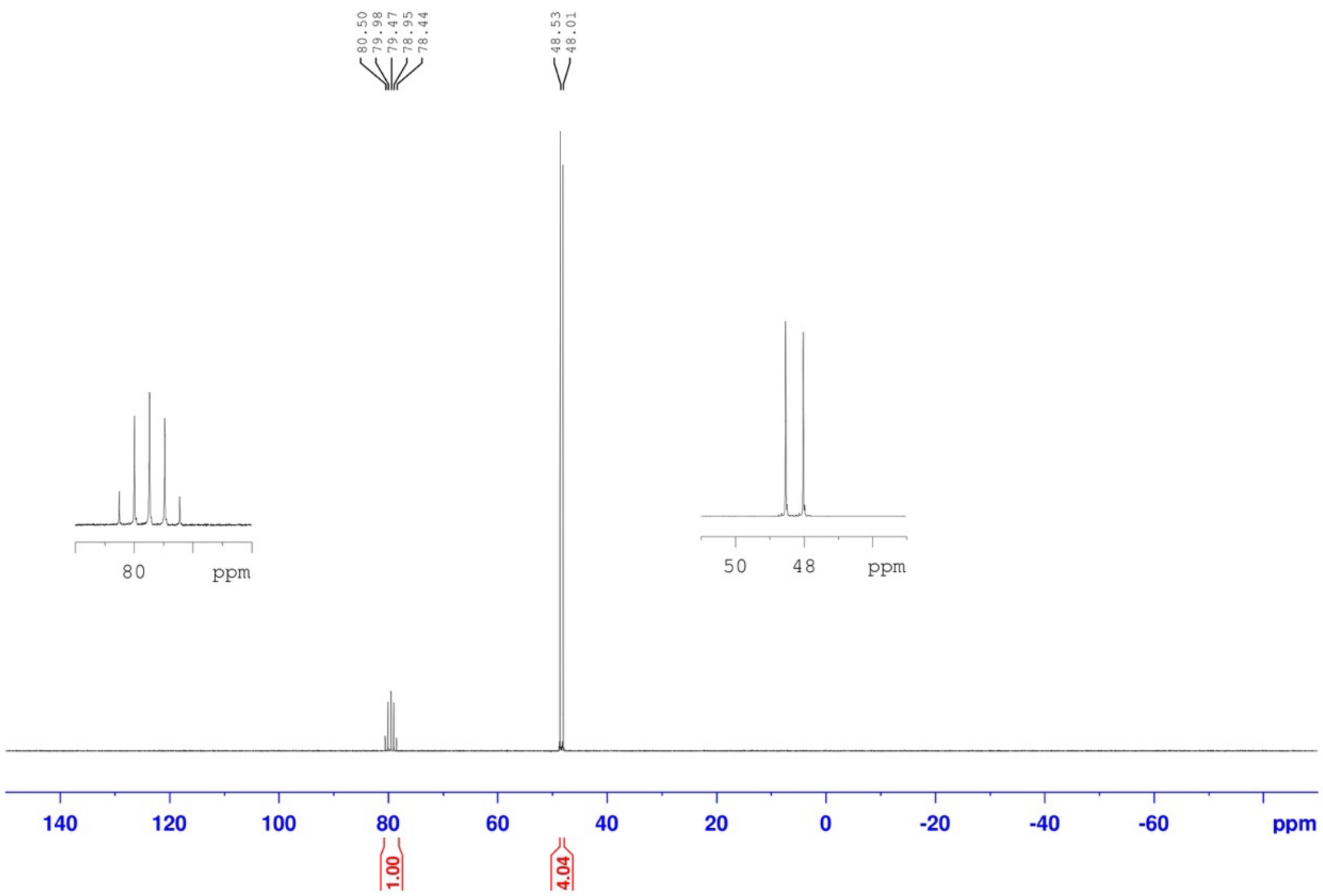

Figure S214. ^{19}F NMR spectrum of benzyl (3'-(pentafluoro-λ^6-sulfanyl)-[1,1'-bi(bicyclo[1.1.1]pentan)]-3-yl)carbamate (**S-23**) in $CDCl_3$.

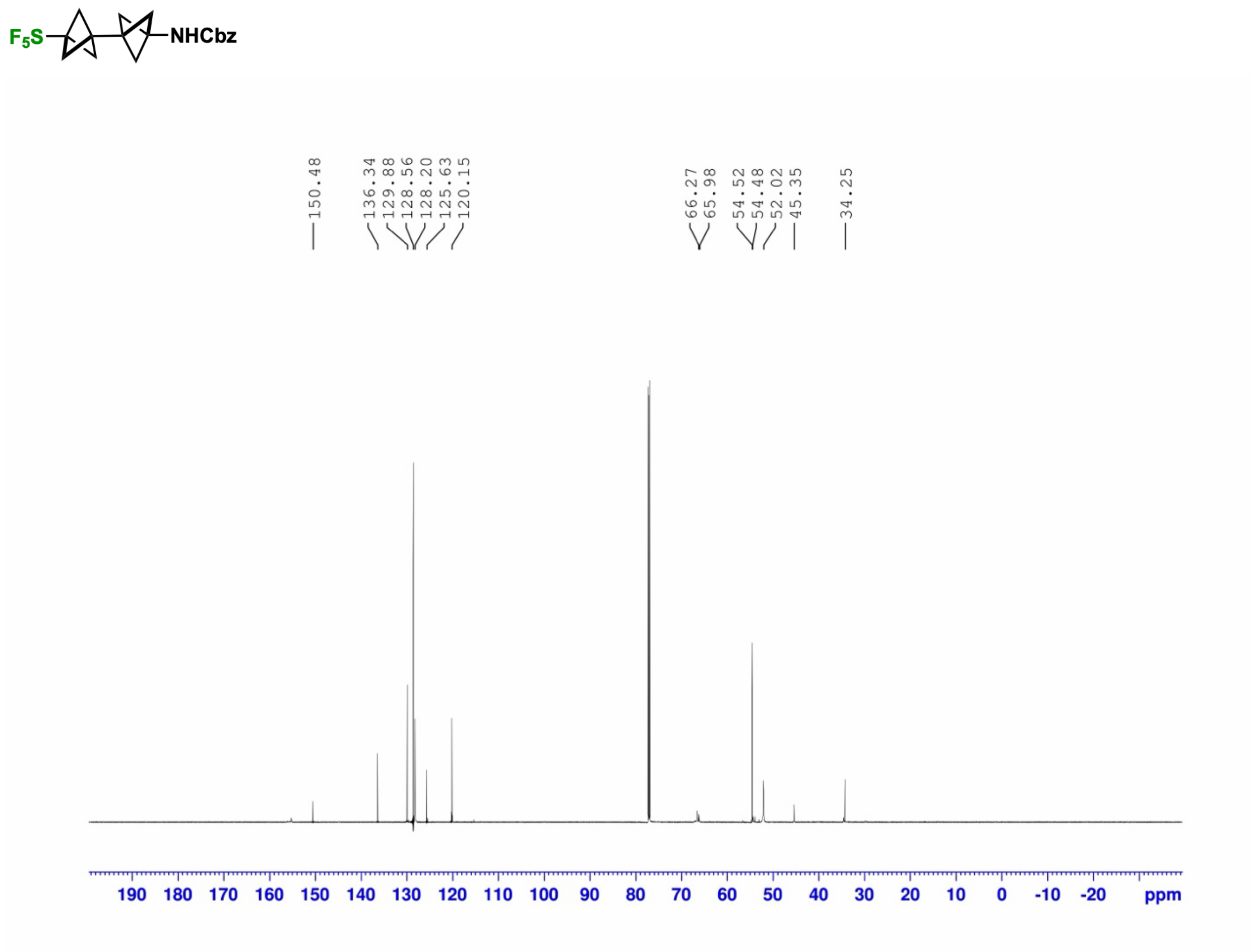

Figure S215. $^{13}C\{^{1}H\}$ NMR spectrum of benzyl (3'-(pentafluoro-λ^6-sulfanyl)-[1,1'-bi(bicyclo[1.1.1]pentan)]-3-yl)carbamate (**S-23**) in $CDCl_3$.

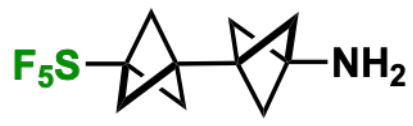

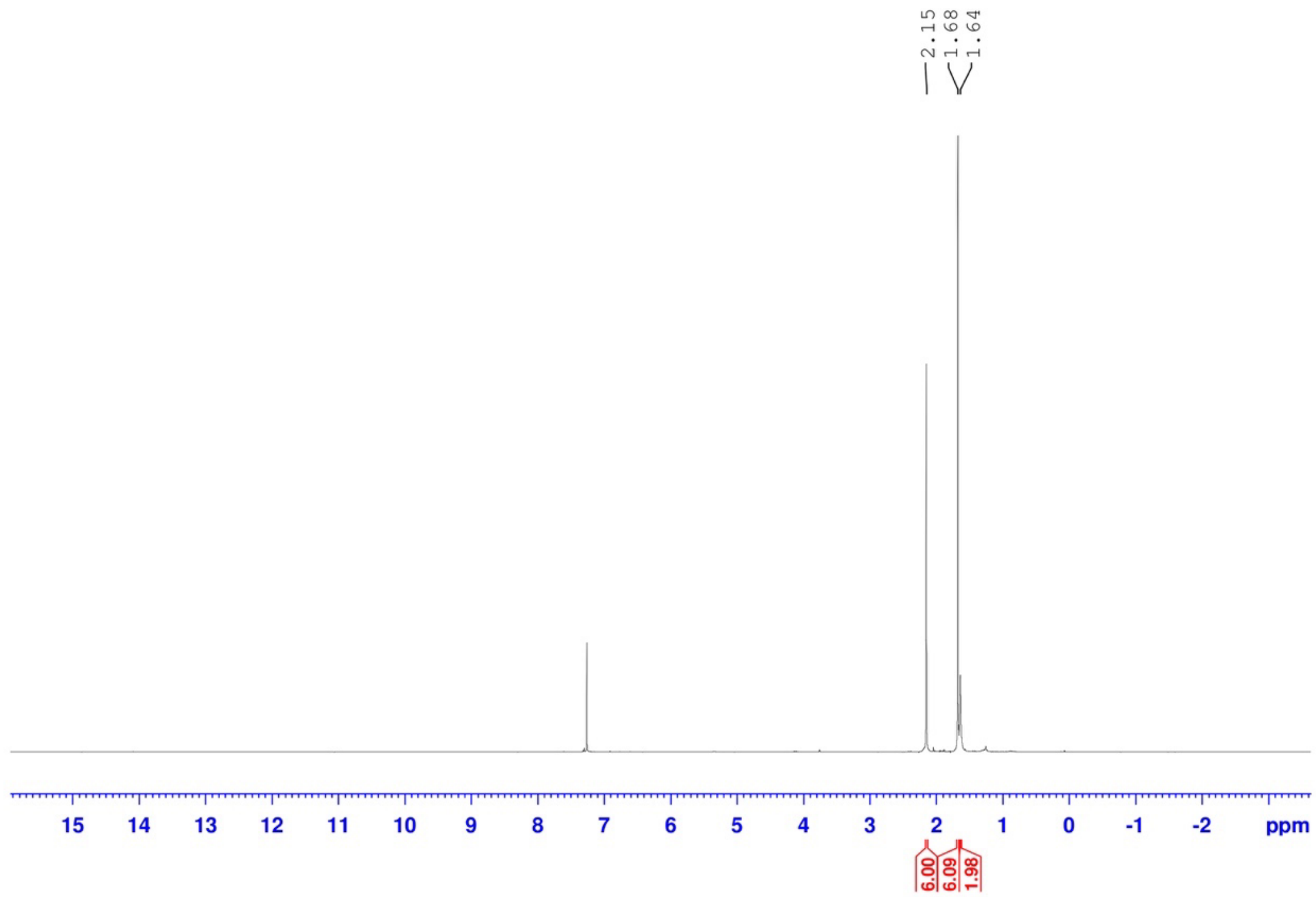

Figure S216. ^{1}H NMR spectrum of 3'-(pentafluoro-λ^6-sulfanyl)-[1,1'-bi(bicyclo[1.1.1]pentan)]-3-amine (**49**) in $CDCl_3$.

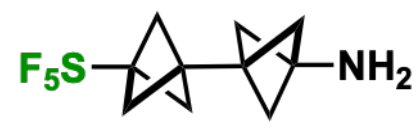

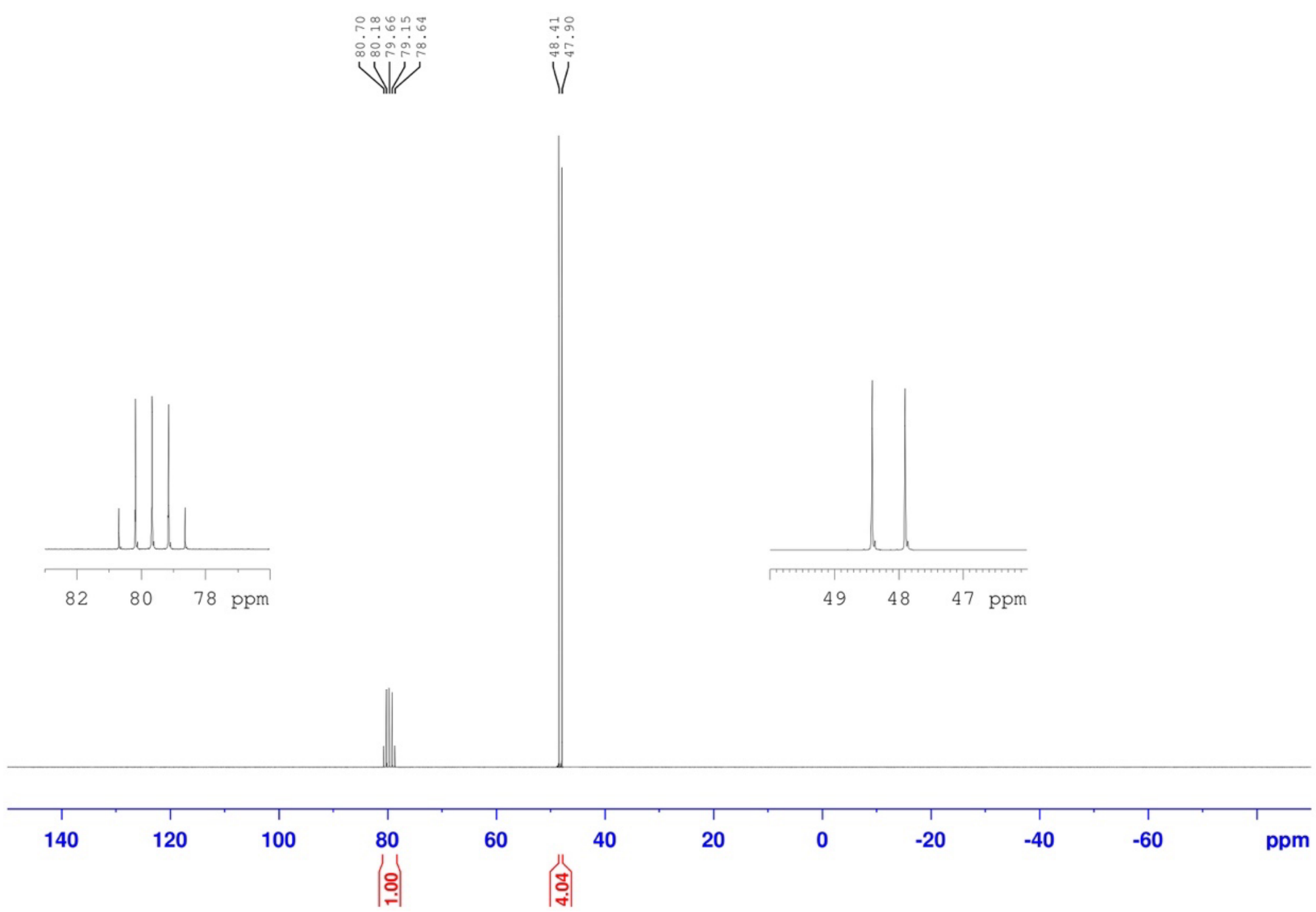

Figure S217. ^{19}F NMR spectrum of 3'-(pentafluoro-λ^6-sulfanyl)-[1,1'-bi(bicyclo[1.1.1]pentan)]-3-amine (**49**) in $CDCl_3$.

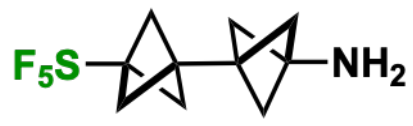

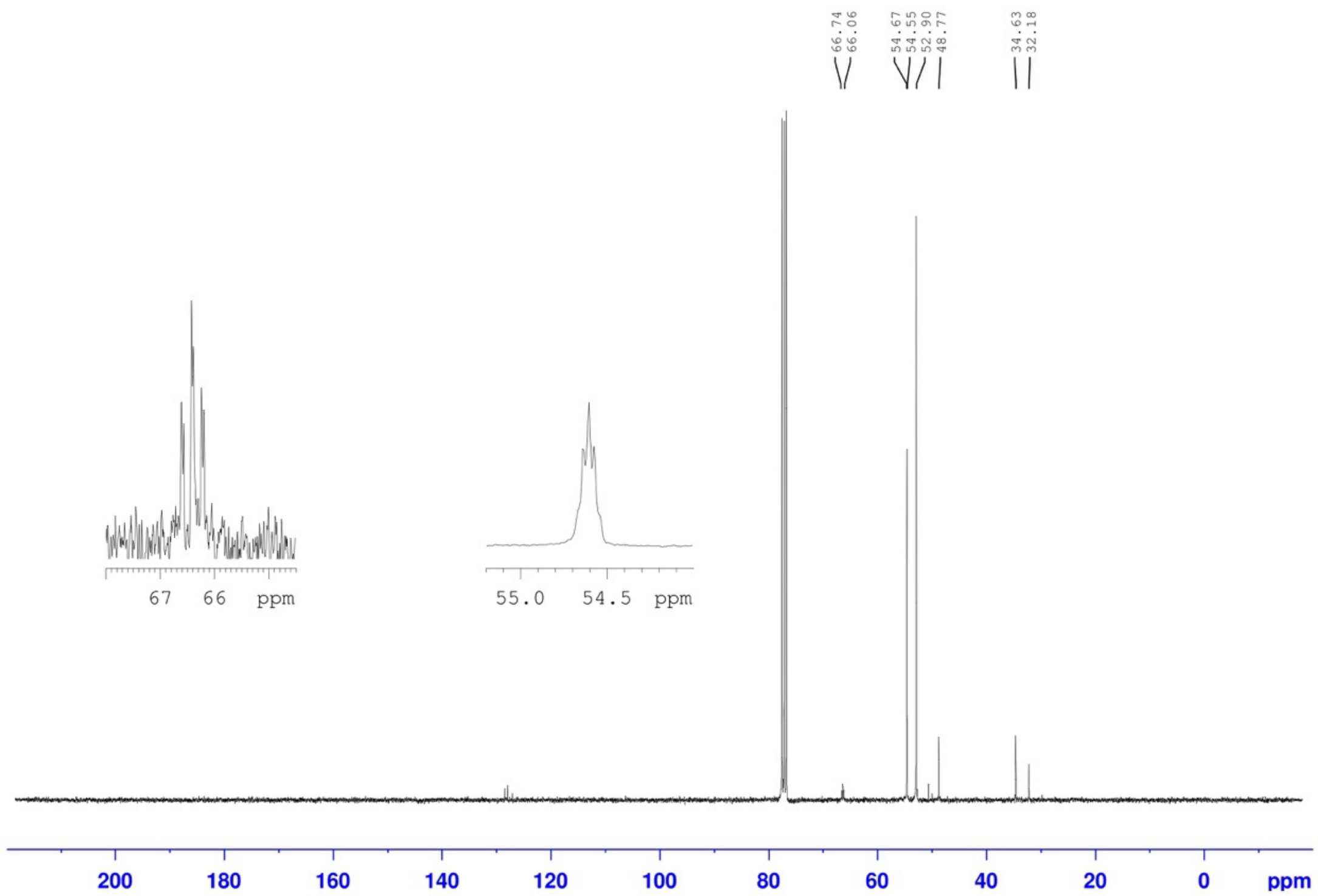

Figure S218. $^{13}C\{^1H\}$ NMR spectrum of 3'-(pentafluoro-λ^6-sulfanyl)-[1,1'-bi(bicyclo[1.1.1]pentan)]-3-amine (**49**) in $CDCl_3$.

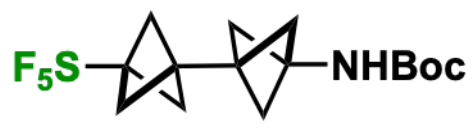

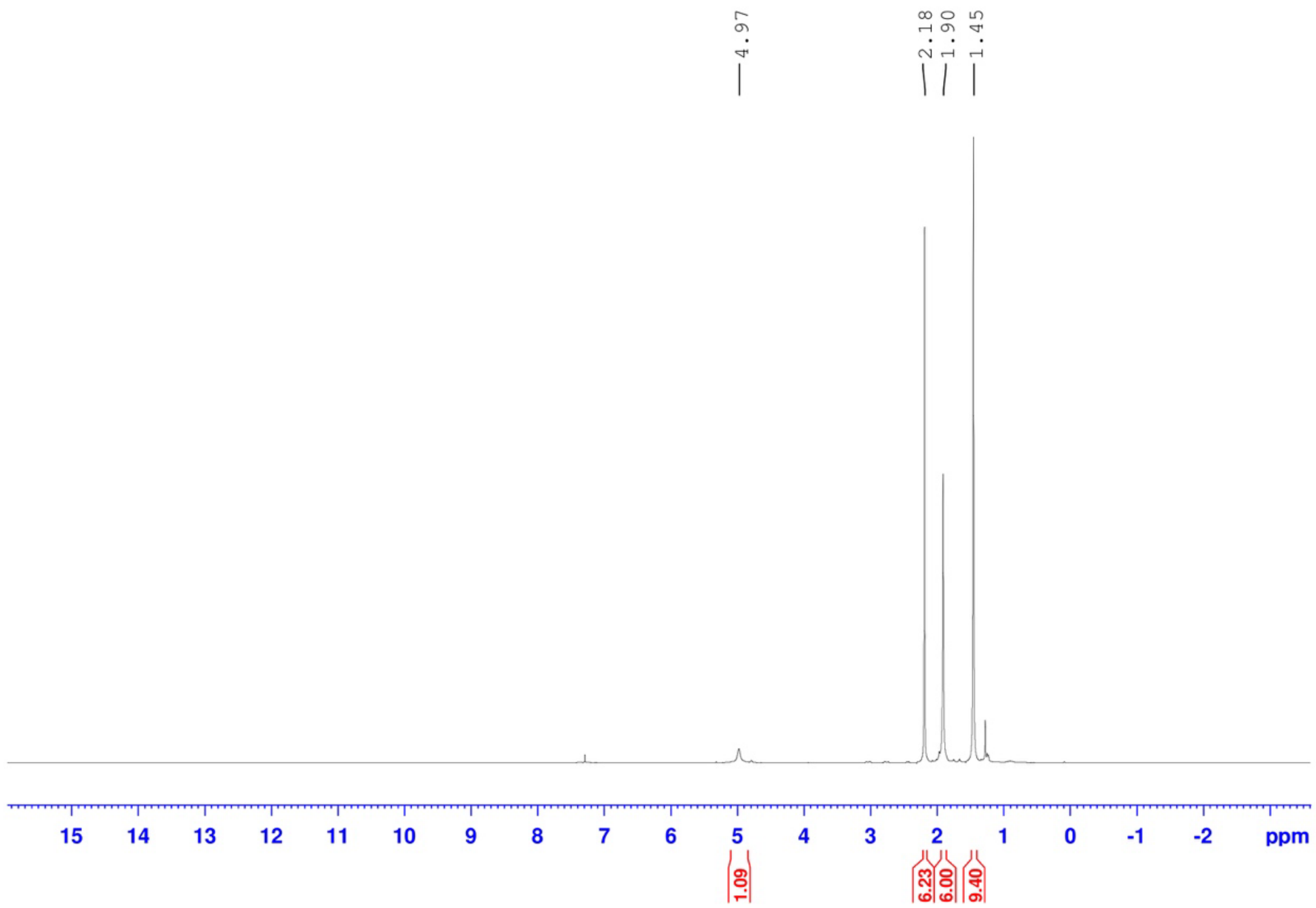

Figure S219. ^{1}H NMR spectrum of *tert*-butyl (3'-(pentafluoro-λ^6-sulfanyl)-[1,1'-bi(bicyclo[1.1.1]pentan)]-3-yl)carbamate (**S-24**) in $CDCl_3$.

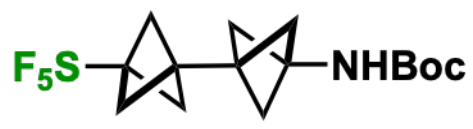

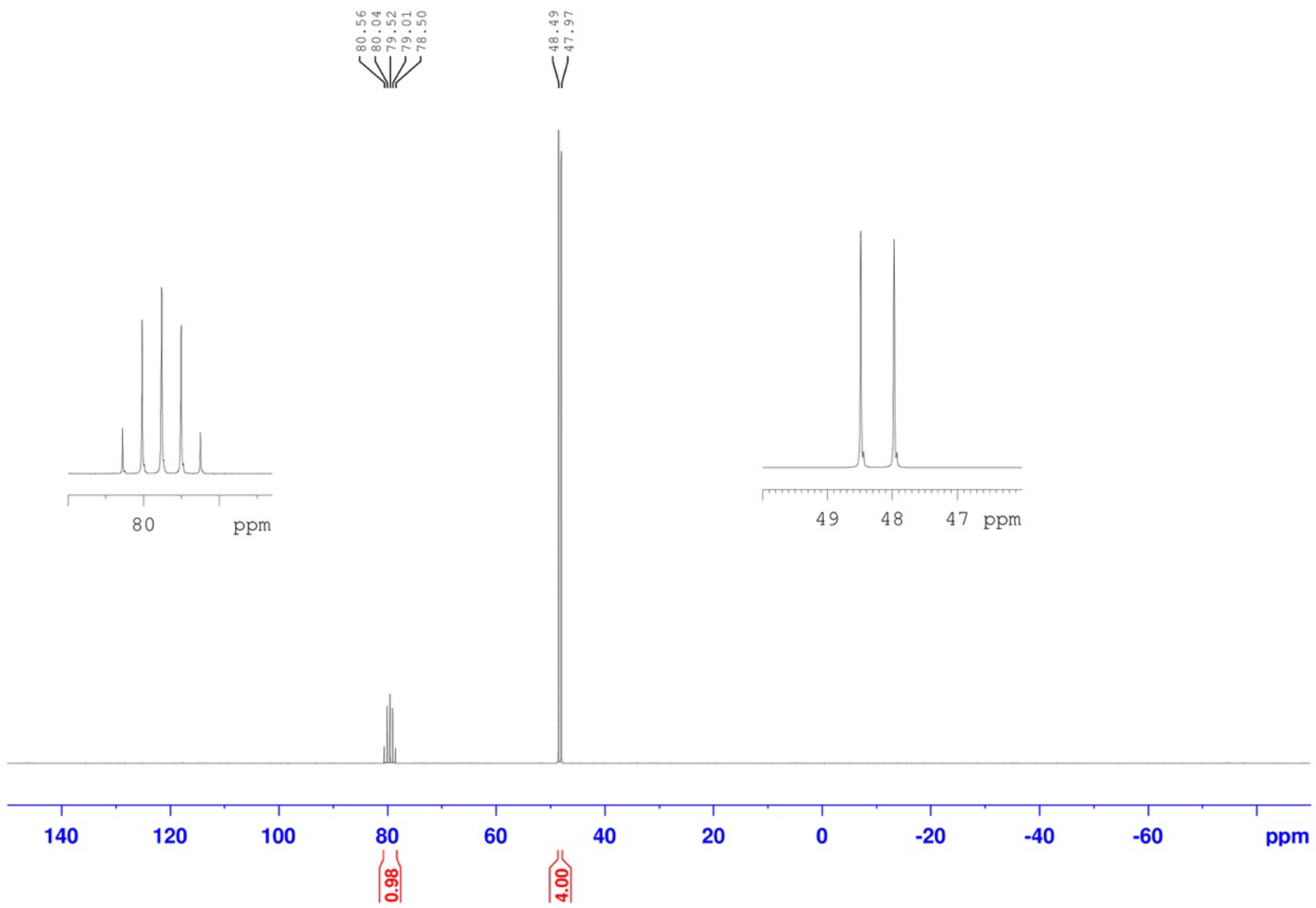

Figure S220. ^{19}F NMR spectrum of *tert*-butyl (3'-(pentafluoro-λ^6-sulfanyl)-[1,1'-bi(bicyclo[1.1.1]pentan)]-3-yl)carbamate (**S-24**) in $CDCl_3$.

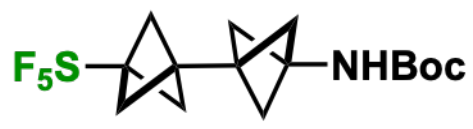

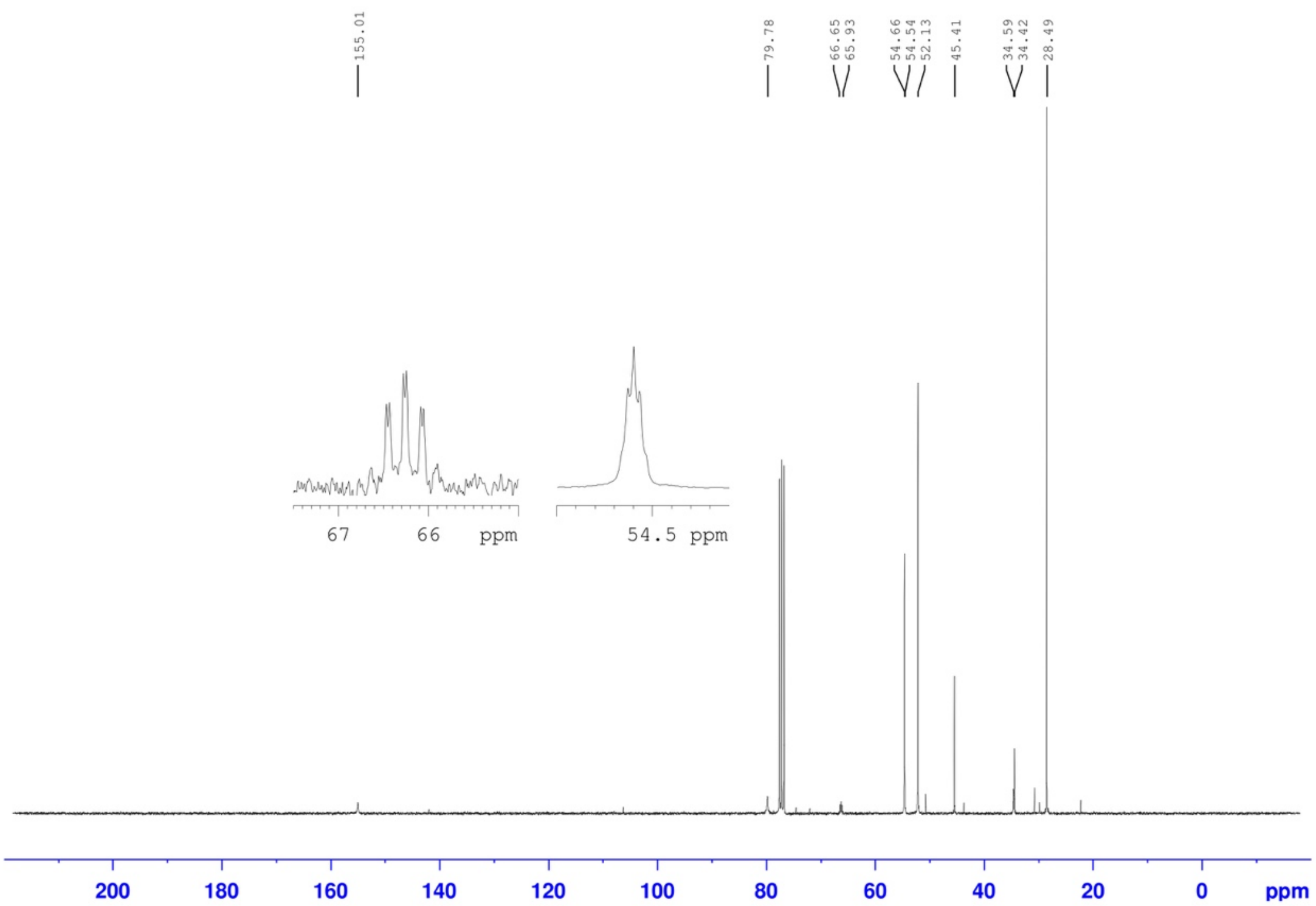

Figure S221. $^{13}C\{^1H\}$ NMR spectrum of *tert*-butyl (3'-(pentafluoro-λ^6-sulfanyl)-[1,1'-bi(bicyclo[1.1.1]pentan)]-3-yl)carbamate (**S-24**) in $CDCl_3$.

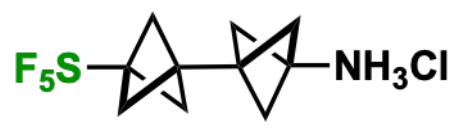

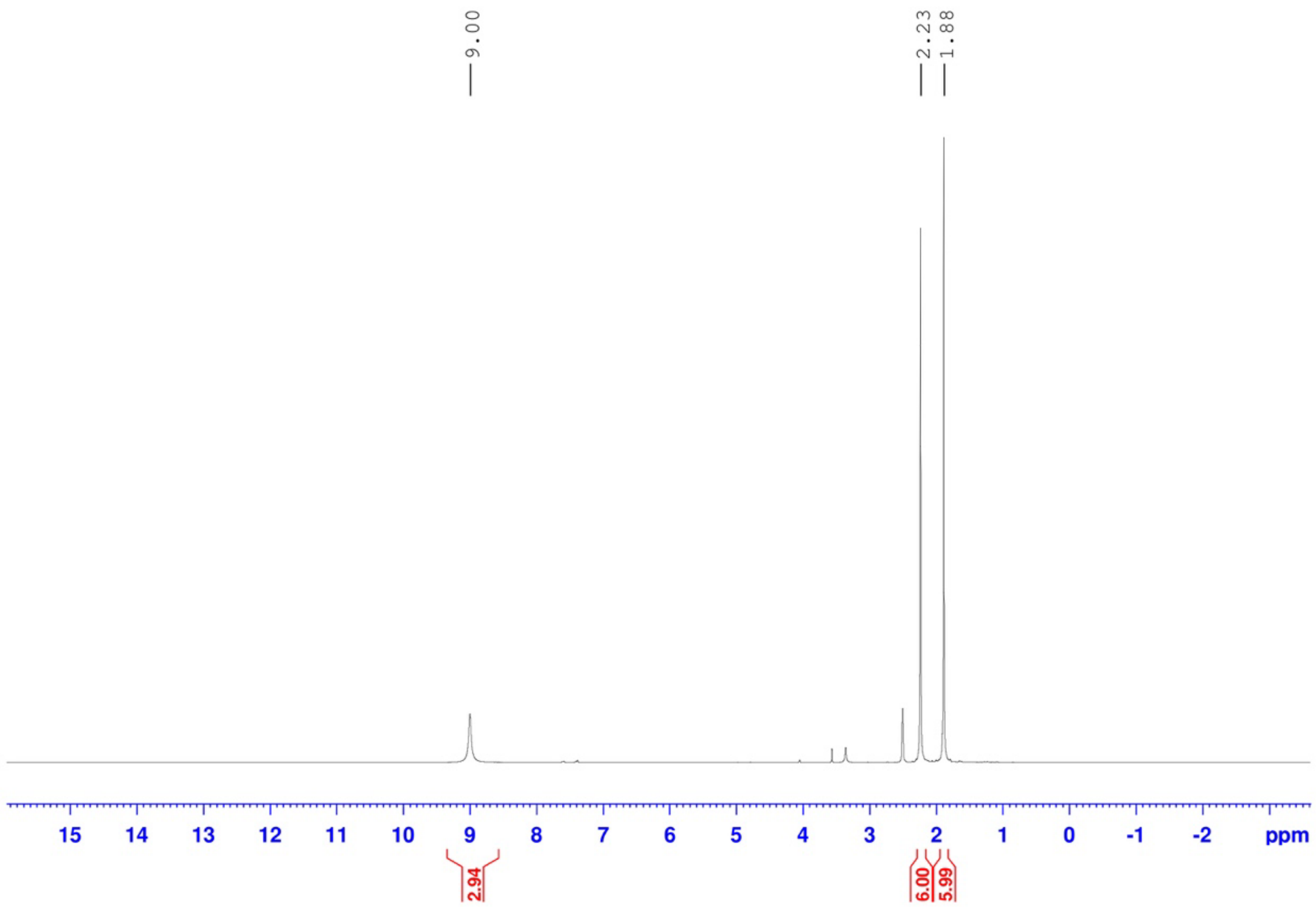

Figure S222. ^{1}H NMR spectrum of chloro(3'-(pentafluoro-λ^6-sulfanyl)-[1,1'-bi(bicyclo[1.1.1]pentan)]-3-yl)-λ^5-azane (**S-25**) in DMSO-*d*$_6$.

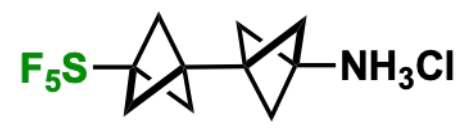

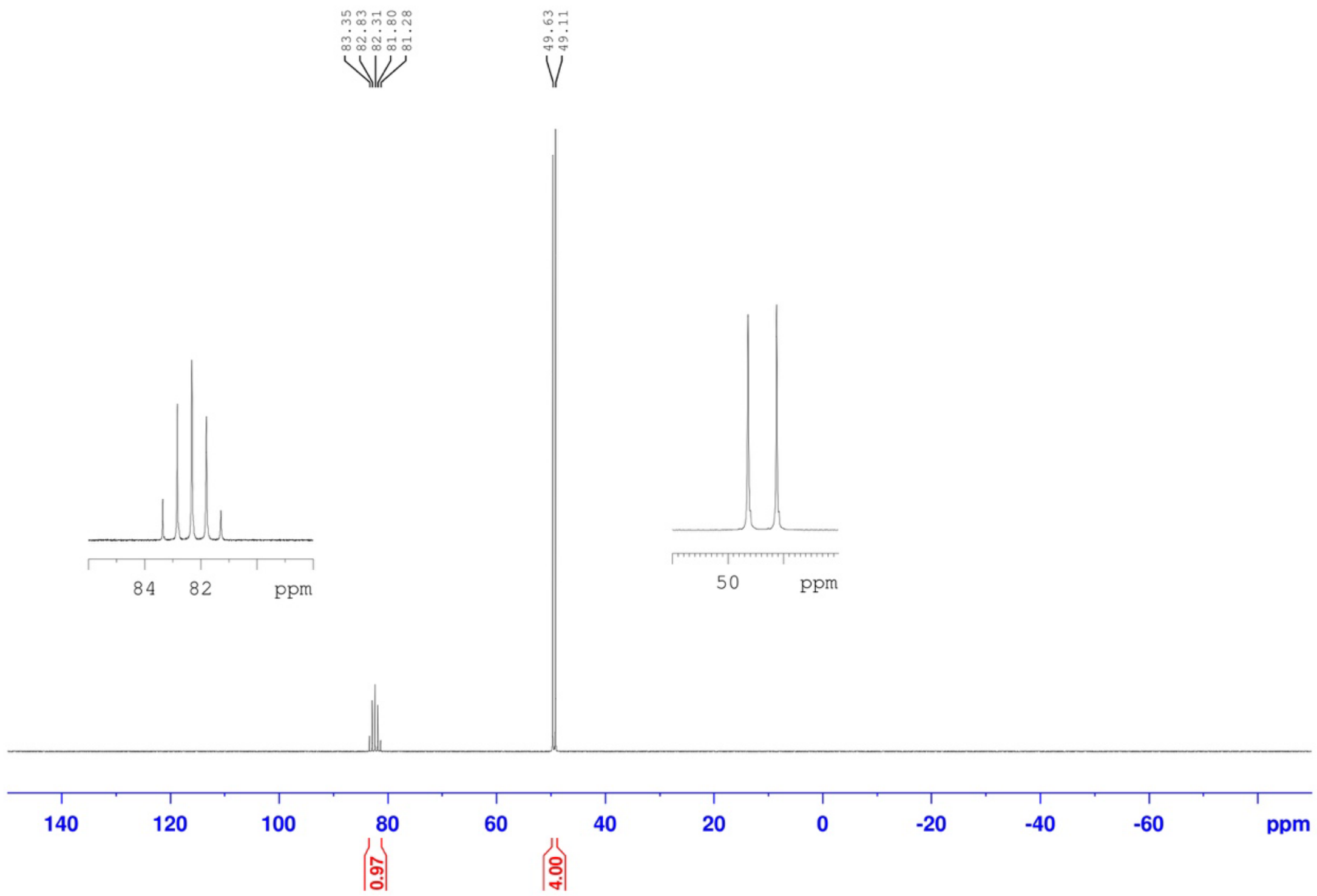

Figure S223. ^{19}F NMR spectrum of chloro(3'-(pentafluoro-λ^6-sulfanyl)-[1,1'-bi(bicyclo[1.1.1]pentan)]-3-yl)-λ^5-azane (**S-25**) in DMSO-d_6.

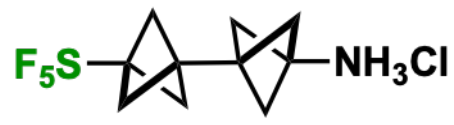

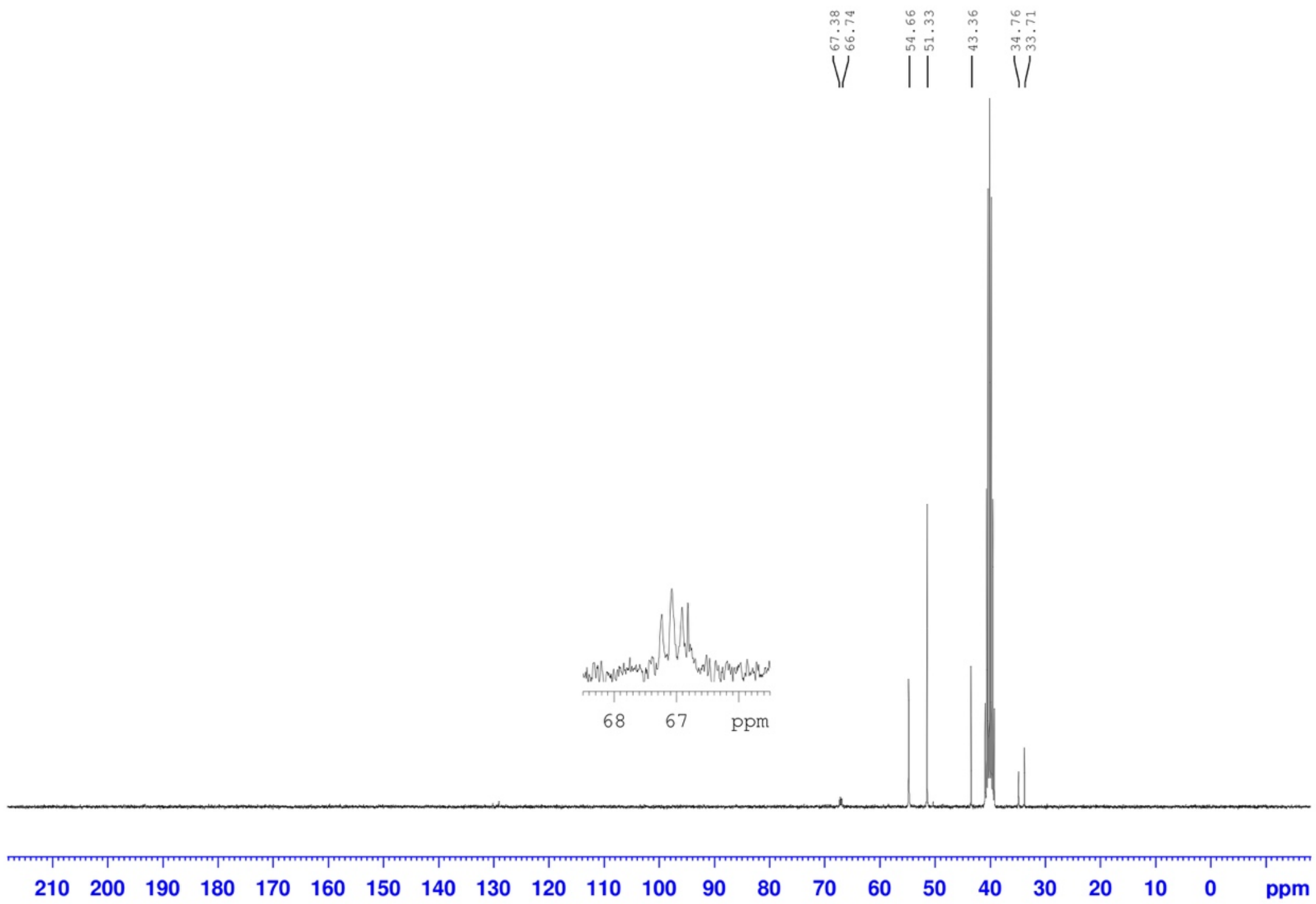

Figure S224. $^{13}C\{^1H\}$ NMR spectrum of chloro(3'-(pentafluoro-λ^6-sulfanyl)-[1,1'-bi(bicyclo[1.1.1]pentan)]-3-yl)-λ^5-azane (**S-25**) in DMSO-*d*6.

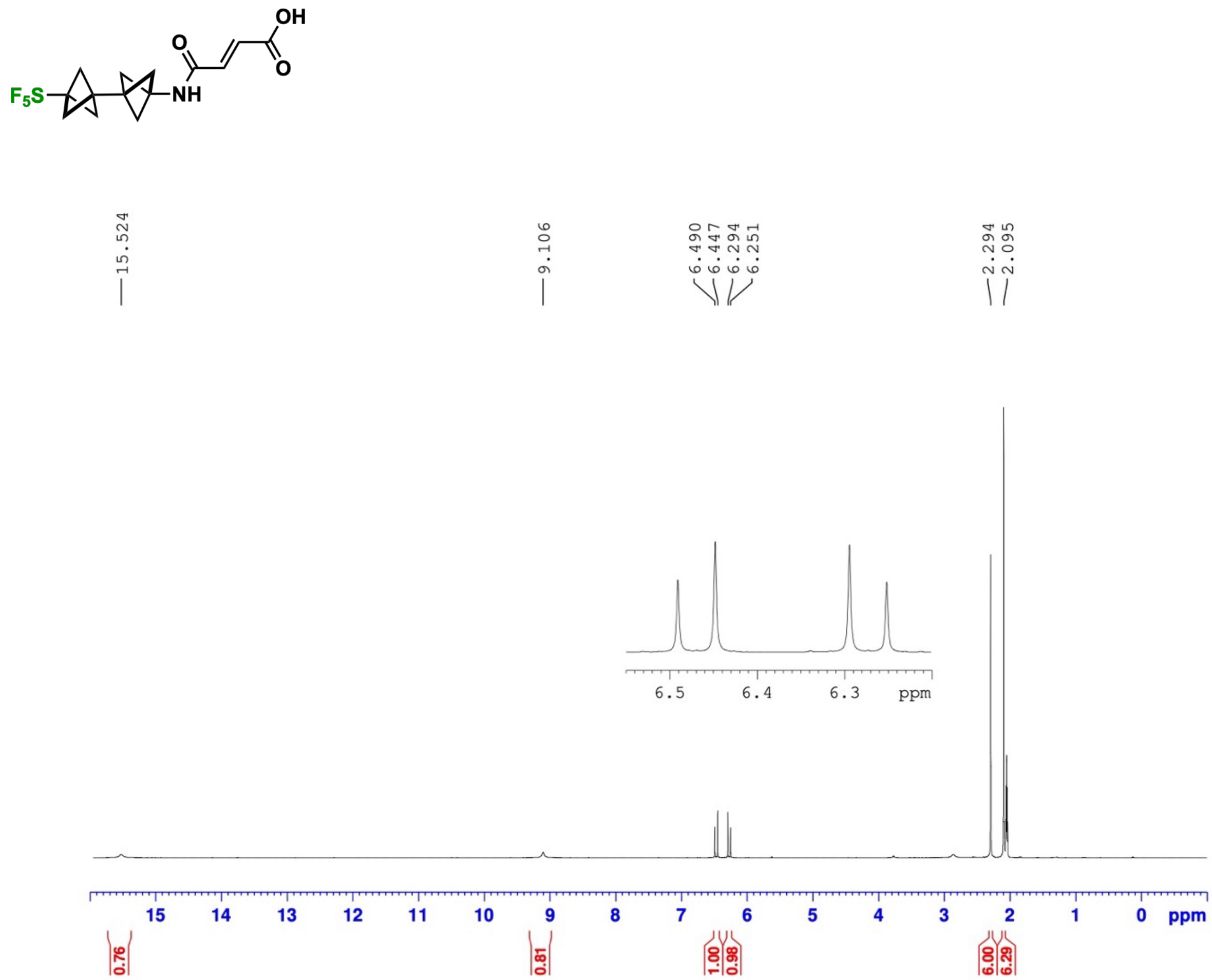

Figure S225. ^{1}H NMR spectrum of (Z) 4-oxo-4-((3'-(pentafluoro-λ^6-sulfanyl)-[1,1'-bi(bicyclo[1.1.1]-pentan)]-3-yl)amino)but-2-enoic acid (**S-26**) in Acetone-d_6.

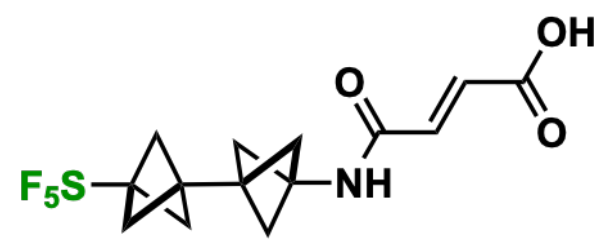

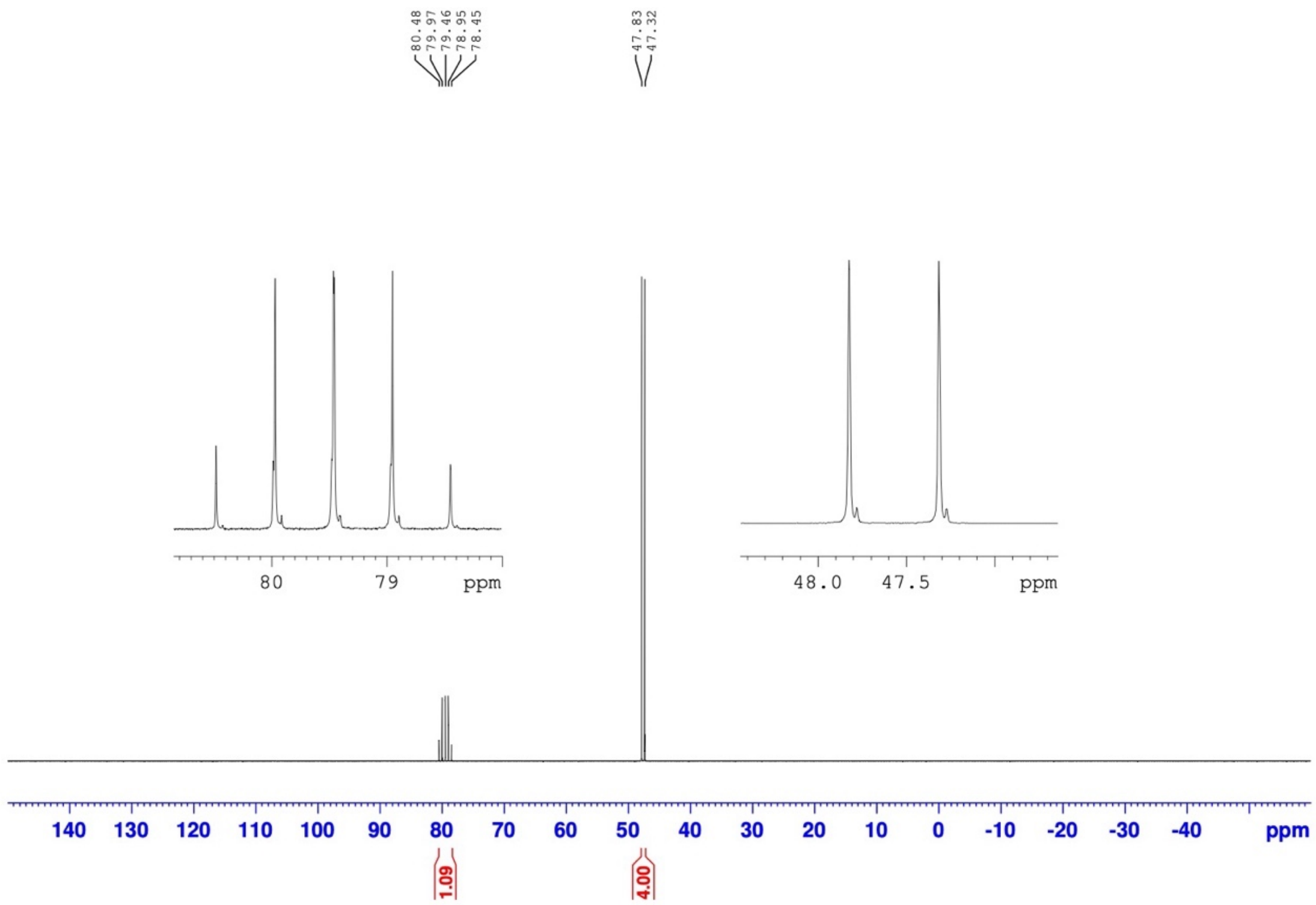

Figure S226. ^{19}F NMR spectrum of (Z) 4-oxo-4-((3'-(pentafluoro-λ^6-sulfanyl)-[1,1'-bi(bicyclo[1.1.1]-pentan)]-3-yl)amino)but-2-enoic acid (**S-26**) in Acetone-*d₆*.

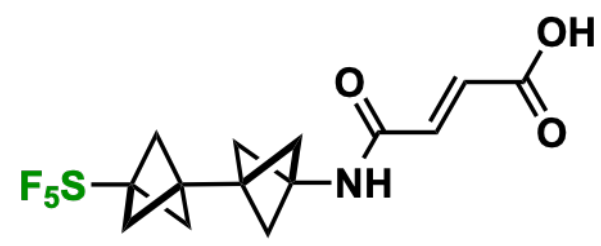

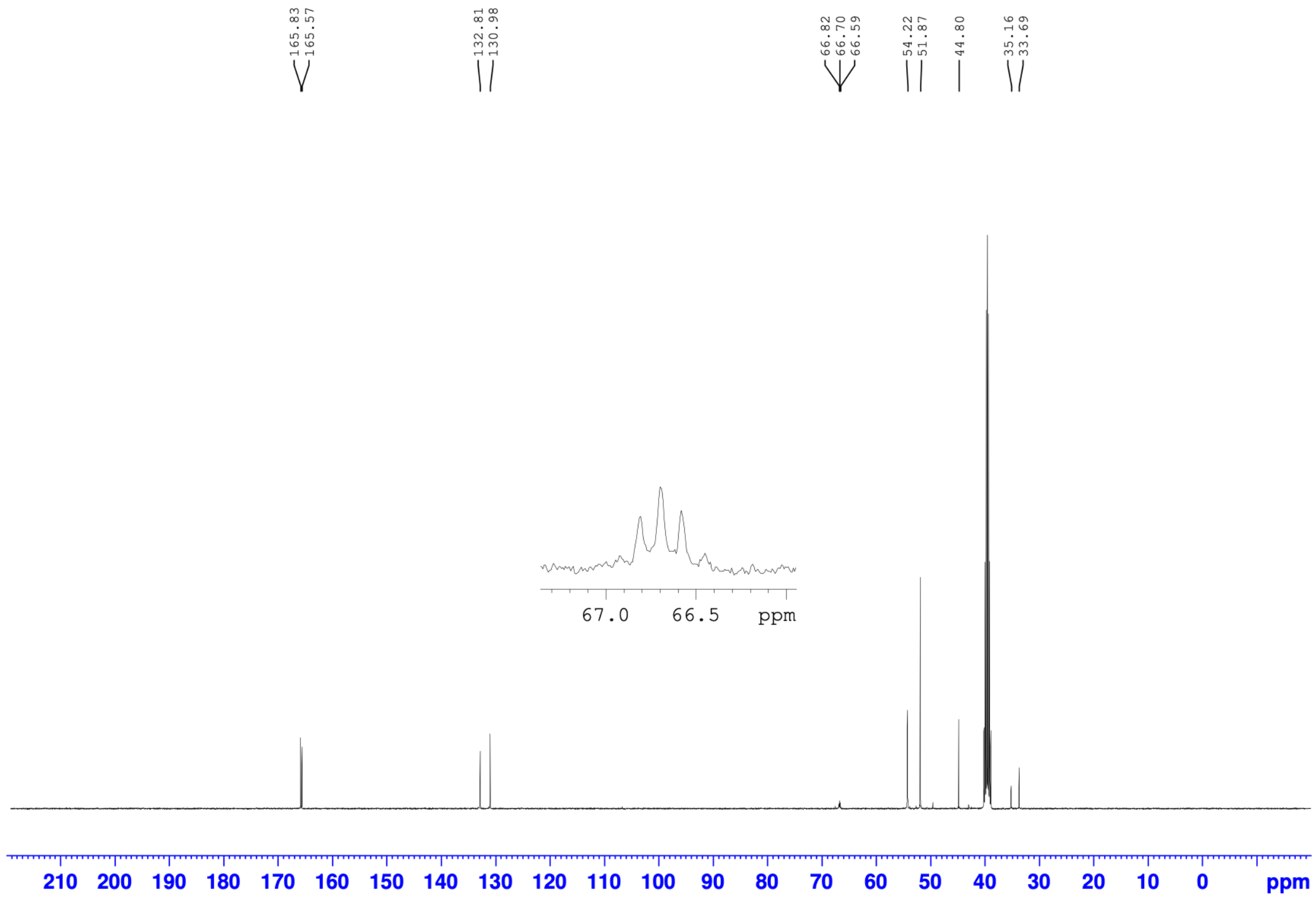

Figure S227. $^{13}C\{^1H\}$ NMR spectrum of (Z) 4-oxo-4-((3'-(pentafluoro-λ^6-sulfanyl)-[1,1'-bi(bicyclo[1.1.1]-pentan)]-3-yl)amino)but-2-enoic acid (**S-26**) in Acetone-*d₆*.

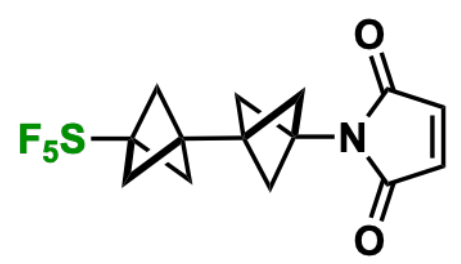

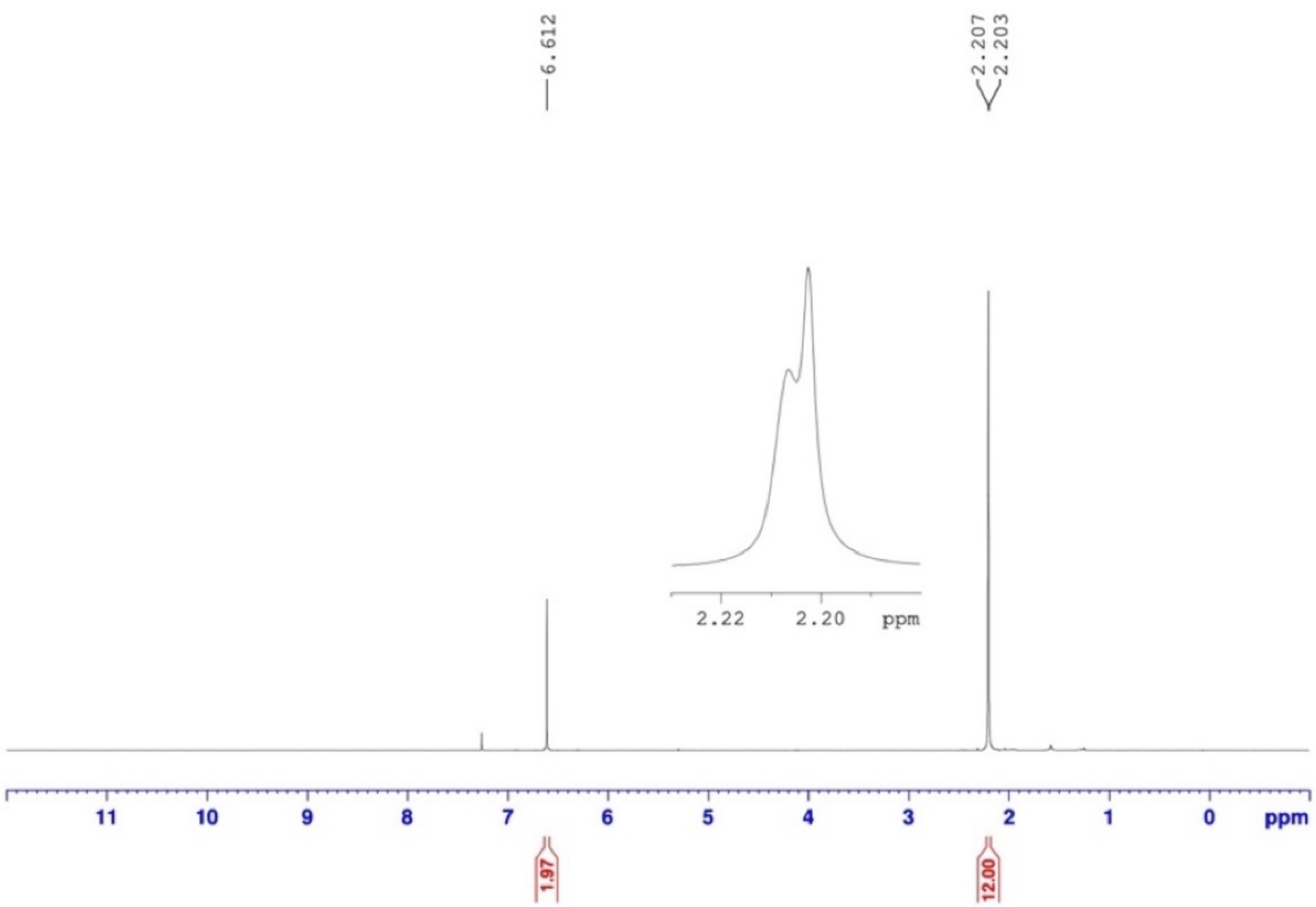

Figure S228. ^{1}H NMR spectrum of 1-(3'-(pentafluoro-λ^{6}-sulfanyl)-[1,1'-bi(bicyclo[1.1.1]pentan)]-3-yl)-1*H*-pyrrole-2,5-dione (**50**) in $CDCl_3$.

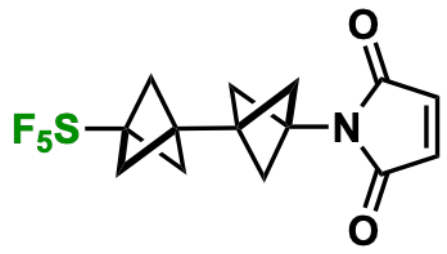

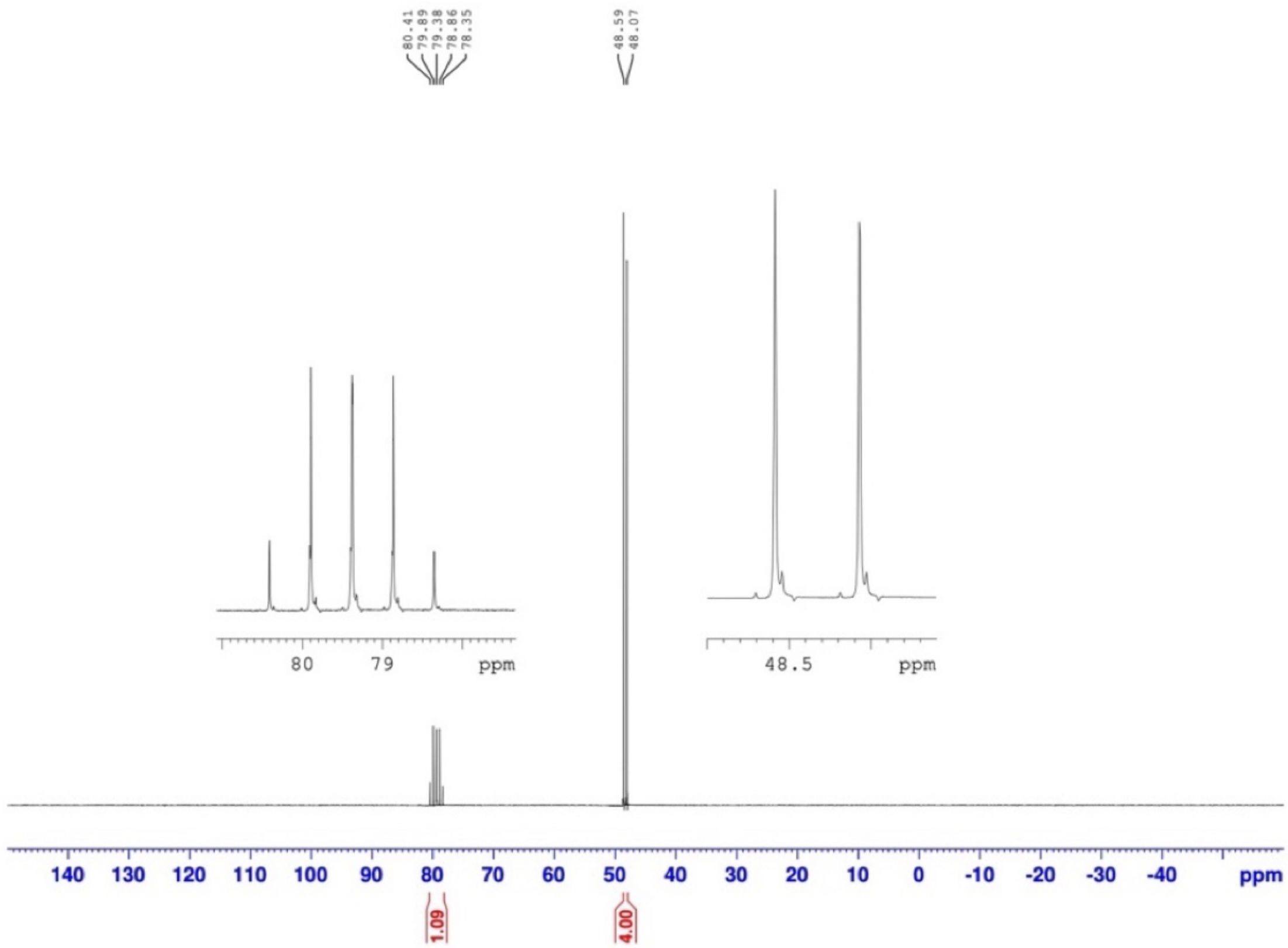

Figure S229. ^{19}F NMR spectrum of 1-(3'-(pentafluoro-λ^6-sulfanyl)-[1,1'-bi(bicyclo[1.1.1]pentan)]-3-yl)-1*H*-pyrrole-2,5-dione (**50**) in $CDCl_3$.

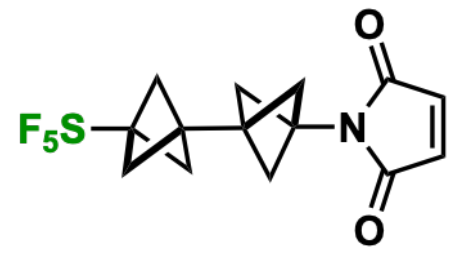

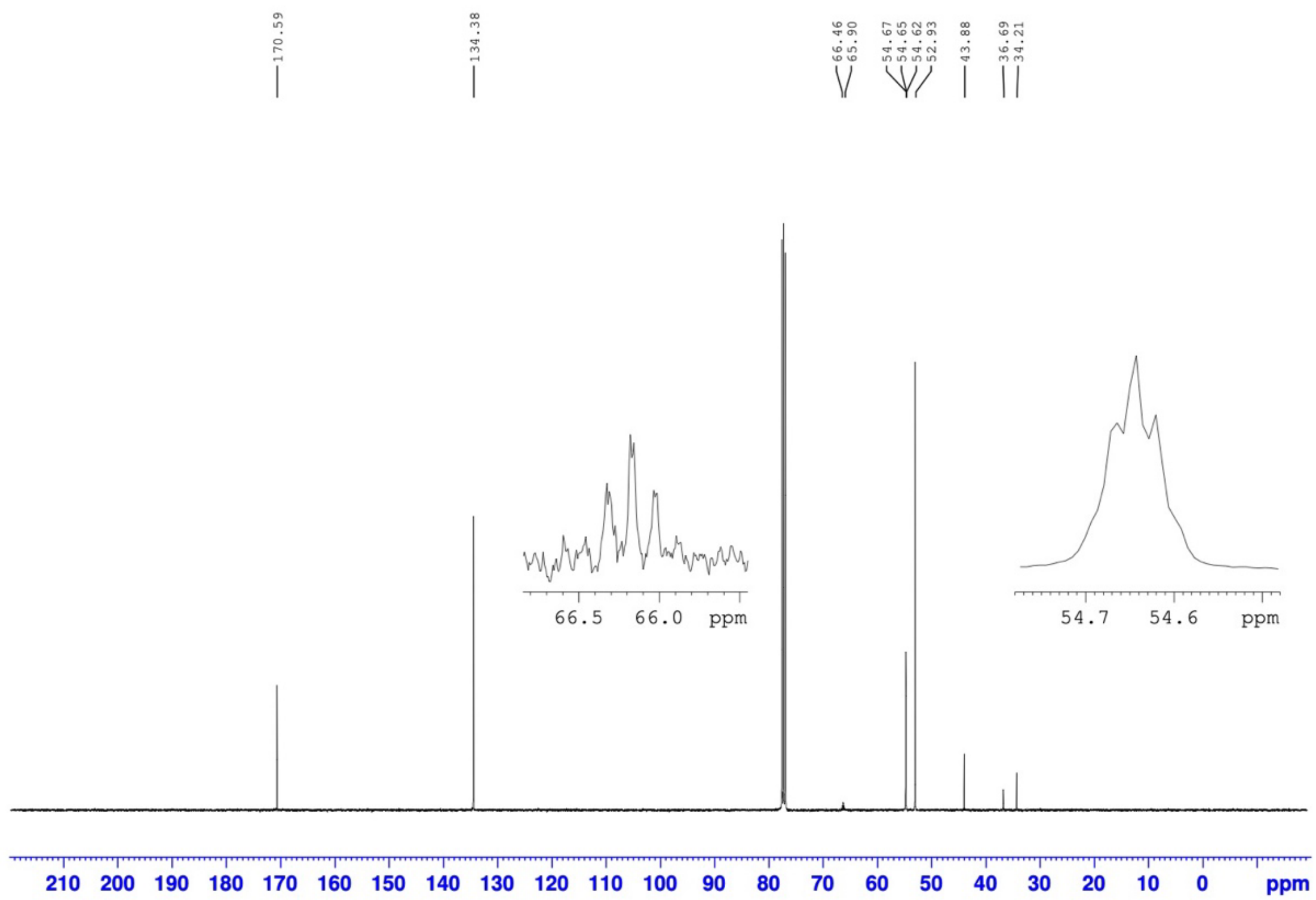

Figure S230. $^{13}C\{^{1}H\}$ NMR spectrum of 1-(3'-(pentafluoro-λ^6-sulfanyl)-[1,1'-bi(bicyclo[1.1.1]pentan)]-3-yl)-1*H*-pyrrole-2,5-dione (**50**) in $CDCl_3$.

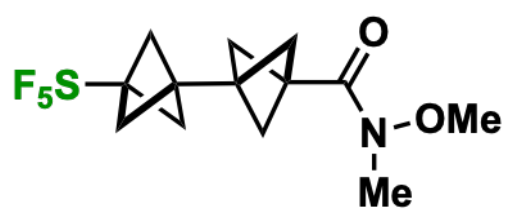

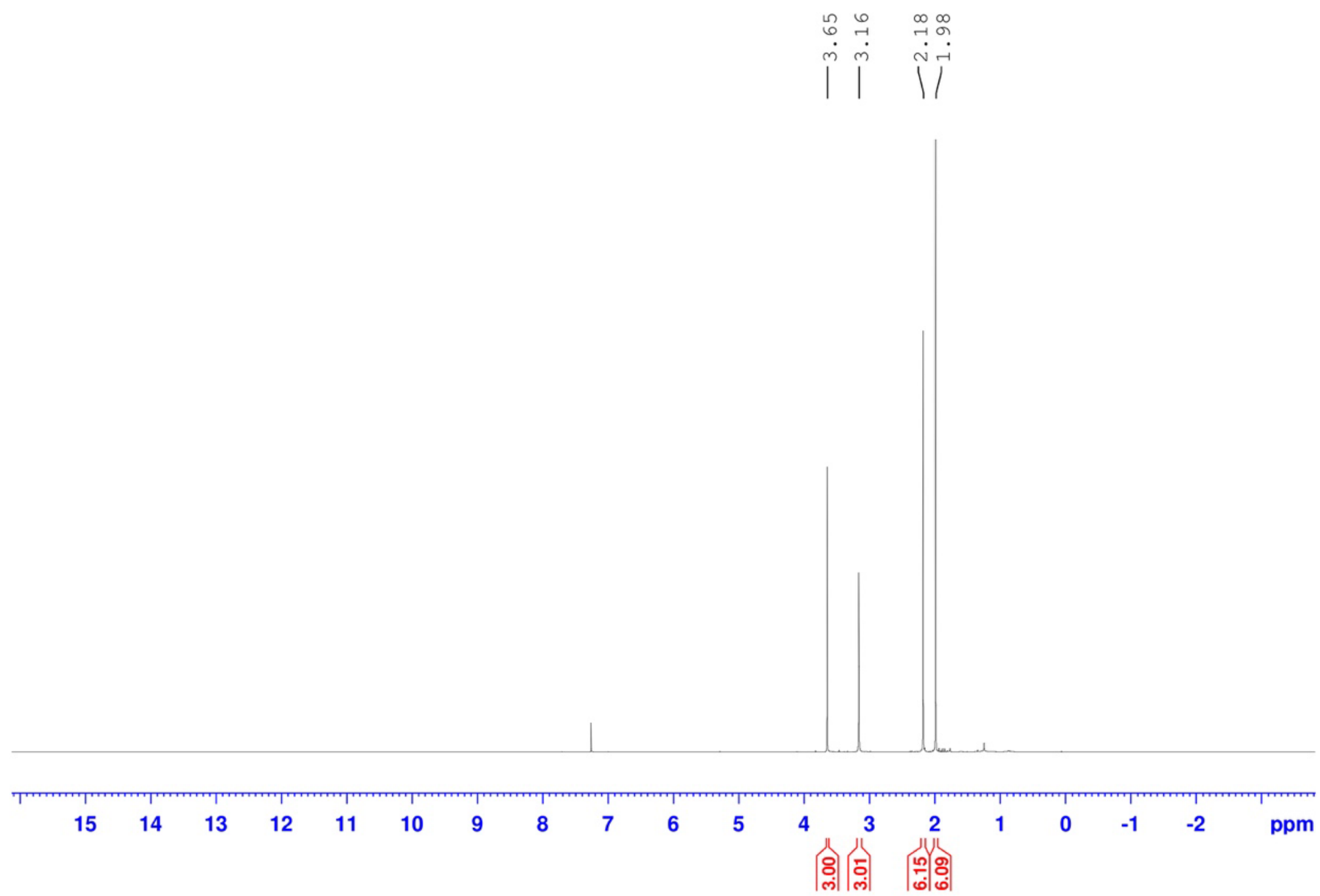

Figure S231. ^{1}H NMR spectrum of *N*-methoxy-*N*-methyl-3'-(pentafluoro-λ^6-sulfanyl)-[1,1'-bi(bicyclo[1.1.1]pentane)]-3-carboxamide (**51**) in $CDCl_3$.

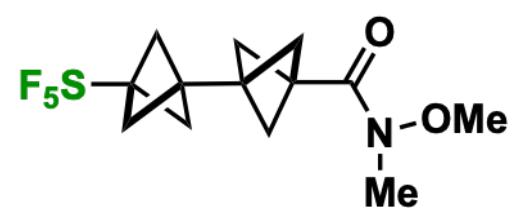

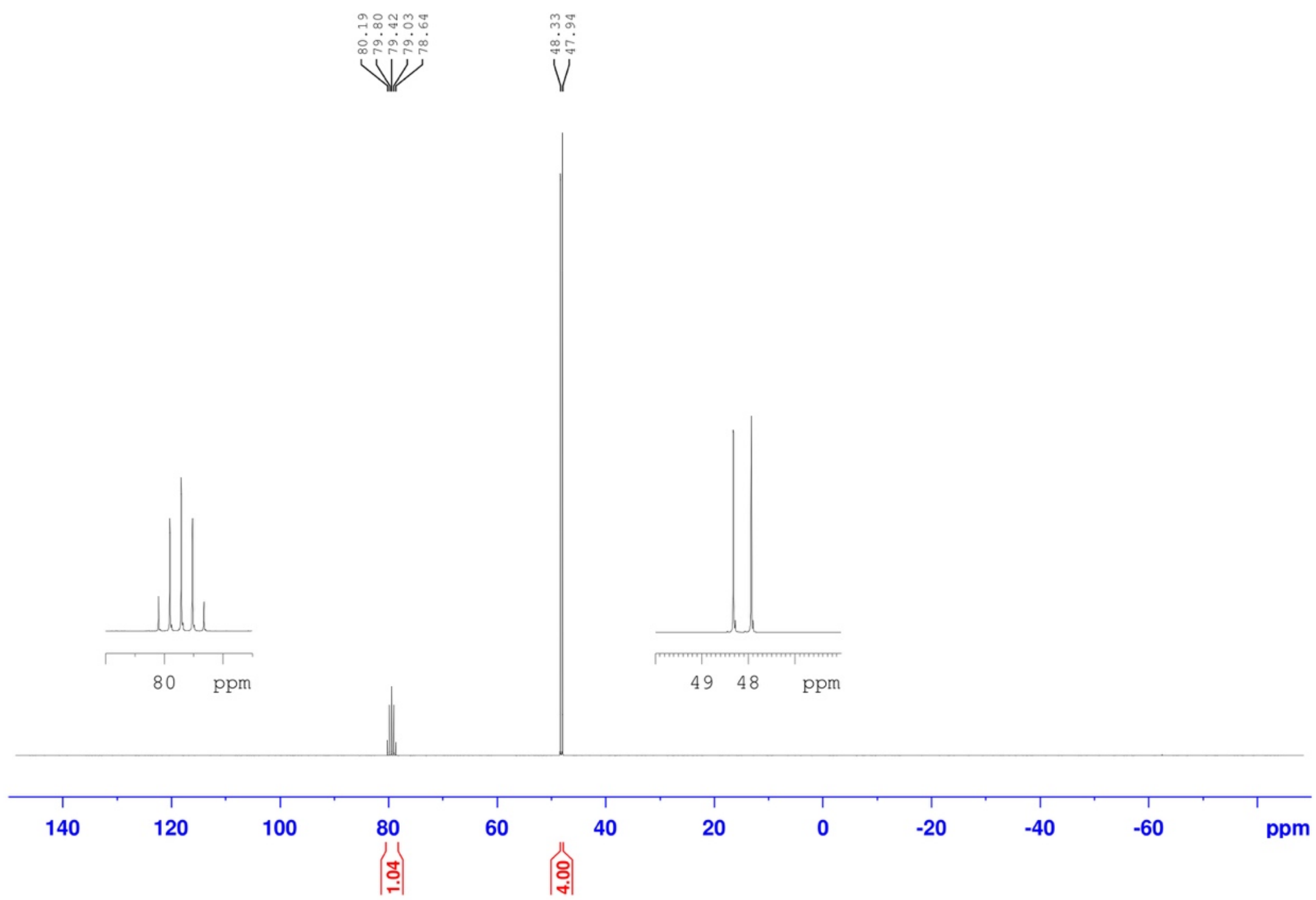

Figure S232. ^{19}F NMR spectrum of *N*-methoxy-*N*-methyl-3'-(pentafluoro-λ^6-sulfanyl)-[1,1'-bi(bicyclo[1.1.1]pentane)]-3-carboxamide (**51**) in $CDCl_3$.

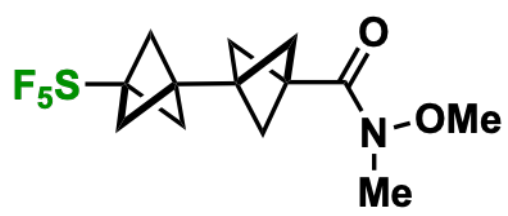

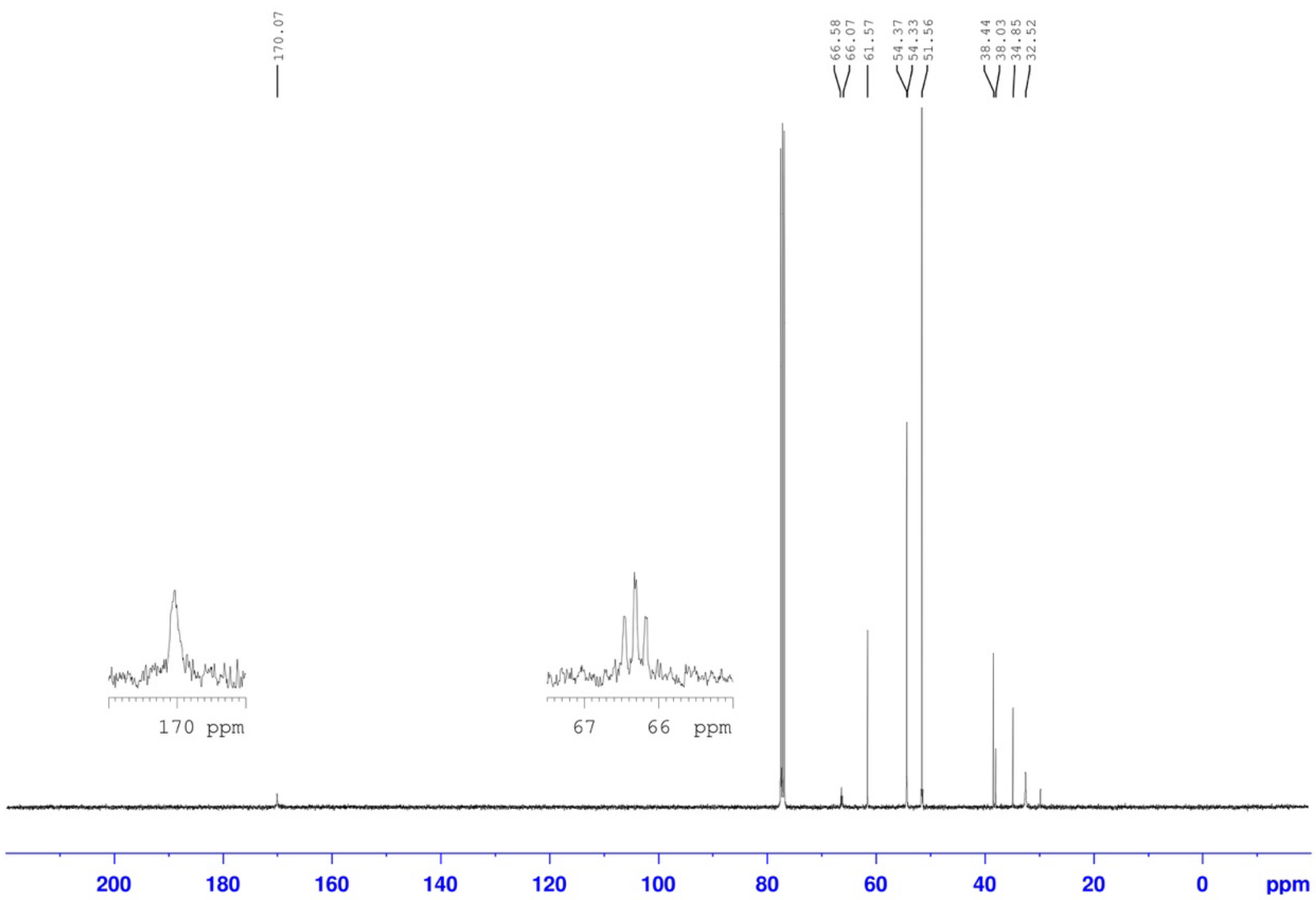

Figure S233. $^{13}C\{^{1}H\}$ NMR spectrum of *N*-methoxy-*N*-methyl-3'-(pentafluoro-λ^6-sulfanyl)-[1,1'-bi(bicyclo[1.1.1]pentane)]-3-carboxamide (**51**) in $CDCl_3$.

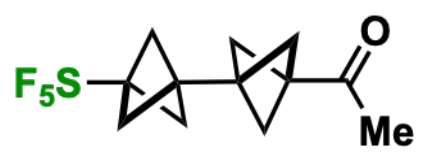

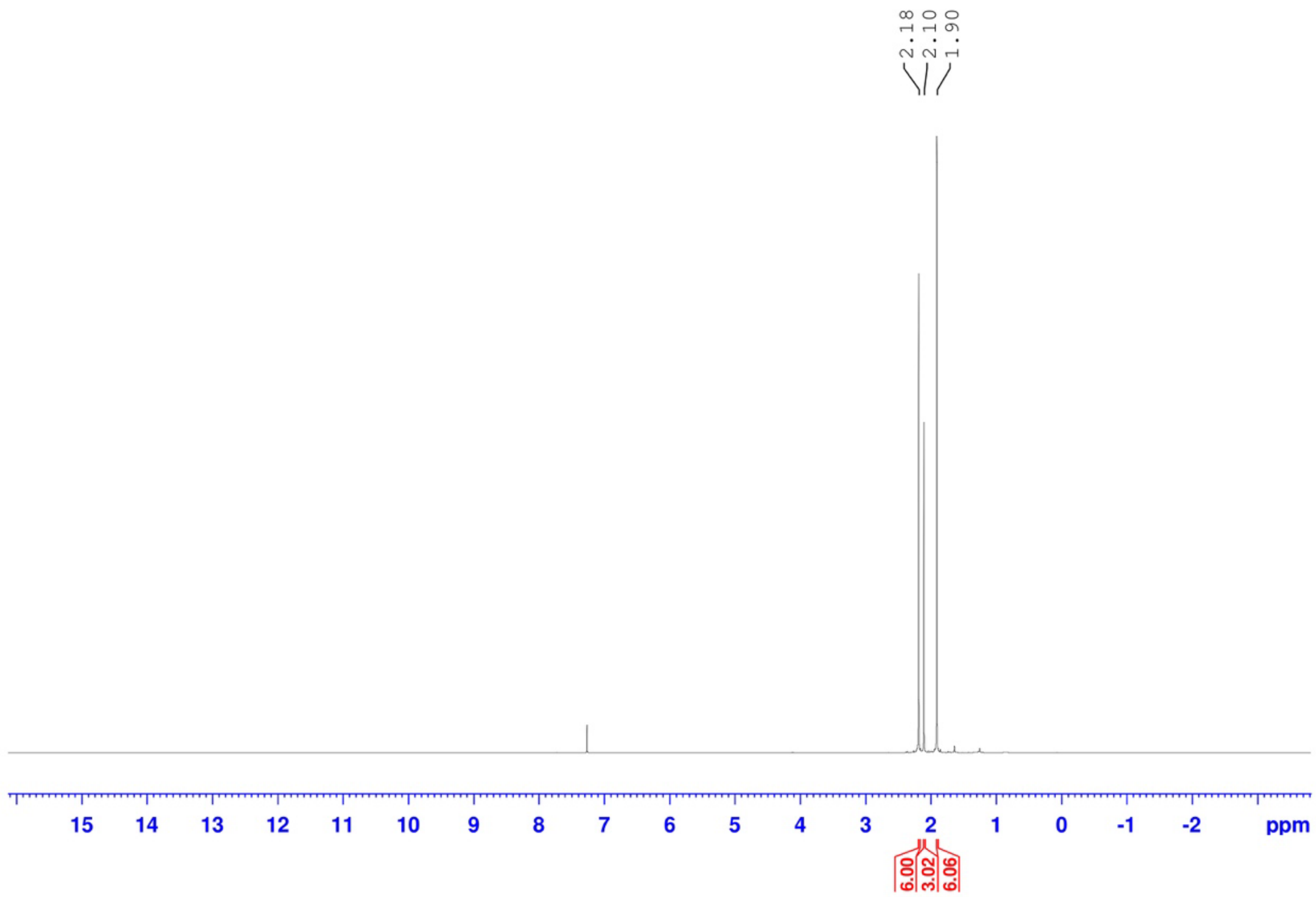

Figure S234. ^{1}H NMR spectrum of 1-(3'-(pentafluoro-λ^6-sulfanyl)-[1,1'-bi(bicyclo[1.1.1]pentan)]-3-yl)ethan-1-one (**32**) in $CDCl_3$.

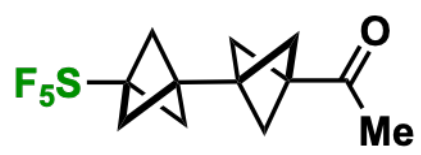

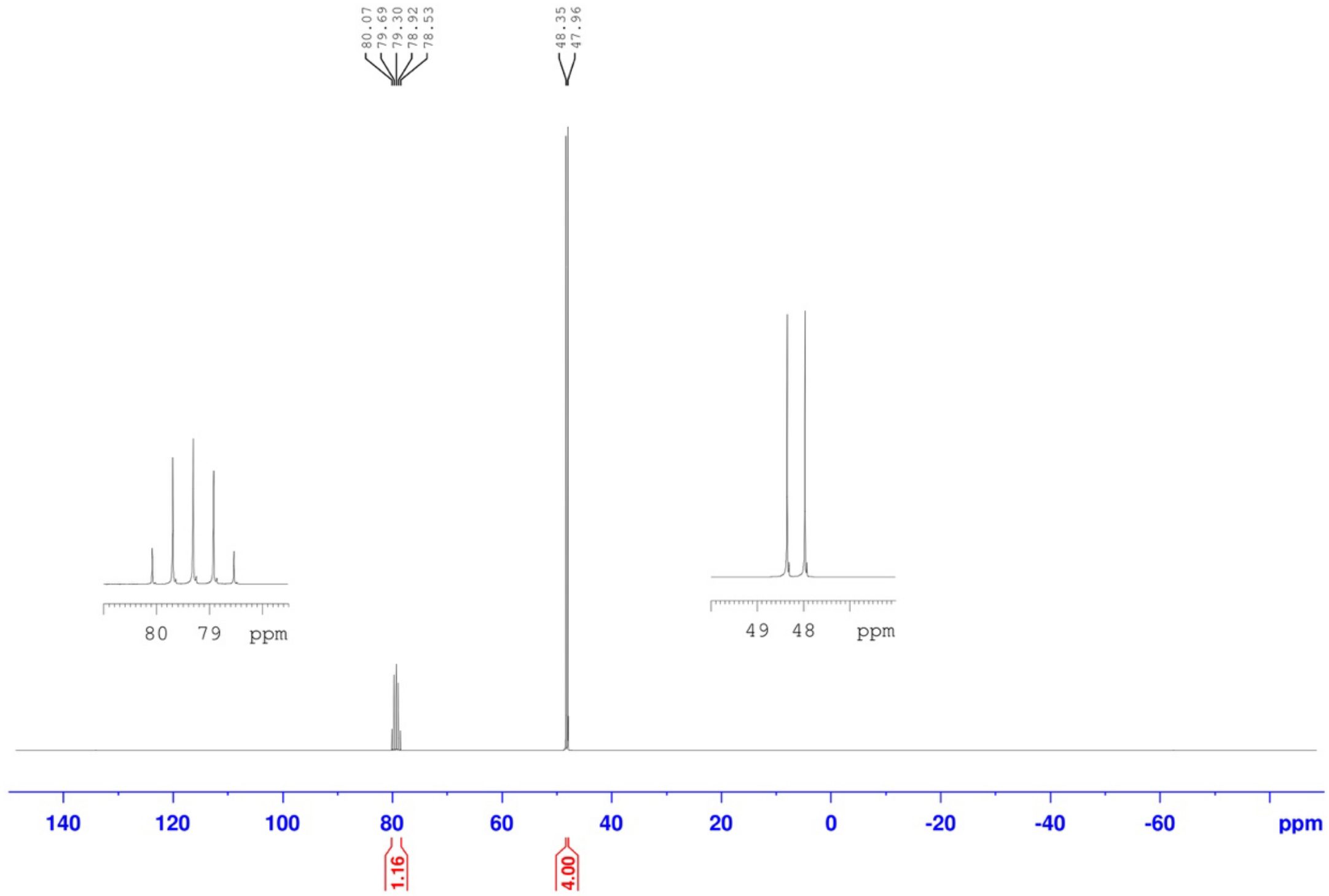

Figure S235. ^{19}F NMR spectrum of 1-(3'-(pentafluoro-λ^6-sulfanyl)-[1,1'-bi(bicyclo[1.1.1]pentan)]-3-yl)ethan-1-one (**32**) in $CDCl_3$.

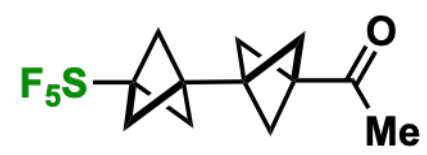

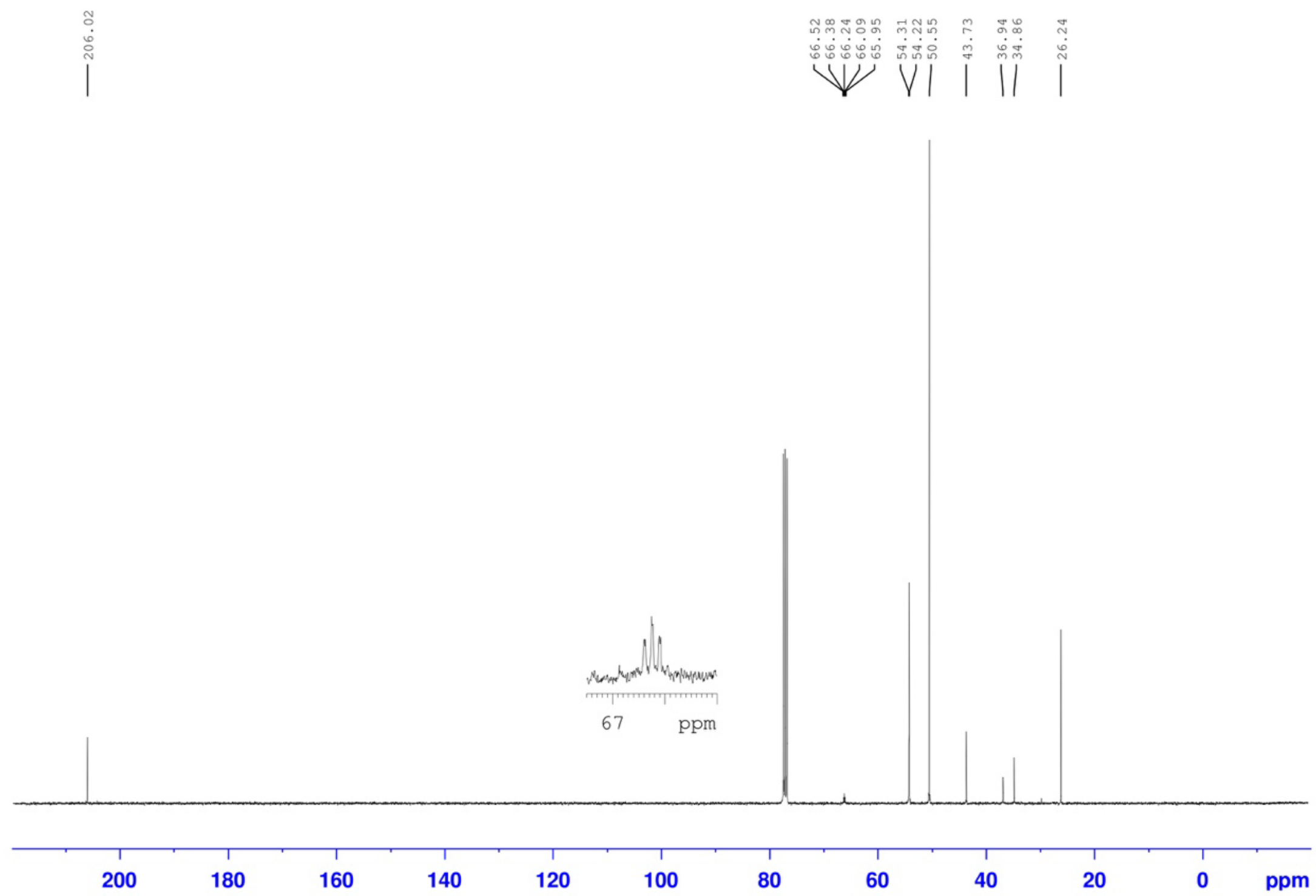

Figure S236. $^{13}C\{^1H\}$ NMR spectrum of 1-(3'-(pentafluoro-λ^6-sulfanyl)-[1,1'-bi(bicyclo[1.1.1]pentan)]-3-yl)ethan-1-one (**32**) in $CDCl_3$.

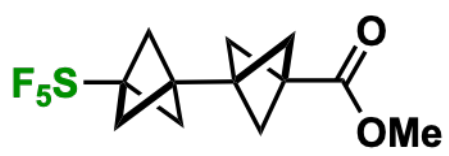

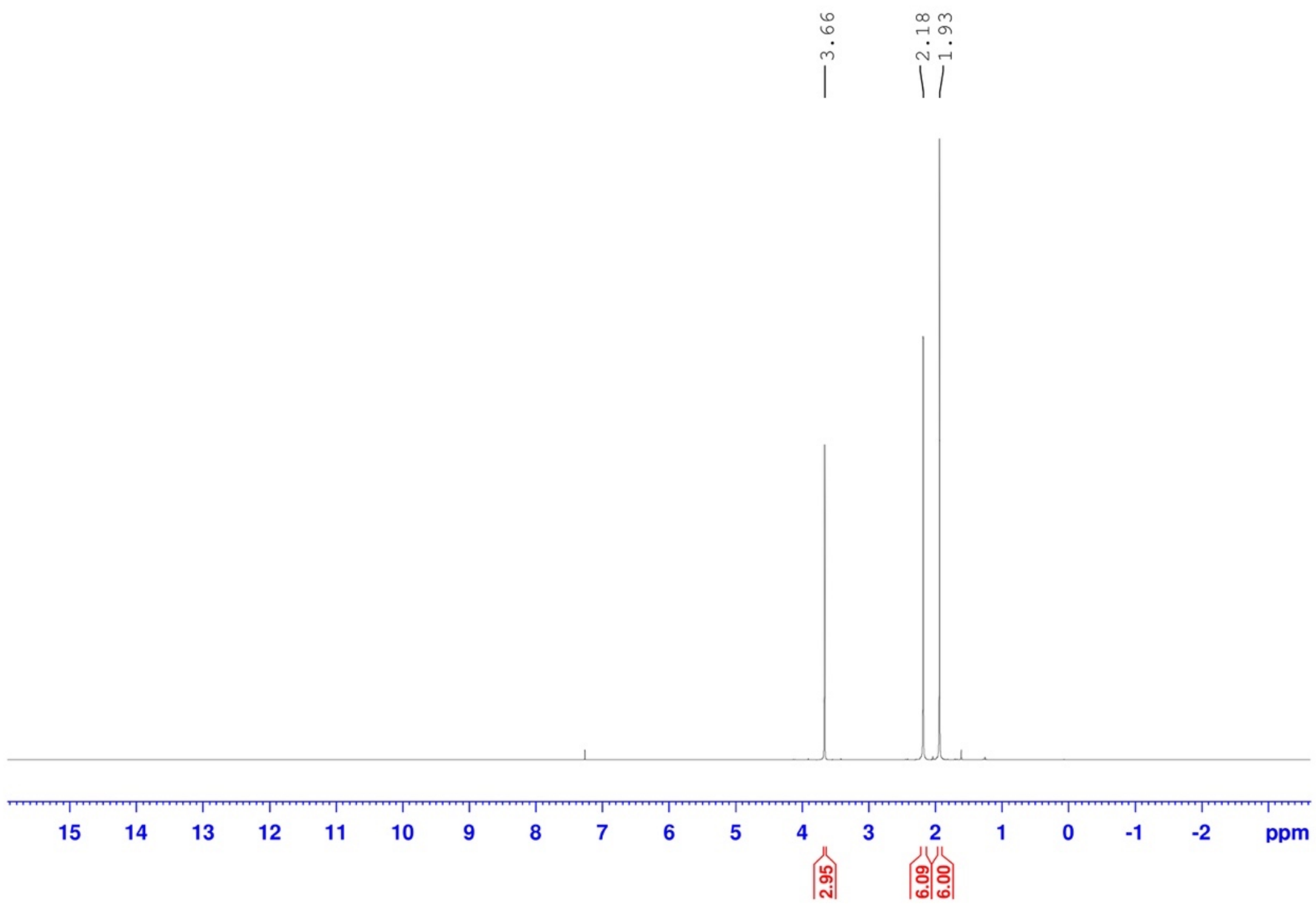

Figure S237. ^{1}H NMR spectrum of methyl 3'-(pentafluoro-λ^6-sulfanyl)-[1,1'-bi(bicyclo[1.1.1]pentane)]-3-carboxylate (**S-27**) in $CDCl_3$.

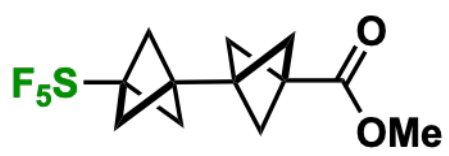

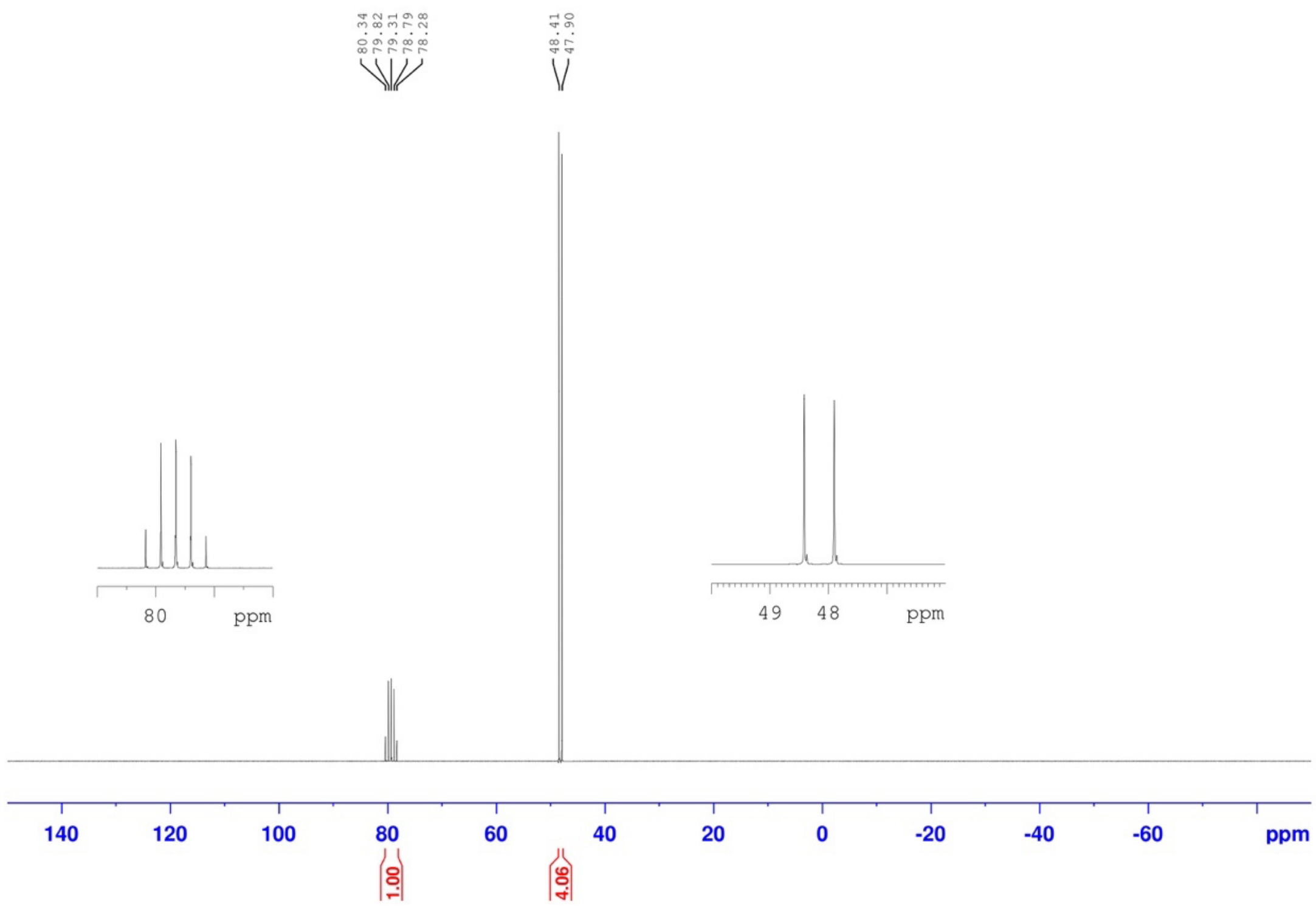

Figure S238. ^{19}F NMR spectrum of methyl 3'-(pentafluoro-λ^6-sulfanyl)-[1,1'-bi(bicyclo[1.1.1]pentane)]-3-carboxylate (**S-27**) in $CDCl_3$.

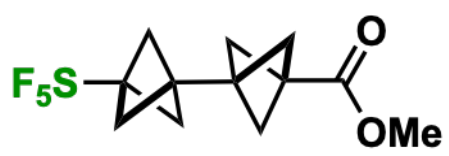

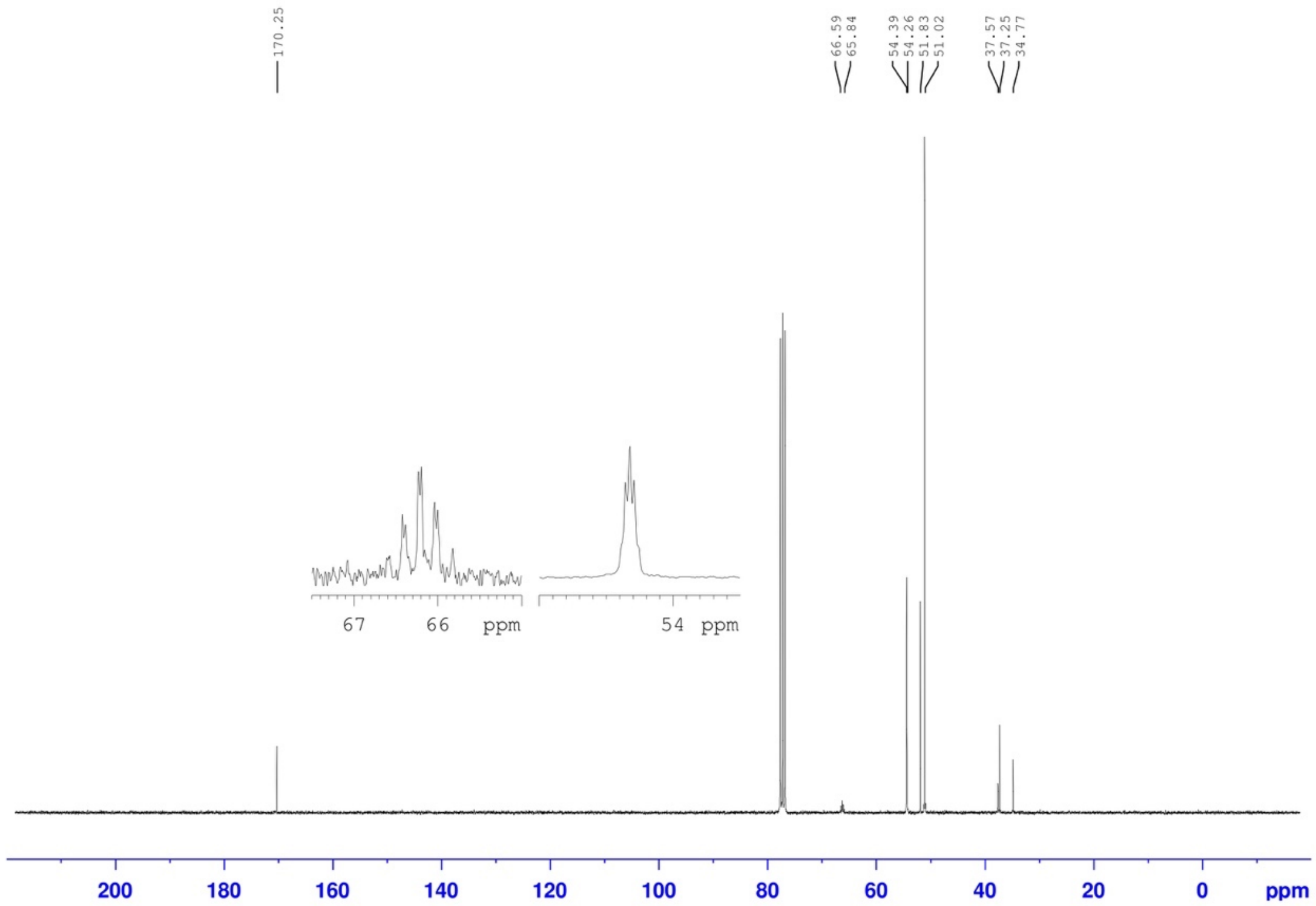

Figure S239. $^{13}C\{^1H\}$ NMR spectrum of methyl 3'-(pentafluoro-λ^6-sulfanyl)-[1,1'-bi(bicyclo[1.1.1]pentane)]-3-carboxylate (**S-27**) in $CDCl_3$.

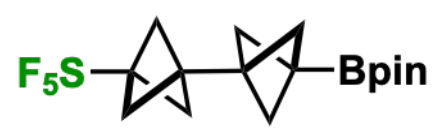

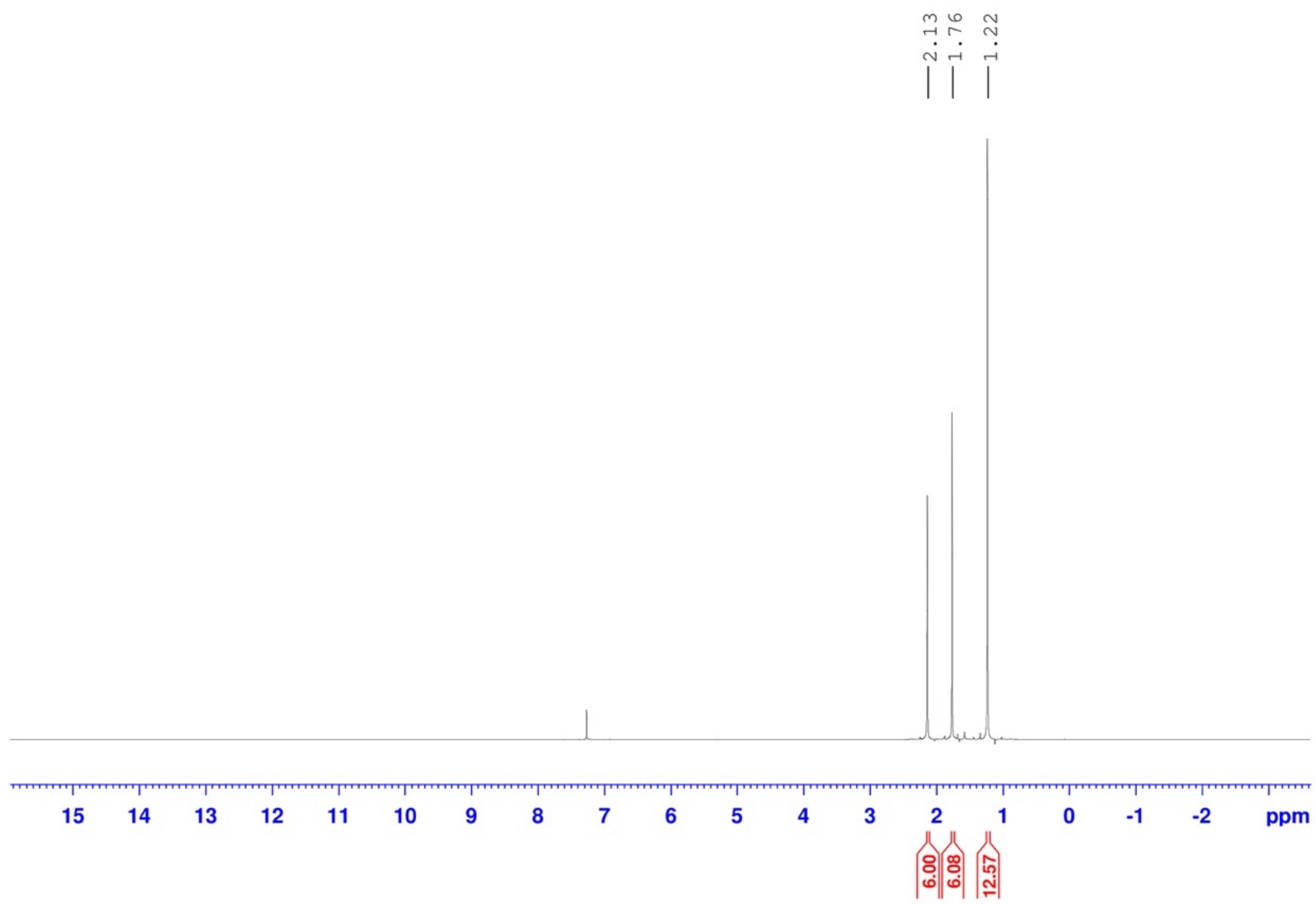

Figure S240. ^{1}H NMR spectrum of 4,4,5,5-tetramethyl-2-(3'-(pentafluoro-λ^6-sulfanyl)-[1,1'-bi(bicyclo[1.1.1]pentan)]-3-yl)-1,3,2-dioxaborolane (**52**) in $CDCl_3$.

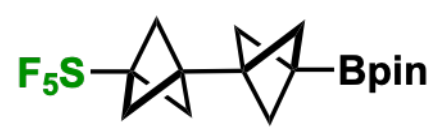

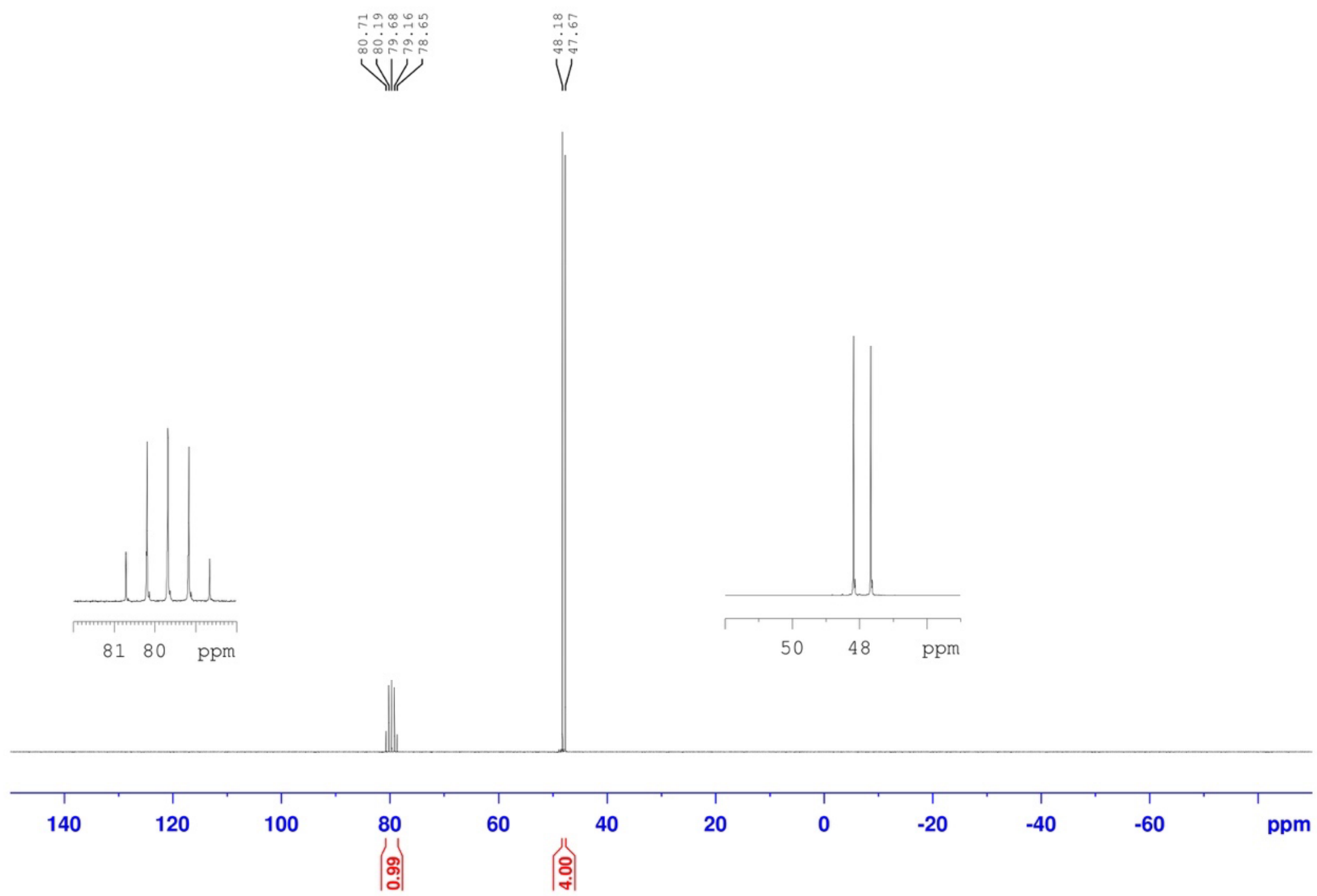

Figure S241. ^{19}F NMR spectrum of 4,4,5,5-tetramethyl-2-(3'-(pentafluoro-λ^6-sulfanyl)-[1,1'-bi(bicyclo[1.1.1]pentan)]-3-yl)-1,3,2-dioxaborolane (**52**) in $CDCl_3$.

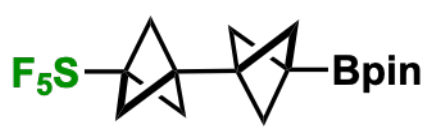

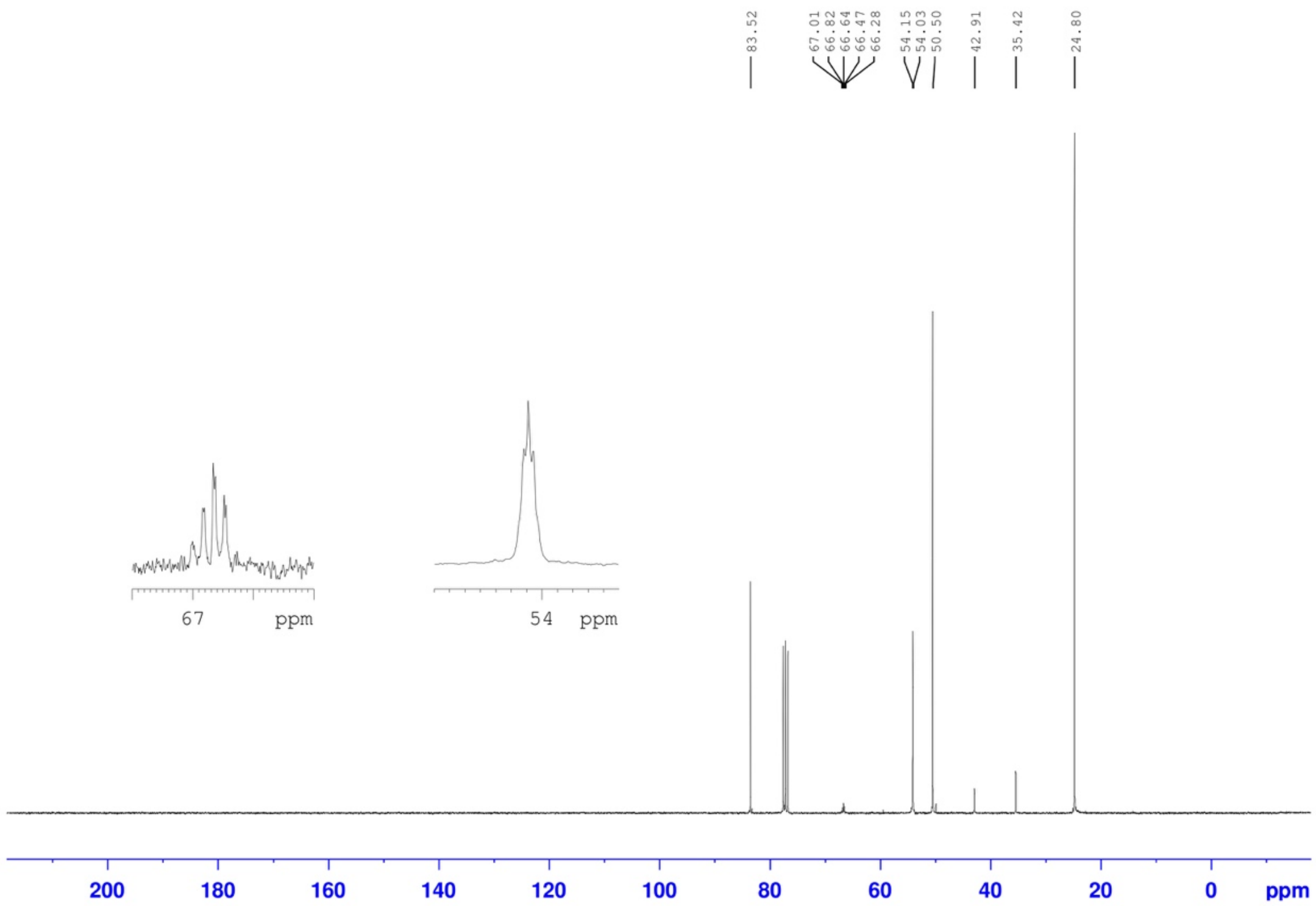

Figure S242. $^{13}C\{^1H\}$ NMR spectrum of 4,4,5,5-tetramethyl-2-(3'-(pentafluoro-λ^6-sulfanyl)-[1,1'-bi(bicyclo[1.1.1]pentan)]-3-yl)-1,3,2-dioxaborolane (**52**) in $CDCl_3$.

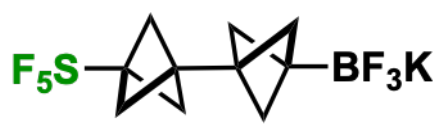

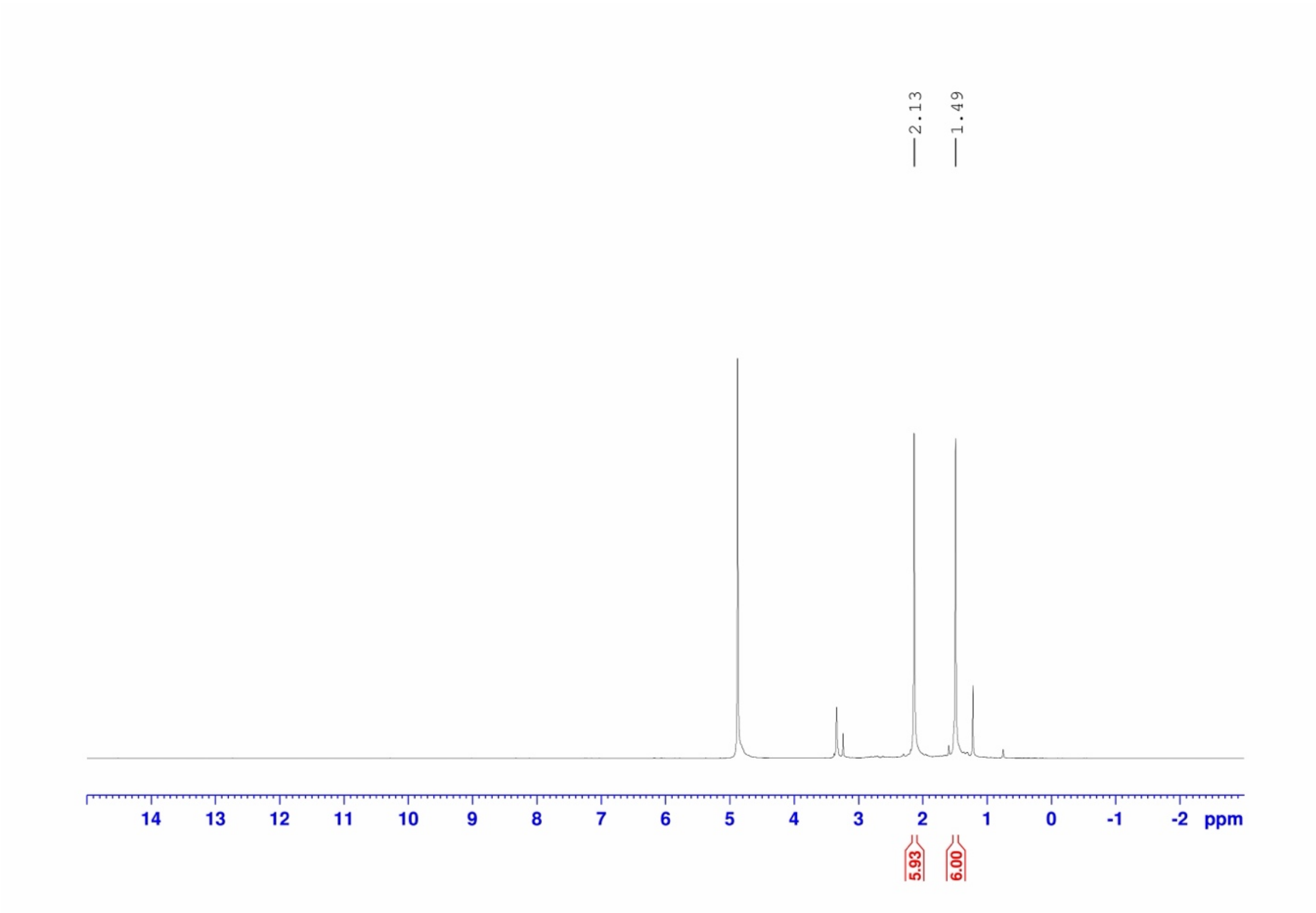

Figure S243. ^{1}H NMR spectrum of trifluoro(3'-(pentafluoro-λ^6-sulfanyl)-[1,1'-bi(bicyclo[1.1.1]pentan)]-3-yl)--λ^4-borane, potassium salt (**53**) in MeOD-*d₄*.

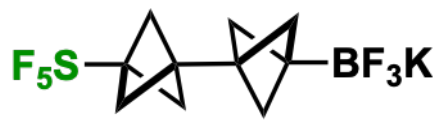

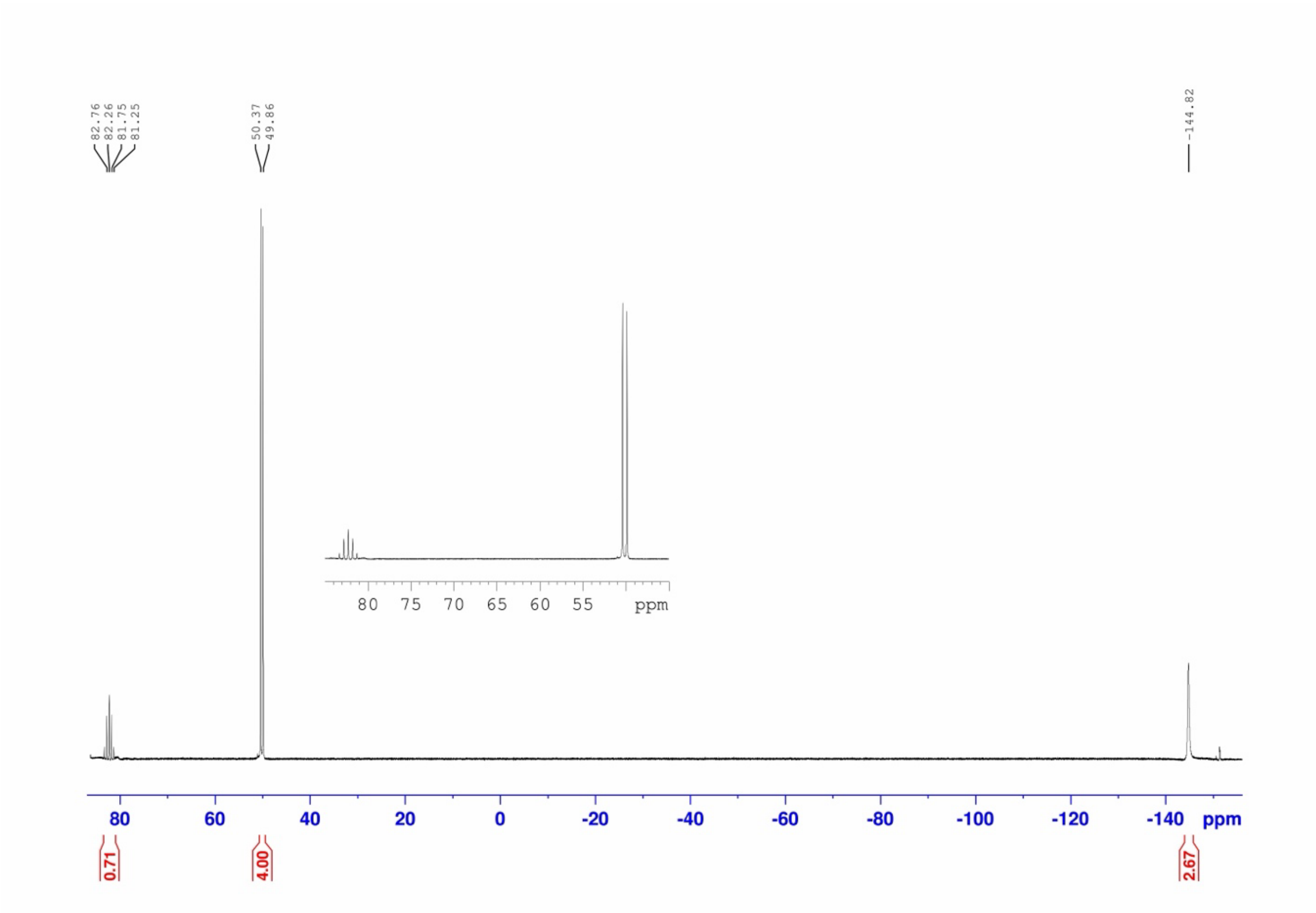

Figure S244. ^{19}F NMR spectrum of trifluoro(3'-(pentafluoro-λ^6-sulfanyl)-[1,1'-bi(bicyclo[1.1.1]pentan)]-3-yl)--λ^4-borane, potassium salt (**53**) in MeOD-d_4.

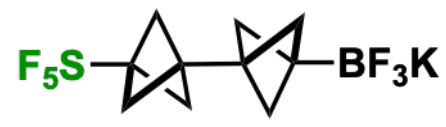

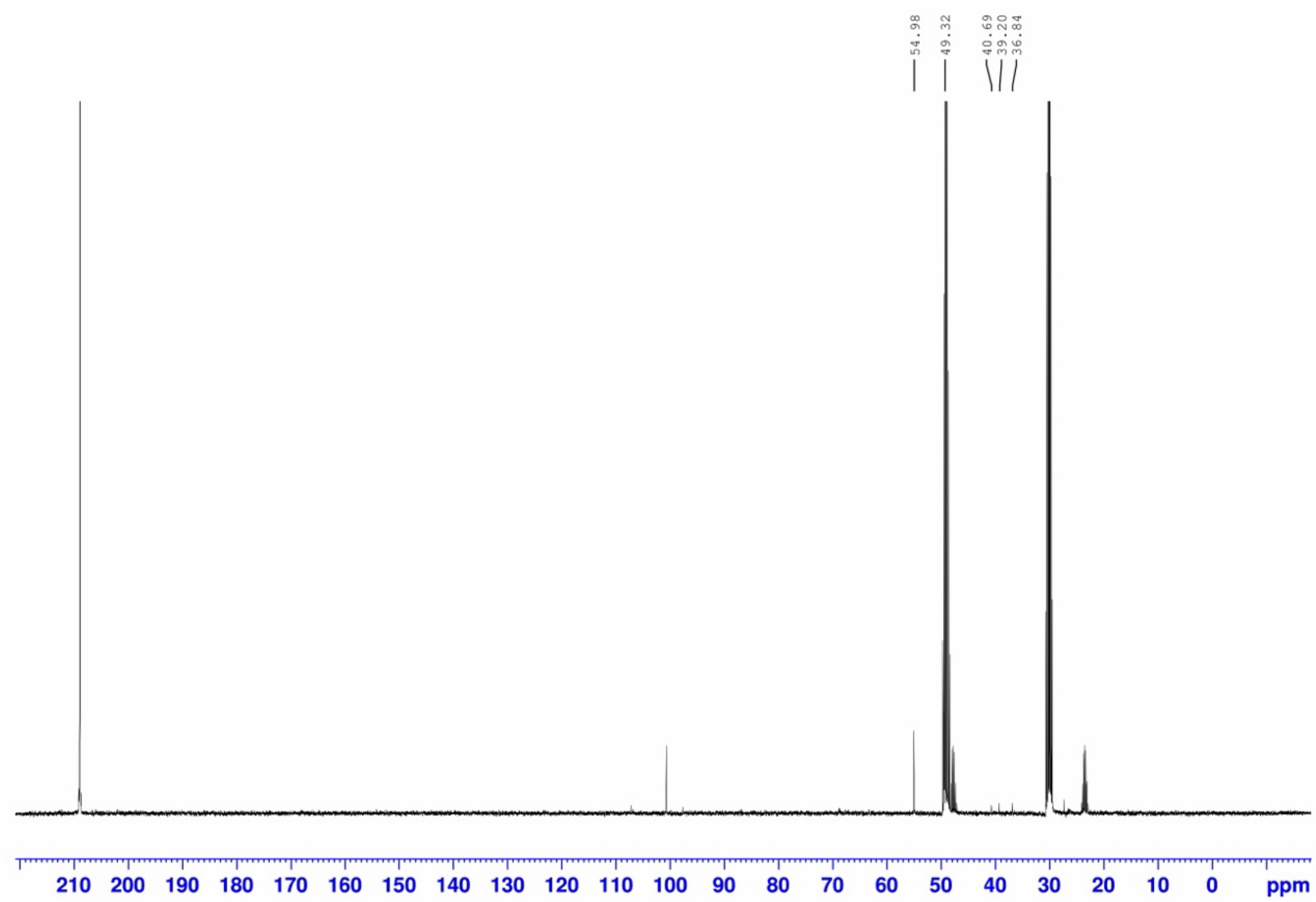

Figure S245. $^{13}C\{^{1}H\}$ NMR spectrum of trifluoro(3'-(pentafluoro-λ^6-sulfanyl)-[1,1'-bi(bicyclo[1.1.1]pentan)]-3-yl)-λ^4-borane, potassium salt (**53**) in MeOD-*d4*:Acetone-*d6*.

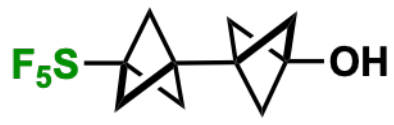

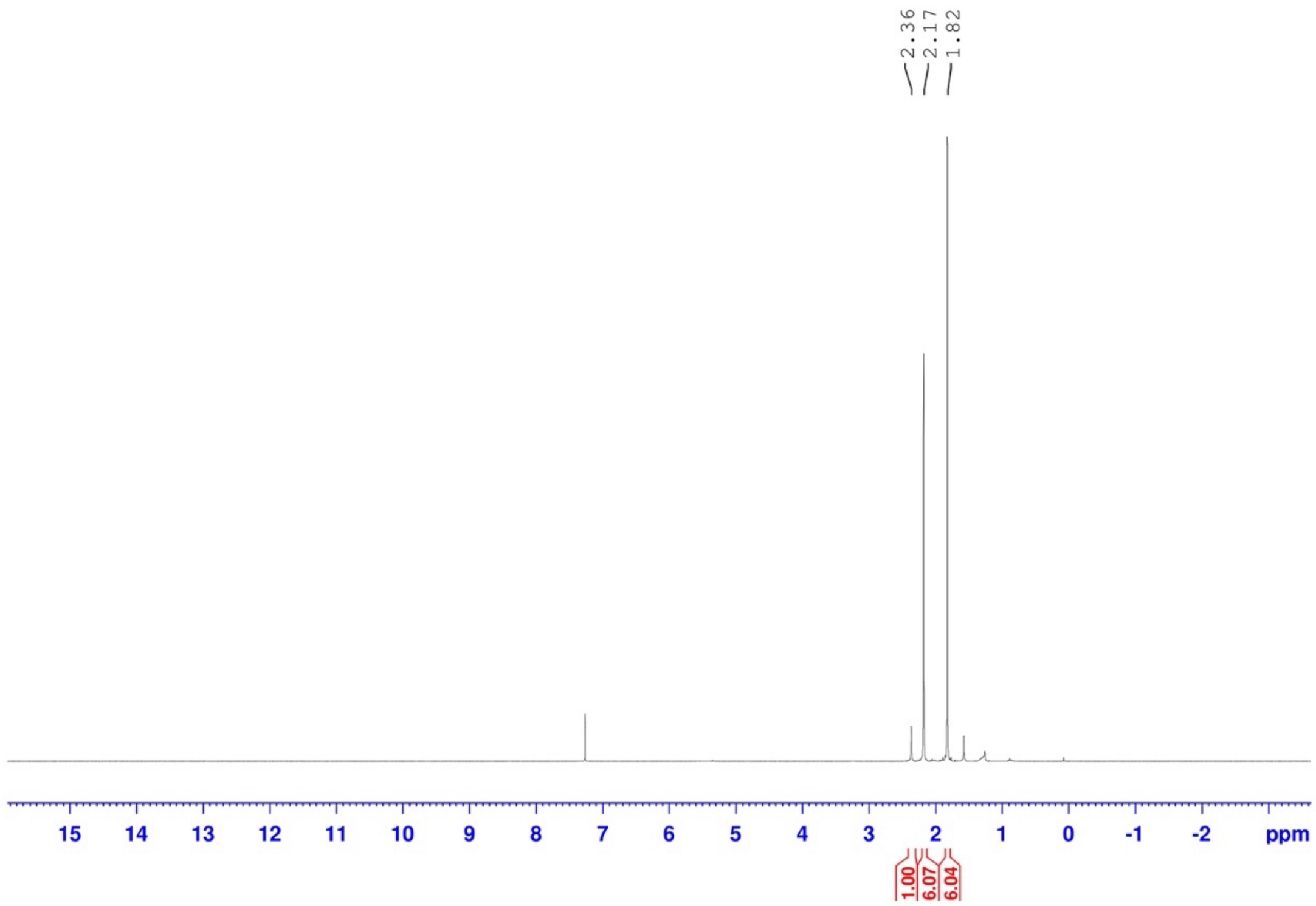

Figure S246. ^{1}H NMR spectrum of 3'-(pentafluoro-λ[6]-sulfanyl)-[1,1'-bi(bicyclo[1.1.1]pentan)]-3-ol (**54**) in $CDCl_3$.

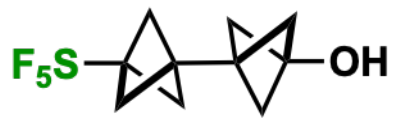

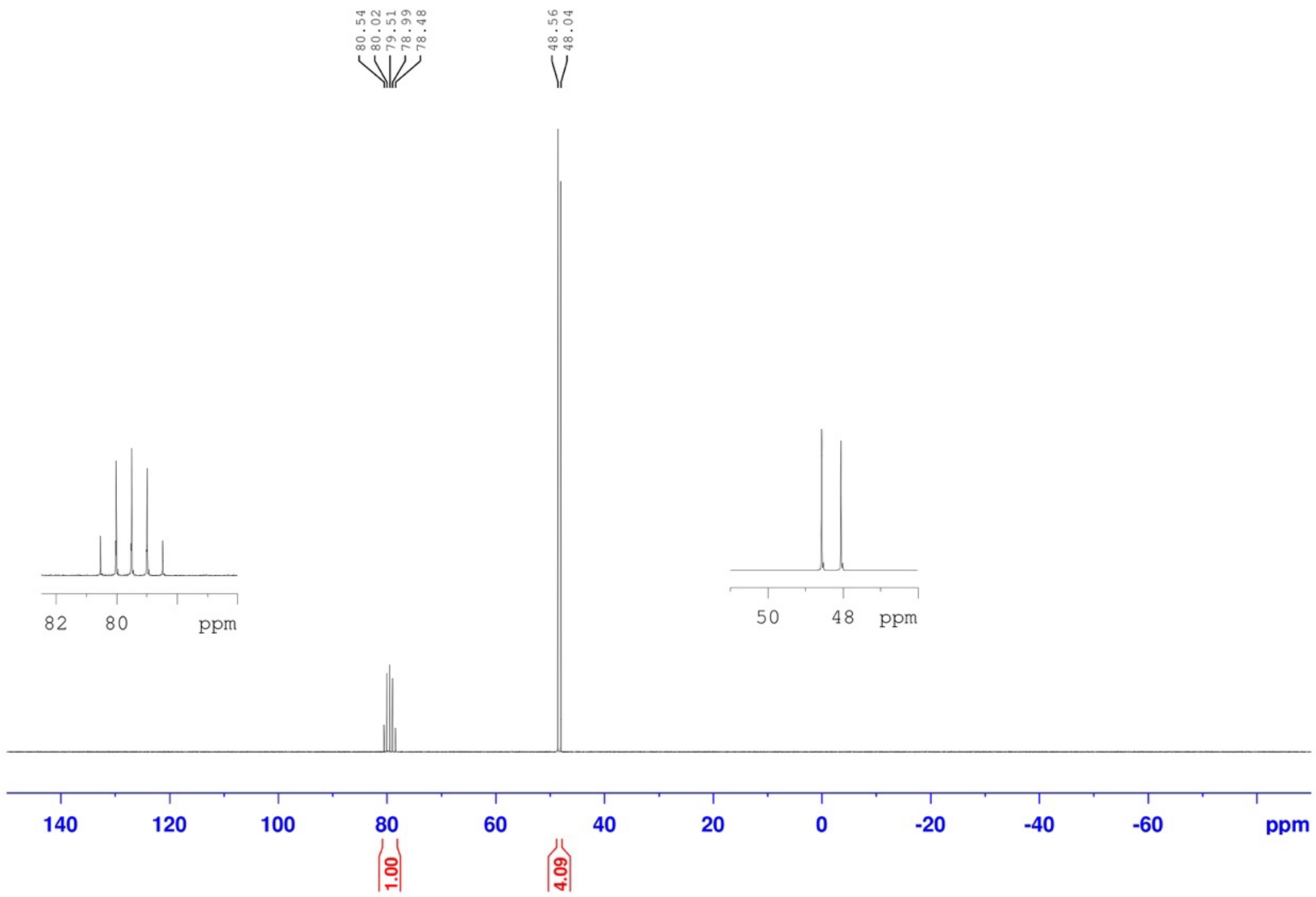

Figure S247. ^{19}F NMR spectrum of 3'-(pentafluoro-λ^6-sulfanyl)-[1,1'-bi(bicyclo[1.1.1]pentan)]-3-ol (**54**) in $CDCl_3$.

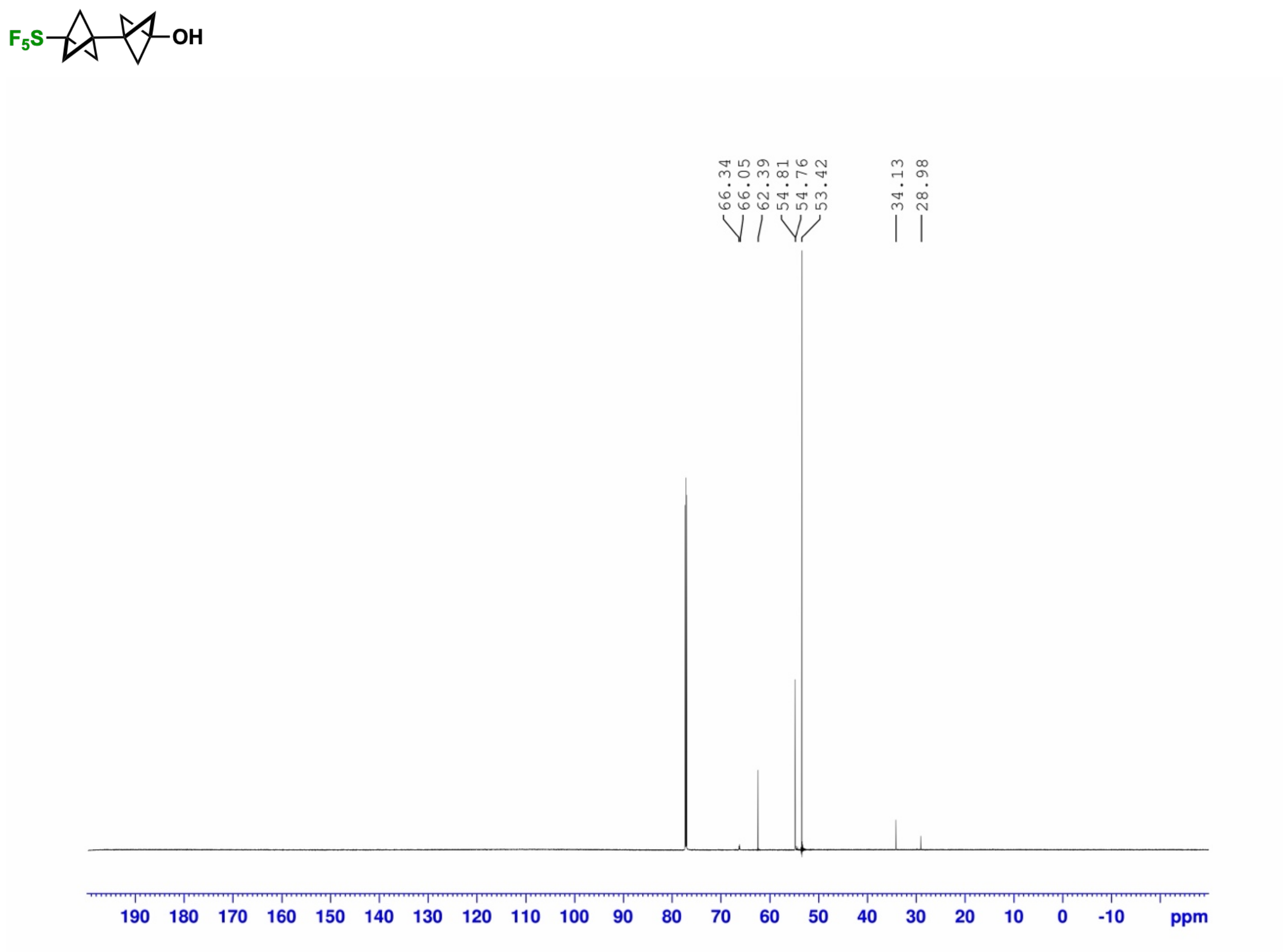

Figure S248. $^{13}C\{^{1}H\}$ NMR spectrum of 3'-(pentafluoro-λ^6-sulfanyl)-[1,1'-bi(bicyclo[1.1.1]pentan)]-3-ol (**54**) in $CDCl_3$.

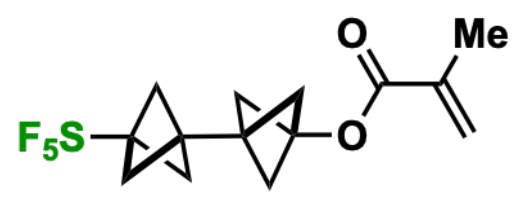

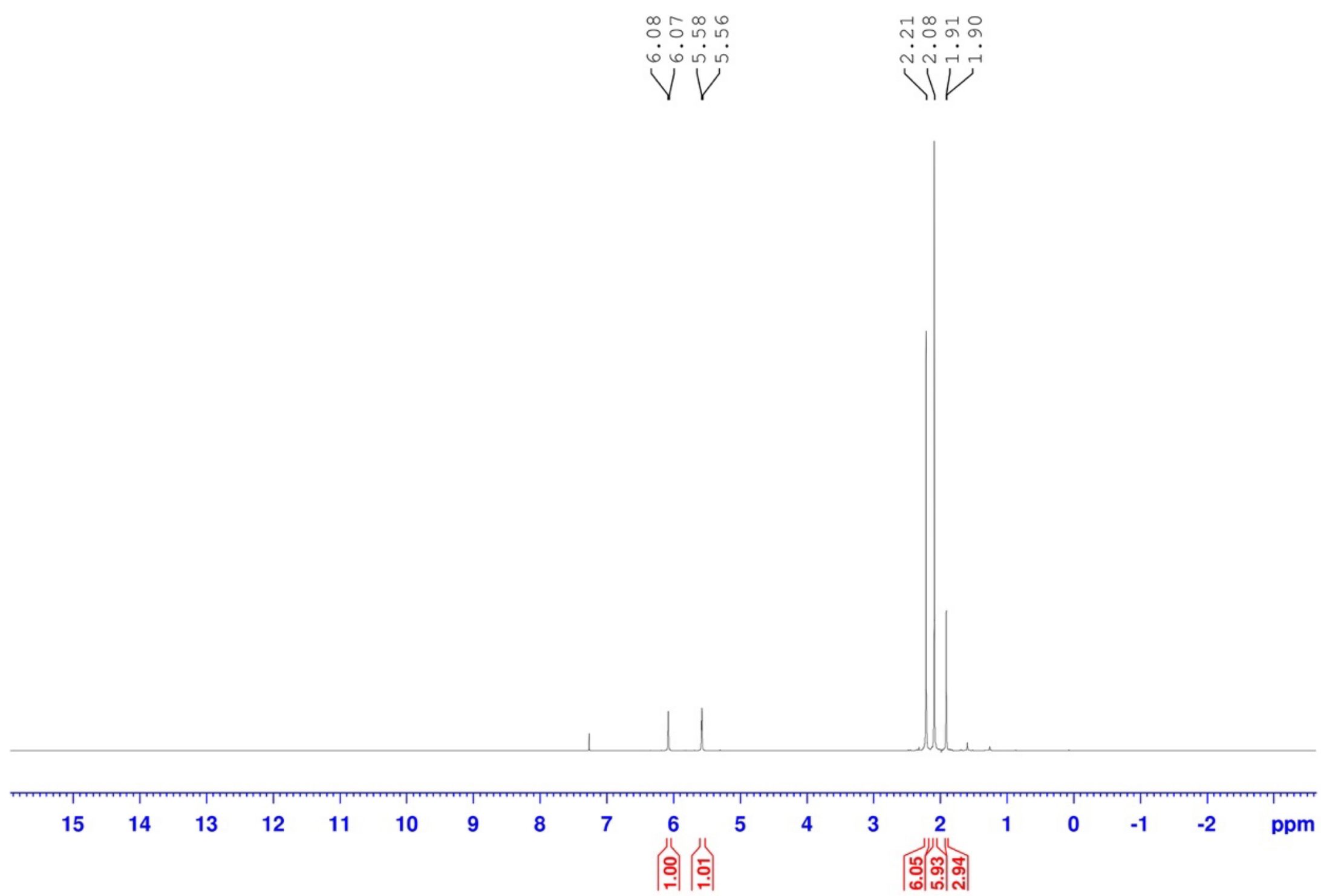

Figure S249. ^{1}H NMR spectrum of 3'-(pentafluoro-λ^6-sulfanyl)-[1,1'-bi(bicyclo[1.1.1]pentan)]-3-yl methacrylate (**55**) in $CDCl_3$.

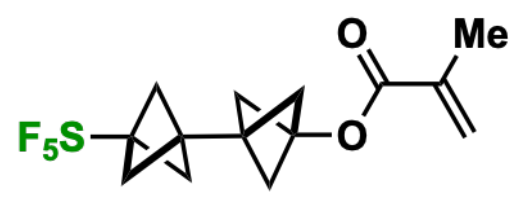

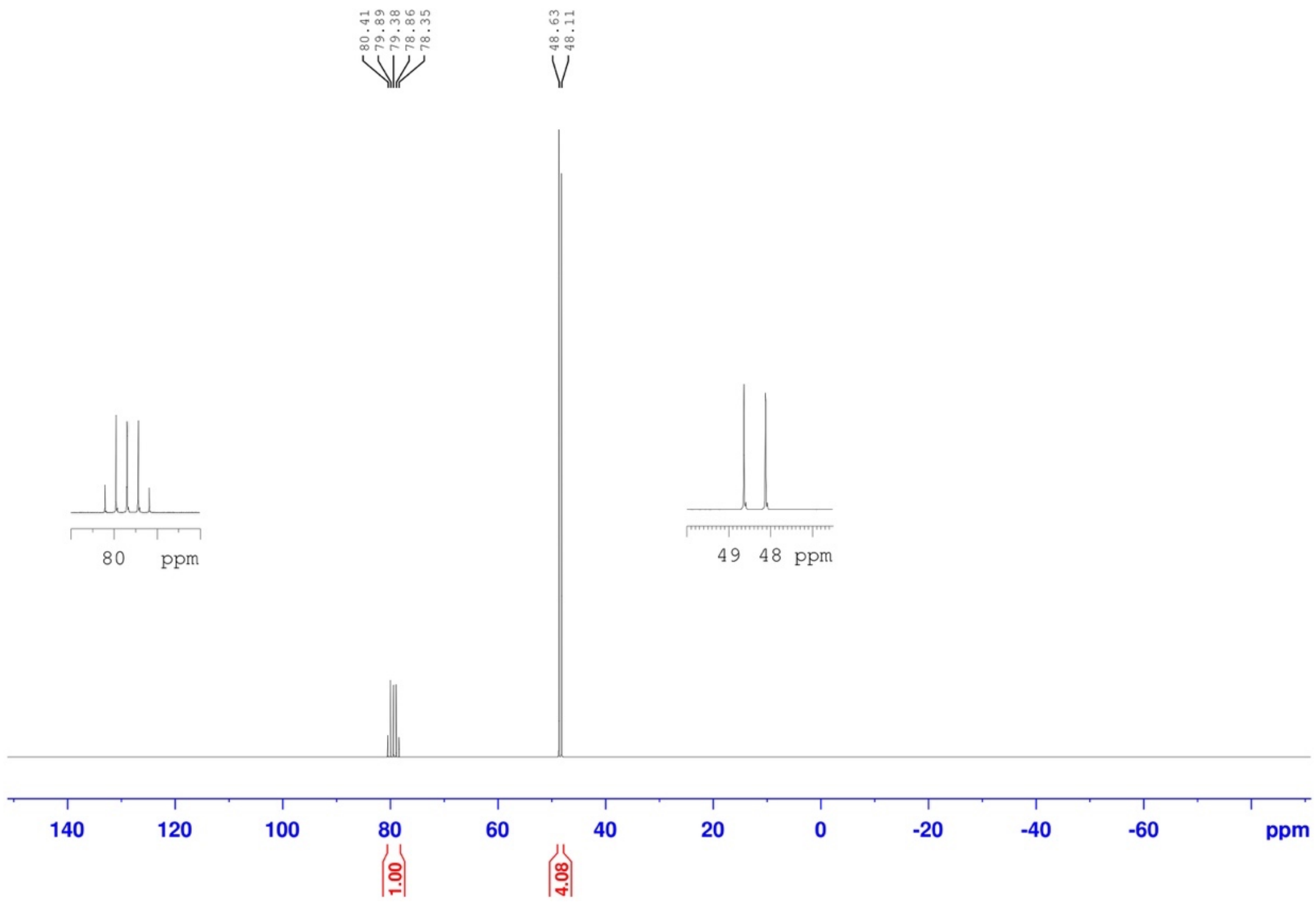

Figure S250. ^{19}F NMR spectrum of 3'-(pentafluoro-λ^6-sulfanyl)-[1,1'-bi(bicyclo[1.1.1]pentan)]-3-yl methacrylate (**55**) in $CDCl_3$.

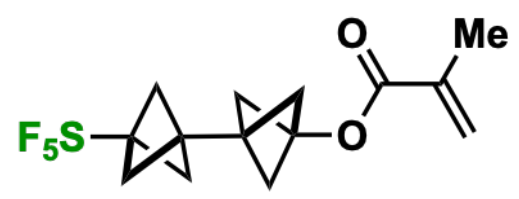

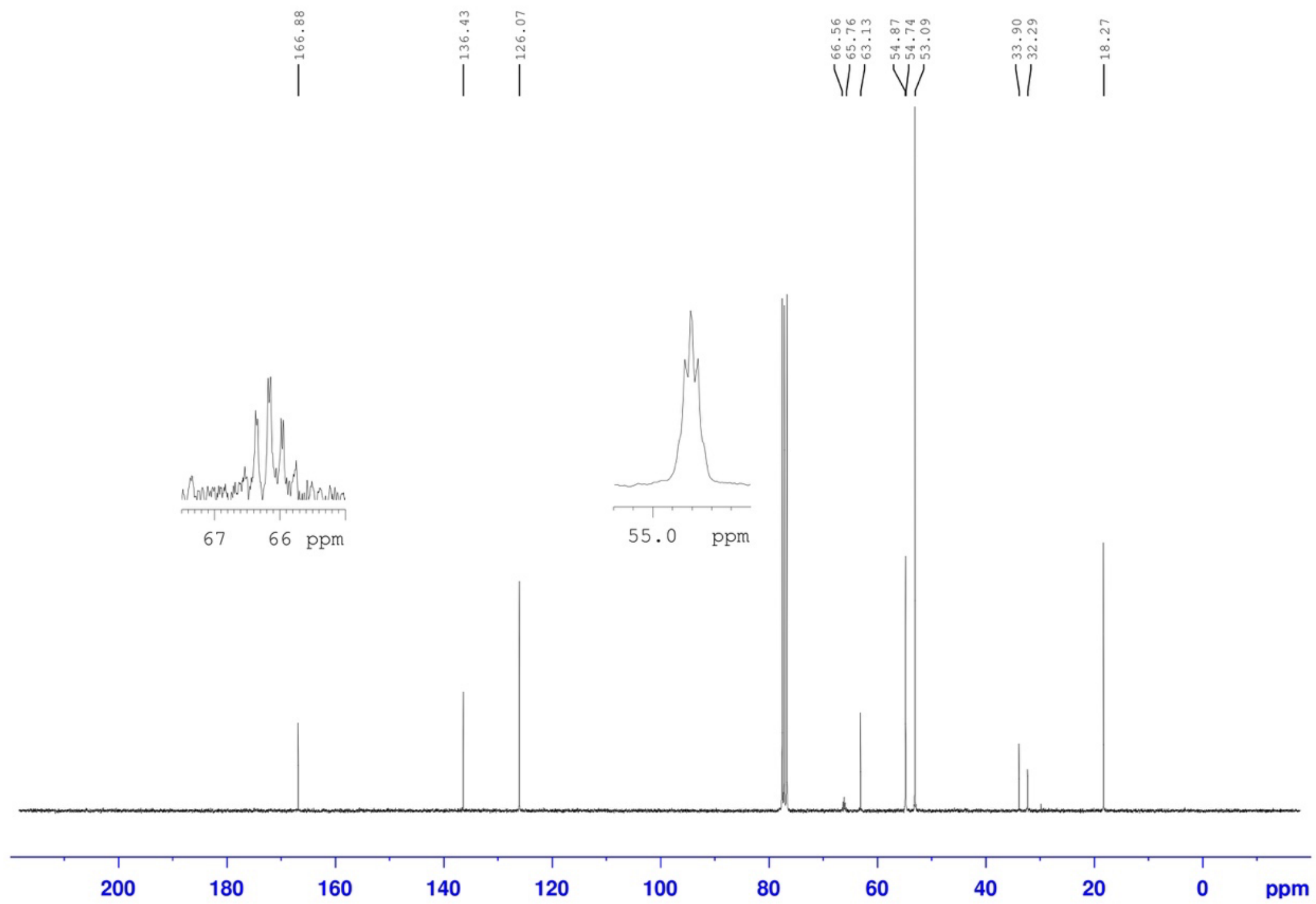

Figure S251. $^{13}C\{^{1}H\}$ NMR spectrum of 3'-(pentafluoro-λ^{6}-sulfanyl)-[1,1'-bi(bicyclo[1.1.1]pentan)]-3-yl methacrylate (**55**) in $CDCl_3$.

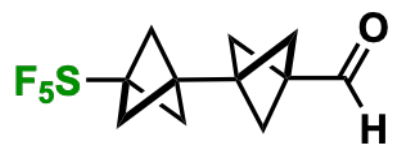

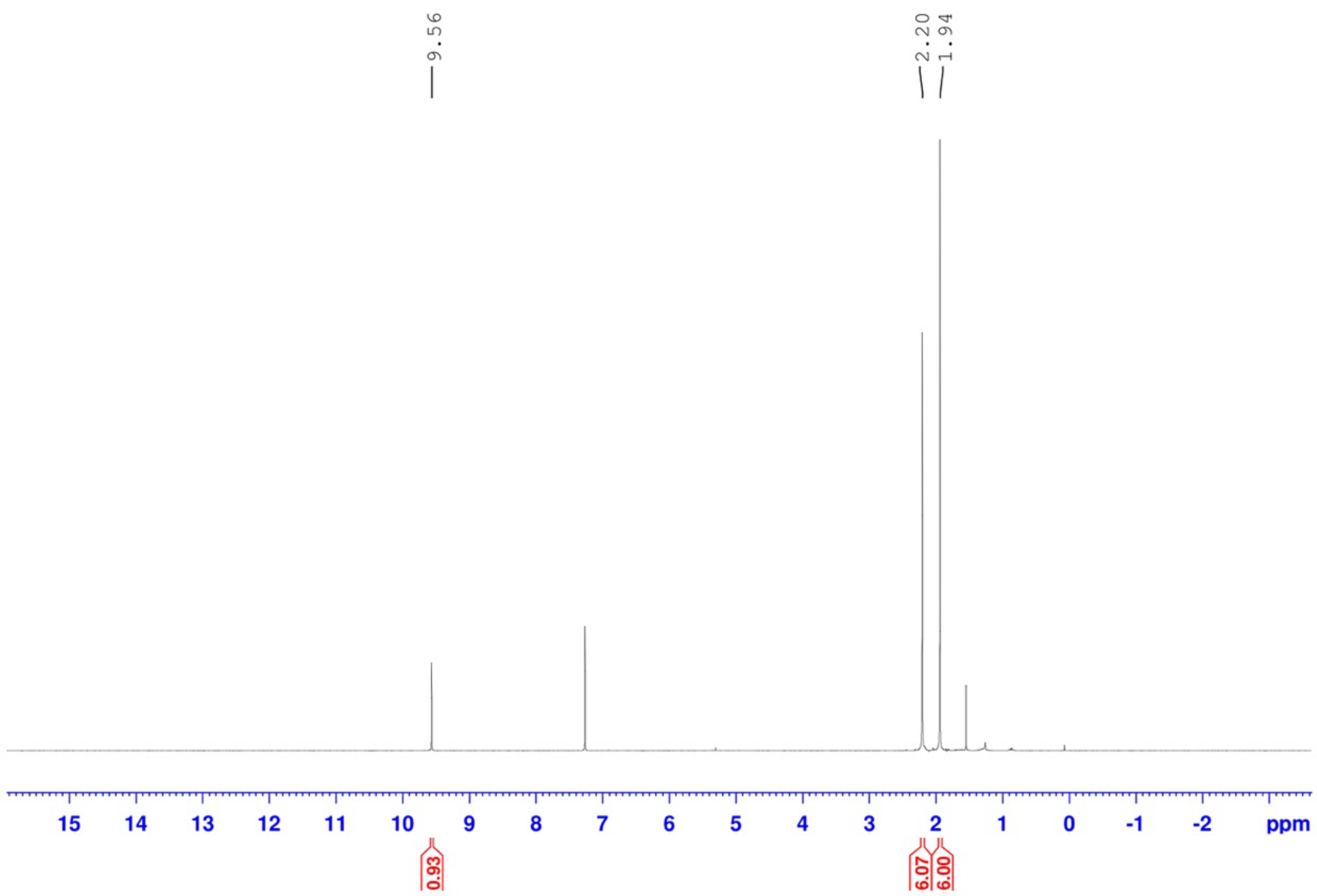

Figure S252.^{1}H NMR spectrum of 3'-(pentafluoro-λ[6]-sulfanyl)-[1,1'-bi(bicyclo[1.1.1]pentane)]-3-carbaldehyde (**56**) in $CDCl_3$.

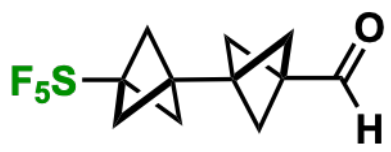

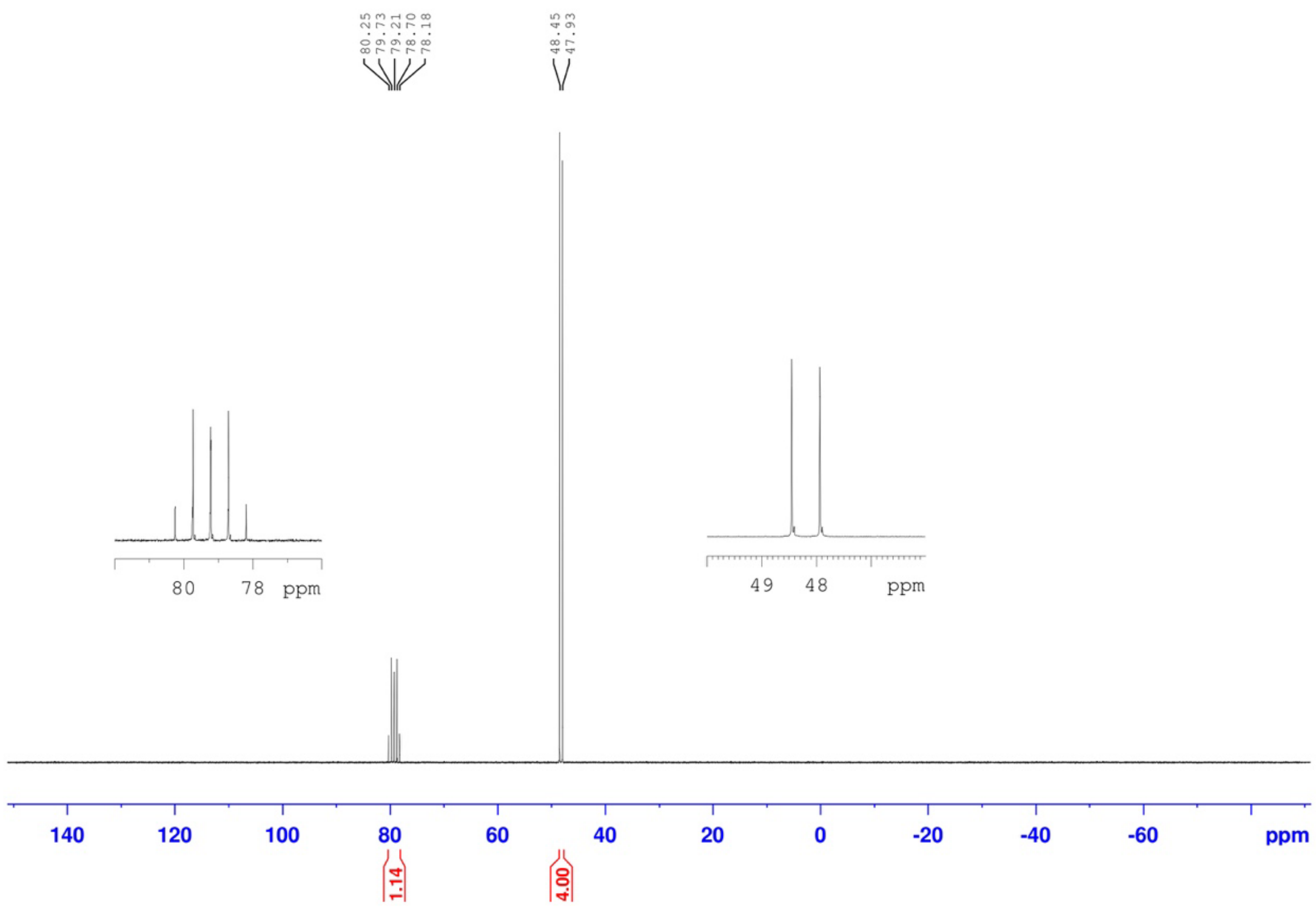

Figure S253. ^{19}F NMR spectrum of 3'-(pentafluoro-λ^6-sulfanyl)-[1,1'-bi(bicyclo[1.1.1]pentane)]-3-carbaldehyde (**56**) in $CDCl_3$.

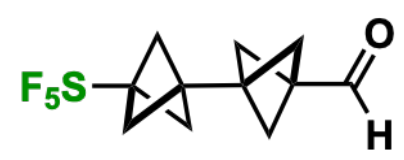

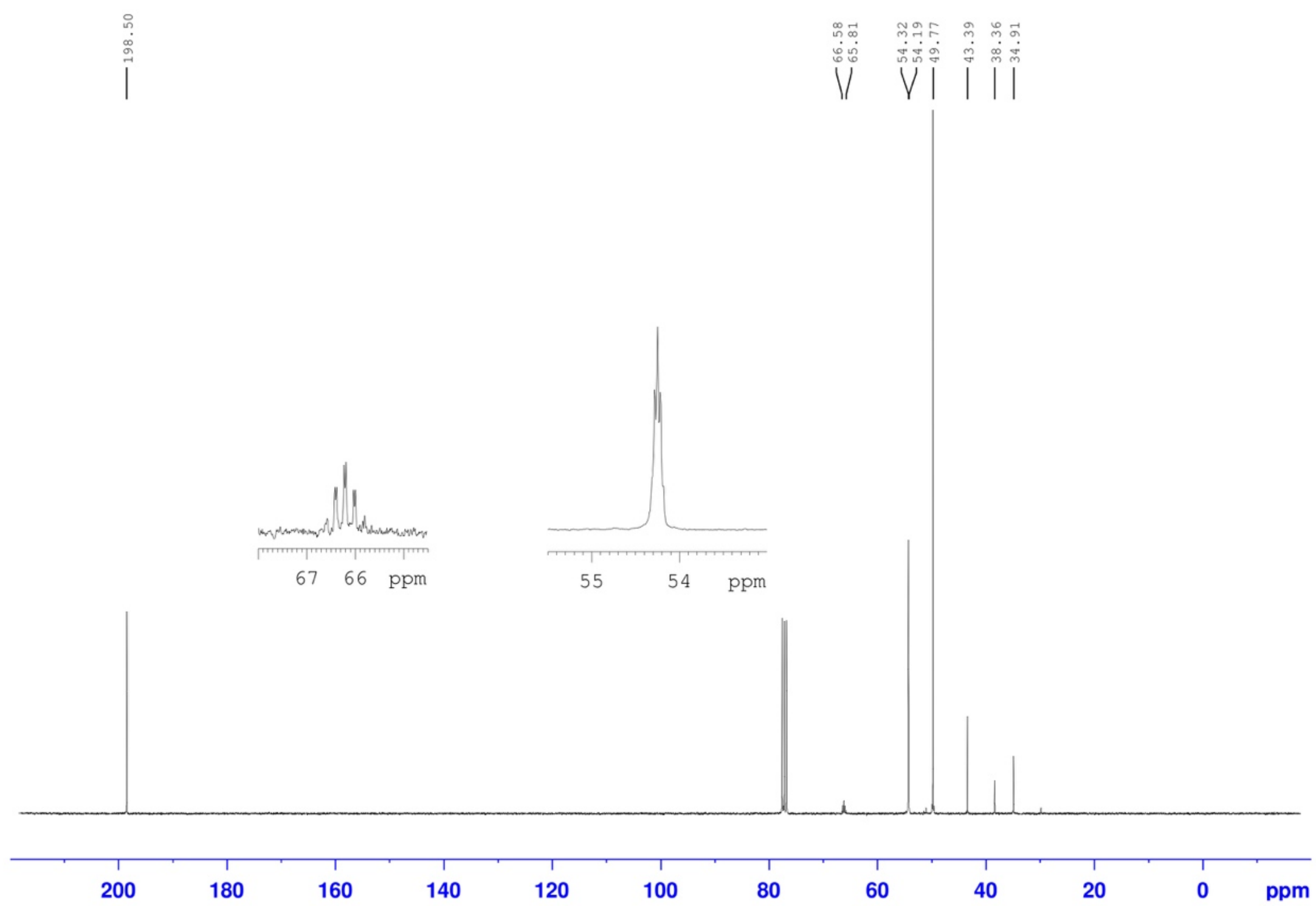

Figure S254. $^{13}C\{^1H\}$ NMR spectrum of 3'-(pentafluoro-λ^6-sulfanyl)-[1,1'-bi(bicyclo[1.1.1]pentane)]-3-carbaldehyde (**56**) in $CDCl_3$.

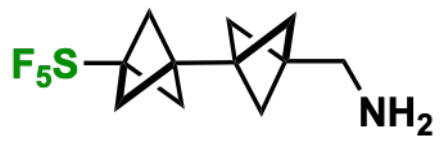

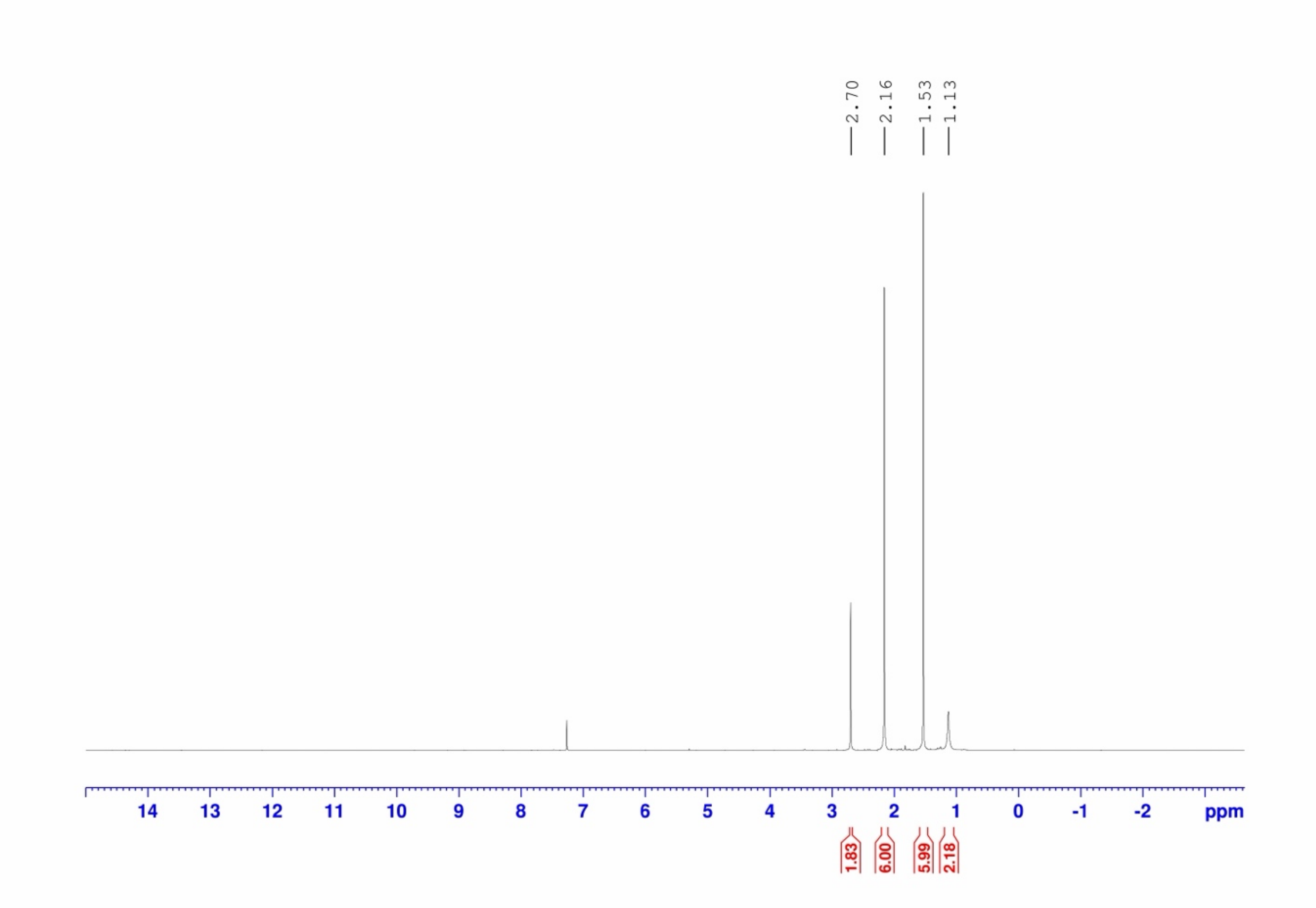

Figure S255. ^{1}H NMR of (3'-(pentafluoro- λ^6-sulfanyl)-[1,1'-bi(bicyclo[1.1.1]pentan)]-3-yl)methanamine (**57**) in $CDCl_3$.

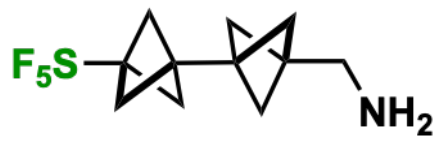

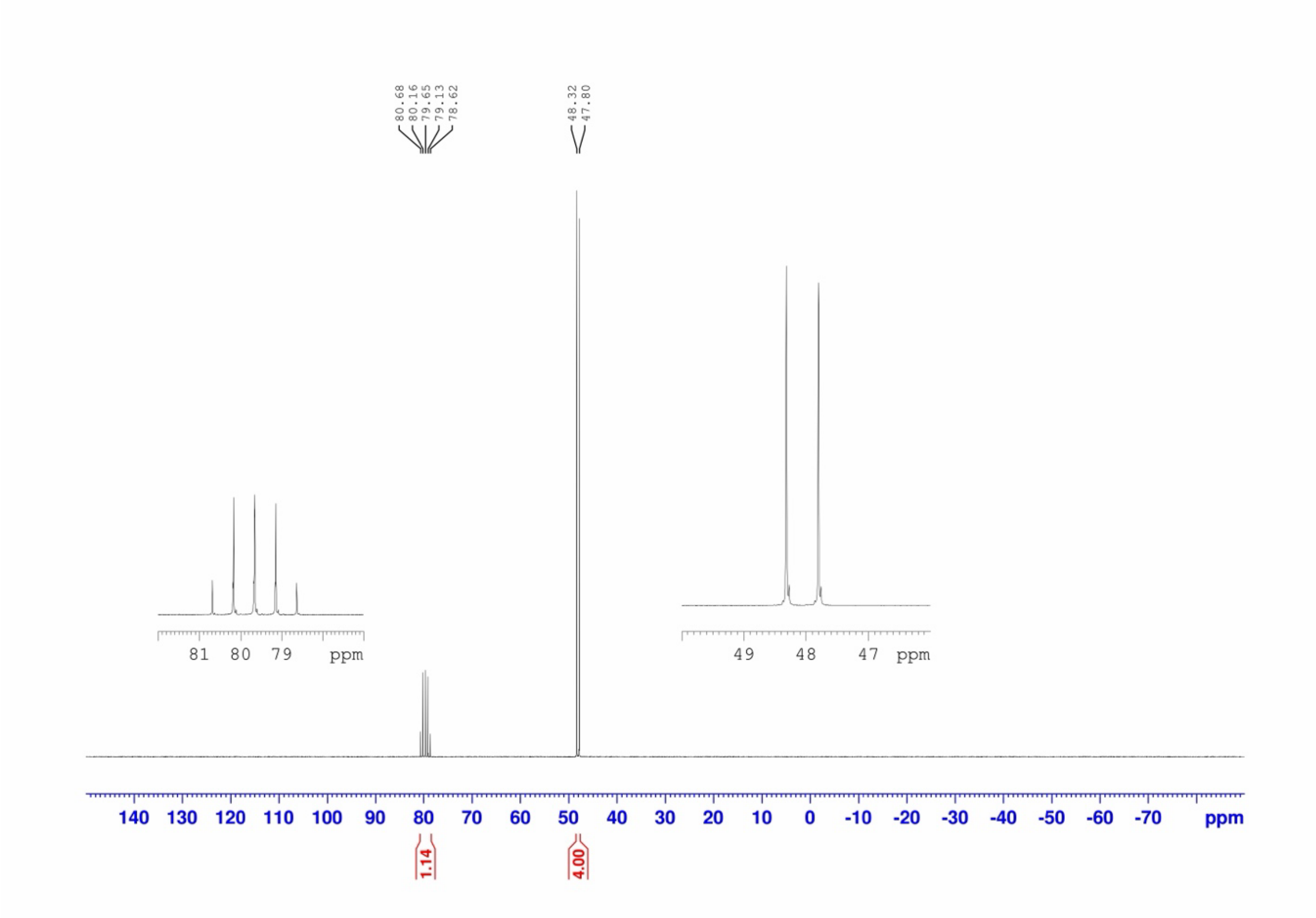

Figure S256. ^{19}F NMR of (3'-(pentafluoro- λ^6-sulfanyl)-[1,1'-bi(bicyclo[1.1.1]pentan)]-3-yl)methanamine (**57**) in $CDCl_3$.

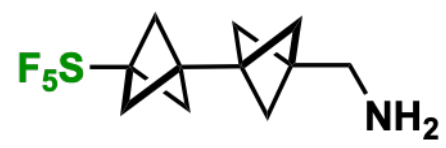

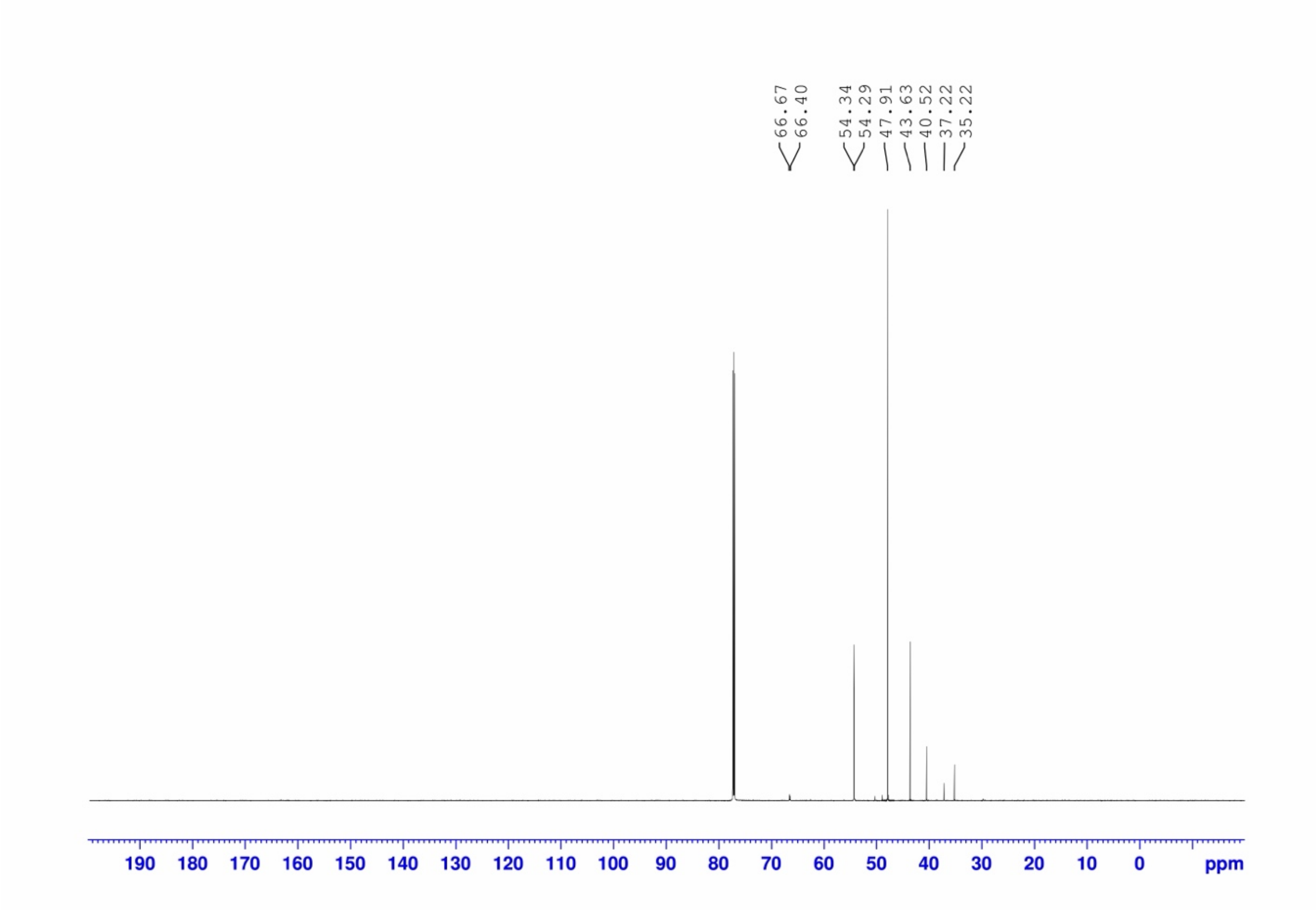

Figure S257. $^{13}C\{^{1}H\}$ NMR of (3'-(pentafluoro- λ^6-sulfanyl)-[1,1'-bi(bicyclo[1.1.1]pentan)]-3-yl)methanamine (**57**) in $CDCl_3$.

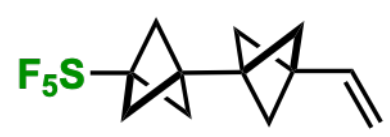

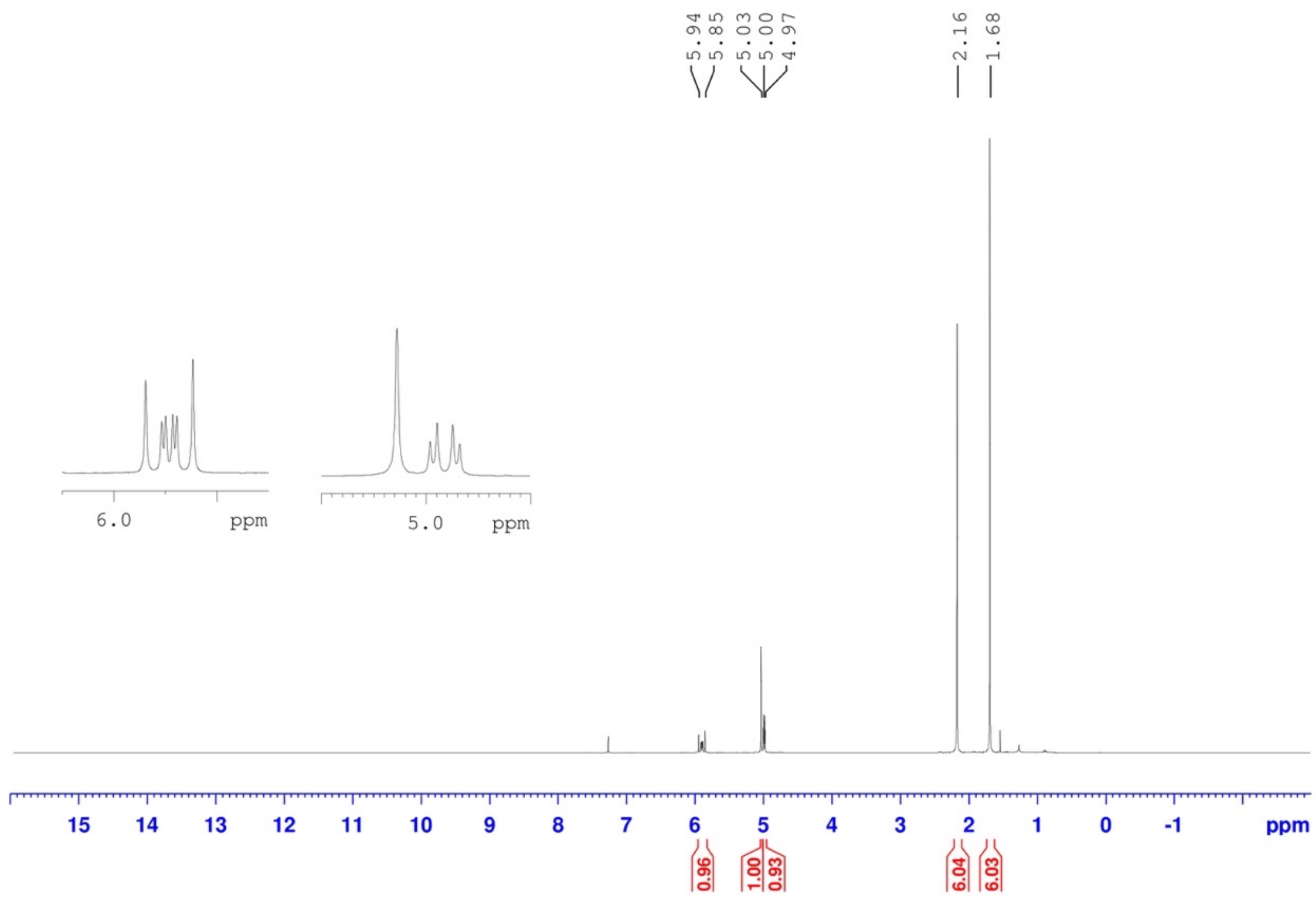

Figure S258.^{1}H NMR spectrum of pentafluoro(3'-vinyl-[1,1'-bi(bicyclo[1.1.1]pentan)]-3-yl)-λ[6]-sulfane (**58**) in $CDCl_3$.

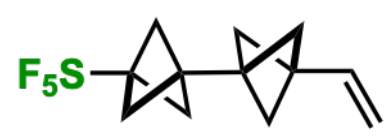

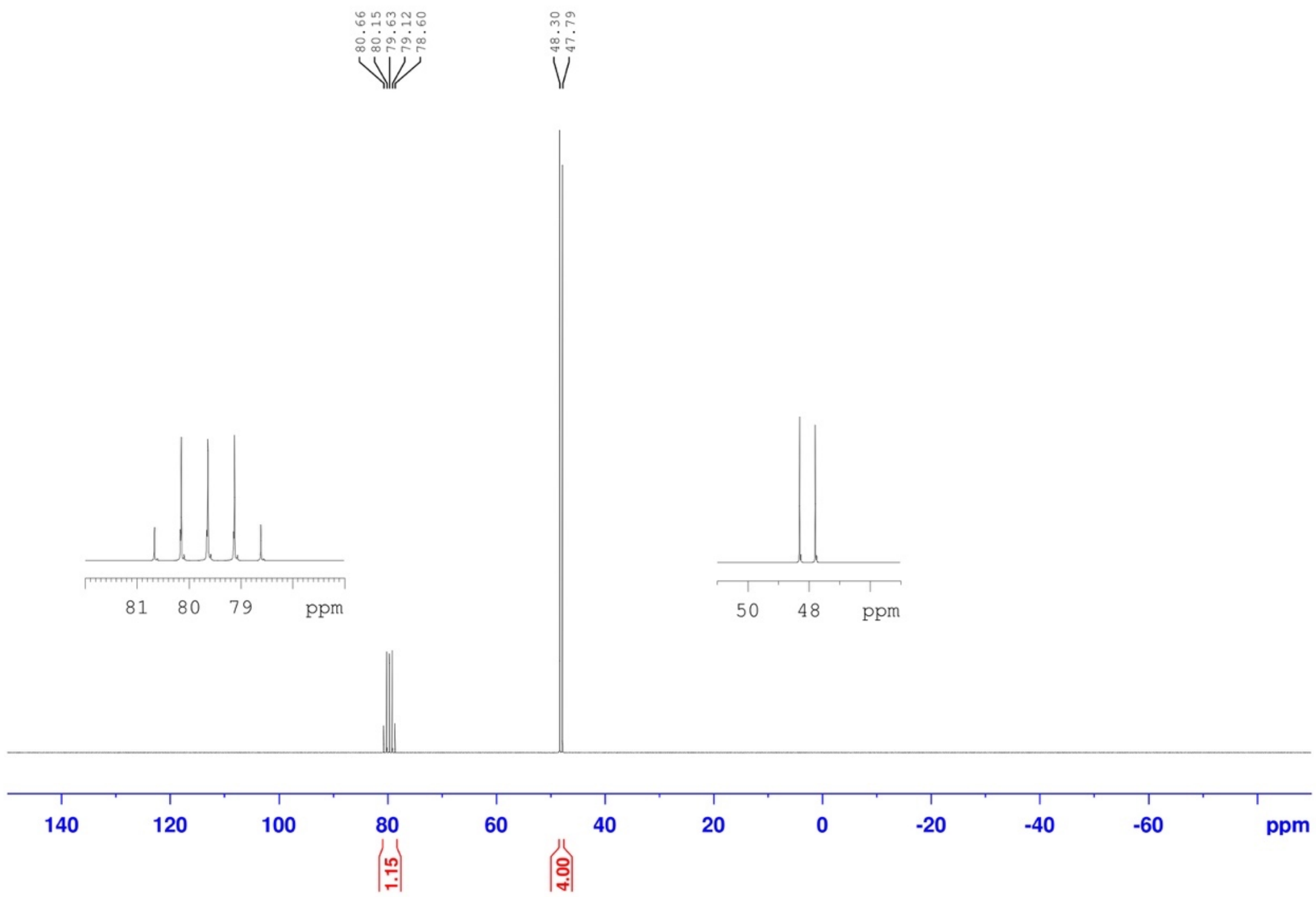

Figure S259.^{19}F NMR spectrum of pentafluoro(3'-vinyl-[1,1'-bi(bicyclo[1.1.1]pentan)]-3-yl)-λ[6]-sulfane (**58**) in $CDCl_3$.

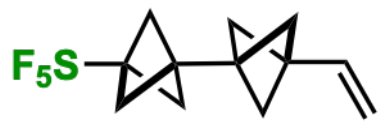

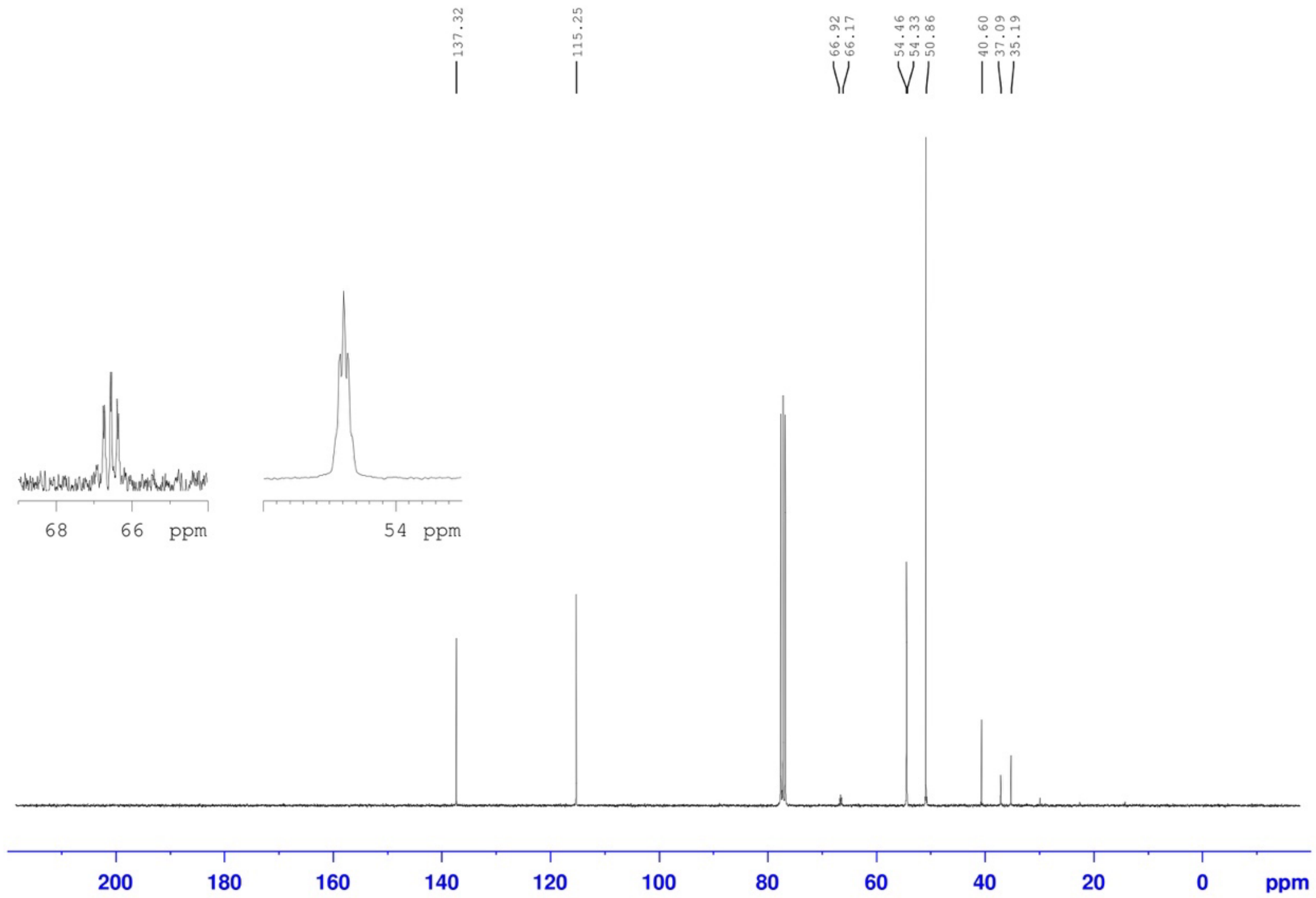

Figure S260. $^{13}C\{^1H\}$ NMR spectrum of pentafluoro(3'-vinyl-[1,1'-bi(bicyclo[1.1.1]pentan)]-3-yl)-λ^6-sulfane (**58**) in $CDCl_3$.

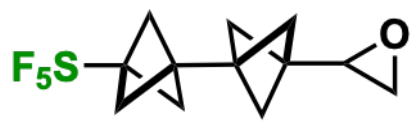

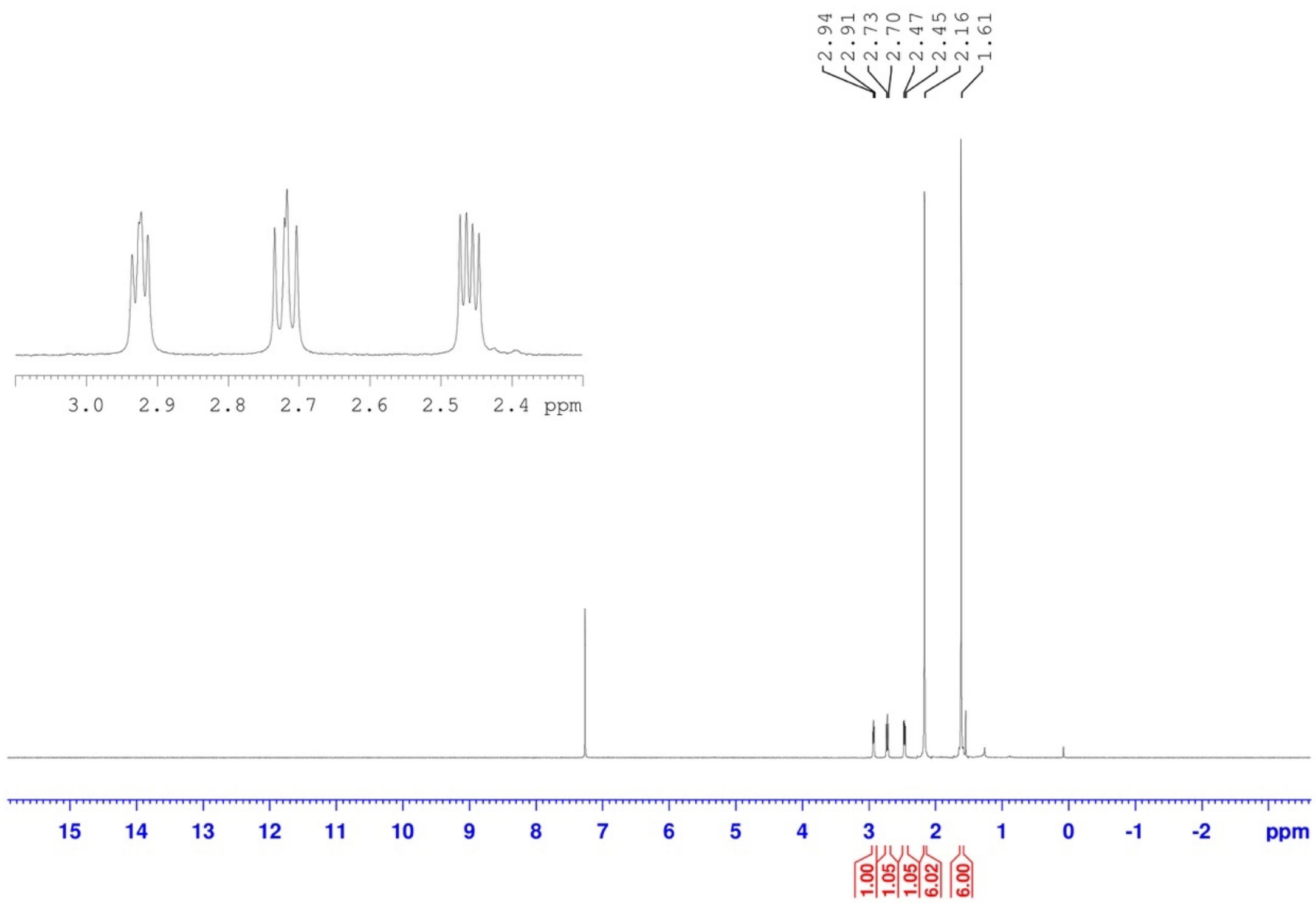

Figure S261. ^1H NMR spectrum of 2-(3'-(pentafluoro-λ^6-sulfanyl)-[1,1'-bi(bicyclo[1.1.1]pentan)]-3-yl)oxirane (**59**) in $CDCl_3$.

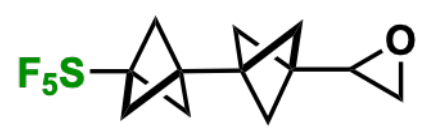

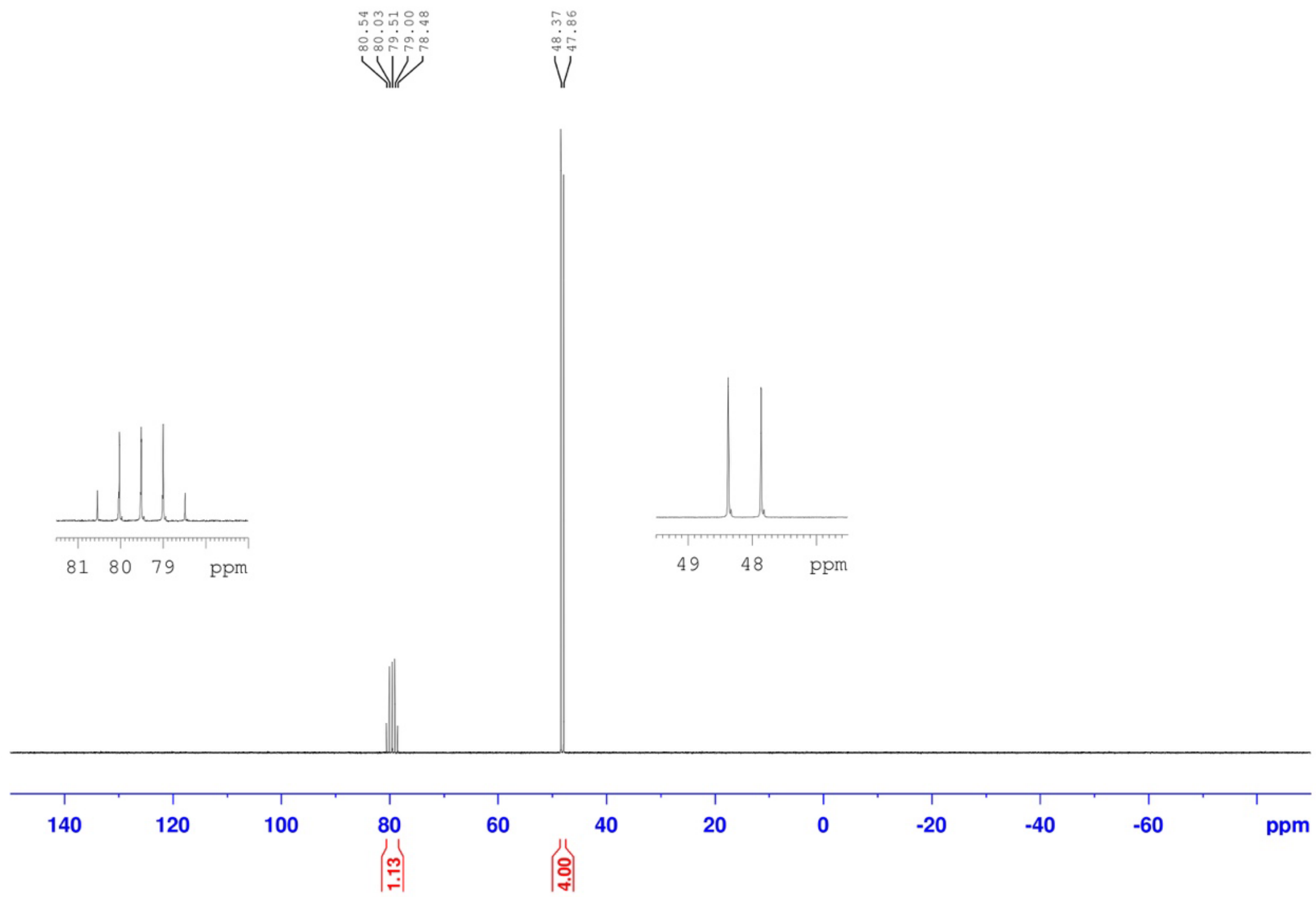

Figure S262. ^{19}F NMR spectrum of 2-(3'-(pentafluoro-λ^6-sulfanyl)-[1,1'-bi(bicyclo[1.1.1]pentan)]-3-yl)oxirane (**59**) in $CDCl_3$.

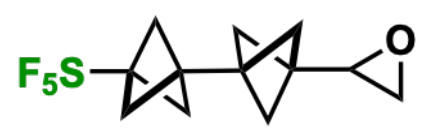

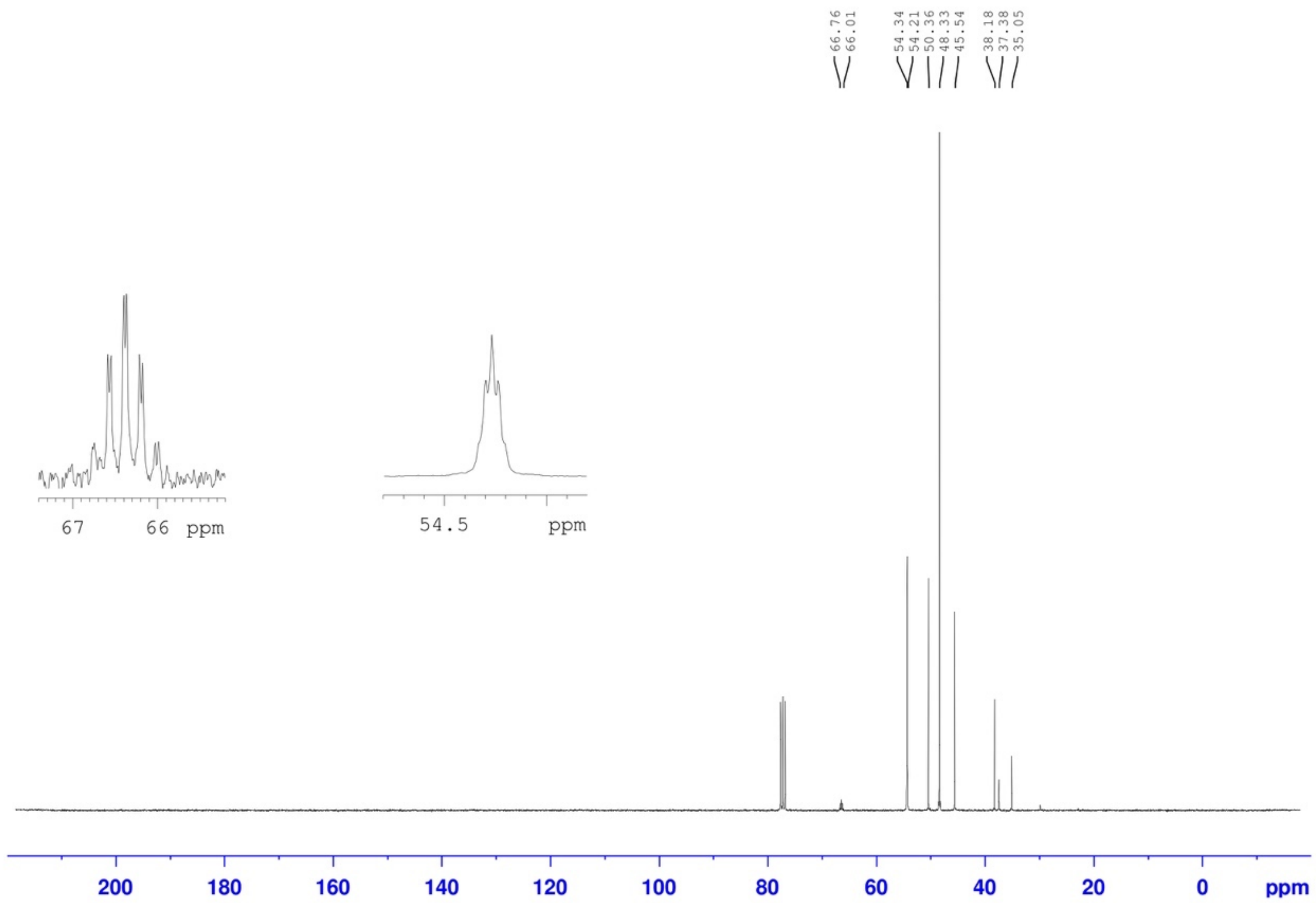

Figure S263. $^{13}C\{^1H\}$ NMR spectrum of 2-(3'-(pentafluoro-λ^6-sulfanyl)-[1,1'-bi(bicyclo[1.1.1]pentan)]-3-yl)oxirane (**59**) in $CDCl_3$.

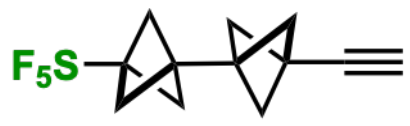

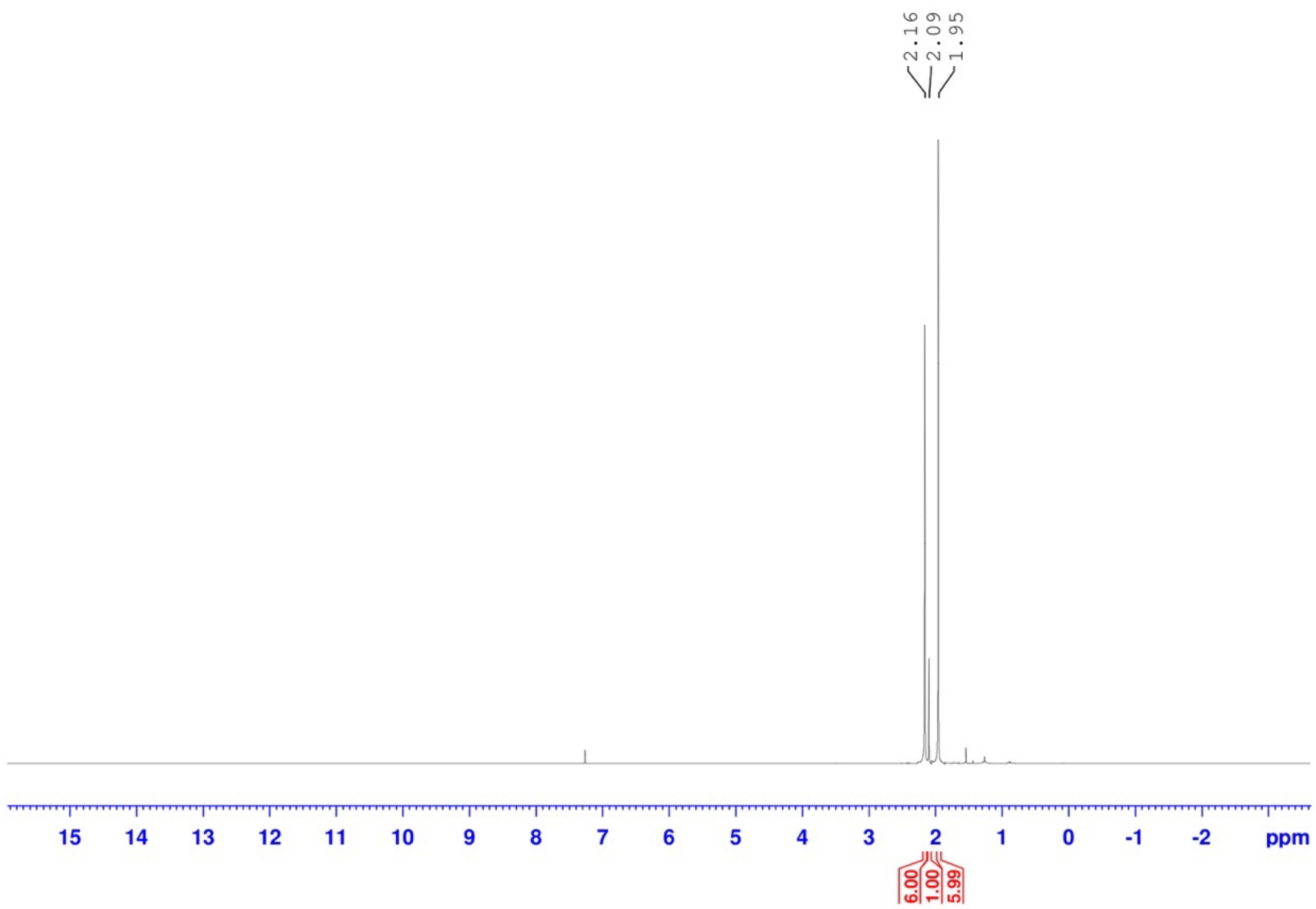

Figure S264. ^{1}H NMR spectrum of (3'-ethynyl-[1,1'-bi(bicyclo[1.1.1]pentan)]-3-yl)pentafluoro-λ^6-sulfane (**60**) in $CDCl_3$.

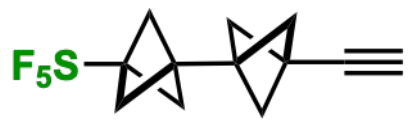

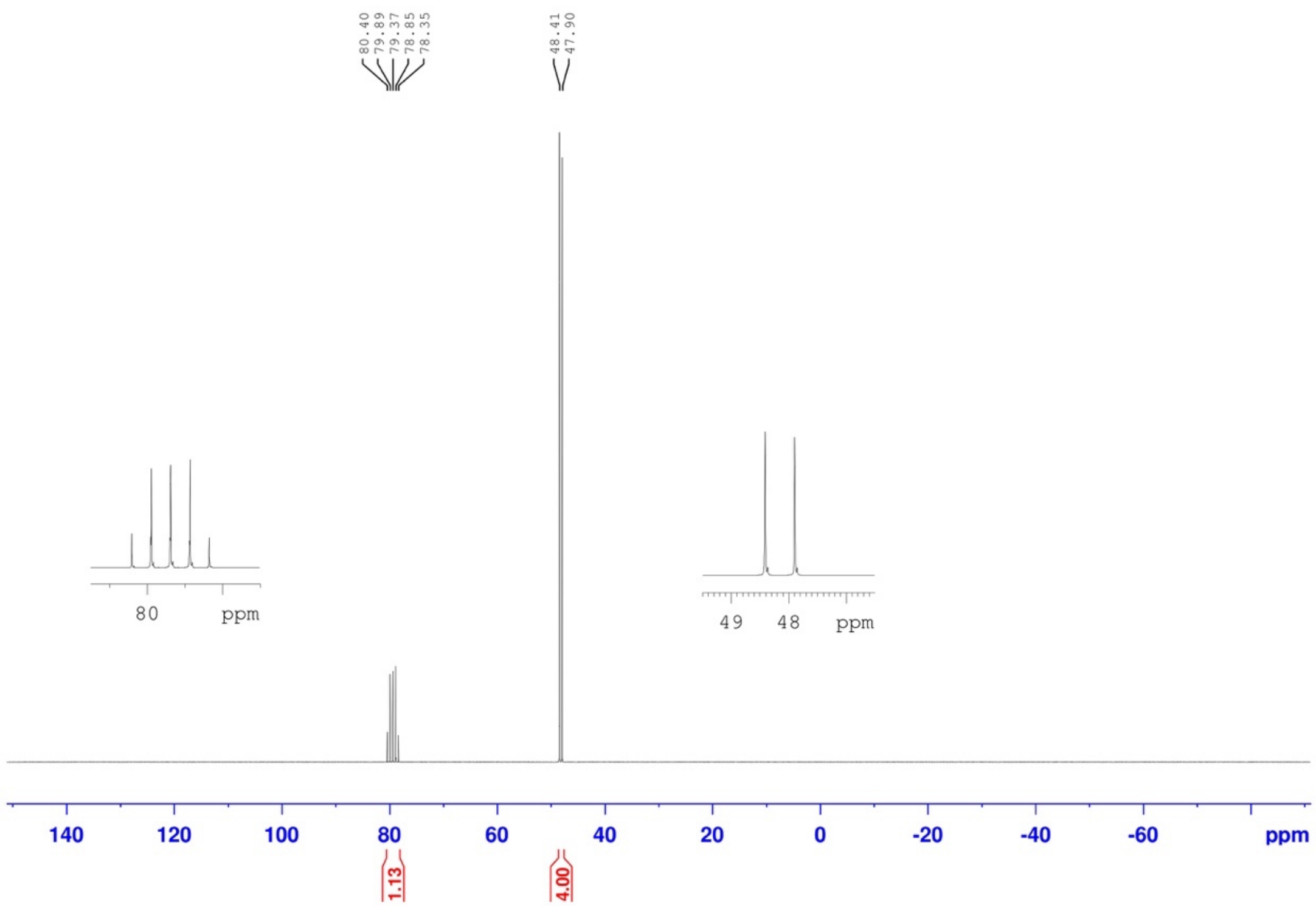

Figure S265. ^{19}F NMR spectrum of (3'-ethynyl-[1,1'-bi(bicyclo[1.1.1]pentan)]-3-yl)pentafluoro-λ^6-sulfane (**60**) in $CDCl_3$.

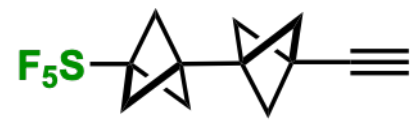

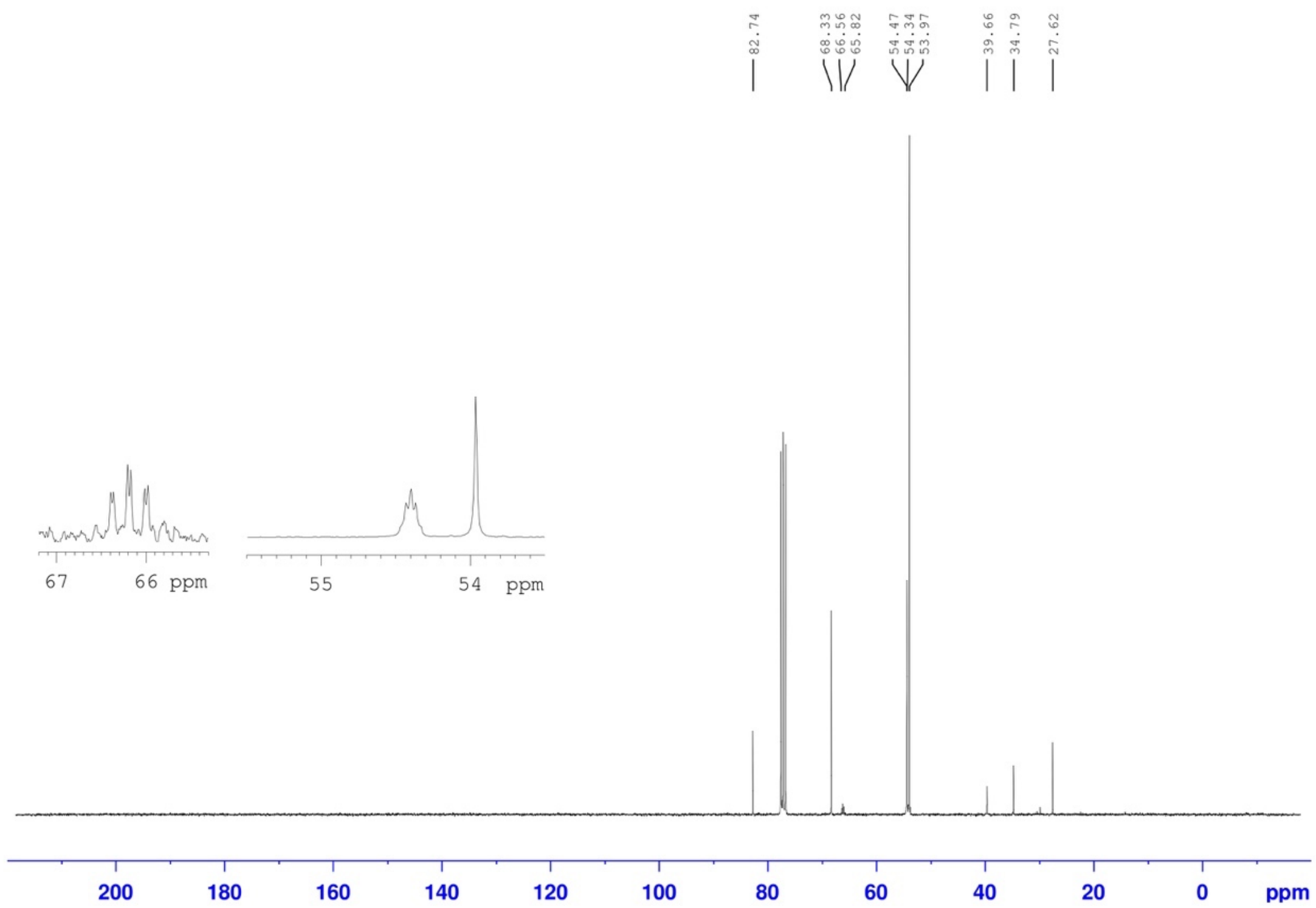

Figure S266. $^{13}C\{^1H\}$ NMR spectrum of (3'-ethynyl-[1,1'-bi(bicyclo[1.1.1]pentan)]-3-yl)pentafluoro-λ^6-sulfane (**60**) in $CDCl_3$.

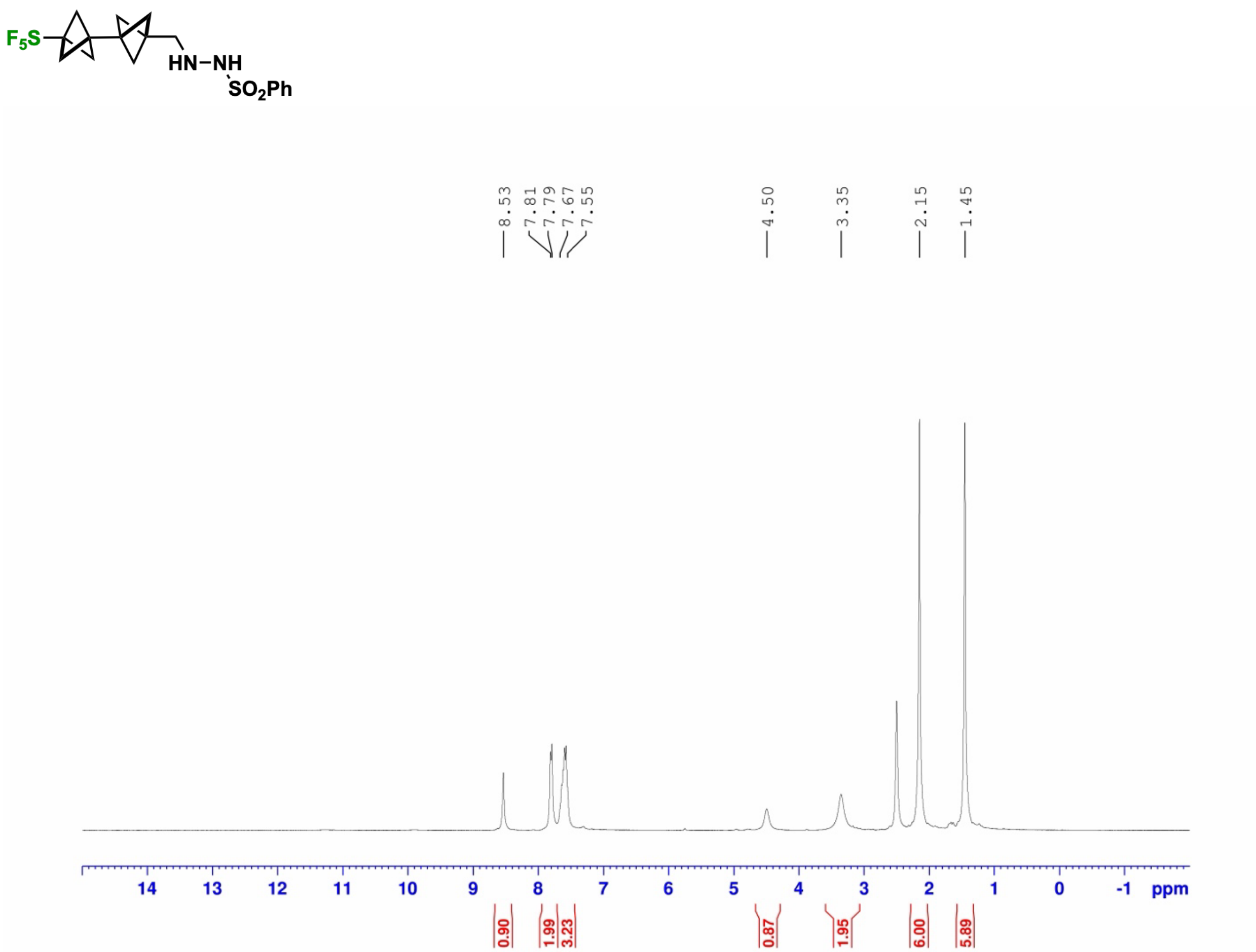

Figure S267. ^{1}H NMR spectrum of *N'*-((3'-(pentafluoro- λ^6-sulfanyl)-[1,1'-bi(bicyclo[1.1.1]pentan)]-3-yl)methyl)benzenesulfonohydrazide (**61**) in $CDCl_3$.

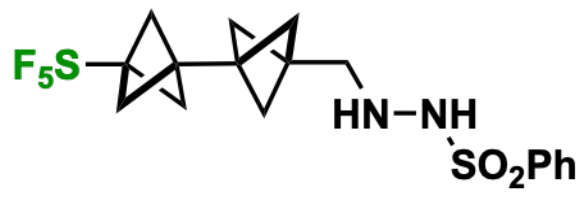

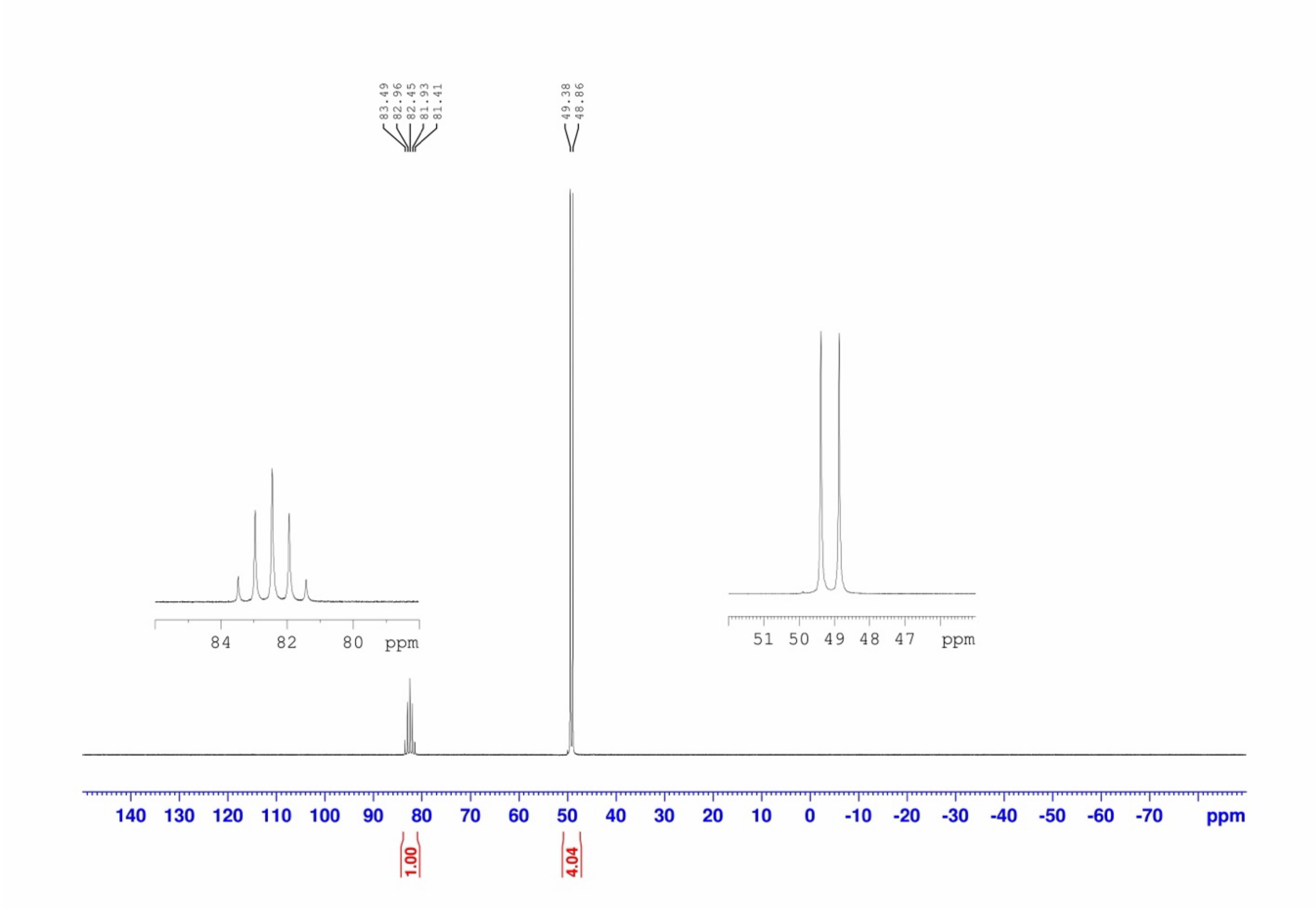

Figure S268. ^{19}F NMR of *N'*-((3'-(pentafluoro- λ^6-sulfanyl)-[1,1'-bi(bicyclo[1.1.1]pentan)]-3-yl)methyl)benzenesulfonohydrazide (**61**) in $CDCl_3$.

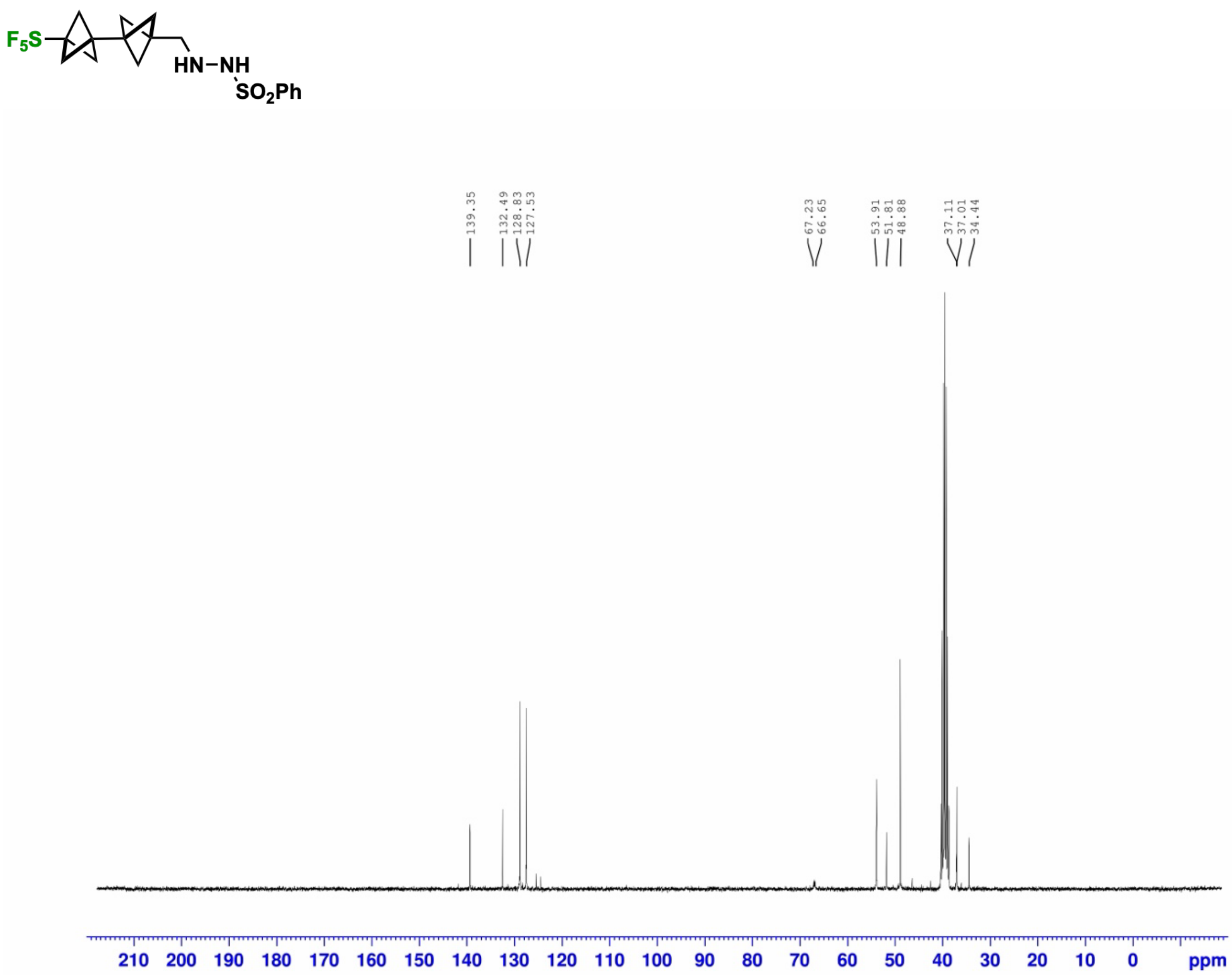

Figure S269. $^{13}C\{^{1}H\}$ NMR spectrum of *N'*-((3'-(pentafluoro-λ^6-sulfanyl)-[1,1'-bi(bicyclo[1.1.1]pentan)]-3-yl)methyl)benzenesulfonohydrazide (**61**) in $CDCl_3$.

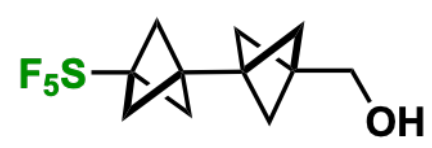

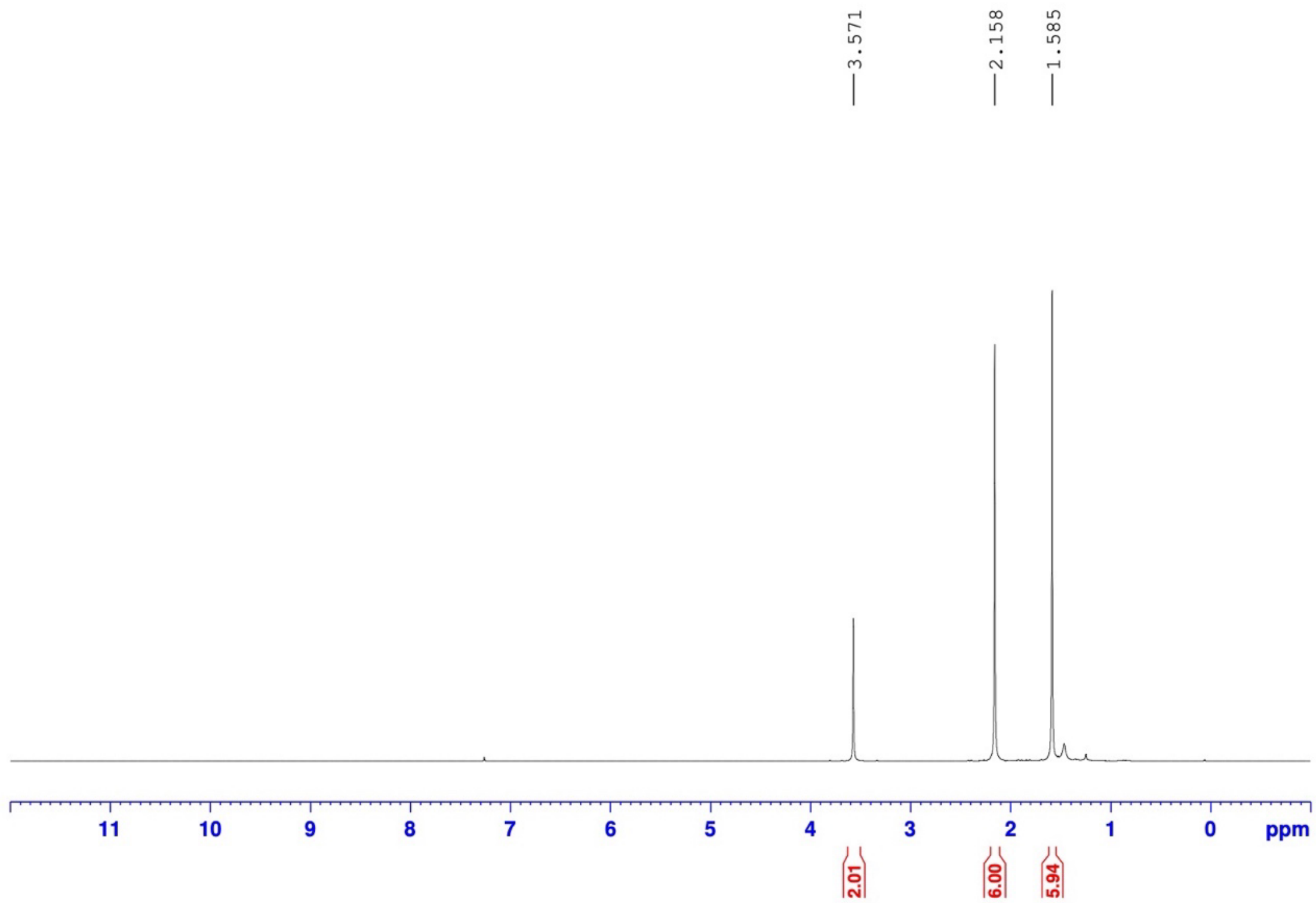

Figure S270. ¹H NMR spectrum of (3'-(pentafluoro- λ ^{6}-sulfanyl)-[1,1'-bi(bicyclo[1.1.1]pentan)]-3-yl)methanol (**62**) in $CDCl_3$.

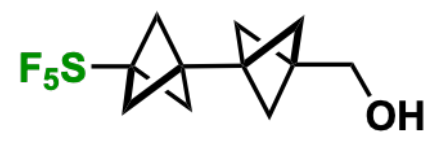

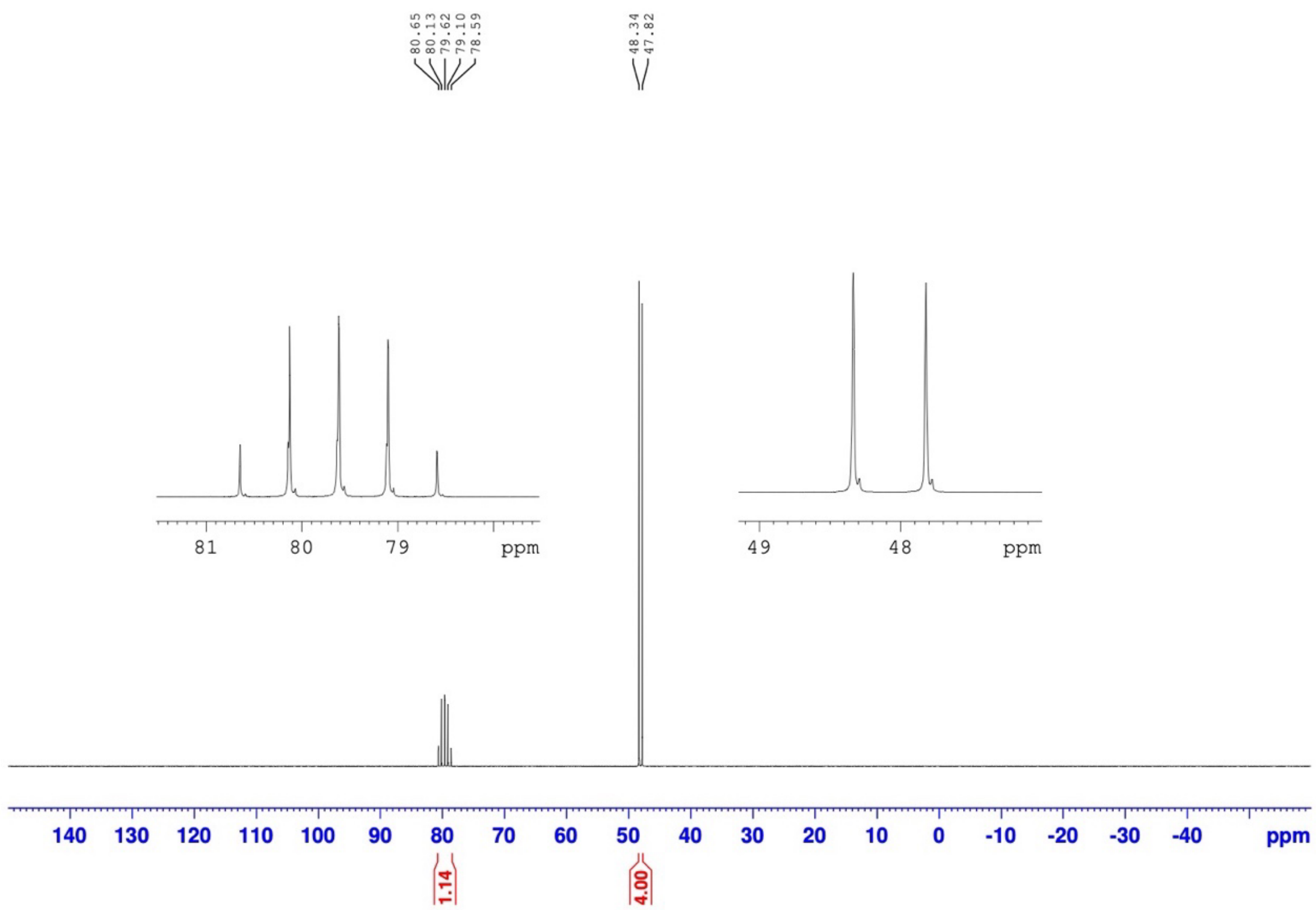

Figure S271. ^{19}F NMR spectrum of (3'-(pentafluoro- λ ^{6}-sulfanyl)-[1,1'-bi(bicyclo[1.1.1]pentan)]-3-yl)methanol (**62**) in $CDCl_3$.

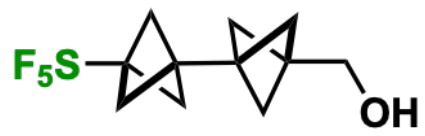

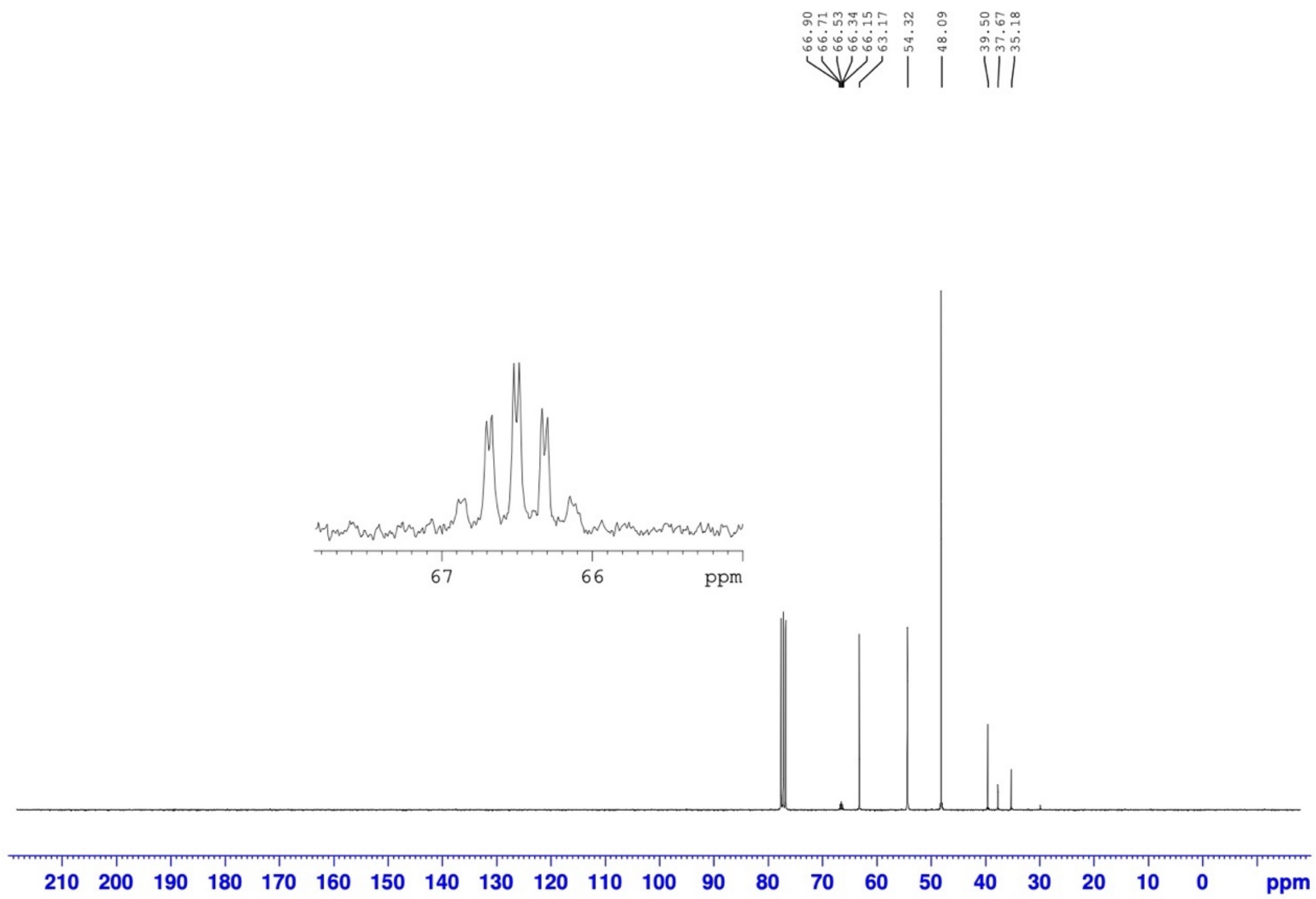

Figure S272. $^{13}C\{^1H\}$ NMR spectrum of (3'-(pentafluoro- λ ^{6}-sulfanyl)-[1,1'-bi(bicyclo[1.1.1]pentan)]-3-yl)methanol (**62**) in $CDCl_3$.

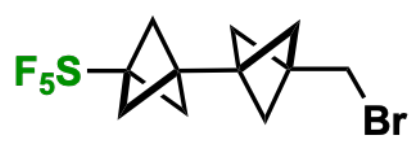

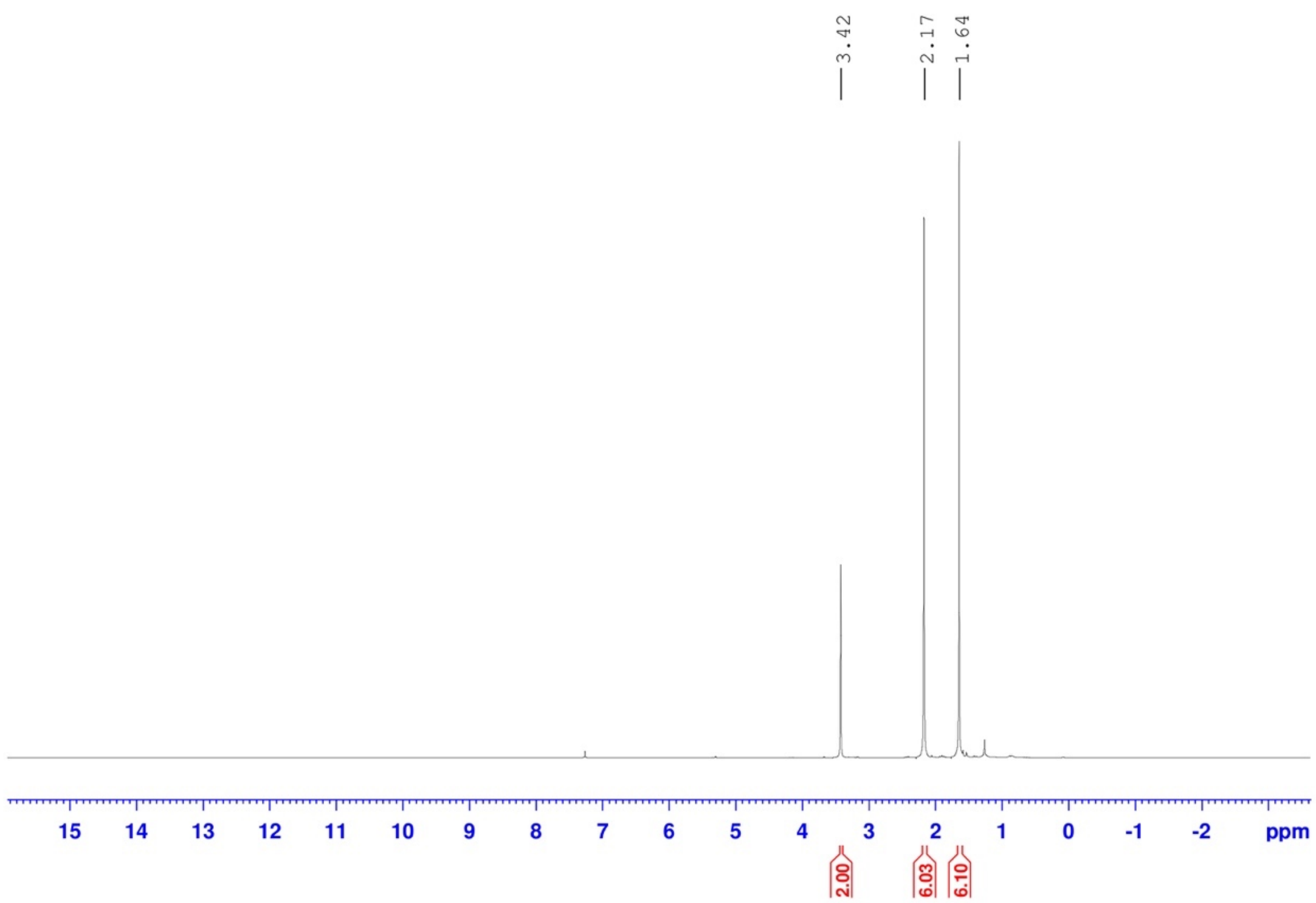

Figure S273. ^{1}H NMR spectrum of (3'-(bromomethyl)-[1,1'-bi(bicyclo[1.1.1]pentan)]-3-yl)pentafluoro-λ^6-sulfane (**63**) in $CDCl_3$.

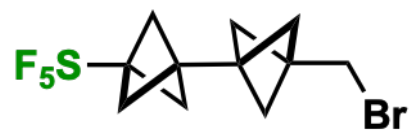

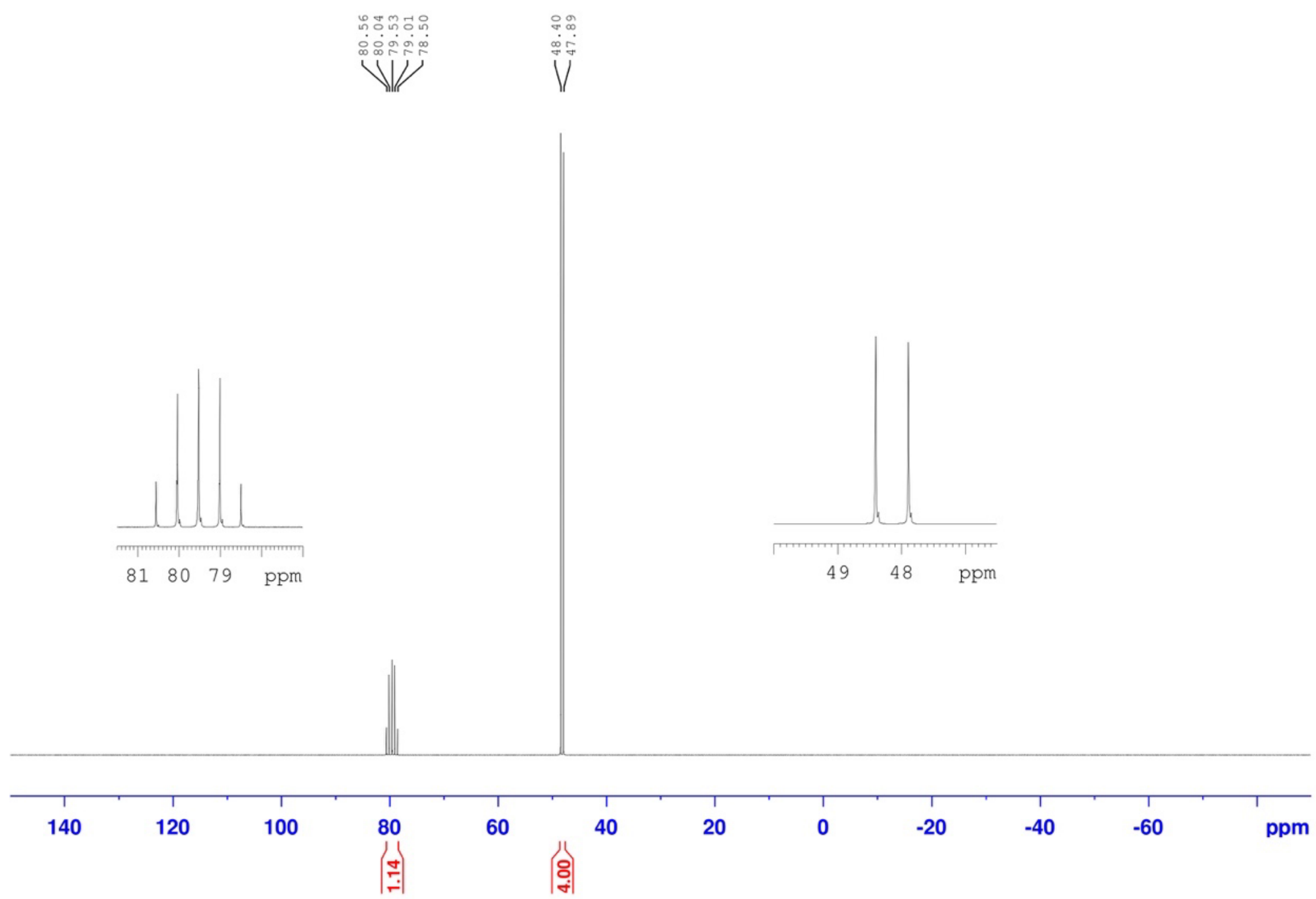

Figure S274. ^{19}F NMR spectrum of (3'-(bromomethyl)-[1,1'-bi(bicyclo[1.1.1]pentan)]-3-yl)pentafluoro-λ^6-sulfane (**63**) in $CDCl_3$.

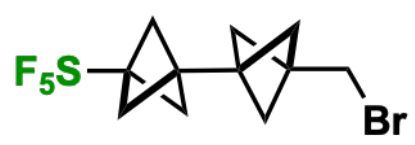

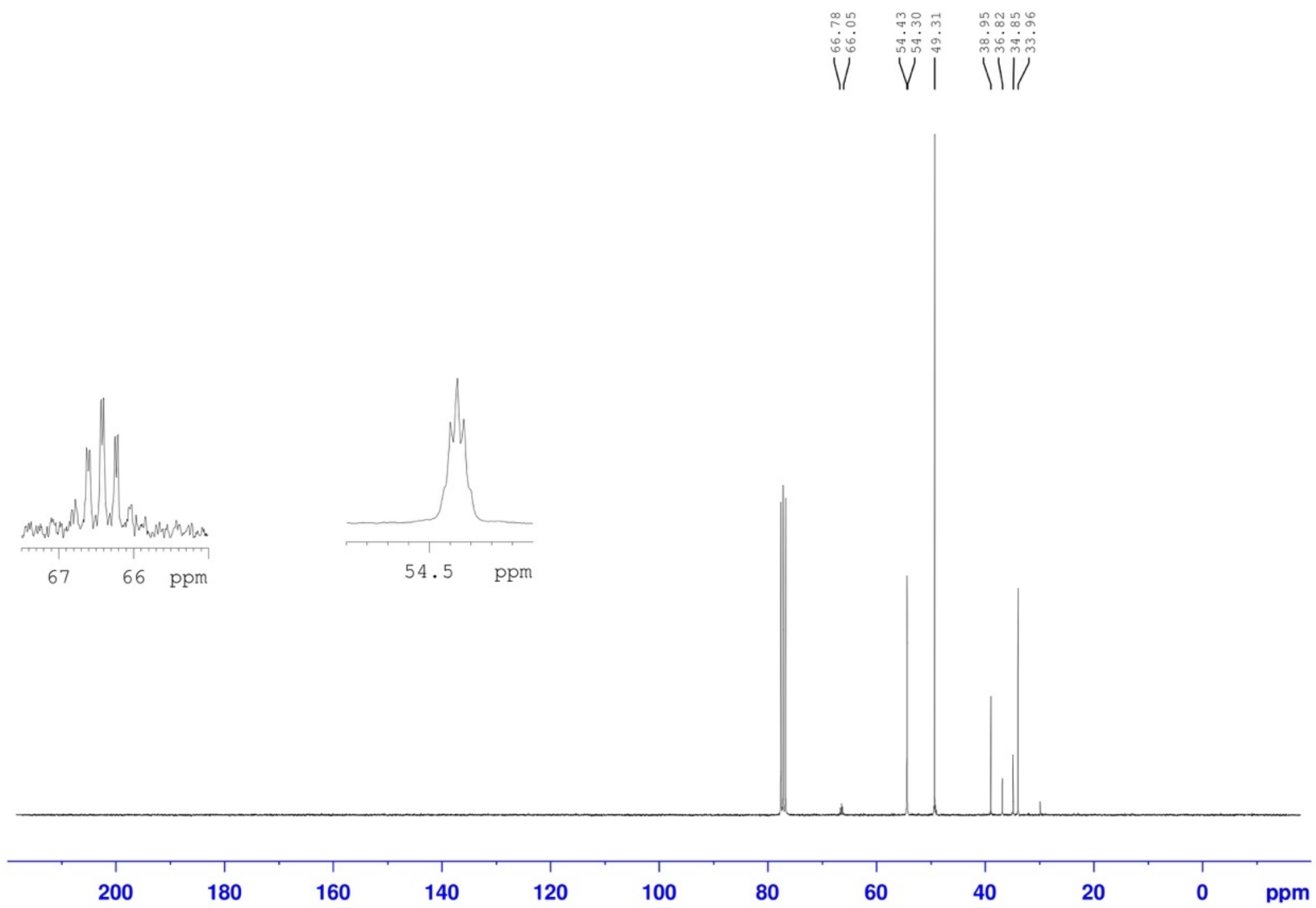

Figure S275. $^{13}C\{^{1}H\}$ NMR spectrum of (3'-(bromomethyl)-[1,1'-bi(bicyclo[1.1.1]pentan)]-3-yl)pentafluoro-λ^6-sulfane (**63**) in $CDCl_3$.

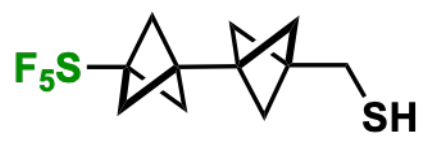

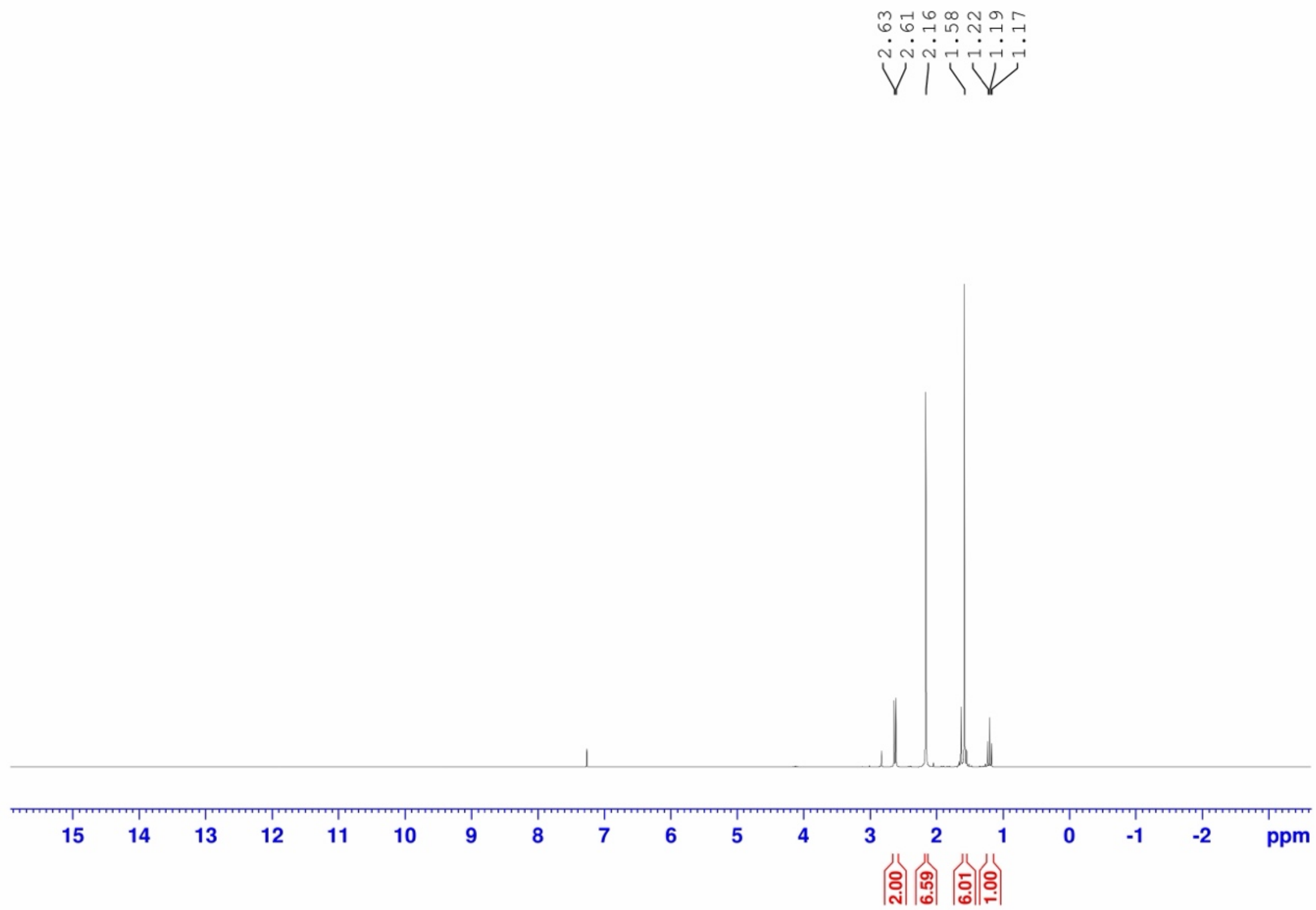

Figure S276. ^{1}H NMR spectrum of (3'-(pentafluoro-λ^6-sulfanyl)-[1,1'-bi(bicyclo[1.1.1]pentan)]-3-yl)methanethiol (**64**) in $CDCl_3$.

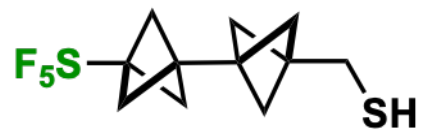

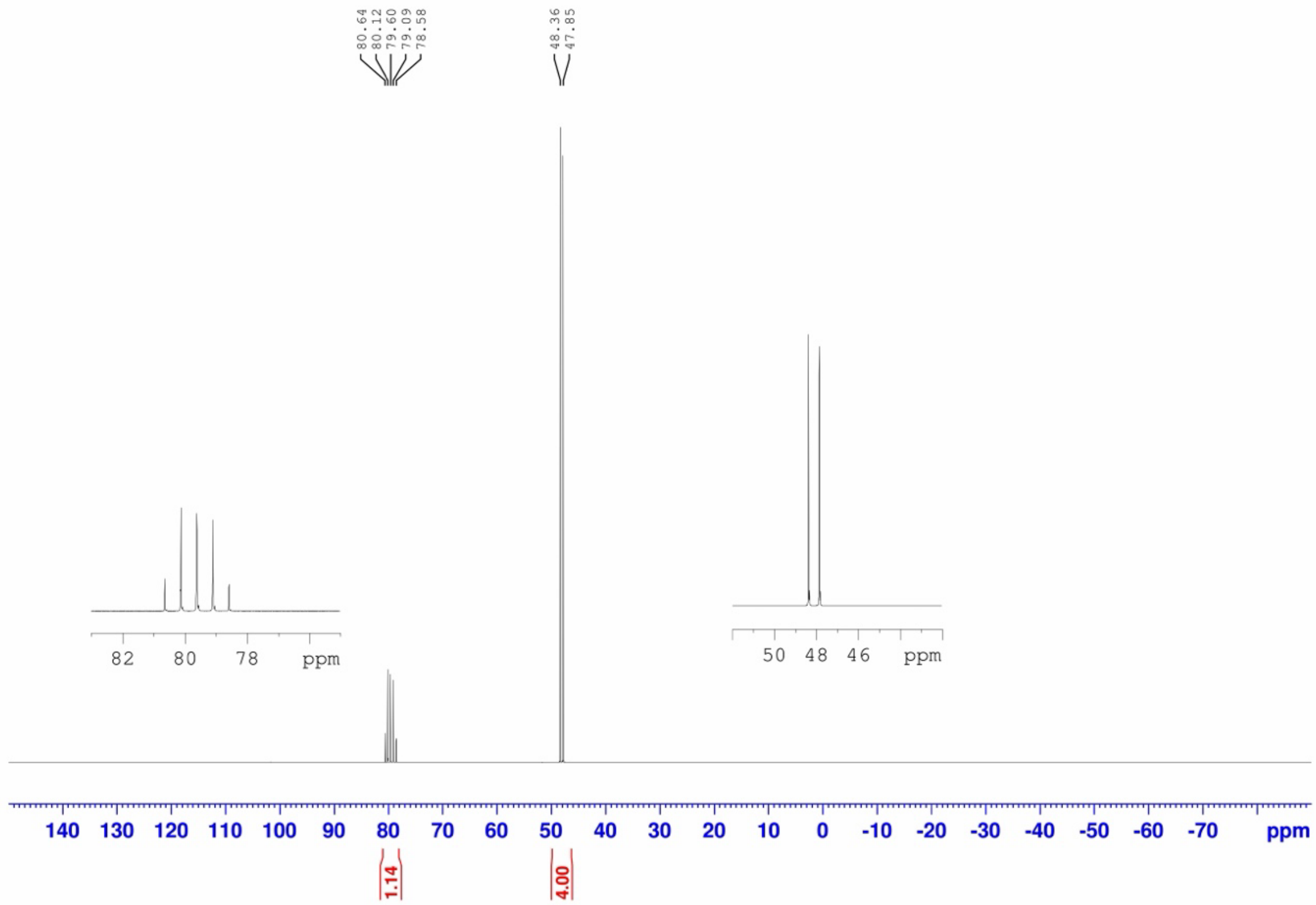

Figure S277. ^{19}F NMR spectrum of (3'-(pentafluoro-λ^6-sulfanyl)-[1,1'-bi(bicyclo[1.1.1]pentan)]-3-yl)methanethiol (**64**) in $CDCl_3$.

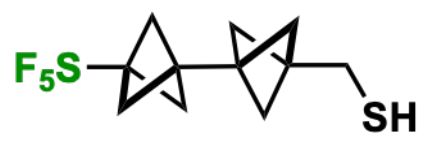

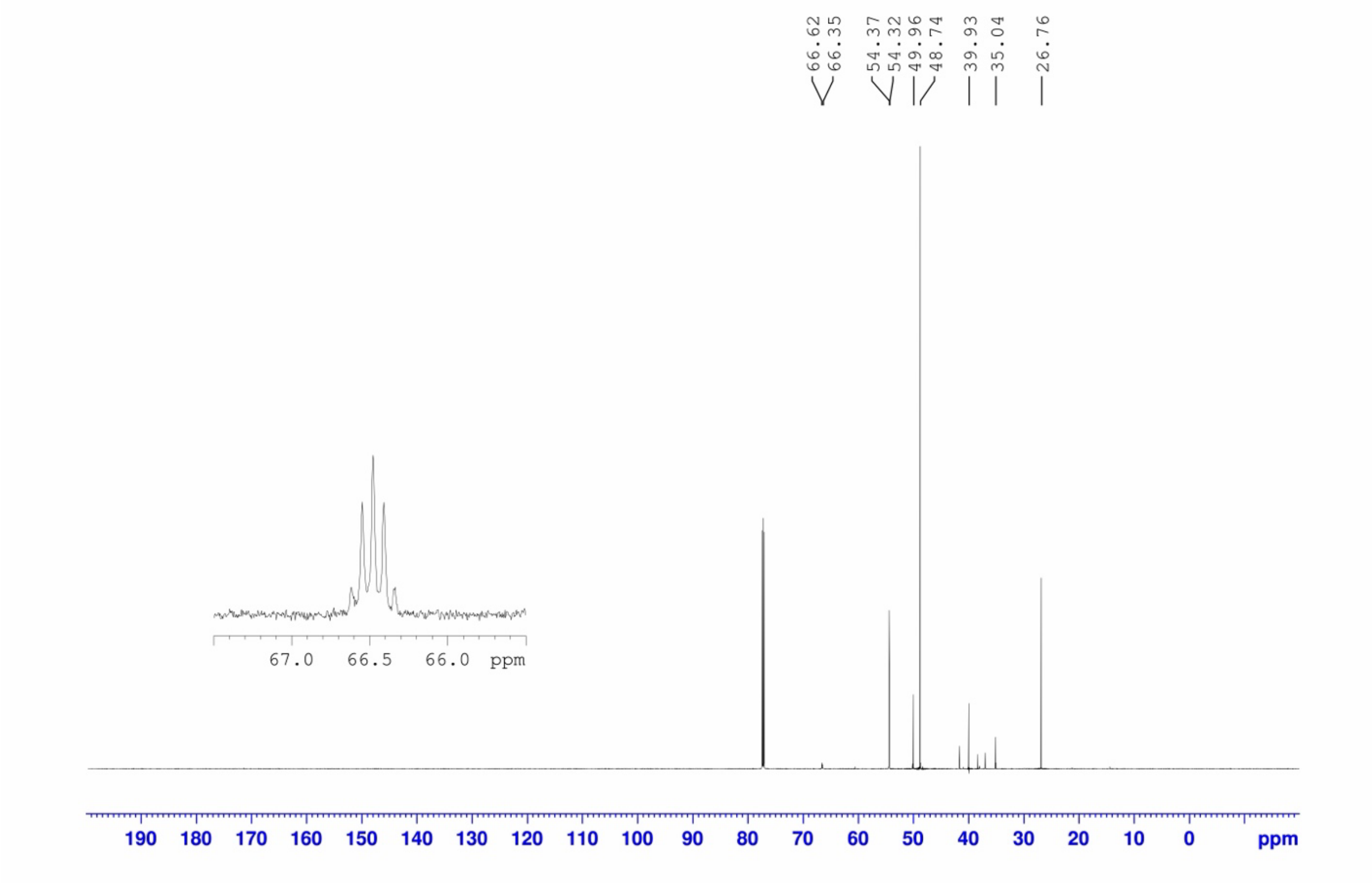

Figure S278. $^{13}C\{^1H\}$ NMR spectrum of (3'-(pentafluoro-λ^6-sulfanyl)-[1,1'-bi(bicyclo[1.1.1]pentan)]-3-yl)methanethiol (**64**) in $CDCl_3$.

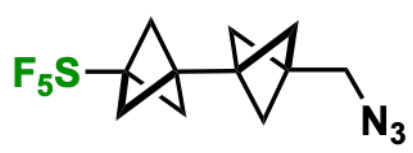

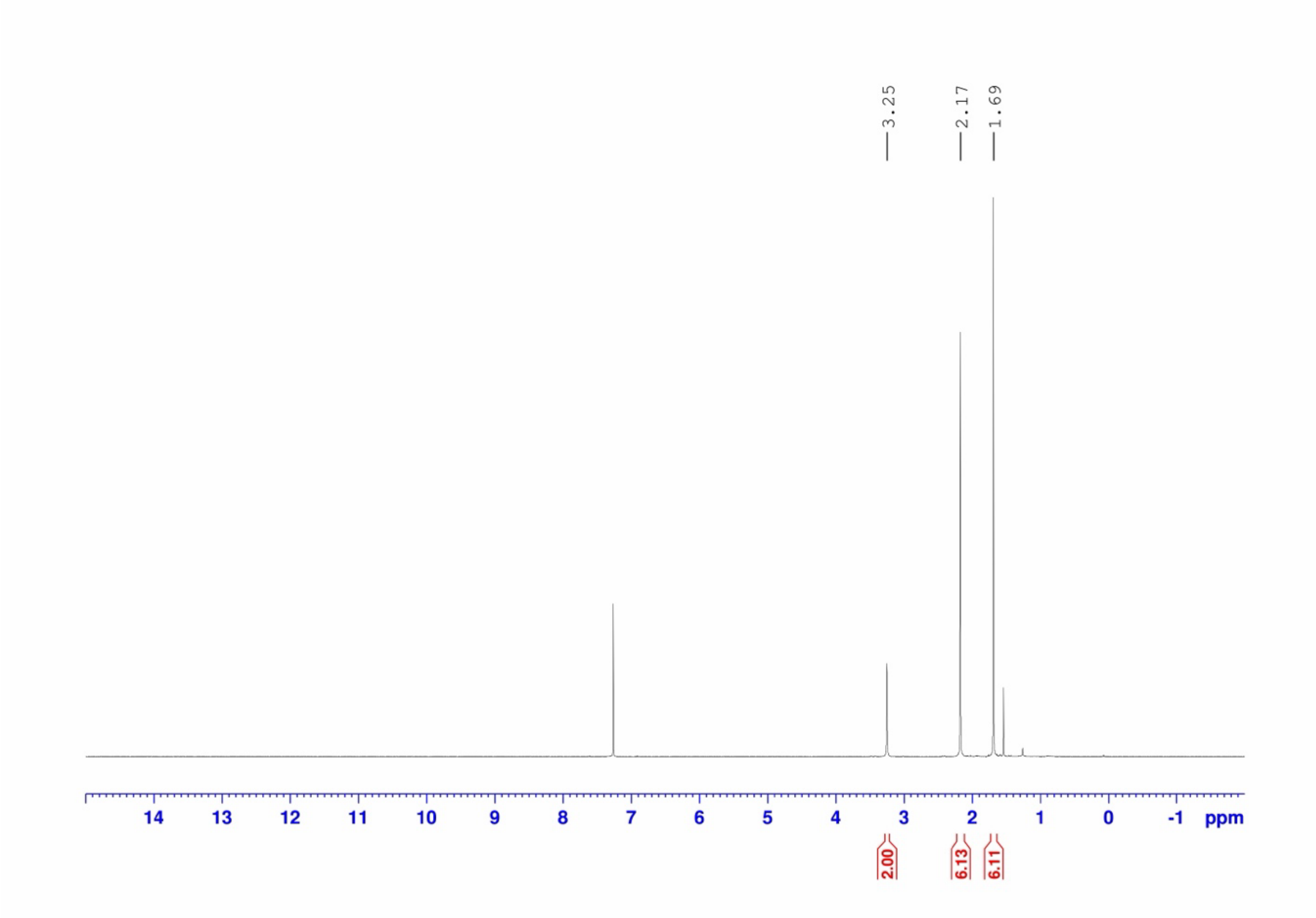

Figure S279. ^{1}H NMR spectrum of (3'-(azidomethyl)-[1,1'-bi(bicyclo[1.1.1]pentan)]-3-yl)pentafluoro-λ^6-sulfane (**65**) in $CDCl_3$.

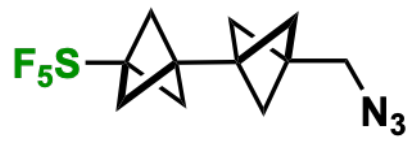

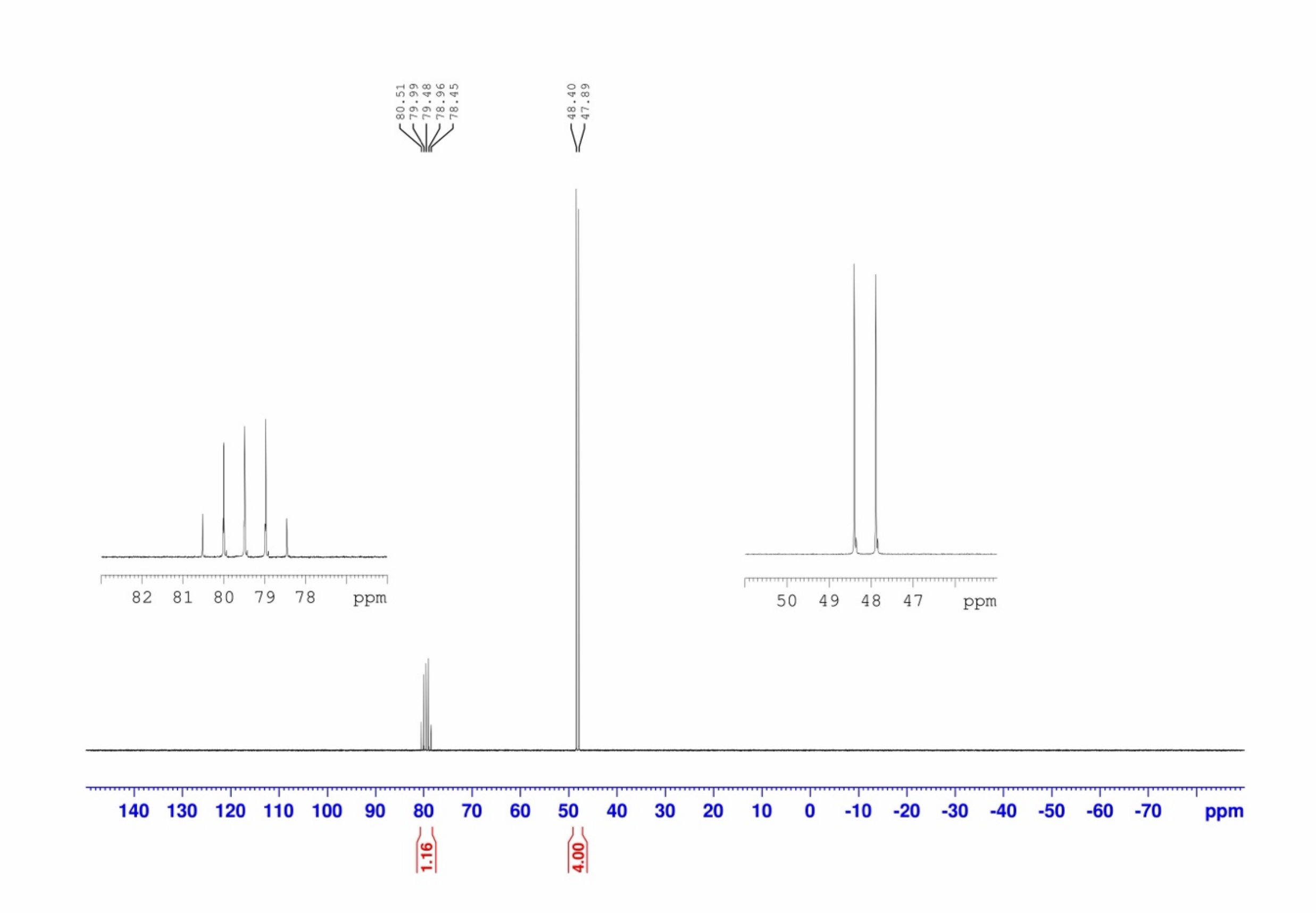

Figure S280. ^{19}F NMR spectrum of (3'-(azidomethyl)-[1,1'-bi(bicyclo[1.1.1]pentan)]-3-yl)pentafluoro-λ^6-sulfane (**65**) in $CDCl_3$.

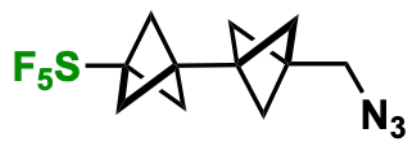

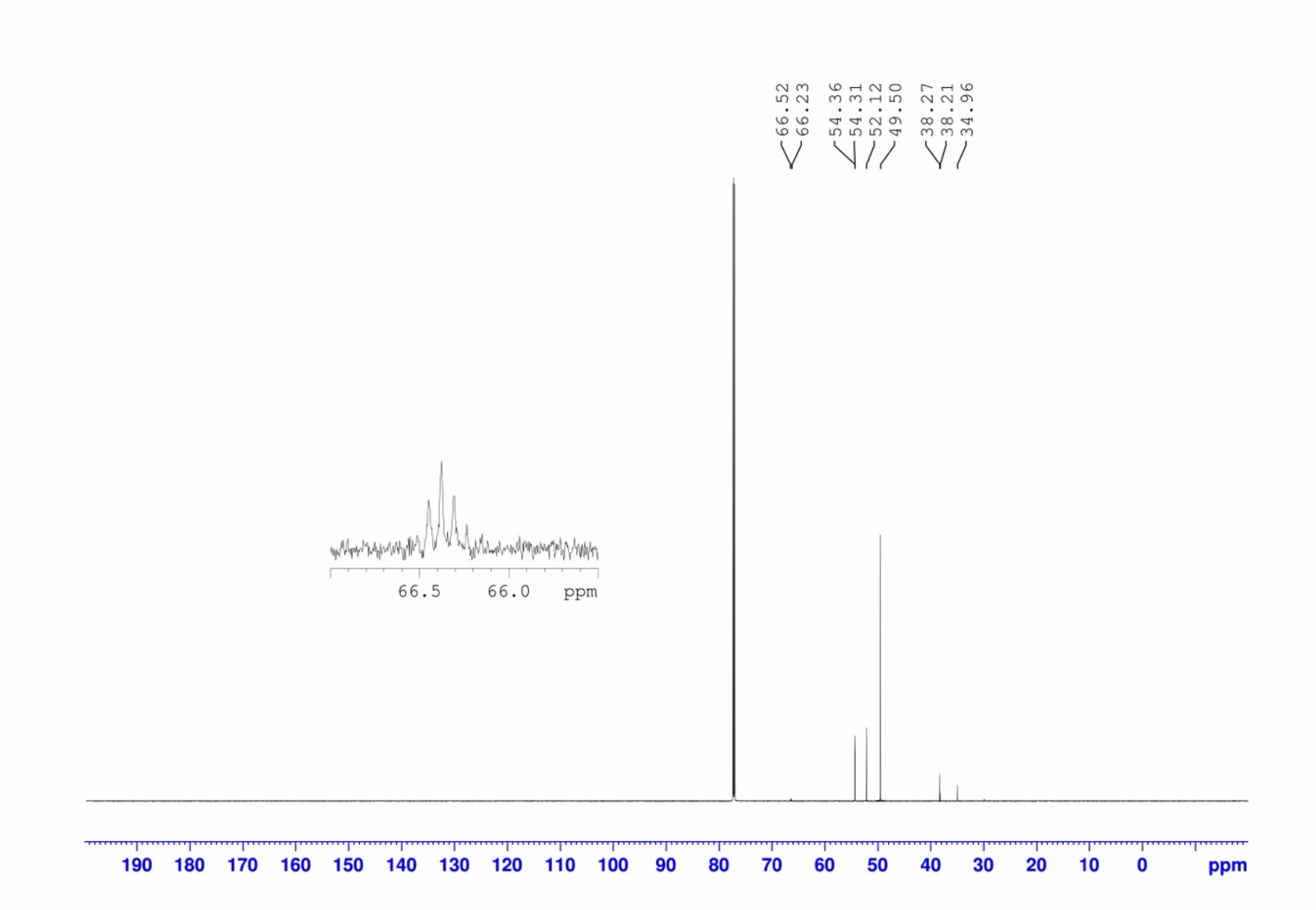

Figure S281. $^{13}C\{^{1}H\}$ NMR spectrum of (3'-(azidomethyl)-[1,1'-bi(bicyclo[1.1.1]pentan)]-3-yl)pentafluoro-λ^6-sulfane **(65)** in $CDCl_3$.

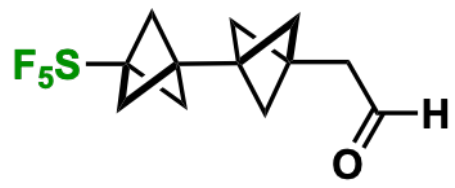

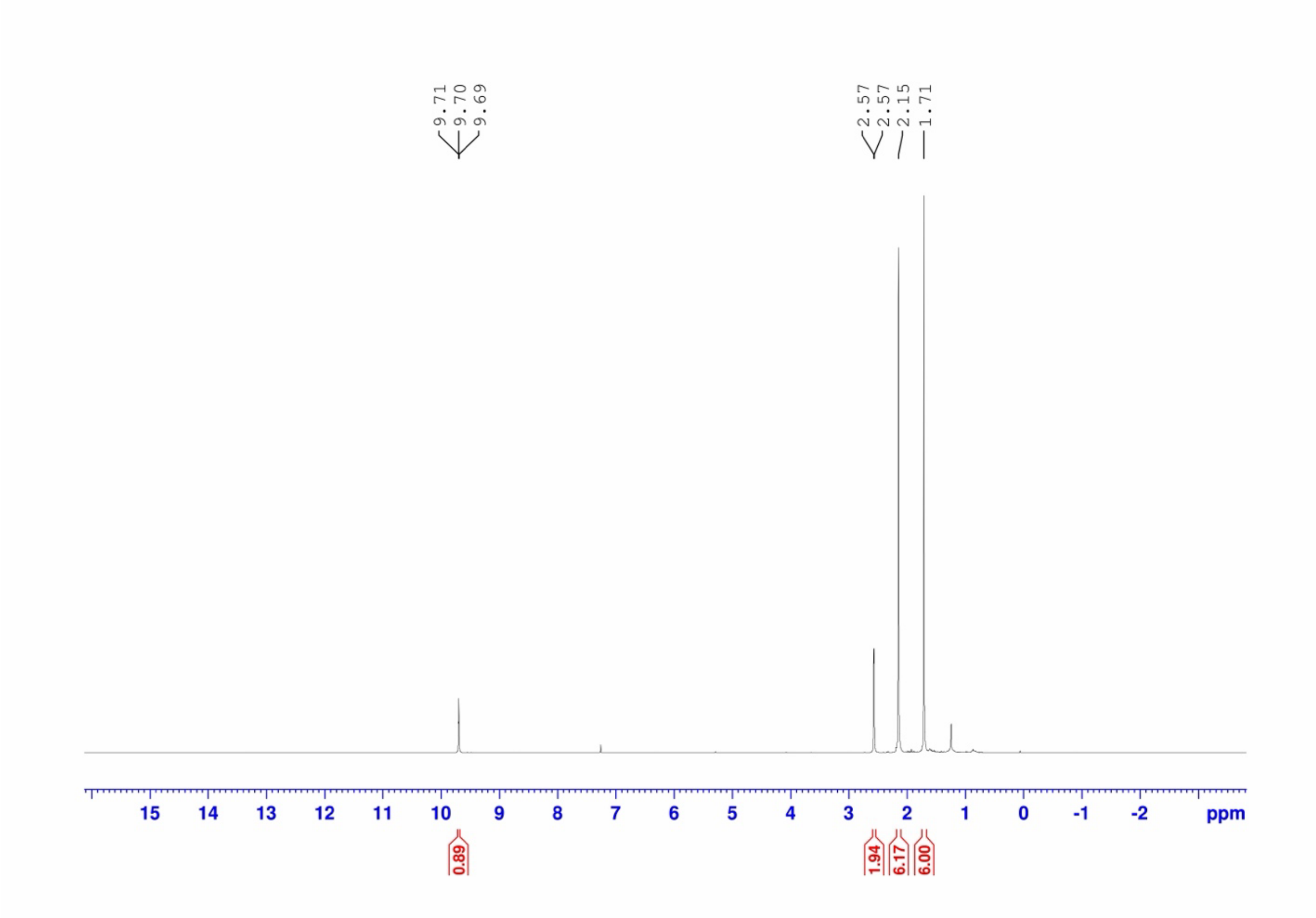

Figure S282. ^{1}H NMR spectrum of 2-(3'-(pentafluoro- λ^6-sulfanyl)-[1,1'-bi(bicyclo[1.1.1]pentan)]-3-yl)acetaldehyde **(66)** in $CDCl_3$.

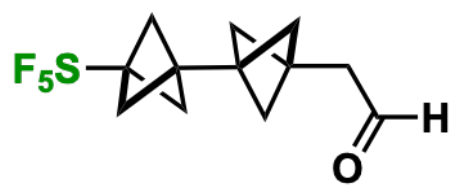

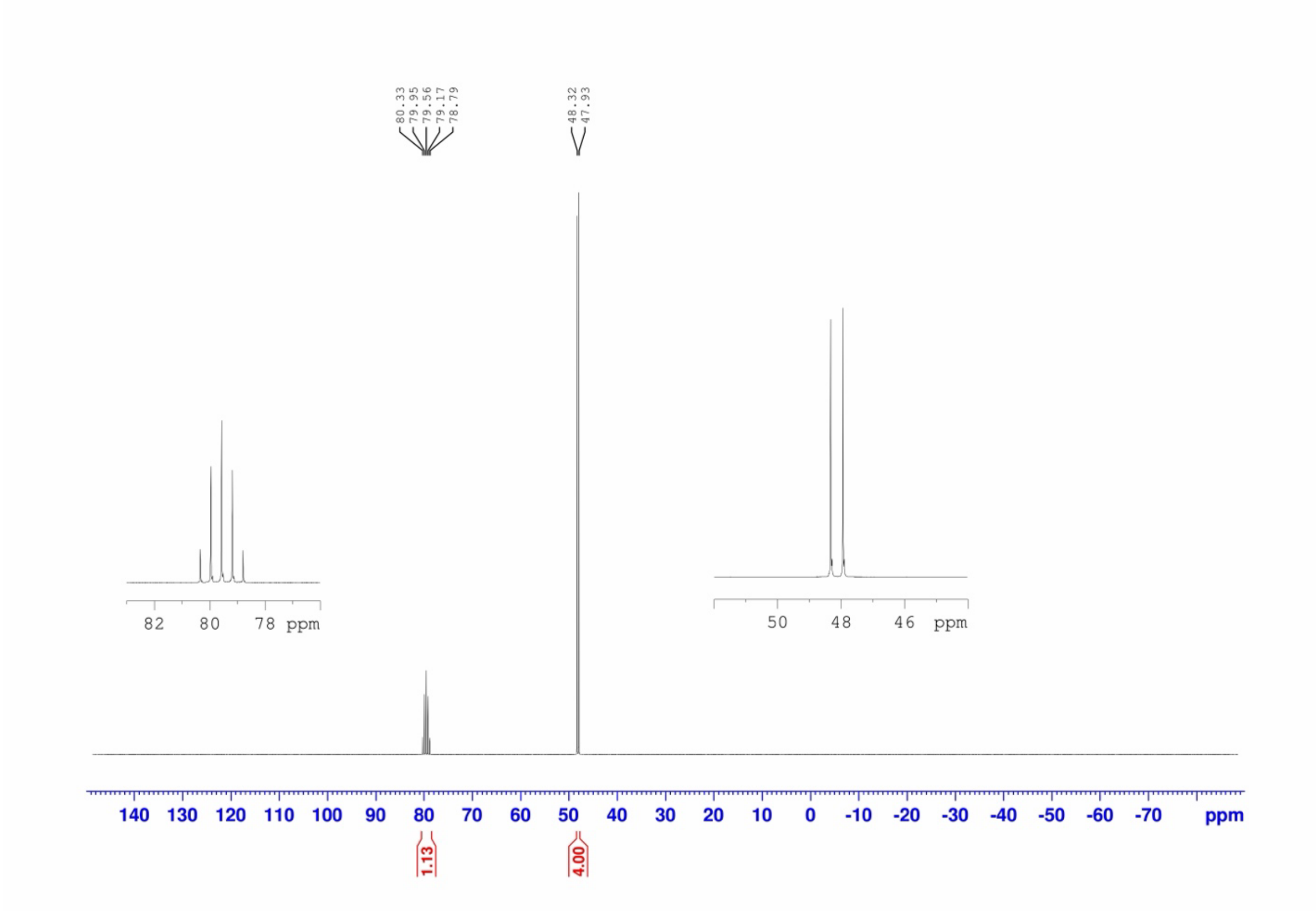

Figure S283. ^{19}F NMR spectrum of 2-(3'-(pentafluoro- λ^6-sulfanyl)-[1,1'-bi(bicyclo[1.1.1]pentan)]-3-yl)acetaldehyde **(66)** in $CDCl_3$.

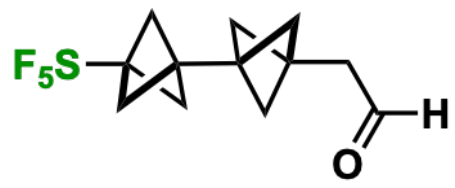

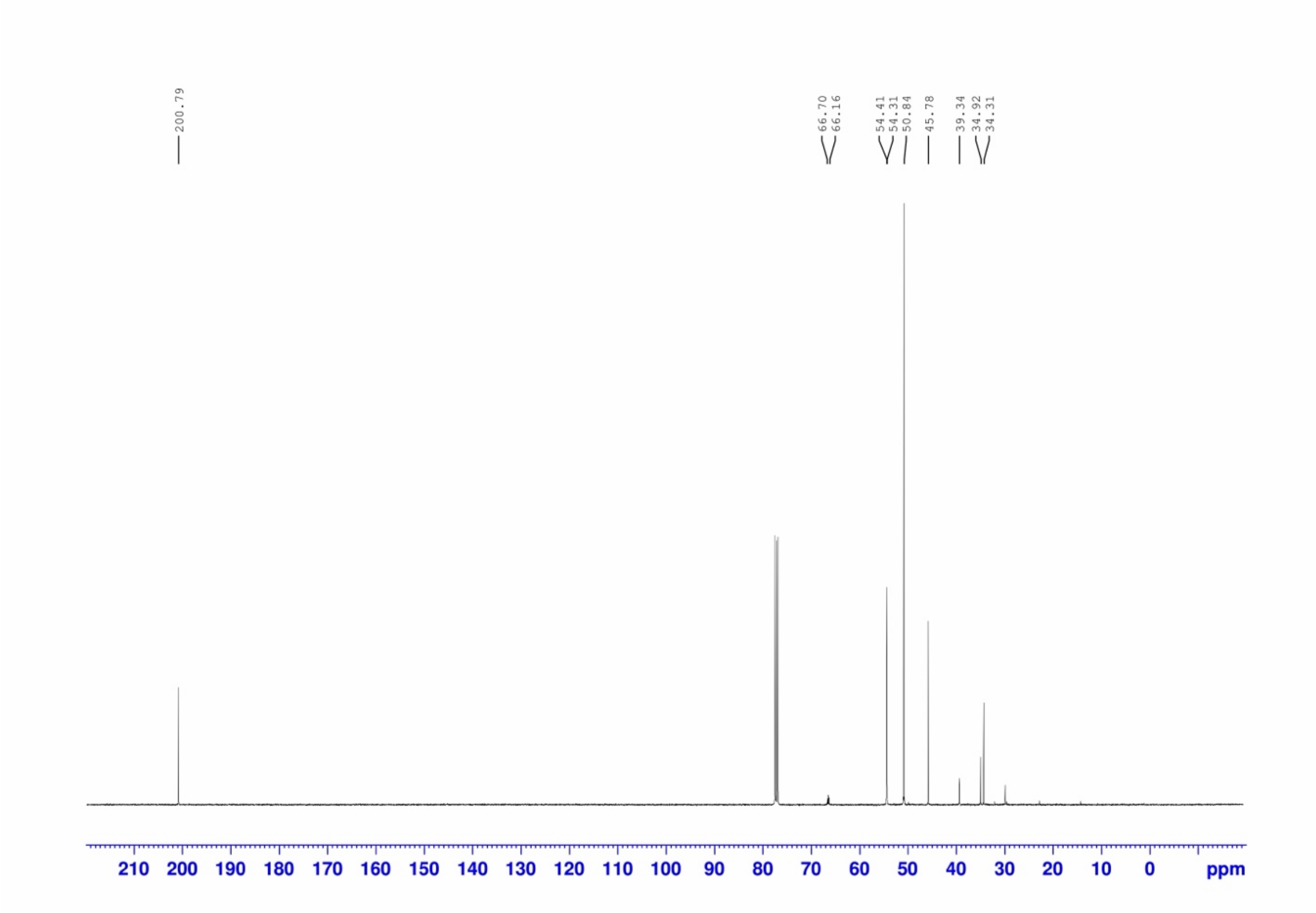

Figure S284. $^{13}C\{^1H\}$ NMR spectrum of 2-(3'-(pentafluoro- λ^6-sulfanyl)-[1,1'-bi(bicyclo[1.1.1]pentan)]-3-yl)acetaldehyde **(66)** in $CDCl_3$.

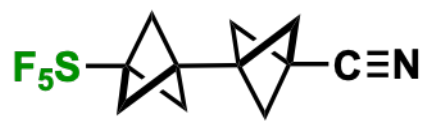

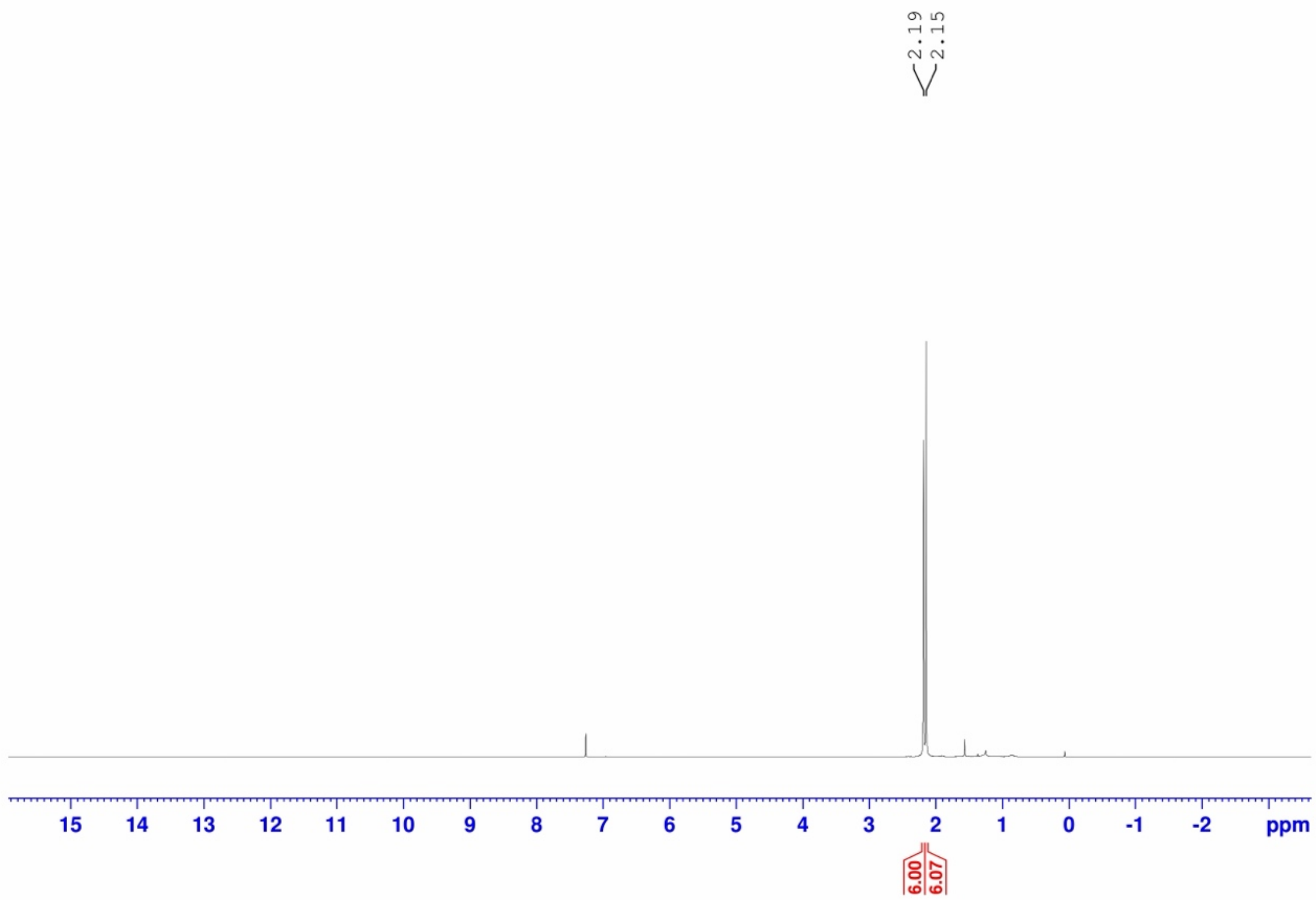

Figure S285.^{1}H NMR spectrum of 3'-(pentafluoro-λ^6-sulfanyl)-[1,1'-bi(bicyclo[1.1.1]pentane)]-3-carbonitrile **(67)** in $CDCl_3$.

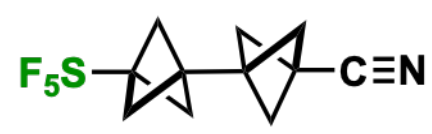

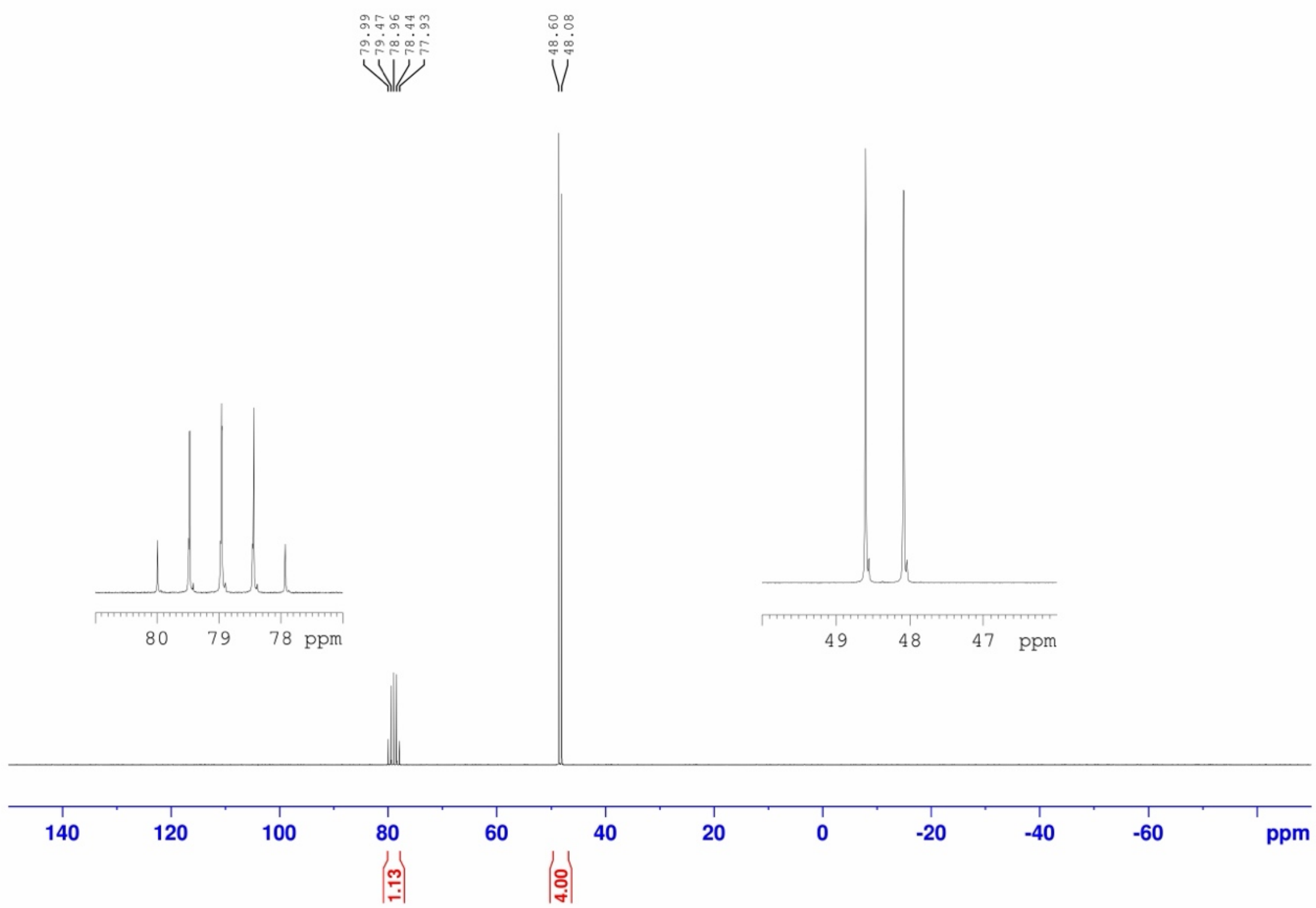

Figure S286. ^{19}F NMR spectrum of 3'-(pentafluoro-λ^6-sulfanyl)-[1,1'-bi(bicyclo[1.1.1]pentane)]-3-carbonitrile **(67)** in $CDCl_3$.

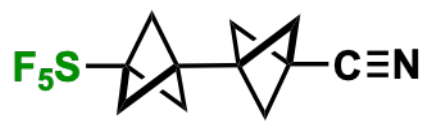

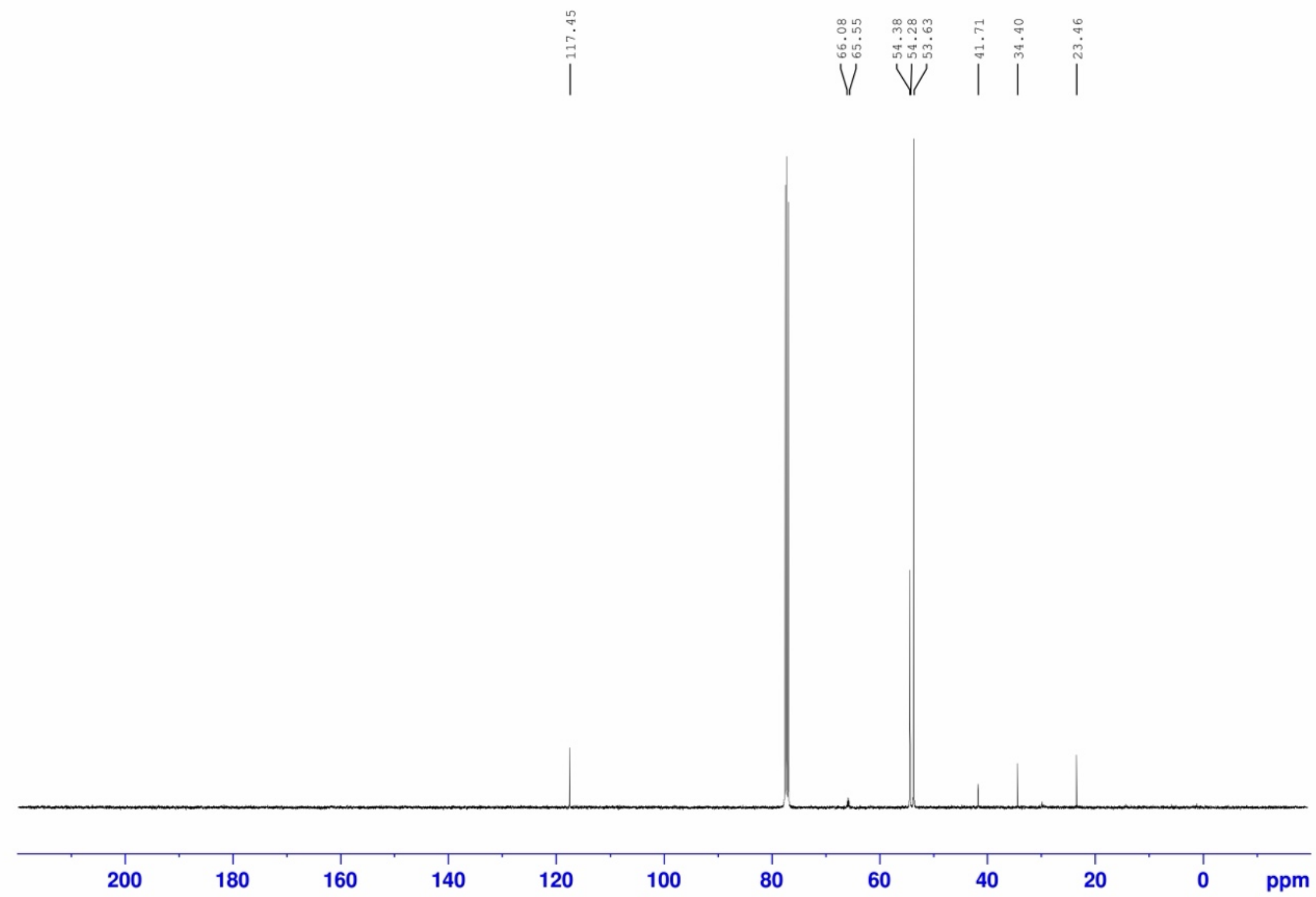

Figure S287. $^{13}C\{^1H\}$ NMR spectrum of 3'-(pentafluoro-λ6-sulfanyl)-[1,1'-bi(bicyclo[1.1.1]pentane)]-3-carbonitrile **(67)** in $CDCl_3$.

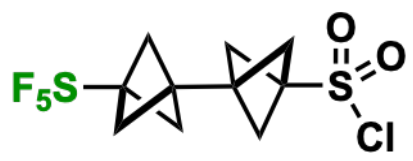

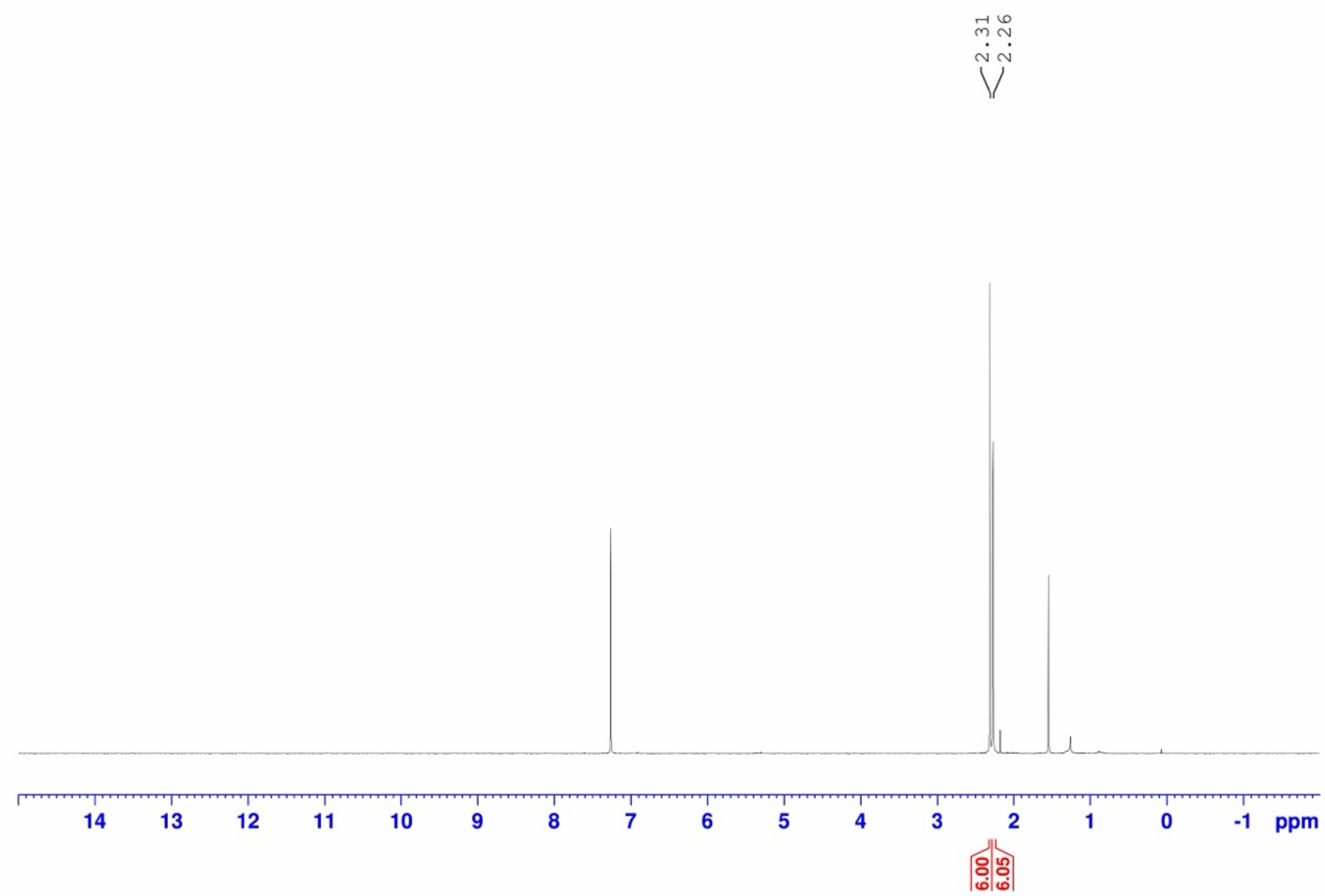

Figure S288. ^{1}H NMR spectrum of 3'-(pentafluoro- λ^6--sulfanyl)-[1,1'-bi(bicyclo[1.1.1]pentane)]-3-sulfonyl chloride **(S-28)** in $CDCl_3$.

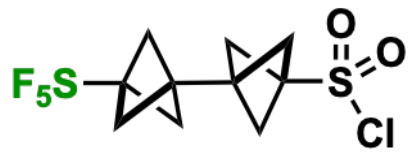

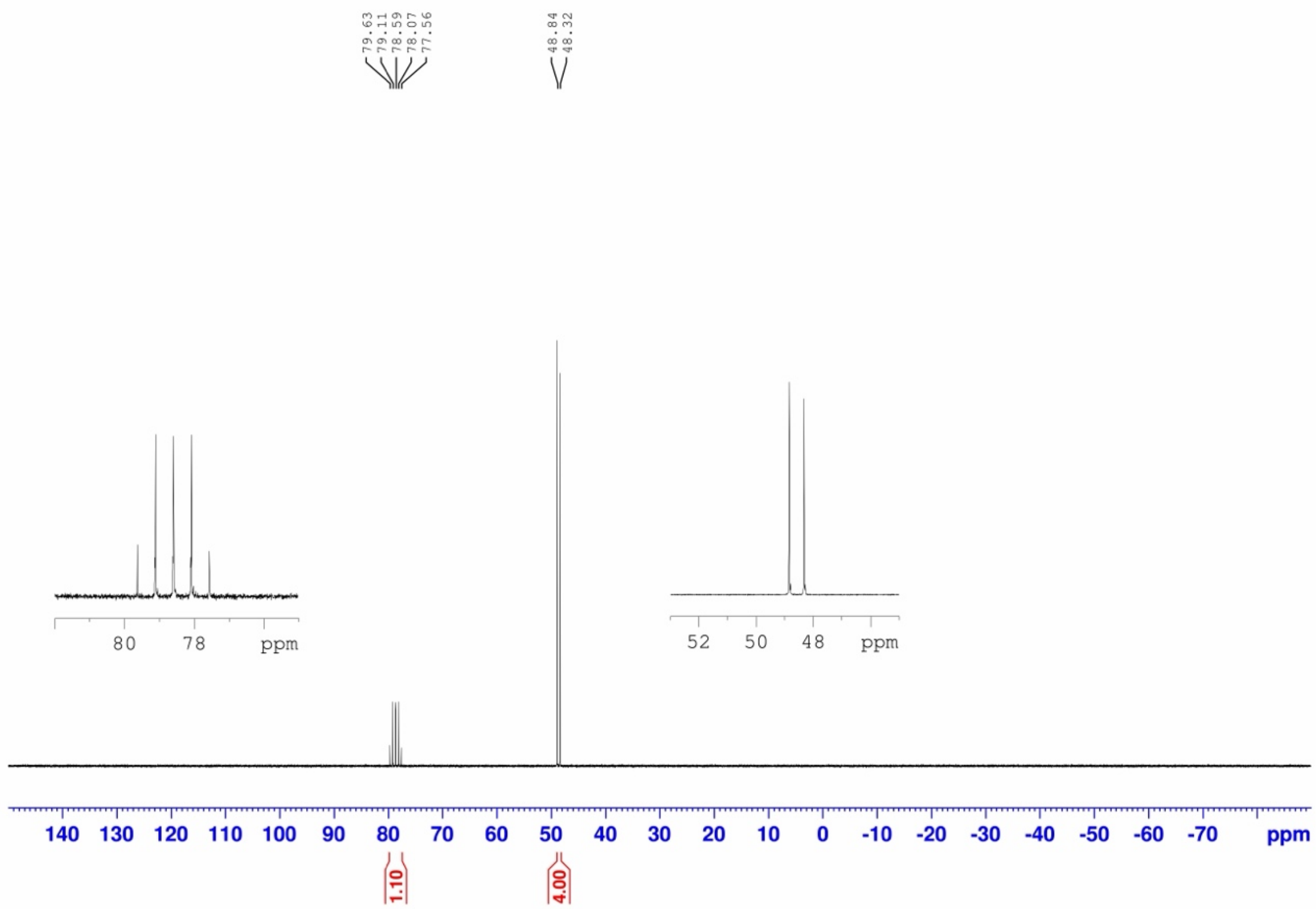

Figure S289. ^{19}F NMR spectrum of 3'-(pentafluoro- λ^6--sulfanyl)-[1,1'-bi(bicyclo[1.1.1]pentane)]-3-sulfonyl chloride **(S-28)** in $CDCl_3$.

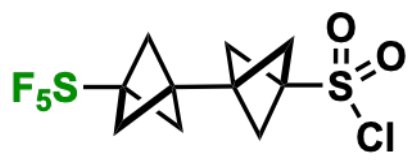

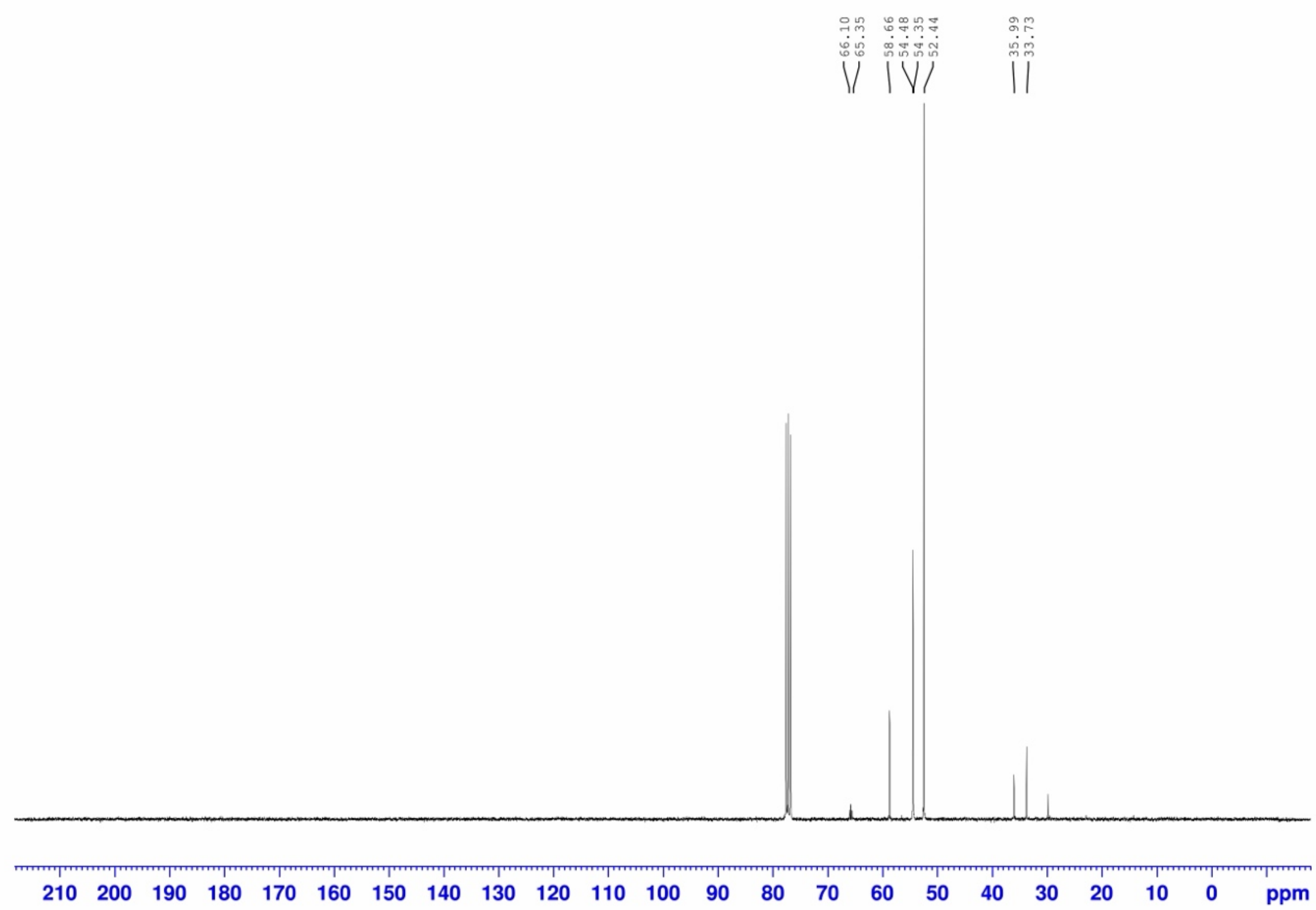

Figure S290. $^{13}C\{^{1}H\}$ NMR spectrum of 3'-(pentafluoro-λ^6-sulfanyl)-[1,1'-bi(bicyclo[1.1.1]pentane)]-3-sulfonyl chloride **(S-28)** in $CDCl_3$.

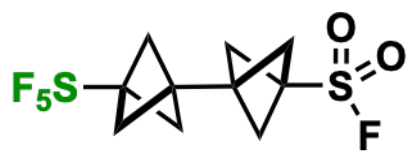

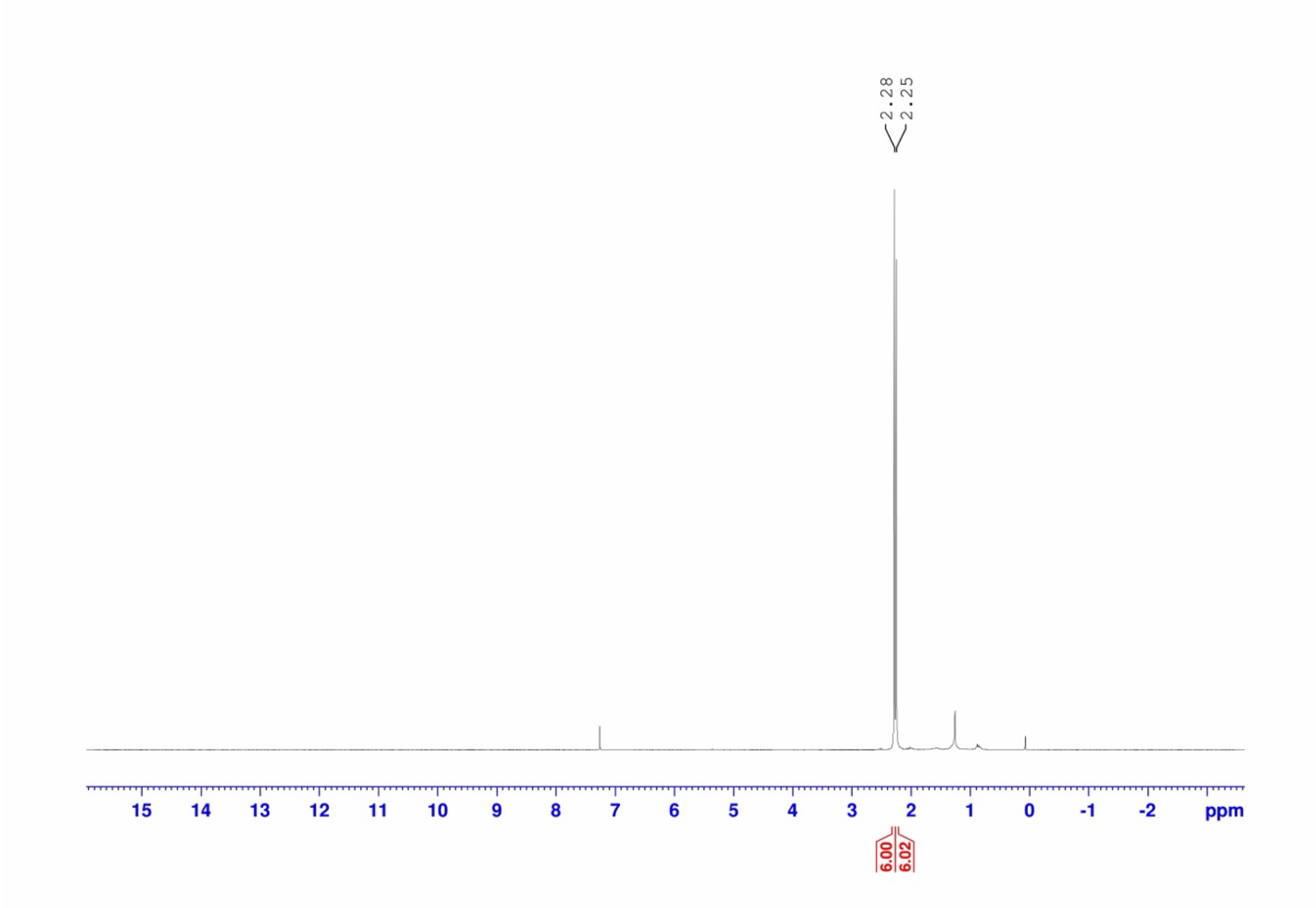

Figure S291. ^{1}H NMR spectrum of 3'-(pentafluoro-λ^6-sulfanyl)-[1,1'-bi(bicyclo[1.1.1]pentane)]-3-sulfonyl fluoride **(68)** in $CDCl_3$.

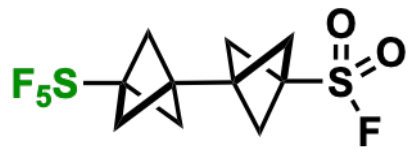

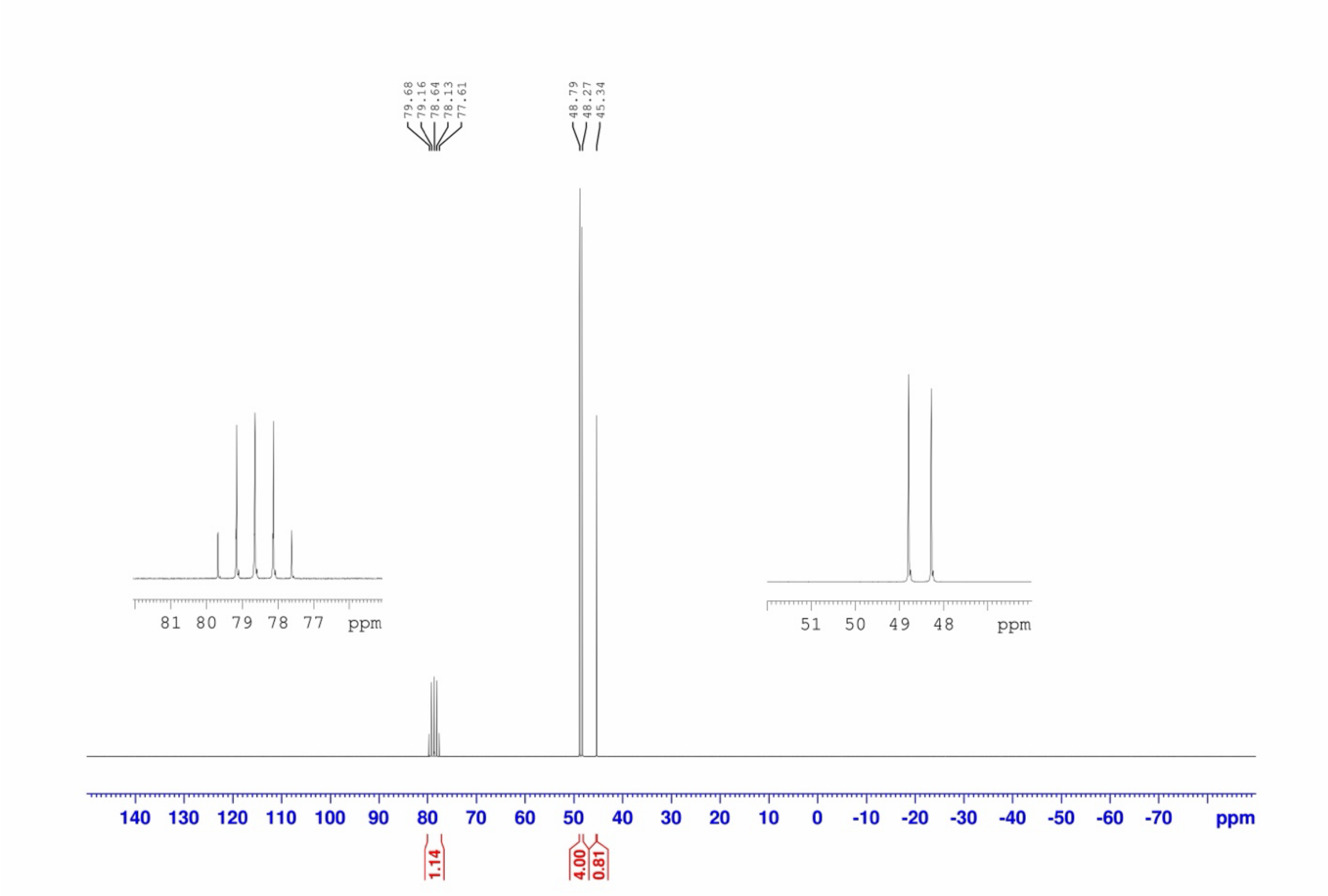

Figure S292. ^{19}F NMR spectrum of 3'-(pentafluoro-λ^6-sulfanyl)-[1,1'-bi(bicyclo[1.1.1]pentane)]-3-sulfonyl fluoride **(68)** in $CDCl_3$.

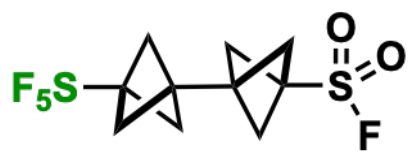

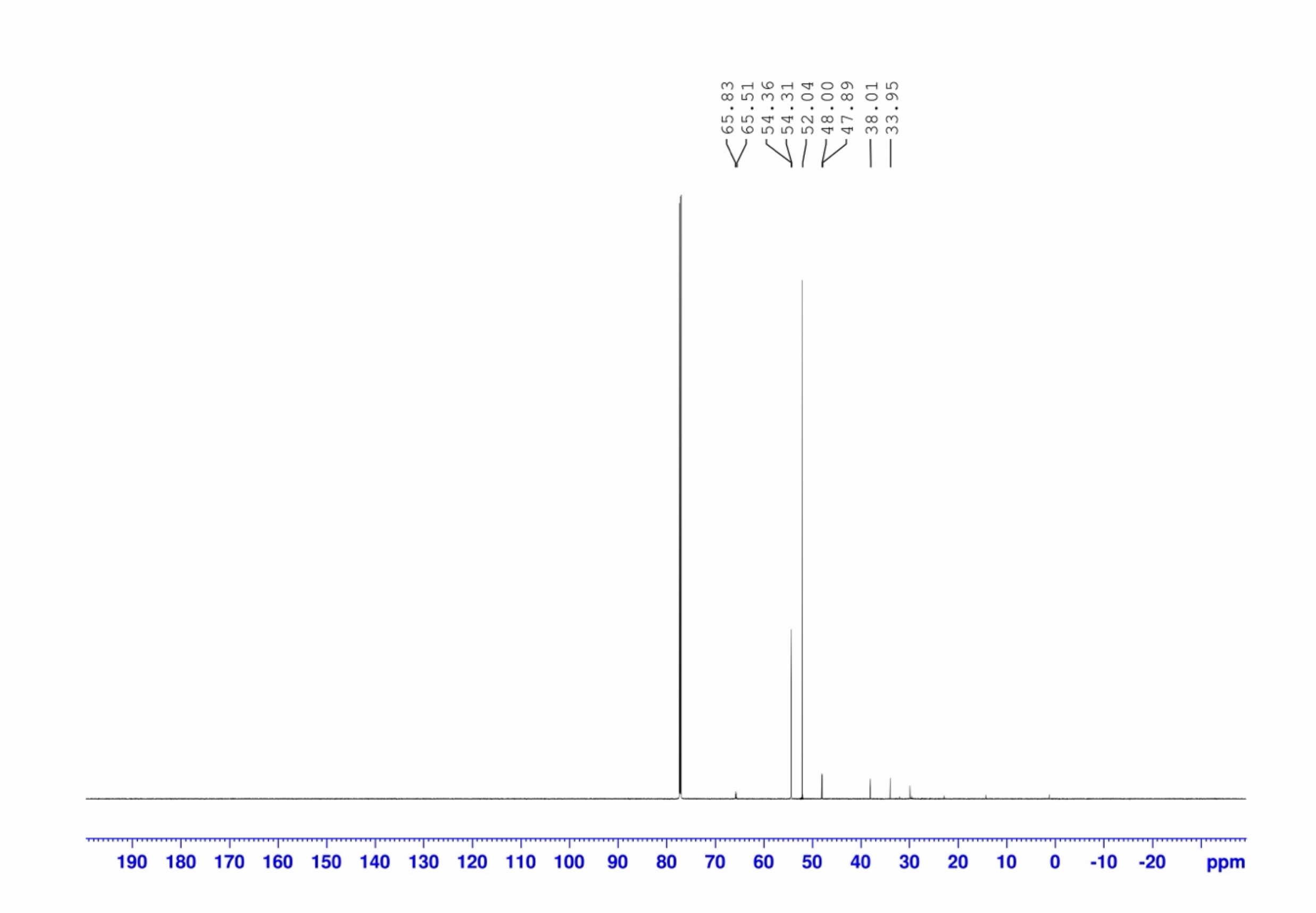

Figure S293. $^{13}C\{^1H\}$ NMR spectrum of 3'-(pentafluoro-λ^6-sulfanyl)-[1,1'-bi(bicyclo[1.1.1]pentane)]-3-sulfonyl fluoride **(68)** in $CDCl_3$.

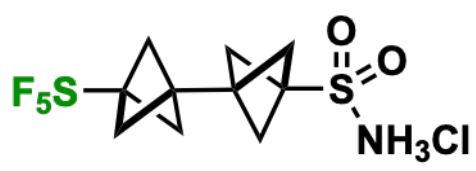

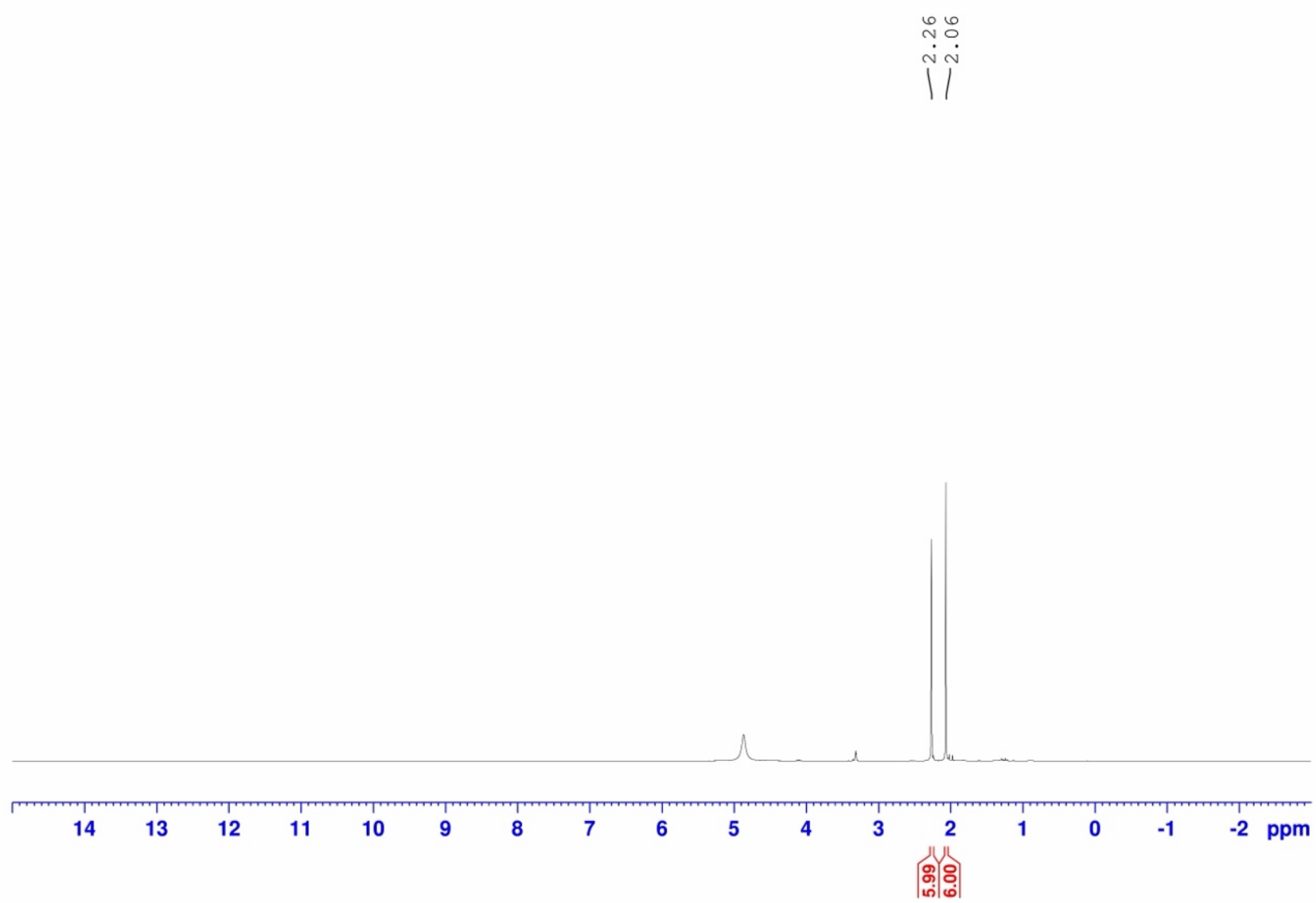

Figure S294. ^{1}H NMR spectrum of chloro((3'-(pentafluoro-λ^6-sulfanyl)-[1,1'-bi(bicyclo[1.1.1]pentan)]-3-yl)sulfonyl)-λ^5-azane **(69)** in $CDCl_3$.

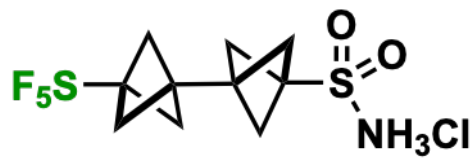

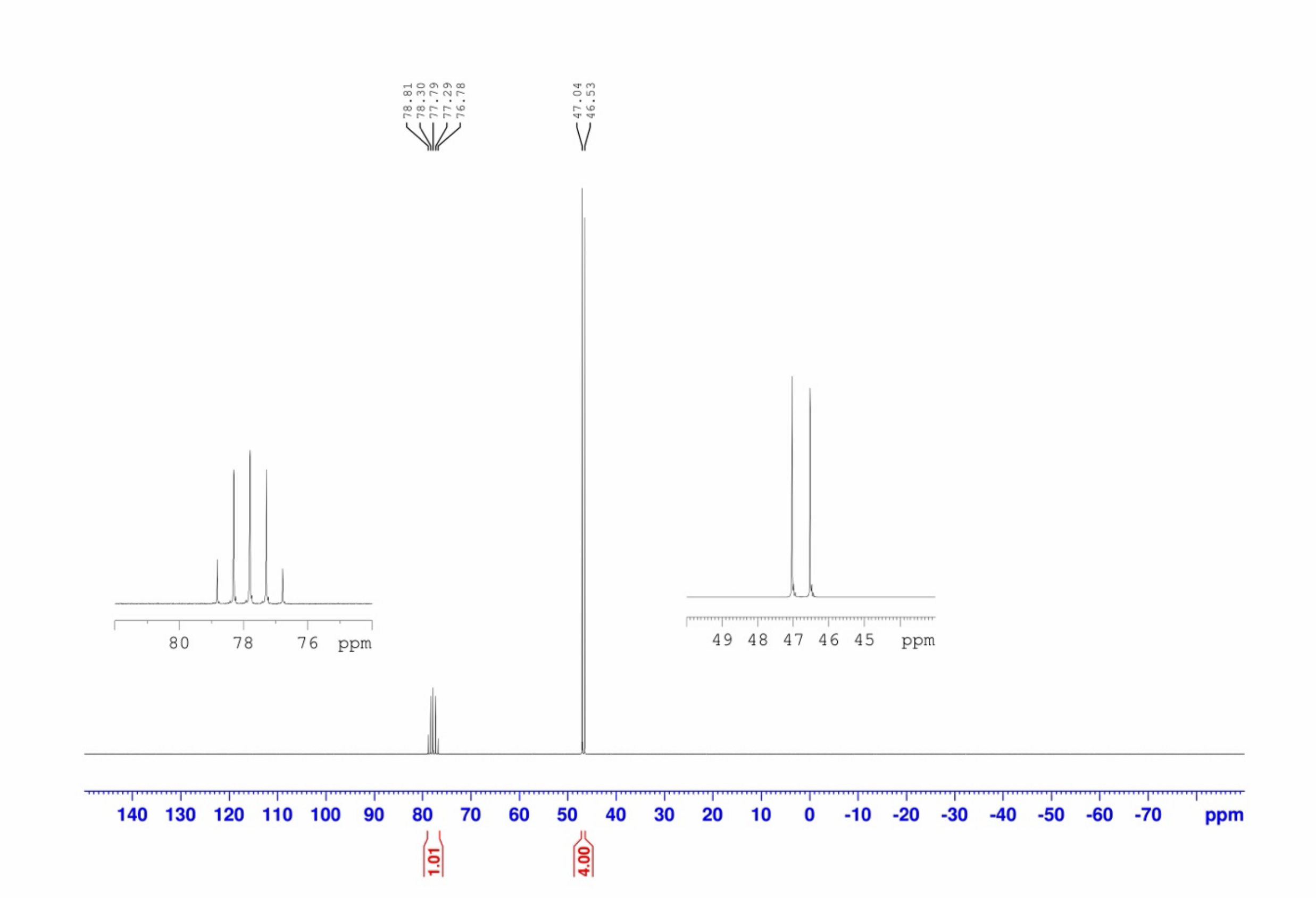

Figure S295. ^{19}F NMR spectrum of chloro((3'-(pentafluoro-λ^6-sulfanyl)-[1,1'-bi(bicyclo[1.1.1]pentan)]-3-yl)sulfonyl)-λ^5-azane **(69)** in $CDCl_3$.

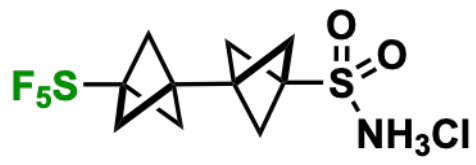

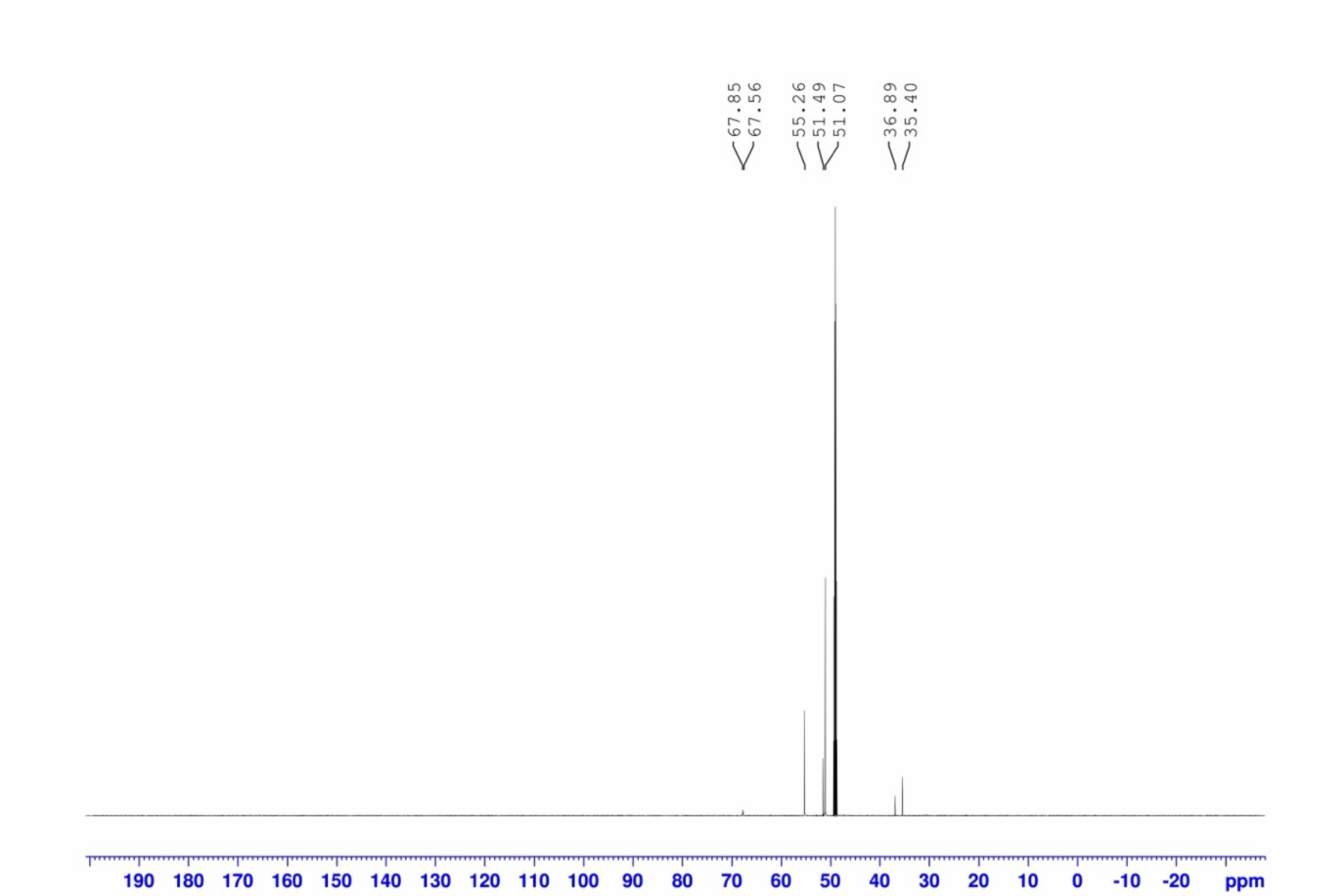

Figure S296. $^{13}C\{^{1}H\}$ NMR spectrum of chloro((3'-(pentafluoro-λ^6-sulfanyl)-[1,1'-bi(bicyclo[1.1.1]pentan)]-3-yl)sulfonyl)- λ^5-azane **(69)** in $CDCl_3$.

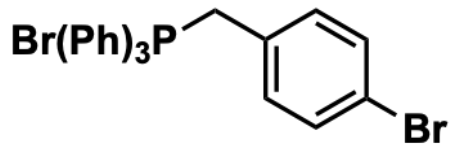

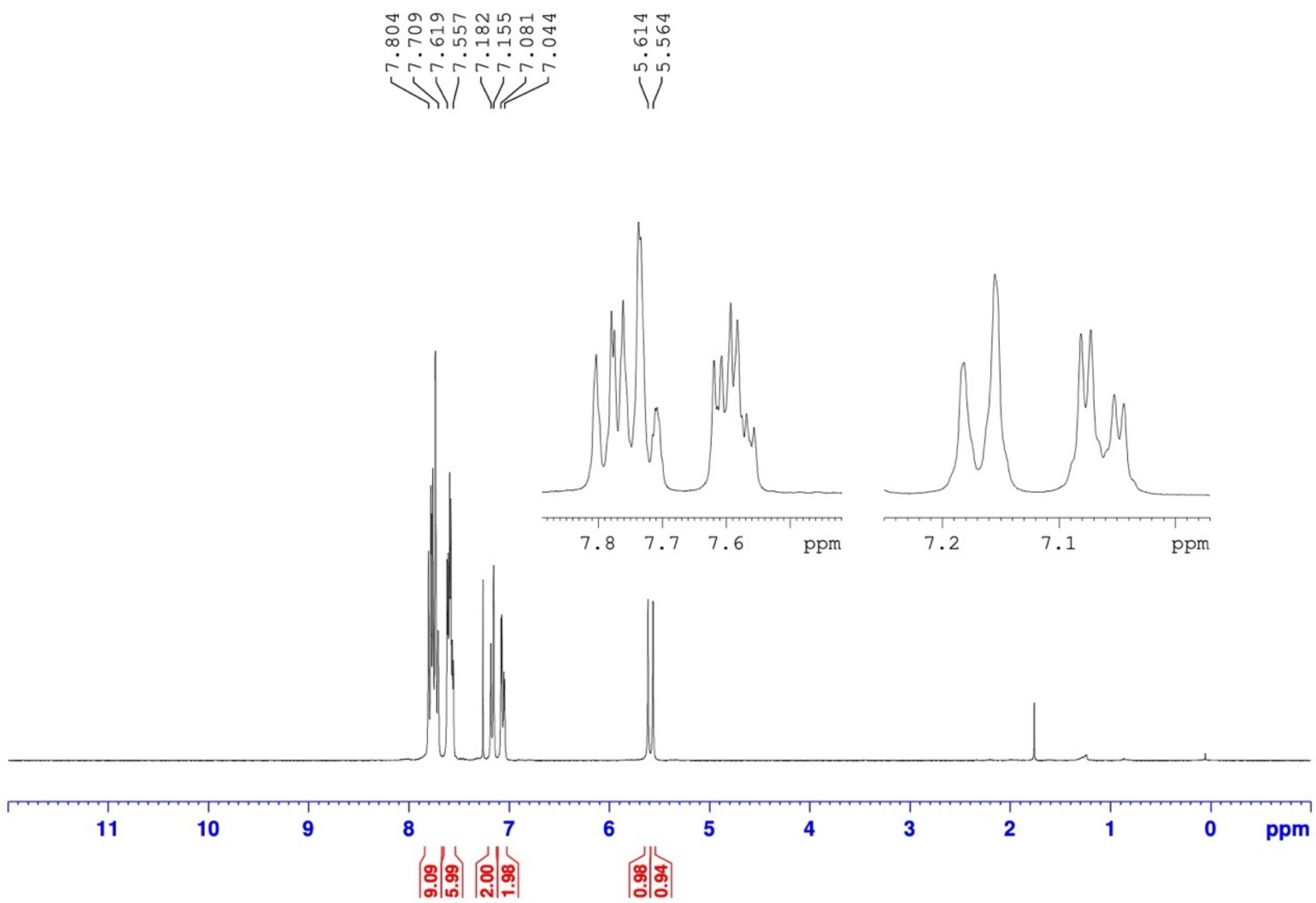

Figure S297. ^{1}H NMR spectrum of (4-bromobenzyl)triphenylphosphonium bromide **(S-29)** in $CDCl_3$.

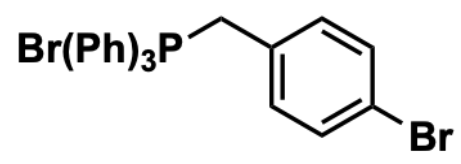

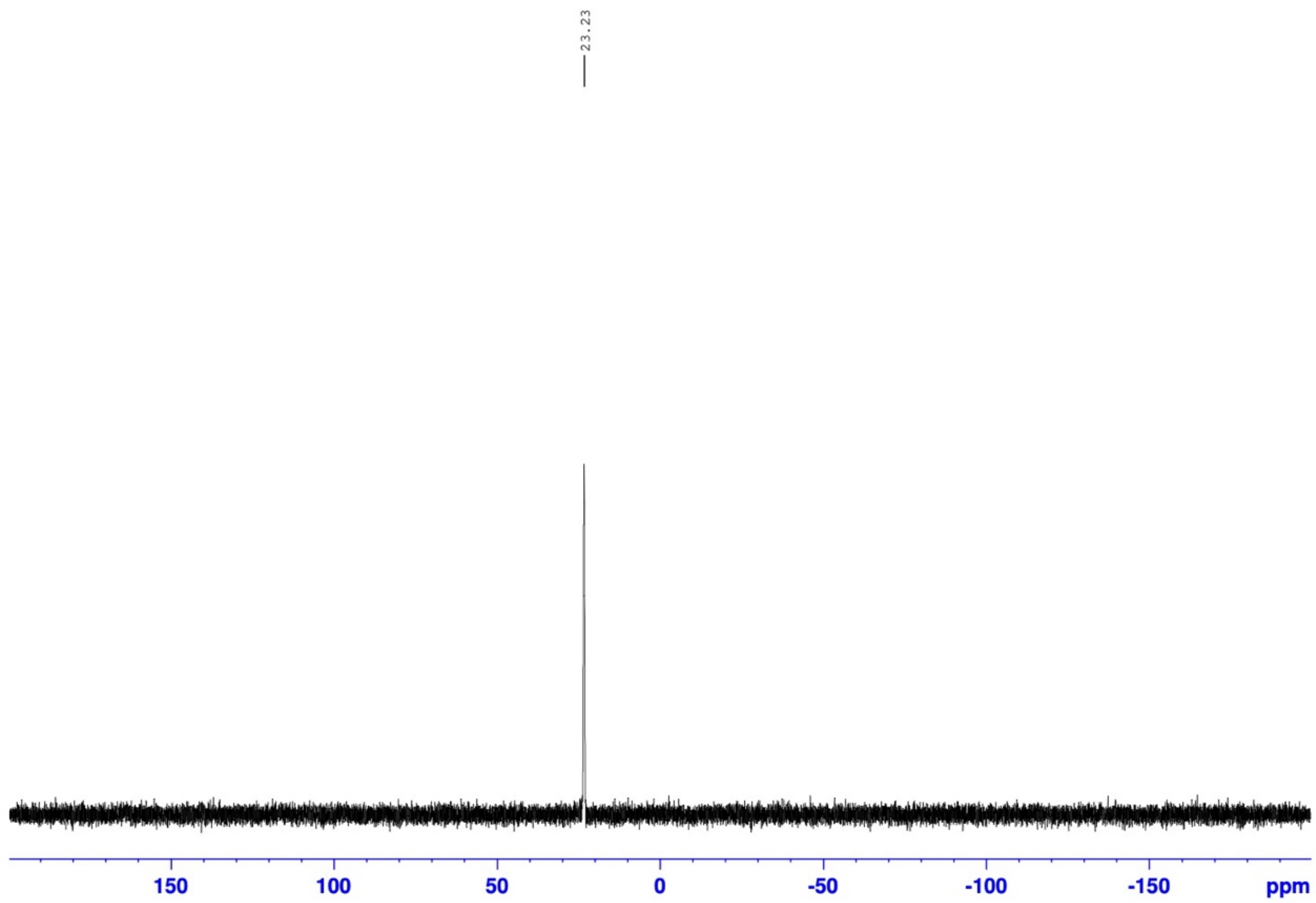

Figure S298. $^{31}P\{^{1}H\}$ NMR spectrum of (4-bromobenzyl)triphenylphosphonium bromide **(S-29)** in $CDCl_3$.

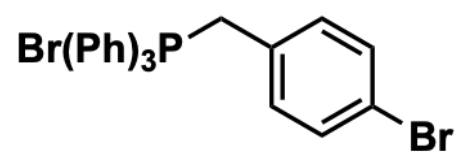

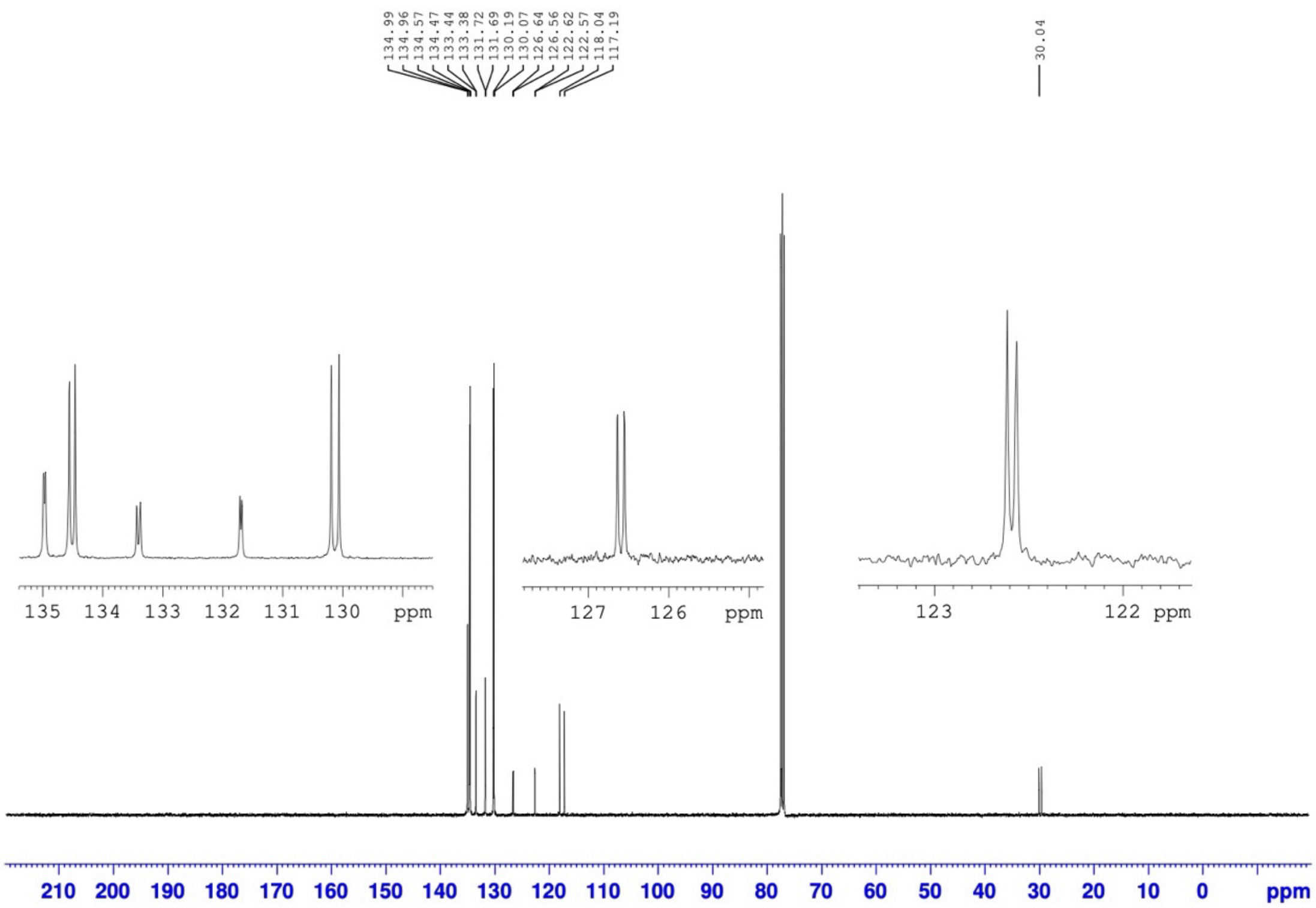

Figure S299. $^{13}C\{^1H\}$ NMR spectrum of (4-bromobenzyl)triphenylphosphonium bromide **(S-29)** in $CDCl_3$.

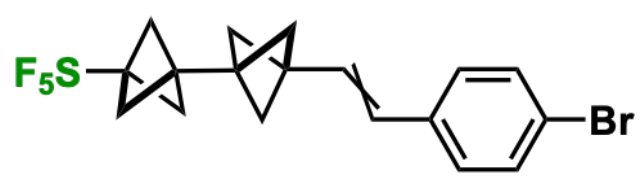

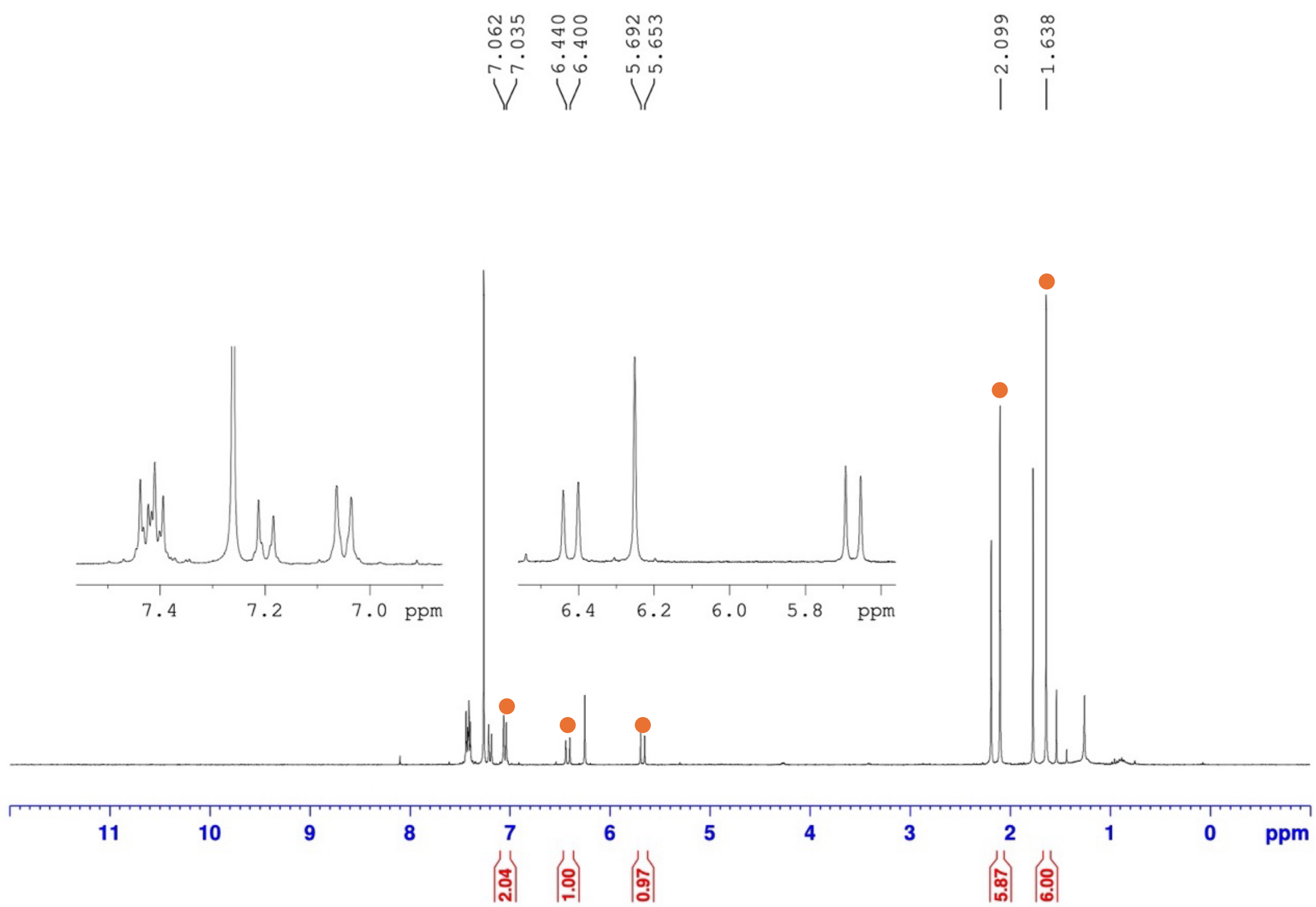

Figure S300. ^{1}H NMR spectrum of (3'-(4-bromostyryl)-[1,1'-bi(bicyclo[1.1.1]pentan)]-3-yl)pentafluoro-λ^6-sulfane **(S-30)** in $CDCl_3$. The *cis* isomer is indicated by orange dots.

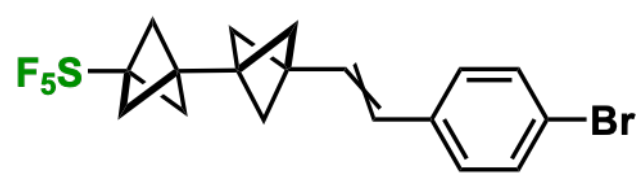

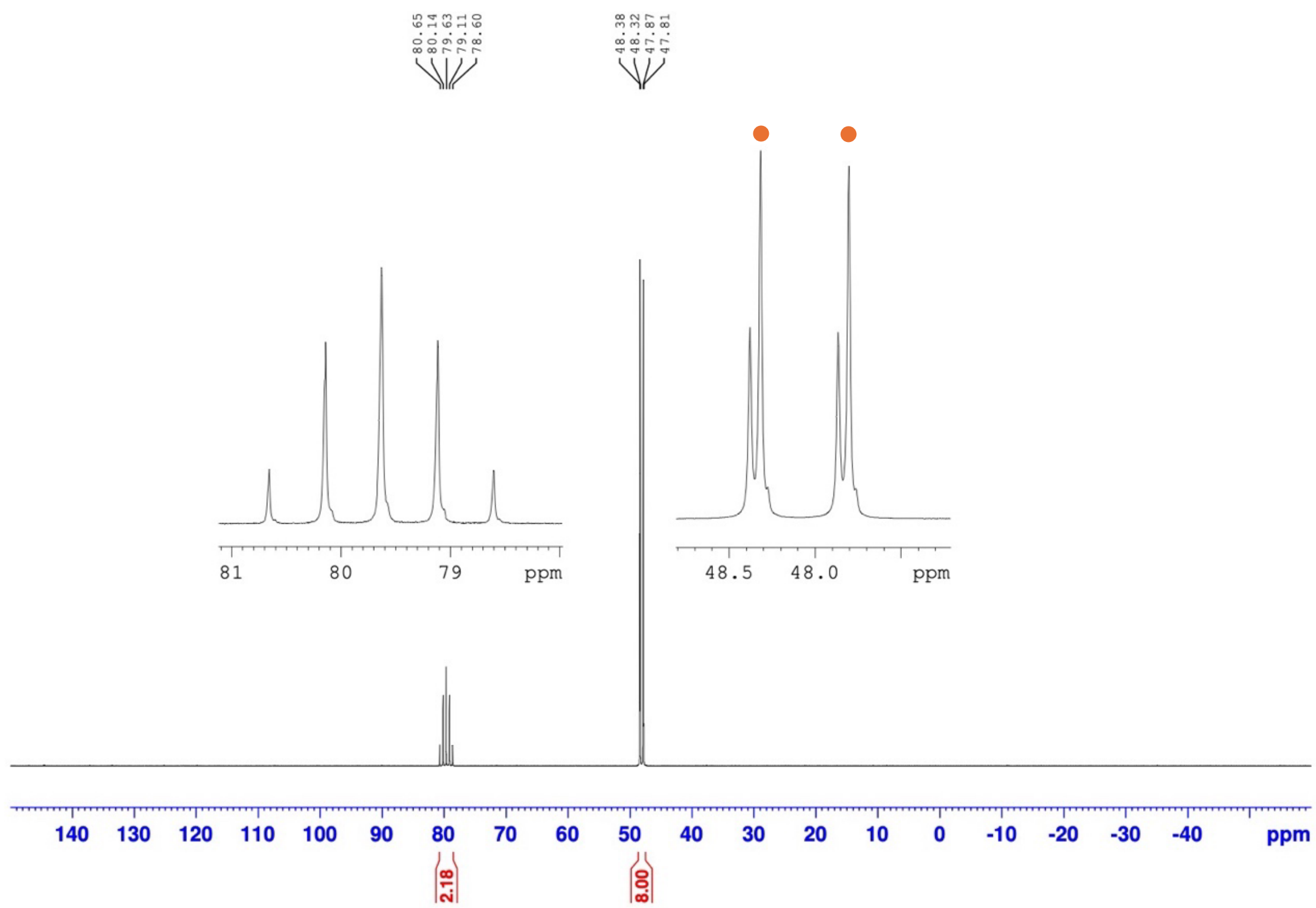

Figure S301. ^{19}F NMR spectrum of (3'-(4-bromostyryl)-[1,1'-bi(bicyclo[1.1.1]pentan)]-3-yl)pentafluoro-λ^6-sulfane **(S-30)** in $CDCl_3$. The *cis* isomer is indicated by orange dots.

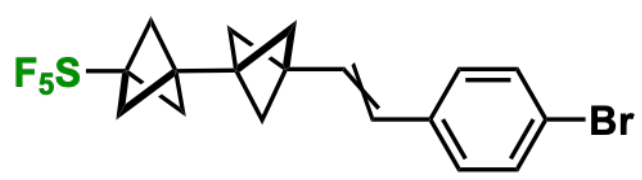

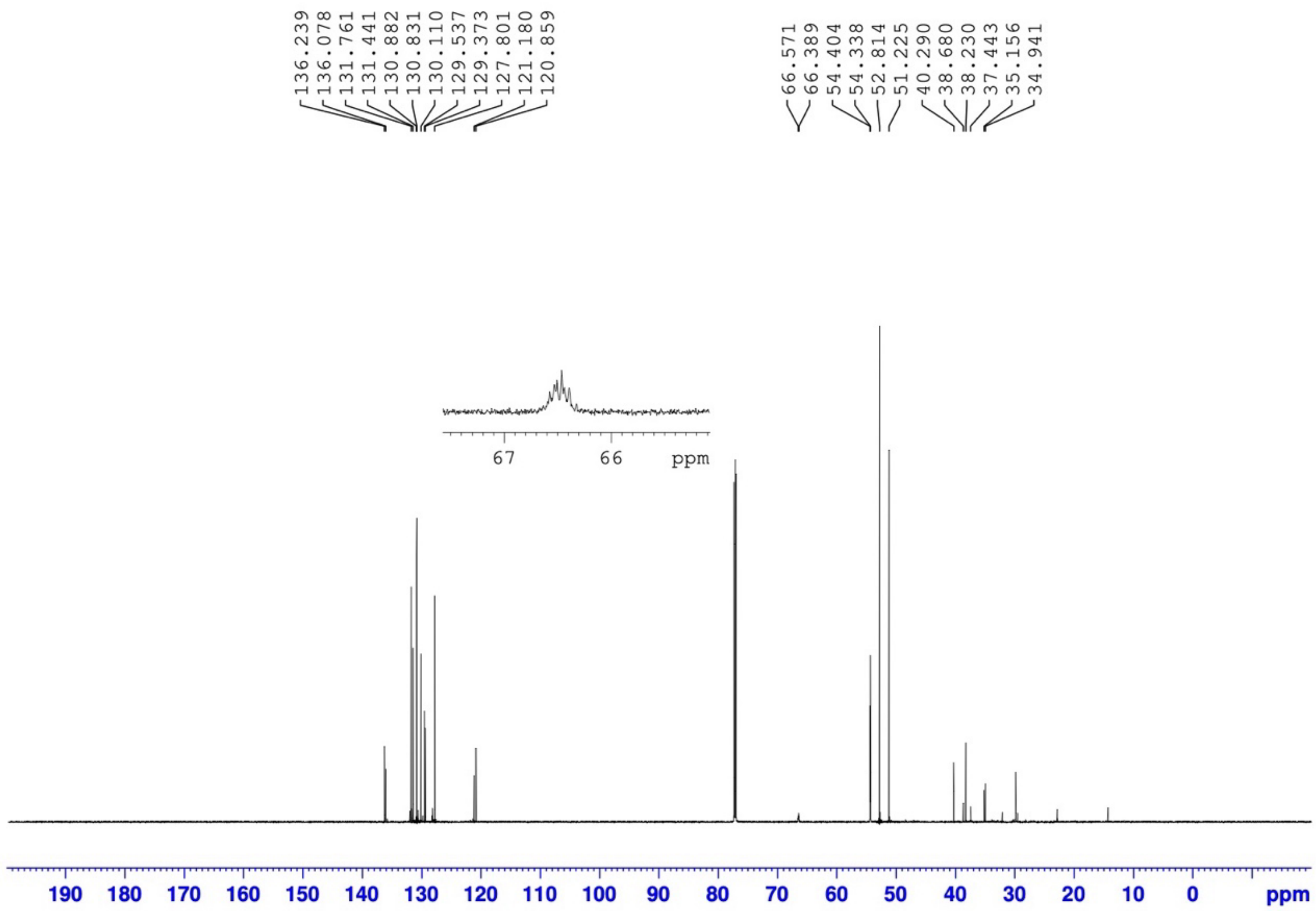

Figure S302. $^{13}C\{^1H\}$ NMR spectrum of (3'-(4-bromostyryl)-[1,1'-bi(bicyclo[1.1.1]pentan)]-3-yl)pentafluoro-λ^6-sulfane **(S-30)** in $CDCl_3$.

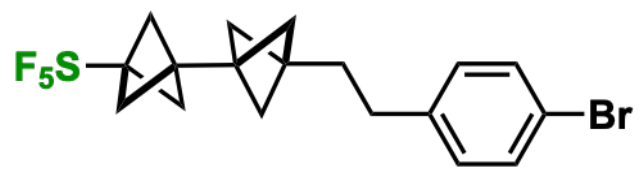

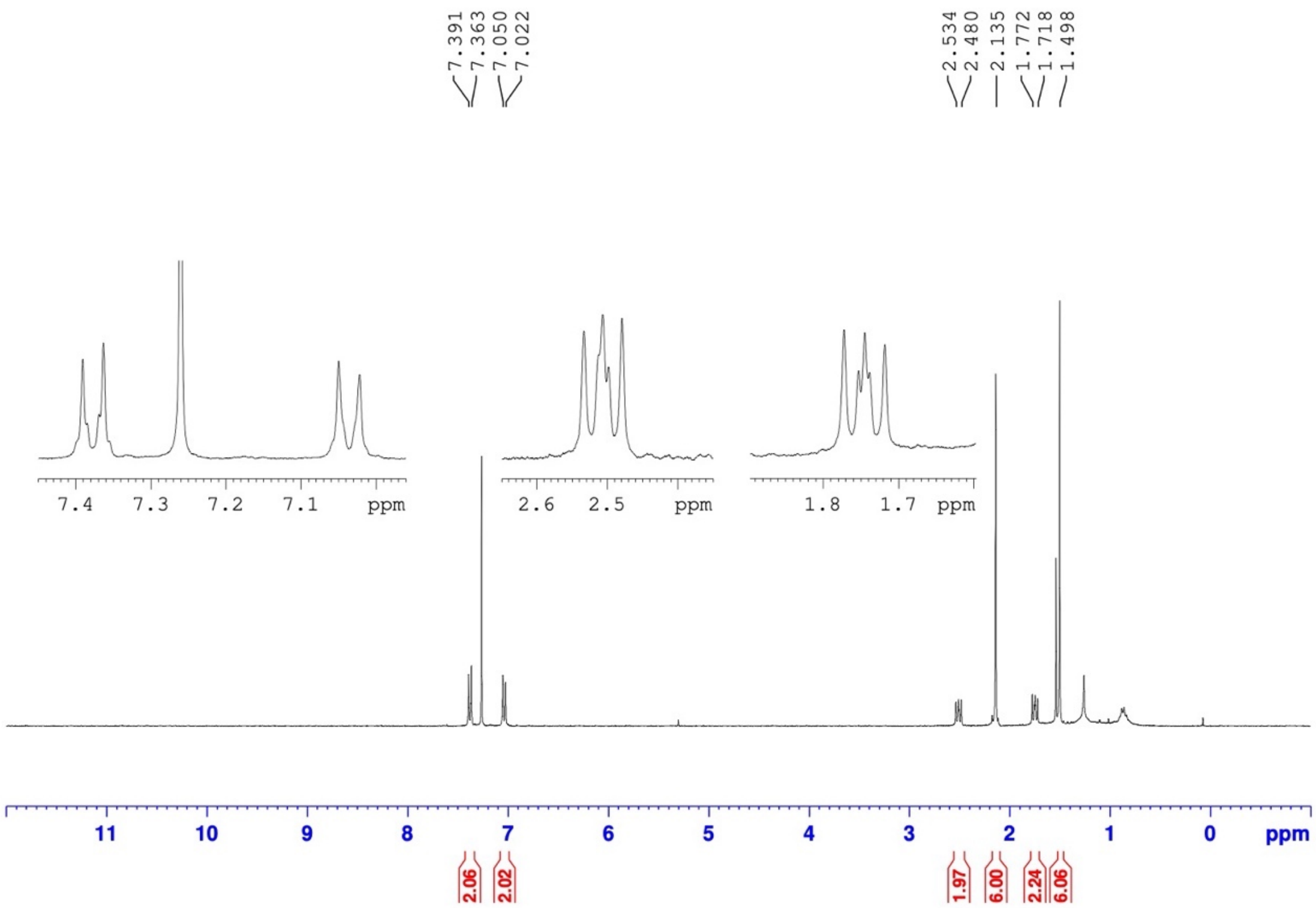

Figure S303.^{1}H NMR spectrum of (3'-(4-bromophenethyl)-[1,1'-bi(bicyclo[1.1.1]pentan)]-3-yl)pentafluoro-λ^6-sulfane **(70)** in $CDCl_3$.

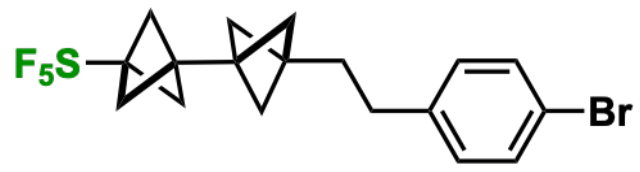

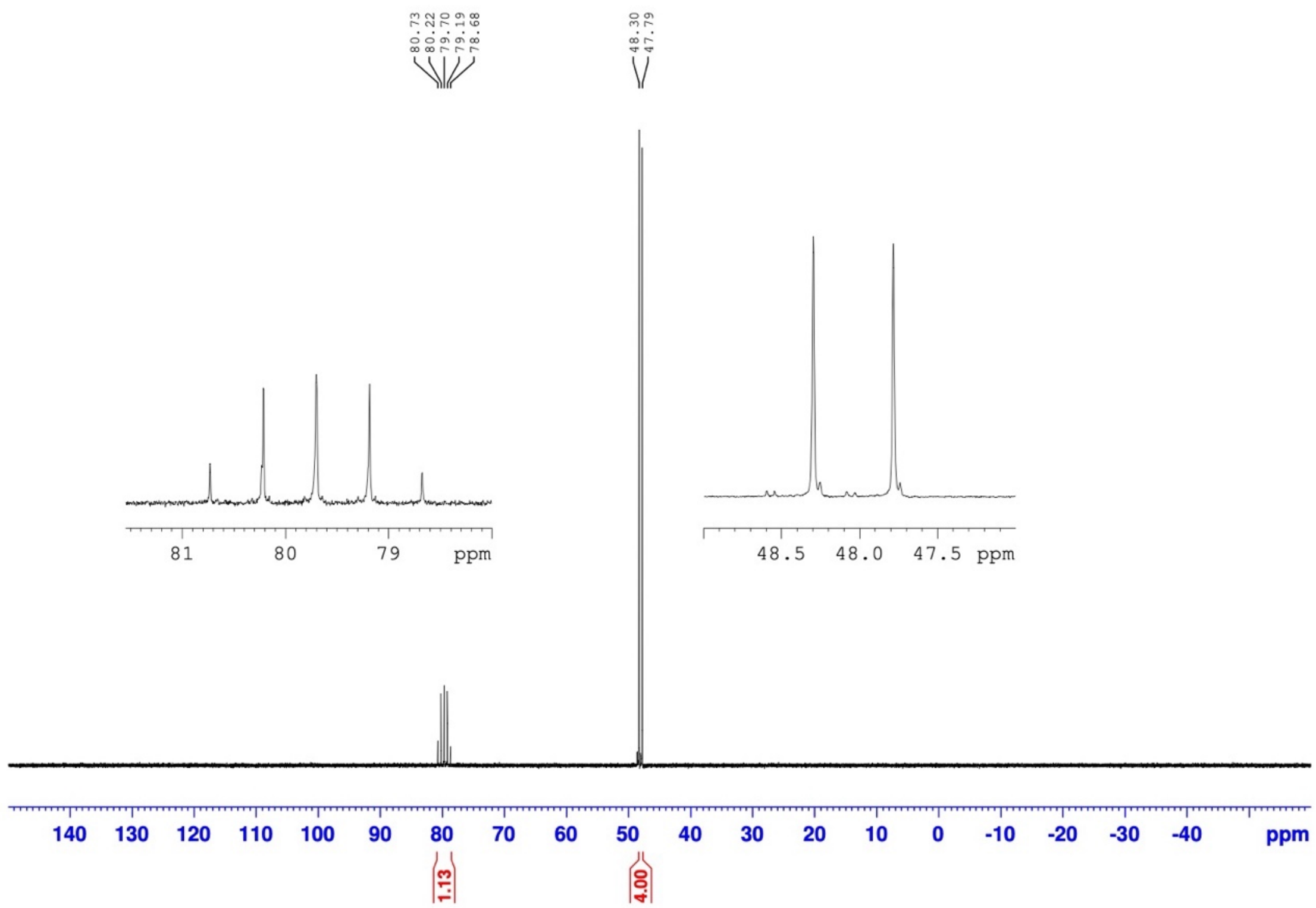

Figure S304. ^{19}F NMR spectrum of (3'-(4-bromophenethyl)-[1,1'-bi(bicyclo[1.1.1]pentan)]-3-yl)pentafluoro-λ^6-sulfane **(70)** in $CDCl_3$.

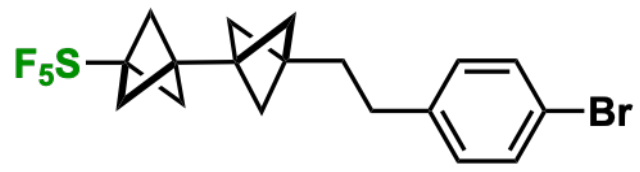

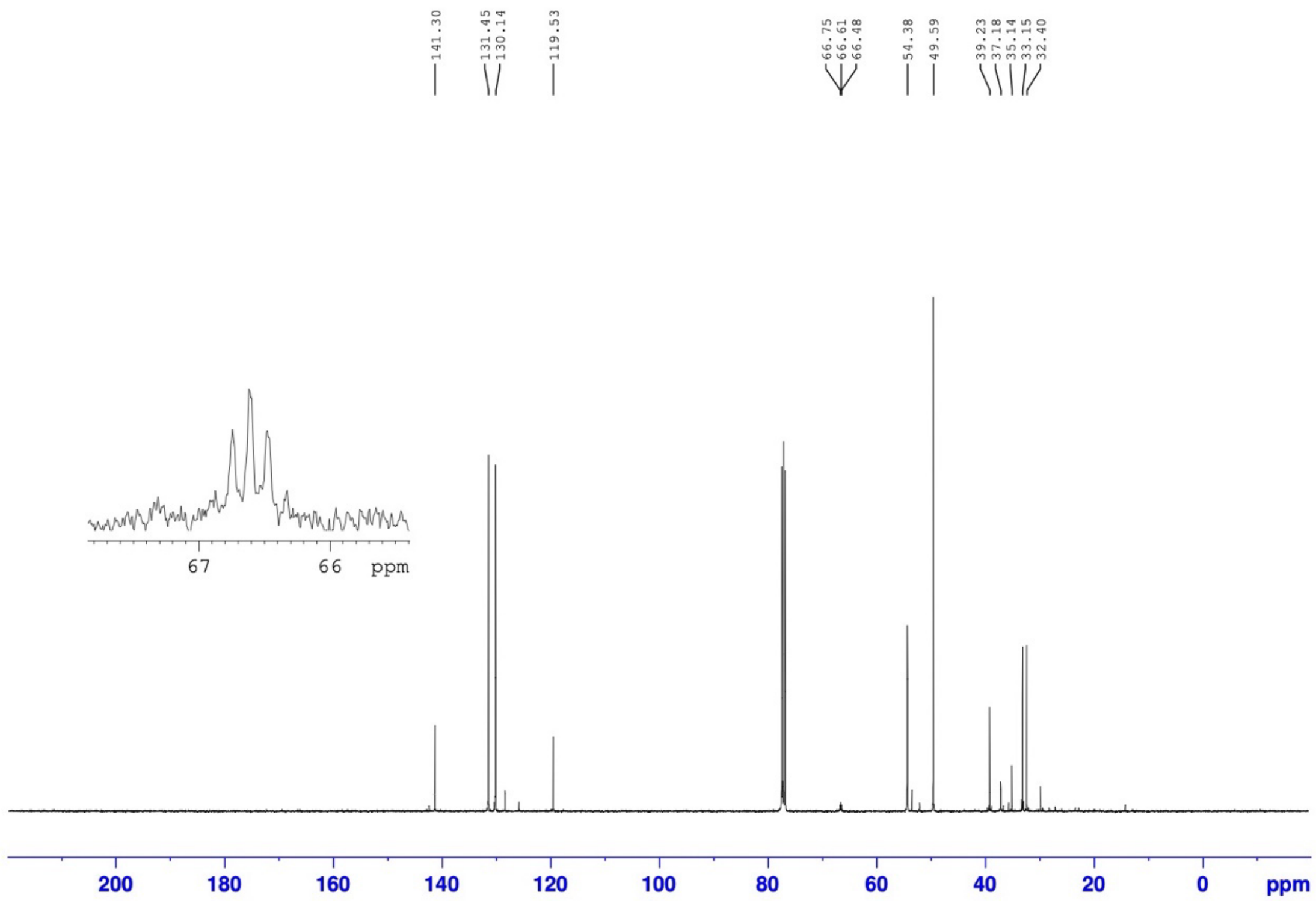

Figure S305. $^{13}C\{^{1}H\}$ NMR spectrum of (3'-(4-bromophenethyl)-[1,1'-bi(bicyclo[1.1.1]pentan)]-3-yl)pentafluoro-λ^6-sulfane **(70)** in $CDCl_3$.

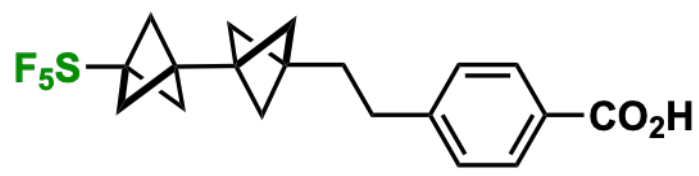

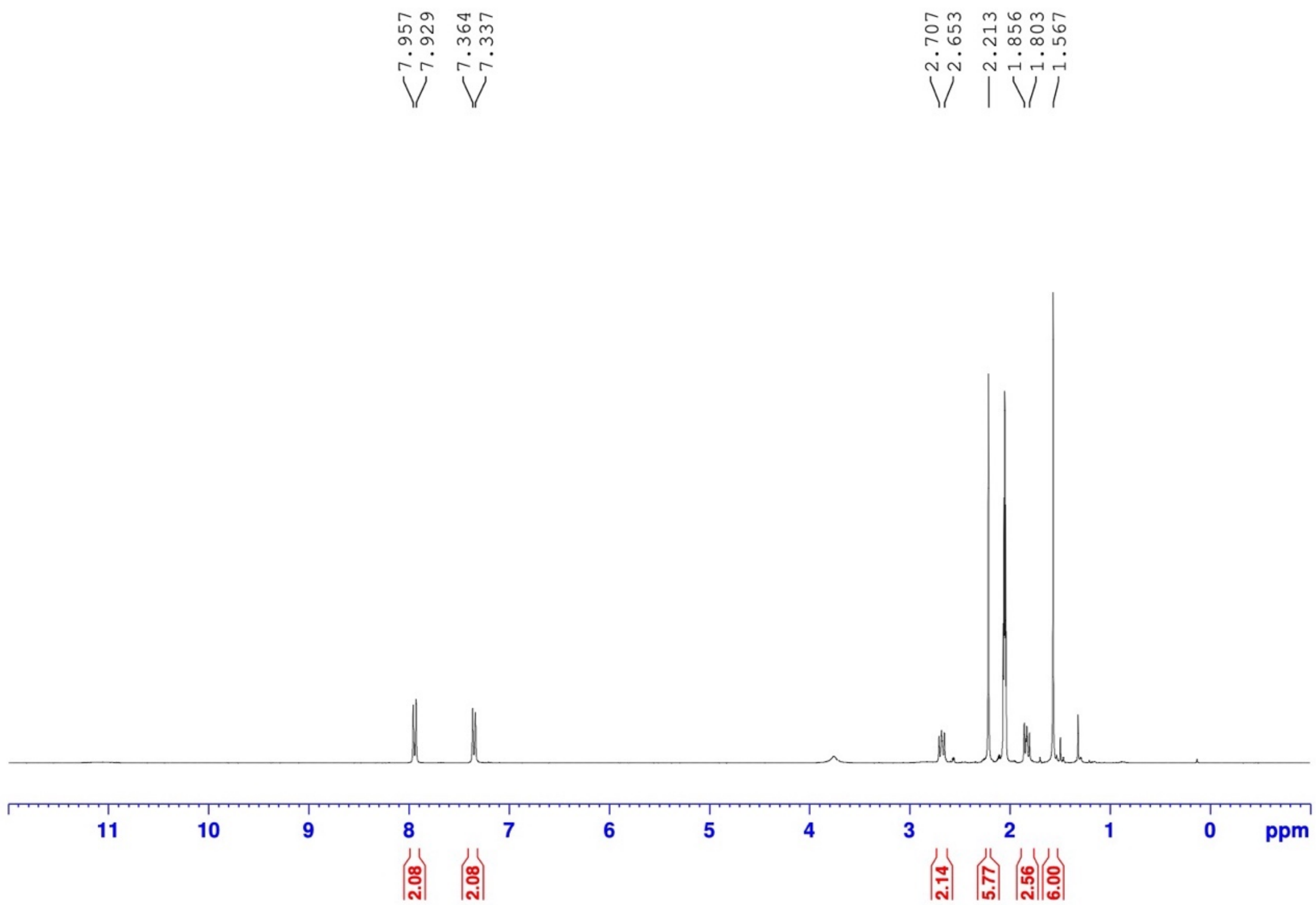

Figure S306. ^{1}H NMR spectrum of 4-(2-(3'-(pentafluoro-λ^6-sulfanyl)-[1,1'-bi(bicyclo[1.1.1]pentan)]-3-yl)ethyl)benzoic acid **(71)** in Acetone-*d*$_6$.

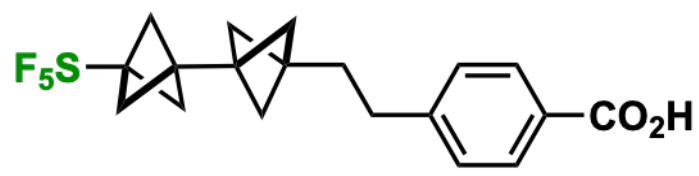

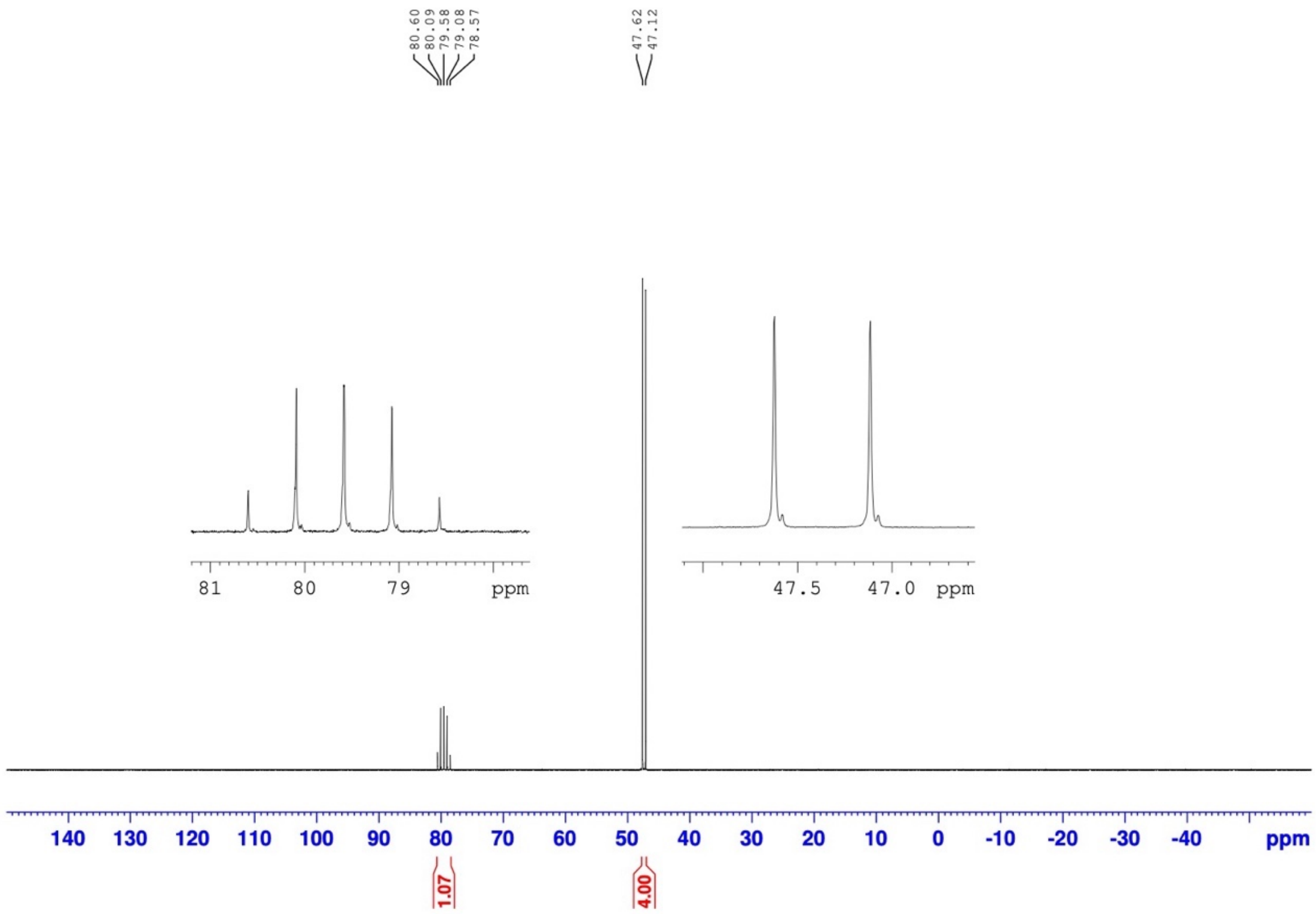

Figure S307.^{19}F NMR spectrum of 4-(2-(3'-(pentafluoro-λ[6]-sulfanyl)-[1,1'-bi(bicyclo[1.1.1]pentan)]-3-yl)ethyl)benzoic acid **(71)** in Acetone-d_6.

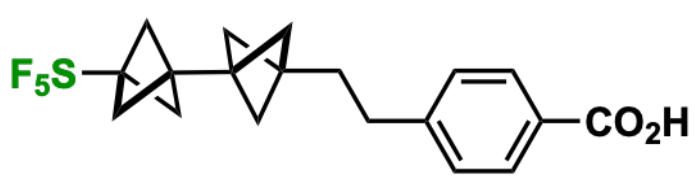

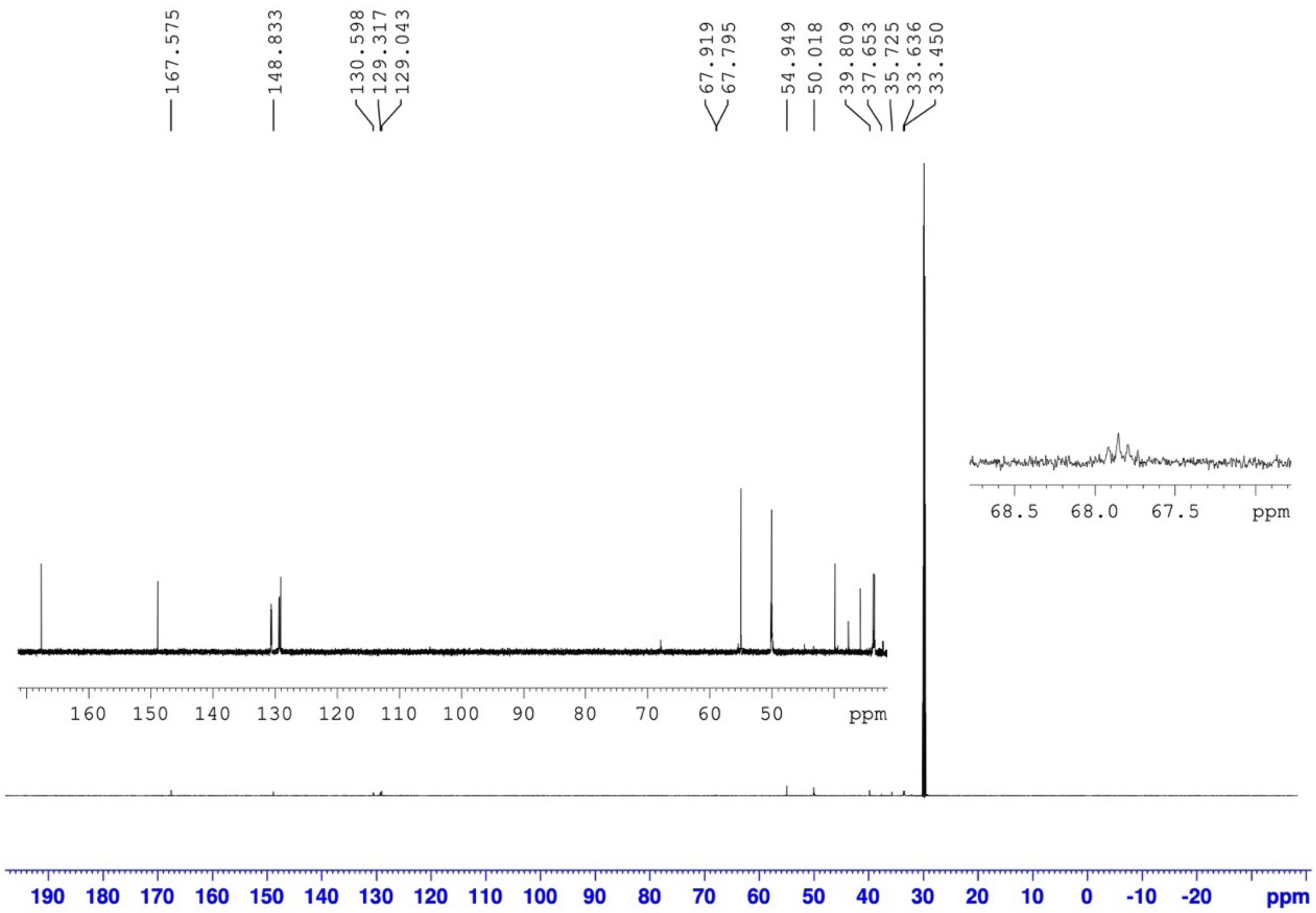

Figure S308. $^{13}C\{^{1}H\}$ NMR spectrum of 4-(2-(3'-(pentafluoro-λ^6-sulfanyl)-[1,1'-bi(bicyclo[1.1.1]pentan)]-3-yl)ethyl)benzoic acid **(71)** in Acetone-*d6.*

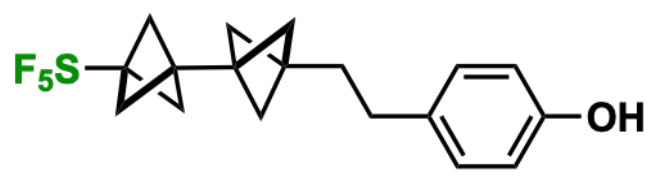

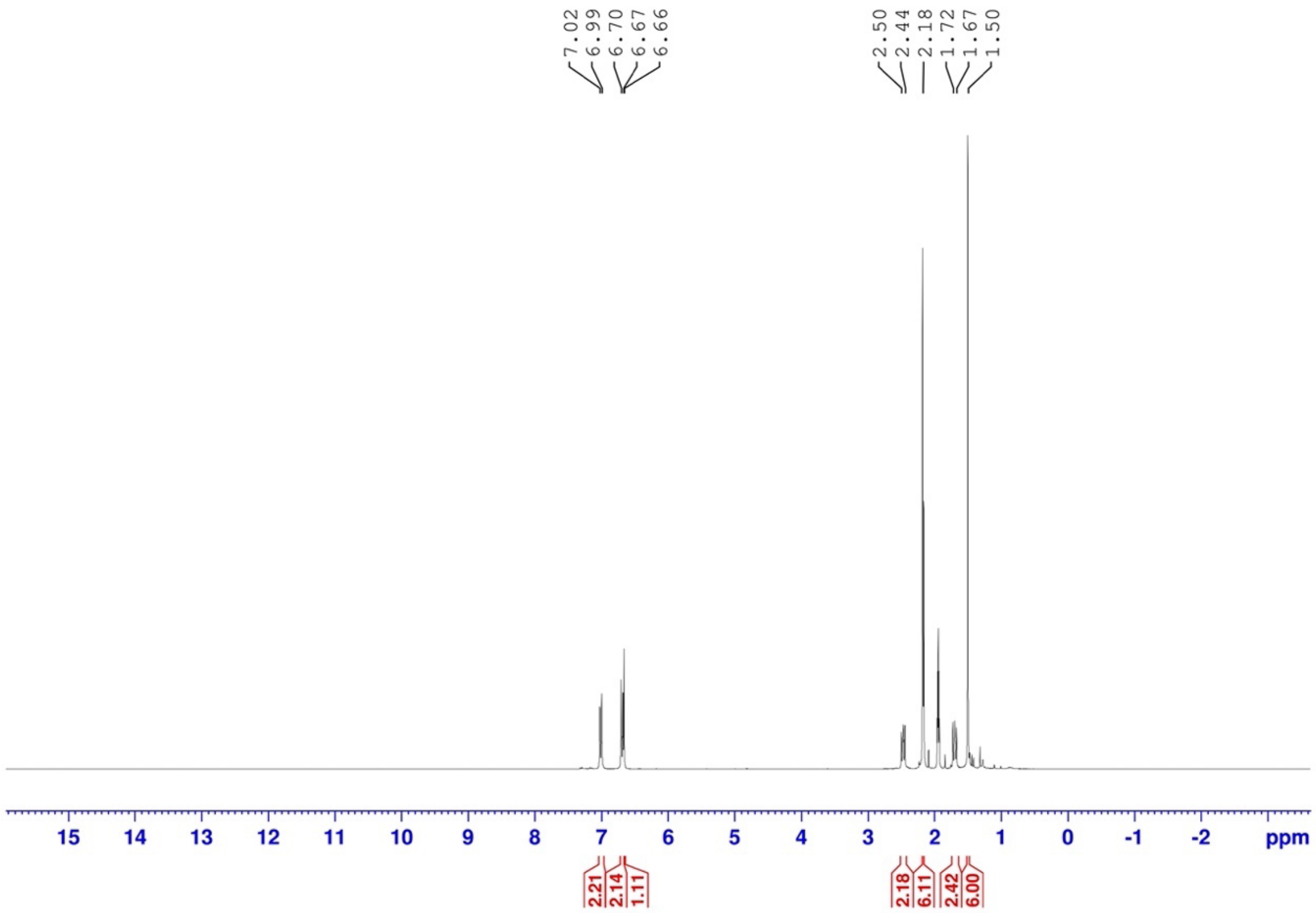

Figure S309. ^{1}H NMR spectrum of 4-(2-(3'-(pentafluoro-λ^6-sulfanyl)-[1,1'-bi(bicyclo[1.1.1]pentan)]-3-yl)ethyl)phenol (**S-31**) in $CDCl_3$.

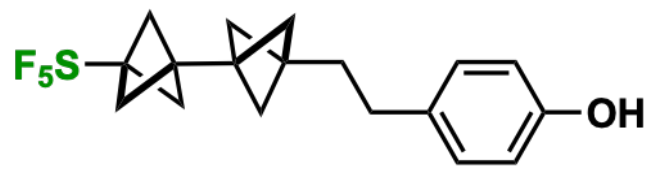

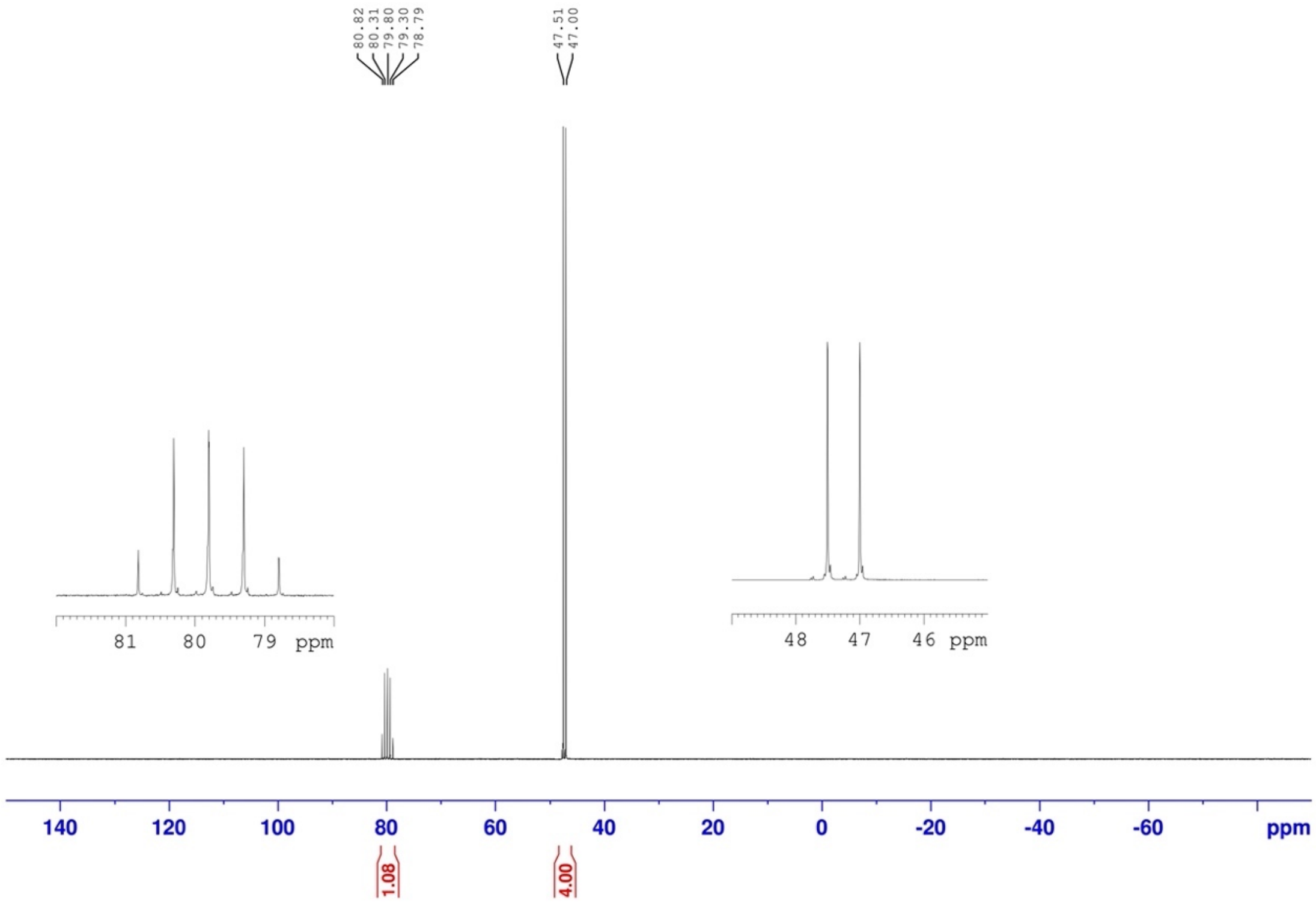

Figure S310. ^{19}F NMR spectrum of 4-(2-(3'-(pentafluoro-λ^6-sulfanyl)-[1,1'-bi(bicyclo[1.1.1]pentan)]-3-yl)ethyl)phenol (**S-31**) in $CDCl_3$.

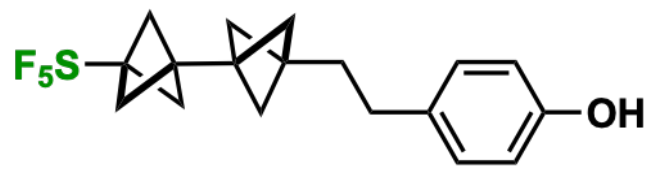

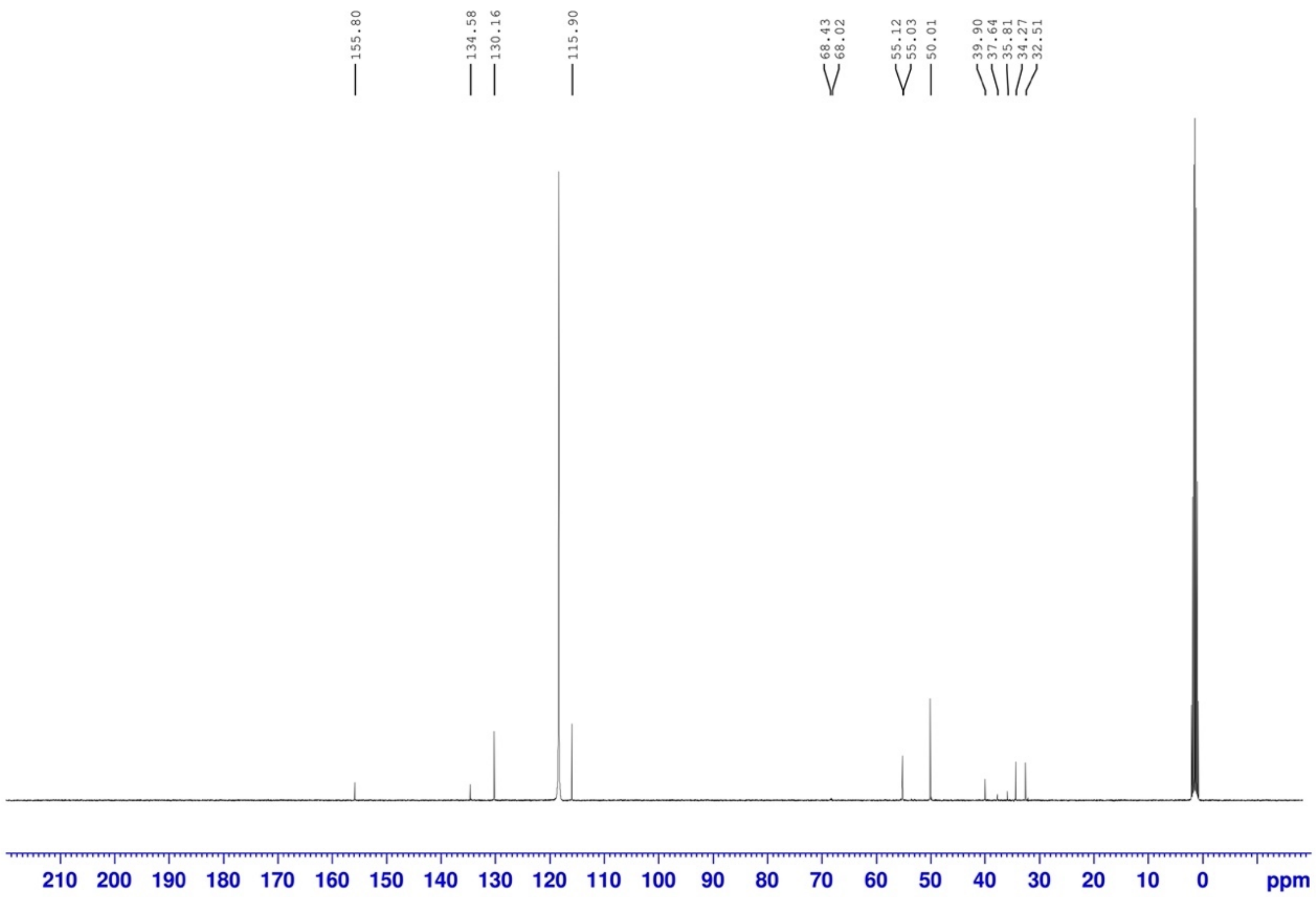

Figure S311. $^{13}C\{^{1}H\}$ NMR spectrum of 4-(2-(3'-(pentafluoro-λ^6-sulfanyl)-[1,1'-bi(bicyclo[1.1.1]pentan)]-3-yl)ethyl)phenol (**S-31**) in $CDCl_3$.

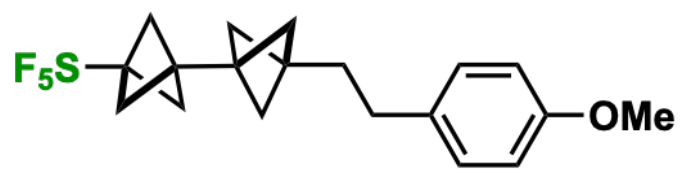

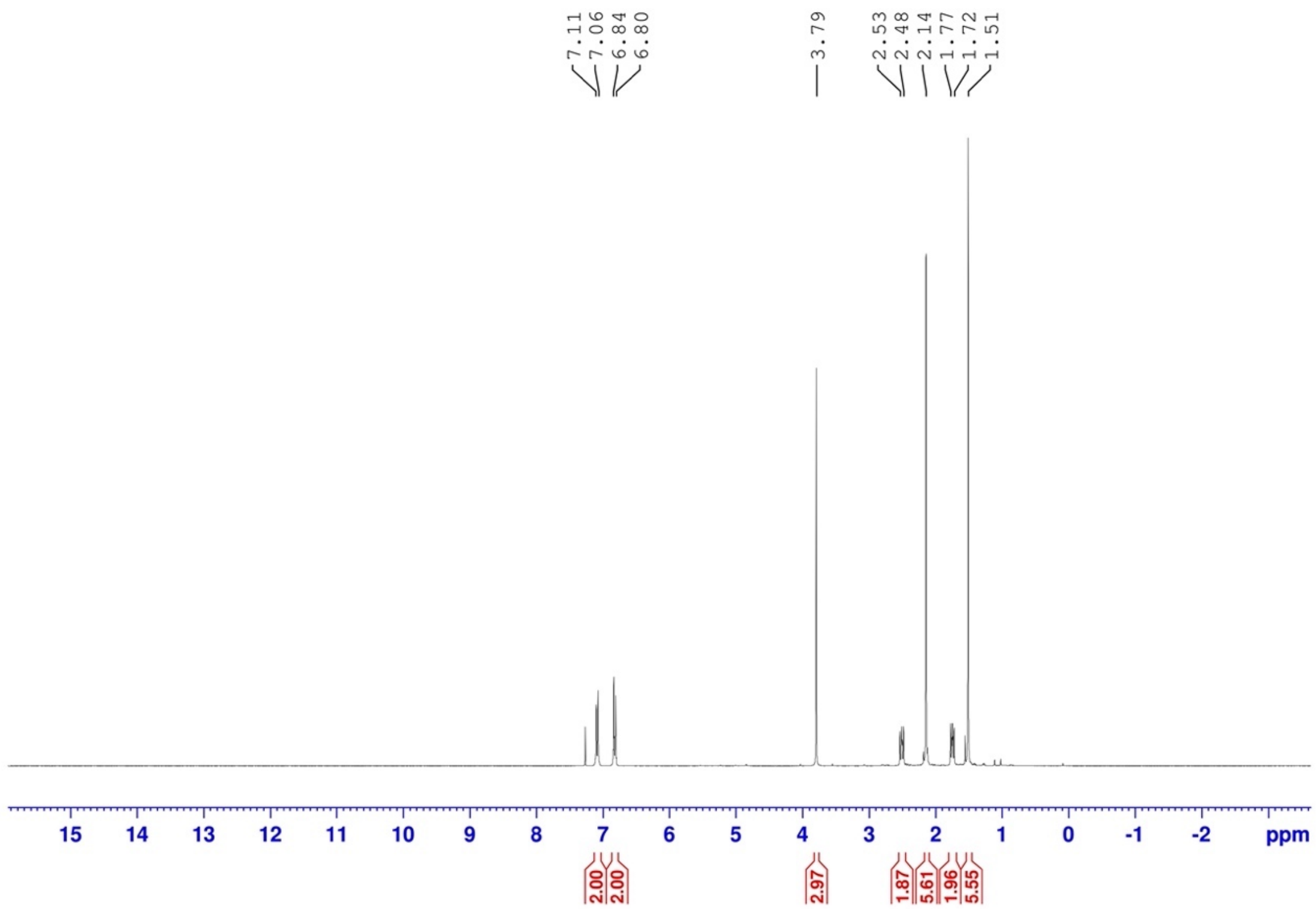

Figure S312. ^{1}H NMR spectrum of pentafluoro(3'-(4-methoxyphenethyl)-[1,1'-bi(bicyclo[1.1.1]pentan)]-3-yl)- λ^6-sulfane (**72**) in $CDCl_3$.

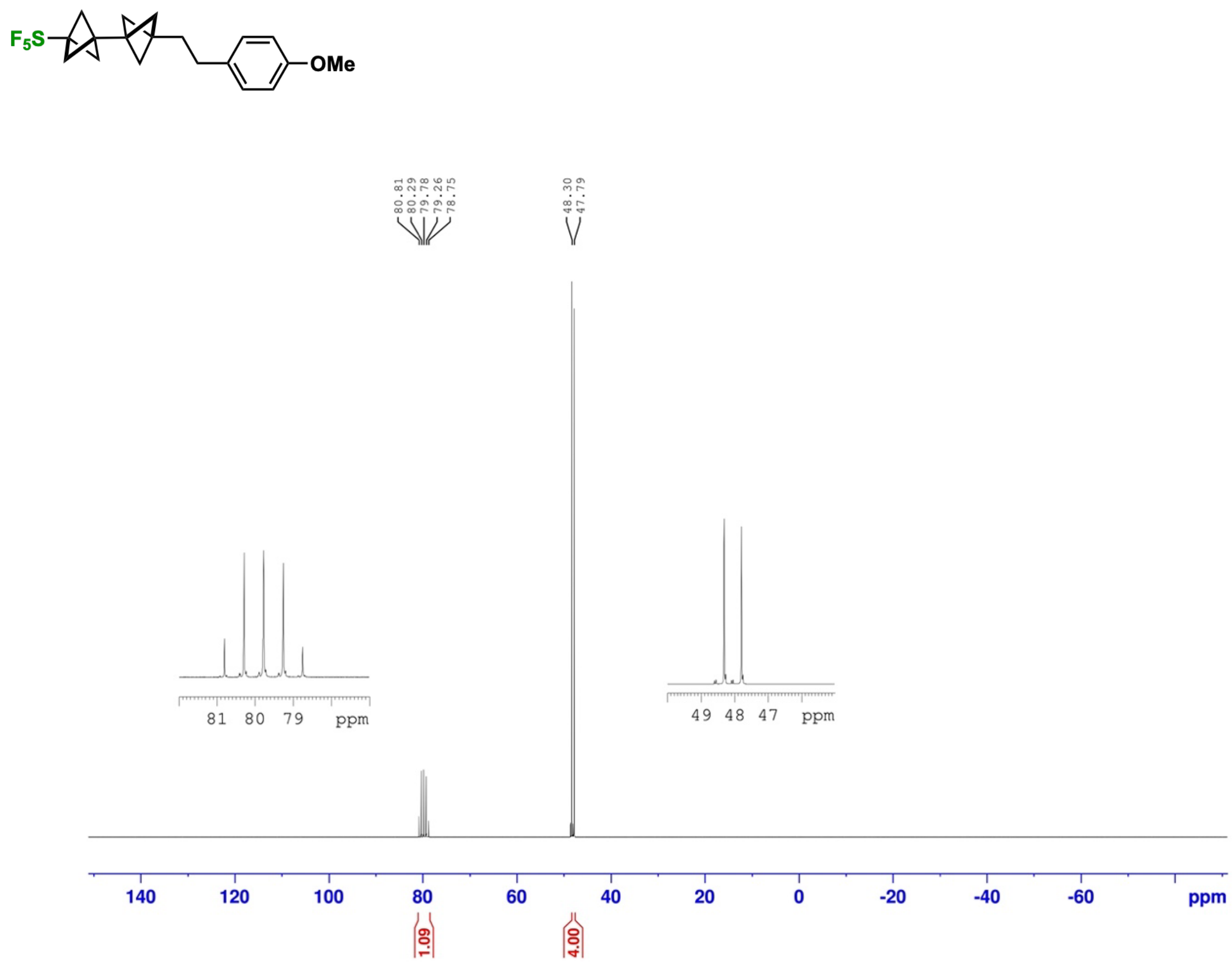

Figure S313. ^{19}F NMR spectrum of pentafluoro(3'-(4-methoxyphenethyl)-[1,1'-bi(bicyclo[1.1.1]pentan)]-3-yl)- λ^6-sulfane (**72**) in $CDCl_3$.

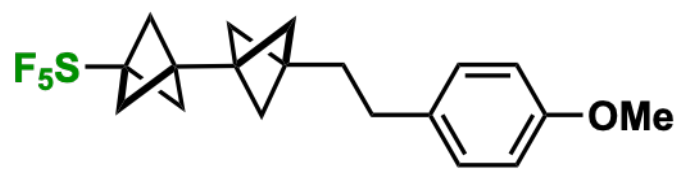

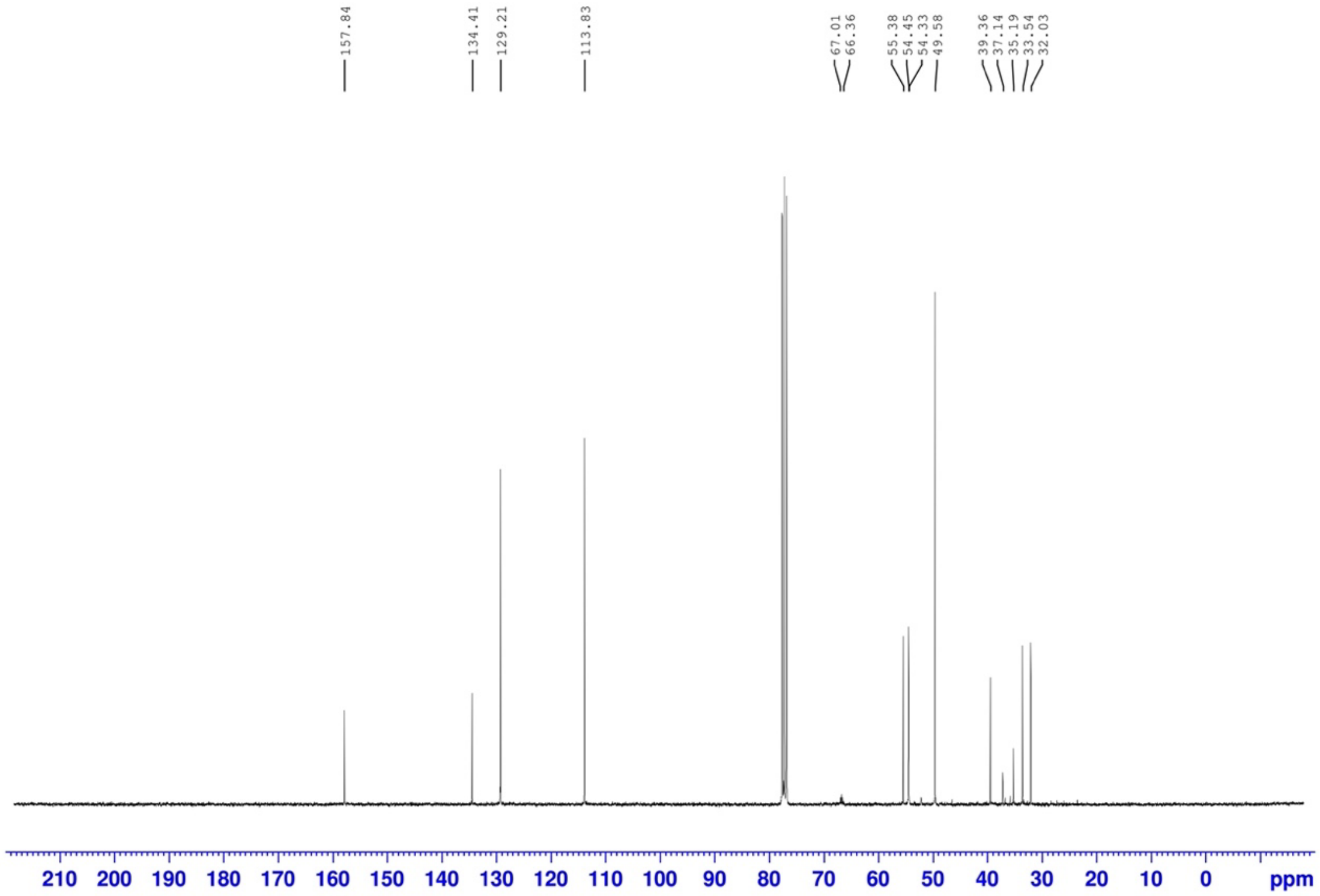

Figure S314. $^{13}C\{^1H\}$ NMR spectrum of pentafluoro(3'-(4-methoxyphenethyl)-[1,1'-bi(bicyclo[1.1.1]pentan)]-3-yl)- λ^6-sulfane (**72**) in $CDCl_3$.

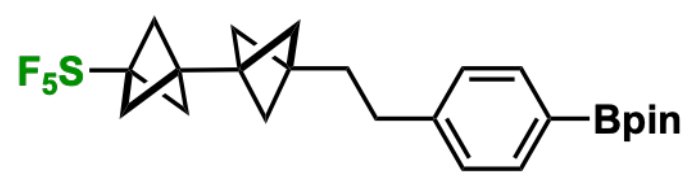

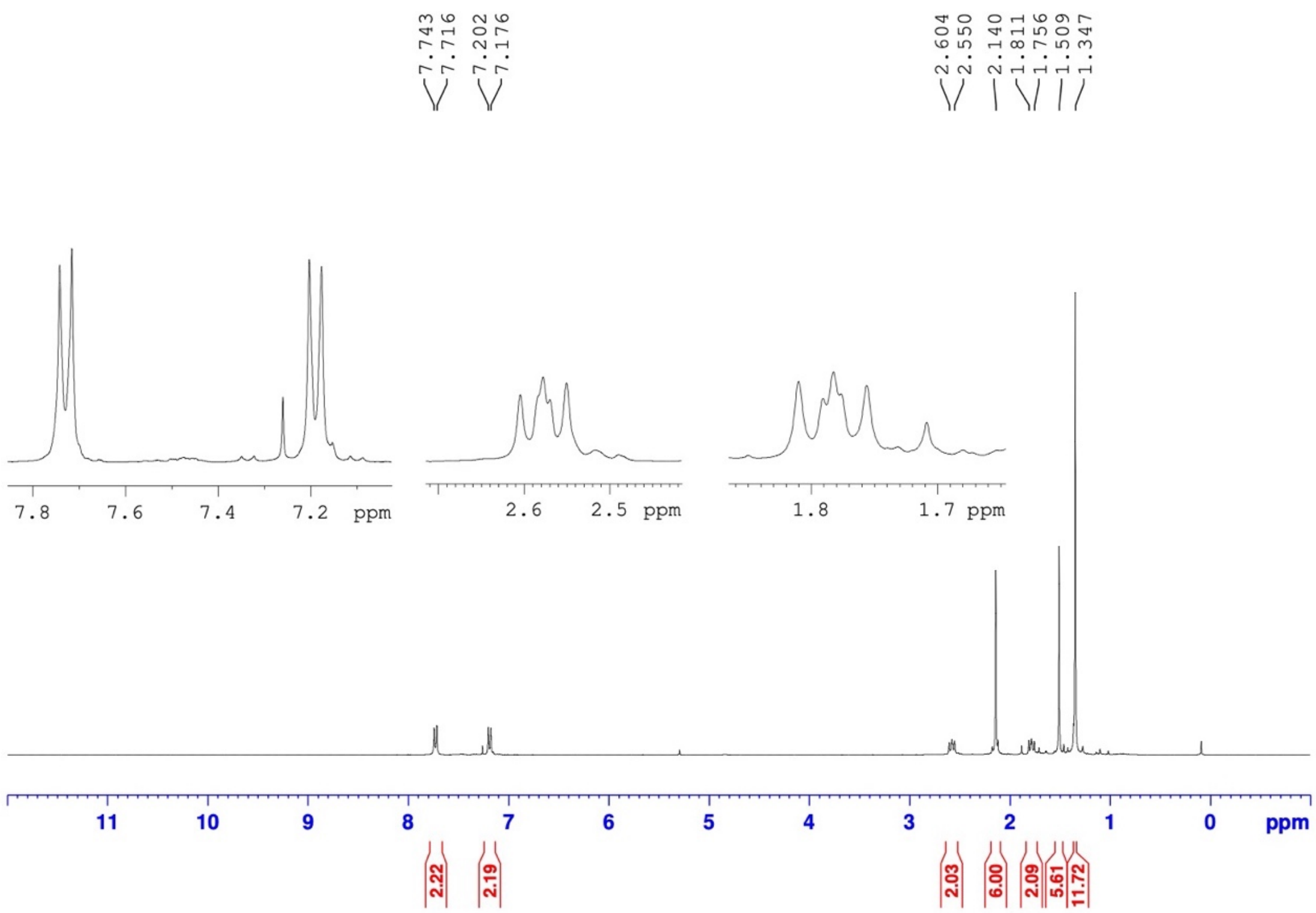

Figure S315. ^{1}H NMR spectrum of 4,4,5,5-tetramethyl-2-(4-(2-(3'-(pentafluoro-λ^{6}-sulfanyl)-[1,1'-bi(bicyclo[1.1.1]pentan)]-3-yl)ethyl)phenyl)-1,3,2-dioxaborolane (**S-32**) in $CDCl_3$.

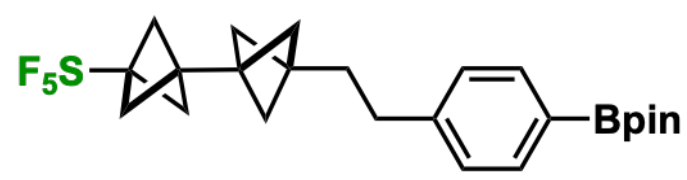

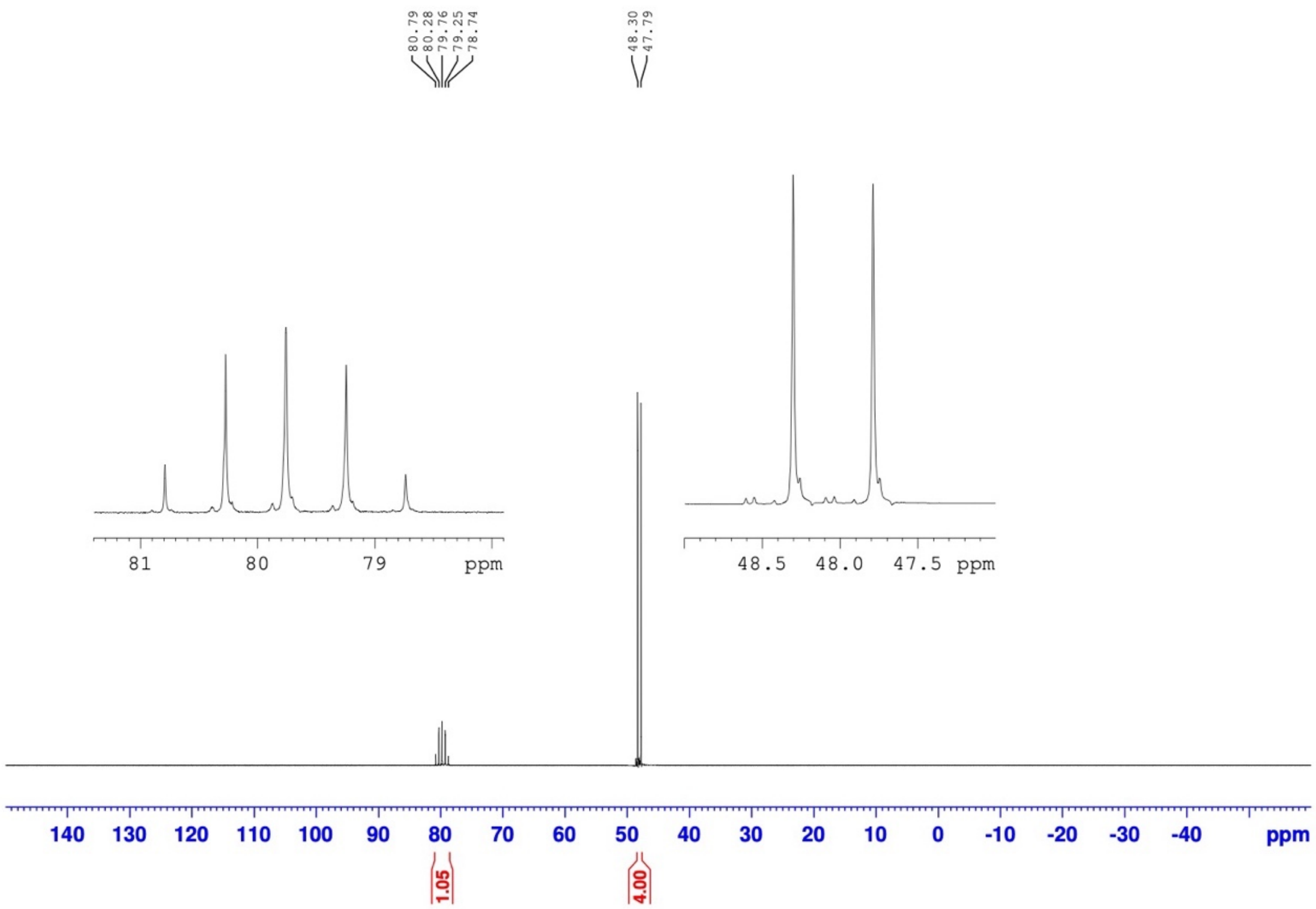

Figure S316. ^{19}F NMR spectrum of 4,4,5,5-tetramethyl-2-(4-(2-(3'-(pentafluoro-λ^6-sulfanyl)-[1,1'-bi(bicyclo[1.1.1]pentan)]-3-yl)ethyl)phenyl)-1,3,2-dioxaborolane (**S-32**) in $CDCl_3$.

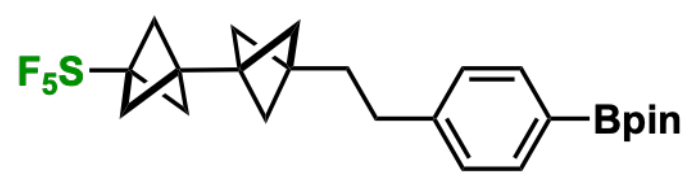

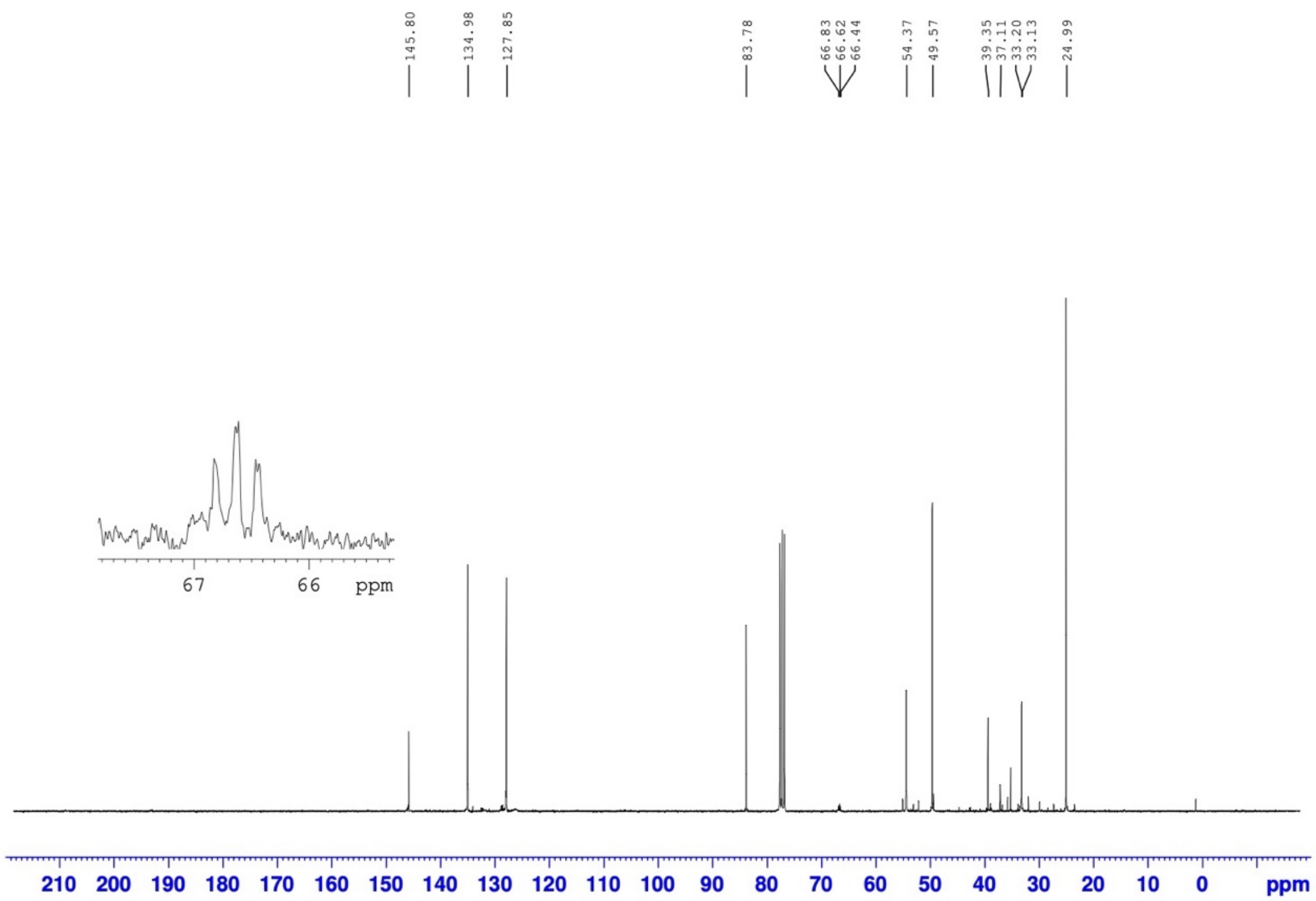

Figure S317. $^{13}C\{^{1}H\}$ NMR spectrum of 4,4,5,5-tetramethyl-2-(4-(2-(3'-(pentafluoro-λ^6-sulfanyl)-[1,1'-bi(bicyclo[1.1.1]pentan)]-3-yl)ethyl)phenyl)-1,3,2-dioxaborolane (**S-32**) in $CDCl_3$.

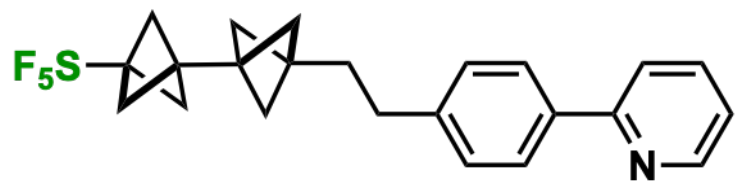

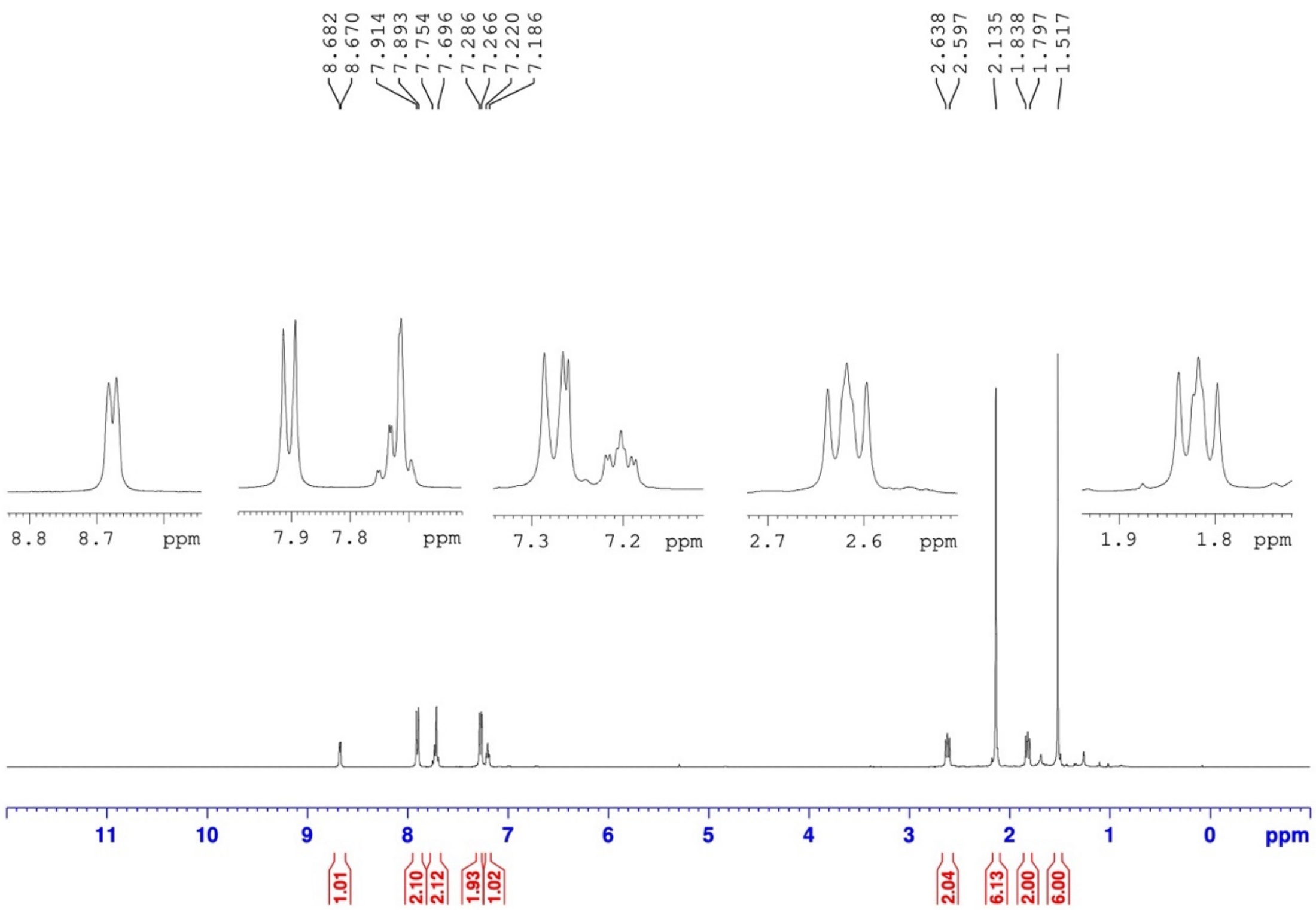

Figure S318. ^{1}H NMR spectrum of 2-(4-(2-(3'-(pentafluoro-λ^6-sulfanyl)-[1,1'-bi(bicyclo[1.1.1]pentan)]-3-yl)ethyl)phenyl)pyridine (**73**) in $CDCl_3$.

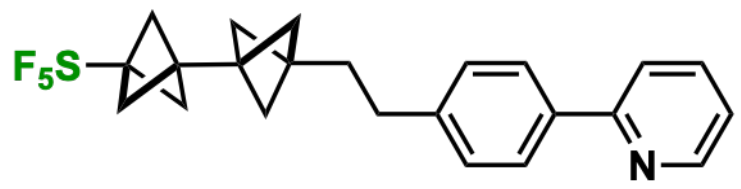

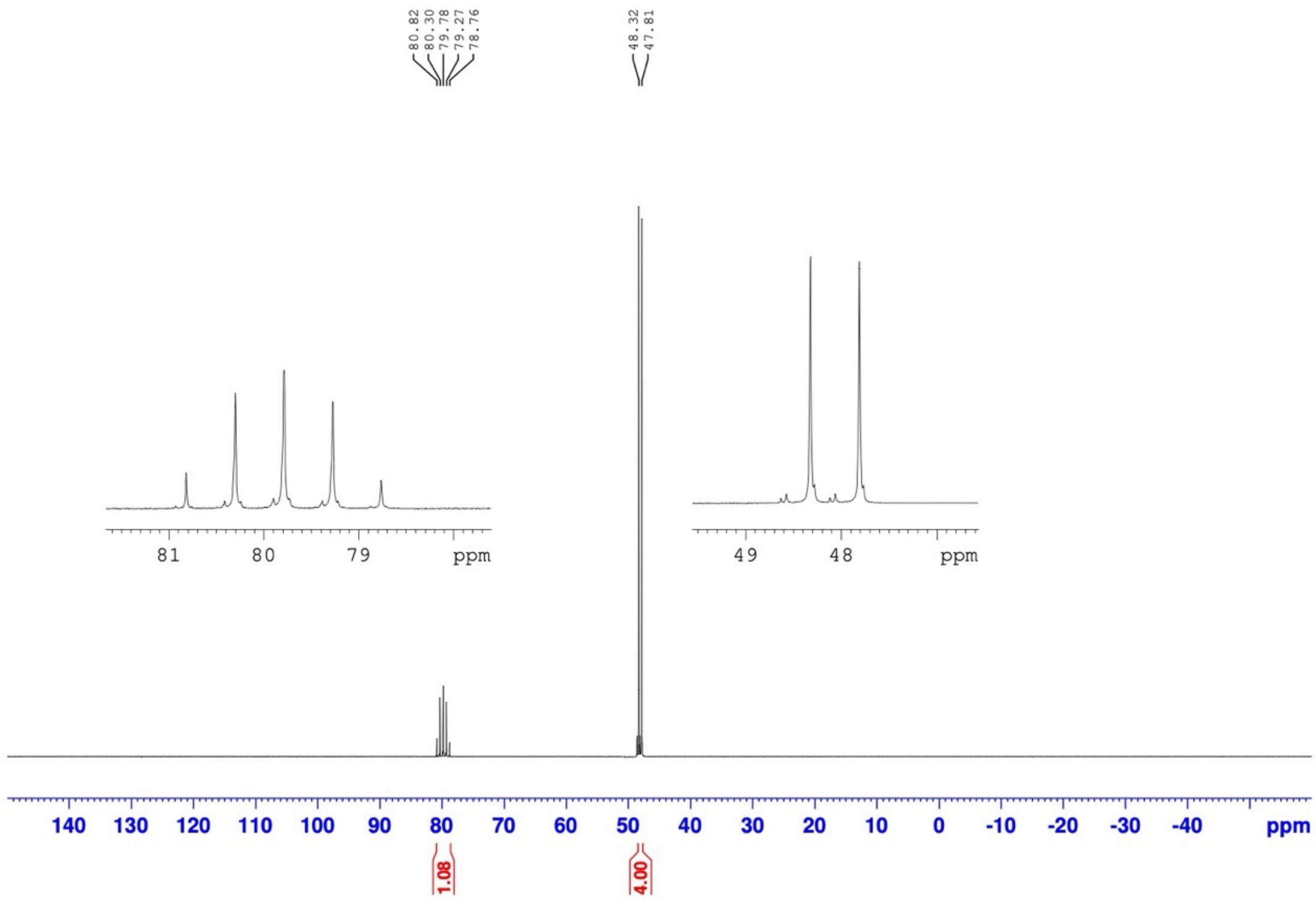

Figure S319. ^{19}F NMR spectrum of 2-(4-(2-(3'-(pentafluoro-λ^6-sulfanyl)-[1,1'-bi(bicyclo[1.1.1]pentan)]-3-yl)ethyl)phenyl)pyridine (**73**) in $CDCl_3$.

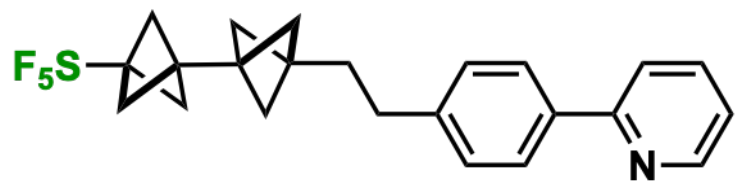

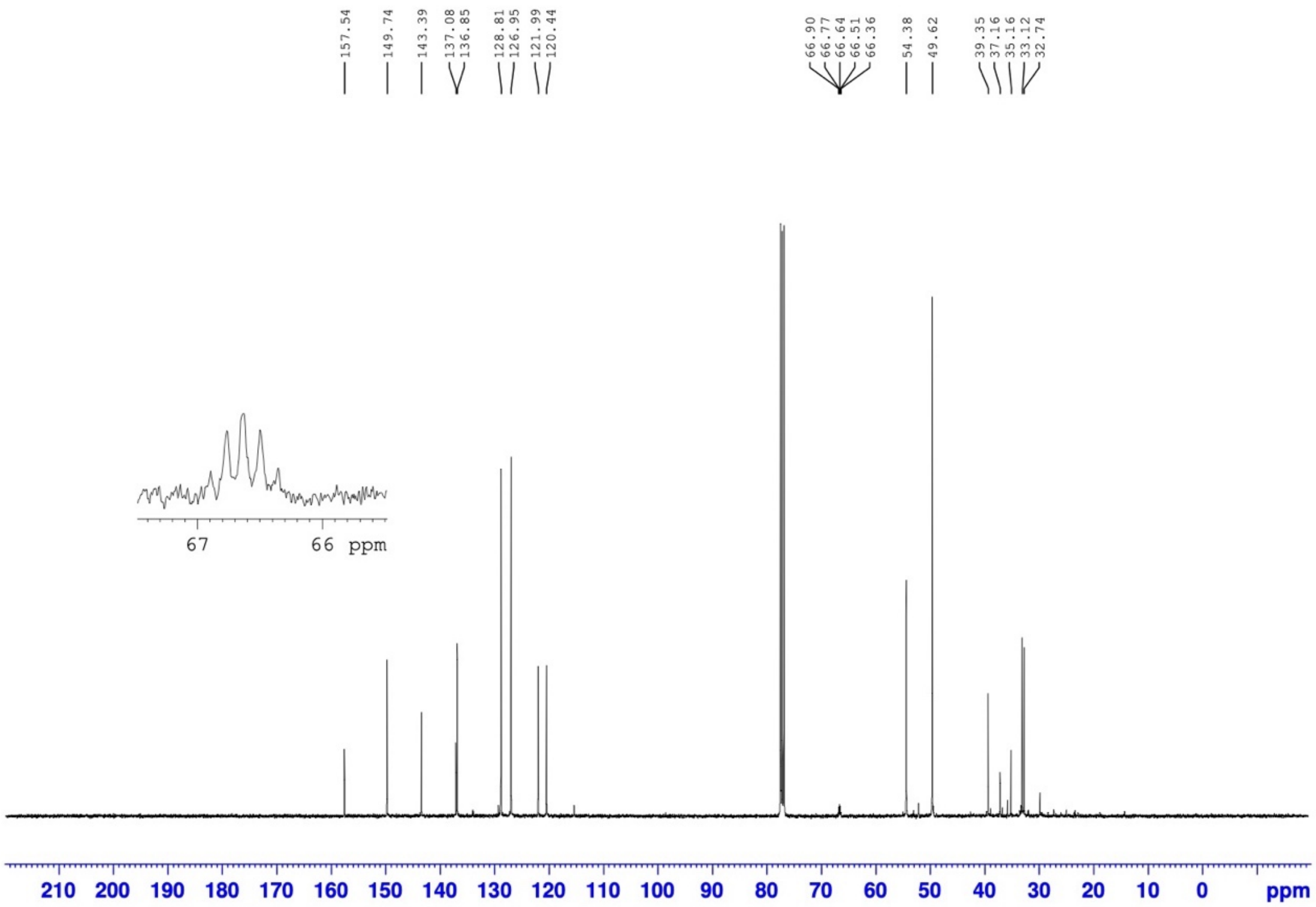

Figure S320. $^{13}C\{^1H\}$ NMR spectrum of 2-(4-(2-(3'-(pentafluoro-λ^6-sulfanyl)-[1,1'-bi(bicyclo[1.1.1]pentan)]-3-yl)ethyl)phenyl)pyridine (**73**) in $CDCl_3$.

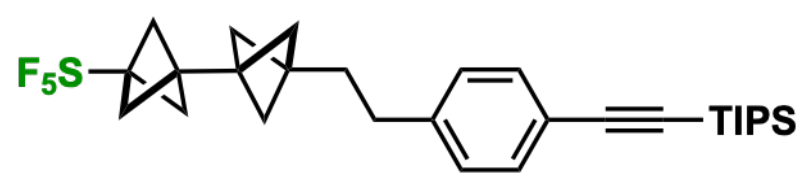

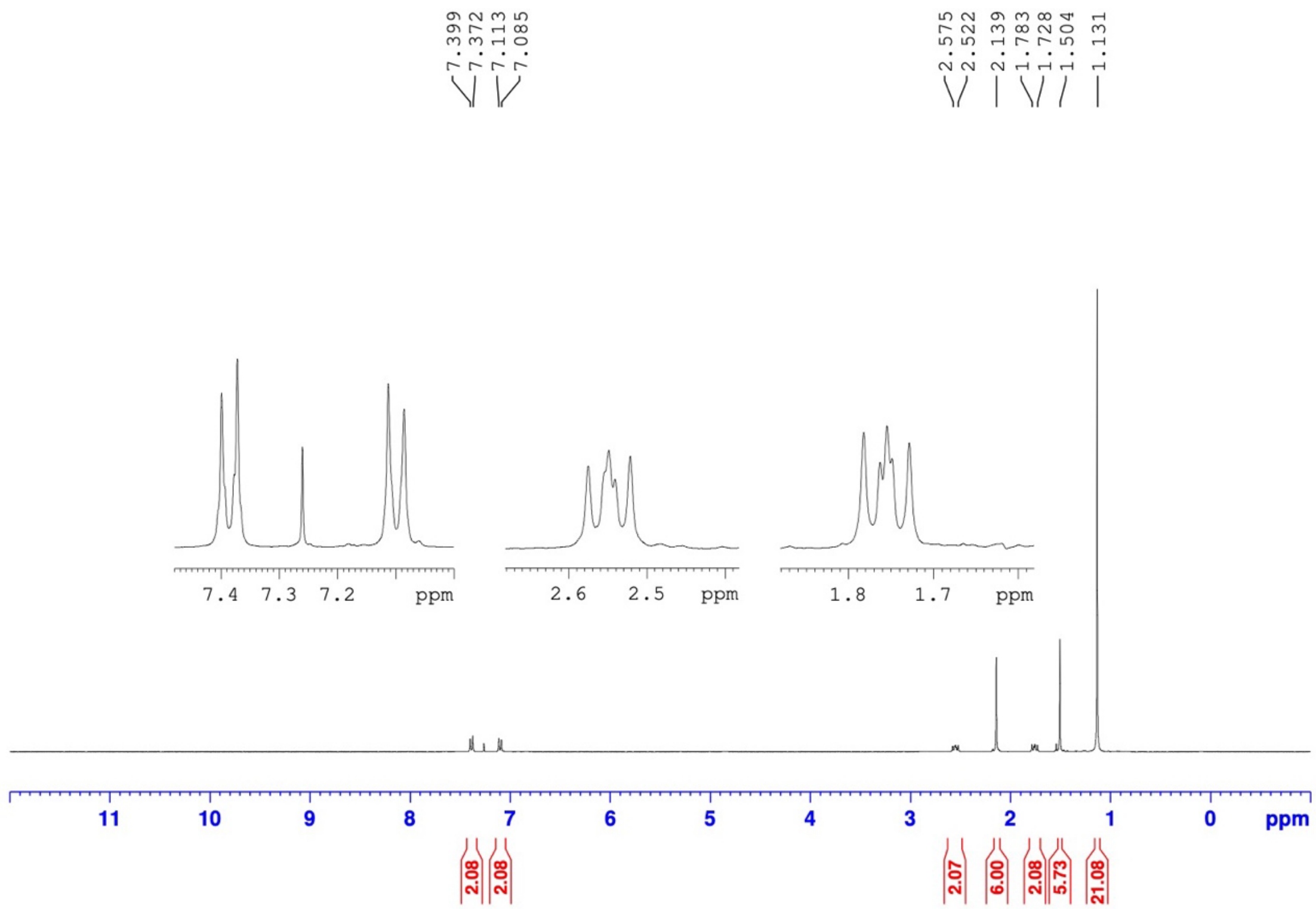

Figure S321. ^{1}H NMR spectrum of triisopropyl((4-(2-(3'-(pentafluoro-λ^6-sulfanyl)-[1,1'-bi(bicyclo[1.1.1]pentan)]-3-yl)ethyl)phenyl)ethynyl)silane (**74**) in $CDCl_3$.

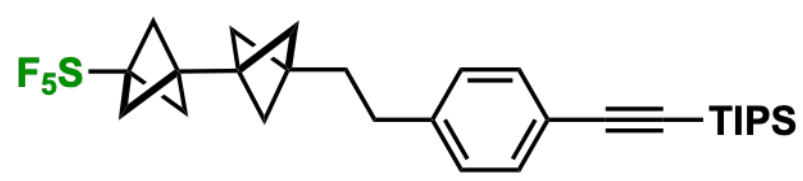

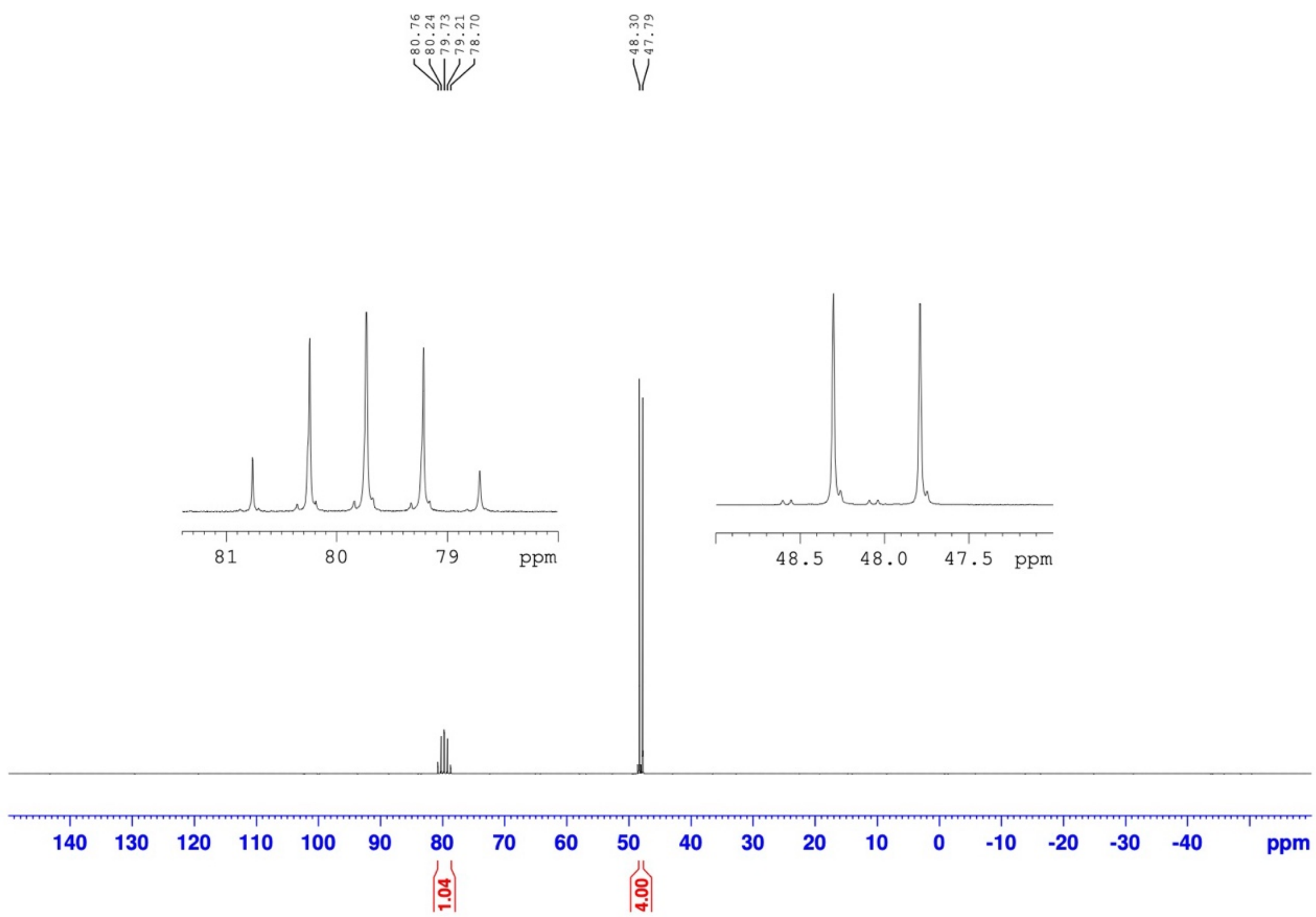

Figure S322. ^{19}F NMR spectrum of triisopropyl((4-(2-(3'-(pentafluoro-λ^{6}-sulfanyl)-[1,1'-bi(bicyclo[1.1.1]pentan)]-3-yl)ethyl)phenyl)ethynyl)silane (**74**) in $CDCl_3$.

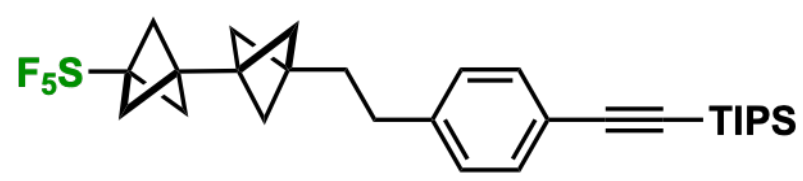

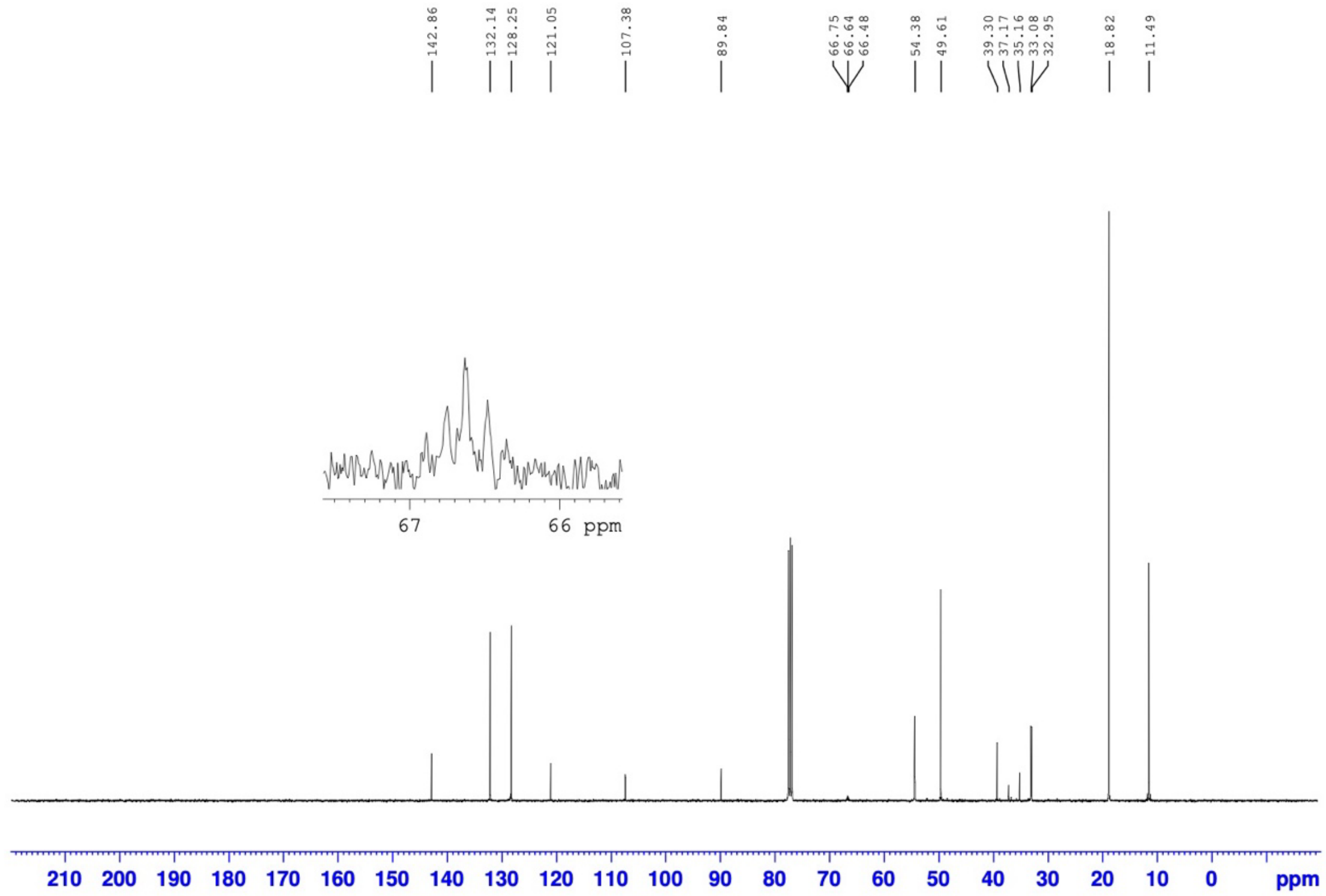

Figure S323. $^{13}C\{^{1}H\}$ NMR spectrum of triisopropyl((4-(2-(3'-(pentafluoro-λ^6-sulfanyl)-[1,1'-bi(bicyclo[1.1.1]pentan)]-3-yl)ethyl)phenyl)ethynyl)silane (**74**) in $CDCl_3$.

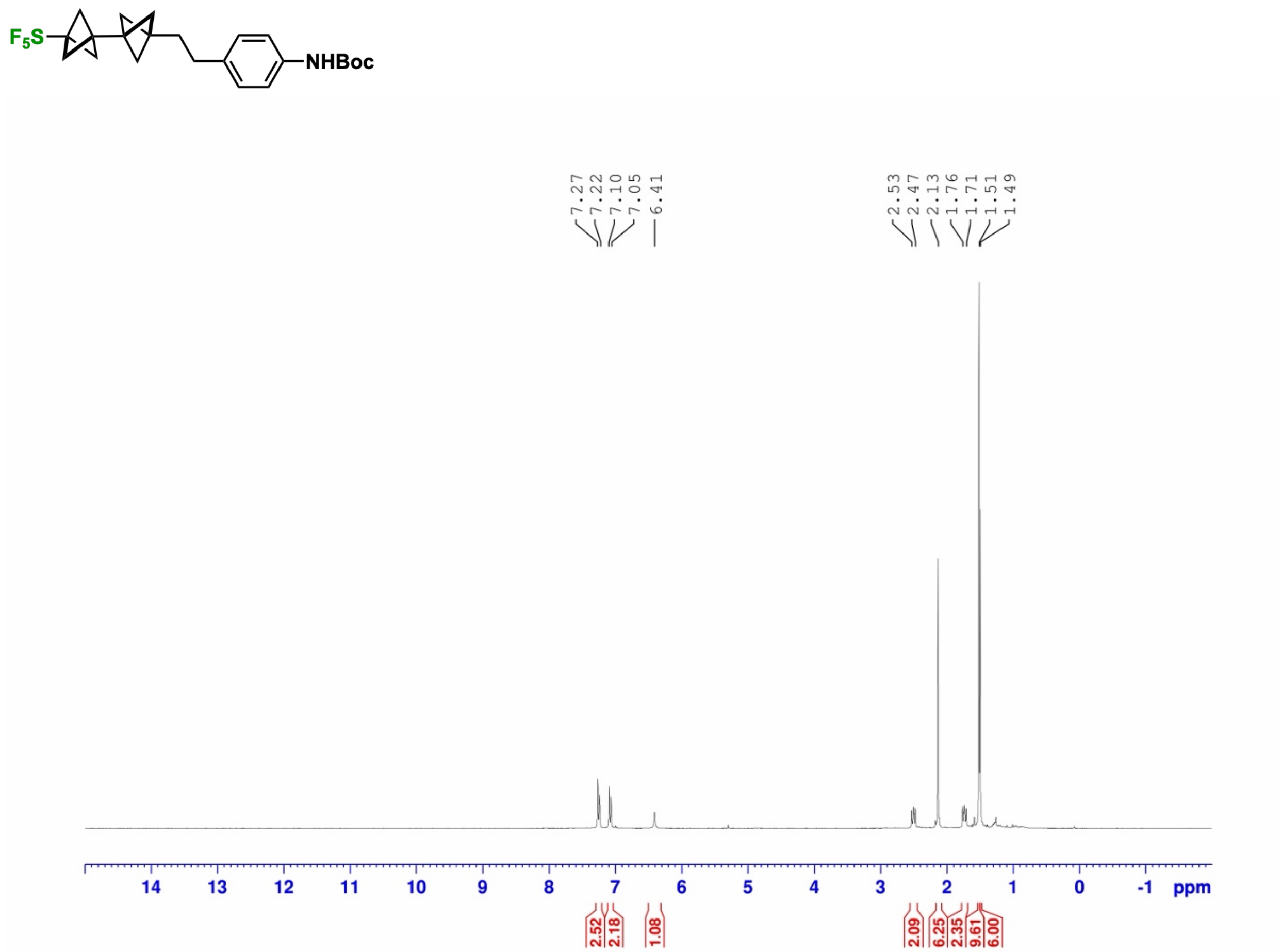

Figure S324. ^{1}H NMR spectrum of *tert*-butyl (4-(2-(3'-(pentafluoro-λ[6]-sulfanyl)-[1,1'-bi(bicyclo[1.1.1]pentan)]-3-yl)ethyl)phenyl)carbamate (**75**) in $CDCl_3$.

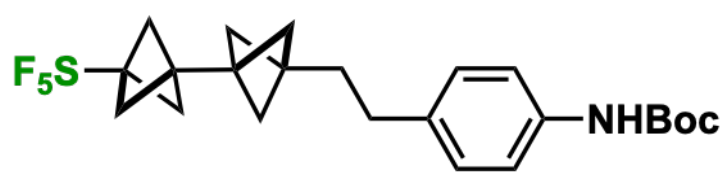

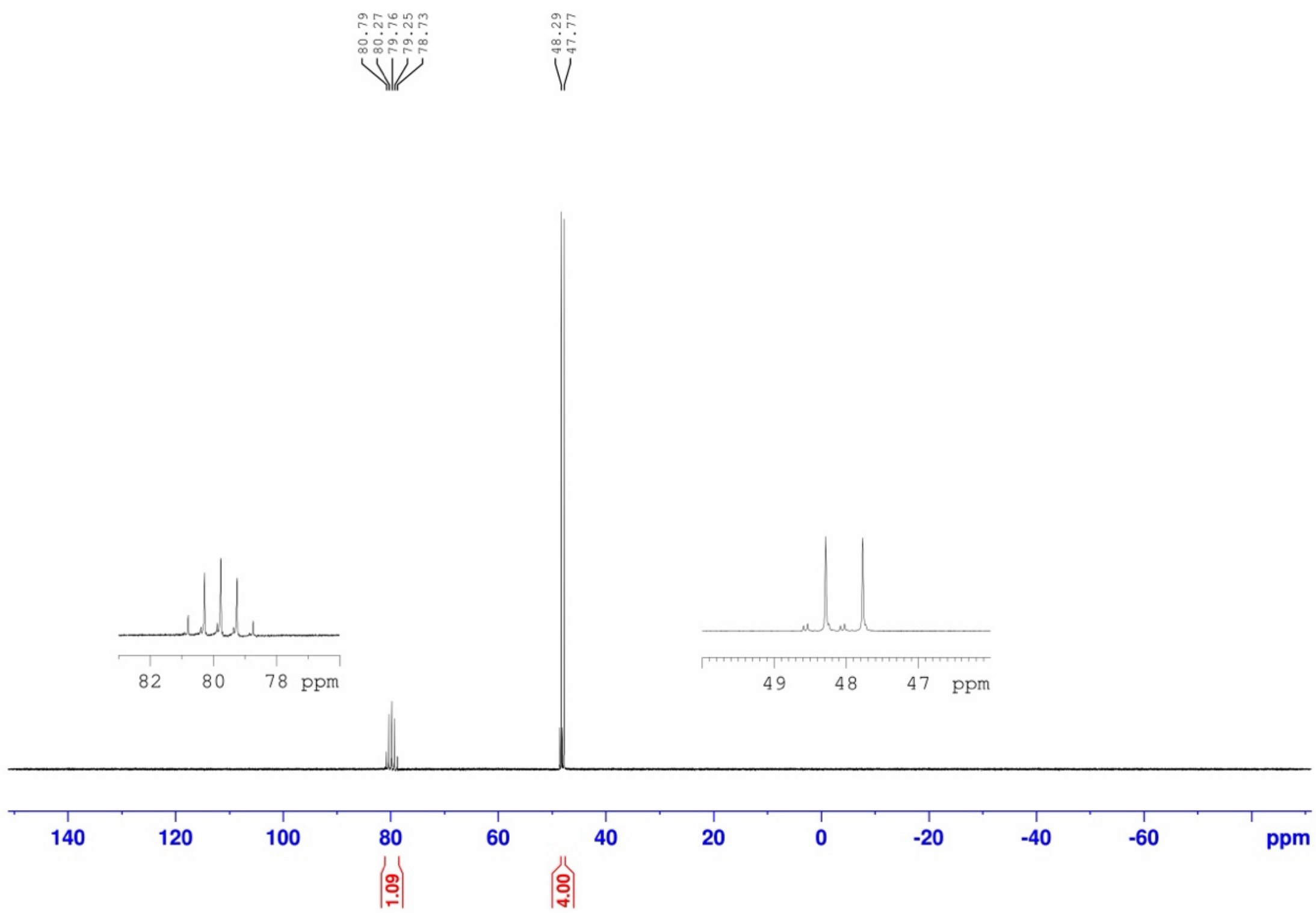

Figure S325. ^{19}F NMR spectrum of *tert*-butyl (4-(2-(3'-(pentafluoro-λ^6-sulfanyl)-[1,1'-bi(bicyclo[1.1.1]pentan)]-3-yl)ethyl)phenyl)carbamate (**75**) in $CDCl_3$.

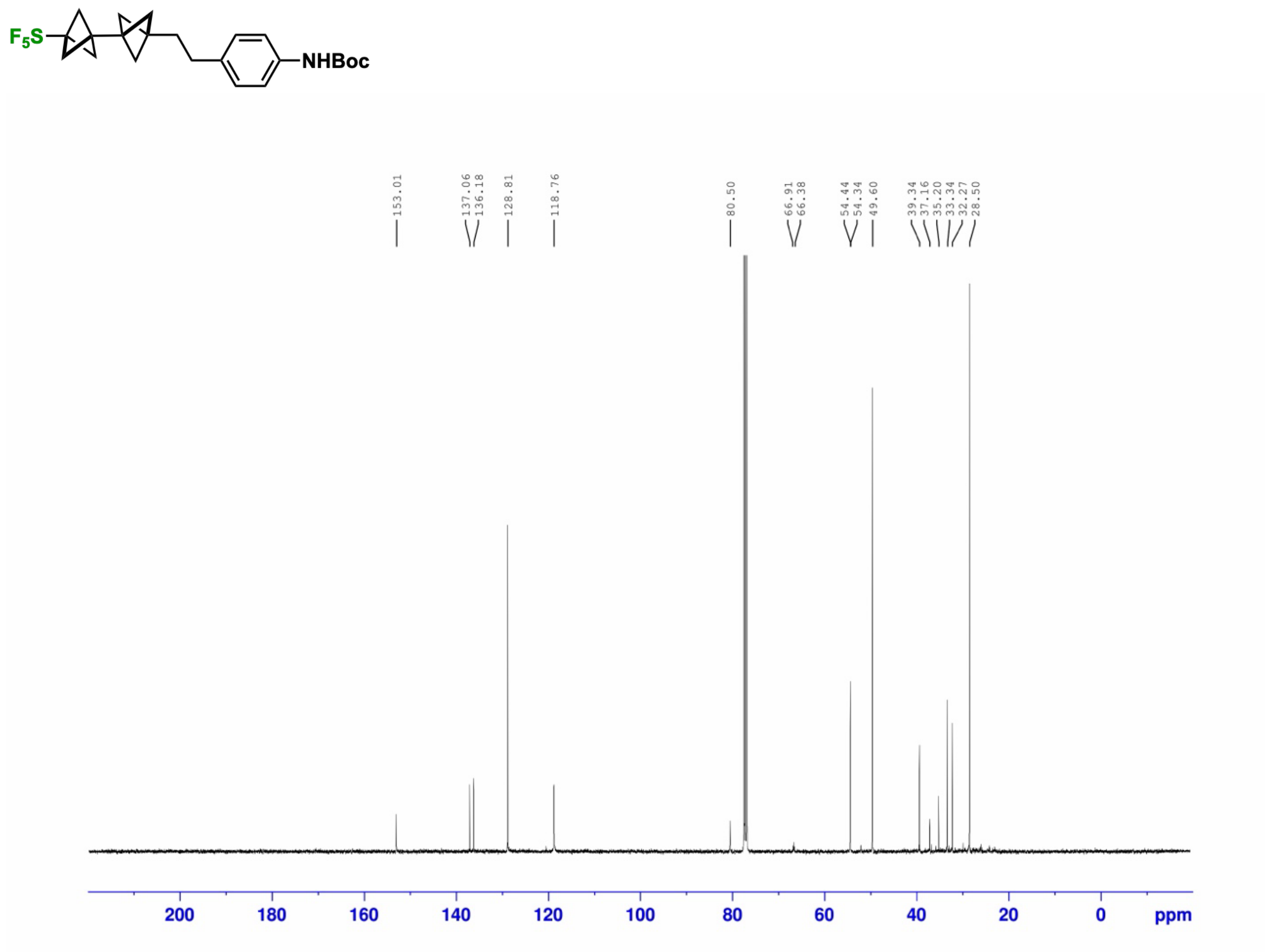

Figure S326. ^{19}F NMR spectrum of *tert*-butyl (4-(2-(3'-(pentafluoro-λ^6-sulfanyl)-[1,1'-bi(bicyclo[1.1.1]pentan)]-3-yl)ethyl)phenyl)carbamate (**75**) in $CDCl_3$.

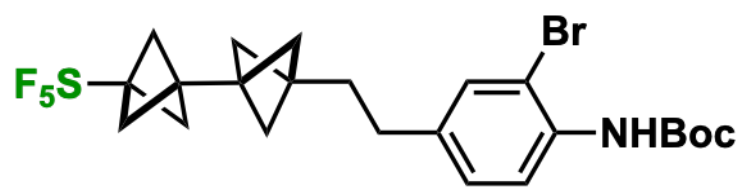

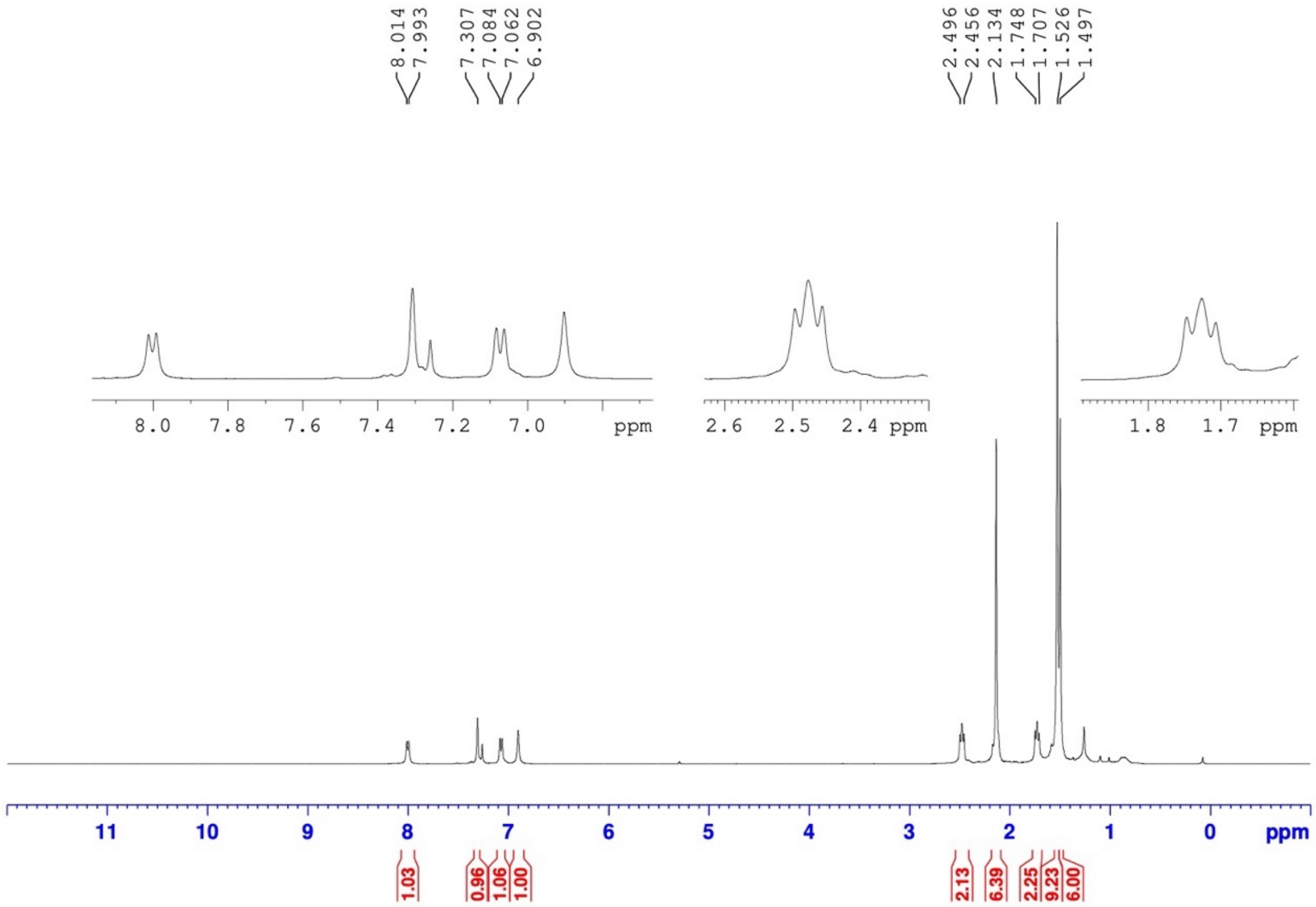

Figure S327. ^{1}H NMR spectrum of *tert*-butyl (2-bromo-4-(2-(3'-(pentafluoro-λ^6-sulfanyl)-[1,1'-bi(bicyclo[1.1.1]pentan)]-3-yl)ethyl)phenyl)carbamate (**76**) in $CDCl_3$.

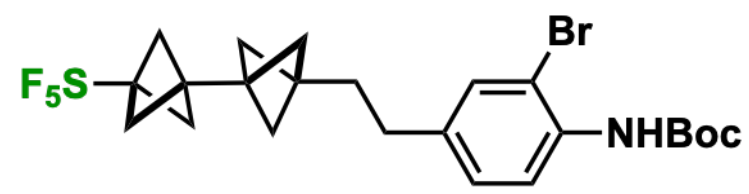

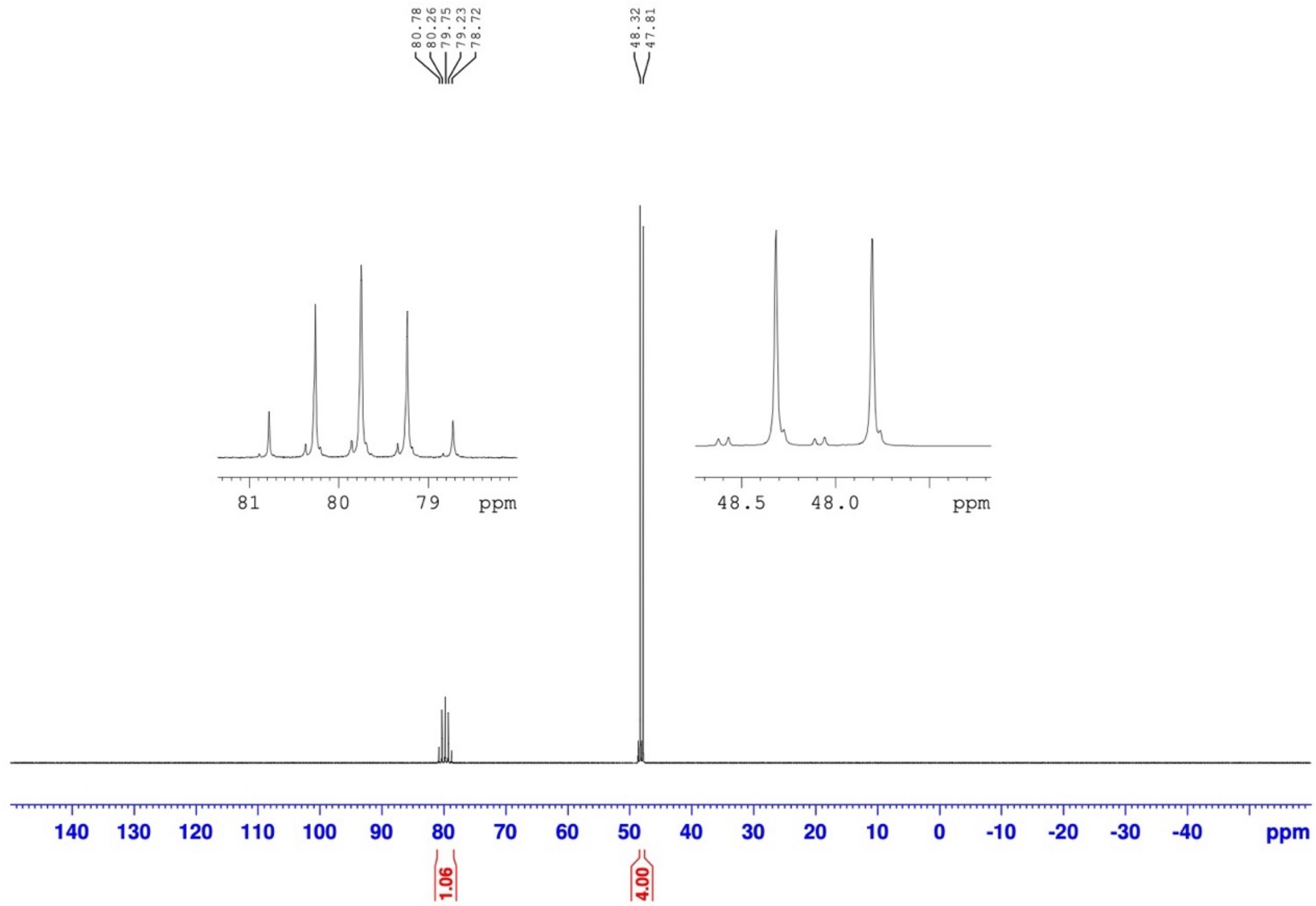

Figure S328. ^{19}F NMR spectrum of *tert*-butyl (2-bromo-4-(2-(3'-(pentafluoro-λ^6-sulfanyl)-[1,1'-bi(bicyclo[1.1.1]pentan)]-3-yl)ethyl)phenyl)carbamate (**76**) in $CDCl_3$.

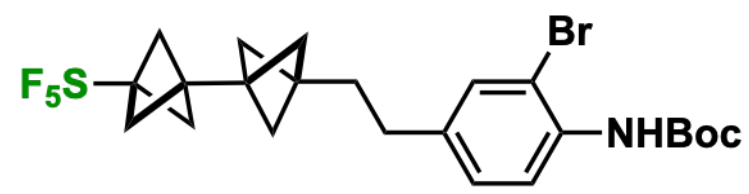

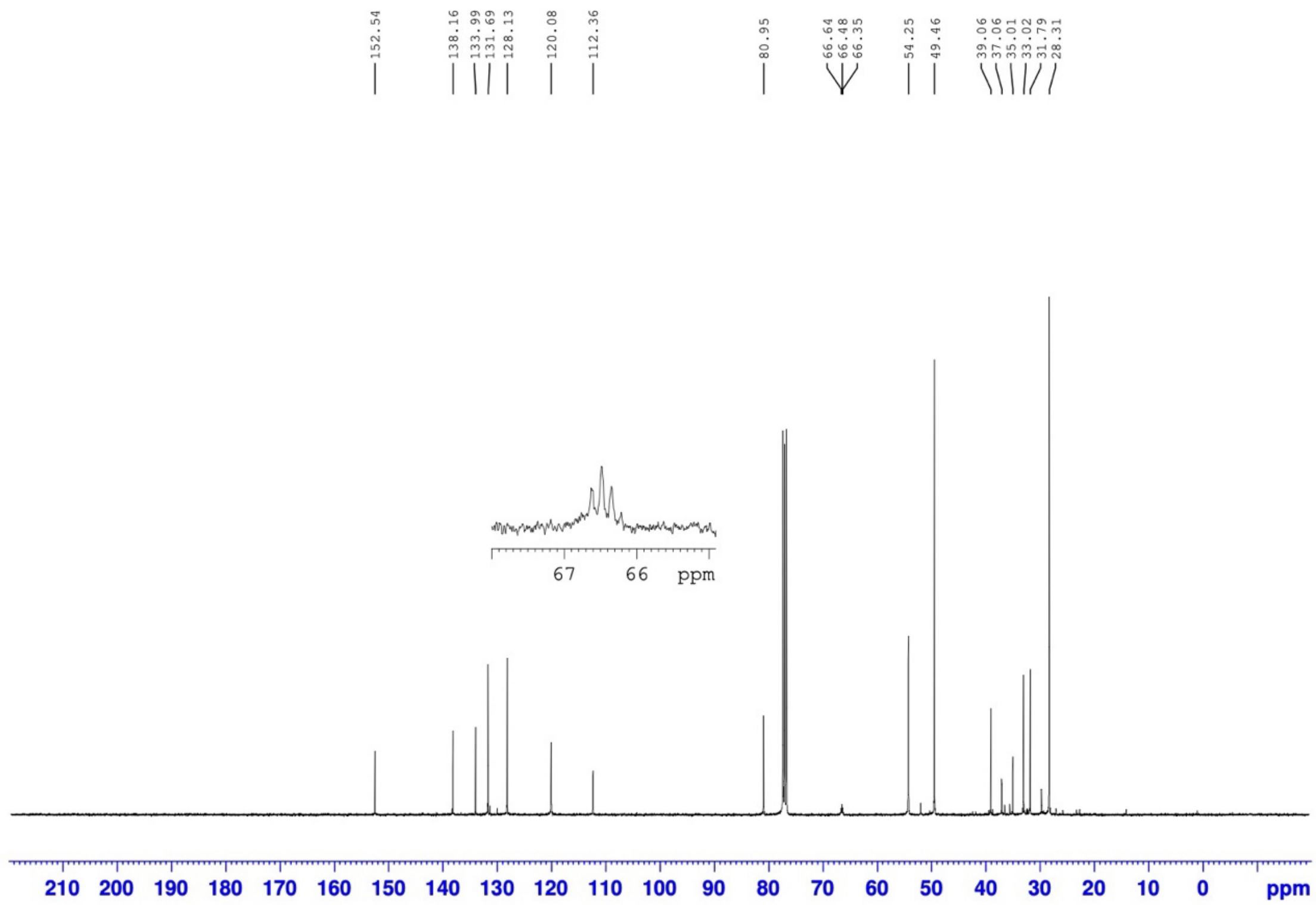

Figure S329. $^{13}C\{^1H\}$ NMR spectrum of *tert*-butyl (2-bromo-4-(2-(3'-(pentafluoro-λ^6-sulfanyl)-[1,1'-bi(bicyclo[1.1.1]pentan)]-3-yl)ethyl)phenyl)carbamate (**76**) in $CDCl_3$.

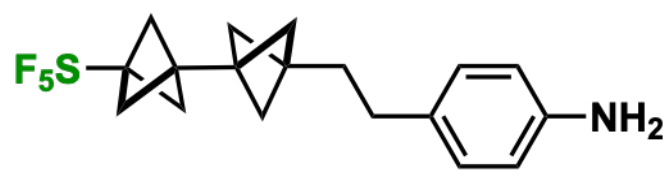

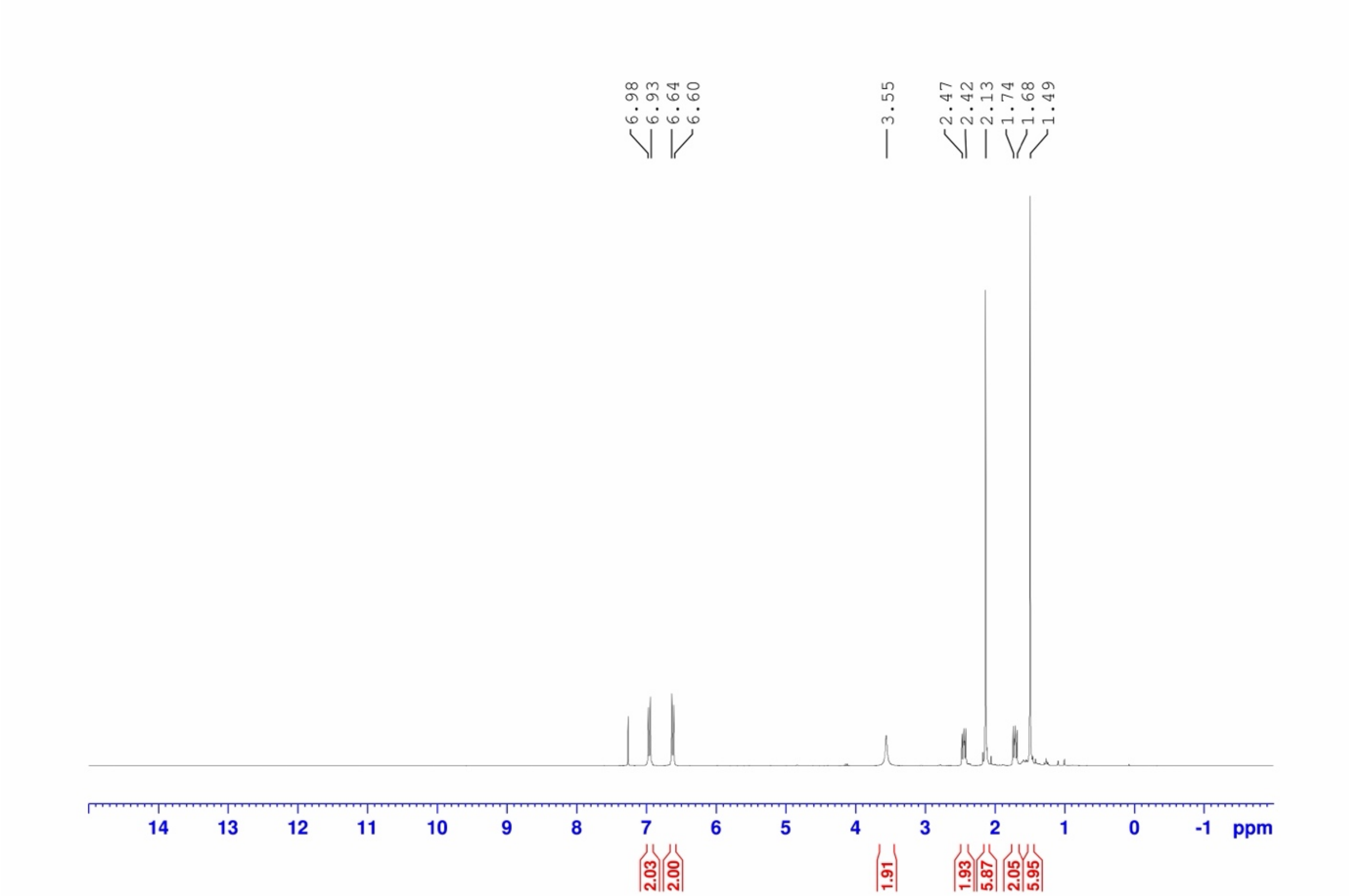

Figure S330. ^{1}H NMR spectrum of 4-(2-(3'-(pentafluoro-λ^6-sulfanyl)-[1,1'-bi(bicyclo[1.1.1]pentan)]-3-yl)ethyl)aniline (**S-33**) in $CDCl_3$.

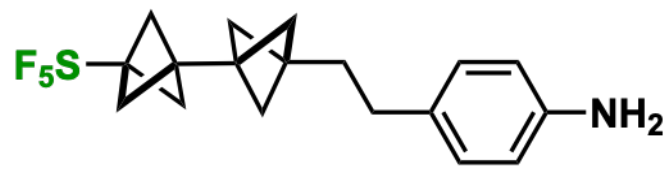

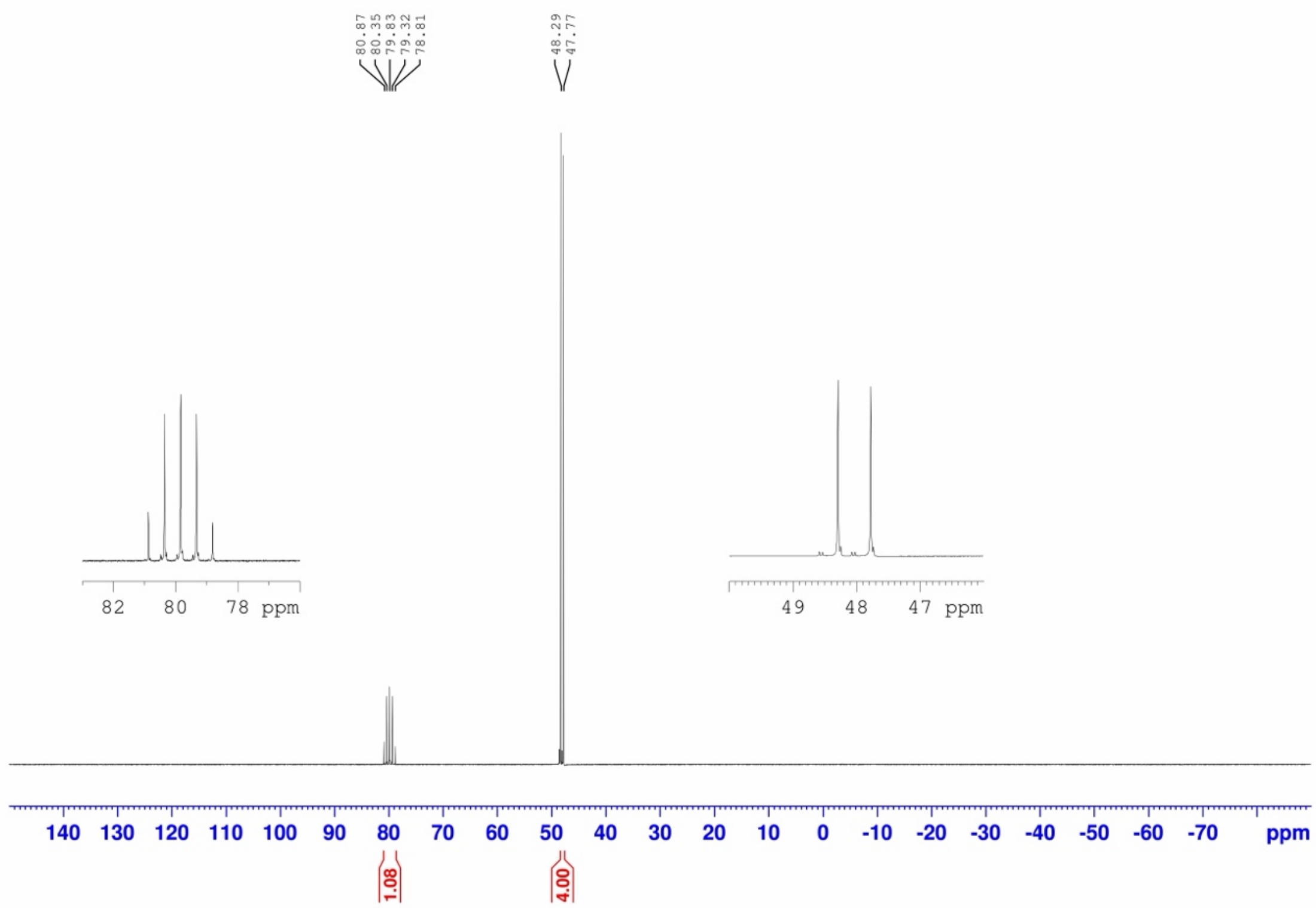

Figure S331. ^{19}F NMR spectrum of 4-(2-(3'-(pentafluoro-λ^6-sulfanyl)-[1,1'-bi(bicyclo[1.1.1]pentan)]-3-yl)ethyl)aniline (**S-33**) in $CDCl_3$.

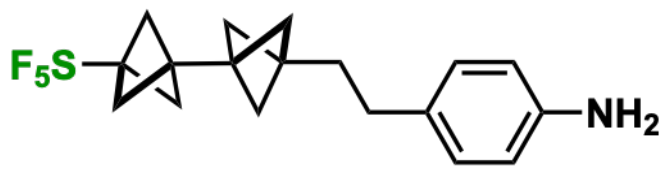

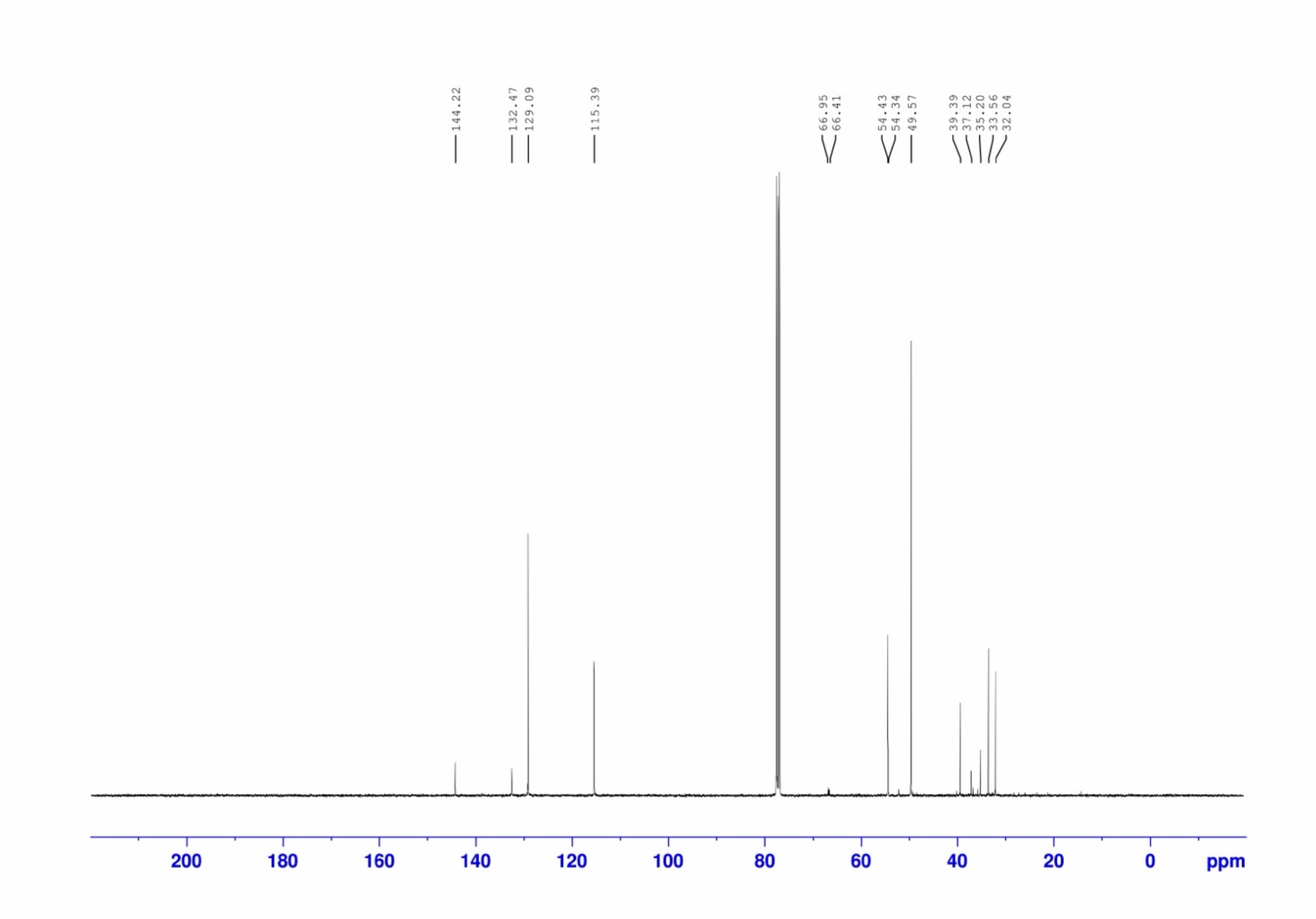

Figure S332. $^{13}C\{^{1}H\}$ NMR spectrum of 4-(2-(3'-(pentafluoro-λ^{6}-sulfanyl)-[1,1'-bi(bicyclo[1.1.1]pentan)]-3-yl)ethyl)aniline (**S-33**) in $CDCl_3$.

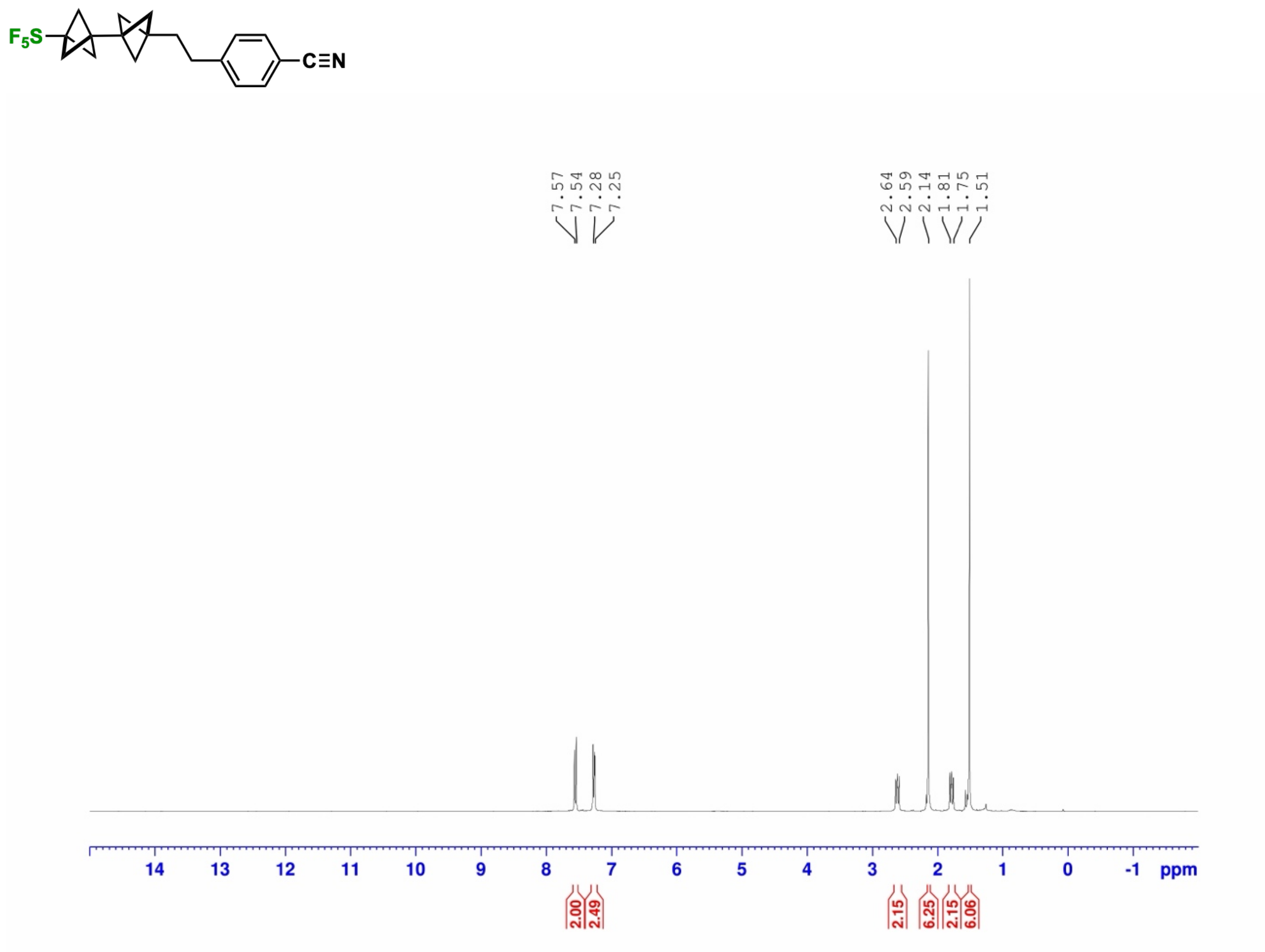

Figure S333. ^{1}H NMR spectrum of 4-(2-(3'-(pentafluoro-λ[6]-sulfanyl)-[1,1'-bi(bicyclo[1.1.1]pentan)]-3-yl)ethyl)benzonitrile (**77**) in $CDCl_3$.

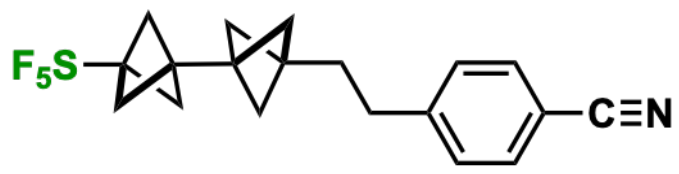

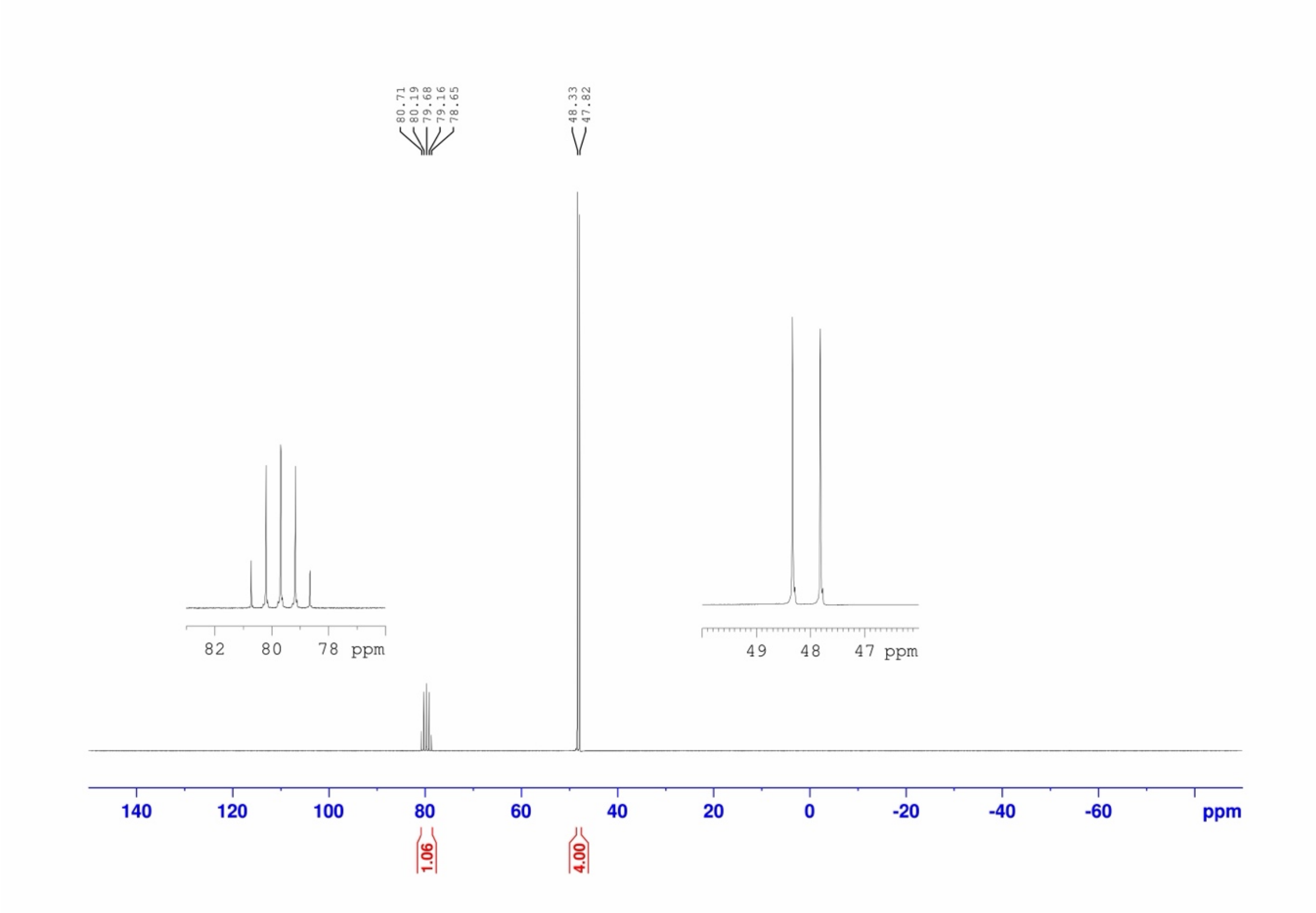

Figure S334. ^{19}F NMR spectrum of 4-(2-(3'-(pentafluoro-λ^6-sulfanyl)-[1,1'-bi(bicyclo[1.1.1]pentan)]-3-yl)ethyl)benzonitrile (**77**) in $CDCl_3$.

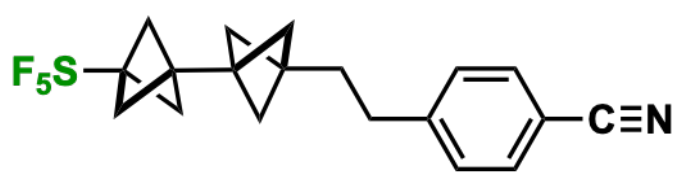

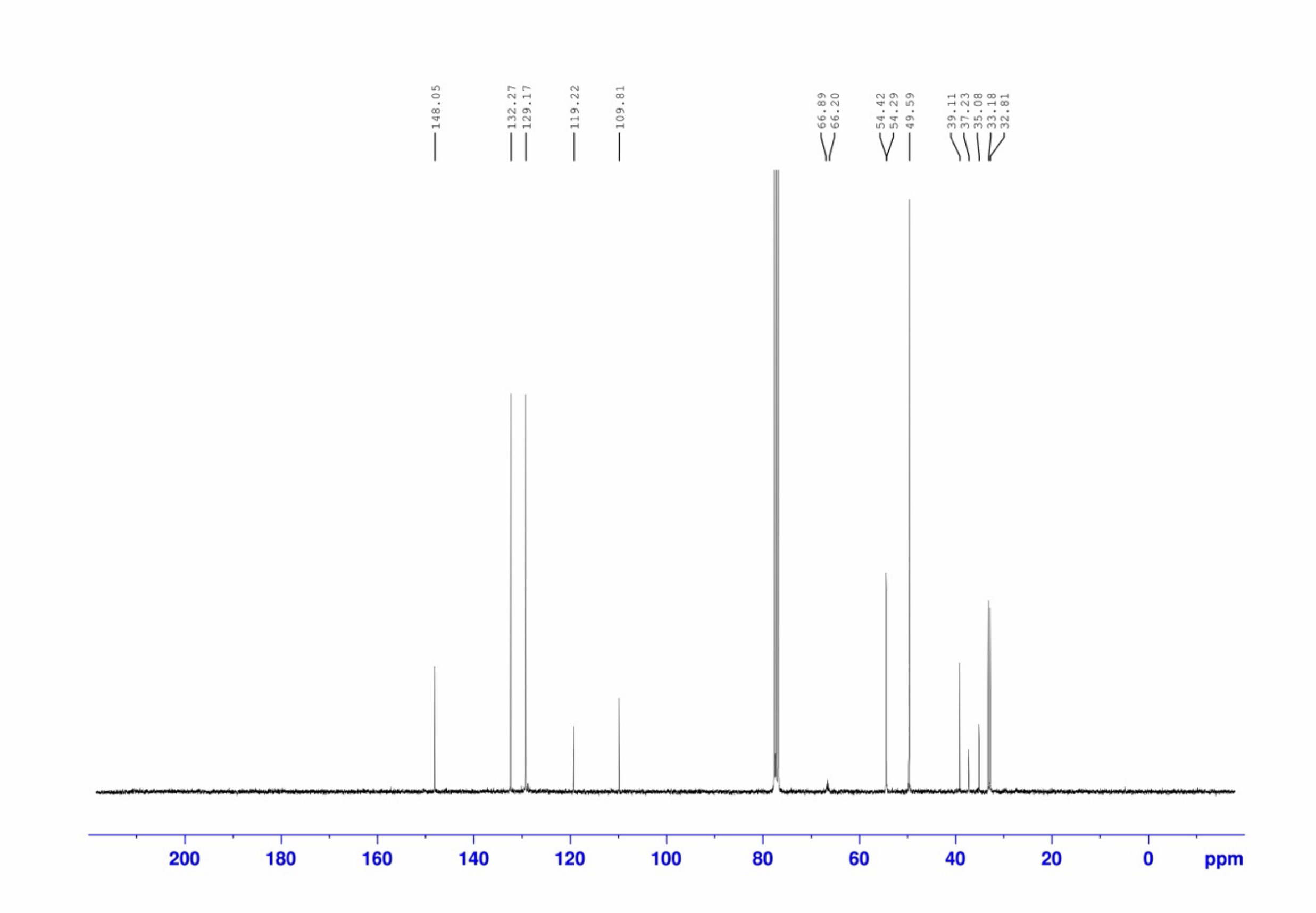

Figure S335. $^{13}C\{^{1}H\}$ NMR spectrum of 4-(2-(3'-(pentafluoro-λ^6-sulfanyl)-[1,1'-bi(bicyclo[1.1.1]pentan)]-3-yl)ethyl)benzonitrile (**77**) in $CDCl_3$.

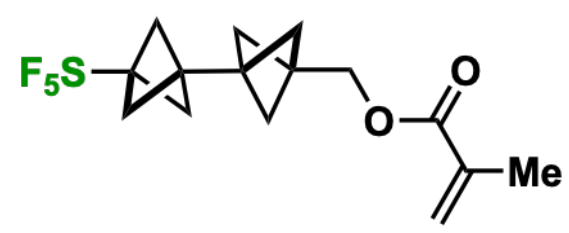

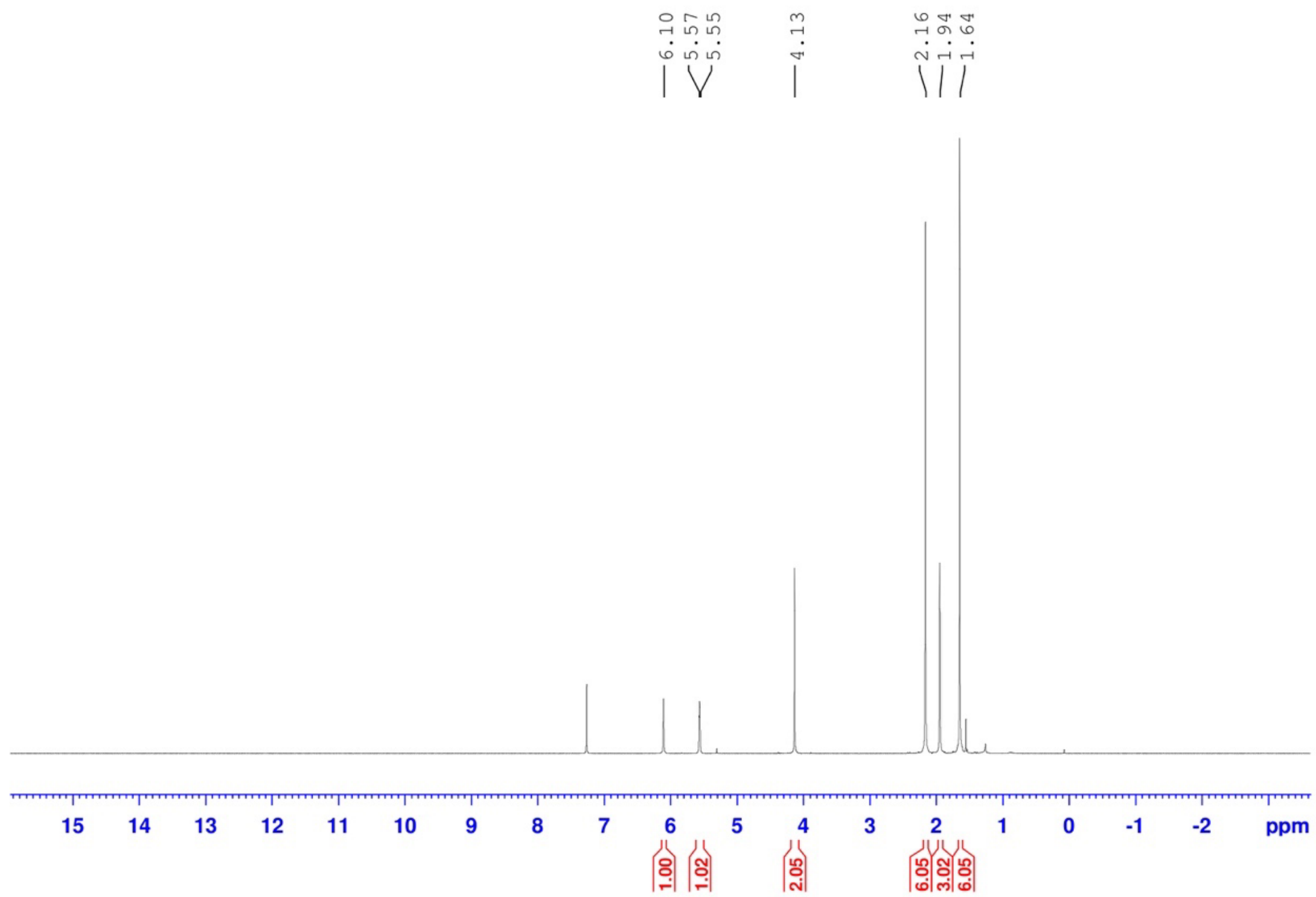

Figure S336. ^{1}H NMR spectrum of (3'-(pentafluoro-λ^{6}-sulfanyl)-[1,1'-bi(bicyclo[1.1.1]pentan)]-3-yl)methyl methacrylate (**S-34**) in $CDCl_3$.

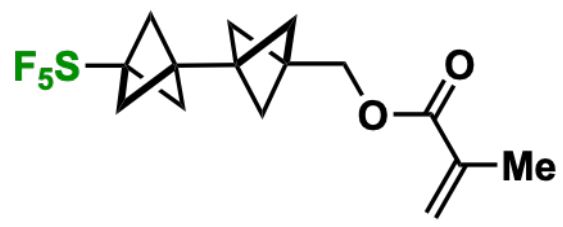

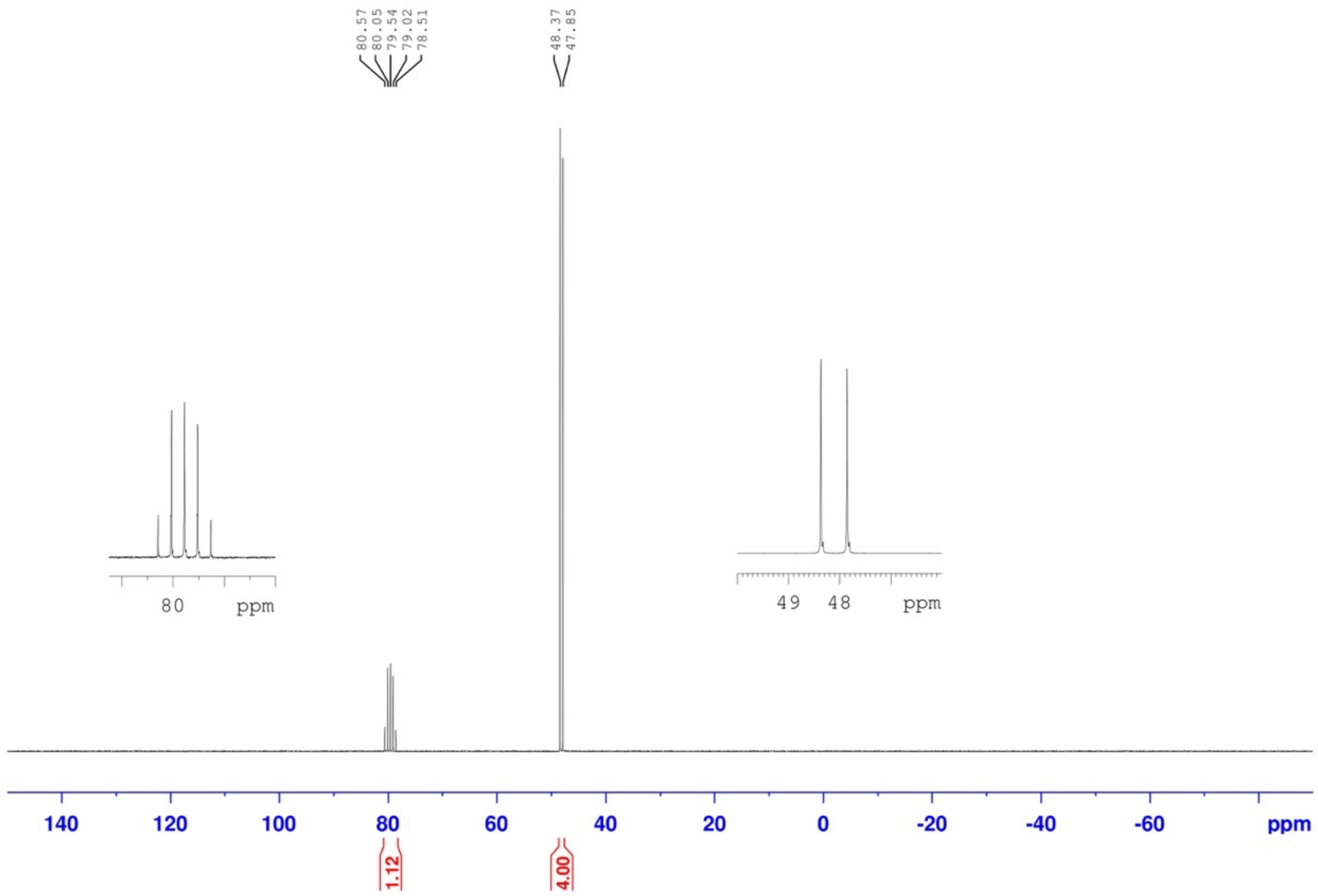

Figure S337. ^{19}F NMR spectrum of (3'-(pentafluoro-λ^6-sulfanyl)-[1,1'-bi(bicyclo[1.1.1]pentan)]-3-yl)methyl methacrylate (**S-34**) in $CDCl_3$.

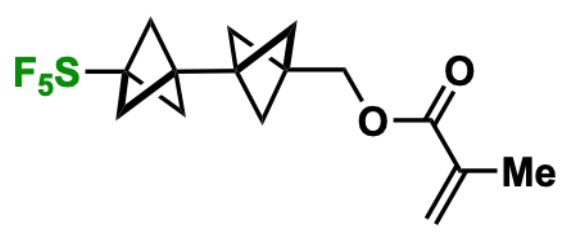

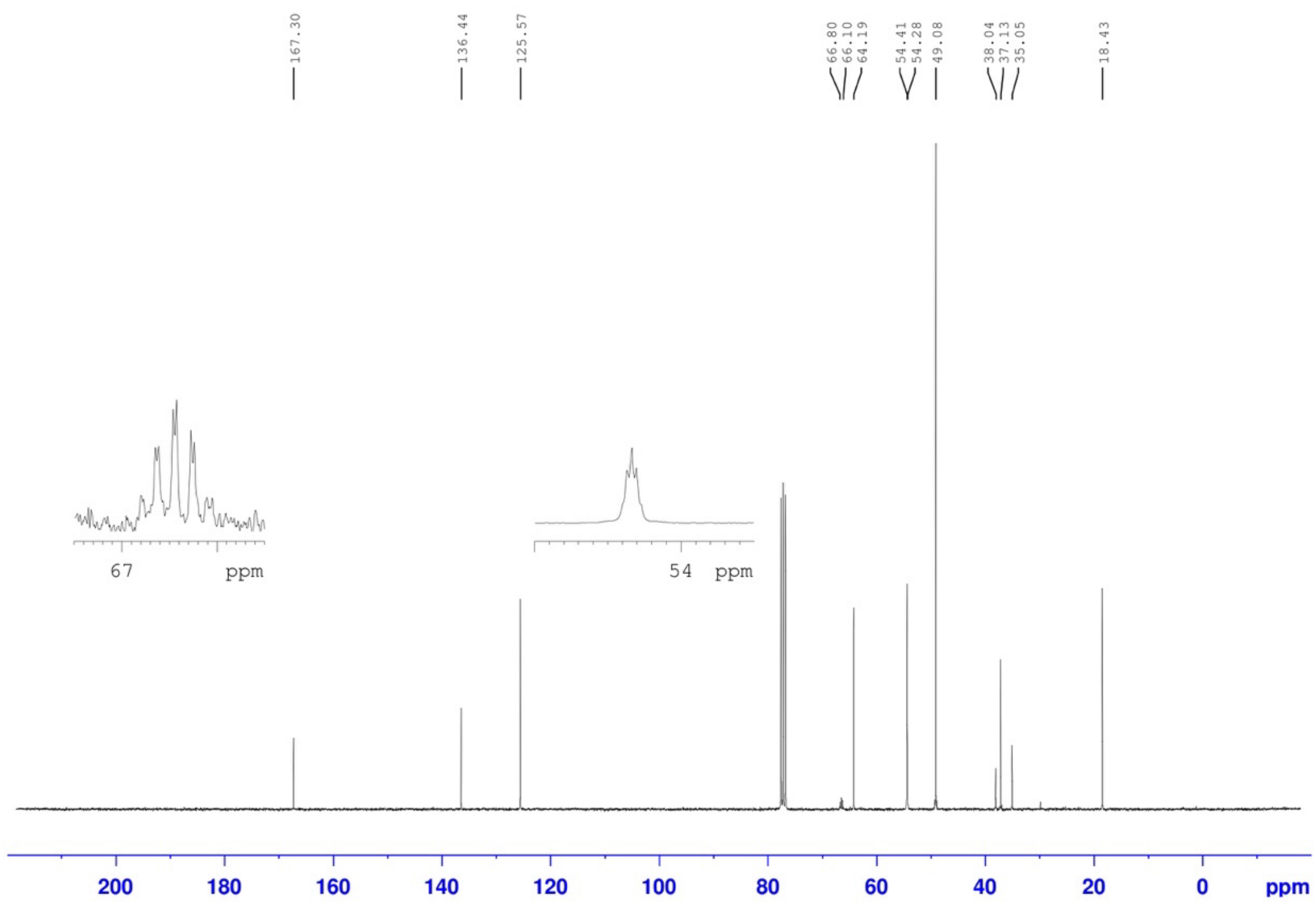

Figure S338. $^{13}C\{^{1}H\}$ NMR spectrum of (3'-(pentafluoro-λ^6-sulfanyl)-[1,1'-bi(bicyclo[1.1.1]pentan)]-3-yl)methyl methacrylate (**S-34**) in $CDCl_3$.

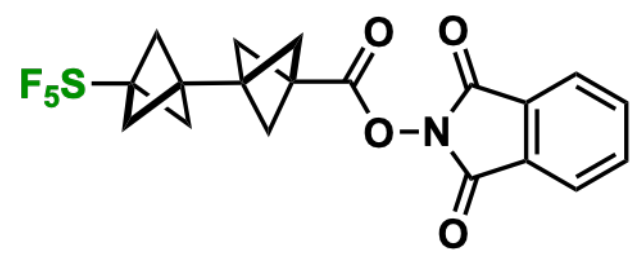

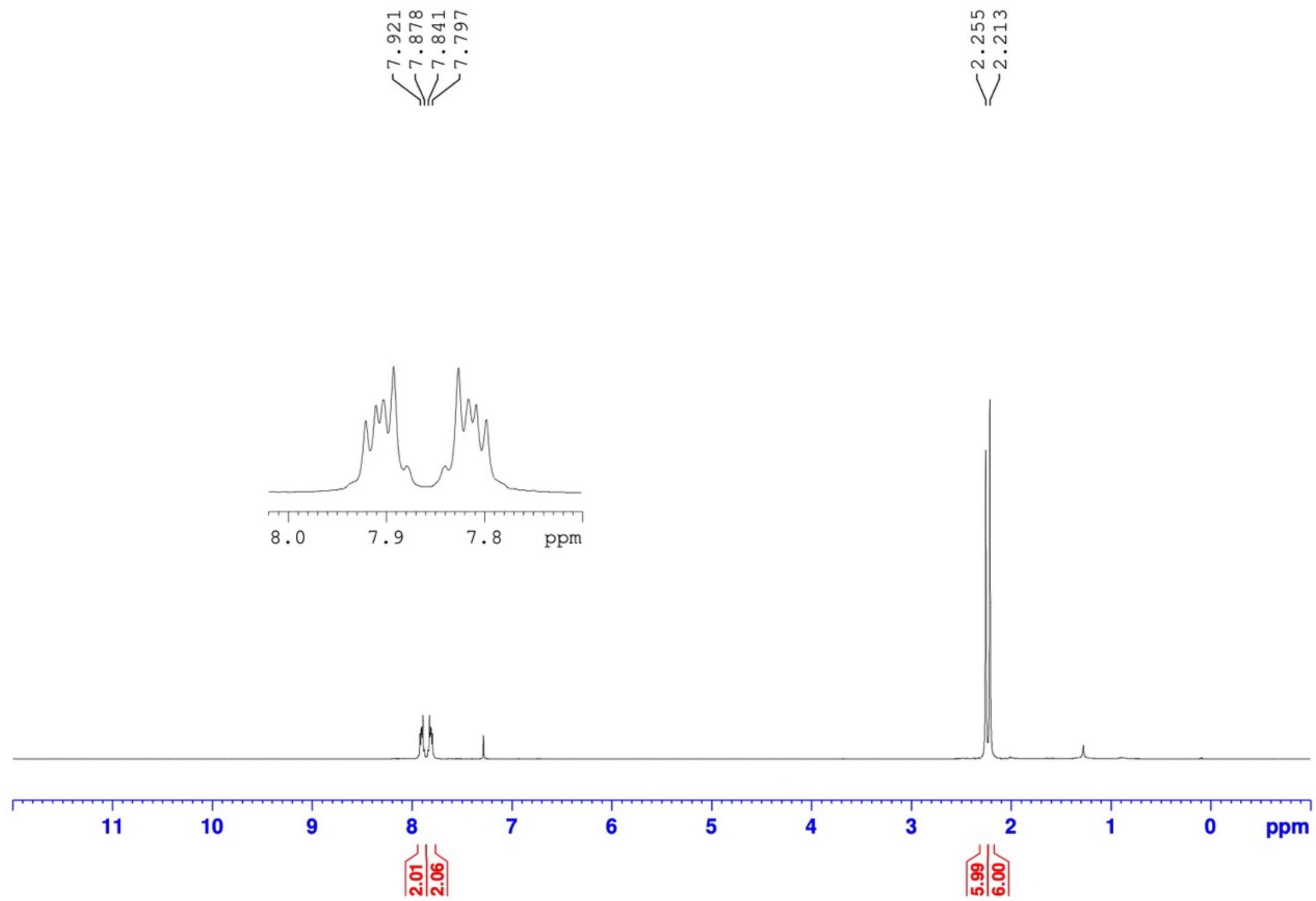

Figure S339. ^{1}H NMR spectrum of 1,3-dioxoisoindolin-2-yl 3'-(pentafluoro-λ^6-sulfaneyl)-[1,1'-bi(bicyclo[1.1.1]pentane)]-3-carboxylate (**S-35**) in $CDCl_3$.

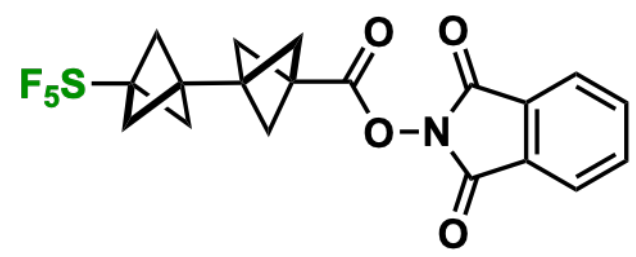

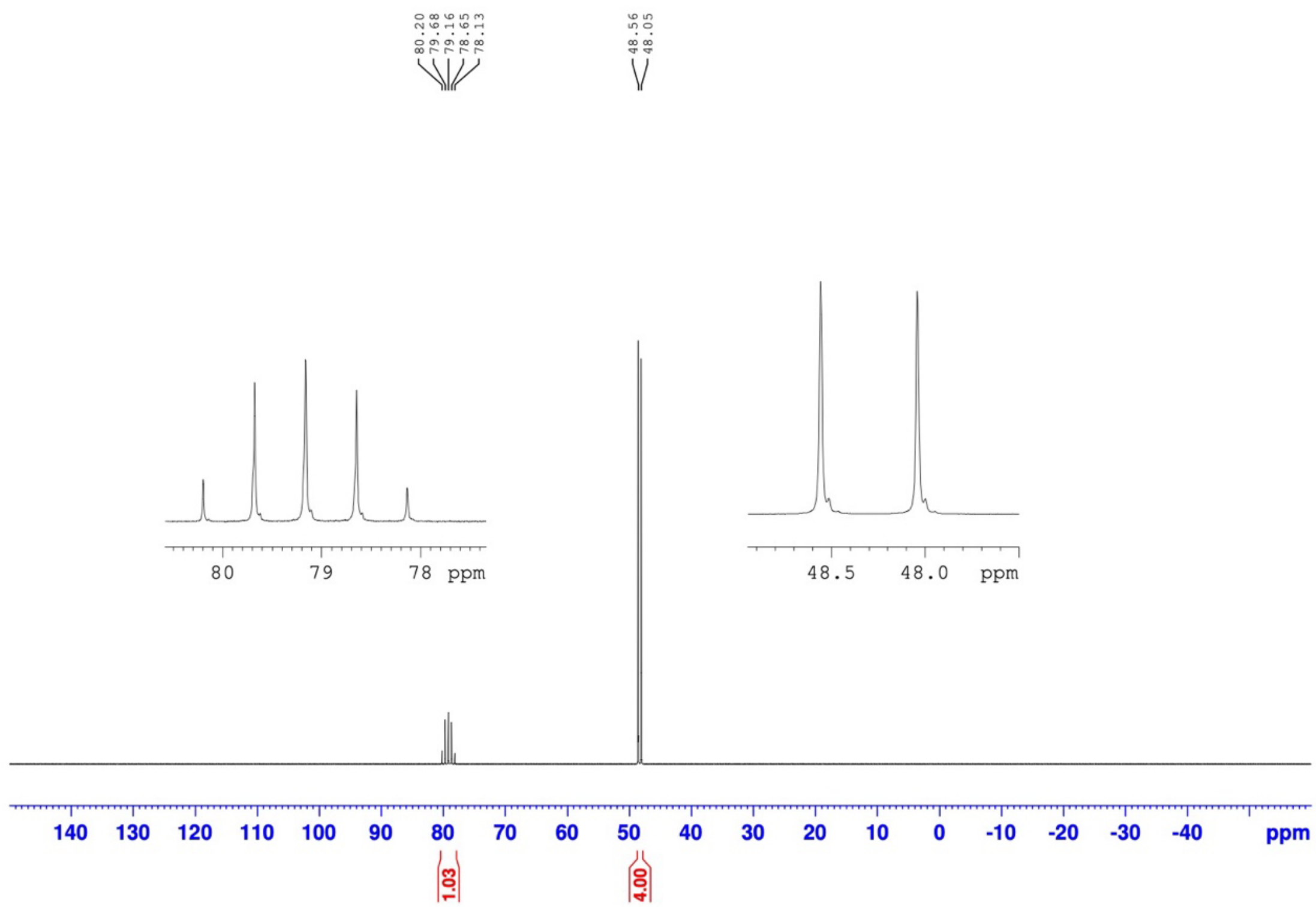

Figure S340. ^{19}F NMR spectrum of 1,3-dioxoisoindolin-2-yl 3'-(pentafluoro-λ^6-sulfaneyl)-[1,1'-bi(bicyclo[1.1.1]pentane)]-3-carboxylate (**S-35**) in $CDCl_3$.

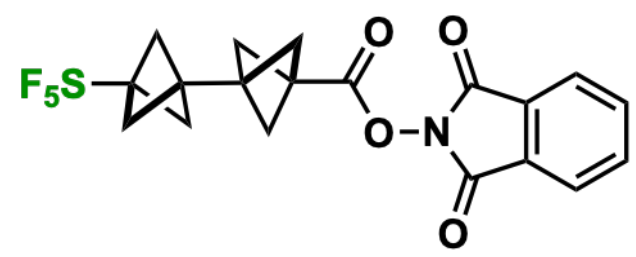

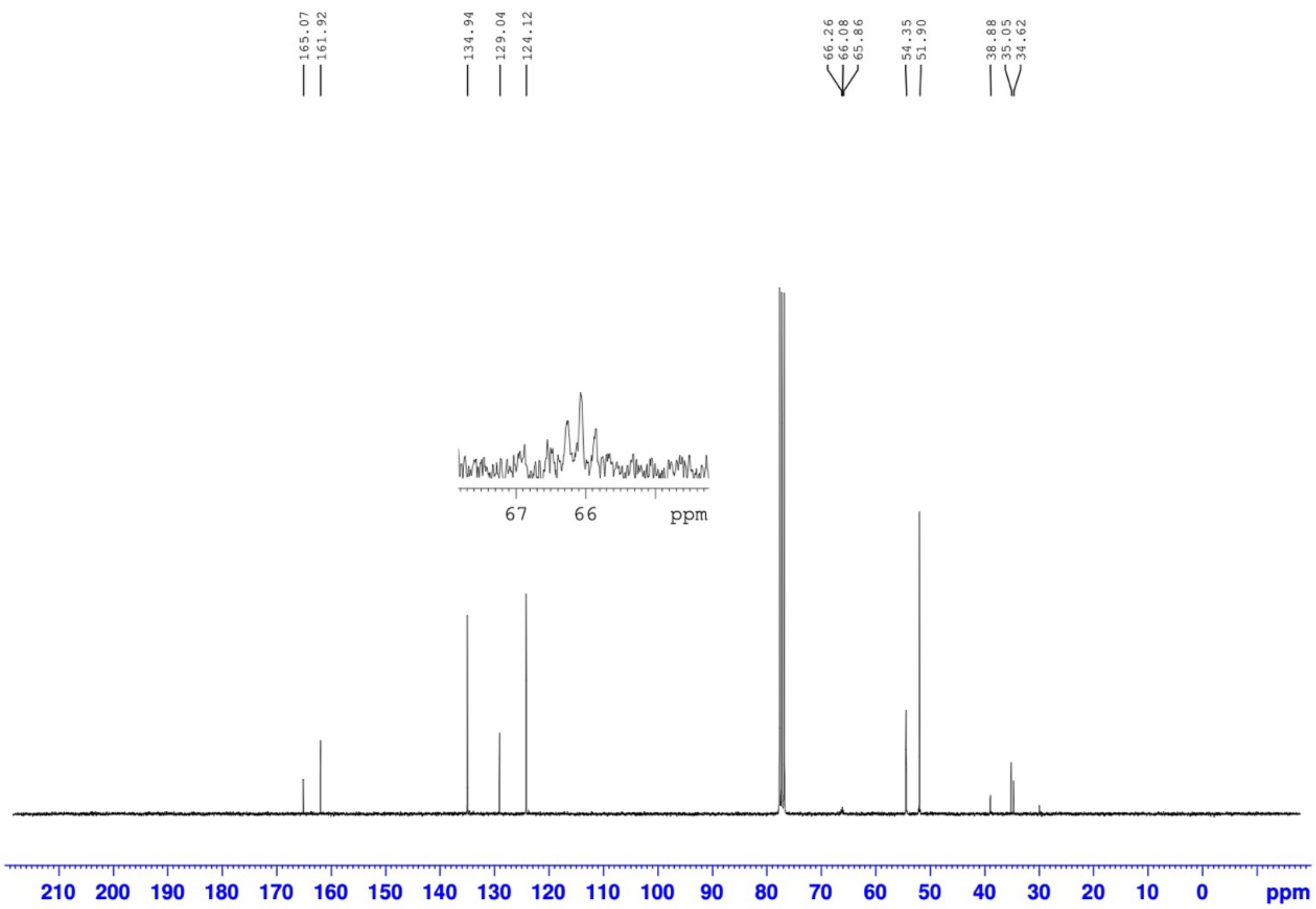

Figure S341. $^{13}C\{^{1}H\}$ NMR spectrum of 1,3-dioxoisoindolin-2-yl 3'-(pentafluoro-λ^6-sulfaneyl)-[1,1'-bi(bicyclo[1.1.1]pentane)]-3-carboxylate (**S-35**) in $CDCl_3$.

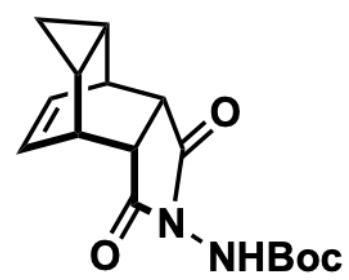

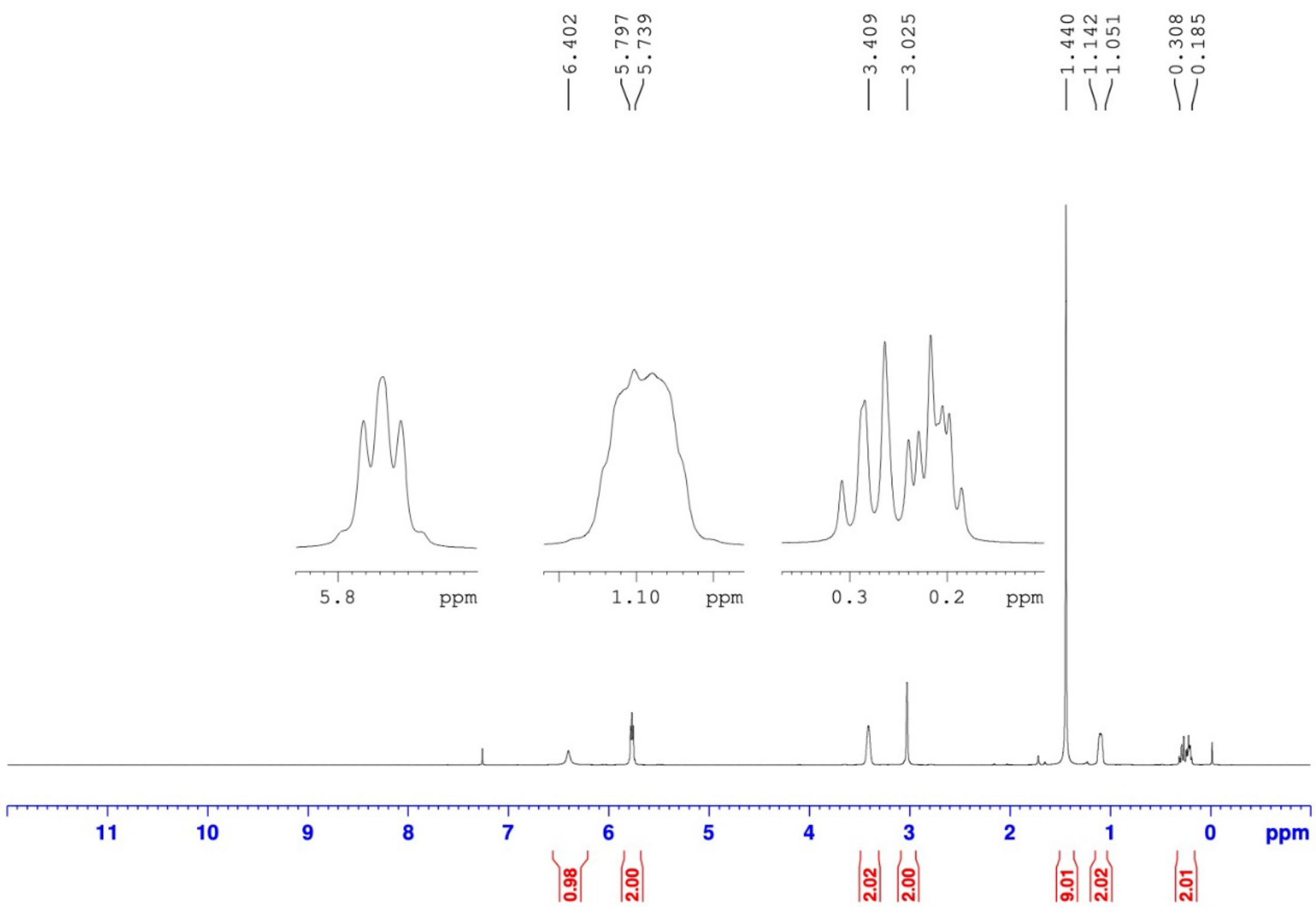

Figure S342. ^{1}H NMR spectrum of (*tert*-butyl ((4*R*,4a*R*,5a*S*,6*S*,6a*S*)-1,3-dioxo-3,3a,4,4a,5,5a,6,6a-octahydro-4,6-ethenocyclopropa[*f*]isoindol-2(1*H*)-yl)carbamate (**S-36**) in $CDCl_3$.

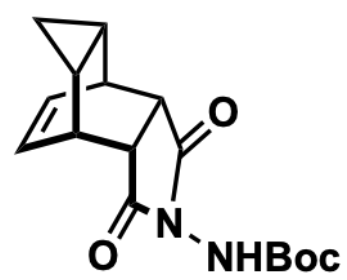

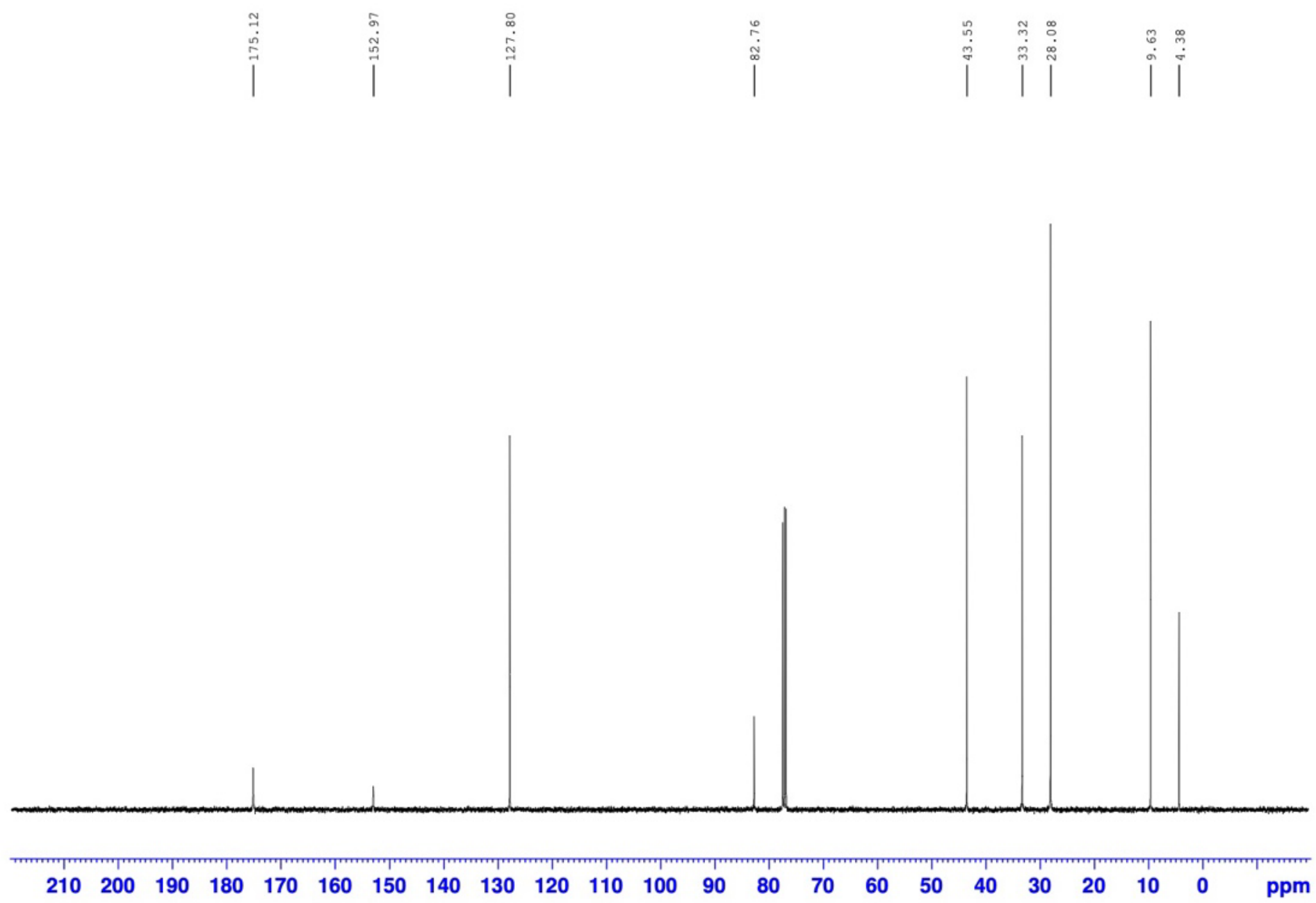

Figure S343. $^{13}C\{^1H\}$ NMR spectrum of (*tert*-butyl (4*R*,4a*R*,5a*S*,6*S*,6a*S*)-1,3-dioxo-3,3a,4,4a,5,5a,6,6a-octahydro-4,6-ethenocyclopropa[*f*]isoindol-2(1*H*)-yl)carbamate (**S-36**) in $CDCl_3$.

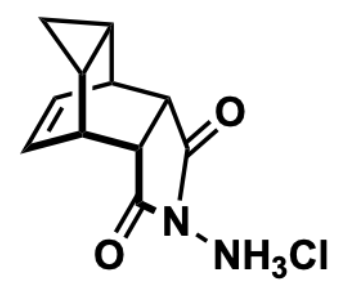

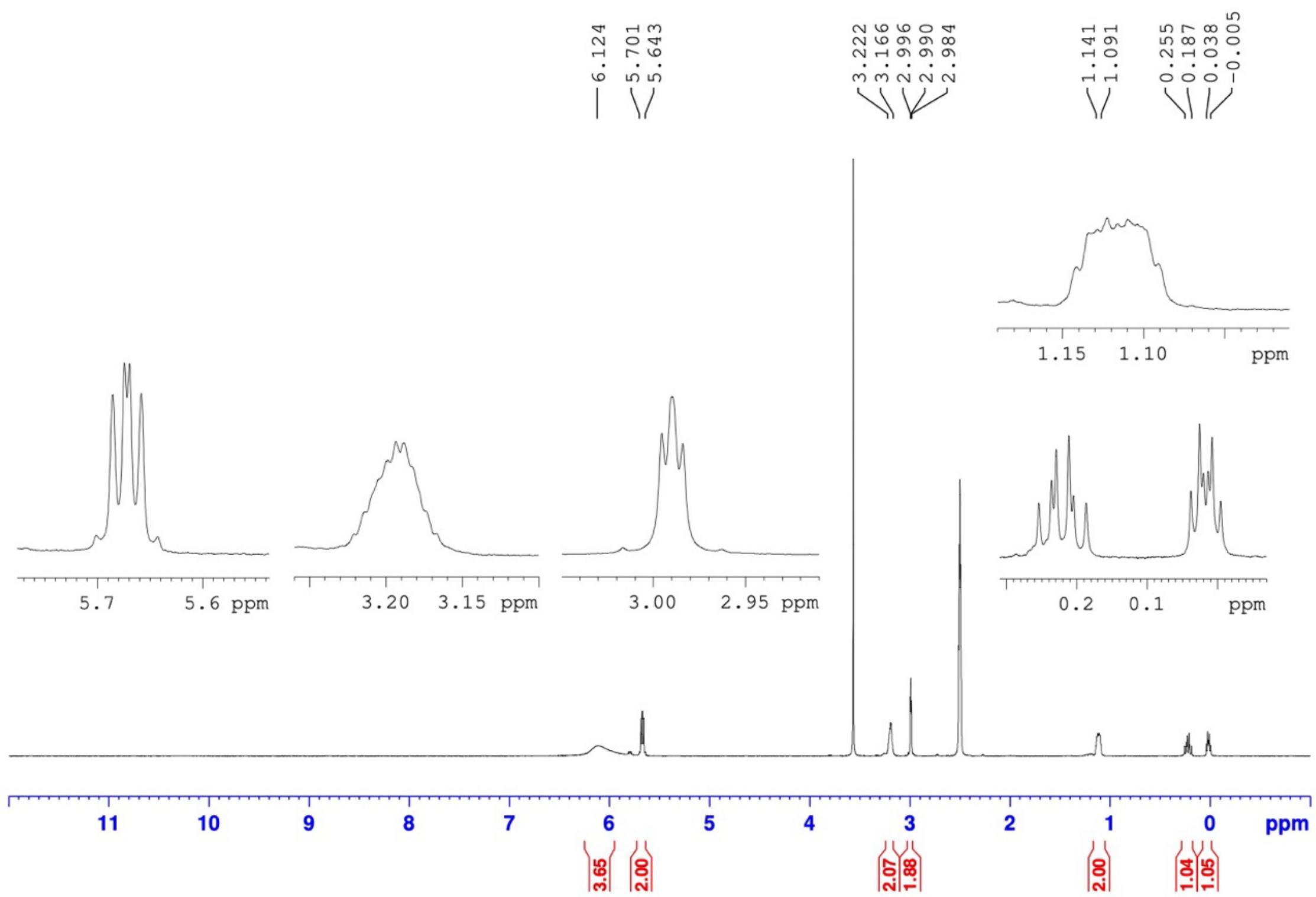

Figure S344. ^{1}H NMR spectrum of (4*R*,4a*R*,5a*S*,6*S*,6a*S*)-2-(chloro-λ^5-azaneyl)-4,4a,5,5a,6,6a-hexahydro-4,6-ethenocyclopropa[*f*]isoindole-1,3(2*H*,3a*H*)-dione (**S-37**) in DMSO-*d*$_6$.

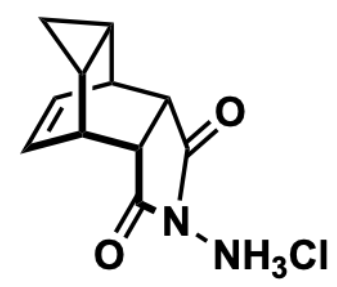

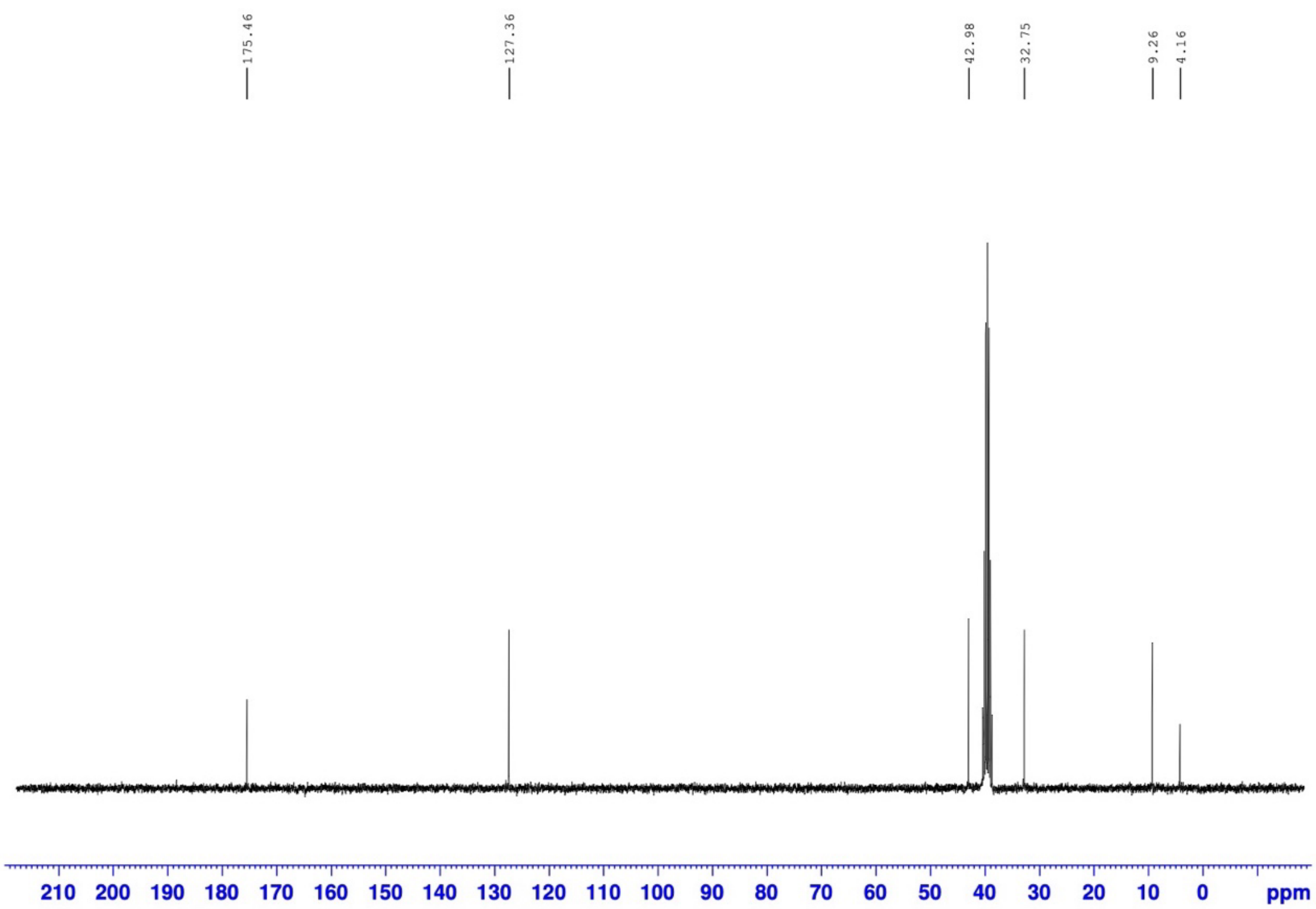

Figure S345. $^{13}C\{^1H\}$ NMR spectrum of (4*R*,4a*R*,5a*S*,6*S*,6a*S*)-2-(chloro-λ^5-azaneyl)-4,4a,5,5a,6,6a-hexahydro-4,6-ethenocyclopropa[*f*]isoindole-1,3(2*H*,3a*H*)-dione (**S-37**) in DMSO-*d*$_6$.

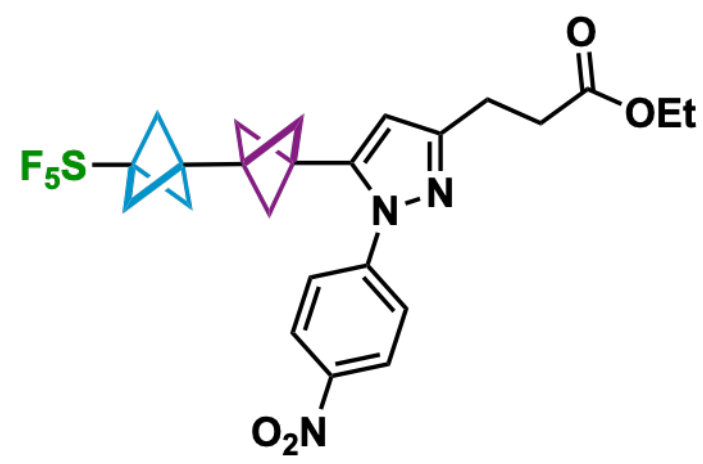

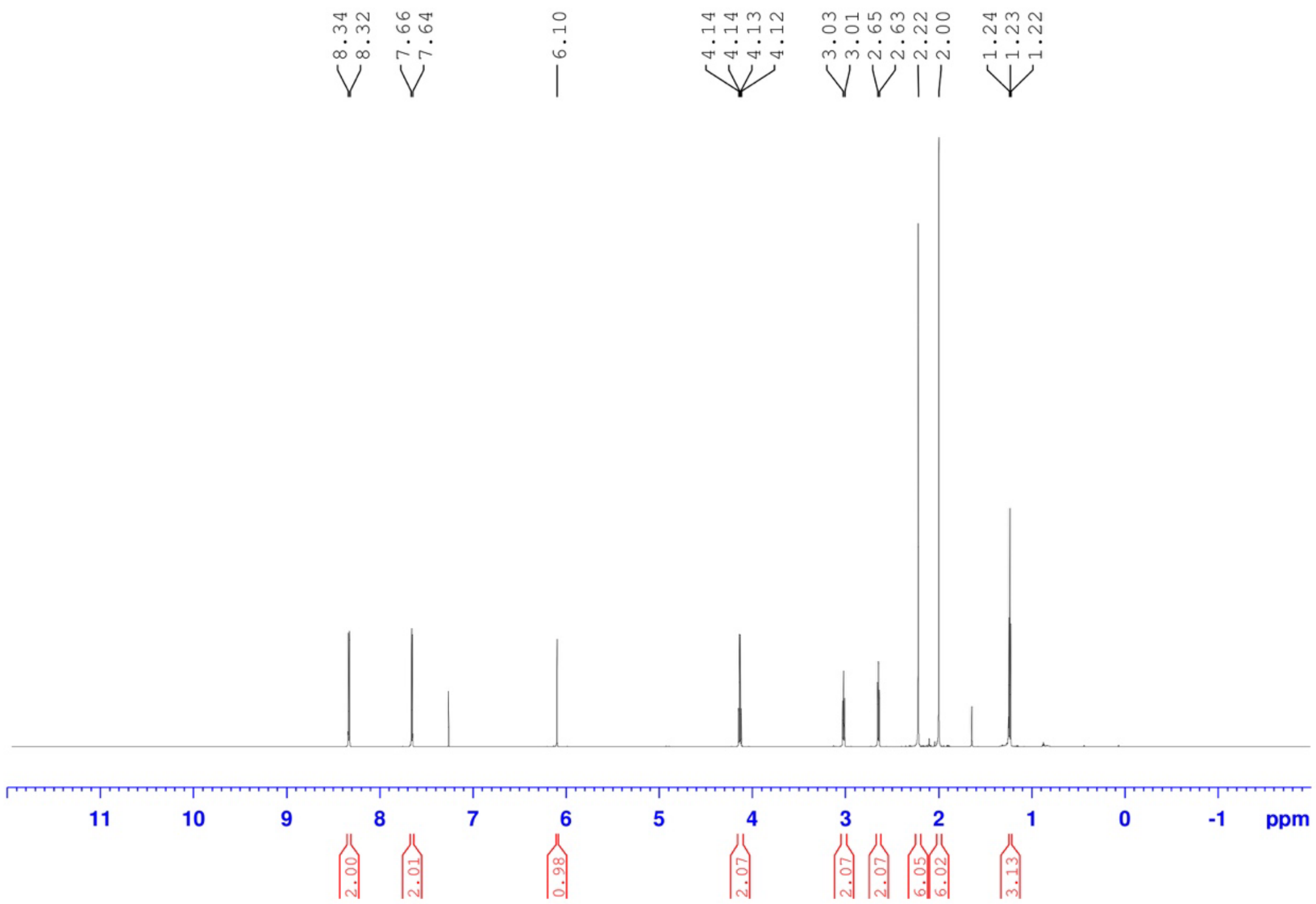

Figure S346. ^{1}H NMR spectrum of ethyl 3-(1-(4-nitrophenyl)-5-(3'-(pentafluoro-λ^6-sulfanyl)-[1,1'-bi(bicyclo[1.1.1]pentan)]-3-yl)-1*H*-pyrazol-3-yl)propanoate (**S-38**) in $CDCl_3$.

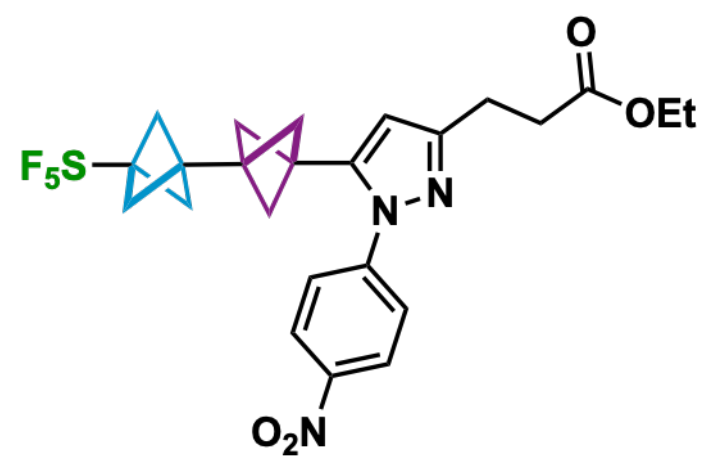

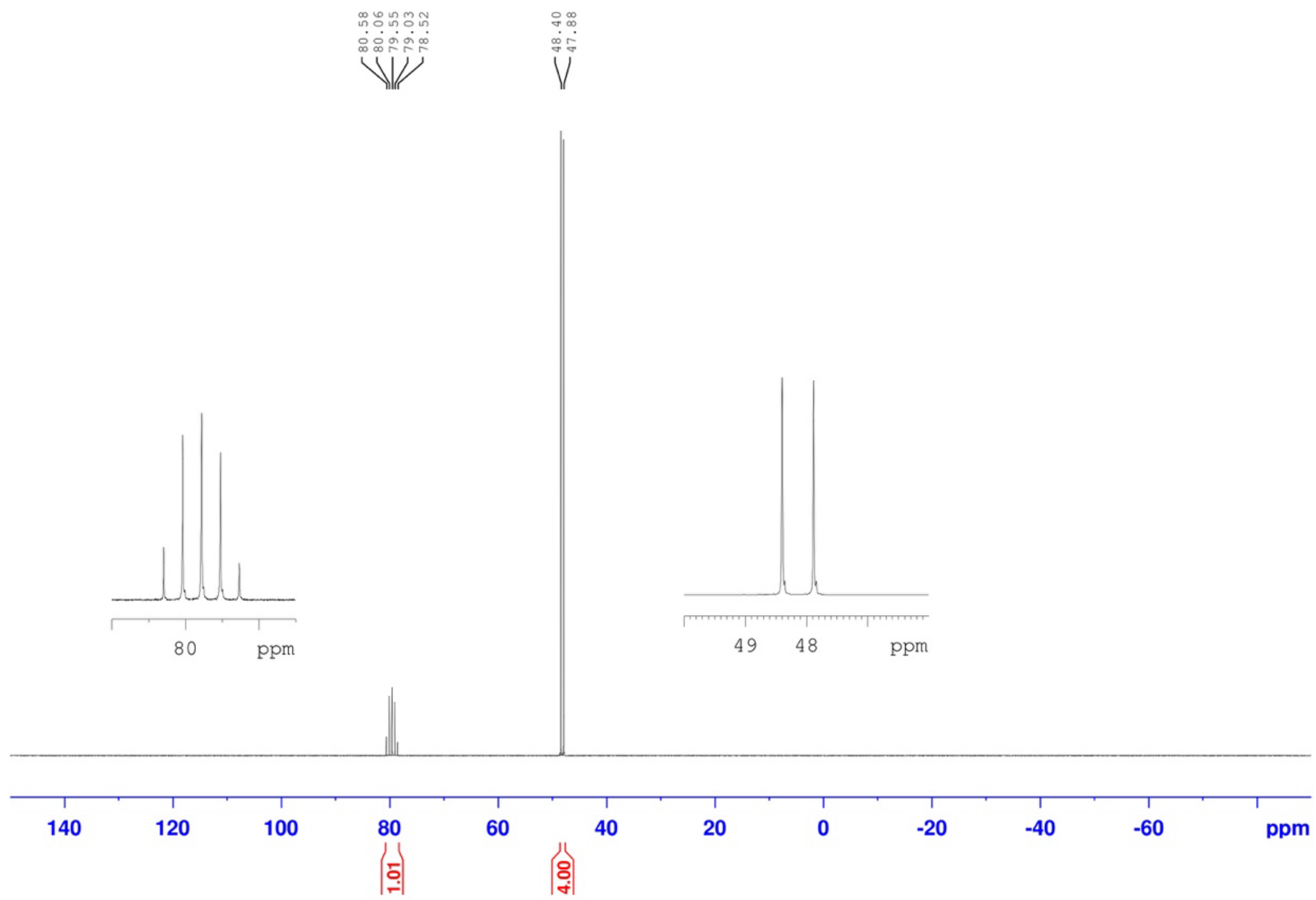

Figure S347. ^{19}F NMR spectrum of ethyl 3-(1-(4-nitrophenyl)-5-(3'-(pentafluoro-λ^6-sulfanyl)-[1,1'-bi(bicyclo[1.1.1]pentan)]-3-yl)-1*H*-pyrazol-3-yl)propanoate (**S-38**) in $CDCl_3$.

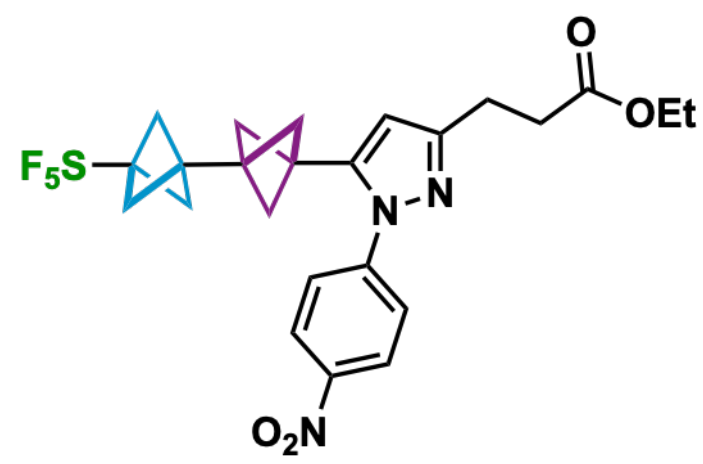

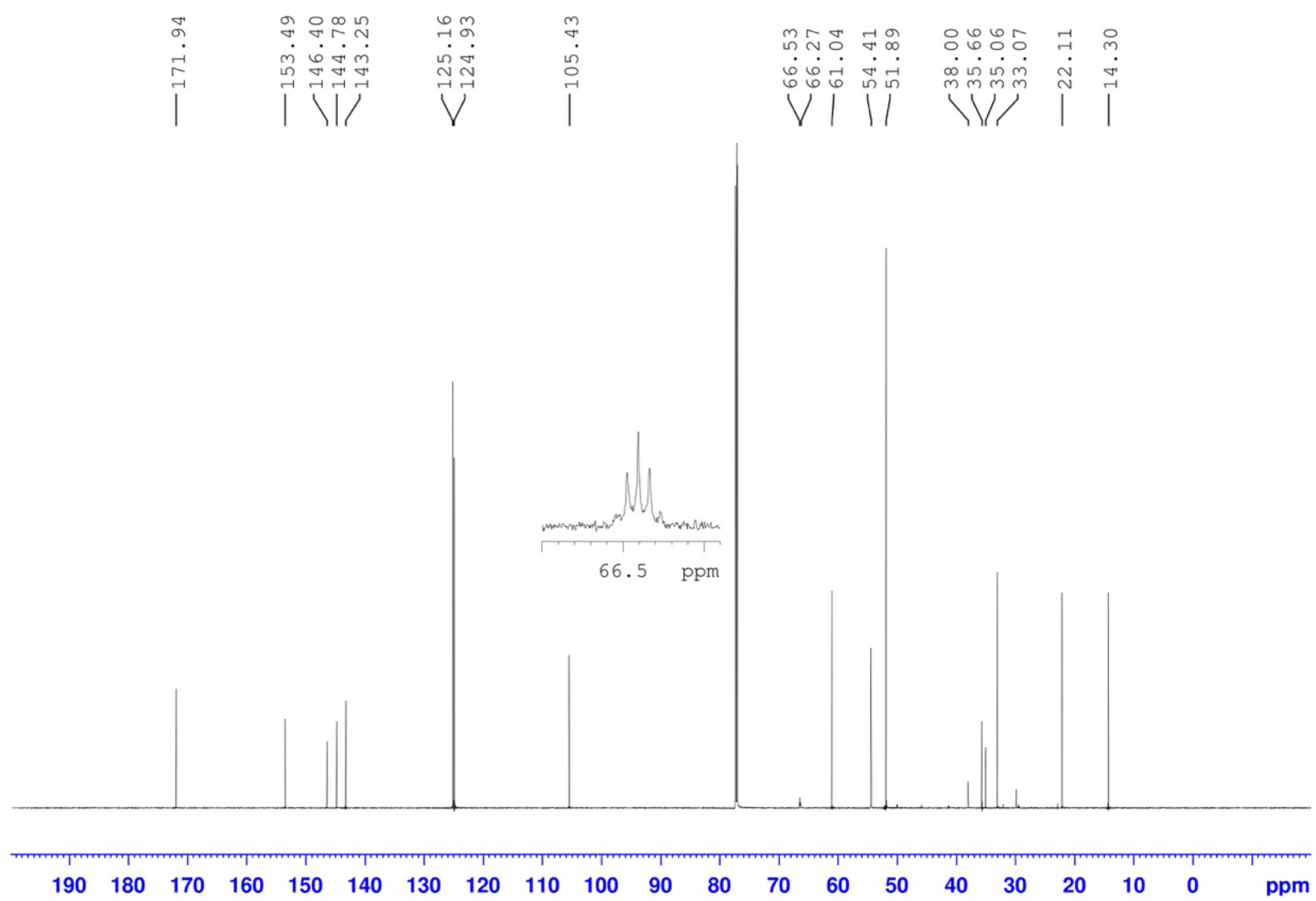

Figure S348. $^{13}C\{^{1}H\}$ NMR spectrum of ethyl 3-(1-(4-nitrophenyl)-5-(3'-(pentafluoro-λ^6-sulfanyl)-[1,1'-bi(bicyclo[1.1.1]pentan)]-3-yl)-1*H*-pyrazol-3-yl)propanoate (**S-38**) in $CDCl_3$.

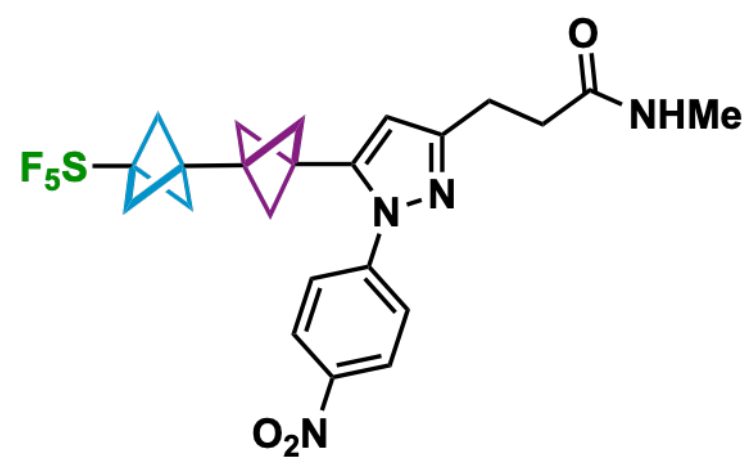

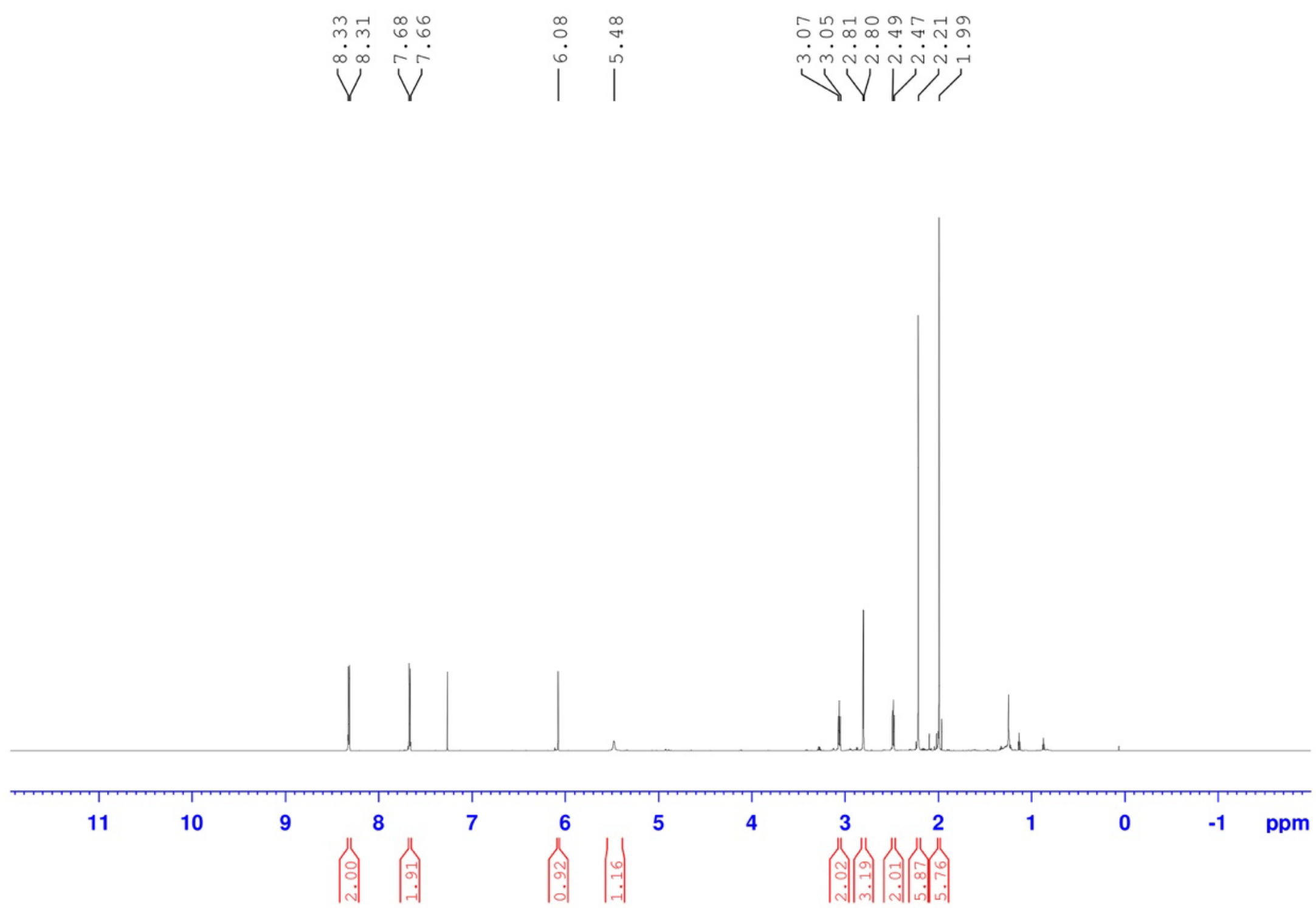

Figure S349. ^{1}H NMR spectrum of *N*-methyl-3-(1-(4-nitrophenyl)-5-(3'-(pentafluoro-λ^6-sulfaneyl)-[1,1'-bi(bicyclo[1.1.1]pentan)]-3-yl)-1*H*-pyrazol-3-yl)propanamide (**S-39**) in $CDCl_3$.

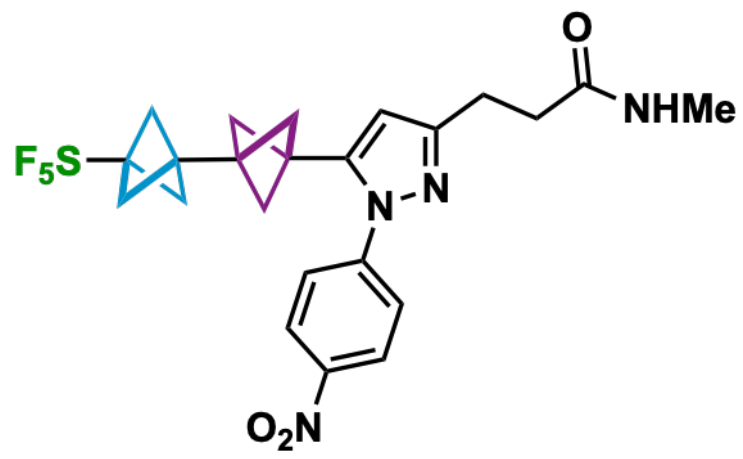

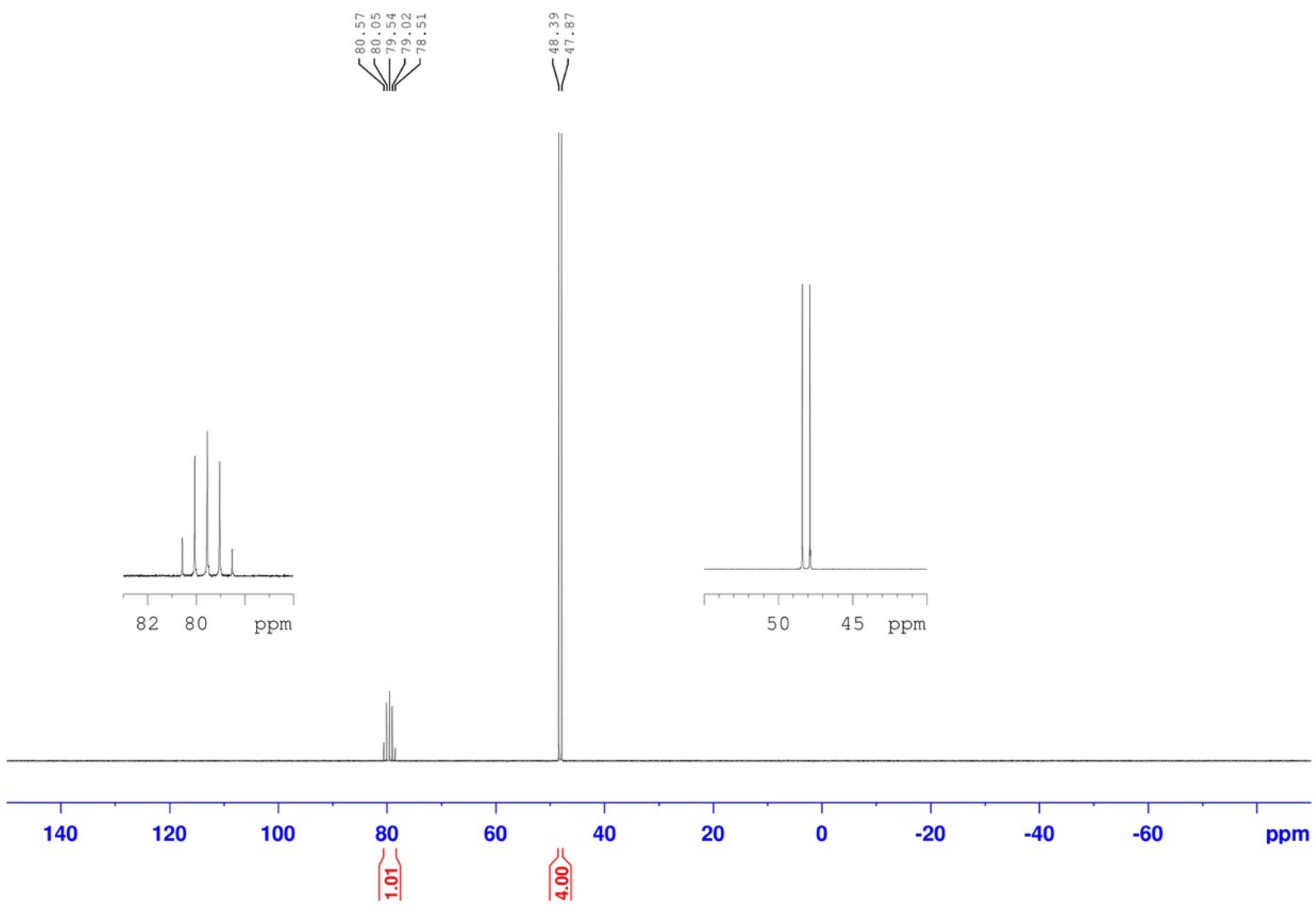

Figure S350. ^{19}F NMR spectrum of *N*-methyl-3-(1-(4-nitrophenyl)-5-(3'-(pentafluoro-λ^6-sulfaneyl)-[1,1'-bi(bicyclo[1.1.1]pentan)]-3-yl)-1*H*-pyrazol-3-yl)propanamide (**S-39**) in $CDCl_3$.

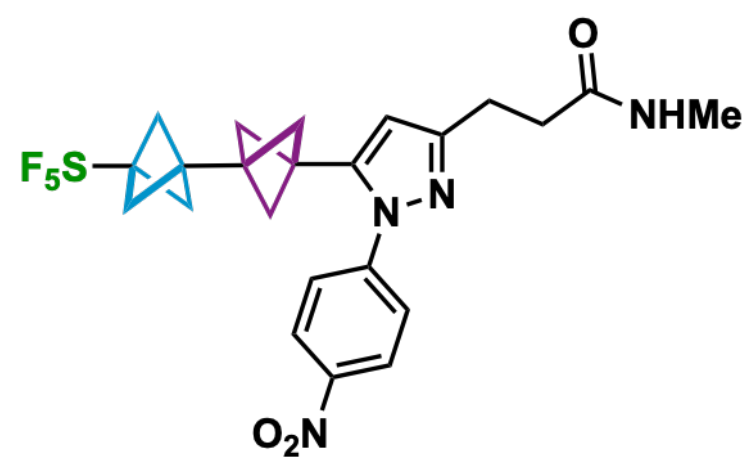

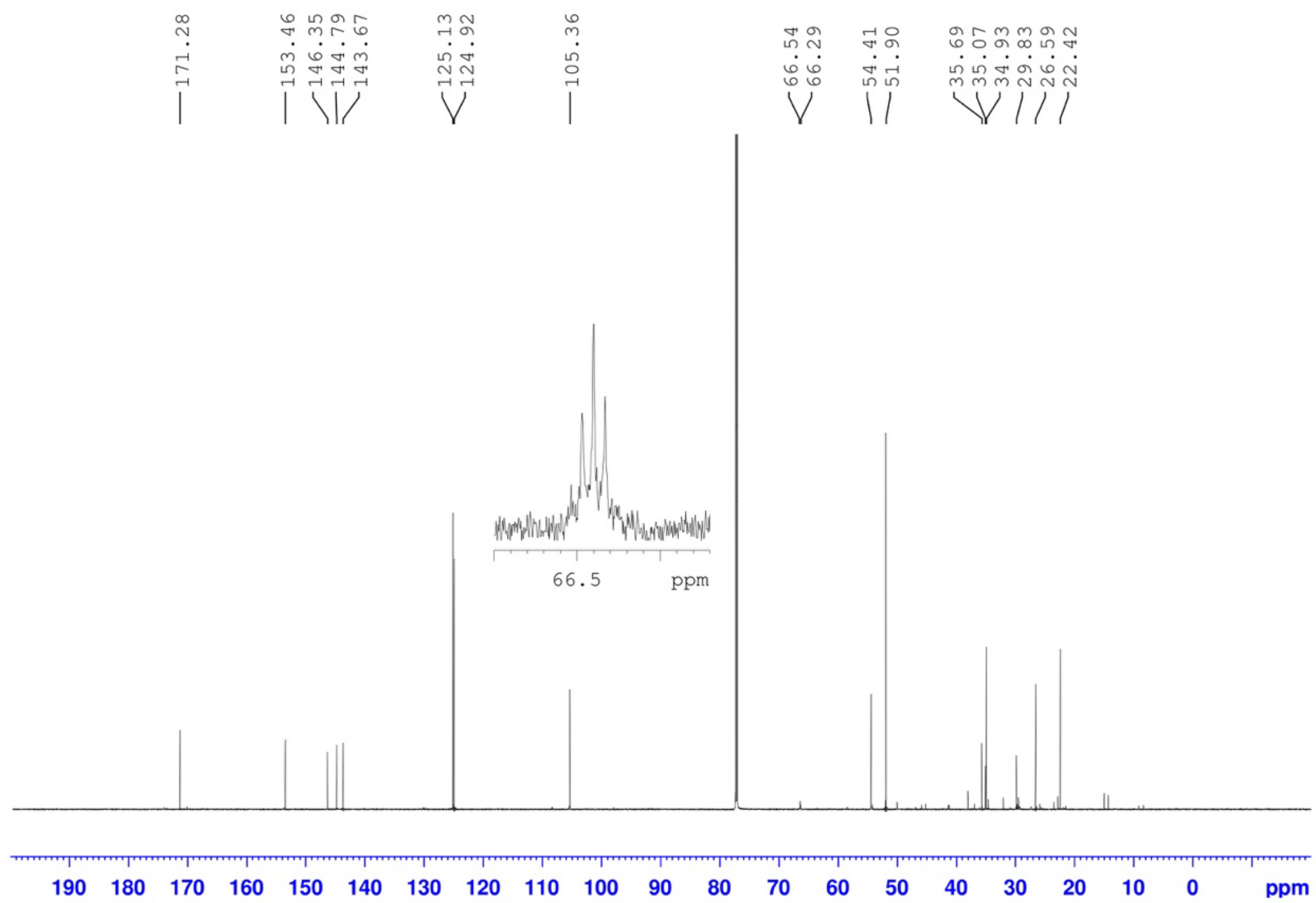

Figure S351. $^{13}C\{^1H\}$ NMR spectrum of *N*-methyl-3-(1-(4-nitrophenyl)-5-(3'-(pentafluoro-λ^6-sulfaneyl)-[1,1'-bi(bicyclo[1.1.1]pentan)]-3-yl)-1*H*-pyrazol-3-yl)propanamide (**S-39**) in $CDCl_3$.

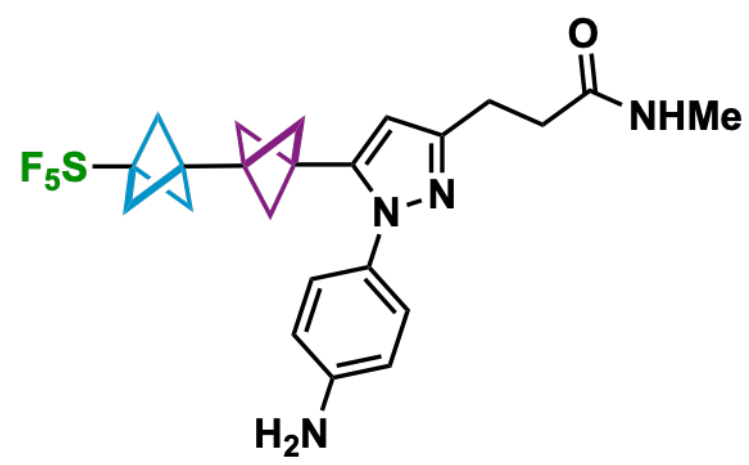

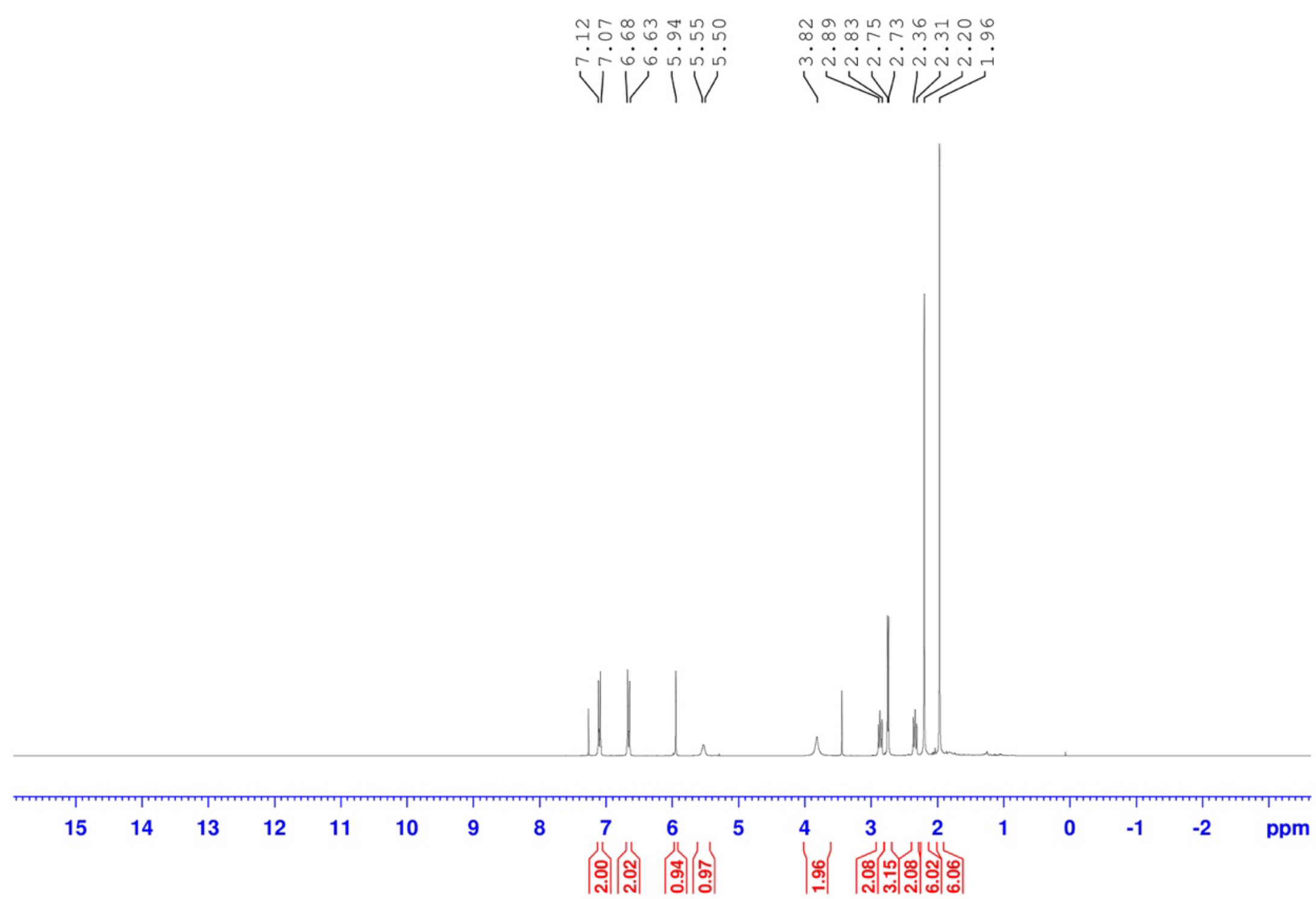

Figure S352. ^{1}H NMR spectrum of 3-(1-(4-aminophenyl)-5-(3'-(pentafluoro-λ^6-sulfaneyl)-[1,1'-bi(bicyclo[1.1.1]pentan)]-3-yl)-1*H*-pyrazol-3-yl)-*N*-methylpropanamide (**S-40**) in $CDCl_3$.

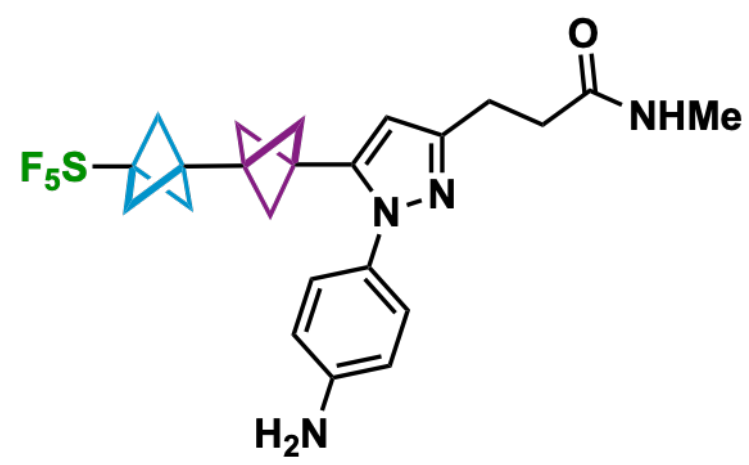

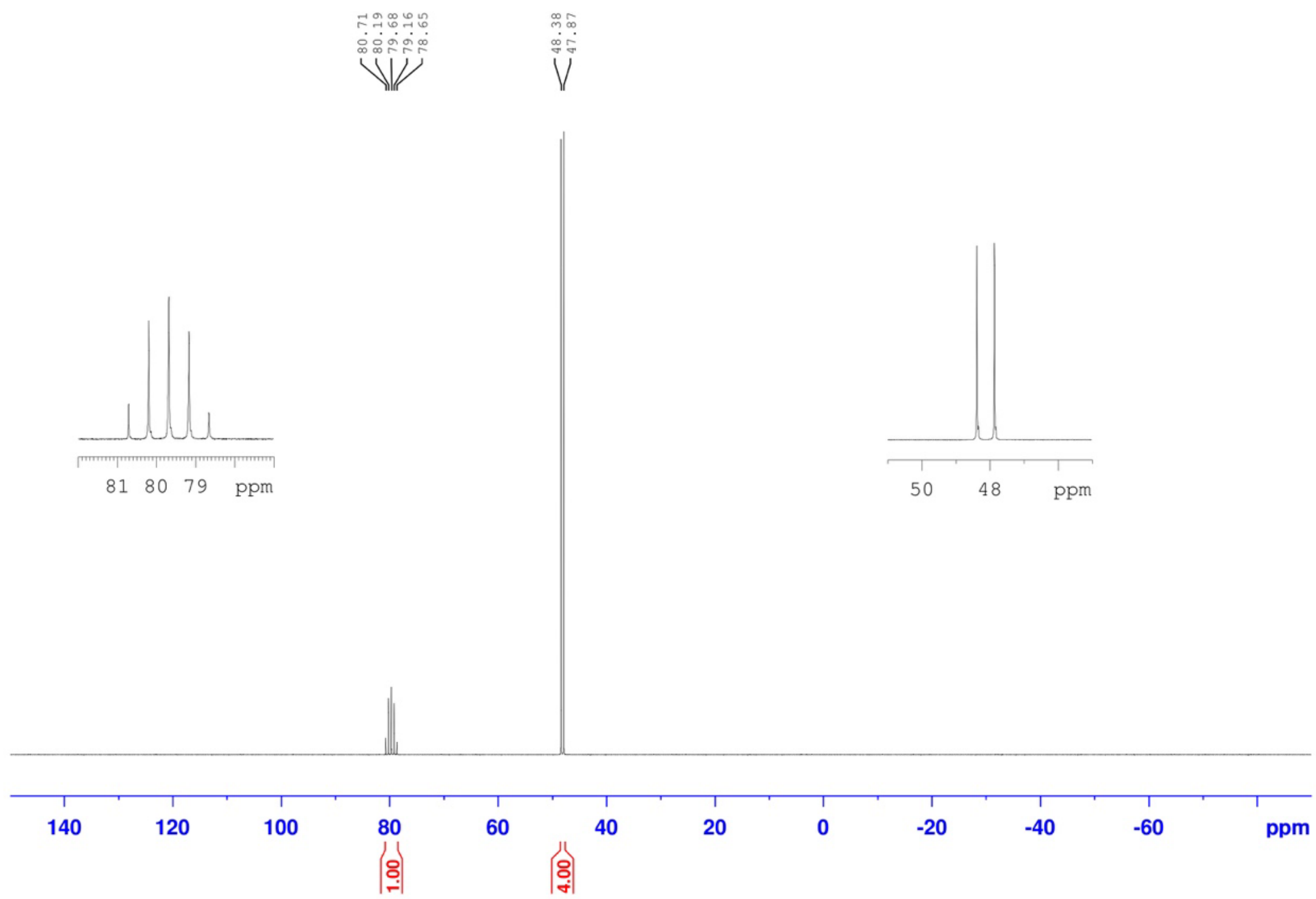

Figure S353. ^{19}F NMR spectrum of 3-(1-(4-aminophenyl)-5-(3'-(pentafluoro-λ^6-sulfaneyl)-[1,1'-bi(bicyclo[1.1.1]pentan)]-3-yl)-1*H*-pyrazol-3-yl)-*N*-methylpropanamide (**S-40**) in $CDCl_3$.

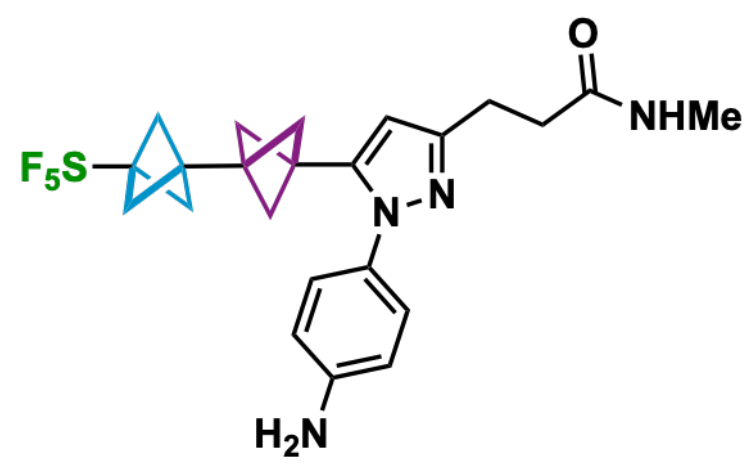

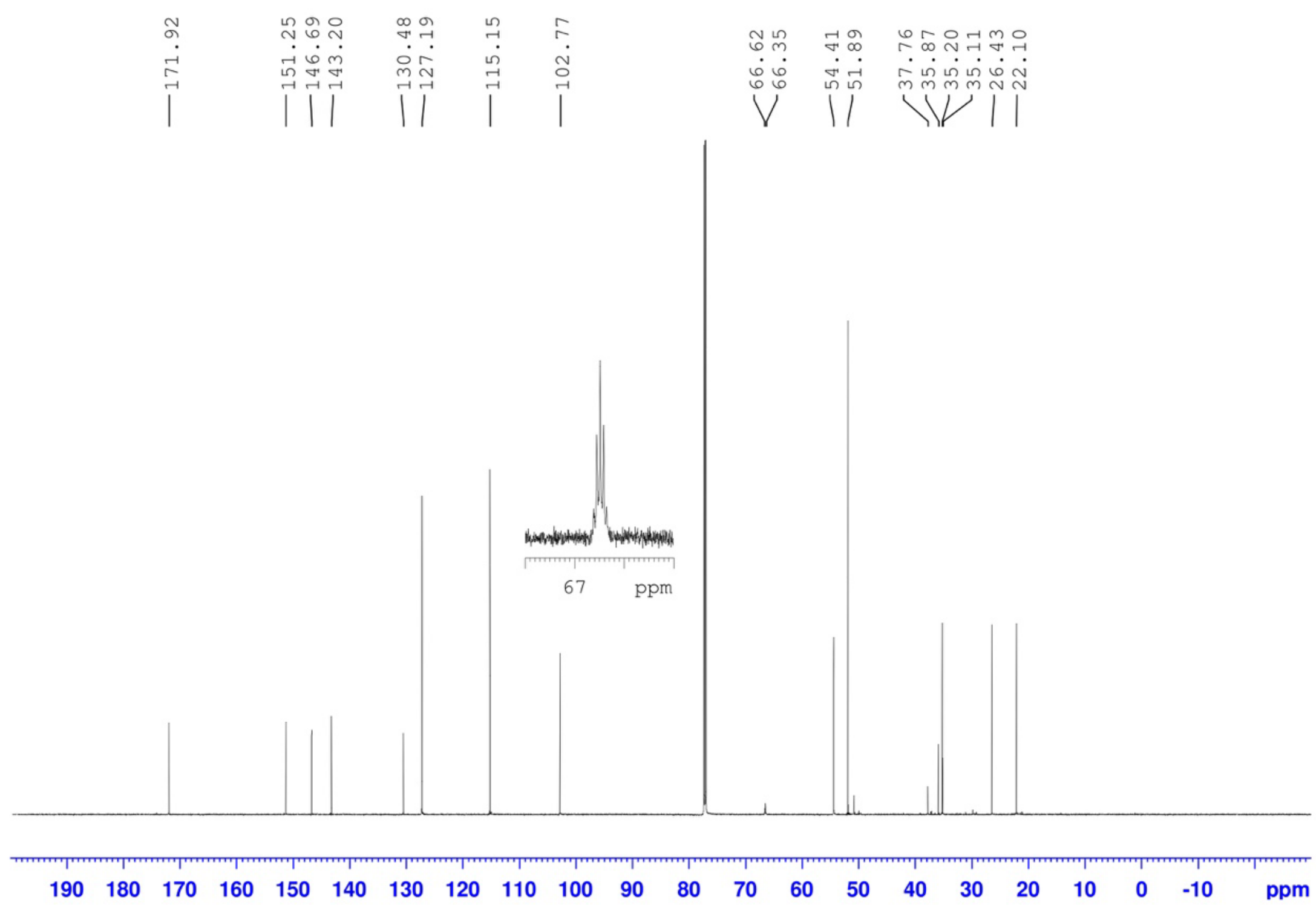

Figure S354. $^{13}C\{^1H\}$ NMR spectrum of 3-(1-(4-aminophenyl)-5-(3'-(pentafluoro-λ^6-sulfaneyl)-[1,1'-bi(bicyclo[1.1.1]pentan)]-3-yl)-1*H*-pyrazol-3-yl)-*N*-methylpropanamide (**S-40**) in $CDCl_3$.

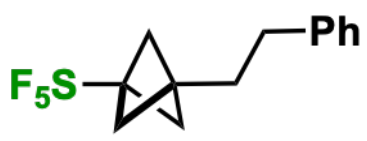

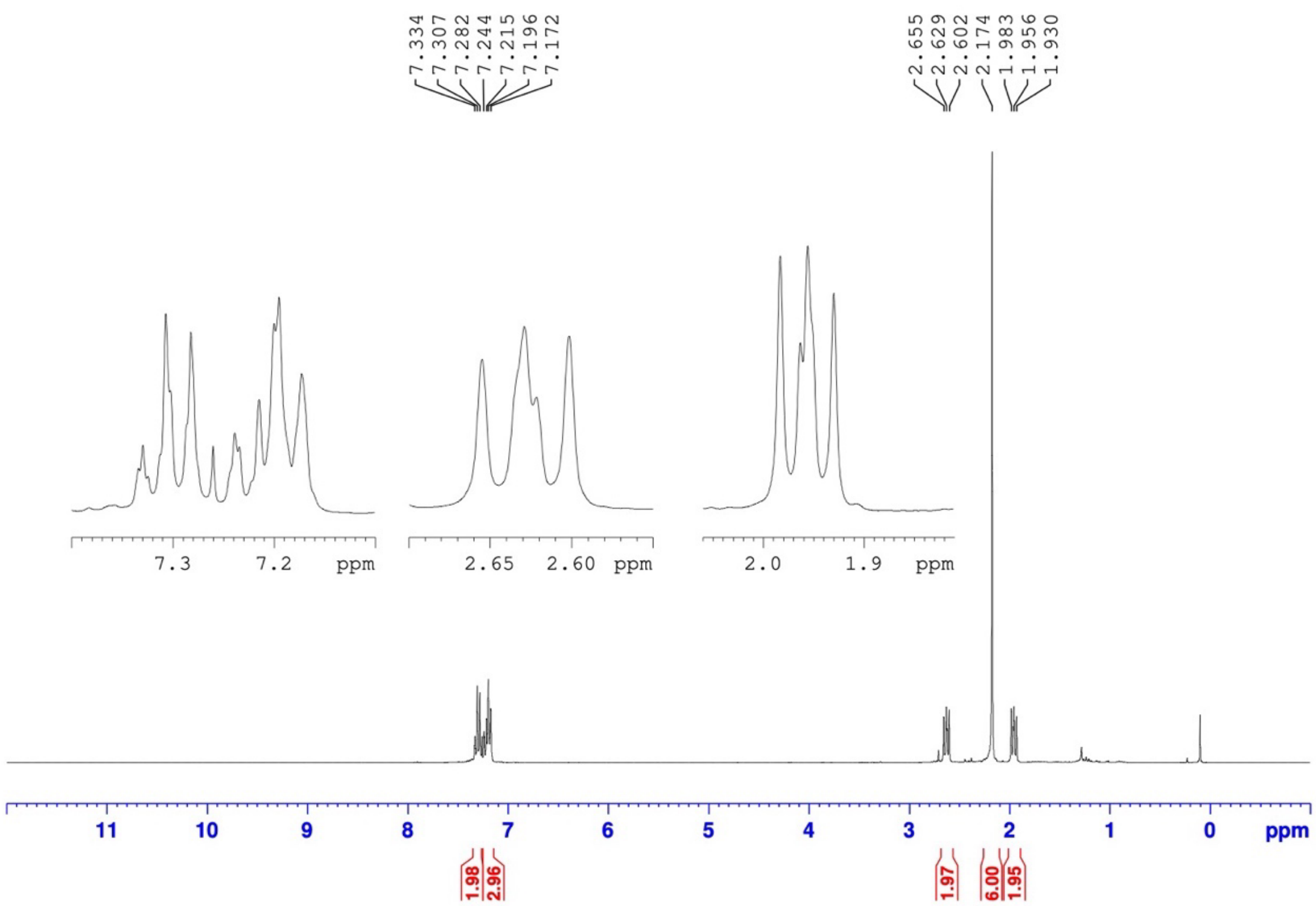

Figure S355.^{1}H NMR spectrum of pentafluoro(3-phenethylbicyclo[1.1.1]pentan-1-yl)- λ^6-sulfane (**S-41**) in $CDCl_3$.

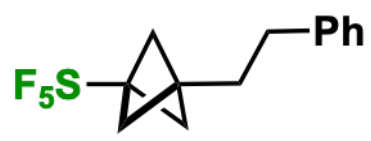

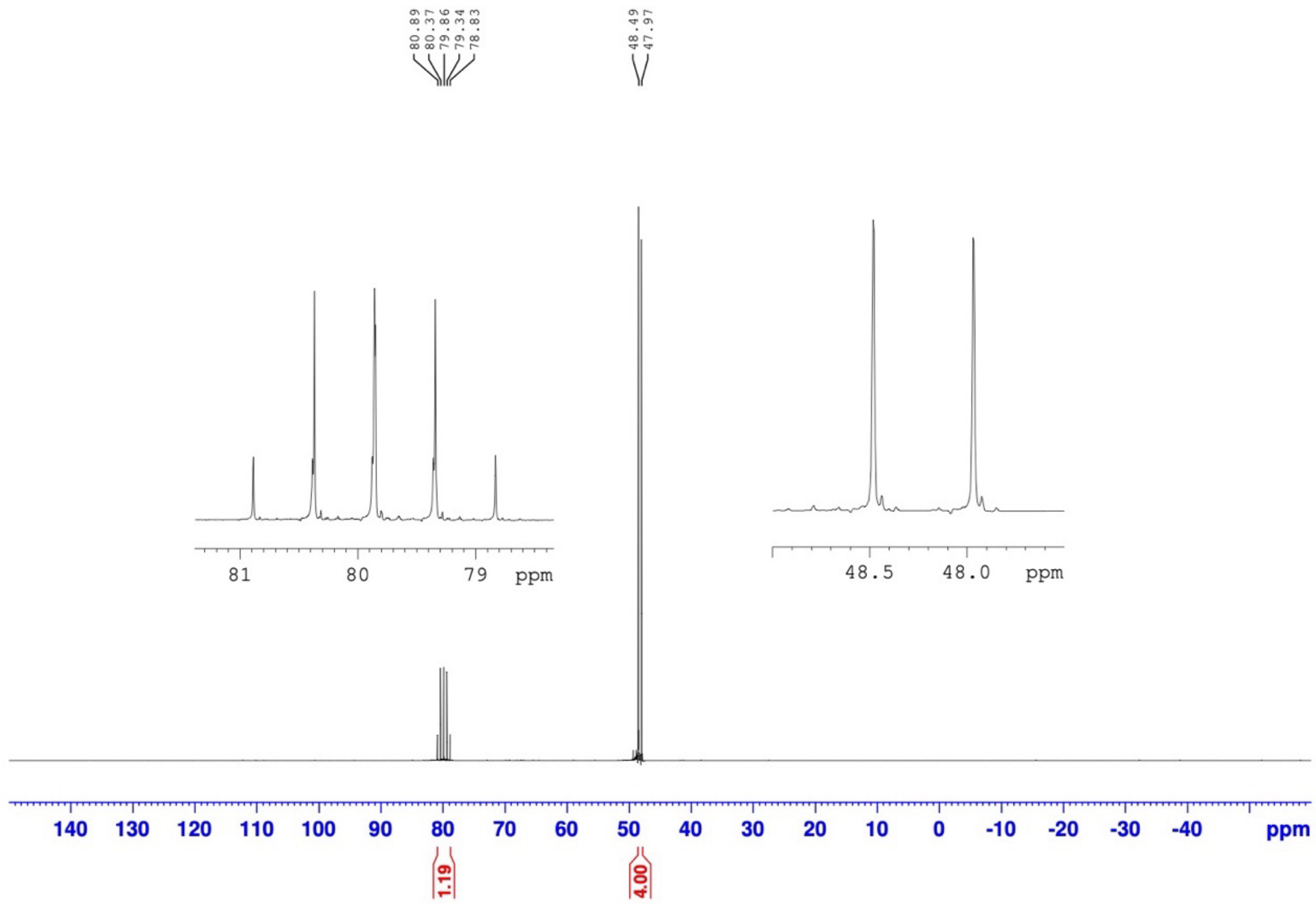

Figure S356. ^{19}F NMR spectrum of pentafluoro(3-phenethylbicyclo[1.1.1]pentan-1-yl)- λ^6-sulfane (**S-41**) in $CDCl_3$.

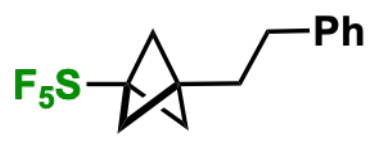

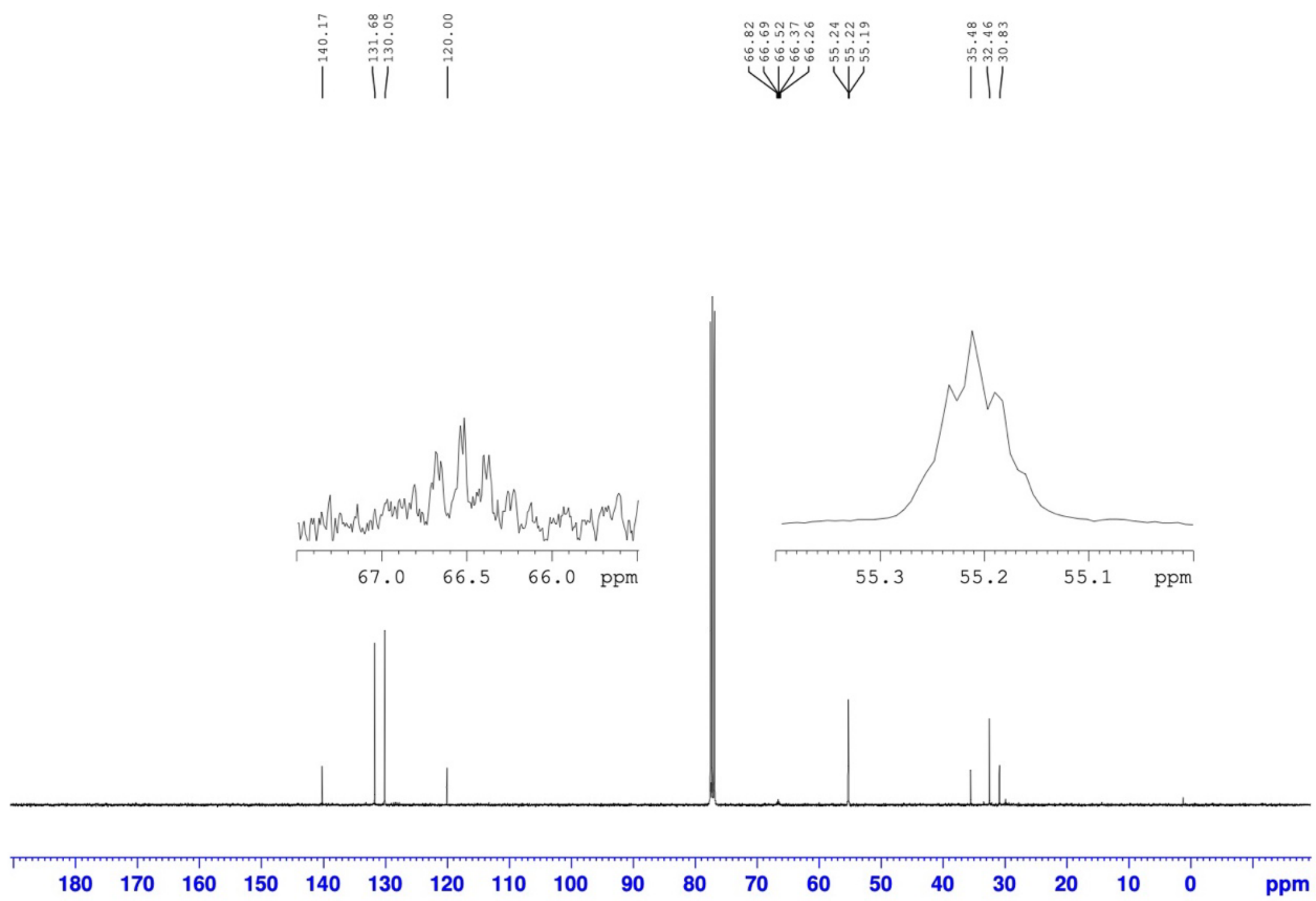

Figure S357. $^{13}C\{^{1}H\}$ NMR spectrum of pentafluoro(3-phenethylbicyclo[1.1.1]pentan-1-yl)- λ^6-sulfane (**S-41**) in $CDCl_3$.

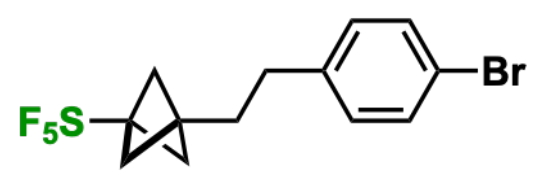

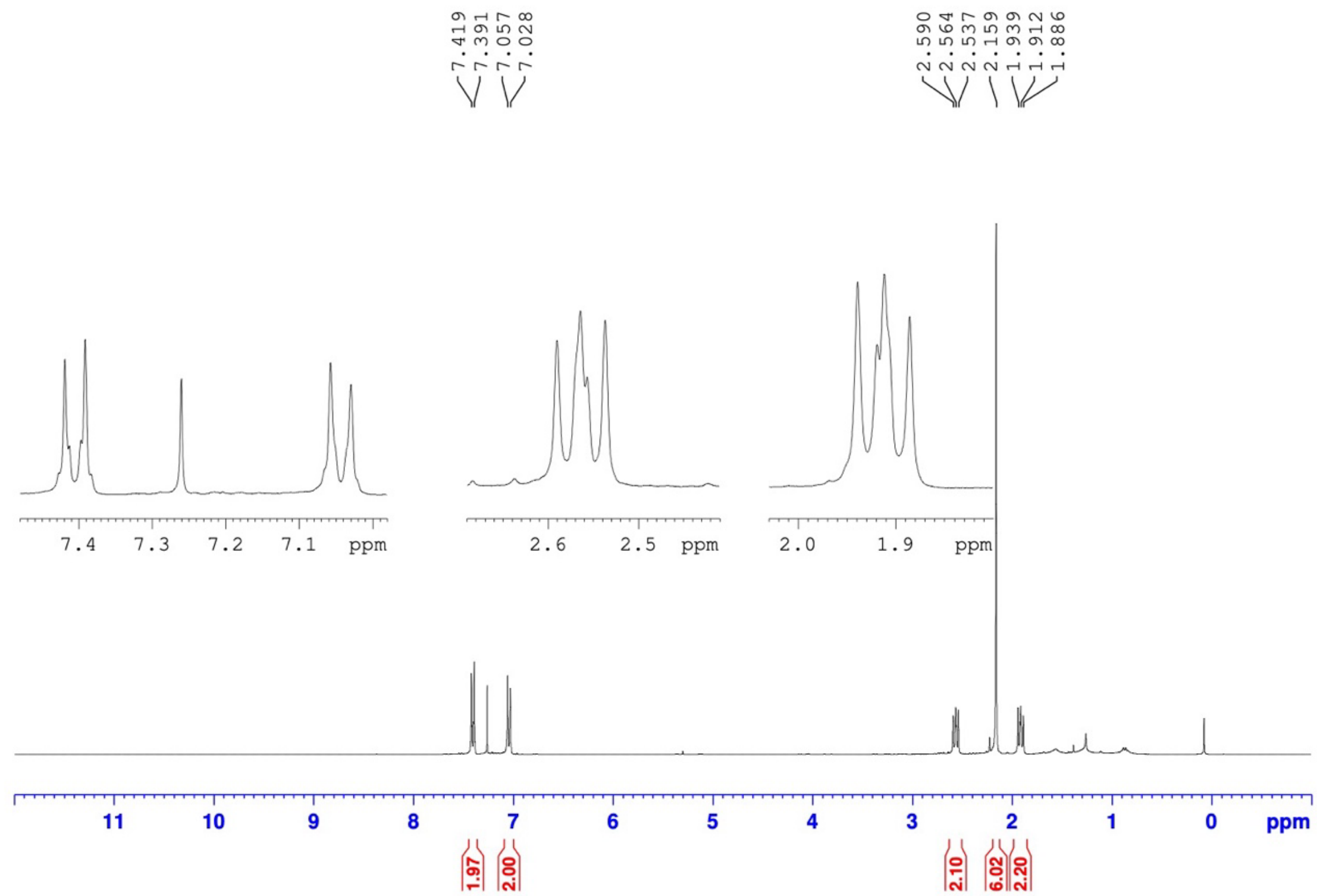

Figure S358. ^{1}H NMR spectrum of (3-(4-bromophenethyl)bicyclo[1.1.1]pentan-1-yl)pentafluoro-λ^{6}-sulfane (**S-42**) in $CDCl_3$.

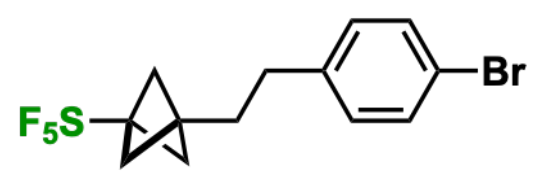

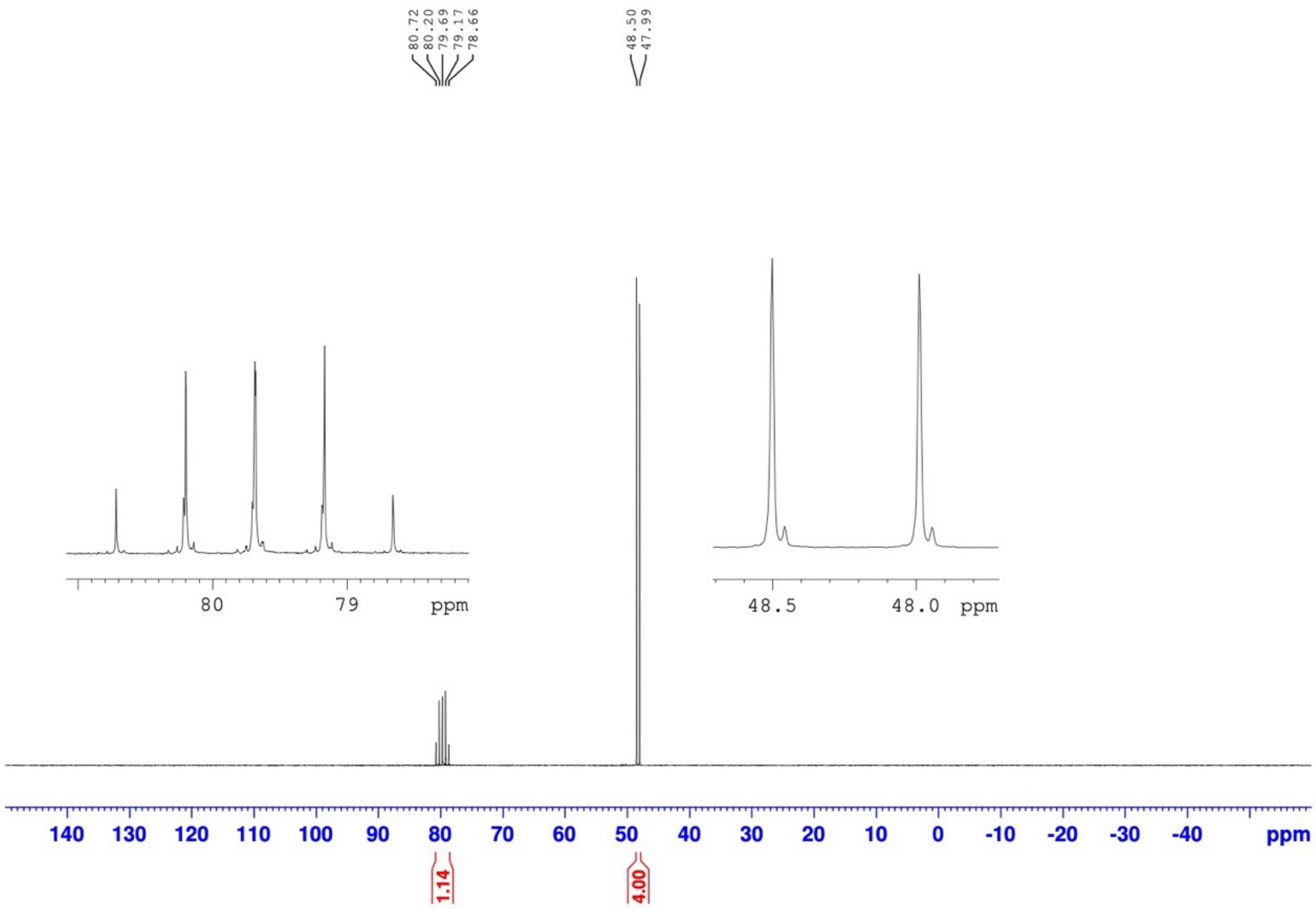

Figure S359. ^{19}F NMR spectrum of (3-(4-bromophenethyl)bicyclo[1.1.1]pentan-1-yl)pentafluoro-λ^6-sulfane (**S-42**) in $CDCl_3$.

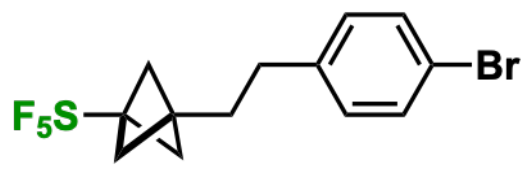

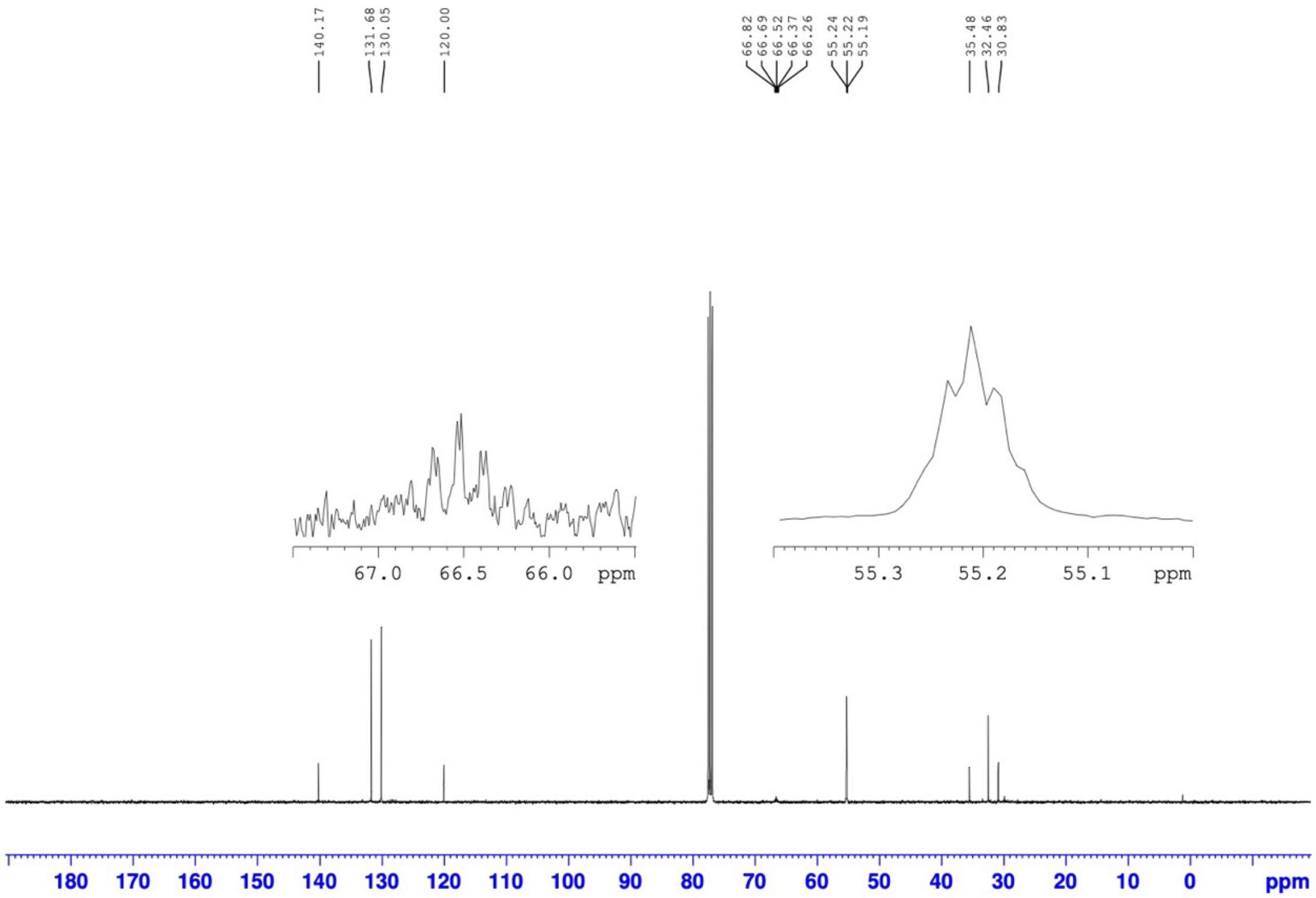

Figure S360. $^{13}C\{^1H\}$ NMR spectrum of (3-(4-bromophenethyl)bicyclo[1.1.1]pentan-1-yl)pentafluoro-λ^6-sulfane (**S-42**) in $CDCl_3$.

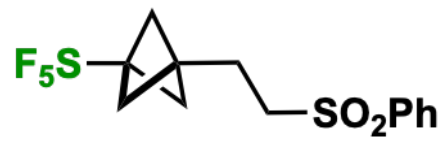

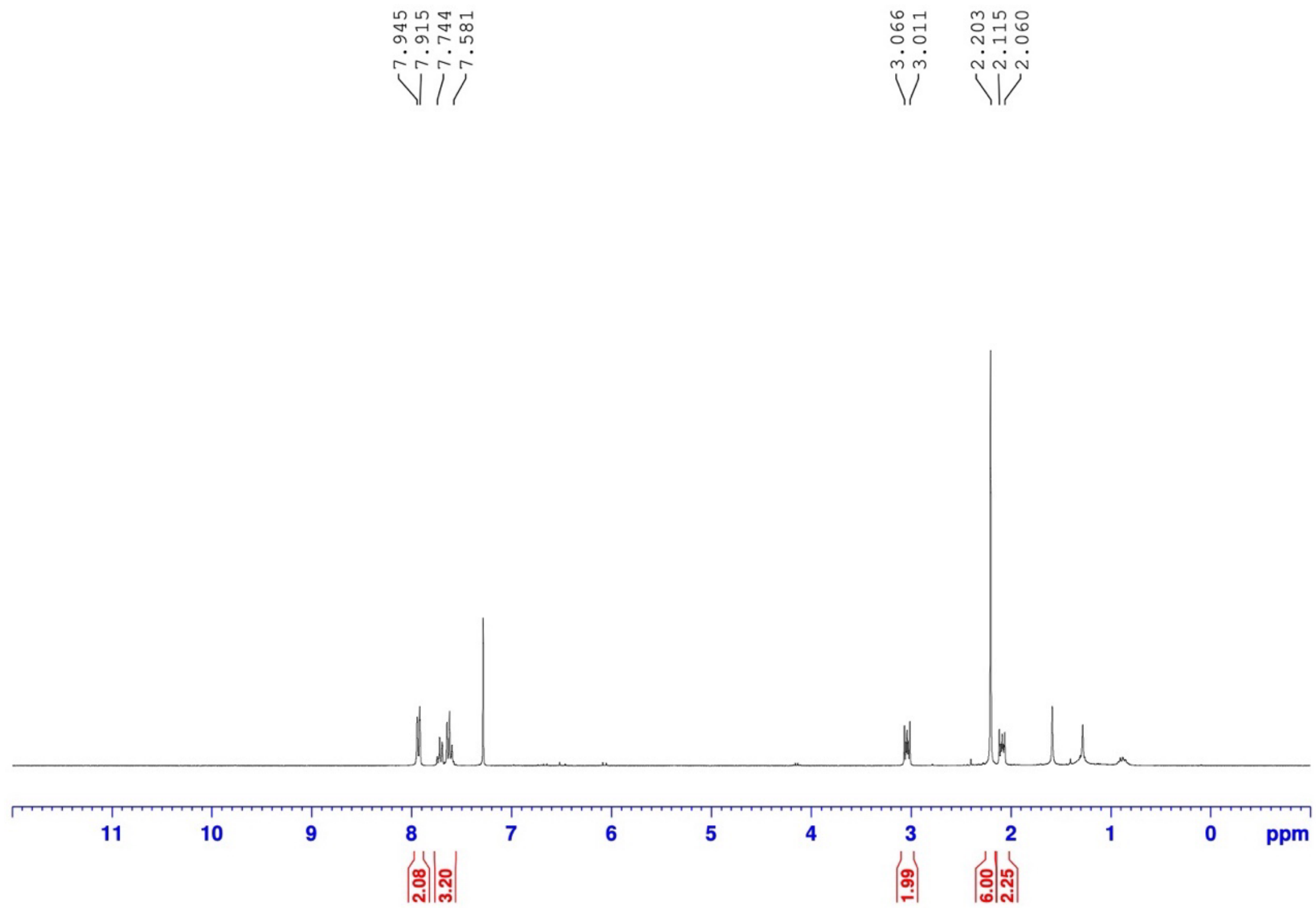

Figure S361. ^{1}H NMR spectrum of (pentafluoro(3-(2-(phenylsulfonyl)ethyl)bicyclo[1.1.1]pentan-1-yl)-λ^6-sulfane (**S-43**) in $CDCl_3$.

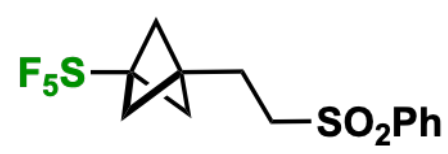

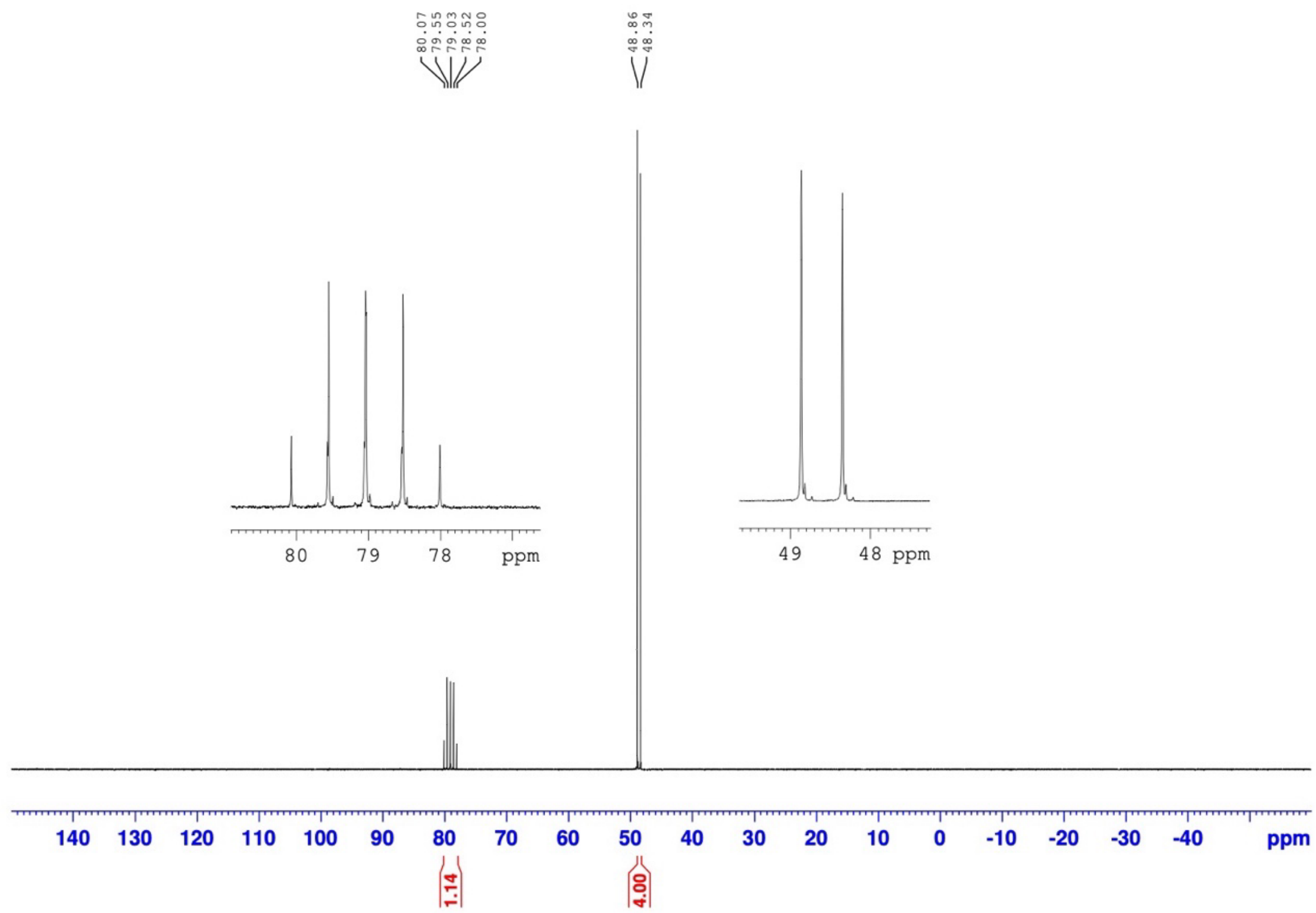

Figure S362. ^{19}F NMR spectrum of (pentafluoro(3-(2-(phenylsulfonyl)ethyl)bicyclo[1.1.1]pentan-1-yl)-λ^6-sulfane (**S-43**) in $CDCl_3$.

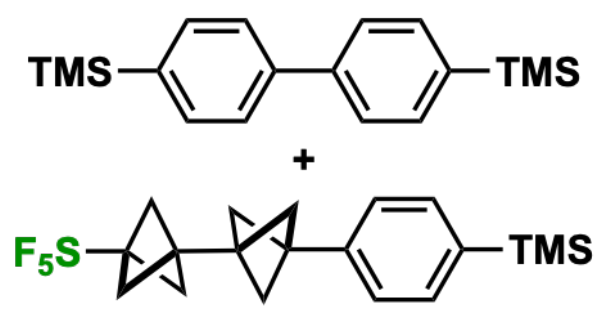

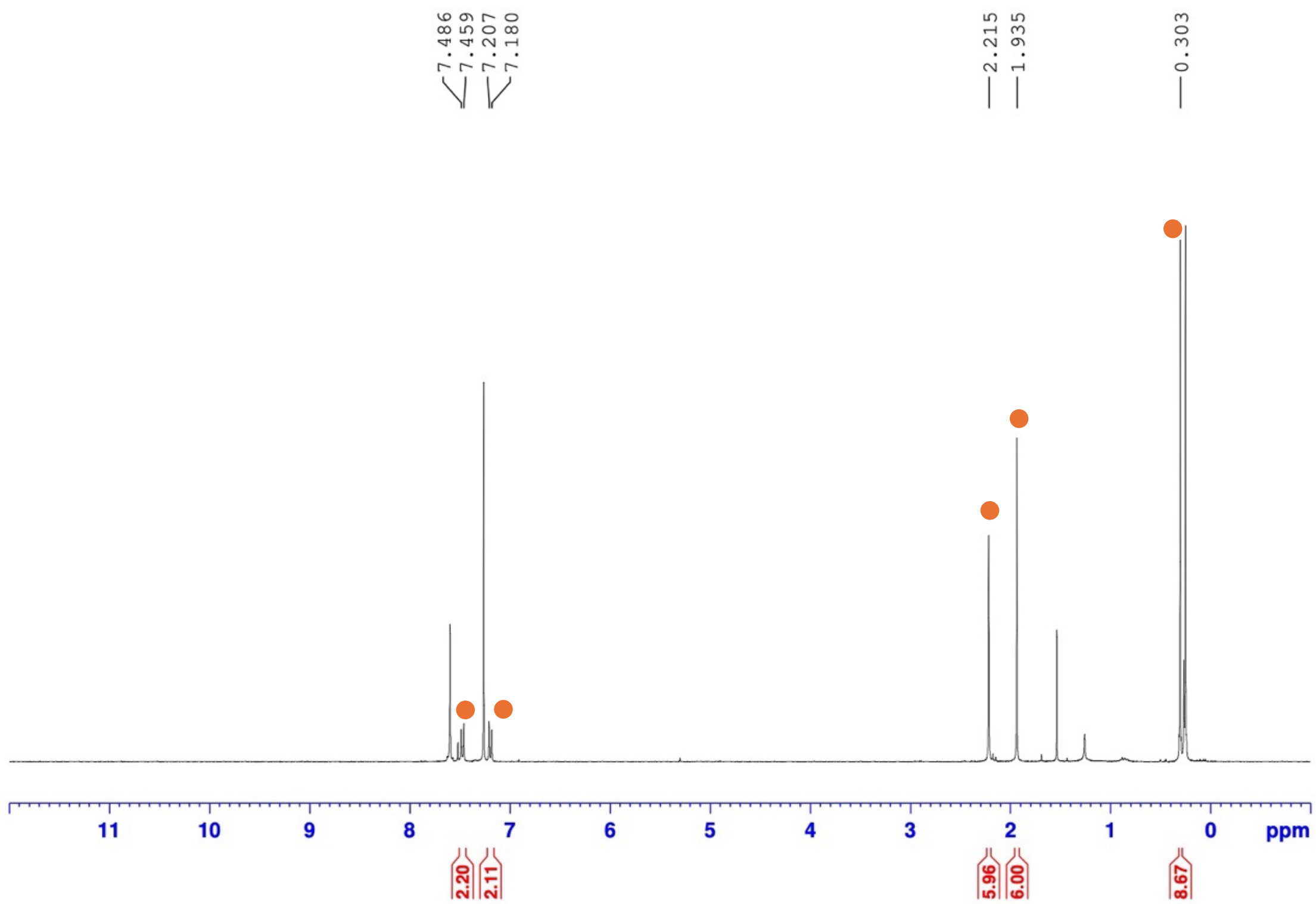

Figure S363. ^{1}H NMR spectrum of a mixture containing (trimethyl(4-(3'-(pentafluoro-λ^6-sulfanyl)-[1,1'-bi(bicyclo[1.1.1]pentan)]-3-yl)phenyl)silane (**S-45**) in $CDCl_3$. (**S-45** peaks highlighted with orange dots).

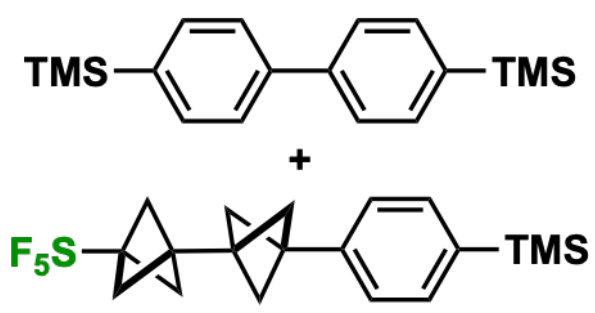

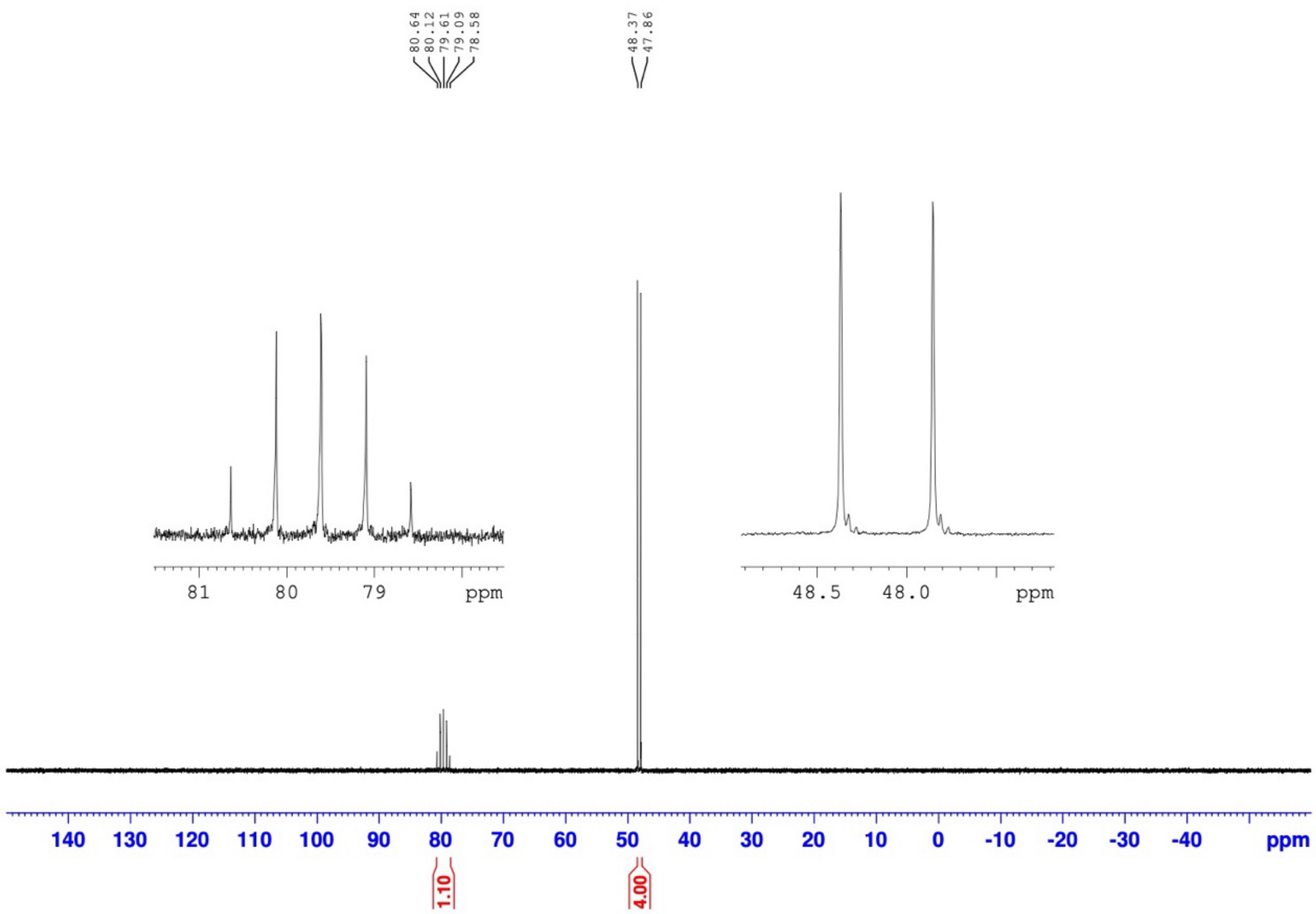

Figure S364. ^{19}F NMR spectrum of a mixture containing (trimethyl(4-(3'-(pentafluoro-λ^6-sulfanyl)-[1,1'-bi(bicyclo[1.1.1]pentan)]-3-yl)phenyl)silane (**S-45**) in $CDCl_3$.

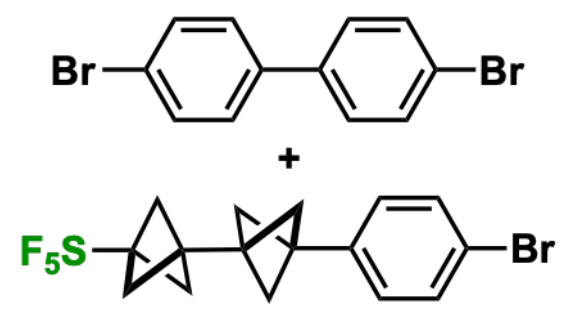

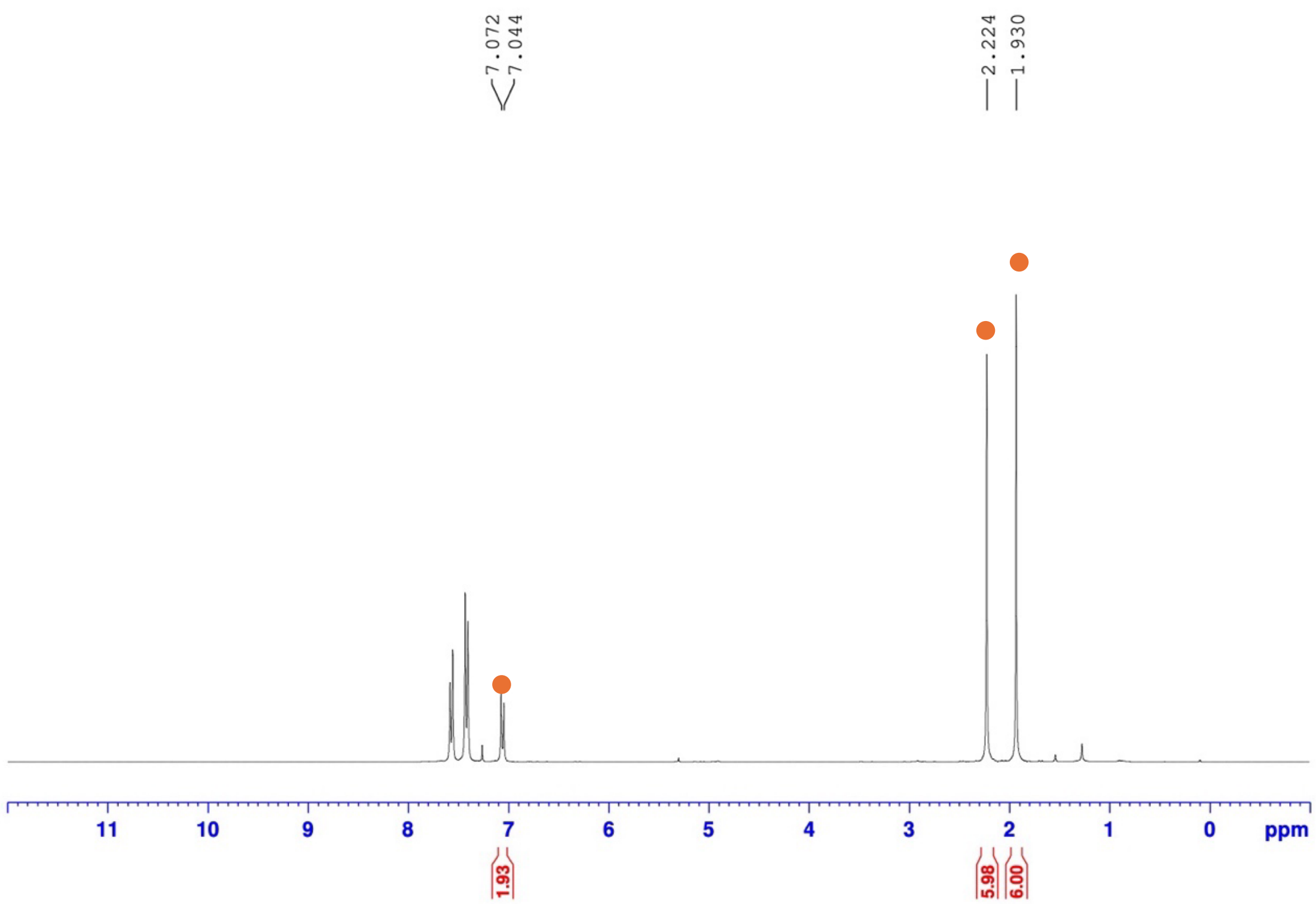

Figure S365. ^{1}H NMR spectrum of a mixture containing (3'-(4-bromophenyl)-[1,1'-bi(bicyclo[1.1.1]pentan)]-3-yl)pentafluoro-λ[6]-sulfane (**S-46**) in $CDCl_3$. (**S-46** peaks highlighted with orange dots).

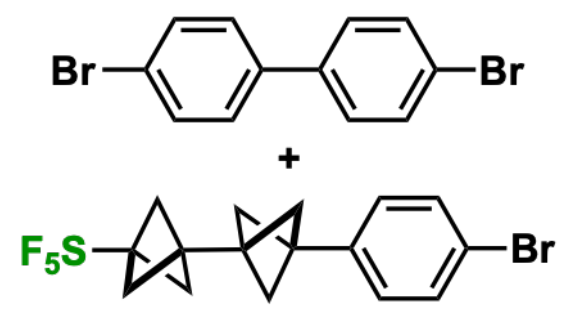

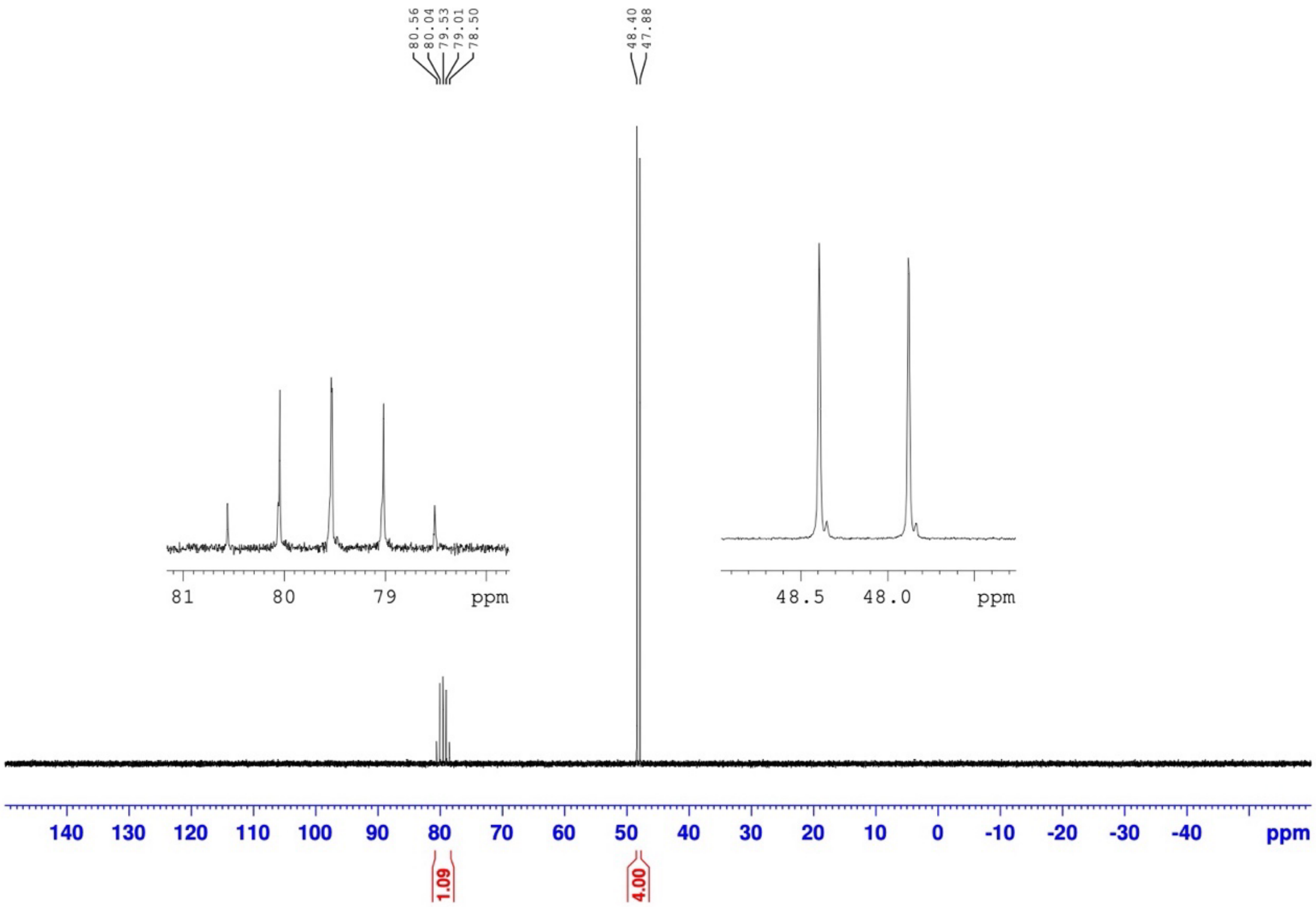

Figure S366. ^{19}F NMR spectrum of a mixture containing (3'-(4-bromophenyl)-[1,1'-bi(bicyclo[1.1.1]pentan)]-3-yl)pentafluoro-λ^6-sulfane (**S-46**) in $CDCl_3$.

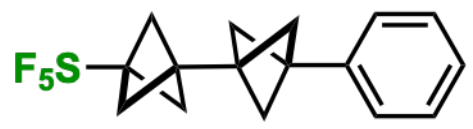

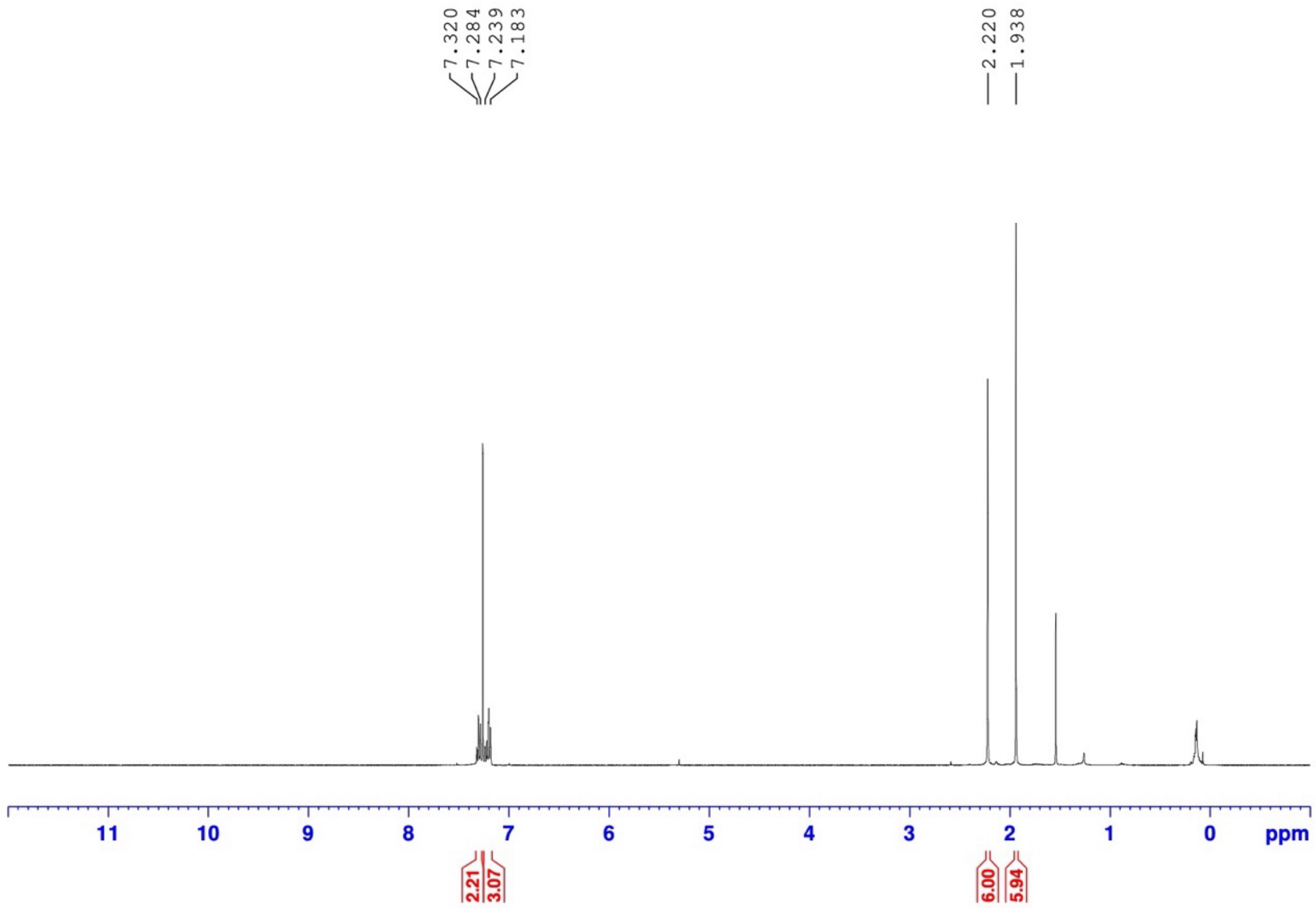

Figure S367. ^{1}H NMR spectrum of pentafluoro(3'-phenyl-[1,1'-bi(bicyclo[1.1.1]pentan)]-3-yl)- λ^6-sulfane (**S-47**) in $CDCl_3$.

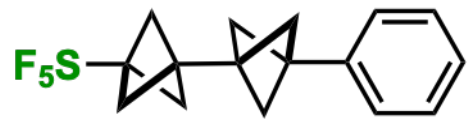

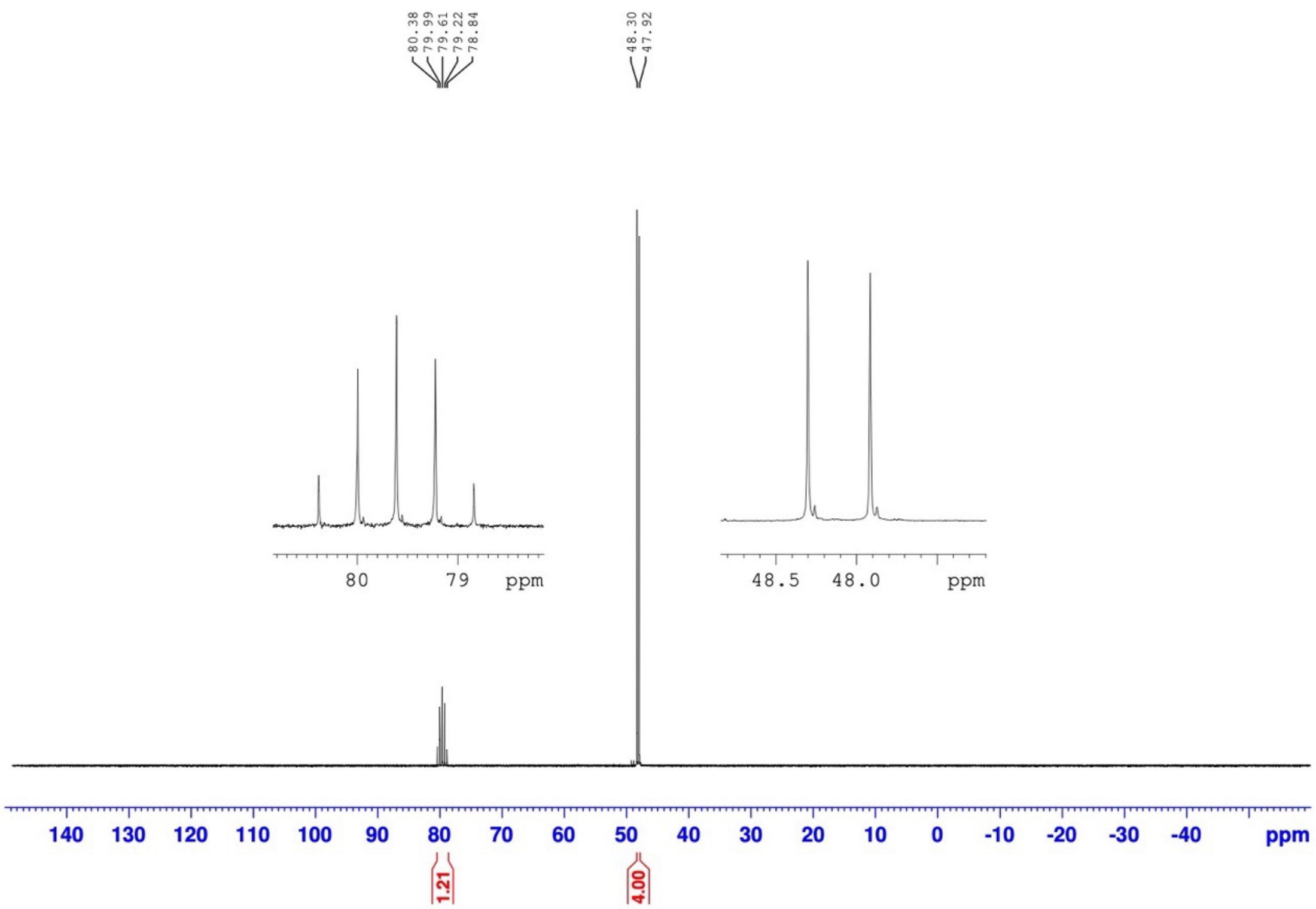

Figure S368. ^{19}F NMR spectrum of pentafluoro(3'-phenyl-[1,1'-bi(bicyclo[1.1.1]pentan)]-3-yl)- λ^6-sulfane (**S-47**) in $CDCl_3$.

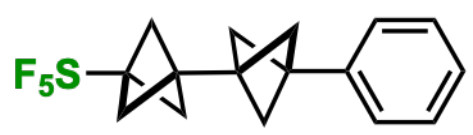

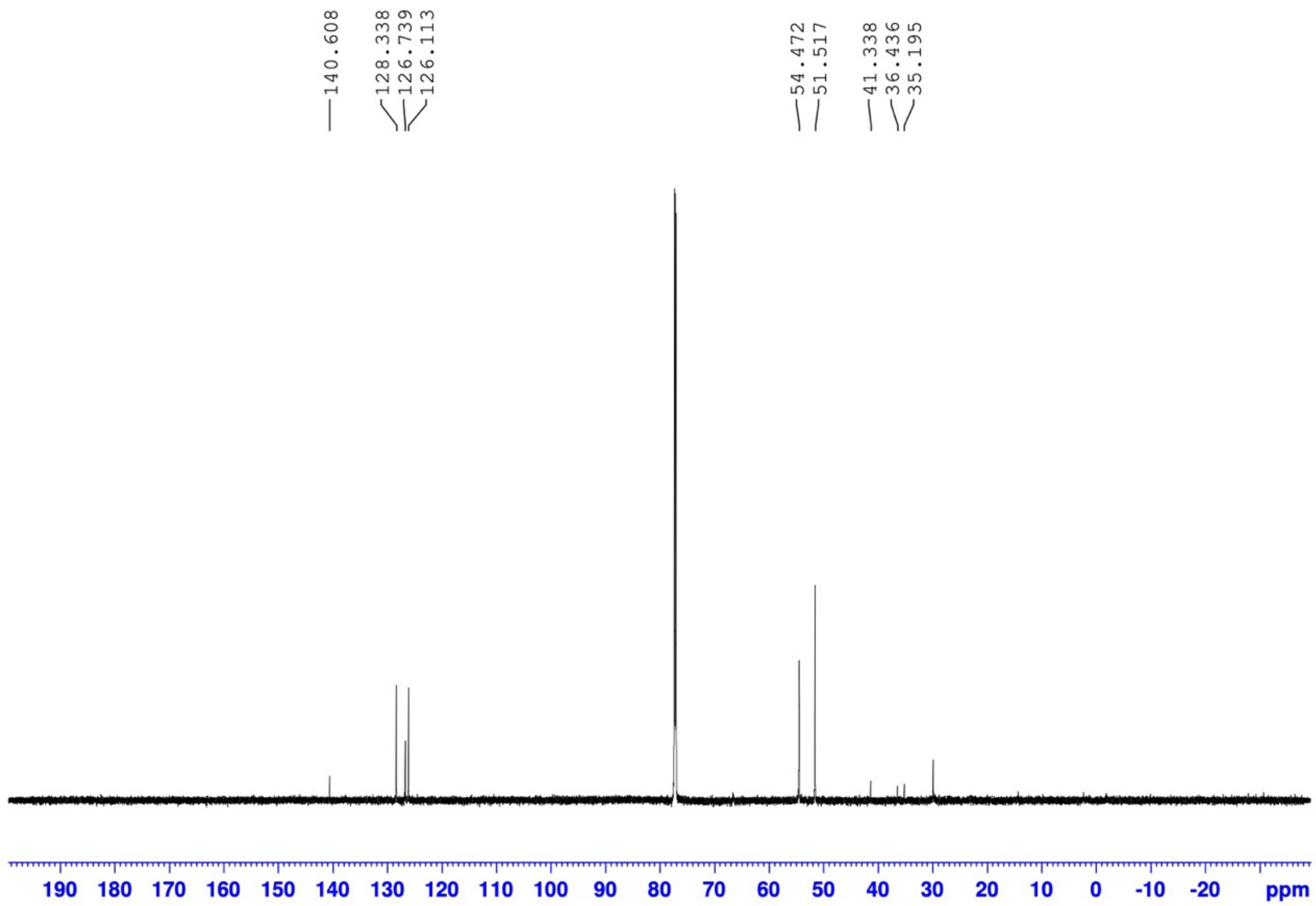

Figure S369. ^{13}C NMR spectrum of pentafluoro(3'-phenyl-[1,1'-bi(bicyclo[1.1.1]pentan)]-3-yl)- λ^6-sulfane (**S-47**) in $CDCl_3$.

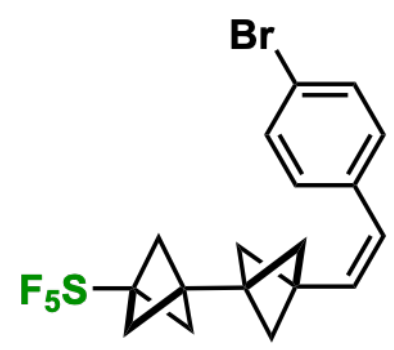

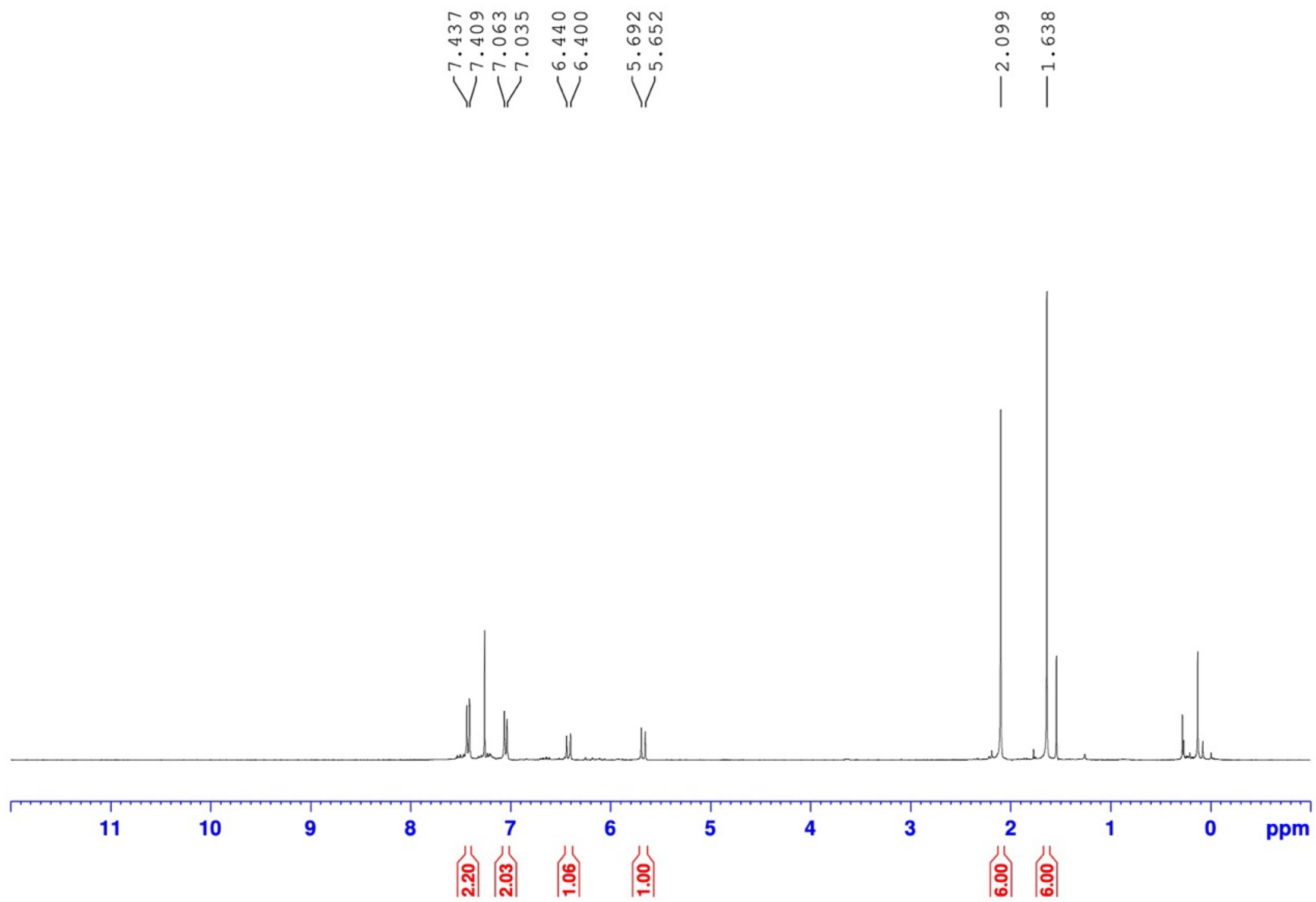

Figure S370. ^{1}H NMR spectrum (*Z*)-(3'-(4-bromostyryl)-[1,1'-bi(bicyclo[1.1.1]pentan)]-3-yl)pentafluoro-λ^6-sulfane (**S-48**) in $CDCl_3$.

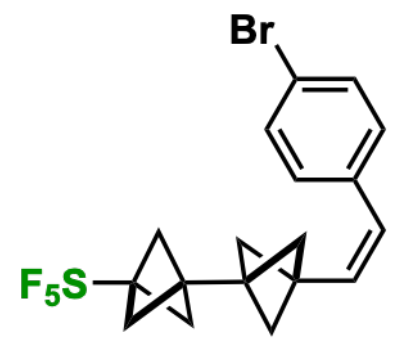

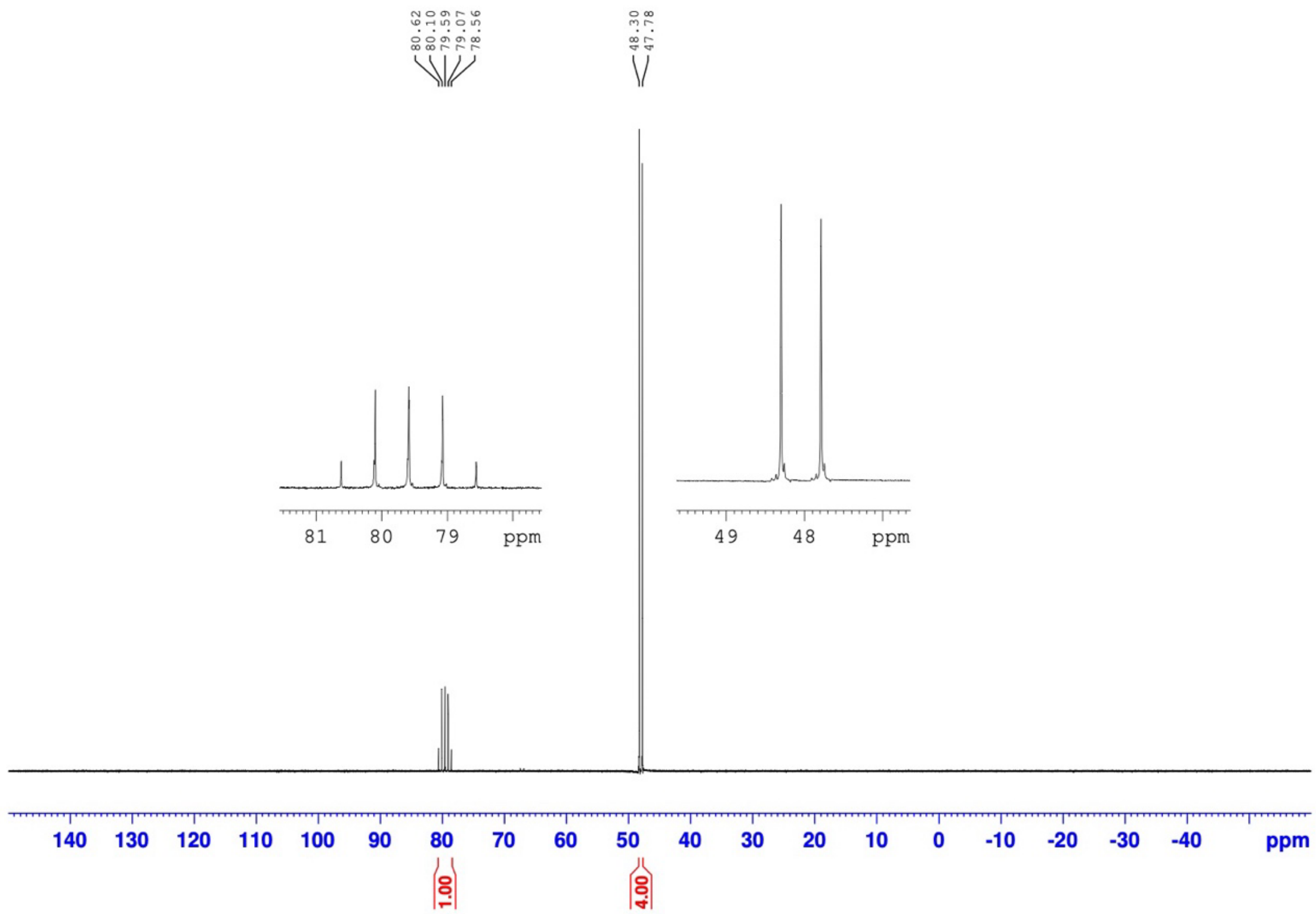

Figure S371. ^{19}F NMR spectrum (*Z*)-(3'-(4-bromostyryl)-[1,1'-bi(bicyclo[1.1.1]pentan)]-3-yl)pentafluoro-λ^6-sulfane (**S-48**) in $CDCl_3$.

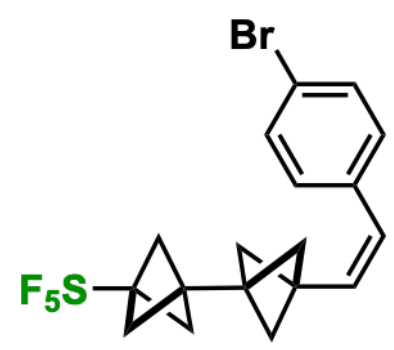

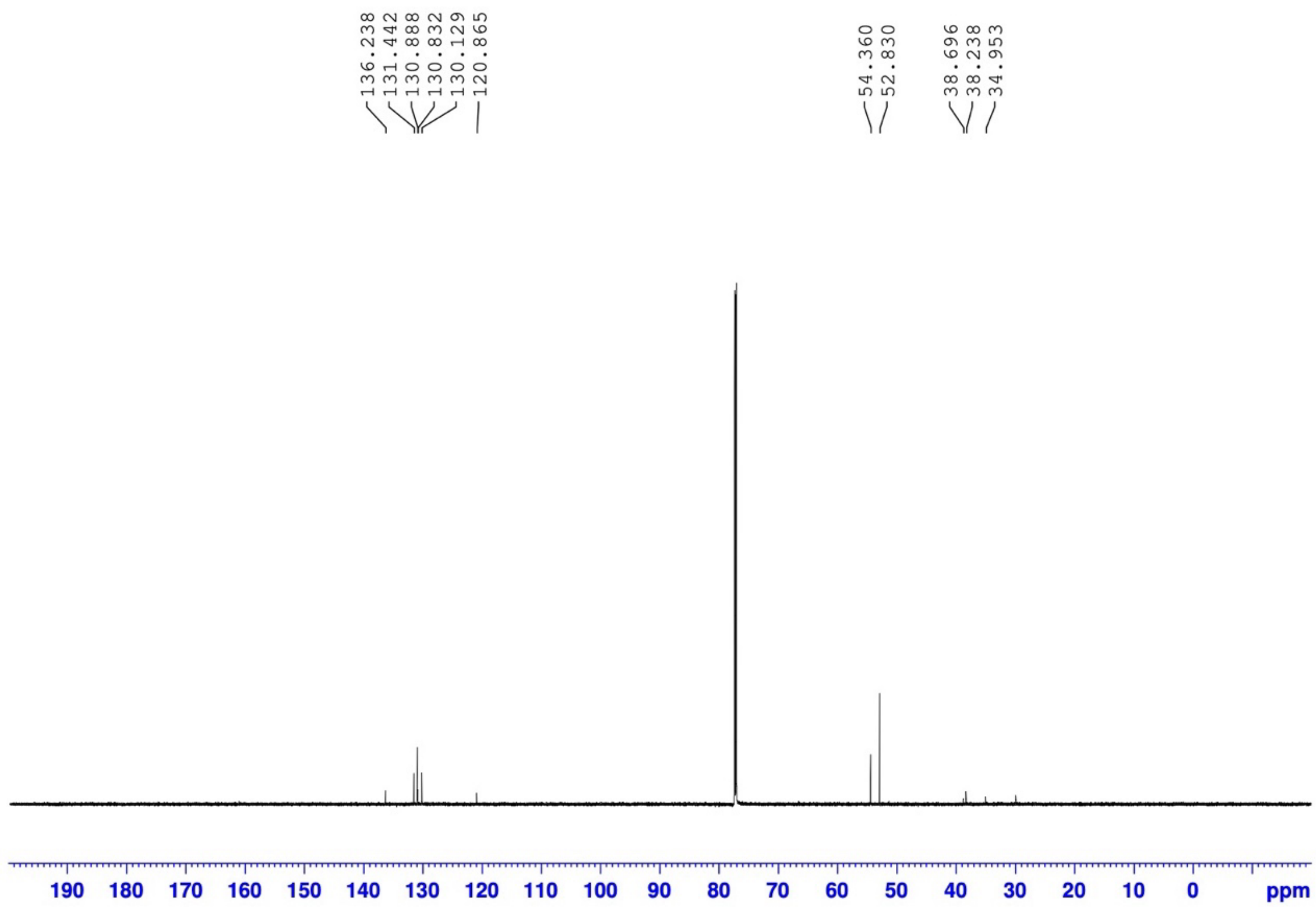

Figure S372. ^{13}C NMR spectrum (*Z*)-(3'-(4-bromostyryl)-[1,1'-bi(bicyclo[1.1.1]pentan)]-3-yl)pentafluoro-λ^6-sulfane (**S-48**) in $CDCl_3$.

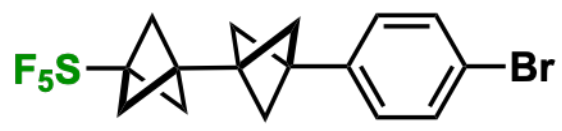

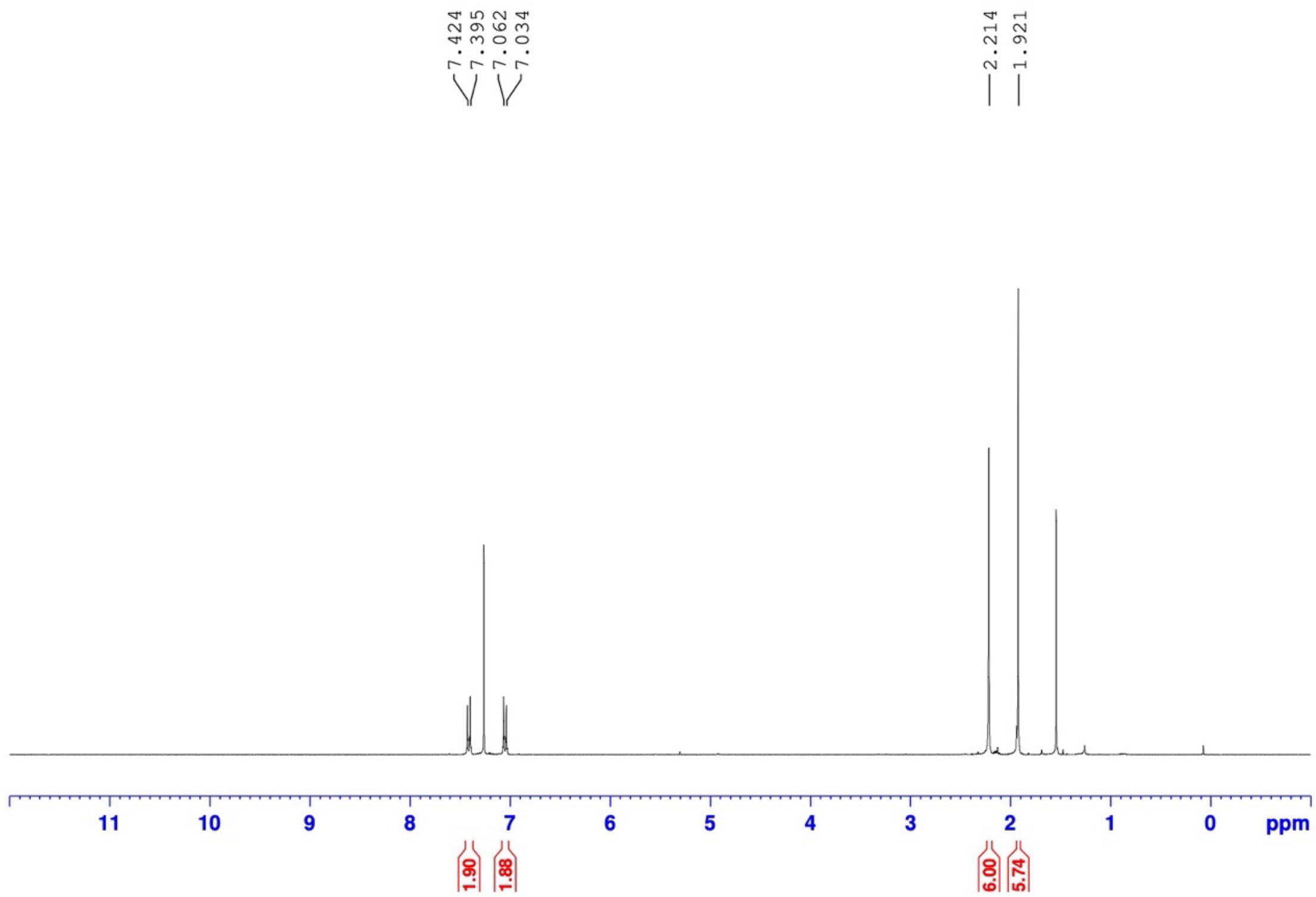

Figure S373. ^{1}H NMR spectrum of (3'-(4-bromophenyl)-[1,1'-bi(bicyclo[1.1.1]pentan)]-3-yl)pentafluoro-λ^6-sulfane (**S-49**) in $CDCl_3$.

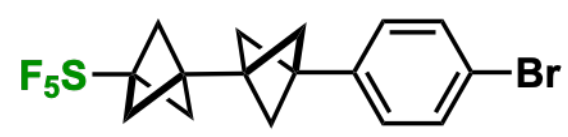

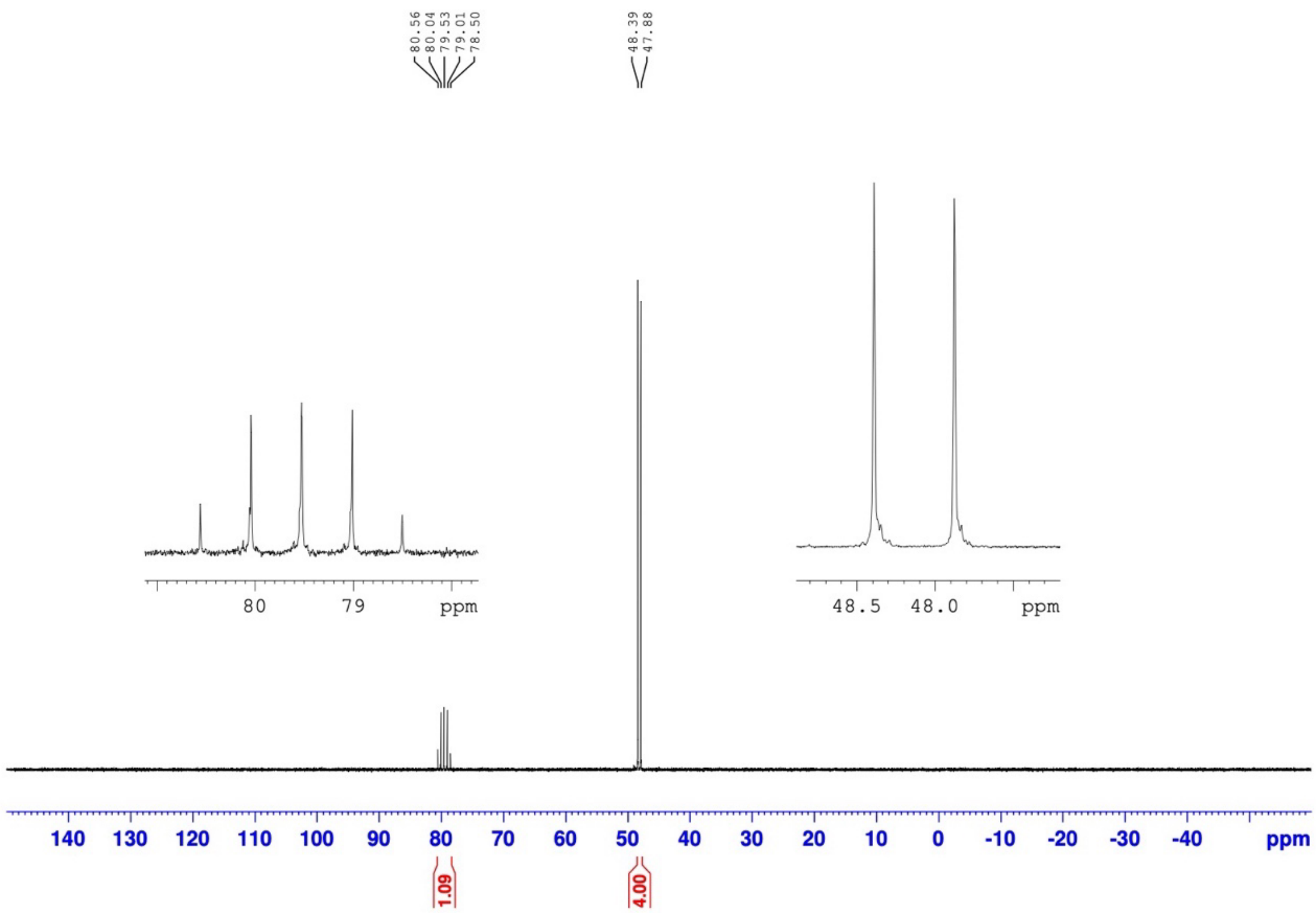

Figure S374. ^{19}F NMR spectrum of (3'-(4-bromophenyl)-[1,1'-bi(bicyclo[1.1.1]pentan)]-3-yl)pentafluoro-λ^6-sulfane (**S-49**) in $CDCl_3$.

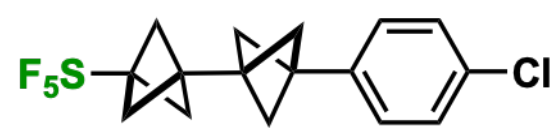

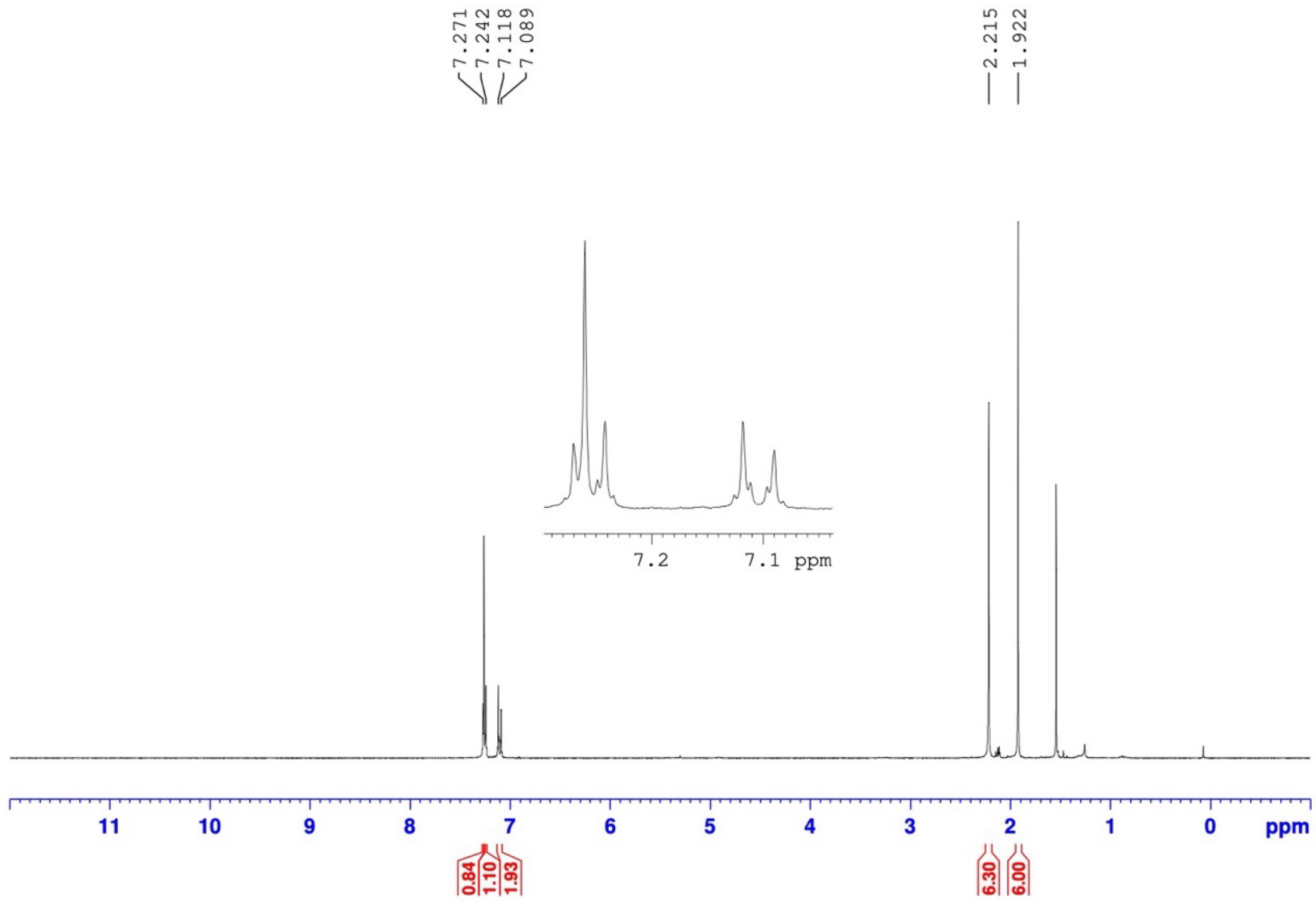

Figure S375. ^{1}H NMR spectrum of (3'-(4-chlorophenyl)-[1,1'-bi(bicyclo[1.1.1]pentan)]-3-yl)pentafluoro-λ^6-sulfane (**S-50**) in $CDCl_3$.

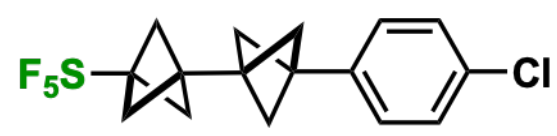

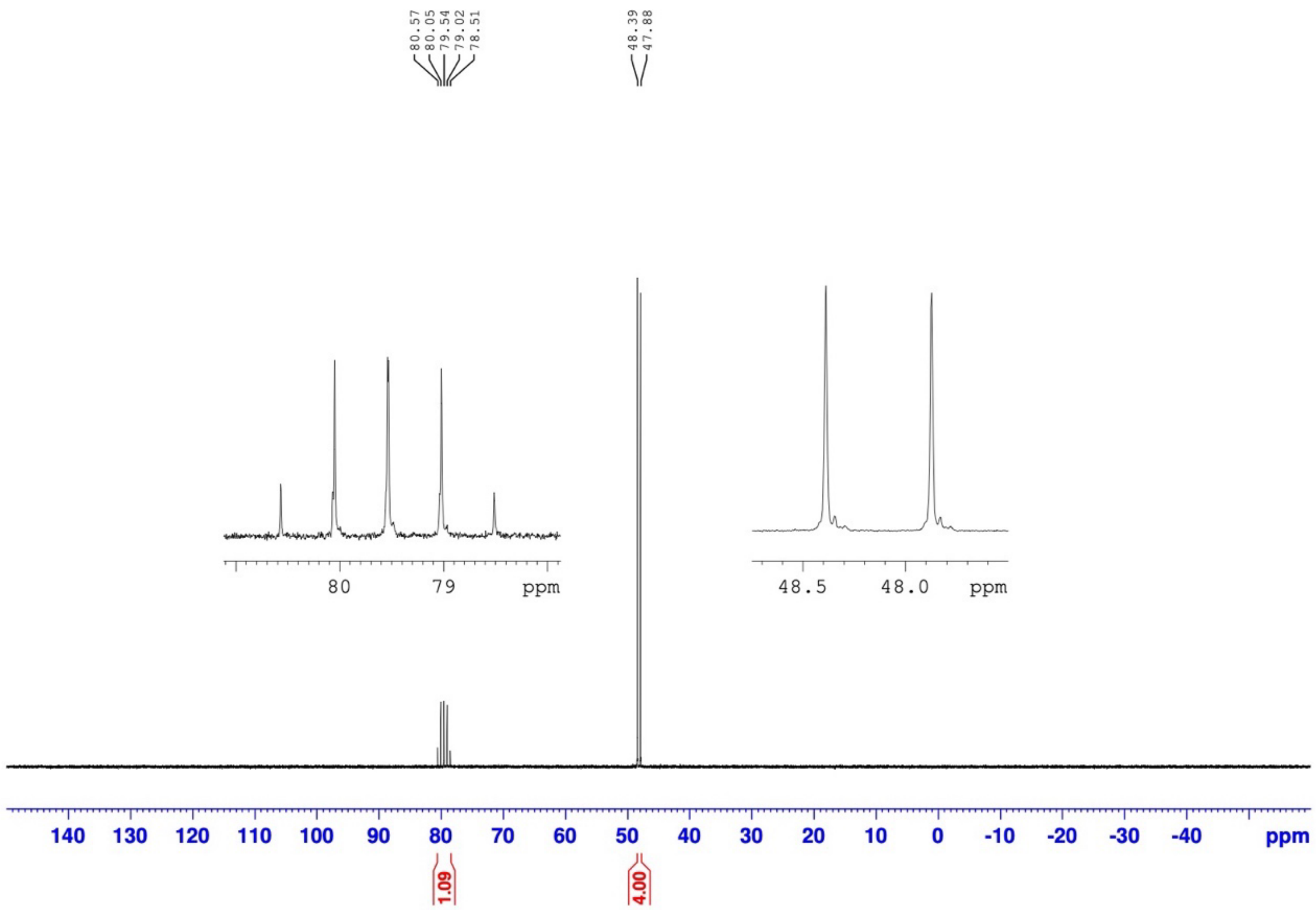

Figure S376. ^{19}F NMR spectrum of (3'-(4-chlorophenyl)-[1,1'-bi(bicyclo[1.1.1]pentan)]-3-yl)pentafluoro-λ^6-sulfane (**S-50**) in $CDCl_3$.

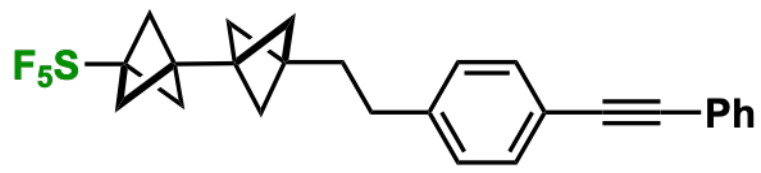

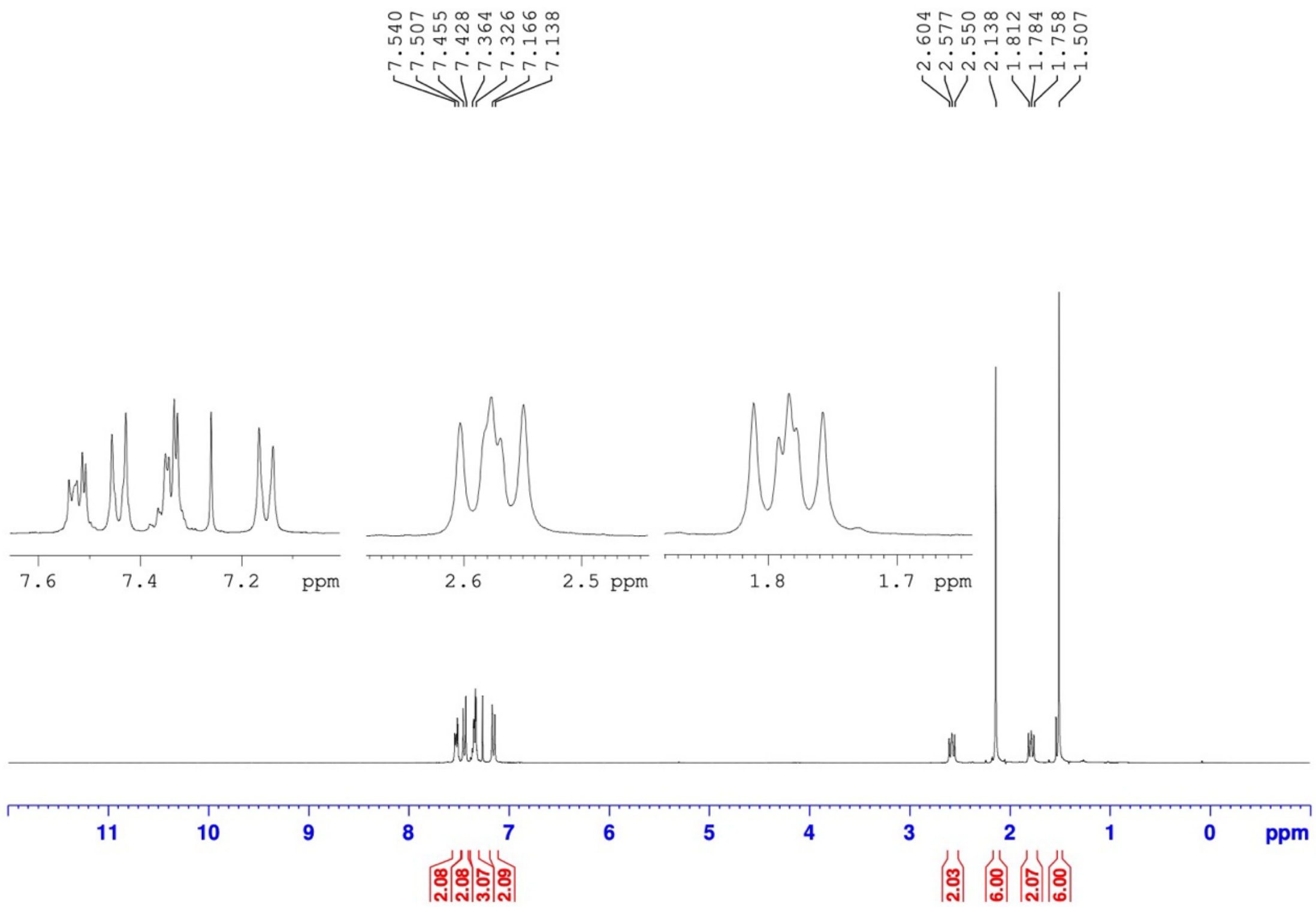

Figure S377. ^{1}H NMR spectrum of pentafluoro(3'-(4-(phenylethynyl)phenethyl)-[1,1'-bi(bicyclo[1.1.1]pentan)]-3-yl)- λ^6-sulfane (**S-52**) in $CDCl_3$.

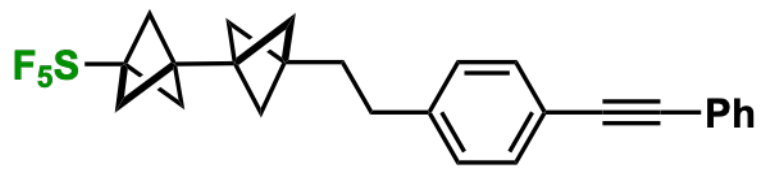

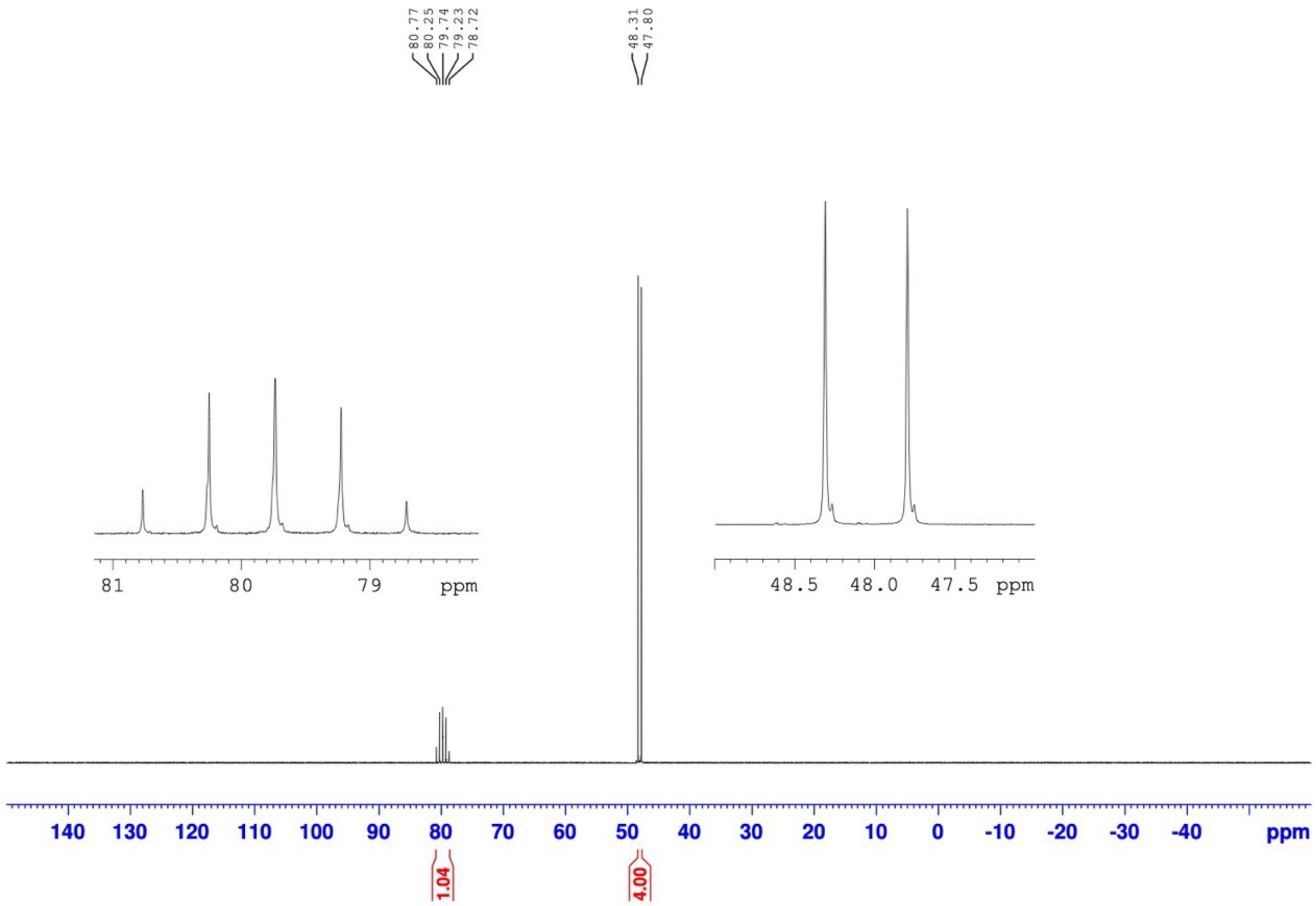

Figure S378. ^{19}F NMR spectrum of pentafluoro(3'-(4-(phenylethynyl)phenethyl)-[1,1'-bi(bicyclo[1.1.1]pentan)]-3-yl)- λ^6-sulfane (**S-52**) in $CDCl_3$.

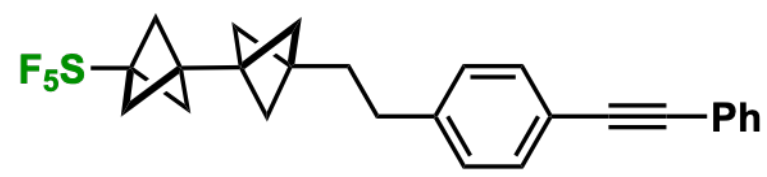

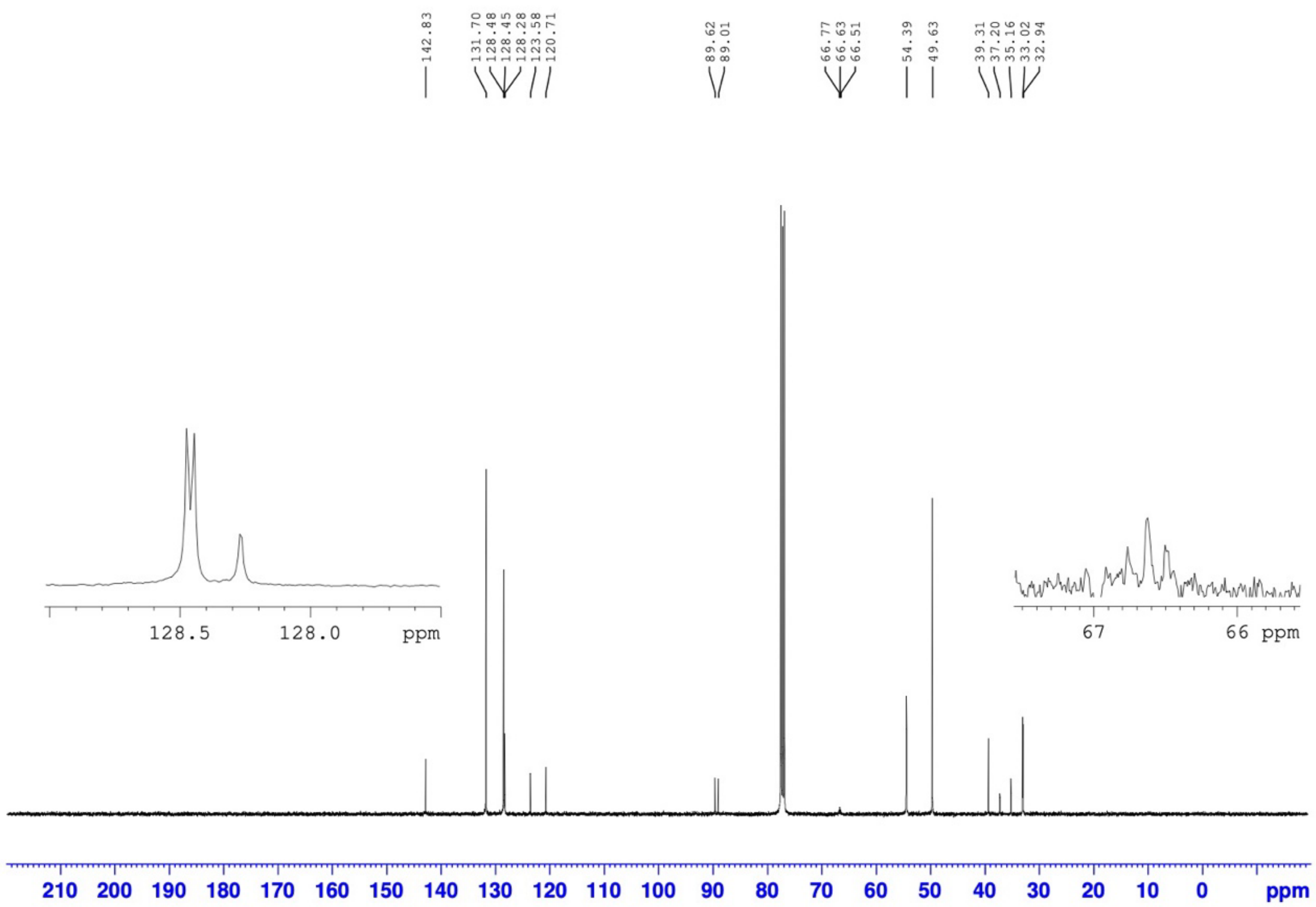

Figure S379. ^{113}C{^{1}H} NMR spectrum of pentafluoro(3'-(4-(phenylethynyl)phenethyl)-[1,1'-bi(bicyclo[1.1.1]pentan)]-3-yl)- λ^6-sulfane (**S-52**) in $CDCl_3$.

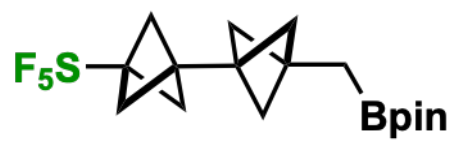

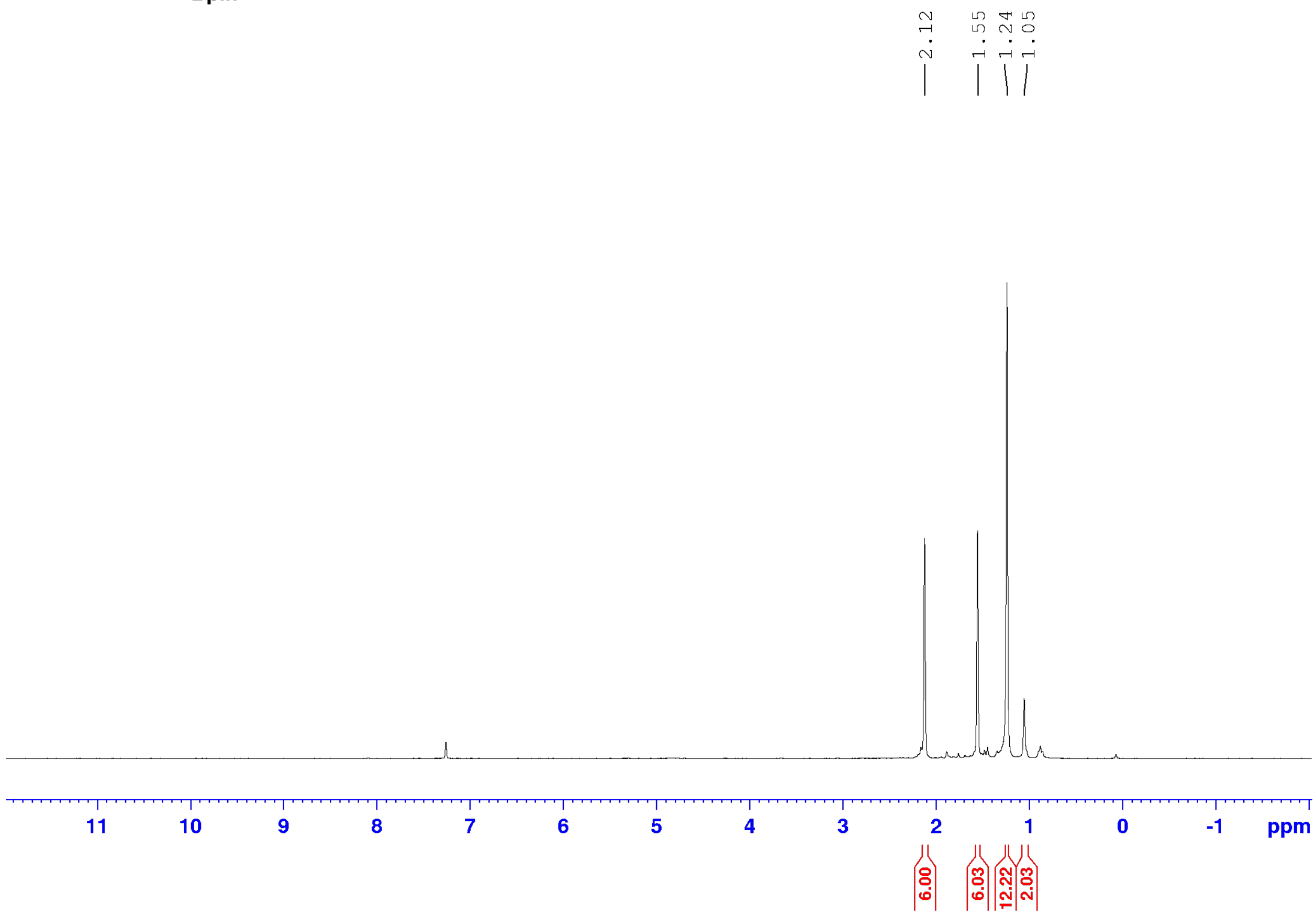

Figure S380. ^{1}H NMR spectrum of 4,4,5,5-tetramethyl-2-((3'-(pentafluoro-λ^6-sulfaneyl)-[1,1'-bi(bicyclo[1.1.1]pentan)]-3-yl)methyl)-1,3,2-dioxaborolane (**S-53**) in $CDCl_3$.

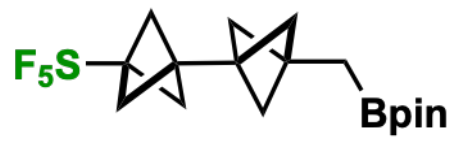

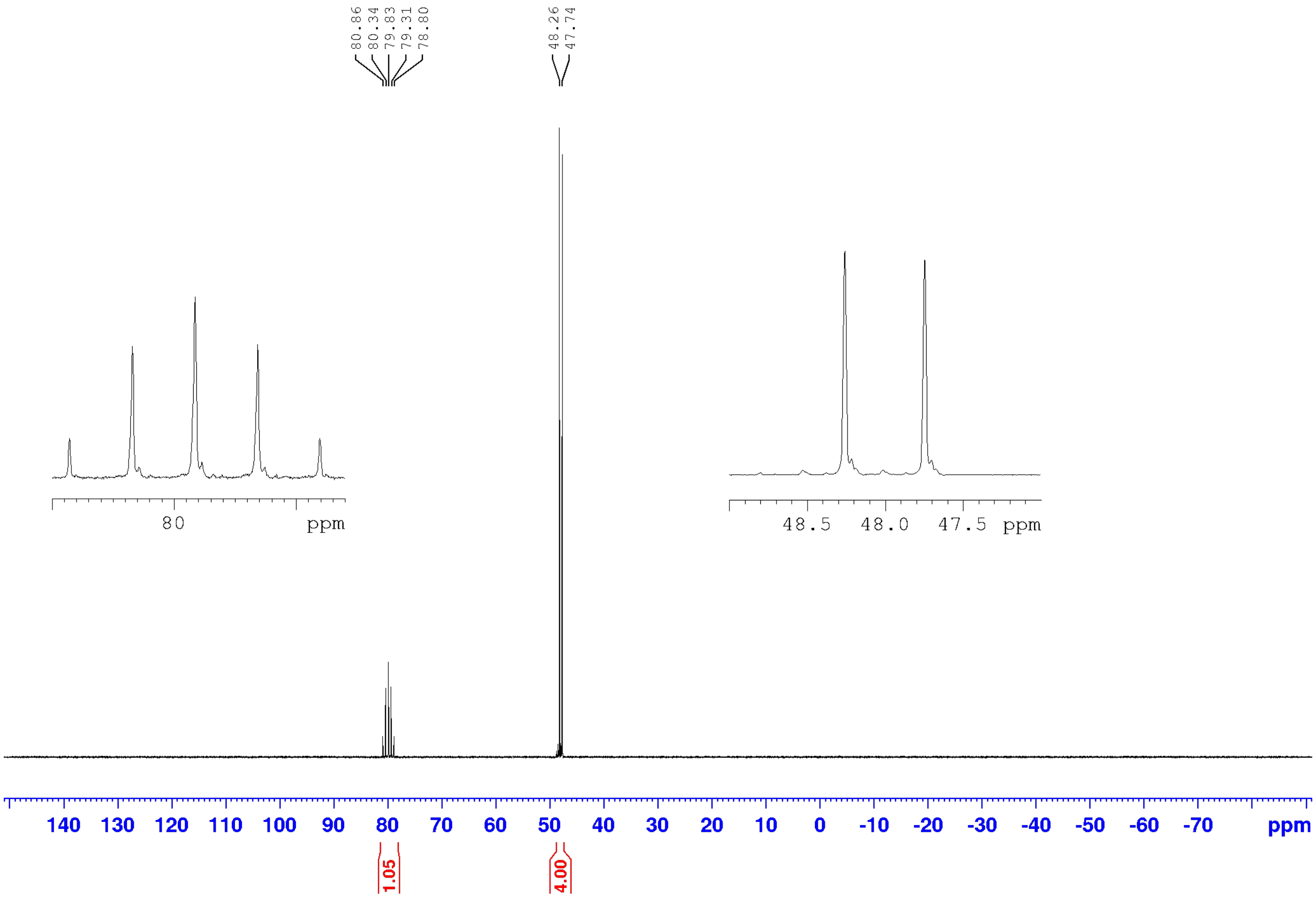

Figure S381. ^{19}F NMR spectrum of 4,4,5,5-tetramethyl-2-((3'-(pentafluoro-λ^6-sulfaneyl)-[1,1'-bi(bicyclo[1.1.1]pentan)]-3-yl)methyl)-1,3,2-dioxaborolane (**S-53**) in $CDCl_3$.

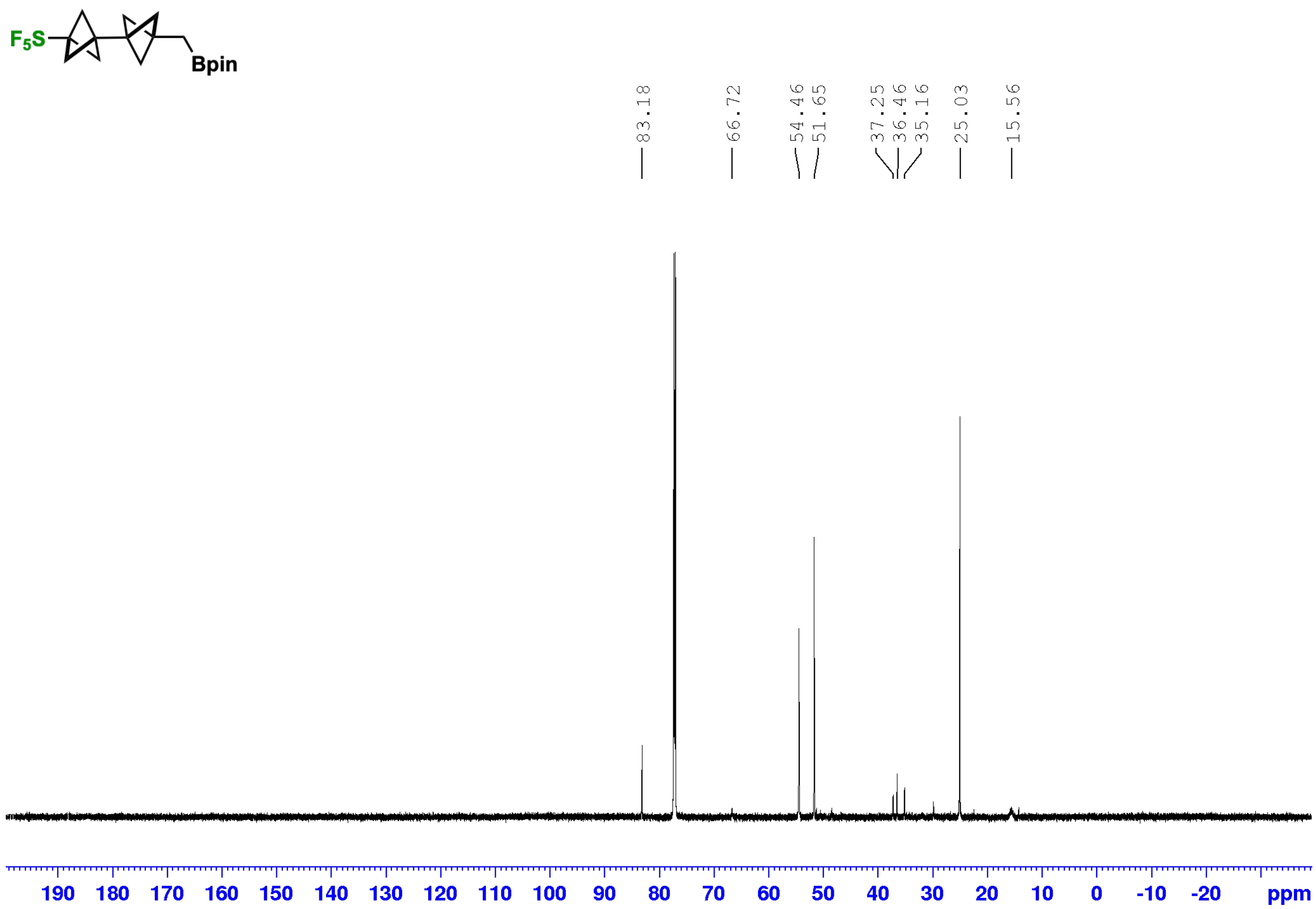

Figure S382. $^{13}C\{^1H\}$ NMR spectrum of 4,4,5,5-tetramethyl-2-((3'-(pentafluoro-λ^6-sulfaneyl)-[1,1'-bi(bicyclo[1.1.1]pentan)]-3-yl)methyl)-1,3,2-dioxaborolane (**S-53**) in $CDCl_3$.

References

[1] Fulmer, G. R.; Miller, A. J. M.; Sherden, N. H.; Gottlieb, H. E.; Nudelman, A.; Stolz, B. M.; Bercaw, J. E.; Goldberg, K. I. NMR Chemical Shifts of Trace Impurities: Common Laboratory Solvents, Organics, and Gases in Deuterated Solvents Relevant to the Organometallic Chemist. *Organometallics* **2010**, *29*, 2176-2179. DOI: https://doi.org/10.1021/om100106e.

[2] Rosenau, C. P.; Jelier, B. J.; Gossert, A. D.; Togni, A. Exposing the Origins of Irreproducibility in Fluorine NMR Spectroscopy. *Angew. Chem. Int. Ed.* **2018**, *57*, 9528-9533. DOI: 10.1002/anie.201802620.

[3] Kraemer, Y.; Park, S.; Kong, W.-Y.; Chen, Y.; Witt, A. J.; Buldt, J. A.; Ragan, A. N.; Holder, L. M.; Tantillo, D. J.; Pitts, C. R. Direct N–SF_5 and N–SF_4CF_3 Bond Formation through Strain-Release Functionalization of 3-Substituted [1.1.0]Azabicyclobutanes. *J. Am. Chem. Soc.* **2025**, *147*, 27892-27904. DOI: 10.1021/jacs.5c07137.

[4] Gianatassio, R.; Lopchuk, J. M.; Wang, J.; Pan, C.-M.; Malins, L. R.; Prieto, L.; Brandt, T. A.; Collins, M. R.; Gallego, G.M.; Sach, N. W.; Spangler, J. E.; Zhu, H.; Zhu, J.; Baran, P. S. *Science* **2016**, *351*, 241-246.

[5] Elek, M.; Djokovic, N.; Frank, A.; Oljacic, S.; Zivkovic, A.; Nikolic, K.; Stark, H. Synthesis, In Silico, and In Vitro Studies of Novel Dopamine D_2 and D_3 Receptor Ligands. *Arch. Pharm. (Weinheim)* **2021**, *354*, 2000486. DOI: 10.1002/ardp.202000486.

[6] Wei, Z.-L.; Nguyen, M. T.; O'Mahony, D. J. R.; Acevedo, A.; Zipfel, S.; Zhang, Q.; Liu, L.; Dourado, M.; Chi, C.; Yip, V.; DeFalco, J.; Gustafson, A.; Emerling, D. E.; Kelly, M. G.; Kincaid, J.; Vincent, F.; Duncton, M. A. J. Identification of Orally Bioavailable Antagonists of the TRPV4 Ion Channel. *Bioorg. Med. Chem. Lett.* **2015**, *25*, 4011-4015. DOI: 10.1016/j.bmcl.2015.06.098.

[7] Satoh, E.; Kasahara, R.; Fukatsu, K.; Aoki, T.; Harayama, H.; Murata, T. Benzpyrimoxan: Design, Synthesis, and Biological Activity of a Novel Insecticide. *J. Pestic. Sci.* **2021**, *46*, 109-114. DOI: 10.1584/jpestics.D20-069.

[8] Bonku, E. M.; Qin, H.; Odilov, A.; Yang, F.; Xing, X.; Wang, X.; Guma, S. D.; Shen, J. Efficient Large-Scale Process for Tecovirimat via Reactive Distillation for the Preparation of Cycloheptatriene. *Org. Process Res. Dev.* **2023**, *27*, 1984-1991. DOI: 10.1021/acs.oprd.3c00151.

[9] Measom, N. D.; Down, K. D.; Hirst, D. J.; Jamieson, C.; Manas, E. S.; Patel, V. K.; Somers, D. O. Investigation of a Bicyclo[1.1.1]pentane as a Phenyl Replacement within an $LpPLA_2$ Inhibitor. *ACS Med. Chem. Lett.* **2017**, *8*, 43-48. DOI: 10.1021/acsmedchemlett.6b00281.

[10] Lee, S.-L.; Hsu, E.-C.; Chou, C.-C.; Chuang, H.-C.; Bai, L.-Y.; Kulp, S. K.; Chen, C.-S. Identification and Characterization of a Novel Integrin-Linked Kinase Inhibitor. *J. Med. Chem.* **2011**, *54*, 6364-6374. DOI: 10.1021/jm2007744.

[11] Li, C.; Kawamata, Y.; Nakamura, H.; Vantourout, J. C.; Liu, Z.; Hou, Q.; Bao, D.; Starr, J. T.; Chen, J.; Yan, M.; Baran, P. S. Electrochemically Enabled, Nickel-Catalyzed Amination. *Angew. Chem. Int. Ed.* **2017**, *56*, 13088-13093. DOI: 10.1002/anie.201707906.

[12] Zipp, G. G.; Barbosa, J.; Green, M. A.; Terranova, K. M.; Fink, C.; Yu, X.-C.; Nouraldeen, A.; Wilson, A.; Savelieva, K.; Lanthorn, T. H.; Kimball, S. D. Novel Inhibitors of the High-affinity L-Proline Transporter as Potential Therapeutic Agents for the Treatment of Cognitive Disorders. *Bioorg. Med. Chem. Lett.* **2014**, *24*, 3886-3890. DOI: 10.1016/j.bmcl.2014.06.049.

[13] Buldt, J. A.; Kong, W.-Y.; Kraemer, Y.; Belsuzarri, M. M.; Patel, A. H.; Fettinger, J. C.; Tantillo, D. J.; Pitts, C. R. Controlled Oligomerization of [1.1.1]Propellane through Radical Polarity Matching: Selective Synthesis of SF_5- and CF_3SF_4-Containing [2]Staffanes. *Beilstein J. Org. Chem.* **2024**, *20*, 3134-3143. DOI: 10.3762/bjoc.20.259.

[14] Zhao, X.; Shou, J.-Y.; Qing, F.-L. Iodopentafluorosulfanylation of [1.1.1]Propellane and Further Functionalizations. *Sci. China Chem.* **2023**, *66*, 2871-2877. DOI: 10.1007/s11426-023-1560-3.

[15] Kokhan, S. O.; Valter, Y. B.; Tymtsunik, A. V.; Komarov, I. V.; Grygorenko, O. O. Bicyclo[1.1.1]pentane-Derived Building Blocks for Click Chemistry. *Eur. J. Org. Chem.* **2017**, *2017*, 6450-6456. DOI: 10.1002/ejoc.201701296.

[16] Callahan, R.; Prip, R.; Shariff, N.; Sklyut, O.; Rothchild, R.; Bynum, K. ^1H and ^{13}C Nuclear Magnetic Resonance Studies of the Hindered Phencyclone Adducts of Some Smaller Branched N-Alkyl Maleimides: Rigorous Aryl Proton Assignments with High-Resolution Two-Dimensional (COSY45) Spectroscopy, and

Anisotropic Shielding Effects and Ab Initio Geometry Optimizations. *Appl. Spectrosc.* **2005**, *59*, 354-365. DOI: 10.1366/0003702053585426.

[17] de Robichon, M.; Kratz, T.; Beyer, F.; Zuber, J.; Merten, C.; Bach, T. Enantioselective, Intermolecular [$_{\pi}$2+$_{\sigma}$2] Photocycloaddition Reactions of 2(1H)-Quinolones and Bicyclo[1.1.0]butanes. *J. Am. Chem. Soc.* **2023**, *145*, 24466-24470. DOI: 10.1021/jacs.3c08404.

[18] Zielke, F. M.; Rutjes, F. P. J. T. Recent Advances in Bioorthogonal Ligation and Bioconjugation. *Top. Curr. Chem.* **2023**, *381*, 35. DOI: 10.1007/s41061-023-00445-6.

[19] Altman, M. D.; Cash, B. D.; Childers, M. L.; Cumming, J. N.; DeMong, D. E.; Haidle, A. M.; Henderson, T.J.; Jewell, J. P.; Larsen, M. A.; Lim, J.; Lu, M.; Otte, R.D.; Trotter, B.W. Sting Agonist Compounds. US20190300513A1, 2019.

[20] Zhao-Kui, W.; Yimin, J.; Xuedong, D.; Qian, L.; Shun, C. W., Gang, D.; Liqiang, F. Heteroaryldihydropyrimidein Derivatives and Methods of Treating Hepatitis B. WO2019001420A1, 2019.

[21] Ripenko, V.; Sham, V.; Levchenko, V.; Holovchuk, S.; Vysochyn, D.; Klymov, I.; Kyslyi, D.; Veselvych, S.; Zhersh, S.; Dmytriv, Y.; Tolmachev, A.; Sadkova, I.; Pishel, I.; Horbatok, K.; Kosach, V.; Nikandrova, Y.; Mykhailiuk, P.K. Light-enabled scalable synthesis of bicyclo[1.1.1]pentane halides and their functionalizations. *Nat. Synth.* **2024**, *3*, 1538-1549. DOI: 10.1038/s44160-024-00637-y.

[22] Yu, I.F.; Manske, J.L.; Diéguez-Vázquez, A.; Misale, A.; Pashenko, A. E.; Mykhailiuk, P. K.; Ryabukhin, S. V.; Volochnyuk, D. M.; Hartwig, J.F. Catalytic undirected borylation of tertiary C–H bonds in bicyclo[1.1.1]pentanes and bicyclo[2.1.1]hexanes. *Nat. Chem.* **2023**, *15*, 685-693. DOI: 10.1038/s41557-023-01159-4.

[23] Galant, O.; Donmez, H. B.; Barner-Kowollik, C.; Diesendruck, C. E. Flow Photochemistry for Single-Chain Polymer Nanoparticle Synthesis. *Angew. Chem. Int. Ed.* **2021**, *60*, 2042-2046. DOI: 10.1002/anie.202010429.

[24] Link, J.; Chen, Y.; Jae, HS.; Patel, J.; Pliushchev, M.; Rohde, J.; Shuai, Q.; Sorensen, B.; Winn, M.; Wodka, D.; Yong, H. Inhibitor of the 11-beta-hydroxysteroid dehydrogenase Type 1 enzyme. US200502776471A1, 2005.

[25] Friedrich, J.; Walczak, K.; Dolg, M.; Piestert, F.; Lauterbach, T.; Worgull, D.; Gansäuer, A. Titanocene Catalyzed 4-exo Cyclizations: Mechanism, Experiment, Catalyst Design. *J. Am. Chem. Soc.* **2008**, *130*, 1788-1796. DOI: 10.1021/ja077596b.

[26] Sun, J.; Peter, A.; He, J.; Tsien, J.; Zhang, H.; Cagan, D.A.; Vokits, B.p.; Peters, D. S.; Oderinde, M. S.; Mandler, M. D.; Richardson, P.; Chen, D.; Palkowitz, M. D.; Raheja, N.; Kawamata, Y.; Baran, P. Sulfonyl hydrazides as a general redox-neutral platform for radical cross-coupling. *Science* **2025**, *387*,1377-1383. DOI: 10.1126/science.adu6406.

[27] Colletto, C. Reductive Transformations with Sodium Borohydride and Their Applications. *Org. Process Res. Dev.* **2026**, *30* (5), 1082-1118 DOI: 10.1021/acs.oprd.5c00357.

[28] Jelínková, K.; Závodná, A.; Kaleta, J.; Janovský, P.; Zatloukal, F.; Nečas, M.; Prucková, Z.; Dastychová, L.; Rouchal, M.: Vícha, R. Two Squares in a Barrel: An Axially Disubstituted Conformationally Rigid Aliphatic Binding Motif for Cucurbit[6]uril. *J. Org. Chem.* **2023**, *88*, 15615-15625. DOI: 10.1021/acs.joc.3c01556.

[29] Zheng, TC.; Burkart, M.; Richardson, D. E. A general and mild synthesis of thioesters and thiols from halides, *Tetrahedron Lett.* **1999**, *40*, 603-606. DOI: 10.1016/S0040-4039(98)02545-3.

[30] Andrade, J. T.; Lima, W. G.; Sousa, J. F.; Saldanha, A. A.; Pereira De Sá, N.; Morais, F. B.; Silva, M. K. P.; Viana, G. H. R.; Johann, S.; Soares, A. C.; Araújo, L. A.; Fernandes, S. O. A.; Cardoso, V. N.; Ferreira, J. M. S. Design, synthesis, and biodistribution studies of new analogues of marine alkaloids: Potent in vitro and in vivo fungicidal agents against Candida spp. *Eur. J. Med. Chem.* **2021**, *210*, 113048. DOI: 10.1016/j.ejmech.2020.113048.

[31] Liu, X.; Zhou, Y.; Qi, X.; Li, R.; Liu, P.; Dong, G. Palladium/Norbornene-Catalyzed Direct Vicinal Di-Carbo-Functionalization of Indoles: Reaction Development and Mechanistic Study. *Angew. Chem. Int. Ed.* **2023**, *62*, e202310697. DOI: 10.1002/anie.202310697.

[32] Laulhé, S.; Gori, S. S.; Nantz, M. H. A Chemoselective, One-Pot Transformation of Aldehydes to Nitriles. *J. Org. Chem.* **2012**, *77*, 9334-9337. DOI: 10.1021/jo301133y.

[33] Alapafuja, S. O.; Nikas, S. P.; Bharathan, I. T.; Shukla, V. G.; Nasr, M. L.; Bowman, A. L.; Zvonok, N.; Li, J.; Shi, X.; Engen, J. R.; Makriyannis, A. Sulfonyl Fluoride Inhibitors of Fatty Acid Amide Hydrolase. *J. Med. Chem.* **2012**, *55*, 10074-10089. DOI: 10.1021/jm301205j.

[34] Orbe, J.; Rodríguez, J. A.; Sánchez-Arias, J. A.; Salicio, A.; Belzunce, M.; Ugarte, A.; Chang, H.C.Y.; Rabal, O.; Oyarzabal, J.; Páramo, J. A. Discovery and Safety Profiling of a Potent Preclinical Candidate, (4-[4-[[(3R)-3-(Hydroxycarbamoyl)-8-azaspiro[4.5]decan-3-yl]sulfonyl]phenoxy]-N-methylbenzamide) (CM-352), for the Prevention and Treatment of Hemorrhage. *J. Med. Chem.* **2015**, *58*, 2941-2957. DOI: 10.1021/jm501939z.

[35] Thomas, J.; Fokin, V. V. Regioselective Synthesis of Fluorosulfonyl 1,2,3-Triazoles from Bromovinylsulfonyl Fluoride. *Org. Lett.* **2018**, *20*, 3749-3752. DOI: 10.1021/acs.orglett.8b01309.

[36] Cai, X.; Tohti, A.; Ramirez, C.; Harb, H.; Fettinger, J. C.; Hratchian, H. P.; Stokes, B. J. Dispersion-Controlled Regioselective Acid-Catalyzed Intramolecular Hydroindolation of *cis*-Methindolylstyrenes To Access Tetrahydrobenzo[cd]indoles. *Org. Lett.* **2019**, *21*, 1574-1577. DOI: 10.1021/acs.orglett.9b00043.

[37] Karmakar, A.; Nimje, R. Y.; Silamkoti, A.; Pitchai, M.; Basha, M.; Singarayer, C.; Ramasamy, D.; Babu, G. T. V.; Samikannu, R.; Subramaniam, S.; Anjanappa, P.; Vetrichelvan, M.; Kumar, H.; Dikundwar, A. G.; Gupta, A.; Gupta, A. K.; Rampulla, R.; Dhar, T. G. M.; Mathur, A. Practical Synthesis of (3aR, 9bR)-8-Fluoro-7-(perfluoropropan-2-yl)-9b-(phenylsulfonyl)-2,3,3a,4,5,9b-hexahydro-1H-benzo[e]indole: An Advanced Intermediate to Access the RORγt Inverse Agonist BMT-362265. *Org. Process Res. Dev.* **2021**, *25*, 1001-1014. DOI: 10.1021/acs.oprd.1c00019.

[38] Kendall, J.T. Synthesis of [2,3,3,2′,3′,5′,6′-2H7]-L-tyrosine from phenol-d6. J. Label Compd. *Radiopharm.* **2000**, *43*, 917-924. DOI: 10.1002/1099-1344.

[39] Satoh, Y.; Gude, C.; Chan, K.; Firooznia, F. Synthesis of 4-Substituted Phenylalanine Derivatives by Cross-Coupling Reaction of p-Boronophenylalanines. *Tetrahedron Lett.* **1997**, *38*, 7645-7648. DOI: 10.1016/S0040-4039(97)10082-X.

[40] Ye, L.; Tian, Y.; Meng, X.; Gu, Q.-S.; Liu, X.-Y. Enantioselective Copper(I)/Chiral Phosphoric Acid Catalyzed Intramolecular Amination of Allylic and Benzylic C–H Bonds. *Angew. Chem. Int. Ed.* **2020**, *59*, 1129-1133. DOI: 10.1002/anie.201911742.

[41] Qin, L.; Cui, H.; Zou, D.; Li, J.; Wu, Y.; Zhu, Z.; Wu, Y. Pd-catalyzed amidation of aryl(Het) halides with tert-butyl carbamate. *Tetrahedron Lett.* ***2010***, *51*, 4445-4448. DOI: 10.1016/j.tetlet.2010.06.083.

[42] Kim, B.; Chinn, A. J.; Fandrick, D. R.; Senanayake, C. H.; Singer, R. A.; Miller, S. J. Distal Stereocontrol Using Guanidinylated Peptides as Multifunctional Ligands: Desymmetrization of Diarylmethanes via Ullman Cross-Coupling. *J. Am. Chem. Soc.* **2016**, *138*, 7939-7945. DOI: 10.1021/jacs.6b03444.

[43] Testolin, G.; Richter, J.; Ritter, A.; Prochnow, H.; Köhnke, J.; Brönstrup, M. Optical Modulation of Antibiotic Resistance by Photoswitchable Cystobactamids. *Chem. Eur. J.* **2022**, *28*, e202201297. DOI: 10.1002/chem.202201297.

[44] Munawar, S.; Zahoor, A. F.; Hussain, S. M.; Ahmad, S.; Mansha, A.; Parveen, B.; Ali, K. G.; Irfan, A. Steglich Esterification: A Versatile Synthetic Approach toward the Synthesis of Natural Products, Their Analogues/Derivatives. *Heliyon* **2024**, *10*, e23416. DOI: 10.1016/j.heliyon.2023.e23416.

[45] Dai, D. Methods of preparing tecovirimat. WO2014028545A1, 2014. DOI: WO2014028545A1.

[46] Nugent, J.; Shire, B.R.; Caputo, D.F.J.; Pickford, H.D.; Nightingale, F.; Houlsby, I.T.T.; Mousseau, J.J.; Anderson, E. A. Synthesis of All-Carbon Disubstituted Bicyclo[1.1.1]pentanes by Iron-Catalyzed Kumada Cross-Coupling. *Angew. Chem., Int. Ed*. **2020**, *59*, 11866-11870. DOI: 10.1002/anie.202004090.

[47] Cassera, E.; Del Tito, A. M.; Merli, D.; Ravelli, D.; Lefebvre, Q.; Fagnoni, M.; Direct decarboxylative alkylation of bicycloalkyl and cubane carboxylic acids enabled by redox-mediated visible-light photoorganocatalysis. *Green. Chem.* 2025, 28, 12740-12747. DOI: 10.1039/d6gc02631a.

[48] Thirumoorthi, N. T.; Shen, C. J.; Adsool, V. A. Expedient synthesis of 3-phenylbicyclo[1.1.1]pentan-1-amine via metal-free homolytic aromatic alkylation of benzene. *Chem. Commun*. **2015**, *51,* 3139-3142. DOI: 10.1039/C4CC09655J.

[49] Polites, V. C.; Badir, S. O.; Keess, S.; Jolit, A.; Molander, G. A. Nickel-Catalyzed Decarboxylative Cross-Coupling of Bicyclo[1.1.1]pentyl Radicals Enabled by Electron Donor–Acceptor Complex Photoactivation. *Org. Lett*. **2021**, *23*, 4828-4833. DOI: 10.1021/acs.orglett.1c01558.

[50] Yang, Y.; Tsien, J.; Hughes, J.M.E.; Peters, B.K.; Merchant, R. R.; Qin, T. An intramolecular coupling approach to alkyl bioisosteres for the synthesis of multisubstituted bicycloalkyl boronates. *Nat. Chem.* **2024**, *13*, 950-955. DOI: https://doi.org/10.1038/s41557-021-00786-z.

[51] Shi, S-H.; Song, S.; Jiao, N.; Nitromethane assisted Brønsted acid catalyzed regioselective halogenation of alkyl aromatics. *Mol. Catal*. **2024**, *553*, 113777. DOI: 10.1016/j.mcat.2023.113777.

[52] Liu, C.; Wang, W.; Li, Z.; Cui, I.; Li, C. Silver-Catalyzed Decarboxylative Radical Azidation of Aliphatic Carboxylic Acids in Aqueous Solution. *J. Am. Chem. Soc.* **2015**, 137, 9820-9823. DOI: 10.1021/jacs.5b06821.

[53] Harwood, S.J.; Palkowitz, M. D.; Gannett, C. N.; Perez, P.; Yao, Z.; Sun, L.; Abruña, H.; Anderson S. L.; Baran, P. S. Modular terpene synthesis enabled by mild electrochemical couplings. *Science*. **2022**, *375*, 745-752. DOI: 10.1126/science.abn1395.

[54] Fawcett, A.; Pradeilles, J.; Wang, Y.; Mutsuga, T.; Myers, E. L.; Aggarwal, V. K. Photoinduced decarboxylative borylation of carboxylic acids. *Science.* **2017***, 357*, 283-286. DOI: 10.1126/science.aan3679.

[55] VanHeyst, M. D.; Qi, J.; Roecker, A. J.; Hughes, J. M. E.; Chend, L.; Zhao, Z.; Yin, J. Continuous Flow-Enabled Synthesis of Bench-Stable Bicyclo[1.1.1]pentane Trifluoroborate Salts and Their Utilization in Metallaphotoredox Cross-Couplings. *Org. Lett.* **2020**, *22*, 1648-1654. DOI: 10.1021/acs.orglett.0c00242.

[56] Dolomanov, O. V.; Bourhis, L. J.; Gildea, R. J.; Howard, J. A. K.; Puschmann, H. OLEX2: a complete structure solution, refinement and analysis program. *J. Appl. Cryst*. **2009**, *42*, 339-341. DOI: 10.1107/S0021889808042726.

[57] Macrae, C. F.; Sovago, S. J.; Galek, P. T. A.; McCabe, P.; Pidcock, E.; Platings, M.; Shields, G. P.; Stevens, J. S.; Towler, M.; Wood, P. A. *J. Appl. Cryst*. **2020**, *53*, 226-235. DOI: 10.1107/S1600576719014092.

[58] Frisch, M. J.; Trucks, G. W.; Schlegel, H. B.; Scuseria, G. E.; Robb, M. A.; Cheeseman, J. R.; Scalmani, G.; Barone, V.; Petersson, G. A; Nakatsuji, H.; Li, X.; Caricato, M.; Marenich, A. V.; Bloino, J.; Janesko, B. G.; Gomperts, R.; Mennucci, B.; Hratchian, H. P.; Ortiz, J. V.; Izmaylov, A. F.; Sonnenberg, J. L.; Williams; Ding, F.; Lipparini, F.; Egidi, F.; Goings, J.; Peng, B.; Petrone, A.; Henderson, T.; Ranasinghe, D.; Zakrzewski, V. G.; Gao, J.; Rega, N.; Zheng, G.; Liang, W.; Hada, M.; Ehara, M.; Toyota, K.; Fukuda, R.; Hasegawa, J.; Ishida, M.; Nakajima, T.; Honda, Y.; Kitao, O.; Nakai, H.; Vreven, T.; Throssell, K.; Montgomery Jr, J. A.; Peralta, J. E.; Ogliaro, F.; Bearpark, M. J.; Heyd, J. J.; Brothers, E. N.; Kudin, K. N.; Staroverov, V. N.; Keith, T. A.; Kobayashi, R.; Normand, J.; Raghavachari, K.; Rendell, A. P.; Burant, J. C; Iyengar, S. S.; Tomasi, J.; Cossi, M.; Millam, J. M.; Klene, M.; Adamo, C.; Cammi, R.; Ochterski, J. W.; Martin, L.; Morokuma, K.; Farkas, O.; Foresman, J. B.; Fox, D. J. Gaussian 16, Revision C.01. Gaussian, Inc., Wallingford CT, **2016**.

[59] Chai, J.-D.; Head-Gordon, M. Long-Range Corrected Hybrid Density Functionals with Damped Atom–Atom Dispersion Corrections. *Phys. Chem. Chem. Phys*. **2008**, *10*, 6615-6620. DOI: 10.1039/B810189B.

[60] Weigend, F.; Ahlrichs, R. Balanced Basis Sets of Split Valence, Triple Zeta Valence and Quadruple Zeta Valence Quality for H to Rn: Design and Assessment of Accuracy. *Phys. Chem. Chem. Phys*. **2005**, *7*, 3297-3305. DOI: 10.1039/B508541A.

[61] Cancès, E.; Mennucci, B.; Tomasi, J. A New Integral Equation Formalism for the Polarizable Continuum Model: Theoretical Background and Applications to Isotropic and Anisotropic Dielectrics. *J. Chem. Phys.* **1997**, *107*, 3032-3041. DOI: 10.1063/1.474659.

[62] Neese, F. Software Update: The ORCA Program System—Version 6.0. *WIREs Comput. Mol. Sci.* **2025**, *15*, DOI: 10.1002/wcms.70019.

[63] De Souza, B. GOAT: A Global Optimization Algorithm for Molecules and Atomic Clusters. *Angew. Chem. Int. Ed.* **2025**, *64*, e202500393. DOI: 10.1002/anie.202500393.

[64] Bannwarth, C.; Ehlert, S.; Grimme, S. GFN2-xTB—An Accurate and Broadly Parametrized Self-Consistent Tight-Binding Quantum Chemical Method with Multipole Electrostatics and Density-Dependent Dispersion Contributions. *J. Chem. Theory Comput.* **2019**, *15*, 1652-1671. DOI: 10.1021/acs.jctc.8b01176.
[65] Fukui, K. The Path of Chemical Reactions - the IRC Approach. *Acc. Chem. Res.* **1981**, *14*, 363-368. DOI: 10.1021/ar00072a001.
[66] Page, M.; McIver, J. W. On Evaluating the Reaction Path Hamiltonian. *J. Chem. Phys.* **1988**, *88*, 922-935. DOI: 10.1063/1.454172.
[67] Grimme, S. Supramolecular Binding Thermodynamics by Dispersion-Corrected Density Functional Theory. *Chem. Eur. J.* **2012**, *18*, 9955-9964. DOI: 10.1002/chem.201200497.
[68] Luchini, G.; Alegre-Requena, J. V.; Funes-Ardoiz, I.; Paton, R. S. GoodVibes: Automated Thermochemistry for Heterogeneous Computational Chemistry Data. *F1000Research.* **2020**, *9*, 291. DOI: 10.12688/f1000research.22758.1.
[69] Reed, A. E.; Weinstock, R. B.; Weinhold, F. Natural population analysis. *J. Chem. Phys.* **1985**, *83*, 735-746. DOI: 10.1063/1.449486.
[70] Glendening, E. D.; Landis, C. R.; Weinhold, F. NBO 7.0 : New vistas in localized and delocalized chemical bonding theory. *J. Comput. Chem.* **2019**, *40*, 2234-2241. DOI: 10.1002/jcc.25873.
[71] Álvarez-Moreno, M.; De Graaf, C.; López, N.; Maseras, F.; Poblet, J. M.; Bo, C. Managing the Computational Chemistry Big Data Problem: The ioChem-BD Platform. *J. Chem. Inf. Model.* **2015**, *55*, 95-103. DOI: 10.1021/ci500593j.
